# Cavity nesting birds show behavioural plasticity to simulated territorial intrusions in response to natural resource pulses

**DOI:** 10.1038/s41598-025-93109-y

**Published:** 2025-03-18

**Authors:** Andrea R. Norris, Kathy Martin

**Affiliations:** 1 Department of Forest and Conservation Sciences, University of British Columbia, 2424 Main Mall, Vancouver, BC V6T 1Z4 Canada; 2 Science & Technology Branch, Environment and Climate Change Canada, 60 Front Street, Nanaimo, BC V9R 5H7 Canada

**Keywords:** Inter-specific competition, Dominance hierarchies, Social resilience, Ecological resilience, Ecological disruption, Behavioural interactions, Behavioural ecology, Community ecology

## Abstract

We investigated the impact of two natural pulses (food and nesting resources) on intra- and inter-specific territorial behaviour of species that co-occur year-round in multi-species groups. We simulated conspecific and heterospecific territorial intrusions in two cavity-nesting species using 974 model presentations with territorial song playbacks during and after a dual resource pulse of insect (bark beetle) prey and nest cavities across 5 years in British Columbia, Canada. As beetle abundance increased, both species increased aggression toward conspecific intruders. At peak beetle abundance the (typically) subordinate generalist insectivore, mountain chickadee (*Poecile gambeli*), attacked model intruders more frequently than did the dominant bark insectivore, red-breasted nuthatch (*Sitta canadensis*), and responded more aggressively to nuthatch intruders than to conspecifics. The reversal in the inter-specific dominance hierarchy suggests that behavioural mechanisms governing community structure may change during resource pulses. Overall, we suggest that social interactions between chickadees and nuthatches are dynamic with high complexity and flexibility to major ecological changes. Future work that examines the fitness consequences of temporal variation in community dynamics and resiliency could help to reveal evolutionary mechanisms by which these species co-exist.

**Supplementary Information:**

The online version contains supplementary material available at 10.1038/s41598-025-93109-y.

## Introduction

Assessing the resiliency of social networks and predicting the responses of animal social systems to ecological change are emerging information needs in ecology^1–3^. Many ecological and evolutionary processes depend on social hierarchies, which are shaped by their surrounding biotic and abiotic conditions^4–6^. Environmental change can destabilize existing social structures, leading to variable responses in social behaviour^7^. Resource pulses – brief, occasional, and intense events of high resource availability - may permeate through multiple levels of terrestrial and aquatic food webs to influence social networks^8–10^. Examining thresholds of ecological change that lead to shifts in social network structure can help to reveal links between social and ecological resilience^11^. Studies of the effects of resource pulses on the variation in species interactions may help to fill key gaps in the functional dynamics of community ecology^12^. In particular, there have been recent calls for more information on social networks and dominance hierarchies among multi-species groups^3^. Variation in foraging niches within and among species has been shown to affect variation in social network structure^13,14^, a relationship demonstrated previously with experimentally pulsed resources^10^ but the opportunity to explore how natural resource pulses affect social interactions across species is rare.

Forest bird communities are structured around complex social networks according to foraging and nesting guilds, respectively^15–18^. In temperate regions, tits/chickadees (Paridae), nuthatches (Sittidae), and woodpeckers (Picidae) are among the bird species groups most frequently studied with respect to social networks due to their co-occurrence in both foraging and nesting guilds^15,16^. Chickadees show a great diversity of foraging behaviours as insect generalists, and while all species require tree cavities for nesting, some species are considered secondary cavity-nesters as they do not typically excavate their cavities^19,20^. Nuthatches are bark beetle-foraging specialists that are facultative excavators (i.e., they exhibit flexibility as they may excavate a new nesting cavity or use an existing cavity and are hereafter considered excavators)^19,21^. These differences in partitioning of the foraging and nesting niches (i.e., resource specialization) within the cavity-nesting community are hypothesized to allow for co-occurrence driving patterns in year-round coexistence^16,22^. Species considered to be resource specialists are often stronger competitors due to their stronger reliance of some shared but limited resource^22^; thus, cavity limitation can drive competitive interactions among secondary tree cavity-nesters^23^, and bark beetle limitation could drive competitive interactions among bark beetle specialists. Community ecology lacks studies that experimentally test whether patterns in co-occurrence indicate ecological interactions driving community co-existence^24^. A dual resource pulse of both bark beetles and nesting cavities offers a unique opportunity to explore coexistence among species that vary in their specialization to each resource.

Despite the niche differentiation nuthatches are found to be consistently dominant behaviourally over chickadees^19,25^ with some exceptions reported^26^. Both species exhibit aggressive behaviour towards conspecific and heterospecific individuals that threaten access to mates or food, which includes dominant individuals moving toward and supplanting their adversaries, and aggressive calls and displays that are unique to each species^26,27^. Socially, chickadees give alarm calls when predators are present, which nuthatches recognize and use to avoid predators^28,29^. In addition, chickadees use nesting cavities excavated by nuthatches, and their breeding populations show lagged positive functional and numerical responses to nuthatch densities^30–32^. Due to their shared reliance on common resources and year-round co-occurrences including overlapping breeding territories and in rare instances nesting in the same tree cavity in the same year^33^, chickadees and nuthatches provide an excellent model in which to examine how a dual resource pulse in insect food and tree cavities affects their social structure.

**Fig. 1:**
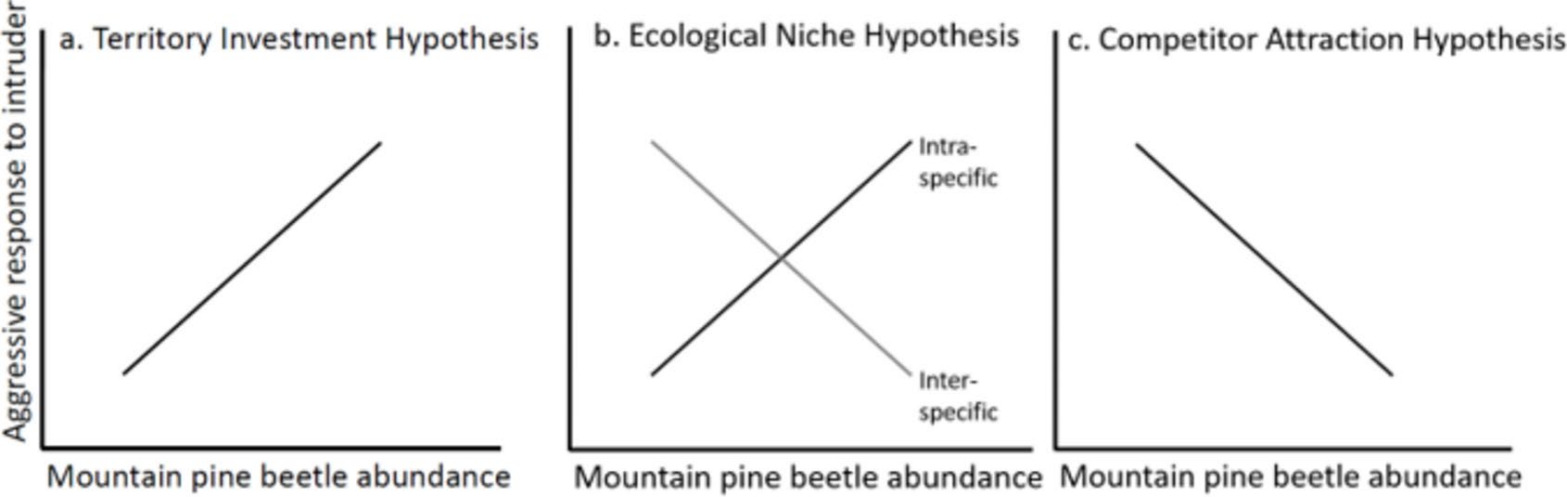
Predicted changes in aggressive responses of mountain chickadees and red-breasted nuthatches to conspecific and heterospecific intruders with a dual pulse of mountain pine beetle abundance and nest cavities. Competitive interactions increase with resource availability through greater incentive to defend high quality resources and obtain greater potential reproductive benefits (Territory Investment Hypothesis; TIH; (**a**); ^14,56,58^). Increased intra-specific competition but decreased inter-specific competition may result from expanded niche breadths that occur with pulsed resources, and inter-specific dominance hierarchies of resource specialists over generalists are relaxed, (Ecological Niche Hypothesis; ENH; (**b**); ^6,22,76,77^). Finally, both intra- and inter-specific competition may decrease if individuals use the presence of conspecifics^78^ or heterospecifics with similar habitat requirements^79^ to assess territory quality in unpredictable environments (The Competitor Attraction Hypothesis; CAH; (**c**)).

Previously, we reported that annual increases in chickadee and nuthatch populations were correlated with two food resource pulses of western spruce budworm (*Choristoneura occidentalis*) and mountain pine (bark) beetle (*Dendroctonus ponderosae*), from 2004 to 2006 in British Columbia^31,34^. In addition, chickadees used more cavities excavated by nuthatches, as nuthatches had excavated proportionately more new nest cavities in response to the beetle outbreak^31,32,34,35^. Here we used an experimental approach to infer the nature and strength of interactions within a population and between coexisting species^36,37^ to explore how changes in social dynamics may have contributed to the observed plasticity in nesting behaviour that led to increased reproductive output for both species^32,35^. Specifically, we examined temporal variation in intra- and inter-specific interactions of mountain chickadee and red-breasted nuthatch, two small cavity-nesting competitors that show plasticity in their nest excavation behaviours and foraging strategies (on bark beetles). The identity of intruders often plays an important role in territoriality, particularly with respect to their relationship to the territory occupant such that neighbours and non-neighbours, or strangers, can elicit different responses depending on various factors such as environmental conditions and life history characteristics^38–41^. For example, individuals that breed at lower densities and/or with sparse food resources can show higher aggression to territorial intrusions by novel intruders (strangers; dear enemy effect) relative to familiar intruders (neighbours), and individuals breeding at higher densities and/or with abundant food can show higher aggression to neighbours (nasty neighbour effect^42^). We simulated territorial intrusions of conspecifics and heterospecifics to determine how the bark beetle outbreak and other environmental factors influenced the responses of chickadees and nuthatches to territorial invaders. We evaluated our results with respect to three competing hypotheses suggested to influence species interactions (Fig. 1). Aggressive responses are predicted to: (a) increase with increasing beetle abundance as the energy and potential reproductive benefits for individuals to defend high quality sites increases (Territory Investment Hypothesis), (b) decrease towards heterospecifics but increase towards conspecifics, by expanding niche breadth, and reducing inter-specific dominance of resource specialists over generalists (Ecological Niche Hypothesis), or; (c) decrease towards both species as individuals use the presence of conspecifics or heterospecifics with similar habitat requirements to assess territory quality (The Competitor Attraction Hypothesis). For example, if resource pulses impact these social networks according to the Territory Investment Hypothesis in which high quality resources are defended more aggressively, then intra- and inter-specific aggression among chickadees and nuthatches would both increase with higher beetle availability. The Ecological Niche Hypothesis (intra-specific mate competition is higher when inter-specific competition for the resources that most limit both species is low^22^) predicts that intra-specific aggression would increase with increasing beetle abundance, and inter-specific dominance of nuthatches over chickadees would be reduced. Alternatively, the Competitor Attraction Hypothesis in which tolerance of competitors increases as food resources are more abundant, predicts that both intra- and inter-specific aggression would decline with increasing beetle abundance (Fig. 1). We experimentally simulated intra- and inter-specific territorial intrusions of two cavity-nesting species across breeding territories with a range in availability of nesting cavities and food to test whether resource pulses correlated with changes in inter-specific interactions.

## Results

**Fig. 2:**
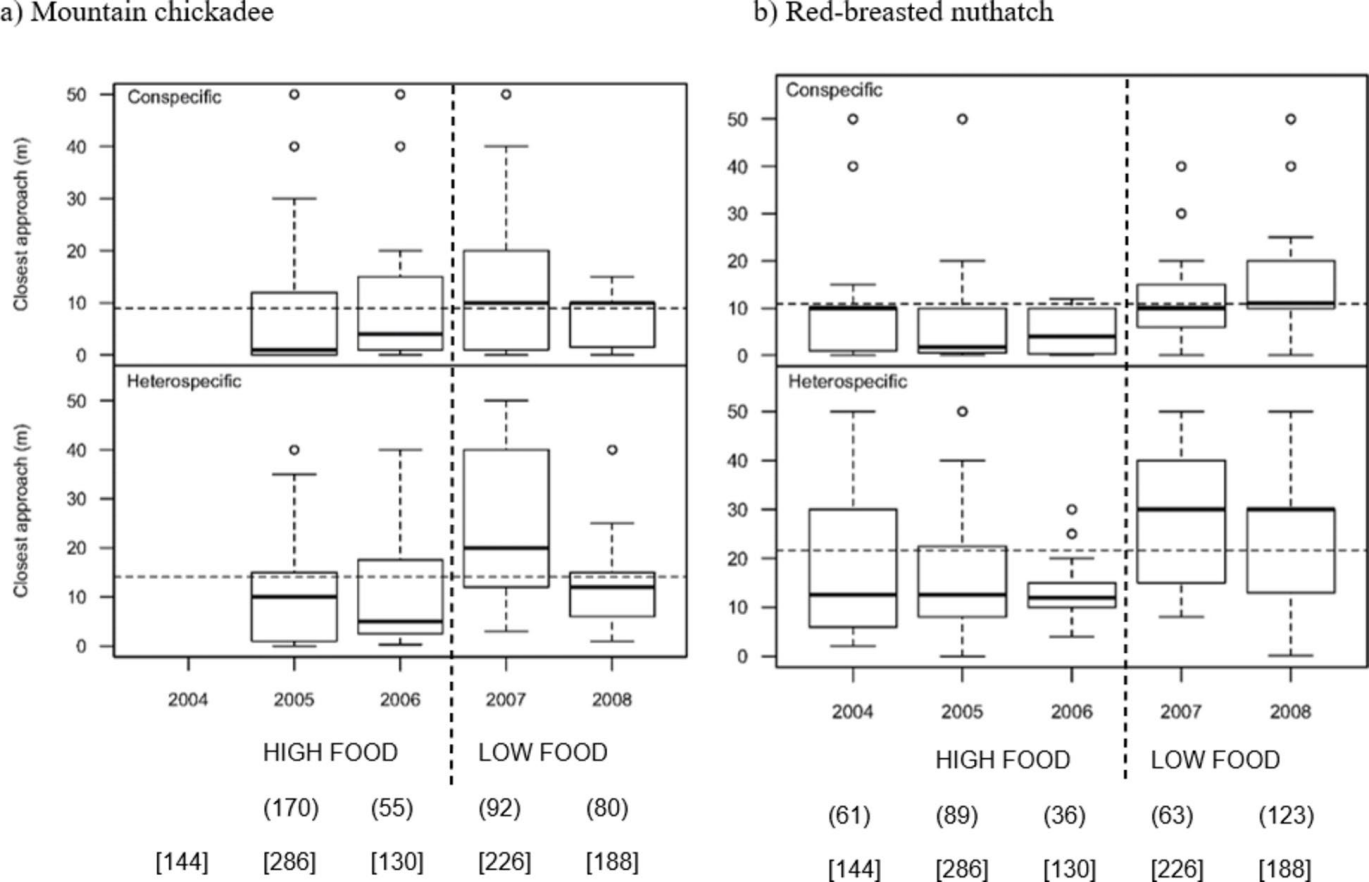
Annual variation in median closest distance approached by (**a**) mountain chickadees, and (**b**) red-breasted nuthatches to conspecific and heterospecific intruders, with overall means indicated by horizontal dashed lines, across 974 intrusions simulated at 25 sites in interior British Columbia. Vertical dashed lines indicate observed annual differences in insect prey availability (mountain pine beetle and western spruce budworm) at the study area, and summarized as “HIGH FOOD” and “LOW FOOD” availability^31,35^. Round brackets indicate the total number of responses elicited, and square brackets show the total number of intruder simulations in each year, boxes show data within the 75th percentiles, whiskers show the maximum and minimum within the 90th percentiles, and circles show outliers.

We simulated 974 territorial intrusions across 25 sites during 2004–2008. We detected 397 responses (i.e., at least one adult detected < 50 m from intruder, 181 of these responses (45.6%) had more than one adult detected) from the bark beetle generalist, mountain chickadee. We detected 372 responses from red-breasted nuthatch (bark beetle specialist), of which 118 responses (31.7%) occurred in the presence of more than one adult. In 95 cases (23.9%) for chickadees and in 30 cases (8.1%) for nuthatches, respondents struck the intruder, knocking it to the ground, and then repeatedly attacked the intruder until the observer removed it. Overall, we found very strong evidence (*p* < 0.001) that both species approached intruders closer when intrusions were simulated on active nest plots (~ 1 m from nest tree; ACN, ANN) compared to inactive territories. We found evidence that both species responded strongly (chickadee) and very strongly (nuthatch) to conspecific intruders (mean closest distance approached by chickadee, 9.1 m ± 0.77 SE; nuthatch, 11 m ± 0.80 SE). However, chickadees approached heterospecific intruders much closer than did nuthatches (14 m ± 1.1 SE, and 21 m ± 1.2 SE, respectively; Fig. 2). During the beetle outbreak (2004–2006), both species approached closer than the overall mean, and approached heterospecifics at least as close as the overall mean approach to conspecifics. After the beetle outbreak (2007–2008) neither species approached the intruder as close as those respondents measured during the peak of the beetle outbreak.

### Mountain chickadee

**Fig. 3:**
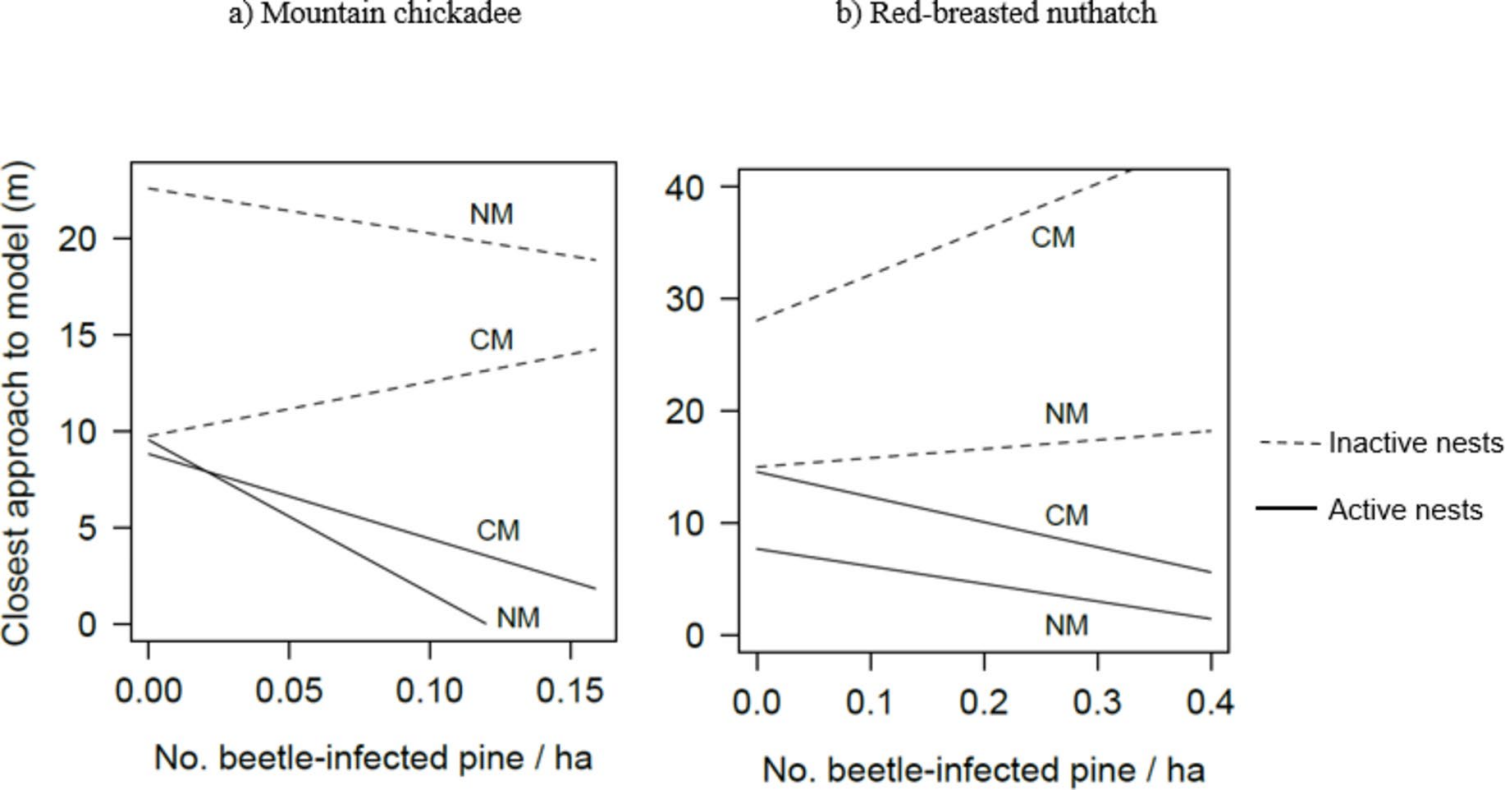
Mean closest distance approached by respondent (**a**) mountain chickadees and (**b**) red-breasted nuthatches, to chickadee (CM) and nuthatch (NM) intruders across sites and years with increasing levels of beetle abundance, at active (solid lines) and inactive (dashed lines) nests occupied by conspecifics, in interior British Columbia, during 2004–2008 (2004 excluded in Fig. 3b). Lines were generated from the linear models: Distance ~ Beetle (Be) + Plot type (PT) + Be PT (see Tables 1 and 2), and responses were noted from any individual respondents observed within a 50 m radius of the simulated intrusion.

On average, chickadees approached all intruders closest at intrusions simulated in the immediate proximity of (~ 1 m from) active chickadee nests (Table 1) and conspecific intruders closer than heterospecific intruders, (*β*_*NM*_ = 6.4 ± 1.5 SE), which provided strong evidence for the Ecological Niche Hypothesis. However, during high beetle abundance (2005 and 2006), chickadees approached heterospecific intruders closer than conspecific intruders at active chickadee nests (*β*_*BexACN*_= -140 ± 70 SE; Fig. 3), providing weak evidence for the Territory Investment Hypothesis (Table 1). Chickadees showed a weak nasty neighbour effect towards conspecifics (*β* = -8.7 ± 4.8 SE), approaching intruders closer with increasing chickadee densities, and we found moderate evidence for a dear enemy effect towards nuthatches, approaching less closely with increasing nuthatch densities (*β* = 25 ± 12 SE).

**Table 1:** Mountain Chickadee responses to 830 experimental intrusions of conspecific and nuthatch intruders across 112 plots at 25 sites in interior British Columbia, from 2005–2008.

Fixed effect	Estimate	SE	DF	t-value	*p*-value
Model Intercept	15	2.6	211	5.9	< 0.001
**Nuthatch model (NM; relative to CM)**	6.4	1.5	211	4.2	**< 0.002**
**Beetle-infected pine density (Be)**	130	70	211	1.8	**0.076**
**Chickadee density**	−8.7	4.8	211	−1.8	**0.073**
**Nuthatch density**	25	12	211	2.1	**0.041**
**PT: Active chickadee nest (ACN)**	−9	2.3	211	−3.8	**0.0002**
**Be x ACN interaction**	−140	70	211	−2	**0.052**

Parameter estimates (Estimate), standard errors (SE), and degrees of freedom (DF) of the final mixture distribution (normal and binomial), linear mixed-effects model, generated using penalized quasi-likelihood of closest distance approached by mountain chickadees to simulated intruders of red-breasted nuthatch (NM) and mountain chickadee (CM) at various locations (plots) within territories compared to inactive territories, with spatial and temporal variation in beetle abundance (beetle-infected pine densities; Be), and nuthatch densities. Estimates were generated from the final model, Closest distance ~ Intruder + Beetle density (Be) + Chickadee density + Nuthatch density + Plot type (PT) + Be × PT; random effects = Site / Plot ID, with continuous autoregressive correlation within Plot ID. Evidence to support the hypotheses that fixed effect variables correlated with distance approached to intruders was considered absent (*p* > 0.1), weak (0.1 > *p* > 0.05), moderate (0.05 > *p* > 0.01), strong (0.01 > *p* > 0.001), or very strong (0.001 > *p* > 0.0001), in bold. Although the responses in five classes of plot type were compared to those in random plots in inactive territories, only classes where some evidence of correlation with fixed effects was found are listed.

**Table 2:** Red-breasted nuthatch responses to 974 experimental intrusions of conspecific and Chickadee territorial intruders across 114 plots at 25 sites in interior British Columbia, from 2004–2008.

Fixed Effect	Estimate	SE	DF	t-value	*p*-value
(Intercept)	24	2.4	157	10	< 0.001
**Nuthatch model (relative to CM)**	−12	1.5	157	−8.4	**< 0.001**
Beetle-infected pine density (Be)	16	18	157	0.89	0.37
**Nuthatch density**	22	9.3	157	2.3	**0.021**
PT: Active nuthatch territory (ANT)	−4.7	3.1	157	−1.5	0.13
**PT: Active nuthatch nest (ANN)**	−12	2.4	157	−4.9	**< 0.001**
**Be x ANT interaction**	−44	26	157	−1.7	**0.098**
**Be x ANN interaction**	−37	21	157	−1.8	**0.08**

Parameter estimates (Estimate), standard errors (SE), and degrees of freedom (DF) of the final mixture distribution (normal and binomial), linear mixed-effects model generated using penalized quasi-likelihood for closest distance approached by red-breasted nuthatches to simulated intruders of red-breasted nuthatch (NM) and mountain chickadee (CM), at various locations (plots) within active territories compared to inactive territories with spatial and temporal variation in beetle abundance (beetle-infected pine densities; Be), and Nuthatch densities. Estimates were generated from the final model, Closest distance ~ Intruder + Beetle (Be) + Nuthatch density + Plot type (PT) + Be × PT; random effects = Site/Plot ID, with continuous autoregressive correlation within Plot ID. Evidence to support the hypotheses that fixed effect variables correlated with distance approached to intruders was considered absent (*p* > 0.1), weak (0.1 > *p* > 0.05), moderate (0.05 > *p* > 0.01), strong (0.01 > *p* > 0.001), or very strong (0.001 > *p* > 0.0001), in bold. Although responses in five classes of plot type were compared to those in inactive territories, where some evidence of correlation with fixed effects was found are listed.

### Red-breasted nuthatch

Very strongly consistent with the Ecological Niche Hypothesis, the final model describing variation in the closest distance approached to intruders predicted that nuthatches approached conspecifics closer than to chickadees (*β*_*NM*_ = -12 ± 1.5 SE; Table 2). Nuthatches showed a strong dear enemy effect towards conspecifics, approaching intruders less closely with increasing nuthatch densities (*β* = 22 ± 9.3 SE). The relationship between response distance and beetle abundance depended upon whether the nest plot was active or inactive. Weakly consistent with the Territory Investment Hypothesis, nuthatches approached intruders closer with increasing beetle abundance in active nuthatch territories (both at active nests and ~ 50 m from the nest; *β*_*BexANN*_ = -37 ± 21 SE, *β*_*BexANT*_ = -44 ± 26 SE, respectively), compared to inactive territories.

## Discussion

We found support for both the Ecological Niche and Territory Investment Hypotheses at different spatial and temporal scales. Chickadees showed greater annual variability in their responses and were more aggressive than nuthatches across all territories, sites, and years, consistent with the Ecological Niche Hypothesis. Nuthatches approached conspecific intruders twice as close as heterospecific intruders, overall, and approached intruders closest at both active nests and at suitable nest trees near active nuthatch nests (active nuthatch territories) relative to inactive territories. Both species were more aggressive to nuthatch intruders at active nest territories at sites and in years with increasing beetle abundance, suggesting that: (1) the more efficient beetle-foragers (nuthatches) may pose a greater threat to territory intrusion during beetle outbreaks, and (2) individuals increased their investment in territory defence with increases in food availability (Territory Investment Hypothesis).

As with any experimental study design, our results are limited to those variables examined. Our primary response measure was distance approached to the intruder, but respondents exhibited a range of behaviours that may have differed disproportionately with respect to territorial aggression. Furthermore, we did not explore the speed at which territorial aggression escalated in response to intruders, even though responses can be expressed in many ways, such as speed and intensity of initial reaction, duration of response, and the level of escalation^43^. Behavioural responses are likely influenced by other factors affecting communication in animals such as demographic features, sex differences, visual, auditory and other sensory cues such as vibrational signals^44,45^, and the presence of other species including humans^3^. Our experimental approach of presenting a model and broadcasting auditory signals, although a standard approach to measuring behavioural responses might have influenced the ways in which these species normally interact with one another within this spatial and temporal context. For example, we found weak evidence to support the idea that the order in which we simulated intrusions of these two species influenced some responses. On average, nuthatches approached simulated intruders somewhat closer when conspecific intruders were presented before chickadees (*β* = -2.90 ± 1.68 SE, *p* = 0.09) but chickadees showed no difference in the distance approached to intruders when presented first with conspecific vs. nuthatch intruders (*β* = -1.00 ± 1.45 SE, *p* = 0.49). Therefore, for nuthatches we were unable to rule out the possibility that order had other indirect effects on their responses.

### Ecological niche hypothesis

We found the strongest evidence for the Ecological Niche Hypothesis. In earlier work, we found that shifts in habitat and cavity preferences led to increases in reproductive output for both species, and hypothesized that increases in food availability could reduce territorial disputes^31,32,35^. Our finding that responses to heterospecifics were stronger in chickadees than nuthatches (24% of responses by chickadees led to an attack vs. 8% by nuthatches) is similar to another study approximately 300 km north of our study area, where mountain chickadees showed similar responses to conspecific intruders and to the behaviourally dominant heterospecific intruder, black-capped chickadee (*Poecile atricapillus*), another small cavity excavator^46^. Despite previous observations and generally accepted knowledge among titmice experts that nuthatches are behaviourally dominant over chickadees^19,25,47^, others occasionally have found chickadees or titmice to be socially dominant over nuthatches in foraging flocks^26^. Dhondt^48,49^ has even suggested that greater inter-specific (relative to intra-specific) competition during the breeding season is an evolutionary balancing mechanism promoting year-round coexistence in tits. As with the Dhondt studies, chickadees in our study showed unexpectedly higher aggression to their dominant heterospecific competitor during the breeding season. Notably, most studies of dominance hierarchies in titmice are conducted outside the breeding season and where patterns in social dominance are inferred mainly from access to food^20,26,50^, rather than breeding resource supply.

At the nesting guild, nest cavity resource specialists, or those who exclusively reuse old nest-sites can behaviourally dominate generalists (in this case, we refer to those species capable of excavating their own cavity^23^). For example, the secondary cavity-nester, European starling (*Sturnus vulgaris*), can usurp cavities from would-be woodpecker competitors by initiating their nests in contested cavities before woodpeckers^51,52^. Thus, starlings are often considered a behaviourally dominant species in the nest web, and can have negative impacts on breeding success of rivals through interference competition^23,51–53^. Mountain chickadees initiated nests ~ 15d earlier than nuthatches, suggesting that earlier nesting facilitated their dominance over nuthatches^32,35^. In the first year of our study we reported an instance of nest sharing between mountain chickadee and red-breasted nuthatch, with a female nuthatch displacing a chickadee pair during the nestling stage and successfully fledging both nuthatch and chickadee young from the nest^33^ suggesting that chickadees have reproductive incentive to defend their nests against the more dominant heterospecific to prevent nest usurpation. Thus, our findings support the Ecological Niche Hypothesis and suggest that the dominance hierarchy between chickadees and nuthatches varies and can be reversed on breeding territories during resource pulses.

Chickadees and nuthatches are the only cavity-nesting passerines to remain in boreal and hemi-boreal temperate regions during winter and due to their year-round co-occurrence and niche overlap probably have highly complex social networks and other important social dynamics not tested here. Model systems where rank can be manipulated are suggested to be extremely useful for testing hypotheses about social dominance dynamics^2^. We suggest that future work should explore the generality of our findings that chickadees that are subordinate in foraging groups can become dominant to excavating nuthatches on breeding territories during resource fluctuations.

### Competitor attraction hypothesis

Since both species approached nuthatch intruders closest at active nest territories, it is possible that both species used nuthatches as cues in territory establishment and the distance approached to nuthatch intruders represented territory prospecting rather than defence. However, we found a strongly negative correlation between the elicited behaviour of attacking the model and distance approached to the model (in the simple linear model, Closest distance ~ Attacker vs. non-attacker, for chickadees, $\hat{\beta }_{{\text{A}}}$ = -13 m ±1.8 SE, *t* = -6.8, *p* < 0.01, and nuthatches, $\hat{\beta }_{{\text{A}}}$ = -16 m ± 3.3 SE, *t* = -4.7, *p* < 0.01) suggesting that the closest distance approached indeed represented an aggressive response rather than a passive, exploratory response. Further, if individuals were using the presence of nuthatches to assess new territories, we would expect a closer approach to nuthatch intruders at all territories. Yet, both species approached similar distances to both nuthatch and chickadee intruders at heterospecific territories (*p* > 0.1; Interaction effect of heterospecific nest plot and nuthatch intruder for both species), and the increased responses to nuthatch intruders were only observed at conspecific territories (Tables 3 and 1), indicating territorial defence responses. Since distance approached was correlated with an aggressive response, and both species approached intruders closer with increasing beetle abundance, neither intra- nor inter-specific aggression was reduced with increases in food availability, as was predicted under the competitor attraction hypothesis. Thus, we were able to reject both the conspecific and heterospecific attraction hypotheses for both chickadees and nuthatches.

**Table 3:** We simulated 974 territorial intrusions according to six plot types (distance to nest tree, species using the cavity, and active or inactive nest status) assigned by mean distance to nest tree (m) observed to be occupied by a chickadee or nuthatch breeding pair (Territory holder species) in the same year as the experiment or in a previous year (active/inactive nest status, respectively).

Plot type	Distance (m)	Territory holder species	Nest status	Simulated intrusions
Active chickadee nest (ACN)	1	Chickadee	Active	124 (c) 116 (n)
Active nuthatch nest (ANN)	1	Nuthatch	Active	63 (c) 66 (n)
Inactive nest	1	Either chickadee or nuthatch	Inactive	113 (c) 119 (n)
Active chickadee territory (ACT)	50	Chickadee	Active	124 (c) 116 (n)
Active nuthatch territory (ANT)	50	Nuthatch	Active	63 (c) 66 (n)
Inactive territory	50	Neither	Inactive	105 (c) 104 (n)

To test the additional hypothesis that proximity to nest affects territoriality we simulated intrusions located in a random direction ~ 50 m from active and inactive nests at (~ 1 m from) any available tree suitable for excavation or nesting (aspen tree ≥ 15 cm DBH) but was never to our knowledge used by either species (i.e., Inactive territory). For each plot type we simulated intrusions of taxidermically-prepared specimens of mountain chickadee (c) and red-breasted nuthatch (n) paired with song and call recordings at 25 study sites in interior British Columbia, Canada, from 2004–2008. We compared the intra- and inter-specific responses measured for each species at active and inactive territories to those at inactive territories.

### Territory investment hypothesis

Both species showed an increasingly aggressive response to all intruders at active nest trees and nearby suitable nest trees within territories, with increasing beetle abundance, providing weak to moderate evidence that investment in territorial defence increased with the beetle outbreak (or more accurately, investment declined with reductions in beetle availability at the temporal scale measured in this study). Food-supplemented song sparrows (*Melospiza melodia*) in the northeastern United States were more aggressive to territorial intruders than non-supplemented birds, particularly in rural environments where birds were less aggressive overall and resources were potentially more limited relative to those in urban areas^54^. High levels of aggression and territorial defence often require elevated energy expenditures, but territories containing ample resources required for increased defence may also provide greater reproductive benefits as the energy spent on territorial defence is readily recouped^55^. In cavity-nesting prothonotary warblers (*Protonotaria citrea*), pairs occupying higher quality territories produced more fledglings and competitively excluded other pairs from territories^56^. Mountain pine beetles provide a year-round food source from late summer, when adult beetles lay eggs beneath the bark, to the following summer, when larval development is completed^57^. As both chickadees and nuthatches are winter residents, the beetle outbreak likely increased the energy reserves of individuals over winter and in spring before the breeding season. During the beetle outbreak, chickadees laid earlier and larger clutches, and had a higher probability of fledging nestlings, and nuthatches laid larger clutches later in the breeding season compared to before the outbreak^32,35^. Thus, it is likely that territories with high beetle abundance provided more food resulting in earlier nesting and higher fecundity, leading to an increase in territoriality before and during territory establishment in both species.

Our result that chickadees approached intruders closer at sites and in years with increasing chickadee densities, supported (weakly; *p* = 0.073) the nasty neighbour effect that neighbouring conspecifics pose a greater threat than strangers due to higher potential for competition over resources and access to mates leading to positive density-dependent aggression^42,58^. Contrary to their response to conspecific densities, however, chickadees showed lower aggression with rising nuthatch densities (moderate evidence; *p* = 0.041). Nuthatches also showed lower aggression with rising nuthatch densities (moderate evidence; *p* = 0.021), and both species were most aggressive to nuthatch intruders in the years of lowest nuthatch population densities (2005–2006; Fig. 2^34^). Breeding populations of both chickadees and nuthatches showed high annual variability, and the result that aggression toward nuthatch intruders was higher when nuthatch densities were lowest and lower when densities were highest suggests that unfamiliar nuthatches (strangers) may pose a greater threat than neighbouring nuthatches. We provided moderate support for the ‘dear enemy’ effect that both species were less aggressive towards familiar nuthatches (i.e., a known threat), and avoided the costs associated with repetitive territorial disputes. The result that chickadees could switch between the nasty neighbour effect to conspecifics and the dear enemy effect to nuthatches indicates behavioural plasticity in territoriality, a pattern shown to occur across the breeding season for dusky warblers (*Phylloscopus fuscatus*) in Hebei, China^59^. We provided some evidence to support both the nasty neighbour (weak) and dear enemy (moderate) effects in chickadees and nuthatches and suggest that future work examine how plasticity in territoriality across the year may contribute to the complex social networks and co-existence in this species group.

## Conclusion

We found that a cavity specialist and foraging generalist chickadee dominated nuthatch (a beetle specialist and cavity generalist), suggesting that inter-specific dominance hierarchies observed at foraging guilds can be reversed in the breeding season during resource pulses. Increases in intra- and inter-specific competition with increases in beetle abundance suggest that behavioural mechanisms governing community structure may change dramatically during resource pulses that increase the disparity in territory quality. Our observation that aggressive responses declined following the beetle outbreak, returning to the typically reported relationships among chickadees and nuthatches suggests that these social networks exhibit resiliency following resource pulses. Future work that examines the variation in responses to conspecifics and heterospecifics and their associated fitness consequences within the context of resource limitation might reveal how social interactions may regulate year-round coexistence among these species groups^3,49^.

## Methods

### Study area

We studied behaviour, fecundity, and habitat characteristics of cavity-nesting birds in 25 mixed coniferous-deciduous forest stands on the lands of the Tŝilhqot’in, Secwépemc, and Southern Dakelh Peoples, an area surrounding Williams Lake, British Columbia, Canada (51°52’N, 122°21’W), from 2004 to 2008. The predominant coniferous trees were Douglas-fir (*Pseudotsuga menziesii* var. *glauca*), lodgepole pine (*Pinus contorta* var. *latifolia*; hereafter, pine), and white and Engelmann hybrid spruce (*Picea glauca x engelmannii*^60^). The predominant broadleaf tree was trembling aspen (*Populus tremuloides*). Study sites ranged from 15 to 32 ha (one 7-ha site) in size and varied in composition from continuous forest to five sites that comprised a series of ‘forest groves’ (0.2 to 5 ha) within a grassland matrix.

### Study system

The small tree cavity-nesting community comprised two species of insectivorous excavators and one secondary cavity-nester^61^. As many cavity excavators are also insectivores, large-scale insect outbreaks can lead to dual pulses in food and nest sites, potentially influencing the competitive interactions among tree cavity-dependent insectivores. Mountain pine beetle is a native bark-boring insect that feeds on the phloem of pine trees and is a common disturbance agent in temperate forests that undergoes occasional patchy outbreaks in western North American forests^62^. Recent mountain pine beetle outbreaks in British Columbia increased year-round food availability and, subsequently, population densities of many insectivorous birds, including many excavators^60,62^. Mountain chickadee a secondary cavity-nester (cavity specialist) that relies on excavators and natural decay processes for nest cavities, is primarily a foliage gleaner but can switch to other foraging substrates depending on forest insect abundance^20^. Red-breasted nuthatch is a facultative tree cavity-nesting excavator and is primarily a bark forager^50^.

The mountain pine beetle outbreak led to increases in population densities of both chickadees and nuthatches at the study area^31,34^. Red-breasted nuthatch shifted nest site preference from areas of high nest site availability to those of high mountain pine beetle availability, where they excavated a greater proportion of nests^63,64^. Mountain chickadee populations showed a one-year lag in increases following increased nuthatch populations and used a greater proportion of smaller, safer nuthatch cavities following the beetle outbreak, suggesting that chickadee populations benefited from the higher densities of nuthatches^31,32^. However, the beetle outbreak also led to increased densities of American red squirrel (*Tamiasciurus hudsonicus*, a common nest predator for chickadees and nuthatches^65^. Because increased predator presence can lead to reduced parental activity around the nest resulting in reduced fecundity^66^, high squirrel densities may diminish territory quality and impede territory defence strategies. Such changes in territory characteristics could lead to increases or decreases in agonistic behaviour within and between species.

We located nest trees of chickadees and nuthatches by checking all nesting cavities in trees used by other cavity-nesters in previous years (1995–2007) with a camera monitoring system on an extendable pole and by following individuals to their nests. We considered nests to be active if we found eggs or chicks in a cavity and monitored all nests until fledging or failure. We color-banded 115 adult mountain chickadees and 65 red-breasted nuthatches on breeding territories by capturing birds with mist nets or by covering the nest entrance with a dip net when the adult was inside. Birds were banded using a unique combination of three plastic color bands and one numbered aluminium band, and released at the capture site within 10 min. Additional study area and nest monitoring details are given in^30,61^.

All experiments were performed in accordance with the Animal Care Committees of the University of British Columbia and Environment and Climate Change Canada, under triennially renewed permits from the University of British Columbia Animal Care Protocol and Environment and Climate Change Canada’s scientific permit and banding permit numbers A07-0130 and 10,365, respectively.

### Territorial intrusions

We used song playbacks with intruder simulations to investigate interference competition within and between species^36^ during territory establishment and before eggs were laid until after chicks fledged. To examine territorial responses of chickadees and nuthatches, we simulated conspecific and heterospecific intrusions (two trials) at six treatment types that represented two temporal and two spatial scales with respect to nesting (Table 3). We were unable to access all nest cavities using a ladder and therefore couldn’t simulate the intrusions directly next to nest cavities. To ensure that the height of the models above ground was consistent across all individuals and years we simulated intrusions 1 m above ground within ~ 1 m of trees at active nests of chickadees and nuthatches, and at nest cavities that were active in a previous year by either species (inactive nests). To explore the additional hypothesis that proximity to nest affects territoriality we simulated intrusions located in a random direction ~ 50 m from active and inactive nests at ~ 1 m from any available tree suitable for excavation or nesting (aspen tree *≥* 15 cm DBH) but was never to our knowledge used by either species.

To assess the level of territorial aggression of chickadees and nuthatches we elicited responses by simulating intrusions at one to three sites per day, and at two to three treatment types (active nest, inactive nest, and inactive territory), depending on the breeding status of territory holders. To avoid the possibility of eliciting responses from neighbouring pairs on active nests, we simulated intrusions only at non-overlapping territories (usually where active nests were > 200 m apart) and ensured that all presentations were at least 50 m apart. Throughout the breeding season, if an inactive territory where we simulated intrusions became active in an area within 200 m of another active nest, we stopped simulating intrusions at both nests. The two species exhibit unique behaviours with respect to aggressive calls and displays, but both species exhibit the same behaviour of moving towards and supplanting intruders, which allows researchers to compare inter-specific responses^27,46,47^. Therefore, we used the closest distance (in m) respondents approached the intruder during each simulated intrusion by measuring the distance between the simulated intruder and the ground (1 m) as a reference distance for those approaches within 5 m of the intruder and a laser rangefinder for approaches > 5 m away. Although it was not possible to record data blind because our study involved focal animals in the field, we simulated intrusions of both species for each trial and presented each intruder species in a random order.

In 2004, we used song recordings from the second edition (1992) of the Peterson Field Guide audio compact disc from Cornell Lab of Ornithology (non-local birds), and during 2005–2008, we used recordings of songs of local chickadees and nuthatches collected ~ 20 km outside the study area (local birds). We used Sound Studio software^67^ to digitally manipulate song recordings so that each song playback, lasting 150 s, contained 1–3 exemplars that were repeated consistently and separated with 3–6 s of silence 20–28 times for chickadee and 90–106 times for nuthatch. For each species we alternated randomly between 2 song playbacks that differed in the number of exemplars and individuals originally recorded in the field. We transferred the recordings onto a Panasonic portable media player (Model no. SL-SX280) and broadcast over portable Sony mini audio speakers (Model no. SRS-P7) at an amplitude that approximated the natural amplitude of songs, evaluated by ear, estimated between 65 and 70 dB. A taxidermic model specimen (intruder) of the appropriate species was placed on a wire stand ~ 1 m above the speakers and presented with the appropriate song type for each trial, with a 5-min period of silence following each intruder species presented. For each respondent, observers sat on the ground *≥* 15 m away from the model and speakers for the duration of the simulation, where we recorded the species, individual (if colour banded), sex (where possible), time of day, behaviour (whether the respondent called, call type, sang, swooped, attacked, etc.), and the closest distance (m) that they approached to the model intruder. In cases where the respondent attacked the intruder, and aggression levels remained high, we stopped the recording, removed the model, and waited ≥ 10 min to start the presentation of the next intruder species until the aggressive individual returned to displaying the behaviour observed before the first intruder was presented. We used song recordings collected from individuals in different geographic regions across North America between 2004 (non-local birds) and similar regions for the remainder of the study, 2005–2008 (local birds). Since territorial behaviour can be strongly influenced by geographic differences in populations^39,40,43^, we compared the annual mean distances between the respondent and the simulated intruder to assess whether annual response patterns could be attributed to differences in recordings. In 2004, simulated intrusions using the non-local birds elicited a weaker response by mountain chickadees compared to recordings of local birds in all other years except 2007 when there was no difference in responses (mean distance approached to conspecific intruder in 2004 was greater than those in 2005, 2006, and 2008; F_4,251_=5.17, *p* < 0.01). As a result, we excluded 2004 in analyses examining responses of mountain chickadees. The responses by nuthatches to the conspecific intruders did not differ between 2004 and the other years, except in 2008 when nuthatches unexpectedly responded weaker (proximity to intruder was greater) relative to the non-local bird recordings (F_4,226_=5.34, *p* < 0.01). Since the responses by nuthatches seemed largely unaffected by differences in geographic location of recordings (non-local vs. local birds), and in 2008, showed the opposite trend of having non-local birds eliciting a stronger response than local birds, 2004 was included in all nuthatch analyses. Where intrusions were simulated at active nest territories, we inspected the nest cavity using a pole-mounted video camera, and recorded fecundity characteristics (number of eggs or nestlings) and the stage of the nest to determine breeding status (pre-nest, egg-laying, incubating, chick-rearing).

### Population densities

To determine how changes in population densities of conspecifics and heterospecifics (including predators) associated with the beetle outbreak in the study area^31,34^ influenced territorial behaviours, we conducted point count surveys to estimate population densities per ha of mountain chickadee, red-breasted nuthatch, and American red squirrel at 25 sites, during 2004–2008. Point count stations were spaced within a 100 m square grid ≥ 50 m from a grassland or wetland edge (one station ha^− 1^) in continuous forest sites, and at least 100 m apart in forest groves. From 0500 to 0930 h, we recorded for 6 min the species, and number of individual birds and squirrels detected within 50-m radius at each station (7–32 stations site^− 1^). Each station across the 25 sites was surveyed three times (rounds) in each of the 5 years. We divided the total number of individuals observed on all rounds by the total number of point counts to obtain estimates of mean individuals ha^− 1^ for each site and year. Further details of population monitoring methods were reported in earlier studies^34,61^.

### Vegetation surveys

To determine whether spatial and temporal variation in food and nest site availability influenced species interactions, we established 0.04-ha circular vegetation plots centered at each point count station every year during 2004–2008. For all trees ≥12.5 cm diameter at breast height (DBH; measured at 1.3 m above ground) in each plot, we recorded tree species, DBH, general health (e.g., presence of boring insects on the bole), and decay class. Decay class 1 was a live, healthy tree, 2 a live tree with visible sign of bark boring insects or heart rot fungus, and 3–8 were standing dead trees in progressive states of decay^68^. Before and during the study period, an outbreak of mountain pine beetle occurred across all sites, with incidence of beetle attacks on pines increasing sharply after 2002, and by 2005 over 95% of the mature lodgepole pine trees (40% of the trees on the sites) were dead^69^. However, the onset of the beetle outbreak showed temporal and spatial variation in the number of trees showing sign of beetle attack^70^. Therefore, we could examine the effects of beetle abundance at the site-year level. Beetle eggs are laid beneath the bark in late summer where they overwinter and beetle larvae complete development in the following summer before emerging as adults^57^. Thus, beetle larvae provided a rich food source throughout the winters and following breeding seasons for insectivores. We determined beetle-infected pine densities as the total number of decay class 2 pine trees with bark boring insects, which was evident by the presence of dried resin outflows, or small entry holes (~ 2 mm in diameter) on the bark, expressed on a per ha basis divided by the total number of 0.04 ha vegetation plots, for each site and year. Since over 90% of chickadee and nuthatch nests were in aspen trees^61^, we determined the densities of potential nest trees per ha from number of aspen trees divided by the total number of 0.04 ha plots, for each site and year.

### Statistical analyses

We examined how territory characteristics (proximity to active and inactive nests, food and nest site availability, and population densities of conspecifics, and heterospecifics, including squirrels) influenced intra- and inter-specific aggression of chickadees and nuthatches. We used the closest distance (m) that a respondent approached each intruder during the territory intrusion simulations as the metric of aggression. For both species, the data for closest distance approached were heavily skewed toward zero and showed an uneven distribution in the number of response variables between 0 and 50 m. As a result of the truncated normal distribution and the high number of zero values, we applied a mixture of the binomial (for closest distance = 0 or > 0) and left-truncated normal (for closest distance > 0) distributions. We used linear mixed-effects models^71^ to examine how variation in closest distance approached was explained by the fixed-effects variables: intruder species (conspecific or heterospecific), plot type of the experiment (active chickadee nest; ACN, active nuthatch nest; ANN, inactive nest, active chickadee territory; ACT, active nuthatch territory; ANT, or inactive territory), breeding status of territory owners (if on an active nest plot), presentation sequence of intruders, and abundance of food (beetle-infected live pine densities per ha for the corresponding site and year in which the intrusion was simulated) and nest sites (nest-tree densities per ha), and densities per ha of red-breasted nuthatch, mountain chickadee, and red squirrel, and all biologically relevant secondary interaction terms. Because we simulated intrusions at multiple locations (plots) within sites and at multiple sites within years and respondents included unmarked birds that could have been tested at multiple sites across years, we included plot nested within site as a random effect to account for the hierarchical error structure due to the potential and actual repeated measures, in all models. Since the repeated measures were not evenly spaced within and across plots, we added a continuous autoregressive correlation within plots. These hierarchical errors were assumed to have normal distributions.

We used penalized quasi-likelihood (PQL) methods to generate parameter estimates^72^. We used Wald’s t-test to eliminate fixed-effects variables from fully parameterized models, and to determine the strength of evidence to support whether fixed-effect variables correlated with the response variables given the other fixed-effect variables and the hierarchical error structure in the best-fit (final) model^72^. Negative signs of coefficients indicated that the closest distance approached decreased (i.e., the responding bird approached closer to the model, and was more aggressive) and positive signs indicated that distance approached increased (the bird did not approach as closely, and was less aggressive) with increases in the fixed effect variables^71^. Penalized quasi-likelihood estimates were obtained using the function glmmPQL in the library MASS, and all data analyses were conducted in the program R version 2023.06.1^73,74^. To address problems associated with null-hypothesis significance testing based on arbitrary *P*-value thresholds, we reported statistical results using language of evidence consistent with current reporting approaches of international research networks^75^.

## Electronic supplementary material

Below is the link to the electronic supplementary material.

Supplementary Material 1

Supplementary Material 2

Supplementary Material 3

Supplementary Material 4

Supplementary Material 5

## Data Availability

All data generated or analyzed during this study are included in this published article (and its supplementary information files).
