# Supplementary figures and images for: Cavity nesting birds show behavioural plasticity to simulated territorial intrusions in response to natural resource pulses
Source: Sci Rep. 2025 Mar 18;15:9338. doi: 10.1038/s41598-025-93109-y (PMC11920054; doi:10.1038/s41598-025-93109-y)

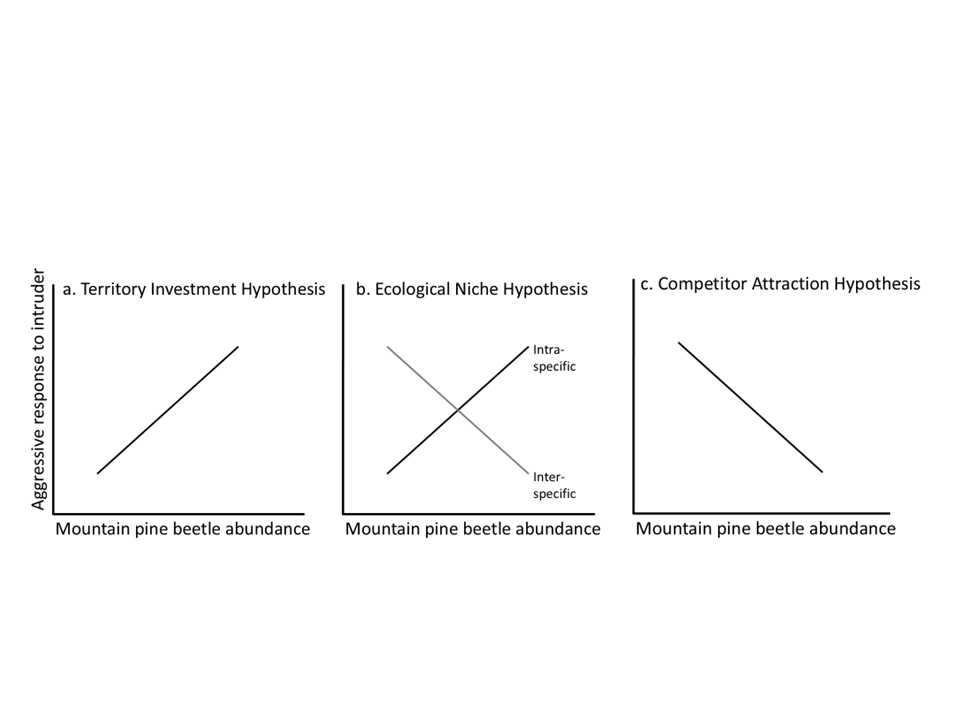

Supplement: Supplementary file 1 — Supplementary Material 1 [file 41598_2025_93109_MOESM1_ESM.tif]

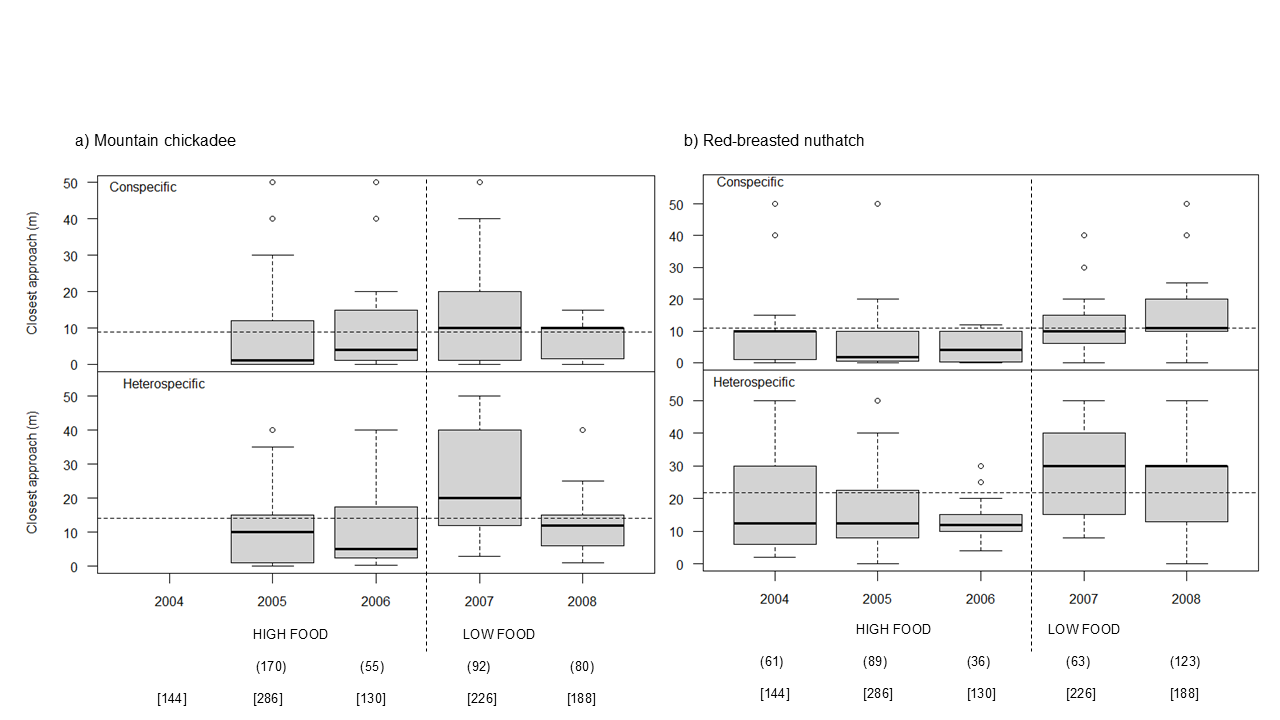

Supplement: Supplementary file 2 — Supplementary Material 2 [file 41598_2025_93109_MOESM2_ESM.tif]

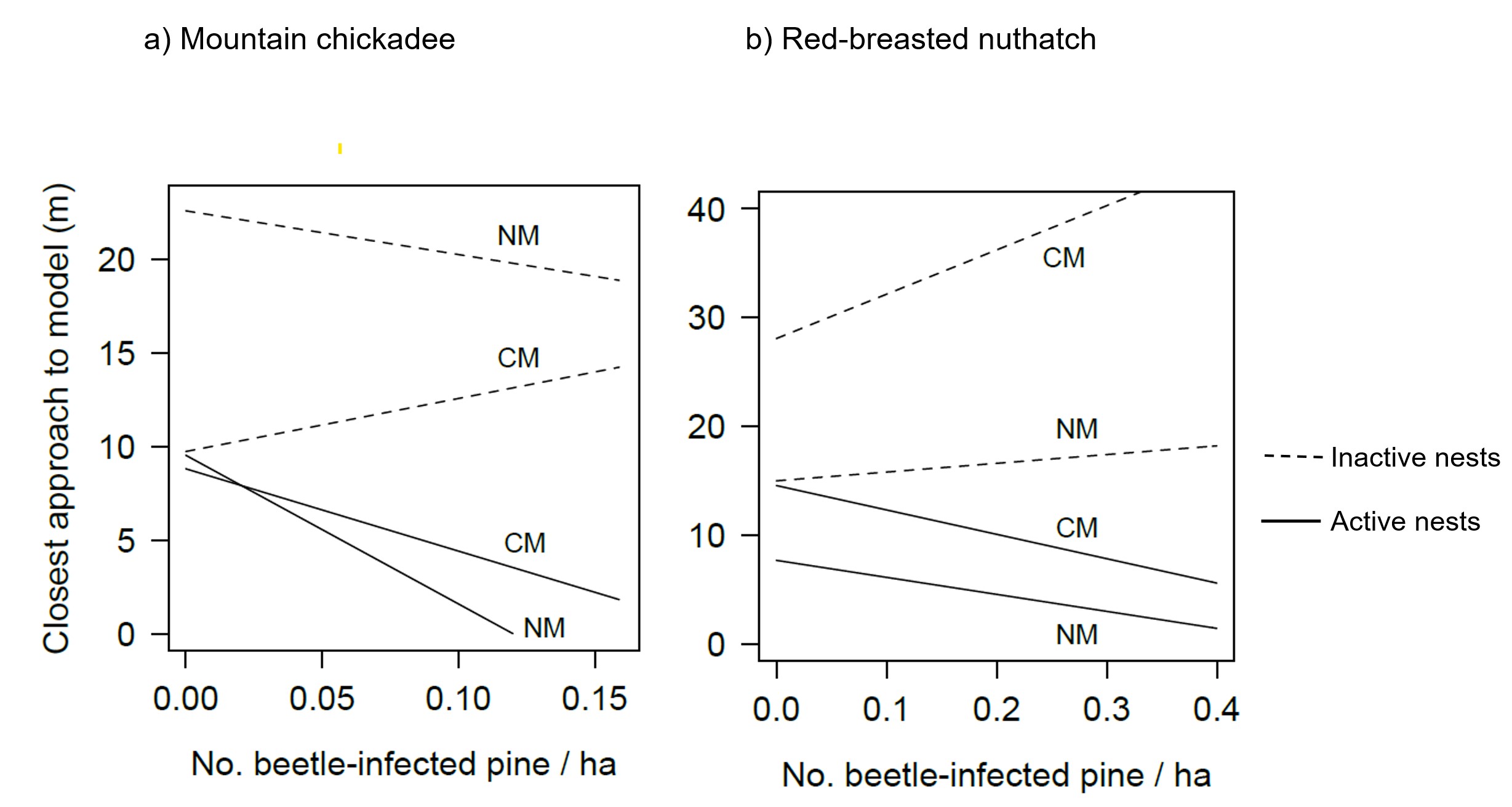

Supplement: Supplementary file 3 — Supplementary Material 3 [file 41598_2025_93109_MOESM3_ESM.jpg]
