# Supplementary material for: Cavity nesting birds show behavioural plasticity to simulated territorial intrusions in response to natural resource pulses
Source: Sci Rep. 2025 Mar 18;15:9338. doi: 10.1038/s41598-025-93109-y (PMC11920054; doi:10.1038/s41598-025-93109-y)
Supplement: Supplementary file 4 — Supplementary Material 4 [file 41598_2025_93109_MOESM4_ESM.pdf]

data.entry.	Date	Year	Site	MOCH	RBNU	BCCH	TAHU	LP2BI	LiveAT.ha
315	30-May-04	2004	RP	0.095238	0.142857	0.095238	0.071429	0.323383	2
316	30-May-04	2004	RP	0.095238	0.142857	0.095238	0.071429	0.323383	2
317	30-May-04	2004	RP	0.095238	0.142857	0.095238	0.071429	0.323383	2
318	30-May-04	2004	RP	0.095238	0.142857	0.095238	0.071429	0.323383	2
322	30-May-04	2004	RP	0.095238	0.142857	0.095238	0.071429	0.323383	2
326	30-May-04	2004	RP	0.095238	0.142857	0.095238	0.071429	0.323383	2
329	30-May-04	2004	RP	0.095238	0.142857	0.095238	0.071429	0.323383	2
332	30-May-04	2004	RP	0.095238	0.142857	0.095238	0.071429	0.323383	2
333	30-May-04	2004	RP	0.095238	0.142857	0.095238	0.071429	0.323383	2
330	30-May-04	2004	RP	0.095238	0.142857	0.095238	0.071429	0.323383	2
321	30-May-04	2004	RP	0.095238	0.142857	0.095238	0.071429	0.323383	2
319	30-May-04	2004	RP	0.095238	0.142857	0.095238	0.071429	0.323383	2
320	30-May-04	2004	RP	0.095238	0.142857	0.095238	0.071429	0.323383	2
324	30-May-04	2004	RP	0.095238	0.142857	0.095238	0.071429	0.323383	2
327	30-May-04	2004	RP	0.095238	0.142857	0.095238	0.071429	0.323383	2
331	30-May-04	2004	RP	0.095238	0.142857	0.095238	0.071429	0.323383	2
328	30-May-04	2004	RP	0.095238	0.142857	0.095238	0.071429	0.323383	2
314	30-May-04	2004	RP	0.095238	0.142857	0.095238	0.071429	0.323383	2
325	30-May-04	2004	RP	0.095238	0.142857	0.095238	0.071429	0.323383	2
323	30-May-04	2004	RP	0.095238	0.142857	0.095238	0.071429	0.323383	2
1343	9-May-08	2008	SC	0.191781	0.013699	0.123288	0	0	8.615385
1362	9-May-08	2008	SC	0.191781	0.013699	0.123288	0	0	8.615385
1337	9-May-08	2008	SC	0.191781	0.013699	0.123288	0	0	8.615385
1333	9-May-08	2008	SC	0.191781	0.013699	0.123288	0	0	8.615385
1334	9-May-08	2008	SC	0.191781	0.013699	0.123288	0	0	8.615385
1335	9-May-08	2008	SC	0.191781	0.013699	0.123288	0	0	8.615385
1338	9-May-08	2008	SC	0.191781	0.013699	0.123288	0	0	8.615385
1340	9-May-08	2008	SC	0.191781	0.013699	0.123288	0	0	8.615385
1342	9-May-08	2008	SC	0.191781	0.013699	0.123288	0	0	8.615385
1344	9-May-08	2008	SC	0.191781	0.013699	0.123288	0	0	8.615385
1345	9-May-08	2008	SC	0.191781	0.013699	0.123288	0	0	8.615385
1347	9-May-08	2008	SC	0.191781	0.013699	0.123288	0	0	8.615385
1348	9-May-08	2008	SC	0.191781	0.013699	0.123288	0	0	8.615385
1349	9-May-08	2008	SC	0.191781	0.013699	0.123288	0	0	8.615385
1352	9-May-08	2008	SC	0.191781	0.013699	0.123288	0	0	8.615385
1359	9-May-08	2008	SC	0.191781	0.013699	0.123288	0	0	8.615385
1360	9-May-08	2008	SC	0.191781	0.013699	0.123288	0	0	8.615385
1361	9-May-08	2008	SC	0.191781	0.013699	0.123288	0	0	8.615385
1364	9-May-08	2008	SC	0.191781	0.013699	0.123288	0	0	8.615385
1332	9-May-08	2008	SC	0.191781	0.013699	0.123288	0	0	8.615385
1339	9-May-08	2008	SC	0.191781	0.013699	0.123288	0	0	8.615385
1341	9-May-08	2008	SC	0.191781	0.013699	0.123288	0	0	8.615385
1350	9-May-08	2008	SC	0.191781	0.013699	0.123288	0	0	8.615385

1357	9-May-08	2008 SC	0.191781	0.013699	0.123288	0	0	8.615385
1351	9-May-08	2008 SC	0.191781	0.013699	0.123288	0	0	8.615385
1363	9-May-08	2008 SC	0.191781	0.013699	0.123288	0	0	8.615385
1331	9-May-08	2008 SC	0.191781	0.013699	0.123288	0	0	8.615385
1353	9-May-08	2008 SC	0.191781	0.013699	0.123288	0	0	8.615385
1336	9-May-08	2008 SC	0.191781	0.013699	0.123288	0	0	8.615385
1354	9-May-08	2008 SC	0.191781	0.013699	0.123288	0	0	8.615385
1355	9-May-08	2008 SC	0.191781	0.013699	0.123288	0	0	8.615385
1356	9-May-08	2008 SC	0.191781	0.013699	0.123288	0	0	8.615385
1346	9-May-08	2008 SC	0.191781	0.013699	0.123288	0	0	8.615385
1358	9-May-08	2008 SC	0.191781	0.013699	0.123288	0	0	8.615385
1330	9-May-08	2008 SC	0.191781	0.013699	0.123288	0	0	8.615385
895	29-May-08	2008 LT1	0.925926	0.222222	0.037037	0.04	0	3.666667
894	29-May-08	2008 LT1	0.925926	0.222222	0.037037	0.04	0	3.666667
896	29-May-08	2008 LT1	0.925926	0.222222	0.037037	0.04	0	3.666667
887	29-May-08	2008 LT1	0.925926	0.222222	0.037037	0.04	0	3.666667
889	29-May-08	2008 LT1	0.925926	0.222222	0.037037	0.04	0	3.666667
885	29-May-08	2008 LT1	0.925926	0.222222	0.037037	0.04	0	3.666667
888	29-May-08	2008 LT1	0.925926	0.222222	0.037037	0.04	0	3.666667
897	29-May-08	2008 LT1	0.925926	0.222222	0.037037	0.04	0	3.666667
884	29-May-08	2008 LT1	0.925926	0.222222	0.037037	0.04	0	3.666667
890	29-May-08	2008 LT1	0.925926	0.222222	0.037037	0.04	0	3.666667
892	29-May-08	2008 LT1	0.925926	0.222222	0.037037	0.04	0	3.666667
886	29-May-08	2008 LT1	0.925926	0.222222	0.037037	0.04	0	3.666667
883	29-May-08	2008 LT1	0.925926	0.222222	0.037037	0.04	0	3.666667
891	29-May-08	2008 LT1	0.925926	0.222222	0.037037	0.04	0	3.666667
893	29-May-08	2008 LT1	0.925926	0.222222	0.037037	0.04	0	3.666667
898	29-May-08	2008 LT1	0.925926	0.222222	0.037037	0.04	0	3.666667
882	29-May-08	2008 LT1	0.925926	0.222222	0.037037	0.04	0	3.666667
1632	15-Jun-08	2008 LT1	0.925926	0.222222	0.037037	0.04	0	3.666667
1634	15-Jun-08	2008 LT1	0.925926	0.222222	0.037037	0.04	0	3.666667
1628	15-Jun-08	2008 LT1	0.925926	0.222222	0.037037	0.04	0	3.666667
1629	15-Jun-08	2008 LT1	0.925926	0.222222	0.037037	0.04	0	3.666667
1635	15-Jun-08	2008 LT1	0.925926	0.222222	0.037037	0.04	0	3.666667
1636	15-Jun-08	2008 LT1	0.925926	0.222222	0.037037	0.04	0	3.666667
1637	15-Jun-08	2008 LT1	0.925926	0.222222	0.037037	0.04	0	3.666667
1639	15-Jun-08	2008 LT1	0.925926	0.222222	0.037037	0.04	0	3.666667
1638	15-Jun-08	2008 LT1	0.925926	0.222222	0.037037	0.04	0	3.666667
1640	15-Jun-08	2008 LT1	0.925926	0.222222	0.037037	0.04	0	3.666667
1641	15-Jun-08	2008 LT1	0.925926	0.222222	0.037037	0.04	0	3.666667
1633	15-Jun-08	2008 LT1	0.925926	0.222222	0.037037	0.04	0	3.666667
1642	15-Jun-08	2008 LT1	0.925926	0.222222	0.037037	0.04	0	3.666667
1627	15-Jun-08	2008 LT1	0.925926	0.222222	0.037037	0.04	0	3.666667
1630	15-Jun-08	2008 LT1	0.925926	0.222222	0.037037	0.04	0	3.666667

1631	15-Jun-08	2008	LT1	0.925926	0.222222	0.037037	0.04	0	3.666667
1643	15-Jun-08	2008	LT1	0.925926	0.222222	0.037037	0.04	0	3.666667
1626	15-Jun-08	2008	LT1	0.925926	0.222222	0.037037	0.04	0	3.666667
3045	5-Jun-07	2007	MC	0.157895	0.157895	0	0	0.05973	2.4
3046	5-Jun-07	2007	MC	0.157895	0.157895	0	0	0.05973	2.4
3047	5-Jun-07	2007	MC	0.157895	0.157895	0	0	0.05973	2.4
3048	5-Jun-07	2007	MC	0.157895	0.157895	0	0	0.05973	2.4
3049	5-Jun-07	2007	MC	0.157895	0.157895	0	0	0.05973	2.4
3050	5-Jun-07	2007	MC	0.157895	0.157895	0	0	0.05973	2.4
3051	5-Jun-07	2007	MC	0.157895	0.157895	0	0	0.05973	2.4
3052	5-Jun-07	2007	MC	0.157895	0.157895	0	0	0.05973	2.4
3044	5-Jun-07	2007	MC	0.157895	0.157895	0	0	0.05973	2.4
3053	5-Jun-07	2007	MC	0.157895	0.157895	0	0	0.05973	2.4
3054	5-Jun-07	2007	MC	0.157895	0.157895	0	0	0.05973	2.4
407	2-Jun-04	2004	7M	0.283019	0.471698	0.113208	0.679245	0.035088	8.333333
409	2-Jun-04	2004	7M	0.283019	0.471698	0.113208	0.679245	0.035088	8.333333
402	2-Jun-04	2004	7M	0.283019	0.471698	0.113208	0.679245	0.035088	8.333333
399	2-Jun-04	2004	7M	0.283019	0.471698	0.113208	0.679245	0.035088	8.333333
397	2-Jun-04	2004	7M	0.283019	0.471698	0.113208	0.679245	0.035088	8.333333
398	2-Jun-04	2004	7M	0.283019	0.471698	0.113208	0.679245	0.035088	8.333333
400	2-Jun-04	2004	7M	0.283019	0.471698	0.113208	0.679245	0.035088	8.333333
401	2-Jun-04	2004	7M	0.283019	0.471698	0.113208	0.679245	0.035088	8.333333
403	2-Jun-04	2004	7M	0.283019	0.471698	0.113208	0.679245	0.035088	8.333333
404	2-Jun-04	2004	7M	0.283019	0.471698	0.113208	0.679245	0.035088	8.333333
405	2-Jun-04	2004	7M	0.283019	0.471698	0.113208	0.679245	0.035088	8.333333
406	2-Jun-04	2004	7M	0.283019	0.471698	0.113208	0.679245	0.035088	8.333333
408	2-Jun-04	2004	7M	0.283019	0.471698	0.113208	0.679245	0.035088	8.333333
410	2-Jun-04	2004	7M	0.283019	0.471698	0.113208	0.679245	0.035088	8.333333
411	2-Jun-04	2004	7M	0.283019	0.471698	0.113208	0.679245	0.035088	8.333333
412	2-Jun-04	2004	7M	0.283019	0.471698	0.113208	0.679245	0.035088	8.333333
413	2-Jun-04	2004	7M	0.283019	0.471698	0.113208	0.679245	0.035088	8.333333
396	2-Jun-04	2004	7M	0.283019	0.471698	0.113208	0.679245	0.035088	8.333333
282	16-May-05	2005	RL	0.465517	0.103448	0	0.137931	0.108209	0.333333
283	16-May-05	2005	RL	0.465517	0.103448	0	0.137931	0.108209	0.333333
272	16-May-05	2005	RL	0.465517	0.103448	0	0.137931	0.108209	0.333333
279	16-May-05	2005	RL	0.465517	0.103448	0	0.137931	0.108209	0.333333
267	16-May-05	2005	RL	0.465517	0.103448	0	0.137931	0.108209	0.333333
277	16-May-05	2005	RL	0.465517	0.103448	0	0.137931	0.108209	0.333333
271	16-May-05	2005	RL	0.465517	0.103448	0	0.137931	0.108209	0.333333
280	16-May-05	2005	RL	0.465517	0.103448	0	0.137931	0.108209	0.333333
274	16-May-05	2005	RL	0.465517	0.103448	0	0.137931	0.108209	0.333333
284	16-May-05	2005	RL	0.465517	0.103448	0	0.137931	0.108209	0.333333
266	16-May-05	2005	RL	0.465517	0.103448	0	0.137931	0.108209	0.333333
285	16-May-05	2005	RL	0.465517	0.103448	0	0.137931	0.108209	0.333333

268	16-May-05	2005	RL	0.465517	0.103448	0	0.137931	0.108209	0.333333
275	16-May-05	2005	RL	0.465517	0.103448	0	0.137931	0.108209	0.333333
281	16-May-05	2005	RL	0.465517	0.103448	0	0.137931	0.108209	0.333333
270	16-May-05	2005	RL	0.465517	0.103448	0	0.137931	0.108209	0.333333
269	16-May-05	2005	RL	0.465517	0.103448	0	0.137931	0.108209	0.333333
273	16-May-05	2005	RL	0.465517	0.103448	0	0.137931	0.108209	0.333333
276	16-May-05	2005	RL	0.465517	0.103448	0	0.137931	0.108209	0.333333
278	16-May-05	2005	RL	0.465517	0.103448	0	0.137931	0.108209	0.333333
286	16-May-05	2005	RL	0.465517	0.103448	0	0.137931	0.108209	0.333333
621	21-May-05	2005	SC	0.183486	0	0.073394	0	0.006329	6.769231
617	21-May-05	2005	SC	0.183486	0	0.073394	0	0.006329	6.769231
619	21-May-05	2005	SC	0.183486	0	0.073394	0	0.006329	6.769231
620	21-May-05	2005	SC	0.183486	0	0.073394	0	0.006329	6.769231
622	21-May-05	2005	SC	0.183486	0	0.073394	0	0.006329	6.769231
615	21-May-05	2005	SC	0.183486	0	0.073394	0	0.006329	6.769231
618	21-May-05	2005	SC	0.183486	0	0.073394	0	0.006329	6.769231
613	21-May-05	2005	SC	0.183486	0	0.073394	0	0.006329	6.769231
624	21-May-05	2005	SC	0.183486	0	0.073394	0	0.006329	6.769231
614	21-May-05	2005	SC	0.183486	0	0.073394	0	0.006329	6.769231
616	21-May-05	2005	SC	0.183486	0	0.073394	0	0.006329	6.769231
623	21-May-05	2005	SC	0.183486	0	0.073394	0	0.006329	6.769231
625	21-May-05	2005	SC	0.183486	0	0.073394	0	0.006329	6.769231
612	21-May-05	2005	SC	0.183486	0	0.073394	0	0.006329	6.769231
645	21-May-05	2005	DE						
644	21-May-05	2005	DE						
648	21-May-05	2005	DE						
643	21-May-05	2005	DE						
650	21-May-05	2005	DE						
649	21-May-05	2005	DE						
642	21-May-05	2005	DE						
654	21-May-05	2005	DE						
637	21-May-05	2005	DE						
640	21-May-05	2005	DE						
641	21-May-05	2005	DE						
646	21-May-05	2005	DE						
647	21-May-05	2005	DE						
653	21-May-05	2005	DE						
651	21-May-05	2005	DE						
638	21-May-05	2005	DE						
652	21-May-05	2005	DE						
636	21-May-05	2005	DE						
639	21-May-05	2005	DE						
984	1-Jun-05	2005	D1/D2						
990	1-Jun-05	2005	D1/D2						

991	1-Jun-05	2005	D1/D2						
983	1-Jun-05	2005	D1/D2						
982	1-Jun-05	2005	D1/D2						
985	1-Jun-05	2005	D1/D2						
988	1-Jun-05	2005	D1/D2						
992	1-Jun-05	2005	D1/D2						
996	1-Jun-05	2005	D1/D2						
986	1-Jun-05	2005	D1/D2						
987	1-Jun-05	2005	D1/D2						
989	1-Jun-05	2005	D1/D2						
993	1-Jun-05	2005	D1/D2						
994	1-Jun-05	2005	D1/D2						
995	1-Jun-05	2005	D1/D2						
1426	10-Jun-05	2005	SC	0.183486	0	0.073394	0	0.006329	6.769231
1427	10-Jun-05	2005	SC	0.183486	0	0.073394	0	0.006329	6.769231
1430	10-Jun-05	2005	SC	0.183486	0	0.073394	0	0.006329	6.769231
1431	10-Jun-05	2005	SC	0.183486	0	0.073394	0	0.006329	6.769231
1424	10-Jun-05	2005	SC	0.183486	0	0.073394	0	0.006329	6.769231
1432	10-Jun-05	2005	SC	0.183486	0	0.073394	0	0.006329	6.769231
1429	10-Jun-05	2005	SC	0.183486	0	0.073394	0	0.006329	6.769231
1422	10-Jun-05	2005	SC	0.183486	0	0.073394	0	0.006329	6.769231
1421	10-Jun-05	2005	SC	0.183486	0	0.073394	0	0.006329	6.769231
1428	10-Jun-05	2005	SC	0.183486	0	0.073394	0	0.006329	6.769231
1425	10-Jun-05	2005	SC	0.183486	0	0.073394	0	0.006329	6.769231
1420	10-Jun-05	2005	SC	0.183486	0	0.073394	0	0.006329	6.769231
1423	10-Jun-05	2005	SC	0.183486	0	0.073394	0	0.006329	6.769231
1708	18-Jun-05	2005	RL	0.465517	0.103448	0	0.137931	0.108209	0.333333
1705	18-Jun-05	2005	RL	0.465517	0.103448	0	0.137931	0.108209	0.333333
1700	18-Jun-05	2005	RL	0.465517	0.103448	0	0.137931	0.108209	0.333333
1701	18-Jun-05	2005	RL	0.465517	0.103448	0	0.137931	0.108209	0.333333
1702	18-Jun-05	2005	RL	0.465517	0.103448	0	0.137931	0.108209	0.333333
1704	18-Jun-05	2005	RL	0.465517	0.103448	0	0.137931	0.108209	0.333333
1706	18-Jun-05	2005	RL	0.465517	0.103448	0	0.137931	0.108209	0.333333
1707	18-Jun-05	2005	RL	0.465517	0.103448	0	0.137931	0.108209	0.333333
1709	18-Jun-05	2005	RL	0.465517	0.103448	0	0.137931	0.108209	0.333333
1703	18-Jun-05	2005	RL	0.465517	0.103448	0	0.137931	0.108209	0.333333
1534	18-Jun-05	2005	RL	0.465517	0.103448	0	0.137931	0.108209	0.333333
1530	18-Jun-05	2005	RL	0.465517	0.103448	0	0.137931	0.108209	0.333333
1533	18-Jun-05	2005	RL	0.465517	0.103448	0	0.137931	0.108209	0.333333
1532	18-Jun-05	2005	RL	0.465517	0.103448	0	0.137931	0.108209	0.333333
1536	18-Jun-05	2005	RL	0.465517	0.103448	0	0.137931	0.108209	0.333333
1524	18-Jun-05	2005	RL	0.465517	0.103448	0	0.137931	0.108209	0.333333
1535	18-Jun-05	2005	RL	0.465517	0.103448	0	0.137931	0.108209	0.333333
1519	18-Jun-05	2005	RL	0.465517	0.103448	0	0.137931	0.108209	0.333333

1529	18-Jun-05	2005	RL	0.465517	0.103448	0	0.137931	0.108209	0.333333
1531	18-Jun-05	2005	RL	0.465517	0.103448	0	0.137931	0.108209	0.333333
1520	18-Jun-05	2005	RL	0.465517	0.103448	0	0.137931	0.108209	0.333333
1528	18-Jun-05	2005	RL	0.465517	0.103448	0	0.137931	0.108209	0.333333
1527	18-Jun-05	2005	RL	0.465517	0.103448	0	0.137931	0.108209	0.333333
1518	18-Jun-05	2005	RL	0.465517	0.103448	0	0.137931	0.108209	0.333333
1521	18-Jun-05	2005	RL	0.465517	0.103448	0	0.137931	0.108209	0.333333
1525	18-Jun-05	2005	RL	0.465517	0.103448	0	0.137931	0.108209	0.333333
1526	18-Jun-05	2005	RL	0.465517	0.103448	0	0.137931	0.108209	0.333333
1522	18-Jun-05	2005	RL	0.465517	0.103448	0	0.137931	0.108209	0.333333
1523	18-Jun-05	2005	RL	0.465517	0.103448	0	0.137931	0.108209	0.333333
1515	18-Jun-05	2005	RL	0.465517	0.103448	0	0.137931	0.108209	0.333333
1516	18-Jun-05	2005	RL	0.465517	0.103448	0	0.137931	0.108209	0.333333
1517	18-Jun-05	2005	RL	0.465517	0.103448	0	0.137931	0.108209	0.333333
	25-Jun-06	2006	ML						
	25-Jun-06	2006	ML						
	25-Jun-06	2006	ML						
	25-Jun-06	2006	ML						
	25-Jun-06	2006	ML						
	25-Jun-06	2006	ML						
	25-Jun-06	2006	ML						
2549	25-May-07	2007	MG	0.24	0.28	0.12	0.02	0.009615	6.15
2550	25-May-07	2007	MG	0.24	0.28	0.12	0.02	0.009615	6.15
2540	25-May-07	2007	MG	0.24	0.28	0.12	0.02	0.009615	6.15
2545	25-May-07	2007	MG	0.24	0.28	0.12	0.02	0.009615	6.15
2546	25-May-07	2007	MG	0.24	0.28	0.12	0.02	0.009615	6.15
2537	25-May-07	2007	MG	0.24	0.28	0.12	0.02	0.009615	6.15
2539	25-May-07	2007	MG	0.24	0.28	0.12	0.02	0.009615	6.15
2541	25-May-07	2007	MG	0.24	0.28	0.12	0.02	0.009615	6.15
2542	25-May-07	2007	MG	0.24	0.28	0.12	0.02	0.009615	6.15
2544	25-May-07	2007	MG	0.24	0.28	0.12	0.02	0.009615	6.15
2548	25-May-07	2007	MG	0.24	0.28	0.12	0.02	0.009615	6.15
2547	25-May-07	2007	MG	0.24	0.28	0.12	0.02	0.009615	6.15
2536	25-May-07	2007	MG	0.24	0.28	0.12	0.02	0.009615	6.15
2538	25-May-07	2007	MG	0.24	0.28	0.12	0.02	0.009615	6.15
2543	25-May-07	2007	MG	0.24	0.28	0.12	0.02	0.009615	6.15
2911	31-May-07	2007	SC	0.136986	0.041096	0.027397	0	0	8.576923
2918	31-May-07	2007	SC	0.136986	0.041096	0.027397	0	0	8.576923
2919	31-May-07	2007	SC	0.136986	0.041096	0.027397	0	0	8.576923
2904	31-May-07	2007	SC	0.136986	0.041096	0.027397	0	0	8.576923
2901	31-May-07	2007	SC	0.136986	0.041096	0.027397	0	0	8.576923
2900	31-May-07	2007	SC	0.136986	0.041096	0.027397	0	0	8.576923
2902	31-May-07	2007	SC	0.136986	0.041096	0.027397	0	0	8.576923
2903	31-May-07	2007	SC	0.136986	0.041096	0.027397	0	0	8.576923

2899	31-May-07	2007 SC	0.136986	0.041096	0.027397	0	0	8.576923
2905	31-May-07	2007 SC	0.136986	0.041096	0.027397	0	0	8.576923
2898	31-May-07	2007 SC	0.136986	0.041096	0.027397	0	0	8.576923
2906	31-May-07	2007 SC	0.136986	0.041096	0.027397	0	0	8.576923
2921	31-May-07	2007 SC	0.136986	0.041096	0.027397	0	0	8.576923
2907	31-May-07	2007 SC	0.136986	0.041096	0.027397	0	0	8.576923
2920	31-May-07	2007 SC	0.136986	0.041096	0.027397	0	0	8.576923
2908	31-May-07	2007 SC	0.136986	0.041096	0.027397	0	0	8.576923
2909	31-May-07	2007 SC	0.136986	0.041096	0.027397	0	0	8.576923
2910	31-May-07	2007 SC	0.136986	0.041096	0.027397	0	0	8.576923
2913	31-May-07	2007 SC	0.136986	0.041096	0.027397	0	0	8.576923
2914	31-May-07	2007 SC	0.136986	0.041096	0.027397	0	0	8.576923
2915	31-May-07	2007 SC	0.136986	0.041096	0.027397	0	0	8.576923
2916	31-May-07	2007 SC	0.136986	0.041096	0.027397	0	0	8.576923
2917	31-May-07	2007 SC	0.136986	0.041096	0.027397	0	0	8.576923
2893	31-May-07	2007 SC	0.136986	0.041096	0.027397	0	0	8.576923
2894	31-May-07	2007 SC	0.136986	0.041096	0.027397	0	0	8.576923
2895	31-May-07	2007 SC	0.136986	0.041096	0.027397	0	0	8.576923
2897	31-May-07	2007 SC	0.136986	0.041096	0.027397	0	0	8.576923
2892	31-May-07	2007 SC	0.136986	0.041096	0.027397	0	0	8.576923
2891	31-May-07	2007 SC	0.136986	0.041096	0.027397	0	0	8.576923
2896	31-May-07	2007 SC	0.136986	0.041096	0.027397	0	0	8.576923
2912	31-May-07	2007 SC	0.136986	0.041096	0.027397	0	0	8.576923
	31-May-07	2007 SC	0.136986	0.041096	0.027397	0	0	8.576923
	31-May-07	2007 SC	0.136986	0.041096	0.027397	0	0	8.576923
	31-May-07	2007 SC	0.136986	0.041096	0.027397	0	0	8.576923
	31-May-07	2007 SC	0.136986	0.041096	0.027397	0	0	8.576923
	31-May-07	2007 SC	0.136986	0.041096	0.027397	0	0	8.576923
	31-May-07	2007 SC	0.136986	0.041096	0.027397	0	0	8.576923
	31-May-07	2007 SC	0.136986	0.041096	0.027397	0	0	8.576923
	31-May-07	2007 SC	0.136986	0.041096	0.027397	0	0	8.576923
	31-May-07	2007 SC	0.136986	0.041096	0.027397	0	0	8.576923
	31-May-07	2007 SC	0.136986	0.041096	0.027397	0	0	8.576923
	31-May-07	2007 SC	0.136986	0.041096	0.027397	0	0	8.576923
	31-May-07	2007 SC	0.136986	0.041096	0.027397	0	0	8.576923
	31-May-07	2007 SC	0.136986	0.041096	0.027397	0	0	8.576923
	31-May-07	2007 SC	0.136986	0.041096	0.027397	0	0	8.576923
	15-Jun-07	2007 SC	0.136986	0.041096	0.027397	0	0	8.576923
	15-Jun-07	2007 SC	0.136986	0.041096	0.027397	0	0	8.576923
	15-Jun-07	2007 SC	0.136986	0.041096	0.027397	0	0	8.576923
	15-Jun-07	2007 SC	0.136986	0.041096	0.027397	0	0	8.576923
	15-Jun-07	2007 SC	0.136986	0.041096	0.027397	0	0	8.576923
	15-Jun-07	2007 SC	0.136986	0.041096	0.027397	0	0	8.576923
	15-Jun-07	2007 SC	0.136986	0.041096	0.027397	0	0	8.576923

	15-Jun-07	2007	SC	0.136986	0.041096	0.027397	0	0	8.576923
	15-Jun-07	2007	SC	0.136986	0.041096	0.027397	0	0	8.576923
	15-Jun-07	2007	SC	0.136986	0.041096	0.027397	0	0	8.576923
	15-Jun-07	2007	SC	0.136986	0.041096	0.027397	0	0	8.576923
	15-Jun-07	2007	SC	0.136986	0.041096	0.027397	0	0	8.576923
	15-Jun-07	2007	SC	0.136986	0.041096	0.027397	0	0	8.576923
	15-Jun-07	2007	SC	0.136986	0.041096	0.027397	0	0	8.576923
1056	30-May-08	2008	ML						
1057	30-May-08	2008	ML						
1058	30-May-08	2008	ML						
1059	30-May-08	2008	ML						
1050	30-May-08	2008	ML						
1064	30-May-08	2008	ML						
1031	30-May-08	2008	ML						
1038	30-May-08	2008	ML						
1040	30-May-08	2008	ML						
1041	30-May-08	2008	ML						
1044	30-May-08	2008	ML						
1048	30-May-08	2008	ML						
1051	30-May-08	2008	ML						
1061	30-May-08	2008	ML						
1037	30-May-08	2008	ML						
1046	30-May-08	2008	ML						
1043	30-May-08	2008	ML						
1052	30-May-08	2008	ML						
1063	30-May-08	2008	ML						
1035	30-May-08	2008	ML						
1034	30-May-08	2008	ML						
1033	30-May-08	2008	ML						
1036	30-May-08	2008	ML						
1039	30-May-08	2008	ML						
1042	30-May-08	2008	ML						
1047	30-May-08	2008	ML						
1045	30-May-08	2008	ML						
1049	30-May-08	2008	ML						
1053	30-May-08	2008	ML						
1055	30-May-08	2008	ML						
1032	30-May-08	2008	ML						
1054	30-May-08	2008	ML						
1060	30-May-08	2008	ML						
1065	30-May-08	2008	ML						
1062	30-May-08	2008	ML						
1030	30-May-08	2008	ML						
1495	10-Jun-08	2008	TO	0.2	0.2	0.05	0.025	0.00289	0.875

1493	10-Jun-08	2008	TO	0.2	0.2	0.05	0.025	0.00289	0.875
1494	10-Jun-08	2008	TO	0.2	0.2	0.05	0.025	0.00289	0.875
1481	10-Jun-08	2008	TO	0.2	0.2	0.05	0.025	0.00289	0.875
1487	10-Jun-08	2008	TO	0.2	0.2	0.05	0.025	0.00289	0.875
1486	10-Jun-08	2008	TO	0.2	0.2	0.05	0.025	0.00289	0.875
1484	10-Jun-08	2008	TO	0.2	0.2	0.05	0.025	0.00289	0.875
1490	10-Jun-08	2008	TO	0.2	0.2	0.05	0.025	0.00289	0.875
1491	10-Jun-08	2008	TO	0.2	0.2	0.05	0.025	0.00289	0.875
1478	10-Jun-08	2008	TO	0.2	0.2	0.05	0.025	0.00289	0.875
1480	10-Jun-08	2008	TO	0.2	0.2	0.05	0.025	0.00289	0.875
1482	10-Jun-08	2008	TO	0.2	0.2	0.05	0.025	0.00289	0.875
1489	10-Jun-08	2008	TO	0.2	0.2	0.05	0.025	0.00289	0.875
1485	10-Jun-08	2008	TO	0.2	0.2	0.05	0.025	0.00289	0.875
1488	10-Jun-08	2008	TO	0.2	0.2	0.05	0.025	0.00289	0.875
1479	10-Jun-08	2008	TO	0.2	0.2	0.05	0.025	0.00289	0.875
1496	10-Jun-08	2008	TO	0.2	0.2	0.05	0.025	0.00289	0.875
1497	10-Jun-08	2008	TO	0.2	0.2	0.05	0.025	0.00289	0.875
1477	10-Jun-08	2008	TO	0.2	0.2	0.05	0.025	0.00289	0.875
1483	10-Jun-08	2008	TO	0.2	0.2	0.05	0.025	0.00289	0.875
1492	10-Jun-08	2008	TO	0.2	0.2	0.05	0.025	0.00289	0.875
1498	10-Jun-08	2008	TO	0.2	0.2	0.05	0.025	0.00289	0.875
1476	10-Jun-08	2008	TO	0.2	0.2	0.05	0.025	0.00289	0.875
1527	15-Jun-08	2008	LT1	0.925926	0.222222	0.037037	0.04	0	3.666667
1528	15-Jun-08	2008	LT1	0.925926	0.222222	0.037037	0.04	0	3.666667
1529	15-Jun-08	2008	LT1	0.925926	0.222222	0.037037	0.04	0	3.666667
1530	15-Jun-08	2008	LT1	0.925926	0.222222	0.037037	0.04	0	3.666667
1532	15-Jun-08	2008	LT1	0.925926	0.222222	0.037037	0.04	0	3.666667
1533	15-Jun-08	2008	LT1	0.925926	0.222222	0.037037	0.04	0	3.666667
1534	15-Jun-08	2008	LT1	0.925926	0.222222	0.037037	0.04	0	3.666667
1535	15-Jun-08	2008	LT1	0.925926	0.222222	0.037037	0.04	0	3.666667
1536	15-Jun-08	2008	LT1	0.925926	0.222222	0.037037	0.04	0	3.666667
1537	15-Jun-08	2008	LT1	0.925926	0.222222	0.037037	0.04	0	3.666667
1538	15-Jun-08	2008	LT1	0.925926	0.222222	0.037037	0.04	0	3.666667
1523	15-Jun-08	2008	LT1	0.925926	0.222222	0.037037	0.04	0	3.666667
1524	15-Jun-08	2008	LT1	0.925926	0.222222	0.037037	0.04	0	3.666667
1526	15-Jun-08	2008	LT1	0.925926	0.222222	0.037037	0.04	0	3.666667
1522	15-Jun-08	2008	LT1	0.925926	0.222222	0.037037	0.04	0	3.666667
1521	15-Jun-08	2008	LT1	0.925926	0.222222	0.037037	0.04	0	3.666667
1525	15-Jun-08	2008	LT1	0.925926	0.222222	0.037037	0.04	0	3.666667
1531	15-Jun-08	2008	LT1	0.925926	0.222222	0.037037	0.04	0	3.666667
1539	15-Jun-08	2008	LT1	0.925926	0.222222	0.037037	0.04	0	3.666667
1520	15-Jun-08	2008	LT1	0.925926	0.222222	0.037037	0.04	0	3.666667
1574	15-Jun-08	2008	LT1	0.925926	0.222222	0.037037	0.04	0	3.666667
1578	15-Jun-08	2008	LT1	0.925926	0.222222	0.037037	0.04	0	3.666667

1582	15-Jun-08	2008	LT1	0.925926	0.222222	0.037037	0.04	0	3.666667
1558	15-Jun-08	2008	LT1	0.925926	0.222222	0.037037	0.04	0	3.666667
1559	15-Jun-08	2008	LT1	0.925926	0.222222	0.037037	0.04	0	3.666667
1560	15-Jun-08	2008	LT1	0.925926	0.222222	0.037037	0.04	0	3.666667
1562	15-Jun-08	2008	LT1	0.925926	0.222222	0.037037	0.04	0	3.666667
1563	15-Jun-08	2008	LT1	0.925926	0.222222	0.037037	0.04	0	3.666667
1564	15-Jun-08	2008	LT1	0.925926	0.222222	0.037037	0.04	0	3.666667
1567	15-Jun-08	2008	LT1	0.925926	0.222222	0.037037	0.04	0	3.666667
1568	15-Jun-08	2008	LT1	0.925926	0.222222	0.037037	0.04	0	3.666667
1569	15-Jun-08	2008	LT1	0.925926	0.222222	0.037037	0.04	0	3.666667
1570	15-Jun-08	2008	LT1	0.925926	0.222222	0.037037	0.04	0	3.666667
1572	15-Jun-08	2008	LT1	0.925926	0.222222	0.037037	0.04	0	3.666667
1573	15-Jun-08	2008	LT1	0.925926	0.222222	0.037037	0.04	0	3.666667
1575	15-Jun-08	2008	LT1	0.925926	0.222222	0.037037	0.04	0	3.666667
1576	15-Jun-08	2008	LT1	0.925926	0.222222	0.037037	0.04	0	3.666667
1579	15-Jun-08	2008	LT1	0.925926	0.222222	0.037037	0.04	0	3.666667
1580	15-Jun-08	2008	LT1	0.925926	0.222222	0.037037	0.04	0	3.666667
1581	15-Jun-08	2008	LT1	0.925926	0.222222	0.037037	0.04	0	3.666667
1583	15-Jun-08	2008	LT1	0.925926	0.222222	0.037037	0.04	0	3.666667
1584	15-Jun-08	2008	LT1	0.925926	0.222222	0.037037	0.04	0	3.666667
1585	15-Jun-08	2008	LT1	0.925926	0.222222	0.037037	0.04	0	3.666667
1586	15-Jun-08	2008	LT1	0.925926	0.222222	0.037037	0.04	0	3.666667
1587	15-Jun-08	2008	LT1	0.925926	0.222222	0.037037	0.04	0	3.666667
1588	15-Jun-08	2008	LT1	0.925926	0.222222	0.037037	0.04	0	3.666667
1557	15-Jun-08	2008	LT1	0.925926	0.222222	0.037037	0.04	0	3.666667
1571	15-Jun-08	2008	LT1	0.925926	0.222222	0.037037	0.04	0	3.666667
1566	15-Jun-08	2008	LT1	0.925926	0.222222	0.037037	0.04	0	3.666667
1561	15-Jun-08	2008	LT1	0.925926	0.222222	0.037037	0.04	0	3.666667
1556	15-Jun-08	2008	LT1	0.925926	0.222222	0.037037	0.04	0	3.666667
1565	15-Jun-08	2008	LT1	0.925926	0.222222	0.037037	0.04	0	3.666667
1577	15-Jun-08	2008	LT1	0.925926	0.222222	0.037037	0.04	0	3.666667
1589	15-Jun-08	2008	LT1	0.925926	0.222222	0.037037	0.04	0	3.666667
1555	15-Jun-08	2008	LT1	0.925926	0.222222	0.037037	0.04	0	3.666667
1898	23-Jun-08	2008	TO	0.2	0.2	0.05	0.025	0.00289	0.875
1900	23-Jun-08	2008	TO	0.2	0.2	0.05	0.025	0.00289	0.875
1899	23-Jun-08	2008	TO	0.2	0.2	0.05	0.025	0.00289	0.875
1901	23-Jun-08	2008	TO	0.2	0.2	0.05	0.025	0.00289	0.875
1902	23-Jun-08	2008	TO	0.2	0.2	0.05	0.025	0.00289	0.875
1903	23-Jun-08	2008	TO	0.2	0.2	0.05	0.025	0.00289	0.875
1897	23-Jun-08	2008	TO	0.2	0.2	0.05	0.025	0.00289	0.875
629	18-Jun-04	2004	DE						
630	18-Jun-04	2004	DE						
627	18-Jun-04	2004	DE						
626	18-Jun-04	2004	DE						

637	18-Jun-04	2004	DE						
638	18-Jun-04	2004	DE						
625	18-Jun-04	2004	DE						
623	18-Jun-04	2004	DE						
624	18-Jun-04	2004	DE						
628	18-Jun-04	2004	DE						
632	18-Jun-04	2004	DE						
633	18-Jun-04	2004	DE						
634	18-Jun-04	2004	DE						
635	18-Jun-04	2004	DE						
636	18-Jun-04	2004	DE						
639	18-Jun-04	2004	DE						
631	18-Jun-04	2004	DE						
307	21-Jun-04	2004	SD	0.412698	0.301587	0.063492	0.634921	0.097297	2.642857
297	21-Jun-04	2004	SD	0.412698	0.301587	0.063492	0.634921	0.097297	2.642857
305	21-Jun-04	2004	SD	0.412698	0.301587	0.063492	0.634921	0.097297	2.642857
300	21-Jun-04	2004	SD	0.412698	0.301587	0.063492	0.634921	0.097297	2.642857
301	21-Jun-04	2004	SD	0.412698	0.301587	0.063492	0.634921	0.097297	2.642857
304	21-Jun-04	2004	SD	0.412698	0.301587	0.063492	0.634921	0.097297	2.642857
302	21-Jun-04	2004	SD	0.412698	0.301587	0.063492	0.634921	0.097297	2.642857
309	21-Jun-04	2004	SD	0.412698	0.301587	0.063492	0.634921	0.097297	2.642857
308	21-Jun-04	2004	SD	0.412698	0.301587	0.063492	0.634921	0.097297	2.642857
299	21-Jun-04	2004	SD	0.412698	0.301587	0.063492	0.634921	0.097297	2.642857
303	21-Jun-04	2004	SD	0.412698	0.301587	0.063492	0.634921	0.097297	2.642857
306	21-Jun-04	2004	SD	0.412698	0.301587	0.063492	0.634921	0.097297	2.642857
310	21-Jun-04	2004	SD	0.412698	0.301587	0.063492	0.634921	0.097297	2.642857
298	21-Jun-04	2004	SD	0.412698	0.301587	0.063492	0.634921	0.097297	2.642857
805	22-Jun-04	2004	RP	0.095238	0.142857	0.095238	0.071429	0.323383	2
802	22-Jun-04	2004	RP	0.095238	0.142857	0.095238	0.071429	0.323383	2
806	22-Jun-04	2004	RP	0.095238	0.142857	0.095238	0.071429	0.323383	2
807	22-Jun-04	2004	RP	0.095238	0.142857	0.095238	0.071429	0.323383	2
804	22-Jun-04	2004	RP	0.095238	0.142857	0.095238	0.071429	0.323383	2
801	22-Jun-04	2004	RP	0.095238	0.142857	0.095238	0.071429	0.323383	2
803	22-Jun-04	2004	RP	0.095238	0.142857	0.095238	0.071429	0.323383	2
795	30-Jun-04	2004	RP	0.095238	0.142857	0.095238	0.071429	0.323383	2
793	30-Jun-04	2004	RP	0.095238	0.142857	0.095238	0.071429	0.323383	2
792	30-Jun-04	2004	RP	0.095238	0.142857	0.095238	0.071429	0.323383	2
788	30-Jun-04	2004	RP	0.095238	0.142857	0.095238	0.071429	0.323383	2
787	30-Jun-04	2004	RP	0.095238	0.142857	0.095238	0.071429	0.323383	2
791	30-Jun-04	2004	RP	0.095238	0.142857	0.095238	0.071429	0.323383	2
797	30-Jun-04	2004	RP	0.095238	0.142857	0.095238	0.071429	0.323383	2
798	30-Jun-04	2004	RP	0.095238	0.142857	0.095238	0.071429	0.323383	2
799	30-Jun-04	2004	RP	0.095238	0.142857	0.095238	0.071429	0.323383	2
800	30-Jun-04	2004	RP	0.095238	0.142857	0.095238	0.071429	0.323383	2

796	30-Jun-04	2004	RP	0.095238	0.142857	0.095238	0.071429	0.323383	2
794	30-Jun-04	2004	RP	0.095238	0.142857	0.095238	0.071429	0.323383	2
789	30-Jun-04	2004	RP	0.095238	0.142857	0.095238	0.071429	0.323383	2
790	30-Jun-04	2004	RP	0.095238	0.142857	0.095238	0.071429	0.323383	2
123	11-May-05	2005	RL	0.465517	0.103448	0	0.137931	0.108209	0.333333
125	11-May-05	2005	RL	0.465517	0.103448	0	0.137931	0.108209	0.333333
128	11-May-05	2005	RL	0.465517	0.103448	0	0.137931	0.108209	0.333333
130	11-May-05	2005	RL	0.465517	0.103448	0	0.137931	0.108209	0.333333
134	11-May-05	2005	RL	0.465517	0.103448	0	0.137931	0.108209	0.333333
120	11-May-05	2005	RL	0.465517	0.103448	0	0.137931	0.108209	0.333333
121	11-May-05	2005	RL	0.465517	0.103448	0	0.137931	0.108209	0.333333
124	11-May-05	2005	RL	0.465517	0.103448	0	0.137931	0.108209	0.333333
137	11-May-05	2005	RL	0.465517	0.103448	0	0.137931	0.108209	0.333333
118	11-May-05	2005	RL	0.465517	0.103448	0	0.137931	0.108209	0.333333
132	11-May-05	2005	RL	0.465517	0.103448	0	0.137931	0.108209	0.333333
133	11-May-05	2005	RL	0.465517	0.103448	0	0.137931	0.108209	0.333333
135	11-May-05	2005	RL	0.465517	0.103448	0	0.137931	0.108209	0.333333
136	11-May-05	2005	RL	0.465517	0.103448	0	0.137931	0.108209	0.333333
139	11-May-05	2005	RL	0.465517	0.103448	0	0.137931	0.108209	0.333333
126	11-May-05	2005	RL	0.465517	0.103448	0	0.137931	0.108209	0.333333
131	11-May-05	2005	RL	0.465517	0.103448	0	0.137931	0.108209	0.333333
119	11-May-05	2005	RL	0.465517	0.103448	0	0.137931	0.108209	0.333333
138	11-May-05	2005	RL	0.465517	0.103448	0	0.137931	0.108209	0.333333
129	11-May-05	2005	RL	0.465517	0.103448	0	0.137931	0.108209	0.333333
122	11-May-05	2005	RL	0.465517	0.103448	0	0.137931	0.108209	0.333333
127	11-May-05	2005	RL	0.465517	0.103448	0	0.137931	0.108209	0.333333
166	12-May-05	2005	DE						
168	12-May-05	2005	DE						
169	12-May-05	2005	DE						
158	12-May-05	2005	DE						
171	12-May-05	2005	DE						
174	12-May-05	2005	DE						
173	12-May-05	2005	DE						
160	12-May-05	2005	DE						
161	12-May-05	2005	DE						
163	12-May-05	2005	DE						
164	12-May-05	2005	DE						
165	12-May-05	2005	DE						
172	12-May-05	2005	DE						
175	12-May-05	2005	DE						
159	12-May-05	2005	DE						
157	12-May-05	2005	DE						
162	12-May-05	2005	DE						
167	12-May-05	2005	DE						

170	12-May-05	2005	DE							
910	29-May-05	2005	DE							
909	29-May-05	2005	DE							
907	29-May-05	2005	DE							
905	29-May-05	2005	DE							
903	29-May-05	2005	DE							
904	29-May-05	2005	DE							
902	29-May-05	2005	DE							
906	29-May-05	2005	DE							
901	29-May-05	2005	DE							
908	29-May-05	2005	DE							
911	29-May-05	2005	DE							
900	29-May-05	2005	DE							
1007	2-Jun-05	2005	RL	0.465517	0.103448		0	0.137931	0.108209	0.333333
1009	2-Jun-05	2005	RL	0.465517	0.103448		0	0.137931	0.108209	0.333333
1003	2-Jun-05	2005	RL	0.465517	0.103448		0	0.137931	0.108209	0.333333
1000	2-Jun-05	2005	RL	0.465517	0.103448		0	0.137931	0.108209	0.333333
1004	2-Jun-05	2005	RL	0.465517	0.103448		0	0.137931	0.108209	0.333333
1008	2-Jun-05	2005	RL	0.465517	0.103448		0	0.137931	0.108209	0.333333
1006	2-Jun-05	2005	RL	0.465517	0.103448		0	0.137931	0.108209	0.333333
1010	2-Jun-05	2005	RL	0.465517	0.103448		0	0.137931	0.108209	0.333333
1002	2-Jun-05	2005	RL	0.465517	0.103448		0	0.137931	0.108209	0.333333
1014	2-Jun-05	2005	RL	0.465517	0.103448		0	0.137931	0.108209	0.333333
1011	2-Jun-05	2005	RL	0.465517	0.103448		0	0.137931	0.108209	0.333333
1013	2-Jun-05	2005	RL	0.465517	0.103448		0	0.137931	0.108209	0.333333
1001	2-Jun-05	2005	RL	0.465517	0.103448		0	0.137931	0.108209	0.333333
1005	2-Jun-05	2005	RL	0.465517	0.103448		0	0.137931	0.108209	0.333333
1012	2-Jun-05	2005	RL	0.465517	0.103448		0	0.137931	0.108209	0.333333
999	2-Jun-05	2005	RL	0.465517	0.103448		0	0.137931	0.108209	0.333333
1017	2-Jun-05	2005	RL	0.465517	0.103448		0	0.137931	0.108209	0.333333
1016	2-Jun-05	2005	RL	0.465517	0.103448		0	0.137931	0.108209	0.333333
1015	2-Jun-05	2005	RL	0.465517	0.103448		0	0.137931	0.108209	0.333333
1018	2-Jun-05	2005	RL	0.465517	0.103448		0	0.137931	0.108209	0.333333
1019	2-Jun-05	2005	RL	0.465517	0.103448		0	0.137931	0.108209	0.333333
1021	2-Jun-05	2005	RL	0.465517	0.103448		0	0.137931	0.108209	0.333333
1020	2-Jun-05	2005	RL	0.465517	0.103448		0	0.137931	0.108209	0.333333
1358	5-Jun-05	2005	SHAC	0.076923	0.153846		0	0.384615	0.026042	2.090909
1357	5-Jun-05	2005	SHAC	0.076923	0.153846		0	0.384615	0.026042	2.090909
1356	5-Jun-05	2005	SHAC	0.076923	0.153846		0	0.384615	0.026042	2.090909
1355	5-Jun-05	2005	SHAC	0.076923	0.153846		0	0.384615	0.026042	2.090909
1351	5-Jun-05	2005	SHAC	0.076923	0.153846		0	0.384615	0.026042	2.090909
1359	5-Jun-05	2005	SHAC	0.076923	0.153846		0	0.384615	0.026042	2.090909
1346	5-Jun-05	2005	SHAC	0.076923	0.153846		0	0.384615	0.026042	2.090909
1353	5-Jun-05	2005	SHAC	0.076923	0.153846		0	0.384615	0.026042	2.090909

1344	5-Jun-05	2005	SHAC	0.076923	0.153846	0	0.384615	0.026042	2.090909
1345	5-Jun-05	2005	SHAC	0.076923	0.153846	0	0.384615	0.026042	2.090909
1347	5-Jun-05	2005	SHAC	0.076923	0.153846	0	0.384615	0.026042	2.090909
1352	5-Jun-05	2005	SHAC	0.076923	0.153846	0	0.384615	0.026042	2.090909
1354	5-Jun-05	2005	SHAC	0.076923	0.153846	0	0.384615	0.026042	2.090909
1348	5-Jun-05	2005	SHAC	0.076923	0.153846	0	0.384615	0.026042	2.090909
1350	5-Jun-05	2005	SHAC	0.076923	0.153846	0	0.384615	0.026042	2.090909
1349	5-Jun-05	2005	SHAC	0.076923	0.153846	0	0.384615	0.026042	2.090909
1343	5-Jun-05	2005	SHAC	0.076923	0.153846	0	0.384615	0.026042	2.090909
1593	23-Jun-05	2005	MM	0.220779	0.064935	0.025974	0.12987	0.044118	1.727273
1589	23-Jun-05	2005	MM	0.220779	0.064935	0.025974	0.12987	0.044118	1.727273
1592	23-Jun-05	2005	MM	0.220779	0.064935	0.025974	0.12987	0.044118	1.727273
1594	23-Jun-05	2005	MM	0.220779	0.064935	0.025974	0.12987	0.044118	1.727273
1590	23-Jun-05	2005	MM	0.220779	0.064935	0.025974	0.12987	0.044118	1.727273
1591	23-Jun-05	2005	MM	0.220779	0.064935	0.025974	0.12987	0.044118	1.727273
1588	23-Jun-05	2005	MM	0.220779	0.064935	0.025974	0.12987	0.044118	1.727273
1595	23-Jun-05	2005	MM	0.220779	0.064935	0.025974	0.12987	0.044118	1.727273
1586	23-Jun-05	2005	MM	0.220779	0.064935	0.025974	0.12987	0.044118	1.727273
1577	23-Jun-05	2005	MM	0.220779	0.064935	0.025974	0.12987	0.044118	1.727273
1576	23-Jun-05	2005	MM	0.220779	0.064935	0.025974	0.12987	0.044118	1.727273
1575	23-Jun-05	2005	MM	0.220779	0.064935	0.025974	0.12987	0.044118	1.727273
1581	23-Jun-05	2005	MM	0.220779	0.064935	0.025974	0.12987	0.044118	1.727273
1572	23-Jun-05	2005	MM	0.220779	0.064935	0.025974	0.12987	0.044118	1.727273
1574	23-Jun-05	2005	MM	0.220779	0.064935	0.025974	0.12987	0.044118	1.727273
1579	23-Jun-05	2005	MM	0.220779	0.064935	0.025974	0.12987	0.044118	1.727273
1582	23-Jun-05	2005	MM	0.220779	0.064935	0.025974	0.12987	0.044118	1.727273
1583	23-Jun-05	2005	MM	0.220779	0.064935	0.025974	0.12987	0.044118	1.727273
1584	23-Jun-05	2005	MM	0.220779	0.064935	0.025974	0.12987	0.044118	1.727273
1585	23-Jun-05	2005	MM	0.220779	0.064935	0.025974	0.12987	0.044118	1.727273
1580	23-Jun-05	2005	MM	0.220779	0.064935	0.025974	0.12987	0.044118	1.727273
1573	23-Jun-05	2005	MM	0.220779	0.064935	0.025974	0.12987	0.044118	1.727273
1578	23-Jun-05	2005	MM	0.220779	0.064935	0.025974	0.12987	0.044118	1.727273
1587	23-Jun-05	2005	MM	0.220779	0.064935	0.025974	0.12987	0.044118	1.727273
160	1-Jun-06	2006	FO	0.157895	0.131579	0.052632	0.157895	0.015152	0.066667
163	1-Jun-06	2006	FO	0.157895	0.131579	0.052632	0.157895	0.015152	0.066667
165	1-Jun-06	2006	FO	0.157895	0.131579	0.052632	0.157895	0.015152	0.066667
161	1-Jun-06	2006	FO	0.157895	0.131579	0.052632	0.157895	0.015152	0.066667
159	1-Jun-06	2006	FO	0.157895	0.131579	0.052632	0.157895	0.015152	0.066667
162	1-Jun-06	2006	FO	0.157895	0.131579	0.052632	0.157895	0.015152	0.066667
164	1-Jun-06	2006	FO	0.157895	0.131579	0.052632	0.157895	0.015152	0.066667
251	10-Jun-06	2006	HH	0.102941	0.161765	0	0.352941	0	0.615385
258	10-Jun-06	2006	HH	0.102941	0.161765	0	0.352941	0	0.615385
250	10-Jun-06	2006	HH	0.102941	0.161765	0	0.352941	0	0.615385
248	10-Jun-06	2006	HH	0.102941	0.161765	0	0.352941	0	0.615385

252	10-Jun-06	2006	HH	0.102941	0.161765	0	0.352941	0	0.615385
254	10-Jun-06	2006	HH	0.102941	0.161765	0	0.352941	0	0.615385
255	10-Jun-06	2006	HH	0.102941	0.161765	0	0.352941	0	0.615385
256	10-Jun-06	2006	HH	0.102941	0.161765	0	0.352941	0	0.615385
249	10-Jun-06	2006	HH	0.102941	0.161765	0	0.352941	0	0.615385
253	10-Jun-06	2006	HH	0.102941	0.161765	0	0.352941	0	0.615385
257	10-Jun-06	2006	HH	0.102941	0.161765	0	0.352941	0	0.615385
	16-Jun-06	2006	RC	0.2	0	0.1	0.15	0.033898	3
	16-Jun-06	2006	RC	0.2	0	0.1	0.15	0.033898	3
	16-Jun-06	2006	RC	0.2	0	0.1	0.15	0.033898	3
	16-Jun-06	2006	RC	0.2	0	0.1	0.15	0.033898	3
	16-Jun-06	2006	RC	0.2	0	0.1	0.15	0.033898	3
	16-Jun-06	2006	RC	0.2	0	0.1	0.15	0.033898	3
	16-Jun-06	2006	RC	0.2	0	0.1	0.15	0.033898	3
	16-Jun-06	2006	RC	0.2	0	0.1	0.15	0.033898	3
	16-Jun-06	2006	RC	0.2	0	0.1	0.15	0.033898	3
	16-Jun-06	2006	RC	0.2	0	0.1	0.15	0.033898	3
	16-Jun-06	2006	RC	0.2	0	0.1	0.15	0.033898	3
	16-Jun-06	2006	RC	0.2	0	0.1	0.15	0.033898	3
	16-Jun-06	2006	RC	0.2	0	0.1	0.15	0.033898	3
	16-Jun-06	2006	RC	0.2	0	0.1	0.15	0.033898	3
	16-Jun-06	2006	RC	0.2	0	0.1	0.15	0.033898	3
	16-Jun-06	2006	RC	0.2	0	0.1	0.15	0.033898	3
	16-Jun-06	2006	RC	0.2	0	0.1	0.15	0.033898	3
	16-Jun-06	2006	RC	0.2	0	0.1	0.15	0.033898	3
	24-Jun-06	2006	TO	0.075	0.1	0.05	0.225	0.005814	0.9375
	24-Jun-06	2006	TO	0.075	0.1	0.05	0.225	0.005814	0.9375
	24-Jun-06	2006	TO	0.075	0.1	0.05	0.225	0.005814	0.9375
	24-Jun-06	2006	TO	0.075	0.1	0.05	0.225	0.005814	0.9375
	24-Jun-06	2006	TO	0.075	0.1	0.05	0.225	0.005814	0.9375
	24-Jun-06	2006	TO	0.075	0.1	0.05	0.225	0.005814	0.9375
	24-Jun-06	2006	TO	0.075	0.1	0.05	0.225	0.005814	0.9375
	24-Jun-06	2006	TO	0.075	0.1	0.05	0.225	0.005814	0.9375
	24-Jun-06	2006	TO	0.075	0.1	0.05	0.225	0.005814	0.9375
	24-Jun-06	2006	TO	0.075	0.1	0.05	0.225	0.005814	0.9375
	24-Jun-06	2006	TO	0.075	0.1	0.05	0.225	0.005814	0.9375
	24-Jun-06	2006	TO	0.075	0.1	0.05	0.225	0.005814	0.9375
	24-Jun-06	2006	TO	0.075	0.1	0.05	0.225	0.005814	0.9375
	24-Jun-06	2006	TO	0.075	0.1	0.05	0.225	0.005814	0.9375
	24-Jun-06	2006	TO	0.075	0.1	0.05	0.225	0.005814	0.9375
	24-Jun-06	2006	TO	0.075	0.1	0.05	0.225	0.005814	0.9375
	25-Jun-06	2006	LT1	0.222222	0.111111	0	0.148148	0	4
	25-Jun-06	2006	LT1	0.222222	0.111111	0	0.148148	0	4
	25-Jun-06	2006	LT1	0.222222	0.111111	0	0.148148	0	4

	25-Jun-06	2006	LT1	0.222222	0.111111	0	0.148148	0	4
	25-Jun-06	2006	LT1	0.222222	0.111111	0	0.148148	0	4
	25-Jun-06	2006	LT1	0.222222	0.111111	0	0.148148	0	4
	25-Jun-06	2006	LT1	0.222222	0.111111	0	0.148148	0	4
	25-Jun-06	2006	LT1	0.222222	0.111111	0	0.148148	0	4
	25-Jun-06	2006	LT1	0.222222	0.111111	0	0.148148	0	4
	25-Jun-06	2006	LT1	0.222222	0.111111	0	0.148148	0	4
	25-Jun-06	2006	LT1	0.222222	0.111111	0	0.148148	0	4
	25-Jun-06	2006	LT1	0.222222	0.111111	0	0.148148	0	4
	28-Jun-06	2006	HH	0.102941	0.161765	0	0.352941	0	0.615385
	28-Jun-06	2006	HH	0.102941	0.161765	0	0.352941	0	0.615385
	28-Jun-06	2006	HH	0.102941	0.161765	0	0.352941	0	0.615385
	28-Jun-06	2006	HH	0.102941	0.161765	0	0.352941	0	0.615385
	28-Jun-06	2006	HH	0.102941	0.161765	0	0.352941	0	0.615385
	28-Jun-06	2006	HH	0.102941	0.161765	0	0.352941	0	0.615385
	28-Jun-06	2006	HH	0.102941	0.161765	0	0.352941	0	0.615385
	28-Jun-06	2006	HH	0.102941	0.161765	0	0.352941	0	0.615385
	28-Jun-06	2006	HH	0.102941	0.161765	0	0.352941	0	0.615385
	28-Jun-06	2006	HH	0.102941	0.161765	0	0.352941	0	0.615385
	28-Jun-06	2006	HH	0.102941	0.161765	0	0.352941	0	0.615385
	28-Jun-06	2006	HH	0.102941	0.161765	0	0.352941	0	0.615385
456	22-May-08	2008	7M	0.078947	0.342105	0.078947	0.263158	0.01023	7.733333
455	22-May-08	2008	7M	0.078947	0.342105	0.078947	0.263158	0.01023	7.733333
454	22-May-08	2008	7M	0.078947	0.342105	0.078947	0.263158	0.01023	7.733333
451	22-May-08	2008	7M	0.078947	0.342105	0.078947	0.263158	0.01023	7.733333
452	22-May-08	2008	7M	0.078947	0.342105	0.078947	0.263158	0.01023	7.733333
450	22-May-08	2008	7M	0.078947	0.342105	0.078947	0.263158	0.01023	7.733333
446	22-May-08	2008	7M	0.078947	0.342105	0.078947	0.263158	0.01023	7.733333
445	22-May-08	2008	7M	0.078947	0.342105	0.078947	0.263158	0.01023	7.733333
447	22-May-08	2008	7M	0.078947	0.342105	0.078947	0.263158	0.01023	7.733333
449	22-May-08	2008	7M	0.078947	0.342105	0.078947	0.263158	0.01023	7.733333
457	22-May-08	2008	7M	0.078947	0.342105	0.078947	0.263158	0.01023	7.733333
443	22-May-08	2008	7M	0.078947	0.342105	0.078947	0.263158	0.01023	7.733333
444	22-May-08	2008	7M	0.078947	0.342105	0.078947	0.263158	0.01023	7.733333
448	22-May-08	2008	7M	0.078947	0.342105	0.078947	0.263158	0.01023	7.733333
453	22-May-08	2008	7M	0.078947	0.342105	0.078947	0.263158	0.01023	7.733333
458	22-May-08	2008	7M	0.078947	0.342105	0.078947	0.263158	0.01023	7.733333
442	22-May-08	2008	7M	0.078947	0.342105	0.078947	0.263158	0.01023	7.733333
1022	30-May-08	2008	ML						
1019	30-May-08	2008	ML						
1025	30-May-08	2008	ML						
1023	30-May-08	2008	ML						
1011	30-May-08	2008	ML						
1024	30-May-08	2008	ML						

1012	30-May-08	2008	ML						
1013	30-May-08	2008	ML						
1017	30-May-08	2008	ML						
1020	30-May-08	2008	ML						
1016	30-May-08	2008	ML						
1018	30-May-08	2008	ML						
1026	30-May-08	2008	ML						
1027	30-May-08	2008	ML						
1014	30-May-08	2008	ML						
1010	30-May-08	2008	ML						
1015	30-May-08	2008	ML						
1021	30-May-08	2008	ML						
1029	30-May-08	2008	ML						
1028	30-May-08	2008	ML						
1009	30-May-08	2008	ML						
1428	10-Jun-08	2008	TO	0.2	0.2	0.05	0.025	0.00289	0.875
1434	10-Jun-08	2008	TO	0.2	0.2	0.05	0.025	0.00289	0.875
1435	10-Jun-08	2008	TO	0.2	0.2	0.05	0.025	0.00289	0.875
1436	10-Jun-08	2008	TO	0.2	0.2	0.05	0.025	0.00289	0.875
1421	10-Jun-08	2008	TO	0.2	0.2	0.05	0.025	0.00289	0.875
1422	10-Jun-08	2008	TO	0.2	0.2	0.05	0.025	0.00289	0.875
1424	10-Jun-08	2008	TO	0.2	0.2	0.05	0.025	0.00289	0.875
1429	10-Jun-08	2008	TO	0.2	0.2	0.05	0.025	0.00289	0.875
1420	10-Jun-08	2008	TO	0.2	0.2	0.05	0.025	0.00289	0.875
1425	10-Jun-08	2008	TO	0.2	0.2	0.05	0.025	0.00289	0.875
1426	10-Jun-08	2008	TO	0.2	0.2	0.05	0.025	0.00289	0.875
1430	10-Jun-08	2008	TO	0.2	0.2	0.05	0.025	0.00289	0.875
1431	10-Jun-08	2008	TO	0.2	0.2	0.05	0.025	0.00289	0.875
1423	10-Jun-08	2008	TO	0.2	0.2	0.05	0.025	0.00289	0.875
1432	10-Jun-08	2008	TO	0.2	0.2	0.05	0.025	0.00289	0.875
1433	10-Jun-08	2008	TO	0.2	0.2	0.05	0.025	0.00289	0.875
1446	10-Jun-08	2008	TO	0.2	0.2	0.05	0.025	0.00289	0.875
1445	10-Jun-08	2008	TO	0.2	0.2	0.05	0.025	0.00289	0.875
1447	10-Jun-08	2008	TO	0.2	0.2	0.05	0.025	0.00289	0.875
1443	10-Jun-08	2008	TO	0.2	0.2	0.05	0.025	0.00289	0.875
1442	10-Jun-08	2008	TO	0.2	0.2	0.05	0.025	0.00289	0.875
1448	10-Jun-08	2008	TO	0.2	0.2	0.05	0.025	0.00289	0.875
1437	10-Jun-08	2008	TO	0.2	0.2	0.05	0.025	0.00289	0.875
1438	10-Jun-08	2008	TO	0.2	0.2	0.05	0.025	0.00289	0.875
1439	10-Jun-08	2008	TO	0.2	0.2	0.05	0.025	0.00289	0.875
1441	10-Jun-08	2008	TO	0.2	0.2	0.05	0.025	0.00289	0.875
1449	10-Jun-08	2008	TO	0.2	0.2	0.05	0.025	0.00289	0.875
1450	10-Jun-08	2008	TO	0.2	0.2	0.05	0.025	0.00289	0.875
1451	10-Jun-08	2008	TO	0.2	0.2	0.05	0.025	0.00289	0.875

1456	10-Jun-08	2008	TO	0.2	0.2	0.05	0.025	0.00289	0.875
1452	10-Jun-08	2008	TO	0.2	0.2	0.05	0.025	0.00289	0.875
1455	10-Jun-08	2008	TO	0.2	0.2	0.05	0.025	0.00289	0.875
1457	10-Jun-08	2008	TO	0.2	0.2	0.05	0.025	0.00289	0.875
1444	10-Jun-08	2008	TO	0.2	0.2	0.05	0.025	0.00289	0.875
1453	10-Jun-08	2008	TO	0.2	0.2	0.05	0.025	0.00289	0.875
1458	10-Jun-08	2008	TO	0.2	0.2	0.05	0.025	0.00289	0.875
1454	10-Jun-08	2008	TO	0.2	0.2	0.05	0.025	0.00289	0.875
1459	10-Jun-08	2008	TO	0.2	0.2	0.05	0.025	0.00289	0.875
1419	10-Jun-08	2008	TO	0.2	0.2	0.05	0.025	0.00289	0.875
1418	10-Jun-08	2008	TO	0.2	0.2	0.05	0.025	0.00289	0.875
1427	10-Jun-08	2008	TO	0.2	0.2	0.05	0.025	0.00289	0.875
1440	10-Jun-08	2008	TO	0.2	0.2	0.05	0.025	0.00289	0.875
1460	10-Jun-08	2008	TO	0.2	0.2	0.05	0.025	0.00289	0.875
1417	10-Jun-08	2008	TO	0.2	0.2	0.05	0.025	0.00289	0.875
1738	16-Jun-08	2008	ML						
1739	16-Jun-08	2008	ML						
1741	16-Jun-08	2008	ML						
1731	16-Jun-08	2008	ML						
1734	16-Jun-08	2008	ML						
1735	16-Jun-08	2008	ML						
1742	16-Jun-08	2008	ML						
1744	16-Jun-08	2008	ML						
1745	16-Jun-08	2008	ML						
1746	16-Jun-08	2008	ML						
1747	16-Jun-08	2008	ML						
1748	16-Jun-08	2008	ML						
1749	16-Jun-08	2008	ML						
1732	16-Jun-08	2008	ML						
1736	16-Jun-08	2008	ML						
1743	16-Jun-08	2008	ML						
1733	16-Jun-08	2008	ML						
1737	16-Jun-08	2008	ML						
1740	16-Jun-08	2008	ML						
1750	16-Jun-08	2008	ML						
1730	16-Jun-08	2008	ML						
1863	23-Jun-08	2008	TO	0.2	0.2	0.05	0.025	0.00289	0.875
1864	23-Jun-08	2008	TO	0.2	0.2	0.05	0.025	0.00289	0.875
1865	23-Jun-08	2008	TO	0.2	0.2	0.05	0.025	0.00289	0.875
1866	23-Jun-08	2008	TO	0.2	0.2	0.05	0.025	0.00289	0.875
1867	23-Jun-08	2008	TO	0.2	0.2	0.05	0.025	0.00289	0.875
1868	23-Jun-08	2008	TO	0.2	0.2	0.05	0.025	0.00289	0.875
1862	23-Jun-08	2008	TO	0.2	0.2	0.05	0.025	0.00289	0.875
1872	23-Jun-08	2008	TO	0.2	0.2	0.05	0.025	0.00289	0.875

1861	23-Jun-08	2008 TO	0.2	0.2	0.05	0.025	0.00289	0.875
1871	23-Jun-08	2008 TO	0.2	0.2	0.05	0.025	0.00289	0.875
1860	23-Jun-08	2008 TO	0.2	0.2	0.05	0.025	0.00289	0.875
1869	23-Jun-08	2008 TO	0.2	0.2	0.05	0.025	0.00289	0.875
1870	23-Jun-08	2008 TO	0.2	0.2	0.05	0.025	0.00289	0.875
1873	23-Jun-08	2008 TO	0.2	0.2	0.05	0.025	0.00289	0.875
1859	23-Jun-08	2008 TO	0.2	0.2	0.05	0.025	0.00289	0.875
1879	23-Jun-08	2008 TO	0.2	0.2	0.05	0.025	0.00289	0.875
1877	23-Jun-08	2008 TO	0.2	0.2	0.05	0.025	0.00289	0.875
1878	23-Jun-08	2008 TO	0.2	0.2	0.05	0.025	0.00289	0.875
1880	23-Jun-08	2008 TO	0.2	0.2	0.05	0.025	0.00289	0.875
1881	23-Jun-08	2008 TO	0.2	0.2	0.05	0.025	0.00289	0.875
1886	23-Jun-08	2008 TO	0.2	0.2	0.05	0.025	0.00289	0.875
1887	23-Jun-08	2008 TO	0.2	0.2	0.05	0.025	0.00289	0.875
1892	23-Jun-08	2008 TO	0.2	0.2	0.05	0.025	0.00289	0.875
1894	23-Jun-08	2008 TO	0.2	0.2	0.05	0.025	0.00289	0.875
1883	23-Jun-08	2008 TO	0.2	0.2	0.05	0.025	0.00289	0.875
1890	23-Jun-08	2008 TO	0.2	0.2	0.05	0.025	0.00289	0.875
1891	23-Jun-08	2008 TO	0.2	0.2	0.05	0.025	0.00289	0.875
1893	23-Jun-08	2008 TO	0.2	0.2	0.05	0.025	0.00289	0.875
1884	23-Jun-08	2008 TO	0.2	0.2	0.05	0.025	0.00289	0.875
1885	23-Jun-08	2008 TO	0.2	0.2	0.05	0.025	0.00289	0.875
1889	23-Jun-08	2008 TO	0.2	0.2	0.05	0.025	0.00289	0.875
1888	23-Jun-08	2008 TO	0.2	0.2	0.05	0.025	0.00289	0.875
1895	23-Jun-08	2008 TO	0.2	0.2	0.05	0.025	0.00289	0.875
1875	23-Jun-08	2008 TO	0.2	0.2	0.05	0.025	0.00289	0.875
1876	23-Jun-08	2008 TO	0.2	0.2	0.05	0.025	0.00289	0.875
1882	23-Jun-08	2008 TO	0.2	0.2	0.05	0.025	0.00289	0.875
1896	23-Jun-08	2008 TO	0.2	0.2	0.05	0.025	0.00289	0.875
1874	23-Jun-08	2008 TO	0.2	0.2	0.05	0.025	0.00289	0.875
		2007 TO	0.175	0.125	0.175	0	0.00289	0.875
		2007 TO	0.175	0.125	0.175	0	0.00289	0.875
		2007 TO	0.175	0.125	0.175	0	0.00289	0.875
		2007 TO	0.175	0.125	0.175	0	0.00289	0.875
		2007 TO	0.175	0.125	0.175	0	0.00289	0.875
		2007 TO	0.175	0.125	0.175	0	0.00289	0.875
		2007 TO	0.175	0.125	0.175	0	0.00289	0.875
		2007 TO	0.175	0.125	0.175	0	0.00289	0.875
		2007 TO	0.175	0.125	0.175	0	0.00289	0.875
		2007 TO	0.175	0.125	0.175	0	0.00289	0.875
		2007 TO	0.175	0.125	0.175	0	0.00289	0.875
		2007 TO	0.175	0.125	0.175	0	0.00289	0.875
		2007 TO	0.175	0.125	0.175	0	0.00289	0.875
		2007 TO	0.175	0.125	0.175	0	0.00289	0.875

		2007	TO	0.175	0.125	0.175	0	0.00289	0.875
		2007	TO	0.175	0.125	0.175	0	0.00289	0.875
1219	21-May-07	2007	D2	0.181818	0.254545	0	0.145455	0.005814	3
1217	21-May-07	2007	D2	0.181818	0.254545	0	0.145455	0.005814	3
1218	21-May-07	2007	D2	0.181818	0.254545	0	0.145455	0.005814	3
1216	21-May-07	2007	D2	0.181818	0.254545	0	0.145455	0.005814	3
1220	21-May-07	2007	D2	0.181818	0.254545	0	0.145455	0.005814	3
1221	21-May-07	2007	D2	0.181818	0.254545	0	0.145455	0.005814	3
1215	21-May-07	2007	D2	0.181818	0.254545	0	0.145455	0.005814	3
1214	21-May-07	2007	D2	0.181818	0.254545	0	0.145455	0.005814	3
1213	21-May-07	2007	D2	0.181818	0.254545	0	0.145455	0.005814	3
1212	21-May-07	2007	D2	0.181818	0.254545	0	0.145455	0.005814	3
1211	21-May-07	2007	D2	0.181818	0.254545	0	0.145455	0.005814	3
1239	21-May-07	2007	D2	0.181818	0.254545	0	0.145455	0.005814	3
1248	21-May-07	2007	D2	0.181818	0.254545	0	0.145455	0.005814	3
1250	21-May-07	2007	D2	0.181818	0.254545	0	0.145455	0.005814	3
1235	21-May-07	2007	D2	0.181818	0.254545	0	0.145455	0.005814	3
1234	21-May-07	2007	D2	0.181818	0.254545	0	0.145455	0.005814	3
1232	21-May-07	2007	D2	0.181818	0.254545	0	0.145455	0.005814	3
1224	21-May-07	2007	D2	0.181818	0.254545	0	0.145455	0.005814	3
1226	21-May-07	2007	D2	0.181818	0.254545	0	0.145455	0.005814	3
1230	21-May-07	2007	D2	0.181818	0.254545	0	0.145455	0.005814	3
1229	21-May-07	2007	D2	0.181818	0.254545	0	0.145455	0.005814	3
1223	21-May-07	2007	D2	0.181818	0.254545	0	0.145455	0.005814	3
1233	21-May-07	2007	D2	0.181818	0.254545	0	0.145455	0.005814	3
1225	21-May-07	2007	D2	0.181818	0.254545	0	0.145455	0.005814	3
1227	21-May-07	2007	D2	0.181818	0.254545	0	0.145455	0.005814	3
1236	21-May-07	2007	D2	0.181818	0.254545	0	0.145455	0.005814	3
1237	21-May-07	2007	D2	0.181818	0.254545	0	0.145455	0.005814	3
1228	21-May-07	2007	D2	0.181818	0.254545	0	0.145455	0.005814	3
1245	21-May-07	2007	D2	0.181818	0.254545	0	0.145455	0.005814	3
1246	21-May-07	2007	D2	0.181818	0.254545	0	0.145455	0.005814	3
1242	21-May-07	2007	D2	0.181818	0.254545	0	0.145455	0.005814	3
1244	21-May-07	2007	D2	0.181818	0.254545	0	0.145455	0.005814	3
1241	21-May-07	2007	D2	0.181818	0.254545	0	0.145455	0.005814	3
1247	21-May-07	2007	D2	0.181818	0.254545	0	0.145455	0.005814	3
1243	21-May-07	2007	D2	0.181818	0.254545	0	0.145455	0.005814	3
1240	21-May-07	2007	D2	0.181818	0.254545	0	0.145455	0.005814	3
1249	21-May-07	2007	D2	0.181818	0.254545	0	0.145455	0.005814	3
1251	21-May-07	2007	D2	0.181818	0.254545	0	0.145455	0.005814	3
1252	21-May-07	2007	D2	0.181818	0.254545	0	0.145455	0.005814	3
1253	21-May-07	2007	D2	0.181818	0.254545	0	0.145455	0.005814	3
1254	21-May-07	2007	D2	0.181818	0.254545	0	0.145455	0.005814	3
1255	21-May-07	2007	D2	0.181818	0.254545	0	0.145455	0.005814	3

1231	21-May-07	2007	D2	0.181818	0.254545	0	0.145455	0.005814	3
1210	21-May-07	2007	D2	0.181818	0.254545	0	0.145455	0.005814	3
1222	21-May-07	2007	D2	0.181818	0.254545	0	0.145455	0.005814	3
1238	21-May-07	2007	D2	0.181818	0.254545	0	0.145455	0.005814	3
1053	5-Jun-05	2005	7M	0.264151	0.09434	0	0.301887	0.01519	8.266667
1052	5-Jun-05	2005	7M	0.264151	0.09434	0	0.301887	0.01519	8.266667
1054	5-Jun-05	2005	7M	0.264151	0.09434	0	0.301887	0.01519	8.266667
1050	5-Jun-05	2005	7M	0.264151	0.09434	0	0.301887	0.01519	8.266667
1051	5-Jun-05	2005	7M	0.264151	0.09434	0	0.301887	0.01519	8.266667
1056	5-Jun-05	2005	7M	0.264151	0.09434	0	0.301887	0.01519	8.266667
1055	5-Jun-05	2005	7M	0.264151	0.09434	0	0.301887	0.01519	8.266667
1057	5-Jun-05	2005	7M	0.264151	0.09434	0	0.301887	0.01519	8.266667
1058	5-Jun-05	2005	7M	0.264151	0.09434	0	0.301887	0.01519	8.266667
1059	5-Jun-05	2005	7M	0.264151	0.09434	0	0.301887	0.01519	8.266667
1060	5-Jun-05	2005	7M	0.264151	0.09434	0	0.301887	0.01519	8.266667
1635	13-Jun-05	2005	SW	0.264151	0.113208	0.018868	0.169811	0.044444	1.4
1634	13-Jun-05	2005	SW	0.264151	0.113208	0.018868	0.169811	0.044444	1.4
1636	13-Jun-05	2005	SW	0.264151	0.113208	0.018868	0.169811	0.044444	1.4
1633	13-Jun-05	2005	SW	0.264151	0.113208	0.018868	0.169811	0.044444	1.4
1637	13-Jun-05	2005	SW	0.264151	0.113208	0.018868	0.169811	0.044444	1.4
1638	13-Jun-05	2005	SW	0.264151	0.113208	0.018868	0.169811	0.044444	1.4
1639	13-Jun-05	2005	SW	0.264151	0.113208	0.018868	0.169811	0.044444	1.4
1640	13-Jun-05	2005	SW	0.264151	0.113208	0.018868	0.169811	0.044444	1.4
1641	13-Jun-05	2005	SW	0.264151	0.113208	0.018868	0.169811	0.044444	1.4
1	19-May-04	2004	SW	0.226415	0.245283	0.056604	0.09434	0.157658	1.4
2	19-May-04	2004	SW	0.226415	0.245283	0.056604	0.09434	0.157658	1.4
3	19-May-04	2004	SW	0.226415	0.245283	0.056604	0.09434	0.157658	1.4
4	19-May-04	2004	SW	0.226415	0.245283	0.056604	0.09434	0.157658	1.4
22	19-May-04	2004	MG	0.405797	0.144928	0.028986	0.275362	0.054326	5.45
23	19-May-04	2004	MG	0.405797	0.144928	0.028986	0.275362	0.054326	5.45
26	19-May-04	2004	MG	0.405797	0.144928	0.028986	0.275362	0.054326	5.45
24	19-May-04	2004	MG	0.405797	0.144928	0.028986	0.275362	0.054326	5.45
25	19-May-04	2004	MG	0.405797	0.144928	0.028986	0.275362	0.054326	5.45
28	19-May-04	2004	MG	0.405797	0.144928	0.028986	0.275362	0.054326	5.45
29	19-May-04	2004	MG	0.405797	0.144928	0.028986	0.275362	0.054326	5.45
30	19-May-04	2004	MG	0.405797	0.144928	0.028986	0.275362	0.054326	5.45
27	19-May-04	2004	MG	0.405797	0.144928	0.028986	0.275362	0.054326	5.45
31	19-May-04	2004	MG	0.405797	0.144928	0.028986	0.275362	0.054326	5.45
32	20-May-04	2004	DE						
33	20-May-04	2004	DE						
34	20-May-04	2004	DE						
35	21-May-04	2004	DE						
36	21-May-04	2004	DE						
37	21-May-04	2004	DE						

62	23-May-04	2004	RL	0.396226	0.113208	0.09434	0.320755	0.203008	0.333333
64	23-May-04	2004	RL	0.396226	0.113208	0.09434	0.320755	0.203008	0.333333
63	23-May-04	2004	RL	0.396226	0.113208	0.09434	0.320755	0.203008	0.333333
167	28-May-04	2004	RC	0.428571	0.107143	0	0	0.401709	3
168	28-May-04	2004	RC	0.428571	0.107143	0	0	0.401709	3
163	28-May-04	2004	RC	0.428571	0.107143	0	0	0.401709	3
165	28-May-04	2004	RC	0.428571	0.107143	0	0	0.401709	3
166	28-May-04	2004	RC	0.428571	0.107143	0	0	0.401709	3
164	28-May-04	2004	RC	0.428571	0.107143	0	0	0.401709	3
162	28-May-04	2004	RC	0.428571	0.107143	0	0	0.401709	3
161	28-May-04	2004	RC	0.428571	0.107143	0	0	0.401709	3
202	29-May-04	2004	RP	0.095238	0.142857	0.095238	0.071429	0.323383	2
206	29-May-04	2004	RP	0.095238	0.142857	0.095238	0.071429	0.323383	2
205	29-May-04	2004	RP	0.095238	0.142857	0.095238	0.071429	0.323383	2
204	29-May-04	2004	RP	0.095238	0.142857	0.095238	0.071429	0.323383	2
207	29-May-04	2004	RP	0.095238	0.142857	0.095238	0.071429	0.323383	2
208	29-May-04	2004	RP	0.095238	0.142857	0.095238	0.071429	0.323383	2
203	29-May-04	2004	RP	0.095238	0.142857	0.095238	0.071429	0.323383	2
201	29-May-04	2004	RP	0.095238	0.142857	0.095238	0.071429	0.323383	2
357	30-May-04	2004	SD	0.412698	0.301587	0.063492	0.634921	0.097297	2.642857
420	2-Jun-04	2004	7M	0.283019	0.471698	0.113208	0.679245	0.035088	8.333333
424	2-Jun-04	2004	7M	0.283019	0.471698	0.113208	0.679245	0.035088	8.333333
419	2-Jun-04	2004	7M	0.283019	0.471698	0.113208	0.679245	0.035088	8.333333
422	2-Jun-04	2004	7M	0.283019	0.471698	0.113208	0.679245	0.035088	8.333333
425	2-Jun-04	2004	7M	0.283019	0.471698	0.113208	0.679245	0.035088	8.333333
421	2-Jun-04	2004	7M	0.283019	0.471698	0.113208	0.679245	0.035088	8.333333
423	2-Jun-04	2004	7M	0.283019	0.471698	0.113208	0.679245	0.035088	8.333333
431	2-Jun-04	2004	7M	0.283019	0.471698	0.113208	0.679245	0.035088	8.333333
434	2-Jun-04	2004	7M	0.283019	0.471698	0.113208	0.679245	0.035088	8.333333
436	2-Jun-04	2004	7M	0.283019	0.471698	0.113208	0.679245	0.035088	8.333333
428	2-Jun-04	2004	7M	0.283019	0.471698	0.113208	0.679245	0.035088	8.333333
429	2-Jun-04	2004	7M	0.283019	0.471698	0.113208	0.679245	0.035088	8.333333
427	2-Jun-04	2004	7M	0.283019	0.471698	0.113208	0.679245	0.035088	8.333333
426	2-Jun-04	2004	7M	0.283019	0.471698	0.113208	0.679245	0.035088	8.333333
430	2-Jun-04	2004	7M	0.283019	0.471698	0.113208	0.679245	0.035088	8.333333
432	2-Jun-04	2004	7M	0.283019	0.471698	0.113208	0.679245	0.035088	8.333333
435	2-Jun-04	2004	7M	0.283019	0.471698	0.113208	0.679245	0.035088	8.333333
433	2-Jun-04	2004	7M	0.283019	0.471698	0.113208	0.679245	0.035088	8.333333
439	2-Jun-04	2004	7M	0.283019	0.471698	0.113208	0.679245	0.035088	8.333333
437	2-Jun-04	2004	7M	0.283019	0.471698	0.113208	0.679245	0.035088	8.333333
438	2-Jun-04	2004	7M	0.283019	0.471698	0.113208	0.679245	0.035088	8.333333
465	3-Jun-04	2004	D1/D2						
464	3-Jun-04	2004	D1/D2						
466	3-Jun-04	2004	D1/D2						

467	3-Jun-04	2004	D1/D2						
469	3-Jun-04	2004	D1/D2						
468	3-Jun-04	2004	D1/D2						
471	3-Jun-04	2004	D1/D2						
473	3-Jun-04	2004	D1/D2						
474	3-Jun-04	2004	D1/D2						
470	3-Jun-04	2004	D1/D2						
472	3-Jun-04	2004	D1/D2						
475	3-Jun-04	2004	D1/D2						
509	4-Jun-04	2004	ML						
507	4-Jun-04	2004	ML						
508	4-Jun-04	2004	ML						
270	12-Jun-04	2004	RL	0.396226	0.113208	0.09434	0.320755	0.203008	0.333333
278	12-Jun-04	2004	RL	0.396226	0.113208	0.09434	0.320755	0.203008	0.333333
281	12-Jun-04	2004	RL	0.396226	0.113208	0.09434	0.320755	0.203008	0.333333
276	12-Jun-04	2004	RL	0.396226	0.113208	0.09434	0.320755	0.203008	0.333333
277	12-Jun-04	2004	RL	0.396226	0.113208	0.09434	0.320755	0.203008	0.333333
275	12-Jun-04	2004	RL	0.396226	0.113208	0.09434	0.320755	0.203008	0.333333
279	12-Jun-04	2004	RL	0.396226	0.113208	0.09434	0.320755	0.203008	0.333333
264	12-Jun-04	2004	RL	0.396226	0.113208	0.09434	0.320755	0.203008	0.333333
284	12-Jun-04	2004	RL	0.396226	0.113208	0.09434	0.320755	0.203008	0.333333
262	12-Jun-04	2004	RL	0.396226	0.113208	0.09434	0.320755	0.203008	0.333333
265	12-Jun-04	2004	RL	0.396226	0.113208	0.09434	0.320755	0.203008	0.333333
263	12-Jun-04	2004	RL	0.396226	0.113208	0.09434	0.320755	0.203008	0.333333
280	12-Jun-04	2004	RL	0.396226	0.113208	0.09434	0.320755	0.203008	0.333333
267	12-Jun-04	2004	RL	0.396226	0.113208	0.09434	0.320755	0.203008	0.333333
266	12-Jun-04	2004	RL	0.396226	0.113208	0.09434	0.320755	0.203008	0.333333
269	12-Jun-04	2004	RL	0.396226	0.113208	0.09434	0.320755	0.203008	0.333333
273	12-Jun-04	2004	RL	0.396226	0.113208	0.09434	0.320755	0.203008	0.333333
274	12-Jun-04	2004	RL	0.396226	0.113208	0.09434	0.320755	0.203008	0.333333
282	12-Jun-04	2004	RL	0.396226	0.113208	0.09434	0.320755	0.203008	0.333333
258	12-Jun-04	2004	RL	0.396226	0.113208	0.09434	0.320755	0.203008	0.333333
260	12-Jun-04	2004	RL	0.396226	0.113208	0.09434	0.320755	0.203008	0.333333
259	12-Jun-04	2004	RL	0.396226	0.113208	0.09434	0.320755	0.203008	0.333333
261	12-Jun-04	2004	RL	0.396226	0.113208	0.09434	0.320755	0.203008	0.333333
268	12-Jun-04	2004	RL	0.396226	0.113208	0.09434	0.320755	0.203008	0.333333
283	12-Jun-04	2004	RL	0.396226	0.113208	0.09434	0.320755	0.203008	0.333333
271	12-Jun-04	2004	RL	0.396226	0.113208	0.09434	0.320755	0.203008	0.333333
272	12-Jun-04	2004	RL	0.396226	0.113208	0.09434	0.320755	0.203008	0.333333
537	13-Jun-04	2004	SD	0.412698	0.301587	0.063492	0.634921	0.097297	2.642857
538	13-Jun-04	2004	SD	0.412698	0.301587	0.063492	0.634921	0.097297	2.642857
539	13-Jun-04	2004	SD	0.412698	0.301587	0.063492	0.634921	0.097297	2.642857
569	16-Jun-04	2004	RP	0.095238	0.142857	0.095238	0.071429	0.323383	2
668	18-Jun-04	2004	DE						

669	18-Jun-04	2004	DE							
671	18-Jun-04	2004	DE							
667	18-Jun-04	2004	DE							
666	18-Jun-04	2004	DE							
670	18-Jun-04	2004	DE							
665	18-Jun-04	2004	DE							
673	18-Jun-04	2004	DE							
672	18-Jun-04	2004	DE							
701	20-Jun-04	2004	ML							
704	20-Jun-04	2004	ML							
706	20-Jun-04	2004	ML							
705	20-Jun-04	2004	ML							
707	20-Jun-04	2004	ML							
702	20-Jun-04	2004	ML							
697	20-Jun-04	2004	ML							
699	20-Jun-04	2004	ML							
690	20-Jun-04	2004	ML							
694	20-Jun-04	2004	ML							
695	20-Jun-04	2004	ML							
691	20-Jun-04	2004	ML							
693	20-Jun-04	2004	ML							
692	20-Jun-04	2004	ML							
696	20-Jun-04	2004	ML							
698	20-Jun-04	2004	ML							
700	20-Jun-04	2004	ML							
703	20-Jun-04	2004	ML							
818	22-Jun-04	2004	RP	0.095238	0.142857	0.095238	0.071429	0.323383	2
768	24-Jun-04	2004	RL	0.396226	0.113208	0.09434	0.320755	0.203008	0.333333
778	24-Jun-04	2004	RL	0.396226	0.113208	0.09434	0.320755	0.203008	0.333333
763	24-Jun-04	2004	RL	0.396226	0.113208	0.09434	0.320755	0.203008	0.333333
762	24-Jun-04	2004	RL	0.396226	0.113208	0.09434	0.320755	0.203008	0.333333
759	24-Jun-04	2004	RL	0.396226	0.113208	0.09434	0.320755	0.203008	0.333333
761	24-Jun-04	2004	RL	0.396226	0.113208	0.09434	0.320755	0.203008	0.333333
775	24-Jun-04	2004	RL	0.396226	0.113208	0.09434	0.320755	0.203008	0.333333
776	24-Jun-04	2004	RL	0.396226	0.113208	0.09434	0.320755	0.203008	0.333333
774	24-Jun-04	2004	RL	0.396226	0.113208	0.09434	0.320755	0.203008	0.333333
770	24-Jun-04	2004	RL	0.396226	0.113208	0.09434	0.320755	0.203008	0.333333
771	24-Jun-04	2004	RL	0.396226	0.113208	0.09434	0.320755	0.203008	0.333333
772	24-Jun-04	2004	RL	0.396226	0.113208	0.09434	0.320755	0.203008	0.333333
773	24-Jun-04	2004	RL	0.396226	0.113208	0.09434	0.320755	0.203008	0.333333
777	24-Jun-04	2004	RL	0.396226	0.113208	0.09434	0.320755	0.203008	0.333333
766	24-Jun-04	2004	RL	0.396226	0.113208	0.09434	0.320755	0.203008	0.333333
765	24-Jun-04	2004	RL	0.396226	0.113208	0.09434	0.320755	0.203008	0.333333
767	24-Jun-04	2004	RL	0.396226	0.113208	0.09434	0.320755	0.203008	0.333333

769	24-Jun-04	2004	RL	0.396226	0.113208	0.09434	0.320755	0.203008	0.333333
760	24-Jun-04	2004	RL	0.396226	0.113208	0.09434	0.320755	0.203008	0.333333
764	24-Jun-04	2004	RL	0.396226	0.113208	0.09434	0.320755	0.203008	0.333333
780	30-Jun-04	2004	RP	0.095238	0.142857	0.095238	0.071429	0.323383	2
779	30-Jun-04	2004	RP	0.095238	0.142857	0.095238	0.071429	0.323383	2
23	8-May-05	2005	SC	0.183486	0	0.073394	0	0.006329	6.769231
20	8-May-05	2005	SC	0.183486	0	0.073394	0	0.006329	6.769231
22	8-May-05	2005	SC	0.183486	0	0.073394	0	0.006329	6.769231
16	8-May-05	2005	SC	0.183486	0	0.073394	0	0.006329	6.769231
17	8-May-05	2005	SC	0.183486	0	0.073394	0	0.006329	6.769231
18	8-May-05	2005	SC	0.183486	0	0.073394	0	0.006329	6.769231
19	8-May-05	2005	SC	0.183486	0	0.073394	0	0.006329	6.769231
24	8-May-05	2005	SC	0.183486	0	0.073394	0	0.006329	6.769231
25	8-May-05	2005	SC	0.183486	0	0.073394	0	0.006329	6.769231
21	8-May-05	2005	SC	0.183486	0	0.073394	0	0.006329	6.769231
26	8-May-05	2005	SC	0.183486	0	0.073394	0	0.006329	6.769231
15	8-May-05	2005	SC	0.183486	0	0.073394	0	0.006329	6.769231
43	9-May-05	2005	ML						
45	9-May-05	2005	ML						
44	9-May-05	2005	ML						
46	9-May-05	2005	ML						
63	9-May-05	2005	ML						
62	9-May-05	2005	ML						
58	9-May-05	2005	ML						
59	9-May-05	2005	ML						
60	9-May-05	2005	ML						
61	9-May-05	2005	ML						
64	9-May-05	2005	ML						
65	9-May-05	2005	ML						
91	10-May-05	2005	SC	0.183486	0	0.073394	0	0.006329	6.769231
89	10-May-05	2005	SC	0.183486	0	0.073394	0	0.006329	6.769231
90	10-May-05	2005	SC	0.183486	0	0.073394	0	0.006329	6.769231
88	10-May-05	2005	SC	0.183486	0	0.073394	0	0.006329	6.769231
92	10-May-05	2005	SC	0.183486	0	0.073394	0	0.006329	6.769231
97	10-May-05	2005	SC	0.183486	0	0.073394	0	0.006329	6.769231
98	10-May-05	2005	SC	0.183486	0	0.073394	0	0.006329	6.769231
94	10-May-05	2005	SC	0.183486	0	0.073394	0	0.006329	6.769231
93	10-May-05	2005	SC	0.183486	0	0.073394	0	0.006329	6.769231
95	10-May-05	2005	SC	0.183486	0	0.073394	0	0.006329	6.769231
96	10-May-05	2005	SC	0.183486	0	0.073394	0	0.006329	6.769231
344	17-May-05	2005	SD	0.365079	0.095238	0.095238	0.380952	0.048387	2.5
345	17-May-05	2005	SD	0.365079	0.095238	0.095238	0.380952	0.048387	2.5
342	17-May-05	2005	SD	0.365079	0.095238	0.095238	0.380952	0.048387	2.5
343	17-May-05	2005	SD	0.365079	0.095238	0.095238	0.380952	0.048387	2.5

346	17-May-05	2005 SD	0.365079	0.095238	0.095238	0.380952	0.048387	2.5
457	20-May-05	2005 7M	0.264151	0.09434	0	0.301887	0.01519	8.266667
458	20-May-05	2005 7M	0.264151	0.09434	0	0.301887	0.01519	8.266667
462	20-May-05	2005 7M	0.264151	0.09434	0	0.301887	0.01519	8.266667
455	20-May-05	2005 7M	0.264151	0.09434	0	0.301887	0.01519	8.266667
456	20-May-05	2005 7M	0.264151	0.09434	0	0.301887	0.01519	8.266667
460	20-May-05	2005 7M	0.264151	0.09434	0	0.301887	0.01519	8.266667
461	20-May-05	2005 7M	0.264151	0.09434	0	0.301887	0.01519	8.266667
463	20-May-05	2005 7M	0.264151	0.09434	0	0.301887	0.01519	8.266667
459	20-May-05	2005 7M	0.264151	0.09434	0	0.301887	0.01519	8.266667
698	23-May-05	2005 RL	0.465517	0.103448	0	0.137931	0.108209	0.333333
699	23-May-05	2005 RL	0.465517	0.103448	0	0.137931	0.108209	0.333333
700	23-May-05	2005 RL	0.465517	0.103448	0	0.137931	0.108209	0.333333
702	23-May-05	2005 RL	0.465517	0.103448	0	0.137931	0.108209	0.333333
703	23-May-05	2005 RL	0.465517	0.103448	0	0.137931	0.108209	0.333333
701	23-May-05	2005 RL	0.465517	0.103448	0	0.137931	0.108209	0.333333
704	23-May-05	2005 RL	0.465517	0.103448	0	0.137931	0.108209	0.333333
709	23-May-05	2005 RL	0.465517	0.103448	0	0.137931	0.108209	0.333333
708	23-May-05	2005 RL	0.465517	0.103448	0	0.137931	0.108209	0.333333
705	23-May-05	2005 RL	0.465517	0.103448	0	0.137931	0.108209	0.333333
706	23-May-05	2005 RL	0.465517	0.103448	0	0.137931	0.108209	0.333333
707	23-May-05	2005 RL	0.465517	0.103448	0	0.137931	0.108209	0.333333
861	26-May-05	2005 SD	0.365079	0.095238	0.095238	0.380952	0.048387	2.5
862	26-May-05	2005 SD	0.365079	0.095238	0.095238	0.380952	0.048387	2.5
854	26-May-05	2005 SD	0.365079	0.095238	0.095238	0.380952	0.048387	2.5
855	26-May-05	2005 SD	0.365079	0.095238	0.095238	0.380952	0.048387	2.5
856	26-May-05	2005 SD	0.365079	0.095238	0.095238	0.380952	0.048387	2.5
857	26-May-05	2005 SD	0.365079	0.095238	0.095238	0.380952	0.048387	2.5
860	26-May-05	2005 SD	0.365079	0.095238	0.095238	0.380952	0.048387	2.5
853	26-May-05	2005 SD	0.365079	0.095238	0.095238	0.380952	0.048387	2.5
858	26-May-05	2005 SD	0.365079	0.095238	0.095238	0.380952	0.048387	2.5
859	26-May-05	2005 SD	0.365079	0.095238	0.095238	0.380952	0.048387	2.5
1	7-May-06	2006 SC	0.092308	0	0.030769	0.046154	0.002941	7.961538
2	7-May-06	2006 SC	0.092308	0	0.030769	0.046154	0.002941	7.961538
7	7-May-06	2006 SC	0.092308	0	0.030769	0.046154	0.002941	7.961538
6	7-May-06	2006 SC	0.092308	0	0.030769	0.046154	0.002941	7.961538
4	7-May-06	2006 SC	0.092308	0	0.030769	0.046154	0.002941	7.961538
5	7-May-06	2006 SC	0.092308	0	0.030769	0.046154	0.002941	7.961538
11	7-May-06	2006 SC	0.092308	0	0.030769	0.046154	0.002941	7.961538
3	7-May-06	2006 SC	0.092308	0	0.030769	0.046154	0.002941	7.961538
9	7-May-06	2006 SC	0.092308	0	0.030769	0.046154	0.002941	7.961538
10	7-May-06	2006 SC	0.092308	0	0.030769	0.046154	0.002941	7.961538
8	7-May-06	2006 SC	0.092308	0	0.030769	0.046154	0.002941	7.961538
21	10-May-06	2006 SC	0.092308	0	0.030769	0.046154	0.002941	7.961538

22	10-May-06	2006	SC	0.092308	0	0.030769	0.046154	0.002941	7.961538
26	10-May-06	2006	SC	0.092308	0	0.030769	0.046154	0.002941	7.961538
25	10-May-06	2006	SC	0.092308	0	0.030769	0.046154	0.002941	7.961538
24	10-May-06	2006	SC	0.092308	0	0.030769	0.046154	0.002941	7.961538
23	10-May-06	2006	SC	0.092308	0	0.030769	0.046154	0.002941	7.961538
27	10-May-06	2006	SC	0.092308	0	0.030769	0.046154	0.002941	7.961538
28	10-May-06	2006	SC	0.092308	0	0.030769	0.046154	0.002941	7.961538
32	10-May-06	2006	SC	0.092308	0	0.030769	0.046154	0.002941	7.961538
33	10-May-06	2006	SC	0.092308	0	0.030769	0.046154	0.002941	7.961538
31	10-May-06	2006	SC	0.092308	0	0.030769	0.046154	0.002941	7.961538
30	10-May-06	2006	SC	0.092308	0	0.030769	0.046154	0.002941	7.961538
29	10-May-06	2006	SC	0.092308	0	0.030769	0.046154	0.002941	7.961538
34	10-May-06	2006	SC	0.092308	0	0.030769	0.046154	0.002941	7.961538
59	17-May-06	2006	LT1	0.222222	0.111111	0	0.148148	0	4
57	17-May-06	2006	LT1	0.222222	0.111111	0	0.148148	0	4
56	17-May-06	2006	LT1	0.222222	0.111111	0	0.148148	0	4
58	17-May-06	2006	LT1	0.222222	0.111111	0	0.148148	0	4
55	17-May-06	2006	LT1	0.222222	0.111111	0	0.148148	0	4
60	17-May-06	2006	LT1	0.222222	0.111111	0	0.148148	0	4
65	17-May-06	2006	LT1	0.222222	0.111111	0	0.148148	0	4
61	17-May-06	2006	LT1	0.222222	0.111111	0	0.148148	0	4
53	17-May-06	2006	LT1	0.222222	0.111111	0	0.148148	0	4
54	17-May-06	2006	LT1	0.222222	0.111111	0	0.148148	0	4
62	17-May-06	2006	LT1	0.222222	0.111111	0	0.148148	0	4
63	17-May-06	2006	LT1	0.222222	0.111111	0	0.148148	0	4
64	17-May-06	2006	LT1	0.222222	0.111111	0	0.148148	0	4
66	17-May-06	2006	LT1	0.222222	0.111111	0	0.148148	0	4
91	18-May-06	2006	SD						
93	18-May-06	2006	SD						
94	18-May-06	2006	SD						
92	18-May-06	2006	SD						
95	22-May-06	2006	RL	0.135135	0.027027	0	0.594595	0.046931	0.333333
	25-May-06	2006	SW	0.210526	0.026316	0.105263	0.131579	0.017778	1.4
	25-Jun-06	2006	ML						
	25-Jun-06	2006	ML						
	25-Jun-06	2006	ML						
	25-Jun-06	2006	ML						
	25-Jun-06	2006	ML						
64	7-May-07	2007	MM	0.036364	0.090909	0.054545	0.181818	0.030769	1.545455
62	7-May-07	2007	MM	0.036364	0.090909	0.054545	0.181818	0.030769	1.545455
76	7-May-07	2007	MM	0.036364	0.090909	0.054545	0.181818	0.030769	1.545455
75	7-May-07	2007	MM	0.036364	0.090909	0.054545	0.181818	0.030769	1.545455
78	7-May-07	2007	MM	0.036364	0.090909	0.054545	0.181818	0.030769	1.545455
74	7-May-07	2007	MM	0.036364	0.090909	0.054545	0.181818	0.030769	1.545455

77	7-May-07	2007	MM	0.036364	0.090909	0.054545	0.181818	0.030769	1.545455
79	7-May-07	2007	MM	0.036364	0.090909	0.054545	0.181818	0.030769	1.545455
80	7-May-07	2007	MM	0.036364	0.090909	0.054545	0.181818	0.030769	1.545455
81	7-May-07	2007	MM	0.036364	0.090909	0.054545	0.181818	0.030769	1.545455
82	7-May-07	2007	MM	0.036364	0.090909	0.054545	0.181818	0.030769	1.545455
73	7-May-07	2007	MM	0.036364	0.090909	0.054545	0.181818	0.030769	1.545455
84	7-May-07	2007	MM	0.036364	0.090909	0.054545	0.181818	0.030769	1.545455
85	7-May-07	2007	MM	0.036364	0.090909	0.054545	0.181818	0.030769	1.545455
72	7-May-07	2007	MM	0.036364	0.090909	0.054545	0.181818	0.030769	1.545455
69	7-May-07	2007	MM	0.036364	0.090909	0.054545	0.181818	0.030769	1.545455
70	7-May-07	2007	MM	0.036364	0.090909	0.054545	0.181818	0.030769	1.545455
67	7-May-07	2007	MM	0.036364	0.090909	0.054545	0.181818	0.030769	1.545455
71	7-May-07	2007	MM	0.036364	0.090909	0.054545	0.181818	0.030769	1.545455
68	7-May-07	2007	MM	0.036364	0.090909	0.054545	0.181818	0.030769	1.545455
86	7-May-07	2007	MM	0.036364	0.090909	0.054545	0.181818	0.030769	1.545455
87	7-May-07	2007	MM	0.036364	0.090909	0.054545	0.181818	0.030769	1.545455
88	7-May-07	2007	MM	0.036364	0.090909	0.054545	0.181818	0.030769	1.545455
66	7-May-07	2007	MM	0.036364	0.090909	0.054545	0.181818	0.030769	1.545455
65	7-May-07	2007	MM	0.036364	0.090909	0.054545	0.181818	0.030769	1.545455
89	7-May-07	2007	MM	0.036364	0.090909	0.054545	0.181818	0.030769	1.545455
61	7-May-07	2007	MM	0.036364	0.090909	0.054545	0.181818	0.030769	1.545455
63	7-May-07	2007	MM	0.036364	0.090909	0.054545	0.181818	0.030769	1.545455
83	7-May-07	2007	MM	0.036364	0.090909	0.054545	0.181818	0.030769	1.545455
105	7-May-07	2007	MM	0.036364	0.090909	0.054545	0.181818	0.030769	1.545455
107	7-May-07	2007	MM	0.036364	0.090909	0.054545	0.181818	0.030769	1.545455
108	7-May-07	2007	MM	0.036364	0.090909	0.054545	0.181818	0.030769	1.545455
109	7-May-07	2007	MM	0.036364	0.090909	0.054545	0.181818	0.030769	1.545455
106	7-May-07	2007	MM	0.036364	0.090909	0.054545	0.181818	0.030769	1.545455
104	7-May-07	2007	MM	0.036364	0.090909	0.054545	0.181818	0.030769	1.545455
110	7-May-07	2007	MM	0.036364	0.090909	0.054545	0.181818	0.030769	1.545455
111	8-May-07	2007	MG	0.24	0.28	0.12	0.02	0.009615	6.15
120	8-May-07	2007	MG	0.24	0.28	0.12	0.02	0.009615	6.15
112	8-May-07	2007	MG	0.24	0.28	0.12	0.02	0.009615	6.15
113	8-May-07	2007	MG	0.24	0.28	0.12	0.02	0.009615	6.15
118	8-May-07	2007	MG	0.24	0.28	0.12	0.02	0.009615	6.15
121	8-May-07	2007	MG	0.24	0.28	0.12	0.02	0.009615	6.15
122	8-May-07	2007	MG	0.24	0.28	0.12	0.02	0.009615	6.15
116	8-May-07	2007	MG	0.24	0.28	0.12	0.02	0.009615	6.15
117	8-May-07	2007	MG	0.24	0.28	0.12	0.02	0.009615	6.15
115	8-May-07	2007	MG	0.24	0.28	0.12	0.02	0.009615	6.15
114	8-May-07	2007	MG	0.24	0.28	0.12	0.02	0.009615	6.15
119	8-May-07	2007	MG	0.24	0.28	0.12	0.02	0.009615	6.15
635	9-May-07	2007	SW	0.157895	0.078947	0.026316	0	0	1.4
607	9-May-07	2007	SW	0.157895	0.078947	0.026316	0	0	1.4

610	9-May-07	2007	SW	0.157895	0.078947	0.026316	0	0	1.4
614	9-May-07	2007	SW	0.157895	0.078947	0.026316	0	0	1.4
616	9-May-07	2007	SW	0.157895	0.078947	0.026316	0	0	1.4
630	9-May-07	2007	SW	0.157895	0.078947	0.026316	0	0	1.4
632	9-May-07	2007	SW	0.157895	0.078947	0.026316	0	0	1.4
633	9-May-07	2007	SW	0.157895	0.078947	0.026316	0	0	1.4
608	9-May-07	2007	SW	0.157895	0.078947	0.026316	0	0	1.4
609	9-May-07	2007	SW	0.157895	0.078947	0.026316	0	0	1.4
612	9-May-07	2007	SW	0.157895	0.078947	0.026316	0	0	1.4
613	9-May-07	2007	SW	0.157895	0.078947	0.026316	0	0	1.4
615	9-May-07	2007	SW	0.157895	0.078947	0.026316	0	0	1.4
628	9-May-07	2007	SW	0.157895	0.078947	0.026316	0	0	1.4
629	9-May-07	2007	SW	0.157895	0.078947	0.026316	0	0	1.4
636	9-May-07	2007	SW	0.157895	0.078947	0.026316	0	0	1.4
606	9-May-07	2007	SW	0.157895	0.078947	0.026316	0	0	1.4
631	9-May-07	2007	SW	0.157895	0.078947	0.026316	0	0	1.4
617	9-May-07	2007	SW	0.157895	0.078947	0.026316	0	0	1.4
637	9-May-07	2007	SW	0.157895	0.078947	0.026316	0	0	1.4
605	9-May-07	2007	SW	0.157895	0.078947	0.026316	0	0	1.4
634	9-May-07	2007	SW	0.157895	0.078947	0.026316	0	0	1.4
604	9-May-07	2007	SW	0.157895	0.078947	0.026316	0	0	1.4
602	9-May-07	2007	SW	0.157895	0.078947	0.026316	0	0	1.4
603	9-May-07	2007	SW	0.157895	0.078947	0.026316	0	0	1.4
601	9-May-07	2007	SW	0.157895	0.078947	0.026316	0	0	1.4
611	9-May-07	2007	SW	0.157895	0.078947	0.026316	0	0	1.4
638	9-May-07	2007	SW	0.157895	0.078947	0.026316	0	0	1.4
639	9-May-07	2007	SW	0.157895	0.078947	0.026316	0	0	1.4
640	9-May-07	2007	SW	0.157895	0.078947	0.026316	0	0	1.4
619	9-May-07	2007	SW	0.157895	0.078947	0.026316	0	0	1.4
620	9-May-07	2007	SW	0.157895	0.078947	0.026316	0	0	1.4
621	9-May-07	2007	SW	0.157895	0.078947	0.026316	0	0	1.4
627	9-May-07	2007	SW	0.157895	0.078947	0.026316	0	0	1.4
622	9-May-07	2007	SW	0.157895	0.078947	0.026316	0	0	1.4
624	9-May-07	2007	SW	0.157895	0.078947	0.026316	0	0	1.4
623	9-May-07	2007	SW	0.157895	0.078947	0.026316	0	0	1.4
625	9-May-07	2007	SW	0.157895	0.078947	0.026316	0	0	1.4
600	9-May-07	2007	SW	0.157895	0.078947	0.026316	0	0	1.4
599	9-May-07	2007	SW	0.157895	0.078947	0.026316	0	0	1.4
618	9-May-07	2007	SW	0.157895	0.078947	0.026316	0	0	1.4
626	9-May-07	2007	SW	0.157895	0.078947	0.026316	0	0	1.4
740	10-May-07	2007	SHAC	0.071429	0.071429	0	0.098361	0.008818	2.227273
743	10-May-07	2007	SHAC	0.071429	0.071429	0	0.098361	0.008818	2.227273
750	10-May-07	2007	SHAC	0.071429	0.071429	0	0.098361	0.008818	2.227273
751	10-May-07	2007	SHAC	0.071429	0.071429	0	0.098361	0.008818	2.227273

752	10-May-07	2007	SHAC	0.071429	0.071429		0	0.098361	0.008818	2.227273
753	10-May-07	2007	SHAC	0.071429	0.071429		0	0.098361	0.008818	2.227273
748	10-May-07	2007	SHAC	0.071429	0.071429		0	0.098361	0.008818	2.227273
744	10-May-07	2007	SHAC	0.071429	0.071429		0	0.098361	0.008818	2.227273
746	10-May-07	2007	SHAC	0.071429	0.071429		0	0.098361	0.008818	2.227273
755	10-May-07	2007	SHAC	0.071429	0.071429		0	0.098361	0.008818	2.227273
756	10-May-07	2007	SHAC	0.071429	0.071429		0	0.098361	0.008818	2.227273
737	10-May-07	2007	SHAC	0.071429	0.071429		0	0.098361	0.008818	2.227273
736	10-May-07	2007	SHAC	0.071429	0.071429		0	0.098361	0.008818	2.227273
747	10-May-07	2007	SHAC	0.071429	0.071429		0	0.098361	0.008818	2.227273
749	10-May-07	2007	SHAC	0.071429	0.071429		0	0.098361	0.008818	2.227273
738	10-May-07	2007	SHAC	0.071429	0.071429		0	0.098361	0.008818	2.227273
754	10-May-07	2007	SHAC	0.071429	0.071429		0	0.098361	0.008818	2.227273
758	10-May-07	2007	SHAC	0.071429	0.071429		0	0.098361	0.008818	2.227273
735	10-May-07	2007	SHAC	0.071429	0.071429		0	0.098361	0.008818	2.227273
739	10-May-07	2007	SHAC	0.071429	0.071429		0	0.098361	0.008818	2.227273
742	10-May-07	2007	SHAC	0.071429	0.071429		0	0.098361	0.008818	2.227273
741	10-May-07	2007	SHAC	0.071429	0.071429		0	0.098361	0.008818	2.227273
759	10-May-07	2007	SHAC	0.071429	0.071429		0	0.098361	0.008818	2.227273
734	10-May-07	2007	SHAC	0.071429	0.071429		0	0.098361	0.008818	2.227273
745	10-May-07	2007	SHAC	0.071429	0.071429		0	0.098361	0.008818	2.227273
757	10-May-07	2007	SHAC	0.071429	0.071429		0	0.098361	0.008818	2.227273
788	10-May-07	2007	SHAC	0.071429	0.071429		0	0.098361	0.008818	2.227273
776	10-May-07	2007	SHAC	0.071429	0.071429		0	0.098361	0.008818	2.227273
775	10-May-07	2007	SHAC	0.071429	0.071429		0	0.098361	0.008818	2.227273
783	10-May-07	2007	SHAC	0.071429	0.071429		0	0.098361	0.008818	2.227273
780	10-May-07	2007	SHAC	0.071429	0.071429		0	0.098361	0.008818	2.227273
786	10-May-07	2007	SHAC	0.071429	0.071429		0	0.098361	0.008818	2.227273
774	10-May-07	2007	SHAC	0.071429	0.071429		0	0.098361	0.008818	2.227273
778	10-May-07	2007	SHAC	0.071429	0.071429		0	0.098361	0.008818	2.227273
779	10-May-07	2007	SHAC	0.071429	0.071429		0	0.098361	0.008818	2.227273
784	10-May-07	2007	SHAC	0.071429	0.071429		0	0.098361	0.008818	2.227273
768	10-May-07	2007	SHAC	0.071429	0.071429		0	0.098361	0.008818	2.227273
770	10-May-07	2007	SHAC	0.071429	0.071429		0	0.098361	0.008818	2.227273
785	10-May-07	2007	SHAC	0.071429	0.071429		0	0.098361	0.008818	2.227273
787	10-May-07	2007	SHAC	0.071429	0.071429		0	0.098361	0.008818	2.227273
773	10-May-07	2007	SHAC	0.071429	0.071429		0	0.098361	0.008818	2.227273
761	10-May-07	2007	SHAC	0.071429	0.071429		0	0.098361	0.008818	2.227273
762	10-May-07	2007	SHAC	0.071429	0.071429		0	0.098361	0.008818	2.227273
764	10-May-07	2007	SHAC	0.071429	0.071429		0	0.098361	0.008818	2.227273
765	10-May-07	2007	SHAC	0.071429	0.071429		0	0.098361	0.008818	2.227273
766	10-May-07	2007	SHAC	0.071429	0.071429		0	0.098361	0.008818	2.227273
767	10-May-07	2007	SHAC	0.071429	0.071429		0	0.098361	0.008818	2.227273
769	10-May-07	2007	SHAC	0.071429	0.071429		0	0.098361	0.008818	2.227273

763	10-May-07	2007	SHAC	0.071429	0.071429		0	0.098361	0.008818	2.227273
781	10-May-07	2007	SHAC	0.071429	0.071429		0	0.098361	0.008818	2.227273
777	10-May-07	2007	SHAC	0.071429	0.071429		0	0.098361	0.008818	2.227273
772	10-May-07	2007	SHAC	0.071429	0.071429		0	0.098361	0.008818	2.227273
760	10-May-07	2007	SHAC	0.071429	0.071429		0	0.098361	0.008818	2.227273
771	10-May-07	2007	SHAC	0.071429	0.071429		0	0.098361	0.008818	2.227273
782	10-May-07	2007	SHAC	0.071429	0.071429		0	0.098361	0.008818	2.227273
180	11-May-07	2007	AFF							
181	11-May-07	2007	AFF							
182	11-May-07	2007	AFF							
184	11-May-07	2007	AFF							
183	11-May-07	2007	AFF							
185	11-May-07	2007	AFF							
186	11-May-07	2007	AFF							
187	11-May-07	2007	AFF							
188	11-May-07	2007	AFF							
189	11-May-07	2007	AFF							
190	11-May-07	2007	AFF							
191	11-May-07	2007	AFF							
192	11-May-07	2007	AFF							
193	11-May-07	2007	AFF							
194	11-May-07	2007	AFF							
178	11-May-07	2007	AFF							
179	11-May-07	2007	AFF							
235	11-May-07	2007	AFC							
249	11-May-07	2007	AFC							
234	11-May-07	2007	AFC							
244	11-May-07	2007	AFC							
251	11-May-07	2007	AFC							
226	11-May-07	2007	AFC							
229	11-May-07	2007	AFC							
231	11-May-07	2007	AFC							
225	11-May-07	2007	AFC							
230	11-May-07	2007	AFC							
232	11-May-07	2007	AFC							
245	11-May-07	2007	AFC							
252	11-May-07	2007	AFC							
253	11-May-07	2007	AFC							
254	11-May-07	2007	AFC							
236	11-May-07	2007	AFC							
237	11-May-07	2007	AFC							
238	11-May-07	2007	AFC							
239	11-May-07	2007	AFC							
246	11-May-07	2007	AFC							

224	11-May-07	2007	AFC						
227	11-May-07	2007	AFC						
228	11-May-07	2007	AFC						
233	11-May-07	2007	AFC						
247	11-May-07	2007	AFC						
250	11-May-07	2007	AFC						
248	11-May-07	2007	AFC						
255	11-May-07	2007	AFC						
256	11-May-07	2007	AFC						
223	11-May-07	2007	AFC						
240	11-May-07	2007	AFC						
257	11-May-07	2007	AFC						
222	11-May-07	2007	AFC						
243	11-May-07	2007	AFC						
241	11-May-07	2007	AFC						
258	11-May-07	2007	AFC						
220	11-May-07	2007	AFC						
218	11-May-07	2007	AFC						
221	11-May-07	2007	AFC						
242	11-May-07	2007	AFC						
219	11-May-07	2007	AFC						
274	12-May-07	2007	RL	0.157895	0.157895	0	0	0.028571	0.333333
275	12-May-07	2007	RL	0.157895	0.157895	0	0	0.028571	0.333333
276	12-May-07	2007	RL	0.157895	0.157895	0	0	0.028571	0.333333
283	12-May-07	2007	RL	0.157895	0.157895	0	0	0.028571	0.333333
284	12-May-07	2007	RL	0.157895	0.157895	0	0	0.028571	0.333333
300	12-May-07	2007	RL	0.157895	0.157895	0	0	0.028571	0.333333
282	12-May-07	2007	RL	0.157895	0.157895	0	0	0.028571	0.333333
301	12-May-07	2007	RL	0.157895	0.157895	0	0	0.028571	0.333333
281	12-May-07	2007	RL	0.157895	0.157895	0	0	0.028571	0.333333
299	12-May-07	2007	RL	0.157895	0.157895	0	0	0.028571	0.333333
280	12-May-07	2007	RL	0.157895	0.157895	0	0	0.028571	0.333333
293	12-May-07	2007	RL	0.157895	0.157895	0	0	0.028571	0.333333
297	12-May-07	2007	RL	0.157895	0.157895	0	0	0.028571	0.333333
285	12-May-07	2007	RL	0.157895	0.157895	0	0	0.028571	0.333333
286	12-May-07	2007	RL	0.157895	0.157895	0	0	0.028571	0.333333
287	12-May-07	2007	RL	0.157895	0.157895	0	0	0.028571	0.333333
304	12-May-07	2007	RL	0.157895	0.157895	0	0	0.028571	0.333333
306	12-May-07	2007	RL	0.157895	0.157895	0	0	0.028571	0.333333
279	12-May-07	2007	RL	0.157895	0.157895	0	0	0.028571	0.333333
288	12-May-07	2007	RL	0.157895	0.157895	0	0	0.028571	0.333333
289	12-May-07	2007	RL	0.157895	0.157895	0	0	0.028571	0.333333
295	12-May-07	2007	RL	0.157895	0.157895	0	0	0.028571	0.333333
296	12-May-07	2007	RL	0.157895	0.157895	0	0	0.028571	0.333333

278	12-May-07	2007	RL	0.157895	0.157895	0	0	0.028571	0.333333
294	12-May-07	2007	RL	0.157895	0.157895	0	0	0.028571	0.333333
307	12-May-07	2007	RL	0.157895	0.157895	0	0	0.028571	0.333333
291	12-May-07	2007	RL	0.157895	0.157895	0	0	0.028571	0.333333
290	12-May-07	2007	RL	0.157895	0.157895	0	0	0.028571	0.333333
303	12-May-07	2007	RL	0.157895	0.157895	0	0	0.028571	0.333333
305	12-May-07	2007	RL	0.157895	0.157895	0	0	0.028571	0.333333
302	12-May-07	2007	RL	0.157895	0.157895	0	0	0.028571	0.333333
277	12-May-07	2007	RL	0.157895	0.157895	0	0	0.028571	0.333333
292	12-May-07	2007	RL	0.157895	0.157895	0	0	0.028571	0.333333
298	12-May-07	2007	RL	0.157895	0.157895	0	0	0.028571	0.333333
929	16-May-07	2007	ML						
926	16-May-07	2007	ML						
924	16-May-07	2007	ML						
925	16-May-07	2007	ML						
927	16-May-07	2007	ML						
931	16-May-07	2007	ML						
932	16-May-07	2007	ML						
933	16-May-07	2007	ML						
935	16-May-07	2007	ML						
923	16-May-07	2007	ML						
937	16-May-07	2007	ML						
918	16-May-07	2007	ML						
919	16-May-07	2007	ML						
920	16-May-07	2007	ML						
921	16-May-07	2007	ML						
928	16-May-07	2007	ML						
934	16-May-07	2007	ML						
936	16-May-07	2007	ML						
917	16-May-07	2007	ML						
922	16-May-07	2007	ML						
930	16-May-07	2007	ML						
938	16-May-07	2007	ML						
916	16-May-07	2007	ML						
973	16-May-07	2007	YY	0.2	0.08	0.06	0.08	0	0.65
975	16-May-07	2007	YY	0.2	0.08	0.06	0.08	0	0.65
977	16-May-07	2007	YY	0.2	0.08	0.06	0.08	0	0.65
979	16-May-07	2007	YY	0.2	0.08	0.06	0.08	0	0.65
980	16-May-07	2007	YY	0.2	0.08	0.06	0.08	0	0.65
978	16-May-07	2007	YY	0.2	0.08	0.06	0.08	0	0.65
981	16-May-07	2007	YY	0.2	0.08	0.06	0.08	0	0.65
982	16-May-07	2007	YY	0.2	0.08	0.06	0.08	0	0.65
972	16-May-07	2007	YY	0.2	0.08	0.06	0.08	0	0.65
974	16-May-07	2007	YY	0.2	0.08	0.06	0.08	0	0.65

976	16-May-07	2007 YY	0.2	0.08	0.06	0.08	0	0.65
1036	18-May-07	2007 7M	0.131579	0.105263	0.078947	0.052632	0.007712	8.066667
1038	18-May-07	2007 7M	0.131579	0.105263	0.078947	0.052632	0.007712	8.066667
1039	18-May-07	2007 7M	0.131579	0.105263	0.078947	0.052632	0.007712	8.066667
1035	18-May-07	2007 7M	0.131579	0.105263	0.078947	0.052632	0.007712	8.066667
1034	18-May-07	2007 7M	0.131579	0.105263	0.078947	0.052632	0.007712	8.066667
1033	18-May-07	2007 7M	0.131579	0.105263	0.078947	0.052632	0.007712	8.066667
1045	18-May-07	2007 7M	0.131579	0.105263	0.078947	0.052632	0.007712	8.066667
1046	18-May-07	2007 7M	0.131579	0.105263	0.078947	0.052632	0.007712	8.066667
1047	18-May-07	2007 7M	0.131579	0.105263	0.078947	0.052632	0.007712	8.066667
1052	18-May-07	2007 7M	0.131579	0.105263	0.078947	0.052632	0.007712	8.066667
1053	18-May-07	2007 7M	0.131579	0.105263	0.078947	0.052632	0.007712	8.066667
1054	18-May-07	2007 7M	0.131579	0.105263	0.078947	0.052632	0.007712	8.066667
1055	18-May-07	2007 7M	0.131579	0.105263	0.078947	0.052632	0.007712	8.066667
1056	18-May-07	2007 7M	0.131579	0.105263	0.078947	0.052632	0.007712	8.066667
1040	18-May-07	2007 7M	0.131579	0.105263	0.078947	0.052632	0.007712	8.066667
1041	18-May-07	2007 7M	0.131579	0.105263	0.078947	0.052632	0.007712	8.066667
1042	18-May-07	2007 7M	0.131579	0.105263	0.078947	0.052632	0.007712	8.066667
1044	18-May-07	2007 7M	0.131579	0.105263	0.078947	0.052632	0.007712	8.066667
1058	18-May-07	2007 7M	0.131579	0.105263	0.078947	0.052632	0.007712	8.066667
1059	18-May-07	2007 7M	0.131579	0.105263	0.078947	0.052632	0.007712	8.066667
1037	18-May-07	2007 7M	0.131579	0.105263	0.078947	0.052632	0.007712	8.066667
1048	18-May-07	2007 7M	0.131579	0.105263	0.078947	0.052632	0.007712	8.066667
1049	18-May-07	2007 7M	0.131579	0.105263	0.078947	0.052632	0.007712	8.066667
1050	18-May-07	2007 7M	0.131579	0.105263	0.078947	0.052632	0.007712	8.066667
1057	18-May-07	2007 7M	0.131579	0.105263	0.078947	0.052632	0.007712	8.066667
1032	18-May-07	2007 7M	0.131579	0.105263	0.078947	0.052632	0.007712	8.066667
1043	18-May-07	2007 7M	0.131579	0.105263	0.078947	0.052632	0.007712	8.066667
1051	18-May-07	2007 7M	0.131579	0.105263	0.078947	0.052632	0.007712	8.066667
1144	20-May-07	2007 FO	0.236842	0.078947	0	0	0	0.066667
1148	20-May-07	2007 FO	0.236842	0.078947	0	0	0	0.066667
1149	20-May-07	2007 FO	0.236842	0.078947	0	0	0	0.066667
1150	20-May-07	2007 FO	0.236842	0.078947	0	0	0	0.066667
1157	20-May-07	2007 FO	0.236842	0.078947	0	0	0	0.066667
1159	20-May-07	2007 FO	0.236842	0.078947	0	0	0	0.066667
1151	20-May-07	2007 FO	0.236842	0.078947	0	0	0	0.066667
1153	20-May-07	2007 FO	0.236842	0.078947	0	0	0	0.066667
1154	20-May-07	2007 FO	0.236842	0.078947	0	0	0	0.066667
1155	20-May-07	2007 FO	0.236842	0.078947	0	0	0	0.066667
1156	20-May-07	2007 FO	0.236842	0.078947	0	0	0	0.066667
1145	20-May-07	2007 FO	0.236842	0.078947	0	0	0	0.066667
1146	20-May-07	2007 FO	0.236842	0.078947	0	0	0	0.066667
1147	20-May-07	2007 FO	0.236842	0.078947	0	0	0	0.066667
1143	20-May-07	2007 FO	0.236842	0.078947	0	0	0	0.066667

1152	20-May-07	2007	FO	0.236842	0.078947	0	0	0	0.066667
1158	20-May-07	2007	FO	0.236842	0.078947	0	0	0	0.066667
1163	20-May-07	2007	FO	0.236842	0.078947	0	0	0	0.066667
1164	20-May-07	2007	FO	0.236842	0.078947	0	0	0	0.066667
1165	20-May-07	2007	FO	0.236842	0.078947	0	0	0	0.066667
1166	20-May-07	2007	FO	0.236842	0.078947	0	0	0	0.066667
1162	20-May-07	2007	FO	0.236842	0.078947	0	0	0	0.066667
1168	20-May-07	2007	FO	0.236842	0.078947	0	0	0	0.066667
1169	20-May-07	2007	FO	0.236842	0.078947	0	0	0	0.066667
1161	20-May-07	2007	FO	0.236842	0.078947	0	0	0	0.066667
1167	20-May-07	2007	FO	0.236842	0.078947	0	0	0	0.066667
1172	20-May-07	2007	FO	0.236842	0.078947	0	0	0	0.066667
1160	20-May-07	2007	FO	0.236842	0.078947	0	0	0	0.066667
1170	20-May-07	2007	FO	0.236842	0.078947	0	0	0	0.066667
1171	20-May-07	2007	FO	0.236842	0.078947	0	0	0	0.066667
1173	20-May-07	2007	FO	0.236842	0.078947	0	0	0	0.066667
1189	20-May-07	2007	FO	0.236842	0.078947	0	0	0	0.066667
1188	20-May-07	2007	FO	0.236842	0.078947	0	0	0	0.066667
1192	20-May-07	2007	FO	0.236842	0.078947	0	0	0	0.066667
1187	20-May-07	2007	FO	0.236842	0.078947	0	0	0	0.066667
1184	20-May-07	2007	FO	0.236842	0.078947	0	0	0	0.066667
1186	20-May-07	2007	FO	0.236842	0.078947	0	0	0	0.066667
1191	20-May-07	2007	FO	0.236842	0.078947	0	0	0	0.066667
1194	20-May-07	2007	FO	0.236842	0.078947	0	0	0	0.066667
1178	20-May-07	2007	FO	0.236842	0.078947	0	0	0	0.066667
1180	20-May-07	2007	FO	0.236842	0.078947	0	0	0	0.066667
1182	20-May-07	2007	FO	0.236842	0.078947	0	0	0	0.066667
1185	20-May-07	2007	FO	0.236842	0.078947	0	0	0	0.066667
1179	20-May-07	2007	FO	0.236842	0.078947	0	0	0	0.066667
1190	20-May-07	2007	FO	0.236842	0.078947	0	0	0	0.066667
1174	20-May-07	2007	FO	0.236842	0.078947	0	0	0	0.066667
1181	20-May-07	2007	FO	0.236842	0.078947	0	0	0	0.066667
1176	20-May-07	2007	FO	0.236842	0.078947	0	0	0	0.066667
1177	20-May-07	2007	FO	0.236842	0.078947	0	0	0	0.066667
1175	20-May-07	2007	FO	0.236842	0.078947	0	0	0	0.066667
1183	20-May-07	2007	FO	0.236842	0.078947	0	0	0	0.066667
1193	20-May-07	2007	FO	0.236842	0.078947	0	0	0	0.066667
2133	24-May-07	2007	ML						
2113	24-May-07	2007	ML						
2120	24-May-07	2007	ML						
2127	24-May-07	2007	ML						
2128	24-May-07	2007	ML						
2131	24-May-07	2007	ML						
2119	24-May-07	2007	ML						

2123	24-May-07	2007	ML
2125	24-May-07	2007	ML
2126	24-May-07	2007	ML
2129	24-May-07	2007	ML
2118	24-May-07	2007	ML
2117	24-May-07	2007	ML
2114	24-May-07	2007	ML
2121	24-May-07	2007	ML
2132	24-May-07	2007	ML
2134	24-May-07	2007	ML
2135	24-May-07	2007	ML
2124	24-May-07	2007	ML
2115	24-May-07	2007	ML
2122	24-May-07	2007	ML
2112	24-May-07	2007	ML
2116	24-May-07	2007	ML
2130	24-May-07	2007	ML
2136	24-May-07	2007	ML
2111	24-May-07	2007	ML
2641	26-May-07	2007	SD
2642	26-May-07	2007	SD
2643	26-May-07	2007	SD
2639	26-May-07	2007	SD
2658	26-May-07	2007	SD
2633	26-May-07	2007	SD
2634	26-May-07	2007	SD
2651	26-May-07	2007	SD
2636	26-May-07	2007	SD
2637	26-May-07	2007	SD
2654	26-May-07	2007	SD
2650	26-May-07	2007	SD
2648	26-May-07	2007	SD
2649	26-May-07	2007	SD
2647	26-May-07	2007	SD
2653	26-May-07	2007	SD
2646	26-May-07	2007	SD
2652	26-May-07	2007	SD
2645	26-May-07	2007	SD
2655	26-May-07	2007	SD
2656	26-May-07	2007	SD
2657	26-May-07	2007	SD
2659	26-May-07	2007	SD
2660	26-May-07	2007	SD
2632	26-May-07	2007	SD

2635	26-May-07	2007	SD						
2640	26-May-07	2007	SD						
2631	26-May-07	2007	SD						
2638	26-May-07	2007	SD						
2644	26-May-07	2007	SD						
2740	29-May-07	2007	SHAC	0.071429	0.071429	0	0.098361	0.008818	2.227273
2742	29-May-07	2007	SHAC	0.071429	0.071429	0	0.098361	0.008818	2.227273
2743	29-May-07	2007	SHAC	0.071429	0.071429	0	0.098361	0.008818	2.227273
2744	29-May-07	2007	SHAC	0.071429	0.071429	0	0.098361	0.008818	2.227273
2739	29-May-07	2007	SHAC	0.071429	0.071429	0	0.098361	0.008818	2.227273
2738	29-May-07	2007	SHAC	0.071429	0.071429	0	0.098361	0.008818	2.227273
2734	29-May-07	2007	SHAC	0.071429	0.071429	0	0.098361	0.008818	2.227273
2731	29-May-07	2007	SHAC	0.071429	0.071429	0	0.098361	0.008818	2.227273
2729	29-May-07	2007	SHAC	0.071429	0.071429	0	0.098361	0.008818	2.227273
2727	29-May-07	2007	SHAC	0.071429	0.071429	0	0.098361	0.008818	2.227273
2728	29-May-07	2007	SHAC	0.071429	0.071429	0	0.098361	0.008818	2.227273
2730	29-May-07	2007	SHAC	0.071429	0.071429	0	0.098361	0.008818	2.227273
2725	29-May-07	2007	SHAC	0.071429	0.071429	0	0.098361	0.008818	2.227273
2726	29-May-07	2007	SHAC	0.071429	0.071429	0	0.098361	0.008818	2.227273
2732	29-May-07	2007	SHAC	0.071429	0.071429	0	0.098361	0.008818	2.227273
2733	29-May-07	2007	SHAC	0.071429	0.071429	0	0.098361	0.008818	2.227273
2735	29-May-07	2007	SHAC	0.071429	0.071429	0	0.098361	0.008818	2.227273
2741	29-May-07	2007	SHAC	0.071429	0.071429	0	0.098361	0.008818	2.227273
2724	29-May-07	2007	SHAC	0.071429	0.071429	0	0.098361	0.008818	2.227273
2736	29-May-07	2007	SHAC	0.071429	0.071429	0	0.098361	0.008818	2.227273
2737	29-May-07	2007	SHAC	0.071429	0.071429	0	0.098361	0.008818	2.227273
2749	29-May-07	2007	SHAC	0.071429	0.071429	0	0.098361	0.008818	2.227273
2747	29-May-07	2007	SHAC	0.071429	0.071429	0	0.098361	0.008818	2.227273
2745	29-May-07	2007	SHAC	0.071429	0.071429	0	0.098361	0.008818	2.227273
2746	29-May-07	2007	SHAC	0.071429	0.071429	0	0.098361	0.008818	2.227273
2748	29-May-07	2007	SHAC	0.071429	0.071429	0	0.098361	0.008818	2.227273
2800	30-May-07	2007	YY	0.2	0.08	0.06	0.08	0	0.65
2801	30-May-07	2007	YY	0.2	0.08	0.06	0.08	0	0.65
2802	30-May-07	2007	YY	0.2	0.08	0.06	0.08	0	0.65
2803	30-May-07	2007	YY	0.2	0.08	0.06	0.08	0	0.65
2804	30-May-07	2007	YY	0.2	0.08	0.06	0.08	0	0.65
2807	30-May-07	2007	YY	0.2	0.08	0.06	0.08	0	0.65
2809	30-May-07	2007	YY	0.2	0.08	0.06	0.08	0	0.65
2810	30-May-07	2007	YY	0.2	0.08	0.06	0.08	0	0.65
2793	30-May-07	2007	YY	0.2	0.08	0.06	0.08	0	0.65
2794	30-May-07	2007	YY	0.2	0.08	0.06	0.08	0	0.65
2795	30-May-07	2007	YY	0.2	0.08	0.06	0.08	0	0.65
2796	30-May-07	2007	YY	0.2	0.08	0.06	0.08	0	0.65
2797	30-May-07	2007	YY	0.2	0.08	0.06	0.08	0	0.65

2805	30-May-07	2007	YY	0.2	0.08	0.06	0.08	0	0.65
2806	30-May-07	2007	YY	0.2	0.08	0.06	0.08	0	0.65
2811	30-May-07	2007	YY	0.2	0.08	0.06	0.08	0	0.65
2798	30-May-07	2007	YY	0.2	0.08	0.06	0.08	0	0.65
2792	30-May-07	2007	YY	0.2	0.08	0.06	0.08	0	0.65
2799	30-May-07	2007	YY	0.2	0.08	0.06	0.08	0	0.65
2808	30-May-07	2007	YY	0.2	0.08	0.06	0.08	0	0.65
2949	1-Jun-07	2007	RP	0.133333	0.033333	0.133333	0	0.019139	2.75
2950	1-Jun-07	2007	RP	0.133333	0.033333	0.133333	0	0.019139	2.75
2951	1-Jun-07	2007	RP	0.133333	0.033333	0.133333	0	0.019139	2.75
1325	7-May-08	2008	MG	0.387755	0.102041	0.061224	0.020408	0.007491	6.75
1324	7-May-08	2008	MG	0.387755	0.102041	0.061224	0.020408	0.007491	6.75
1326	7-May-08	2008	MG	0.387755	0.102041	0.061224	0.020408	0.007491	6.75
1328	7-May-08	2008	MG	0.387755	0.102041	0.061224	0.020408	0.007491	6.75
1321	7-May-08	2008	MG	0.387755	0.102041	0.061224	0.020408	0.007491	6.75
1319	7-May-08	2008	MG	0.387755	0.102041	0.061224	0.020408	0.007491	6.75
1322	7-May-08	2008	MG	0.387755	0.102041	0.061224	0.020408	0.007491	6.75
1320	7-May-08	2008	MG	0.387755	0.102041	0.061224	0.020408	0.007491	6.75
1318	7-May-08	2008	MG	0.387755	0.102041	0.061224	0.020408	0.007491	6.75
1323	7-May-08	2008	MG	0.387755	0.102041	0.061224	0.020408	0.007491	6.75
1327	7-May-08	2008	MG	0.387755	0.102041	0.061224	0.020408	0.007491	6.75
1329	7-May-08	2008	MG	0.387755	0.102041	0.061224	0.020408	0.007491	6.75
1317	7-May-08	2008	MG	0.387755	0.102041	0.061224	0.020408	0.007491	6.75
113	16-May-08	2008	TO	0.2	0.2	0.05	0.025	0.00289	0.875
116	16-May-08	2008	TO	0.2	0.2	0.05	0.025	0.00289	0.875
110	16-May-08	2008	TO	0.2	0.2	0.05	0.025	0.00289	0.875
108	16-May-08	2008	TO	0.2	0.2	0.05	0.025	0.00289	0.875
111	16-May-08	2008	TO	0.2	0.2	0.05	0.025	0.00289	0.875
115	16-May-08	2008	TO	0.2	0.2	0.05	0.025	0.00289	0.875
114	16-May-08	2008	TO	0.2	0.2	0.05	0.025	0.00289	0.875
118	16-May-08	2008	TO	0.2	0.2	0.05	0.025	0.00289	0.875
119	16-May-08	2008	TO	0.2	0.2	0.05	0.025	0.00289	0.875
120	16-May-08	2008	TO	0.2	0.2	0.05	0.025	0.00289	0.875
109	16-May-08	2008	TO	0.2	0.2	0.05	0.025	0.00289	0.875
107	16-May-08	2008	TO	0.2	0.2	0.05	0.025	0.00289	0.875
112	16-May-08	2008	TO	0.2	0.2	0.05	0.025	0.00289	0.875
117	16-May-08	2008	TO	0.2	0.2	0.05	0.025	0.00289	0.875
121	16-May-08	2008	TO	0.2	0.2	0.05	0.025	0.00289	0.875
106	16-May-08	2008	TO	0.2	0.2	0.05	0.025	0.00289	0.875
158	16-May-08	2008	TO	0.2	0.2	0.05	0.025	0.00289	0.875
159	16-May-08	2008	TO	0.2	0.2	0.05	0.025	0.00289	0.875
157	16-May-08	2008	TO	0.2	0.2	0.05	0.025	0.00289	0.875
160	16-May-08	2008	TO	0.2	0.2	0.05	0.025	0.00289	0.875
162	16-May-08	2008	TO	0.2	0.2	0.05	0.025	0.00289	0.875

165	16-May-08	2008	TO	0.2	0.2	0.05	0.025	0.00289	0.875
156	16-May-08	2008	TO	0.2	0.2	0.05	0.025	0.00289	0.875
164	16-May-08	2008	TO	0.2	0.2	0.05	0.025	0.00289	0.875
155	16-May-08	2008	TO	0.2	0.2	0.05	0.025	0.00289	0.875
161	16-May-08	2008	TO	0.2	0.2	0.05	0.025	0.00289	0.875
163	16-May-08	2008	TO	0.2	0.2	0.05	0.025	0.00289	0.875
166	16-May-08	2008	TO	0.2	0.2	0.05	0.025	0.00289	0.875
154	16-May-08	2008	TO	0.2	0.2	0.05	0.025	0.00289	0.875
1374	17-May-08	2008	YY	0.14	0.24	0.08	0.02	0	0.65
1373	17-May-08	2008	YY	0.14	0.24	0.08	0.02	0	0.65
1372	17-May-08	2008	YY	0.14	0.24	0.08	0.02	0	0.65
1370	17-May-08	2008	YY	0.14	0.24	0.08	0.02	0	0.65
1371	17-May-08	2008	YY	0.14	0.24	0.08	0.02	0	0.65
1375	17-May-08	2008	YY	0.14	0.24	0.08	0.02	0	0.65
1376	17-May-08	2008	YY	0.14	0.24	0.08	0.02	0	0.65
1367	17-May-08	2008	YY	0.14	0.24	0.08	0.02	0	0.65
1366	17-May-08	2008	YY	0.14	0.24	0.08	0.02	0	0.65
1368	17-May-08	2008	YY	0.14	0.24	0.08	0.02	0	0.65
1369	17-May-08	2008	YY	0.14	0.24	0.08	0.02	0	0.65
1377	17-May-08	2008	YY	0.14	0.24	0.08	0.02	0	0.65
1365	17-May-08	2008	YY	0.14	0.24	0.08	0.02	0	0.65
279	18-May-08	2008	SW	0.368421	0.157895	0.052632	0	0	1.466667
278	18-May-08	2008	SW	0.368421	0.157895	0.052632	0	0	1.466667
259	18-May-08	2008	SW	0.368421	0.157895	0.052632	0	0	1.466667
272	18-May-08	2008	SW	0.368421	0.157895	0.052632	0	0	1.466667
238	18-May-08	2008	SW	0.368421	0.157895	0.052632	0	0	1.466667
240	18-May-08	2008	SW	0.368421	0.157895	0.052632	0	0	1.466667
241	18-May-08	2008	SW	0.368421	0.157895	0.052632	0	0	1.466667
244	18-May-08	2008	SW	0.368421	0.157895	0.052632	0	0	1.466667
245	18-May-08	2008	SW	0.368421	0.157895	0.052632	0	0	1.466667
246	18-May-08	2008	SW	0.368421	0.157895	0.052632	0	0	1.466667
249	18-May-08	2008	SW	0.368421	0.157895	0.052632	0	0	1.466667
250	18-May-08	2008	SW	0.368421	0.157895	0.052632	0	0	1.466667
251	18-May-08	2008	SW	0.368421	0.157895	0.052632	0	0	1.466667
252	18-May-08	2008	SW	0.368421	0.157895	0.052632	0	0	1.466667
268	18-May-08	2008	SW	0.368421	0.157895	0.052632	0	0	1.466667
269	18-May-08	2008	SW	0.368421	0.157895	0.052632	0	0	1.466667
236	18-May-08	2008	SW	0.368421	0.157895	0.052632	0	0	1.466667
237	18-May-08	2008	SW	0.368421	0.157895	0.052632	0	0	1.466667
262	18-May-08	2008	SW	0.368421	0.157895	0.052632	0	0	1.466667
265	18-May-08	2008	SW	0.368421	0.157895	0.052632	0	0	1.466667
234	18-May-08	2008	SW	0.368421	0.157895	0.052632	0	0	1.466667
235	18-May-08	2008	SW	0.368421	0.157895	0.052632	0	0	1.466667
247	18-May-08	2008	SW	0.368421	0.157895	0.052632	0	0	1.466667

266	18-May-08	2008	SW	0.368421	0.157895	0.052632	0	0	1.466667
275	18-May-08	2008	SW	0.368421	0.157895	0.052632	0	0	1.466667
239	18-May-08	2008	SW	0.368421	0.157895	0.052632	0	0	1.466667
263	18-May-08	2008	SW	0.368421	0.157895	0.052632	0	0	1.466667
271	18-May-08	2008	SW	0.368421	0.157895	0.052632	0	0	1.466667
243	18-May-08	2008	SW	0.368421	0.157895	0.052632	0	0	1.466667
248	18-May-08	2008	SW	0.368421	0.157895	0.052632	0	0	1.466667
258	18-May-08	2008	SW	0.368421	0.157895	0.052632	0	0	1.466667
276	18-May-08	2008	SW	0.368421	0.157895	0.052632	0	0	1.466667
257	18-May-08	2008	SW	0.368421	0.157895	0.052632	0	0	1.466667
260	18-May-08	2008	SW	0.368421	0.157895	0.052632	0	0	1.466667
267	18-May-08	2008	SW	0.368421	0.157895	0.052632	0	0	1.466667
273	18-May-08	2008	SW	0.368421	0.157895	0.052632	0	0	1.466667
253	18-May-08	2008	SW	0.368421	0.157895	0.052632	0	0	1.466667
255	18-May-08	2008	SW	0.368421	0.157895	0.052632	0	0	1.466667
270	18-May-08	2008	SW	0.368421	0.157895	0.052632	0	0	1.466667
274	18-May-08	2008	SW	0.368421	0.157895	0.052632	0	0	1.466667
256	18-May-08	2008	SW	0.368421	0.157895	0.052632	0	0	1.466667
261	18-May-08	2008	SW	0.368421	0.157895	0.052632	0	0	1.466667
242	18-May-08	2008	SW	0.368421	0.157895	0.052632	0	0	1.466667
277	18-May-08	2008	SW	0.368421	0.157895	0.052632	0	0	1.466667
233	18-May-08	2008	SW	0.368421	0.157895	0.052632	0	0	1.466667
254	18-May-08	2008	SW	0.368421	0.157895	0.052632	0	0	1.466667
264	18-May-08	2008	SW	0.368421	0.157895	0.052632	0	0	1.466667
280	18-May-08	2008	SW	0.368421	0.157895	0.052632	0	0	1.466667
232	18-May-08	2008	SW	0.368421	0.157895	0.052632	0	0	1.466667
398	21-May-08	2008	AFF						
394	21-May-08	2008	AFF						
385	21-May-08	2008	AFF						
387	21-May-08	2008	AFF						
393	21-May-08	2008	AFF						
399	21-May-08	2008	AFF						
386	21-May-08	2008	AFF						
388	21-May-08	2008	AFF						
390	21-May-08	2008	AFF						
392	21-May-08	2008	AFF						
396	21-May-08	2008	AFF						
397	21-May-08	2008	AFF						
401	21-May-08	2008	AFF						
402	21-May-08	2008	AFF						
405	21-May-08	2008	AFF						
384	21-May-08	2008	AFF						
379	21-May-08	2008	AFF						
400	21-May-08	2008	AFF						

403	21-May-08	2008	AFF						
406	21-May-08	2008	AFF						
380	21-May-08	2008	AFF						
381	21-May-08	2008	AFF						
389	21-May-08	2008	AFF						
391	21-May-08	2008	AFF						
395	21-May-08	2008	AFF						
382	21-May-08	2008	AFF						
378	21-May-08	2008	AFF						
383	21-May-08	2008	AFF						
404	21-May-08	2008	AFF						
407	21-May-08	2008	AFF						
377	21-May-08	2008	AFF						
552	23-May-08	2008	RL	0.210526	0.131579	0.026316	0.078947	0.018018	0.333333
555	23-May-08	2008	RL	0.210526	0.131579	0.026316	0.078947	0.018018	0.333333
553	23-May-08	2008	RL	0.210526	0.131579	0.026316	0.078947	0.018018	0.333333
554	23-May-08	2008	RL	0.210526	0.131579	0.026316	0.078947	0.018018	0.333333
556	23-May-08	2008	RL	0.210526	0.131579	0.026316	0.078947	0.018018	0.333333
557	23-May-08	2008	RL	0.210526	0.131579	0.026316	0.078947	0.018018	0.333333
562	23-May-08	2008	RL	0.210526	0.131579	0.026316	0.078947	0.018018	0.333333
561	23-May-08	2008	RL	0.210526	0.131579	0.026316	0.078947	0.018018	0.333333
560	23-May-08	2008	RL	0.210526	0.131579	0.026316	0.078947	0.018018	0.333333
563	23-May-08	2008	RL	0.210526	0.131579	0.026316	0.078947	0.018018	0.333333
564	23-May-08	2008	RL	0.210526	0.131579	0.026316	0.078947	0.018018	0.333333
565	23-May-08	2008	RL	0.210526	0.131579	0.026316	0.078947	0.018018	0.333333
566	23-May-08	2008	RL	0.210526	0.131579	0.026316	0.078947	0.018018	0.333333
559	23-May-08	2008	RL	0.210526	0.131579	0.026316	0.078947	0.018018	0.333333
550	23-May-08	2008	RL	0.210526	0.131579	0.026316	0.078947	0.018018	0.333333
551	23-May-08	2008	RL	0.210526	0.131579	0.026316	0.078947	0.018018	0.333333
558	23-May-08	2008	RL	0.210526	0.131579	0.026316	0.078947	0.018018	0.333333
567	23-May-08	2008	RL	0.210526	0.131579	0.026316	0.078947	0.018018	0.333333
549	23-May-08	2008	RL	0.210526	0.131579	0.026316	0.078947	0.018018	0.333333
626	24-May-08	2008	SC	0.191781	0.013699	0.123288	0	0	8.615385
629	24-May-08	2008	SC	0.191781	0.013699	0.123288	0	0	8.615385
628	24-May-08	2008	SC	0.191781	0.013699	0.123288	0	0	8.615385
630	24-May-08	2008	SC	0.191781	0.013699	0.123288	0	0	8.615385
631	24-May-08	2008	SC	0.191781	0.013699	0.123288	0	0	8.615385
627	24-May-08	2008	SC	0.191781	0.013699	0.123288	0	0	8.615385
624	24-May-08	2008	SC	0.191781	0.013699	0.123288	0	0	8.615385
625	24-May-08	2008	SC	0.191781	0.013699	0.123288	0	0	8.615385
632	24-May-08	2008	SC	0.191781	0.013699	0.123288	0	0	8.615385
636	24-May-08	2008	SC	0.191781	0.013699	0.123288	0	0	8.615385
637	24-May-08	2008	SC	0.191781	0.013699	0.123288	0	0	8.615385
633	24-May-08	2008	SC	0.191781	0.013699	0.123288	0	0	8.615385

664	24-May-08	2008 SC	0.191781	0.013699	0.123288	0	0	8.615385
652	24-May-08	2008 SC	0.191781	0.013699	0.123288	0	0	8.615385
650	24-May-08	2008 SC	0.191781	0.013699	0.123288	0	0	8.615385
647	24-May-08	2008 SC	0.191781	0.013699	0.123288	0	0	8.615385
651	24-May-08	2008 SC	0.191781	0.013699	0.123288	0	0	8.615385
654	24-May-08	2008 SC	0.191781	0.013699	0.123288	0	0	8.615385
641	24-May-08	2008 SC	0.191781	0.013699	0.123288	0	0	8.615385
642	24-May-08	2008 SC	0.191781	0.013699	0.123288	0	0	8.615385
644	24-May-08	2008 SC	0.191781	0.013699	0.123288	0	0	8.615385
648	24-May-08	2008 SC	0.191781	0.013699	0.123288	0	0	8.615385
646	24-May-08	2008 SC	0.191781	0.013699	0.123288	0	0	8.615385
643	24-May-08	2008 SC	0.191781	0.013699	0.123288	0	0	8.615385
645	24-May-08	2008 SC	0.191781	0.013699	0.123288	0	0	8.615385
655	24-May-08	2008 SC	0.191781	0.013699	0.123288	0	0	8.615385
649	24-May-08	2008 SC	0.191781	0.013699	0.123288	0	0	8.615385
653	24-May-08	2008 SC	0.191781	0.013699	0.123288	0	0	8.615385
656	24-May-08	2008 SC	0.191781	0.013699	0.123288	0	0	8.615385
657	24-May-08	2008 SC	0.191781	0.013699	0.123288	0	0	8.615385
658	24-May-08	2008 SC	0.191781	0.013699	0.123288	0	0	8.615385
659	24-May-08	2008 SC	0.191781	0.013699	0.123288	0	0	8.615385
660	24-May-08	2008 SC	0.191781	0.013699	0.123288	0	0	8.615385
639	24-May-08	2008 SC	0.191781	0.013699	0.123288	0	0	8.615385
640	24-May-08	2008 SC	0.191781	0.013699	0.123288	0	0	8.615385
662	24-May-08	2008 SC	0.191781	0.013699	0.123288	0	0	8.615385
666	24-May-08	2008 SC	0.191781	0.013699	0.123288	0	0	8.615385
663	24-May-08	2008 SC	0.191781	0.013699	0.123288	0	0	8.615385
634	24-May-08	2008 SC	0.191781	0.013699	0.123288	0	0	8.615385
665	24-May-08	2008 SC	0.191781	0.013699	0.123288	0	0	8.615385
635	24-May-08	2008 SC	0.191781	0.013699	0.123288	0	0	8.615385
623	24-May-08	2008 SC	0.191781	0.013699	0.123288	0	0	8.615385
638	24-May-08	2008 SC	0.191781	0.013699	0.123288	0	0	8.615385
661	24-May-08	2008 SC	0.191781	0.013699	0.123288	0	0	8.615385
667	24-May-08	2008 SC	0.191781	0.013699	0.123288	0	0	8.615385
622	24-May-08	2008 SC	0.191781	0.013699	0.123288	0	0	8.615385
699	24-May-08	2008 SC	0.191781	0.013699	0.123288	0	0	8.615385
712	24-May-08	2008 SC	0.191781	0.013699	0.123288	0	0	8.615385
726	24-May-08	2008 SC	0.191781	0.013699	0.123288	0	0	8.615385
727	24-May-08	2008 SC	0.191781	0.013699	0.123288	0	0	8.615385
725	24-May-08	2008 SC	0.191781	0.013699	0.123288	0	0	8.615385
703	24-May-08	2008 SC	0.191781	0.013699	0.123288	0	0	8.615385
723	24-May-08	2008 SC	0.191781	0.013699	0.123288	0	0	8.615385
728	24-May-08	2008 SC	0.191781	0.013699	0.123288	0	0	8.615385
710	24-May-08	2008 SC	0.191781	0.013699	0.123288	0	0	8.615385
717	24-May-08	2008 SC	0.191781	0.013699	0.123288	0	0	8.615385

716	24-May-08	2008	SC	0.191781	0.013699	0.123288	0	0	8.615385
718	24-May-08	2008	SC	0.191781	0.013699	0.123288	0	0	8.615385
719	24-May-08	2008	SC	0.191781	0.013699	0.123288	0	0	8.615385
715	24-May-08	2008	SC	0.191781	0.013699	0.123288	0	0	8.615385
708	24-May-08	2008	SC	0.191781	0.013699	0.123288	0	0	8.615385
714	24-May-08	2008	SC	0.191781	0.013699	0.123288	0	0	8.615385
720	24-May-08	2008	SC	0.191781	0.013699	0.123288	0	0	8.615385
706	24-May-08	2008	SC	0.191781	0.013699	0.123288	0	0	8.615385
721	24-May-08	2008	SC	0.191781	0.013699	0.123288	0	0	8.615385
704	24-May-08	2008	SC	0.191781	0.013699	0.123288	0	0	8.615385
701	24-May-08	2008	SC	0.191781	0.013699	0.123288	0	0	8.615385
702	24-May-08	2008	SC	0.191781	0.013699	0.123288	0	0	8.615385
709	24-May-08	2008	SC	0.191781	0.013699	0.123288	0	0	8.615385
713	24-May-08	2008	SC	0.191781	0.013699	0.123288	0	0	8.615385
722	24-May-08	2008	SC	0.191781	0.013699	0.123288	0	0	8.615385
711	24-May-08	2008	SC	0.191781	0.013699	0.123288	0	0	8.615385
700	24-May-08	2008	SC	0.191781	0.013699	0.123288	0	0	8.615385
707	24-May-08	2008	SC	0.191781	0.013699	0.123288	0	0	8.615385
724	24-May-08	2008	SC	0.191781	0.013699	0.123288	0	0	8.615385
729	24-May-08	2008	SC	0.191781	0.013699	0.123288	0	0	8.615385
705	24-May-08	2008	SC	0.191781	0.013699	0.123288	0	0	8.615385
698	24-May-08	2008	SC	0.191781	0.013699	0.123288	0	0	8.615385
770	25-May-08	2008	YY	0.14	0.24	0.08	0.02	0	0.65
767	25-May-08	2008	YY	0.14	0.24	0.08	0.02	0	0.65
769	25-May-08	2008	YY	0.14	0.24	0.08	0.02	0	0.65
772	25-May-08	2008	YY	0.14	0.24	0.08	0.02	0	0.65
774	25-May-08	2008	YY	0.14	0.24	0.08	0.02	0	0.65
775	25-May-08	2008	YY	0.14	0.24	0.08	0.02	0	0.65
776	25-May-08	2008	YY	0.14	0.24	0.08	0.02	0	0.65
777	25-May-08	2008	YY	0.14	0.24	0.08	0.02	0	0.65
778	25-May-08	2008	YY	0.14	0.24	0.08	0.02	0	0.65
766	25-May-08	2008	YY	0.14	0.24	0.08	0.02	0	0.65
771	25-May-08	2008	YY	0.14	0.24	0.08	0.02	0	0.65
765	25-May-08	2008	YY	0.14	0.24	0.08	0.02	0	0.65
768	25-May-08	2008	YY	0.14	0.24	0.08	0.02	0	0.65
773	25-May-08	2008	YY	0.14	0.24	0.08	0.02	0	0.65
779	25-May-08	2008	YY	0.14	0.24	0.08	0.02	0	0.65
764	25-May-08	2008	YY	0.14	0.24	0.08	0.02	0	0.65
1093	3-Jun-08	2008	D1	0.2	0.28	0.04	0.02	0.006036	0.8
1094	3-Jun-08	2008	D1	0.2	0.28	0.04	0.02	0.006036	0.8
1098	3-Jun-08	2008	D1	0.2	0.28	0.04	0.02	0.006036	0.8
1099	3-Jun-08	2008	D1	0.2	0.28	0.04	0.02	0.006036	0.8
1097	3-Jun-08	2008	D1	0.2	0.28	0.04	0.02	0.006036	0.8
1092	3-Jun-08	2008	D1	0.2	0.28	0.04	0.02	0.006036	0.8

1100	3-Jun-08	2008	D1	0.2	0.28	0.04	0.02	0.006036	0.8
1091	3-Jun-08	2008	D1	0.2	0.28	0.04	0.02	0.006036	0.8
1069	3-Jun-08	2008	D1	0.2	0.28	0.04	0.02	0.006036	0.8
1095	3-Jun-08	2008	D1	0.2	0.28	0.04	0.02	0.006036	0.8
1068	3-Jun-08	2008	D1	0.2	0.28	0.04	0.02	0.006036	0.8
1096	3-Jun-08	2008	D1	0.2	0.28	0.04	0.02	0.006036	0.8
1073	3-Jun-08	2008	D1	0.2	0.28	0.04	0.02	0.006036	0.8
1072	3-Jun-08	2008	D1	0.2	0.28	0.04	0.02	0.006036	0.8
1074	3-Jun-08	2008	D1	0.2	0.28	0.04	0.02	0.006036	0.8
1075	3-Jun-08	2008	D1	0.2	0.28	0.04	0.02	0.006036	0.8
1071	3-Jun-08	2008	D1	0.2	0.28	0.04	0.02	0.006036	0.8
1076	3-Jun-08	2008	D1	0.2	0.28	0.04	0.02	0.006036	0.8
1077	3-Jun-08	2008	D1	0.2	0.28	0.04	0.02	0.006036	0.8
1078	3-Jun-08	2008	D1	0.2	0.28	0.04	0.02	0.006036	0.8
1079	3-Jun-08	2008	D1	0.2	0.28	0.04	0.02	0.006036	0.8
1080	3-Jun-08	2008	D1	0.2	0.28	0.04	0.02	0.006036	0.8
1081	3-Jun-08	2008	D1	0.2	0.28	0.04	0.02	0.006036	0.8
1082	3-Jun-08	2008	D1	0.2	0.28	0.04	0.02	0.006036	0.8
1084	3-Jun-08	2008	D1	0.2	0.28	0.04	0.02	0.006036	0.8
1086	3-Jun-08	2008	D1	0.2	0.28	0.04	0.02	0.006036	0.8
1088	3-Jun-08	2008	D1	0.2	0.28	0.04	0.02	0.006036	0.8
1085	3-Jun-08	2008	D1	0.2	0.28	0.04	0.02	0.006036	0.8
1090	3-Jun-08	2008	D1	0.2	0.28	0.04	0.02	0.006036	0.8
1083	3-Jun-08	2008	D1	0.2	0.28	0.04	0.02	0.006036	0.8
1087	3-Jun-08	2008	D1	0.2	0.28	0.04	0.02	0.006036	0.8
1101	3-Jun-08	2008	D1	0.2	0.28	0.04	0.02	0.006036	0.8
1067	3-Jun-08	2008	D1	0.2	0.28	0.04	0.02	0.006036	0.8
1070	3-Jun-08	2008	D1	0.2	0.28	0.04	0.02	0.006036	0.8
1089	3-Jun-08	2008	D1	0.2	0.28	0.04	0.02	0.006036	0.8
1102	3-Jun-08	2008	D1	0.2	0.28	0.04	0.02	0.006036	0.8
1066	3-Jun-08	2008	D1	0.2	0.28	0.04	0.02	0.006036	0.8
2093	same date	2007	SD						
2088	same date	2007	SD						
2087	same date	2007	SD						
2091	same date	2007	SD						
2081	same date	2007	SD						
2084	same date	2007	SD						
2079	same date	2007	SD						
2080	same date	2007	SD						
2085	same date	2007	SD						
2077	same date	2007	SD						
2076	same date	2007	SD						
2082	same date	2007	SD						
2070	same date	2007	SD						

2090	same date	2007	SD
2092	same date	2007	SD
2094	same date	2007	SD
2066	same date	2007	SD
2067	same date	2007	SD
2065	same date	2007	SD
2072	same date	2007	SD
2064	same date	2007	SD
2068	same date	2007	SD
2073	same date	2007	SD
2086	same date	2007	SD
2078	same date	2007	SD
2095	same date	2007	SD
2071	same date	2007	SD
2069	same date	2007	SD
2083	same date	2007	SD
2062	same date	2007	SD
2075	same date	2007	SD
2089	same date	2007	SD
2096	same date	2007	SD
2063	same date	2007	SD
2074	same date	2007	SD
2061	same date	2007	SD
1997	UK 1	2007	SD
2004	UK 1	2007	SD
1998	UK 1	2007	SD
2012	UK 1	2007	SD
2013	UK 1	2007	SD
2014	UK 1	2007	SD
2015	UK 1	2007	SD
1999	UK 1	2007	SD
2011	UK 1	2007	SD
2010	UK 1	2007	SD
2016	UK 1	2007	SD
2017	UK 1	2007	SD
1996	UK 1	2007	SD
2003	UK 1	2007	SD
2024	UK 1	2007	SD
2025	UK 1	2007	SD
2002	UK 1	2007	SD
2005	UK 1	2007	SD
2006	UK 1	2007	SD
2022	UK 1	2007	SD
2023	UK 1	2007	SD

1995	UK 1	2007	SD						
2018	UK 1	2007	SD						
2000	UK 1	2007	SD						
2021	UK 1	2007	SD						
2020	UK 1	2007	SD						
2001	UK 1	2007	SD						
2009	UK 1	2007	SD						
1994	UK 1	2007	SD						
2007	UK 1	2007	SD						
2019	UK 1	2007	SD						
2026	UK 1	2007	SD						
2008	UK 1	2007	SD						
1993	UK 1	2007	SD						
126	27-May-04	2004	SC	0.174312	0.009174	0.06422	0.018349	0.103343	6.846154
130	27-May-04	2004	SC	0.174312	0.009174	0.06422	0.018349	0.103343	6.846154
131	27-May-04	2004	SC	0.174312	0.009174	0.06422	0.018349	0.103343	6.846154
135	27-May-04	2004	SC	0.174312	0.009174	0.06422	0.018349	0.103343	6.846154
136	27-May-04	2004	SC	0.174312	0.009174	0.06422	0.018349	0.103343	6.846154
140	27-May-04	2004	SC	0.174312	0.009174	0.06422	0.018349	0.103343	6.846154
144	27-May-04	2004	SC	0.174312	0.009174	0.06422	0.018349	0.103343	6.846154
133	27-May-04	2004	SC	0.174312	0.009174	0.06422	0.018349	0.103343	6.846154
134	27-May-04	2004	SC	0.174312	0.009174	0.06422	0.018349	0.103343	6.846154
141	27-May-04	2004	SC	0.174312	0.009174	0.06422	0.018349	0.103343	6.846154
143	27-May-04	2004	SC	0.174312	0.009174	0.06422	0.018349	0.103343	6.846154
129	27-May-04	2004	SC	0.174312	0.009174	0.06422	0.018349	0.103343	6.846154
124	27-May-04	2004	SC	0.174312	0.009174	0.06422	0.018349	0.103343	6.846154
125	27-May-04	2004	SC	0.174312	0.009174	0.06422	0.018349	0.103343	6.846154
128	27-May-04	2004	SC	0.174312	0.009174	0.06422	0.018349	0.103343	6.846154
132	27-May-04	2004	SC	0.174312	0.009174	0.06422	0.018349	0.103343	6.846154
137	27-May-04	2004	SC	0.174312	0.009174	0.06422	0.018349	0.103343	6.846154
139	27-May-04	2004	SC	0.174312	0.009174	0.06422	0.018349	0.103343	6.846154
142	27-May-04	2004	SC	0.174312	0.009174	0.06422	0.018349	0.103343	6.846154
127	27-May-04	2004	SC	0.174312	0.009174	0.06422	0.018349	0.103343	6.846154
123	27-May-04	2004	SC	0.174312	0.009174	0.06422	0.018349	0.103343	6.846154
138	27-May-04	2004	SC	0.174312	0.009174	0.06422	0.018349	0.103343	6.846154
235	11-Jun-04	2004	SC	0.174312	0.009174	0.06422	0.018349	0.103343	6.846154
229	11-Jun-04	2004	SC	0.174312	0.009174	0.06422	0.018349	0.103343	6.846154
231	11-Jun-04	2004	SC	0.174312	0.009174	0.06422	0.018349	0.103343	6.846154
227	11-Jun-04	2004	SC	0.174312	0.009174	0.06422	0.018349	0.103343	6.846154
228	11-Jun-04	2004	SC	0.174312	0.009174	0.06422	0.018349	0.103343	6.846154
238	11-Jun-04	2004	SC	0.174312	0.009174	0.06422	0.018349	0.103343	6.846154
240	11-Jun-04	2004	SC	0.174312	0.009174	0.06422	0.018349	0.103343	6.846154
241	11-Jun-04	2004	SC	0.174312	0.009174	0.06422	0.018349	0.103343	6.846154
220	11-Jun-04	2004	SC	0.174312	0.009174	0.06422	0.018349	0.103343	6.846154

233	11-Jun-04	2004	SC	0.174312	0.009174	0.06422	0.018349	0.103343	6.846154
221	11-Jun-04	2004	SC	0.174312	0.009174	0.06422	0.018349	0.103343	6.846154
223	11-Jun-04	2004	SC	0.174312	0.009174	0.06422	0.018349	0.103343	6.846154
224	11-Jun-04	2004	SC	0.174312	0.009174	0.06422	0.018349	0.103343	6.846154
232	11-Jun-04	2004	SC	0.174312	0.009174	0.06422	0.018349	0.103343	6.846154
236	11-Jun-04	2004	SC	0.174312	0.009174	0.06422	0.018349	0.103343	6.846154
237	11-Jun-04	2004	SC	0.174312	0.009174	0.06422	0.018349	0.103343	6.846154
239	11-Jun-04	2004	SC	0.174312	0.009174	0.06422	0.018349	0.103343	6.846154
245	11-Jun-04	2004	SC	0.174312	0.009174	0.06422	0.018349	0.103343	6.846154
246	11-Jun-04	2004	SC	0.174312	0.009174	0.06422	0.018349	0.103343	6.846154
225	11-Jun-04	2004	SC	0.174312	0.009174	0.06422	0.018349	0.103343	6.846154
226	11-Jun-04	2004	SC	0.174312	0.009174	0.06422	0.018349	0.103343	6.846154
234	11-Jun-04	2004	SC	0.174312	0.009174	0.06422	0.018349	0.103343	6.846154
242	11-Jun-04	2004	SC	0.174312	0.009174	0.06422	0.018349	0.103343	6.846154
243	11-Jun-04	2004	SC	0.174312	0.009174	0.06422	0.018349	0.103343	6.846154
244	11-Jun-04	2004	SC	0.174312	0.009174	0.06422	0.018349	0.103343	6.846154
230	11-Jun-04	2004	SC	0.174312	0.009174	0.06422	0.018349	0.103343	6.846154
222	11-Jun-04	2004	SC	0.174312	0.009174	0.06422	0.018349	0.103343	6.846154
551	16-Jun-04	2004	RP	0.095238	0.142857	0.095238	0.071429	0.323383	2
558	16-Jun-04	2004	RP	0.095238	0.142857	0.095238	0.071429	0.323383	2
563	16-Jun-04	2004	RP	0.095238	0.142857	0.095238	0.071429	0.323383	2
564	16-Jun-04	2004	RP	0.095238	0.142857	0.095238	0.071429	0.323383	2
561	16-Jun-04	2004	RP	0.095238	0.142857	0.095238	0.071429	0.323383	2
562	16-Jun-04	2004	RP	0.095238	0.142857	0.095238	0.071429	0.323383	2
554	16-Jun-04	2004	RP	0.095238	0.142857	0.095238	0.071429	0.323383	2
555	16-Jun-04	2004	RP	0.095238	0.142857	0.095238	0.071429	0.323383	2
557	16-Jun-04	2004	RP	0.095238	0.142857	0.095238	0.071429	0.323383	2
565	16-Jun-04	2004	RP	0.095238	0.142857	0.095238	0.071429	0.323383	2
556	16-Jun-04	2004	RP	0.095238	0.142857	0.095238	0.071429	0.323383	2
567	16-Jun-04	2004	RP	0.095238	0.142857	0.095238	0.071429	0.323383	2
568	16-Jun-04	2004	RP	0.095238	0.142857	0.095238	0.071429	0.323383	2
552	16-Jun-04	2004	RP	0.095238	0.142857	0.095238	0.071429	0.323383	2
560	16-Jun-04	2004	RP	0.095238	0.142857	0.095238	0.071429	0.323383	2
566	16-Jun-04	2004	RP	0.095238	0.142857	0.095238	0.071429	0.323383	2
559	16-Jun-04	2004	RP	0.095238	0.142857	0.095238	0.071429	0.323383	2
553	16-Jun-04	2004	RP	0.095238	0.142857	0.095238	0.071429	0.323383	2
1300	1-Jun-05	2005	D1/D2						
1295	1-Jun-05	2005	D1/D2						
1292	1-Jun-05	2005	D1/D2						
1294	1-Jun-05	2005	D1/D2						
1297	1-Jun-05	2005	D1/D2						
1299	1-Jun-05	2005	D1/D2						
1296	1-Jun-05	2005	D1/D2						
1298	1-Jun-05	2005	D1/D2						

1293	1-Jun-05	2005	D1/D2						
1301	1-Jun-05	2005	D1/D2						
1291	1-Jun-05	2005	D1/D2						
1646	14-Jun-05	2005	7M	0.264151	0.09434	0	0.301887	0.01519	8.266667
1647	14-Jun-05	2005	7M	0.264151	0.09434	0	0.301887	0.01519	8.266667
1648	14-Jun-05	2005	7M	0.264151	0.09434	0	0.301887	0.01519	8.266667
1649	14-Jun-05	2005	7M	0.264151	0.09434	0	0.301887	0.01519	8.266667
1645	14-Jun-05	2005	7M	0.264151	0.09434	0	0.301887	0.01519	8.266667
1644	14-Jun-05	2005	7M	0.264151	0.09434	0	0.301887	0.01519	8.266667
1650	14-Jun-05	2005	7M	0.264151	0.09434	0	0.301887	0.01519	8.266667
1651	14-Jun-05	2005	7M	0.264151	0.09434	0	0.301887	0.01519	8.266667
1642	14-Jun-05	2005	7M	0.264151	0.09434	0	0.301887	0.01519	8.266667
1643	14-Jun-05	2005	7M	0.264151	0.09434	0	0.301887	0.01519	8.266667
1653	14-Jun-05	2005	7M	0.264151	0.09434	0	0.301887	0.01519	8.266667
1652	14-Jun-05	2005	7M	0.264151	0.09434	0	0.301887	0.01519	8.266667
1654	14-Jun-05	2005	7M	0.264151	0.09434	0	0.301887	0.01519	8.266667
2574	26-May-07	2007	SD						
2590	26-May-07	2007	SD						
2571	26-May-07	2007	SD						
2572	26-May-07	2007	SD						
2591	26-May-07	2007	SD						
2576	26-May-07	2007	SD						
2588	26-May-07	2007	SD						
2570	26-May-07	2007	SD						
2559	26-May-07	2007	SD						
2560	26-May-07	2007	SD						
2561	26-May-07	2007	SD						
2563	26-May-07	2007	SD						
2569	26-May-07	2007	SD						
2562	26-May-07	2007	SD						
2564	26-May-07	2007	SD						
2579	26-May-07	2007	SD						
2558	26-May-07	2007	SD						
2575	26-May-07	2007	SD						
2568	26-May-07	2007	SD						
2553	26-May-07	2007	SD						
2557	26-May-07	2007	SD						
2578	26-May-07	2007	SD						
2555	26-May-07	2007	SD						
2556	26-May-07	2007	SD						
2566	26-May-07	2007	SD						
2593	26-May-07	2007	SD						
2552	26-May-07	2007	SD						
2554	26-May-07	2007	SD						

2583	26-May-07	2007	SD						
2580	26-May-07	2007	SD						
2582	26-May-07	2007	SD						
2581	26-May-07	2007	SD						
2584	26-May-07	2007	SD						
2585	26-May-07	2007	SD						
2587	26-May-07	2007	SD						
2573	26-May-07	2007	SD						
2589	26-May-07	2007	SD						
2592	26-May-07	2007	SD						
2565	26-May-07	2007	SD						
2586	26-May-07	2007	SD						
2551	26-May-07	2007	SD						
2567	26-May-07	2007	SD						
2577	26-May-07	2007	SD						
2999	2-Jun-07	2007	LT1	0.521739	0	0.130435	0	0	3.555556
2998	2-Jun-07	2007	LT1	0.521739	0	0.130435	0	0	3.555556
2990	2-Jun-07	2007	LT1	0.521739	0	0.130435	0	0	3.555556
2986	2-Jun-07	2007	LT1	0.521739	0	0.130435	0	0	3.555556
2991	2-Jun-07	2007	LT1	0.521739	0	0.130435	0	0	3.555556
2985	2-Jun-07	2007	LT1	0.521739	0	0.130435	0	0	3.555556
2987	2-Jun-07	2007	LT1	0.521739	0	0.130435	0	0	3.555556
2988	2-Jun-07	2007	LT1	0.521739	0	0.130435	0	0	3.555556
2989	2-Jun-07	2007	LT1	0.521739	0	0.130435	0	0	3.555556
2992	2-Jun-07	2007	LT1	0.521739	0	0.130435	0	0	3.555556
2993	2-Jun-07	2007	LT1	0.521739	0	0.130435	0	0	3.555556
2994	2-Jun-07	2007	LT1	0.521739	0	0.130435	0	0	3.555556
2995	2-Jun-07	2007	LT1	0.521739	0	0.130435	0	0	3.555556
2996	2-Jun-07	2007	LT1	0.521739	0	0.130435	0	0	3.555556
2997	2-Jun-07	2007	LT1	0.521739	0	0.130435	0	0	3.555556
2983	2-Jun-07	2007	LT1	0.521739	0	0.130435	0	0	3.555556
2984	2-Jun-07	2007	LT1	0.521739	0	0.130435	0	0	3.555556
3000	2-Jun-07	2007	LT1	0.521739	0	0.130435	0	0	3.555556
3001	2-Jun-07	2007	LT1	0.521739	0	0.130435	0	0	3.555556
2981	2-Jun-07	2007	LT1	0.521739	0	0.130435	0	0	3.555556
2982	2-Jun-07	2007	LT1	0.521739	0	0.130435	0	0	3.555556
3002	2-Jun-07	2007	LT1	0.521739	0	0.130435	0	0	3.555556
	8-Jun-07	2007	SD						
	8-Jun-07	2007	SD						
	8-Jun-07	2007	SD						
	8-Jun-07	2007	SD						
	8-Jun-07	2007	SD						
	8-Jun-07	2007	SD						
	8-Jun-07	2007	SD						

	8-Jun-07	2007	SD							
	8-Jun-07	2007	SD							
	8-Jun-07	2007	SD							
	8-Jun-07	2007	SD							
	8-Jun-07	2007	SD							
	8-Jun-07	2007	SD							
	8-Jun-07	2007	SD							
	8-Jun-07	2007	SD							
	8-Jun-07	2007	SD							
	8-Jun-07	2007	SD							
	8-Jun-07	2007	SD							
	8-Jun-07	2007	SD							
	8-Jun-07	2007	SD							
	8-Jun-07	2007	SD							
	8-Jun-07	2007	SD							
	8-Jun-07	2007	SD							
	8-Jun-07	2007	SD							
	8-Jun-07	2007	SD							
	8-Jun-07	2007	SD							
	8-Jun-07	2007	SD							
	8-Jun-07	2007	SD							
	8-Jun-07	2007	SD							
	8-Jun-07	2007	SD							
	8-Jun-07	2007	SD							
1387	18-May-08	2008	SD							
1385	18-May-08	2008	SD							
1383	18-May-08	2008	SD							
1384	18-May-08	2008	SD							
1382	18-May-08	2008	SD							
1388	18-May-08	2008	SD							
1389	18-May-08	2008	SD							
1380	18-May-08	2008	SD							
1391	18-May-08	2008	SD							
1386	18-May-08	2008	SD							
1395	18-May-08	2008	SD							
1393	18-May-08	2008	SD							
1381	18-May-08	2008	SD							
1390	18-May-08	2008	SD							
1392	18-May-08	2008	SD							
1396	18-May-08	2008	SD							
1394	18-May-08	2008	SD							
1378	18-May-08	2008	SD							
1379	18-May-08	2008	SD							
148	27-May-04	2004	SC		0.174312	0.009174	0.06422	0.018349	0.103343	6.846154

149	27-May-04	2004	SC	0.174312	0.009174	0.06422	0.018349	0.103343	6.846154
151	27-May-04	2004	SC	0.174312	0.009174	0.06422	0.018349	0.103343	6.846154
152	27-May-04	2004	SC	0.174312	0.009174	0.06422	0.018349	0.103343	6.846154
147	27-May-04	2004	SC	0.174312	0.009174	0.06422	0.018349	0.103343	6.846154
150	27-May-04	2004	SC	0.174312	0.009174	0.06422	0.018349	0.103343	6.846154
145	27-May-04	2004	SC	0.174312	0.009174	0.06422	0.018349	0.103343	6.846154
146	27-May-04	2004	SC	0.174312	0.009174	0.06422	0.018349	0.103343	6.846154
189	28-May-04	2004	RC	0.428571	0.107143	0	0	0.401709	3
169	28-May-04	2004	RC	0.428571	0.107143	0	0	0.401709	3
179	28-May-04	2004	RC	0.428571	0.107143	0	0	0.401709	3
184	28-May-04	2004	RC	0.428571	0.107143	0	0	0.401709	3
186	28-May-04	2004	RC	0.428571	0.107143	0	0	0.401709	3
192	28-May-04	2004	RC	0.428571	0.107143	0	0	0.401709	3
193	28-May-04	2004	RC	0.428571	0.107143	0	0	0.401709	3
182	28-May-04	2004	RC	0.428571	0.107143	0	0	0.401709	3
187	28-May-04	2004	RC	0.428571	0.107143	0	0	0.401709	3
178	28-May-04	2004	RC	0.428571	0.107143	0	0	0.401709	3
176	28-May-04	2004	RC	0.428571	0.107143	0	0	0.401709	3
177	28-May-04	2004	RC	0.428571	0.107143	0	0	0.401709	3
175	28-May-04	2004	RC	0.428571	0.107143	0	0	0.401709	3
172	28-May-04	2004	RC	0.428571	0.107143	0	0	0.401709	3
174	28-May-04	2004	RC	0.428571	0.107143	0	0	0.401709	3
180	28-May-04	2004	RC	0.428571	0.107143	0	0	0.401709	3
185	28-May-04	2004	RC	0.428571	0.107143	0	0	0.401709	3
170	28-May-04	2004	RC	0.428571	0.107143	0	0	0.401709	3
171	28-May-04	2004	RC	0.428571	0.107143	0	0	0.401709	3
173	28-May-04	2004	RC	0.428571	0.107143	0	0	0.401709	3
181	28-May-04	2004	RC	0.428571	0.107143	0	0	0.401709	3
183	28-May-04	2004	RC	0.428571	0.107143	0	0	0.401709	3
190	28-May-04	2004	RC	0.428571	0.107143	0	0	0.401709	3
191	28-May-04	2004	RC	0.428571	0.107143	0	0	0.401709	3
188	28-May-04	2004	RC	0.428571	0.107143	0	0	0.401709	3
194	28-May-04	2004	RC	0.428571	0.107143	0	0	0.401709	3
342	30-May-04	2004	SD	0.412698	0.301587	0.063492	0.634921	0.097297	2.642857
343	30-May-04	2004	SD	0.412698	0.301587	0.063492	0.634921	0.097297	2.642857
341	30-May-04	2004	SD	0.412698	0.301587	0.063492	0.634921	0.097297	2.642857
350	30-May-04	2004	SD	0.412698	0.301587	0.063492	0.634921	0.097297	2.642857
351	30-May-04	2004	SD	0.412698	0.301587	0.063492	0.634921	0.097297	2.642857
345	30-May-04	2004	SD	0.412698	0.301587	0.063492	0.634921	0.097297	2.642857
334	30-May-04	2004	SD	0.412698	0.301587	0.063492	0.634921	0.097297	2.642857
335	30-May-04	2004	SD	0.412698	0.301587	0.063492	0.634921	0.097297	2.642857
338	30-May-04	2004	SD	0.412698	0.301587	0.063492	0.634921	0.097297	2.642857
339	30-May-04	2004	SD	0.412698	0.301587	0.063492	0.634921	0.097297	2.642857
340	30-May-04	2004	SD	0.412698	0.301587	0.063492	0.634921	0.097297	2.642857

344	30-May-04	2004	SD	0.412698	0.301587	0.063492	0.634921	0.097297	2.642857
346	30-May-04	2004	SD	0.412698	0.301587	0.063492	0.634921	0.097297	2.642857
347	30-May-04	2004	SD	0.412698	0.301587	0.063492	0.634921	0.097297	2.642857
348	30-May-04	2004	SD	0.412698	0.301587	0.063492	0.634921	0.097297	2.642857
336	30-May-04	2004	SD	0.412698	0.301587	0.063492	0.634921	0.097297	2.642857
337	30-May-04	2004	SD	0.412698	0.301587	0.063492	0.634921	0.097297	2.642857
349	30-May-04	2004	SD	0.412698	0.301587	0.063492	0.634921	0.097297	2.642857
540	13-Jun-04	2004	SD	0.412698	0.301587	0.063492	0.634921	0.097297	2.642857
541	13-Jun-04	2004	SD	0.412698	0.301587	0.063492	0.634921	0.097297	2.642857
542	13-Jun-04	2004	SD	0.412698	0.301587	0.063492	0.634921	0.097297	2.642857
544	13-Jun-04	2004	SD	0.412698	0.301587	0.063492	0.634921	0.097297	2.642857
543	13-Jun-04	2004	SD	0.412698	0.301587	0.063492	0.634921	0.097297	2.642857
77	10-May-05	2005	SC	0.183486	0	0.073394	0	0.006329	6.769231
81	10-May-05	2005	SC	0.183486	0	0.073394	0	0.006329	6.769231
82	10-May-05	2005	SC	0.183486	0	0.073394	0	0.006329	6.769231
87	10-May-05	2005	SC	0.183486	0	0.073394	0	0.006329	6.769231
80	10-May-05	2005	SC	0.183486	0	0.073394	0	0.006329	6.769231
84	10-May-05	2005	SC	0.183486	0	0.073394	0	0.006329	6.769231
85	10-May-05	2005	SC	0.183486	0	0.073394	0	0.006329	6.769231
73	10-May-05	2005	SC	0.183486	0	0.073394	0	0.006329	6.769231
74	10-May-05	2005	SC	0.183486	0	0.073394	0	0.006329	6.769231
75	10-May-05	2005	SC	0.183486	0	0.073394	0	0.006329	6.769231
79	10-May-05	2005	SC	0.183486	0	0.073394	0	0.006329	6.769231
72	10-May-05	2005	SC	0.183486	0	0.073394	0	0.006329	6.769231
83	10-May-05	2005	SC	0.183486	0	0.073394	0	0.006329	6.769231
78	10-May-05	2005	SC	0.183486	0	0.073394	0	0.006329	6.769231
86	10-May-05	2005	SC	0.183486	0	0.073394	0	0.006329	6.769231
71	10-May-05	2005	SC	0.183486	0	0.073394	0	0.006329	6.769231
76	10-May-05	2005	SC	0.183486	0	0.073394	0	0.006329	6.769231
146	12-May-05	2005	DE						
148	12-May-05	2005	DE						
149	12-May-05	2005	DE						
150	12-May-05	2005	DE						
147	12-May-05	2005	DE						
144	12-May-05	2005	DE						
142	12-May-05	2005	DE						
143	12-May-05	2005	DE						
140	12-May-05	2005	DE						
156	12-May-05	2005	DE						
151	12-May-05	2005	DE						
153	12-May-05	2005	DE						
154	12-May-05	2005	DE						
155	12-May-05	2005	DE						
145	12-May-05	2005	DE						

152	12-May-05	2005	DE						
141	12-May-05	2005	DE						
182	12-May-05	2005	RP	0.333333	0	0	0.047619	0.072115	2.25
183	12-May-05	2005	RP	0.333333	0	0	0.047619	0.072115	2.25
184	12-May-05	2005	RP	0.333333	0	0	0.047619	0.072115	2.25
179	12-May-05	2005	RP	0.333333	0	0	0.047619	0.072115	2.25
180	12-May-05	2005	RP	0.333333	0	0	0.047619	0.072115	2.25
181	12-May-05	2005	RP	0.333333	0	0	0.047619	0.072115	2.25
176	12-May-05	2005	RP	0.333333	0	0	0.047619	0.072115	2.25
177	12-May-05	2005	RP	0.333333	0	0	0.047619	0.072115	2.25
178	12-May-05	2005	RP	0.333333	0	0	0.047619	0.072115	2.25
189	14-May-05	2005	RP	0.333333	0	0	0.047619	0.072115	2.25
191	14-May-05	2005	RP	0.333333	0	0	0.047619	0.072115	2.25
193	14-May-05	2005	RP	0.333333	0	0	0.047619	0.072115	2.25
190	14-May-05	2005	RP	0.333333	0	0	0.047619	0.072115	2.25
192	14-May-05	2005	RP	0.333333	0	0	0.047619	0.072115	2.25
187	14-May-05	2005	RP	0.333333	0	0	0.047619	0.072115	2.25
188	14-May-05	2005	RP	0.333333	0	0	0.047619	0.072115	2.25
185	14-May-05	2005	RP	0.333333	0	0	0.047619	0.072115	2.25
186	14-May-05	2005	RP	0.333333	0	0	0.047619	0.072115	2.25
202	14-May-05	2005	SW	0.264151	0.113208	0.018868	0.169811	0.044444	1.4
203	14-May-05	2005	SW	0.264151	0.113208	0.018868	0.169811	0.044444	1.4
200	14-May-05	2005	SW	0.264151	0.113208	0.018868	0.169811	0.044444	1.4
201	14-May-05	2005	SW	0.264151	0.113208	0.018868	0.169811	0.044444	1.4
204	14-May-05	2005	SW	0.264151	0.113208	0.018868	0.169811	0.044444	1.4
194	14-May-05	2005	SW	0.264151	0.113208	0.018868	0.169811	0.044444	1.4
198	14-May-05	2005	SW	0.264151	0.113208	0.018868	0.169811	0.044444	1.4
199	14-May-05	2005	SW	0.264151	0.113208	0.018868	0.169811	0.044444	1.4
206	14-May-05	2005	SW	0.264151	0.113208	0.018868	0.169811	0.044444	1.4
209	14-May-05	2005	SW	0.264151	0.113208	0.018868	0.169811	0.044444	1.4
210	14-May-05	2005	SW	0.264151	0.113208	0.018868	0.169811	0.044444	1.4
195	14-May-05	2005	SW	0.264151	0.113208	0.018868	0.169811	0.044444	1.4
211	14-May-05	2005	SW	0.264151	0.113208	0.018868	0.169811	0.044444	1.4
196	14-May-05	2005	SW	0.264151	0.113208	0.018868	0.169811	0.044444	1.4
207	14-May-05	2005	SW	0.264151	0.113208	0.018868	0.169811	0.044444	1.4
197	14-May-05	2005	SW	0.264151	0.113208	0.018868	0.169811	0.044444	1.4
208	14-May-05	2005	SW	0.264151	0.113208	0.018868	0.169811	0.044444	1.4
205	14-May-05	2005	SW	0.264151	0.113208	0.018868	0.169811	0.044444	1.4
264	15-May-05	2005	TO	0.285714	0.142857	0.017857	0.142857	0.014577	1
255	15-May-05	2005	TO	0.285714	0.142857	0.017857	0.142857	0.014577	1
246	15-May-05	2005	TO	0.285714	0.142857	0.017857	0.142857	0.014577	1
254	15-May-05	2005	TO	0.285714	0.142857	0.017857	0.142857	0.014577	1
250	15-May-05	2005	TO	0.285714	0.142857	0.017857	0.142857	0.014577	1
248	15-May-05	2005	TO	0.285714	0.142857	0.017857	0.142857	0.014577	1

259	15-May-05	2005 TO	0.285714	0.142857	0.017857	0.142857	0.014577	1
258	15-May-05	2005 TO	0.285714	0.142857	0.017857	0.142857	0.014577	1
252	15-May-05	2005 TO	0.285714	0.142857	0.017857	0.142857	0.014577	1
253	15-May-05	2005 TO	0.285714	0.142857	0.017857	0.142857	0.014577	1
257	15-May-05	2005 TO	0.285714	0.142857	0.017857	0.142857	0.014577	1
260	15-May-05	2005 TO	0.285714	0.142857	0.017857	0.142857	0.014577	1
261	15-May-05	2005 TO	0.285714	0.142857	0.017857	0.142857	0.014577	1
262	15-May-05	2005 TO	0.285714	0.142857	0.017857	0.142857	0.014577	1
247	15-May-05	2005 TO	0.285714	0.142857	0.017857	0.142857	0.014577	1
249	15-May-05	2005 TO	0.285714	0.142857	0.017857	0.142857	0.014577	1
256	15-May-05	2005 TO	0.285714	0.142857	0.017857	0.142857	0.014577	1
251	15-May-05	2005 TO	0.285714	0.142857	0.017857	0.142857	0.014577	1
265	15-May-05	2005 TO	0.285714	0.142857	0.017857	0.142857	0.014577	1
263	15-May-05	2005 TO	0.285714	0.142857	0.017857	0.142857	0.014577	1
352	17-May-05	2005 RC	0.185185	0	0	0.037037	0.155172	2.875
351	17-May-05	2005 RC	0.185185	0	0	0.037037	0.155172	2.875
347	17-May-05	2005 RC	0.185185	0	0	0.037037	0.155172	2.875
348	17-May-05	2005 RC	0.185185	0	0	0.037037	0.155172	2.875
349	17-May-05	2005 RC	0.185185	0	0	0.037037	0.155172	2.875
350	17-May-05	2005 RC	0.185185	0	0	0.037037	0.155172	2.875
353	17-May-05	2005 RC	0.185185	0	0	0.037037	0.155172	2.875
354	17-May-05	2005 RC	0.185185	0	0	0.037037	0.155172	2.875
359	17-May-05	2005 RC	0.185185	0	0	0.037037	0.155172	2.875
360	17-May-05	2005 RC	0.185185	0	0	0.037037	0.155172	2.875
361	17-May-05	2005 RC	0.185185	0	0	0.037037	0.155172	2.875
362	17-May-05	2005 RC	0.185185	0	0	0.037037	0.155172	2.875
363	17-May-05	2005 RC	0.185185	0	0	0.037037	0.155172	2.875
365	17-May-05	2005 RC	0.185185	0	0	0.037037	0.155172	2.875
366	17-May-05	2005 RC	0.185185	0	0	0.037037	0.155172	2.875
355	17-May-05	2005 RC	0.185185	0	0	0.037037	0.155172	2.875
364	17-May-05	2005 RC	0.185185	0	0	0.037037	0.155172	2.875
356	17-May-05	2005 RC	0.185185	0	0	0.037037	0.155172	2.875
357	17-May-05	2005 RC	0.185185	0	0	0.037037	0.155172	2.875
358	17-May-05	2005 RC	0.185185	0	0	0.037037	0.155172	2.875
312	17-May-05	2005 RC	0.185185	0	0	0.037037	0.155172	2.875
317	17-May-05	2005 RC	0.185185	0	0	0.037037	0.155172	2.875
316	17-May-05	2005 RC	0.185185	0	0	0.037037	0.155172	2.875
314	17-May-05	2005 RC	0.185185	0	0	0.037037	0.155172	2.875
313	17-May-05	2005 RC	0.185185	0	0	0.037037	0.155172	2.875
305	17-May-05	2005 RC	0.185185	0	0	0.037037	0.155172	2.875
308	17-May-05	2005 RC	0.185185	0	0	0.037037	0.155172	2.875
309	17-May-05	2005 RC	0.185185	0	0	0.037037	0.155172	2.875
310	17-May-05	2005 RC	0.185185	0	0	0.037037	0.155172	2.875
315	17-May-05	2005 RC	0.185185	0	0	0.037037	0.155172	2.875

307	17-May-05	2005	RC	0.185185	0	0	0.037037	0.155172	2.875
319	17-May-05	2005	RC	0.185185	0	0	0.037037	0.155172	2.875
306	17-May-05	2005	RC	0.185185	0	0	0.037037	0.155172	2.875
311	17-May-05	2005	RC	0.185185	0	0	0.037037	0.155172	2.875
318	17-May-05	2005	RC	0.185185	0	0	0.037037	0.155172	2.875
386	17-May-05	2005	SD	0.365079	0.095238	0.095238	0.380952	0.048387	2.5
389	17-May-05	2005	SD	0.365079	0.095238	0.095238	0.380952	0.048387	2.5
388	17-May-05	2005	SD	0.365079	0.095238	0.095238	0.380952	0.048387	2.5
387	17-May-05	2005	SD	0.365079	0.095238	0.095238	0.380952	0.048387	2.5
390	17-May-05	2005	SD	0.365079	0.095238	0.095238	0.380952	0.048387	2.5
385	17-May-05	2005	SD	0.365079	0.095238	0.095238	0.380952	0.048387	2.5
391	17-May-05	2005	SD	0.365079	0.095238	0.095238	0.380952	0.048387	2.5
392	17-May-05	2005	SD	0.365079	0.095238	0.095238	0.380952	0.048387	2.5
395	17-May-05	2005	SD	0.365079	0.095238	0.095238	0.380952	0.048387	2.5
398	17-May-05	2005	SD	0.365079	0.095238	0.095238	0.380952	0.048387	2.5
399	17-May-05	2005	SD	0.365079	0.095238	0.095238	0.380952	0.048387	2.5
400	17-May-05	2005	SD	0.365079	0.095238	0.095238	0.380952	0.048387	2.5
397	17-May-05	2005	SD	0.365079	0.095238	0.095238	0.380952	0.048387	2.5
401	17-May-05	2005	SD	0.365079	0.095238	0.095238	0.380952	0.048387	2.5
402	17-May-05	2005	SD	0.365079	0.095238	0.095238	0.380952	0.048387	2.5
396	17-May-05	2005	SD	0.365079	0.095238	0.095238	0.380952	0.048387	2.5
394	17-May-05	2005	SD	0.365079	0.095238	0.095238	0.380952	0.048387	2.5
393	17-May-05	2005	SD	0.365079	0.095238	0.095238	0.380952	0.048387	2.5
416	18-May-05	2005	SC	0.183486	0	0.073394	0	0.006329	6.769231
417	18-May-05	2005	SC	0.183486	0	0.073394	0	0.006329	6.769231
410	18-May-05	2005	SC	0.183486	0	0.073394	0	0.006329	6.769231
419	18-May-05	2005	SC	0.183486	0	0.073394	0	0.006329	6.769231
420	18-May-05	2005	SC	0.183486	0	0.073394	0	0.006329	6.769231
411	18-May-05	2005	SC	0.183486	0	0.073394	0	0.006329	6.769231
412	18-May-05	2005	SC	0.183486	0	0.073394	0	0.006329	6.769231
421	18-May-05	2005	SC	0.183486	0	0.073394	0	0.006329	6.769231
418	18-May-05	2005	SC	0.183486	0	0.073394	0	0.006329	6.769231
414	18-May-05	2005	SC	0.183486	0	0.073394	0	0.006329	6.769231
415	18-May-05	2005	SC	0.183486	0	0.073394	0	0.006329	6.769231
413	18-May-05	2005	SC	0.183486	0	0.073394	0	0.006329	6.769231
422	18-May-05	2005	SC	0.183486	0	0.073394	0	0.006329	6.769231
446	19-May-05	2005	ML						
435	19-May-05	2005	ML						
436	19-May-05	2005	ML						
437	19-May-05	2005	ML						
439	19-May-05	2005	ML						
440	19-May-05	2005	ML						
441	19-May-05	2005	ML						
442	19-May-05	2005	ML						

430	19-May-05	2005	ML						
438	19-May-05	2005	ML						
445	19-May-05	2005	ML						
434	19-May-05	2005	ML						
443	19-May-05	2005	ML						
444	19-May-05	2005	ML						
431	19-May-05	2005	ML						
432	19-May-05	2005	ML						
433	19-May-05	2005	ML						
494	20-May-05	2005	SHAC	0.076923	0.153846	0	0.384615	0.026042	2.090909
487	20-May-05	2005	SHAC	0.076923	0.153846	0	0.384615	0.026042	2.090909
493	20-May-05	2005	SHAC	0.076923	0.153846	0	0.384615	0.026042	2.090909
486	20-May-05	2005	SHAC	0.076923	0.153846	0	0.384615	0.026042	2.090909
484	20-May-05	2005	SHAC	0.076923	0.153846	0	0.384615	0.026042	2.090909
489	20-May-05	2005	SHAC	0.076923	0.153846	0	0.384615	0.026042	2.090909
491	20-May-05	2005	SHAC	0.076923	0.153846	0	0.384615	0.026042	2.090909
499	20-May-05	2005	SHAC	0.076923	0.153846	0	0.384615	0.026042	2.090909
492	20-May-05	2005	SHAC	0.076923	0.153846	0	0.384615	0.026042	2.090909
482	20-May-05	2005	SHAC	0.076923	0.153846	0	0.384615	0.026042	2.090909
488	20-May-05	2005	SHAC	0.076923	0.153846	0	0.384615	0.026042	2.090909
490	20-May-05	2005	SHAC	0.076923	0.153846	0	0.384615	0.026042	2.090909
500	20-May-05	2005	SHAC	0.076923	0.153846	0	0.384615	0.026042	2.090909
483	20-May-05	2005	SHAC	0.076923	0.153846	0	0.384615	0.026042	2.090909
485	20-May-05	2005	SHAC	0.076923	0.153846	0	0.384615	0.026042	2.090909
498	20-May-05	2005	SHAC	0.076923	0.153846	0	0.384615	0.026042	2.090909
501	20-May-05	2005	SHAC	0.076923	0.153846	0	0.384615	0.026042	2.090909
497	20-May-05	2005	SHAC	0.076923	0.153846	0	0.384615	0.026042	2.090909
495	20-May-05	2005	SHAC	0.076923	0.153846	0	0.384615	0.026042	2.090909
496	20-May-05	2005	SHAC	0.076923	0.153846	0	0.384615	0.026042	2.090909
536	20-May-05	2005	SHCC	0.319149	0.106383	0	0.191489	0.034483	0.230769
537	20-May-05	2005	SHCC	0.319149	0.106383	0	0.191489	0.034483	0.230769
542	20-May-05	2005	SHCC	0.319149	0.106383	0	0.191489	0.034483	0.230769
543	20-May-05	2005	SHCC	0.319149	0.106383	0	0.191489	0.034483	0.230769
533	20-May-05	2005	SHCC	0.319149	0.106383	0	0.191489	0.034483	0.230769
539	20-May-05	2005	SHCC	0.319149	0.106383	0	0.191489	0.034483	0.230769
532	20-May-05	2005	SHCC	0.319149	0.106383	0	0.191489	0.034483	0.230769
534	20-May-05	2005	SHCC	0.319149	0.106383	0	0.191489	0.034483	0.230769
535	20-May-05	2005	SHCC	0.319149	0.106383	0	0.191489	0.034483	0.230769
538	20-May-05	2005	SHCC	0.319149	0.106383	0	0.191489	0.034483	0.230769
540	20-May-05	2005	SHCC	0.319149	0.106383	0	0.191489	0.034483	0.230769
541	20-May-05	2005	SHCC	0.319149	0.106383	0	0.191489	0.034483	0.230769
599	21-May-05	2005	SC	0.183486	0	0.073394	0	0.006329	6.769231
598	21-May-05	2005	SC	0.183486	0	0.073394	0	0.006329	6.769231
627	21-May-05	2005	SC	0.183486	0	0.073394	0	0.006329	6.769231

631	21-May-05	2005 SC	0.183486	0	0.073394	0	0.006329	6.769231
633	21-May-05	2005 SC	0.183486	0	0.073394	0	0.006329	6.769231
630	21-May-05	2005 SC	0.183486	0	0.073394	0	0.006329	6.769231
632	21-May-05	2005 SC	0.183486	0	0.073394	0	0.006329	6.769231
635	21-May-05	2005 SC	0.183486	0	0.073394	0	0.006329	6.769231
628	21-May-05	2005 SC	0.183486	0	0.073394	0	0.006329	6.769231
629	21-May-05	2005 SC	0.183486	0	0.073394	0	0.006329	6.769231
634	21-May-05	2005 SC	0.183486	0	0.073394	0	0.006329	6.769231
626	21-May-05	2005 SC	0.183486	0	0.073394	0	0.006329	6.769231
674	22-May-05	2005 SW	0.264151	0.113208	0.018868	0.169811	0.044444	1.4
675	22-May-05	2005 SW	0.264151	0.113208	0.018868	0.169811	0.044444	1.4
676	22-May-05	2005 SW	0.264151	0.113208	0.018868	0.169811	0.044444	1.4
677	22-May-05	2005 SW	0.264151	0.113208	0.018868	0.169811	0.044444	1.4
678	22-May-05	2005 SW	0.264151	0.113208	0.018868	0.169811	0.044444	1.4
688	22-May-05	2005 TO	0.285714	0.142857	0.017857	0.142857	0.014577	1
687	22-May-05	2005 TO	0.285714	0.142857	0.017857	0.142857	0.014577	1
683	22-May-05	2005 TO	0.285714	0.142857	0.017857	0.142857	0.014577	1
685	22-May-05	2005 TO	0.285714	0.142857	0.017857	0.142857	0.014577	1
686	22-May-05	2005 TO	0.285714	0.142857	0.017857	0.142857	0.014577	1
689	22-May-05	2005 TO	0.285714	0.142857	0.017857	0.142857	0.014577	1
684	22-May-05	2005 TO	0.285714	0.142857	0.017857	0.142857	0.014577	1
679	22-May-05	2005 TO	0.285714	0.142857	0.017857	0.142857	0.014577	1
680	22-May-05	2005 TO	0.285714	0.142857	0.017857	0.142857	0.014577	1
681	22-May-05	2005 TO	0.285714	0.142857	0.017857	0.142857	0.014577	1
682	22-May-05	2005 TO	0.285714	0.142857	0.017857	0.142857	0.014577	1
715	25-May-05	2005 SD	0.365079	0.095238	0.095238	0.380952	0.048387	2.5
716	25-May-05	2005 SD	0.365079	0.095238	0.095238	0.380952	0.048387	2.5
717	25-May-05	2005 SD	0.365079	0.095238	0.095238	0.380952	0.048387	2.5
711	25-May-05	2005 SD	0.365079	0.095238	0.095238	0.380952	0.048387	2.5
713	25-May-05	2005 SD	0.365079	0.095238	0.095238	0.380952	0.048387	2.5
714	25-May-05	2005 SD	0.365079	0.095238	0.095238	0.380952	0.048387	2.5
710	25-May-05	2005 SD	0.365079	0.095238	0.095238	0.380952	0.048387	2.5
712	25-May-05	2005 SD	0.365079	0.095238	0.095238	0.380952	0.048387	2.5
718	25-May-05	2005 SD	0.365079	0.095238	0.095238	0.380952	0.048387	2.5
720	25-May-05	2005 SD	0.365079	0.095238	0.095238	0.380952	0.048387	2.5
719	25-May-05	2005 SD	0.365079	0.095238	0.095238	0.380952	0.048387	2.5
804	25-May-05	2005 SD	0.365079	0.095238	0.095238	0.380952	0.048387	2.5
802	25-May-05	2005 SD	0.365079	0.095238	0.095238	0.380952	0.048387	2.5
803	25-May-05	2005 SD	0.365079	0.095238	0.095238	0.380952	0.048387	2.5
805	25-May-05	2005 SD	0.365079	0.095238	0.095238	0.380952	0.048387	2.5
806	25-May-05	2005 SD	0.365079	0.095238	0.095238	0.380952	0.048387	2.5
795	25-May-05	2005 SD	0.365079	0.095238	0.095238	0.380952	0.048387	2.5
797	25-May-05	2005 SD	0.365079	0.095238	0.095238	0.380952	0.048387	2.5
807	25-May-05	2005 SD	0.365079	0.095238	0.095238	0.380952	0.048387	2.5

794	25-May-05	2005 SD	0.365079	0.095238	0.095238	0.380952	0.048387	2.5
791	25-May-05	2005 SD	0.365079	0.095238	0.095238	0.380952	0.048387	2.5
792	25-May-05	2005 SD	0.365079	0.095238	0.095238	0.380952	0.048387	2.5
793	25-May-05	2005 SD	0.365079	0.095238	0.095238	0.380952	0.048387	2.5
796	25-May-05	2005 SD	0.365079	0.095238	0.095238	0.380952	0.048387	2.5
790	25-May-05	2005 SD	0.365079	0.095238	0.095238	0.380952	0.048387	2.5
800	25-May-05	2005 SD	0.365079	0.095238	0.095238	0.380952	0.048387	2.5
798	25-May-05	2005 SD	0.365079	0.095238	0.095238	0.380952	0.048387	2.5
799	25-May-05	2005 SD	0.365079	0.095238	0.095238	0.380952	0.048387	2.5
801	25-May-05	2005 SD	0.365079	0.095238	0.095238	0.380952	0.048387	2.5
808	26-May-05	2005 SC	0.183486	0	0.073394	0	0.006329	6.769231
811	26-May-05	2005 SC	0.183486	0	0.073394	0	0.006329	6.769231
812	26-May-05	2005 SC	0.183486	0	0.073394	0	0.006329	6.769231
813	26-May-05	2005 SC	0.183486	0	0.073394	0	0.006329	6.769231
815	26-May-05	2005 SC	0.183486	0	0.073394	0	0.006329	6.769231
809	26-May-05	2005 SC	0.183486	0	0.073394	0	0.006329	6.769231
810	26-May-05	2005 SC	0.183486	0	0.073394	0	0.006329	6.769231
814	26-May-05	2005 SC	0.183486	0	0.073394	0	0.006329	6.769231
816	26-May-05	2005 SC	0.183486	0	0.073394	0	0.006329	6.769231
817	26-May-05	2005 SC	0.183486	0	0.073394	0	0.006329	6.769231
819	26-May-05	2005 SC	0.183486	0	0.073394	0	0.006329	6.769231
820	26-May-05	2005 SC	0.183486	0	0.073394	0	0.006329	6.769231
821	26-May-05	2005 SC	0.183486	0	0.073394	0	0.006329	6.769231
818	26-May-05	2005 SC	0.183486	0	0.073394	0	0.006329	6.769231
823	26-May-05	2005 SC	0.183486	0	0.073394	0	0.006329	6.769231
824	26-May-05	2005 SC	0.183486	0	0.073394	0	0.006329	6.769231
825	26-May-05	2005 SC	0.183486	0	0.073394	0	0.006329	6.769231
828	26-May-05	2005 SC	0.183486	0	0.073394	0	0.006329	6.769231
829	26-May-05	2005 SC	0.183486	0	0.073394	0	0.006329	6.769231
830	26-May-05	2005 SC	0.183486	0	0.073394	0	0.006329	6.769231
831	26-May-05	2005 SC	0.183486	0	0.073394	0	0.006329	6.769231
832	26-May-05	2005 SC	0.183486	0	0.073394	0	0.006329	6.769231
834	26-May-05	2005 SC	0.183486	0	0.073394	0	0.006329	6.769231
835	26-May-05	2005 SC	0.183486	0	0.073394	0	0.006329	6.769231
822	26-May-05	2005 SC	0.183486	0	0.073394	0	0.006329	6.769231
826	26-May-05	2005 SC	0.183486	0	0.073394	0	0.006329	6.769231
833	26-May-05	2005 SC	0.183486	0	0.073394	0	0.006329	6.769231
827	26-May-05	2005 SC	0.183486	0	0.073394	0	0.006329	6.769231
842	26-May-05	2005 SD	0.365079	0.095238	0.095238	0.380952	0.048387	2.5
849	26-May-05	2005 SD	0.365079	0.095238	0.095238	0.380952	0.048387	2.5
850	26-May-05	2005 SD	0.365079	0.095238	0.095238	0.380952	0.048387	2.5
851	26-May-05	2005 SD	0.365079	0.095238	0.095238	0.380952	0.048387	2.5
837	26-May-05	2005 SD	0.365079	0.095238	0.095238	0.380952	0.048387	2.5
839	26-May-05	2005 SD	0.365079	0.095238	0.095238	0.380952	0.048387	2.5

840	26-May-05	2005 SD	0.365079	0.095238	0.095238	0.380952	0.048387	2.5
841	26-May-05	2005 SD	0.365079	0.095238	0.095238	0.380952	0.048387	2.5
844	26-May-05	2005 SD	0.365079	0.095238	0.095238	0.380952	0.048387	2.5
845	26-May-05	2005 SD	0.365079	0.095238	0.095238	0.380952	0.048387	2.5
846	26-May-05	2005 SD	0.365079	0.095238	0.095238	0.380952	0.048387	2.5
847	26-May-05	2005 SD	0.365079	0.095238	0.095238	0.380952	0.048387	2.5
848	26-May-05	2005 SD	0.365079	0.095238	0.095238	0.380952	0.048387	2.5
852	26-May-05	2005 SD	0.365079	0.095238	0.095238	0.380952	0.048387	2.5
843	26-May-05	2005 SD	0.365079	0.095238	0.095238	0.380952	0.048387	2.5
838	26-May-05	2005 SD	0.365079	0.095238	0.095238	0.380952	0.048387	2.5
836	26-May-05	2005 SD	0.365079	0.095238	0.095238	0.380952	0.048387	2.5
1220	26-May-05	2005 SD	0.365079	0.095238	0.095238	0.380952	0.048387	2.5
1221	26-May-05	2005 SD	0.365079	0.095238	0.095238	0.380952	0.048387	2.5
1216	26-May-05	2005 SD	0.365079	0.095238	0.095238	0.380952	0.048387	2.5
1218	26-May-05	2005 SD	0.365079	0.095238	0.095238	0.380952	0.048387	2.5
1219	26-May-05	2005 SD	0.365079	0.095238	0.095238	0.380952	0.048387	2.5
1217	26-May-05	2005 SD	0.365079	0.095238	0.095238	0.380952	0.048387	2.5
1235	28-May-05	2005 YY	0.114286	0.071429	0	0.342857	0	0.65
1222	28-May-05	2005 YY	0.114286	0.071429	0	0.342857	0	0.65
1224	28-May-05	2005 YY	0.114286	0.071429	0	0.342857	0	0.65
1225	28-May-05	2005 YY	0.114286	0.071429	0	0.342857	0	0.65
1226	28-May-05	2005 YY	0.114286	0.071429	0	0.342857	0	0.65
1227	28-May-05	2005 YY	0.114286	0.071429	0	0.342857	0	0.65
1233	28-May-05	2005 YY	0.114286	0.071429	0	0.342857	0	0.65
1228	28-May-05	2005 YY	0.114286	0.071429	0	0.342857	0	0.65
1229	28-May-05	2005 YY	0.114286	0.071429	0	0.342857	0	0.65
1230	28-May-05	2005 YY	0.114286	0.071429	0	0.342857	0	0.65
1232	28-May-05	2005 YY	0.114286	0.071429	0	0.342857	0	0.65
1234	28-May-05	2005 YY	0.114286	0.071429	0	0.342857	0	0.65
1237	28-May-05	2005 YY	0.114286	0.071429	0	0.342857	0	0.65
1223	28-May-05	2005 YY	0.114286	0.071429	0	0.342857	0	0.65
1236	28-May-05	2005 YY	0.114286	0.071429	0	0.342857	0	0.65
1231	28-May-05	2005 YY	0.114286	0.071429	0	0.342857	0	0.65
1263	28-May-05	2005 MG	0.142857	0.128571	0	0.071429	0.043738	5.65
1264	28-May-05	2005 MG	0.142857	0.128571	0	0.071429	0.043738	5.65
1262	28-May-05	2005 MG	0.142857	0.128571	0	0.071429	0.043738	5.65
1261	28-May-05	2005 MG	0.142857	0.128571	0	0.071429	0.043738	5.65
1270	28-May-05	2005 MG	0.142857	0.128571	0	0.071429	0.043738	5.65
1258	28-May-05	2005 MG	0.142857	0.128571	0	0.071429	0.043738	5.65
1259	28-May-05	2005 MG	0.142857	0.128571	0	0.071429	0.043738	5.65
1267	28-May-05	2005 MG	0.142857	0.128571	0	0.071429	0.043738	5.65
1268	28-May-05	2005 MG	0.142857	0.128571	0	0.071429	0.043738	5.65
1271	28-May-05	2005 MG	0.142857	0.128571	0	0.071429	0.043738	5.65
1272	28-May-05	2005 MG	0.142857	0.128571	0	0.071429	0.043738	5.65

1275	28-May-05	2005	MG	0.142857	0.128571	0	0.071429	0.043738	5.65
1276	28-May-05	2005	MG	0.142857	0.128571	0	0.071429	0.043738	5.65
1277	28-May-05	2005	MG	0.142857	0.128571	0	0.071429	0.043738	5.65
1266	28-May-05	2005	MG	0.142857	0.128571	0	0.071429	0.043738	5.65
1269	28-May-05	2005	MG	0.142857	0.128571	0	0.071429	0.043738	5.65
1265	28-May-05	2005	MG	0.142857	0.128571	0	0.071429	0.043738	5.65
1260	28-May-05	2005	MG	0.142857	0.128571	0	0.071429	0.043738	5.65
1273	28-May-05	2005	MG	0.142857	0.128571	0	0.071429	0.043738	5.65
1274	28-May-05	2005	MG	0.142857	0.128571	0	0.071429	0.043738	5.65
877	28-May-05	2005	MG	0.142857	0.128571	0	0.071429	0.043738	5.65
874	28-May-05	2005	MG	0.142857	0.128571	0	0.071429	0.043738	5.65
873	28-May-05	2005	MG	0.142857	0.128571	0	0.071429	0.043738	5.65
875	28-May-05	2005	MG	0.142857	0.128571	0	0.071429	0.043738	5.65
876	28-May-05	2005	MG	0.142857	0.128571	0	0.071429	0.043738	5.65
878	28-May-05	2005	MG	0.142857	0.128571	0	0.071429	0.043738	5.65
882	28-May-05	2005	MG	0.142857	0.128571	0	0.071429	0.043738	5.65
885	28-May-05	2005	MG	0.142857	0.128571	0	0.071429	0.043738	5.65
886	28-May-05	2005	MG	0.142857	0.128571	0	0.071429	0.043738	5.65
887	28-May-05	2005	MG	0.142857	0.128571	0	0.071429	0.043738	5.65
880	28-May-05	2005	MG	0.142857	0.128571	0	0.071429	0.043738	5.65
881	28-May-05	2005	MG	0.142857	0.128571	0	0.071429	0.043738	5.65
879	28-May-05	2005	MG	0.142857	0.128571	0	0.071429	0.043738	5.65
883	28-May-05	2005	MG	0.142857	0.128571	0	0.071429	0.043738	5.65
884	28-May-05	2005	MG	0.142857	0.128571	0	0.071429	0.043738	5.65
912	29-May-05	2005	RP	0.333333	0	0	0.047619	0.072115	2.25
925	29-May-05	2005	RP	0.333333	0	0	0.047619	0.072115	2.25
926	29-May-05	2005	RP	0.333333	0	0	0.047619	0.072115	2.25
928	29-May-05	2005	RP	0.333333	0	0	0.047619	0.072115	2.25
913	29-May-05	2005	RP	0.333333	0	0	0.047619	0.072115	2.25
919	29-May-05	2005	RP	0.333333	0	0	0.047619	0.072115	2.25
924	29-May-05	2005	RP	0.333333	0	0	0.047619	0.072115	2.25
927	29-May-05	2005	RP	0.333333	0	0	0.047619	0.072115	2.25
914	29-May-05	2005	RP	0.333333	0	0	0.047619	0.072115	2.25
918	29-May-05	2005	RP	0.333333	0	0	0.047619	0.072115	2.25
921	29-May-05	2005	RP	0.333333	0	0	0.047619	0.072115	2.25
920	29-May-05	2005	RP	0.333333	0	0	0.047619	0.072115	2.25
922	29-May-05	2005	RP	0.333333	0	0	0.047619	0.072115	2.25
923	29-May-05	2005	RP	0.333333	0	0	0.047619	0.072115	2.25
916	29-May-05	2005	RP	0.333333	0	0	0.047619	0.072115	2.25
917	29-May-05	2005	RP	0.333333	0	0	0.047619	0.072115	2.25
915	29-May-05	2005	RP	0.333333	0	0	0.047619	0.072115	2.25
929	29-May-05	2005	RP	0.333333	0	0	0.047619	0.072115	2.25
940	30-May-05	2005	LT1	0.393939	0.030303	0	0.242424	0	4.444444
941	30-May-05	2005	LT1	0.393939	0.030303	0	0.242424	0	4.444444

942	30-May-05	2005	LT1	0.393939	0.030303	0	0.242424	0	4.444444
944	30-May-05	2005	LT1	0.393939	0.030303	0	0.242424	0	4.444444
945	30-May-05	2005	LT1	0.393939	0.030303	0	0.242424	0	4.444444
955	30-May-05	2005	LT1	0.393939	0.030303	0	0.242424	0	4.444444
956	30-May-05	2005	LT1	0.393939	0.030303	0	0.242424	0	4.444444
952	30-May-05	2005	LT1	0.393939	0.030303	0	0.242424	0	4.444444
954	30-May-05	2005	LT1	0.393939	0.030303	0	0.242424	0	4.444444
948	30-May-05	2005	LT1	0.393939	0.030303	0	0.242424	0	4.444444
939	30-May-05	2005	LT1	0.393939	0.030303	0	0.242424	0	4.444444
946	30-May-05	2005	LT1	0.393939	0.030303	0	0.242424	0	4.444444
957	30-May-05	2005	LT1	0.393939	0.030303	0	0.242424	0	4.444444
938	30-May-05	2005	LT1	0.393939	0.030303	0	0.242424	0	4.444444
943	30-May-05	2005	LT1	0.393939	0.030303	0	0.242424	0	4.444444
951	30-May-05	2005	LT1	0.393939	0.030303	0	0.242424	0	4.444444
950	30-May-05	2005	LT1	0.393939	0.030303	0	0.242424	0	4.444444
947	30-May-05	2005	LT1	0.393939	0.030303	0	0.242424	0	4.444444
949	30-May-05	2005	LT1	0.393939	0.030303	0	0.242424	0	4.444444
953	30-May-05	2005	LT1	0.393939	0.030303	0	0.242424	0	4.444444
1290	30-May-05	2005	LT1	0.393939	0.030303	0	0.242424	0	4.444444
1289	30-May-05	2005	LT1	0.393939	0.030303	0	0.242424	0	4.444444
1288	30-May-05	2005	LT1	0.393939	0.030303	0	0.242424	0	4.444444
1287	30-May-05	2005	LT1	0.393939	0.030303	0	0.242424	0	4.444444
1312	4-Jun-05	2005	SC	0.183486	0	0.073394	0	0.006329	6.769231
1323	4-Jun-05	2005	SC	0.183486	0	0.073394	0	0.006329	6.769231
1305	4-Jun-05	2005	SC	0.183486	0	0.073394	0	0.006329	6.769231
1306	4-Jun-05	2005	SC	0.183486	0	0.073394	0	0.006329	6.769231
1309	4-Jun-05	2005	SC	0.183486	0	0.073394	0	0.006329	6.769231
1313	4-Jun-05	2005	SC	0.183486	0	0.073394	0	0.006329	6.769231
1315	4-Jun-05	2005	SC	0.183486	0	0.073394	0	0.006329	6.769231
1321	4-Jun-05	2005	SC	0.183486	0	0.073394	0	0.006329	6.769231
1322	4-Jun-05	2005	SC	0.183486	0	0.073394	0	0.006329	6.769231
1304	4-Jun-05	2005	SC	0.183486	0	0.073394	0	0.006329	6.769231
1310	4-Jun-05	2005	SC	0.183486	0	0.073394	0	0.006329	6.769231
1311	4-Jun-05	2005	SC	0.183486	0	0.073394	0	0.006329	6.769231
1314	4-Jun-05	2005	SC	0.183486	0	0.073394	0	0.006329	6.769231
1318	4-Jun-05	2005	SC	0.183486	0	0.073394	0	0.006329	6.769231
1319	4-Jun-05	2005	SC	0.183486	0	0.073394	0	0.006329	6.769231
1320	4-Jun-05	2005	SC	0.183486	0	0.073394	0	0.006329	6.769231
1308	4-Jun-05	2005	SC	0.183486	0	0.073394	0	0.006329	6.769231
1317	4-Jun-05	2005	SC	0.183486	0	0.073394	0	0.006329	6.769231
1316	4-Jun-05	2005	SC	0.183486	0	0.073394	0	0.006329	6.769231
1307	4-Jun-05	2005	SC	0.183486	0	0.073394	0	0.006329	6.769231
1303	4-Jun-05	2005	SC	0.183486	0	0.073394	0	0.006329	6.769231
1025	5-Jun-05	2005	SHAC	0.076923	0.153846	0	0.384615	0.026042	2.090909

1035	5-Jun-05	2005	SHAC	0.076923	0.153846	0	0.384615	0.026042	2.090909
1027	5-Jun-05	2005	SHAC	0.076923	0.153846	0	0.384615	0.026042	2.090909
1026	5-Jun-05	2005	SHAC	0.076923	0.153846	0	0.384615	0.026042	2.090909
1034	5-Jun-05	2005	SHAC	0.076923	0.153846	0	0.384615	0.026042	2.090909
1036	5-Jun-05	2005	SHAC	0.076923	0.153846	0	0.384615	0.026042	2.090909
1022	5-Jun-05	2005	SHAC	0.076923	0.153846	0	0.384615	0.026042	2.090909
1037	5-Jun-05	2005	SHAC	0.076923	0.153846	0	0.384615	0.026042	2.090909
1023	5-Jun-05	2005	SHAC	0.076923	0.153846	0	0.384615	0.026042	2.090909
1029	5-Jun-05	2005	SHAC	0.076923	0.153846	0	0.384615	0.026042	2.090909
1030	5-Jun-05	2005	SHAC	0.076923	0.153846	0	0.384615	0.026042	2.090909
1031	5-Jun-05	2005	SHAC	0.076923	0.153846	0	0.384615	0.026042	2.090909
1033	5-Jun-05	2005	SHAC	0.076923	0.153846	0	0.384615	0.026042	2.090909
1028	5-Jun-05	2005	SHAC	0.076923	0.153846	0	0.384615	0.026042	2.090909
1032	5-Jun-05	2005	SHAC	0.076923	0.153846	0	0.384615	0.026042	2.090909
1024	5-Jun-05	2005	SHAC	0.076923	0.153846	0	0.384615	0.026042	2.090909
1102	9-Jun-05	2005	SC	0.183486	0	0.073394	0	0.006329	6.769231
1108	9-Jun-05	2005	SC	0.183486	0	0.073394	0	0.006329	6.769231
1113	9-Jun-05	2005	SC	0.183486	0	0.073394	0	0.006329	6.769231
1098	9-Jun-05	2005	SC	0.183486	0	0.073394	0	0.006329	6.769231
1095	9-Jun-05	2005	SC	0.183486	0	0.073394	0	0.006329	6.769231
1096	9-Jun-05	2005	SC	0.183486	0	0.073394	0	0.006329	6.769231
1097	9-Jun-05	2005	SC	0.183486	0	0.073394	0	0.006329	6.769231
1099	9-Jun-05	2005	SC	0.183486	0	0.073394	0	0.006329	6.769231
1100	9-Jun-05	2005	SC	0.183486	0	0.073394	0	0.006329	6.769231
1101	9-Jun-05	2005	SC	0.183486	0	0.073394	0	0.006329	6.769231
1105	9-Jun-05	2005	SC	0.183486	0	0.073394	0	0.006329	6.769231
1106	9-Jun-05	2005	SC	0.183486	0	0.073394	0	0.006329	6.769231
1111	9-Jun-05	2005	SC	0.183486	0	0.073394	0	0.006329	6.769231
1116	9-Jun-05	2005	SC	0.183486	0	0.073394	0	0.006329	6.769231
1117	9-Jun-05	2005	SC	0.183486	0	0.073394	0	0.006329	6.769231
1109	9-Jun-05	2005	SC	0.183486	0	0.073394	0	0.006329	6.769231
1107	9-Jun-05	2005	SC	0.183486	0	0.073394	0	0.006329	6.769231
1114	9-Jun-05	2005	SC	0.183486	0	0.073394	0	0.006329	6.769231
1094	9-Jun-05	2005	SC	0.183486	0	0.073394	0	0.006329	6.769231
1112	9-Jun-05	2005	SC	0.183486	0	0.073394	0	0.006329	6.769231
1110	9-Jun-05	2005	SC	0.183486	0	0.073394	0	0.006329	6.769231
1115	9-Jun-05	2005	SC	0.183486	0	0.073394	0	0.006329	6.769231
1103	9-Jun-05	2005	SC	0.183486	0	0.073394	0	0.006329	6.769231
1104	9-Jun-05	2005	SC	0.183486	0	0.073394	0	0.006329	6.769231
1402	10-Jun-05	2005	SC	0.183486	0	0.073394	0	0.006329	6.769231
1404	10-Jun-05	2005	SC	0.183486	0	0.073394	0	0.006329	6.769231
1406	10-Jun-05	2005	SC	0.183486	0	0.073394	0	0.006329	6.769231
1407	10-Jun-05	2005	SC	0.183486	0	0.073394	0	0.006329	6.769231
1412	10-Jun-05	2005	SC	0.183486	0	0.073394	0	0.006329	6.769231

1405	10-Jun-05	2005	SC	0.183486	0	0.073394	0	0.006329	6.769231
1403	10-Jun-05	2005	SC	0.183486	0	0.073394	0	0.006329	6.769231
1410	10-Jun-05	2005	SC	0.183486	0	0.073394	0	0.006329	6.769231
1408	10-Jun-05	2005	SC	0.183486	0	0.073394	0	0.006329	6.769231
1409	10-Jun-05	2005	SC	0.183486	0	0.073394	0	0.006329	6.769231
1411	10-Jun-05	2005	SC	0.183486	0	0.073394	0	0.006329	6.769231
1168	11-Jun-05	2005	SW	0.264151	0.113208	0.018868	0.169811	0.044444	1.4
1167	11-Jun-05	2005	SW	0.264151	0.113208	0.018868	0.169811	0.044444	1.4
1160	11-Jun-05	2005	SW	0.264151	0.113208	0.018868	0.169811	0.044444	1.4
1161	11-Jun-05	2005	SW	0.264151	0.113208	0.018868	0.169811	0.044444	1.4
1169	11-Jun-05	2005	SW	0.264151	0.113208	0.018868	0.169811	0.044444	1.4
1166	11-Jun-05	2005	SW	0.264151	0.113208	0.018868	0.169811	0.044444	1.4
1152	11-Jun-05	2005	SW	0.264151	0.113208	0.018868	0.169811	0.044444	1.4
1155	11-Jun-05	2005	SW	0.264151	0.113208	0.018868	0.169811	0.044444	1.4
1162	11-Jun-05	2005	SW	0.264151	0.113208	0.018868	0.169811	0.044444	1.4
1164	11-Jun-05	2005	SW	0.264151	0.113208	0.018868	0.169811	0.044444	1.4
1165	11-Jun-05	2005	SW	0.264151	0.113208	0.018868	0.169811	0.044444	1.4
1170	11-Jun-05	2005	SW	0.264151	0.113208	0.018868	0.169811	0.044444	1.4
1158	11-Jun-05	2005	SW	0.264151	0.113208	0.018868	0.169811	0.044444	1.4
1159	11-Jun-05	2005	SW	0.264151	0.113208	0.018868	0.169811	0.044444	1.4
1163	11-Jun-05	2005	SW	0.264151	0.113208	0.018868	0.169811	0.044444	1.4
1154	11-Jun-05	2005	SW	0.264151	0.113208	0.018868	0.169811	0.044444	1.4
1157	11-Jun-05	2005	SW	0.264151	0.113208	0.018868	0.169811	0.044444	1.4
1153	11-Jun-05	2005	SW	0.264151	0.113208	0.018868	0.169811	0.044444	1.4
1156	11-Jun-05	2005	SW	0.264151	0.113208	0.018868	0.169811	0.044444	1.4
1171	11-Jun-05	2005	SW	0.264151	0.113208	0.018868	0.169811	0.044444	1.4
1600	12-Jun-05	2005	SD	0.365079	0.095238	0.095238	0.380952	0.048387	2.5
1606	12-Jun-05	2005	SD	0.365079	0.095238	0.095238	0.380952	0.048387	2.5
1612	12-Jun-05	2005	SD	0.365079	0.095238	0.095238	0.380952	0.048387	2.5
1613	12-Jun-05	2005	SD	0.365079	0.095238	0.095238	0.380952	0.048387	2.5
1614	12-Jun-05	2005	SD	0.365079	0.095238	0.095238	0.380952	0.048387	2.5
1616	12-Jun-05	2005	SD	0.365079	0.095238	0.095238	0.380952	0.048387	2.5
1619	12-Jun-05	2005	SD	0.365079	0.095238	0.095238	0.380952	0.048387	2.5
1621	12-Jun-05	2005	SD	0.365079	0.095238	0.095238	0.380952	0.048387	2.5
1609	12-Jun-05	2005	SD	0.365079	0.095238	0.095238	0.380952	0.048387	2.5
1611	12-Jun-05	2005	SD	0.365079	0.095238	0.095238	0.380952	0.048387	2.5
1602	12-Jun-05	2005	SD	0.365079	0.095238	0.095238	0.380952	0.048387	2.5
1603	12-Jun-05	2005	SD	0.365079	0.095238	0.095238	0.380952	0.048387	2.5
1604	12-Jun-05	2005	SD	0.365079	0.095238	0.095238	0.380952	0.048387	2.5
1618	12-Jun-05	2005	SD	0.365079	0.095238	0.095238	0.380952	0.048387	2.5
1607	12-Jun-05	2005	SD	0.365079	0.095238	0.095238	0.380952	0.048387	2.5
1608	12-Jun-05	2005	SD	0.365079	0.095238	0.095238	0.380952	0.048387	2.5
1615	12-Jun-05	2005	SD	0.365079	0.095238	0.095238	0.380952	0.048387	2.5
1620	12-Jun-05	2005	SD	0.365079	0.095238	0.095238	0.380952	0.048387	2.5

1622	12-Jun-05	2005	SD	0.365079	0.095238	0.095238	0.380952	0.048387	2.5
1601	12-Jun-05	2005	SD	0.365079	0.095238	0.095238	0.380952	0.048387	2.5
1605	12-Jun-05	2005	SD	0.365079	0.095238	0.095238	0.380952	0.048387	2.5
1617	12-Jun-05	2005	SD	0.365079	0.095238	0.095238	0.380952	0.048387	2.5
1610	12-Jun-05	2005	SD	0.365079	0.095238	0.095238	0.380952	0.048387	2.5
1623	12-Jun-05	2005	SD	0.365079	0.095238	0.095238	0.380952	0.048387	2.5
1205	13-Jun-05	2005	TO	0.285714	0.142857	0.017857	0.142857	0.014577	1
1209	13-Jun-05	2005	TO	0.285714	0.142857	0.017857	0.142857	0.014577	1
1198	13-Jun-05	2005	TO	0.285714	0.142857	0.017857	0.142857	0.014577	1
1208	13-Jun-05	2005	TO	0.285714	0.142857	0.017857	0.142857	0.014577	1
1204	13-Jun-05	2005	TO	0.285714	0.142857	0.017857	0.142857	0.014577	1
1197	13-Jun-05	2005	TO	0.285714	0.142857	0.017857	0.142857	0.014577	1
1199	13-Jun-05	2005	TO	0.285714	0.142857	0.017857	0.142857	0.014577	1
1201	13-Jun-05	2005	TO	0.285714	0.142857	0.017857	0.142857	0.014577	1
1203	13-Jun-05	2005	TO	0.285714	0.142857	0.017857	0.142857	0.014577	1
1206	13-Jun-05	2005	TO	0.285714	0.142857	0.017857	0.142857	0.014577	1
1207	13-Jun-05	2005	TO	0.285714	0.142857	0.017857	0.142857	0.014577	1
1200	13-Jun-05	2005	TO	0.285714	0.142857	0.017857	0.142857	0.014577	1
1202	13-Jun-05	2005	TO	0.285714	0.142857	0.017857	0.142857	0.014577	1
1679	17-Jun-05	2005	LT1	0.393939	0.030303	0	0.242424	0	4.444444
1678	17-Jun-05	2005	LT1	0.393939	0.030303	0	0.242424	0	4.444444
1677	17-Jun-05	2005	LT1	0.393939	0.030303	0	0.242424	0	4.444444
1676	17-Jun-05	2005	LT1	0.393939	0.030303	0	0.242424	0	4.444444
1675	17-Jun-05	2005	LT1	0.393939	0.030303	0	0.242424	0	4.444444
1667	17-Jun-05	2005	LT1	0.393939	0.030303	0	0.242424	0	4.444444
1668	17-Jun-05	2005	LT1	0.393939	0.030303	0	0.242424	0	4.444444
1669	17-Jun-05	2005	LT1	0.393939	0.030303	0	0.242424	0	4.444444
1671	17-Jun-05	2005	LT1	0.393939	0.030303	0	0.242424	0	4.444444
1672	17-Jun-05	2005	LT1	0.393939	0.030303	0	0.242424	0	4.444444
1674	17-Jun-05	2005	LT1	0.393939	0.030303	0	0.242424	0	4.444444
1670	17-Jun-05	2005	LT1	0.393939	0.030303	0	0.242424	0	4.444444
1673	17-Jun-05	2005	LT1	0.393939	0.030303	0	0.242424	0	4.444444
1486	17-Jun-05	2005	LT1	0.393939	0.030303	0	0.242424	0	4.444444
1491	17-Jun-05	2005	LT1	0.393939	0.030303	0	0.242424	0	4.444444
1489	17-Jun-05	2005	LT1	0.393939	0.030303	0	0.242424	0	4.444444
1484	17-Jun-05	2005	LT1	0.393939	0.030303	0	0.242424	0	4.444444
1472	17-Jun-05	2005	LT1	0.393939	0.030303	0	0.242424	0	4.444444
1473	17-Jun-05	2005	LT1	0.393939	0.030303	0	0.242424	0	4.444444
1474	17-Jun-05	2005	LT1	0.393939	0.030303	0	0.242424	0	4.444444
1476	17-Jun-05	2005	LT1	0.393939	0.030303	0	0.242424	0	4.444444
1475	17-Jun-05	2005	LT1	0.393939	0.030303	0	0.242424	0	4.444444
1477	17-Jun-05	2005	LT1	0.393939	0.030303	0	0.242424	0	4.444444
1478	17-Jun-05	2005	LT1	0.393939	0.030303	0	0.242424	0	4.444444
1479	17-Jun-05	2005	LT1	0.393939	0.030303	0	0.242424	0	4.444444

1480	17-Jun-05	2005	LT1	0.393939	0.030303	0	0.242424	0	4.444444
1481	17-Jun-05	2005	LT1	0.393939	0.030303	0	0.242424	0	4.444444
1483	17-Jun-05	2005	LT1	0.393939	0.030303	0	0.242424	0	4.444444
1485	17-Jun-05	2005	LT1	0.393939	0.030303	0	0.242424	0	4.444444
1487	17-Jun-05	2005	LT1	0.393939	0.030303	0	0.242424	0	4.444444
1490	17-Jun-05	2005	LT1	0.393939	0.030303	0	0.242424	0	4.444444
1492	17-Jun-05	2005	LT1	0.393939	0.030303	0	0.242424	0	4.444444
1482	17-Jun-05	2005	LT1	0.393939	0.030303	0	0.242424	0	4.444444
1488	17-Jun-05	2005	LT1	0.393939	0.030303	0	0.242424	0	4.444444
1550	18-Jun-05	2005	SD	0.365079	0.095238	0.095238	0.380952	0.048387	2.5
1547	18-Jun-05	2005	SD	0.365079	0.095238	0.095238	0.380952	0.048387	2.5
1549	18-Jun-05	2005	SD	0.365079	0.095238	0.095238	0.380952	0.048387	2.5
1545	18-Jun-05	2005	SD	0.365079	0.095238	0.095238	0.380952	0.048387	2.5
1548	18-Jun-05	2005	SD	0.365079	0.095238	0.095238	0.380952	0.048387	2.5
1560	18-Jun-05	2005	SD	0.365079	0.095238	0.095238	0.380952	0.048387	2.5
1537	18-Jun-05	2005	SD	0.365079	0.095238	0.095238	0.380952	0.048387	2.5
1539	18-Jun-05	2005	SD	0.365079	0.095238	0.095238	0.380952	0.048387	2.5
1540	18-Jun-05	2005	SD	0.365079	0.095238	0.095238	0.380952	0.048387	2.5
1541	18-Jun-05	2005	SD	0.365079	0.095238	0.095238	0.380952	0.048387	2.5
1542	18-Jun-05	2005	SD	0.365079	0.095238	0.095238	0.380952	0.048387	2.5
1544	18-Jun-05	2005	SD	0.365079	0.095238	0.095238	0.380952	0.048387	2.5
1553	18-Jun-05	2005	SD	0.365079	0.095238	0.095238	0.380952	0.048387	2.5
1555	18-Jun-05	2005	SD	0.365079	0.095238	0.095238	0.380952	0.048387	2.5
1557	18-Jun-05	2005	SD	0.365079	0.095238	0.095238	0.380952	0.048387	2.5
1559	18-Jun-05	2005	SD	0.365079	0.095238	0.095238	0.380952	0.048387	2.5
1561	18-Jun-05	2005	SD	0.365079	0.095238	0.095238	0.380952	0.048387	2.5
1546	18-Jun-05	2005	SD	0.365079	0.095238	0.095238	0.380952	0.048387	2.5
1551	18-Jun-05	2005	SD	0.365079	0.095238	0.095238	0.380952	0.048387	2.5
1558	18-Jun-05	2005	SD	0.365079	0.095238	0.095238	0.380952	0.048387	2.5
1562	18-Jun-05	2005	SD	0.365079	0.095238	0.095238	0.380952	0.048387	2.5
1556	18-Jun-05	2005	SD	0.365079	0.095238	0.095238	0.380952	0.048387	2.5
1554	18-Jun-05	2005	SD	0.365079	0.095238	0.095238	0.380952	0.048387	2.5
1563	18-Jun-05	2005	SD	0.365079	0.095238	0.095238	0.380952	0.048387	2.5
1538	18-Jun-05	2005	SD	0.365079	0.095238	0.095238	0.380952	0.048387	2.5
1552	18-Jun-05	2005	SD	0.365079	0.095238	0.095238	0.380952	0.048387	2.5
1543	18-Jun-05	2005	SD	0.365079	0.095238	0.095238	0.380952	0.048387	2.5
1738	19-Jun-05	2005	ML						
1737	19-Jun-05	2005	ML						
1743	19-Jun-05	2005	ML						
1739	19-Jun-05	2005	ML						
1736	19-Jun-05	2005	ML						
1742	19-Jun-05	2005	ML						
1732	19-Jun-05	2005	ML						
1734	19-Jun-05	2005	ML						

1735	19-Jun-05	2005	ML						
1741	19-Jun-05	2005	ML						
1733	19-Jun-05	2005	ML						
1740	19-Jun-05	2005	ML						
52	16-May-06	2006	RP	0.233333	0	0.066667	0.3	0.027778	2.75
42	16-May-06	2006	RP	0.233333	0	0.066667	0.3	0.027778	2.75
43	16-May-06	2006	RP	0.233333	0	0.066667	0.3	0.027778	2.75
45	16-May-06	2006	RP	0.233333	0	0.066667	0.3	0.027778	2.75
46	16-May-06	2006	RP	0.233333	0	0.066667	0.3	0.027778	2.75
49	16-May-06	2006	RP	0.233333	0	0.066667	0.3	0.027778	2.75
50	16-May-06	2006	RP	0.233333	0	0.066667	0.3	0.027778	2.75
51	16-May-06	2006	RP	0.233333	0	0.066667	0.3	0.027778	2.75
44	16-May-06	2006	RP	0.233333	0	0.066667	0.3	0.027778	2.75
48	16-May-06	2006	RP	0.233333	0	0.066667	0.3	0.027778	2.75
47	16-May-06	2006	RP	0.233333	0	0.066667	0.3	0.027778	2.75
76	18-May-06	2006	SW	0.210526	0.026316	0.105263	0.131579	0.017778	1.4
79	18-May-06	2006	SW	0.210526	0.026316	0.105263	0.131579	0.017778	1.4
78	18-May-06	2006	SW	0.210526	0.026316	0.105263	0.131579	0.017778	1.4
80	18-May-06	2006	SW	0.210526	0.026316	0.105263	0.131579	0.017778	1.4
81	18-May-06	2006	SW	0.210526	0.026316	0.105263	0.131579	0.017778	1.4
77	18-May-06	2006	SW	0.210526	0.026316	0.105263	0.131579	0.017778	1.4
98	27-May-06	2006	HH	0.102941	0.161765	0	0.352941	0	0.615385
100	27-May-06	2006	HH	0.102941	0.161765	0	0.352941	0	0.615385
107	27-May-06	2006	HH	0.102941	0.161765	0	0.352941	0	0.615385
103	27-May-06	2006	HH	0.102941	0.161765	0	0.352941	0	0.615385
97	27-May-06	2006	HH	0.102941	0.161765	0	0.352941	0	0.615385
96	27-May-06	2006	HH	0.102941	0.161765	0	0.352941	0	0.615385
99	27-May-06	2006	HH	0.102941	0.161765	0	0.352941	0	0.615385
101	27-May-06	2006	HH	0.102941	0.161765	0	0.352941	0	0.615385
102	27-May-06	2006	HH	0.102941	0.161765	0	0.352941	0	0.615385
106	27-May-06	2006	HH	0.102941	0.161765	0	0.352941	0	0.615385
109	27-May-06	2006	HH	0.102941	0.161765	0	0.352941	0	0.615385
104	27-May-06	2006	HH	0.102941	0.161765	0	0.352941	0	0.615385
105	27-May-06	2006	HH	0.102941	0.161765	0	0.352941	0	0.615385
108	27-May-06	2006	HH	0.102941	0.161765	0	0.352941	0	0.615385
108	28-May-06	2006	TO	0.075	0.1	0.05	0.225	0.005814	0.9375
107	28-May-06	2006	TO	0.075	0.1	0.05	0.225	0.005814	0.9375
104	28-May-06	2006	TO	0.075	0.1	0.05	0.225	0.005814	0.9375
106	28-May-06	2006	TO	0.075	0.1	0.05	0.225	0.005814	0.9375
105	28-May-06	2006	TO	0.075	0.1	0.05	0.225	0.005814	0.9375
102	28-May-06	2006	TO	0.075	0.1	0.05	0.225	0.005814	0.9375
109	28-May-06	2006	TO	0.075	0.1	0.05	0.225	0.005814	0.9375
101	28-May-06	2006	TO	0.075	0.1	0.05	0.225	0.005814	0.9375
107	28-May-06	2006	TO	0.075	0.1	0.05	0.225	0.005814	0.9375

108	28-May-06	2006	TO	0.075	0.1	0.05	0.225	0.005814	0.9375
101	28-May-06	2006	TO	0.075	0.1	0.05	0.225	0.005814	0.9375
109	28-May-06	2006	TO	0.075	0.1	0.05	0.225	0.005814	0.9375
103	28-May-06	2006	TO	0.075	0.1	0.05	0.225	0.005814	0.9375
102	29-May-06	2006	RP	0.233333	0	0.066667	0.3	0.027778	2.75
104	29-May-06	2006	RP	0.233333	0	0.066667	0.3	0.027778	2.75
105	29-May-06	2006	RP	0.233333	0	0.066667	0.3	0.027778	2.75
106	29-May-06	2006	RP	0.233333	0	0.066667	0.3	0.027778	2.75
107	29-May-06	2006	RP	0.233333	0	0.066667	0.3	0.027778	2.75
108	29-May-06	2006	RP	0.233333	0	0.066667	0.3	0.027778	2.75
103	29-May-06	2006	RP	0.233333	0	0.066667	0.3	0.027778	2.75
109	29-May-06	2006	RP	0.233333	0	0.066667	0.3	0.027778	2.75
126	29-May-06	2006	SW	0.210526	0.026316	0.105263	0.131579	0.017778	1.4
127	29-May-06	2006	SW	0.210526	0.026316	0.105263	0.131579	0.017778	1.4
123	29-May-06	2006	SW	0.210526	0.026316	0.105263	0.131579	0.017778	1.4
128	29-May-06	2006	SW	0.210526	0.026316	0.105263	0.131579	0.017778	1.4
119	29-May-06	2006	SW	0.210526	0.026316	0.105263	0.131579	0.017778	1.4
122	29-May-06	2006	SW	0.210526	0.026316	0.105263	0.131579	0.017778	1.4
121	29-May-06	2006	SW	0.210526	0.026316	0.105263	0.131579	0.017778	1.4
118	29-May-06	2006	SW	0.210526	0.026316	0.105263	0.131579	0.017778	1.4
125	29-May-06	2006	SW	0.210526	0.026316	0.105263	0.131579	0.017778	1.4
117	29-May-06	2006	SW	0.210526	0.026316	0.105263	0.131579	0.017778	1.4
120	29-May-06	2006	SW	0.210526	0.026316	0.105263	0.131579	0.017778	1.4
124	29-May-06	2006	SW	0.210526	0.026316	0.105263	0.131579	0.017778	1.4
140	31-May-06	2006	SC	0.092308	0	0.030769	0.046154	0.002941	7.961538
137	31-May-06	2006	SC	0.092308	0	0.030769	0.046154	0.002941	7.961538
138	31-May-06	2006	SC	0.092308	0	0.030769	0.046154	0.002941	7.961538
146	31-May-06	2006	SC	0.092308	0	0.030769	0.046154	0.002941	7.961538
147	31-May-06	2006	SC	0.092308	0	0.030769	0.046154	0.002941	7.961538
144	31-May-06	2006	SC	0.092308	0	0.030769	0.046154	0.002941	7.961538
141	31-May-06	2006	SC	0.092308	0	0.030769	0.046154	0.002941	7.961538
142	31-May-06	2006	SC	0.092308	0	0.030769	0.046154	0.002941	7.961538
145	31-May-06	2006	SC	0.092308	0	0.030769	0.046154	0.002941	7.961538
136	31-May-06	2006	SC	0.092308	0	0.030769	0.046154	0.002941	7.961538
139	31-May-06	2006	SC	0.092308	0	0.030769	0.046154	0.002941	7.961538
143	31-May-06	2006	SC	0.092308	0	0.030769	0.046154	0.002941	7.961538
150	1-Jun-06	2006	KN	0.157895	0.105263	0.026316	0.368421	0	1.133333
149	1-Jun-06	2006	KN	0.157895	0.105263	0.026316	0.368421	0	1.133333
152	1-Jun-06	2006	KN	0.157895	0.105263	0.026316	0.368421	0	1.133333
154	1-Jun-06	2006	KN	0.157895	0.105263	0.026316	0.368421	0	1.133333
148	1-Jun-06	2006	KN	0.157895	0.105263	0.026316	0.368421	0	1.133333
151	1-Jun-06	2006	KN	0.157895	0.105263	0.026316	0.368421	0	1.133333
153	1-Jun-06	2006	KN	0.157895	0.105263	0.026316	0.368421	0	1.133333
179	3-Jun-06	2006	SC	0.092308	0	0.030769	0.046154	0.002941	7.961538

178	3-Jun-06	2006	SC	0.092308	0	0.030769	0.046154	0.002941	7.961538
181	3-Jun-06	2006	SC	0.092308	0	0.030769	0.046154	0.002941	7.961538
177	3-Jun-06	2006	SC	0.092308	0	0.030769	0.046154	0.002941	7.961538
176	3-Jun-06	2006	SC	0.092308	0	0.030769	0.046154	0.002941	7.961538
183	3-Jun-06	2006	SC	0.092308	0	0.030769	0.046154	0.002941	7.961538
175	3-Jun-06	2006	SC	0.092308	0	0.030769	0.046154	0.002941	7.961538
180	3-Jun-06	2006	SC	0.092308	0	0.030769	0.046154	0.002941	7.961538
182	3-Jun-06	2006	SC	0.092308	0	0.030769	0.046154	0.002941	7.961538
209	6-Jun-06	2006	ML						
208	6-Jun-06	2006	ML						
210	6-Jun-06	2006	ML						
211	6-Jun-06	2006	ML						
228	8-Jun-06	2006	LT1	0.222222	0.111111	0	0.148148	0	4
232	8-Jun-06	2006	LT1	0.222222	0.111111	0	0.148148	0	4
227	8-Jun-06	2006	LT1	0.222222	0.111111	0	0.148148	0	4
229	8-Jun-06	2006	LT1	0.222222	0.111111	0	0.148148	0	4
231	8-Jun-06	2006	LT1	0.222222	0.111111	0	0.148148	0	4
225	8-Jun-06	2006	LT1	0.222222	0.111111	0	0.148148	0	4
226	8-Jun-06	2006	LT1	0.222222	0.111111	0	0.148148	0	4
230	8-Jun-06	2006	LT1	0.222222	0.111111	0	0.148148	0	4
264	11-Jun-06	2006	D1	0.04	0.04	0.06	0.22	0.008386	0.75
260	11-Jun-06	2006	D1	0.04	0.04	0.06	0.22	0.008386	0.75
262	11-Jun-06	2006	D1	0.04	0.04	0.06	0.22	0.008386	0.75
266	11-Jun-06	2006	D1	0.04	0.04	0.06	0.22	0.008386	0.75
261	11-Jun-06	2006	D1	0.04	0.04	0.06	0.22	0.008386	0.75
259	11-Jun-06	2006	D1	0.04	0.04	0.06	0.22	0.008386	0.75
263	11-Jun-06	2006	D1	0.04	0.04	0.06	0.22	0.008386	0.75
265	11-Jun-06	2006	D1	0.04	0.04	0.06	0.22	0.008386	0.75
	11-Jun-06	2006	D2	0.111111	0	0.055556	0.327273	0	3.181818
	11-Jun-06	2006	D2	0.111111	0	0.055556	0.327273	0	3.181818
	11-Jun-06	2006	D2	0.111111	0	0.055556	0.327273	0	3.181818
	11-Jun-06	2006	D2	0.111111	0	0.055556	0.327273	0	3.181818
	11-Jun-06	2006	D2	0.111111	0	0.055556	0.327273	0	3.181818
	11-Jun-06	2006	D2	0.111111	0	0.055556	0.327273	0	3.181818
	11-Jun-06	2006	D2	0.111111	0	0.055556	0.327273	0	3.181818
	11-Jun-06	2006	D2	0.111111	0	0.055556	0.327273	0	3.181818
	11-Jun-06	2006	D2	0.111111	0	0.055556	0.327273	0	3.181818
	11-Jun-06	2006	D2	0.111111	0	0.055556	0.327273	0	3.181818
	11-Jun-06	2006	D2	0.111111	0	0.055556	0.327273	0	3.181818
	11-Jun-06	2006	D2	0.111111	0	0.055556	0.327273	0	3.181818
	15-Jun-06	2006	SC	0.092308	0	0.030769	0.046154	0.002941	7.961538
	15-Jun-06	2006	SC	0.092308	0	0.030769	0.046154	0.002941	7.961538
	15-Jun-06	2006	SC	0.092308	0	0.030769	0.046154	0.002941	7.961538
	15-Jun-06	2006	SC	0.092308	0	0.030769	0.046154	0.002941	7.961538

15-Jun-06	2006 SC	0.092308	0	0.030769	0.046154	0.002941	7.961538
15-Jun-06	2006 SC	0.092308	0	0.030769	0.046154	0.002941	7.961538
15-Jun-06	2006 SC	0.092308	0	0.030769	0.046154	0.002941	7.961538
15-Jun-06	2006 SC	0.092308	0	0.030769	0.046154	0.002941	7.961538
15-Jun-06	2006 SC	0.092308	0	0.030769	0.046154	0.002941	7.961538
15-Jun-06	2006 SC	0.092308	0	0.030769	0.046154	0.002941	7.961538
15-Jun-06	2006 SC	0.092308	0	0.030769	0.046154	0.002941	7.961538
15-Jun-06	2006 SC	0.092308	0	0.030769	0.046154	0.002941	7.961538
15-Jun-06	2006 SC	0.092308	0	0.030769	0.046154	0.002941	7.961538
15-Jun-06	2006 SC	0.092308	0	0.030769	0.046154	0.002941	7.961538
15-Jun-06	2006 SC	0.092308	0	0.030769	0.046154	0.002941	7.961538
15-Jun-06	2006 SC	0.092308	0	0.030769	0.046154	0.002941	7.961538
15-Jun-06	2006 SC	0.092308	0	0.030769	0.046154	0.002941	7.961538
15-Jun-06	2006 SC	0.092308	0	0.030769	0.046154	0.002941	7.961538
15-Jun-06	2006 SC	0.092308	0	0.030769	0.046154	0.002941	7.961538
15-Jun-06	2006 SC	0.092308	0	0.030769	0.046154	0.002941	7.961538
15-Jun-06	2006 SC	0.092308	0	0.030769	0.046154	0.002941	7.961538
15-Jun-06	2006 SC	0.092308	0	0.030769	0.046154	0.002941	7.961538
15-Jun-06	2006 SC	0.092308	0	0.030769	0.046154	0.002941	7.961538
15-Jun-06	2006 SC	0.092308	0	0.030769	0.046154	0.002941	7.961538
15-Jun-06	2006 SC	0.092308	0	0.030769	0.046154	0.002941	7.961538
15-Jun-06	2006 SC	0.092308	0	0.030769	0.046154	0.002941	7.961538
15-Jun-06	2006 SC	0.092308	0	0.030769	0.046154	0.002941	7.961538
15-Jun-06	2006 SC	0.092308	0	0.030769	0.046154	0.002941	7.961538
15-Jun-06	2006 SC	0.092308	0	0.030769	0.046154	0.002941	7.961538
15-Jun-06	2006 SC	0.092308	0	0.030769	0.046154	0.002941	7.961538
15-Jun-06	2006 SC	0.092308	0	0.030769	0.046154	0.002941	7.961538
15-Jun-06	2006 SC	0.092308	0	0.030769	0.046154	0.002941	7.961538
15-Jun-06	2006 SC	0.092308	0	0.030769	0.046154	0.002941	7.961538
15-Jun-06	2006 SC	0.092308	0	0.030769	0.046154	0.002941	7.961538
15-Jun-06	2006 SC	0.092308	0	0.030769	0.046154	0.002941	7.961538
15-Jun-06	2006 SC	0.092308	0	0.030769	0.046154	0.002941	7.961538
16-Jun-06	2006 RP	0.233333	0	0.066667	0.3	0.027778	2.75
16-Jun-06	2006 RP	0.233333	0	0.066667	0.3	0.027778	2.75
16-Jun-06	2006 RP	0.233333	0	0.066667	0.3	0.027778	2.75
16-Jun-06	2006 RP	0.233333	0	0.066667	0.3	0.027778	2.75
16-Jun-06	2006 RP	0.233333	0	0.066667	0.3	0.027778	2.75
16-Jun-06	2006 RP	0.233333	0	0.066667	0.3	0.027778	2.75
16-Jun-06	2006 RP	0.233333	0	0.066667	0.3	0.027778	2.75
16-Jun-06	2006 RP	0.233333	0	0.066667	0.3	0.027778	2.75
16-Jun-06	2006 RP	0.233333	0	0.066667	0.3	0.027778	2.75
16-Jun-06	2006 RP	0.233333	0	0.066667	0.3	0.027778	2.75
16-Jun-06	2006 RP	0.233333	0	0.066667	0.3	0.027778	2.75
16-Jun-06	2006 RP	0.233333	0	0.066667	0.3	0.027778	2.75

18-Jun-06	2006	HH	0.102941	0.161765	0	0.352941	0	0.615385
18-Jun-06	2006	HH	0.102941	0.161765	0	0.352941	0	0.615385
18-Jun-06	2006	HH	0.102941	0.161765	0	0.352941	0	0.615385
18-Jun-06	2006	HH	0.102941	0.161765	0	0.352941	0	0.615385
18-Jun-06	2006	HH	0.102941	0.161765	0	0.352941	0	0.615385
18-Jun-06	2006	HH	0.102941	0.161765	0	0.352941	0	0.615385
18-Jun-06	2006	HH	0.102941	0.161765	0	0.352941	0	0.615385
18-Jun-06	2006	HH	0.102941	0.161765	0	0.352941	0	0.615385
18-Jun-06	2006	HH	0.102941	0.161765	0	0.352941	0	0.615385
18-Jun-06	2006	HH	0.102941	0.161765	0	0.352941	0	0.615385
18-Jun-06	2006	HH	0.102941	0.161765	0	0.352941	0	0.615385
18-Jun-06	2006	HH	0.102941	0.161765	0	0.352941	0	0.615385
18-Jun-06	2006	HH	0.102941	0.161765	0	0.352941	0	0.615385
18-Jun-06	2006	HH	0.102941	0.161765	0	0.352941	0	0.615385
18-Jun-06	2006	HH	0.102941	0.161765	0	0.352941	0	0.615385
18-Jun-06	2006	HH	0.102941	0.161765	0	0.352941	0	0.615385
18-Jun-06	2006	HH	0.102941	0.161765	0	0.352941	0	0.615385
22-Jun-06	2006	SC	0.092308	0	0.030769	0.046154	0.002941	7.961538
22-Jun-06	2006	SC	0.092308	0	0.030769	0.046154	0.002941	7.961538
22-Jun-06	2006	SC	0.092308	0	0.030769	0.046154	0.002941	7.961538
22-Jun-06	2006	SC	0.092308	0	0.030769	0.046154	0.002941	7.961538
22-Jun-06	2006	SC	0.092308	0	0.030769	0.046154	0.002941	7.961538
22-Jun-06	2006	SC	0.092308	0	0.030769	0.046154	0.002941	7.961538
22-Jun-06	2006	SC	0.092308	0	0.030769	0.046154	0.002941	7.961538
22-Jun-06	2006	SC	0.092308	0	0.030769	0.046154	0.002941	7.961538
22-Jun-06	2006	SC	0.092308	0	0.030769	0.046154	0.002941	7.961538
22-Jun-06	2006	SC	0.092308	0	0.030769	0.046154	0.002941	7.961538
22-Jun-06	2006	SC	0.092308	0	0.030769	0.046154	0.002941	7.961538
22-Jun-06	2006	SC	0.092308	0	0.030769	0.046154	0.002941	7.961538
22-Jun-06	2006	SC	0.092308	0	0.030769	0.046154	0.002941	7.961538
22-Jun-06	2006	SC	0.092308	0	0.030769	0.046154	0.002941	7.961538
22-Jun-06	2006	SC	0.092308	0	0.030769	0.046154	0.002941	7.961538
22-Jun-06	2006	SC	0.092308	0	0.030769	0.046154	0.002941	7.961538
22-Jun-06	2006	SC	0.092308	0	0.030769	0.046154	0.002941	7.961538
22-Jun-06	2006	SC	0.092308	0	0.030769	0.046154	0.002941	7.961538
22-Jun-06	2006	SC	0.092308	0	0.030769	0.046154	0.002941	7.961538
28-Jun-06	2006	LT1	0.222222	0.111111	0	0.148148	0	4
28-Jun-06	2006	LT1	0.222222	0.111111	0	0.148148	0	4
28-Jun-06	2006	LT1	0.222222	0.111111	0	0.148148	0	4
28-Jun-06	2006	LT1	0.222222	0.111111	0	0.148148	0	4
28-Jun-06	2006	LT1	0.222222	0.111111	0	0.148148	0	4
28-Jun-06	2006	LT1	0.222222	0.111111	0	0.148148	0	4
28-Jun-06	2006	LT1	0.222222	0.111111	0	0.148148	0	4
28-Jun-06	2006	LT1	0.222222	0.111111	0	0.148148	0	4

	28-Jun-06	2006	LT1	0.222222	0.111111	0	0.148148	0	4
	28-Jun-06	2006	LT1	0.222222	0.111111	0	0.148148	0	4
	28-Jun-06	2006	LT1	0.222222	0.111111	0	0.148148	0	4
	28-Jun-06	2006	LT1	0.222222	0.111111	0	0.148148	0	4
590	8-May-07	2007	MG	0.24	0.28	0.12	0.02	0.009615	6.15
592	8-May-07	2007	MG	0.24	0.28	0.12	0.02	0.009615	6.15
598	8-May-07	2007	MG	0.24	0.28	0.12	0.02	0.009615	6.15
594	8-May-07	2007	MG	0.24	0.28	0.12	0.02	0.009615	6.15
595	8-May-07	2007	MG	0.24	0.28	0.12	0.02	0.009615	6.15
596	8-May-07	2007	MG	0.24	0.28	0.12	0.02	0.009615	6.15
591	8-May-07	2007	MG	0.24	0.28	0.12	0.02	0.009615	6.15
593	8-May-07	2007	MG	0.24	0.28	0.12	0.02	0.009615	6.15
597	8-May-07	2007	MG	0.24	0.28	0.12	0.02	0.009615	6.15
695	9-May-07	2007	TO	0.175	0.125	0.175	0	0.00289	0.875
696	9-May-07	2007	TO	0.175	0.125	0.175	0	0.00289	0.875
697	9-May-07	2007	TO	0.175	0.125	0.175	0	0.00289	0.875
693	9-May-07	2007	TO	0.175	0.125	0.175	0	0.00289	0.875
698	9-May-07	2007	TO	0.175	0.125	0.175	0	0.00289	0.875
692	9-May-07	2007	TO	0.175	0.125	0.175	0	0.00289	0.875
694	9-May-07	2007	TO	0.175	0.125	0.175	0	0.00289	0.875
699	9-May-07	2007	TO	0.175	0.125	0.175	0	0.00289	0.875
691	9-May-07	2007	TO	0.175	0.125	0.175	0	0.00289	0.875
700	9-May-07	2007	TO	0.175	0.125	0.175	0	0.00289	0.875
701	9-May-07	2007	TO	0.175	0.125	0.175	0	0.00289	0.875
689	9-May-07	2007	TO	0.175	0.125	0.175	0	0.00289	0.875
690	9-May-07	2007	TO	0.175	0.125	0.175	0	0.00289	0.875
681	9-May-07	2007	TO	0.175	0.125	0.175	0	0.00289	0.875
685	9-May-07	2007	TO	0.175	0.125	0.175	0	0.00289	0.875
686	9-May-07	2007	TO	0.175	0.125	0.175	0	0.00289	0.875
687	9-May-07	2007	TO	0.175	0.125	0.175	0	0.00289	0.875
683	9-May-07	2007	TO	0.175	0.125	0.175	0	0.00289	0.875
684	9-May-07	2007	TO	0.175	0.125	0.175	0	0.00289	0.875
702	9-May-07	2007	TO	0.175	0.125	0.175	0	0.00289	0.875
678	9-May-07	2007	TO	0.175	0.125	0.175	0	0.00289	0.875
679	9-May-07	2007	TO	0.175	0.125	0.175	0	0.00289	0.875
680	9-May-07	2007	TO	0.175	0.125	0.175	0	0.00289	0.875
677	9-May-07	2007	TO	0.175	0.125	0.175	0	0.00289	0.875
682	9-May-07	2007	TO	0.175	0.125	0.175	0	0.00289	0.875
688	9-May-07	2007	TO	0.175	0.125	0.175	0	0.00289	0.875
372	13-May-07	2007	SC	0.136986	0.041096	0.027397	0	0	8.576923
351	13-May-07	2007	SC	0.136986	0.041096	0.027397	0	0	8.576923
354	13-May-07	2007	SC	0.136986	0.041096	0.027397	0	0	8.576923
356	13-May-07	2007	SC	0.136986	0.041096	0.027397	0	0	8.576923
358	13-May-07	2007	SC	0.136986	0.041096	0.027397	0	0	8.576923

367	13-May-07	2007 SC	0.136986	0.041096	0.027397	0	0	8.576923
370	13-May-07	2007 SC	0.136986	0.041096	0.027397	0	0	8.576923
352	13-May-07	2007 SC	0.136986	0.041096	0.027397	0	0	8.576923
368	13-May-07	2007 SC	0.136986	0.041096	0.027397	0	0	8.576923
374	13-May-07	2007 SC	0.136986	0.041096	0.027397	0	0	8.576923
375	13-May-07	2007 SC	0.136986	0.041096	0.027397	0	0	8.576923
376	13-May-07	2007 SC	0.136986	0.041096	0.027397	0	0	8.576923
355	13-May-07	2007 SC	0.136986	0.041096	0.027397	0	0	8.576923
350	13-May-07	2007 SC	0.136986	0.041096	0.027397	0	0	8.576923
349	13-May-07	2007 SC	0.136986	0.041096	0.027397	0	0	8.576923
366	13-May-07	2007 SC	0.136986	0.041096	0.027397	0	0	8.576923
369	13-May-07	2007 SC	0.136986	0.041096	0.027397	0	0	8.576923
377	13-May-07	2007 SC	0.136986	0.041096	0.027397	0	0	8.576923
365	13-May-07	2007 SC	0.136986	0.041096	0.027397	0	0	8.576923
347	13-May-07	2007 SC	0.136986	0.041096	0.027397	0	0	8.576923
364	13-May-07	2007 SC	0.136986	0.041096	0.027397	0	0	8.576923
371	13-May-07	2007 SC	0.136986	0.041096	0.027397	0	0	8.576923
348	13-May-07	2007 SC	0.136986	0.041096	0.027397	0	0	8.576923
361	13-May-07	2007 SC	0.136986	0.041096	0.027397	0	0	8.576923
362	13-May-07	2007 SC	0.136986	0.041096	0.027397	0	0	8.576923
360	13-May-07	2007 SC	0.136986	0.041096	0.027397	0	0	8.576923
359	13-May-07	2007 SC	0.136986	0.041096	0.027397	0	0	8.576923
357	13-May-07	2007 SC	0.136986	0.041096	0.027397	0	0	8.576923
353	13-May-07	2007 SC	0.136986	0.041096	0.027397	0	0	8.576923
373	13-May-07	2007 SC	0.136986	0.041096	0.027397	0	0	8.576923
346	13-May-07	2007 SC	0.136986	0.041096	0.027397	0	0	8.576923
363	13-May-07	2007 SC	0.136986	0.041096	0.027397	0	0	8.576923
378	13-May-07	2007 SC	0.136986	0.041096	0.027397	0	0	8.576923
470	13-May-07	2007 RP	0.133333	0.033333	0.133333	0	0.019139	2.75
471	13-May-07	2007 RP	0.133333	0.033333	0.133333	0	0.019139	2.75
472	13-May-07	2007 RP	0.133333	0.033333	0.133333	0	0.019139	2.75
476	13-May-07	2007 RP	0.133333	0.033333	0.133333	0	0.019139	2.75
453	13-May-07	2007 RP	0.133333	0.033333	0.133333	0	0.019139	2.75
468	13-May-07	2007 RP	0.133333	0.033333	0.133333	0	0.019139	2.75
469	13-May-07	2007 RP	0.133333	0.033333	0.133333	0	0.019139	2.75
475	13-May-07	2007 RP	0.133333	0.033333	0.133333	0	0.019139	2.75
454	13-May-07	2007 RP	0.133333	0.033333	0.133333	0	0.019139	2.75
477	13-May-07	2007 RP	0.133333	0.033333	0.133333	0	0.019139	2.75
478	13-May-07	2007 RP	0.133333	0.033333	0.133333	0	0.019139	2.75
479	13-May-07	2007 RP	0.133333	0.033333	0.133333	0	0.019139	2.75
480	13-May-07	2007 RP	0.133333	0.033333	0.133333	0	0.019139	2.75
481	13-May-07	2007 RP	0.133333	0.033333	0.133333	0	0.019139	2.75
455	13-May-07	2007 RP	0.133333	0.033333	0.133333	0	0.019139	2.75
456	13-May-07	2007 RP	0.133333	0.033333	0.133333	0	0.019139	2.75

473	13-May-07	2007 RP	0.133333	0.033333	0.133333	0	0.019139	2.75
474	13-May-07	2007 RP	0.133333	0.033333	0.133333	0	0.019139	2.75
451	13-May-07	2007 RP	0.133333	0.033333	0.133333	0	0.019139	2.75
452	13-May-07	2007 RP	0.133333	0.033333	0.133333	0	0.019139	2.75
465	13-May-07	2007 RP	0.133333	0.033333	0.133333	0	0.019139	2.75
466	13-May-07	2007 RP	0.133333	0.033333	0.133333	0	0.019139	2.75
467	13-May-07	2007 RP	0.133333	0.033333	0.133333	0	0.019139	2.75
462	13-May-07	2007 RP	0.133333	0.033333	0.133333	0	0.019139	2.75
463	13-May-07	2007 RP	0.133333	0.033333	0.133333	0	0.019139	2.75
461	13-May-07	2007 RP	0.133333	0.033333	0.133333	0	0.019139	2.75
459	13-May-07	2007 RP	0.133333	0.033333	0.133333	0	0.019139	2.75
460	13-May-07	2007 RP	0.133333	0.033333	0.133333	0	0.019139	2.75
445	13-May-07	2007 RP	0.133333	0.033333	0.133333	0	0.019139	2.75
446	13-May-07	2007 RP	0.133333	0.033333	0.133333	0	0.019139	2.75
447	13-May-07	2007 RP	0.133333	0.033333	0.133333	0	0.019139	2.75
444	13-May-07	2007 RP	0.133333	0.033333	0.133333	0	0.019139	2.75
448	13-May-07	2007 RP	0.133333	0.033333	0.133333	0	0.019139	2.75
449	13-May-07	2007 RP	0.133333	0.033333	0.133333	0	0.019139	2.75
443	13-May-07	2007 RP	0.133333	0.033333	0.133333	0	0.019139	2.75
458	13-May-07	2007 RP	0.133333	0.033333	0.133333	0	0.019139	2.75
450	13-May-07	2007 RP	0.133333	0.033333	0.133333	0	0.019139	2.75
442	13-May-07	2007 RP	0.133333	0.033333	0.133333	0	0.019139	2.75
457	13-May-07	2007 RP	0.133333	0.033333	0.133333	0	0.019139	2.75
464	13-May-07	2007 RP	0.133333	0.033333	0.133333	0	0.019139	2.75
991	17-May-07	2007 TO	0.175	0.125	0.175	0	0.00289	0.875
992	17-May-07	2007 TO	0.175	0.125	0.175	0	0.00289	0.875
993	17-May-07	2007 TO	0.175	0.125	0.175	0	0.00289	0.875
998	17-May-07	2007 TO	0.175	0.125	0.175	0	0.00289	0.875
999	17-May-07	2007 TO	0.175	0.125	0.175	0	0.00289	0.875
1000	17-May-07	2007 TO	0.175	0.125	0.175	0	0.00289	0.875
994	17-May-07	2007 TO	0.175	0.125	0.175	0	0.00289	0.875
995	17-May-07	2007 TO	0.175	0.125	0.175	0	0.00289	0.875
996	17-May-07	2007 TO	0.175	0.125	0.175	0	0.00289	0.875
997	17-May-07	2007 TO	0.175	0.125	0.175	0	0.00289	0.875
1001	17-May-07	2007 TO	0.175	0.125	0.175	0	0.00289	0.875
1003	17-May-07	2007 TO	0.175	0.125	0.175	0	0.00289	0.875
1002	17-May-07	2007 TO	0.175	0.125	0.175	0	0.00289	0.875
986	17-May-07	2007 TO	0.175	0.125	0.175	0	0.00289	0.875
987	17-May-07	2007 TO	0.175	0.125	0.175	0	0.00289	0.875
988	17-May-07	2007 TO	0.175	0.125	0.175	0	0.00289	0.875
984	17-May-07	2007 TO	0.175	0.125	0.175	0	0.00289	0.875
985	17-May-07	2007 TO	0.175	0.125	0.175	0	0.00289	0.875
983	17-May-07	2007 TO	0.175	0.125	0.175	0	0.00289	0.875
989	17-May-07	2007 TO	0.175	0.125	0.175	0	0.00289	0.875

990	17-May-07	2007	TO	0.175	0.125	0.175	0	0.00289	0.875
1108	19-May-07	2007	D1	0.26	0.14	0.02	0.06	0.006036	0.8
1097	19-May-07	2007	D1	0.26	0.14	0.02	0.06	0.006036	0.8
1098	19-May-07	2007	D1	0.26	0.14	0.02	0.06	0.006036	0.8
1099	19-May-07	2007	D1	0.26	0.14	0.02	0.06	0.006036	0.8
1100	19-May-07	2007	D1	0.26	0.14	0.02	0.06	0.006036	0.8
1101	19-May-07	2007	D1	0.26	0.14	0.02	0.06	0.006036	0.8
1103	19-May-07	2007	D1	0.26	0.14	0.02	0.06	0.006036	0.8
1113	19-May-07	2007	D1	0.26	0.14	0.02	0.06	0.006036	0.8
1116	19-May-07	2007	D1	0.26	0.14	0.02	0.06	0.006036	0.8
1096	19-May-07	2007	D1	0.26	0.14	0.02	0.06	0.006036	0.8
1106	19-May-07	2007	D1	0.26	0.14	0.02	0.06	0.006036	0.8
1107	19-May-07	2007	D1	0.26	0.14	0.02	0.06	0.006036	0.8
1109	19-May-07	2007	D1	0.26	0.14	0.02	0.06	0.006036	0.8
1112	19-May-07	2007	D1	0.26	0.14	0.02	0.06	0.006036	0.8
1126	19-May-07	2007	D1	0.26	0.14	0.02	0.06	0.006036	0.8
1128	19-May-07	2007	D1	0.26	0.14	0.02	0.06	0.006036	0.8
1133	19-May-07	2007	D1	0.26	0.14	0.02	0.06	0.006036	0.8
1134	19-May-07	2007	D1	0.26	0.14	0.02	0.06	0.006036	0.8
1110	19-May-07	2007	D1	0.26	0.14	0.02	0.06	0.006036	0.8
1111	19-May-07	2007	D1	0.26	0.14	0.02	0.06	0.006036	0.8
1141	19-May-07	2007	D1	0.26	0.14	0.02	0.06	0.006036	0.8
1095	19-May-07	2007	D1	0.26	0.14	0.02	0.06	0.006036	0.8
1114	19-May-07	2007	D1	0.26	0.14	0.02	0.06	0.006036	0.8
1138	19-May-07	2007	D1	0.26	0.14	0.02	0.06	0.006036	0.8
1139	19-May-07	2007	D1	0.26	0.14	0.02	0.06	0.006036	0.8
1129	19-May-07	2007	D1	0.26	0.14	0.02	0.06	0.006036	0.8
1130	19-May-07	2007	D1	0.26	0.14	0.02	0.06	0.006036	0.8
1131	19-May-07	2007	D1	0.26	0.14	0.02	0.06	0.006036	0.8
1132	19-May-07	2007	D1	0.26	0.14	0.02	0.06	0.006036	0.8
1142	19-May-07	2007	D1	0.26	0.14	0.02	0.06	0.006036	0.8
1122	19-May-07	2007	D1	0.26	0.14	0.02	0.06	0.006036	0.8
1118	19-May-07	2007	D1	0.26	0.14	0.02	0.06	0.006036	0.8
1137	19-May-07	2007	D1	0.26	0.14	0.02	0.06	0.006036	0.8
1140	19-May-07	2007	D1	0.26	0.14	0.02	0.06	0.006036	0.8
1117	19-May-07	2007	D1	0.26	0.14	0.02	0.06	0.006036	0.8
1094	19-May-07	2007	D1	0.26	0.14	0.02	0.06	0.006036	0.8
1127	19-May-07	2007	D1	0.26	0.14	0.02	0.06	0.006036	0.8
1124	19-May-07	2007	D1	0.26	0.14	0.02	0.06	0.006036	0.8
1125	19-May-07	2007	D1	0.26	0.14	0.02	0.06	0.006036	0.8
1123	19-May-07	2007	D1	0.26	0.14	0.02	0.06	0.006036	0.8
1121	19-May-07	2007	D1	0.26	0.14	0.02	0.06	0.006036	0.8
1135	19-May-07	2007	D1	0.26	0.14	0.02	0.06	0.006036	0.8
1120	19-May-07	2007	D1	0.26	0.14	0.02	0.06	0.006036	0.8

1136	19-May-07	2007	D1	0.26	0.14	0.02	0.06	0.006036	0.8
1115	19-May-07	2007	D1	0.26	0.14	0.02	0.06	0.006036	0.8
1104	19-May-07	2007	D1	0.26	0.14	0.02	0.06	0.006036	0.8
1105	19-May-07	2007	D1	0.26	0.14	0.02	0.06	0.006036	0.8
1102	19-May-07	2007	D1	0.26	0.14	0.02	0.06	0.006036	0.8
1092	19-May-07	2007	D1	0.26	0.14	0.02	0.06	0.006036	0.8
1093	19-May-07	2007	D1	0.26	0.14	0.02	0.06	0.006036	0.8
1119	19-May-07	2007	D1	0.26	0.14	0.02	0.06	0.006036	0.8
2375	22-May-07	2007	HH	0.089552	0.149254	0.104478	0.029851	0	0.615385
2374	22-May-07	2007	HH	0.089552	0.149254	0.104478	0.029851	0	0.615385
2383	22-May-07	2007	HH	0.089552	0.149254	0.104478	0.029851	0	0.615385
2378	22-May-07	2007	HH	0.089552	0.149254	0.104478	0.029851	0	0.615385
2384	22-May-07	2007	HH	0.089552	0.149254	0.104478	0.029851	0	0.615385
2382	22-May-07	2007	HH	0.089552	0.149254	0.104478	0.029851	0	0.615385
2385	22-May-07	2007	HH	0.089552	0.149254	0.104478	0.029851	0	0.615385
2372	22-May-07	2007	HH	0.089552	0.149254	0.104478	0.029851	0	0.615385
2373	22-May-07	2007	HH	0.089552	0.149254	0.104478	0.029851	0	0.615385
2376	22-May-07	2007	HH	0.089552	0.149254	0.104478	0.029851	0	0.615385
2370	22-May-07	2007	HH	0.089552	0.149254	0.104478	0.029851	0	0.615385
2379	22-May-07	2007	HH	0.089552	0.149254	0.104478	0.029851	0	0.615385
2381	22-May-07	2007	HH	0.089552	0.149254	0.104478	0.029851	0	0.615385
2380	22-May-07	2007	HH	0.089552	0.149254	0.104478	0.029851	0	0.615385
2369	22-May-07	2007	HH	0.089552	0.149254	0.104478	0.029851	0	0.615385
2371	22-May-07	2007	HH	0.089552	0.149254	0.104478	0.029851	0	0.615385
2377	22-May-07	2007	HH	0.089552	0.149254	0.104478	0.029851	0	0.615385
2389	23-May-07	2007	SD						
2388	23-May-07	2007	SD						
2390	23-May-07	2007	SD						
2401	23-May-07	2007	SD						
2402	23-May-07	2007	SD						
2403	23-May-07	2007	SD						
2387	23-May-07	2007	SD						
2391	23-May-07	2007	SD						
2400	23-May-07	2007	SD						
2405	23-May-07	2007	SD						
2399	23-May-07	2007	SD						
2404	23-May-07	2007	SD						
2394	23-May-07	2007	SD						
2411	23-May-07	2007	SD						
2412	23-May-07	2007	SD						
2393	23-May-07	2007	SD						
2395	23-May-07	2007	SD						
2409	23-May-07	2007	SD						
2410	23-May-07	2007	SD						

2406	23-May-07	2007	SD
2392	23-May-07	2007	SD
2408	23-May-07	2007	SD
2398	23-May-07	2007	SD
2386	23-May-07	2007	SD
2396	23-May-07	2007	SD
2407	23-May-07	2007	SD
2397	23-May-07	2007	SD
2476	23-May-07	2007	SD
2470	23-May-07	2007	SD
2469	23-May-07	2007	SD
2474	23-May-07	2007	SD
2463	23-May-07	2007	SD
2466	23-May-07	2007	SD
2461	23-May-07	2007	SD
2462	23-May-07	2007	SD
2467	23-May-07	2007	SD
2453	23-May-07	2007	SD
2459	23-May-07	2007	SD
2464	23-May-07	2007	SD
2472	23-May-07	2007	SD
2473	23-May-07	2007	SD
2475	23-May-07	2007	SD
2477	23-May-07	2007	SD
2450	23-May-07	2007	SD
2451	23-May-07	2007	SD
2449	23-May-07	2007	SD
2455	23-May-07	2007	SD
2448	23-May-07	2007	SD
2452	23-May-07	2007	SD
2456	23-May-07	2007	SD
2468	23-May-07	2007	SD
2460	23-May-07	2007	SD
2478	23-May-07	2007	SD
2454	23-May-07	2007	SD
2465	23-May-07	2007	SD
2445	23-May-07	2007	SD
2458	23-May-07	2007	SD
2471	23-May-07	2007	SD
2446	23-May-07	2007	SD
2447	23-May-07	2007	SD
2457	23-May-07	2007	SD
2511	24-May-07	2007	ML
2491	24-May-07	2007	ML

2499	24-May-07	2007	ML						
2500	24-May-07	2007	ML						
2505	24-May-07	2007	ML						
2506	24-May-07	2007	ML						
2509	24-May-07	2007	ML						
2492	24-May-07	2007	ML						
2493	24-May-07	2007	ML						
2498	24-May-07	2007	ML						
2502	24-May-07	2007	ML						
2504	24-May-07	2007	ML						
2507	24-May-07	2007	ML						
2497	24-May-07	2007	ML						
2496	24-May-07	2007	ML						
2510	24-May-07	2007	ML						
2512	24-May-07	2007	ML						
2503	24-May-07	2007	ML						
2494	24-May-07	2007	ML						
2501	24-May-07	2007	ML						
2490	24-May-07	2007	ML						
2495	24-May-07	2007	ML						
2508	24-May-07	2007	ML						
2781	29-May-07	2007	D1	0.26	0.14	0.02	0.06	0.006036	0.8
2782	29-May-07	2007	D1	0.26	0.14	0.02	0.06	0.006036	0.8
2780	29-May-07	2007	D1	0.26	0.14	0.02	0.06	0.006036	0.8
2785	29-May-07	2007	D1	0.26	0.14	0.02	0.06	0.006036	0.8
2788	29-May-07	2007	D1	0.26	0.14	0.02	0.06	0.006036	0.8
2783	29-May-07	2007	D1	0.26	0.14	0.02	0.06	0.006036	0.8
2789	29-May-07	2007	D1	0.26	0.14	0.02	0.06	0.006036	0.8
2790	29-May-07	2007	D1	0.26	0.14	0.02	0.06	0.006036	0.8
2791	29-May-07	2007	D1	0.26	0.14	0.02	0.06	0.006036	0.8
2787	29-May-07	2007	D1	0.26	0.14	0.02	0.06	0.006036	0.8
2786	29-May-07	2007	D1	0.26	0.14	0.02	0.06	0.006036	0.8
2778	29-May-07	2007	D1	0.26	0.14	0.02	0.06	0.006036	0.8
2777	29-May-07	2007	D1	0.26	0.14	0.02	0.06	0.006036	0.8
2776	29-May-07	2007	D1	0.26	0.14	0.02	0.06	0.006036	0.8
2775	29-May-07	2007	D1	0.26	0.14	0.02	0.06	0.006036	0.8
2779	29-May-07	2007	D1	0.26	0.14	0.02	0.06	0.006036	0.8
2784	29-May-07	2007	D1	0.26	0.14	0.02	0.06	0.006036	0.8
2838	30-May-07	2007	MG	0.24	0.28	0.12	0.02	0.009615	6.15
2837	30-May-07	2007	MG	0.24	0.28	0.12	0.02	0.009615	6.15
2842	30-May-07	2007	MG	0.24	0.28	0.12	0.02	0.009615	6.15
2844	30-May-07	2007	MG	0.24	0.28	0.12	0.02	0.009615	6.15
2846	30-May-07	2007	MG	0.24	0.28	0.12	0.02	0.009615	6.15
2853	30-May-07	2007	MG	0.24	0.28	0.12	0.02	0.009615	6.15

2836	30-May-07	2007	MG	0.24	0.28	0.12	0.02	0.009615	6.15
2839	30-May-07	2007	MG	0.24	0.28	0.12	0.02	0.009615	6.15
2840	30-May-07	2007	MG	0.24	0.28	0.12	0.02	0.009615	6.15
2841	30-May-07	2007	MG	0.24	0.28	0.12	0.02	0.009615	6.15
2843	30-May-07	2007	MG	0.24	0.28	0.12	0.02	0.009615	6.15
2847	30-May-07	2007	MG	0.24	0.28	0.12	0.02	0.009615	6.15
2848	30-May-07	2007	MG	0.24	0.28	0.12	0.02	0.009615	6.15
2852	30-May-07	2007	MG	0.24	0.28	0.12	0.02	0.009615	6.15
2849	30-May-07	2007	MG	0.24	0.28	0.12	0.02	0.009615	6.15
2850	30-May-07	2007	MG	0.24	0.28	0.12	0.02	0.009615	6.15
2851	30-May-07	2007	MG	0.24	0.28	0.12	0.02	0.009615	6.15
2835	30-May-07	2007	MG	0.24	0.28	0.12	0.02	0.009615	6.15
2854	30-May-07	2007	MG	0.24	0.28	0.12	0.02	0.009615	6.15
2845	30-May-07	2007	MG	0.24	0.28	0.12	0.02	0.009615	6.15
2855	30-May-07	2007	MG	0.24	0.28	0.12	0.02	0.009615	6.15
2834	30-May-07	2007	MG	0.24	0.28	0.12	0.02	0.009615	6.15
2856	30-May-07	2007	MG	0.24	0.28	0.12	0.02	0.009615	6.15
2857	30-May-07	2007	MG	0.24	0.28	0.12	0.02	0.009615	6.15
	31-May-07	2007	SC	0.136986	0.041096	0.027397	0	0	8.576923
	31-May-07	2007	SC	0.136986	0.041096	0.027397	0	0	8.576923
	31-May-07	2007	SC	0.136986	0.041096	0.027397	0	0	8.576923
	31-May-07	2007	SC	0.136986	0.041096	0.027397	0	0	8.576923
	31-May-07	2007	SC	0.136986	0.041096	0.027397	0	0	8.576923
	31-May-07	2007	SC	0.136986	0.041096	0.027397	0	0	8.576923
	31-May-07	2007	SC	0.136986	0.041096	0.027397	0	0	8.576923
	31-May-07	2007	SC	0.136986	0.041096	0.027397	0	0	8.576923
	31-May-07	2007	SC	0.136986	0.041096	0.027397	0	0	8.576923
	31-May-07	2007	SC	0.136986	0.041096	0.027397	0	0	8.576923
	31-May-07	2007	SC	0.136986	0.041096	0.027397	0	0	8.576923
	31-May-07	2007	SC	0.136986	0.041096	0.027397	0	0	8.576923
	31-May-07	2007	SC	0.136986	0.041096	0.027397	0	0	8.576923
2925	31-May-07	2007	SC	0.136986	0.041096	0.027397	0	0	8.576923
2927	31-May-07	2007	SC	0.136986	0.041096	0.027397	0	0	8.576923
2929	31-May-07	2007	SC	0.136986	0.041096	0.027397	0	0	8.576923
2926	31-May-07	2007	SC	0.136986	0.041096	0.027397	0	0	8.576923
2928	31-May-07	2007	SC	0.136986	0.041096	0.027397	0	0	8.576923
2930	31-May-07	2007	SC	0.136986	0.041096	0.027397	0	0	8.576923
2931	31-May-07	2007	SC	0.136986	0.041096	0.027397	0	0	8.576923
2924	31-May-07	2007	SC	0.136986	0.041096	0.027397	0	0	8.576923
2922	31-May-07	2007	SC	0.136986	0.041096	0.027397	0	0	8.576923
2923	31-May-07	2007	SC	0.136986	0.041096	0.027397	0	0	8.576923
2932	31-May-07	2007	SC	0.136986	0.041096	0.027397	0	0	8.576923
2939	1-Jun-07	2007	RP	0.133333	0.033333	0.133333	0	0.019139	2.75
2937	1-Jun-07	2007	RP	0.133333	0.033333	0.133333	0	0.019139	2.75

2938	1-Jun-07	2007	RP	0.133333	0.033333	0.133333		0	0.019139	2.75
2940	1-Jun-07	2007	RP	0.133333	0.033333	0.133333		0	0.019139	2.75
2941	1-Jun-07	2007	RP	0.133333	0.033333	0.133333		0	0.019139	2.75
2943	1-Jun-07	2007	RP	0.133333	0.033333	0.133333		0	0.019139	2.75
2945	1-Jun-07	2007	RP	0.133333	0.033333	0.133333		0	0.019139	2.75
2946	1-Jun-07	2007	RP	0.133333	0.033333	0.133333		0	0.019139	2.75
2947	1-Jun-07	2007	RP	0.133333	0.033333	0.133333		0	0.019139	2.75
2948	1-Jun-07	2007	RP	0.133333	0.033333	0.133333		0	0.019139	2.75
2936	1-Jun-07	2007	RP	0.133333	0.033333	0.133333		0	0.019139	2.75
2942	1-Jun-07	2007	RP	0.133333	0.033333	0.133333		0	0.019139	2.75
2944	1-Jun-07	2007	RP	0.133333	0.033333	0.133333		0	0.019139	2.75
2971	1-Jun-07	2007	RL	0.157895	0.157895	0		0	0.028571	0.333333
2970	1-Jun-07	2007	RL	0.157895	0.157895	0		0	0.028571	0.333333
2969	1-Jun-07	2007	RL	0.157895	0.157895	0		0	0.028571	0.333333
2968	1-Jun-07	2007	RL	0.157895	0.157895	0		0	0.028571	0.333333
2972	1-Jun-07	2007	RL	0.157895	0.157895	0		0	0.028571	0.333333
2980	1-Jun-07	2007	RL	0.157895	0.157895	0		0	0.028571	0.333333
2961	1-Jun-07	2007	RL	0.157895	0.157895	0		0	0.028571	0.333333
2959	1-Jun-07	2007	RL	0.157895	0.157895	0		0	0.028571	0.333333
2960	1-Jun-07	2007	RL	0.157895	0.157895	0		0	0.028571	0.333333
2963	1-Jun-07	2007	RL	0.157895	0.157895	0		0	0.028571	0.333333
2967	1-Jun-07	2007	RL	0.157895	0.157895	0		0	0.028571	0.333333
2973	1-Jun-07	2007	RL	0.157895	0.157895	0		0	0.028571	0.333333
2975	1-Jun-07	2007	RL	0.157895	0.157895	0		0	0.028571	0.333333
2976	1-Jun-07	2007	RL	0.157895	0.157895	0		0	0.028571	0.333333
2962	1-Jun-07	2007	RL	0.157895	0.157895	0		0	0.028571	0.333333
2964	1-Jun-07	2007	RL	0.157895	0.157895	0		0	0.028571	0.333333
2965	1-Jun-07	2007	RL	0.157895	0.157895	0		0	0.028571	0.333333
2977	1-Jun-07	2007	RL	0.157895	0.157895	0		0	0.028571	0.333333
2978	1-Jun-07	2007	RL	0.157895	0.157895	0		0	0.028571	0.333333
2979	1-Jun-07	2007	RL	0.157895	0.157895	0		0	0.028571	0.333333
2958	1-Jun-07	2007	RL	0.157895	0.157895	0		0	0.028571	0.333333
2966	1-Jun-07	2007	RL	0.157895	0.157895	0		0	0.028571	0.333333
2974	1-Jun-07	2007	RL	0.157895	0.157895	0		0	0.028571	0.333333
3043	2-Jun-07	2007	ML							
3037	2-Jun-07	2007	ML							
3039	2-Jun-07	2007	ML							
3042	2-Jun-07	2007	ML							
3040	2-Jun-07	2007	ML							
3036	2-Jun-07	2007	ML							
3038	2-Jun-07	2007	ML							
3041	2-Jun-07	2007	ML							
	8-Jun-07	2007	ML							
	8-Jun-07	2007	ML							

8-Jun-07	2007	ML						
8-Jun-07	2007	ML						
8-Jun-07	2007	ML						
8-Jun-07	2007	ML						
8-Jun-07	2007	ML						
8-Jun-07	2007	ML						
8-Jun-07	2007	ML						
12-Jun-07	2007	SD						
12-Jun-07	2007	SD						
12-Jun-07	2007	SD						
12-Jun-07	2007	SD						
12-Jun-07	2007	SD						
12-Jun-07	2007	SD						
12-Jun-07	2007	SD						
12-Jun-07	2007	SD						
12-Jun-07	2007	SD						
12-Jun-07	2007	SD						
12-Jun-07	2007	SD						
12-Jun-07	2007	SD						
12-Jun-07	2007	SD						
12-Jun-07	2007	SD						
12-Jun-07	2007	SD						
12-Jun-07	2007	SD						
13-Jun-07	2007	RP	0.133333	0.033333	0.133333	0	0.019139	2.75
13-Jun-07	2007	RP	0.133333	0.033333	0.133333	0	0.019139	2.75
13-Jun-07	2007	RP	0.133333	0.033333	0.133333	0	0.019139	2.75
13-Jun-07	2007	RP	0.133333	0.033333	0.133333	0	0.019139	2.75
13-Jun-07	2007	RP	0.133333	0.033333	0.133333	0	0.019139	2.75
13-Jun-07	2007	RP	0.133333	0.033333	0.133333	0	0.019139	2.75
13-Jun-07	2007	RP	0.133333	0.033333	0.133333	0	0.019139	2.75
13-Jun-07	2007	RP	0.133333	0.033333	0.133333	0	0.019139	2.75
13-Jun-07	2007	RP	0.133333	0.033333	0.133333	0	0.019139	2.75
13-Jun-07	2007	RP	0.133333	0.033333	0.133333	0	0.019139	2.75
13-Jun-07	2007	RP	0.133333	0.033333	0.133333	0	0.019139	2.75
13-Jun-07	2007	RP	0.133333	0.033333	0.133333	0	0.019139	2.75
13-Jun-07	2007	RP	0.133333	0.033333	0.133333	0	0.019139	2.75
13-Jun-07	2007	RP	0.133333	0.033333	0.133333	0	0.019139	2.75
13-Jun-07	2007	RP	0.133333	0.033333	0.133333	0	0.019139	2.75
13-Jun-07	2007	RP	0.133333	0.033333	0.133333	0	0.019139	2.75
13-Jun-07	2007	RP	0.133333	0.033333	0.133333	0	0.019139	2.75
13-Jun-07	2007	RP	0.133333	0.033333	0.133333	0	0.019139	2.75
13-Jun-07	2007	RP	0.133333	0.033333	0.133333	0	0.019139	2.75
13-Jun-07	2007	RP	0.133333	0.033333	0.133333	0	0.019139	2.75
13-Jun-07	2007	RP	0.133333	0.033333	0.133333	0	0.019139	2.75

	13-Jun-07	2007	RP	0.133333	0.033333	0.133333	0	0.019139	2.75
	13-Jun-07	2007	RP	0.133333	0.033333	0.133333	0	0.019139	2.75
	15-Jun-07	2007	SC	0.136986	0.041096	0.027397	0	0	8.576923
	15-Jun-07	2007	SC	0.136986	0.041096	0.027397	0	0	8.576923
	15-Jun-07	2007	SC	0.136986	0.041096	0.027397	0	0	8.576923
	15-Jun-07	2007	SC	0.136986	0.041096	0.027397	0	0	8.576923
2	10-May-08	2008	D1	0.2	0.28	0.04	0.02	0.006036	0.8
6	10-May-08	2008	D1	0.2	0.28	0.04	0.02	0.006036	0.8
7	10-May-08	2008	D1	0.2	0.28	0.04	0.02	0.006036	0.8
8	10-May-08	2008	D1	0.2	0.28	0.04	0.02	0.006036	0.8
9	10-May-08	2008	D1	0.2	0.28	0.04	0.02	0.006036	0.8
3	10-May-08	2008	D1	0.2	0.28	0.04	0.02	0.006036	0.8
4	10-May-08	2008	D1	0.2	0.28	0.04	0.02	0.006036	0.8
5	10-May-08	2008	D1	0.2	0.28	0.04	0.02	0.006036	0.8
10	10-May-08	2008	D1	0.2	0.28	0.04	0.02	0.006036	0.8
11	10-May-08	2008	D1	0.2	0.28	0.04	0.02	0.006036	0.8
1	10-May-08	2008	D1	0.2	0.28	0.04	0.02	0.006036	0.8
28	12-May-08	2008	LT1	0.925926	0.222222	0.037037	0.04	0	3.666667
26	12-May-08	2008	LT1	0.925926	0.222222	0.037037	0.04	0	3.666667
27	12-May-08	2008	LT1	0.925926	0.222222	0.037037	0.04	0	3.666667
22	12-May-08	2008	LT1	0.925926	0.222222	0.037037	0.04	0	3.666667
23	12-May-08	2008	LT1	0.925926	0.222222	0.037037	0.04	0	3.666667
21	12-May-08	2008	LT1	0.925926	0.222222	0.037037	0.04	0	3.666667
20	12-May-08	2008	LT1	0.925926	0.222222	0.037037	0.04	0	3.666667
29	12-May-08	2008	LT1	0.925926	0.222222	0.037037	0.04	0	3.666667
30	12-May-08	2008	LT1	0.925926	0.222222	0.037037	0.04	0	3.666667
31	12-May-08	2008	LT1	0.925926	0.222222	0.037037	0.04	0	3.666667
32	12-May-08	2008	LT1	0.925926	0.222222	0.037037	0.04	0	3.666667
38	12-May-08	2008	LT1	0.925926	0.222222	0.037037	0.04	0	3.666667
39	12-May-08	2008	LT1	0.925926	0.222222	0.037037	0.04	0	3.666667
16	12-May-08	2008	LT1	0.925926	0.222222	0.037037	0.04	0	3.666667
19	12-May-08	2008	LT1	0.925926	0.222222	0.037037	0.04	0	3.666667
24	12-May-08	2008	LT1	0.925926	0.222222	0.037037	0.04	0	3.666667
25	12-May-08	2008	LT1	0.925926	0.222222	0.037037	0.04	0	3.666667
17	12-May-08	2008	LT1	0.925926	0.222222	0.037037	0.04	0	3.666667
37	12-May-08	2008	LT1	0.925926	0.222222	0.037037	0.04	0	3.666667
34	12-May-08	2008	LT1	0.925926	0.222222	0.037037	0.04	0	3.666667
36	12-May-08	2008	LT1	0.925926	0.222222	0.037037	0.04	0	3.666667
13	12-May-08	2008	LT1	0.925926	0.222222	0.037037	0.04	0	3.666667
33	12-May-08	2008	LT1	0.925926	0.222222	0.037037	0.04	0	3.666667
40	12-May-08	2008	LT1	0.925926	0.222222	0.037037	0.04	0	3.666667
15	12-May-08	2008	LT1	0.925926	0.222222	0.037037	0.04	0	3.666667
14	12-May-08	2008	LT1	0.925926	0.222222	0.037037	0.04	0	3.666667
18	12-May-08	2008	LT1	0.925926	0.222222	0.037037	0.04	0	3.666667

35	12-May-08	2008	LT1	0.925926	0.222222	0.037037	0.04	0	3.666667
41	12-May-08	2008	LT1	0.925926	0.222222	0.037037	0.04	0	3.666667
12	12-May-08	2008	LT1	0.925926	0.222222	0.037037	0.04	0	3.666667
67	15-May-08	2008	RP	0.176471	0	0.029412	0.029412	0.015228	3.083333
90	15-May-08	2008	RP	0.176471	0	0.029412	0.029412	0.015228	3.083333
66	15-May-08	2008	RP	0.176471	0	0.029412	0.029412	0.015228	3.083333
65	15-May-08	2008	RP	0.176471	0	0.029412	0.029412	0.015228	3.083333
86	15-May-08	2008	RP	0.176471	0	0.029412	0.029412	0.015228	3.083333
87	15-May-08	2008	RP	0.176471	0	0.029412	0.029412	0.015228	3.083333
89	15-May-08	2008	RP	0.176471	0	0.029412	0.029412	0.015228	3.083333
64	15-May-08	2008	RP	0.176471	0	0.029412	0.029412	0.015228	3.083333
68	15-May-08	2008	RP	0.176471	0	0.029412	0.029412	0.015228	3.083333
69	15-May-08	2008	RP	0.176471	0	0.029412	0.029412	0.015228	3.083333
77	15-May-08	2008	RP	0.176471	0	0.029412	0.029412	0.015228	3.083333
80	15-May-08	2008	RP	0.176471	0	0.029412	0.029412	0.015228	3.083333
84	15-May-08	2008	RP	0.176471	0	0.029412	0.029412	0.015228	3.083333
75	15-May-08	2008	RP	0.176471	0	0.029412	0.029412	0.015228	3.083333
82	15-May-08	2008	RP	0.176471	0	0.029412	0.029412	0.015228	3.083333
85	15-May-08	2008	RP	0.176471	0	0.029412	0.029412	0.015228	3.083333
76	15-May-08	2008	RP	0.176471	0	0.029412	0.029412	0.015228	3.083333
78	15-May-08	2008	RP	0.176471	0	0.029412	0.029412	0.015228	3.083333
79	15-May-08	2008	RP	0.176471	0	0.029412	0.029412	0.015228	3.083333
62	15-May-08	2008	RP	0.176471	0	0.029412	0.029412	0.015228	3.083333
70	15-May-08	2008	RP	0.176471	0	0.029412	0.029412	0.015228	3.083333
91	15-May-08	2008	RP	0.176471	0	0.029412	0.029412	0.015228	3.083333
92	15-May-08	2008	RP	0.176471	0	0.029412	0.029412	0.015228	3.083333
72	15-May-08	2008	RP	0.176471	0	0.029412	0.029412	0.015228	3.083333
73	15-May-08	2008	RP	0.176471	0	0.029412	0.029412	0.015228	3.083333
74	15-May-08	2008	RP	0.176471	0	0.029412	0.029412	0.015228	3.083333
83	15-May-08	2008	RP	0.176471	0	0.029412	0.029412	0.015228	3.083333
63	15-May-08	2008	RP	0.176471	0	0.029412	0.029412	0.015228	3.083333
71	15-May-08	2008	RP	0.176471	0	0.029412	0.029412	0.015228	3.083333
81	15-May-08	2008	RP	0.176471	0	0.029412	0.029412	0.015228	3.083333
93	15-May-08	2008	RP	0.176471	0	0.029412	0.029412	0.015228	3.083333
88	15-May-08	2008	RP	0.176471	0	0.029412	0.029412	0.015228	3.083333
61	15-May-08	2008	RP	0.176471	0	0.029412	0.029412	0.015228	3.083333
286	19-May-08	2008	ML						
290	19-May-08	2008	ML						
288	19-May-08	2008	ML						
289	19-May-08	2008	ML						
294	19-May-08	2008	ML						
295	19-May-08	2008	ML						
296	19-May-08	2008	ML						
299	19-May-08	2008	ML						

287	19-May-08	2008	ML							
292	19-May-08	2008	ML							
297	19-May-08	2008	ML							
298	19-May-08	2008	ML							
300	19-May-08	2008	ML							
303	19-May-08	2008	ML							
291	19-May-08	2008	ML							
302	19-May-08	2008	ML							
301	19-May-08	2008	ML							
282	19-May-08	2008	ML							
284	19-May-08	2008	ML							
283	19-May-08	2008	ML							
285	19-May-08	2008	ML							
293	19-May-08	2008	ML							
304	19-May-08	2008	ML							
281	19-May-08	2008	ML							
597	23-May-08	2008	RL		0.210526	0.131579	0.026316	0.078947	0.018018	0.333333
592	23-May-08	2008	RL		0.210526	0.131579	0.026316	0.078947	0.018018	0.333333
594	23-May-08	2008	RL		0.210526	0.131579	0.026316	0.078947	0.018018	0.333333
593	23-May-08	2008	RL		0.210526	0.131579	0.026316	0.078947	0.018018	0.333333
595	23-May-08	2008	RL		0.210526	0.131579	0.026316	0.078947	0.018018	0.333333
598	23-May-08	2008	RL		0.210526	0.131579	0.026316	0.078947	0.018018	0.333333
601	23-May-08	2008	RL		0.210526	0.131579	0.026316	0.078947	0.018018	0.333333
590	23-May-08	2008	RL		0.210526	0.131579	0.026316	0.078947	0.018018	0.333333
602	23-May-08	2008	RL		0.210526	0.131579	0.026316	0.078947	0.018018	0.333333
596	23-May-08	2008	RL		0.210526	0.131579	0.026316	0.078947	0.018018	0.333333
599	23-May-08	2008	RL		0.210526	0.131579	0.026316	0.078947	0.018018	0.333333
600	23-May-08	2008	RL		0.210526	0.131579	0.026316	0.078947	0.018018	0.333333
604	23-May-08	2008	RL		0.210526	0.131579	0.026316	0.078947	0.018018	0.333333
589	23-May-08	2008	RL		0.210526	0.131579	0.026316	0.078947	0.018018	0.333333
588	23-May-08	2008	RL		0.210526	0.131579	0.026316	0.078947	0.018018	0.333333
591	23-May-08	2008	RL		0.210526	0.131579	0.026316	0.078947	0.018018	0.333333
603	23-May-08	2008	RL		0.210526	0.131579	0.026316	0.078947	0.018018	0.333333
605	23-May-08	2008	RL		0.210526	0.131579	0.026316	0.078947	0.018018	0.333333
587	23-May-08	2008	RL		0.210526	0.131579	0.026316	0.078947	0.018018	0.333333
822	27-May-08	2008	RP		0.176471	0	0.029412	0.029412	0.015228	3.083333
824	27-May-08	2008	RP		0.176471	0	0.029412	0.029412	0.015228	3.083333
820	27-May-08	2008	RP		0.176471	0	0.029412	0.029412	0.015228	3.083333
821	27-May-08	2008	RP		0.176471	0	0.029412	0.029412	0.015228	3.083333
823	27-May-08	2008	RP		0.176471	0	0.029412	0.029412	0.015228	3.083333
825	27-May-08	2008	RP		0.176471	0	0.029412	0.029412	0.015228	3.083333
819	27-May-08	2008	RP		0.176471	0	0.029412	0.029412	0.015228	3.083333
832	27-May-08	2008	RC		0.15	0	0.05	0	0.009346	2.875
828	27-May-08	2008	RC		0.15	0	0.05	0	0.009346	2.875

831	27-May-08	2008	RC	0.15	0	0.05	0	0.009346	2.875
830	27-May-08	2008	RC	0.15	0	0.05	0	0.009346	2.875
827	27-May-08	2008	RC	0.15	0	0.05	0	0.009346	2.875
829	27-May-08	2008	RC	0.15	0	0.05	0	0.009346	2.875
833	27-May-08	2008	RC	0.15	0	0.05	0	0.009346	2.875
834	27-May-08	2008	RC	0.15	0	0.05	0	0.009346	2.875
826	27-May-08	2008	RC	0.15	0	0.05	0	0.009346	2.875
924	29-May-08	2008	LT1	0.925926	0.222222	0.037037	0.04	0	3.666667
932	29-May-08	2008	LT1	0.925926	0.222222	0.037037	0.04	0	3.666667
930	29-May-08	2008	LT1	0.925926	0.222222	0.037037	0.04	0	3.666667
931	29-May-08	2008	LT1	0.925926	0.222222	0.037037	0.04	0	3.666667
933	29-May-08	2008	LT1	0.925926	0.222222	0.037037	0.04	0	3.666667
929	29-May-08	2008	LT1	0.925926	0.222222	0.037037	0.04	0	3.666667
934	29-May-08	2008	LT1	0.925926	0.222222	0.037037	0.04	0	3.666667
935	29-May-08	2008	LT1	0.925926	0.222222	0.037037	0.04	0	3.666667
925	29-May-08	2008	LT1	0.925926	0.222222	0.037037	0.04	0	3.666667
926	29-May-08	2008	LT1	0.925926	0.222222	0.037037	0.04	0	3.666667
927	29-May-08	2008	LT1	0.925926	0.222222	0.037037	0.04	0	3.666667
928	29-May-08	2008	LT1	0.925926	0.222222	0.037037	0.04	0	3.666667
937	29-May-08	2008	LT1	0.925926	0.222222	0.037037	0.04	0	3.666667
938	29-May-08	2008	LT1	0.925926	0.222222	0.037037	0.04	0	3.666667
941	29-May-08	2008	LT1	0.925926	0.222222	0.037037	0.04	0	3.666667
942	29-May-08	2008	LT1	0.925926	0.222222	0.037037	0.04	0	3.666667
944	29-May-08	2008	LT1	0.925926	0.222222	0.037037	0.04	0	3.666667
940	29-May-08	2008	LT1	0.925926	0.222222	0.037037	0.04	0	3.666667
943	29-May-08	2008	LT1	0.925926	0.222222	0.037037	0.04	0	3.666667
936	29-May-08	2008	LT1	0.925926	0.222222	0.037037	0.04	0	3.666667
923	29-May-08	2008	LT1	0.925926	0.222222	0.037037	0.04	0	3.666667
939	29-May-08	2008	LT1	0.925926	0.222222	0.037037	0.04	0	3.666667
945	29-May-08	2008	LT1	0.925926	0.222222	0.037037	0.04	0	3.666667
946	29-May-08	2008	LT1	0.925926	0.222222	0.037037	0.04	0	3.666667
922	29-May-08	2008	LT1	0.925926	0.222222	0.037037	0.04	0	3.666667
975	30-May-08	2008	ML						
976	30-May-08	2008	ML						
980	30-May-08	2008	ML						
982	30-May-08	2008	ML						
977	30-May-08	2008	ML						
972	30-May-08	2008	ML						
973	30-May-08	2008	ML						
971	30-May-08	2008	ML						
974	30-May-08	2008	ML						
983	30-May-08	2008	ML						
978	30-May-08	2008	ML						
984	30-May-08	2008	ML						

985	30-May-08	2008	ML						
965	30-May-08	2008	ML						
966	30-May-08	2008	ML						
968	30-May-08	2008	ML						
988	30-May-08	2008	ML						
964	30-May-08	2008	ML						
987	30-May-08	2008	ML						
969	30-May-08	2008	ML						
979	30-May-08	2008	ML						
986	30-May-08	2008	ML						
962	30-May-08	2008	ML						
963	30-May-08	2008	ML						
970	30-May-08	2008	ML						
981	30-May-08	2008	ML						
989	30-May-08	2008	ML						
967	30-May-08	2008	ML						
961	30-May-08	2008	ML						
1139	3-Jun-08	2008	D1		0.2	0.28	0.04	0.02	0.006036	0.8
1131	3-Jun-08	2008	D1		0.2	0.28	0.04	0.02	0.006036	0.8
1130	3-Jun-08	2008	D1		0.2	0.28	0.04	0.02	0.006036	0.8
1132	3-Jun-08	2008	D1		0.2	0.28	0.04	0.02	0.006036	0.8
1133	3-Jun-08	2008	D1		0.2	0.28	0.04	0.02	0.006036	0.8
1134	3-Jun-08	2008	D1		0.2	0.28	0.04	0.02	0.006036	0.8
1141	3-Jun-08	2008	D1		0.2	0.28	0.04	0.02	0.006036	0.8
1129	3-Jun-08	2008	D1		0.2	0.28	0.04	0.02	0.006036	0.8
1136	3-Jun-08	2008	D1		0.2	0.28	0.04	0.02	0.006036	0.8
1126	3-Jun-08	2008	D1		0.2	0.28	0.04	0.02	0.006036	0.8
1124	3-Jun-08	2008	D1		0.2	0.28	0.04	0.02	0.006036	0.8
1144	3-Jun-08	2008	D1		0.2	0.28	0.04	0.02	0.006036	0.8
1145	3-Jun-08	2008	D1		0.2	0.28	0.04	0.02	0.006036	0.8
1147	3-Jun-08	2008	D1		0.2	0.28	0.04	0.02	0.006036	0.8
1148	3-Jun-08	2008	D1		0.2	0.28	0.04	0.02	0.006036	0.8
1120	3-Jun-08	2008	D1		0.2	0.28	0.04	0.02	0.006036	0.8
1121	3-Jun-08	2008	D1		0.2	0.28	0.04	0.02	0.006036	0.8
1116	3-Jun-08	2008	D1		0.2	0.28	0.04	0.02	0.006036	0.8
1119	3-Jun-08	2008	D1		0.2	0.28	0.04	0.02	0.006036	0.8
1118	3-Jun-08	2008	D1		0.2	0.28	0.04	0.02	0.006036	0.8
1117	3-Jun-08	2008	D1		0.2	0.28	0.04	0.02	0.006036	0.8
1142	3-Jun-08	2008	D1		0.2	0.28	0.04	0.02	0.006036	0.8
1137	3-Jun-08	2008	D1		0.2	0.28	0.04	0.02	0.006036	0.8
1109	3-Jun-08	2008	D1		0.2	0.28	0.04	0.02	0.006036	0.8
1110	3-Jun-08	2008	D1		0.2	0.28	0.04	0.02	0.006036	0.8
1107	3-Jun-08	2008	D1		0.2	0.28	0.04	0.02	0.006036	0.8
1114	3-Jun-08	2008	D1		0.2	0.28	0.04	0.02	0.006036	0.8

1115	3-Jun-08	2008	D1	0.2	0.28	0.04	0.02	0.006036	0.8
1135	3-Jun-08	2008	D1	0.2	0.28	0.04	0.02	0.006036	0.8
1106	3-Jun-08	2008	D1	0.2	0.28	0.04	0.02	0.006036	0.8
1108	3-Jun-08	2008	D1	0.2	0.28	0.04	0.02	0.006036	0.8
1111	3-Jun-08	2008	D1	0.2	0.28	0.04	0.02	0.006036	0.8
1112	3-Jun-08	2008	D1	0.2	0.28	0.04	0.02	0.006036	0.8
1105	3-Jun-08	2008	D1	0.2	0.28	0.04	0.02	0.006036	0.8
1123	3-Jun-08	2008	D1	0.2	0.28	0.04	0.02	0.006036	0.8
1125	3-Jun-08	2008	D1	0.2	0.28	0.04	0.02	0.006036	0.8
1143	3-Jun-08	2008	D1	0.2	0.28	0.04	0.02	0.006036	0.8
1127	3-Jun-08	2008	D1	0.2	0.28	0.04	0.02	0.006036	0.8
1138	3-Jun-08	2008	D1	0.2	0.28	0.04	0.02	0.006036	0.8
1146	3-Jun-08	2008	D1	0.2	0.28	0.04	0.02	0.006036	0.8
1128	3-Jun-08	2008	D1	0.2	0.28	0.04	0.02	0.006036	0.8
1113	3-Jun-08	2008	D1	0.2	0.28	0.04	0.02	0.006036	0.8
1104	3-Jun-08	2008	D1	0.2	0.28	0.04	0.02	0.006036	0.8
1122	3-Jun-08	2008	D1	0.2	0.28	0.04	0.02	0.006036	0.8
1140	3-Jun-08	2008	D1	0.2	0.28	0.04	0.02	0.006036	0.8
1149	3-Jun-08	2008	D1	0.2	0.28	0.04	0.02	0.006036	0.8
1103	3-Jun-08	2008	D1	0.2	0.28	0.04	0.02	0.006036	0.8
1232	7-Jun-08	2008	LT1	0.925926	0.222222	0.037037	0.04	0	3.666667
1241	7-Jun-08	2008	LT1	0.925926	0.222222	0.037037	0.04	0	3.666667
1243	7-Jun-08	2008	LT1	0.925926	0.222222	0.037037	0.04	0	3.666667
1233	7-Jun-08	2008	LT1	0.925926	0.222222	0.037037	0.04	0	3.666667
1235	7-Jun-08	2008	LT1	0.925926	0.222222	0.037037	0.04	0	3.666667
1240	7-Jun-08	2008	LT1	0.925926	0.222222	0.037037	0.04	0	3.666667
1231	7-Jun-08	2008	LT1	0.925926	0.222222	0.037037	0.04	0	3.666667
1237	7-Jun-08	2008	LT1	0.925926	0.222222	0.037037	0.04	0	3.666667
1242	7-Jun-08	2008	LT1	0.925926	0.222222	0.037037	0.04	0	3.666667
1230	7-Jun-08	2008	LT1	0.925926	0.222222	0.037037	0.04	0	3.666667
1236	7-Jun-08	2008	LT1	0.925926	0.222222	0.037037	0.04	0	3.666667
1238	7-Jun-08	2008	LT1	0.925926	0.222222	0.037037	0.04	0	3.666667
1229	7-Jun-08	2008	LT1	0.925926	0.222222	0.037037	0.04	0	3.666667
1234	7-Jun-08	2008	LT1	0.925926	0.222222	0.037037	0.04	0	3.666667
1239	7-Jun-08	2008	LT1	0.925926	0.222222	0.037037	0.04	0	3.666667
1244	7-Jun-08	2008	LT1	0.925926	0.222222	0.037037	0.04	0	3.666667
1228	7-Jun-08	2008	LT1	0.925926	0.222222	0.037037	0.04	0	3.666667
1285	7-Jun-08	2008	LT1	0.925926	0.222222	0.037037	0.04	0	3.666667
1289	7-Jun-08	2008	LT1	0.925926	0.222222	0.037037	0.04	0	3.666667
1290	7-Jun-08	2008	LT1	0.925926	0.222222	0.037037	0.04	0	3.666667
1291	7-Jun-08	2008	LT1	0.925926	0.222222	0.037037	0.04	0	3.666667
1293	7-Jun-08	2008	LT1	0.925926	0.222222	0.037037	0.04	0	3.666667
1295	7-Jun-08	2008	LT1	0.925926	0.222222	0.037037	0.04	0	3.666667
1294	7-Jun-08	2008	LT1	0.925926	0.222222	0.037037	0.04	0	3.666667

1284	7-Jun-08	2008	LT1	0.925926	0.222222	0.037037	0.04	0	3.666667
1288	7-Jun-08	2008	LT1	0.925926	0.222222	0.037037	0.04	0	3.666667
1296	7-Jun-08	2008	LT1	0.925926	0.222222	0.037037	0.04	0	3.666667
1286	7-Jun-08	2008	LT1	0.925926	0.222222	0.037037	0.04	0	3.666667
1287	7-Jun-08	2008	LT1	0.925926	0.222222	0.037037	0.04	0	3.666667
1292	7-Jun-08	2008	LT1	0.925926	0.222222	0.037037	0.04	0	3.666667
1297	7-Jun-08	2008	LT1	0.925926	0.222222	0.037037	0.04	0	3.666667
1298	7-Jun-08	2008	LT1	0.925926	0.222222	0.037037	0.04	0	3.666667
1306	7-Jun-08	2008	LT1	0.925926	0.222222	0.037037	0.04	0	3.666667
1301	7-Jun-08	2008	LT1	0.925926	0.222222	0.037037	0.04	0	3.666667
1299	7-Jun-08	2008	LT1	0.925926	0.222222	0.037037	0.04	0	3.666667
1308	7-Jun-08	2008	LT1	0.925926	0.222222	0.037037	0.04	0	3.666667
1302	7-Jun-08	2008	LT1	0.925926	0.222222	0.037037	0.04	0	3.666667
1307	7-Jun-08	2008	LT1	0.925926	0.222222	0.037037	0.04	0	3.666667
1305	7-Jun-08	2008	LT1	0.925926	0.222222	0.037037	0.04	0	3.666667
1304	7-Jun-08	2008	LT1	0.925926	0.222222	0.037037	0.04	0	3.666667
1283	7-Jun-08	2008	LT1	0.925926	0.222222	0.037037	0.04	0	3.666667
1300	7-Jun-08	2008	LT1	0.925926	0.222222	0.037037	0.04	0	3.666667
1303	7-Jun-08	2008	LT1	0.925926	0.222222	0.037037	0.04	0	3.666667
1309	7-Jun-08	2008	LT1	0.925926	0.222222	0.037037	0.04	0	3.666667
1282	7-Jun-08	2008	LT1	0.925926	0.222222	0.037037	0.04	0	3.666667
1600	15-Jun-08	2008	LT1	0.925926	0.222222	0.037037	0.04	0	3.666667
1607	15-Jun-08	2008	LT1	0.925926	0.222222	0.037037	0.04	0	3.666667
1608	15-Jun-08	2008	LT1	0.925926	0.222222	0.037037	0.04	0	3.666667
1609	15-Jun-08	2008	LT1	0.925926	0.222222	0.037037	0.04	0	3.666667
1610	15-Jun-08	2008	LT1	0.925926	0.222222	0.037037	0.04	0	3.666667
1611	15-Jun-08	2008	LT1	0.925926	0.222222	0.037037	0.04	0	3.666667
1612	15-Jun-08	2008	LT1	0.925926	0.222222	0.037037	0.04	0	3.666667
1613	15-Jun-08	2008	LT1	0.925926	0.222222	0.037037	0.04	0	3.666667
1614	15-Jun-08	2008	LT1	0.925926	0.222222	0.037037	0.04	0	3.666667
1616	15-Jun-08	2008	LT1	0.925926	0.222222	0.037037	0.04	0	3.666667
1618	15-Jun-08	2008	LT1	0.925926	0.222222	0.037037	0.04	0	3.666667
1619	15-Jun-08	2008	LT1	0.925926	0.222222	0.037037	0.04	0	3.666667
1620	15-Jun-08	2008	LT1	0.925926	0.222222	0.037037	0.04	0	3.666667
1621	15-Jun-08	2008	LT1	0.925926	0.222222	0.037037	0.04	0	3.666667
1622	15-Jun-08	2008	LT1	0.925926	0.222222	0.037037	0.04	0	3.666667
1623	15-Jun-08	2008	LT1	0.925926	0.222222	0.037037	0.04	0	3.666667
1624	15-Jun-08	2008	LT1	0.925926	0.222222	0.037037	0.04	0	3.666667
1599	15-Jun-08	2008	LT1	0.925926	0.222222	0.037037	0.04	0	3.666667
1617	15-Jun-08	2008	LT1	0.925926	0.222222	0.037037	0.04	0	3.666667
1601	15-Jun-08	2008	LT1	0.925926	0.222222	0.037037	0.04	0	3.666667
1604	15-Jun-08	2008	LT1	0.925926	0.222222	0.037037	0.04	0	3.666667
1603	15-Jun-08	2008	LT1	0.925926	0.222222	0.037037	0.04	0	3.666667
1605	15-Jun-08	2008	LT1	0.925926	0.222222	0.037037	0.04	0	3.666667

1602	15-Jun-08	2008	LT1	0.925926	0.222222	0.037037	0.04	0	3.666667
1598	15-Jun-08	2008	LT1	0.925926	0.222222	0.037037	0.04	0	3.666667
1606	15-Jun-08	2008	LT1	0.925926	0.222222	0.037037	0.04	0	3.666667
1615	15-Jun-08	2008	LT1	0.925926	0.222222	0.037037	0.04	0	3.666667
1625	15-Jun-08	2008	LT1	0.925926	0.222222	0.037037	0.04	0	3.666667
1597	15-Jun-08	2008	LT1	0.925926	0.222222	0.037037	0.04	0	3.666667
1680	16-Jun-08	2008	ML						
1683	16-Jun-08	2008	ML						
1684	16-Jun-08	2008	ML						
1686	16-Jun-08	2008	ML						
1687	16-Jun-08	2008	ML						
1688	16-Jun-08	2008	ML						
1689	16-Jun-08	2008	ML						
1691	16-Jun-08	2008	ML						
1692	16-Jun-08	2008	ML						
1696	16-Jun-08	2008	ML						
1697	16-Jun-08	2008	ML						
1701	16-Jun-08	2008	ML						
1702	16-Jun-08	2008	ML						
1703	16-Jun-08	2008	ML						
1707	16-Jun-08	2008	ML						
1699	16-Jun-08	2008	ML						
1700	16-Jun-08	2008	ML						
1706	16-Jun-08	2008	ML						
1695	16-Jun-08	2008	ML						
1705	16-Jun-08	2008	ML						
1681	16-Jun-08	2008	ML						
1698	16-Jun-08	2008	ML						
1694	16-Jun-08	2008	ML						
1690	16-Jun-08	2008	ML						
1679	16-Jun-08	2008	ML						
1704	16-Jun-08	2008	ML						
1685	16-Jun-08	2008	ML						
1678	16-Jun-08	2008	ML						
1682	16-Jun-08	2008	ML						
1693	16-Jun-08	2008	ML						
1708	16-Jun-08	2008	ML						
1677	16-Jun-08	2008	ML						
1752	17-Jun-08	2008	RP	0.176471	0	0.029412	0.029412	0.015228	3.083333
1753	17-Jun-08	2008	RP	0.176471	0	0.029412	0.029412	0.015228	3.083333
1761	17-Jun-08	2008	RP	0.176471	0	0.029412	0.029412	0.015228	3.083333
1762	17-Jun-08	2008	RP	0.176471	0	0.029412	0.029412	0.015228	3.083333
1764	17-Jun-08	2008	RP	0.176471	0	0.029412	0.029412	0.015228	3.083333
1766	17-Jun-08	2008	RP	0.176471	0	0.029412	0.029412	0.015228	3.083333

1771	17-Jun-08	2008	RP	0.176471	0	0.029412	0.029412	0.015228	3.083333
1772	17-Jun-08	2008	RP	0.176471	0	0.029412	0.029412	0.015228	3.083333
1774	17-Jun-08	2008	RP	0.176471	0	0.029412	0.029412	0.015228	3.083333
1777	17-Jun-08	2008	RP	0.176471	0	0.029412	0.029412	0.015228	3.083333
1778	17-Jun-08	2008	RP	0.176471	0	0.029412	0.029412	0.015228	3.083333
1763	17-Jun-08	2008	RP	0.176471	0	0.029412	0.029412	0.015228	3.083333
1768	17-Jun-08	2008	RP	0.176471	0	0.029412	0.029412	0.015228	3.083333
1756	17-Jun-08	2008	RP	0.176471	0	0.029412	0.029412	0.015228	3.083333
1757	17-Jun-08	2008	RP	0.176471	0	0.029412	0.029412	0.015228	3.083333
1755	17-Jun-08	2008	RP	0.176471	0	0.029412	0.029412	0.015228	3.083333
1767	17-Jun-08	2008	RP	0.176471	0	0.029412	0.029412	0.015228	3.083333
1773	17-Jun-08	2008	RP	0.176471	0	0.029412	0.029412	0.015228	3.083333
1776	17-Jun-08	2008	RP	0.176471	0	0.029412	0.029412	0.015228	3.083333
1758	17-Jun-08	2008	RP	0.176471	0	0.029412	0.029412	0.015228	3.083333
1769	17-Jun-08	2008	RP	0.176471	0	0.029412	0.029412	0.015228	3.083333
1770	17-Jun-08	2008	RP	0.176471	0	0.029412	0.029412	0.015228	3.083333
1759	17-Jun-08	2008	RP	0.176471	0	0.029412	0.029412	0.015228	3.083333
1760	17-Jun-08	2008	RP	0.176471	0	0.029412	0.029412	0.015228	3.083333
1754	17-Jun-08	2008	RP	0.176471	0	0.029412	0.029412	0.015228	3.083333
1765	17-Jun-08	2008	RP	0.176471	0	0.029412	0.029412	0.015228	3.083333
1775	17-Jun-08	2008	RP	0.176471	0	0.029412	0.029412	0.015228	3.083333
1779	17-Jun-08	2008	RP	0.176471	0	0.029412	0.029412	0.015228	3.083333
1751	17-Jun-08	2008	RP	0.176471	0	0.029412	0.029412	0.015228	3.083333
1798	17-Jun-08	2008	RP	0.176471	0	0.029412	0.029412	0.015228	3.083333
1802	17-Jun-08	2008	RP	0.176471	0	0.029412	0.029412	0.015228	3.083333
1803	17-Jun-08	2008	RP	0.176471	0	0.029412	0.029412	0.015228	3.083333
1800	17-Jun-08	2008	RP	0.176471	0	0.029412	0.029412	0.015228	3.083333
1804	17-Jun-08	2008	RP	0.176471	0	0.029412	0.029412	0.015228	3.083333
1799	17-Jun-08	2008	RP	0.176471	0	0.029412	0.029412	0.015228	3.083333
1797	17-Jun-08	2008	RP	0.176471	0	0.029412	0.029412	0.015228	3.083333
1801	17-Jun-08	2008	RP	0.176471	0	0.029412	0.029412	0.015228	3.083333
1805	17-Jun-08	2008	RP	0.176471	0	0.029412	0.029412	0.015228	3.083333
1806	17-Jun-08	2008	RP	0.176471	0	0.029412	0.029412	0.015228	3.083333
1796	17-Jun-08	2008	RP	0.176471	0	0.029412	0.029412	0.015228	3.083333
2220	17-Jun-08	2008	RC	0.15	0	0.05	0	0.009346	2.875
2224	17-Jun-08	2008	RC	0.15	0	0.05	0	0.009346	2.875
2225	17-Jun-08	2008	RC	0.15	0	0.05	0	0.009346	2.875
2209	17-Jun-08	2008	RC	0.15	0	0.05	0	0.009346	2.875
2226	17-Jun-08	2008	RC	0.15	0	0.05	0	0.009346	2.875
2227	17-Jun-08	2008	RC	0.15	0	0.05	0	0.009346	2.875
2203	17-Jun-08	2008	RC	0.15	0	0.05	0	0.009346	2.875
2205	17-Jun-08	2008	RC	0.15	0	0.05	0	0.009346	2.875
2206	17-Jun-08	2008	RC	0.15	0	0.05	0	0.009346	2.875
2210	17-Jun-08	2008	RC	0.15	0	0.05	0	0.009346	2.875

2215	17-Jun-08	2008	RC	0.15	0	0.05	0	0.009346	2.875
2216	17-Jun-08	2008	RC	0.15	0	0.05	0	0.009346	2.875
2223	17-Jun-08	2008	RC	0.15	0	0.05	0	0.009346	2.875
2228	17-Jun-08	2008	RC	0.15	0	0.05	0	0.009346	2.875
2229	17-Jun-08	2008	RC	0.15	0	0.05	0	0.009346	2.875
2211	17-Jun-08	2008	RC	0.15	0	0.05	0	0.009346	2.875
2214	17-Jun-08	2008	RC	0.15	0	0.05	0	0.009346	2.875
2219	17-Jun-08	2008	RC	0.15	0	0.05	0	0.009346	2.875
2222	17-Jun-08	2008	RC	0.15	0	0.05	0	0.009346	2.875
2217	17-Jun-08	2008	RC	0.15	0	0.05	0	0.009346	2.875
2212	17-Jun-08	2008	RC	0.15	0	0.05	0	0.009346	2.875
2221	17-Jun-08	2008	RC	0.15	0	0.05	0	0.009346	2.875
2213	17-Jun-08	2008	RC	0.15	0	0.05	0	0.009346	2.875
2204	17-Jun-08	2008	RC	0.15	0	0.05	0	0.009346	2.875
2208	17-Jun-08	2008	RC	0.15	0	0.05	0	0.009346	2.875
2202	17-Jun-08	2008	RC	0.15	0	0.05	0	0.009346	2.875
2207	17-Jun-08	2008	RC	0.15	0	0.05	0	0.009346	2.875
2218	17-Jun-08	2008	RC	0.15	0	0.05	0	0.009346	2.875
2164	20-Jun-08	2008	D1	0.2	0.28	0.04	0.02	0.006036	0.8
2173	20-Jun-08	2008	D1	0.2	0.28	0.04	0.02	0.006036	0.8
2166	20-Jun-08	2008	D1	0.2	0.28	0.04	0.02	0.006036	0.8
2168	20-Jun-08	2008	D1	0.2	0.28	0.04	0.02	0.006036	0.8
2171	20-Jun-08	2008	D1	0.2	0.28	0.04	0.02	0.006036	0.8
2174	20-Jun-08	2008	D1	0.2	0.28	0.04	0.02	0.006036	0.8
2162	20-Jun-08	2008	D1	0.2	0.28	0.04	0.02	0.006036	0.8
2140	20-Jun-08	2008	D1	0.2	0.28	0.04	0.02	0.006036	0.8
2144	20-Jun-08	2008	D1	0.2	0.28	0.04	0.02	0.006036	0.8
2154	20-Jun-08	2008	D1	0.2	0.28	0.04	0.02	0.006036	0.8
2167	20-Jun-08	2008	D1	0.2	0.28	0.04	0.02	0.006036	0.8
2170	20-Jun-08	2008	D1	0.2	0.28	0.04	0.02	0.006036	0.8
2176	20-Jun-08	2008	D1	0.2	0.28	0.04	0.02	0.006036	0.8
2177	20-Jun-08	2008	D1	0.2	0.28	0.04	0.02	0.006036	0.8
2157	20-Jun-08	2008	D1	0.2	0.28	0.04	0.02	0.006036	0.8
2169	20-Jun-08	2008	D1	0.2	0.28	0.04	0.02	0.006036	0.8
2139	20-Jun-08	2008	D1	0.2	0.28	0.04	0.02	0.006036	0.8
2141	20-Jun-08	2008	D1	0.2	0.28	0.04	0.02	0.006036	0.8
2142	20-Jun-08	2008	D1	0.2	0.28	0.04	0.02	0.006036	0.8
2143	20-Jun-08	2008	D1	0.2	0.28	0.04	0.02	0.006036	0.8
2156	20-Jun-08	2008	D1	0.2	0.28	0.04	0.02	0.006036	0.8
2158	20-Jun-08	2008	D1	0.2	0.28	0.04	0.02	0.006036	0.8
2159	20-Jun-08	2008	D1	0.2	0.28	0.04	0.02	0.006036	0.8
2163	20-Jun-08	2008	D1	0.2	0.28	0.04	0.02	0.006036	0.8
2165	20-Jun-08	2008	D1	0.2	0.28	0.04	0.02	0.006036	0.8
2153	20-Jun-08	2008	D1	0.2	0.28	0.04	0.02	0.006036	0.8

2151	20-Jun-08	2008	D1	0.2	0.28	0.04	0.02	0.006036	0.8
2161	20-Jun-08	2008	D1	0.2	0.28	0.04	0.02	0.006036	0.8
2152	20-Jun-08	2008	D1	0.2	0.28	0.04	0.02	0.006036	0.8
2148	20-Jun-08	2008	D1	0.2	0.28	0.04	0.02	0.006036	0.8
2149	20-Jun-08	2008	D1	0.2	0.28	0.04	0.02	0.006036	0.8
2147	20-Jun-08	2008	D1	0.2	0.28	0.04	0.02	0.006036	0.8
2146	20-Jun-08	2008	D1	0.2	0.28	0.04	0.02	0.006036	0.8
2150	20-Jun-08	2008	D1	0.2	0.28	0.04	0.02	0.006036	0.8
2175	20-Jun-08	2008	D1	0.2	0.28	0.04	0.02	0.006036	0.8
2155	20-Jun-08	2008	D1	0.2	0.28	0.04	0.02	0.006036	0.8
2172	20-Jun-08	2008	D1	0.2	0.28	0.04	0.02	0.006036	0.8
2138	20-Jun-08	2008	D1	0.2	0.28	0.04	0.02	0.006036	0.8
2145	20-Jun-08	2008	D1	0.2	0.28	0.04	0.02	0.006036	0.8
2160	20-Jun-08	2008	D1	0.2	0.28	0.04	0.02	0.006036	0.8
2178	20-Jun-08	2008	D1	0.2	0.28	0.04	0.02	0.006036	0.8
2137	20-Jun-08	2008	D1	0.2	0.28	0.04	0.02	0.006036	0.8
2255	24-Jun-08	2008	SC	0.191781	0.013699	0.123288	0	0	8.615385
2254	24-Jun-08	2008	SC	0.191781	0.013699	0.123288	0	0	8.615385
2238	24-Jun-08	2008	SC	0.191781	0.013699	0.123288	0	0	8.615385
2244	24-Jun-08	2008	SC	0.191781	0.013699	0.123288	0	0	8.615385
2245	24-Jun-08	2008	SC	0.191781	0.013699	0.123288	0	0	8.615385
2234	24-Jun-08	2008	SC	0.191781	0.013699	0.123288	0	0	8.615385
2239	24-Jun-08	2008	SC	0.191781	0.013699	0.123288	0	0	8.615385
2250	24-Jun-08	2008	SC	0.191781	0.013699	0.123288	0	0	8.615385
2252	24-Jun-08	2008	SC	0.191781	0.013699	0.123288	0	0	8.615385
2256	24-Jun-08	2008	SC	0.191781	0.013699	0.123288	0	0	8.615385
2243	24-Jun-08	2008	SC	0.191781	0.013699	0.123288	0	0	8.615385
2257	24-Jun-08	2008	SC	0.191781	0.013699	0.123288	0	0	8.615385
2246	24-Jun-08	2008	SC	0.191781	0.013699	0.123288	0	0	8.615385
2235	24-Jun-08	2008	SC	0.191781	0.013699	0.123288	0	0	8.615385
2237	24-Jun-08	2008	SC	0.191781	0.013699	0.123288	0	0	8.615385
2242	24-Jun-08	2008	SC	0.191781	0.013699	0.123288	0	0	8.615385
2247	24-Jun-08	2008	SC	0.191781	0.013699	0.123288	0	0	8.615385
2248	24-Jun-08	2008	SC	0.191781	0.013699	0.123288	0	0	8.615385
2249	24-Jun-08	2008	SC	0.191781	0.013699	0.123288	0	0	8.615385
2253	24-Jun-08	2008	SC	0.191781	0.013699	0.123288	0	0	8.615385
2236	24-Jun-08	2008	SC	0.191781	0.013699	0.123288	0	0	8.615385
2240	24-Jun-08	2008	SC	0.191781	0.013699	0.123288	0	0	8.615385
2233	24-Jun-08	2008	SC	0.191781	0.013699	0.123288	0	0	8.615385
2241	24-Jun-08	2008	SC	0.191781	0.013699	0.123288	0	0	8.615385
2251	24-Jun-08	2008	SC	0.191781	0.013699	0.123288	0	0	8.615385
2294	24-Jun-08	2008	RP	0.176471	0	0.029412	0.029412	0.015228	3.083333
2304	24-Jun-08	2008	RP	0.176471	0	0.029412	0.029412	0.015228	3.083333
2310	24-Jun-08	2008	RP	0.176471	0	0.029412	0.029412	0.015228	3.083333

2309	24-Jun-08	2008	RP	0.176471	0	0.029412	0.029412	0.015228	3.083333
2307	24-Jun-08	2008	RP	0.176471	0	0.029412	0.029412	0.015228	3.083333
2308	24-Jun-08	2008	RP	0.176471	0	0.029412	0.029412	0.015228	3.083333
2306	24-Jun-08	2008	RP	0.176471	0	0.029412	0.029412	0.015228	3.083333
2311	24-Jun-08	2008	RP	0.176471	0	0.029412	0.029412	0.015228	3.083333
2312	24-Jun-08	2008	RP	0.176471	0	0.029412	0.029412	0.015228	3.083333
2305	24-Jun-08	2008	RP	0.176471	0	0.029412	0.029412	0.015228	3.083333
2313	24-Jun-08	2008	RP	0.176471	0	0.029412	0.029412	0.015228	3.083333
2282	24-Jun-08	2008	RP	0.176471	0	0.029412	0.029412	0.015228	3.083333
2290	24-Jun-08	2008	RP	0.176471	0	0.029412	0.029412	0.015228	3.083333
2291	24-Jun-08	2008	RP	0.176471	0	0.029412	0.029412	0.015228	3.083333
2297	24-Jun-08	2008	RP	0.176471	0	0.029412	0.029412	0.015228	3.083333
2293	24-Jun-08	2008	RP	0.176471	0	0.029412	0.029412	0.015228	3.083333
2298	24-Jun-08	2008	RP	0.176471	0	0.029412	0.029412	0.015228	3.083333
2314	24-Jun-08	2008	RP	0.176471	0	0.029412	0.029412	0.015228	3.083333
2288	24-Jun-08	2008	RP	0.176471	0	0.029412	0.029412	0.015228	3.083333
2281	24-Jun-08	2008	RP	0.176471	0	0.029412	0.029412	0.015228	3.083333
2303	24-Jun-08	2008	RP	0.176471	0	0.029412	0.029412	0.015228	3.083333
2302	24-Jun-08	2008	RP	0.176471	0	0.029412	0.029412	0.015228	3.083333
2299	24-Jun-08	2008	RP	0.176471	0	0.029412	0.029412	0.015228	3.083333
2315	24-Jun-08	2008	RP	0.176471	0	0.029412	0.029412	0.015228	3.083333
2279	24-Jun-08	2008	RP	0.176471	0	0.029412	0.029412	0.015228	3.083333
2283	24-Jun-08	2008	RP	0.176471	0	0.029412	0.029412	0.015228	3.083333
2284	24-Jun-08	2008	RP	0.176471	0	0.029412	0.029412	0.015228	3.083333
2285	24-Jun-08	2008	RP	0.176471	0	0.029412	0.029412	0.015228	3.083333
2286	24-Jun-08	2008	RP	0.176471	0	0.029412	0.029412	0.015228	3.083333
2280	24-Jun-08	2008	RP	0.176471	0	0.029412	0.029412	0.015228	3.083333
2289	24-Jun-08	2008	RP	0.176471	0	0.029412	0.029412	0.015228	3.083333
2292	24-Jun-08	2008	RP	0.176471	0	0.029412	0.029412	0.015228	3.083333
2296	24-Jun-08	2008	RP	0.176471	0	0.029412	0.029412	0.015228	3.083333
2295	24-Jun-08	2008	RP	0.176471	0	0.029412	0.029412	0.015228	3.083333
2301	24-Jun-08	2008	RP	0.176471	0	0.029412	0.029412	0.015228	3.083333
2278	24-Jun-08	2008	RP	0.176471	0	0.029412	0.029412	0.015228	3.083333
2287	24-Jun-08	2008	RP	0.176471	0	0.029412	0.029412	0.015228	3.083333
2300	24-Jun-08	2008	RP	0.176471	0	0.029412	0.029412	0.015228	3.083333
		2007	TO	0.175	0.125	0.175	0	0.00289	0.875
		2007	TO	0.175	0.125	0.175	0	0.00289	0.875
		2007	TO	0.175	0.125	0.175	0	0.00289	0.875
		2007	TO	0.175	0.125	0.175	0	0.00289	0.875
		2007	TO	0.175	0.125	0.175	0	0.00289	0.875
		2007	TO	0.175	0.125	0.175	0	0.00289	0.875
		2007	TO	0.175	0.125	0.175	0	0.00289	0.875
		2007	TO	0.175	0.125	0.175	0	0.00289	0.875
		2007	TO	0.175	0.125	0.175	0	0.00289	0.875

		2007	TO	0.175	0.125	0.175	0	0.00289	0.875
		2007	TO	0.175	0.125	0.175	0	0.00289	0.875
		2007	TO	0.175	0.125	0.175	0	0.00289	0.875
		2007	TO	0.175	0.125	0.175	0	0.00289	0.875
		2007	TO	0.175	0.125	0.175	0	0.00289	0.875
		2007	TO	0.175	0.125	0.175	0	0.00289	0.875
		2007	TO	0.175	0.125	0.175	0	0.00289	0.875
		2007	TO	0.175	0.125	0.175	0	0.00289	0.875
		2007	TO	0.175	0.125	0.175	0	0.00289	0.875
		2007	TO	0.175	0.125	0.175	0	0.00289	0.875
		2007	TO	0.175	0.125	0.175	0	0.00289	0.875
		2007	TO	0.175	0.125	0.175	0	0.00289	0.875
6	19-May-04	2004	ML						
5	19-May-04	2004	ML						
12	19-May-04	2004	ML						
10	19-May-04	2004	ML						
11	19-May-04	2004	ML						
13	19-May-04	2004	ML						
14	19-May-04	2004	ML						
15	19-May-04	2004	ML						
17	19-May-04	2004	ML						
18	19-May-04	2004	ML						
19	19-May-04	2004	ML						
8	19-May-04	2004	ML						
9	19-May-04	2004	ML						
16	19-May-04	2004	ML						
7	19-May-04	2004	ML						
20	19-May-04	2004	ML						
21	19-May-04	2004	ML						
61	21-May-04	2004	DE						
51	21-May-04	2004	DE						
47	21-May-04	2004	DE						
49	21-May-04	2004	DE						
50	21-May-04	2004	DE						
48	21-May-04	2004	DE						
46	21-May-04	2004	DE						
52	21-May-04	2004	DE						
53	21-May-04	2004	DE						
60	21-May-04	2004	DE						
54	21-May-04	2004	DE						
59	21-May-04	2004	DE						
55	21-May-04	2004	DE						
57	21-May-04	2004	DE						
56	21-May-04	2004	DE						

58	21-May-04	2004	DE						
392	31-May-04	2004	RC	0.428571	0.107143	0	0	0.401709	3
384	31-May-04	2004	RC	0.428571	0.107143	0	0	0.401709	3
385	31-May-04	2004	RC	0.428571	0.107143	0	0	0.401709	3
386	31-May-04	2004	RC	0.428571	0.107143	0	0	0.401709	3
387	31-May-04	2004	RC	0.428571	0.107143	0	0	0.401709	3
388	31-May-04	2004	RC	0.428571	0.107143	0	0	0.401709	3
390	31-May-04	2004	RC	0.428571	0.107143	0	0	0.401709	3
391	31-May-04	2004	RC	0.428571	0.107143	0	0	0.401709	3
393	31-May-04	2004	RC	0.428571	0.107143	0	0	0.401709	3
394	31-May-04	2004	RC	0.428571	0.107143	0	0	0.401709	3
395	31-May-04	2004	RC	0.428571	0.107143	0	0	0.401709	3
389	31-May-04	2004	RC	0.428571	0.107143	0	0	0.401709	3
476	3-Jun-04	2004	D1/D2						
477	3-Jun-04	2004	D1/D2						
496	4-Jun-04	2004	ML						
495	4-Jun-04	2004	ML						
499	4-Jun-04	2004	ML						
506	4-Jun-04	2004	ML						
492	4-Jun-04	2004	ML						
502	4-Jun-04	2004	ML						
483	4-Jun-04	2004	ML						
484	4-Jun-04	2004	ML						
480	4-Jun-04	2004	ML						
479	4-Jun-04	2004	ML						
481	4-Jun-04	2004	ML						
482	4-Jun-04	2004	ML						
493	4-Jun-04	2004	ML						
494	4-Jun-04	2004	ML						
498	4-Jun-04	2004	ML						
504	4-Jun-04	2004	ML						
505	4-Jun-04	2004	ML						
485	4-Jun-04	2004	ML						
486	4-Jun-04	2004	ML						
487	4-Jun-04	2004	ML						
500	4-Jun-04	2004	ML						
497	4-Jun-04	2004	ML						
488	4-Jun-04	2004	ML						
501	4-Jun-04	2004	ML						
489	4-Jun-04	2004	ML						
503	4-Jun-04	2004	ML						
490	4-Jun-04	2004	ML						
491	4-Jun-04	2004	ML						
478	4-Jun-04	2004	ML						

110	7-Jun-04	2004	SD	0.412698	0.301587	0.063492	0.634921	0.097297	2.642857
111	7-Jun-04	2004	SD	0.412698	0.301587	0.063492	0.634921	0.097297	2.642857
105	7-Jun-04	2004	SD	0.412698	0.301587	0.063492	0.634921	0.097297	2.642857
106	7-Jun-04	2004	SD	0.412698	0.301587	0.063492	0.634921	0.097297	2.642857
107	7-Jun-04	2004	SD	0.412698	0.301587	0.063492	0.634921	0.097297	2.642857
108	7-Jun-04	2004	SD	0.412698	0.301587	0.063492	0.634921	0.097297	2.642857
109	7-Jun-04	2004	SD	0.412698	0.301587	0.063492	0.634921	0.097297	2.642857
103	7-Jun-04	2004	SD	0.412698	0.301587	0.063492	0.634921	0.097297	2.642857
104	7-Jun-04	2004	SD	0.412698	0.301587	0.063492	0.634921	0.097297	2.642857
102	7-Jun-04	2004	SD	0.412698	0.301587	0.063492	0.634921	0.097297	2.642857
115	7-Jun-04	2004	SD	0.412698	0.301587	0.063492	0.634921	0.097297	2.642857
116	7-Jun-04	2004	SD	0.412698	0.301587	0.063492	0.634921	0.097297	2.642857
120	7-Jun-04	2004	SD	0.412698	0.301587	0.063492	0.634921	0.097297	2.642857
112	7-Jun-04	2004	SD	0.412698	0.301587	0.063492	0.634921	0.097297	2.642857
113	7-Jun-04	2004	SD	0.412698	0.301587	0.063492	0.634921	0.097297	2.642857
119	7-Jun-04	2004	SD	0.412698	0.301587	0.063492	0.634921	0.097297	2.642857
122	7-Jun-04	2004	SD	0.412698	0.301587	0.063492	0.634921	0.097297	2.642857
114	7-Jun-04	2004	SD	0.412698	0.301587	0.063492	0.634921	0.097297	2.642857
117	7-Jun-04	2004	SD	0.412698	0.301587	0.063492	0.634921	0.097297	2.642857
118	7-Jun-04	2004	SD	0.412698	0.301587	0.063492	0.634921	0.097297	2.642857
101	7-Jun-04	2004	SD	0.412698	0.301587	0.063492	0.634921	0.097297	2.642857
121	7-Jun-04	2004	SD	0.412698	0.301587	0.063492	0.634921	0.097297	2.642857
288	12-Jun-04	2004	RL	0.396226	0.113208	0.09434	0.320755	0.203008	0.333333
291	12-Jun-04	2004	RL	0.396226	0.113208	0.09434	0.320755	0.203008	0.333333
289	12-Jun-04	2004	RL	0.396226	0.113208	0.09434	0.320755	0.203008	0.333333
292	12-Jun-04	2004	RL	0.396226	0.113208	0.09434	0.320755	0.203008	0.333333
290	12-Jun-04	2004	RL	0.396226	0.113208	0.09434	0.320755	0.203008	0.333333
293	12-Jun-04	2004	RL	0.396226	0.113208	0.09434	0.320755	0.203008	0.333333
294	12-Jun-04	2004	RL	0.396226	0.113208	0.09434	0.320755	0.203008	0.333333
578	17-Jun-04	2004	RC	0.428571	0.107143	0	0	0.401709	3
582	17-Jun-04	2004	RC	0.428571	0.107143	0	0	0.401709	3
580	17-Jun-04	2004	RC	0.428571	0.107143	0	0	0.401709	3
570	17-Jun-04	2004	RC	0.428571	0.107143	0	0	0.401709	3
571	17-Jun-04	2004	RC	0.428571	0.107143	0	0	0.401709	3
573	17-Jun-04	2004	RC	0.428571	0.107143	0	0	0.401709	3
574	17-Jun-04	2004	RC	0.428571	0.107143	0	0	0.401709	3
575	17-Jun-04	2004	RC	0.428571	0.107143	0	0	0.401709	3
576	17-Jun-04	2004	RC	0.428571	0.107143	0	0	0.401709	3
577	17-Jun-04	2004	RC	0.428571	0.107143	0	0	0.401709	3
583	17-Jun-04	2004	RC	0.428571	0.107143	0	0	0.401709	3
572	17-Jun-04	2004	RC	0.428571	0.107143	0	0	0.401709	3
579	17-Jun-04	2004	RC	0.428571	0.107143	0	0	0.401709	3
581	17-Jun-04	2004	RC	0.428571	0.107143	0	0	0.401709	3
617	18-Jun-04	2004	DE						

606	18-Jun-04	2004	DE						
611	18-Jun-04	2004	DE						
610	18-Jun-04	2004	DE						
604	18-Jun-04	2004	DE						
605	18-Jun-04	2004	DE						
609	18-Jun-04	2004	DE						
613	18-Jun-04	2004	DE						
614	18-Jun-04	2004	DE						
615	18-Jun-04	2004	DE						
616	18-Jun-04	2004	DE						
608	18-Jun-04	2004	DE						
603	18-Jun-04	2004	DE						
607	18-Jun-04	2004	DE						
612	18-Jun-04	2004	DE						
654	19-Jun-04	2004	RP	0.095238	0.142857	0.095238	0.071429	0.323383	2
659	19-Jun-04	2004	RP	0.095238	0.142857	0.095238	0.071429	0.323383	2
640	19-Jun-04	2004	RP	0.095238	0.142857	0.095238	0.071429	0.323383	2
641	19-Jun-04	2004	RP	0.095238	0.142857	0.095238	0.071429	0.323383	2
663	19-Jun-04	2004	RP	0.095238	0.142857	0.095238	0.071429	0.323383	2
646	19-Jun-04	2004	RP	0.095238	0.142857	0.095238	0.071429	0.323383	2
651	19-Jun-04	2004	RP	0.095238	0.142857	0.095238	0.071429	0.323383	2
652	19-Jun-04	2004	RP	0.095238	0.142857	0.095238	0.071429	0.323383	2
645	19-Jun-04	2004	RP	0.095238	0.142857	0.095238	0.071429	0.323383	2
642	19-Jun-04	2004	RP	0.095238	0.142857	0.095238	0.071429	0.323383	2
643	19-Jun-04	2004	RP	0.095238	0.142857	0.095238	0.071429	0.323383	2
647	19-Jun-04	2004	RP	0.095238	0.142857	0.095238	0.071429	0.323383	2
648	19-Jun-04	2004	RP	0.095238	0.142857	0.095238	0.071429	0.323383	2
649	19-Jun-04	2004	RP	0.095238	0.142857	0.095238	0.071429	0.323383	2
650	19-Jun-04	2004	RP	0.095238	0.142857	0.095238	0.071429	0.323383	2
656	19-Jun-04	2004	RP	0.095238	0.142857	0.095238	0.071429	0.323383	2
657	19-Jun-04	2004	RP	0.095238	0.142857	0.095238	0.071429	0.323383	2
660	19-Jun-04	2004	RP	0.095238	0.142857	0.095238	0.071429	0.323383	2
661	19-Jun-04	2004	RP	0.095238	0.142857	0.095238	0.071429	0.323383	2
653	19-Jun-04	2004	RP	0.095238	0.142857	0.095238	0.071429	0.323383	2
655	19-Jun-04	2004	RP	0.095238	0.142857	0.095238	0.071429	0.323383	2
658	19-Jun-04	2004	RP	0.095238	0.142857	0.095238	0.071429	0.323383	2
644	19-Jun-04	2004	RP	0.095238	0.142857	0.095238	0.071429	0.323383	2
664	19-Jun-04	2004	RP	0.095238	0.142857	0.095238	0.071429	0.323383	2
662	19-Jun-04	2004	RP	0.095238	0.142857	0.095238	0.071429	0.323383	2
727	20-Jun-04	2004	ML						
724	20-Jun-04	2004	ML						
723	20-Jun-04	2004	ML						
726	20-Jun-04	2004	ML						
708	20-Jun-04	2004	ML						

709	20-Jun-04	2004	ML						
711	20-Jun-04	2004	ML						
712	20-Jun-04	2004	ML						
713	20-Jun-04	2004	ML						
714	20-Jun-04	2004	ML						
715	20-Jun-04	2004	ML						
716	20-Jun-04	2004	ML						
718	20-Jun-04	2004	ML						
719	20-Jun-04	2004	ML						
722	20-Jun-04	2004	ML						
728	20-Jun-04	2004	ML						
721	20-Jun-04	2004	ML						
725	20-Jun-04	2004	ML						
717	20-Jun-04	2004	ML						
720	20-Jun-04	2004	ML						
710	20-Jun-04	2004	ML						
730	24-Jun-04	2004	RL	0.396226	0.113208	0.09434	0.320755	0.203008	0.333333
733	24-Jun-04	2004	RL	0.396226	0.113208	0.09434	0.320755	0.203008	0.333333
734	24-Jun-04	2004	RL	0.396226	0.113208	0.09434	0.320755	0.203008	0.333333
737	24-Jun-04	2004	RL	0.396226	0.113208	0.09434	0.320755	0.203008	0.333333
741	24-Jun-04	2004	RL	0.396226	0.113208	0.09434	0.320755	0.203008	0.333333
731	24-Jun-04	2004	RL	0.396226	0.113208	0.09434	0.320755	0.203008	0.333333
732	24-Jun-04	2004	RL	0.396226	0.113208	0.09434	0.320755	0.203008	0.333333
735	24-Jun-04	2004	RL	0.396226	0.113208	0.09434	0.320755	0.203008	0.333333
738	24-Jun-04	2004	RL	0.396226	0.113208	0.09434	0.320755	0.203008	0.333333
739	24-Jun-04	2004	RL	0.396226	0.113208	0.09434	0.320755	0.203008	0.333333
736	24-Jun-04	2004	RL	0.396226	0.113208	0.09434	0.320755	0.203008	0.333333
729	24-Jun-04	2004	RL	0.396226	0.113208	0.09434	0.320755	0.203008	0.333333
740	24-Jun-04	2004	RL	0.396226	0.113208	0.09434	0.320755	0.203008	0.333333
7	8-May-05	2005	SC	0.183486	0	0.073394	0	0.006329	6.769231
8	8-May-05	2005	SC	0.183486	0	0.073394	0	0.006329	6.769231
11	8-May-05	2005	SC	0.183486	0	0.073394	0	0.006329	6.769231
13	8-May-05	2005	SC	0.183486	0	0.073394	0	0.006329	6.769231
12	8-May-05	2005	SC	0.183486	0	0.073394	0	0.006329	6.769231
	8-May-05	2005	SC	0.183486	0	0.073394	0	0.006329	6.769231
9	8-May-05	2005	SC	0.183486	0	0.073394	0	0.006329	6.769231
2	8-May-05	2005	SC	0.183486	0	0.073394	0	0.006329	6.769231
14	8-May-05	2005	SC	0.183486	0	0.073394	0	0.006329	6.769231
4	8-May-05	2005	SC	0.183486	0	0.073394	0	0.006329	6.769231
10	8-May-05	2005	SC	0.183486	0	0.073394	0	0.006329	6.769231
1	8-May-05	2005	SC	0.183486	0	0.073394	0	0.006329	6.769231
3	8-May-05	2005	SC	0.183486	0	0.073394	0	0.006329	6.769231
6	8-May-05	2005	SC	0.183486	0	0.073394	0	0.006329	6.769231
5	8-May-05	2005	SC	0.183486	0	0.073394	0	0.006329	6.769231

521	20-May-05	2005	SHCC	0.319149	0.106383	0	0.191489	0.034483	0.230769
525	20-May-05	2005	SHCC	0.319149	0.106383	0	0.191489	0.034483	0.230769
555	20-May-05	2005	SHCC	0.319149	0.106383	0	0.191489	0.034483	0.230769
559	20-May-05	2005	SHCC	0.319149	0.106383	0	0.191489	0.034483	0.230769
520	20-May-05	2005	SHCC	0.319149	0.106383	0	0.191489	0.034483	0.230769
554	20-May-05	2005	SHCC	0.319149	0.106383	0	0.191489	0.034483	0.230769
528	20-May-05	2005	SHCC	0.319149	0.106383	0	0.191489	0.034483	0.230769
529	20-May-05	2005	SHCC	0.319149	0.106383	0	0.191489	0.034483	0.230769
562	20-May-05	2005	SHCC	0.319149	0.106383	0	0.191489	0.034483	0.230769
563	20-May-05	2005	SHCC	0.319149	0.106383	0	0.191489	0.034483	0.230769
518	20-May-05	2005	SHCC	0.319149	0.106383	0	0.191489	0.034483	0.230769
552	20-May-05	2005	SHCC	0.319149	0.106383	0	0.191489	0.034483	0.230769
514	20-May-05	2005	SHCC	0.319149	0.106383	0	0.191489	0.034483	0.230769
548	20-May-05	2005	SHCC	0.319149	0.106383	0	0.191489	0.034483	0.230769
513	20-May-05	2005	SHCC	0.319149	0.106383	0	0.191489	0.034483	0.230769
526	20-May-05	2005	SHCC	0.319149	0.106383	0	0.191489	0.034483	0.230769
547	20-May-05	2005	SHCC	0.319149	0.106383	0	0.191489	0.034483	0.230769
560	20-May-05	2005	SHCC	0.319149	0.106383	0	0.191489	0.034483	0.230769
510	20-May-05	2005	SHCC	0.319149	0.106383	0	0.191489	0.034483	0.230769
517	20-May-05	2005	SHCC	0.319149	0.106383	0	0.191489	0.034483	0.230769
527	20-May-05	2005	SHCC	0.319149	0.106383	0	0.191489	0.034483	0.230769
544	20-May-05	2005	SHCC	0.319149	0.106383	0	0.191489	0.034483	0.230769
551	20-May-05	2005	SHCC	0.319149	0.106383	0	0.191489	0.034483	0.230769
561	20-May-05	2005	SHCC	0.319149	0.106383	0	0.191489	0.034483	0.230769
512	20-May-05	2005	SHCC	0.319149	0.106383	0	0.191489	0.034483	0.230769
519	20-May-05	2005	SHCC	0.319149	0.106383	0	0.191489	0.034483	0.230769
522	20-May-05	2005	SHCC	0.319149	0.106383	0	0.191489	0.034483	0.230769
524	20-May-05	2005	SHCC	0.319149	0.106383	0	0.191489	0.034483	0.230769
546	20-May-05	2005	SHCC	0.319149	0.106383	0	0.191489	0.034483	0.230769
553	20-May-05	2005	SHCC	0.319149	0.106383	0	0.191489	0.034483	0.230769
556	20-May-05	2005	SHCC	0.319149	0.106383	0	0.191489	0.034483	0.230769
558	20-May-05	2005	SHCC	0.319149	0.106383	0	0.191489	0.034483	0.230769
515	20-May-05	2005	SHCC	0.319149	0.106383	0	0.191489	0.034483	0.230769
516	20-May-05	2005	SHCC	0.319149	0.106383	0	0.191489	0.034483	0.230769
530	20-May-05	2005	SHCC	0.319149	0.106383	0	0.191489	0.034483	0.230769
531	20-May-05	2005	SHCC	0.319149	0.106383	0	0.191489	0.034483	0.230769
549	20-May-05	2005	SHCC	0.319149	0.106383	0	0.191489	0.034483	0.230769
550	20-May-05	2005	SHCC	0.319149	0.106383	0	0.191489	0.034483	0.230769
564	20-May-05	2005	SHCC	0.319149	0.106383	0	0.191489	0.034483	0.230769
565	20-May-05	2005	SHCC	0.319149	0.106383	0	0.191489	0.034483	0.230769
511	20-May-05	2005	SHCC	0.319149	0.106383	0	0.191489	0.034483	0.230769
545	20-May-05	2005	SHCC	0.319149	0.106383	0	0.191489	0.034483	0.230769
523	20-May-05	2005	SHCC	0.319149	0.106383	0	0.191489	0.034483	0.230769
557	20-May-05	2005	SHCC	0.319149	0.106383	0	0.191489	0.034483	0.230769

588	21-May-05	2005	SC	0.183486	0	0.073394	0	0.006329	6.769231
586	21-May-05	2005	SC	0.183486	0	0.073394	0	0.006329	6.769231
568	21-May-05	2005	SC	0.183486	0	0.073394	0	0.006329	6.769231
571	21-May-05	2005	SC	0.183486	0	0.073394	0	0.006329	6.769231
573	21-May-05	2005	SC	0.183486	0	0.073394	0	0.006329	6.769231
577	21-May-05	2005	SC	0.183486	0	0.073394	0	0.006329	6.769231
567	21-May-05	2005	SC	0.183486	0	0.073394	0	0.006329	6.769231
579	21-May-05	2005	SC	0.183486	0	0.073394	0	0.006329	6.769231
570	21-May-05	2005	SC	0.183486	0	0.073394	0	0.006329	6.769231
572	21-May-05	2005	SC	0.183486	0	0.073394	0	0.006329	6.769231
584	21-May-05	2005	SC	0.183486	0	0.073394	0	0.006329	6.769231
585	21-May-05	2005	SC	0.183486	0	0.073394	0	0.006329	6.769231
580	21-May-05	2005	SC	0.183486	0	0.073394	0	0.006329	6.769231
583	21-May-05	2005	SC	0.183486	0	0.073394	0	0.006329	6.769231
576	21-May-05	2005	SC	0.183486	0	0.073394	0	0.006329	6.769231
569	21-May-05	2005	SC	0.183486	0	0.073394	0	0.006329	6.769231
581	21-May-05	2005	SC	0.183486	0	0.073394	0	0.006329	6.769231
582	21-May-05	2005	SC	0.183486	0	0.073394	0	0.006329	6.769231
587	21-May-05	2005	SC	0.183486	0	0.073394	0	0.006329	6.769231
575	21-May-05	2005	SC	0.183486	0	0.073394	0	0.006329	6.769231
574	21-May-05	2005	SC	0.183486	0	0.073394	0	0.006329	6.769231
566	21-May-05	2005	SC	0.183486	0	0.073394	0	0.006329	6.769231
578	21-May-05	2005	SC	0.183486	0	0.073394	0	0.006329	6.769231
742	25-May-05	2005	SD	0.365079	0.095238	0.095238	0.380952	0.048387	2.5
747	25-May-05	2005	SD	0.365079	0.095238	0.095238	0.380952	0.048387	2.5
749	25-May-05	2005	SD	0.365079	0.095238	0.095238	0.380952	0.048387	2.5
737	25-May-05	2005	SD	0.365079	0.095238	0.095238	0.380952	0.048387	2.5
736	25-May-05	2005	SD	0.365079	0.095238	0.095238	0.380952	0.048387	2.5
738	25-May-05	2005	SD	0.365079	0.095238	0.095238	0.380952	0.048387	2.5
739	25-May-05	2005	SD	0.365079	0.095238	0.095238	0.380952	0.048387	2.5
740	25-May-05	2005	SD	0.365079	0.095238	0.095238	0.380952	0.048387	2.5
744	25-May-05	2005	SD	0.365079	0.095238	0.095238	0.380952	0.048387	2.5
746	25-May-05	2005	SD	0.365079	0.095238	0.095238	0.380952	0.048387	2.5
741	25-May-05	2005	SD	0.365079	0.095238	0.095238	0.380952	0.048387	2.5
743	25-May-05	2005	SD	0.365079	0.095238	0.095238	0.380952	0.048387	2.5
748	25-May-05	2005	SD	0.365079	0.095238	0.095238	0.380952	0.048387	2.5
745	25-May-05	2005	SD	0.365079	0.095238	0.095238	0.380952	0.048387	2.5
977	1-Jun-05	2005	D1/D2						
978	1-Jun-05	2005	D1/D2						
974	1-Jun-05	2005	D1/D2						
976	1-Jun-05	2005	D1/D2						
975	1-Jun-05	2005	D1/D2						
973	1-Jun-05	2005	D1/D2						
980	1-Jun-05	2005	D1/D2						

979	1-Jun-05	2005	D1/D2						
981	1-Jun-05	2005	D1/D2						
1360	5-Jun-05	2005	7M	0.264151	0.09434	0	0.301887	0.01519	8.266667
1362	5-Jun-05	2005	7M	0.264151	0.09434	0	0.301887	0.01519	8.266667
1364	5-Jun-05	2005	7M	0.264151	0.09434	0	0.301887	0.01519	8.266667
1366	5-Jun-05	2005	7M	0.264151	0.09434	0	0.301887	0.01519	8.266667
1361	5-Jun-05	2005	7M	0.264151	0.09434	0	0.301887	0.01519	8.266667
1367	5-Jun-05	2005	7M	0.264151	0.09434	0	0.301887	0.01519	8.266667
1363	5-Jun-05	2005	7M	0.264151	0.09434	0	0.301887	0.01519	8.266667
1365	5-Jun-05	2005	7M	0.264151	0.09434	0	0.301887	0.01519	8.266667
1368	5-Jun-05	2005	7M	0.264151	0.09434	0	0.301887	0.01519	8.266667
1084	5-Jun-05	2005	MM	0.220779	0.064935	0.025974	0.12987	0.044118	1.727273
1083	5-Jun-05	2005	MM	0.220779	0.064935	0.025974	0.12987	0.044118	1.727273
1085	5-Jun-05	2005	MM	0.220779	0.064935	0.025974	0.12987	0.044118	1.727273
1082	5-Jun-05	2005	MM	0.220779	0.064935	0.025974	0.12987	0.044118	1.727273
1092	5-Jun-05	2005	MM	0.220779	0.064935	0.025974	0.12987	0.044118	1.727273
1093	5-Jun-05	2005	MM	0.220779	0.064935	0.025974	0.12987	0.044118	1.727273
1080	5-Jun-05	2005	MM	0.220779	0.064935	0.025974	0.12987	0.044118	1.727273
1088	5-Jun-05	2005	MM	0.220779	0.064935	0.025974	0.12987	0.044118	1.727273
1086	5-Jun-05	2005	MM	0.220779	0.064935	0.025974	0.12987	0.044118	1.727273
1090	5-Jun-05	2005	MM	0.220779	0.064935	0.025974	0.12987	0.044118	1.727273
1091	5-Jun-05	2005	MM	0.220779	0.064935	0.025974	0.12987	0.044118	1.727273
1089	5-Jun-05	2005	MM	0.220779	0.064935	0.025974	0.12987	0.044118	1.727273
1078	5-Jun-05	2005	MM	0.220779	0.064935	0.025974	0.12987	0.044118	1.727273
1087	5-Jun-05	2005	MM	0.220779	0.064935	0.025974	0.12987	0.044118	1.727273
1079	5-Jun-05	2005	MM	0.220779	0.064935	0.025974	0.12987	0.044118	1.727273
1081	5-Jun-05	2005	MM	0.220779	0.064935	0.025974	0.12987	0.044118	1.727273
1398	5-Jun-05	2005	MM	0.220779	0.064935	0.025974	0.12987	0.044118	1.727273
1401	5-Jun-05	2005	MM	0.220779	0.064935	0.025974	0.12987	0.044118	1.727273
1400	5-Jun-05	2005	MM	0.220779	0.064935	0.025974	0.12987	0.044118	1.727273
1397	5-Jun-05	2005	MM	0.220779	0.064935	0.025974	0.12987	0.044118	1.727273
1399	5-Jun-05	2005	MM	0.220779	0.064935	0.025974	0.12987	0.044118	1.727273
1396	5-Jun-05	2005	MM	0.220779	0.064935	0.025974	0.12987	0.044118	1.727273
1395	5-Jun-05	2005	MM	0.220779	0.064935	0.025974	0.12987	0.044118	1.727273
1141	11-Jun-05	2005	SC	0.183486	0	0.073394	0	0.006329	6.769231
1143	11-Jun-05	2005	SC	0.183486	0	0.073394	0	0.006329	6.769231
1147	11-Jun-05	2005	SC	0.183486	0	0.073394	0	0.006329	6.769231
1149	11-Jun-05	2005	SC	0.183486	0	0.073394	0	0.006329	6.769231
1134	11-Jun-05	2005	SC	0.183486	0	0.073394	0	0.006329	6.769231
1136	11-Jun-05	2005	SC	0.183486	0	0.073394	0	0.006329	6.769231
1137	11-Jun-05	2005	SC	0.183486	0	0.073394	0	0.006329	6.769231
1140	11-Jun-05	2005	SC	0.183486	0	0.073394	0	0.006329	6.769231
1133	11-Jun-05	2005	SC	0.183486	0	0.073394	0	0.006329	6.769231
1135	11-Jun-05	2005	SC	0.183486	0	0.073394	0	0.006329	6.769231

1138	11-Jun-05	2005	SC	0.183486	0	0.073394	0	0.006329	6.769231
1145	11-Jun-05	2005	SC	0.183486	0	0.073394	0	0.006329	6.769231
1146	11-Jun-05	2005	SC	0.183486	0	0.073394	0	0.006329	6.769231
1148	11-Jun-05	2005	SC	0.183486	0	0.073394	0	0.006329	6.769231
1150	11-Jun-05	2005	SC	0.183486	0	0.073394	0	0.006329	6.769231
1151	11-Jun-05	2005	SC	0.183486	0	0.073394	0	0.006329	6.769231
1139	11-Jun-05	2005	SC	0.183486	0	0.073394	0	0.006329	6.769231
1144	11-Jun-05	2005	SC	0.183486	0	0.073394	0	0.006329	6.769231
1142	11-Jun-05	2005	SC	0.183486	0	0.073394	0	0.006329	6.769231
1699	17-Jun-05	2005	LT1	0.393939	0.030303	0	0.242424	0	4.444444
1695	17-Jun-05	2005	LT1	0.393939	0.030303	0	0.242424	0	4.444444
1696	17-Jun-05	2005	LT1	0.393939	0.030303	0	0.242424	0	4.444444
1697	17-Jun-05	2005	LT1	0.393939	0.030303	0	0.242424	0	4.444444
1694	17-Jun-05	2005	LT1	0.393939	0.030303	0	0.242424	0	4.444444
1693	17-Jun-05	2005	LT1	0.393939	0.030303	0	0.242424	0	4.444444
1698	17-Jun-05	2005	LT1	0.393939	0.030303	0	0.242424	0	4.444444
1498	18-Jun-05	2005	RP	0.333333	0	0	0.047619	0.072115	2.25
1493	18-Jun-05	2005	RP	0.333333	0	0	0.047619	0.072115	2.25
1494	18-Jun-05	2005	RP	0.333333	0	0	0.047619	0.072115	2.25
1495	18-Jun-05	2005	RP	0.333333	0	0	0.047619	0.072115	2.25
1496	18-Jun-05	2005	RP	0.333333	0	0	0.047619	0.072115	2.25
1499	18-Jun-05	2005	RP	0.333333	0	0	0.047619	0.072115	2.25
1500	18-Jun-05	2005	RP	0.333333	0	0	0.047619	0.072115	2.25
1497	18-Jun-05	2005	RP	0.333333	0	0	0.047619	0.072115	2.25
1501	18-Jun-05	2005	RP	0.333333	0	0	0.047619	0.072115	2.25
1721	19-Jun-05	2005	HH	0.272727	0.072727	0	0.145455	0.005076	0.653846
1717	19-Jun-05	2005	HH	0.272727	0.072727	0	0.145455	0.005076	0.653846
1718	19-Jun-05	2005	HH	0.272727	0.072727	0	0.145455	0.005076	0.653846
1720	19-Jun-05	2005	HH	0.272727	0.072727	0	0.145455	0.005076	0.653846
1722	19-Jun-05	2005	HH	0.272727	0.072727	0	0.145455	0.005076	0.653846
1723	19-Jun-05	2005	HH	0.272727	0.072727	0	0.145455	0.005076	0.653846
1725	19-Jun-05	2005	HH	0.272727	0.072727	0	0.145455	0.005076	0.653846
1711	19-Jun-05	2005	HH	0.272727	0.072727	0	0.145455	0.005076	0.653846
1728	19-Jun-05	2005	HH	0.272727	0.072727	0	0.145455	0.005076	0.653846
1727	19-Jun-05	2005	HH	0.272727	0.072727	0	0.145455	0.005076	0.653846
1714	19-Jun-05	2005	HH	0.272727	0.072727	0	0.145455	0.005076	0.653846
1715	19-Jun-05	2005	HH	0.272727	0.072727	0	0.145455	0.005076	0.653846
1724	19-Jun-05	2005	HH	0.272727	0.072727	0	0.145455	0.005076	0.653846
1729	19-Jun-05	2005	HH	0.272727	0.072727	0	0.145455	0.005076	0.653846
1730	19-Jun-05	2005	HH	0.272727	0.072727	0	0.145455	0.005076	0.653846
1731	19-Jun-05	2005	HH	0.272727	0.072727	0	0.145455	0.005076	0.653846
1713	19-Jun-05	2005	HH	0.272727	0.072727	0	0.145455	0.005076	0.653846
1726	19-Jun-05	2005	HH	0.272727	0.072727	0	0.145455	0.005076	0.653846
1712	19-Jun-05	2005	HH	0.272727	0.072727	0	0.145455	0.005076	0.653846

1716	19-Jun-05	2005	HH	0.272727	0.072727	0	0.145455	0.005076	0.653846
1719	19-Jun-05	2005	HH	0.272727	0.072727	0	0.145455	0.005076	0.653846
12	8-May-06	2006	RC	0.2	0	0.1	0.15	0.033898	3
15	8-May-06	2006	RC	0.2	0	0.1	0.15	0.033898	3
14	8-May-06	2006	RC	0.2	0	0.1	0.15	0.033898	3
16	8-May-06	2006	RC	0.2	0	0.1	0.15	0.033898	3
19	8-May-06	2006	RC	0.2	0	0.1	0.15	0.033898	3
18	8-May-06	2006	RC	0.2	0	0.1	0.15	0.033898	3
17	8-May-06	2006	RC	0.2	0	0.1	0.15	0.033898	3
20	8-May-06	2006	RC	0.2	0	0.1	0.15	0.033898	3
13	8-May-06	2006	RC	0.2	0	0.1	0.15	0.033898	3
188	4-Jun-06	2006	LT1	0.222222	0.111111	0	0.148148	0	4
192	4-Jun-06	2006	LT1	0.222222	0.111111	0	0.148148	0	4
193	4-Jun-06	2006	LT1	0.222222	0.111111	0	0.148148	0	4
195	4-Jun-06	2006	LT1	0.222222	0.111111	0	0.148148	0	4
196	4-Jun-06	2006	LT1	0.222222	0.111111	0	0.148148	0	4
197	4-Jun-06	2006	LT1	0.222222	0.111111	0	0.148148	0	4
198	4-Jun-06	2006	LT1	0.222222	0.111111	0	0.148148	0	4
187	4-Jun-06	2006	LT1	0.222222	0.111111	0	0.148148	0	4
185	4-Jun-06	2006	LT1	0.222222	0.111111	0	0.148148	0	4
186	4-Jun-06	2006	LT1	0.222222	0.111111	0	0.148148	0	4
189	4-Jun-06	2006	LT1	0.222222	0.111111	0	0.148148	0	4
190	4-Jun-06	2006	LT1	0.222222	0.111111	0	0.148148	0	4
184	4-Jun-06	2006	LT1	0.222222	0.111111	0	0.148148	0	4
191	4-Jun-06	2006	LT1	0.222222	0.111111	0	0.148148	0	4
194	4-Jun-06	2006	LT1	0.222222	0.111111	0	0.148148	0	4
4	6-May-07	2007	ML						
11	6-May-07	2007	ML						
23	6-May-07	2007	ML						
5	6-May-07	2007	ML						
10	6-May-07	2007	ML						
6	6-May-07	2007	ML						
13	6-May-07	2007	ML						
14	6-May-07	2007	ML						
15	6-May-07	2007	ML						
17	6-May-07	2007	ML						
25	6-May-07	2007	ML						
26	6-May-07	2007	ML						
27	6-May-07	2007	ML						
28	6-May-07	2007	ML						
29	6-May-07	2007	ML						
30	6-May-07	2007	ML						
3	6-May-07	2007	ML						
12	6-May-07	2007	ML						

16	6-May-07	2007	ML							
1	6-May-07	2007	ML							
2	6-May-07	2007	ML							
22	6-May-07	2007	ML							
24	6-May-07	2007	ML							
7	6-May-07	2007	ML							
18	6-May-07	2007	ML							
8	6-May-07	2007	ML							
19	6-May-07	2007	ML							
21	6-May-07	2007	ML							
9	6-May-07	2007	ML							
20	6-May-07	2007	ML							
500	13-May-07	2007	RC		0.25	0	0.05	0.05	0.018182	2.875
501	13-May-07	2007	RC		0.25	0	0.05	0.05	0.018182	2.875
517	13-May-07	2007	RC		0.25	0	0.05	0.05	0.018182	2.875
496	13-May-07	2007	RC		0.25	0	0.05	0.05	0.018182	2.875
497	13-May-07	2007	RC		0.25	0	0.05	0.05	0.018182	2.875
499	13-May-07	2007	RC		0.25	0	0.05	0.05	0.018182	2.875
498	13-May-07	2007	RC		0.25	0	0.05	0.05	0.018182	2.875
515	13-May-07	2007	RC		0.25	0	0.05	0.05	0.018182	2.875
516	13-May-07	2007	RC		0.25	0	0.05	0.05	0.018182	2.875
506	13-May-07	2007	RC		0.25	0	0.05	0.05	0.018182	2.875
507	13-May-07	2007	RC		0.25	0	0.05	0.05	0.018182	2.875
510	13-May-07	2007	RC		0.25	0	0.05	0.05	0.018182	2.875
514	13-May-07	2007	RC		0.25	0	0.05	0.05	0.018182	2.875
512	13-May-07	2007	RC		0.25	0	0.05	0.05	0.018182	2.875
518	13-May-07	2007	RC		0.25	0	0.05	0.05	0.018182	2.875
520	13-May-07	2007	RC		0.25	0	0.05	0.05	0.018182	2.875
521	13-May-07	2007	RC		0.25	0	0.05	0.05	0.018182	2.875
492	13-May-07	2007	RC		0.25	0	0.05	0.05	0.018182	2.875
487	13-May-07	2007	RC		0.25	0	0.05	0.05	0.018182	2.875
485	13-May-07	2007	RC		0.25	0	0.05	0.05	0.018182	2.875
502	13-May-07	2007	RC		0.25	0	0.05	0.05	0.018182	2.875
503	13-May-07	2007	RC		0.25	0	0.05	0.05	0.018182	2.875
486	13-May-07	2007	RC		0.25	0	0.05	0.05	0.018182	2.875
504	13-May-07	2007	RC		0.25	0	0.05	0.05	0.018182	2.875
484	13-May-07	2007	RC		0.25	0	0.05	0.05	0.018182	2.875
488	13-May-07	2007	RC		0.25	0	0.05	0.05	0.018182	2.875
489	13-May-07	2007	RC		0.25	0	0.05	0.05	0.018182	2.875
483	13-May-07	2007	RC		0.25	0	0.05	0.05	0.018182	2.875
490	13-May-07	2007	RC		0.25	0	0.05	0.05	0.018182	2.875
491	13-May-07	2007	RC		0.25	0	0.05	0.05	0.018182	2.875
505	13-May-07	2007	RC		0.25	0	0.05	0.05	0.018182	2.875
495	13-May-07	2007	RC		0.25	0	0.05	0.05	0.018182	2.875

508	13-May-07	2007	RC	0.25	0	0.05	0.05	0.018182	2.875
509	13-May-07	2007	RC	0.25	0	0.05	0.05	0.018182	2.875
511	13-May-07	2007	RC	0.25	0	0.05	0.05	0.018182	2.875
519	13-May-07	2007	RC	0.25	0	0.05	0.05	0.018182	2.875
493	13-May-07	2007	RC	0.25	0	0.05	0.05	0.018182	2.875
482	13-May-07	2007	RC	0.25	0	0.05	0.05	0.018182	2.875
494	13-May-07	2007	RC	0.25	0	0.05	0.05	0.018182	2.875
513	13-May-07	2007	RC	0.25	0	0.05	0.05	0.018182	2.875
580	15-May-07	2007	RL	0.157895	0.157895	0	0	0.028571	0.333333
583	15-May-07	2007	RL	0.157895	0.157895	0	0	0.028571	0.333333
585	15-May-07	2007	RL	0.157895	0.157895	0	0	0.028571	0.333333
586	15-May-07	2007	RL	0.157895	0.157895	0	0	0.028571	0.333333
579	15-May-07	2007	RL	0.157895	0.157895	0	0	0.028571	0.333333
584	15-May-07	2007	RL	0.157895	0.157895	0	0	0.028571	0.333333
587	15-May-07	2007	RL	0.157895	0.157895	0	0	0.028571	0.333333
588	15-May-07	2007	RL	0.157895	0.157895	0	0	0.028571	0.333333
582	15-May-07	2007	RL	0.157895	0.157895	0	0	0.028571	0.333333
569	15-May-07	2007	RL	0.157895	0.157895	0	0	0.028571	0.333333
589	15-May-07	2007	RL	0.157895	0.157895	0	0	0.028571	0.333333
551	15-May-07	2007	RL	0.157895	0.157895	0	0	0.028571	0.333333
568	15-May-07	2007	RL	0.157895	0.157895	0	0	0.028571	0.333333
552	15-May-07	2007	RL	0.157895	0.157895	0	0	0.028571	0.333333
554	15-May-07	2007	RL	0.157895	0.157895	0	0	0.028571	0.333333
567	15-May-07	2007	RL	0.157895	0.157895	0	0	0.028571	0.333333
555	15-May-07	2007	RL	0.157895	0.157895	0	0	0.028571	0.333333
556	15-May-07	2007	RL	0.157895	0.157895	0	0	0.028571	0.333333
581	15-May-07	2007	RL	0.157895	0.157895	0	0	0.028571	0.333333
550	15-May-07	2007	RL	0.157895	0.157895	0	0	0.028571	0.333333
566	15-May-07	2007	RL	0.157895	0.157895	0	0	0.028571	0.333333
562	15-May-07	2007	RL	0.157895	0.157895	0	0	0.028571	0.333333
572	15-May-07	2007	RL	0.157895	0.157895	0	0	0.028571	0.333333
571	15-May-07	2007	RL	0.157895	0.157895	0	0	0.028571	0.333333
565	15-May-07	2007	RL	0.157895	0.157895	0	0	0.028571	0.333333
563	15-May-07	2007	RL	0.157895	0.157895	0	0	0.028571	0.333333
564	15-May-07	2007	RL	0.157895	0.157895	0	0	0.028571	0.333333
573	15-May-07	2007	RL	0.157895	0.157895	0	0	0.028571	0.333333
560	15-May-07	2007	RL	0.157895	0.157895	0	0	0.028571	0.333333
561	15-May-07	2007	RL	0.157895	0.157895	0	0	0.028571	0.333333
570	15-May-07	2007	RL	0.157895	0.157895	0	0	0.028571	0.333333
574	15-May-07	2007	RL	0.157895	0.157895	0	0	0.028571	0.333333
575	15-May-07	2007	RL	0.157895	0.157895	0	0	0.028571	0.333333
576	15-May-07	2007	RL	0.157895	0.157895	0	0	0.028571	0.333333
578	15-May-07	2007	RL	0.157895	0.157895	0	0	0.028571	0.333333
557	15-May-07	2007	RL	0.157895	0.157895	0	0	0.028571	0.333333

558	15-May-07	2007	RL	0.157895	0.157895	0	0	0.028571	0.333333
549	15-May-07	2007	RL	0.157895	0.157895	0	0	0.028571	0.333333
553	15-May-07	2007	RL	0.157895	0.157895	0	0	0.028571	0.333333
548	15-May-07	2007	RL	0.157895	0.157895	0	0	0.028571	0.333333
559	15-May-07	2007	RL	0.157895	0.157895	0	0	0.028571	0.333333
577	15-May-07	2007	RL	0.157895	0.157895	0	0	0.028571	0.333333
836	15-May-07	2007	ML						
845	15-May-07	2007	ML						
841	15-May-07	2007	ML						
842	15-May-07	2007	ML						
839	15-May-07	2007	ML						
843	15-May-07	2007	ML						
834	15-May-07	2007	ML						
835	15-May-07	2007	ML						
844	15-May-07	2007	ML						
846	15-May-07	2007	ML						
833	15-May-07	2007	ML						
838	15-May-07	2007	ML						
840	15-May-07	2007	ML						
847	15-May-07	2007	ML						
944	16-May-07	2007	HH	0.089552	0.149254	0.104478	0.029851	0	0.615385
945	16-May-07	2007	HH	0.089552	0.149254	0.104478	0.029851	0	0.615385
946	16-May-07	2007	HH	0.089552	0.149254	0.104478	0.029851	0	0.615385
947	16-May-07	2007	HH	0.089552	0.149254	0.104478	0.029851	0	0.615385
948	16-May-07	2007	HH	0.089552	0.149254	0.104478	0.029851	0	0.615385
943	16-May-07	2007	HH	0.089552	0.149254	0.104478	0.029851	0	0.615385
949	16-May-07	2007	HH	0.089552	0.149254	0.104478	0.029851	0	0.615385
940	16-May-07	2007	HH	0.089552	0.149254	0.104478	0.029851	0	0.615385
941	16-May-07	2007	HH	0.089552	0.149254	0.104478	0.029851	0	0.615385
942	16-May-07	2007	HH	0.089552	0.149254	0.104478	0.029851	0	0.615385
950	16-May-07	2007	HH	0.089552	0.149254	0.104478	0.029851	0	0.615385
939	16-May-07	2007	HH	0.089552	0.149254	0.104478	0.029851	0	0.615385
	30-May-07	2007	YY	0.2	0.08	0.06	0.08	0	0.65
	30-May-07	2007	YY	0.2	0.08	0.06	0.08	0	0.65
	30-May-07	2007	YY	0.2	0.08	0.06	0.08	0	0.65
	30-May-07	2007	YY	0.2	0.08	0.06	0.08	0	0.65
	30-May-07	2007	YY	0.2	0.08	0.06	0.08	0	0.65
	30-May-07	2007	YY	0.2	0.08	0.06	0.08	0	0.65
	30-May-07	2007	YY	0.2	0.08	0.06	0.08	0	0.65
	30-May-07	2007	YY	0.2	0.08	0.06	0.08	0	0.65
	30-May-07	2007	YY	0.2	0.08	0.06	0.08	0	0.65
	30-May-07	2007	YY	0.2	0.08	0.06	0.08	0	0.65
	30-May-07	2007	YY	0.2	0.08	0.06	0.08	0	0.65
	30-May-07	2007	YY	0.2	0.08	0.06	0.08	0	0.65

	30-May-07	2007	YY	0.2	0.08	0.06	0.08	0	0.65
	30-May-07	2007	YY	0.2	0.08	0.06	0.08	0	0.65
	30-May-07	2007	YY	0.2	0.08	0.06	0.08	0	0.65
	30-May-07	2007	YY	0.2	0.08	0.06	0.08	0	0.65
	30-May-07	2007	YY	0.2	0.08	0.06	0.08	0	0.65
	30-May-07	2007	YY	0.2	0.08	0.06	0.08	0	0.65
	30-May-07	2007	YY	0.2	0.08	0.06	0.08	0	0.65
	30-May-07	2007	YY	0.2	0.08	0.06	0.08	0	0.65
	30-May-07	2007	YY	0.2	0.08	0.06	0.08	0	0.65
	30-May-07	2007	YY	0.2	0.08	0.06	0.08	0	0.65
	30-May-07	2007	YY	0.2	0.08	0.06	0.08	0	0.65
	30-May-07	2007	YY	0.2	0.08	0.06	0.08	0	0.65
	30-May-07	2007	YY	0.2	0.08	0.06	0.08	0	0.65
	9-Jun-07	2007	RC	0.25	0	0.05	0.05	0.018182	2.875
	9-Jun-07	2007	RC	0.25	0	0.05	0.05	0.018182	2.875
	9-Jun-07	2007	RC	0.25	0	0.05	0.05	0.018182	2.875
	9-Jun-07	2007	RC	0.25	0	0.05	0.05	0.018182	2.875
	9-Jun-07	2007	RC	0.25	0	0.05	0.05	0.018182	2.875
	9-Jun-07	2007	RC	0.25	0	0.05	0.05	0.018182	2.875
	9-Jun-07	2007	RC	0.25	0	0.05	0.05	0.018182	2.875
	9-Jun-07	2007	RC	0.25	0	0.05	0.05	0.018182	2.875
	9-Jun-07	2007	RC	0.25	0	0.05	0.05	0.018182	2.875
	9-Jun-07	2007	RC	0.25	0	0.05	0.05	0.018182	2.875
	9-Jun-07	2007	RC	0.25	0	0.05	0.05	0.018182	2.875
	9-Jun-07	2007	RC	0.25	0	0.05	0.05	0.018182	2.875
	9-Jun-07	2007	RC	0.25	0	0.05	0.05	0.018182	2.875
	9-Jun-07	2007	RC	0.25	0	0.05	0.05	0.018182	2.875
	9-Jun-07	2007	RC	0.25	0	0.05	0.05	0.018182	2.875
	9-Jun-07	2007	RC	0.25	0	0.05	0.05	0.018182	2.875
	9-Jun-07	2007	RC	0.25	0	0.05	0.05	0.018182	2.875
1402	18-May-08	2008	SD						
1401	18-May-08	2008	SD						
1409	18-May-08	2008	SD						
1410	18-May-08	2008	SD						
1406	18-May-08	2008	SD						
1400	18-May-08	2008	SD						
1414	18-May-08	2008	SD						
1398	18-May-08	2008	SD						
1412	18-May-08	2008	SD						
1415	18-May-08	2008	SD						
1403	18-May-08	2008	SD						
1404	18-May-08	2008	SD						
1405	18-May-08	2008	SD						
1411	18-May-08	2008	SD						

1408	18-May-08	2008	SD							
1399	18-May-08	2008	SD							
1407	18-May-08	2008	SD							
1413	18-May-08	2008	SD							
1416	18-May-08	2008	SD							
1397	18-May-08	2008	SD							
313	19-May-08	2008	ML							
324	19-May-08	2008	ML							
325	19-May-08	2008	ML							
327	19-May-08	2008	ML							
315	19-May-08	2008	ML							
328	19-May-08	2008	ML							
307	19-May-08	2008	ML							
306	19-May-08	2008	ML							
309	19-May-08	2008	ML							
316	19-May-08	2008	ML							
329	19-May-08	2008	ML							
334	19-May-08	2008	ML							
335	19-May-08	2008	ML							
311	19-May-08	2008	ML							
319	19-May-08	2008	ML							
321	19-May-08	2008	ML							
323	19-May-08	2008	ML							
326	19-May-08	2008	ML							
332	19-May-08	2008	ML							
336	19-May-08	2008	ML							
330	19-May-08	2008	ML							
331	19-May-08	2008	ML							
320	19-May-08	2008	ML							
314	19-May-08	2008	ML							
317	19-May-08	2008	ML							
333	19-May-08	2008	ML							
308	19-May-08	2008	ML							
310	19-May-08	2008	ML							
312	19-May-08	2008	ML							
322	19-May-08	2008	ML							
337	19-May-08	2008	ML							
318	19-May-08	2008	ML							
305	19-May-08	2008	ML							
523	22-May-08	2008	7M		0.078947	0.342105	0.078947	0.263158	0.01023	7.733333
521	22-May-08	2008	7M		0.078947	0.342105	0.078947	0.263158	0.01023	7.733333
528	22-May-08	2008	7M		0.078947	0.342105	0.078947	0.263158	0.01023	7.733333
494	22-May-08	2008	7M		0.078947	0.342105	0.078947	0.263158	0.01023	7.733333
492	22-May-08	2008	7M		0.078947	0.342105	0.078947	0.263158	0.01023	7.733333

493	22-May-08	2008	7M	0.078947	0.342105	0.078947	0.263158	0.01023	7.733333
491	22-May-08	2008	7M	0.078947	0.342105	0.078947	0.263158	0.01023	7.733333
497	22-May-08	2008	7M	0.078947	0.342105	0.078947	0.263158	0.01023	7.733333
514	22-May-08	2008	7M	0.078947	0.342105	0.078947	0.263158	0.01023	7.733333
515	22-May-08	2008	7M	0.078947	0.342105	0.078947	0.263158	0.01023	7.733333
496	22-May-08	2008	7M	0.078947	0.342105	0.078947	0.263158	0.01023	7.733333
502	22-May-08	2008	7M	0.078947	0.342105	0.078947	0.263158	0.01023	7.733333
503	22-May-08	2008	7M	0.078947	0.342105	0.078947	0.263158	0.01023	7.733333
486	22-May-08	2008	7M	0.078947	0.342105	0.078947	0.263158	0.01023	7.733333
504	22-May-08	2008	7M	0.078947	0.342105	0.078947	0.263158	0.01023	7.733333
505	22-May-08	2008	7M	0.078947	0.342105	0.078947	0.263158	0.01023	7.733333
500	22-May-08	2008	7M	0.078947	0.342105	0.078947	0.263158	0.01023	7.733333
522	22-May-08	2008	7M	0.078947	0.342105	0.078947	0.263158	0.01023	7.733333
487	22-May-08	2008	7M	0.078947	0.342105	0.078947	0.263158	0.01023	7.733333
489	22-May-08	2008	7M	0.078947	0.342105	0.078947	0.263158	0.01023	7.733333
506	22-May-08	2008	7M	0.078947	0.342105	0.078947	0.263158	0.01023	7.733333
507	22-May-08	2008	7M	0.078947	0.342105	0.078947	0.263158	0.01023	7.733333
513	22-May-08	2008	7M	0.078947	0.342105	0.078947	0.263158	0.01023	7.733333
516	22-May-08	2008	7M	0.078947	0.342105	0.078947	0.263158	0.01023	7.733333
519	22-May-08	2008	7M	0.078947	0.342105	0.078947	0.263158	0.01023	7.733333
488	22-May-08	2008	7M	0.078947	0.342105	0.078947	0.263158	0.01023	7.733333
495	22-May-08	2008	7M	0.078947	0.342105	0.078947	0.263158	0.01023	7.733333
508	22-May-08	2008	7M	0.078947	0.342105	0.078947	0.263158	0.01023	7.733333
520	22-May-08	2008	7M	0.078947	0.342105	0.078947	0.263158	0.01023	7.733333
524	22-May-08	2008	7M	0.078947	0.342105	0.078947	0.263158	0.01023	7.733333
490	22-May-08	2008	7M	0.078947	0.342105	0.078947	0.263158	0.01023	7.733333
509	22-May-08	2008	7M	0.078947	0.342105	0.078947	0.263158	0.01023	7.733333
510	22-May-08	2008	7M	0.078947	0.342105	0.078947	0.263158	0.01023	7.733333
511	22-May-08	2008	7M	0.078947	0.342105	0.078947	0.263158	0.01023	7.733333
526	22-May-08	2008	7M	0.078947	0.342105	0.078947	0.263158	0.01023	7.733333
512	22-May-08	2008	7M	0.078947	0.342105	0.078947	0.263158	0.01023	7.733333
517	22-May-08	2008	7M	0.078947	0.342105	0.078947	0.263158	0.01023	7.733333
529	22-May-08	2008	7M	0.078947	0.342105	0.078947	0.263158	0.01023	7.733333
525	22-May-08	2008	7M	0.078947	0.342105	0.078947	0.263158	0.01023	7.733333
527	22-May-08	2008	7M	0.078947	0.342105	0.078947	0.263158	0.01023	7.733333
498	22-May-08	2008	7M	0.078947	0.342105	0.078947	0.263158	0.01023	7.733333
499	22-May-08	2008	7M	0.078947	0.342105	0.078947	0.263158	0.01023	7.733333
485	22-May-08	2008	7M	0.078947	0.342105	0.078947	0.263158	0.01023	7.733333
501	22-May-08	2008	7M	0.078947	0.342105	0.078947	0.263158	0.01023	7.733333
518	22-May-08	2008	7M	0.078947	0.342105	0.078947	0.263158	0.01023	7.733333
530	22-May-08	2008	7M	0.078947	0.342105	0.078947	0.263158	0.01023	7.733333
484	22-May-08	2008	7M	0.078947	0.342105	0.078947	0.263158	0.01023	7.733333
804	25-May-08	2008	YY	0.14	0.24	0.08	0.02	0	0.65
806	25-May-08	2008	YY	0.14	0.24	0.08	0.02	0	0.65

813	25-May-08	2008	YY	0.14	0.24	0.08	0.02	0	0.65
811	25-May-08	2008	YY	0.14	0.24	0.08	0.02	0	0.65
812	25-May-08	2008	YY	0.14	0.24	0.08	0.02	0	0.65
808	25-May-08	2008	YY	0.14	0.24	0.08	0.02	0	0.65
809	25-May-08	2008	YY	0.14	0.24	0.08	0.02	0	0.65
810	25-May-08	2008	YY	0.14	0.24	0.08	0.02	0	0.65
816	25-May-08	2008	YY	0.14	0.24	0.08	0.02	0	0.65
814	25-May-08	2008	YY	0.14	0.24	0.08	0.02	0	0.65
817	25-May-08	2008	YY	0.14	0.24	0.08	0.02	0	0.65
805	25-May-08	2008	YY	0.14	0.24	0.08	0.02	0	0.65
803	25-May-08	2008	YY	0.14	0.24	0.08	0.02	0	0.65
807	25-May-08	2008	YY	0.14	0.24	0.08	0.02	0	0.65
815	25-May-08	2008	YY	0.14	0.24	0.08	0.02	0	0.65
818	25-May-08	2008	YY	0.14	0.24	0.08	0.02	0	0.65
802	25-May-08	2008	YY	0.14	0.24	0.08	0.02	0	0.65
863	28-May-08	2008	HH	0.161765	0.102941	0.014706	0.147059	0	0.615385
857	28-May-08	2008	HH	0.161765	0.102941	0.014706	0.147059	0	0.615385
860	28-May-08	2008	HH	0.161765	0.102941	0.014706	0.147059	0	0.615385
853	28-May-08	2008	HH	0.161765	0.102941	0.014706	0.147059	0	0.615385
854	28-May-08	2008	HH	0.161765	0.102941	0.014706	0.147059	0	0.615385
855	28-May-08	2008	HH	0.161765	0.102941	0.014706	0.147059	0	0.615385
858	28-May-08	2008	HH	0.161765	0.102941	0.014706	0.147059	0	0.615385
856	28-May-08	2008	HH	0.161765	0.102941	0.014706	0.147059	0	0.615385
852	28-May-08	2008	HH	0.161765	0.102941	0.014706	0.147059	0	0.615385
859	28-May-08	2008	HH	0.161765	0.102941	0.014706	0.147059	0	0.615385
849	28-May-08	2008	HH	0.161765	0.102941	0.014706	0.147059	0	0.615385
850	28-May-08	2008	HH	0.161765	0.102941	0.014706	0.147059	0	0.615385
851	28-May-08	2008	HH	0.161765	0.102941	0.014706	0.147059	0	0.615385
862	28-May-08	2008	HH	0.161765	0.102941	0.014706	0.147059	0	0.615385
864	28-May-08	2008	HH	0.161765	0.102941	0.014706	0.147059	0	0.615385
843	28-May-08	2008	HH	0.161765	0.102941	0.014706	0.147059	0	0.615385
845	28-May-08	2008	HH	0.161765	0.102941	0.014706	0.147059	0	0.615385
846	28-May-08	2008	HH	0.161765	0.102941	0.014706	0.147059	0	0.615385
847	28-May-08	2008	HH	0.161765	0.102941	0.014706	0.147059	0	0.615385
844	28-May-08	2008	HH	0.161765	0.102941	0.014706	0.147059	0	0.615385
861	28-May-08	2008	HH	0.161765	0.102941	0.014706	0.147059	0	0.615385
841	28-May-08	2008	HH	0.161765	0.102941	0.014706	0.147059	0	0.615385
842	28-May-08	2008	HH	0.161765	0.102941	0.014706	0.147059	0	0.615385
848	28-May-08	2008	HH	0.161765	0.102941	0.014706	0.147059	0	0.615385
865	28-May-08	2008	HH	0.161765	0.102941	0.014706	0.147059	0	0.615385
840	28-May-08	2008	HH	0.161765	0.102941	0.014706	0.147059	0	0.615385
1210	3-Jun-08	2008	D1	0.2	0.28	0.04	0.02	0.006036	0.8
1196	3-Jun-08	2008	D1	0.2	0.28	0.04	0.02	0.006036	0.8
1197	3-Jun-08	2008	D1	0.2	0.28	0.04	0.02	0.006036	0.8

1205	3-Jun-08	2008	D1	0.2	0.28	0.04	0.02	0.006036	0.8
1201	3-Jun-08	2008	D1	0.2	0.28	0.04	0.02	0.006036	0.8
1202	3-Jun-08	2008	D1	0.2	0.28	0.04	0.02	0.006036	0.8
1209	3-Jun-08	2008	D1	0.2	0.28	0.04	0.02	0.006036	0.8
1211	3-Jun-08	2008	D1	0.2	0.28	0.04	0.02	0.006036	0.8
1195	3-Jun-08	2008	D1	0.2	0.28	0.04	0.02	0.006036	0.8
1198	3-Jun-08	2008	D1	0.2	0.28	0.04	0.02	0.006036	0.8
1200	3-Jun-08	2008	D1	0.2	0.28	0.04	0.02	0.006036	0.8
1207	3-Jun-08	2008	D1	0.2	0.28	0.04	0.02	0.006036	0.8
1199	3-Jun-08	2008	D1	0.2	0.28	0.04	0.02	0.006036	0.8
1212	3-Jun-08	2008	D1	0.2	0.28	0.04	0.02	0.006036	0.8
1190	3-Jun-08	2008	D1	0.2	0.28	0.04	0.02	0.006036	0.8
1194	3-Jun-08	2008	D1	0.2	0.28	0.04	0.02	0.006036	0.8
1203	3-Jun-08	2008	D1	0.2	0.28	0.04	0.02	0.006036	0.8
1217	3-Jun-08	2008	D1	0.2	0.28	0.04	0.02	0.006036	0.8
1220	3-Jun-08	2008	D1	0.2	0.28	0.04	0.02	0.006036	0.8
1223	3-Jun-08	2008	D1	0.2	0.28	0.04	0.02	0.006036	0.8
1213	3-Jun-08	2008	D1	0.2	0.28	0.04	0.02	0.006036	0.8
1218	3-Jun-08	2008	D1	0.2	0.28	0.04	0.02	0.006036	0.8
1157	3-Jun-08	2008	D1	0.2	0.28	0.04	0.02	0.006036	0.8
1159	3-Jun-08	2008	D1	0.2	0.28	0.04	0.02	0.006036	0.8
1162	3-Jun-08	2008	D1	0.2	0.28	0.04	0.02	0.006036	0.8
1163	3-Jun-08	2008	D1	0.2	0.28	0.04	0.02	0.006036	0.8
1167	3-Jun-08	2008	D1	0.2	0.28	0.04	0.02	0.006036	0.8
1168	3-Jun-08	2008	D1	0.2	0.28	0.04	0.02	0.006036	0.8
1169	3-Jun-08	2008	D1	0.2	0.28	0.04	0.02	0.006036	0.8
1172	3-Jun-08	2008	D1	0.2	0.28	0.04	0.02	0.006036	0.8
1175	3-Jun-08	2008	D1	0.2	0.28	0.04	0.02	0.006036	0.8
1176	3-Jun-08	2008	D1	0.2	0.28	0.04	0.02	0.006036	0.8
1180	3-Jun-08	2008	D1	0.2	0.28	0.04	0.02	0.006036	0.8
1184	3-Jun-08	2008	D1	0.2	0.28	0.04	0.02	0.006036	0.8
1185	3-Jun-08	2008	D1	0.2	0.28	0.04	0.02	0.006036	0.8
1188	3-Jun-08	2008	D1	0.2	0.28	0.04	0.02	0.006036	0.8
1158	3-Jun-08	2008	D1	0.2	0.28	0.04	0.02	0.006036	0.8
1164	3-Jun-08	2008	D1	0.2	0.28	0.04	0.02	0.006036	0.8
1173	3-Jun-08	2008	D1	0.2	0.28	0.04	0.02	0.006036	0.8
1160	3-Jun-08	2008	D1	0.2	0.28	0.04	0.02	0.006036	0.8
1165	3-Jun-08	2008	D1	0.2	0.28	0.04	0.02	0.006036	0.8
1171	3-Jun-08	2008	D1	0.2	0.28	0.04	0.02	0.006036	0.8
1174	3-Jun-08	2008	D1	0.2	0.28	0.04	0.02	0.006036	0.8
1181	3-Jun-08	2008	D1	0.2	0.28	0.04	0.02	0.006036	0.8
1183	3-Jun-08	2008	D1	0.2	0.28	0.04	0.02	0.006036	0.8
1189	3-Jun-08	2008	D1	0.2	0.28	0.04	0.02	0.006036	0.8
1214	3-Jun-08	2008	D1	0.2	0.28	0.04	0.02	0.006036	0.8

1166	3-Jun-08	2008	D1	0.2	0.28	0.04	0.02	0.006036	0.8
1187	3-Jun-08	2008	D1	0.2	0.28	0.04	0.02	0.006036	0.8
1154	3-Jun-08	2008	D1	0.2	0.28	0.04	0.02	0.006036	0.8
1155	3-Jun-08	2008	D1	0.2	0.28	0.04	0.02	0.006036	0.8
1170	3-Jun-08	2008	D1	0.2	0.28	0.04	0.02	0.006036	0.8
1153	3-Jun-08	2008	D1	0.2	0.28	0.04	0.02	0.006036	0.8
1191	3-Jun-08	2008	D1	0.2	0.28	0.04	0.02	0.006036	0.8
1222	3-Jun-08	2008	D1	0.2	0.28	0.04	0.02	0.006036	0.8
1161	3-Jun-08	2008	D1	0.2	0.28	0.04	0.02	0.006036	0.8
1182	3-Jun-08	2008	D1	0.2	0.28	0.04	0.02	0.006036	0.8
1204	3-Jun-08	2008	D1	0.2	0.28	0.04	0.02	0.006036	0.8
1206	3-Jun-08	2008	D1	0.2	0.28	0.04	0.02	0.006036	0.8
1208	3-Jun-08	2008	D1	0.2	0.28	0.04	0.02	0.006036	0.8
1177	3-Jun-08	2008	D1	0.2	0.28	0.04	0.02	0.006036	0.8
1178	3-Jun-08	2008	D1	0.2	0.28	0.04	0.02	0.006036	0.8
1193	3-Jun-08	2008	D1	0.2	0.28	0.04	0.02	0.006036	0.8
1152	3-Jun-08	2008	D1	0.2	0.28	0.04	0.02	0.006036	0.8
1156	3-Jun-08	2008	D1	0.2	0.28	0.04	0.02	0.006036	0.8
1192	3-Jun-08	2008	D1	0.2	0.28	0.04	0.02	0.006036	0.8
1219	3-Jun-08	2008	D1	0.2	0.28	0.04	0.02	0.006036	0.8
1221	3-Jun-08	2008	D1	0.2	0.28	0.04	0.02	0.006036	0.8
1226	3-Jun-08	2008	D1	0.2	0.28	0.04	0.02	0.006036	0.8
1224	3-Jun-08	2008	D1	0.2	0.28	0.04	0.02	0.006036	0.8
1179	3-Jun-08	2008	D1	0.2	0.28	0.04	0.02	0.006036	0.8
1215	3-Jun-08	2008	D1	0.2	0.28	0.04	0.02	0.006036	0.8
1225	3-Jun-08	2008	D1	0.2	0.28	0.04	0.02	0.006036	0.8
1151	3-Jun-08	2008	D1	0.2	0.28	0.04	0.02	0.006036	0.8
1186	3-Jun-08	2008	D1	0.2	0.28	0.04	0.02	0.006036	0.8
1216	3-Jun-08	2008	D1	0.2	0.28	0.04	0.02	0.006036	0.8
1227	3-Jun-08	2008	D1	0.2	0.28	0.04	0.02	0.006036	0.8
1150	3-Jun-08	2008	D1	0.2	0.28	0.04	0.02	0.006036	0.8
1248	7-Jun-08	2008	LT1	0.925926	0.222222	0.037037	0.04	0	3.666667
1256	7-Jun-08	2008	LT1	0.925926	0.222222	0.037037	0.04	0	3.666667
1252	7-Jun-08	2008	LT1	0.925926	0.222222	0.037037	0.04	0	3.666667
1254	7-Jun-08	2008	LT1	0.925926	0.222222	0.037037	0.04	0	3.666667
1247	7-Jun-08	2008	LT1	0.925926	0.222222	0.037037	0.04	0	3.666667
1255	7-Jun-08	2008	LT1	0.925926	0.222222	0.037037	0.04	0	3.666667
1269	7-Jun-08	2008	LT1	0.925926	0.222222	0.037037	0.04	0	3.666667
1271	7-Jun-08	2008	LT1	0.925926	0.222222	0.037037	0.04	0	3.666667
1259	7-Jun-08	2008	LT1	0.925926	0.222222	0.037037	0.04	0	3.666667
1264	7-Jun-08	2008	LT1	0.925926	0.222222	0.037037	0.04	0	3.666667
1265	7-Jun-08	2008	LT1	0.925926	0.222222	0.037037	0.04	0	3.666667
1273	7-Jun-08	2008	LT1	0.925926	0.222222	0.037037	0.04	0	3.666667
1258	7-Jun-08	2008	LT1	0.925926	0.222222	0.037037	0.04	0	3.666667

1274	7-Jun-08	2008	LT1	0.925926	0.222222	0.037037	0.04	0	3.666667
1267	7-Jun-08	2008	LT1	0.925926	0.222222	0.037037	0.04	0	3.666667
1272	7-Jun-08	2008	LT1	0.925926	0.222222	0.037037	0.04	0	3.666667
1251	7-Jun-08	2008	LT1	0.925926	0.222222	0.037037	0.04	0	3.666667
1266	7-Jun-08	2008	LT1	0.925926	0.222222	0.037037	0.04	0	3.666667
1276	7-Jun-08	2008	LT1	0.925926	0.222222	0.037037	0.04	0	3.666667
1279	7-Jun-08	2008	LT1	0.925926	0.222222	0.037037	0.04	0	3.666667
1270	7-Jun-08	2008	LT1	0.925926	0.222222	0.037037	0.04	0	3.666667
1249	7-Jun-08	2008	LT1	0.925926	0.222222	0.037037	0.04	0	3.666667
1250	7-Jun-08	2008	LT1	0.925926	0.222222	0.037037	0.04	0	3.666667
1268	7-Jun-08	2008	LT1	0.925926	0.222222	0.037037	0.04	0	3.666667
1277	7-Jun-08	2008	LT1	0.925926	0.222222	0.037037	0.04	0	3.666667
1278	7-Jun-08	2008	LT1	0.925926	0.222222	0.037037	0.04	0	3.666667
1275	7-Jun-08	2008	LT1	0.925926	0.222222	0.037037	0.04	0	3.666667
1280	7-Jun-08	2008	LT1	0.925926	0.222222	0.037037	0.04	0	3.666667
1253	7-Jun-08	2008	LT1	0.925926	0.222222	0.037037	0.04	0	3.666667
1260	7-Jun-08	2008	LT1	0.925926	0.222222	0.037037	0.04	0	3.666667
1261	7-Jun-08	2008	LT1	0.925926	0.222222	0.037037	0.04	0	3.666667
1262	7-Jun-08	2008	LT1	0.925926	0.222222	0.037037	0.04	0	3.666667
1246	7-Jun-08	2008	LT1	0.925926	0.222222	0.037037	0.04	0	3.666667
1257	7-Jun-08	2008	LT1	0.925926	0.222222	0.037037	0.04	0	3.666667
1263	7-Jun-08	2008	LT1	0.925926	0.222222	0.037037	0.04	0	3.666667
1281	7-Jun-08	2008	LT1	0.925926	0.222222	0.037037	0.04	0	3.666667
1245	7-Jun-08	2008	LT1	0.925926	0.222222	0.037037	0.04	0	3.666667
1712	16-Jun-08	2008	ML						
1713	16-Jun-08	2008	ML						
1715	16-Jun-08	2008	ML						
1714	16-Jun-08	2008	ML						
1711	16-Jun-08	2008	ML						
1718	16-Jun-08	2008	ML						
1710	16-Jun-08	2008	ML						
1716	16-Jun-08	2008	ML						
1717	16-Jun-08	2008	ML						
1719	16-Jun-08	2008	ML						
1709	16-Jun-08	2008	ML						
2197	20-Jun-08	2008	D1	0.2	0.28	0.04	0.02	0.006036	0.8
2199	20-Jun-08	2008	D1	0.2	0.28	0.04	0.02	0.006036	0.8
2193	20-Jun-08	2008	D1	0.2	0.28	0.04	0.02	0.006036	0.8
2189	20-Jun-08	2008	D1	0.2	0.28	0.04	0.02	0.006036	0.8
2196	20-Jun-08	2008	D1	0.2	0.28	0.04	0.02	0.006036	0.8
2192	20-Jun-08	2008	D1	0.2	0.28	0.04	0.02	0.006036	0.8
2198	20-Jun-08	2008	D1	0.2	0.28	0.04	0.02	0.006036	0.8
2200	20-Jun-08	2008	D1	0.2	0.28	0.04	0.02	0.006036	0.8
2191	20-Jun-08	2008	D1	0.2	0.28	0.04	0.02	0.006036	0.8

2190	20-Jun-08	2008	D1	0.2	0.28	0.04	0.02	0.006036	0.8
2194	20-Jun-08	2008	D1	0.2	0.28	0.04	0.02	0.006036	0.8
2195	20-Jun-08	2008	D1	0.2	0.28	0.04	0.02	0.006036	0.8
2201	20-Jun-08	2008	D1	0.2	0.28	0.04	0.02	0.006036	0.8
2188	20-Jun-08	2008	D1	0.2	0.28	0.04	0.02	0.006036	0.8
1819	23-Jun-08	2008	YY	0.14	0.24	0.08	0.02	0	0.65
1818	23-Jun-08	2008	YY	0.14	0.24	0.08	0.02	0	0.65
1820	23-Jun-08	2008	YY	0.14	0.24	0.08	0.02	0	0.65
1823	23-Jun-08	2008	YY	0.14	0.24	0.08	0.02	0	0.65
1821	23-Jun-08	2008	YY	0.14	0.24	0.08	0.02	0	0.65
1817	23-Jun-08	2008	YY	0.14	0.24	0.08	0.02	0	0.65
1822	23-Jun-08	2008	YY	0.14	0.24	0.08	0.02	0	0.65
1824	23-Jun-08	2008	YY	0.14	0.24	0.08	0.02	0	0.65
1825	23-Jun-08	2008	YY	0.14	0.24	0.08	0.02	0	0.65
1816	23-Jun-08	2008	YY	0.14	0.24	0.08	0.02	0	0.65
1931	27-Jun-08	2008	LT1	0.925926	0.222222	0.037037	0.04	0	3.666667
1933	27-Jun-08	2008	LT1	0.925926	0.222222	0.037037	0.04	0	3.666667
1928	27-Jun-08	2008	LT1	0.925926	0.222222	0.037037	0.04	0	3.666667
1915	27-Jun-08	2008	LT1	0.925926	0.222222	0.037037	0.04	0	3.666667
1932	27-Jun-08	2008	LT1	0.925926	0.222222	0.037037	0.04	0	3.666667
1916	27-Jun-08	2008	LT1	0.925926	0.222222	0.037037	0.04	0	3.666667
1917	27-Jun-08	2008	LT1	0.925926	0.222222	0.037037	0.04	0	3.666667
1919	27-Jun-08	2008	LT1	0.925926	0.222222	0.037037	0.04	0	3.666667
1920	27-Jun-08	2008	LT1	0.925926	0.222222	0.037037	0.04	0	3.666667
1922	27-Jun-08	2008	LT1	0.925926	0.222222	0.037037	0.04	0	3.666667
1923	27-Jun-08	2008	LT1	0.925926	0.222222	0.037037	0.04	0	3.666667
1924	27-Jun-08	2008	LT1	0.925926	0.222222	0.037037	0.04	0	3.666667
1925	27-Jun-08	2008	LT1	0.925926	0.222222	0.037037	0.04	0	3.666667
1929	27-Jun-08	2008	LT1	0.925926	0.222222	0.037037	0.04	0	3.666667
1930	27-Jun-08	2008	LT1	0.925926	0.222222	0.037037	0.04	0	3.666667
1918	27-Jun-08	2008	LT1	0.925926	0.222222	0.037037	0.04	0	3.666667
1926	27-Jun-08	2008	LT1	0.925926	0.222222	0.037037	0.04	0	3.666667
1913	27-Jun-08	2008	LT1	0.925926	0.222222	0.037037	0.04	0	3.666667
1934	27-Jun-08	2008	LT1	0.925926	0.222222	0.037037	0.04	0	3.666667
1914	27-Jun-08	2008	LT1	0.925926	0.222222	0.037037	0.04	0	3.666667
1921	27-Jun-08	2008	LT1	0.925926	0.222222	0.037037	0.04	0	3.666667
1927	27-Jun-08	2008	LT1	0.925926	0.222222	0.037037	0.04	0	3.666667
1935	27-Jun-08	2008	LT1	0.925926	0.222222	0.037037	0.04	0	3.666667
1912	27-Jun-08	2008	LT1	0.925926	0.222222	0.037037	0.04	0	3.666667
2683	28-May-07	2007	D1	0.26	0.14	0.02	0.06	0.006036	0.8
2690	28-May-07	2007	D1	0.26	0.14	0.02	0.06	0.006036	0.8
2692	28-May-07	2007	D1	0.26	0.14	0.02	0.06	0.006036	0.8
2694	28-May-07	2007	D1	0.26	0.14	0.02	0.06	0.006036	0.8
2696	28-May-07	2007	D1	0.26	0.14	0.02	0.06	0.006036	0.8

2698	28-May-07	2007	D1	0.26	0.14	0.02	0.06	0.006036	0.8
2702	28-May-07	2007	D1	0.26	0.14	0.02	0.06	0.006036	0.8
2710	28-May-07	2007	D1	0.26	0.14	0.02	0.06	0.006036	0.8
2713	28-May-07	2007	D1	0.26	0.14	0.02	0.06	0.006036	0.8
2716	28-May-07	2007	D1	0.26	0.14	0.02	0.06	0.006036	0.8
2717	28-May-07	2007	D1	0.26	0.14	0.02	0.06	0.006036	0.8
2723	28-May-07	2007	D1	0.26	0.14	0.02	0.06	0.006036	0.8
2682	28-May-07	2007	D1	0.26	0.14	0.02	0.06	0.006036	0.8
2721	28-May-07	2007	D1	0.26	0.14	0.02	0.06	0.006036	0.8
2699	28-May-07	2007	D1	0.26	0.14	0.02	0.06	0.006036	0.8
2697	28-May-07	2007	D1	0.26	0.14	0.02	0.06	0.006036	0.8
2691	28-May-07	2007	D1	0.26	0.14	0.02	0.06	0.006036	0.8
2703	28-May-07	2007	D1	0.26	0.14	0.02	0.06	0.006036	0.8
2688	28-May-07	2007	D1	0.26	0.14	0.02	0.06	0.006036	0.8
2689	28-May-07	2007	D1	0.26	0.14	0.02	0.06	0.006036	0.8
2714	28-May-07	2007	D1	0.26	0.14	0.02	0.06	0.006036	0.8
2712	28-May-07	2007	D1	0.26	0.14	0.02	0.06	0.006036	0.8
2711	28-May-07	2007	D1	0.26	0.14	0.02	0.06	0.006036	0.8
2687	28-May-07	2007	D1	0.26	0.14	0.02	0.06	0.006036	0.8
2709	28-May-07	2007	D1	0.26	0.14	0.02	0.06	0.006036	0.8
2686	28-May-07	2007	D1	0.26	0.14	0.02	0.06	0.006036	0.8
2707	28-May-07	2007	D1	0.26	0.14	0.02	0.06	0.006036	0.8
2708	28-May-07	2007	D1	0.26	0.14	0.02	0.06	0.006036	0.8
2684	28-May-07	2007	D1	0.26	0.14	0.02	0.06	0.006036	0.8
2719	28-May-07	2007	D1	0.26	0.14	0.02	0.06	0.006036	0.8
2685	28-May-07	2007	D1	0.26	0.14	0.02	0.06	0.006036	0.8
2704	28-May-07	2007	D1	0.26	0.14	0.02	0.06	0.006036	0.8
2706	28-May-07	2007	D1	0.26	0.14	0.02	0.06	0.006036	0.8
2722	28-May-07	2007	D1	0.26	0.14	0.02	0.06	0.006036	0.8
2718	28-May-07	2007	D1	0.26	0.14	0.02	0.06	0.006036	0.8
2720	28-May-07	2007	D1	0.26	0.14	0.02	0.06	0.006036	0.8
2693	28-May-07	2007	D1	0.26	0.14	0.02	0.06	0.006036	0.8
2700	28-May-07	2007	D1	0.26	0.14	0.02	0.06	0.006036	0.8
2701	28-May-07	2007	D1	0.26	0.14	0.02	0.06	0.006036	0.8
2715	28-May-07	2007	D1	0.26	0.14	0.02	0.06	0.006036	0.8
2681	28-May-07	2007	D1	0.26	0.14	0.02	0.06	0.006036	0.8
2695	28-May-07	2007	D1	0.26	0.14	0.02	0.06	0.006036	0.8
2705	28-May-07	2007	D1	0.26	0.14	0.02	0.06	0.006036	0.8
1596	12-Jun-05	2005	SD	0.365079	0.095238	0.095238	0.380952	0.048387	2.5
1598	12-Jun-05	2005	SD	0.365079	0.095238	0.095238	0.380952	0.048387	2.5
1599	12-Jun-05	2005	SD	0.365079	0.095238	0.095238	0.380952	0.048387	2.5
1597	12-Jun-05	2005	SD	0.365079	0.095238	0.095238	0.380952	0.048387	2.5
219	6-Jun-06	2006	ML						
220	6-Jun-06	2006	ML						

222	6-Jun-06	2006	ML						
215	6-Jun-06	2006	ML						
218	6-Jun-06	2006	ML						
214	6-Jun-06	2006	ML						
223	6-Jun-06	2006	ML						
213	6-Jun-06	2006	ML						
216	6-Jun-06	2006	ML						
224	6-Jun-06	2006	ML						
212	6-Jun-06	2006	ML						
217	6-Jun-06	2006	ML						
221	6-Jun-06	2006	ML						
267	11-Jun-06	2006	D1	0.04	0.04	0.06	0.22	0.008386	0.75
268	11-Jun-06	2006	D1	0.04	0.04	0.06	0.22	0.008386	0.75
269	11-Jun-06	2006	D1	0.04	0.04	0.06	0.22	0.008386	0.75
83	18-May-06	2006	TO	0.075	0.1	0.05	0.225	0.005814	0.9375
84	18-May-06	2006	TO	0.075	0.1	0.05	0.225	0.005814	0.9375
82	18-May-06	2006	TO	0.075	0.1	0.05	0.225	0.005814	0.9375
85	18-May-06	2006	TO	0.075	0.1	0.05	0.225	0.005814	0.9375
89	18-May-06	2006	TO	0.075	0.1	0.05	0.225	0.005814	0.9375
86	18-May-06	2006	TO	0.075	0.1	0.05	0.225	0.005814	0.9375
87	18-May-06	2006	TO	0.075	0.1	0.05	0.225	0.005814	0.9375
88	18-May-06	2006	TO	0.075	0.1	0.05	0.225	0.005814	0.9375
90	18-May-06	2006	TO	0.075	0.1	0.05	0.225	0.005814	0.9375
103	28-May-06	2006	TO	0.075	0.1	0.05	0.225	0.005814	0.9375
102	28-May-06	2006	TO	0.075	0.1	0.05	0.225	0.005814	0.9375
101	28-May-06	2006	TO	0.075	0.1	0.05	0.225	0.005814	0.9375
109	28-May-06	2006	TO	0.075	0.1	0.05	0.225	0.005814	0.9375
104	28-May-06	2006	TO	0.075	0.1	0.05	0.225	0.005814	0.9375
105	28-May-06	2006	TO	0.075	0.1	0.05	0.225	0.005814	0.9375
106	28-May-06	2006	TO	0.075	0.1	0.05	0.225	0.005814	0.9375
108	28-May-06	2006	TO	0.075	0.1	0.05	0.225	0.005814	0.9375
	18-May-06	2006	TO	0.075	0.1	0.05	0.225	0.005814	0.9375
	18-May-06	2006	TO	0.075	0.1	0.05	0.225	0.005814	0.9375
	18-May-06	2006	TO	0.075	0.1	0.05	0.225	0.005814	0.9375
	18-May-06	2006	TO	0.075	0.1	0.05	0.225	0.005814	0.9375
	18-May-06	2006	TO	0.075	0.1	0.05	0.225	0.005814	0.9375
	18-May-06	2006	TO	0.075	0.1	0.05	0.225	0.005814	0.9375
	18-May-06	2006	TO	0.075	0.1	0.05	0.225	0.005814	0.9375
	18-May-06	2006	TO	0.075	0.1	0.05	0.225	0.005814	0.9375
	18-May-06	2006	TO	0.075	0.1	0.05	0.225	0.005814	0.9375
	28-May-06	2006	TO	0.075	0.1	0.05	0.225	0.005814	0.9375
	28-May-06	2006	TO	0.075	0.1	0.05	0.225	0.005814	0.9375
	28-May-06	2006	TO	0.075	0.1	0.05	0.225	0.005814	0.9375
	28-May-06	2006	TO	0.075	0.1	0.05	0.225	0.005814	0.9375

	28-May-06	2006	TO	0.075	0.1	0.05	0.225	0.005814	0.9375
	28-May-06	2006	TO	0.075	0.1	0.05	0.225	0.005814	0.9375
	28-May-06	2006	TO	0.075	0.1	0.05	0.225	0.005814	0.9375
	28-May-06	2006	TO	0.075	0.1	0.05	0.225	0.005814	0.9375
1383	5-Jun-05	2005	MM	0.220779	0.064935	0.025974	0.12987	0.044118	1.727273
1377	5-Jun-05	2005	MM	0.220779	0.064935	0.025974	0.12987	0.044118	1.727273
1378	5-Jun-05	2005	MM	0.220779	0.064935	0.025974	0.12987	0.044118	1.727273
1382	5-Jun-05	2005	MM	0.220779	0.064935	0.025974	0.12987	0.044118	1.727273
1384	5-Jun-05	2005	MM	0.220779	0.064935	0.025974	0.12987	0.044118	1.727273
1380	5-Jun-05	2005	MM	0.220779	0.064935	0.025974	0.12987	0.044118	1.727273
1385	5-Jun-05	2005	MM	0.220779	0.064935	0.025974	0.12987	0.044118	1.727273
1379	5-Jun-05	2005	MM	0.220779	0.064935	0.025974	0.12987	0.044118	1.727273
1381	5-Jun-05	2005	MM	0.220779	0.064935	0.025974	0.12987	0.044118	1.727273
1504	18-Jun-05	2005	RP	0.333333	0	0	0.047619	0.072115	2.25
1503	18-Jun-05	2005	RP	0.333333	0	0	0.047619	0.072115	2.25
1502	18-Jun-05	2005	RP	0.333333	0	0	0.047619	0.072115	2.25
1508	18-Jun-05	2005	RP	0.333333	0	0	0.047619	0.072115	2.25
1509	18-Jun-05	2005	RP	0.333333	0	0	0.047619	0.072115	2.25
1507	18-Jun-05	2005	RP	0.333333	0	0	0.047619	0.072115	2.25
1505	18-Jun-05	2005	RP	0.333333	0	0	0.047619	0.072115	2.25
1506	18-Jun-05	2005	RP	0.333333	0	0	0.047619	0.072115	2.25
1510	18-Jun-05	2005	RP	0.333333	0	0	0.047619	0.072115	2.25
200	4-Jun-06	2006	LT1	0.222222	0.111111	0	0.148148	0	4
201	4-Jun-06	2006	LT1	0.222222	0.111111	0	0.148148	0	4
202	4-Jun-06	2006	LT1	0.222222	0.111111	0	0.148148	0	4
205	4-Jun-06	2006	LT1	0.222222	0.111111	0	0.148148	0	4
207	4-Jun-06	2006	LT1	0.222222	0.111111	0	0.148148	0	4
203	4-Jun-06	2006	LT1	0.222222	0.111111	0	0.148148	0	4
199	4-Jun-06	2006	LT1	0.222222	0.111111	0	0.148148	0	4
204	4-Jun-06	2006	LT1	0.222222	0.111111	0	0.148148	0	4
206	4-Jun-06	2006	LT1	0.222222	0.111111	0	0.148148	0	4
	25-Jun-06	2006	LT1	0.222222	0.111111	0	0.148148	0	4
	25-Jun-06	2006	LT1	0.222222	0.111111	0	0.148148	0	4
	25-Jun-06	2006	LT1	0.222222	0.111111	0	0.148148	0	4
	25-Jun-06	2006	LT1	0.222222	0.111111	0	0.148148	0	4
	25-Jun-06	2006	LT1	0.222222	0.111111	0	0.148148	0	4
	25-Jun-06	2006	LT1	0.222222	0.111111	0	0.148148	0	4
	25-Jun-06	2006	LT1	0.222222	0.111111	0	0.148148	0	4
	25-Jun-06	2006	LT1	0.222222	0.111111	0	0.148148	0	4
667	9-May-07	2007	TO	0.175	0.125	0.175	0	0.00289	0.875
669	9-May-07	2007	TO	0.175	0.125	0.175	0	0.00289	0.875
670	9-May-07	2007	TO	0.175	0.125	0.175	0	0.00289	0.875
668	9-May-07	2007	TO	0.175	0.125	0.175	0	0.00289	0.875
666	9-May-07	2007	TO	0.175	0.125	0.175	0	0.00289	0.875

671	9-May-07	2007	TO	0.175	0.125	0.175	0	0.00289	0.875
672	9-May-07	2007	TO	0.175	0.125	0.175	0	0.00289	0.875
673	9-May-07	2007	TO	0.175	0.125	0.175	0	0.00289	0.875
674	9-May-07	2007	TO	0.175	0.125	0.175	0	0.00289	0.875
675	9-May-07	2007	TO	0.175	0.125	0.175	0	0.00289	0.875
676	9-May-07	2007	TO	0.175	0.125	0.175	0	0.00289	0.875
663	9-May-07	2007	TO	0.175	0.125	0.175	0	0.00289	0.875
662	9-May-07	2007	TO	0.175	0.125	0.175	0	0.00289	0.875
664	9-May-07	2007	TO	0.175	0.125	0.175	0	0.00289	0.875
665	9-May-07	2007	TO	0.175	0.125	0.175	0	0.00289	0.875
311	29-May-04	2004	RP	0.095238	0.142857	0.095238	0.071429	0.323383	2
312	29-May-04	2004	RP	0.095238	0.142857	0.095238	0.071429	0.323383	2
313	29-May-04	2004	RP	0.095238	0.142857	0.095238	0.071429	0.323383	2
286	11-Jun-04	2004	SC	0.174312	0.009174	0.06422	0.018349	0.103343	6.846154
285	11-Jun-04	2004	SC	0.174312	0.009174	0.06422	0.018349	0.103343	6.846154
287	11-Jun-04	2004	SC	0.174312	0.009174	0.06422	0.018349	0.103343	6.846154
1302	1-Jun-05	2005	D1/D2						
1661	14-Jun-05	2005	7M	0.264151	0.09434	0	0.301887	0.01519	8.266667
1658	14-Jun-05	2005	7M	0.264151	0.09434	0	0.301887	0.01519	8.266667
1657	14-Jun-05	2005	7M	0.264151	0.09434	0	0.301887	0.01519	8.266667
1656	14-Jun-05	2005	7M	0.264151	0.09434	0	0.301887	0.01519	8.266667
1659	14-Jun-05	2005	7M	0.264151	0.09434	0	0.301887	0.01519	8.266667
1660	14-Jun-05	2005	7M	0.264151	0.09434	0	0.301887	0.01519	8.266667
1655	14-Jun-05	2005	7M	0.264151	0.09434	0	0.301887	0.01519	8.266667
1666	14-Jun-05	2005	7M	0.264151	0.09434	0	0.301887	0.01519	8.266667
1662	14-Jun-05	2005	7M	0.264151	0.09434	0	0.301887	0.01519	8.266667
1665	14-Jun-05	2005	7M	0.264151	0.09434	0	0.301887	0.01519	8.266667
1664	14-Jun-05	2005	7M	0.264151	0.09434	0	0.301887	0.01519	8.266667
1663	14-Jun-05	2005	7M	0.264151	0.09434	0	0.301887	0.01519	8.266667
352	30-May-04	2004	SD	0.412698	0.301587	0.063492	0.634921	0.097297	2.642857
353	30-May-04	2004	SD	0.412698	0.301587	0.063492	0.634921	0.097297	2.642857
354	30-May-04	2004	SD	0.412698	0.301587	0.063492	0.634921	0.097297	2.642857
355	30-May-04	2004	SD	0.412698	0.301587	0.063492	0.634921	0.097297	2.642857
356	30-May-04	2004	SD	0.412698	0.301587	0.063492	0.634921	0.097297	2.642857
414	2-Jun-04	2004	7M	0.283019	0.471698	0.113208	0.679245	0.035088	8.333333
418	2-Jun-04	2004	7M	0.283019	0.471698	0.113208	0.679245	0.035088	8.333333
416	2-Jun-04	2004	7M	0.283019	0.471698	0.113208	0.679245	0.035088	8.333333
415	2-Jun-04	2004	7M	0.283019	0.471698	0.113208	0.679245	0.035088	8.333333
417	2-Jun-04	2004	7M	0.283019	0.471698	0.113208	0.679245	0.035088	8.333333
31	8-May-05	2005	SC	0.183486	0	0.073394	0	0.006329	6.769231
33	8-May-05	2005	SC	0.183486	0	0.073394	0	0.006329	6.769231
32	8-May-05	2005	SC	0.183486	0	0.073394	0	0.006329	6.769231
29	8-May-05	2005	SC	0.183486	0	0.073394	0	0.006329	6.769231
30	8-May-05	2005	SC	0.183486	0	0.073394	0	0.006329	6.769231

34	8-May-05	2005	SC	0.183486	0	0.073394	0	0.006329	6.769231
27	8-May-05	2005	SC	0.183486	0	0.073394	0	0.006329	6.769231
28	8-May-05	2005	SC	0.183486	0	0.073394	0	0.006329	6.769231
217	14-May-05	2005	SW	0.264151	0.113208	0.018868	0.169811	0.044444	1.4
222	14-May-05	2005	SW	0.264151	0.113208	0.018868	0.169811	0.044444	1.4
223	14-May-05	2005	SW	0.264151	0.113208	0.018868	0.169811	0.044444	1.4
216	14-May-05	2005	SW	0.264151	0.113208	0.018868	0.169811	0.044444	1.4
218	14-May-05	2005	SW	0.264151	0.113208	0.018868	0.169811	0.044444	1.4
219	14-May-05	2005	SW	0.264151	0.113208	0.018868	0.169811	0.044444	1.4
220	14-May-05	2005	SW	0.264151	0.113208	0.018868	0.169811	0.044444	1.4
215	14-May-05	2005	SW	0.264151	0.113208	0.018868	0.169811	0.044444	1.4
221	14-May-05	2005	SW	0.264151	0.113208	0.018868	0.169811	0.044444	1.4
213	14-May-05	2005	SW	0.264151	0.113208	0.018868	0.169811	0.044444	1.4
214	14-May-05	2005	SW	0.264151	0.113208	0.018868	0.169811	0.044444	1.4
224	14-May-05	2005	SW	0.264151	0.113208	0.018868	0.169811	0.044444	1.4
212	14-May-05	2005	SW	0.264151	0.113208	0.018868	0.169811	0.044444	1.4
245	15-May-05	2005	TO	0.285714	0.142857	0.017857	0.142857	0.014577	1
244	15-May-05	2005	TO	0.285714	0.142857	0.017857	0.142857	0.014577	1
242	15-May-05	2005	TO	0.285714	0.142857	0.017857	0.142857	0.014577	1
239	15-May-05	2005	TO	0.285714	0.142857	0.017857	0.142857	0.014577	1
240	15-May-05	2005	TO	0.285714	0.142857	0.017857	0.142857	0.014577	1
238	15-May-05	2005	TO	0.285714	0.142857	0.017857	0.142857	0.014577	1
241	15-May-05	2005	TO	0.285714	0.142857	0.017857	0.142857	0.014577	1
243	15-May-05	2005	TO	0.285714	0.142857	0.017857	0.142857	0.014577	1
296	16-May-05	2005	RL	0.465517	0.103448	0	0.137931	0.108209	0.333333
297	16-May-05	2005	RL	0.465517	0.103448	0	0.137931	0.108209	0.333333
295	16-May-05	2005	RL	0.465517	0.103448	0	0.137931	0.108209	0.333333
300	16-May-05	2005	RL	0.465517	0.103448	0	0.137931	0.108209	0.333333
289	16-May-05	2005	RL	0.465517	0.103448	0	0.137931	0.108209	0.333333
299	16-May-05	2005	RL	0.465517	0.103448	0	0.137931	0.108209	0.333333
287	16-May-05	2005	RL	0.465517	0.103448	0	0.137931	0.108209	0.333333
302	16-May-05	2005	RL	0.465517	0.103448	0	0.137931	0.108209	0.333333
303	16-May-05	2005	RL	0.465517	0.103448	0	0.137931	0.108209	0.333333
298	16-May-05	2005	RL	0.465517	0.103448	0	0.137931	0.108209	0.333333
291	16-May-05	2005	RL	0.465517	0.103448	0	0.137931	0.108209	0.333333
288	16-May-05	2005	RL	0.465517	0.103448	0	0.137931	0.108209	0.333333
294	16-May-05	2005	RL	0.465517	0.103448	0	0.137931	0.108209	0.333333
304	16-May-05	2005	RL	0.465517	0.103448	0	0.137931	0.108209	0.333333
301	16-May-05	2005	RL	0.465517	0.103448	0	0.137931	0.108209	0.333333
290	16-May-05	2005	RL	0.465517	0.103448	0	0.137931	0.108209	0.333333
293	16-May-05	2005	RL	0.465517	0.103448	0	0.137931	0.108209	0.333333
292	16-May-05	2005	RL	0.465517	0.103448	0	0.137931	0.108209	0.333333
374	17-May-05	2005	RC	0.185185	0	0	0.037037	0.155172	2.875
373	17-May-05	2005	RC	0.185185	0	0	0.037037	0.155172	2.875

372	17-May-05	2005	RC	0.185185	0	0	0.037037	0.155172	2.875
369	17-May-05	2005	RC	0.185185	0	0	0.037037	0.155172	2.875
370	17-May-05	2005	RC	0.185185	0	0	0.037037	0.155172	2.875
371	17-May-05	2005	RC	0.185185	0	0	0.037037	0.155172	2.875
368	17-May-05	2005	RC	0.185185	0	0	0.037037	0.155172	2.875
375	17-May-05	2005	RC	0.185185	0	0	0.037037	0.155172	2.875
367	17-May-05	2005	RC	0.185185	0	0	0.037037	0.155172	2.875
376	17-May-05	2005	RC	0.185185	0	0	0.037037	0.155172	2.875
377	17-May-05	2005	RC	0.185185	0	0	0.037037	0.155172	2.875
326	17-May-05	2005	RC	0.185185	0	0	0.037037	0.155172	2.875
325	17-May-05	2005	RC	0.185185	0	0	0.037037	0.155172	2.875
324	17-May-05	2005	RC	0.185185	0	0	0.037037	0.155172	2.875
322	17-May-05	2005	RC	0.185185	0	0	0.037037	0.155172	2.875
328	17-May-05	2005	RC	0.185185	0	0	0.037037	0.155172	2.875
323	17-May-05	2005	RC	0.185185	0	0	0.037037	0.155172	2.875
327	17-May-05	2005	RC	0.185185	0	0	0.037037	0.155172	2.875
320	17-May-05	2005	RC	0.185185	0	0	0.037037	0.155172	2.875
321	17-May-05	2005	RC	0.185185	0	0	0.037037	0.155172	2.875
383	17-May-05	2005	SD	0.365079	0.095238	0.095238	0.380952	0.048387	2.5
384	17-May-05	2005	SD	0.365079	0.095238	0.095238	0.380952	0.048387	2.5
382	17-May-05	2005	SD	0.365079	0.095238	0.095238	0.380952	0.048387	2.5
378	17-May-05	2005	SD	0.365079	0.095238	0.095238	0.380952	0.048387	2.5
381	17-May-05	2005	SD	0.365079	0.095238	0.095238	0.380952	0.048387	2.5
379	17-May-05	2005	SD	0.365079	0.095238	0.095238	0.380952	0.048387	2.5
380	17-May-05	2005	SD	0.365079	0.095238	0.095238	0.380952	0.048387	2.5
427	19-May-05	2005	ML						
428	19-May-05	2005	ML						
426	19-May-05	2005	ML						
425	19-May-05	2005	ML						
424	19-May-05	2005	ML						
429	19-May-05	2005	ML						
423	19-May-05	2005	ML						
503	20-May-05	2005	SHAC	0.076923	0.153846	0	0.384615	0.026042	2.090909
505	20-May-05	2005	SHAC	0.076923	0.153846	0	0.384615	0.026042	2.090909
507	20-May-05	2005	SHAC	0.076923	0.153846	0	0.384615	0.026042	2.090909
502	20-May-05	2005	SHAC	0.076923	0.153846	0	0.384615	0.026042	2.090909
508	20-May-05	2005	SHAC	0.076923	0.153846	0	0.384615	0.026042	2.090909
509	20-May-05	2005	SHAC	0.076923	0.153846	0	0.384615	0.026042	2.090909
504	20-May-05	2005	SHAC	0.076923	0.153846	0	0.384615	0.026042	2.090909
506	20-May-05	2005	SHAC	0.076923	0.153846	0	0.384615	0.026042	2.090909
593	21-May-05	2005	SC	0.183486	0	0.073394	0	0.006329	6.769231
592	21-May-05	2005	SC	0.183486	0	0.073394	0	0.006329	6.769231
595	21-May-05	2005	SC	0.183486	0	0.073394	0	0.006329	6.769231
594	21-May-05	2005	SC	0.183486	0	0.073394	0	0.006329	6.769231

591	21-May-05	2005	SC	0.183486	0	0.073394	0	0.006329	6.769231
590	21-May-05	2005	SC	0.183486	0	0.073394	0	0.006329	6.769231
596	21-May-05	2005	SC	0.183486	0	0.073394	0	0.006329	6.769231
597	21-May-05	2005	SC	0.183486	0	0.073394	0	0.006329	6.769231
589	21-May-05	2005	SC	0.183486	0	0.073394	0	0.006329	6.769231
601	21-May-05	2005	SC	0.183486	0	0.073394	0	0.006329	6.769231
605	21-May-05	2005	SC	0.183486	0	0.073394	0	0.006329	6.769231
609	21-May-05	2005	SC	0.183486	0	0.073394	0	0.006329	6.769231
602	21-May-05	2005	SC	0.183486	0	0.073394	0	0.006329	6.769231
606	21-May-05	2005	SC	0.183486	0	0.073394	0	0.006329	6.769231
610	21-May-05	2005	SC	0.183486	0	0.073394	0	0.006329	6.769231
600	21-May-05	2005	SC	0.183486	0	0.073394	0	0.006329	6.769231
604	21-May-05	2005	SC	0.183486	0	0.073394	0	0.006329	6.769231
608	21-May-05	2005	SC	0.183486	0	0.073394	0	0.006329	6.769231
603	21-May-05	2005	SC	0.183486	0	0.073394	0	0.006329	6.769231
607	21-May-05	2005	SC	0.183486	0	0.073394	0	0.006329	6.769231
611	21-May-05	2005	SC	0.183486	0	0.073394	0	0.006329	6.769231
659	21-May-05	2005	DE						
663	21-May-05	2005	DE						
664	21-May-05	2005	DE						
660	21-May-05	2005	DE						
658	21-May-05	2005	DE						
656	21-May-05	2005	DE						
657	21-May-05	2005	DE						
665	21-May-05	2005	DE						
662	21-May-05	2005	DE						
661	21-May-05	2005	DE						
655	21-May-05	2005	DE						
669	22-May-05	2005	SW	0.264151	0.113208	0.018868	0.169811	0.044444	1.4
668	22-May-05	2005	SW	0.264151	0.113208	0.018868	0.169811	0.044444	1.4
670	22-May-05	2005	SW	0.264151	0.113208	0.018868	0.169811	0.044444	1.4
671	22-May-05	2005	SW	0.264151	0.113208	0.018868	0.169811	0.044444	1.4
667	22-May-05	2005	SW	0.264151	0.113208	0.018868	0.169811	0.044444	1.4
672	22-May-05	2005	SW	0.264151	0.113208	0.018868	0.169811	0.044444	1.4
673	22-May-05	2005	SW	0.264151	0.113208	0.018868	0.169811	0.044444	1.4
666	22-May-05	2005	SW	0.264151	0.113208	0.018868	0.169811	0.044444	1.4
690	22-May-05	2005	TO	0.285714	0.142857	0.017857	0.142857	0.014577	1
692	23-May-05	2005	RL	0.465517	0.103448	0	0.137931	0.108209	0.333333
691	23-May-05	2005	RL	0.465517	0.103448	0	0.137931	0.108209	0.333333
696	23-May-05	2005	RL	0.465517	0.103448	0	0.137931	0.108209	0.333333
694	23-May-05	2005	RL	0.465517	0.103448	0	0.137931	0.108209	0.333333
695	23-May-05	2005	RL	0.465517	0.103448	0	0.137931	0.108209	0.333333
693	23-May-05	2005	RL	0.465517	0.103448	0	0.137931	0.108209	0.333333
697	23-May-05	2005	RL	0.465517	0.103448	0	0.137931	0.108209	0.333333

727	25-May-05	2005 SD	0.365079	0.095238	0.095238	0.380952	0.048387	2.5
729	25-May-05	2005 SD	0.365079	0.095238	0.095238	0.380952	0.048387	2.5
726	25-May-05	2005 SD	0.365079	0.095238	0.095238	0.380952	0.048387	2.5
725	25-May-05	2005 SD	0.365079	0.095238	0.095238	0.380952	0.048387	2.5
728	25-May-05	2005 SD	0.365079	0.095238	0.095238	0.380952	0.048387	2.5
723	25-May-05	2005 SD	0.365079	0.095238	0.095238	0.380952	0.048387	2.5
724	25-May-05	2005 SD	0.365079	0.095238	0.095238	0.380952	0.048387	2.5
730	25-May-05	2005 SD	0.365079	0.095238	0.095238	0.380952	0.048387	2.5
732	25-May-05	2005 SD	0.365079	0.095238	0.095238	0.380952	0.048387	2.5
731	25-May-05	2005 SD	0.365079	0.095238	0.095238	0.380952	0.048387	2.5
734	25-May-05	2005 SD	0.365079	0.095238	0.095238	0.380952	0.048387	2.5
735	25-May-05	2005 SD	0.365079	0.095238	0.095238	0.380952	0.048387	2.5
721	25-May-05	2005 SD	0.365079	0.095238	0.095238	0.380952	0.048387	2.5
722	25-May-05	2005 SD	0.365079	0.095238	0.095238	0.380952	0.048387	2.5
733	25-May-05	2005 SD	0.365079	0.095238	0.095238	0.380952	0.048387	2.5
779	25-May-05	2005 SD	0.365079	0.095238	0.095238	0.380952	0.048387	2.5
781	25-May-05	2005 SD	0.365079	0.095238	0.095238	0.380952	0.048387	2.5
778	25-May-05	2005 SD	0.365079	0.095238	0.095238	0.380952	0.048387	2.5
777	25-May-05	2005 SD	0.365079	0.095238	0.095238	0.380952	0.048387	2.5
776	25-May-05	2005 SD	0.365079	0.095238	0.095238	0.380952	0.048387	2.5
784	25-May-05	2005 SD	0.365079	0.095238	0.095238	0.380952	0.048387	2.5
786	25-May-05	2005 SD	0.365079	0.095238	0.095238	0.380952	0.048387	2.5
787	25-May-05	2005 SD	0.365079	0.095238	0.095238	0.380952	0.048387	2.5
775	25-May-05	2005 SD	0.365079	0.095238	0.095238	0.380952	0.048387	2.5
788	25-May-05	2005 SD	0.365079	0.095238	0.095238	0.380952	0.048387	2.5
789	25-May-05	2005 SD	0.365079	0.095238	0.095238	0.380952	0.048387	2.5
771	25-May-05	2005 SD	0.365079	0.095238	0.095238	0.380952	0.048387	2.5
772	25-May-05	2005 SD	0.365079	0.095238	0.095238	0.380952	0.048387	2.5
782	25-May-05	2005 SD	0.365079	0.095238	0.095238	0.380952	0.048387	2.5
770	25-May-05	2005 SD	0.365079	0.095238	0.095238	0.380952	0.048387	2.5
780	25-May-05	2005 SD	0.365079	0.095238	0.095238	0.380952	0.048387	2.5
774	25-May-05	2005 SD	0.365079	0.095238	0.095238	0.380952	0.048387	2.5
785	25-May-05	2005 SD	0.365079	0.095238	0.095238	0.380952	0.048387	2.5
773	25-May-05	2005 SD	0.365079	0.095238	0.095238	0.380952	0.048387	2.5
783	25-May-05	2005 SD	0.365079	0.095238	0.095238	0.380952	0.048387	2.5
871	26-May-05	2005 SD	0.365079	0.095238	0.095238	0.380952	0.048387	2.5
872	26-May-05	2005 SD	0.365079	0.095238	0.095238	0.380952	0.048387	2.5
864	26-May-05	2005 SD	0.365079	0.095238	0.095238	0.380952	0.048387	2.5
865	26-May-05	2005 SD	0.365079	0.095238	0.095238	0.380952	0.048387	2.5
866	26-May-05	2005 SD	0.365079	0.095238	0.095238	0.380952	0.048387	2.5
867	26-May-05	2005 SD	0.365079	0.095238	0.095238	0.380952	0.048387	2.5
870	26-May-05	2005 SD	0.365079	0.095238	0.095238	0.380952	0.048387	2.5
863	26-May-05	2005 SD	0.365079	0.095238	0.095238	0.380952	0.048387	2.5
868	26-May-05	2005 SD	0.365079	0.095238	0.095238	0.380952	0.048387	2.5

869	26-May-05	2005	SD	0.365079	0.095238	0.095238	0.380952	0.048387	2.5
1215	26-May-05	2005	SD	0.365079	0.095238	0.095238	0.380952	0.048387	2.5
1238	28-May-05	2005	YY	0.114286	0.071429	0	0.342857	0	0.65
1242	28-May-05	2005	YY	0.114286	0.071429	0	0.342857	0	0.65
1241	28-May-05	2005	YY	0.114286	0.071429	0	0.342857	0	0.65
1240	28-May-05	2005	YY	0.114286	0.071429	0	0.342857	0	0.65
1249	28-May-05	2005	YY	0.114286	0.071429	0	0.342857	0	0.65
1245	28-May-05	2005	YY	0.114286	0.071429	0	0.342857	0	0.65
1246	28-May-05	2005	YY	0.114286	0.071429	0	0.342857	0	0.65
1247	28-May-05	2005	YY	0.114286	0.071429	0	0.342857	0	0.65
1244	28-May-05	2005	YY	0.114286	0.071429	0	0.342857	0	0.65
1248	28-May-05	2005	YY	0.114286	0.071429	0	0.342857	0	0.65
1239	28-May-05	2005	YY	0.114286	0.071429	0	0.342857	0	0.65
1243	28-May-05	2005	YY	0.114286	0.071429	0	0.342857	0	0.65
1254	28-May-05	2005	MG	0.142857	0.128571	0	0.071429	0.043738	5.65
1255	28-May-05	2005	MG	0.142857	0.128571	0	0.071429	0.043738	5.65
1250	28-May-05	2005	MG	0.142857	0.128571	0	0.071429	0.043738	5.65
1251	28-May-05	2005	MG	0.142857	0.128571	0	0.071429	0.043738	5.65
1253	28-May-05	2005	MG	0.142857	0.128571	0	0.071429	0.043738	5.65
1256	28-May-05	2005	MG	0.142857	0.128571	0	0.071429	0.043738	5.65
1257	28-May-05	2005	MG	0.142857	0.128571	0	0.071429	0.043738	5.65
1252	28-May-05	2005	MG	0.142857	0.128571	0	0.071429	0.043738	5.65
1278	28-May-05	2005	MG	0.142857	0.128571	0	0.071429	0.043738	5.65
1279	28-May-05	2005	MG	0.142857	0.128571	0	0.071429	0.043738	5.65
1280	28-May-05	2005	MG	0.142857	0.128571	0	0.071429	0.043738	5.65
1281	28-May-05	2005	MG	0.142857	0.128571	0	0.071429	0.043738	5.65
1282	28-May-05	2005	MG	0.142857	0.128571	0	0.071429	0.043738	5.65
1283	28-May-05	2005	MG	0.142857	0.128571	0	0.071429	0.043738	5.65
1284	28-May-05	2005	MG	0.142857	0.128571	0	0.071429	0.043738	5.65
934	29-May-05	2005	RP	0.333333	0	0	0.047619	0.072115	2.25
936	29-May-05	2005	RP	0.333333	0	0	0.047619	0.072115	2.25
935	29-May-05	2005	RP	0.333333	0	0	0.047619	0.072115	2.25
933	29-May-05	2005	RP	0.333333	0	0	0.047619	0.072115	2.25
937	29-May-05	2005	RP	0.333333	0	0	0.047619	0.072115	2.25
930	29-May-05	2005	RP	0.333333	0	0	0.047619	0.072115	2.25
931	29-May-05	2005	RP	0.333333	0	0	0.047619	0.072115	2.25
932	29-May-05	2005	RP	0.333333	0	0	0.047619	0.072115	2.25
1285	30-May-05	2005	LT1	0.393939	0.030303	0	0.242424	0	4.444444
1286	30-May-05	2005	LT1	0.393939	0.030303	0	0.242424	0	4.444444
970	30-May-05	2005	LT1	0.393939	0.030303	0	0.242424	0	4.444444
972	30-May-05	2005	LT1	0.393939	0.030303	0	0.242424	0	4.444444
965	30-May-05	2005	LT1	0.393939	0.030303	0	0.242424	0	4.444444
964	30-May-05	2005	LT1	0.393939	0.030303	0	0.242424	0	4.444444
966	30-May-05	2005	LT1	0.393939	0.030303	0	0.242424	0	4.444444

962	30-May-05	2005	LT1	0.393939	0.030303	0	0.242424	0	4.444444
963	30-May-05	2005	LT1	0.393939	0.030303	0	0.242424	0	4.444444
961	30-May-05	2005	LT1	0.393939	0.030303	0	0.242424	0	4.444444
958	30-May-05	2005	LT1	0.393939	0.030303	0	0.242424	0	4.444444
967	30-May-05	2005	LT1	0.393939	0.030303	0	0.242424	0	4.444444
971	30-May-05	2005	LT1	0.393939	0.030303	0	0.242424	0	4.444444
969	30-May-05	2005	LT1	0.393939	0.030303	0	0.242424	0	4.444444
960	30-May-05	2005	LT1	0.393939	0.030303	0	0.242424	0	4.444444
968	30-May-05	2005	LT1	0.393939	0.030303	0	0.242424	0	4.444444
959	30-May-05	2005	LT1	0.393939	0.030303	0	0.242424	0	4.444444
998	1-Jun-05	2005	D1/D2						
997	1-Jun-05	2005	D1/D2						
1329	4-Jun-05	2005	SC	0.183486	0	0.073394	0	0.006329	6.769231
1325	4-Jun-05	2005	SC	0.183486	0	0.073394	0	0.006329	6.769231
1326	4-Jun-05	2005	SC	0.183486	0	0.073394	0	0.006329	6.769231
1327	4-Jun-05	2005	SC	0.183486	0	0.073394	0	0.006329	6.769231
1324	4-Jun-05	2005	SC	0.183486	0	0.073394	0	0.006329	6.769231
1328	4-Jun-05	2005	SC	0.183486	0	0.073394	0	0.006329	6.769231
1330	4-Jun-05	2005	SC	0.183486	0	0.073394	0	0.006329	6.769231
1331	4-Jun-05	2005	SC	0.183486	0	0.073394	0	0.006329	6.769231
1332	4-Jun-05	2005	SC	0.183486	0	0.073394	0	0.006329	6.769231
1038	5-Jun-05	2005	SHAC	0.076923	0.153846	0	0.384615	0.026042	2.090909
1042	5-Jun-05	2005	SHAC	0.076923	0.153846	0	0.384615	0.026042	2.090909
1048	5-Jun-05	2005	SHAC	0.076923	0.153846	0	0.384615	0.026042	2.090909
1040	5-Jun-05	2005	SHAC	0.076923	0.153846	0	0.384615	0.026042	2.090909
1039	5-Jun-05	2005	SHAC	0.076923	0.153846	0	0.384615	0.026042	2.090909
1041	5-Jun-05	2005	SHAC	0.076923	0.153846	0	0.384615	0.026042	2.090909
1043	5-Jun-05	2005	SHAC	0.076923	0.153846	0	0.384615	0.026042	2.090909
1044	5-Jun-05	2005	SHAC	0.076923	0.153846	0	0.384615	0.026042	2.090909
1047	5-Jun-05	2005	SHAC	0.076923	0.153846	0	0.384615	0.026042	2.090909
1049	5-Jun-05	2005	SHAC	0.076923	0.153846	0	0.384615	0.026042	2.090909
1046	5-Jun-05	2005	SHAC	0.076923	0.153846	0	0.384615	0.026042	2.090909
1045	5-Jun-05	2005	SHAC	0.076923	0.153846	0	0.384615	0.026042	2.090909
1392	5-Jun-05	2005	MM	0.220779	0.064935	0.025974	0.12987	0.044118	1.727273
1386	5-Jun-05	2005	MM	0.220779	0.064935	0.025974	0.12987	0.044118	1.727273
1387	5-Jun-05	2005	MM	0.220779	0.064935	0.025974	0.12987	0.044118	1.727273
1391	5-Jun-05	2005	MM	0.220779	0.064935	0.025974	0.12987	0.044118	1.727273
1393	5-Jun-05	2005	MM	0.220779	0.064935	0.025974	0.12987	0.044118	1.727273
1389	5-Jun-05	2005	MM	0.220779	0.064935	0.025974	0.12987	0.044118	1.727273
1394	5-Jun-05	2005	MM	0.220779	0.064935	0.025974	0.12987	0.044118	1.727273
1388	5-Jun-05	2005	MM	0.220779	0.064935	0.025974	0.12987	0.044118	1.727273
1390	5-Jun-05	2005	MM	0.220779	0.064935	0.025974	0.12987	0.044118	1.727273
1118	9-Jun-05	2005	SC	0.183486	0	0.073394	0	0.006329	6.769231
1417	10-Jun-05	2005	SC	0.183486	0	0.073394	0	0.006329	6.769231

1437	10-Jun-05	2005	SC	0.183486	0	0.073394	0	0.006329	6.769231
1415	10-Jun-05	2005	SC	0.183486	0	0.073394	0	0.006329	6.769231
1416	10-Jun-05	2005	SC	0.183486	0	0.073394	0	0.006329	6.769231
1418	10-Jun-05	2005	SC	0.183486	0	0.073394	0	0.006329	6.769231
1435	10-Jun-05	2005	SC	0.183486	0	0.073394	0	0.006329	6.769231
1436	10-Jun-05	2005	SC	0.183486	0	0.073394	0	0.006329	6.769231
1438	10-Jun-05	2005	SC	0.183486	0	0.073394	0	0.006329	6.769231
1413	10-Jun-05	2005	SC	0.183486	0	0.073394	0	0.006329	6.769231
1433	10-Jun-05	2005	SC	0.183486	0	0.073394	0	0.006329	6.769231
1419	10-Jun-05	2005	SC	0.183486	0	0.073394	0	0.006329	6.769231
1439	10-Jun-05	2005	SC	0.183486	0	0.073394	0	0.006329	6.769231
1414	10-Jun-05	2005	SC	0.183486	0	0.073394	0	0.006329	6.769231
1434	10-Jun-05	2005	SC	0.183486	0	0.073394	0	0.006329	6.769231
1185	11-Jun-05	2005	SW	0.264151	0.113208	0.018868	0.169811	0.044444	1.4
1184	11-Jun-05	2005	SW	0.264151	0.113208	0.018868	0.169811	0.044444	1.4
1183	11-Jun-05	2005	SW	0.264151	0.113208	0.018868	0.169811	0.044444	1.4
1180	11-Jun-05	2005	SW	0.264151	0.113208	0.018868	0.169811	0.044444	1.4
1175	11-Jun-05	2005	SW	0.264151	0.113208	0.018868	0.169811	0.044444	1.4
1178	11-Jun-05	2005	SW	0.264151	0.113208	0.018868	0.169811	0.044444	1.4
1181	11-Jun-05	2005	SW	0.264151	0.113208	0.018868	0.169811	0.044444	1.4
1182	11-Jun-05	2005	SW	0.264151	0.113208	0.018868	0.169811	0.044444	1.4
1174	11-Jun-05	2005	SW	0.264151	0.113208	0.018868	0.169811	0.044444	1.4
1179	11-Jun-05	2005	SW	0.264151	0.113208	0.018868	0.169811	0.044444	1.4
1172	11-Jun-05	2005	SW	0.264151	0.113208	0.018868	0.169811	0.044444	1.4
1173	11-Jun-05	2005	SW	0.264151	0.113208	0.018868	0.169811	0.044444	1.4
1176	11-Jun-05	2005	SW	0.264151	0.113208	0.018868	0.169811	0.044444	1.4
1177	11-Jun-05	2005	SW	0.264151	0.113208	0.018868	0.169811	0.044444	1.4
1447	12-Jun-05	2005	SD	0.365079	0.095238	0.095238	0.380952	0.048387	2.5
1211	13-Jun-05	2005	TO	0.285714	0.142857	0.017857	0.142857	0.014577	1
1210	13-Jun-05	2005	TO	0.285714	0.142857	0.017857	0.142857	0.014577	1
1213	13-Jun-05	2005	TO	0.285714	0.142857	0.017857	0.142857	0.014577	1
1212	13-Jun-05	2005	TO	0.285714	0.142857	0.017857	0.142857	0.014577	1
1214	13-Jun-05	2005	TO	0.285714	0.142857	0.017857	0.142857	0.014577	1
1467	17-Jun-05	2005	LT1	0.393939	0.030303	0	0.242424	0	4.444444
1459	17-Jun-05	2005	LT1	0.393939	0.030303	0	0.242424	0	4.444444
1463	17-Jun-05	2005	LT1	0.393939	0.030303	0	0.242424	0	4.444444
1465	17-Jun-05	2005	LT1	0.393939	0.030303	0	0.242424	0	4.444444
1453	17-Jun-05	2005	LT1	0.393939	0.030303	0	0.242424	0	4.444444
1455	17-Jun-05	2005	LT1	0.393939	0.030303	0	0.242424	0	4.444444
1457	17-Jun-05	2005	LT1	0.393939	0.030303	0	0.242424	0	4.444444
1466	17-Jun-05	2005	LT1	0.393939	0.030303	0	0.242424	0	4.444444
1469	17-Jun-05	2005	LT1	0.393939	0.030303	0	0.242424	0	4.444444
1454	17-Jun-05	2005	LT1	0.393939	0.030303	0	0.242424	0	4.444444
1456	17-Jun-05	2005	LT1	0.393939	0.030303	0	0.242424	0	4.444444

1461	17-Jun-05	2005	LT1	0.393939	0.030303	0	0.242424	0	4.444444
1464	17-Jun-05	2005	LT1	0.393939	0.030303	0	0.242424	0	4.444444
1448	17-Jun-05	2005	LT1	0.393939	0.030303	0	0.242424	0	4.444444
1449	17-Jun-05	2005	LT1	0.393939	0.030303	0	0.242424	0	4.444444
1452	17-Jun-05	2005	LT1	0.393939	0.030303	0	0.242424	0	4.444444
1462	17-Jun-05	2005	LT1	0.393939	0.030303	0	0.242424	0	4.444444
1450	17-Jun-05	2005	LT1	0.393939	0.030303	0	0.242424	0	4.444444
1460	17-Jun-05	2005	LT1	0.393939	0.030303	0	0.242424	0	4.444444
1470	17-Jun-05	2005	LT1	0.393939	0.030303	0	0.242424	0	4.444444
1451	17-Jun-05	2005	LT1	0.393939	0.030303	0	0.242424	0	4.444444
1468	17-Jun-05	2005	LT1	0.393939	0.030303	0	0.242424	0	4.444444
1458	17-Jun-05	2005	LT1	0.393939	0.030303	0	0.242424	0	4.444444
1471	17-Jun-05	2005	LT1	0.393939	0.030303	0	0.242424	0	4.444444
1511	18-Jun-05	2005	RL	0.465517	0.103448	0	0.137931	0.108209	0.333333
1512	18-Jun-05	2005	RL	0.465517	0.103448	0	0.137931	0.108209	0.333333
1513	18-Jun-05	2005	RL	0.465517	0.103448	0	0.137931	0.108209	0.333333
1514	18-Jun-05	2005	RL	0.465517	0.103448	0	0.137931	0.108209	0.333333
1710	18-Jun-05	2005	RL	0.465517	0.103448	0	0.137931	0.108209	0.333333
1564	18-Jun-05	2005	SD	0.365079	0.095238	0.095238	0.380952	0.048387	2.5
1567	18-Jun-05	2005	SD	0.365079	0.095238	0.095238	0.380952	0.048387	2.5
1568	18-Jun-05	2005	SD	0.365079	0.095238	0.095238	0.380952	0.048387	2.5
1571	18-Jun-05	2005	SD	0.365079	0.095238	0.095238	0.380952	0.048387	2.5
1565	18-Jun-05	2005	SD	0.365079	0.095238	0.095238	0.380952	0.048387	2.5
1569	18-Jun-05	2005	SD	0.365079	0.095238	0.095238	0.380952	0.048387	2.5
1566	18-Jun-05	2005	SD	0.365079	0.095238	0.095238	0.380952	0.048387	2.5
1570	18-Jun-05	2005	SD	0.365079	0.095238	0.095238	0.380952	0.048387	2.5
1747	19-Jun-05	2005	ML						
1746	19-Jun-05	2005	ML						
1745	19-Jun-05	2005	ML						
1752	19-Jun-05	2005	ML						
1750	19-Jun-05	2005	ML						
1753	19-Jun-05	2005	ML						
1754	19-Jun-05	2005	ML						
1749	19-Jun-05	2005	ML						
1751	19-Jun-05	2005	ML						
1748	19-Jun-05	2005	ML						
1744	19-Jun-05	2005	ML						
105	27-May-06	2006	HH	0.102941	0.161765	0	0.352941	0	0.615385
104	27-May-06	2006	HH	0.102941	0.161765	0	0.352941	0	0.615385
101	27-May-06	2006	HH	0.102941	0.161765	0	0.352941	0	0.615385
102	27-May-06	2006	HH	0.102941	0.161765	0	0.352941	0	0.615385
106	27-May-06	2006	HH	0.102941	0.161765	0	0.352941	0	0.615385
107	27-May-06	2006	HH	0.102941	0.161765	0	0.352941	0	0.615385
103	27-May-06	2006	HH	0.102941	0.161765	0	0.352941	0	0.615385

110	29-May-06	2006	RP	0.233333	0	0.066667	0.3	0.027778	2.75
115	29-May-06	2006	SW	0.210526	0.026316	0.105263	0.131579	0.017778	1.4
116	29-May-06	2006	SW	0.210526	0.026316	0.105263	0.131579	0.017778	1.4
114	29-May-06	2006	SW	0.210526	0.026316	0.105263	0.131579	0.017778	1.4
113	29-May-06	2006	SW	0.210526	0.026316	0.105263	0.131579	0.017778	1.4
112	29-May-06	2006	SW	0.210526	0.026316	0.105263	0.131579	0.017778	1.4
111	29-May-06	2006	SW	0.210526	0.026316	0.105263	0.131579	0.017778	1.4
134	31-May-06	2006	SC	0.092308	0	0.030769	0.046154	0.002941	7.961538
135	31-May-06	2006	SC	0.092308	0	0.030769	0.046154	0.002941	7.961538
132	31-May-06	2006	SC	0.092308	0	0.030769	0.046154	0.002941	7.961538
131	31-May-06	2006	SC	0.092308	0	0.030769	0.046154	0.002941	7.961538
129	31-May-06	2006	SC	0.092308	0	0.030769	0.046154	0.002941	7.961538
130	31-May-06	2006	SC	0.092308	0	0.030769	0.046154	0.002941	7.961538
133	31-May-06	2006	SC	0.092308	0	0.030769	0.046154	0.002941	7.961538
157	1-Jun-06	2006	KN	0.157895	0.105263	0.026316	0.368421	0	1.133333
155	1-Jun-06	2006	KN	0.157895	0.105263	0.026316	0.368421	0	1.133333
156	1-Jun-06	2006	KN	0.157895	0.105263	0.026316	0.368421	0	1.133333
158	1-Jun-06	2006	KN	0.157895	0.105263	0.026316	0.368421	0	1.133333
172	3-Jun-06	2006	SC	0.092308	0	0.030769	0.046154	0.002941	7.961538
173	3-Jun-06	2006	SC	0.092308	0	0.030769	0.046154	0.002941	7.961538
174	3-Jun-06	2006	SC	0.092308	0	0.030769	0.046154	0.002941	7.961538
233	8-Jun-06	2006	LT1	0.222222	0.111111	0	0.148148	0	4
234	8-Jun-06	2006	LT1	0.222222	0.111111	0	0.148148	0	4
235	8-Jun-06	2006	LT1	0.222222	0.111111	0	0.148148	0	4
	18-Jun-06	2006	HH	0.102941	0.161765	0	0.352941	0	0.615385
	18-Jun-06	2006	HH	0.102941	0.161765	0	0.352941	0	0.615385
	18-Jun-06	2006	HH	0.102941	0.161765	0	0.352941	0	0.615385
	18-Jun-06	2006	HH	0.102941	0.161765	0	0.352941	0	0.615385
	18-Jun-06	2006	HH	0.102941	0.161765	0	0.352941	0	0.615385
	18-Jun-06	2006	HH	0.102941	0.161765	0	0.352941	0	0.615385
	18-Jun-06	2006	HH	0.102941	0.161765	0	0.352941	0	0.615385
145	8-May-07	2007	MG	0.24	0.28	0.12	0.02	0.009615	6.15
144	8-May-07	2007	MG	0.24	0.28	0.12	0.02	0.009615	6.15
146	8-May-07	2007	MG	0.24	0.28	0.12	0.02	0.009615	6.15
147	8-May-07	2007	MG	0.24	0.28	0.12	0.02	0.009615	6.15
142	8-May-07	2007	MG	0.24	0.28	0.12	0.02	0.009615	6.15
141	8-May-07	2007	MG	0.24	0.28	0.12	0.02	0.009615	6.15
149	8-May-07	2007	MG	0.24	0.28	0.12	0.02	0.009615	6.15
148	8-May-07	2007	MG	0.24	0.28	0.12	0.02	0.009615	6.15
150	8-May-07	2007	MG	0.24	0.28	0.12	0.02	0.009615	6.15
151	8-May-07	2007	MG	0.24	0.28	0.12	0.02	0.009615	6.15
153	8-May-07	2007	MG	0.24	0.28	0.12	0.02	0.009615	6.15
157	8-May-07	2007	MG	0.24	0.28	0.12	0.02	0.009615	6.15
160	8-May-07	2007	MG	0.24	0.28	0.12	0.02	0.009615	6.15

164	8-May-07	2007 MG	0.24	0.28	0.12	0.02	0.009615	6.15
166	8-May-07	2007 MG	0.24	0.28	0.12	0.02	0.009615	6.15
143	8-May-07	2007 MG	0.24	0.28	0.12	0.02	0.009615	6.15
159	8-May-07	2007 MG	0.24	0.28	0.12	0.02	0.009615	6.15
162	8-May-07	2007 MG	0.24	0.28	0.12	0.02	0.009615	6.15
165	8-May-07	2007 MG	0.24	0.28	0.12	0.02	0.009615	6.15
161	8-May-07	2007 MG	0.24	0.28	0.12	0.02	0.009615	6.15
163	8-May-07	2007 MG	0.24	0.28	0.12	0.02	0.009615	6.15
167	8-May-07	2007 MG	0.24	0.28	0.12	0.02	0.009615	6.15
158	8-May-07	2007 MG	0.24	0.28	0.12	0.02	0.009615	6.15
156	8-May-07	2007 MG	0.24	0.28	0.12	0.02	0.009615	6.15
155	8-May-07	2007 MG	0.24	0.28	0.12	0.02	0.009615	6.15
169	8-May-07	2007 MG	0.24	0.28	0.12	0.02	0.009615	6.15
174	8-May-07	2007 MG	0.24	0.28	0.12	0.02	0.009615	6.15
170	8-May-07	2007 MG	0.24	0.28	0.12	0.02	0.009615	6.15
175	8-May-07	2007 MG	0.24	0.28	0.12	0.02	0.009615	6.15
171	8-May-07	2007 MG	0.24	0.28	0.12	0.02	0.009615	6.15
176	8-May-07	2007 MG	0.24	0.28	0.12	0.02	0.009615	6.15
177	8-May-07	2007 MG	0.24	0.28	0.12	0.02	0.009615	6.15
152	8-May-07	2007 MG	0.24	0.28	0.12	0.02	0.009615	6.15
172	8-May-07	2007 MG	0.24	0.28	0.12	0.02	0.009615	6.15
173	8-May-07	2007 MG	0.24	0.28	0.12	0.02	0.009615	6.15
154	8-May-07	2007 MG	0.24	0.28	0.12	0.02	0.009615	6.15
168	8-May-07	2007 MG	0.24	0.28	0.12	0.02	0.009615	6.15
392	13-May-07	2007 SC	0.136986	0.041096	0.027397	0	0	8.576923
393	13-May-07	2007 SC	0.136986	0.041096	0.027397	0	0	8.576923
390	13-May-07	2007 SC	0.136986	0.041096	0.027397	0	0	8.576923
389	13-May-07	2007 SC	0.136986	0.041096	0.027397	0	0	8.576923
391	13-May-07	2007 SC	0.136986	0.041096	0.027397	0	0	8.576923
380	13-May-07	2007 SC	0.136986	0.041096	0.027397	0	0	8.576923
382	13-May-07	2007 SC	0.136986	0.041096	0.027397	0	0	8.576923
381	13-May-07	2007 SC	0.136986	0.041096	0.027397	0	0	8.576923
386	13-May-07	2007 SC	0.136986	0.041096	0.027397	0	0	8.576923
387	13-May-07	2007 SC	0.136986	0.041096	0.027397	0	0	8.576923
383	13-May-07	2007 SC	0.136986	0.041096	0.027397	0	0	8.576923
384	13-May-07	2007 SC	0.136986	0.041096	0.027397	0	0	8.576923
402	13-May-07	2007 SC	0.136986	0.041096	0.027397	0	0	8.576923
403	13-May-07	2007 SC	0.136986	0.041096	0.027397	0	0	8.576923
405	13-May-07	2007 SC	0.136986	0.041096	0.027397	0	0	8.576923
385	13-May-07	2007 SC	0.136986	0.041096	0.027397	0	0	8.576923
397	13-May-07	2007 SC	0.136986	0.041096	0.027397	0	0	8.576923
396	13-May-07	2007 SC	0.136986	0.041096	0.027397	0	0	8.576923
395	13-May-07	2007 SC	0.136986	0.041096	0.027397	0	0	8.576923
398	13-May-07	2007 SC	0.136986	0.041096	0.027397	0	0	8.576923

399	13-May-07	2007	SC	0.136986	0.041096	0.027397	0	0	8.576923
400	13-May-07	2007	SC	0.136986	0.041096	0.027397	0	0	8.576923
401	13-May-07	2007	SC	0.136986	0.041096	0.027397	0	0	8.576923
404	13-May-07	2007	SC	0.136986	0.041096	0.027397	0	0	8.576923
406	13-May-07	2007	SC	0.136986	0.041096	0.027397	0	0	8.576923
379	13-May-07	2007	SC	0.136986	0.041096	0.027397	0	0	8.576923
388	13-May-07	2007	SC	0.136986	0.041096	0.027397	0	0	8.576923
394	13-May-07	2007	SC	0.136986	0.041096	0.027397	0	0	8.576923
410	13-May-07	2007	RP	0.133333	0.033333	0.133333	0	0.019139	2.75
411	13-May-07	2007	RP	0.133333	0.033333	0.133333	0	0.019139	2.75
424	13-May-07	2007	RP	0.133333	0.033333	0.133333	0	0.019139	2.75
409	13-May-07	2007	RP	0.133333	0.033333	0.133333	0	0.019139	2.75
423	13-May-07	2007	RP	0.133333	0.033333	0.133333	0	0.019139	2.75
425	13-May-07	2007	RP	0.133333	0.033333	0.133333	0	0.019139	2.75
408	13-May-07	2007	RP	0.133333	0.033333	0.133333	0	0.019139	2.75
426	13-May-07	2007	RP	0.133333	0.033333	0.133333	0	0.019139	2.75
427	13-May-07	2007	RP	0.133333	0.033333	0.133333	0	0.019139	2.75
433	13-May-07	2007	RP	0.133333	0.033333	0.133333	0	0.019139	2.75
422	13-May-07	2007	RP	0.133333	0.033333	0.133333	0	0.019139	2.75
434	13-May-07	2007	RP	0.133333	0.033333	0.133333	0	0.019139	2.75
428	13-May-07	2007	RP	0.133333	0.033333	0.133333	0	0.019139	2.75
429	13-May-07	2007	RP	0.133333	0.033333	0.133333	0	0.019139	2.75
412	13-May-07	2007	RP	0.133333	0.033333	0.133333	0	0.019139	2.75
421	13-May-07	2007	RP	0.133333	0.033333	0.133333	0	0.019139	2.75
414	13-May-07	2007	RP	0.133333	0.033333	0.133333	0	0.019139	2.75
420	13-May-07	2007	RP	0.133333	0.033333	0.133333	0	0.019139	2.75
435	13-May-07	2007	RP	0.133333	0.033333	0.133333	0	0.019139	2.75
436	13-May-07	2007	RP	0.133333	0.033333	0.133333	0	0.019139	2.75
437	13-May-07	2007	RP	0.133333	0.033333	0.133333	0	0.019139	2.75
413	13-May-07	2007	RP	0.133333	0.033333	0.133333	0	0.019139	2.75
415	13-May-07	2007	RP	0.133333	0.033333	0.133333	0	0.019139	2.75
416	13-May-07	2007	RP	0.133333	0.033333	0.133333	0	0.019139	2.75
417	13-May-07	2007	RP	0.133333	0.033333	0.133333	0	0.019139	2.75
419	13-May-07	2007	RP	0.133333	0.033333	0.133333	0	0.019139	2.75
431	13-May-07	2007	RP	0.133333	0.033333	0.133333	0	0.019139	2.75
430	13-May-07	2007	RP	0.133333	0.033333	0.133333	0	0.019139	2.75
438	13-May-07	2007	RP	0.133333	0.033333	0.133333	0	0.019139	2.75
439	13-May-07	2007	RP	0.133333	0.033333	0.133333	0	0.019139	2.75
440	13-May-07	2007	RP	0.133333	0.033333	0.133333	0	0.019139	2.75
441	13-May-07	2007	RP	0.133333	0.033333	0.133333	0	0.019139	2.75
407	13-May-07	2007	RP	0.133333	0.033333	0.133333	0	0.019139	2.75
418	13-May-07	2007	RP	0.133333	0.033333	0.133333	0	0.019139	2.75
432	13-May-07	2007	RP	0.133333	0.033333	0.133333	0	0.019139	2.75
1087	19-May-07	2007	D1	0.26	0.14	0.02	0.06	0.006036	0.8

1088	19-May-07	2007	D1	0.26	0.14	0.02	0.06	0.006036	0.8
1089	19-May-07	2007	D1	0.26	0.14	0.02	0.06	0.006036	0.8
1086	19-May-07	2007	D1	0.26	0.14	0.02	0.06	0.006036	0.8
1085	19-May-07	2007	D1	0.26	0.14	0.02	0.06	0.006036	0.8
1090	19-May-07	2007	D1	0.26	0.14	0.02	0.06	0.006036	0.8
1091	19-May-07	2007	D1	0.26	0.14	0.02	0.06	0.006036	0.8
1084	19-May-07	2007	D1	0.26	0.14	0.02	0.06	0.006036	0.8
1073	19-May-07	2007	D1	0.26	0.14	0.02	0.06	0.006036	0.8
1070	19-May-07	2007	D1	0.26	0.14	0.02	0.06	0.006036	0.8
1072	19-May-07	2007	D1	0.26	0.14	0.02	0.06	0.006036	0.8
1071	19-May-07	2007	D1	0.26	0.14	0.02	0.06	0.006036	0.8
1069	19-May-07	2007	D1	0.26	0.14	0.02	0.06	0.006036	0.8
1068	19-May-07	2007	D1	0.26	0.14	0.02	0.06	0.006036	0.8
1067	19-May-07	2007	D1	0.26	0.14	0.02	0.06	0.006036	0.8
1061	19-May-07	2007	D1	0.26	0.14	0.02	0.06	0.006036	0.8
1062	19-May-07	2007	D1	0.26	0.14	0.02	0.06	0.006036	0.8
1066	19-May-07	2007	D1	0.26	0.14	0.02	0.06	0.006036	0.8
1074	19-May-07	2007	D1	0.26	0.14	0.02	0.06	0.006036	0.8
1075	19-May-07	2007	D1	0.26	0.14	0.02	0.06	0.006036	0.8
1076	19-May-07	2007	D1	0.26	0.14	0.02	0.06	0.006036	0.8
1065	19-May-07	2007	D1	0.26	0.14	0.02	0.06	0.006036	0.8
1079	19-May-07	2007	D1	0.26	0.14	0.02	0.06	0.006036	0.8
1083	19-May-07	2007	D1	0.26	0.14	0.02	0.06	0.006036	0.8
1077	19-May-07	2007	D1	0.26	0.14	0.02	0.06	0.006036	0.8
1078	19-May-07	2007	D1	0.26	0.14	0.02	0.06	0.006036	0.8
1080	19-May-07	2007	D1	0.26	0.14	0.02	0.06	0.006036	0.8
1081	19-May-07	2007	D1	0.26	0.14	0.02	0.06	0.006036	0.8
1063	19-May-07	2007	D1	0.26	0.14	0.02	0.06	0.006036	0.8
1060	19-May-07	2007	D1	0.26	0.14	0.02	0.06	0.006036	0.8
1064	19-May-07	2007	D1	0.26	0.14	0.02	0.06	0.006036	0.8
1082	19-May-07	2007	D1	0.26	0.14	0.02	0.06	0.006036	0.8
2230	17-Jun-08	2008	RC	0.15	0	0.05	0	0.009346	2.875
2231	17-Jun-08	2008	RC	0.15	0	0.05	0	0.009346	2.875
2232	17-Jun-08	2008	RC	0.15	0	0.05	0	0.009346	2.875
2267	24-Jun-08	2008	SC	0.191781	0.013699	0.123288	0	0	8.615385
2259	24-Jun-08	2008	SC	0.191781	0.013699	0.123288	0	0	8.615385
2260	24-Jun-08	2008	SC	0.191781	0.013699	0.123288	0	0	8.615385
2262	24-Jun-08	2008	SC	0.191781	0.013699	0.123288	0	0	8.615385
2265	24-Jun-08	2008	SC	0.191781	0.013699	0.123288	0	0	8.615385
2266	24-Jun-08	2008	SC	0.191781	0.013699	0.123288	0	0	8.615385
2268	24-Jun-08	2008	SC	0.191781	0.013699	0.123288	0	0	8.615385
2269	24-Jun-08	2008	SC	0.191781	0.013699	0.123288	0	0	8.615385
2270	24-Jun-08	2008	SC	0.191781	0.013699	0.123288	0	0	8.615385
2272	24-Jun-08	2008	SC	0.191781	0.013699	0.123288	0	0	8.615385

2274	24-Jun-08	2008	SC	0.191781	0.013699	0.123288	0	0	8.615385
2263	24-Jun-08	2008	SC	0.191781	0.013699	0.123288	0	0	8.615385
2264	24-Jun-08	2008	SC	0.191781	0.013699	0.123288	0	0	8.615385
2275	24-Jun-08	2008	SC	0.191781	0.013699	0.123288	0	0	8.615385
2276	24-Jun-08	2008	SC	0.191781	0.013699	0.123288	0	0	8.615385
2277	24-Jun-08	2008	SC	0.191781	0.013699	0.123288	0	0	8.615385
2261	24-Jun-08	2008	SC	0.191781	0.013699	0.123288	0	0	8.615385
2258	24-Jun-08	2008	SC	0.191781	0.013699	0.123288	0	0	8.615385
2271	24-Jun-08	2008	SC	0.191781	0.013699	0.123288	0	0	8.615385
2273	24-Jun-08	2008	SC	0.191781	0.013699	0.123288	0	0	8.615385
2339	24-Jun-08	2008	RP	0.176471	0	0.029412	0.029412	0.015228	3.083333
2341	24-Jun-08	2008	RP	0.176471	0	0.029412	0.029412	0.015228	3.083333
2338	24-Jun-08	2008	RP	0.176471	0	0.029412	0.029412	0.015228	3.083333
2343	24-Jun-08	2008	RP	0.176471	0	0.029412	0.029412	0.015228	3.083333
2344	24-Jun-08	2008	RP	0.176471	0	0.029412	0.029412	0.015228	3.083333
2337	24-Jun-08	2008	RP	0.176471	0	0.029412	0.029412	0.015228	3.083333
2336	24-Jun-08	2008	RP	0.176471	0	0.029412	0.029412	0.015228	3.083333
2334	24-Jun-08	2008	RP	0.176471	0	0.029412	0.029412	0.015228	3.083333
2335	24-Jun-08	2008	RP	0.176471	0	0.029412	0.029412	0.015228	3.083333
2323	24-Jun-08	2008	RP	0.176471	0	0.029412	0.029412	0.015228	3.083333
2324	24-Jun-08	2008	RP	0.176471	0	0.029412	0.029412	0.015228	3.083333
2327	24-Jun-08	2008	RP	0.176471	0	0.029412	0.029412	0.015228	3.083333
2331	24-Jun-08	2008	RP	0.176471	0	0.029412	0.029412	0.015228	3.083333
2326	24-Jun-08	2008	RP	0.176471	0	0.029412	0.029412	0.015228	3.083333
2318	24-Jun-08	2008	RP	0.176471	0	0.029412	0.029412	0.015228	3.083333
2319	24-Jun-08	2008	RP	0.176471	0	0.029412	0.029412	0.015228	3.083333
2317	24-Jun-08	2008	RP	0.176471	0	0.029412	0.029412	0.015228	3.083333
2325	24-Jun-08	2008	RP	0.176471	0	0.029412	0.029412	0.015228	3.083333
2320	24-Jun-08	2008	RP	0.176471	0	0.029412	0.029412	0.015228	3.083333
2329	24-Jun-08	2008	RP	0.176471	0	0.029412	0.029412	0.015228	3.083333
2321	24-Jun-08	2008	RP	0.176471	0	0.029412	0.029412	0.015228	3.083333
2322	24-Jun-08	2008	RP	0.176471	0	0.029412	0.029412	0.015228	3.083333
2330	24-Jun-08	2008	RP	0.176471	0	0.029412	0.029412	0.015228	3.083333
2332	24-Jun-08	2008	RP	0.176471	0	0.029412	0.029412	0.015228	3.083333
2340	24-Jun-08	2008	RP	0.176471	0	0.029412	0.029412	0.015228	3.083333
2342	24-Jun-08	2008	RP	0.176471	0	0.029412	0.029412	0.015228	3.083333
2316	24-Jun-08	2008	RP	0.176471	0	0.029412	0.029412	0.015228	3.083333
2328	24-Jun-08	2008	RP	0.176471	0	0.029412	0.029412	0.015228	3.083333
2333	24-Jun-08	2008	RP	0.176471	0	0.029412	0.029412	0.015228	3.083333
1680	17-Jun-05	2005	LT1	0.393939	0.030303	0	0.242424	0	4.444444
1681	17-Jun-05	2005	LT1	0.393939	0.030303	0	0.242424	0	4.444444
1688	17-Jun-05	2005	LT1	0.393939	0.030303	0	0.242424	0	4.444444
1691	17-Jun-05	2005	LT1	0.393939	0.030303	0	0.242424	0	4.444444
1689	17-Jun-05	2005	LT1	0.393939	0.030303	0	0.242424	0	4.444444

1690	17-Jun-05	2005	LT1	0.393939	0.030303	0	0.242424	0	4.444444
1687	17-Jun-05	2005	LT1	0.393939	0.030303	0	0.242424	0	4.444444
1692	17-Jun-05	2005	LT1	0.393939	0.030303	0	0.242424	0	4.444444
1684	17-Jun-05	2005	LT1	0.393939	0.030303	0	0.242424	0	4.444444
1683	17-Jun-05	2005	LT1	0.393939	0.030303	0	0.242424	0	4.444444
1682	17-Jun-05	2005	LT1	0.393939	0.030303	0	0.242424	0	4.444444
1685	17-Jun-05	2005	LT1	0.393939	0.030303	0	0.242424	0	4.444444
1686	17-Jun-05	2005	LT1	0.393939	0.030303	0	0.242424	0	4.444444
	17-Jun-05	2005	LT1	0.393939	0.030303	0	0.242424	0	4.444444
	17-Jun-05	2005	LT1	0.393939	0.030303	0	0.242424	0	4.444444
	17-Jun-05	2005	LT1	0.393939	0.030303	0	0.242424	0	4.444444
	17-Jun-05	2005	LT1	0.393939	0.030303	0	0.242424	0	4.444444
	17-Jun-05	2005	LT1	0.393939	0.030303	0	0.242424	0	4.444444
	17-Jun-05	2005	LT1	0.393939	0.030303	0	0.242424	0	4.444444
	17-Jun-05	2005	LT1	0.393939	0.030303	0	0.242424	0	4.444444
	17-Jun-05	2005	LT1	0.393939	0.030303	0	0.242424	0	4.444444
	17-Jun-05	2005	LT1	0.393939	0.030303	0	0.242424	0	4.444444
	17-Jun-05	2005	LT1	0.393939	0.030303	0	0.242424	0	4.444444
	17-Jun-05	2005	LT1	0.393939	0.030303	0	0.242424	0	4.444444
	17-Jun-05	2005	LT1	0.393939	0.030303	0	0.242424	0	4.444444
	17-Jun-05	2005	LT1	0.393939	0.030303	0	0.242424	0	4.444444
1192	11-Jun-05	2005	SW	0.264151	0.113208	0.018868	0.169811	0.044444	1.4
1193	11-Jun-05	2005	SW	0.264151	0.113208	0.018868	0.169811	0.044444	1.4
1191	11-Jun-05	2005	SW	0.264151	0.113208	0.018868	0.169811	0.044444	1.4
1190	11-Jun-05	2005	SW	0.264151	0.113208	0.018868	0.169811	0.044444	1.4
1188	11-Jun-05	2005	SW	0.264151	0.113208	0.018868	0.169811	0.044444	1.4
1189	11-Jun-05	2005	SW	0.264151	0.113208	0.018868	0.169811	0.044444	1.4
1195	11-Jun-05	2005	SW	0.264151	0.113208	0.018868	0.169811	0.044444	1.4
1194	11-Jun-05	2005	SW	0.264151	0.113208	0.018868	0.169811	0.044444	1.4
1187	11-Jun-05	2005	SW	0.264151	0.113208	0.018868	0.169811	0.044444	1.4
1196	11-Jun-05	2005	SW	0.264151	0.113208	0.018868	0.169811	0.044444	1.4
1186	11-Jun-05	2005	SW	0.264151	0.113208	0.018868	0.169811	0.044444	1.4
1627	13-Jun-05	2005	SW	0.264151	0.113208	0.018868	0.169811	0.044444	1.4
1626	13-Jun-05	2005	SW	0.264151	0.113208	0.018868	0.169811	0.044444	1.4
1631	13-Jun-05	2005	SW	0.264151	0.113208	0.018868	0.169811	0.044444	1.4
1632	13-Jun-05	2005	SW	0.264151	0.113208	0.018868	0.169811	0.044444	1.4
1628	13-Jun-05	2005	SW	0.264151	0.113208	0.018868	0.169811	0.044444	1.4
1629	13-Jun-05	2005	SW	0.264151	0.113208	0.018868	0.169811	0.044444	1.4
1630	13-Jun-05	2005	SW	0.264151	0.113208	0.018868	0.169811	0.044444	1.4
1625	13-Jun-05	2005	SW	0.264151	0.113208	0.018868	0.169811	0.044444	1.4
1624	13-Jun-05	2005	SW	0.264151	0.113208	0.018868	0.169811	0.044444	1.4
363	31-May-04	2004	RC	0.428571	0.107143	0	0	0.401709	3
360	31-May-04	2004	RC	0.428571	0.107143	0	0	0.401709	3
358	31-May-04	2004	RC	0.428571	0.107143	0	0	0.401709	3

362	31-May-04	2004	RC	0.428571	0.107143	0	0	0.401709	3
364	31-May-04	2004	RC	0.428571	0.107143	0	0	0.401709	3
368	31-May-04	2004	RC	0.428571	0.107143	0	0	0.401709	3
365	31-May-04	2004	RC	0.428571	0.107143	0	0	0.401709	3
359	31-May-04	2004	RC	0.428571	0.107143	0	0	0.401709	3
367	31-May-04	2004	RC	0.428571	0.107143	0	0	0.401709	3
361	31-May-04	2004	RC	0.428571	0.107143	0	0	0.401709	3
366	31-May-04	2004	RC	0.428571	0.107143	0	0	0.401709	3
381	31-May-04	2004	RC	0.428571	0.107143	0	0	0.401709	3
383	31-May-04	2004	RC	0.428571	0.107143	0	0	0.401709	3
372	31-May-04	2004	RC	0.428571	0.107143	0	0	0.401709	3
371	31-May-04	2004	RC	0.428571	0.107143	0	0	0.401709	3
373	31-May-04	2004	RC	0.428571	0.107143	0	0	0.401709	3
374	31-May-04	2004	RC	0.428571	0.107143	0	0	0.401709	3
370	31-May-04	2004	RC	0.428571	0.107143	0	0	0.401709	3
369	31-May-04	2004	RC	0.428571	0.107143	0	0	0.401709	3
375	31-May-04	2004	RC	0.428571	0.107143	0	0	0.401709	3
376	31-May-04	2004	RC	0.428571	0.107143	0	0	0.401709	3
377	31-May-04	2004	RC	0.428571	0.107143	0	0	0.401709	3
380	31-May-04	2004	RC	0.428571	0.107143	0	0	0.401709	3
378	31-May-04	2004	RC	0.428571	0.107143	0	0	0.401709	3
379	31-May-04	2004	RC	0.428571	0.107143	0	0	0.401709	3
382	31-May-04	2004	RC	0.428571	0.107143	0	0	0.401709	3
213	7-Jun-04	2004	SD	0.412698	0.301587	0.063492	0.634921	0.097297	2.642857
216	7-Jun-04	2004	SD	0.412698	0.301587	0.063492	0.634921	0.097297	2.642857
218	7-Jun-04	2004	SD	0.412698	0.301587	0.063492	0.634921	0.097297	2.642857
214	7-Jun-04	2004	SD	0.412698	0.301587	0.063492	0.634921	0.097297	2.642857
215	7-Jun-04	2004	SD	0.412698	0.301587	0.063492	0.634921	0.097297	2.642857
211	7-Jun-04	2004	SD	0.412698	0.301587	0.063492	0.634921	0.097297	2.642857
210	7-Jun-04	2004	SD	0.412698	0.301587	0.063492	0.634921	0.097297	2.642857
217	7-Jun-04	2004	SD	0.412698	0.301587	0.063492	0.634921	0.097297	2.642857
219	7-Jun-04	2004	SD	0.412698	0.301587	0.063492	0.634921	0.097297	2.642857
209	7-Jun-04	2004	SD	0.412698	0.301587	0.063492	0.634921	0.097297	2.642857
212	7-Jun-04	2004	SD	0.412698	0.301587	0.063492	0.634921	0.097297	2.642857
250	12-Jun-04	2004	RL	0.396226	0.113208	0.09434	0.320755	0.203008	0.333333
254	12-Jun-04	2004	RL	0.396226	0.113208	0.09434	0.320755	0.203008	0.333333
255	12-Jun-04	2004	RL	0.396226	0.113208	0.09434	0.320755	0.203008	0.333333
256	12-Jun-04	2004	RL	0.396226	0.113208	0.09434	0.320755	0.203008	0.333333
248	12-Jun-04	2004	RL	0.396226	0.113208	0.09434	0.320755	0.203008	0.333333
249	12-Jun-04	2004	RL	0.396226	0.113208	0.09434	0.320755	0.203008	0.333333
251	12-Jun-04	2004	RL	0.396226	0.113208	0.09434	0.320755	0.203008	0.333333
252	12-Jun-04	2004	RL	0.396226	0.113208	0.09434	0.320755	0.203008	0.333333
253	12-Jun-04	2004	RL	0.396226	0.113208	0.09434	0.320755	0.203008	0.333333
247	12-Jun-04	2004	RL	0.396226	0.113208	0.09434	0.320755	0.203008	0.333333

257	12-Jun-04	2004	RL	0.396226	0.113208	0.09434	0.320755	0.203008	0.333333
295	21-Jun-04	2004	SD	0.412698	0.301587	0.063492	0.634921	0.097297	2.642857
296	21-Jun-04	2004	SD	0.412698	0.301587	0.063492	0.634921	0.097297	2.642857
759	25-May-05	2005	SD	0.365079	0.095238	0.095238	0.380952	0.048387	2.5
761	25-May-05	2005	SD	0.365079	0.095238	0.095238	0.380952	0.048387	2.5
758	25-May-05	2005	SD	0.365079	0.095238	0.095238	0.380952	0.048387	2.5
757	25-May-05	2005	SD	0.365079	0.095238	0.095238	0.380952	0.048387	2.5
756	25-May-05	2005	SD	0.365079	0.095238	0.095238	0.380952	0.048387	2.5
764	25-May-05	2005	SD	0.365079	0.095238	0.095238	0.380952	0.048387	2.5
766	25-May-05	2005	SD	0.365079	0.095238	0.095238	0.380952	0.048387	2.5
767	25-May-05	2005	SD	0.365079	0.095238	0.095238	0.380952	0.048387	2.5
755	25-May-05	2005	SD	0.365079	0.095238	0.095238	0.380952	0.048387	2.5
768	25-May-05	2005	SD	0.365079	0.095238	0.095238	0.380952	0.048387	2.5
769	25-May-05	2005	SD	0.365079	0.095238	0.095238	0.380952	0.048387	2.5
751	25-May-05	2005	SD	0.365079	0.095238	0.095238	0.380952	0.048387	2.5
752	25-May-05	2005	SD	0.365079	0.095238	0.095238	0.380952	0.048387	2.5
762	25-May-05	2005	SD	0.365079	0.095238	0.095238	0.380952	0.048387	2.5
750	25-May-05	2005	SD	0.365079	0.095238	0.095238	0.380952	0.048387	2.5
760	25-May-05	2005	SD	0.365079	0.095238	0.095238	0.380952	0.048387	2.5
754	25-May-05	2005	SD	0.365079	0.095238	0.095238	0.380952	0.048387	2.5
765	25-May-05	2005	SD	0.365079	0.095238	0.095238	0.380952	0.048387	2.5
753	25-May-05	2005	SD	0.365079	0.095238	0.095238	0.380952	0.048387	2.5
763	25-May-05	2005	SD	0.365079	0.095238	0.095238	0.380952	0.048387	2.5
891	29-May-05	2005	DE						
897	29-May-05	2005	DE						
894	29-May-05	2005	DE						
896	29-May-05	2005	DE						
899	29-May-05	2005	DE						
893	29-May-05	2005	DE						
888	29-May-05	2005	DE						
889	29-May-05	2005	DE						
895	29-May-05	2005	DE						
898	29-May-05	2005	DE						
890	29-May-05	2005	DE						
892	29-May-05	2005	DE						
1338	5-Jun-05	2005	SHAC	0.076923	0.153846	0	0.384615	0.026042	2.090909
1339	5-Jun-05	2005	SHAC	0.076923	0.153846	0	0.384615	0.026042	2.090909
1340	5-Jun-05	2005	SHAC	0.076923	0.153846	0	0.384615	0.026042	2.090909
1341	5-Jun-05	2005	SHAC	0.076923	0.153846	0	0.384615	0.026042	2.090909
1342	5-Jun-05	2005	SHAC	0.076923	0.153846	0	0.384615	0.026042	2.090909
1333	5-Jun-05	2005	SHAC	0.076923	0.153846	0	0.384615	0.026042	2.090909
1334	5-Jun-05	2005	SHAC	0.076923	0.153846	0	0.384615	0.026042	2.090909
1336	5-Jun-05	2005	SHAC	0.076923	0.153846	0	0.384615	0.026042	2.090909
1337	5-Jun-05	2005	SHAC	0.076923	0.153846	0	0.384615	0.026042	2.090909

1335	5-Jun-05	2005	SHAC	0.076923	0.153846	0	0.384615	0.026042	2.090909
1374	5-Jun-05	2005	7M	0.264151	0.09434	0	0.301887	0.01519	8.266667
1372	5-Jun-05	2005	7M	0.264151	0.09434	0	0.301887	0.01519	8.266667
1371	5-Jun-05	2005	7M	0.264151	0.09434	0	0.301887	0.01519	8.266667
1370	5-Jun-05	2005	7M	0.264151	0.09434	0	0.301887	0.01519	8.266667
1373	5-Jun-05	2005	7M	0.264151	0.09434	0	0.301887	0.01519	8.266667
1375	5-Jun-05	2005	7M	0.264151	0.09434	0	0.301887	0.01519	8.266667
1376	5-Jun-05	2005	7M	0.264151	0.09434	0	0.301887	0.01519	8.266667
1369	5-Jun-05	2005	7M	0.264151	0.09434	0	0.301887	0.01519	8.266667
1064	5-Jun-05	2005	MM	0.220779	0.064935	0.025974	0.12987	0.044118	1.727273
1066	5-Jun-05	2005	MM	0.220779	0.064935	0.025974	0.12987	0.044118	1.727273
1062	5-Jun-05	2005	MM	0.220779	0.064935	0.025974	0.12987	0.044118	1.727273
1072	5-Jun-05	2005	MM	0.220779	0.064935	0.025974	0.12987	0.044118	1.727273
1074	5-Jun-05	2005	MM	0.220779	0.064935	0.025974	0.12987	0.044118	1.727273
1076	5-Jun-05	2005	MM	0.220779	0.064935	0.025974	0.12987	0.044118	1.727273
1071	5-Jun-05	2005	MM	0.220779	0.064935	0.025974	0.12987	0.044118	1.727273
1069	5-Jun-05	2005	MM	0.220779	0.064935	0.025974	0.12987	0.044118	1.727273
1075	5-Jun-05	2005	MM	0.220779	0.064935	0.025974	0.12987	0.044118	1.727273
1073	5-Jun-05	2005	MM	0.220779	0.064935	0.025974	0.12987	0.044118	1.727273
1061	5-Jun-05	2005	MM	0.220779	0.064935	0.025974	0.12987	0.044118	1.727273
1065	5-Jun-05	2005	MM	0.220779	0.064935	0.025974	0.12987	0.044118	1.727273
1063	5-Jun-05	2005	MM	0.220779	0.064935	0.025974	0.12987	0.044118	1.727273
1077	5-Jun-05	2005	MM	0.220779	0.064935	0.025974	0.12987	0.044118	1.727273
1070	5-Jun-05	2005	MM	0.220779	0.064935	0.025974	0.12987	0.044118	1.727273
1067	5-Jun-05	2005	MM	0.220779	0.064935	0.025974	0.12987	0.044118	1.727273
1068	5-Jun-05	2005	MM	0.220779	0.064935	0.025974	0.12987	0.044118	1.727273
1444	10-Jun-05	2005	SC	0.183486	0	0.073394	0	0.006329	6.769231
1442	10-Jun-05	2005	SC	0.183486	0	0.073394	0	0.006329	6.769231
1443	10-Jun-05	2005	SC	0.183486	0	0.073394	0	0.006329	6.769231
1445	10-Jun-05	2005	SC	0.183486	0	0.073394	0	0.006329	6.769231
1440	10-Jun-05	2005	SC	0.183486	0	0.073394	0	0.006329	6.769231
1446	10-Jun-05	2005	SC	0.183486	0	0.073394	0	0.006329	6.769231
1441	10-Jun-05	2005	SC	0.183486	0	0.073394	0	0.006329	6.769231
1129	11-Jun-05	2005	SC	0.183486	0	0.073394	0	0.006329	6.769231
1131	11-Jun-05	2005	SC	0.183486	0	0.073394	0	0.006329	6.769231
1132	11-Jun-05	2005	SC	0.183486	0	0.073394	0	0.006329	6.769231
1127	11-Jun-05	2005	SC	0.183486	0	0.073394	0	0.006329	6.769231
1123	11-Jun-05	2005	SC	0.183486	0	0.073394	0	0.006329	6.769231
1121	11-Jun-05	2005	SC	0.183486	0	0.073394	0	0.006329	6.769231
1122	11-Jun-05	2005	SC	0.183486	0	0.073394	0	0.006329	6.769231
1125	11-Jun-05	2005	SC	0.183486	0	0.073394	0	0.006329	6.769231
1119	11-Jun-05	2005	SC	0.183486	0	0.073394	0	0.006329	6.769231
1120	11-Jun-05	2005	SC	0.183486	0	0.073394	0	0.006329	6.769231
1128	11-Jun-05	2005	SC	0.183486	0	0.073394	0	0.006329	6.769231

1124	11-Jun-05	2005 SC	0.183486	0	0.073394	0	0.006329	6.769231
1126	11-Jun-05	2005 SC	0.183486	0	0.073394	0	0.006329	6.769231
1130	11-Jun-05	2005 SC	0.183486	0	0.073394	0	0.006329	6.769231
168	1-Jun-06	2006 FO	0.157895	0.131579	0.052632	0.157895	0.015152	0.066667
169	1-Jun-06	2006 FO	0.157895	0.131579	0.052632	0.157895	0.015152	0.066667
167	1-Jun-06	2006 FO	0.157895	0.131579	0.052632	0.157895	0.015152	0.066667
166	1-Jun-06	2006 FO	0.157895	0.131579	0.052632	0.157895	0.015152	0.066667
170	1-Jun-06	2006 FO	0.157895	0.131579	0.052632	0.157895	0.015152	0.066667
171	1-Jun-06	2006 FO	0.157895	0.131579	0.052632	0.157895	0.015152	0.066667
237	10-Jun-06	2006 HH	0.102941	0.161765	0	0.352941	0	0.615385
239	10-Jun-06	2006 HH	0.102941	0.161765	0	0.352941	0	0.615385
240	10-Jun-06	2006 HH	0.102941	0.161765	0	0.352941	0	0.615385
241	10-Jun-06	2006 HH	0.102941	0.161765	0	0.352941	0	0.615385
243	10-Jun-06	2006 HH	0.102941	0.161765	0	0.352941	0	0.615385
245	10-Jun-06	2006 HH	0.102941	0.161765	0	0.352941	0	0.615385
247	10-Jun-06	2006 HH	0.102941	0.161765	0	0.352941	0	0.615385
244	10-Jun-06	2006 HH	0.102941	0.161765	0	0.352941	0	0.615385
238	10-Jun-06	2006 HH	0.102941	0.161765	0	0.352941	0	0.615385
236	10-Jun-06	2006 HH	0.102941	0.161765	0	0.352941	0	0.615385
242	10-Jun-06	2006 HH	0.102941	0.161765	0	0.352941	0	0.615385
246	10-Jun-06	2006 HH	0.102941	0.161765	0	0.352941	0	0.615385
56	6-May-07	2007 ML						
34	6-May-07	2007 ML						
38	6-May-07	2007 ML						
44	6-May-07	2007 ML						
50	6-May-07	2007 ML						
52	6-May-07	2007 ML						
35	6-May-07	2007 ML						
51	6-May-07	2007 ML						
43	6-May-07	2007 ML						
36	6-May-07	2007 ML						
31	6-May-07	2007 ML						
32	6-May-07	2007 ML						
33	6-May-07	2007 ML						
42	6-May-07	2007 ML						
45	6-May-07	2007 ML						
49	6-May-07	2007 ML						
37	6-May-07	2007 ML						
41	6-May-07	2007 ML						
46	6-May-07	2007 ML						
47	6-May-07	2007 ML						
39	6-May-07	2007 ML						
40	6-May-07	2007 ML						
53	6-May-07	2007 ML						

57	6-May-07	2007	ML						
59	6-May-07	2007	ML						
60	6-May-07	2007	ML						
55	6-May-07	2007	ML						
58	6-May-07	2007	ML						
48	6-May-07	2007	ML						
54	6-May-07	2007	ML						
531	13-May-07	2007	RC	0.25	0	0.05	0.05	0.018182	2.875
529	13-May-07	2007	RC	0.25	0	0.05	0.05	0.018182	2.875
533	13-May-07	2007	RC	0.25	0	0.05	0.05	0.018182	2.875
530	13-May-07	2007	RC	0.25	0	0.05	0.05	0.018182	2.875
534	13-May-07	2007	RC	0.25	0	0.05	0.05	0.018182	2.875
528	13-May-07	2007	RC	0.25	0	0.05	0.05	0.018182	2.875
546	13-May-07	2007	RC	0.25	0	0.05	0.05	0.018182	2.875
532	13-May-07	2007	RC	0.25	0	0.05	0.05	0.018182	2.875
527	13-May-07	2007	RC	0.25	0	0.05	0.05	0.018182	2.875
535	13-May-07	2007	RC	0.25	0	0.05	0.05	0.018182	2.875
536	13-May-07	2007	RC	0.25	0	0.05	0.05	0.018182	2.875
547	13-May-07	2007	RC	0.25	0	0.05	0.05	0.018182	2.875
537	13-May-07	2007	RC	0.25	0	0.05	0.05	0.018182	2.875
544	13-May-07	2007	RC	0.25	0	0.05	0.05	0.018182	2.875
539	13-May-07	2007	RC	0.25	0	0.05	0.05	0.018182	2.875
543	13-May-07	2007	RC	0.25	0	0.05	0.05	0.018182	2.875
538	13-May-07	2007	RC	0.25	0	0.05	0.05	0.018182	2.875
540	13-May-07	2007	RC	0.25	0	0.05	0.05	0.018182	2.875
541	13-May-07	2007	RC	0.25	0	0.05	0.05	0.018182	2.875
542	13-May-07	2007	RC	0.25	0	0.05	0.05	0.018182	2.875
522	13-May-07	2007	RC	0.25	0	0.05	0.05	0.018182	2.875
523	13-May-07	2007	RC	0.25	0	0.05	0.05	0.018182	2.875
525	13-May-07	2007	RC	0.25	0	0.05	0.05	0.018182	2.875
524	13-May-07	2007	RC	0.25	0	0.05	0.05	0.018182	2.875
526	13-May-07	2007	RC	0.25	0	0.05	0.05	0.018182	2.875
545	13-May-07	2007	RC	0.25	0	0.05	0.05	0.018182	2.875
952	16-May-07	2007	HH	0.089552	0.149254	0.104478	0.029851	0	0.615385
956	16-May-07	2007	HH	0.089552	0.149254	0.104478	0.029851	0	0.615385
955	16-May-07	2007	HH	0.089552	0.149254	0.104478	0.029851	0	0.615385
957	16-May-07	2007	HH	0.089552	0.149254	0.104478	0.029851	0	0.615385
958	16-May-07	2007	HH	0.089552	0.149254	0.104478	0.029851	0	0.615385
959	16-May-07	2007	HH	0.089552	0.149254	0.104478	0.029851	0	0.615385
951	16-May-07	2007	HH	0.089552	0.149254	0.104478	0.029851	0	0.615385
953	16-May-07	2007	HH	0.089552	0.149254	0.104478	0.029851	0	0.615385
954	16-May-07	2007	HH	0.089552	0.149254	0.104478	0.029851	0	0.615385
3028	2-Jun-07	2007	ML						
3029	2-Jun-07	2007	ML						

3034	2-Jun-07	2007	ML						
3022	2-Jun-07	2007	ML						
3030	2-Jun-07	2007	ML						
3031	2-Jun-07	2007	ML						
3032	2-Jun-07	2007	ML						
3024	2-Jun-07	2007	ML						
3020	2-Jun-07	2007	ML						
3019	2-Jun-07	2007	ML						
3021	2-Jun-07	2007	ML						
3023	2-Jun-07	2007	ML						
3025	2-Jun-07	2007	ML						
3026	2-Jun-07	2007	ML						
3033	2-Jun-07	2007	ML						
3018	2-Jun-07	2007	ML						
3027	2-Jun-07	2007	ML						
3035	2-Jun-07	2007	ML						
38	21-May-04	2004	DE						
40	21-May-04	2004	DE						
43	21-May-04	2004	DE						
41	21-May-04	2004	DE						
44	21-May-04	2004	DE						
39	21-May-04	2004	DE						
42	21-May-04	2004	DE						
45	21-May-04	2004	DE						
67	23-May-04	2004	RL	0.396226	0.113208	0.09434	0.320755	0.203008	0.333333
68	23-May-04	2004	RL	0.396226	0.113208	0.09434	0.320755	0.203008	0.333333
69	23-May-04	2004	RL	0.396226	0.113208	0.09434	0.320755	0.203008	0.333333
76	23-May-04	2004	RL	0.396226	0.113208	0.09434	0.320755	0.203008	0.333333
77	23-May-04	2004	RL	0.396226	0.113208	0.09434	0.320755	0.203008	0.333333
78	23-May-04	2004	RL	0.396226	0.113208	0.09434	0.320755	0.203008	0.333333
84	23-May-04	2004	RL	0.396226	0.113208	0.09434	0.320755	0.203008	0.333333
73	23-May-04	2004	RL	0.396226	0.113208	0.09434	0.320755	0.203008	0.333333
74	23-May-04	2004	RL	0.396226	0.113208	0.09434	0.320755	0.203008	0.333333
83	23-May-04	2004	RL	0.396226	0.113208	0.09434	0.320755	0.203008	0.333333
80	23-May-04	2004	RL	0.396226	0.113208	0.09434	0.320755	0.203008	0.333333
81	23-May-04	2004	RL	0.396226	0.113208	0.09434	0.320755	0.203008	0.333333
82	23-May-04	2004	RL	0.396226	0.113208	0.09434	0.320755	0.203008	0.333333
86	23-May-04	2004	RL	0.396226	0.113208	0.09434	0.320755	0.203008	0.333333
72	23-May-04	2004	RL	0.396226	0.113208	0.09434	0.320755	0.203008	0.333333
85	23-May-04	2004	RL	0.396226	0.113208	0.09434	0.320755	0.203008	0.333333
79	23-May-04	2004	RL	0.396226	0.113208	0.09434	0.320755	0.203008	0.333333
65	23-May-04	2004	RL	0.396226	0.113208	0.09434	0.320755	0.203008	0.333333
75	23-May-04	2004	RL	0.396226	0.113208	0.09434	0.320755	0.203008	0.333333
71	23-May-04	2004	RL	0.396226	0.113208	0.09434	0.320755	0.203008	0.333333

87	23-May-04	2004	RL	0.396226	0.113208	0.09434	0.320755	0.203008	0.333333
88	23-May-04	2004	RL	0.396226	0.113208	0.09434	0.320755	0.203008	0.333333
70	23-May-04	2004	RL	0.396226	0.113208	0.09434	0.320755	0.203008	0.333333
89	23-May-04	2004	RL	0.396226	0.113208	0.09434	0.320755	0.203008	0.333333
66	23-May-04	2004	RL	0.396226	0.113208	0.09434	0.320755	0.203008	0.333333
90	27-May-04	2004	SC	0.174312	0.009174	0.06422	0.018349	0.103343	6.846154
91	27-May-04	2004	SC	0.174312	0.009174	0.06422	0.018349	0.103343	6.846154
95	27-May-04	2004	SC	0.174312	0.009174	0.06422	0.018349	0.103343	6.846154
94	27-May-04	2004	SC	0.174312	0.009174	0.06422	0.018349	0.103343	6.846154
96	27-May-04	2004	SC	0.174312	0.009174	0.06422	0.018349	0.103343	6.846154
93	27-May-04	2004	SC	0.174312	0.009174	0.06422	0.018349	0.103343	6.846154
97	27-May-04	2004	SC	0.174312	0.009174	0.06422	0.018349	0.103343	6.846154
92	27-May-04	2004	SC	0.174312	0.009174	0.06422	0.018349	0.103343	6.846154
98	27-May-04	2004	SC	0.174312	0.009174	0.06422	0.018349	0.103343	6.846154
99	27-May-04	2004	SC	0.174312	0.009174	0.06422	0.018349	0.103343	6.846154
100	27-May-04	2004	SC	0.174312	0.009174	0.06422	0.018349	0.103343	6.846154
154	27-May-04	2004	SC	0.174312	0.009174	0.06422	0.018349	0.103343	6.846154
155	27-May-04	2004	SC	0.174312	0.009174	0.06422	0.018349	0.103343	6.846154
157	27-May-04	2004	SC	0.174312	0.009174	0.06422	0.018349	0.103343	6.846154
153	27-May-04	2004	SC	0.174312	0.009174	0.06422	0.018349	0.103343	6.846154
156	27-May-04	2004	SC	0.174312	0.009174	0.06422	0.018349	0.103343	6.846154
158	27-May-04	2004	SC	0.174312	0.009174	0.06422	0.018349	0.103343	6.846154
160	27-May-04	2004	SC	0.174312	0.009174	0.06422	0.018349	0.103343	6.846154
159	27-May-04	2004	SC	0.174312	0.009174	0.06422	0.018349	0.103343	6.846154
197	28-May-04	2004	RC	0.428571	0.107143	0	0	0.401709	3
198	28-May-04	2004	RC	0.428571	0.107143	0	0	0.401709	3
200	28-May-04	2004	RC	0.428571	0.107143	0	0	0.401709	3
196	28-May-04	2004	RC	0.428571	0.107143	0	0	0.401709	3
199	28-May-04	2004	RC	0.428571	0.107143	0	0	0.401709	3
195	28-May-04	2004	RC	0.428571	0.107143	0	0	0.401709	3
457	3-Jun-04	2004	D1/D2						
458	3-Jun-04	2004	D1/D2						
451	3-Jun-04	2004	D1/D2						
453	3-Jun-04	2004	D1/D2						
454	3-Jun-04	2004	D1/D2						
459	3-Jun-04	2004	D1/D2						
460	3-Jun-04	2004	D1/D2						
443	3-Jun-04	2004	D1/D2						
455	3-Jun-04	2004	D1/D2						
442	3-Jun-04	2004	D1/D2						
450	3-Jun-04	2004	D1/D2						
456	3-Jun-04	2004	D1/D2						
441	3-Jun-04	2004	D1/D2						
449	3-Jun-04	2004	D1/D2						

452	3-Jun-04	2004	D1/D2						
444	3-Jun-04	2004	D1/D2						
440	3-Jun-04	2004	D1/D2						
461	3-Jun-04	2004	D1/D2						
445	3-Jun-04	2004	D1/D2						
447	3-Jun-04	2004	D1/D2						
448	3-Jun-04	2004	D1/D2						
463	3-Jun-04	2004	D1/D2						
446	3-Jun-04	2004	D1/D2						
462	3-Jun-04	2004	D1/D2						
534	4-Jun-04	2004	ML						
535	4-Jun-04	2004	ML						
525	4-Jun-04	2004	ML						
527	4-Jun-04	2004	ML						
528	4-Jun-04	2004	ML						
529	4-Jun-04	2004	ML						
531	4-Jun-04	2004	ML						
530	4-Jun-04	2004	ML						
533	4-Jun-04	2004	ML						
524	4-Jun-04	2004	ML						
532	4-Jun-04	2004	ML						
522	4-Jun-04	2004	ML						
510	4-Jun-04	2004	ML						
513	4-Jun-04	2004	ML						
517	4-Jun-04	2004	ML						
518	4-Jun-04	2004	ML						
515	4-Jun-04	2004	ML						
514	4-Jun-04	2004	ML						
516	4-Jun-04	2004	ML						
520	4-Jun-04	2004	ML						
526	4-Jun-04	2004	ML						
519	4-Jun-04	2004	ML						
511	4-Jun-04	2004	ML						
523	4-Jun-04	2004	ML						
512	4-Jun-04	2004	ML						
521	4-Jun-04	2004	ML						
536	13-Jun-04	2004	SD	0.412698	0.301587	0.063492	0.634921	0.097297	2.642857
549	16-Jun-04	2004	RP	0.095238	0.142857	0.095238	0.071429	0.323383	2
550	16-Jun-04	2004	RP	0.095238	0.142857	0.095238	0.071429	0.323383	2
547	16-Jun-04	2004	RP	0.095238	0.142857	0.095238	0.071429	0.323383	2
548	16-Jun-04	2004	RP	0.095238	0.142857	0.095238	0.071429	0.323383	2
545	16-Jun-04	2004	RP	0.095238	0.142857	0.095238	0.071429	0.323383	2
546	16-Jun-04	2004	RP	0.095238	0.142857	0.095238	0.071429	0.323383	2
586	17-Jun-04	2004	RC	0.428571	0.107143	0	0	0.401709	3

585	17-Jun-04	2004	RC	0.428571	0.107143	0	0	0.401709	3
587	17-Jun-04	2004	RC	0.428571	0.107143	0	0	0.401709	3
584	17-Jun-04	2004	RC	0.428571	0.107143	0	0	0.401709	3
600	18-Jun-04	2004	DE						
593	18-Jun-04	2004	DE						
599	18-Jun-04	2004	DE						
591	18-Jun-04	2004	DE						
596	18-Jun-04	2004	DE						
602	18-Jun-04	2004	DE						
592	18-Jun-04	2004	DE						
589	18-Jun-04	2004	DE						
590	18-Jun-04	2004	DE						
595	18-Jun-04	2004	DE						
594	18-Jun-04	2004	DE						
601	18-Jun-04	2004	DE						
597	18-Jun-04	2004	DE						
598	18-Jun-04	2004	DE						
588	18-Jun-04	2004	DE						
621	18-Jun-04	2004	DE						
620	18-Jun-04	2004	DE						
619	18-Jun-04	2004	DE						
622	18-Jun-04	2004	DE						
618	18-Jun-04	2004	DE						
676	19-Jun-04	2004	RP	0.095238	0.142857	0.095238	0.071429	0.323383	2
678	19-Jun-04	2004	RP	0.095238	0.142857	0.095238	0.071429	0.323383	2
680	19-Jun-04	2004	RP	0.095238	0.142857	0.095238	0.071429	0.323383	2
675	19-Jun-04	2004	RP	0.095238	0.142857	0.095238	0.071429	0.323383	2
674	19-Jun-04	2004	RP	0.095238	0.142857	0.095238	0.071429	0.323383	2
677	19-Jun-04	2004	RP	0.095238	0.142857	0.095238	0.071429	0.323383	2
679	19-Jun-04	2004	RP	0.095238	0.142857	0.095238	0.071429	0.323383	2
689	20-Jun-04	2004	ML						
688	20-Jun-04	2004	ML						
686	20-Jun-04	2004	ML						
687	20-Jun-04	2004	ML						
684	20-Jun-04	2004	ML						
681	20-Jun-04	2004	ML						
682	20-Jun-04	2004	ML						
685	20-Jun-04	2004	ML						
683	20-Jun-04	2004	ML						
811	22-Jun-04	2004	RP	0.095238	0.142857	0.095238	0.071429	0.323383	2
812	22-Jun-04	2004	RP	0.095238	0.142857	0.095238	0.071429	0.323383	2
814	22-Jun-04	2004	RP	0.095238	0.142857	0.095238	0.071429	0.323383	2
815	22-Jun-04	2004	RP	0.095238	0.142857	0.095238	0.071429	0.323383	2
816	22-Jun-04	2004	RP	0.095238	0.142857	0.095238	0.071429	0.323383	2

817	22-Jun-04	2004	RP	0.095238	0.142857	0.095238	0.071429	0.323383	2
810	22-Jun-04	2004	RP	0.095238	0.142857	0.095238	0.071429	0.323383	2
813	22-Jun-04	2004	RP	0.095238	0.142857	0.095238	0.071429	0.323383	2
809	22-Jun-04	2004	RP	0.095238	0.142857	0.095238	0.071429	0.323383	2
808	22-Jun-04	2004	RP	0.095238	0.142857	0.095238	0.071429	0.323383	2
746	24-Jun-04	2004	RL	0.396226	0.113208	0.09434	0.320755	0.203008	0.333333
755	24-Jun-04	2004	RL	0.396226	0.113208	0.09434	0.320755	0.203008	0.333333
743	24-Jun-04	2004	RL	0.396226	0.113208	0.09434	0.320755	0.203008	0.333333
747	24-Jun-04	2004	RL	0.396226	0.113208	0.09434	0.320755	0.203008	0.333333
753	24-Jun-04	2004	RL	0.396226	0.113208	0.09434	0.320755	0.203008	0.333333
756	24-Jun-04	2004	RL	0.396226	0.113208	0.09434	0.320755	0.203008	0.333333
757	24-Jun-04	2004	RL	0.396226	0.113208	0.09434	0.320755	0.203008	0.333333
758	24-Jun-04	2004	RL	0.396226	0.113208	0.09434	0.320755	0.203008	0.333333
742	24-Jun-04	2004	RL	0.396226	0.113208	0.09434	0.320755	0.203008	0.333333
750	24-Jun-04	2004	RL	0.396226	0.113208	0.09434	0.320755	0.203008	0.333333
751	24-Jun-04	2004	RL	0.396226	0.113208	0.09434	0.320755	0.203008	0.333333
754	24-Jun-04	2004	RL	0.396226	0.113208	0.09434	0.320755	0.203008	0.333333
749	24-Jun-04	2004	RL	0.396226	0.113208	0.09434	0.320755	0.203008	0.333333
748	24-Jun-04	2004	RL	0.396226	0.113208	0.09434	0.320755	0.203008	0.333333
745	24-Jun-04	2004	RL	0.396226	0.113208	0.09434	0.320755	0.203008	0.333333
752	24-Jun-04	2004	RL	0.396226	0.113208	0.09434	0.320755	0.203008	0.333333
744	24-Jun-04	2004	RL	0.396226	0.113208	0.09434	0.320755	0.203008	0.333333
783	30-Jun-04	2004	RP	0.095238	0.142857	0.095238	0.071429	0.323383	2
784	30-Jun-04	2004	RP	0.095238	0.142857	0.095238	0.071429	0.323383	2
781	30-Jun-04	2004	RP	0.095238	0.142857	0.095238	0.071429	0.323383	2
782	30-Jun-04	2004	RP	0.095238	0.142857	0.095238	0.071429	0.323383	2
786	30-Jun-04	2004	RP	0.095238	0.142857	0.095238	0.071429	0.323383	2
785	30-Jun-04	2004	RP	0.095238	0.142857	0.095238	0.071429	0.323383	2
39	8-May-05	2005	SC	0.183486	0	0.073394	0	0.006329	6.769231
37	8-May-05	2005	SC	0.183486	0	0.073394	0	0.006329	6.769231
36	8-May-05	2005	SC	0.183486	0	0.073394	0	0.006329	6.769231
41	8-May-05	2005	SC	0.183486	0	0.073394	0	0.006329	6.769231
35	8-May-05	2005	SC	0.183486	0	0.073394	0	0.006329	6.769231
40	8-May-05	2005	SC	0.183486	0	0.073394	0	0.006329	6.769231
42	8-May-05	2005	SC	0.183486	0	0.073394	0	0.006329	6.769231
38	8-May-05	2005	SC	0.183486	0	0.073394	0	0.006329	6.769231
53	9-May-05	2005	ML						
49	9-May-05	2005	ML						
51	9-May-05	2005	ML						
50	9-May-05	2005	ML						
48	9-May-05	2005	ML						
52	9-May-05	2005	ML						
47	9-May-05	2005	ML						
54	9-May-05	2005	ML						

57	9-May-05	2005	ML							
56	9-May-05	2005	ML							
55	9-May-05	2005	ML							
104	11-May-05	2005	RL		0.465517	0.103448	0	0.137931	0.108209	0.333333
103	11-May-05	2005	RL		0.465517	0.103448	0	0.137931	0.108209	0.333333
107	11-May-05	2005	RL		0.465517	0.103448	0	0.137931	0.108209	0.333333
108	11-May-05	2005	RL		0.465517	0.103448	0	0.137931	0.108209	0.333333
106	11-May-05	2005	RL		0.465517	0.103448	0	0.137931	0.108209	0.333333
109	11-May-05	2005	RL		0.465517	0.103448	0	0.137931	0.108209	0.333333
115	11-May-05	2005	RL		0.465517	0.103448	0	0.137931	0.108209	0.333333
105	11-May-05	2005	RL		0.465517	0.103448	0	0.137931	0.108209	0.333333
114	11-May-05	2005	RL		0.465517	0.103448	0	0.137931	0.108209	0.333333
113	11-May-05	2005	RL		0.465517	0.103448	0	0.137931	0.108209	0.333333
112	11-May-05	2005	RL		0.465517	0.103448	0	0.137931	0.108209	0.333333
111	11-May-05	2005	RL		0.465517	0.103448	0	0.137931	0.108209	0.333333
116	11-May-05	2005	RL		0.465517	0.103448	0	0.137931	0.108209	0.333333
117	11-May-05	2005	RL		0.465517	0.103448	0	0.137931	0.108209	0.333333
110	11-May-05	2005	RL		0.465517	0.103448	0	0.137931	0.108209	0.333333
102	11-May-05	2005	RL		0.465517	0.103448	0	0.137931	0.108209	0.333333
336	17-May-05	2005	SD		0.365079	0.095238	0.095238	0.380952	0.048387	2.5
338	17-May-05	2005	SD		0.365079	0.095238	0.095238	0.380952	0.048387	2.5
335	17-May-05	2005	SD		0.365079	0.095238	0.095238	0.380952	0.048387	2.5
337	17-May-05	2005	SD		0.365079	0.095238	0.095238	0.380952	0.048387	2.5
334	17-May-05	2005	SD		0.365079	0.095238	0.095238	0.380952	0.048387	2.5
329	17-May-05	2005	SD		0.365079	0.095238	0.095238	0.380952	0.048387	2.5
331	17-May-05	2005	SD		0.365079	0.095238	0.095238	0.380952	0.048387	2.5
332	17-May-05	2005	SD		0.365079	0.095238	0.095238	0.380952	0.048387	2.5
333	17-May-05	2005	SD		0.365079	0.095238	0.095238	0.380952	0.048387	2.5
339	17-May-05	2005	SD		0.365079	0.095238	0.095238	0.380952	0.048387	2.5
330	17-May-05	2005	SD		0.365079	0.095238	0.095238	0.380952	0.048387	2.5
340	17-May-05	2005	SD		0.365079	0.095238	0.095238	0.380952	0.048387	2.5
341	17-May-05	2005	SD		0.365079	0.095238	0.095238	0.380952	0.048387	2.5
404	18-May-05	2005	SC		0.183486	0	0.073394	0	0.006329	6.769231
405	18-May-05	2005	SC		0.183486	0	0.073394	0	0.006329	6.769231
408	18-May-05	2005	SC		0.183486	0	0.073394	0	0.006329	6.769231
407	18-May-05	2005	SC		0.183486	0	0.073394	0	0.006329	6.769231
406	18-May-05	2005	SC		0.183486	0	0.073394	0	0.006329	6.769231
409	18-May-05	2005	SC		0.183486	0	0.073394	0	0.006329	6.769231
403	18-May-05	2005	SC		0.183486	0	0.073394	0	0.006329	6.769231
447	19-May-05	2005	RC		0.185185	0	0	0.037037	0.155172	2.875
450	20-May-05	2005	7M		0.264151	0.09434	0	0.301887	0.01519	8.266667
449	20-May-05	2005	7M		0.264151	0.09434	0	0.301887	0.01519	8.266667
448	20-May-05	2005	7M		0.264151	0.09434	0	0.301887	0.01519	8.266667
451	20-May-05	2005	7M		0.264151	0.09434	0	0.301887	0.01519	8.266667

454	20-May-05	2005	7M	0.264151	0.09434	0	0.301887	0.01519	8.266667
453	20-May-05	2005	7M	0.264151	0.09434	0	0.301887	0.01519	8.266667
452	20-May-05	2005	7M	0.264151	0.09434	0	0.301887	0.01519	8.266667
468	20-May-05	2005	7M	0.264151	0.09434	0	0.301887	0.01519	8.266667
467	20-May-05	2005	7M	0.264151	0.09434	0	0.301887	0.01519	8.266667
469	20-May-05	2005	7M	0.264151	0.09434	0	0.301887	0.01519	8.266667
466	20-May-05	2005	7M	0.264151	0.09434	0	0.301887	0.01519	8.266667
471	20-May-05	2005	7M	0.264151	0.09434	0	0.301887	0.01519	8.266667
464	20-May-05	2005	7M	0.264151	0.09434	0	0.301887	0.01519	8.266667
465	20-May-05	2005	7M	0.264151	0.09434	0	0.301887	0.01519	8.266667
478	20-May-05	2005	7M	0.264151	0.09434	0	0.301887	0.01519	8.266667
480	20-May-05	2005	7M	0.264151	0.09434	0	0.301887	0.01519	8.266667
481	20-May-05	2005	7M	0.264151	0.09434	0	0.301887	0.01519	8.266667
473	20-May-05	2005	7M	0.264151	0.09434	0	0.301887	0.01519	8.266667
476	20-May-05	2005	7M	0.264151	0.09434	0	0.301887	0.01519	8.266667
475	20-May-05	2005	7M	0.264151	0.09434	0	0.301887	0.01519	8.266667
477	20-May-05	2005	7M	0.264151	0.09434	0	0.301887	0.01519	8.266667
474	20-May-05	2005	7M	0.264151	0.09434	0	0.301887	0.01519	8.266667
479	20-May-05	2005	7M	0.264151	0.09434	0	0.301887	0.01519	8.266667
472	20-May-05	2005	7M	0.264151	0.09434	0	0.301887	0.01519	8.266667
470	20-May-05	2005	7M	0.264151	0.09434	0	0.301887	0.01519	8.266667
36	10-May-06	2006	SC	0.092308	0	0.030769	0.046154	0.002941	7.961538
37	10-May-06	2006	SC	0.092308	0	0.030769	0.046154	0.002941	7.961538
38	10-May-06	2006	SC	0.092308	0	0.030769	0.046154	0.002941	7.961538
35	10-May-06	2006	SC	0.092308	0	0.030769	0.046154	0.002941	7.961538
39	10-May-06	2006	SC	0.092308	0	0.030769	0.046154	0.002941	7.961538
40	10-May-06	2006	SC	0.092308	0	0.030769	0.046154	0.002941	7.961538
41	10-May-06	2006	SC	0.092308	0	0.030769	0.046154	0.002941	7.961538
75	17-May-06	2006	LT1	0.222222	0.111111	0	0.148148	0	4
72	17-May-06	2006	LT1	0.222222	0.111111	0	0.148148	0	4
69	17-May-06	2006	LT1	0.222222	0.111111	0	0.148148	0	4
71	17-May-06	2006	LT1	0.222222	0.111111	0	0.148148	0	4
73	17-May-06	2006	LT1	0.222222	0.111111	0	0.148148	0	4
74	17-May-06	2006	LT1	0.222222	0.111111	0	0.148148	0	4
68	17-May-06	2006	LT1	0.222222	0.111111	0	0.148148	0	4
67	17-May-06	2006	LT1	0.222222	0.111111	0	0.148148	0	4
70	17-May-06	2006	LT1	0.222222	0.111111	0	0.148148	0	4
	11-Jun-06	2006	D2	0.111111	0	0.055556	0.327273	0	3.181818
	11-Jun-06	2006	D2	0.111111	0	0.055556	0.327273	0	3.181818
	11-Jun-06	2006	D2	0.111111	0	0.055556	0.327273	0	3.181818
	11-Jun-06	2006	D2	0.111111	0	0.055556	0.327273	0	3.181818
	11-Jun-06	2006	D2	0.111111	0	0.055556	0.327273	0	3.181818
	11-Jun-06	2006	D2	0.111111	0	0.055556	0.327273	0	3.181818
	11-Jun-06	2006	D2	0.111111	0	0.055556	0.327273	0	3.181818

	11-Jun-06	2006	D2	0.111111	0	0.055556	0.327273	0	3.181818
	11-Jun-06	2006	D2	0.111111	0	0.055556	0.327273	0	3.181818
	11-Jun-06	2006	D2	0.111111	0	0.055556	0.327273	0	3.181818
	22-Jun-06	2006	SC	0.092308	0	0.030769	0.046154	0.002941	7.961538
	22-Jun-06	2006	SC	0.092308	0	0.030769	0.046154	0.002941	7.961538
	22-Jun-06	2006	SC	0.092308	0	0.030769	0.046154	0.002941	7.961538
	22-Jun-06	2006	SC	0.092308	0	0.030769	0.046154	0.002941	7.961538
	22-Jun-06	2006	SC	0.092308	0	0.030769	0.046154	0.002941	7.961538
	28-Jun-06	2006	HH	0.102941	0.161765	0	0.352941	0	0.615385
	28-Jun-06	2006	HH	0.102941	0.161765	0	0.352941	0	0.615385
	28-Jun-06	2006	HH	0.102941	0.161765	0	0.352941	0	0.615385
	28-Jun-06	2006	HH	0.102941	0.161765	0	0.352941	0	0.615385
	28-Jun-06	2006	HH	0.102941	0.161765	0	0.352941	0	0.615385
	28-Jun-06	2006	HH	0.102941	0.161765	0	0.352941	0	0.615385
	28-Jun-06	2006	HH	0.102941	0.161765	0	0.352941	0	0.615385
	28-Jun-06	2006	HH	0.102941	0.161765	0	0.352941	0	0.615385
	28-Jun-06	2006	HH	0.102941	0.161765	0	0.352941	0	0.615385
	28-Jun-06	2006	HH	0.102941	0.161765	0	0.352941	0	0.615385
	28-Jun-06	2006	HH	0.102941	0.161765	0	0.352941	0	0.615385
	28-Jun-06	2006	HH	0.102941	0.161765	0	0.352941	0	0.615385
	28-Jun-06	2006	LT1	0.222222	0.111111	0	0.148148	0	4
	28-Jun-06	2006	LT1	0.222222	0.111111	0	0.148148	0	4
	28-Jun-06	2006	LT1	0.222222	0.111111	0	0.148148	0	4
	28-Jun-06	2006	LT1	0.222222	0.111111	0	0.148148	0	4
	28-Jun-06	2006	LT1	0.222222	0.111111	0	0.148148	0	4
	28-Jun-06	2006	LT1	0.222222	0.111111	0	0.148148	0	4
91	7-May-07	2007	MM	0.036364	0.090909	0.054545	0.181818	0.030769	1.545455
92	7-May-07	2007	MM	0.036364	0.090909	0.054545	0.181818	0.030769	1.545455
90	7-May-07	2007	MM	0.036364	0.090909	0.054545	0.181818	0.030769	1.545455
93	7-May-07	2007	MM	0.036364	0.090909	0.054545	0.181818	0.030769	1.545455
94	7-May-07	2007	MM	0.036364	0.090909	0.054545	0.181818	0.030769	1.545455
99	7-May-07	2007	MM	0.036364	0.090909	0.054545	0.181818	0.030769	1.545455
100	7-May-07	2007	MM	0.036364	0.090909	0.054545	0.181818	0.030769	1.545455
101	7-May-07	2007	MM	0.036364	0.090909	0.054545	0.181818	0.030769	1.545455
102	7-May-07	2007	MM	0.036364	0.090909	0.054545	0.181818	0.030769	1.545455
103	7-May-07	2007	MM	0.036364	0.090909	0.054545	0.181818	0.030769	1.545455
98	7-May-07	2007	MM	0.036364	0.090909	0.054545	0.181818	0.030769	1.545455
95	7-May-07	2007	MM	0.036364	0.090909	0.054545	0.181818	0.030769	1.545455
96	7-May-07	2007	MM	0.036364	0.090909	0.054545	0.181818	0.030769	1.545455
97	7-May-07	2007	MM	0.036364	0.090909	0.054545	0.181818	0.030769	1.545455
128	8-May-07	2007	MG	0.24	0.28	0.12	0.02	0.009615	6.15
129	8-May-07	2007	MG	0.24	0.28	0.12	0.02	0.009615	6.15
131	8-May-07	2007	MG	0.24	0.28	0.12	0.02	0.009615	6.15
132	8-May-07	2007	MG	0.24	0.28	0.12	0.02	0.009615	6.15

134	8-May-07	2007	MG	0.24	0.28	0.12	0.02	0.009615	6.15
135	8-May-07	2007	MG	0.24	0.28	0.12	0.02	0.009615	6.15
130	8-May-07	2007	MG	0.24	0.28	0.12	0.02	0.009615	6.15
133	8-May-07	2007	MG	0.24	0.28	0.12	0.02	0.009615	6.15
136	8-May-07	2007	MG	0.24	0.28	0.12	0.02	0.009615	6.15
137	8-May-07	2007	MG	0.24	0.28	0.12	0.02	0.009615	6.15
138	8-May-07	2007	MG	0.24	0.28	0.12	0.02	0.009615	6.15
139	8-May-07	2007	MG	0.24	0.28	0.12	0.02	0.009615	6.15
140	8-May-07	2007	MG	0.24	0.28	0.12	0.02	0.009615	6.15
124	8-May-07	2007	MG	0.24	0.28	0.12	0.02	0.009615	6.15
127	8-May-07	2007	MG	0.24	0.28	0.12	0.02	0.009615	6.15
125	8-May-07	2007	MG	0.24	0.28	0.12	0.02	0.009615	6.15
126	8-May-07	2007	MG	0.24	0.28	0.12	0.02	0.009615	6.15
123	8-May-07	2007	MG	0.24	0.28	0.12	0.02	0.009615	6.15
644	9-May-07	2007	SW	0.157895	0.078947	0.026316	0	0	1.4
646	9-May-07	2007	SW	0.157895	0.078947	0.026316	0	0	1.4
648	9-May-07	2007	SW	0.157895	0.078947	0.026316	0	0	1.4
647	9-May-07	2007	SW	0.157895	0.078947	0.026316	0	0	1.4
642	9-May-07	2007	SW	0.157895	0.078947	0.026316	0	0	1.4
645	9-May-07	2007	SW	0.157895	0.078947	0.026316	0	0	1.4
643	9-May-07	2007	SW	0.157895	0.078947	0.026316	0	0	1.4
649	9-May-07	2007	SW	0.157895	0.078947	0.026316	0	0	1.4
650	9-May-07	2007	SW	0.157895	0.078947	0.026316	0	0	1.4
651	9-May-07	2007	SW	0.157895	0.078947	0.026316	0	0	1.4
652	9-May-07	2007	SW	0.157895	0.078947	0.026316	0	0	1.4
653	9-May-07	2007	SW	0.157895	0.078947	0.026316	0	0	1.4
655	9-May-07	2007	SW	0.157895	0.078947	0.026316	0	0	1.4
656	9-May-07	2007	SW	0.157895	0.078947	0.026316	0	0	1.4
657	9-May-07	2007	SW	0.157895	0.078947	0.026316	0	0	1.4
659	9-May-07	2007	SW	0.157895	0.078947	0.026316	0	0	1.4
660	9-May-07	2007	SW	0.157895	0.078947	0.026316	0	0	1.4
661	9-May-07	2007	SW	0.157895	0.078947	0.026316	0	0	1.4
641	9-May-07	2007	SW	0.157895	0.078947	0.026316	0	0	1.4
654	9-May-07	2007	SW	0.157895	0.078947	0.026316	0	0	1.4
658	9-May-07	2007	SW	0.157895	0.078947	0.026316	0	0	1.4
719	10-May-07	2007	SHAC	0.071429	0.071429	0	0.098361	0.008818	2.227273
720	10-May-07	2007	SHAC	0.071429	0.071429	0	0.098361	0.008818	2.227273
721	10-May-07	2007	SHAC	0.071429	0.071429	0	0.098361	0.008818	2.227273
722	10-May-07	2007	SHAC	0.071429	0.071429	0	0.098361	0.008818	2.227273
723	10-May-07	2007	SHAC	0.071429	0.071429	0	0.098361	0.008818	2.227273
716	10-May-07	2007	SHAC	0.071429	0.071429	0	0.098361	0.008818	2.227273
717	10-May-07	2007	SHAC	0.071429	0.071429	0	0.098361	0.008818	2.227273
728	10-May-07	2007	SHAC	0.071429	0.071429	0	0.098361	0.008818	2.227273
715	10-May-07	2007	SHAC	0.071429	0.071429	0	0.098361	0.008818	2.227273

731	10-May-07	2007	SHAC	0.071429	0.071429	0	0.098361	0.008818	2.227273
732	10-May-07	2007	SHAC	0.071429	0.071429	0	0.098361	0.008818	2.227273
733	10-May-07	2007	SHAC	0.071429	0.071429	0	0.098361	0.008818	2.227273
704	10-May-07	2007	SHAC	0.071429	0.071429	0	0.098361	0.008818	2.227273
724	10-May-07	2007	SHAC	0.071429	0.071429	0	0.098361	0.008818	2.227273
725	10-May-07	2007	SHAC	0.071429	0.071429	0	0.098361	0.008818	2.227273
726	10-May-07	2007	SHAC	0.071429	0.071429	0	0.098361	0.008818	2.227273
730	10-May-07	2007	SHAC	0.071429	0.071429	0	0.098361	0.008818	2.227273
705	10-May-07	2007	SHAC	0.071429	0.071429	0	0.098361	0.008818	2.227273
711	10-May-07	2007	SHAC	0.071429	0.071429	0	0.098361	0.008818	2.227273
729	10-May-07	2007	SHAC	0.071429	0.071429	0	0.098361	0.008818	2.227273
707	10-May-07	2007	SHAC	0.071429	0.071429	0	0.098361	0.008818	2.227273
706	10-May-07	2007	SHAC	0.071429	0.071429	0	0.098361	0.008818	2.227273
708	10-May-07	2007	SHAC	0.071429	0.071429	0	0.098361	0.008818	2.227273
709	10-May-07	2007	SHAC	0.071429	0.071429	0	0.098361	0.008818	2.227273
710	10-May-07	2007	SHAC	0.071429	0.071429	0	0.098361	0.008818	2.227273
718	10-May-07	2007	SHAC	0.071429	0.071429	0	0.098361	0.008818	2.227273
712	10-May-07	2007	SHAC	0.071429	0.071429	0	0.098361	0.008818	2.227273
713	10-May-07	2007	SHAC	0.071429	0.071429	0	0.098361	0.008818	2.227273
703	10-May-07	2007	SHAC	0.071429	0.071429	0	0.098361	0.008818	2.227273
714	10-May-07	2007	SHAC	0.071429	0.071429	0	0.098361	0.008818	2.227273
727	10-May-07	2007	SHAC	0.071429	0.071429	0	0.098361	0.008818	2.227273
803	10-May-07	2007	SHAC	0.071429	0.071429	0	0.098361	0.008818	2.227273
795	10-May-07	2007	SHAC	0.071429	0.071429	0	0.098361	0.008818	2.227273
794	10-May-07	2007	SHAC	0.071429	0.071429	0	0.098361	0.008818	2.227273
792	10-May-07	2007	SHAC	0.071429	0.071429	0	0.098361	0.008818	2.227273
793	10-May-07	2007	SHAC	0.071429	0.071429	0	0.098361	0.008818	2.227273
791	10-May-07	2007	SHAC	0.071429	0.071429	0	0.098361	0.008818	2.227273
790	10-May-07	2007	SHAC	0.071429	0.071429	0	0.098361	0.008818	2.227273
796	10-May-07	2007	SHAC	0.071429	0.071429	0	0.098361	0.008818	2.227273
799	10-May-07	2007	SHAC	0.071429	0.071429	0	0.098361	0.008818	2.227273
800	10-May-07	2007	SHAC	0.071429	0.071429	0	0.098361	0.008818	2.227273
801	10-May-07	2007	SHAC	0.071429	0.071429	0	0.098361	0.008818	2.227273
798	10-May-07	2007	SHAC	0.071429	0.071429	0	0.098361	0.008818	2.227273
789	10-May-07	2007	SHAC	0.071429	0.071429	0	0.098361	0.008818	2.227273
797	10-May-07	2007	SHAC	0.071429	0.071429	0	0.098361	0.008818	2.227273
802	10-May-07	2007	SHAC	0.071429	0.071429	0	0.098361	0.008818	2.227273
207	11-May-07	2007	AFF						
204	11-May-07	2007	AFF						
205	11-May-07	2007	AFF						
206	11-May-07	2007	AFF						
203	11-May-07	2007	AFF						
202	11-May-07	2007	AFF						
208	11-May-07	2007	AFF						

209	11-May-07	2007	AFF						
210	11-May-07	2007	AFF						
212	11-May-07	2007	AFF						
214	11-May-07	2007	AFF						
199	11-May-07	2007	AFF						
200	11-May-07	2007	AFF						
211	11-May-07	2007	AFF						
197	11-May-07	2007	AFF						
198	11-May-07	2007	AFF						
213	11-May-07	2007	AFF						
195	11-May-07	2007	AFF						
196	11-May-07	2007	AFF						
201	11-May-07	2007	AFF						
215	11-May-07	2007	AFC						
216	11-May-07	2007	AFC						
217	11-May-07	2007	AFC						
272	12-May-07	2007	RL	0.157895	0.157895	0	0	0.028571	0.333333
268	12-May-07	2007	RL	0.157895	0.157895	0	0	0.028571	0.333333
269	12-May-07	2007	RL	0.157895	0.157895	0	0	0.028571	0.333333
270	12-May-07	2007	RL	0.157895	0.157895	0	0	0.028571	0.333333
271	12-May-07	2007	RL	0.157895	0.157895	0	0	0.028571	0.333333
267	12-May-07	2007	RL	0.157895	0.157895	0	0	0.028571	0.333333
266	12-May-07	2007	RL	0.157895	0.157895	0	0	0.028571	0.333333
260	12-May-07	2007	RL	0.157895	0.157895	0	0	0.028571	0.333333
261	12-May-07	2007	RL	0.157895	0.157895	0	0	0.028571	0.333333
262	12-May-07	2007	RL	0.157895	0.157895	0	0	0.028571	0.333333
263	12-May-07	2007	RL	0.157895	0.157895	0	0	0.028571	0.333333
273	12-May-07	2007	RL	0.157895	0.157895	0	0	0.028571	0.333333
259	12-May-07	2007	RL	0.157895	0.157895	0	0	0.028571	0.333333
264	12-May-07	2007	RL	0.157895	0.157895	0	0	0.028571	0.333333
265	12-May-07	2007	RL	0.157895	0.157895	0	0	0.028571	0.333333
332	12-May-07	2007	RL	0.157895	0.157895	0	0	0.028571	0.333333
334	12-May-07	2007	RL	0.157895	0.157895	0	0	0.028571	0.333333
331	12-May-07	2007	RL	0.157895	0.157895	0	0	0.028571	0.333333
312	12-May-07	2007	RL	0.157895	0.157895	0	0	0.028571	0.333333
313	12-May-07	2007	RL	0.157895	0.157895	0	0	0.028571	0.333333
314	12-May-07	2007	RL	0.157895	0.157895	0	0	0.028571	0.333333
315	12-May-07	2007	RL	0.157895	0.157895	0	0	0.028571	0.333333
316	12-May-07	2007	RL	0.157895	0.157895	0	0	0.028571	0.333333
310	12-May-07	2007	RL	0.157895	0.157895	0	0	0.028571	0.333333
318	12-May-07	2007	RL	0.157895	0.157895	0	0	0.028571	0.333333
319	12-May-07	2007	RL	0.157895	0.157895	0	0	0.028571	0.333333
308	12-May-07	2007	RL	0.157895	0.157895	0	0	0.028571	0.333333
309	12-May-07	2007	RL	0.157895	0.157895	0	0	0.028571	0.333333

322	12-May-07	2007	RL	0.157895	0.157895	0	0	0.028571	0.333333
328	12-May-07	2007	RL	0.157895	0.157895	0	0	0.028571	0.333333
329	12-May-07	2007	RL	0.157895	0.157895	0	0	0.028571	0.333333
333	12-May-07	2007	RL	0.157895	0.157895	0	0	0.028571	0.333333
335	12-May-07	2007	RL	0.157895	0.157895	0	0	0.028571	0.333333
336	12-May-07	2007	RL	0.157895	0.157895	0	0	0.028571	0.333333
337	12-May-07	2007	RL	0.157895	0.157895	0	0	0.028571	0.333333
338	12-May-07	2007	RL	0.157895	0.157895	0	0	0.028571	0.333333
339	12-May-07	2007	RL	0.157895	0.157895	0	0	0.028571	0.333333
340	12-May-07	2007	RL	0.157895	0.157895	0	0	0.028571	0.333333
341	12-May-07	2007	RL	0.157895	0.157895	0	0	0.028571	0.333333
342	12-May-07	2007	RL	0.157895	0.157895	0	0	0.028571	0.333333
317	12-May-07	2007	RL	0.157895	0.157895	0	0	0.028571	0.333333
321	12-May-07	2007	RL	0.157895	0.157895	0	0	0.028571	0.333333
323	12-May-07	2007	RL	0.157895	0.157895	0	0	0.028571	0.333333
324	12-May-07	2007	RL	0.157895	0.157895	0	0	0.028571	0.333333
343	12-May-07	2007	RL	0.157895	0.157895	0	0	0.028571	0.333333
325	12-May-07	2007	RL	0.157895	0.157895	0	0	0.028571	0.333333
326	12-May-07	2007	RL	0.157895	0.157895	0	0	0.028571	0.333333
344	12-May-07	2007	RL	0.157895	0.157895	0	0	0.028571	0.333333
345	12-May-07	2007	RL	0.157895	0.157895	0	0	0.028571	0.333333
311	12-May-07	2007	RL	0.157895	0.157895	0	0	0.028571	0.333333
327	12-May-07	2007	RL	0.157895	0.157895	0	0	0.028571	0.333333
320	12-May-07	2007	RL	0.157895	0.157895	0	0	0.028571	0.333333
330	12-May-07	2007	RL	0.157895	0.157895	0	0	0.028571	0.333333
808	15-May-07	2007	RL	0.157895	0.157895	0	0	0.028571	0.333333
813	15-May-07	2007	RL	0.157895	0.157895	0	0	0.028571	0.333333
815	15-May-07	2007	RL	0.157895	0.157895	0	0	0.028571	0.333333
814	15-May-07	2007	RL	0.157895	0.157895	0	0	0.028571	0.333333
816	15-May-07	2007	RL	0.157895	0.157895	0	0	0.028571	0.333333
818	15-May-07	2007	RL	0.157895	0.157895	0	0	0.028571	0.333333
809	15-May-07	2007	RL	0.157895	0.157895	0	0	0.028571	0.333333
810	15-May-07	2007	RL	0.157895	0.157895	0	0	0.028571	0.333333
812	15-May-07	2007	RL	0.157895	0.157895	0	0	0.028571	0.333333
806	15-May-07	2007	RL	0.157895	0.157895	0	0	0.028571	0.333333
807	15-May-07	2007	RL	0.157895	0.157895	0	0	0.028571	0.333333
817	15-May-07	2007	RL	0.157895	0.157895	0	0	0.028571	0.333333
819	15-May-07	2007	RL	0.157895	0.157895	0	0	0.028571	0.333333
805	15-May-07	2007	RL	0.157895	0.157895	0	0	0.028571	0.333333
811	15-May-07	2007	RL	0.157895	0.157895	0	0	0.028571	0.333333
820	15-May-07	2007	RL	0.157895	0.157895	0	0	0.028571	0.333333
822	15-May-07	2007	RL	0.157895	0.157895	0	0	0.028571	0.333333
821	15-May-07	2007	RL	0.157895	0.157895	0	0	0.028571	0.333333
826	15-May-07	2007	ML						

825	15-May-07	2007	ML
829	15-May-07	2007	ML
830	15-May-07	2007	ML
831	15-May-07	2007	ML
824	15-May-07	2007	ML
827	15-May-07	2007	ML
828	15-May-07	2007	ML
832	15-May-07	2007	ML
905	16-May-07	2007	ML
899	16-May-07	2007	ML
907	16-May-07	2007	ML
898	16-May-07	2007	ML
911	16-May-07	2007	ML
900	16-May-07	2007	ML
891	16-May-07	2007	ML
893	16-May-07	2007	ML
903	16-May-07	2007	ML
904	16-May-07	2007	ML
902	16-May-07	2007	ML
910	16-May-07	2007	ML
859	16-May-07	2007	ML
897	16-May-07	2007	ML
888	16-May-07	2007	ML
854	16-May-07	2007	ML
858	16-May-07	2007	ML
855	16-May-07	2007	ML
856	16-May-07	2007	ML
860	16-May-07	2007	ML
861	16-May-07	2007	ML
863	16-May-07	2007	ML
864	16-May-07	2007	ML
865	16-May-07	2007	ML
866	16-May-07	2007	ML
868	16-May-07	2007	ML
869	16-May-07	2007	ML
873	16-May-07	2007	ML
875	16-May-07	2007	ML
878	16-May-07	2007	ML
880	16-May-07	2007	ML
885	16-May-07	2007	ML
886	16-May-07	2007	ML
894	16-May-07	2007	ML
895	16-May-07	2007	ML
912	16-May-07	2007	ML

913	16-May-07	2007	ML						
852	16-May-07	2007	ML						
853	16-May-07	2007	ML						
857	16-May-07	2007	ML						
867	16-May-07	2007	ML						
871	16-May-07	2007	ML						
872	16-May-07	2007	ML						
851	16-May-07	2007	ML						
870	16-May-07	2007	ML						
883	16-May-07	2007	ML						
850	16-May-07	2007	ML						
881	16-May-07	2007	ML						
862	16-May-07	2007	ML						
874	16-May-07	2007	ML						
876	16-May-07	2007	ML						
877	16-May-07	2007	ML						
879	16-May-07	2007	ML						
882	16-May-07	2007	ML						
906	16-May-07	2007	ML						
908	16-May-07	2007	ML						
890	16-May-07	2007	ML						
889	16-May-07	2007	ML						
887	16-May-07	2007	ML						
914	16-May-07	2007	ML						
909	16-May-07	2007	ML						
892	16-May-07	2007	ML						
901	16-May-07	2007	ML						
849	16-May-07	2007	ML						
884	16-May-07	2007	ML						
896	16-May-07	2007	ML						
915	16-May-07	2007	ML						
848	16-May-07	2007	ML						
968	16-May-07	2007	YY	0.2	0.08	0.06	0.08	0	0.65
971	16-May-07	2007	YY	0.2	0.08	0.06	0.08	0	0.65
967	16-May-07	2007	YY	0.2	0.08	0.06	0.08	0	0.65
969	16-May-07	2007	YY	0.2	0.08	0.06	0.08	0	0.65
970	16-May-07	2007	YY	0.2	0.08	0.06	0.08	0	0.65
962	16-May-07	2007	YY	0.2	0.08	0.06	0.08	0	0.65
963	16-May-07	2007	YY	0.2	0.08	0.06	0.08	0	0.65
964	16-May-07	2007	YY	0.2	0.08	0.06	0.08	0	0.65
965	16-May-07	2007	YY	0.2	0.08	0.06	0.08	0	0.65
960	16-May-07	2007	YY	0.2	0.08	0.06	0.08	0	0.65
961	16-May-07	2007	YY	0.2	0.08	0.06	0.08	0	0.65
966	16-May-07	2007	YY	0.2	0.08	0.06	0.08	0	0.65

1017	18-May-07	2007	7M	0.131579	0.105263	0.078947	0.052632	0.007712	8.066667
1015	18-May-07	2007	7M	0.131579	0.105263	0.078947	0.052632	0.007712	8.066667
1016	18-May-07	2007	7M	0.131579	0.105263	0.078947	0.052632	0.007712	8.066667
1018	18-May-07	2007	7M	0.131579	0.105263	0.078947	0.052632	0.007712	8.066667
1020	18-May-07	2007	7M	0.131579	0.105263	0.078947	0.052632	0.007712	8.066667
1014	18-May-07	2007	7M	0.131579	0.105263	0.078947	0.052632	0.007712	8.066667
1005	18-May-07	2007	7M	0.131579	0.105263	0.078947	0.052632	0.007712	8.066667
1008	18-May-07	2007	7M	0.131579	0.105263	0.078947	0.052632	0.007712	8.066667
1031	18-May-07	2007	7M	0.131579	0.105263	0.078947	0.052632	0.007712	8.066667
1024	18-May-07	2007	7M	0.131579	0.105263	0.078947	0.052632	0.007712	8.066667
1025	18-May-07	2007	7M	0.131579	0.105263	0.078947	0.052632	0.007712	8.066667
1026	18-May-07	2007	7M	0.131579	0.105263	0.078947	0.052632	0.007712	8.066667
1023	18-May-07	2007	7M	0.131579	0.105263	0.078947	0.052632	0.007712	8.066667
1012	18-May-07	2007	7M	0.131579	0.105263	0.078947	0.052632	0.007712	8.066667
1027	18-May-07	2007	7M	0.131579	0.105263	0.078947	0.052632	0.007712	8.066667
1028	18-May-07	2007	7M	0.131579	0.105263	0.078947	0.052632	0.007712	8.066667
1029	18-May-07	2007	7M	0.131579	0.105263	0.078947	0.052632	0.007712	8.066667
1030	18-May-07	2007	7M	0.131579	0.105263	0.078947	0.052632	0.007712	8.066667
1011	18-May-07	2007	7M	0.131579	0.105263	0.078947	0.052632	0.007712	8.066667
1021	18-May-07	2007	7M	0.131579	0.105263	0.078947	0.052632	0.007712	8.066667
1019	18-May-07	2007	7M	0.131579	0.105263	0.078947	0.052632	0.007712	8.066667
1006	18-May-07	2007	7M	0.131579	0.105263	0.078947	0.052632	0.007712	8.066667
1007	18-May-07	2007	7M	0.131579	0.105263	0.078947	0.052632	0.007712	8.066667
1009	18-May-07	2007	7M	0.131579	0.105263	0.078947	0.052632	0.007712	8.066667
1010	18-May-07	2007	7M	0.131579	0.105263	0.078947	0.052632	0.007712	8.066667
1004	18-May-07	2007	7M	0.131579	0.105263	0.078947	0.052632	0.007712	8.066667
1013	18-May-07	2007	7M	0.131579	0.105263	0.078947	0.052632	0.007712	8.066667
1022	18-May-07	2007	7M	0.131579	0.105263	0.078947	0.052632	0.007712	8.066667
1204	20-May-07	2007	FO	0.236842	0.078947	0	0	0	0.066667
1197	20-May-07	2007	FO	0.236842	0.078947	0	0	0	0.066667
1198	20-May-07	2007	FO	0.236842	0.078947	0	0	0	0.066667
1199	20-May-07	2007	FO	0.236842	0.078947	0	0	0	0.066667
1200	20-May-07	2007	FO	0.236842	0.078947	0	0	0	0.066667
1201	20-May-07	2007	FO	0.236842	0.078947	0	0	0	0.066667
1196	20-May-07	2007	FO	0.236842	0.078947	0	0	0	0.066667
1202	20-May-07	2007	FO	0.236842	0.078947	0	0	0	0.066667
1195	20-May-07	2007	FO	0.236842	0.078947	0	0	0	0.066667
1203	20-May-07	2007	FO	0.236842	0.078947	0	0	0	0.066667
1205	20-May-07	2007	FO	0.236842	0.078947	0	0	0	0.066667
1965	22-May-07	2007	HH	0.089552	0.149254	0.104478	0.029851	0	0.615385
2364	22-May-07	2007	HH	0.089552	0.149254	0.104478	0.029851	0	0.615385
1968	22-May-07	2007	HH	0.089552	0.149254	0.104478	0.029851	0	0.615385
2367	22-May-07	2007	HH	0.089552	0.149254	0.104478	0.029851	0	0.615385
1963	22-May-07	2007	HH	0.089552	0.149254	0.104478	0.029851	0	0.615385

2362	22-May-07	2007	HH	0.089552	0.149254	0.104478	0.029851	0	0.615385
1969	22-May-07	2007	HH	0.089552	0.149254	0.104478	0.029851	0	0.615385
2368	22-May-07	2007	HH	0.089552	0.149254	0.104478	0.029851	0	0.615385
1957	22-May-07	2007	HH	0.089552	0.149254	0.104478	0.029851	0	0.615385
2356	22-May-07	2007	HH	0.089552	0.149254	0.104478	0.029851	0	0.615385
1961	22-May-07	2007	HH	0.089552	0.149254	0.104478	0.029851	0	0.615385
2360	22-May-07	2007	HH	0.089552	0.149254	0.104478	0.029851	0	0.615385
1949	22-May-07	2007	HH	0.089552	0.149254	0.104478	0.029851	0	0.615385
1950	22-May-07	2007	HH	0.089552	0.149254	0.104478	0.029851	0	0.615385
2349	22-May-07	2007	HH	0.089552	0.149254	0.104478	0.029851	0	0.615385
1947	22-May-07	2007	HH	0.089552	0.149254	0.104478	0.029851	0	0.615385
1948	22-May-07	2007	HH	0.089552	0.149254	0.104478	0.029851	0	0.615385
1952	22-May-07	2007	HH	0.089552	0.149254	0.104478	0.029851	0	0.615385
2347	22-May-07	2007	HH	0.089552	0.149254	0.104478	0.029851	0	0.615385
2348	22-May-07	2007	HH	0.089552	0.149254	0.104478	0.029851	0	0.615385
2351	22-May-07	2007	HH	0.089552	0.149254	0.104478	0.029851	0	0.615385
1958	22-May-07	2007	HH	0.089552	0.149254	0.104478	0.029851	0	0.615385
1960	22-May-07	2007	HH	0.089552	0.149254	0.104478	0.029851	0	0.615385
2357	22-May-07	2007	HH	0.089552	0.149254	0.104478	0.029851	0	0.615385
2359	22-May-07	2007	HH	0.089552	0.149254	0.104478	0.029851	0	0.615385
1964	22-May-07	2007	HH	0.089552	0.149254	0.104478	0.029851	0	0.615385
2363	22-May-07	2007	HH	0.089552	0.149254	0.104478	0.029851	0	0.615385
1955	22-May-07	2007	HH	0.089552	0.149254	0.104478	0.029851	0	0.615385
2354	22-May-07	2007	HH	0.089552	0.149254	0.104478	0.029851	0	0.615385
1954	22-May-07	2007	HH	0.089552	0.149254	0.104478	0.029851	0	0.615385
2353	22-May-07	2007	HH	0.089552	0.149254	0.104478	0.029851	0	0.615385
1956	22-May-07	2007	HH	0.089552	0.149254	0.104478	0.029851	0	0.615385
2355	22-May-07	2007	HH	0.089552	0.149254	0.104478	0.029851	0	0.615385
1967	22-May-07	2007	HH	0.089552	0.149254	0.104478	0.029851	0	0.615385
2366	22-May-07	2007	HH	0.089552	0.149254	0.104478	0.029851	0	0.615385
1966	22-May-07	2007	HH	0.089552	0.149254	0.104478	0.029851	0	0.615385
2365	22-May-07	2007	HH	0.089552	0.149254	0.104478	0.029851	0	0.615385
1946	22-May-07	2007	HH	0.089552	0.149254	0.104478	0.029851	0	0.615385
1951	22-May-07	2007	HH	0.089552	0.149254	0.104478	0.029851	0	0.615385
1962	22-May-07	2007	HH	0.089552	0.149254	0.104478	0.029851	0	0.615385
2346	22-May-07	2007	HH	0.089552	0.149254	0.104478	0.029851	0	0.615385
2350	22-May-07	2007	HH	0.089552	0.149254	0.104478	0.029851	0	0.615385
2361	22-May-07	2007	HH	0.089552	0.149254	0.104478	0.029851	0	0.615385
1953	22-May-07	2007	HH	0.089552	0.149254	0.104478	0.029851	0	0.615385
1959	22-May-07	2007	HH	0.089552	0.149254	0.104478	0.029851	0	0.615385
2345	22-May-07	2007	HH	0.089552	0.149254	0.104478	0.029851	0	0.615385
2352	22-May-07	2007	HH	0.089552	0.149254	0.104478	0.029851	0	0.615385
2358	22-May-07	2007	HH	0.089552	0.149254	0.104478	0.029851	0	0.615385
1970	22-May-07	2007	HH	0.089552	0.149254	0.104478	0.029851	0	0.615385

1945	22-May-07	2007	HH	0.089552	0.149254	0.104478	0.029851	0	0.615385
2414	23-May-07	2007	SD						
2416	23-May-07	2007	SD						
2418	23-May-07	2007	SD						
2430	23-May-07	2007	SD						
2424	23-May-07	2007	SD						
2431	23-May-07	2007	SD						
2426	23-May-07	2007	SD						
2429	23-May-07	2007	SD						
2433	23-May-07	2007	SD						
2427	23-May-07	2007	SD						
2428	23-May-07	2007	SD						
2434	23-May-07	2007	SD						
2439	23-May-07	2007	SD						
2435	23-May-07	2007	SD						
2440	23-May-07	2007	SD						
2438	23-May-07	2007	SD						
2442	23-May-07	2007	SD						
2417	23-May-07	2007	SD						
2415	23-May-07	2007	SD						
2420	23-May-07	2007	SD						
2419	23-May-07	2007	SD						
2421	23-May-07	2007	SD						
2423	23-May-07	2007	SD						
2437	23-May-07	2007	SD						
2443	23-May-07	2007	SD						
2432	23-May-07	2007	SD						
2441	23-May-07	2007	SD						
2444	23-May-07	2007	SD						
2413	23-May-07	2007	SD						
2422	23-May-07	2007	SD						
2436	23-May-07	2007	SD						
2425	23-May-07	2007	SD						
2103	24-May-07	2007	ML						
2484	24-May-07	2007	ML						
2100	24-May-07	2007	ML						
2481	24-May-07	2007	ML						
2104	24-May-07	2007	ML						
2106	24-May-07	2007	ML						
2486	24-May-07	2007	ML						
2107	24-May-07	2007	ML						
2487	24-May-07	2007	ML						
2108	24-May-07	2007	ML						
2488	24-May-07	2007	ML						

2101	24-May-07	2007	ML						
2482	24-May-07	2007	ML						
2109	24-May-07	2007	ML						
2489	24-May-07	2007	ML						
2099	24-May-07	2007	ML						
2480	24-May-07	2007	ML						
2098	24-May-07	2007	ML						
2102	24-May-07	2007	ML						
2105	24-May-07	2007	ML						
2479	24-May-07	2007	ML						
2483	24-May-07	2007	ML						
2485	24-May-07	2007	ML						
2110	24-May-07	2007	ML						
2097	24-May-07	2007	ML						
2533	25-May-07	2007	MG	0.24	0.28	0.12	0.02	0.009615	6.15
2531	25-May-07	2007	MG	0.24	0.28	0.12	0.02	0.009615	6.15
2529	25-May-07	2007	MG	0.24	0.28	0.12	0.02	0.009615	6.15
2534	25-May-07	2007	MG	0.24	0.28	0.12	0.02	0.009615	6.15
2528	25-May-07	2007	MG	0.24	0.28	0.12	0.02	0.009615	6.15
2519	25-May-07	2007	MG	0.24	0.28	0.12	0.02	0.009615	6.15
2520	25-May-07	2007	MG	0.24	0.28	0.12	0.02	0.009615	6.15
2518	25-May-07	2007	MG	0.24	0.28	0.12	0.02	0.009615	6.15
2532	25-May-07	2007	MG	0.24	0.28	0.12	0.02	0.009615	6.15
2515	25-May-07	2007	MG	0.24	0.28	0.12	0.02	0.009615	6.15
2516	25-May-07	2007	MG	0.24	0.28	0.12	0.02	0.009615	6.15
2522	25-May-07	2007	MG	0.24	0.28	0.12	0.02	0.009615	6.15
2517	25-May-07	2007	MG	0.24	0.28	0.12	0.02	0.009615	6.15
2521	25-May-07	2007	MG	0.24	0.28	0.12	0.02	0.009615	6.15
2524	25-May-07	2007	MG	0.24	0.28	0.12	0.02	0.009615	6.15
2526	25-May-07	2007	MG	0.24	0.28	0.12	0.02	0.009615	6.15
2530	25-May-07	2007	MG	0.24	0.28	0.12	0.02	0.009615	6.15
2523	25-May-07	2007	MG	0.24	0.28	0.12	0.02	0.009615	6.15
2525	25-May-07	2007	MG	0.24	0.28	0.12	0.02	0.009615	6.15
2535	25-May-07	2007	MG	0.24	0.28	0.12	0.02	0.009615	6.15
2513	25-May-07	2007	MG	0.24	0.28	0.12	0.02	0.009615	6.15
2514	25-May-07	2007	MG	0.24	0.28	0.12	0.02	0.009615	6.15
2527	25-May-07	2007	MG	0.24	0.28	0.12	0.02	0.009615	6.15
2598	26-May-07	2007	SD						
2596	26-May-07	2007	SD						
2609	26-May-07	2007	SD						
2610	26-May-07	2007	SD						
2600	26-May-07	2007	SD						
2606	26-May-07	2007	SD						
2621	26-May-07	2007	SD						

2599	26-May-07	2007	SD						
2604	26-May-07	2007	SD						
2625	26-May-07	2007	SD						
2618	26-May-07	2007	SD						
2597	26-May-07	2007	SD						
2613	26-May-07	2007	SD						
2614	26-May-07	2007	SD						
2595	26-May-07	2007	SD						
2624	26-May-07	2007	SD						
2623	26-May-07	2007	SD						
2616	26-May-07	2007	SD						
2617	26-May-07	2007	SD						
2615	26-May-07	2007	SD						
2607	26-May-07	2007	SD						
2627	26-May-07	2007	SD						
2619	26-May-07	2007	SD						
2620	26-May-07	2007	SD						
2622	26-May-07	2007	SD						
2611	26-May-07	2007	SD						
2612	26-May-07	2007	SD						
2602	26-May-07	2007	SD						
2601	26-May-07	2007	SD						
2608	26-May-07	2007	SD						
2629	26-May-07	2007	SD						
2630	26-May-07	2007	SD						
2603	26-May-07	2007	SD						
2626	26-May-07	2007	SD						
2628	26-May-07	2007	SD						
2594	26-May-07	2007	SD						
2605	26-May-07	2007	SD						
2662	28-May-07	2007	D1	0.26	0.14	0.02	0.06	0.006036	0.8
2668	28-May-07	2007	D1	0.26	0.14	0.02	0.06	0.006036	0.8
2664	28-May-07	2007	D1	0.26	0.14	0.02	0.06	0.006036	0.8
2669	28-May-07	2007	D1	0.26	0.14	0.02	0.06	0.006036	0.8
2665	28-May-07	2007	D1	0.26	0.14	0.02	0.06	0.006036	0.8
2672	28-May-07	2007	D1	0.26	0.14	0.02	0.06	0.006036	0.8
2666	28-May-07	2007	D1	0.26	0.14	0.02	0.06	0.006036	0.8
2670	28-May-07	2007	D1	0.26	0.14	0.02	0.06	0.006036	0.8
2671	28-May-07	2007	D1	0.26	0.14	0.02	0.06	0.006036	0.8
2677	28-May-07	2007	D1	0.26	0.14	0.02	0.06	0.006036	0.8
2680	28-May-07	2007	D1	0.26	0.14	0.02	0.06	0.006036	0.8
2679	28-May-07	2007	D1	0.26	0.14	0.02	0.06	0.006036	0.8
2673	28-May-07	2007	D1	0.26	0.14	0.02	0.06	0.006036	0.8
2676	28-May-07	2007	D1	0.26	0.14	0.02	0.06	0.006036	0.8

2675	28-May-07	2007	D1	0.26	0.14	0.02	0.06	0.006036	0.8
2667	28-May-07	2007	D1	0.26	0.14	0.02	0.06	0.006036	0.8
2678	28-May-07	2007	D1	0.26	0.14	0.02	0.06	0.006036	0.8
2661	28-May-07	2007	D1	0.26	0.14	0.02	0.06	0.006036	0.8
2663	28-May-07	2007	D1	0.26	0.14	0.02	0.06	0.006036	0.8
2674	28-May-07	2007	D1	0.26	0.14	0.02	0.06	0.006036	0.8
2755	29-May-07	2007	D1	0.26	0.14	0.02	0.06	0.006036	0.8
2757	29-May-07	2007	D1	0.26	0.14	0.02	0.06	0.006036	0.8
2758	29-May-07	2007	D1	0.26	0.14	0.02	0.06	0.006036	0.8
2760	29-May-07	2007	D1	0.26	0.14	0.02	0.06	0.006036	0.8
2761	29-May-07	2007	D1	0.26	0.14	0.02	0.06	0.006036	0.8
2765	29-May-07	2007	D1	0.26	0.14	0.02	0.06	0.006036	0.8
2753	29-May-07	2007	D1	0.26	0.14	0.02	0.06	0.006036	0.8
2763	29-May-07	2007	D1	0.26	0.14	0.02	0.06	0.006036	0.8
2769	29-May-07	2007	D1	0.26	0.14	0.02	0.06	0.006036	0.8
2770	29-May-07	2007	D1	0.26	0.14	0.02	0.06	0.006036	0.8
2773	29-May-07	2007	D1	0.26	0.14	0.02	0.06	0.006036	0.8
2766	29-May-07	2007	D1	0.26	0.14	0.02	0.06	0.006036	0.8
2772	29-May-07	2007	D1	0.26	0.14	0.02	0.06	0.006036	0.8
2756	29-May-07	2007	D1	0.26	0.14	0.02	0.06	0.006036	0.8
2771	29-May-07	2007	D1	0.26	0.14	0.02	0.06	0.006036	0.8
2762	29-May-07	2007	D1	0.26	0.14	0.02	0.06	0.006036	0.8
2768	29-May-07	2007	D1	0.26	0.14	0.02	0.06	0.006036	0.8
2754	29-May-07	2007	D1	0.26	0.14	0.02	0.06	0.006036	0.8
2759	29-May-07	2007	D1	0.26	0.14	0.02	0.06	0.006036	0.8
2764	29-May-07	2007	D1	0.26	0.14	0.02	0.06	0.006036	0.8
2767	29-May-07	2007	D1	0.26	0.14	0.02	0.06	0.006036	0.8
2752	29-May-07	2007	D1	0.26	0.14	0.02	0.06	0.006036	0.8
2774	29-May-07	2007	D1	0.26	0.14	0.02	0.06	0.006036	0.8
2750	29-May-07	2007	D1	0.26	0.14	0.02	0.06	0.006036	0.8
2751	29-May-07	2007	D1	0.26	0.14	0.02	0.06	0.006036	0.8
	30-May-07	2007	YY	0.2	0.08	0.06	0.08	0	0.65
	30-May-07	2007	YY	0.2	0.08	0.06	0.08	0	0.65
	30-May-07	2007	YY	0.2	0.08	0.06	0.08	0	0.65
	30-May-07	2007	YY	0.2	0.08	0.06	0.08	0	0.65
	30-May-07	2007	YY	0.2	0.08	0.06	0.08	0	0.65
	30-May-07	2007	YY	0.2	0.08	0.06	0.08	0	0.65
	30-May-07	2007	YY	0.2	0.08	0.06	0.08	0	0.65
	30-May-07	2007	YY	0.2	0.08	0.06	0.08	0	0.65
	30-May-07	2007	YY	0.2	0.08	0.06	0.08	0	0.65
	30-May-07	2007	YY	0.2	0.08	0.06	0.08	0	0.65
	30-May-07	2007	YY	0.2	0.08	0.06	0.08	0	0.65
	30-May-07	2007	YY	0.2	0.08	0.06	0.08	0	0.65
	30-May-07	2007	YY	0.2	0.08	0.06	0.08	0	0.65

	30-May-07	2007	YY	0.2	0.08	0.06	0.08	0	0.65
	30-May-07	2007	YY	0.2	0.08	0.06	0.08	0	0.65
	30-May-07	2007	YY	0.2	0.08	0.06	0.08	0	0.65
	30-May-07	2007	YY	0.2	0.08	0.06	0.08	0	0.65
	30-May-07	2007	YY	0.2	0.08	0.06	0.08	0	0.65
	30-May-07	2007	YY	0.2	0.08	0.06	0.08	0	0.65
2831	30-May-07	2007	MG	0.24	0.28	0.12	0.02	0.009615	6.15
2833	30-May-07	2007	MG	0.24	0.28	0.12	0.02	0.009615	6.15
2829	30-May-07	2007	MG	0.24	0.28	0.12	0.02	0.009615	6.15
2830	30-May-07	2007	MG	0.24	0.28	0.12	0.02	0.009615	6.15
2832	30-May-07	2007	MG	0.24	0.28	0.12	0.02	0.009615	6.15
2814	30-May-07	2007	YY	0.2	0.08	0.06	0.08	0	0.65
2815	30-May-07	2007	YY	0.2	0.08	0.06	0.08	0	0.65
2816	30-May-07	2007	YY	0.2	0.08	0.06	0.08	0	0.65
2813	30-May-07	2007	YY	0.2	0.08	0.06	0.08	0	0.65
2818	30-May-07	2007	YY	0.2	0.08	0.06	0.08	0	0.65
2819	30-May-07	2007	YY	0.2	0.08	0.06	0.08	0	0.65
2822	30-May-07	2007	YY	0.2	0.08	0.06	0.08	0	0.65
2823	30-May-07	2007	YY	0.2	0.08	0.06	0.08	0	0.65
2820	30-May-07	2007	YY	0.2	0.08	0.06	0.08	0	0.65
2825	30-May-07	2007	YY	0.2	0.08	0.06	0.08	0	0.65
2827	30-May-07	2007	YY	0.2	0.08	0.06	0.08	0	0.65
2828	30-May-07	2007	YY	0.2	0.08	0.06	0.08	0	0.65
2817	30-May-07	2007	YY	0.2	0.08	0.06	0.08	0	0.65
2824	30-May-07	2007	YY	0.2	0.08	0.06	0.08	0	0.65
2812	30-May-07	2007	YY	0.2	0.08	0.06	0.08	0	0.65
2821	30-May-07	2007	YY	0.2	0.08	0.06	0.08	0	0.65
2826	30-May-07	2007	YY	0.2	0.08	0.06	0.08	0	0.65
2878	31-May-07	2007	SC	0.136986	0.041096	0.027397	0	0	8.576923
2876	31-May-07	2007	SC	0.136986	0.041096	0.027397	0	0	8.576923
2859	31-May-07	2007	SC	0.136986	0.041096	0.027397	0	0	8.576923
2881	31-May-07	2007	SC	0.136986	0.041096	0.027397	0	0	8.576923
2869	31-May-07	2007	SC	0.136986	0.041096	0.027397	0	0	8.576923
2867	31-May-07	2007	SC	0.136986	0.041096	0.027397	0	0	8.576923
2866	31-May-07	2007	SC	0.136986	0.041096	0.027397	0	0	8.576923
2863	31-May-07	2007	SC	0.136986	0.041096	0.027397	0	0	8.576923
2864	31-May-07	2007	SC	0.136986	0.041096	0.027397	0	0	8.576923
2868	31-May-07	2007	SC	0.136986	0.041096	0.027397	0	0	8.576923
2880	31-May-07	2007	SC	0.136986	0.041096	0.027397	0	0	8.576923
2879	31-May-07	2007	SC	0.136986	0.041096	0.027397	0	0	8.576923
2862	31-May-07	2007	SC	0.136986	0.041096	0.027397	0	0	8.576923
2882	31-May-07	2007	SC	0.136986	0.041096	0.027397	0	0	8.576923
2861	31-May-07	2007	SC	0.136986	0.041096	0.027397	0	0	8.576923
2865	31-May-07	2007	SC	0.136986	0.041096	0.027397	0	0	8.576923

2877	31-May-07	2007	SC	0.136986	0.041096	0.027397	0	0	8.576923
2885	31-May-07	2007	SC	0.136986	0.041096	0.027397	0	0	8.576923
2886	31-May-07	2007	SC	0.136986	0.041096	0.027397	0	0	8.576923
2875	31-May-07	2007	SC	0.136986	0.041096	0.027397	0	0	8.576923
2883	31-May-07	2007	SC	0.136986	0.041096	0.027397	0	0	8.576923
2872	31-May-07	2007	SC	0.136986	0.041096	0.027397	0	0	8.576923
2874	31-May-07	2007	SC	0.136986	0.041096	0.027397	0	0	8.576923
2884	31-May-07	2007	SC	0.136986	0.041096	0.027397	0	0	8.576923
2888	31-May-07	2007	SC	0.136986	0.041096	0.027397	0	0	8.576923
2890	31-May-07	2007	SC	0.136986	0.041096	0.027397	0	0	8.576923
2870	31-May-07	2007	SC	0.136986	0.041096	0.027397	0	0	8.576923
2871	31-May-07	2007	SC	0.136986	0.041096	0.027397	0	0	8.576923
2887	31-May-07	2007	SC	0.136986	0.041096	0.027397	0	0	8.576923
2889	31-May-07	2007	SC	0.136986	0.041096	0.027397	0	0	8.576923
2858	31-May-07	2007	SC	0.136986	0.041096	0.027397	0	0	8.576923
2860	31-May-07	2007	SC	0.136986	0.041096	0.027397	0	0	8.576923
2873	31-May-07	2007	SC	0.136986	0.041096	0.027397	0	0	8.576923
	31-May-07	2007	SC	0.136986	0.041096	0.027397	0	0	8.576923
	31-May-07	2007	SC	0.136986	0.041096	0.027397	0	0	8.576923
	31-May-07	2007	SC	0.136986	0.041096	0.027397	0	0	8.576923
	31-May-07	2007	SC	0.136986	0.041096	0.027397	0	0	8.576923
	31-May-07	2007	SC	0.136986	0.041096	0.027397	0	0	8.576923
	31-May-07	2007	SC	0.136986	0.041096	0.027397	0	0	8.576923
	31-May-07	2007	SC	0.136986	0.041096	0.027397	0	0	8.576923
	31-May-07	2007	SC	0.136986	0.041096	0.027397	0	0	8.576923
	31-May-07	2007	SC	0.136986	0.041096	0.027397	0	0	8.576923
	31-May-07	2007	SC	0.136986	0.041096	0.027397	0	0	8.576923
	31-May-07	2007	SC	0.136986	0.041096	0.027397	0	0	8.576923
	31-May-07	2007	SC	0.136986	0.041096	0.027397	0	0	8.576923
	31-May-07	2007	SC	0.136986	0.041096	0.027397	0	0	8.576923
	31-May-07	2007	SC	0.136986	0.041096	0.027397	0	0	8.576923
	31-May-07	2007	SC	0.136986	0.041096	0.027397	0	0	8.576923
	31-May-07	2007	SC	0.136986	0.041096	0.027397	0	0	8.576923
	31-May-07	2007	SC	0.136986	0.041096	0.027397	0	0	8.576923
2933	31-May-07	2007	SC	0.136986	0.041096	0.027397	0	0	8.576923
2934	31-May-07	2007	SC	0.136986	0.041096	0.027397	0	0	8.576923
2935	31-May-07	2007	SC	0.136986	0.041096	0.027397	0	0	8.576923
2955	1-Jun-07	2007	RL	0.157895	0.157895	0	0	0.028571	0.333333
2956	1-Jun-07	2007	RL	0.157895	0.157895	0	0	0.028571	0.333333
2957	1-Jun-07	2007	RL	0.157895	0.157895	0	0	0.028571	0.333333
2952	1-Jun-07	2007	RL	0.157895	0.157895	0	0	0.028571	0.333333
2953	1-Jun-07	2007	RL	0.157895	0.157895	0	0	0.028571	0.333333
2954	1-Jun-07	2007	RL	0.157895	0.157895	0	0	0.028571	0.333333
3012	2-Jun-07	2007	LT1	0.521739	0	0.130435	0	0	3.555556

3013	2-Jun-07	2007 LT1	0.521739	0	0.130435	0	0	3.555556
3014	2-Jun-07	2007 LT1	0.521739	0	0.130435	0	0	3.555556
3011	2-Jun-07	2007 LT1	0.521739	0	0.130435	0	0	3.555556
3010	2-Jun-07	2007 LT1	0.521739	0	0.130435	0	0	3.555556
3006	2-Jun-07	2007 LT1	0.521739	0	0.130435	0	0	3.555556
3015	2-Jun-07	2007 LT1	0.521739	0	0.130435	0	0	3.555556
3016	2-Jun-07	2007 LT1	0.521739	0	0.130435	0	0	3.555556
3007	2-Jun-07	2007 LT1	0.521739	0	0.130435	0	0	3.555556
3008	2-Jun-07	2007 LT1	0.521739	0	0.130435	0	0	3.555556
3009	2-Jun-07	2007 LT1	0.521739	0	0.130435	0	0	3.555556
3004	2-Jun-07	2007 LT1	0.521739	0	0.130435	0	0	3.555556
3003	2-Jun-07	2007 LT1	0.521739	0	0.130435	0	0	3.555556
3005	2-Jun-07	2007 LT1	0.521739	0	0.130435	0	0	3.555556
3017	2-Jun-07	2007 LT1	0.521739	0	0.130435	0	0	3.555556
	8-Jun-07	2007 ML						
	8-Jun-07	2007 ML						
	8-Jun-07	2007 ML						
	8-Jun-07	2007 ML						
	8-Jun-07	2007 ML						
	8-Jun-07	2007 ML						
	8-Jun-07	2007 ML						
	8-Jun-07	2007 ML						
	8-Jun-07	2007 SD						
	8-Jun-07	2007 SD						
	8-Jun-07	2007 SD						
	8-Jun-07	2007 SD						
	8-Jun-07	2007 SD						
	8-Jun-07	2007 SD						
	8-Jun-07	2007 SD						
	8-Jun-07	2007 SD						
	8-Jun-07	2007 SD						
	8-Jun-07	2007 SD						
	8-Jun-07	2007 SD						
	8-Jun-07	2007 SD						
	8-Jun-07	2007 SD						
	8-Jun-07	2007 SD						
	8-Jun-07	2007 SD						
	8-Jun-07	2007 SD						
	8-Jun-07	2007 SD						
	8-Jun-07	2007 SD						
	8-Jun-07	2007 SD						
	8-Jun-07	2007 SD						
	8-Jun-07	2007 SD						

	8-Jun-07	2007	SD						
	8-Jun-07	2007	SD						
	8-Jun-07	2007	SD						
	8-Jun-07	2007	SD						
	9-Jun-07	2007	RC	0.25	0	0.05	0.05	0.018182	2.875
	9-Jun-07	2007	RC	0.25	0	0.05	0.05	0.018182	2.875
	9-Jun-07	2007	RC	0.25	0	0.05	0.05	0.018182	2.875
	9-Jun-07	2007	RC	0.25	0	0.05	0.05	0.018182	2.875
	9-Jun-07	2007	RC	0.25	0	0.05	0.05	0.018182	2.875
	9-Jun-07	2007	RC	0.25	0	0.05	0.05	0.018182	2.875
	12-Jun-07	2007	SD						
	12-Jun-07	2007	SD						
	12-Jun-07	2007	SD						
	12-Jun-07	2007	SD						
	12-Jun-07	2007	SD						
	12-Jun-07	2007	SD						
	13-Jun-07	2007	RP	0.133333	0.033333	0.133333	0	0.019139	2.75
	13-Jun-07	2007	RP	0.133333	0.033333	0.133333	0	0.019139	2.75
	13-Jun-07	2007	RP	0.133333	0.033333	0.133333	0	0.019139	2.75
	15-Jun-07	2007	SC	0.136986	0.041096	0.027397	0	0	8.576923
	15-Jun-07	2007	SC	0.136986	0.041096	0.027397	0	0	8.576923
	15-Jun-07	2007	SC	0.136986	0.041096	0.027397	0	0	8.576923
	15-Jun-07	2007	SC	0.136986	0.041096	0.027397	0	0	8.576923
	15-Jun-07	2007	SC	0.136986	0.041096	0.027397	0	0	8.576923
	15-Jun-07	2007	SC	0.136986	0.041096	0.027397	0	0	8.576923
	15-Jun-07	2007	SC	0.136986	0.041096	0.027397	0	0	8.576923
	15-Jun-07	2007	SC	0.136986	0.041096	0.027397	0	0	8.576923
	15-Jun-07	2007	SC	0.136986	0.041096	0.027397	0	0	8.576923
	15-Jun-07	2007	SC	0.136986	0.041096	0.027397	0	0	8.576923
46	12-May-08	2008	LT1	0.925926	0.222222	0.037037	0.04	0	3.666667
52	12-May-08	2008	LT1	0.925926	0.222222	0.037037	0.04	0	3.666667
51	12-May-08	2008	LT1	0.925926	0.222222	0.037037	0.04	0	3.666667
53	12-May-08	2008	LT1	0.925926	0.222222	0.037037	0.04	0	3.666667
50	12-May-08	2008	LT1	0.925926	0.222222	0.037037	0.04	0	3.666667
55	12-May-08	2008	LT1	0.925926	0.222222	0.037037	0.04	0	3.666667
54	12-May-08	2008	LT1	0.925926	0.222222	0.037037	0.04	0	3.666667
56	12-May-08	2008	LT1	0.925926	0.222222	0.037037	0.04	0	3.666667
43	12-May-08	2008	LT1	0.925926	0.222222	0.037037	0.04	0	3.666667
49	12-May-08	2008	LT1	0.925926	0.222222	0.037037	0.04	0	3.666667
45	12-May-08	2008	LT1	0.925926	0.222222	0.037037	0.04	0	3.666667
59	12-May-08	2008	LT1	0.925926	0.222222	0.037037	0.04	0	3.666667
47	12-May-08	2008	LT1	0.925926	0.222222	0.037037	0.04	0	3.666667
44	12-May-08	2008	LT1	0.925926	0.222222	0.037037	0.04	0	3.666667
48	12-May-08	2008	LT1	0.925926	0.222222	0.037037	0.04	0	3.666667

57	12-May-08	2008	LT1	0.925926	0.222222	0.037037	0.04	0	3.666667
60	12-May-08	2008	LT1	0.925926	0.222222	0.037037	0.04	0	3.666667
58	12-May-08	2008	LT1	0.925926	0.222222	0.037037	0.04	0	3.666667
42	12-May-08	2008	LT1	0.925926	0.222222	0.037037	0.04	0	3.666667
96	15-May-08	2008	RP	0.176471	0	0.029412	0.029412	0.015228	3.083333
104	15-May-08	2008	RP	0.176471	0	0.029412	0.029412	0.015228	3.083333
103	15-May-08	2008	RP	0.176471	0	0.029412	0.029412	0.015228	3.083333
99	15-May-08	2008	RP	0.176471	0	0.029412	0.029412	0.015228	3.083333
100	15-May-08	2008	RP	0.176471	0	0.029412	0.029412	0.015228	3.083333
97	15-May-08	2008	RP	0.176471	0	0.029412	0.029412	0.015228	3.083333
95	15-May-08	2008	RP	0.176471	0	0.029412	0.029412	0.015228	3.083333
101	15-May-08	2008	RP	0.176471	0	0.029412	0.029412	0.015228	3.083333
102	15-May-08	2008	RP	0.176471	0	0.029412	0.029412	0.015228	3.083333
105	15-May-08	2008	RP	0.176471	0	0.029412	0.029412	0.015228	3.083333
98	15-May-08	2008	RP	0.176471	0	0.029412	0.029412	0.015228	3.083333
94	15-May-08	2008	RP	0.176471	0	0.029412	0.029412	0.015228	3.083333
128	16-May-08	2008	TO	0.2	0.2	0.05	0.025	0.00289	0.875
134	16-May-08	2008	TO	0.2	0.2	0.05	0.025	0.00289	0.875
126	16-May-08	2008	TO	0.2	0.2	0.05	0.025	0.00289	0.875
124	16-May-08	2008	TO	0.2	0.2	0.05	0.025	0.00289	0.875
150	16-May-08	2008	TO	0.2	0.2	0.05	0.025	0.00289	0.875
141	16-May-08	2008	TO	0.2	0.2	0.05	0.025	0.00289	0.875
143	16-May-08	2008	TO	0.2	0.2	0.05	0.025	0.00289	0.875
146	16-May-08	2008	TO	0.2	0.2	0.05	0.025	0.00289	0.875
151	16-May-08	2008	TO	0.2	0.2	0.05	0.025	0.00289	0.875
149	16-May-08	2008	TO	0.2	0.2	0.05	0.025	0.00289	0.875
144	16-May-08	2008	TO	0.2	0.2	0.05	0.025	0.00289	0.875
147	16-May-08	2008	TO	0.2	0.2	0.05	0.025	0.00289	0.875
152	16-May-08	2008	TO	0.2	0.2	0.05	0.025	0.00289	0.875
136	16-May-08	2008	TO	0.2	0.2	0.05	0.025	0.00289	0.875
139	16-May-08	2008	TO	0.2	0.2	0.05	0.025	0.00289	0.875
131	16-May-08	2008	TO	0.2	0.2	0.05	0.025	0.00289	0.875
130	16-May-08	2008	TO	0.2	0.2	0.05	0.025	0.00289	0.875
127	16-May-08	2008	TO	0.2	0.2	0.05	0.025	0.00289	0.875
129	16-May-08	2008	TO	0.2	0.2	0.05	0.025	0.00289	0.875
132	16-May-08	2008	TO	0.2	0.2	0.05	0.025	0.00289	0.875
133	16-May-08	2008	TO	0.2	0.2	0.05	0.025	0.00289	0.875
135	16-May-08	2008	TO	0.2	0.2	0.05	0.025	0.00289	0.875
148	16-May-08	2008	TO	0.2	0.2	0.05	0.025	0.00289	0.875
142	16-May-08	2008	TO	0.2	0.2	0.05	0.025	0.00289	0.875
137	16-May-08	2008	TO	0.2	0.2	0.05	0.025	0.00289	0.875
145	16-May-08	2008	TO	0.2	0.2	0.05	0.025	0.00289	0.875
138	16-May-08	2008	TO	0.2	0.2	0.05	0.025	0.00289	0.875
123	16-May-08	2008	TO	0.2	0.2	0.05	0.025	0.00289	0.875

125	16-May-08	2008	TO	0.2	0.2	0.05	0.025	0.00289	0.875
140	16-May-08	2008	TO	0.2	0.2	0.05	0.025	0.00289	0.875
153	16-May-08	2008	TO	0.2	0.2	0.05	0.025	0.00289	0.875
122	16-May-08	2008	TO	0.2	0.2	0.05	0.025	0.00289	0.875
190	16-May-08	2008	TO	0.2	0.2	0.05	0.025	0.00289	0.875
176	16-May-08	2008	TO	0.2	0.2	0.05	0.025	0.00289	0.875
188	16-May-08	2008	TO	0.2	0.2	0.05	0.025	0.00289	0.875
189	16-May-08	2008	TO	0.2	0.2	0.05	0.025	0.00289	0.875
184	16-May-08	2008	TO	0.2	0.2	0.05	0.025	0.00289	0.875
180	16-May-08	2008	TO	0.2	0.2	0.05	0.025	0.00289	0.875
181	16-May-08	2008	TO	0.2	0.2	0.05	0.025	0.00289	0.875
185	16-May-08	2008	TO	0.2	0.2	0.05	0.025	0.00289	0.875
179	16-May-08	2008	TO	0.2	0.2	0.05	0.025	0.00289	0.875
187	16-May-08	2008	TO	0.2	0.2	0.05	0.025	0.00289	0.875
191	16-May-08	2008	TO	0.2	0.2	0.05	0.025	0.00289	0.875
182	16-May-08	2008	TO	0.2	0.2	0.05	0.025	0.00289	0.875
183	16-May-08	2008	TO	0.2	0.2	0.05	0.025	0.00289	0.875
192	16-May-08	2008	TO	0.2	0.2	0.05	0.025	0.00289	0.875
170	16-May-08	2008	TO	0.2	0.2	0.05	0.025	0.00289	0.875
173	16-May-08	2008	TO	0.2	0.2	0.05	0.025	0.00289	0.875
174	16-May-08	2008	TO	0.2	0.2	0.05	0.025	0.00289	0.875
177	16-May-08	2008	TO	0.2	0.2	0.05	0.025	0.00289	0.875
171	16-May-08	2008	TO	0.2	0.2	0.05	0.025	0.00289	0.875
172	16-May-08	2008	TO	0.2	0.2	0.05	0.025	0.00289	0.875
169	16-May-08	2008	TO	0.2	0.2	0.05	0.025	0.00289	0.875
175	16-May-08	2008	TO	0.2	0.2	0.05	0.025	0.00289	0.875
168	16-May-08	2008	TO	0.2	0.2	0.05	0.025	0.00289	0.875
178	16-May-08	2008	TO	0.2	0.2	0.05	0.025	0.00289	0.875
186	16-May-08	2008	TO	0.2	0.2	0.05	0.025	0.00289	0.875
193	16-May-08	2008	TO	0.2	0.2	0.05	0.025	0.00289	0.875
167	16-May-08	2008	TO	0.2	0.2	0.05	0.025	0.00289	0.875
224	18-May-08	2008	SW	0.368421	0.157895	0.052632	0	0	1.466667
199	18-May-08	2008	SW	0.368421	0.157895	0.052632	0	0	1.466667
201	18-May-08	2008	SW	0.368421	0.157895	0.052632	0	0	1.466667
230	18-May-08	2008	SW	0.368421	0.157895	0.052632	0	0	1.466667
198	18-May-08	2008	SW	0.368421	0.157895	0.052632	0	0	1.466667
196	18-May-08	2008	SW	0.368421	0.157895	0.052632	0	0	1.466667
197	18-May-08	2008	SW	0.368421	0.157895	0.052632	0	0	1.466667
202	18-May-08	2008	SW	0.368421	0.157895	0.052632	0	0	1.466667
203	18-May-08	2008	SW	0.368421	0.157895	0.052632	0	0	1.466667
212	18-May-08	2008	SW	0.368421	0.157895	0.052632	0	0	1.466667
200	18-May-08	2008	SW	0.368421	0.157895	0.052632	0	0	1.466667
206	18-May-08	2008	SW	0.368421	0.157895	0.052632	0	0	1.466667
226	18-May-08	2008	SW	0.368421	0.157895	0.052632	0	0	1.466667

227	18-May-08	2008	SW	0.368421	0.157895	0.052632	0	0	1.466667
217	18-May-08	2008	SW	0.368421	0.157895	0.052632	0	0	1.466667
209	18-May-08	2008	SW	0.368421	0.157895	0.052632	0	0	1.466667
215	18-May-08	2008	SW	0.368421	0.157895	0.052632	0	0	1.466667
216	18-May-08	2008	SW	0.368421	0.157895	0.052632	0	0	1.466667
218	18-May-08	2008	SW	0.368421	0.157895	0.052632	0	0	1.466667
220	18-May-08	2008	SW	0.368421	0.157895	0.052632	0	0	1.466667
208	18-May-08	2008	SW	0.368421	0.157895	0.052632	0	0	1.466667
221	18-May-08	2008	SW	0.368421	0.157895	0.052632	0	0	1.466667
213	18-May-08	2008	SW	0.368421	0.157895	0.052632	0	0	1.466667
210	18-May-08	2008	SW	0.368421	0.157895	0.052632	0	0	1.466667
211	18-May-08	2008	SW	0.368421	0.157895	0.052632	0	0	1.466667
214	18-May-08	2008	SW	0.368421	0.157895	0.052632	0	0	1.466667
222	18-May-08	2008	SW	0.368421	0.157895	0.052632	0	0	1.466667
207	18-May-08	2008	SW	0.368421	0.157895	0.052632	0	0	1.466667
225	18-May-08	2008	SW	0.368421	0.157895	0.052632	0	0	1.466667
223	18-May-08	2008	SW	0.368421	0.157895	0.052632	0	0	1.466667
204	18-May-08	2008	SW	0.368421	0.157895	0.052632	0	0	1.466667
228	18-May-08	2008	SW	0.368421	0.157895	0.052632	0	0	1.466667
229	18-May-08	2008	SW	0.368421	0.157895	0.052632	0	0	1.466667
195	18-May-08	2008	SW	0.368421	0.157895	0.052632	0	0	1.466667
205	18-May-08	2008	SW	0.368421	0.157895	0.052632	0	0	1.466667
219	18-May-08	2008	SW	0.368421	0.157895	0.052632	0	0	1.466667
231	18-May-08	2008	SW	0.368421	0.157895	0.052632	0	0	1.466667
194	18-May-08	2008	SW	0.368421	0.157895	0.052632	0	0	1.466667
348	19-May-08	2008	ML						
349	19-May-08	2008	ML						
352	19-May-08	2008	ML						
353	19-May-08	2008	ML						
342	19-May-08	2008	ML						
343	19-May-08	2008	ML						
341	19-May-08	2008	ML						
340	19-May-08	2008	ML						
345	19-May-08	2008	ML						
346	19-May-08	2008	ML						
351	19-May-08	2008	ML						
354	19-May-08	2008	ML						
347	19-May-08	2008	ML						
355	19-May-08	2008	ML						
339	19-May-08	2008	ML						
344	19-May-08	2008	ML						
350	19-May-08	2008	ML						
356	19-May-08	2008	ML						
338	19-May-08	2008	ML						

370	21-May-08	2008	AFC
374	21-May-08	2008	AFC
362	21-May-08	2008	AFC
361	21-May-08	2008	AFC
363	21-May-08	2008	AFC
375	21-May-08	2008	AFC
360	21-May-08	2008	AFC
373	21-May-08	2008	AFC
359	21-May-08	2008	AFC
371	21-May-08	2008	AFC
367	21-May-08	2008	AFC
372	21-May-08	2008	AFC
366	21-May-08	2008	AFC
365	21-May-08	2008	AFC
368	21-May-08	2008	AFC
358	21-May-08	2008	AFC
364	21-May-08	2008	AFC
369	21-May-08	2008	AFC
376	21-May-08	2008	AFC
357	21-May-08	2008	AFC
440	21-May-08	2008	AFF
435	21-May-08	2008	AFF
429	21-May-08	2008	AFF
425	21-May-08	2008	AFF
427	21-May-08	2008	AFF
422	21-May-08	2008	AFF
423	21-May-08	2008	AFF
437	21-May-08	2008	AFF
421	21-May-08	2008	AFF
438	21-May-08	2008	AFF
431	21-May-08	2008	AFF
420	21-May-08	2008	AFF
418	21-May-08	2008	AFF
439	21-May-08	2008	AFF
432	21-May-08	2008	AFF
417	21-May-08	2008	AFF
424	21-May-08	2008	AFF
433	21-May-08	2008	AFF
414	21-May-08	2008	AFF
416	21-May-08	2008	AFF
419	21-May-08	2008	AFF
436	21-May-08	2008	AFF
412	21-May-08	2008	AFF
411	21-May-08	2008	AFF

413	21-May-08	2008	AFF						
426	21-May-08	2008	AFF						
430	21-May-08	2008	AFF						
434	21-May-08	2008	AFF						
410	21-May-08	2008	AFF						
409	21-May-08	2008	AFF						
415	21-May-08	2008	AFF						
428	21-May-08	2008	AFF						
441	21-May-08	2008	AFF						
408	21-May-08	2008	AFF						
477	22-May-08	2008	7M	0.078947	0.342105	0.078947	0.263158	0.01023	7.733333
476	22-May-08	2008	7M	0.078947	0.342105	0.078947	0.263158	0.01023	7.733333
479	22-May-08	2008	7M	0.078947	0.342105	0.078947	0.263158	0.01023	7.733333
467	22-May-08	2008	7M	0.078947	0.342105	0.078947	0.263158	0.01023	7.733333
468	22-May-08	2008	7M	0.078947	0.342105	0.078947	0.263158	0.01023	7.733333
470	22-May-08	2008	7M	0.078947	0.342105	0.078947	0.263158	0.01023	7.733333
472	22-May-08	2008	7M	0.078947	0.342105	0.078947	0.263158	0.01023	7.733333
475	22-May-08	2008	7M	0.078947	0.342105	0.078947	0.263158	0.01023	7.733333
471	22-May-08	2008	7M	0.078947	0.342105	0.078947	0.263158	0.01023	7.733333
462	22-May-08	2008	7M	0.078947	0.342105	0.078947	0.263158	0.01023	7.733333
466	22-May-08	2008	7M	0.078947	0.342105	0.078947	0.263158	0.01023	7.733333
461	22-May-08	2008	7M	0.078947	0.342105	0.078947	0.263158	0.01023	7.733333
478	22-May-08	2008	7M	0.078947	0.342105	0.078947	0.263158	0.01023	7.733333
463	22-May-08	2008	7M	0.078947	0.342105	0.078947	0.263158	0.01023	7.733333
464	22-May-08	2008	7M	0.078947	0.342105	0.078947	0.263158	0.01023	7.733333
469	22-May-08	2008	7M	0.078947	0.342105	0.078947	0.263158	0.01023	7.733333
473	22-May-08	2008	7M	0.078947	0.342105	0.078947	0.263158	0.01023	7.733333
480	22-May-08	2008	7M	0.078947	0.342105	0.078947	0.263158	0.01023	7.733333
481	22-May-08	2008	7M	0.078947	0.342105	0.078947	0.263158	0.01023	7.733333
482	22-May-08	2008	7M	0.078947	0.342105	0.078947	0.263158	0.01023	7.733333
460	22-May-08	2008	7M	0.078947	0.342105	0.078947	0.263158	0.01023	7.733333
465	22-May-08	2008	7M	0.078947	0.342105	0.078947	0.263158	0.01023	7.733333
474	22-May-08	2008	7M	0.078947	0.342105	0.078947	0.263158	0.01023	7.733333
483	22-May-08	2008	7M	0.078947	0.342105	0.078947	0.263158	0.01023	7.733333
459	22-May-08	2008	7M	0.078947	0.342105	0.078947	0.263158	0.01023	7.733333
546	22-May-08	2008	7M	0.078947	0.342105	0.078947	0.263158	0.01023	7.733333
533	22-May-08	2008	7M	0.078947	0.342105	0.078947	0.263158	0.01023	7.733333
537	22-May-08	2008	7M	0.078947	0.342105	0.078947	0.263158	0.01023	7.733333
534	22-May-08	2008	7M	0.078947	0.342105	0.078947	0.263158	0.01023	7.733333
535	22-May-08	2008	7M	0.078947	0.342105	0.078947	0.263158	0.01023	7.733333
538	22-May-08	2008	7M	0.078947	0.342105	0.078947	0.263158	0.01023	7.733333
541	22-May-08	2008	7M	0.078947	0.342105	0.078947	0.263158	0.01023	7.733333
536	22-May-08	2008	7M	0.078947	0.342105	0.078947	0.263158	0.01023	7.733333
540	22-May-08	2008	7M	0.078947	0.342105	0.078947	0.263158	0.01023	7.733333

543	22-May-08	2008	7M	0.078947	0.342105	0.078947	0.263158	0.01023	7.733333
539	22-May-08	2008	7M	0.078947	0.342105	0.078947	0.263158	0.01023	7.733333
544	22-May-08	2008	7M	0.078947	0.342105	0.078947	0.263158	0.01023	7.733333
545	22-May-08	2008	7M	0.078947	0.342105	0.078947	0.263158	0.01023	7.733333
532	22-May-08	2008	7M	0.078947	0.342105	0.078947	0.263158	0.01023	7.733333
542	22-May-08	2008	7M	0.078947	0.342105	0.078947	0.263158	0.01023	7.733333
547	22-May-08	2008	7M	0.078947	0.342105	0.078947	0.263158	0.01023	7.733333
548	22-May-08	2008	7M	0.078947	0.342105	0.078947	0.263158	0.01023	7.733333
531	22-May-08	2008	7M	0.078947	0.342105	0.078947	0.263158	0.01023	7.733333
572	23-May-08	2008	RL	0.210526	0.131579	0.026316	0.078947	0.018018	0.333333
571	23-May-08	2008	RL	0.210526	0.131579	0.026316	0.078947	0.018018	0.333333
573	23-May-08	2008	RL	0.210526	0.131579	0.026316	0.078947	0.018018	0.333333
579	23-May-08	2008	RL	0.210526	0.131579	0.026316	0.078947	0.018018	0.333333
577	23-May-08	2008	RL	0.210526	0.131579	0.026316	0.078947	0.018018	0.333333
574	23-May-08	2008	RL	0.210526	0.131579	0.026316	0.078947	0.018018	0.333333
575	23-May-08	2008	RL	0.210526	0.131579	0.026316	0.078947	0.018018	0.333333
576	23-May-08	2008	RL	0.210526	0.131579	0.026316	0.078947	0.018018	0.333333
569	23-May-08	2008	RL	0.210526	0.131579	0.026316	0.078947	0.018018	0.333333
585	23-May-08	2008	RL	0.210526	0.131579	0.026316	0.078947	0.018018	0.333333
580	23-May-08	2008	RL	0.210526	0.131579	0.026316	0.078947	0.018018	0.333333
581	23-May-08	2008	RL	0.210526	0.131579	0.026316	0.078947	0.018018	0.333333
583	23-May-08	2008	RL	0.210526	0.131579	0.026316	0.078947	0.018018	0.333333
584	23-May-08	2008	RL	0.210526	0.131579	0.026316	0.078947	0.018018	0.333333
570	23-May-08	2008	RL	0.210526	0.131579	0.026316	0.078947	0.018018	0.333333
578	23-May-08	2008	RL	0.210526	0.131579	0.026316	0.078947	0.018018	0.333333
582	23-May-08	2008	RL	0.210526	0.131579	0.026316	0.078947	0.018018	0.333333
586	23-May-08	2008	RL	0.210526	0.131579	0.026316	0.078947	0.018018	0.333333
568	23-May-08	2008	RL	0.210526	0.131579	0.026316	0.078947	0.018018	0.333333
619	23-May-08	2008	RL	0.210526	0.131579	0.026316	0.078947	0.018018	0.333333
607	23-May-08	2008	RL	0.210526	0.131579	0.026316	0.078947	0.018018	0.333333
611	23-May-08	2008	RL	0.210526	0.131579	0.026316	0.078947	0.018018	0.333333
612	23-May-08	2008	RL	0.210526	0.131579	0.026316	0.078947	0.018018	0.333333
610	23-May-08	2008	RL	0.210526	0.131579	0.026316	0.078947	0.018018	0.333333
614	23-May-08	2008	RL	0.210526	0.131579	0.026316	0.078947	0.018018	0.333333
609	23-May-08	2008	RL	0.210526	0.131579	0.026316	0.078947	0.018018	0.333333
620	23-May-08	2008	RL	0.210526	0.131579	0.026316	0.078947	0.018018	0.333333
613	23-May-08	2008	RL	0.210526	0.131579	0.026316	0.078947	0.018018	0.333333
616	23-May-08	2008	RL	0.210526	0.131579	0.026316	0.078947	0.018018	0.333333
617	23-May-08	2008	RL	0.210526	0.131579	0.026316	0.078947	0.018018	0.333333
608	23-May-08	2008	RL	0.210526	0.131579	0.026316	0.078947	0.018018	0.333333
615	23-May-08	2008	RL	0.210526	0.131579	0.026316	0.078947	0.018018	0.333333
618	23-May-08	2008	RL	0.210526	0.131579	0.026316	0.078947	0.018018	0.333333
621	23-May-08	2008	RL	0.210526	0.131579	0.026316	0.078947	0.018018	0.333333
606	23-May-08	2008	RL	0.210526	0.131579	0.026316	0.078947	0.018018	0.333333

672	24-May-08	2008 SC	0.191781	0.013699	0.123288	0	0	8.615385
683	24-May-08	2008 SC	0.191781	0.013699	0.123288	0	0	8.615385
670	24-May-08	2008 SC	0.191781	0.013699	0.123288	0	0	8.615385
671	24-May-08	2008 SC	0.191781	0.013699	0.123288	0	0	8.615385
673	24-May-08	2008 SC	0.191781	0.013699	0.123288	0	0	8.615385
678	24-May-08	2008 SC	0.191781	0.013699	0.123288	0	0	8.615385
674	24-May-08	2008 SC	0.191781	0.013699	0.123288	0	0	8.615385
676	24-May-08	2008 SC	0.191781	0.013699	0.123288	0	0	8.615385
684	24-May-08	2008 SC	0.191781	0.013699	0.123288	0	0	8.615385
685	24-May-08	2008 SC	0.191781	0.013699	0.123288	0	0	8.615385
682	24-May-08	2008 SC	0.191781	0.013699	0.123288	0	0	8.615385
686	24-May-08	2008 SC	0.191781	0.013699	0.123288	0	0	8.615385
681	24-May-08	2008 SC	0.191781	0.013699	0.123288	0	0	8.615385
690	24-May-08	2008 SC	0.191781	0.013699	0.123288	0	0	8.615385
687	24-May-08	2008 SC	0.191781	0.013699	0.123288	0	0	8.615385
680	24-May-08	2008 SC	0.191781	0.013699	0.123288	0	0	8.615385
692	24-May-08	2008 SC	0.191781	0.013699	0.123288	0	0	8.615385
688	24-May-08	2008 SC	0.191781	0.013699	0.123288	0	0	8.615385
695	24-May-08	2008 SC	0.191781	0.013699	0.123288	0	0	8.615385
696	24-May-08	2008 SC	0.191781	0.013699	0.123288	0	0	8.615385
677	24-May-08	2008 SC	0.191781	0.013699	0.123288	0	0	8.615385
675	24-May-08	2008 SC	0.191781	0.013699	0.123288	0	0	8.615385
669	24-May-08	2008 SC	0.191781	0.013699	0.123288	0	0	8.615385
679	24-May-08	2008 SC	0.191781	0.013699	0.123288	0	0	8.615385
694	24-May-08	2008 SC	0.191781	0.013699	0.123288	0	0	8.615385
697	24-May-08	2008 SC	0.191781	0.013699	0.123288	0	0	8.615385
691	24-May-08	2008 SC	0.191781	0.013699	0.123288	0	0	8.615385
689	24-May-08	2008 SC	0.191781	0.013699	0.123288	0	0	8.615385
693	24-May-08	2008 SC	0.191781	0.013699	0.123288	0	0	8.615385
668	24-May-08	2008 SC	0.191781	0.013699	0.123288	0	0	8.615385
747	24-May-08	2008 SC	0.191781	0.013699	0.123288	0	0	8.615385
748	24-May-08	2008 SC	0.191781	0.013699	0.123288	0	0	8.615385
749	24-May-08	2008 SC	0.191781	0.013699	0.123288	0	0	8.615385
750	24-May-08	2008 SC	0.191781	0.013699	0.123288	0	0	8.615385
751	24-May-08	2008 SC	0.191781	0.013699	0.123288	0	0	8.615385
752	24-May-08	2008 SC	0.191781	0.013699	0.123288	0	0	8.615385
753	24-May-08	2008 SC	0.191781	0.013699	0.123288	0	0	8.615385
756	24-May-08	2008 SC	0.191781	0.013699	0.123288	0	0	8.615385
758	24-May-08	2008 SC	0.191781	0.013699	0.123288	0	0	8.615385
759	24-May-08	2008 SC	0.191781	0.013699	0.123288	0	0	8.615385
745	24-May-08	2008 SC	0.191781	0.013699	0.123288	0	0	8.615385
746	24-May-08	2008 SC	0.191781	0.013699	0.123288	0	0	8.615385
754	24-May-08	2008 SC	0.191781	0.013699	0.123288	0	0	8.615385
760	24-May-08	2008 SC	0.191781	0.013699	0.123288	0	0	8.615385

755	24-May-08	2008	SC	0.191781	0.013699	0.123288	0	0	8.615385
757	24-May-08	2008	SC	0.191781	0.013699	0.123288	0	0	8.615385
742	24-May-08	2008	SC	0.191781	0.013699	0.123288	0	0	8.615385
744	24-May-08	2008	SC	0.191781	0.013699	0.123288	0	0	8.615385
762	24-May-08	2008	SC	0.191781	0.013699	0.123288	0	0	8.615385
743	24-May-08	2008	SC	0.191781	0.013699	0.123288	0	0	8.615385
738	24-May-08	2008	SC	0.191781	0.013699	0.123288	0	0	8.615385
735	24-May-08	2008	SC	0.191781	0.013699	0.123288	0	0	8.615385
736	24-May-08	2008	SC	0.191781	0.013699	0.123288	0	0	8.615385
734	24-May-08	2008	SC	0.191781	0.013699	0.123288	0	0	8.615385
733	24-May-08	2008	SC	0.191781	0.013699	0.123288	0	0	8.615385
739	24-May-08	2008	SC	0.191781	0.013699	0.123288	0	0	8.615385
732	24-May-08	2008	SC	0.191781	0.013699	0.123288	0	0	8.615385
740	24-May-08	2008	SC	0.191781	0.013699	0.123288	0	0	8.615385
741	24-May-08	2008	SC	0.191781	0.013699	0.123288	0	0	8.615385
731	24-May-08	2008	SC	0.191781	0.013699	0.123288	0	0	8.615385
737	24-May-08	2008	SC	0.191781	0.013699	0.123288	0	0	8.615385
761	24-May-08	2008	SC	0.191781	0.013699	0.123288	0	0	8.615385
763	24-May-08	2008	SC	0.191781	0.013699	0.123288	0	0	8.615385
730	24-May-08	2008	SC	0.191781	0.013699	0.123288	0	0	8.615385
791	25-May-08	2008	YY	0.14	0.24	0.08	0.02	0	0.65
784	25-May-08	2008	YY	0.14	0.24	0.08	0.02	0	0.65
785	25-May-08	2008	YY	0.14	0.24	0.08	0.02	0	0.65
786	25-May-08	2008	YY	0.14	0.24	0.08	0.02	0	0.65
792	25-May-08	2008	YY	0.14	0.24	0.08	0.02	0	0.65
793	25-May-08	2008	YY	0.14	0.24	0.08	0.02	0	0.65
783	25-May-08	2008	YY	0.14	0.24	0.08	0.02	0	0.65
787	25-May-08	2008	YY	0.14	0.24	0.08	0.02	0	0.65
790	25-May-08	2008	YY	0.14	0.24	0.08	0.02	0	0.65
794	25-May-08	2008	YY	0.14	0.24	0.08	0.02	0	0.65
795	25-May-08	2008	YY	0.14	0.24	0.08	0.02	0	0.65
782	25-May-08	2008	YY	0.14	0.24	0.08	0.02	0	0.65
788	25-May-08	2008	YY	0.14	0.24	0.08	0.02	0	0.65
797	25-May-08	2008	YY	0.14	0.24	0.08	0.02	0	0.65
798	25-May-08	2008	YY	0.14	0.24	0.08	0.02	0	0.65
799	25-May-08	2008	YY	0.14	0.24	0.08	0.02	0	0.65
800	25-May-08	2008	YY	0.14	0.24	0.08	0.02	0	0.65
781	25-May-08	2008	YY	0.14	0.24	0.08	0.02	0	0.65
789	25-May-08	2008	YY	0.14	0.24	0.08	0.02	0	0.65
796	25-May-08	2008	YY	0.14	0.24	0.08	0.02	0	0.65
801	25-May-08	2008	YY	0.14	0.24	0.08	0.02	0	0.65
780	25-May-08	2008	YY	0.14	0.24	0.08	0.02	0	0.65
836	27-May-08	2008	RC	0.15	0	0.05	0	0.009346	2.875
837	27-May-08	2008	RC	0.15	0	0.05	0	0.009346	2.875

838	27-May-08	2008	RC	0.15	0	0.05	0	0.009346	2.875
839	27-May-08	2008	RC	0.15	0	0.05	0	0.009346	2.875
835	27-May-08	2008	RC	0.15	0	0.05	0	0.009346	2.875
872	28-May-08	2008	HH	0.161765	0.102941	0.014706	0.147059	0	0.615385
873	28-May-08	2008	HH	0.161765	0.102941	0.014706	0.147059	0	0.615385
870	28-May-08	2008	HH	0.161765	0.102941	0.014706	0.147059	0	0.615385
871	28-May-08	2008	HH	0.161765	0.102941	0.014706	0.147059	0	0.615385
874	28-May-08	2008	HH	0.161765	0.102941	0.014706	0.147059	0	0.615385
875	28-May-08	2008	HH	0.161765	0.102941	0.014706	0.147059	0	0.615385
876	28-May-08	2008	HH	0.161765	0.102941	0.014706	0.147059	0	0.615385
877	28-May-08	2008	HH	0.161765	0.102941	0.014706	0.147059	0	0.615385
878	28-May-08	2008	HH	0.161765	0.102941	0.014706	0.147059	0	0.615385
879	28-May-08	2008	HH	0.161765	0.102941	0.014706	0.147059	0	0.615385
880	28-May-08	2008	HH	0.161765	0.102941	0.014706	0.147059	0	0.615385
867	28-May-08	2008	HH	0.161765	0.102941	0.014706	0.147059	0	0.615385
868	28-May-08	2008	HH	0.161765	0.102941	0.014706	0.147059	0	0.615385
869	28-May-08	2008	HH	0.161765	0.102941	0.014706	0.147059	0	0.615385
881	28-May-08	2008	HH	0.161765	0.102941	0.014706	0.147059	0	0.615385
866	28-May-08	2008	HH	0.161765	0.102941	0.014706	0.147059	0	0.615385
916	29-May-08	2008	LT1	0.925926	0.222222	0.037037	0.04	0	3.666667
911	29-May-08	2008	LT1	0.925926	0.222222	0.037037	0.04	0	3.666667
915	29-May-08	2008	LT1	0.925926	0.222222	0.037037	0.04	0	3.666667
913	29-May-08	2008	LT1	0.925926	0.222222	0.037037	0.04	0	3.666667
910	29-May-08	2008	LT1	0.925926	0.222222	0.037037	0.04	0	3.666667
914	29-May-08	2008	LT1	0.925926	0.222222	0.037037	0.04	0	3.666667
909	29-May-08	2008	LT1	0.925926	0.222222	0.037037	0.04	0	3.666667
917	29-May-08	2008	LT1	0.925926	0.222222	0.037037	0.04	0	3.666667
920	29-May-08	2008	LT1	0.925926	0.222222	0.037037	0.04	0	3.666667
903	29-May-08	2008	LT1	0.925926	0.222222	0.037037	0.04	0	3.666667
918	29-May-08	2008	LT1	0.925926	0.222222	0.037037	0.04	0	3.666667
904	29-May-08	2008	LT1	0.925926	0.222222	0.037037	0.04	0	3.666667
912	29-May-08	2008	LT1	0.925926	0.222222	0.037037	0.04	0	3.666667
919	29-May-08	2008	LT1	0.925926	0.222222	0.037037	0.04	0	3.666667
902	29-May-08	2008	LT1	0.925926	0.222222	0.037037	0.04	0	3.666667
905	29-May-08	2008	LT1	0.925926	0.222222	0.037037	0.04	0	3.666667
907	29-May-08	2008	LT1	0.925926	0.222222	0.037037	0.04	0	3.666667
908	29-May-08	2008	LT1	0.925926	0.222222	0.037037	0.04	0	3.666667
900	29-May-08	2008	LT1	0.925926	0.222222	0.037037	0.04	0	3.666667
901	29-May-08	2008	LT1	0.925926	0.222222	0.037037	0.04	0	3.666667
906	29-May-08	2008	LT1	0.925926	0.222222	0.037037	0.04	0	3.666667
921	29-May-08	2008	LT1	0.925926	0.222222	0.037037	0.04	0	3.666667
899	29-May-08	2008	LT1	0.925926	0.222222	0.037037	0.04	0	3.666667
952	29-May-08	2008	LT1	0.925926	0.222222	0.037037	0.04	0	3.666667
953	29-May-08	2008	LT1	0.925926	0.222222	0.037037	0.04	0	3.666667

956	29-May-08	2008	LT1	0.925926	0.222222	0.037037	0.04	0	3.666667
954	29-May-08	2008	LT1	0.925926	0.222222	0.037037	0.04	0	3.666667
955	29-May-08	2008	LT1	0.925926	0.222222	0.037037	0.04	0	3.666667
951	29-May-08	2008	LT1	0.925926	0.222222	0.037037	0.04	0	3.666667
957	29-May-08	2008	LT1	0.925926	0.222222	0.037037	0.04	0	3.666667
959	29-May-08	2008	LT1	0.925926	0.222222	0.037037	0.04	0	3.666667
958	29-May-08	2008	LT1	0.925926	0.222222	0.037037	0.04	0	3.666667
948	29-May-08	2008	LT1	0.925926	0.222222	0.037037	0.04	0	3.666667
949	29-May-08	2008	LT1	0.925926	0.222222	0.037037	0.04	0	3.666667
950	29-May-08	2008	LT1	0.925926	0.222222	0.037037	0.04	0	3.666667
960	29-May-08	2008	LT1	0.925926	0.222222	0.037037	0.04	0	3.666667
947	29-May-08	2008	LT1	0.925926	0.222222	0.037037	0.04	0	3.666667
1004	30-May-08	2008	ML						
1005	30-May-08	2008	ML						
1007	30-May-08	2008	ML						
1003	30-May-08	2008	ML						
995	30-May-08	2008	ML						
993	30-May-08	2008	ML						
994	30-May-08	2008	ML						
996	30-May-08	2008	ML						
998	30-May-08	2008	ML						
1002	30-May-08	2008	ML						
991	30-May-08	2008	ML						
1001	30-May-08	2008	ML						
997	30-May-08	2008	ML						
999	30-May-08	2008	ML						
992	30-May-08	2008	ML						
1000	30-May-08	2008	ML						
1006	30-May-08	2008	ML						
1008	30-May-08	2008	ML						
990	30-May-08	2008	ML						
1314	7-Jun-08	2008	LT1	0.925926	0.222222	0.037037	0.04	0	3.666667
1312	7-Jun-08	2008	LT1	0.925926	0.222222	0.037037	0.04	0	3.666667
1311	7-Jun-08	2008	LT1	0.925926	0.222222	0.037037	0.04	0	3.666667
1313	7-Jun-08	2008	LT1	0.925926	0.222222	0.037037	0.04	0	3.666667
1315	7-Jun-08	2008	LT1	0.925926	0.222222	0.037037	0.04	0	3.666667
1316	7-Jun-08	2008	LT1	0.925926	0.222222	0.037037	0.04	0	3.666667
1310	7-Jun-08	2008	LT1	0.925926	0.222222	0.037037	0.04	0	3.666667
1473	10-Jun-08	2008	TO	0.2	0.2	0.05	0.025	0.00289	0.875
1467	10-Jun-08	2008	TO	0.2	0.2	0.05	0.025	0.00289	0.875
1465	10-Jun-08	2008	TO	0.2	0.2	0.05	0.025	0.00289	0.875
1472	10-Jun-08	2008	TO	0.2	0.2	0.05	0.025	0.00289	0.875
1471	10-Jun-08	2008	TO	0.2	0.2	0.05	0.025	0.00289	0.875
1474	10-Jun-08	2008	TO	0.2	0.2	0.05	0.025	0.00289	0.875

1470	10-Jun-08	2008	TO	0.2	0.2	0.05	0.025	0.00289	0.875
1463	10-Jun-08	2008	TO	0.2	0.2	0.05	0.025	0.00289	0.875
1466	10-Jun-08	2008	TO	0.2	0.2	0.05	0.025	0.00289	0.875
1469	10-Jun-08	2008	TO	0.2	0.2	0.05	0.025	0.00289	0.875
1462	10-Jun-08	2008	TO	0.2	0.2	0.05	0.025	0.00289	0.875
1464	10-Jun-08	2008	TO	0.2	0.2	0.05	0.025	0.00289	0.875
1468	10-Jun-08	2008	TO	0.2	0.2	0.05	0.025	0.00289	0.875
1475	10-Jun-08	2008	TO	0.2	0.2	0.05	0.025	0.00289	0.875
1461	10-Jun-08	2008	TO	0.2	0.2	0.05	0.025	0.00289	0.875
1501	10-Jun-08	2008	TO	0.2	0.2	0.05	0.025	0.00289	0.875
1509	10-Jun-08	2008	TO	0.2	0.2	0.05	0.025	0.00289	0.875
1510	10-Jun-08	2008	TO	0.2	0.2	0.05	0.025	0.00289	0.875
1511	10-Jun-08	2008	TO	0.2	0.2	0.05	0.025	0.00289	0.875
1512	10-Jun-08	2008	TO	0.2	0.2	0.05	0.025	0.00289	0.875
1513	10-Jun-08	2008	TO	0.2	0.2	0.05	0.025	0.00289	0.875
1508	10-Jun-08	2008	TO	0.2	0.2	0.05	0.025	0.00289	0.875
1517	10-Jun-08	2008	TO	0.2	0.2	0.05	0.025	0.00289	0.875
1505	10-Jun-08	2008	TO	0.2	0.2	0.05	0.025	0.00289	0.875
1516	10-Jun-08	2008	TO	0.2	0.2	0.05	0.025	0.00289	0.875
1518	10-Jun-08	2008	TO	0.2	0.2	0.05	0.025	0.00289	0.875
1515	10-Jun-08	2008	TO	0.2	0.2	0.05	0.025	0.00289	0.875
1507	10-Jun-08	2008	TO	0.2	0.2	0.05	0.025	0.00289	0.875
1506	10-Jun-08	2008	TO	0.2	0.2	0.05	0.025	0.00289	0.875
1502	10-Jun-08	2008	TO	0.2	0.2	0.05	0.025	0.00289	0.875
1503	10-Jun-08	2008	TO	0.2	0.2	0.05	0.025	0.00289	0.875
1500	10-Jun-08	2008	TO	0.2	0.2	0.05	0.025	0.00289	0.875
1504	10-Jun-08	2008	TO	0.2	0.2	0.05	0.025	0.00289	0.875
1514	10-Jun-08	2008	TO	0.2	0.2	0.05	0.025	0.00289	0.875
1519	10-Jun-08	2008	TO	0.2	0.2	0.05	0.025	0.00289	0.875
1499	10-Jun-08	2008	TO	0.2	0.2	0.05	0.025	0.00289	0.875
1543	15-Jun-08	2008	LT1	0.925926	0.222222	0.037037	0.04	0	3.666667
1548	15-Jun-08	2008	LT1	0.925926	0.222222	0.037037	0.04	0	3.666667
1550	15-Jun-08	2008	LT1	0.925926	0.222222	0.037037	0.04	0	3.666667
1546	15-Jun-08	2008	LT1	0.925926	0.222222	0.037037	0.04	0	3.666667
1551	15-Jun-08	2008	LT1	0.925926	0.222222	0.037037	0.04	0	3.666667
1545	15-Jun-08	2008	LT1	0.925926	0.222222	0.037037	0.04	0	3.666667
1547	15-Jun-08	2008	LT1	0.925926	0.222222	0.037037	0.04	0	3.666667
1549	15-Jun-08	2008	LT1	0.925926	0.222222	0.037037	0.04	0	3.666667
1553	15-Jun-08	2008	LT1	0.925926	0.222222	0.037037	0.04	0	3.666667
1541	15-Jun-08	2008	LT1	0.925926	0.222222	0.037037	0.04	0	3.666667
1544	15-Jun-08	2008	LT1	0.925926	0.222222	0.037037	0.04	0	3.666667
1552	15-Jun-08	2008	LT1	0.925926	0.222222	0.037037	0.04	0	3.666667
1554	15-Jun-08	2008	LT1	0.925926	0.222222	0.037037	0.04	0	3.666667
1542	15-Jun-08	2008	LT1	0.925926	0.222222	0.037037	0.04	0	3.666667

1540	15-Jun-08	2008	LT1	0.925926	0.222222	0.037037	0.04	0	3.666667
1594	15-Jun-08	2008	LT1	0.925926	0.222222	0.037037	0.04	0	3.666667
1593	15-Jun-08	2008	LT1	0.925926	0.222222	0.037037	0.04	0	3.666667
1591	15-Jun-08	2008	LT1	0.925926	0.222222	0.037037	0.04	0	3.666667
1592	15-Jun-08	2008	LT1	0.925926	0.222222	0.037037	0.04	0	3.666667
1595	15-Jun-08	2008	LT1	0.925926	0.222222	0.037037	0.04	0	3.666667
1596	15-Jun-08	2008	LT1	0.925926	0.222222	0.037037	0.04	0	3.666667
1590	15-Jun-08	2008	LT1	0.925926	0.222222	0.037037	0.04	0	3.666667
1661	15-Jun-08	2008	LT1	0.925926	0.222222	0.037037	0.04	0	3.666667
1657	15-Jun-08	2008	LT1	0.925926	0.222222	0.037037	0.04	0	3.666667
1658	15-Jun-08	2008	LT1	0.925926	0.222222	0.037037	0.04	0	3.666667
1656	15-Jun-08	2008	LT1	0.925926	0.222222	0.037037	0.04	0	3.666667
1648	15-Jun-08	2008	LT1	0.925926	0.222222	0.037037	0.04	0	3.666667
1647	15-Jun-08	2008	LT1	0.925926	0.222222	0.037037	0.04	0	3.666667
1650	15-Jun-08	2008	LT1	0.925926	0.222222	0.037037	0.04	0	3.666667
1652	15-Jun-08	2008	LT1	0.925926	0.222222	0.037037	0.04	0	3.666667
1653	15-Jun-08	2008	LT1	0.925926	0.222222	0.037037	0.04	0	3.666667
1655	15-Jun-08	2008	LT1	0.925926	0.222222	0.037037	0.04	0	3.666667
1649	15-Jun-08	2008	LT1	0.925926	0.222222	0.037037	0.04	0	3.666667
1651	15-Jun-08	2008	LT1	0.925926	0.222222	0.037037	0.04	0	3.666667
1663	15-Jun-08	2008	LT1	0.925926	0.222222	0.037037	0.04	0	3.666667
1660	15-Jun-08	2008	LT1	0.925926	0.222222	0.037037	0.04	0	3.666667
1662	15-Jun-08	2008	LT1	0.925926	0.222222	0.037037	0.04	0	3.666667
1646	15-Jun-08	2008	LT1	0.925926	0.222222	0.037037	0.04	0	3.666667
1664	15-Jun-08	2008	LT1	0.925926	0.222222	0.037037	0.04	0	3.666667
1665	15-Jun-08	2008	LT1	0.925926	0.222222	0.037037	0.04	0	3.666667
1667	15-Jun-08	2008	LT1	0.925926	0.222222	0.037037	0.04	0	3.666667
1670	15-Jun-08	2008	LT1	0.925926	0.222222	0.037037	0.04	0	3.666667
1666	15-Jun-08	2008	LT1	0.925926	0.222222	0.037037	0.04	0	3.666667
1672	15-Jun-08	2008	LT1	0.925926	0.222222	0.037037	0.04	0	3.666667
1673	15-Jun-08	2008	LT1	0.925926	0.222222	0.037037	0.04	0	3.666667
1675	15-Jun-08	2008	LT1	0.925926	0.222222	0.037037	0.04	0	3.666667
1669	15-Jun-08	2008	LT1	0.925926	0.222222	0.037037	0.04	0	3.666667
1671	15-Jun-08	2008	LT1	0.925926	0.222222	0.037037	0.04	0	3.666667
1659	15-Jun-08	2008	LT1	0.925926	0.222222	0.037037	0.04	0	3.666667
1645	15-Jun-08	2008	LT1	0.925926	0.222222	0.037037	0.04	0	3.666667
1668	15-Jun-08	2008	LT1	0.925926	0.222222	0.037037	0.04	0	3.666667
1674	15-Jun-08	2008	LT1	0.925926	0.222222	0.037037	0.04	0	3.666667
1676	15-Jun-08	2008	LT1	0.925926	0.222222	0.037037	0.04	0	3.666667
1654	15-Jun-08	2008	LT1	0.925926	0.222222	0.037037	0.04	0	3.666667
1644	15-Jun-08	2008	LT1	0.925926	0.222222	0.037037	0.04	0	3.666667
1723	16-Jun-08	2008	ML						
1725	16-Jun-08	2008	ML						
1726	16-Jun-08	2008	ML						

1727	16-Jun-08	2008	ML						
1722	16-Jun-08	2008	ML						
1721	16-Jun-08	2008	ML						
1724	16-Jun-08	2008	ML						
1728	16-Jun-08	2008	ML						
1729	16-Jun-08	2008	ML						
1720	16-Jun-08	2008	ML						
1785	17-Jun-08	2008	RP	0.176471	0	0.029412	0.029412	0.015228	3.083333
1786	17-Jun-08	2008	RP	0.176471	0	0.029412	0.029412	0.015228	3.083333
1788	17-Jun-08	2008	RP	0.176471	0	0.029412	0.029412	0.015228	3.083333
1791	17-Jun-08	2008	RP	0.176471	0	0.029412	0.029412	0.015228	3.083333
1789	17-Jun-08	2008	RP	0.176471	0	0.029412	0.029412	0.015228	3.083333
1792	17-Jun-08	2008	RP	0.176471	0	0.029412	0.029412	0.015228	3.083333
1790	17-Jun-08	2008	RP	0.176471	0	0.029412	0.029412	0.015228	3.083333
1793	17-Jun-08	2008	RP	0.176471	0	0.029412	0.029412	0.015228	3.083333
1794	17-Jun-08	2008	RP	0.176471	0	0.029412	0.029412	0.015228	3.083333
1783	17-Jun-08	2008	RP	0.176471	0	0.029412	0.029412	0.015228	3.083333
1782	17-Jun-08	2008	RP	0.176471	0	0.029412	0.029412	0.015228	3.083333
1781	17-Jun-08	2008	RP	0.176471	0	0.029412	0.029412	0.015228	3.083333
1784	17-Jun-08	2008	RP	0.176471	0	0.029412	0.029412	0.015228	3.083333
1787	17-Jun-08	2008	RP	0.176471	0	0.029412	0.029412	0.015228	3.083333
1795	17-Jun-08	2008	RP	0.176471	0	0.029412	0.029412	0.015228	3.083333
1780	17-Jun-08	2008	RP	0.176471	0	0.029412	0.029412	0.015228	3.083333
1808	17-Jun-08	2008	RP	0.176471	0	0.029412	0.029412	0.015228	3.083333
1812	17-Jun-08	2008	RP	0.176471	0	0.029412	0.029412	0.015228	3.083333
1811	17-Jun-08	2008	RP	0.176471	0	0.029412	0.029412	0.015228	3.083333
1813	17-Jun-08	2008	RP	0.176471	0	0.029412	0.029412	0.015228	3.083333
1809	17-Jun-08	2008	RP	0.176471	0	0.029412	0.029412	0.015228	3.083333
1810	17-Jun-08	2008	RP	0.176471	0	0.029412	0.029412	0.015228	3.083333
1814	17-Jun-08	2008	RP	0.176471	0	0.029412	0.029412	0.015228	3.083333
1815	17-Jun-08	2008	RP	0.176471	0	0.029412	0.029412	0.015228	3.083333
1807	17-Jun-08	2008	RP	0.176471	0	0.029412	0.029412	0.015228	3.083333
2181	20-Jun-08	2008	D1	0.2	0.28	0.04	0.02	0.006036	0.8
2183	20-Jun-08	2008	D1	0.2	0.28	0.04	0.02	0.006036	0.8
2185	20-Jun-08	2008	D1	0.2	0.28	0.04	0.02	0.006036	0.8
2186	20-Jun-08	2008	D1	0.2	0.28	0.04	0.02	0.006036	0.8
2180	20-Jun-08	2008	D1	0.2	0.28	0.04	0.02	0.006036	0.8
2182	20-Jun-08	2008	D1	0.2	0.28	0.04	0.02	0.006036	0.8
2184	20-Jun-08	2008	D1	0.2	0.28	0.04	0.02	0.006036	0.8
2187	20-Jun-08	2008	D1	0.2	0.28	0.04	0.02	0.006036	0.8
2179	20-Jun-08	2008	D1	0.2	0.28	0.04	0.02	0.006036	0.8
1828	23-Jun-08	2008	YY	0.14	0.24	0.08	0.02	0	0.65
1830	23-Jun-08	2008	YY	0.14	0.24	0.08	0.02	0	0.65
1831	23-Jun-08	2008	YY	0.14	0.24	0.08	0.02	0	0.65

1833	23-Jun-08	2008	YY	0.14	0.24	0.08	0.02	0	0.65
1827	23-Jun-08	2008	YY	0.14	0.24	0.08	0.02	0	0.65
1829	23-Jun-08	2008	YY	0.14	0.24	0.08	0.02	0	0.65
1832	23-Jun-08	2008	YY	0.14	0.24	0.08	0.02	0	0.65
1834	23-Jun-08	2008	YY	0.14	0.24	0.08	0.02	0	0.65
1826	23-Jun-08	2008	YY	0.14	0.24	0.08	0.02	0	0.65
1838	23-Jun-08	2008	TO	0.2	0.2	0.05	0.025	0.00289	0.875
1840	23-Jun-08	2008	TO	0.2	0.2	0.05	0.025	0.00289	0.875
1841	23-Jun-08	2008	TO	0.2	0.2	0.05	0.025	0.00289	0.875
1839	23-Jun-08	2008	TO	0.2	0.2	0.05	0.025	0.00289	0.875
1842	23-Jun-08	2008	TO	0.2	0.2	0.05	0.025	0.00289	0.875
1843	23-Jun-08	2008	TO	0.2	0.2	0.05	0.025	0.00289	0.875
1845	23-Jun-08	2008	TO	0.2	0.2	0.05	0.025	0.00289	0.875
1846	23-Jun-08	2008	TO	0.2	0.2	0.05	0.025	0.00289	0.875
1837	23-Jun-08	2008	TO	0.2	0.2	0.05	0.025	0.00289	0.875
1844	23-Jun-08	2008	TO	0.2	0.2	0.05	0.025	0.00289	0.875
1847	23-Jun-08	2008	TO	0.2	0.2	0.05	0.025	0.00289	0.875
1848	23-Jun-08	2008	TO	0.2	0.2	0.05	0.025	0.00289	0.875
1849	23-Jun-08	2008	TO	0.2	0.2	0.05	0.025	0.00289	0.875
1850	23-Jun-08	2008	TO	0.2	0.2	0.05	0.025	0.00289	0.875
1855	23-Jun-08	2008	TO	0.2	0.2	0.05	0.025	0.00289	0.875
1852	23-Jun-08	2008	TO	0.2	0.2	0.05	0.025	0.00289	0.875
1857	23-Jun-08	2008	TO	0.2	0.2	0.05	0.025	0.00289	0.875
1854	23-Jun-08	2008	TO	0.2	0.2	0.05	0.025	0.00289	0.875
1853	23-Jun-08	2008	TO	0.2	0.2	0.05	0.025	0.00289	0.875
1836	23-Jun-08	2008	TO	0.2	0.2	0.05	0.025	0.00289	0.875
1851	23-Jun-08	2008	TO	0.2	0.2	0.05	0.025	0.00289	0.875
1856	23-Jun-08	2008	TO	0.2	0.2	0.05	0.025	0.00289	0.875
1858	23-Jun-08	2008	TO	0.2	0.2	0.05	0.025	0.00289	0.875
1835	23-Jun-08	2008	TO	0.2	0.2	0.05	0.025	0.00289	0.875
1910	23-Jun-08	2008	TO	0.2	0.2	0.05	0.025	0.00289	0.875
1906	23-Jun-08	2008	TO	0.2	0.2	0.05	0.025	0.00289	0.875
1907	23-Jun-08	2008	TO	0.2	0.2	0.05	0.025	0.00289	0.875
1905	23-Jun-08	2008	TO	0.2	0.2	0.05	0.025	0.00289	0.875
1908	23-Jun-08	2008	TO	0.2	0.2	0.05	0.025	0.00289	0.875
1909	23-Jun-08	2008	TO	0.2	0.2	0.05	0.025	0.00289	0.875
1911	23-Jun-08	2008	TO	0.2	0.2	0.05	0.025	0.00289	0.875
1904	23-Jun-08	2008	TO	0.2	0.2	0.05	0.025	0.00289	0.875
1943	27-Jun-08	2008	LT1	0.925926	0.222222	0.037037	0.04	0	3.666667
1938	27-Jun-08	2008	LT1	0.925926	0.222222	0.037037	0.04	0	3.666667
1942	27-Jun-08	2008	LT1	0.925926	0.222222	0.037037	0.04	0	3.666667
1940	27-Jun-08	2008	LT1	0.925926	0.222222	0.037037	0.04	0	3.666667
1937	27-Jun-08	2008	LT1	0.925926	0.222222	0.037037	0.04	0	3.666667
1939	27-Jun-08	2008	LT1	0.925926	0.222222	0.037037	0.04	0	3.666667

1941	27-Jun-08	2008	LT1	0.925926	0.222222	0.037037	0.04	0	3.666667
1944	27-Jun-08	2008	LT1	0.925926	0.222222	0.037037	0.04	0	3.666667
1936	27-Jun-08	2008	LT1	0.925926	0.222222	0.037037	0.04	0	3.666667
2029	UK2	2007	SD						
2031	UK2	2007	SD						
2033	UK2	2007	SD						
2039	UK2	2007	SD						
2045	UK2	2007	SD						
2046	UK2	2007	SD						
2041	UK2	2007	SD						
2044	UK2	2007	SD						
2048	UK2	2007	SD						
2042	UK2	2007	SD						
2043	UK2	2007	SD						
2049	UK2	2007	SD						
2050	UK2	2007	SD						
2055	UK2	2007	SD						
2053	UK2	2007	SD						
2054	UK2	2007	SD						
2057	UK2	2007	SD						
2032	UK2	2007	SD						
2030	UK2	2007	SD						
2035	UK2	2007	SD						
2034	UK2	2007	SD						
2036	UK2	2007	SD						
2038	UK2	2007	SD						
2052	UK2	2007	SD						
2058	UK2	2007	SD						
2047	UK2	2007	SD						
2056	UK2	2007	SD						
2059	UK2	2007	SD						
2028	UK2	2007	SD						
2037	UK2	2007	SD						
2051	UK2	2007	SD						
2060	UK2	2007	SD						
2040	UK2	2007	SD						
2027	UK2	2007	SD						
		2007	TO	0.175	0.125	0.175	0	0.00289	0.875
		2007	TO	0.175	0.125	0.175	0	0.00289	0.875
		2007	TO	0.175	0.125	0.175	0	0.00289	0.875
		2007	TO	0.175	0.125	0.175	0	0.00289	0.875
		2007	TO	0.175	0.125	0.175	0	0.00289	0.875
		2007	TO	0.175	0.125	0.175	0	0.00289	0.875
99	11-May-05	2005	ML						

101	11-May-05	2005	ML						
100	11-May-05	2005	ML						
230	15-May-05	2005	SW	0.264151	0.113208	0.018868	0.169811	0.044444	1.4
235	15-May-05	2005	SW	0.264151	0.113208	0.018868	0.169811	0.044444	1.4
234	15-May-05	2005	SW	0.264151	0.113208	0.018868	0.169811	0.044444	1.4
236	15-May-05	2005	SW	0.264151	0.113208	0.018868	0.169811	0.044444	1.4
225	15-May-05	2005	SW	0.264151	0.113208	0.018868	0.169811	0.044444	1.4
226	15-May-05	2005	SW	0.264151	0.113208	0.018868	0.169811	0.044444	1.4
232	15-May-05	2005	SW	0.264151	0.113208	0.018868	0.169811	0.044444	1.4
229	15-May-05	2005	SW	0.264151	0.113208	0.018868	0.169811	0.044444	1.4
231	15-May-05	2005	SW	0.264151	0.113208	0.018868	0.169811	0.044444	1.4
233	15-May-05	2005	SW	0.264151	0.113208	0.018868	0.169811	0.044444	1.4
228	15-May-05	2005	SW	0.264151	0.113208	0.018868	0.169811	0.044444	1.4
237	15-May-05	2005	SW	0.264151	0.113208	0.018868	0.169811	0.044444	1.4
227	15-May-05	2005	SW	0.264151	0.113208	0.018868	0.169811	0.044444	1.4
66	10-May-05	2005	SC	0.183486	0	0.073394	0	0.006329	6.769231
70	10-May-05	2005	SC	0.183486	0	0.073394	0	0.006329	6.769231
69	10-May-05	2005	SC	0.183486	0	0.073394	0	0.006329	6.769231
68	10-May-05	2005	SC	0.183486	0	0.073394	0	0.006329	6.769231
67	10-May-05	2005	SC	0.183486	0	0.073394	0	0.006329	6.769231

OBS	Plot	N.O.R	Current.ne	Nest.ID	Last.nest.s	Last.nest.y	Play.order	Start.time	Model
	70102	N	BCCH	04-ARN-04	NEW	2004	BRM	7:30	BCCH
	70102	N	BCCH	04-ARN-04	NEW	2004	BRM	7:30	BCCH
	70102	N	BCCH	04-ARN-04	NEW	2004	BRM	7:30	BCCH
	70102	N	BCCH	04-ARN-04	NEW	2004	BRM	7:30	BCCH
	70102	N	BCCH	04-ARN-04	NEW	2004	BRM	7:30	RBNU
	70102	N	BCCH	04-ARN-04	NEW	2004	BRM	7:30	RBNU
	70102	N	BCCH	04-ARN-04	NEW	2004	BRM	7:30	MOCH
	70102	N	BCCH	04-ARN-04	NEW	2004	BRM	7:30	MOCH
	70102	N	BCCH	04-ARN-04	NEW	2004	BRM	7:30	MOCH
	70102	N	BCCH	04-ARN-04	NEW	2004	BRM	7:30	MOCH
	70102	N	BCCH	04-ARN-04	NEW	2004	BRM	7:30	RBNU
	70102	N	BCCH	04-ARN-04	NEW	2004	BRM	7:30	BCCH
	70102	N	BCCH	04-ARN-04	NEW	2004	BRM	7:30	BCCH
	70102	N	BCCH	04-ARN-04	NEW	2004	BRM	7:30	RBNU
	70102	N	BCCH	04-ARN-04	NEW	2004	BRM	7:30	RBNU
	70102	N	BCCH	04-ARN-04	NEW	2004	BRM	7:30	MOCH
	70102	N	BCCH	04-ARN-04	NEW	2004	BRM	7:30	MOCH
	70102	N	BCCH	04-ARN-04	NEW	2004	BRM	7:30	none
	70102	N	BCCH	04-ARN-04	NEW	2004	BRM	7:30	RBNU
	70102	N	BCCH	04-ARN-04	NEW	2004	BRM	7:30	RBNU
ARN	70327	N	BCCH	08-ARN-006			BRM	9:50	BCCH
ARN	70327	N	BCCH	08-ARN-006			BRM	9:50	MOCH
ARN	70327	N	BCCH	08-ARN-006			BRM	9:50	BCCH
ARN	70327	N	BCCH	08-ARN-006			BRM	9:50	BCCH
ARN	70327	N	BCCH	08-ARN-006			BRM	9:50	BCCH
ARN	70327	N	BCCH	08-ARN-006			BRM	9:50	BCCH
ARN	70327	N	BCCH	08-ARN-006			BRM	9:50	BCCH
ARN	70327	N	BCCH	08-ARN-006			BRM	9:50	BCCH
ARN	70327	N	BCCH	08-ARN-006			BRM	9:50	BCCH
ARN	70327	N	BCCH	08-ARN-006			BRM	9:50	BCCH
ARN	70327	N	BCCH	08-ARN-006			BRM	9:50	BCCH
ARN	70327	N	BCCH	08-ARN-006			BRM	9:50	RBNU
ARN	70327	N	BCCH	08-ARN-006			BRM	9:50	RBNU
ARN	70327	N	BCCH	08-ARN-006			BRM	9:50	RBNU
ARN	70327	N	BCCH	08-ARN-006			BRM	9:50	RBNU
ARN	70327	N	BCCH	08-ARN-006			BRM	9:50	MOCH
ARN	70327	N	BCCH	08-ARN-006			BRM	9:50	MOCH
ARN	70327	N	BCCH	08-ARN-006			BRM	9:50	MOCH
ARN	70327	N	BCCH	08-ARN-006			BRM	9:50	MOCH
ARN	70327	N	BCCH	08-ARN-006			BRM	9:50	BCCH
ARN	70327	N	BCCH	08-ARN-006			BRM	9:50	BCCH
ARN	70327	N	BCCH	08-ARN-006			BRM	9:50	BCCH
ARN	70327	N	BCCH	08-ARN-006			BRM	9:50	RBNU

ARN	70327	N	BCCH	08-ARN-006	BRM	9:50	RBNU
ARN	70327	N	BCCH	08-ARN-006	BRM	9:50	RBNU
ARN	70327	N	BCCH	08-ARN-006	BRM	9:50	MOCH
ARN	70327	N	BCCH	08-ARN-006	BRM	9:50	BCCH
ARN	70327	N	BCCH	08-ARN-006	BRM	9:50	RBNU
ARN	70327	N	BCCH	08-ARN-006	BRM	9:50	BCCH
ARN	70327	N	BCCH	08-ARN-006	BRM	9:50	RBNU
ARN	70327	N	BCCH	08-ARN-006	BRM	9:50	RBNU
ARN	70327	N	BCCH	08-ARN-006	BRM	9:50	RBNU
ARN	70327	N	BCCH	08-ARN-006	BRM	9:50	RBNU
ARN	70327	N	BCCH	08-ARN-006	BRM	9:50	MOCH
ARN	70327	N	BCCH	08-ARN-006	BRM	9:50	
HK	70221	N	BCCH	08-ARN-017	BRM	9:04	MOCH
HK	70221	N	BCCH	08-ARN-017	BRM	9:04	MOCH
HK	70221	N	BCCH	08-ARN-017	BRM	9:04	MOCH
HK	70221	N	BCCH	08-ARN-017	BRM	9:04	BCCH
HK	70221	N	BCCH	08-ARN-017	BRM	9:04	BCCH
HK	70221	N	BCCH	08-ARN-017	BRM	9:04	BCCH
HK	70221	N	BCCH	08-ARN-017	BRM	9:04	BCCH
HK	70221	N	BCCH	08-ARN-017	BRM	9:04	MOCH
HK	70221	N	BCCH	08-ARN-017	BRM	9:04	BCCH
HK	70221	N	BCCH	08-ARN-017	BRM	9:04	BCCH
HK	70221	N	BCCH	08-ARN-017	BRM	9:04	RBNU
HK	70221	N	BCCH	08-ARN-017	BRM	9:04	BCCH
HK	70221	N	BCCH	08-ARN-017	BRM	9:04	BCCH
HK	70221	N	BCCH	08-ARN-017	BRM	9:04	RBNU
HK	70221	N	BCCH	08-ARN-017	BRM	9:04	MOCH
HK	70221	N	BCCH	08-ARN-017	BRM	9:04	MOCH
HK	70221	N	BCCH	08-ARN-017	BRM	9:04	
HK		N	BCCH	08-ARN-017	MBR	11:22	RBNU
HK		N	BCCH	08-ARN-017	MBR	11:22	RBNU
HK		N	BCCH	08-ARN-017	MBR	11:22	MOCH
HK		N	BCCH	08-ARN-017	MBR	11:22	MOCH
HK		N	BCCH	08-ARN-017	MBR	11:22	RBNU
HK		N	BCCH	08-ARN-017	MBR	11:22	RBNU
HK		N	BCCH	08-ARN-017	MBR	11:22	RBNU
HK		N	BCCH	08-ARN-017	MBR	11:22	RBNU
HK		N	BCCH	08-ARN-017	MBR	11:22	RBNU
HK		N	BCCH	08-ARN-017	MBR	11:22	RBNU
HK		N	BCCH	08-ARN-017	MBR	11:22	RBNU
HK		N	BCCH	08-ARN-017	MBR	11:22	RBNU
HK		N	BCCH	08-ARN-017	MBR	11:22	RBNU
HK		N	BCCH	08-ARN-017	MBR	11:22	MOCH
HK		N	BCCH	08-ARN-017	MBR	11:22	BCCH

HK		N	BCCH	08-ARN-017			MBR	11:22	RBNU
HK		N	BCCH	08-ARN-017			MBR	11:22	RBNU
HK		N	BCCH	08-ARN-017			MBR	11:22	
IB	7897	N	BOCH	07-AE-025	ATTW	2004	MBR	11:50 AM	MOCH
IB	7897	N	BOCH	07-AE-025	ATTW	2004	MBR	11:50 AM	MOCH
IB	7897	N	BOCH	07-AE-025	ATTW	2004	MBR	11:50 AM	MOCH
IB	7897	N	BOCH	07-AE-025	ATTW	2004	MBR	11:50 AM	MOCH
IB	7897	N	BOCH	07-AE-025	ATTW	2004	MBR	11:50 AM	MOCH
IB	7897	N	BOCH	07-AE-025	ATTW	2004	MBR	11:50 AM	MOCH
IB	7897	N	BOCH	07-AE-025	ATTW	2004	MBR	11:50 AM	MOCH
IB	7897	N	BOCH	07-AE-025	ATTW	2004	MBR	11:50 AM	MOCH
IB	7897	N	BOCH	07-AE-025	ATTW	2004	MBR	11:50 AM	MOCH
IB	7897	N	BOCH	07-AE-025	ATTW	2004	MBR	11:50 AM	BCCH
IB	7897	N	BOCH	07-AE-025	ATTW	2004	MBR	11:50 AM	RBNU
	70705	N	MOCH	04-NK-024			BRM	8:15	RBNU
	70705	N	MOCH	04-NK-024			BRM	8:15	RBNU
	70705	N	MOCH	04-NK-024			BRM	8:15	BCCH
	70705	N	MOCH	04-NK-024			BRM	8:15	BCCH
	70705	N	MOCH	04-NK-024			BRM	8:15	none
	70705	N	MOCH	04-NK-024			BRM	8:15	BCCH
	70705	N	MOCH	04-NK-024			BRM	8:15	BCCH
	70705	N	MOCH	04-NK-024			BRM	8:15	BCCH
	70705	N	MOCH	04-NK-024			BRM	8:15	BCCH
	70705	N	MOCH	04-NK-024			BRM	8:15	RBNU
	70705	N	MOCH	04-NK-024			BRM	8:15	RBNU
	70705	N	MOCH	04-NK-024			BRM	8:15	RBNU
	70705	N	MOCH	04-NK-024			BRM	8:15	RBNU
	70705	N	MOCH	04-NK-024			BRM	8:15	RBNU
	70705	N	MOCH	04-NK-024			BRM	8:15	MOCH
	70705	N	MOCH	04-NK-024			BRM	8:15	MOCH
	70705	N	MOCH	04-NK-024			BRM	8:15	MOCH
	70705	N	MOCH	04-NK-024			BRM	8:15	none
	70418	N	MOCH	05-MN-008	none	none	RMB	8:43	MOCH
	70418	N	MOCH	05-MN-008	none	none	RMB	8:43	BCCH
	70418	N	MOCH	05-MN-008	none	none	RMB	8:43	RBNU
	70418	N	MOCH	05-MN-008	none	none	RMB	8:43	MOCH
	70418	N	MOCH	05-MN-008	none	none	RMB	8:43	none
	70418	N	MOCH	05-MN-008	none	none	RMB	8:43	RBNU
	70418	N	MOCH	05-MN-008	none	none	RMB	8:43	RBNU
	70418	N	MOCH	05-MN-008	none	none	RMB	8:43	MOCH
	70418	N	MOCH	05-MN-008	none	none	RMB	8:43	RBNU
	70418	N	MOCH	05-MN-008	none	none	RMB	8:43	BCCH
	70418	N	MOCH	05-MN-008	none	none	RMB	8:43	none
	70418	N	MOCH	05-MN-008	none	none	RMB	8:43	BCCH

70418	N	MOCH	05-MN-008	none	none	RMB	8:43	none
70418	N	MOCH	05-MN-008	none	none	RMB	8:43	RBNU
70418	N	MOCH	05-MN-008	none	none	RMB	8:43	MOCH
70418	N	MOCH	05-MN-008	none	none	RMB	8:43	RBNU
70418	N	MOCH	05-MN-008	none	none	RMB	8:43	RBNU
70418	N	MOCH	05-MN-008	none	none	RMB	8:43	RBNU
70418	N	MOCH	05-MN-008	none	none	RMB	8:43	RBNU
70418	N	MOCH	05-MN-008	none	none	RMB	8:43	MOCH
70418	N	MOCH	05-MN-008	none	none	RMB	8:43	BCCH
70426	N	MOCH	05-ARN-013			RMB	10:40	MOCH
70426	N	MOCH	05-ARN-013			RMB	10:40	RBNU
70426	N	MOCH	05-ARN-013			RMB	10:40	MOCH
70426	N	MOCH	05-ARN-013			RMB	10:40	MOCH
70426	N	MOCH	05-ARN-013			RMB	10:40	MOCH
70426	N	MOCH	05-ARN-013			RMB	10:40	RBNU
70426	N	MOCH	05-ARN-013			RMB	10:40	RBNU
70426	N	MOCH	05-ARN-013			RMB	10:40	RBNU
70426	N	MOCH	05-ARN-013			RMB	10:40	BCCH
70426	N	MOCH	05-ARN-013			RMB	10:40	RBNU
70426	N	MOCH	05-ARN-013			RMB	10:40	RBNU
70426	N	MOCH	05-ARN-013			RMB	10:40	MOCH
70426	N	MOCH	05-ARN-013			RMB	10:40	BCCH
70426	N	MOCH	05-ARN-013			RMB	10:40	none
NEW	N	MOCH	05-ARN-014			RBM	14:03	BCCH
NEW	N	MOCH	05-ARN-014			RBM	14:03	BCCH
NEW	N	MOCH	05-ARN-014			RBM	14:03	MOCH
NEW	N	MOCH	05-ARN-014			RBM	14:03	BCCH
NEW	N	MOCH	05-ARN-014			RBM	14:03	MOCH
NEW	N	MOCH	05-ARN-014			RBM	14:03	MOCH
NEW	N	MOCH	05-ARN-014			RBM	14:03	BCCH
NEW	N	MOCH	05-ARN-014			RBM	14:03	MOCH
NEW	N	MOCH	05-ARN-014			RBM	14:03	RBNU
NEW	N	MOCH	05-ARN-014			RBM	14:03	RBNU
NEW	N	MOCH	05-ARN-014			RBM	14:03	RBNU
NEW	N	MOCH	05-ARN-014			RBM	14:03	BCCH
NEW	N	MOCH	05-ARN-014			RBM	14:03	BCCH
NEW	N	MOCH	05-ARN-014			RBM	14:03	MOCH
NEW	N	MOCH	05-ARN-014			RBM	14:03	MOCH
NEW	N	MOCH	05-ARN-014			RBM	14:03	RBNU
NEW	N	MOCH	05-ARN-014			RBM	14:03	MOCH
NEW	N	MOCH	05-ARN-014			RBM	14:03	RBNU
NEW	N	MOCH	05-ARN-014			RBM	14:03	RBNU
70401	N	MOCH	05-MN-026			MBR	14:05	MOCH
70401	N	MOCH	05-MN-026			MBR	14:05	BCCH

70401	N	MOCH	05-MN-026			MBR	14:05	BCCH
70401	N	MOCH	05-MN-026			MBR	14:05	MOCH
70401	N	MOCH	05-MN-026			MBR	14:05	none
70401	N	MOCH	05-MN-026			MBR	14:05	MOCH
70401	N	MOCH	05-MN-026			MBR	14:05	BCCH
70401	N	MOCH	05-MN-026			MBR	14:05	BCCH
70401	N	MOCH	05-MN-026			MBR	14:05	RBNU
70401	N	MOCH	05-MN-026			MBR	14:05	MOCH
70401	N	MOCH	05-MN-026			MBR	14:05	MOCH
70401	N	MOCH	05-MN-026			MBR	14:05	BCCH
70401	N	MOCH	05-MN-026			MBR	14:05	BCCH
70401	N	MOCH	05-MN-026			MBR	14:05	RBNU
70401	N	MOCH	05-MN-026			MBR	14:05	RBNU
70426	N	MOCH	05-ARN-013			RMB	13:12	MOCH
70426	N	MOCH	05-ARN-013			RMB	13:12	MOCH
70426	N	MOCH	05-ARN-013			RMB	13:12	BCCH
70426	N	MOCH	05-ARN-013			RMB	13:12	BCCH
70426	N	MOCH	05-ARN-013			RMB	13:12	MOCH
70426	N	MOCH	05-ARN-013			RMB	13:12	BCCH
70426	N	MOCH	05-ARN-013			RMB	13:12	BCCH
70426	N	MOCH	05-ARN-013			RMB	13:12	RBNU
70426	N	MOCH	05-ARN-013			RMB	13:12	RBNU
70426	N	MOCH	05-ARN-013			RMB	13:12	BCCH
70426	N	MOCH	05-ARN-013			RMB	13:12	MOCH
70426	N	MOCH	05-ARN-013			RMB	13:12	RBNU
70426	N	MOCH	05-ARN-013			RMB	13:12	RBNU
70416	N	MOCH	05-ARN-03	none	none	RBM	13:35	MOCH
70416	N	MOCH	05-ARN-03	none	none	RBM	13:35	MOCH
70416	N	MOCH	05-ARN-03	none	none	RBM	13:35	none
70416	N	MOCH	05-ARN-03	none	none	RBM	13:35	none
70416	N	MOCH	05-ARN-03	none	none	RBM	13:35	RBNU
70416	N	MOCH	05-ARN-03	none	none	RBM	13:35	BCCH
70416	N	MOCH	05-ARN-03	none	none	RBM	13:35	MOCH
70416	N	MOCH	05-ARN-03	none	none	RBM	13:35	MOCH
70416	N	MOCH	05-ARN-03	none	none	RBM	13:35	MOCH
70416	N	MOCH	05-ARN-03	none	none	RBM	13:35	BCCH
70418	N	MOCH	05-MN-008			RMB	14:05	BCCH
70418	N	MOCH	05-MN-008			RMB	14:05	BCCH
70418	N	MOCH	05-MN-008			RMB	14:05	BCCH
70418	N	MOCH	05-MN-008			RMB	14:05	BCCH
70418	N	MOCH	05-MN-008			RMB	14:05	BCCH
70418	N	MOCH	05-MN-008			RMB	14:05	MOCH
70418	N	MOCH	05-MN-008			RMB	14:05	BCCH
70418	N	MOCH	05-MN-008			RMB	14:05	RBNU

	70418	N	MOCH	05-MN-008			RMB	14:05	BCCH
	70418	N	MOCH	05-MN-008			RMB	14:05	BCCH
	70418	N	MOCH	05-MN-008			RMB	14:05	RBNU
	70418	N	MOCH	05-MN-008			RMB	14:05	BCCH
	70418	N	MOCH	05-MN-008			RMB	14:05	BCCH
	70418	N	MOCH	05-MN-008			RMB	14:05	RBNU
	70418	N	MOCH	05-MN-008			RMB	14:05	RBNU
	70418	N	MOCH	05-MN-008			RMB	14:05	MOCH
	70418	N	MOCH	05-MN-008			RMB	14:05	MOCH
	70418	N	MOCH	05-MN-008			RMB	14:05	MOCH
	70418	N	MOCH	05-MN-008			RMB	14:05	MOCH
	70418	N	MOCH	05-MN-008			RMB	14:05	none
	70418	N	MOCH	05-MN-008			RMB	14:05	none
	70418	N	MOCH	05-MN-008			RMB	14:05	none
	70128	N	MOCH	06-ARN-01	RNSA	2004	MBR	8:15	RBNU
	70128	N	MOCH	06-ARN-01	RNSA	2004	MBR	8:15	BCCH
	70128	N	MOCH	06-ARN-01	RNSA	2004	MBR	8:15	MOCH
	70128	N	MOCH	06-ARN-01	RNSA	2004	MBR	8:15	BCCH
	70128	N	MOCH	06-ARN-01	RNSA	2004	MBR	8:15	MOCH
	70128	N	MOCH	06-ARN-01	RNSA	2004	MBR	8:15	RBNU
	70128	N	MOCH	06-ARN-01	RNSA	2004	MBR	8:15	
IB	7406	N	MOCH	07-LP-015	GLSA	2006	RMB	9:16 AM	BCCH
IB	7406	N	MOCH	07-LP-015	GLSA	2006	RMB	9:16 AM	BCCH
IB	7406	N	MOCH	07-LP-015	GLSA	2006	RMB	9:16 AM	MOCH
IB	7406	N	MOCH	07-LP-015	GLSA	2006	RMB	9:16 AM	BCCH
IB	7406	N	MOCH	07-LP-015	GLSA	2006	RMB	9:16 AM	BCCH
IB	7406	N	MOCH	07-LP-015	GLSA	2006	RMB	9:16 AM	RBNU
IB	7406	N	MOCH	07-LP-015	GLSA	2006	RMB	9:16 AM	MOCH
IB	7406	N	MOCH	07-LP-015	GLSA	2006	RMB	9:16 AM	MOCH
IB	7406	N	MOCH	07-LP-015	GLSA	2006	RMB	9:16 AM	MOCH
IB	7406	N	MOCH	07-LP-015	GLSA	2006	RMB	9:16 AM	BCCH
IB	7406	N	MOCH	07-LP-015	GLSA	2006	RMB	9:16 AM	BCCH
IB	7406	N	MOCH	07-LP-015	GLSA	2006	RMB	9:16 AM	BCCH
IB	7406	N	MOCH	07-LP-015	GLSA	2006	RMB	9:16 AM	RBNU
IB	7406	N	MOCH	07-LP-015	GLSA	2006	RMB	9:16 AM	MOCH
IB	7406	N	MOCH	07-LP-015	GLSA	2006	RMB	9:16 AM	BCCH
IB	7360	N	MOCH	07-KS-041	EUST	2001	BRM	8:20 AM	RBNU
IB	7360	N	MOCH	07-KS-041	EUST	2001	BRM	8:20 AM	MOCH
IB	7360	N	MOCH	07-KS-041	EUST	2001	BRM	8:20 AM	MOCH
IB	7360	N	MOCH	07-KS-041	EUST	2001	BRM	8:20 AM	RBNU
IB	7360	N	MOCH	07-KS-041	EUST	2001	BRM	8:20 AM	RBNU
IB	7360	N	MOCH	07-KS-041	EUST	2001	BRM	8:20 AM	RBNU
IB	7360	N	MOCH	07-KS-041	EUST	2001	BRM	8:20 AM	RBNU
IB	7360	N	MOCH	07-KS-041	EUST	2001	BRM	8:20 AM	RBNU

IB	7360	N	MOCH	07-KS-041	EUST	2001	BRM	8:20 AM	RBNU
IB	7360	N	MOCH	07-KS-041	EUST	2001	BRM	8:20 AM	RBNU
IB	7360	N	MOCH	07-KS-041	EUST	2001	BRM	8:20 AM	RBNU
IB	7360	N	MOCH	07-KS-041	EUST	2001	BRM	8:20 AM	RBNU
IB	7360	N	MOCH	07-KS-041	EUST	2001	BRM	8:20 AM	MOCH
IB	7360	N	MOCH	07-KS-041	EUST	2001	BRM	8:20 AM	RBNU
IB	7360	N	MOCH	07-KS-041	EUST	2001	BRM	8:20 AM	MOCH
IB	7360	N	MOCH	07-KS-041	EUST	2001	BRM	8:20 AM	RBNU
IB	7360	N	MOCH	07-KS-041	EUST	2001	BRM	8:20 AM	RBNU
IB	7360	N	MOCH	07-KS-041	EUST	2001	BRM	8:20 AM	RBNU
IB	7360	N	MOCH	07-KS-041	EUST	2001	BRM	8:20 AM	MOCH
IB	7360	N	MOCH	07-KS-041	EUST	2001	BRM	8:20 AM	MOCH
IB	7360	N	MOCH	07-KS-041	EUST	2001	BRM	8:20 AM	MOCH
IB	7360	N	MOCH	07-KS-041	EUST	2001	BRM	8:20 AM	MOCH
IB	7360	N	MOCH	07-KS-041	EUST	2001	BRM	8:20 AM	MOCH
IB	7360	N	MOCH	07-KS-041	EUST	2001	BRM	8:20 AM	BCCH
IB	7360	N	MOCH	07-KS-041	EUST	2001	BRM	8:20 AM	BCCH
IB	7360	N	MOCH	07-KS-041	EUST	2001	BRM	8:20 AM	BCCH
IB	7360	N	MOCH	07-KS-041	EUST	2001	BRM	8:20 AM	RBNU
IB	7360	N	MOCH	07-KS-041	EUST	2001	BRM	8:20 AM	BCCH
IB	7360	N	MOCH	07-KS-041	EUST	2001	BRM	8:20 AM	BCCH
IB	7360	N	MOCH	07-KS-041	EUST	2001	BRM	8:20 AM	RBNU
IB	7360	N	MOCH	07-KS-041	EUST	2001	BRM	8:20 AM	MOCH
ARN	7349	N	MOCH	07-KS-034	MOBL	2005	MBR	10:07 AM	RBNU
ARN	7349	N	MOCH	07-KS-034	MOBL	2005	MBR	10:07 AM	BCCH
ARN	7349	N	MOCH	07-KS-034	MOBL	2005	MBR	10:07 AM	MOCH
ARN	7349	N	MOCH	07-KS-034	MOBL	2005	MBR	10:07 AM	MOCH
ARN	7349	N	MOCH	07-KS-034	MOBL	2005	MBR	10:07 AM	MOCH
ARN	7349	N	MOCH	07-KS-034	MOBL	2005	MBR	10:07 AM	MOCH
ARN	7349	N	MOCH	07-KS-034	MOBL	2005	MBR	10:07 AM	RBNU
ARN	7349	N	MOCH	07-KS-034	MOBL	2005	MBR	10:07 AM	RBNU
ARN	7349	N	MOCH	07-KS-034	MOBL	2005	MBR	10:07 AM	MOCH
ARN	7349	N	MOCH	07-KS-034	MOBL	2005	MBR	10:07 AM	RBNU
ARN	7349	N	MOCH	07-KS-034	MOBL	2005	MBR	10:07 AM	BCCH
ARN	7349	N	MOCH	07-KS-034	MOBL	2005	MBR	10:07 AM	MOCH
ARN	7349	N	MOCH	07-KS-034	MOBL	2005	MBR	10:07 AM	RBNU
ARN	7349	N	MOCH	07-KS-034	MOBL	2005	MBR	10:07 AM	
IB	7360	N	MOCH	07-KS-041	EUST	2001	BMR	9:20 AM	BCCH
IB	7360	N	MOCH	07-KS-041	EUST	2001	BMR	9:20 AM	MOCH
IB	7360	N	MOCH	07-KS-041	EUST	2001	BMR	9:20 AM	MOCH
IB	7360	N	MOCH	07-KS-041	EUST	2001	BMR	9:20 AM	BCCH
IB	7360	N	MOCH	07-KS-041	EUST	2001	BMR	9:20 AM	MOCH
IB	7360	N	MOCH	07-KS-041	EUST	2001	BMR	9:20 AM	BCCH
IB	7360	N	MOCH	07-KS-041	EUST	2001	BMR	9:20 AM	MOCH

IB	7360	N	MOCH	07-KS-041	EUST	2001	BMR	9:20 AM	MOCH
IB	7360	N	MOCH	07-KS-041	EUST	2001	BMR	9:20 AM	MOCH
IB	7360	N	MOCH	07-KS-041	EUST	2001	BMR	9:20 AM	MOCH
IB	7360	N	MOCH	07-KS-041	EUST	2001	BMR	9:20 AM	BCCH
IB	7360	N	MOCH	07-KS-041	EUST	2001	BMR	9:20 AM	BCCH
IB	7360	N	MOCH	07-KS-041	EUST	2001	BMR	9:20 AM	MOCH
IB	7360	N	MOCH	07-KS-041	EUST	2001	BMR	9:20 AM	RBNU
HK	70225	N	MOCH	08-ARN-013			RBM	9:52	BCCH
HK	70225	N	MOCH	08-ARN-013			RBM	9:52	BCCH
HK	70225	N	MOCH	08-ARN-013			RBM	9:52	BCCH
HK	70225	N	MOCH	08-ARN-013			RBM	9:52	BCCH
HK	70225	N	MOCH	08-ARN-013			RBM	9:52	RBNU
HK	70225	N	MOCH	08-ARN-013			RBM	9:52	MOCH
HK	70225	N	MOCH	08-ARN-013			RBM	9:52	
HK	70225	N	MOCH	08-ARN-013			RBM	9:52	RBNU
HK	70225	N	MOCH	08-ARN-013			RBM	9:52	RBNU
HK	70225	N	MOCH	08-ARN-013			RBM	9:52	RBNU
HK	70225	N	MOCH	08-ARN-013			RBM	9:52	RBNU
HK	70225	N	MOCH	08-ARN-013			RBM	9:52	RBNU
HK	70225	N	MOCH	08-ARN-013			RBM	9:52	RBNU
HK	70225	N	MOCH	08-ARN-013			RBM	9:52	MOCH
HK	70225	N	MOCH	08-ARN-013			RBM	9:52	RBNU
HK	70225	N	MOCH	08-ARN-013			RBM	9:52	RBNU
HK	70225	N	MOCH	08-ARN-013			RBM	9:52	RBNU
HK	70225	N	MOCH	08-ARN-013			RBM	9:52	RBNU
HK	70225	N	MOCH	08-ARN-013			RBM	9:52	MOCH
HK	70225	N	MOCH	08-ARN-013			RBM	9:52	RBNU
HK	70225	N	MOCH	08-ARN-013			RBM	9:52	RBNU
HK	70225	N	MOCH	08-ARN-013			RBM	9:52	RBNU
HK	70225	N	MOCH	08-ARN-013			RBM	9:52	RBNU
HK	70225	N	MOCH	08-ARN-013			RBM	9:52	RBNU
HK	70225	N	MOCH	08-ARN-013			RBM	9:52	RBNU
HK	70225	N	MOCH	08-ARN-013			RBM	9:52	RBNU
HK	70225	N	MOCH	08-ARN-013			RBM	9:52	RBNU
HK	70225	N	MOCH	08-ARN-013			RBM	9:52	RBNU
HK	70225	N	MOCH	08-ARN-013			RBM	9:52	RBNU
HK	70225	N	MOCH	08-ARN-013			RBM	9:52	BCCH
HK	70225	N	MOCH	08-ARN-013			RBM	9:52	RBNU
HK	70225	N	MOCH	08-ARN-013			RBM	9:52	BCCH
HK	70225	N	MOCH	08-ARN-013			RBM	9:52	MOCH
HK	70225	N	MOCH	08-ARN-013			RBM	9:52	MOCH
HK	70225	N	MOCH	08-ARN-013			RBM	9:52	MOCH
HK	70225	N	MOCH	08-ARN-013			RBM	9:52	
AE	70717	N	MOCH	08-ARN-02	RNSA	2005	RMB	9:37	BCCH

AE	70717	N	MOCH	08-ARN-02	RNSA	2005	RMB	9:37	BCCH
AE	70717	N	MOCH	08-ARN-02	RNSA	2005	RMB	9:37	BCCH
AE	70717	N	MOCH	08-ARN-02	RNSA	2005	RMB	9:37	RBNU
AE	70717	N	MOCH	08-ARN-02	RNSA	2005	RMB	9:37	MOCH
AE	70717	N	MOCH	08-ARN-02	RNSA	2005	RMB	9:37	MOCH
AE	70717	N	MOCH	08-ARN-02	RNSA	2005	RMB	9:37	MOCH
AE	70717	N	MOCH	08-ARN-02	RNSA	2005	RMB	9:37	MOCH
AE	70717	N	MOCH	08-ARN-02	RNSA	2005	RMB	9:37	MOCH
AE	70717	N	MOCH	08-ARN-02	RNSA	2005	RMB	9:37	RBNU
AE	70717	N	MOCH	08-ARN-02	RNSA	2005	RMB	9:37	RBNU
AE	70717	N	MOCH	08-ARN-02	RNSA	2005	RMB	9:37	RBNU
AE	70717	N	MOCH	08-ARN-02	RNSA	2005	RMB	9:37	MOCH
AE	70717	N	MOCH	08-ARN-02	RNSA	2005	RMB	9:37	MOCH
AE	70717	N	MOCH	08-ARN-02	RNSA	2005	RMB	9:37	MOCH
AE	70717	N	MOCH	08-ARN-02	RNSA	2005	RMB	9:37	RBNU
AE	70717	N	MOCH	08-ARN-02	RNSA	2005	RMB	9:37	BCCH
AE	70717	N	MOCH	08-ARN-02	RNSA	2005	RMB	9:37	BCCH
AE	70717	N	MOCH	08-ARN-02	RNSA	2005	RMB	9:37	RBNU
AE	70717	N	MOCH	08-ARN-02	RNSA	2005	RMB	9:37	MOCH
AE	70717	N	MOCH	08-ARN-02	RNSA	2005	RMB	9:37	BCCH
AE	70717	N	MOCH	08-ARN-02	RNSA	2005	RMB	9:37	BCCH
AE	70717	N	MOCH	08-ARN-02	RNSA	2005	RMB	9:37	
HK	7839	N	MOCH	08-ARN-02	DOWO	2005	BRM	8:55	RBNU
HK	7839	N	MOCH	08-ARN-02	DOWO	2005	BRM	8:55	RBNU
HK	7839	N	MOCH	08-ARN-02	DOWO	2005	BRM	8:55	RBNU
HK	7839	N	MOCH	08-ARN-02	DOWO	2005	BRM	8:55	RBNU
HK	7839	N	MOCH	08-ARN-02	DOWO	2005	BRM	8:55	MOCH
HK	7839	N	MOCH	08-ARN-02	DOWO	2005	BRM	8:55	MOCH
HK	7839	N	MOCH	08-ARN-02	DOWO	2005	BRM	8:55	MOCH
HK	7839	N	MOCH	08-ARN-02	DOWO	2005	BRM	8:55	MOCH
HK	7839	N	MOCH	08-ARN-02	DOWO	2005	BRM	8:55	MOCH
HK	7839	N	MOCH	08-ARN-02	DOWO	2005	BRM	8:55	MOCH
HK	7839	N	MOCH	08-ARN-02	DOWO	2005	BRM	8:55	MOCH
HK	7839	N	MOCH	08-ARN-02	DOWO	2005	BRM	8:55	BCCH
HK	7839	N	MOCH	08-ARN-02	DOWO	2005	BRM	8:55	BCCH
HK	7839	N	MOCH	08-ARN-02	DOWO	2005	BRM	8:55	RBNU
HK	7839	N	MOCH	08-ARN-02	DOWO	2005	BRM	8:55	BCCH
HK	7839	N	MOCH	08-ARN-02	DOWO	2005	BRM	8:55	BCCH
HK	7839	N	MOCH	08-ARN-02	DOWO	2005	BRM	8:55	RBNU
HK	7839	N	MOCH	08-ARN-02	DOWO	2005	BRM	8:55	MOCH
HK	7839	N	MOCH	08-ARN-02	DOWO	2005	BRM	8:55	MOCH
HK	7839	N	MOCH	08-ARN-02	DOWO	2005	BRM	8:55	
HK	7837	N	MOCH	08-CA-027	TRES	2006	BRM	9:27	RBNU
HK	7837	N	MOCH	08-CA-027	TRES	2006	BRM	9:27	MOCH

HK	7837	N	MOCH	08-CA-027	TRES	2006	BRM	9:27	MOCH
HK	7837	N	MOCH	08-CA-027	TRES	2006	BRM	9:27	BCCH
HK	7837	N	MOCH	08-CA-027	TRES	2006	BRM	9:27	BCCH
HK	7837	N	MOCH	08-CA-027	TRES	2006	BRM	9:27	BCCH
HK	7837	N	MOCH	08-CA-027	TRES	2006	BRM	9:27	BCCH
HK	7837	N	MOCH	08-CA-027	TRES	2006	BRM	9:27	BCCH
HK	7837	N	MOCH	08-CA-027	TRES	2006	BRM	9:27	BCCH
HK	7837	N	MOCH	08-CA-027	TRES	2006	BRM	9:27	RBNU
HK	7837	N	MOCH	08-CA-027	TRES	2006	BRM	9:27	RBNU
HK	7837	N	MOCH	08-CA-027	TRES	2006	BRM	9:27	RBNU
HK	7837	N	MOCH	08-CA-027	TRES	2006	BRM	9:27	RBNU
HK	7837	N	MOCH	08-CA-027	TRES	2006	BRM	9:27	RBNU
HK	7837	N	MOCH	08-CA-027	TRES	2006	BRM	9:27	RBNU
HK	7837	N	MOCH	08-CA-027	TRES	2006	BRM	9:27	RBNU
HK	7837	N	MOCH	08-CA-027	TRES	2006	BRM	9:27	RBNU
HK	7837	N	MOCH	08-CA-027	TRES	2006	BRM	9:27	MOCH
HK	7837	N	MOCH	08-CA-027	TRES	2006	BRM	9:27	MOCH
HK	7837	N	MOCH	08-CA-027	TRES	2006	BRM	9:27	MOCH
HK	7837	N	MOCH	08-CA-027	TRES	2006	BRM	9:27	MOCH
HK	7837	N	MOCH	08-CA-027	TRES	2006	BRM	9:27	MOCH
HK	7837	N	MOCH	08-CA-027	TRES	2006	BRM	9:27	MOCH
HK	7837	N	MOCH	08-CA-027	TRES	2006	BRM	9:27	MOCH
HK	7837	N	MOCH	08-CA-027	TRES	2006	BRM	9:27	MOCH
HK	7837	N	MOCH	08-CA-027	TRES	2006	BRM	9:27	MOCH
HK	7837	N	MOCH	08-CA-027	TRES	2006	BRM	9:27	BCCH
HK	7837	N	MOCH	08-CA-027	TRES	2006	BRM	9:27	RBNU
HK	7837	N	MOCH	08-CA-027	TRES	2006	BRM	9:27	RBNU
HK	7837	N	MOCH	08-CA-027	TRES	2006	BRM	9:27	BCCH
HK	7837	N	MOCH	08-CA-027	TRES	2006	BRM	9:27	BCCH
HK	7837	N	MOCH	08-CA-027	TRES	2006	BRM	9:27	RBNU
HK	7837	N	MOCH	08-CA-027	TRES	2006	BRM	9:27	MOCH
HK	7837	N	MOCH	08-CA-027	TRES	2006	BRM	9:27	MOCH
HK	7837	N	MOCH	08-CA-027	TRES	2006	BRM	9:27	
HK	70717	N	MOCH	08-ARN-02	RNSA	2007	MBR	11:23	
HK	70717	N	MOCH	08-ARN-02	RNSA	2007	MBR	11:23	MOCH
HK	70717	N	MOCH	08-ARN-02	RNSA	2007	MBR	11:23	MOCH
HK	70717	N	MOCH	08-ARN-02	RNSA	2007	MBR	11:23	BCCH
HK	70717	N	MOCH	08-ARN-02	RNSA	2007	MBR	11:23	RBNU
HK	70717	N	MOCH	08-ARN-02	RNSA	2007	MBR	11:23	RBNU
HK	70717	N	MOCH	08-ARN-02	RNSA	2007	MBR	11:23	
	70140	N	RBNU	04-ARN-042			MBR	10:49	BCCH
	70140	N	RBNU	04-ARN-042			MBR	10:49	BCCH
	70140	N	RBNU	04-ARN-042			MBR	10:49	BCCH
	70140	N	RBNU	04-ARN-042			MBR	10:49	MOCH

70140	N	RBNU	04-ARN-042	MBR	10:49	RBNU
70140	N	RBNU	04-ARN-042	MBR	10:49	RBNU
70140	N	RBNU	04-ARN-042	MBR	10:49	MOCH
70140	N	RBNU	04-ARN-042	MBR	10:49	MOCH
70140	N	RBNU	04-ARN-042	MBR	10:49	MOCH
70140	N	RBNU	04-ARN-042	MBR	10:49	BCCH
70140	N	RBNU	04-ARN-042	MBR	10:49	BCCH
70140	N	RBNU	04-ARN-042	MBR	10:49	BCCH
70140	N	RBNU	04-ARN-042	MBR	10:49	BCCH
70140	N	RBNU	04-ARN-042	MBR	10:49	BCCH
70140	N	RBNU	04-ARN-042	MBR	10:49	RBNU
70140	N	RBNU	04-ARN-042	MBR	10:49	RBNU
70140	N	RBNU	04-ARN-042	MBR	10:49	BCCH
7579	N	RBNU	04-PAR-007	BRM	8:28:30	MOCH
7579	N	RBNU	04-PAR-007	BRM	8:28:30	none
7579	N	RBNU	04-PAR-007	BRM	8:28:30	RBNU
7579	N	RBNU	04-PAR-007	BRM	8:28:30	RBNU
7579	N	RBNU	04-PAR-007	BRM	8:28:30	RBNU
7579	N	RBNU	04-PAR-007	BRM	8:28:30	RBNU
7579	N	RBNU	04-PAR-007	BRM	8:28:30	RBNU
7579	N	RBNU	04-PAR-007	BRM	8:28:30	MOCH
7579	N	RBNU	04-PAR-007	BRM	8:28:30	MOCH
7579	N	RBNU	04-PAR-007	BRM	8:28:30	RBNU
7579	N	RBNU	04-PAR-007	BRM	8:28:30	RBNU
7579	N	RBNU	04-PAR-007	BRM	8:28:30	RBNU
7579	N	RBNU	04-PAR-007	BRM	8:28:30	MOCH
7579	N	RBNU	04-PAR-007	BRM	8:28:30	BCCH
70101	N	RBNU	04-PAR-078	BRM	7:35:20	BCCH
70101	N	RBNU	04-PAR-078	BRM	7:35:20	none
70101	N	RBNU	04-PAR-078	BRM	7:35:20	BCCH
70101	N	RBNU	04-PAR-078	BRM	7:35:20	BCCH
70101	N	RBNU	04-PAR-078	BRM	7:35:20	BCCH
70101	N	RBNU	04-PAR-078	BRM	7:35:20	none
70101	N	RBNU	04-PAR-078	BRM	7:35:20	none
70101	N	RBNU	04-PAR-078	RMB	10:12	MOCH
70101	N	RBNU	04-PAR-078	RMB	10:12	MOCH
70101	N	RBNU	04-PAR-078	RMB	10:12	MOCH
70101	N	RBNU	04-PAR-078	RMB	10:12	RBNU
70101	N	RBNU	04-PAR-078	RMB	10:12	none
70101	N	RBNU	04-PAR-078	RMB	10:12	RBNU
70101	N	RBNU	04-PAR-078	RMB	10:12	MOCH
70101	N	RBNU	04-PAR-078	RMB	10:12	MOCH
70101	N	RBNU	04-PAR-078	RMB	10:12	BCCH
70101	N	RBNU	04-PAR-078	RMB	10:12	BCCH

70101	N	RBNU	04-PAR-078			RMB	10:12	MOCH
70101	N	RBNU	04-PAR-078			RMB	10:12	MOCH
70101	N	RBNU	04-PAR-078			RMB	10:12	RBNU
70101	N	RBNU	04-PAR-078			RMB	10:12	RBNU
70415	N	RBNU	05-ARN-026			BMR	11:00	MOCH
70415	N	RBNU	05-ARN-026			BMR	11:00	MOCH
70415	N	RBNU	05-ARN-026			BMR	11:00	MOCH
70415	N	RBNU	05-ARN-026			BMR	11:00	MOCH
70415	N	RBNU	05-ARN-026			BMR	11:00	RBNU
70415	N	RBNU	05-ARN-026			BMR	11:00	BCCH
70415	N	RBNU	05-ARN-026			BMR	11:00	MOCH
70415	N	RBNU	05-ARN-026			BMR	11:00	MOCH
70415	N	RBNU	05-ARN-026			BMR	11:00	RBNU
70415	N	RBNU	05-ARN-026			BMR	11:00	none
70415	N	RBNU	05-ARN-026			BMR	11:00	MOCH
70415	N	RBNU	05-ARN-026			BMR	11:00	MOCH
70415	N	RBNU	05-ARN-026			BMR	11:00	RBNU
70415	N	RBNU	05-ARN-026			BMR	11:00	RBNU
70415	N	RBNU	05-ARN-026			BMR	11:00	RBNU
70415	N	RBNU	05-ARN-026			BMR	11:00	MOCH
70415	N	RBNU	05-ARN-026			BMR	11:00	MOCH
70415	N	RBNU	05-ARN-026			BMR	11:00	none
70415	N	RBNU	05-ARN-026			BMR	11:00	RBNU
70415	N	RBNU	05-ARN-026			BMR	11:00	MOCH
70415	N	RBNU	05-ARN-026			BMR	11:00	MOCH
70415	N	RBNU	05-ARN-026			BMR	11:00	MOCH
70425	N	RBNU	05-MN-019	-	-	BMR	8:46	MOCH
70425	N	RBNU	05-MN-019	-	-	BMR	8:46	MOCH
70425	N	RBNU	05-MN-019	-	-	BMR	8:46	MOCH
70425	N	RBNU	05-MN-019	-	-	BMR	8:46	none
70425	N	RBNU	05-MN-019	-	-	BMR	8:46	MOCH
70425	N	RBNU	05-MN-019	-	-	BMR	8:46	RBNU
70425	N	RBNU	05-MN-019	-	-	BMR	8:46	RBNU
70425	N	RBNU	05-MN-019	-	-	BMR	8:46	BCCH
70425	N	RBNU	05-MN-019	-	-	BMR	8:46	BCCH
70425	N	RBNU	05-MN-019	-	-	BMR	8:46	BCCH
70425	N	RBNU	05-MN-019	-	-	BMR	8:46	BCCH
70425	N	RBNU	05-MN-019	-	-	BMR	8:46	BCCH
70425	N	RBNU	05-MN-019	-	-	BMR	8:46	MOCH
70425	N	RBNU	05-MN-019	-	-	BMR	8:46	RBNU
70425	N	RBNU	05-MN-019	-	-	BMR	8:46	none
70425	N	RBNU	05-MN-019	-	-	BMR	8:46	none
70425	N	RBNU	05-MN-019	-	-	BMR	8:46	BCCH
70425	N	RBNU	05-MN-019	-	-	BMR	8:46	MOCH

70425	N	RBNU	05-MN-019	-	-	BMR	8:46	MOCH
7457	N	RBNU	05-CW-025			MBR	7:12	RBNU
7457	N	RBNU	05-CW-025			MBR	7:12	RBNU
7457	N	RBNU	05-CW-025			MBR	7:12	RBNU
7457	N	RBNU	05-CW-025			MBR	7:12	BCCH
7457	N	RBNU	05-CW-025			MBR	7:12	BCCH
7457	N	RBNU	05-CW-025			MBR	7:12	BCCH
7457	N	RBNU	05-CW-025			MBR	7:12	BCCH
7457	N	RBNU	05-CW-025			MBR	7:12	BCCH
7457	N	RBNU	05-CW-025			MBR	7:12	MOCH
7457	N	RBNU	05-CW-025			MBR	7:12	RBNU
7457	N	RBNU	05-CW-025			MBR	7:12	RBNU
7457	N	RBNU	05-CW-025			MBR	7:12	MOCH
NEW	N	RBNU	05-ARN-026			BRM	8:36	RBNU
NEW	N	RBNU	05-ARN-026			BRM	8:36	RBNU
NEW	N	RBNU	05-ARN-026			BRM	8:36	BCCH
NEW	N	RBNU	05-ARN-026			BRM	8:36	none
NEW	N	RBNU	05-ARN-026			BRM	8:36	BCCH
NEW	N	RBNU	05-ARN-026			BRM	8:36	RBNU
NEW	N	RBNU	05-ARN-026			BRM	8:36	RBNU
NEW	N	RBNU	05-ARN-026			BRM	8:36	RBNU
NEW	N	RBNU	05-ARN-026			BRM	8:36	BCCH
NEW	N	RBNU	05-ARN-026			BRM	8:36	MOCH
NEW	N	RBNU	05-ARN-026			BRM	8:36	RBNU
NEW	N	RBNU	05-ARN-026			BRM	8:36	MOCH
NEW	N	RBNU	05-ARN-026			BRM	8:36	none
NEW	N	RBNU	05-ARN-026			BRM	8:36	BCCH
NEW	N	RBNU	05-ARN-026			BRM	8:36	MOCH
NEW	N	RBNU	05-ARN-026			BRM	8:36	none
NEW	N	RBNU	05-ARN-026			BRM	9:09	RBNU
NEW	N	RBNU	05-ARN-026			BRM	9:09	BCCH
NEW	N	RBNU	05-ARN-026			BRM	9:09	none
NEW	N	RBNU	05-ARN-026			BRM	9:09	RBNU
NEW	N	RBNU	05-ARN-026			BRM	9:09	RBNU
NEW	N	RBNU	05-ARN-026			BRM	9:09	MOCH
NEW	N	RBNU	05-ARN-026			BRM	9:09	MOCH
70406	N	RBNU	05-PAR-021			BMR	10:09	RBNU
70406	N	RBNU	05-PAR-021			BMR	10:09	RBNU
70406	N	RBNU	05-PAR-021			BMR	10:09	RBNU
70406	N	RBNU	05-PAR-021			BMR	10:09	RBNU
70406	N	RBNU	05-PAR-021			BMR	10:09	MOCH
70406	N	RBNU	05-PAR-021			BMR	10:09	RBNU
70406	N	RBNU	05-PAR-021			BMR	10:09	BCCH
70406	N	RBNU	05-PAR-021			BMR	10:09	MOCH

70406	N	RBNU	05-PAR-021			BMR	10:09	BCCH
70406	N	RBNU	05-PAR-021			BMR	10:09	BCCH
70406	N	RBNU	05-PAR-021			BMR	10:09	BCCH
70406	N	RBNU	05-PAR-021			BMR	10:09	MOCH
70406	N	RBNU	05-PAR-021			BMR	10:09	MOCH
70406	N	RBNU	05-PAR-021			BMR	10:09	BCCH
70406	N	RBNU	05-PAR-021			BMR	10:09	BCCH
70406	N	RBNU	05-PAR-021			BMR	10:09	BCCH
70406	N	RBNU	05-PAR-021			BMR	10:09	none
70407	N	RBNU	05-PAR-06	none	none	RBM	15:02	MOCH
70407	N	RBNU	05-PAR-06	none	none	RBM	15:02	MOCH
70407	N	RBNU	05-PAR-06	none	none	RBM	15:02	MOCH
70407	N	RBNU	05-PAR-06	none	none	RBM	15:02	none
70407	N	RBNU	05-PAR-06	none	none	RBM	15:02	MOCH
70407	N	RBNU	05-PAR-06	none	none	RBM	15:02	MOCH
70407	N	RBNU	05-PAR-06	none	none	RBM	15:02	MOCH
70407	N	RBNU	05-PAR-06	none	none	RBM	15:02	none
70407	N	RBNU	05-PAR-06	none	none	RBM	15:02	MOCH
70407	N	RBNU	05-PAR-06	none	none	RBM	15:02	RBNU
70407	N	RBNU	05-PAR-06	none	none	RBM	15:02	RBNU
70407	N	RBNU	05-PAR-06	none	none	RBM	15:02	RBNU
70407	N	RBNU	05-PAR-06	none	none	RBM	15:02	RBNU
70407	N	RBNU	05-PAR-06	none	none	RBM	15:02	RBNU
70407	N	RBNU	05-PAR-06	none	none	RBM	15:02	RBNU
70407	N	RBNU	05-PAR-06	none	none	RBM	15:02	RBNU
70407	N	RBNU	05-PAR-06	none	none	RBM	15:02	BCCH
70407	N	RBNU	05-PAR-06	none	none	RBM	15:02	BCCH
70407	N	RBNU	05-PAR-06	none	none	RBM	15:02	BCCH
70407	N	RBNU	05-PAR-06	none	none	RBM	15:02	BCCH
70407	N	RBNU	05-PAR-06	none	none	RBM	15:02	RBNU
70407	N	RBNU	05-PAR-06	none	none	RBM	15:02	RBNU
70407	N	RBNU	05-PAR-06	none	none	RBM	15:02	RBNU
70407	N	RBNU	05-PAR-06	none	none	RBM	15:02	MOCH
70179	N	RBNU	06-KS-024			BRM	9:46	BCCH
70179	N	RBNU	06-KS-024			BRM	9:46	RBNU
70179	N	RBNU	06-KS-024			BRM	9:46	MOCH
70179	N	RBNU	06-KS-024			BRM	9:46	BCCH
70179	N	RBNU	06-KS-024			BRM	9:46	BCCH
70179	N	RBNU	06-KS-024			BRM	9:46	RBNU
70179	N	RBNU	06-KS-024			BRM	9:46	MOCH
70198	N	RBNU	06-SLD-049			RMB	10:09	RBNU
70198	N	RBNU	06-SLD-049			RMB	10:09	BCCH
70198	N	RBNU	06-SLD-049			RMB	10:09	RBNU
70198	N	RBNU	06-SLD-049			RMB	10:09	none

70198	N	RBNU	06-SLD-049			RMB	10:09	RBNU
70198	N	RBNU	06-SLD-049			RMB	10:09	MOCH
70198	N	RBNU	06-SLD-049			RMB	10:09	MOCH
70198	N	RBNU	06-SLD-049			RMB	10:09	MOCH
70198	N	RBNU	06-SLD-049			RMB	10:09	RBNU
70198	N	RBNU	06-SLD-049			RMB	10:09	MOCH
70198	N	RBNU	06-SLD-049			RMB	10:09	BCCH
70005	N	RBNU	06-ARN-009			RBM	10:07	MOCH
70005	N	RBNU	06-ARN-009			RBM	10:07	BCCH
70005	N	RBNU	06-ARN-009			RBM	10:07	BCCH
70005	N	RBNU	06-ARN-009			RBM	10:07	MOCH
70005	N	RBNU	06-ARN-009			RBM	10:07	MOCH
70005	N	RBNU	06-ARN-009			RBM	10:07	MOCH
70005	N	RBNU	06-ARN-009			RBM	10:07	MOCH
70005	N	RBNU	06-ARN-009			RBM	10:07	RBNU
70005	N	RBNU	06-ARN-009			RBM	10:07	
70005	N	RBNU	06-ARN-009			RBM	10:07	
70005	N	RBNU	06-ARN-009			RBM	10:07	RBNU
70005	N	RBNU	06-ARN-009			RBM	10:07	BCCH
70005	N	RBNU	06-ARN-009			RBM	10:07	BCCH
70005	N	RBNU	06-ARN-009			RBM	10:07	MOCH
70005	N	RBNU	06-ARN-009			RBM	10:07	BCCH
70005	N	RBNU	06-ARN-009			RBM	10:07	MOCH
70005	N	RBNU	06-ARN-009			RBM	10:07	RBNU
70005	N	RBNU	06-ARN-009			RBM	10:07	
7028	N	RBNU	06-ARN-014			RMB	9:18	BCCH
7028	N	RBNU	06-ARN-014			RMB	9:18	MOCH
7028	N	RBNU	06-ARN-014			RMB	9:18	BCCH
7028	N	RBNU	06-ARN-014			RMB	9:18	MOCH
7028	N	RBNU	06-ARN-014			RMB	9:18	MOCH
7028	N	RBNU	06-ARN-014			RMB	9:18	MOCH
7028	N	RBNU	06-ARN-014			RMB	9:18	RBNU
7028	N	RBNU	06-ARN-014			RMB	9:18	RBNU
7028	N	RBNU	06-ARN-014			RMB	9:18	BCCH
7028	N	RBNU	06-ARN-014			RMB	9:18	BCCH
7028	N	RBNU	06-ARN-014			RMB	9:18	MOCH
7028	N	RBNU	06-ARN-014			RMB	9:18	MOCH
7028	N	RBNU	06-ARN-014			RMB	9:18	BCCH
7028	N	RBNU	06-ARN-014			RMB	9:18	MOCH
7028	N	RBNU	06-ARN-014			RMB	9:18	RBNU
7028	N	RBNU	06-ARN-014			RMB	9:18	
70770	N	RBNU	06-ARN-02	DOWO	2005	RMB	10:07	BCCH
70770	N	RBNU	06-ARN-02	DOWO	2005	RMB	10:07	BCCH
70770	N	RBNU	06-ARN-02	DOWO	2005	RMB	10:07	MOCH

	70770	N	RBNU	06-ARN-02	DOWO	2005	RMB	10:07	MOCH
	70770	N	RBNU	06-ARN-02	DOWO	2005	RMB	10:07	BCCH
	70770	N	RBNU	06-ARN-02	DOWO	2005	RMB	10:07	BCCH
	70770	N	RBNU	06-ARN-02	DOWO	2005	RMB	10:07	RBNU
	70770	N	RBNU	06-ARN-02	DOWO	2005	RMB	10:07	RBNU
	70770	N	RBNU	06-ARN-02	DOWO	2005	RMB	10:07	BCCH
	70770	N	RBNU	06-ARN-02	DOWO	2005	RMB	10:07	MOCH
	70770	N	RBNU	06-ARN-02	DOWO	2005	RMB	10:07	RBNU
	70770	N	RBNU	06-ARN-02	DOWO	2005	RMB	10:07	
	70198	N	RBNU	06-SLD-049			RMB	8:50	BCCH
	70198	N	RBNU	06-SLD-049			RMB	8:50	MOCH
	70198	N	RBNU	06-SLD-049			RMB	8:50	RBNU
	70198	N	RBNU	06-SLD-049			RMB	8:50	RBNU
	70198	N	RBNU	06-SLD-049			RMB	8:50	MOCH
	70198	N	RBNU	06-SLD-049			RMB	8:50	BCCH
	70198	N	RBNU	06-SLD-049			RMB	8:50	BCCH
	70198	N	RBNU	06-SLD-049			RMB	8:50	RBNU
	70198	N	RBNU	06-SLD-049			RMB	8:50	BCCH
	70198	N	RBNU	06-SLD-049			RMB	8:50	MOCH
	70198	N	RBNU	06-SLD-049			RMB	8:50	RBNU
	70198	N	RBNU	06-SLD-049			RMB	8:50	
HK	70210	N	RBNU	08-CA-042			MRB	9:44	BCCH
HK	70210	N	RBNU	08-CA-042			MRB	9:44	BCCH
HK	70210	N	RBNU	08-CA-042			MRB	9:44	BCCH
HK	70210	N	RBNU	08-CA-042			MRB	9:44	RBNU
HK	70210	N	RBNU	08-CA-042			MRB	9:44	RBNU
HK	70210	N	RBNU	08-CA-042			MRB	9:44	RBNU
HK	70210	N	RBNU	08-CA-042			MRB	9:44	MOCH
HK	70210	N	RBNU	08-CA-042			MRB	9:44	MOCH
HK	70210	N	RBNU	08-CA-042			MRB	9:44	MOCH
HK	70210	N	RBNU	08-CA-042			MRB	9:44	RBNU
HK	70210	N	RBNU	08-CA-042			MRB	9:44	BCCH
HK	70210	N	RBNU	08-CA-042			MRB	9:44	
HK	70210	N	RBNU	08-CA-042			MRB	9:44	MOCH
HK	70210	N	RBNU	08-CA-042			MRB	9:44	RBNU
HK	70210	N	RBNU	08-CA-042			MRB	9:44	BCCH
HK	70210	N	RBNU	08-CA-042			MRB	9:44	BCCH
HK	70210	N	RBNU	08-CA-042			MRB	9:44	
HK	70223	N	RBNU	08-ARN-012			RBM	9:22	MOCH
HK	70223	N	RBNU	08-ARN-012			RBM	9:22	BCCH
HK	70223	N	RBNU	08-ARN-012			RBM	9:22	MOCH
HK	70223	N	RBNU	08-ARN-012			RBM	9:22	MOCH
HK	70223	N	RBNU	08-ARN-012			RBM	9:22	RBNU
HK	70223	N	RBNU	08-ARN-012			RBM	9:22	MOCH

HK	70223	N	RBNU	08-ARN-012	RBM	9:22 RBNU
HK	70223	N	RBNU	08-ARN-012	RBM	9:22 RBNU
HK	70223	N	RBNU	08-ARN-012	RBM	9:22 BCCH
HK	70223	N	RBNU	08-ARN-012	RBM	9:22 BCCH
HK	70223	N	RBNU	08-ARN-012	RBM	9:22 BCCH
HK	70223	N	RBNU	08-ARN-012	RBM	9:22 BCCH
HK	70223	N	RBNU	08-ARN-012	RBM	9:22 MOCH
HK	70223	N	RBNU	08-ARN-012	RBM	9:22 MOCH
HK	70223	N	RBNU	08-ARN-012	RBM	9:22 RBNU
HK	70223	N	RBNU	08-ARN-012	RBM	9:22 RBNU
HK	70223	N	RBNU	08-ARN-012	RBM	9:22 BCCH
HK	70223	N	RBNU	08-ARN-012	RBM	9:22 MOCH
HK	70223	N	RBNU	08-ARN-012	RBM	9:22 MOCH
HK	70223	N	RBNU	08-ARN-012	RBM	9:22 MOCH
HK	70223	N	RBNU	08-ARN-012	RBM	9:22
HK	70525	N	RBNU	08-ARN-037	MBR	8:28 BCCH
HK	70525	N	RBNU	08-ARN-037	MBR	8:28 BCCH
HK	70525	N	RBNU	08-ARN-037	MBR	8:28 BCCH
HK	70525	N	RBNU	08-ARN-037	MBR	8:28 BCCH
HK	70525	N	RBNU	08-ARN-037	MBR	8:28 MOCH
HK	70525	N	RBNU	08-ARN-037	MBR	8:28 MOCH
HK	70525	N	RBNU	08-ARN-037	MBR	8:28 MOCH
HK	70525	N	RBNU	08-ARN-037	MBR	8:28 BCCH
HK	70525	N	RBNU	08-ARN-037	MBR	8:28 MOCH
HK	70525	N	RBNU	08-ARN-037	MBR	8:28 MOCH
HK	70525	N	RBNU	08-ARN-037	MBR	8:28 MOCH
HK	70525	N	RBNU	08-ARN-037	MBR	8:28 BCCH
HK	70525	N	RBNU	08-ARN-037	MBR	8:28 BCCH
HK	70525	N	RBNU	08-ARN-037	MBR	8:28 MOCH
HK	70525	N	RBNU	08-ARN-037	MBR	8:28 BCCH
HK	70525	N	RBNU	08-ARN-037	MBR	8:28 BCCH
HK	70525	N	RBNU	08-ARN-037	MBR	8:28 RBNU
HK	70525	N	RBNU	08-ARN-037	MBR	8:28 RBNU
HK	70525	N	RBNU	08-ARN-037	MBR	8:28 RBNU
HK	70525	N	RBNU	08-ARN-037	MBR	8:28 RBNU
HK	70525	N	RBNU	08-ARN-037	MBR	8:28 RBNU
HK	70525	N	RBNU	08-ARN-037	MBR	8:28 RBNU
HK	70525	N	RBNU	08-ARN-037	MBR	8:28 BCCH
HK	70525	N	RBNU	08-ARN-037	MBR	8:28 BCCH
HK	70525	N	RBNU	08-ARN-037	MBR	8:28 BCCH
HK	70525	N	RBNU	08-ARN-037	MBR	8:28 RBNU
HK	70525	N	RBNU	08-ARN-037	MBR	8:28 RBNU
HK	70525	N	RBNU	08-ARN-037	MBR	8:28 RBNU
HK	70525	N	RBNU	08-ARN-037	MBR	8:28 RBNU

HK	70525	N	RBNU	08-ARN-037	MBR	8:28	RBNU
HK	70525	N	RBNU	08-ARN-037	MBR	8:28	RBNU
HK	70525	N	RBNU	08-ARN-037	MBR	8:28	RBNU
HK	70525	N	RBNU	08-ARN-037	MBR	8:28	RBNU
HK	70525	N	RBNU	08-ARN-037	MBR	8:28	RBNU
HK	70525	N	RBNU	08-ARN-037	MBR	8:28	RBNU
HK	70525	N	RBNU	08-ARN-037	MBR	8:28	RBNU
HK	70525	N	RBNU	08-ARN-037	MBR	8:28	RBNU
HK	70525	N	RBNU	08-ARN-037	MBR	8:28	RBNU
HK	70525	N	RBNU	08-ARN-037	MBR	8:28	MOCH
HK	70525	N	RBNU	08-ARN-037	MBR	8:28	MOCH
HK	70525	N	RBNU	08-ARN-037	MBR	8:28	BCCH
HK	70525	N	RBNU	08-ARN-037	MBR	8:28	RBNU
HK	70525	N	RBNU	08-ARN-037	MBR	8:28	RBNU
HK	70525	N	RBNU	08-ARN-037	MBR	8:28	
HK	70223	N	RBNU	08-ARN-012	MBR	9:34	BCCH
HK	70223	N	RBNU	08-ARN-012	MBR	9:34	BCCH
HK	70223	N	RBNU	08-ARN-012	MBR	9:34	RBNU
HK	70223	N	RBNU	08-ARN-012	MBR	9:34	
HK	70223	N	RBNU	08-ARN-012	MBR	9:34	MOCH
HK	70223	N	RBNU	08-ARN-012	MBR	9:34	MOCH
HK	70223	N	RBNU	08-ARN-012	MBR	9:34	RBNU
HK	70223	N	RBNU	08-ARN-012	MBR	9:34	RBNU
HK	70223	N	RBNU	08-ARN-012	MBR	9:34	RBNU
HK	70223	N	RBNU	08-ARN-012	MBR	9:34	RBNU
HK	70223	N	RBNU	08-ARN-012	MBR	9:34	RBNU
HK	70223	N	RBNU	08-ARN-012	MBR	9:34	RBNU
HK	70223	N	RBNU	08-ARN-012	MBR	9:34	RBNU
HK	70223	N	RBNU	08-ARN-012	MBR	9:34	
HK	70223	N	RBNU	08-ARN-012	MBR	9:34	MOCH
HK	70223	N	RBNU	08-ARN-012	MBR	9:34	RBNU
HK	70223	N	RBNU	08-ARN-012	MBR	9:34	MOCH
HK	70223	N	RBNU	08-ARN-012	MBR	9:34	BCCH
HK	70223	N	RBNU	08-ARN-012	MBR	9:34	RBNU
HK	70223	N	RBNU	08-ARN-012	MBR	9:34	RBNU
HK	70223	N	RBNU	08-ARN-012	MBR	9:34	
HK	70478	N	RBNU	08-ARN-039	RBM	10:24	RBNU
HK	70478	N	RBNU	08-ARN-039	RBM	10:24	RBNU
HK	70478	N	RBNU	08-ARN-039	RBM	10:24	RBNU
HK	70478	N	RBNU	08-ARN-039	RBM	10:24	RBNU
HK	70478	N	RBNU	08-ARN-039	RBM	10:24	RBNU
HK	70478	N	RBNU	08-ARN-039	RBM	10:24	RBNU
HK	70478	N	RBNU	08-ARN-039	RBM	10:24	RBNU
HK	70478	N	RBNU	08-ARN-039	RBM	10:24	MOCH

HK	70478	N	RBNU	08-ARN-039		RBM	10:24	RBNU
HK	70478	N	RBNU	08-ARN-039		RBM	10:24	MOCH
HK	70478	N	RBNU	08-ARN-039		RBM	10:24	RBNU
HK	70478	N	RBNU	08-ARN-039		RBM	10:24	BCCH
HK	70478	N	RBNU	08-ARN-039		RBM	10:24	MOCH
HK	70478	N	RBNU	08-ARN-039		RBM	10:24	MOCH
HK	70478	N	RBNU	08-ARN-039		RBM	10:24	
HK	70545	N	RBNU	08-ARN-037		MBR	10:51	BCCH
HK	70545	N	RBNU	08-ARN-037		MBR	10:51	BCCH
HK	70545	N	RBNU	08-ARN-037		MBR	10:51	BCCH
HK	70545	N	RBNU	08-ARN-037		MBR	10:51	BCCH
HK	70545	N	RBNU	08-ARN-037		MBR	10:51	BCCH
HK	70545	N	RBNU	08-ARN-037		MBR	10:51	RBNU
HK	70545	N	RBNU	08-ARN-037		MBR	10:51	RBNU
HK	70545	N	RBNU	08-ARN-037		MBR	10:51	RBNU
HK	70545	N	RBNU	08-ARN-037		MBR	10:51	RBNU
HK	70545	N	RBNU	08-ARN-037		MBR	10:51	RBNU
HK	70545	N	RBNU	08-ARN-037		MBR	10:51	RBNU
HK	70545	N	RBNU	08-ARN-037		MBR	10:51	RBNU
HK	70545	N	RBNU	08-ARN-037		MBR	10:51	RBNU
HK	70545	N	RBNU	08-ARN-037		MBR	10:51	RBNU
HK	70545	N	RBNU	08-ARN-037		MBR	10:51	RBNU
HK	70545	N	RBNU	08-ARN-037		MBR	10:51	RBNU
HK	70545	N	RBNU	08-ARN-037		MBR	10:51	RBNU
HK	70545	N	RBNU	08-ARN-037		MBR	10:51	RBNU
HK	70545	N	RBNU	08-ARN-037		MBR	10:51	MOCH
HK	70545	N	RBNU	08-ARN-037		MBR	10:51	BCCH
HK	70545	N	RBNU	08-ARN-037		MBR	10:51	RBNU
HK	70545	N	RBNU	08-ARN-037		MBR	10:51	RBNU
HK	70545	N	RBNU	08-ARN-037		MBR	10:51	
IB	7403	N	RBNU	07-ARN-01 TAHU	2005	BRM	9:21 AM	BCCH
IB	7403	N	RBNU	07-ARN-01 TAHU	2005	BRM	9:21 AM	RBNU
IB	7403	N	RBNU	07-ARN-01 TAHU	2005	BRM	9:21 AM	RBNU
IB	7403	N	RBNU	07-ARN-01 TAHU	2005	BRM	9:21 AM	RBNU
IB	7403	N	RBNU	07-ARN-01 TAHU	2005	BRM	9:21 AM	RBNU
IB	7403	N	RBNU	07-ARN-01 TAHU	2005	BRM	9:21 AM	BCCH
IB	7403	N	RBNU	07-ARN-01 TAHU	2005	BRM	9:21 AM	BCCH
IB	7403	N	RBNU	07-ARN-01 TAHU	2005	BRM	9:21 AM	MOCH
IB	7403	N	RBNU	07-ARN-01 TAHU	2005	BRM	9:21 AM	RBNU
IB	7403	N	RBNU	07-ARN-01 TAHU	2005	BRM	9:21 AM	MOCH
IB	7403	N	RBNU	07-ARN-01 TAHU	2005	BRM	9:21 AM	MOCH
IB	7403	N	RBNU	07-ARN-01 TAHU	2005	BRM	9:21 AM	MOCH
IB	7403	N	RBNU	07-ARN-01 TAHU	2005	BRM	9:21 AM	MOCH
IB	7403	N	RBNU	07-ARN-01 TAHU	2005	BRM	9:21 AM	BCCH

IB	7403	N	RBNU	07-ARN-01	TAHU	2005	BRM	9:21 AM	MOCH
IB	7403	N	RBNU	07-ARN-01	TAHU	2005	BRM	9:21 AM	RBNU
IB	70594	O	DOWO	07-TD-019	RBNU	2006	BMR	9:09	BCCH
IB	70594	O	DOWO	07-TD-019	RBNU	2006	BMR	9:09	BCCH
IB	70594	O	DOWO	07-TD-019	RBNU	2006	BMR	9:09	BCCH
IB	70594	O	DOWO	07-TD-019	RBNU	2006	BMR	9:09	BCCH
IB	70594	O	DOWO	07-TD-019	RBNU	2006	BMR	9:09	BCCH
IB	70594	O	DOWO	07-TD-019	RBNU	2006	BMR	9:09	BCCH
IB	70594	O	DOWO	07-TD-019	RBNU	2006	BMR	9:09	BCCH
IB	70594	O	DOWO	07-TD-019	RBNU	2006	BMR	9:09	BCCH
IB	70594	O	DOWO	07-TD-019	RBNU	2006	BMR	9:09	BCCH
IB	70594	O	DOWO	07-TD-019	RBNU	2006	BMR	9:09	BCCH
IB	70594	O	DOWO	07-TD-019	RBNU	2006	BMR	9:09	BCCH
IB	70594	O	DOWO	07-TD-019	RBNU	2006	BMR	9:09	RBNU
IB	70594	O	DOWO	07-TD-019	RBNU	2006	BMR	9:09	RBNU
IB	70594	O	DOWO	07-TD-019	RBNU	2006	BMR	9:09	RBNU
IB	70594	O	DOWO	07-TD-019	RBNU	2006	BMR	9:09	MOCH
IB	70594	O	DOWO	07-TD-019	RBNU	2006	BMR	9:09	MOCH
IB	70594	O	DOWO	07-TD-019	RBNU	2006	BMR	9:09	MOCH
IB	70594	O	DOWO	07-TD-019	RBNU	2006	BMR	9:09	MOCH
IB	70594	O	DOWO	07-TD-019	RBNU	2006	BMR	9:09	MOCH
IB	70594	O	DOWO	07-TD-019	RBNU	2006	BMR	9:09	MOCH
IB	70594	O	DOWO	07-TD-019	RBNU	2006	BMR	9:09	MOCH
IB	70594	O	DOWO	07-TD-019	RBNU	2006	BMR	9:09	MOCH
IB	70594	O	DOWO	07-TD-019	RBNU	2006	BMR	9:09	MOCH
IB	70594	O	DOWO	07-TD-019	RBNU	2006	BMR	9:09	MOCH
IB	70594	O	DOWO	07-TD-019	RBNU	2006	BMR	9:09	MOCH
IB	70594	O	DOWO	07-TD-019	RBNU	2006	BMR	9:09	MOCH
IB	70594	O	DOWO	07-TD-019	RBNU	2006	BMR	9:09	MOCH
IB	70594	O	DOWO	07-TD-019	RBNU	2006	BMR	9:09	MOCH
IB	70594	O	DOWO	07-TD-019	RBNU	2006	BMR	9:09	RBNU
IB	70594	O	DOWO	07-TD-019	RBNU	2006	BMR	9:09	RBNU
IB	70594	O	DOWO	07-TD-019	RBNU	2006	BMR	9:09	RBNU
IB	70594	O	DOWO	07-TD-019	RBNU	2006	BMR	9:09	RBNU
IB	70594	O	DOWO	07-TD-019	RBNU	2006	BMR	9:09	RBNU
IB	70594	O	DOWO	07-TD-019	RBNU	2006	BMR	9:09	RBNU
IB	70594	O	DOWO	07-TD-019	RBNU	2006	BMR	9:09	RBNU
IB	70594	O	DOWO	07-TD-019	RBNU	2006	BMR	9:09	RBNU
IB	70594	O	DOWO	07-TD-019	RBNU	2006	BMR	9:09	RBNU
IB	70594	O	DOWO	07-TD-019	RBNU	2006	BMR	9:09	RBNU
IB	70594	O	DOWO	07-TD-019	RBNU	2006	BMR	9:09	RBNU
IB	70594	O	DOWO	07-TD-019	RBNU	2006	BMR	9:09	RBNU
IB	70594	O	DOWO	07-TD-019	RBNU	2006	BMR	9:09	RBNU
IB	70594	O	DOWO	07-TD-019	RBNU	2006	BMR	9:09	RBNU

IB	70594	O	DOWO	07-TD-019	RBNU	2006	BMR	9:09	MOCH
IB	70594	O	DOWO	07-TD-019	RBNU	2006	BMR	9:09	BCCH
IB	70594	O	DOWO	07-TD-019	RBNU	2006	BMR	9:09	MOCH
IB	70594	O	DOWO	07-TD-019	RBNU	2006	BMR	9:09	RBNU
	70704	O			RBNU	2004	BMR	12:52	MOCH
	70704	O			RBNU	2004	BMR	12:52	MOCH
	70704	O			RBNU	2004	BMR	12:52	MOCH
	70704	O			RBNU	2004	BMR	12:52	none
	70704	O			RBNU	2004	BMR	12:52	BCCH
	70704	O			RBNU	2004	BMR	12:52	RBNU
	70704	O			RBNU	2004	BMR	12:52	RBNU
	70704	O			RBNU	2004	BMR	12:52	RBNU
	70704	O			RBNU	2004	BMR	12:52	RBNU
	70704	O			RBNU	2004	BMR	12:52	RBNU
	70704	O			RBNU	2004	BMR	12:52	RBNU
	7952	O			MOCH	2003	RBM	10:25	RBNU
	7952	O			MOCH	2003	RBM	10:25	RBNU
	7952	O			MOCH	2003	RBM	10:25	RBNU
	7952	O			MOCH	2003	RBM	10:25	none
	7952	O			MOCH	2003	RBM	10:25	RBNU
	7952	O			MOCH	2003	RBM	10:25	RBNU
	7952	O			MOCH	2003	RBM	10:25	BCCH
	7952	O			MOCH	2003	RBM	10:25	BCCH
	7952	O			MOCH	2003	RBM	10:25	MOCH
	7952	O			MOCH	2003	R	7:53	RBNU
	7952	O			MOCH	2003	R	7:53	RBNU
	7952	O			MOCH	2003	R	7:53	RBNU
	7952	O			MOCH	2003	R	7:53	RBNU
	7849	O			RBNU	2003	R	14:22:10	RBNU
	7950	O			RBNU	2003	R	15:00:12	RBNU
	7950	O			RBNU	2003	R	15:00:12	RBNU
	7950	O			RBNU	2003	R	15:00:12	RBNU
	7950	O			RBNU	2003	R	15:00:12	RBNU
	7950	O			RBNU	2003	R	15:00:12	RBNU
	7950	O			RBNU	2003	R	15:00:12	RBNU
	7950	O			RBNU	2003	R	15:00:12	RBNU
	7950	O			RBNU	2003	R	15:00:12	RBNU
	7950	O			RBNU	2003	R	15:00:12	RBNU
	7564	O			RBNU	2003	MR	13:33:25	MOCH
	7564	O			RBNU	2003	MR	13:33:25	MOCH
	7564	O			RBNU	2003	MR	13:33:25	RBNU
	7564	O			RBNU	2003	BMR	9:15:00	BCCH
	7564	O			RBNU	2003	BMR	9:15:00	MOCH
	7564	O			RBNU	2003	BMR	9:15:00	MOCH

7676	O	MOCH	2003 RBM	9:50	RBNU
7676	O	MOCH	2003 RBM	9:50	MOCH
7676	O	MOCH	2003 RBM	9:50	BCCH
7557	O	RBNU	2003 RMB	8:00	BCCH
7557	O	RBNU	2003 RMB	8:00	BCCH
7557	O	RBNU	2003 RMB	8:00	BCCH
7557	O	RBNU	2003 RMB	8:00	BCCH
7557	O	RBNU	2003 RMB	8:00	BCCH
7557	O	RBNU	2003 RMB	8:00	BCCH
7557	O	RBNU	2003 RMB	8:00	RBNU
7557	O	RBNU	2003 RMB	8:00	RBNU
7386	O	MOCH	2003 BRM	8:05	BCCH
7386	O	MOCH	2003 BRM	8:05	RBNU
7386	O	MOCH	2003 BRM	8:05	RBNU
7386	O	MOCH	2003 BRM	8:05	RBNU
7386	O	MOCH	2003 BRM	8:05	RBNU
7386	O	MOCH	2003 BRM	8:05	RBNU
7386	O	MOCH	2003 BRM	8:05	BCCH
7386	O	MOCH	2003 BRM	8:05	none
7998	O	RBNU	2002 MBR	10:37:00	BCCH
7929	O	RBNU	2002 BRM	9:38	BCCH
7929	O	RBNU	2002 BRM	9:38	BCCH
7929	O	RBNU	2002 BRM	9:38	BCCH
7929	O	RBNU	2002 BRM	9:38	BCCH
7929	O	RBNU	2002 BRM	9:38	BCCH
7929	O	RBNU	2002 BRM	9:38	BCCH
7929	O	RBNU	2002 BRM	9:38	BCCH
7929	O	RBNU	2002 BRM	9:38	RBNU
7929	O	RBNU	2002 BRM	9:38	MOCH
7929	O	RBNU	2002 BRM	9:38	MOCH
7929	O	RBNU	2002 BRM	9:38	RBNU
7929	O	RBNU	2002 BRM	9:38	RBNU
7929	O	RBNU	2002 BRM	9:38	RBNU
7929	O	RBNU	2002 BRM	9:38	RBNU
7929	O	RBNU	2002 BRM	9:38	RBNU
7929	O	RBNU	2002 BRM	9:38	RBNU
7929	O	RBNU	2002 BRM	9:38	MOCH
7929	O	RBNU	2002 BRM	9:38	RBNU
7928	O	RBNU	2003 MRB	10:24	RBNU
7928	O	RBNU	2003 MRB	10:24	MOCH
7928	O	RBNU	2003 MRB	10:24	RBNU
7776	O	RBNU	2003 MBR	9:10	BCCH
7776	O	RBNU	2003 MBR	9:10	BCCH
7776	O	RBNU	2003 MBR	9:10	BCCH

7776	O	RBNU	2003	MBR	9:10	BCCH
7776	O	RBNU	2003	MBR	9:10	RBNU
7776	O	RBNU	2003	MBR	9:10	BCCH
7776	O	RBNU	2003	MBR	9:10	RBNU
7776	O	RBNU	2003	MBR	9:10	RBNU
7776	O	RBNU	2003	MBR	9:10	RBNU
7776	O	RBNU	2003	MBR	9:10	RBNU
7776	O	RBNU	2003	MBR	9:10	RBNU
7776	O	RBNU	2003	MBR	9:10	RBNU
7789	O	RBNU	2000	MRB	9:15	BCCH
7789	O	RBNU	2000	MRB	9:15	MOCH
7789	O	RBNU	2000	MRB	9:15	RBNU
7676	O	MOCH	2003	MBR	8:30	BCCH
7676	O	MOCH	2003	MBR	8:30	BCCH
7676	O	MOCH	2003	MBR	8:30	RBNU
7676	O	MOCH	2003	MBR	8:30	BCCH
7676	O	MOCH	2003	MBR	8:30	BCCH
7676	O	MOCH	2003	MBR	8:30	BCCH
7676	O	MOCH	2003	MBR	8:30	RBNU
7676	O	MOCH	2003	MBR	8:30	MOCH
7676	O	MOCH	2003	MBR	8:30	RBNU
7676	O	MOCH	2003	MBR	8:30	MOCH
7676	O	MOCH	2003	MBR	8:30	MOCH
7676	O	MOCH	2003	MBR	8:30	MOCH
7676	O	MOCH	2003	MBR	8:30	RBNU
7676	O	MOCH	2003	MBR	8:30	MOCH
7676	O	MOCH	2003	MBR	8:30	MOCH
7676	O	MOCH	2003	MBR	8:30	BCCH
7676	O	MOCH	2003	MBR	8:30	BCCH
7676	O	MOCH	2003	MBR	8:30	BCCH
7676	O	MOCH	2003	MBR	8:30	RBNU
7676	O	MOCH	2003	MBR	8:30	none
7676	O	MOCH	2003	MBR	8:30	none
7676	O	MOCH	2003	MBR	8:30	none
7676	O	MOCH	2003	MBR	8:30	MOCH
7676	O	MOCH	2003	MBR	8:30	BCCH
7676	O	MOCH	2003	MBR	8:30	RBNU
7676	O	MOCH	2003	MBR	8:30	BCCH
7676	O	MOCH	2003	MBR	8:30	BCCH
7998	O	RBNU	2002	BRM	8:44	none
7998	O	RBNU	2002	BRM	8:44	RBNU
7998	O	RBNU	2002	BRM	8:44	MOCH
7386	O	MOCH	2003	BMR	10:20	none
7564	O	RBNU	2003	MBR	11:21	MOCH

7564	O	RBNU	2003	MBR	11:21	BCCH
7564	O	RBNU	2003	MBR	11:21	BCCH
7564	O	RBNU	2003	MBR	11:21	MOCH
7564	O	RBNU	2003	MBR	11:21	MOCH
7564	O	RBNU	2003	MBR	11:21	BCCH
7564	O	RBNU	2003	MBR	11:21	MOCH
7564	O	RBNU	2003	MBR	11:21	BCCH
7564	O	RBNU	2003	MBR	11:21	BCCH
7789	O	RBNU	2000	MRB	8:31	BCCH
7789	O	RBNU	2000	MRB	8:31	BCCH
7789	O	RBNU	2000	MRB	8:31	BCCH
7789	O	RBNU	2000	MRB	8:31	BCCH
7789	O	RBNU	2000	MRB	8:31	BCCH
7789	O	RBNU	2000	MRB	8:31	BCCH
7789	O	RBNU	2000	MRB	8:31	BCCH
7789	O	RBNU	2000	MRB	8:31	BCCH
7789	O	RBNU	2000	MRB	8:31	MOCH
7789	O	RBNU	2000	MRB	8:31	RBNU
7789	O	RBNU	2000	MRB	8:31	RBNU
7789	O	RBNU	2000	MRB	8:31	MOCH
7789	O	RBNU	2000	MRB	8:31	RBNU
7789	O	RBNU	2000	MRB	8:31	RBNU
7789	O	RBNU	2000	MRB	8:31	RBNU
7789	O	RBNU	2000	MRB	8:31	BCCH
7789	O	RBNU	2000	MRB	8:31	BCCH
7789	O	RBNU	2000	MRB	8:31	BCCH
7386	O	MOCH	2003	BRM	8:53:30	MOCH
7676	O	MOCH	2003	BRM	9:15:30	RBNU
7676	O	MOCH	2003	BRM	9:15:30	MOCH
7676	O	MOCH	2003	BRM	9:15:30	BCCH
7676	O	MOCH	2003	BRM	9:15:30	BCCH
7676	O	MOCH	2003	BRM	9:15:30	none
7676	O	MOCH	2003	BRM	9:15:30	BCCH
7676	O	MOCH	2003	BRM	9:15:30	MOCH
7676	O	MOCH	2003	BRM	9:15:30	MOCH
7676	O	MOCH	2003	BRM	9:15:30	MOCH
7676	O	MOCH	2003	BRM	9:15:30	RBNU
7676	O	MOCH	2003	BRM	9:15:30	RBNU
7676	O	MOCH	2003	BRM	9:15:30	RBNU
7676	O	MOCH	2003	BRM	9:15:30	RBNU
7676	O	MOCH	2003	BRM	9:15:30	MOCH
7676	O	MOCH	2003	BRM	9:15:30	RBNU
7676	O	MOCH	2003	BRM	9:15:30	RBNU
7676	O	MOCH	2003	BRM	9:15:30	RBNU

7676	O	MOCH	2003	BRM	9:15:30	RBNU
7676	O	MOCH	2003	BRM	9:15:30	none
7676	O	MOCH	2003	BRM	9:15:30	BCCH
7386	O	MOCH	2003	RMB	8:46	BCCH
7386	O	MOCH	2003	RMB	8:46	none
70723	O	BCCH	2004	BMR	9:39	MOCH
70723	O	BCCH	2004	BMR	9:39	MOCH
70723	O	BCCH	2004	BMR	9:39	MOCH
70723	O	BCCH	2004	BMR	9:39	BCCH
70723	O	BCCH	2004	BMR	9:39	BCCH
70723	O	BCCH	2004	BMR	9:39	BCCH
70723	O	BCCH	2004	BMR	9:39	BCCH
70723	O	BCCH	2004	BMR	9:39	RBNU
70723	O	BCCH	2004	BMR	9:39	RBNU
70723	O	BCCH	2004	BMR	9:39	MOCH
70723	O	BCCH	2004	BMR	9:39	RBNU
70723	O	BCCH	2004	BMR	9:39	none
70730	O	BCCH	2004	RBM	8:33	RBNU
70730	O	BCCH	2004	RBM	8:33	BCCH
70730	O	BCCH	2004	RBM	8:33	BCCH
70730	O	BCCH	2004	RBM	8:33	MOCH
7063	O	MOCH	2004	MBR	11:14	BCCH
7063	O	MOCH	2004	MBR	11:14	MOCH
7063	O	MOCH	2004	MBR	11:14	MOCH
7063	O	MOCH	2004	MBR	11:14	MOCH
7063	O	MOCH	2004	MBR	11:14	MOCH
7063	O	MOCH	2004	MBR	11:14	MOCH
7063	O	MOCH	2004	MBR	11:14	BCCH
7063	O	MOCH	2004	MBR	11:14	RBNU
7478	O	TRES	2004	RBM	10:03	BCCH
7478	O	TRES	2004	RBM	10:03	RBNU
7478	O	TRES	2004	RBM	10:03	BCCH
7478	O	TRES	2004	RBM	10:03	RBNU
7478	O	TRES	2004	RBM	10:03	none
7111	O	MOCH	2004	BRM	10:42	BCCH
7111	O	MOCH	2004	BRM	10:42	RBNU
7111	O	MOCH	2004	BRM	10:42	none
7111	O	MOCH	2004	BRM	10:42	none
7111	O	MOCH	2004	BRM	10:42	BCCH
7111	O	MOCH	2004	BRM	10:42	BCCH
7130	O	MOCH	2004	BMR	12:19	MOCH
7130	O	MOCH	2004	BMR	12:19	MOCH
7130	O	MOCH	2004	BMR	12:19	none
7130	O	MOCH	2004	BMR	12:19	BCCH

7130	O	MOCH	2004	BMR	12:19	RBNU
70706	O	RBNU	2004	MRB	9:53	none
70706	O	RBNU	2004	MRB	9:53	none
70706	O	RBNU	2004	MRB	9:53	BCCH
70706	O	RBNU	2004	MRB	9:53	none
70706	O	RBNU	2004	MRB	9:53	none
70706	O	RBNU	2004	MRB	9:53	BCCH
70706	O	RBNU	2004	MRB	9:53	BCCH
70706	O	RBNU	2004	MRB	9:53	BCCH
70706	O	RBNU	2004	MRB	9:53	RBNU
7512	O	RBNU	2004	BMR	9:10	none
7512	O	RBNU	2004	BMR	9:10	none
7512	O	RBNU	2004	BMR	9:10	none
7512	O	RBNU	2004	BMR	9:10	none
7512	O	RBNU	2004	BMR	9:10	BCCH
7512	O	RBNU	2004	BMR	9:10	none
7512	O	RBNU	2004	BMR	9:10	BCCH
7512	O	RBNU	2004	BMR	9:10	MOCH
7512	O	RBNU	2004	BMR	9:10	MOCH
7512	O	RBNU	2004	BMR	9:10	BCCH
7512	O	RBNU	2004	BMR	9:10	BCCH
7512	O	RBNU	2004	BMR	9:10	MOCH
7115	O	MOCH	2004	RMB	10:34	BCCH
7115	O	MOCH	2004	RMB	10:34	BCCH
7115	O	MOCH	2004	RMB	10:34	MOCH
7115	O	MOCH	2004	RMB	10:34	MOCH
7115	O	MOCH	2004	RMB	10:34	MOCH
7115	O	MOCH	2004	RMB	10:34	BCCH
7115	O	MOCH	2004	RMB	10:34	BCCH
7115	O	MOCH	2004	RMB	10:34	MOCH
7115	O	MOCH	2004	RMB	10:34	BCCH
7115	O	MOCH	2004	RMB	10:34	BCCH
7545	O	RBNU	2005	MRB	9:24	none
7545	O	RBNU	2005	MRB	9:24	MOCH
7545	O	RBNU	2005	MRB	9:24	RBNU
7545	O	RBNU	2005	MRB	9:24	RBNU
7545	O	RBNU	2005	MRB	9:24	RBNU
7545	O	RBNU	2005	MRB	9:24	RBNU
7545	O	RBNU	1999	MRB	9:24	BCCH
7545	O	RBNU	2005	MRB	9:24	RBNU
7545	O	RBNU	2005	MRB	9:24	RBNU
7545	O	RBNU	2005	MRB	9:24	RBNU
7545	O	RBNU	2005	MRB	9:24	RBNU
7657	O	RBNU	1999	MBR	9:10	none

	7657	O	RBNU	1999	MBR	9:10	none
	7657	O	RBNU	1999	MBR	9:13	MOCH
	7657	O	RBNU	1999	MBR	9:13	MOCH
	7657	O	RBNU	1999	MBR	9:13	MOCH
	7657	O	RBNU	1999	MBR	9:13	MOCH
	7657	O	RBNU	1999	MBR	9:20	BCCH
	7657	O	RBNU	1999	MBR	9:20	BCCH
	7657	O	RBNU	1999	MBR	9:26	RBNU
	7657	O	RBNU	1999	MBR	9:26	RBNU
	7657	O	RBNU	1999	MBR	9:26	RBNU
	7657	O	RBNU	1999	MBR	9:26	RBNU
	7657	O	RBNU	1999	MBR	9:26	RBNU
	7657	O	RBNU	1999	MBR	9:26	RBNU
	7595	O	MOCH	2005	MRB	8:22	MOCH
	7595	O	MOCH	2005	MRB	8:22	MOCH
	7595	O	MOCH	2005	MRB	8:22	MOCH
	7595	O	MOCH	2005	MRB	8:22	MOCH
	7595	O	MOCH	2005	MRB	8:22	MOCH
	7595	O	MOCH	2005	MRB	8:22	RBNU
	7595	O	MOCH	2005	MRB	8:22	BCCH
	7595	O	MOCH	2005	MRB	8:22	RBNU
	7595	O	MOCH	2005	MRB	8:22	none
	7595	O	MOCH	2005	MRB	8:22	MOCH
	7595	O	MOCH	2005	MRB	8:22	RBNU
	7595	O	MOCH	2005	MRB	8:22	RBNU
	7595	O	MOCH	2005	MRB	8:22	BCCH
	7595	O	MOCH	2005	MRB	8:22	BCCH
	70171	O	DOWO	2005	BMR	12:52	none
	70171	O	DOWO	2005	BMR	12:52	BCCH
	70171	O	DOWO	2005	BMR	12:52	BCCH
	70171	O	DOWO	2005	BMR	12:52	BCCH
	70418	O	MOCH	2005	MBR	9:31	ALL
	7280	O			RBM	11:21	
	7974	O	MOCH	2005	MBR	8:37	RBNU
	7974	O	MOCH	2005	MBR	8:37	BCCH
	7974	O	MOCH	2005	MBR	8:37	MOCH
	7974	O	MOCH	2005	MBR	8:37	RBNU
	7974	O	MOCH	2005	MBR	8:37	
IB	7374	O	RBNU	2005	RMB	9:47	MOCH
IB	7374	O	RBNU	2005	RMB	9:47	RBNU
IB	7374	O	RBNU	2005	RMB	9:47	MOCH
IB	7374	O	RBNU	2005	RMB	9:47	MOCH
IB	7374	O	RBNU	2005	RMB	9:47	MOCH
IB	7374	O	RBNU	2005	RMB	9:47	MOCH

IB	7374	O	RBNU	2005	RMB	9:47	MOCH
IB	7374	O	RBNU	2005	RMB	9:47	MOCH
IB	7374	O	RBNU	2005	RMB	9:47	MOCH
IB	7374	O	RBNU	2005	RMB	9:47	MOCH
IB	7374	O	RBNU	2005	RMB	9:47	MOCH
IB	7374	O	RBNU	2005	RMB	9:47	MOCH
IB	7374	O	RBNU	2005	RMB	9:47	BCCH
IB	7374	O	RBNU	2005	RMB	9:47	BCCH
IB	7374	O	RBNU	2005	RMB	9:47	MOCH
IB	7374	O	RBNU	2005	RMB	9:47	MOCH
IB	7374	O	RBNU	2005	RMB	9:47	MOCH
IB	7374	O	RBNU	2005	RMB	9:47	MOCH
IB	7374	O	RBNU	2005	RMB	9:47	MOCH
IB	7374	O	RBNU	2005	RMB	9:47	MOCH
IB	7374	O	RBNU	2005	RMB	9:47	BCCH
IB	7374	O	RBNU	2005	RMB	9:47	BCCH
IB	7374	O	RBNU	2005	RMB	9:47	BCCH
IB	7374	O	RBNU	2005	RMB	9:47	MOCH
IB	7374	O	RBNU	2005	RMB	9:47	MOCH
IB	7374	O	RBNU	2005	RMB	9:47	BCCH
IB	7374	O	RBNU	2005	RMB	9:47	RBNU
IB	7374	O	RBNU	2005	RMB	9:47	MOCH
IB	7374	O	RBNU	2005	RMB	9:47	BCCH
IB	70755	O	MOCH	2006	RMB	11:50	MOCH
IB	70755	O	MOCH	2006	RMB	11:50	MOCH
IB	70755	O	MOCH	2006	RMB	11:50	MOCH
IB	70755	O	MOCH	2006	RMB	11:50	MOCH
IB	70755	O	MOCH	2006	RMB	11:50	MOCH
IB	70755	O	MOCH	2006	RMB	11:50	RBNU
IB	70755	O	MOCH	2006	RMB	11:50	BCCH
IB	7408	O	MOCH	2006	MRB	9:20	MOCH
IB	7408	O	MOCH	2006	MRB	9:20	BCCH
IB	7408	O	MOCH	2006	MRB	9:20	MOCH
IB	7408	O	MOCH	2006	MRB	9:20	MOCH
IB	7408	O	MOCH	2006	MRB	9:20	RBNU
IB	7408	O	MOCH	2006	MRB	9:20	BCCH
IB	7408	O	MOCH	2006	MRB	9:20	BCCH
IB	7408	O	MOCH	2006	MRB	9:20	RBNU
IB	7408	O	MOCH	2006	MRB	9:20	RBNU
IB	7408	O	MOCH	2006	MRB	9:20	RBNU
IB	7408	O	MOCH	2006	MRB	9:20	RBNU
IB	7408	O	MOCH	2006	MRB	9:20	BCCH
IB	7280	O	MOCH	2006	MRB	9:09	BCCH
IB	7280	O	MOCH	2006	MRB	9:09	MOCH

IB	7280	O	MOCH	2006	MRB	9:09	MOCH
IB	7280	O	MOCH	2006	MRB	9:09	MOCH
IB	7280	O	MOCH	2006	MRB	9:09	MOCH
IB	7280	O	MOCH	2006	MRB	9:09	BCCH
IB	7280	O	MOCH	2006	MRB	9:09	BCCH
IB	7280	O	MOCH	2006	MRB	9:09	BCCH
IB	7280	O	MOCH	2006	MRB	9:09	MOCH
IB	7280	O	MOCH	2006	MRB	9:09	MOCH
IB	7280	O	MOCH	2006	MRB	9:09	MOCH
IB	7280	O	MOCH	2006	MRB	9:09	MOCH
IB	7280	O	MOCH	2006	MRB	9:09	MOCH
IB	7280	O	MOCH	2006	MRB	9:09	BCCH
IB	7280	O	MOCH	2006	MRB	9:09	BCCH
IB	7280	O	MOCH	2006	MRB	9:09	BCCH
IB	7280	O	MOCH	2006	MRB	9:09	MOCH
IB	7280	O	MOCH	2006	MRB	9:09	BCCH
IB	7280	O	MOCH	2006	MRB	9:09	MOCH
IB	7280	O	MOCH	2006	MRB	9:09	BCCH
IB	7280	O	MOCH	2006	MRB	9:09	MOCH
IB	7280	O	MOCH	2006	MRB	9:09	BCCH
IB	7280	O	MOCH	2006	MRB	9:09	MOCH
IB	7280	O	MOCH	2006	MRB	9:09	MOCH
IB	7280	O	MOCH	2006	MRB	9:09	MOCH
IB	7280	O	MOCH	2006	MRB	9:09	MOCH
IB	7280	O	MOCH	2006	MRB	9:09	MOCH
IB	7280	O	MOCH	2006	MRB	9:09	BCCH
IB	7280	O	MOCH	2006	MRB	9:09	BCCH
IB	7280	O	MOCH	2006	MRB	9:09	BCCH
IB	7280	O	MOCH	2006	MRB	9:09	RBNU
IB	7280	O	MOCH	2006	MRB	9:09	RBNU
IB	7280	O	MOCH	2006	MRB	9:09	RBNU
IB	7280	O	MOCH	2006	MRB	9:09	BCCH
IB	7280	O	MOCH	2006	MRB	9:09	RBNU
IB	7280	O	MOCH	2006	MRB	9:09	RBNU
IB	7280	O	MOCH	2006	MRB	9:09	RBNU
IB	7280	O	MOCH	2006	MRB	9:09	RBNU
IB	7280	O	MOCH	2006	MRB	9:09	MOCH
IB	7280	O	MOCH	2006	MRB	9:09	MOCH
IB	7280	O	MOCH	2006	MRB	9:09	RBNU
IB	7280	O	MOCH	2006	MRB	9:09	BCCH
IB	70703	O	RBNU	2006	MBR	10:03	MOCH
IB	70703	O	RBNU	2006	MBR	10:03	MOCH
IB	70703	O	RBNU	2006	MBR	10:03	BCCH
IB	70703	O	RBNU	2006	MBR	10:03	BCCH

IB	70703	O	RBNU	2006	MBR	10:03	BCCH
IB	70703	O	RBNU	2006	MBR	10:03	BCCH
IB	70703	O	RBNU	2006	MBR	10:03	BCCH
IB	70703	O	RBNU	2006	MBR	10:03	MOCH
IB	70703	O	RBNU	2006	MBR	10:03	BCCH
IB	70703	O	RBNU	2006	MBR	10:03	BCCH
IB	70703	O	RBNU	2006	MBR	10:03	BCCH
IB	70703	O	RBNU	2006	MBR	10:03	MOCH
IB	70703	O	RBNU	2006	MBR	10:03	MOCH
IB	70703	O	RBNU	2006	MBR	10:03	BCCH
IB	70703	O	RBNU	2006	MBR	10:03	BCCH
IB	70703	O	RBNU	2006	MBR	10:03	MOCH
IB	70703	O	RBNU	2006	MBR	10:03	BCCH
IB	70703	O	RBNU	2006	MBR	10:03	RBNU
IB	70703	O	RBNU	2006	MBR	10:03	MOCH
IB	70703	O	RBNU	2006	MBR	10:03	MOCH
IB	70703	O	RBNU	2006	MBR	10:03	MOCH
IB	70703	O	RBNU	2006	MBR	10:03	MOCH
IB	70703	O	RBNU	2006	MBR	10:03	RBNU
IB	70703	O	RBNU	2006	MBR	10:03	MOCH
IB	70703	O	RBNU	2006	MBR	10:03	BCCH
IB	70703	O	RBNU	2006	MBR	10:03	RBNU
IB	70110	O	MOCH	2005	RMB	10:49	BCCH
IB	70110	O	MOCH	2005	RMB	10:49	MOCH
IB	70110	O	MOCH	2005	RMB	10:49	MOCH
IB	70110	O	MOCH	2005	RMB	10:49	BCCH
IB	70110	O	MOCH	2005	RMB	10:49	MOCH
IB	70110	O	MOCH	2005	RMB	10:49	BCCH
IB	70110	O	MOCH	2005	RMB	10:49	MOCH
IB	70110	O	MOCH	2005	RMB	10:49	MOCH
IB	70110	O	MOCH	2005	RMB	10:49	MOCH
IB	70110	O	MOCH	2005	RMB	10:49	BCCH
IB	70110	O	MOCH	2005	RMB	10:49	RBNU
IB	70110	O	MOCH	2005	RMB	10:49	RBNU
IB	70110	O	MOCH	2005	RMB	10:49	BCCH
IB	70110	O	MOCH	2005	RMB	10:49	BCCH
IB	70110	O	MOCH	2005	RMB	10:49	MOCH
IB	70110	O	MOCH	2005	RMB	10:49	RBNU
IB	70110	O	MOCH	2005	RMB	10:49	RBNU
IB	70110	O	MOCH	2005	RMB	10:49	RBNU
IB	70110	O	MOCH	2005	RMB	10:49	RBNU
IB	70110	O	MOCH	2005	RMB	10:49	RBNU
IB	70110	O	MOCH	2005	RMB	10:49	RBNU
IB	70110	O	MOCH	2005	RMB	10:49	RBNU

IB	70110	O	MOCH	2005	RMB	10:49	RBNU
IB	70110	O	MOCH	2005	RMB	10:49	MOCH
IB	70110	O	MOCH	2005	RMB	10:49	MOCH
IB	70110	O	MOCH	2005	RMB	10:49	MOCH
IB	70110	O	MOCH	2005	RMB	10:49	RBNU
IB	70110	O	MOCH	2005	RMB	10:49	MOCH
IB	70110	O	MOCH	2005	RMB	10:49	BCCH
IB	06-SLD-06	O	RBNU	2006	BRM	9:04	RBNU
IB	06-SLD-06	O	RBNU	2006	BRM	9:04	RBNU
IB	06-SLD-06	O	RBNU	2006	BRM	9:04	RBNU
IB	06-SLD-06	O	RBNU	2006	BRM	9:04	RBNU
IB	06-SLD-06	O	RBNU	2006	BRM	9:04	RBNU
IB	06-SLD-06	O	RBNU	2006	BRM	9:04	RBNU
IB	06-SLD-06	O	RBNU	2006	BRM	9:04	RBNU
IB	06-SLD-06	O	RBNU	2006	BRM	9:04	RBNU
IB	06-SLD-06	O	RBNU	2006	BRM	9:04	RBNU
IB	06-SLD-06	O	RBNU	2006	BRM	9:04	RBNU
IB	06-SLD-06	O	RBNU	2006	BRM	9:04	MOCH
IB	06-SLD-06	O	RBNU	2006	BRM	9:04	MOCH
IB	06-SLD-06	O	RBNU	2006	BRM	9:04	MOCH
IB	06-SLD-06	O	RBNU	2006	BRM	9:04	MOCH
IB	06-SLD-06	O	RBNU	2006	BRM	9:04	MOCH
IB	06-SLD-06	O	RBNU	2006	BRM	9:04	BCCH
IB	06-SLD-06	O	RBNU	2006	BRM	9:04	RBNU
IB	06-SLD-04	O			RMB	11:18	MOCH
IB	06-SLD-04	O			RMB	11:18	BCCH
IB	06-SLD-04	O			RMB	11:18	MOCH
IB	06-SLD-04	O			RMB	11:18	BCCH
IB	06-SLD-04	O			RMB	11:18	BCCH
IB	06-SLD-04	O			RMB	11:18	MOCH
IB	06-SLD-04	O			RMB	11:18	MOCH
IB	06-SLD-04	O			RMB	11:18	MOCH
IB	06-SLD-04	O			RMB	11:18	MOCH
IB	06-SLD-04	O			RMB	11:18	MOCH
IB	06-SLD-04	O			RMB	11:18	MOCH
IB	06-SLD-04	O			RMB	11:18	BCCH
IB	06-SLD-04	O			RMB	11:18	BCCH
IB	06-SLD-04	O			RMB	11:18	BCCH
IB	06-SLD-04	O			RMB	11:18	BCCH
IB	06-SLD-04	O			RMB	11:18	MOCH
IB	06-SLD-04	O			RMB	11:18	MOCH
IB	06-SLD-04	O			RMB	11:18	MOCH
IB	06-SLD-04	O			RMB	11:18	MOCH
IB	06-SLD-04	O			RMB	11:18	BCCH

IB	06-SLD-04	O			RMB	11:18	MOCH
IB	06-SLD-04	O			RMB	11:18	MOCH
IB	06-SLD-04	O			RMB	11:18	MOCH
IB	06-SLD-04	O			RMB	11:18	MOCH
IB	06-SLD-04	O			RMB	11:18	BCCH
IB	06-SLD-04	O			RMB	11:18	BCCH
IB	06-SLD-04	O			RMB	11:18	BCCH
IB	06-SLD-04	O			RMB	11:18	BCCH
IB	06-SLD-04	O			RMB	11:18	BCCH
IB	06-SLD-04	O			RMB	11:18	MOCH
IB	06-SLD-04	O			RMB	11:18	MOCH
IB	06-SLD-04	O			RMB	11:18	BCCH
IB	06-SLD-04	O			RMB	11:18	MOCH
IB	06-SLD-04	O			RMB	11:18	BCCH
IB	06-SLD-04	O			RMB	11:18	MOCH
IB	06-SLD-04	O			RMB	11:18	BCCH
IB	06-SLD-04	O			RMB	11:18	RBNU
IB	06-SLD-04	O	not in allca	not in allca	RMB	11:18	RBNU
IB	06-SLD-04	O			RMB	11:18	MOCH
IB	06-SLD-04	O			RMB	11:18	BCCH
IB	06-SLD-04	O			RMB	11:18	RBNU
IB	70418	O	MOCH	2005	RBM	10:01	RBNU
IB	70418	O	MOCH	2005	RBM	10:01	BCCH
IB	70418	O	MOCH	2005	RBM	10:01	MOCH
IB	70694	O	MOCH	2006	MRB	10:51	MOCH
IB	70694	O	MOCH	2006	MRB	10:51	MOCH
IB	70694	O	MOCH	2006	MRB	10:51	BCCH
IB	70694	O	MOCH	2006	MRB	10:51	MOCH
IB	70694	O	MOCH	2006	MRB	10:51	BCCH
IB	70694	O	MOCH	2006	MRB	10:51	MOCH
IB	70694	O	MOCH	2006	MRB	10:51	BCCH
IB	70694	O	MOCH	2006	MRB	10:51	MOCH
IB	70694	O	MOCH	2006	MRB	10:51	RBNU
IB	70694	O	MOCH	2006	MRB	10:51	RBNU
IB	70694	O	MOCH	2006	MRB	10:51	MOCH
IB	70694	O	MOCH	2006	MRB	10:51	MOCH
IB	70694	O	MOCH	2006	MRB	10:51	MOCH
IB	70694	O	MOCH	2006	MRB	10:51	BCCH
IB	70694	O	MOCH	2006	MRB	10:51	BCCH
IB	70694	O	MOCH	2006	MRB	10:51	MOCH
IB	70694	O	MOCH	2006	MRB	10:51	MOCH
IB	70694	O	MOCH	2006	MRB	10:51	MOCH
IB	70694	O	MOCH	2006	MRB	10:51	RBNU
IB	70694	O	MOCH	2006	MRB	10:51	RBNU

IB	70694	O	MOCH	2006	MRB	10:51	MOCH
IB	70694	O	MOCH	2006	MRB	10:51	RBNU
IB	70694	O	MOCH	2006	MRB	10:51	BCCH
IB	70694	O	MOCH	2006	MRB	10:51	MOCH
IB	70694	O	MOCH	2006	MRB	10:51	MOCH
IB	70694	O	MOCH	2006	MRB	10:51	BCCH
IB	70694	O	MOCH	2006	MRB	10:51	BCCH
IB	70694	O	MOCH	2006	MRB	10:51	BCCH
IB	70694	O	MOCH	2006	MRB	10:51	MOCH
IB	70694	O	MOCH	2006	MRB	10:51	RBNU
IB	70694	O	MOCH	2006	MRB	10:51	BCCH
IB	70735	O	RBNU	2004	RMB	9:12	MOCH
IB	70735	O	RBNU	2004	RMB	9:12	MOCH
IB	70735	O	RBNU	2004	RMB	9:12	MOCH
IB	70735	O	RBNU	2004	RMB	9:12	MOCH
IB	70735	O	RBNU	2004	RMB	9:12	MOCH
IB	70735	O	RBNU	2004	RMB	9:12	BCCH
IB	70735	O	RBNU	2004	RMB	9:12	BCCH
IB	70735	O	RBNU	2004	RMB	9:12	BCCH
IB	70735	O	RBNU	2004	RMB	9:12	BCCH
IB	70735	O	RBNU	2004	RMB	9:12	MOCH
IB	70735	O	RBNU	2004	RMB	9:12	BCCH
IB	70735	O	RBNU	2004	RMB	9:12	RBNU
IB	70735	O	RBNU	2004	RMB	9:12	RBNU
IB	70735	O	RBNU	2004	RMB	9:12	RBNU
IB	70735	O	RBNU	2004	RMB	9:12	RBNU
IB	70735	O	RBNU	2004	RMB	9:12	MOCH
IB	70735	O	RBNU	2004	RMB	9:12	BCCH
IB	70735	O	RBNU	2004	RMB	9:12	BCCH
IB	70735	O	RBNU	2004	RMB	9:12	RBNU
IB	70735	O	RBNU	2004	RMB	9:12	MOCH
IB	70735	O	RBNU	2004	RMB	9:12	BCCH
IB	70735	O	RBNU	2004	RMB	9:12	BCCH
IB	70735	O	RBNU	2004	RMB	9:12	
IB	70420	O	RBNU	2006	BMR	11:48	BCCH
IB	70420	O	RBNU	2006	BMR	11:48	MOCH
IB	70420	O	RBNU	2006	BMR	11:48	RBNU
IB	70420	O	RBNU	2006	BMR	11:48	RBNU
IB	70420	O	RBNU	2006	BMR	11:48	RBNU
IB	70420	O	RBNU	2006	BMR	11:48	RBNU
IB	70420	O	RBNU	2006	BMR	11:48	RBNU
IB	70420	O	RBNU	2006	BMR	11:48	RBNU
IB	70420	O	RBNU	2006	BMR	11:48	BCCH
IB	70420	O	RBNU	2006	BMR	11:48	MOCH

IB	70420	O	RBNU	2006	BMR	11:48	RBNU
IB	70405	O	RBNU	2005	BMR	10:32	BCCH
IB	70405	O	RBNU	2005	BMR	10:32	BCCH
IB	70405	O	RBNU	2005	BMR	10:32	BCCH
IB	70405	O	RBNU	2005	BMR	10:32	BCCH
IB	70405	O	RBNU	2005	BMR	10:32	BCCH
IB	70405	O	RBNU	2005	BMR	10:32	BCCH
IB	70405	O	RBNU	2005	BMR	10:32	MOCH
IB	70405	O	RBNU	2005	BMR	10:32	MOCH
IB	70405	O	RBNU	2005	BMR	10:32	MOCH
IB	70405	O	RBNU	2005	BMR	10:32	RBNU
IB	70405	O	RBNU	2005	BMR	10:32	RBNU
IB	70405	O	RBNU	2005	BMR	10:32	RBNU
IB	70405	O	RBNU	2005	BMR	10:32	RBNU
IB	70405	O	RBNU	2005	BMR	10:32	RBNU
IB	70405	O	RBNU	2005	BMR	10:32	BCCH
IB	70405	O	RBNU	2005	BMR	10:32	BCCH
IB	70405	O	RBNU	2005	BMR	10:32	BCCH
IB	70405	O	RBNU	2005	BMR	10:32	MOCH
IB	70405	O	RBNU	2005	BMR	10:32	RBNU
IB	70405	O	RBNU	2005	BMR	10:32	RBNU
IB	70405	O	RBNU	2005	BMR	10:32	BCCH
IB	70405	O	RBNU	2005	BMR	10:32	MOCH
IB	70405	O	RBNU	2005	BMR	10:32	MOCH
IB	70405	O	RBNU	2005	BMR	10:32	MOCH
IB	70405	O	RBNU	2005	BMR	10:32	RBNU
IB	70405	O	RBNU	2005	BMR	10:32	BCCH
IB	70405	O	RBNU	2005	BMR	10:32	MOCH
IB	70405	O	RBNU	2005	BMR	10:32	RBNU
IB	7820	O	MOCH	2005	MRB	8:30	MOCH
IB	7820	O	MOCH	2005	MRB	8:30	MOCH
IB	7820	O	MOCH	2005	MRB	8:30	MOCH
IB	7820	O	MOCH	2005	MRB	8:30	MOCH
IB	7820	O	MOCH	2005	MRB	8:30	RBNU
IB	7820	O	MOCH	2005	MRB	8:30	BCCH
IB	7820	O	MOCH	2005	MRB	8:30	MOCH
IB	7820	O	MOCH	2005	MRB	8:30	RBNU
IB	7820	O	MOCH	2005	MRB	8:30	RBNU
IB	7820	O	MOCH	2005	MRB	8:30	RBNU
IB	7820	O	MOCH	2005	MRB	8:30	RBNU
IB	7820	O	MOCH	2005	MRB	8:30	MOCH
IB	7820	O	MOCH	2005	MRB	8:30	MOCH
IB	7820	O	MOCH	2005	MRB	8:30	MOCH
IB	7820	O	MOCH	2005	MRB	8:30	MOCH

IB	7820	O	MOCH	2005	MRB	8:30	RBNU
IB	7820	O	MOCH	2005	MRB	8:30	BCCH
IB	7891	O	TAHU	2006	MRB	9:08	MOCH
IB	7891	O	TAHU	2006	MRB	9:08	MOCH
IB	7891	O	TAHU	2006	MRB	9:08	MOCH
IB	7891	O	TAHU	2006	MRB	9:08	MOCH
IB	7891	O	TAHU	2006	MRB	9:08	MOCH
IB	7891	O	TAHU	2006	MRB	9:08	MOCH
IB	7891	O	TAHU	2006	MRB	9:08	MOCH
IB	7891	O	TAHU	2006	MRB	9:08	MOCH
IB	7891	O	TAHU	2006	MRB	9:08	MOCH
IB	7891	O	TAHU	2006	MRB	9:08	BCCH
IB	7891	O	TAHU	2006	MRB	9:08	MOCH
IB	7891	O	TAHU	2006	MRB	9:08	RBNU
IB	7891	O	TAHU	2006	MRB	9:08	BCCH
IB	7891	O	TAHU	2006	MRB	9:08	BCCH
IB	7888	O	TRES	2006	RBM	10:14	BCCH
IB	7888	O	TRES	2006	RBM	10:14	BCCH
IB	7888	O	TRES	2006	RBM	10:14	BCCH
IB	7888	O	TRES	2006	RBM	10:14	BCCH
IB	7888	O	TRES	2006	RBM	10:14	BCCH
IB	7888	O	TRES	2006	RBM	10:14	BCCH
IB	7888	O	TRES	2006	RBM	10:14	BCCH
IB	7888	O	TRES	2006	RBM	10:14	MOCH
IB	7888	O	TRES	2006	RBM	10:14	RBNU
IB	7888	O	TRES	2006	RBM	10:14	RBNU
IB	7888	O	TRES	2006	RBM	10:14	RBNU
IB	7888	O	TRES	2006	RBM	10:14	BCCH
IB	7888	O	TRES	2006	RBM	10:14	RBNU
IB	7888	O	TRES	2006	RBM	10:14	BCCH
IB	7888	O	TRES	2006	RBM	10:14	none
IB	7888	O	TRES	2006	RBM	10:14	RBNU
IB	7888	O	TRES	2006	RBM	10:14	RBNU
IB	7888	O	TRES	2006	RBM	10:14	RBNU
IB	7888	O	TRES	2006	RBM	10:14	RBNU
IB	7888	O	TRES	2006	RBM	10:14	BCCH
IB	7888	O	TRES	2006	RBM	10:14	MOCH
IB	7563	O			BMR	10:36	RBNU
IB	7563	O			BMR	10:36	BCCH
IB	7563	O			BMR	10:36	MOCH
IB	7563	O			BMR	10:36	MOCH
IB	7563	O			BMR	10:36	MOCH
IB	7563	O			BMR	10:36	RBNU
IB	7563	O			BMR	10:36	MOCH

IB	7563	O			BMR	10:36	MOCH
IB	7563	O			BMR	10:36	MOCH
IB	7563	O			BMR	10:36	MOCH
IB	7563	O			BMR	10:36	MOCH
IB	7563	O			BMR	10:36	MOCH
IB	7563	O			BMR	10:36	MOCH
IB	7563	O			BMR	10:36	BCCH
IB	7563	O			BMR	10:36	MOCH
IB	7563	O			BMR	10:36	RBNU
IB	7563	O			BMR	10:36	RBNU
IB	7563	O			BMR	10:36	RBNU
IB	7563	O			BMR	10:36	MOCH
IB	7563	O			BMR	10:36	BCCH
IB	7563	O			BMR	10:36	MOCH
IB	7563	O			BMR	10:36	BCCH
IB	7563	O			BMR	10:36	MOCH
IB	7563	O			BMR	10:36	RBNU
IB	7563	O			BMR	10:36	RBNU
IB	7563	O			BMR	10:36	
IB	7131	O	MOCH	2006	MBR	11:08 AM	BCCH
IB	7131	O	MOCH	2006	MBR	11:08 AM	BCCH
IB	7131	O	MOCH	2006	MBR	11:08 AM	BCCH
IB	7131	O	MOCH	2006	MBR	11:08 AM	BCCH
IB	7131	O	MOCH	2006	MBR	11:08 AM	RBNU
IB	7131	O	MOCH	2006	MBR	11:08 AM	MOCH
IB	7131	O	MOCH	2006	MBR	11:08 AM	MOCH
IB	7131	O	MOCH	2006	MBR	11:08 AM	RBNU
IB	7131	O	MOCH	2006	MBR	11:08 AM	MOCH
IB	7131	O	MOCH	2006	MBR	11:08 AM	MOCH
IB	7131	O	MOCH	2006	MBR	11:08 AM	RBNU
IB	7131	O	MOCH	2006	MBR	11:08 AM	RBNU
IB	7131	O	MOCH	2006	MBR	11:08 AM	RBNU
IB	7131	O	MOCH	2006	MBR	11:08 AM	RBNU
IB	7131	O	MOCH	2006	MBR	11:08 AM	RBNU
IB	7131	O	MOCH	2006	MBR	11:08 AM	RBNU
IB	7131	O	MOCH	2006	MBR	11:08 AM	RBNU
IB	7131	O	MOCH	2006	MBR	11:08 AM	RBNU
IB	7131	O	MOCH	2006	MBR	11:08 AM	RBNU
IB	7131	O	MOCH	2006	MBR	11:08 AM	RBNU
IB	7131	O	MOCH	2006	MBR	11:08 AM	RBNU
IB	7131	O	MOCH	2006	MBR	11:08 AM	RBNU
IB	7131	O	MOCH	2006	MBR	11:08 AM	RBNU
IB	7131	O	MOCH	2006	MBR	11:08 AM	RBNU
IB	7131	O	MOCH	2006	MBR	11:08 AM	MOCH

IB	7131	O	MOCH	2006	MBR	11:08 AM	MOCH
IB	7131	O	MOCH	2006	MBR	11:08 AM	BCCH
IB	7131	O	MOCH	2006	MBR	11:08 AM	MOCH
IB	7131	O	MOCH	2006	MBR	11:08 AM	BCCH
IB	7131	O	MOCH	2006	MBR	11:08 AM	RBNU
IB	70110	O	MOCH	2005	RBM	7:20 AM	MOCH
IB	70110	O	MOCH	2005	RBM	7:20 AM	MOCH
IB	70110	O	MOCH	2005	RBM	7:20 AM	MOCH
IB	70110	O	MOCH	2005	RBM	7:20 AM	MOCH
IB	70110	O	MOCH	2005	RBM	7:20 AM	MOCH
IB	70110	O	MOCH	2005	RBM	7:20 AM	MOCH
IB	70110	O	MOCH	2005	RBM	7:20 AM	RBNU
IB	70110	O	MOCH	2005	RBM	7:20 AM	RBNU
IB	70110	O	MOCH	2005	RBM	7:20 AM	RBNU
IB	70110	O	MOCH	2005	RBM	7:20 AM	RBNU
IB	70110	O	MOCH	2005	RBM	7:20 AM	RBNU
IB	70110	O	MOCH	2005	RBM	7:20 AM	RBNU
IB	70110	O	MOCH	2005	RBM	7:20 AM	RBNU
IB	70110	O	MOCH	2005	RBM	7:20 AM	RBNU
IB	70110	O	MOCH	2005	RBM	7:20 AM	RBNU
IB	70110	O	MOCH	2005	RBM	7:20 AM	RBNU
IB	70110	O	MOCH	2005	RBM	7:20 AM	RBNU
IB	70110	O	MOCH	2005	RBM	7:20 AM	MOCH
IB	70110	O	MOCH	2005	RBM	7:20 AM	RBNU
IB	70110	O	MOCH	2005	RBM	7:20 AM	BCCH
IB	70110	O	MOCH	2005	RBM	7:20 AM	MOCH
IB	70791	O	RBNU	2006	BRM	9:07 AM	MOCH
IB	70791	O	RBNU	2006	BRM	9:07 AM	RBNU
IB	70791	O	RBNU	2006	BRM	9:07 AM	BCCH
IB	70791	O	RBNU	2006	BRM	9:07 AM	RBNU
IB	70791	O	RBNU	2006	BRM	9:07 AM	MOCH
IB	70163	O	RNSA	2005	RMB	8:55 AM	MOCH
IB	70163	O	RNSA	2005	RMB	8:55 AM	MOCH
IB	70163	O	RNSA	2005	RMB	8:55 AM	MOCH
IB	70163	O	RNSA	2005	RMB	8:55 AM	MOCH
IB	70163	O	RNSA	2005	RMB	8:55 AM	MOCH
IB	70163	O	RNSA	2005	RMB	8:55 AM	MOCH
IB	70163	O	RNSA	2005	RMB	8:55 AM	BCCH
IB	70163	O	RNSA	2005	RMB	8:55 AM	BCCH
IB	70163	O	RNSA	2005	RMB	8:55 AM	RBNU
IB	70163	O	RNSA	2005	RMB	8:55 AM	RBNU
IB	70163	O	RNSA	2005	RMB	8:55 AM	RBNU
IB	70163	O	RNSA	2005	RMB	8:55 AM	RBNU
IB	70163	O	RNSA	2005	RMB	8:55 AM	RBNU

IB	70163	O	RNSA	2005	RMB	8:55 AM	MOCH
IB	70163	O	RNSA	2005	RMB	8:55 AM	MOCH
IB	70163	O	RNSA	2005	RMB	8:55 AM	BCCH
IB	70163	O	RNSA	2005	RMB	8:55 AM	RBNU
IB	70163	O	RNSA	2005	RMB	8:55 AM	RBNU
IB	70163	O	RNSA	2005	RMB	8:55 AM	MOCH
IB	70163	O	RNSA	2005	RMB	8:55 AM	BCCH
IB	70692	O	MOBL	2006	RBM	8:15 AM	RBNU
IB	70692	O	MOBL	2006	RBM	8:15 AM	BCCH
IB	70692	O	MOBL	2006	RBM	8:15 AM	MOCH
ARN	70133	O	MOCH	2007	RMB	10:46	MOCH
ARN	70133	O	MOCH	2007	RMB	10:46	MOCH
ARN	70133	O	MOCH	2007	RMB	10:46	MOCH
ARN	70133	O	MOCH	2007	RMB	10:46	BCCH
ARN	70133	O	MOCH	2007	RMB	10:46	RBNU
ARN	70133	O	MOCH	2007	RMB	10:46	RBNU
ARN	70133	O	MOCH	2007	RMB	10:46	RBNU
ARN	70133	O	MOCH	2007	RMB	10:46	RBNU
ARN	70133	O	MOCH	2007	RMB	10:46	RBNU
ARN	70133	O	MOCH	2007	RMB	10:46	MOCH
ARN	70133	O	MOCH	2007	RMB	10:46	BCCH
ARN	70133	O	MOCH	2007	RMB	10:46	BCCH
ARN	70133	O	MOCH	2007	RMB	10:46	
HK	7403	O	RBNU	2007	BRM	9:41	RBNU
HK	7403	O	RBNU	2007	BRM	9:41	RBNU
HK	7403	O	RBNU	2007	BRM	9:41	BCCH
HK	7403	O	RBNU	2007	BRM	9:41	BCCH
HK	7403	O	RBNU	2007	BRM	9:41	BCCH
HK	7403	O	RBNU	2007	BRM	9:41	RBNU
HK	7403	O	RBNU	2007	BRM	9:41	RBNU
HK	7403	O	RBNU	2007	BRM	9:41	MOCH
HK	7403	O	RBNU	2007	BRM	9:41	MOCH
HK	7403	O	RBNU	2007	BRM	9:41	MOCH
HK	7403	O	RBNU	2007	BRM	9:41	BCCH
HK	7403	O	RBNU	2007	BRM	9:41	BCCH
HK	7403	O	RBNU	2007	BRM	9:41	RBNU
HK	7403	O	RBNU	2007	BRM	9:41	MOCH
HK	7403	O	RBNU	2007	BRM	9:41	MOCH
HK	7403	O	RBNU	2007	BRM	9:41	
HK	7028	O	MOCH	2007	RBM	11:28	RBNU
HK	7028	O	MOCH	2007	RBM	11:28	RBNU
HK	7028	O	MOCH	2007	RBM	11:28	RBNU
HK	7028	O	MOCH	2007	RBM	11:28	RBNU
HK	7028	O	MOCH	2007	RBM	11:28	BCCH

HK	7028	O	MOCH	2007	RBM	11:28 MOCH
HK	7028	O	MOCH	2007	RBM	11:28 RBNU
HK	7028	O	MOCH	2007	RBM	11:28 MOCH
HK	7028	O	MOCH	2007	RBM	11:28 RBNU
HK	7028	O	MOCH	2007	RBM	11:28 BCCH
HK	7028	O	MOCH	2007	RBM	11:28 MOCH
HK	7028	O	MOCH	2007	RBM	11:28 MOCH
HK	7028	O	MOCH	2007	RBM	11:28
ARN	7972	O	RBNU	2007	MBR	9:53 RBNU
ARN	7972	O	RBNU	2007	MBR	9:53 RBNU
ARN	7972	O	RBNU	2007	MBR	9:53 RBNU
ARN	7972	O	RBNU	2007	MBR	9:53 RBNU
ARN	7972	O	RBNU	2007	MBR	9:53 RBNU
ARN	7972	O	RBNU	2007	MBR	9:53 RBNU
ARN	7972	O	RBNU	2007	MBR	9:53 RBNU
ARN	7972	O	RBNU	2007	MBR	9:53 MOCH
ARN	7972	O	RBNU	2007	MBR	9:53 MOCH
ARN	7972	O	RBNU	2007	MBR	9:53 BCCH
ARN	7972	O	RBNU	2007	MBR	9:53 RBNU
ARN	7972	O	RBNU	2007	MBR	9:53 RBNU
ARN	7972	O	RBNU	2007	MBR	9:53
HK	70196	O	MOCH	2007	MRB	9:44 BCCH
HK	70196	O	MOCH	2007	MRB	9:44 BCCH
HK	70196	O	MOCH	2007	MRB	9:44 RBNU
HK	70196	O	MOCH	2007	MRB	9:44 BCCH
HK	70196	O	MOCH	2007	MRB	9:44 MOCH
HK	70196	O	MOCH	2007	MRB	9:44 MOCH
HK	70196	O	MOCH	2007	MRB	9:44 MOCH
HK	70196	O	MOCH	2007	MRB	9:44 MOCH
HK	70196	O	MOCH	2007	MRB	9:44 MOCH
HK	70196	O	MOCH	2007	MRB	9:44 MOCH
HK	70196	O	MOCH	2007	MRB	9:44 MOCH
HK	70196	O	MOCH	2007	MRB	9:44 MOCH
HK	70196	O	MOCH	2007	MRB	9:44 MOCH
HK	70196	O	MOCH	2007	MRB	9:44 MOCH
HK	70196	O	MOCH	2007	MRB	9:44 BCCH
HK	70196	O	MOCH	2007	MRB	9:44 BCCH
HK	70196	O	MOCH	2007	MRB	9:44 MOCH
HK	70196	O	MOCH	2007	MRB	9:44 MOCH
HK	70196	O	MOCH	2007	MRB	9:44 RBNU
HK	70196	O	MOCH	2007	MRB	9:44 BCCH
HK	70196	O	MOCH	2007	MRB	9:44 MOCH
HK	70196	O	MOCH	2007	MRB	9:44 MOCH
HK	70196	O	MOCH	2007	MRB	9:44 MOCH

HK	70196	O	MOCH	2007	MRB	9:44	BCCH
HK	70196	O	MOCH	2007	MRB	9:44	BCCH
HK	70196	O	MOCH	2007	MRB	9:44	MOCH
HK	70196	O	MOCH	2007	MRB	9:44	RBNU
HK	70196	O	MOCH	2007	MRB	9:44	BCCH
HK	70196	O	MOCH	2007	MRB	9:44	MOCH
HK	70196	O	MOCH	2007	MRB	9:44	MOCH
HK	70196	O	MOCH	2007	MRB	9:44	RBNU
HK	70196	O	MOCH	2007	MRB	9:44	BCCH
HK	70196	O	MOCH	2007	MRB	9:44	RBNU
HK	70196	O	MOCH	2007	MRB	9:44	RBNU
HK	70196	O	MOCH	2007	MRB	9:44	BCCH
HK	70196	O	MOCH	2007	MRB	9:44	BCCH
HK	70196	O	MOCH	2007	MRB	9:44	MOCH
HK	70196	O	MOCH	2007	MRB	9:44	RBNU
HK	70196	O	MOCH	2007	MRB	9:44	BCCH
HK	70196	O	MOCH	2007	MRB	9:44	BCCH
HK	70196	O	MOCH	2007	MRB	9:44	RBNU
HK	70196	O	MOCH	2007	MRB	9:44	RBNU
HK	70196	O	MOCH	2007	MRB	9:44	MOCH
HK	70196	O	MOCH	2007	MRB	9:44	BCCH
HK	70196	O	MOCH	2007	MRB	9:44	MOCH
HK	70196	O	MOCH	2007	MRB	9:44	RBNU
HK	70196	O	MOCH	2007	MRB	9:44	BCCH
HK	70196	O	MOCH	2007	MRB	9:44	BCCH
HK	70196	O	MOCH	2007	MRB	9:44	
HK	2006 RBNU	O	RBNU	2006	RBM	11:53	BCCH
HK	2006 RBNU	O	RBNU	2006	RBM	11:53	BCCH
HK	2006 RBNU	O	RBNU	2006	RBM	11:53	BCCH
HK	2006 RBNU	O	RBNU	2006	RBM	11:53	BCCH
HK	2006 RBNU	O	RBNU	2006	RBM	11:53	BCCH
HK	2006 RBNU	O	RBNU	2006	RBM	11:53	BCCH
HK	2006 RBNU	O	RBNU	2006	RBM	11:53	BCCH
HK	2006 RBNU	O	RBNU	2006	RBM	11:53	BCCH
HK	2006 RBNU	O	RBNU	2006	RBM	11:53	BCCH
HK	2006 RBNU	O	RBNU	2006	RBM	11:53	BCCH
HK	2006 RBNU	O	RBNU	2006	RBM	11:53	BCCH
HK	2006 RBNU	O	RBNU	2006	RBM	11:53	BCCH
HK	2006 RBNU	O	RBNU	2006	RBM	11:53	BCCH
HK	2006 RBNU	O	RBNU	2006	RBM	11:53	BCCH
HK	2006 RBNU	O	RBNU	2006	RBM	11:53	MOCH
HK	2006 RBNU	O	RBNU	2006	RBM	11:53	BCCH
HK	2006 RBNU	O	RBNU	2006	RBM	11:53	RBNU
HK	2006 RBNU	O	RBNU	2006	RBM	11:53	BCCH

HK	2006 RBNU	O	RBNU	2006	RBM	11:53	BCCH
HK	2006 RBNU	O	RBNU	2006	RBM	11:53	MOCH
HK	2006 RBNU	O	RBNU	2006	RBM	11:53	RBNU
HK	2006 RBNU	O	RBNU	2006	RBM	11:53	RBNU
HK	2006 RBNU	O	RBNU	2006	RBM	11:53	BCCH
HK	2006 RBNU	O	RBNU	2006	RBM	11:53	BCCH
HK	2006 RBNU	O	RBNU	2006	RBM	11:53	BCCH
HK	2006 RBNU	O	RBNU	2006	RBM	11:53	RBNU
HK	2006 RBNU	O	RBNU	2006	RBM	11:53	RBNU
HK	2006 RBNU	O	RBNU	2006	RBM	11:53	BCCH
HK	2006 RBNU	O	RBNU	2006	RBM	11:53	MOCH
HK	2006 RBNU	O	RBNU	2006	RBM	11:53	MOCH
HK	2006 RBNU	O	RBNU	2006	RBM	11:53	
HK	70415	O	RBNU	2007	MBR	9:14	MOCH
HK	70415	O	RBNU	2007	MBR	9:14	MOCH
HK	70415	O	RBNU	2007	MBR	9:14	MOCH
HK	70415	O	RBNU	2007	MBR	9:14	MOCH
HK	70415	O	RBNU	2007	MBR	9:14	MOCH
HK	70415	O	RBNU	2007	MBR	9:14	MOCH
HK	70415	O	RBNU	2007	MBR	9:14	RBNU
HK	70415	O	RBNU	2007	MBR	9:14	RBNU
HK	70415	O	RBNU	2007	MBR	9:14	RBNU
HK	70415	O	RBNU	2007	MBR	9:14	RBNU
HK	70415	O	RBNU	2007	MBR	9:14	RBNU
HK	70415	O	RBNU	2007	MBR	9:14	RBNU
HK	70415	O	RBNU	2007	MBR	9:14	RBNU
HK	70415	O	RBNU	2007	MBR	9:14	RBNU
HK	70415	O	RBNU	2007	MBR	9:14	MOCH
HK	70415	O	RBNU	2007	MBR	9:14	MOCH
HK	70415	O	RBNU	2007	MBR	9:14	RBNU
HK	70415	O	RBNU	2007	MBR	9:14	RBNU
HK	70415	O	RBNU	2007	MBR	9:14	
HK	7086	O	MOBL/EUS	2007	MRB	8:06	MOCH
HK	7086	O	MOBL/EUS	2007	MRB	8:06	MOCH
HK	7086	O	MOBL/EUS	2007	MRB	8:06	MOCH
HK	7086	O	MOBL/EUS	2007	MRB	8:06	MOCH
HK	7086	O	MOBL/EUS	2007	MRB	8:06	MOCH
HK	7086	O	MOBL/EUS	2007	MRB	8:06	MOCH
HK	7086	O	MOBL/EUS	2007	MRB	8:06	MOCH
HK	7086	O	MOBL/EUS	2007	MRB	8:06	MOCH
HK	7086	O	MOBL/EUS	2007	MRB	8:06	MOCH
HK	7086	O	MOBL/EUS	2007	MRB	8:06	MOCH
HK	7086	O	MOBL/EUS	2007	MRB	8:06	MOCH
HK	7086	O	MOBL/EUS	2007	MRB	8:06	MOCH

HK	7086	O	MOBL/EUS	2007	MRB	8:06	BCCH
HK	7086	O	MOBL/EUS	2007	MRB	8:06	RBNU
HK	7086	O	MOBL/EUS	2007	MRB	8:06	RBNU
HK	7086	O	MOBL/EUS	2007	MRB	8:06	RBNU
HK	7086	O	MOBL/EUS	2007	MRB	8:06	RBNU
HK	7086	O	MOBL/EUS	2007	MRB	8:06	RBNU
HK	7086	O	MOBL/EUS	2007	MRB	8:06	RBNU
HK	7086	O	MOBL/EUS	2007	MRB	8:06	RBNU
HK	7086	O	MOBL/EUS	2007	MRB	8:06	RBNU
HK	7086	O	MOBL/EUS	2007	MRB	8:06	RBNU
HK	7086	O	MOBL/EUS	2007	MRB	8:06	RBNU
HK	7086	O	MOBL/EUS	2007	MRB	8:06	RBNU
HK	7086	O	MOBL/EUS	2007	MRB	8:06	RBNU
HK	7086	O	MOBL/EUS	2007	MRB	8:06	RBNU
HK	7086	O	MOBL/EUS	2007	MRB	8:06	RBNU
HK	7086	O	MOBL/EUS	2007	MRB	8:06	RBNU
HK	7086	O	MOBL/EUS	2007	MRB	8:06	RBNU
HK	7086	O	MOBL/EUS	2007	MRB	8:06	RBNU
HK	7086	O	MOBL/EUS	2007	MRB	8:06	RBNU
HK	7086	O	MOBL/EUS	2007	MRB	8:06	RBNU
HK	7086	O	MOBL/EUS	2007	MRB	8:06	RBNU
HK	7086	O	MOBL/EUS	2007	MRB	8:06	RBNU
HK	7086	O	MOBL/EUS	2007	MRB	8:06	RBNU
HK	7086	O	MOBL/EUS	2007	MRB	8:06	BCCH
HK	7086	O	MOBL/EUS	2007	MRB	8:06	BCCH
HK	7086	O	MOBL/EUS	2007	MRB	8:06	BCCH
HK	7086	O	MOBL/EUS	2007	MRB	8:06	MOCH
HK	7086	O	MOBL/EUS	2007	MRB	8:06	BCCH
HK	7086	O	MOBL/EUS	2007	MRB	8:06	MOCH
HK	7086	O	MOBL/EUS	2007	MRB	8:06	MOCH
HK	7086	O	MOBL/EUS	2007	MRB	8:06	RBNU
HK	7086	O	MOBL/EUS	2007	MRB	8:06	BCCH
HK	7086	O	MOBL/EUS	2007	MRB	8:06	BCCH
HK	7086	O	MOBL/EUS	2007	MRB	8:06	
HK	7803	O	MOBL	2007	MBR	10:00	
HK	7803	O	MOBL	2007	MBR	10:00	BCCH
HK	7803	O	MOBL	2007	MBR	10:00	RBNU
HK	7803	O	MOBL	2007	MBR	10:00	RBNU
HK	7803	O	MOBL	2007	MBR	10:00	RBNU
HK	7803	O	MOBL	2007	MBR	10:00	MOCH
HK	7803	O	MOBL	2007	MBR	10:00	BCCH
HK	7803	O	MOBL	2007	MBR	10:00	RBNU
HK	7803	O	MOBL	2007	MBR	10:00	BCCH
HK	7803	O	MOBL	2007	MBR	10:00	BCCH

HK	7803	O	MOBL	2007	MBR	10:00	BCCH
HK	7803	O	MOBL	2007	MBR	10:00	BCCH
HK	7803	O	MOBL	2007	MBR	10:00	BCCH
HK	7803	O	MOBL	2007	MBR	10:00	BCCH
HK	7803	O	MOBL	2007	MBR	10:00	BCCH
HK	7803	O	MOBL	2007	MBR	10:00	BCCH
HK	7803	O	MOBL	2007	MBR	10:00	BCCH
HK	7803	O	MOBL	2007	MBR	10:00	MOCH
HK	7803	O	MOBL	2007	MBR	10:00	BCCH
HK	7803	O	MOBL	2007	MBR	10:00	MOCH
HK	7803	O	MOBL	2007	MBR	10:00	MOCH
HK	7803	O	MOBL	2007	MBR	10:00	MOCH
HK	7803	O	MOBL	2007	MBR	10:00	BCCH
HK	7803	O	MOBL	2007	MBR	10:00	BCCH
HK	7803	O	MOBL	2007	MBR	10:00	BCCH
HK	7803	O	MOBL	2007	MBR	10:00	BCCH
HK	7803	O	MOBL	2007	MBR	10:00	MOCH
HK	7803	O	MOBL	2007	MBR	10:00	BCCH
HK	7803	O	MOBL	2007	MBR	10:00	RBNU
HK	7803	O	MOBL	2007	MBR	10:00	RBNU
HK	7803	O	MOBL	2007	MBR	10:00	MOCH
HK	7803	O	MOBL	2007	MBR	10:00	
HK	7972	O	RBNU	2007	BMR	8:40	MOCH
HK	7972	O	RBNU	2007	BMR	8:40	BCCH
HK	7972	O	RBNU	2007	BMR	8:40	MOCH
HK	7972	O	RBNU	2007	BMR	8:40	MOCH
HK	7972	O	RBNU	2007	BMR	8:40	RBNU
HK	7972	O	RBNU	2007	BMR	8:40	RBNU
HK	7972	O	RBNU	2007	BMR	8:40	RBNU
HK	7972	O	RBNU	2007	BMR	8:40	RBNU
HK	7972	O	RBNU	2007	BMR	8:40	RBNU
HK	7972	O	RBNU	2007	BMR	8:40	BCCH
HK	7972	O	RBNU	2007	BMR	8:40	MOCH
HK	7972	O	RBNU	2007	BMR	8:40	BCCH
HK	7972	O	RBNU	2007	BMR	8:40	MOCH
HK	7972	O	RBNU	2007	BMR	8:40	RBNU
HK	7972	O	RBNU	2007	BMR	8:40	RBNU
HK	7972	O	RBNU	2007	BMR	8:40	
HK	7865/7015	O	MOCH	2007	MRB	9:04	BCCH
HK	7865/7015	O	MOCH	2007	MRB	9:04	BCCH
HK	7865/7015	O	MOCH	2007	MRB	9:04	BCCH
HK	7865/7015	O	MOCH	2007	MRB	9:04	BCCH
HK	7865/7015	O	MOCH	2007	MRB	9:04	BCCH
HK	7865/7015	O	MOCH	2007	MRB	9:04	BCCH

HK	7865/7015	O	MOCH	2007	MRB	9:04	BCCH
HK	7865/7015	O	MOCH	2007	MRB	9:04	BCCH
HK	7865/7015	O	MOCH	2007	MRB	9:04	MOCH
HK	7865/7015	O	MOCH	2007	MRB	9:04	BCCH
HK	7865/7015	O	MOCH	2007	MRB	9:04	MOCH
HK	7865/7015	O	MOCH	2007	MRB	9:04	BCCH
HK	7865/7015	O	MOCH	2007	MRB	9:04	RBNU
HK	7865/7015	O	MOCH	2007	MRB	9:04	RBNU
HK	7865/7015	O	MOCH	2007	MRB	9:04	RBNU
HK	7865/7015	O	MOCH	2007	MRB	9:04	RBNU
HK	7865/7015	O	MOCH	2007	MRB	9:04	RBNU
HK	7865/7015	O	MOCH	2007	MRB	9:04	RBNU
HK	7865/7015	O	MOCH	2007	MRB	9:04	RBNU
HK	7865/7015	O	MOCH	2007	MRB	9:04	RBNU
HK	7865/7015	O	MOCH	2007	MRB	9:04	RBNU
HK	7865/7015	O	MOCH	2007	MRB	9:04	RBNU
HK	7865/7015	O	MOCH	2007	MRB	9:04	RBNU
HK	7865/7015	O	MOCH	2007	MRB	9:04	RBNU
HK	7865/7015	O	MOCH	2007	MRB	9:04	RBNU
HK	7865/7015	O	MOCH	2007	MRB	9:04	RBNU
HK	7865/7015	O	MOCH	2007	MRB	9:04	RBNU
HK	7865/7015	O	MOCH	2007	MRB	9:04	RBNU
HK	7865/7015	O	MOCH	2007	MRB	9:04	BCCH
HK	7865/7015	O	MOCH	2007	MRB	9:04	RBNU
HK	7865/7015	O	MOCH	2007	MRB	9:04	RBNU
HK	7865/7015	O	MOCH	2007	MRB	9:04	BCCH
HK	7865/7015	O	MOCH	2007	MRB	9:04	MOCH
HK	7865/7015	O	MOCH	2007	MRB	9:04	RBNU
HK	7865/7015	O	MOCH	2007	MRB	9:04	BCCH
HK	7865/7015	O	MOCH	2007	MRB	9:04	BCCH
HK	7865/7015	O	MOCH	2007	MRB	9:04	
IB	70502	O			RMB	10:42	BCCH
IB	70502	O			RMB	10:42	MOCH
IB	70502	O			RMB	10:42	MOCH
IB	70502	O			RMB	10:42	BCCH
IB	70502	O			RMB	10:42	MOCH
IB	70502	O			RMB	10:42	MOCH
IB	70502	O			RMB	10:42	MOCH
IB	70502	O			RMB	10:42	MOCH
IB	70502	O			RMB	10:42	MOCH
IB	70502	O			RMB	10:42	MOCH
IB	70502	O			RMB	10:42	MOCH
IB	70502	O			RMB	10:42	MOCH
IB	70502	O			RMB	10:42	RBNU

IB	70502	O	RMB	10:42	BCCH
IB	70502	O	RMB	10:42	BCCH
IB	70502	O	RMB	10:42	BCCH
IB	70502	O	RMB	10:42	RBNU
IB	70502	O	RMB	10:42	RBNU
IB	70502	O	RMB	10:42	RBNU
IB	70502	O	RMB	10:42	RBNU
IB	70502	O	RMB	10:42	RBNU
IB	70502	O	RMB	10:42	RBNU
IB	70502	O	RMB	10:42	RBNU
IB	70502	O	RMB	10:42	MOCH
IB	70502	O	RMB	10:42	MOCH
IB	70502	O	RMB	10:42	BCCH
IB	70502	O	RMB	10:42	RBNU
IB	70502	O	RMB	10:42	RBNU
IB	70502	O	RMB	10:42	MOCH
IB	70502	O	RMB	10:42	RBNU
IB	70502	O	RMB	10:42	MOCH
IB	70502	O	RMB	10:42	BCCH
IB	70502	O	RMB	10:42	BCCH
IB	70502	O	RMB	10:42	RBNU
IB	70502	O	RMB	10:42	RBNU
IB	70502	O	RMB	10:42	
IB	7788	O	BMR	9:13	BCCH
IB	7788	O	BMR	9:13	BCCH
IB	7788	O	BMR	9:13	BCCH
IB	7788	O	BMR	9:13	MOCH
IB	7788	O	BMR	9:13	MOCH
IB	7788	O	BMR	9:13	MOCH
IB	7788	O	BMR	9:13	MOCH
IB	7788	O	BMR	9:13	BCCH
IB	7788	O	BMR	9:13	MOCH
IB	7788	O	BMR	9:13	MOCH
IB	7788	O	BMR	9:13	MOCH
IB	7788	O	BMR	9:13	MOCH
IB	7788	O	BMR	9:13	BCCH
IB	7788	O	BMR	9:13	BCCH
IB	7788	O	BMR	9:13	RBNU
IB	7788	O	BMR	9:13	RBNU
IB	7788	O	BMR	9:13	BCCH
IB	7788	O	BMR	9:13	BCCH
IB	7788	O	BMR	9:13	BCCH
IB	7788	O	BMR	9:13	RBNU
IB	7788	O	BMR	9:13	RBNU

IB	7788	O					BMR	9:13	BCCH
IB	7788	O					BMR	9:13	MOCH
IB	7788	O					BMR	9:13	BCCH
IB	7788	O					BMR	9:13	RBNU
IB	7788	O					BMR	9:13	RBNU
IB	7788	O					BMR	9:13	BCCH
IB	7788	O					BMR	9:13	MOCH
IB	7788	O					BMR	9:13	BCCH
IB	7788	O					BMR	9:13	MOCH
IB	7788	O					BMR	9:13	RBNU
IB	7788	O					BMR	9:13	RBNU
IB	7788	O					BMR	9:13	MOCH
IB	7788	O					BMR	9:13	
	7825	ON	BCCH	04-DG-015	BCCH	2003	BRM	8:15	BCCH
	7825	ON	BCCH	04-DG-015	BCCH	2003	BRM	8:15	BCCH
	7825	ON	BCCH	04-DG-015	BCCH	2003	BRM	8:15	BCCH
	7825	ON	BCCH	04-DG-015	BCCH	2003	BRM	8:15	RBNU
	7825	ON	BCCH	04-DG-015	BCCH	2003	BRM	8:15	RBNU
	7825	ON	BCCH	04-DG-015	BCCH	2003	BRM	8:15	MOCH
	7825	ON	BCCH	04-DG-015	BCCH	2003	BRM	8:15	MOCH
	7825	ON	BCCH	04-DG-015	BCCH	2003	BRM	8:15	BCCH
	7825	ON	BCCH	04-DG-015	BCCH	2003	BRM	8:15	RBNU
	7825	ON	BCCH	04-DG-015	BCCH	2003	BRM	8:15	MOCH
	7825	ON	BCCH	04-DG-015	BCCH	2003	BRM	8:15	MOCH
	7825	ON	BCCH	04-DG-015	BCCH	2003	BRM	8:15	BCCH
	7825	ON	BCCH	04-DG-015	BCCH	2003	BRM	8:15	BCCH
	7825	ON	BCCH	04-DG-015	BCCH	2003	BRM	8:15	BCCH
	7825	ON	BCCH	04-DG-015	BCCH	2003	BRM	8:15	BCCH
	7825	ON	BCCH	04-DG-015	BCCH	2003	BRM	8:15	BCCH
	7825	ON	BCCH	04-DG-015	BCCH	2003	BRM	8:15	RBNU
	7825	ON	BCCH	04-DG-015	BCCH	2003	BRM	8:15	MOCH
	7825	ON	BCCH	04-DG-015	BCCH	2003	BRM	8:15	MOCH
	7825	ON	BCCH	04-DG-015	BCCH	2003	BRM	8:15	BCCH
	7825	ON	BCCH	04-DG-015	BCCH	2003	BRM	8:15	BCCH
	7825	ON	BCCH	04-DG-015	BCCH	2003	BRM	8:15	RBNU
	7825	ON	BCCH	04-DG-015	BCCH	2003	BRM	8:35	BCCH
	7825	ON	BCCH	04-DG-015	BCCH	2003	BRM	8:35	BCCH
	7825	ON	BCCH	04-DG-015	BCCH	2003	BRM	8:35	BCCH
	7825	ON	BCCH	04-DG-015	BCCH	2003	BRM	8:35	BCCH
	7825	ON	BCCH	04-DG-015	BCCH	2003	BRM	8:35	BCCH
	7825	ON	BCCH	04-DG-015	BCCH	2003	BRM	8:35	RBNU
	7825	ON	BCCH	04-DG-015	BCCH	2003	BRM	8:35	MOCH
	7825	ON	BCCH	04-DG-015	BCCH	2003	BRM	8:35	MOCH
	7825	ON	BCCH	04-DG-015	BCCH	2003	BRM	8:35	none

7825	ON	BCCH	04-DG-015	BCCH	2003	BRM	8:35	BCCH
7825	ON	BCCH	04-DG-015	BCCH	2003	BRM	8:35	none
7825	ON	BCCH	04-DG-015	BCCH	2003	BRM	8:35	none
7825	ON	BCCH	04-DG-015	BCCH	2003	BRM	8:35	none
7825	ON	BCCH	04-DG-015	BCCH	2003	BRM	8:35	BCCH
7825	ON	BCCH	04-DG-015	BCCH	2003	BRM	8:35	BCCH
7825	ON	BCCH	04-DG-015	BCCH	2003	BRM	8:35	RBNU
7825	ON	BCCH	04-DG-015	BCCH	2003	BRM	8:35	RBNU
7825	ON	BCCH	04-DG-015	BCCH	2003	BRM	8:35	MOCH
7825	ON	BCCH	04-DG-015	BCCH	2003	BRM	8:35	MOCH
7825	ON	BCCH	04-DG-015	BCCH	2003	BRM	8:35	BCCH
7825	ON	BCCH	04-DG-015	BCCH	2003	BRM	8:35	BCCH
7825	ON	BCCH	04-DG-015	BCCH	2003	BRM	8:35	BCCH
7825	ON	BCCH	04-DG-015	BCCH	2003	BRM	8:35	MOCH
7825	ON	BCCH	04-DG-015	BCCH	2003	BRM	8:35	MOCH
7825	ON	BCCH	04-DG-015	BCCH	2003	BRM	8:35	MOCH
7825	ON	BCCH	04-DG-015	BCCH	2003	BRM	8:35	BCCH
7825	ON	BCCH	04-DG-015	BCCH	2003	BRM	8:35	none
70102	ON	BCCH	04-ARN-04	MOCH	2003	BMR	9:43	none
70102	ON	BCCH	04-ARN-04	MOCH	2003	BMR	9:43	MOCH
70102	ON	BCCH	04-ARN-04	MOCH	2003	BMR	9:43	RBNU
70102	ON	BCCH	04-ARN-04	MOCH	2003	BMR	9:43	RBNU
70102	ON	BCCH	04-ARN-04	MOCH	2003	BMR	9:43	RBNU
70102	ON	BCCH	04-ARN-04	MOCH	2003	BMR	9:43	RBNU
70102	ON	BCCH	04-ARN-04	MOCH	2003	BMR	9:43	BCCH
70102	ON	BCCH	04-ARN-04	MOCH	2003	BMR	9:43	BCCH
70102	ON	BCCH	04-ARN-04	MOCH	2003	BMR	9:43	MOCH
70102	ON	BCCH	04-ARN-04	MOCH	2003	BMR	9:43	RBNU
70102	ON	BCCH	04-ARN-04	MOCH	2003	BMR	9:43	MOCH
70102	ON	BCCH	04-ARN-04	MOCH	2003	BMR	9:43	RBNU
70102	ON	BCCH	04-ARN-04	MOCH	2003	BMR	9:43	RBNU
70102	ON	BCCH	04-ARN-04	MOCH	2003	BMR	9:43	BCCH
70102	ON	BCCH	04-ARN-04	MOCH	2003	BMR	9:43	RBNU
70102	ON	BCCH	04-ARN-04	MOCH	2003	BMR	9:43	RBNU
70102	ON	BCCH	04-ARN-04	MOCH	2003	BMR	9:43	MOCH
70102	ON	BCCH	04-ARN-04	MOCH	2003	BMR	9:43	BCCH
7694	ON	BCCH	05-RV-026	MOCH	2004	MBR	11:24	BCCH
7694	ON	BCCH	05-RV-026	MOCH	2004	MBR	11:24	MOCH
7694	ON	BCCH	05-RV-026	MOCH	2004	MBR	11:24	MOCH
7694	ON	BCCH	05-RV-026	MOCH	2004	MBR	11:24	MOCH
7694	ON	BCCH	05-RV-026	MOCH	2004	MBR	11:24	BCCH
7694	ON	BCCH	05-RV-026	MOCH	2004	MBR	11:24	BCCH
7694	ON	BCCH	05-RV-026	MOCH	2004	MBR	11:24	BCCH
7694	ON	BCCH	05-RV-026	MOCH	2004	MBR	11:24	BCCH

	7694	ON	BCCH	05-RV-026	MOCH	2004	MBR	11:24	MOCH
	7694	ON	BCCH	05-RV-026	MOCH	2004	MBR	11:24	BCCH
	7694	ON	BCCH	05-RV-026	MOCH	2004	MBR	11:24	MOCH
	7928	ON	BCCH	05-MN-029	RBNU	2003	BMR	9:22	BCCH
	7928	ON	BCCH	05-MN-029	RBNU	2003	BMR	9:22	BCCH
	7928	ON	BCCH	05-MN-029	RBNU	2003	BMR	9:22	BCCH
	7928	ON	BCCH	05-MN-029	RBNU	2003	BMR	9:22	MOCH
	7928	ON	BCCH	05-MN-029	RBNU	2003	BMR	9:22	BCCH
	7928	ON	BCCH	05-MN-029	RBNU	2003	BMR	9:22	BCCH
	7928	ON	BCCH	05-MN-029	RBNU	2003	BMR	9:22	MOCH
	7928	ON	BCCH	05-MN-029	RBNU	2003	BMR	9:22	RBNU
	7928	ON	BCCH	05-MN-029	RBNU	2003	BMR	9:22	none
	7928	ON	BCCH	05-MN-029	RBNU	2003	BMR	9:22	none
	7928	ON	BCCH	05-MN-029	RBNU	2003	BMR	9:22	RBNU
	7928	ON	BCCH	05-MN-029	RBNU	2003	BMR	9:22	RBNU
	7928	ON	BCCH	05-MN-029	RBNU	2003	BMR	9:22	RBNU
IB	70004	ON	BCCH	07-ARN-00	BCCH	2006	MBR	9:40 AM	BCCH
IB	70004	ON	BCCH	07-ARN-00	BCCH	2006	MBR	9:40 AM	RBNU
IB	70004	ON	BCCH	07-ARN-00	BCCH	2006	MBR	9:40 AM	BCCH
IB	70004	ON	BCCH	07-ARN-00	BCCH	2006	MBR	9:40 AM	BCCH
IB	70004	ON	BCCH	07-ARN-00	BCCH	2006	MBR	9:40 AM	RBNU
IB	70004	ON	BCCH	07-ARN-00	BCCH	2006	MBR	9:40 AM	BCCH
IB	70004	ON	BCCH	07-ARN-00	BCCH	2006	MBR	9:40 AM	RBNU
IB	70004	ON	BCCH	07-ARN-00	BCCH	2006	MBR	9:40 AM	BCCH
IB	70004	ON	BCCH	07-ARN-00	BCCH	2006	MBR	9:40 AM	MOCH
IB	70004	ON	BCCH	07-ARN-00	BCCH	2006	MBR	9:40 AM	MOCH
IB	70004	ON	BCCH	07-ARN-00	BCCH	2006	MBR	9:40 AM	MOCH
IB	70004	ON	BCCH	07-ARN-00	BCCH	2006	MBR	9:40 AM	MOCH
IB	70004	ON	BCCH	07-ARN-00	BCCH	2006	MBR	9:40 AM	BCCH
IB	70004	ON	BCCH	07-ARN-00	BCCH	2006	MBR	9:40 AM	MOCH
IB	70004	ON	BCCH	07-ARN-00	BCCH	2006	MBR	9:40 AM	MOCH
IB	70004	ON	BCCH	07-ARN-00	BCCH	2006	MBR	9:40 AM	RBNU
IB	70004	ON	BCCH	07-ARN-00	BCCH	2006	MBR	9:40 AM	MOCH
IB	70004	ON	BCCH	07-ARN-00	BCCH	2006	MBR	9:40 AM	BCCH
IB	70004	ON	BCCH	07-ARN-00	BCCH	2006	MBR	9:40 AM	BCCH
IB	70004	ON	BCCH	07-ARN-00	BCCH	2006	MBR	9:40 AM	MOCH
IB	70004	ON	BCCH	07-ARN-00	BCCH	2006	MBR	9:40 AM	MOCH
IB	70004	ON	BCCH	07-ARN-00	BCCH	2006	MBR	9:40 AM	RBNU
IB	70004	ON	BCCH	07-ARN-00	BCCH	2006	MBR	9:40 AM	MOCH
IB	70004	ON	BCCH	07-ARN-00	BCCH	2006	MBR	9:40 AM	MOCH
IB	70004	ON	BCCH	07-ARN-00	BCCH	2006	MBR	9:40 AM	MOCH
IB	70004	ON	BCCH	07-ARN-00	BCCH	2006	MBR	9:40 AM	RBNU
IB	70004	ON	BCCH	07-ARN-00	BCCH	2006	MBR	9:40 AM	MOCH
IB	70004	ON	BCCH	07-ARN-00	BCCH	2006	MBR	9:40 AM	MOCH

IB	70004	ON	BCCH	07-ARN-00	BCCH	2006	MBR	9:40 AM	RBNU
IB	70004	ON	BCCH	07-ARN-00	BCCH	2006	MBR	9:40 AM	RBNU
IB	70004	ON	BCCH	07-ARN-00	BCCH	2006	MBR	9:40 AM	RBNU
IB	70004	ON	BCCH	07-ARN-00	BCCH	2006	MBR	9:40 AM	RBNU
IB	70004	ON	BCCH	07-ARN-00	BCCH	2006	MBR	9:40 AM	RBNU
IB	70004	ON	BCCH	07-ARN-00	BCCH	2006	MBR	9:40 AM	RBNU
IB	70004	ON	BCCH	07-ARN-00	BCCH	2006	MBR	9:40 AM	RBNU
IB	70004	ON	BCCH	07-ARN-00	BCCH	2006	MBR	9:40 AM	BCCH
IB	70004	ON	BCCH	07-ARN-00	BCCH	2006	MBR	9:40 AM	RBNU
IB	70004	ON	BCCH	07-ARN-00	BCCH	2006	MBR	9:40 AM	RBNU
IB	70004	ON	BCCH	07-ARN-00	BCCH	2006	MBR	9:40 AM	MOCH
IB	70004	ON	BCCH	07-ARN-00	BCCH	2006	MBR	9:40 AM	RBNU
IB	70004	ON	BCCH	07-ARN-00	BCCH	2006	MBR	9:40 AM	MOCH
IB	70004	ON	BCCH	07-ARN-00	BCCH	2006	MBR	9:40 AM	BCCH
IB	70004	ON	BCCH	07-ARN-00	BCCH	2006	MBR	9:40 AM	RBNU
IB	7840	ON	BCCH	07-KS-054	MOCH	2006	MBR	8:30 AM	BCCH
IB	7840	ON	BCCH	07-KS-054	MOCH	2006	MBR	8:30 AM	BCCH
IB	7840	ON	BCCH	07-KS-054	MOCH	2006	MBR	8:30 AM	BCCH
IB	7840	ON	BCCH	07-KS-054	MOCH	2006	MBR	8:30 AM	BCCH
IB	7840	ON	BCCH	07-KS-054	MOCH	2006	MBR	8:30 AM	BCCH
IB	7840	ON	BCCH	07-KS-054	MOCH	2006	MBR	8:30 AM	BCCH
IB	7840	ON	BCCH	07-KS-054	MOCH	2006	MBR	8:30 AM	BCCH
IB	7840	ON	BCCH	07-KS-054	MOCH	2006	MBR	8:30 AM	BCCH
IB	7840	ON	BCCH	07-KS-054	MOCH	2006	MBR	8:30 AM	BCCH
IB	7840	ON	BCCH	07-KS-054	MOCH	2006	MBR	8:30 AM	BCCH
IB	7840	ON	BCCH	07-KS-054	MOCH	2006	MBR	8:30 AM	BCCH
IB	7840	ON	BCCH	07-KS-054	MOCH	2006	MBR	8:30 AM	BCCH
IB	7840	ON	BCCH	07-KS-054	MOCH	2006	MBR	8:30 AM	BCCH
IB	7840	ON	BCCH	07-KS-054	MOCH	2006	MBR	8:30 AM	BCCH
IB	7840	ON	BCCH	07-KS-054	MOCH	2006	MBR	8:30 AM	BCCH
IB	7840	ON	BCCH	07-KS-054	MOCH	2006	MBR	8:30 AM	BCCH
IB	7840	ON	BCCH	07-KS-054	MOCH	2006	MBR	8:30 AM	BCCH
IB	7840	ON	BCCH	07-KS-054	MOCH	2006	MBR	8:30 AM	BCCH
IB	7840	ON	BCCH	07-KS-054	MOCH	2006	MBR	8:30 AM	BCCH
IB	7840	ON	BCCH	07-KS-054	MOCH	2006	MBR	8:30 AM	MOCH
IB	7840	ON	BCCH	07-KS-054	MOCH	2006	MBR	8:30 AM	BCCH
IB	7840	ON	BCCH	07-KS-054	MOCH	2006	MBR	8:30 AM	RBNU
IB	70004	ON	BCCH	07-ARN-00	BCCH	2006	MRB	10:27 AM	RBNU
IB	70004	ON	BCCH	07-ARN-00	BCCH	2006	MRB	10:27 AM	BCCH
IB	70004	ON	BCCH	07-ARN-00	BCCH	2006	MRB	10:27 AM	BCCH
IB	70004	ON	BCCH	07-ARN-00	BCCH	2006	MRB	10:27 AM	BCCH
IB	70004	ON	BCCH	07-ARN-00	BCCH	2006	MRB	10:27 AM	BCCH
IB	70004	ON	BCCH	07-ARN-00	BCCH	2006	MRB	10:27 AM	BCCH
IB	70004	ON	BCCH	07-ARN-00	BCCH	2006	MRB	10:27 AM	RBNU

IB	70004	ON	BCCH	07-ARN-00	BCCH	2006	MRB	10:27 AM	RBNU
IB	70004	ON	BCCH	07-ARN-00	BCCH	2006	MRB	10:27 AM	MOCH
IB	70004	ON	BCCH	07-ARN-00	BCCH	2006	MRB	10:27 AM	MOCH
IB	70004	ON	BCCH	07-ARN-00	BCCH	2006	MRB	10:27 AM	RBNU
IB	70004	ON	BCCH	07-ARN-00	BCCH	2006	MRB	10:27 AM	MOCH
IB	70004	ON	BCCH	07-ARN-00	BCCH	2006	MRB	10:27 AM	MOCH
IB	70004	ON	BCCH	07-ARN-00	BCCH	2006	MRB	10:27 AM	MOCH
IB	70004	ON	BCCH	07-ARN-00	BCCH	2006	MRB	10:27 AM	RBNU
IB	70004	ON	BCCH	07-ARN-00	BCCH	2006	MRB	10:27 AM	MOCH
IB	70004	ON	BCCH	07-ARN-00	BCCH	2006	MRB	10:27 AM	MOCH
IB	70004	ON	BCCH	07-ARN-00	BCCH	2006	MRB	10:27 AM	RBNU
IB	70004	ON	BCCH	07-ARN-00	BCCH	2006	MRB	10:27 AM	RBNU
IB	70004	ON	BCCH	07-ARN-00	BCCH	2006	MRB	10:27 AM	RBNU
IB	70004	ON	BCCH	07-ARN-00	BCCH	2006	MRB	10:27 AM	RBNU
IB	70004	ON	BCCH	07-ARN-00	BCCH	2006	MRB	10:27 AM	RBNU
IB	70004	ON	BCCH	07-ARN-00	BCCH	2006	MRB	10:27 AM	RBNU
IB	70004	ON	BCCH	07-ARN-00	BCCH	2006	MRB	10:27 AM	RBNU
IB	70004	ON	BCCH	07-ARN-00	BCCH	2006	MRB	10:27 AM	BCCH
IB	70004	ON	BCCH	07-ARN-00	BCCH	2006	MRB	10:27 AM	BCCH
IB	70004	ON	BCCH	07-ARN-00	BCCH	2006	MRB	10:27 AM	RBNU
IB	70004	ON	BCCH	07-ARN-00	BCCH	2006	MRB	10:27 AM	RBNU
IB	70004	ON	BCCH	07-ARN-00	BCCH	2006	MRB	10:27 AM	BCCH
IB	70004	ON	BCCH	07-ARN-00	BCCH	2006	MRB	10:27 AM	MOCH
IB	70004	ON	BCCH	07-ARN-00	BCCH	2006	MRB	10:27 AM	RBNU
ARN	70004	ON	BCCH	08-ARN-02	BCCH	2007	MBR	12:38	MOCH
ARN	70004	ON	BCCH	08-ARN-02	BCCH	2007	MBR	12:38	MOCH
ARN	70004	ON	BCCH	08-ARN-02	BCCH	2007	MBR	12:38	MOCH
ARN	70004	ON	BCCH	08-ARN-02	BCCH	2007	MBR	12:38	MOCH
ARN	70004	ON	BCCH	08-ARN-02	BCCH	2007	MBR	12:38	MOCH
ARN	70004	ON	BCCH	08-ARN-02	BCCH	2007	MBR	12:38	MOCH
ARN	70004	ON	BCCH	08-ARN-02	BCCH	2007	MBR	12:38	MOCH
ARN	70004	ON	BCCH	08-ARN-02	BCCH	2007	MBR	12:38	MOCH
ARN	70004	ON	BCCH	08-ARN-02	BCCH	2007	MBR	12:38	BCCH
ARN	70004	ON	BCCH	08-ARN-02	BCCH	2007	MBR	12:38	MOCH
ARN	70004	ON	BCCH	08-ARN-02	BCCH	2007	MBR	12:38	RBNU
ARN	70004	ON	BCCH	08-ARN-02	BCCH	2007	MBR	12:38	RBNU
ARN	70004	ON	BCCH	08-ARN-02	BCCH	2007	MBR	12:38	MOCH
ARN	70004	ON	BCCH	08-ARN-02	BCCH	2007	MBR	12:38	BCCH
ARN	70004	ON	BCCH	08-ARN-02	BCCH	2007	MBR	12:38	RBNU
ARN	70004	ON	BCCH	08-ARN-02	BCCH	2007	MBR	12:38	RBNU
ARN	70004	ON	BCCH	08-ARN-02	BCCH	2007	MBR	12:38	RBNU
ARN	70004	ON	BCCH	08-ARN-02	BCCH	2007	MBR	12:38	
ARN	70004	ON	BCCH	08-ARN-02	BCCH	2007	MBR	12:38	MOCH
	7656	ON	MOCH		RBNU	2001	BRM	9:10	BCCH

7656	ON	MOCH	RBNU	2001	BRM	9:10	RBNU
7656	ON	MOCH	RBNU	2001	BRM	9:10	RBNU
7656	ON	MOCH	RBNU	2001	BRM	9:10	MOCH
7656	ON	MOCH	RBNU	2001	BRM	9:10	BCCH
7656	ON	MOCH	RBNU	2001	BRM	9:10	RBNU
7656	ON	MOCH	RBNU	2001	BRM	9:10	BCCH
7656	ON	MOCH	RBNU	2001	BRM	9:10	BCCH
7679	ON	MOCH	MOCH	2002	RMB	9:50	MOCH
7679	ON	MOCH	MOCH	2002	RMB	9:50	none
7679	ON	MOCH	MOCH	2002	RMB	9:50	RBNU
7679	ON	MOCH	MOCH	2002	RMB	9:50	MOCH
7679	ON	MOCH	MOCH	2002	RMB	9:50	MOCH
7679	ON	MOCH	MOCH	2002	RMB	9:50	BCCH
7679	ON	MOCH	MOCH	2002	RMB	9:50	BCCH
7679	ON	MOCH	MOCH	2002	RMB	9:50	RBNU
7679	ON	MOCH	MOCH	2002	RMB	9:50	MOCH
7679	ON	MOCH	MOCH	2002	RMB	9:50	RBNU
7679	ON	MOCH	MOCH	2002	RMB	9:50	RBNU
7679	ON	MOCH	MOCH	2002	RMB	9:50	RBNU
7679	ON	MOCH	MOCH	2002	RMB	9:50	RBNU
7679	ON	MOCH	MOCH	2002	RMB	9:50	RBNU
7679	ON	MOCH	MOCH	2002	RMB	9:50	RBNU
7679	ON	MOCH	MOCH	2002	RMB	9:50	RBNU
7679	ON	MOCH	MOCH	2002	RMB	9:50	MOCH
7679	ON	MOCH	MOCH	2002	RMB	9:50	RBNU
7679	ON	MOCH	MOCH	2002	RMB	9:50	RBNU
7679	ON	MOCH	MOCH	2002	RMB	9:50	RBNU
7679	ON	MOCH	MOCH	2002	RMB	9:50	RBNU
7679	ON	MOCH	MOCH	2002	RMB	9:50	RBNU
7679	ON	MOCH	MOCH	2002	RMB	9:50	MOCH
7679	ON	MOCH	MOCH	2002	RMB	9:50	MOCH
7679	ON	MOCH	MOCH	2002	RMB	9:50	MOCH
7679	ON	MOCH	MOCH	2002	RMB	9:50	BCCH
7130	ON	MOCH	DOWO	2003	MBR	8:45:00	MOCH
7130	ON	MOCH	DOWO	2003	MBR	8:45:00	MOCH
7130	ON	MOCH	DOWO	2003	MBR	8:45:00	MOCH
7130	ON	MOCH	DOWO	2003	MBR	8:45:00	RBNU
7130	ON	MOCH	DOWO	2003	MBR	8:45:00	RBNU
7130	ON	MOCH	DOWO	2003	MBR	8:45:00	BCCH
7130	ON	MOCH	DOWO	2003	MBR	8:45:00	none
7130	ON	MOCH	DOWO	2003	MBR	8:45:00	none
7130	ON	MOCH	DOWO	2003	MBR	8:45:00	MOCH
7130	ON	MOCH	DOWO	2003	MBR	8:45:00	MOCH
7130	ON	MOCH	DOWO	2003	MBR	8:45:00	MOCH

7130	ON	MOCH		DOWO	2003	MBR	8:45:00	BCCH
7130	ON	MOCH		DOWO	2003	MBR	8:45:00	BCCH
7130	ON	MOCH		DOWO	2003	MBR	8:45:00	BCCH
7130	ON	MOCH		DOWO	2003	MBR	8:45:00	RBNU
7130	ON	MOCH		DOWO	2003	MBR	8:45:00	none
7130	ON	MOCH		DOWO	2003	MBR	8:45:00	none
7130	ON	MOCH		DOWO	2003	MBR	8:45:00	RBNU
7130	ON	MOCH	04-ARN-03	DOWO	2003	BRM	9:22:05	none
7130	ON	MOCH	04-ARN-03	DOWO	2003	BRM	9:22:05	none
7130	ON	MOCH	04-ARN-03	DOWO	2003	BRM	9:22:05	MOCH
7130	ON	MOCH	04-ARN-03	DOWO	2003	BRM	9:22:05	MOCH
7130	ON	MOCH	04-ARN-03	DOWO	2003	BRM	9:22:05	MOCH
7825	ON	MOCH	05-ARN-00	BCCH	2004	RBM	9:02	RBNU
7825	ON	MOCH	05-ARN-00	BCCH	2004	RBM	9:02	BCCH
7825	ON	MOCH	05-ARN-00	BCCH	2004	RBM	9:02	BCCH
7825	ON	MOCH	05-ARN-00	BCCH	2004	RBM	9:02	MOCH
7825	ON	MOCH	05-ARN-00	BCCH	2004	RBM	9:02	BCCH
7825	ON	MOCH	05-ARN-00	BCCH	2004	RBM	9:02	MOCH
7825	ON	MOCH	05-ARN-00	BCCH	2004	RBM	9:02	MOCH
7825	ON	MOCH	05-ARN-00	BCCH	2004	RBM	9:02	RBNU
7825	ON	MOCH	05-ARN-00	BCCH	2004	RBM	9:02	RBNU
7825	ON	MOCH	05-ARN-00	BCCH	2004	RBM	9:02	RBNU
7825	ON	MOCH	05-ARN-00	BCCH	2004	RBM	9:02	BCCH
7825	ON	MOCH	05-ARN-00	BCCH	2004	RBM	9:02	RBNU
7825	ON	MOCH	05-ARN-00	BCCH	2004	RBM	9:02	MOCH
7825	ON	MOCH	05-ARN-00	BCCH	2004	RBM	9:02	BCCH
7825	ON	MOCH	05-ARN-00	BCCH	2004	RBM	9:02	MOCH
7825	ON	MOCH	05-ARN-00	BCCH	2004	RBM	9:02	none
7825	ON	MOCH	05-ARN-00	BCCH	2004	RBM	9:02	RBNU
70507	ON	MOCH	05-ARN-02	BCCH	2004	MRB	8:12	MOCH
70507	ON	MOCH	05-ARN-02	BCCH	2004	MRB	8:12	MOCH
70507	ON	MOCH	05-ARN-02	BCCH	2004	MRB	8:12	MOCH
70507	ON	MOCH	05-ARN-02	BCCH	2004	MRB	8:12	MOCH
70507	ON	MOCH	05-ARN-02	BCCH	2004	MRB	8:12	MOCH
70507	ON	MOCH	05-ARN-02	BCCH	2004	MRB	8:12	MOCH
70507	ON	MOCH	05-ARN-02	BCCH	2004	MRB	8:12	MOCH
70507	ON	MOCH	05-ARN-02	BCCH	2004	MRB	8:12	MOCH
70507	ON	MOCH	05-ARN-02	BCCH	2004	MRB	8:12	none
70507	ON	MOCH	05-ARN-02	BCCH	2004	MRB	8:12	BCCH
70507	ON	MOCH	05-ARN-02	BCCH	2004	MRB	8:12	MOCH
70507	ON	MOCH	05-ARN-02	BCCH	2004	MRB	8:12	RBNU
70507	ON	MOCH	05-ARN-02	BCCH	2004	MRB	8:12	RBNU
70507	ON	MOCH	05-ARN-02	BCCH	2004	MRB	8:12	RBNU
70507	ON	MOCH	05-ARN-02	BCCH	2004	MRB	8:12	MOCH

	70507	ON	MOCH	05-ARN-02	BCCH	2004	MRB	8:12	MOCH
	70507	ON	MOCH	05-ARN-02	BCCH	2004	MRB	8:12	none
	70102	ON	MOCH	05-ARN-02	BCCH	2004	RBM	10:29	BCCH
	70102	ON	MOCH	05-ARN-02	BCCH	2004	RBM	10:29	MOCH
	70102	ON	MOCH	05-ARN-02	BCCH	2004	RBM	10:29	MOCH
	70102	ON	MOCH	05-ARN-02	BCCH	2004	RBM	10:29	BCCH
	70102	ON	MOCH	05-ARN-02	BCCH	2004	RBM	10:29	BCCH
	70102	ON	MOCH	05-ARN-02	BCCH	2004	RBM	10:29	BCCH
	70102	ON	MOCH	05-ARN-02	BCCH	2004	RBM	10:29	none
	70102	ON	MOCH	05-ARN-02	BCCH	2004	RBM	10:29	none
	70102	ON	MOCH	05-ARN-02	BCCH	2004	RBM	10:29	RBNU
	70102	ON	MOCH	05-ARN-02	BCCH	2004	RBM	8:50	RBNU
	70102	ON	MOCH	05-ARN-02	BCCH	2004	RBM	8:50	BCCH
	70102	ON	MOCH	05-ARN-02	BCCH	2004	RBM	8:50	MOCH
	70102	ON	MOCH	05-ARN-02	BCCH	2004	RBM	8:50	RBNU
	70102	ON	MOCH	05-ARN-02	BCCH	2004	RBM	8:50	BCCH
	70102	ON	MOCH	05-ARN-02	BCCH	2004	RBM	8:50	none
	70102	ON	MOCH	05-ARN-02	BCCH	2004	RBM	8:50	none
	70102	ON	MOCH	05-ARN-02	BCCH	2004	RBM	8:50	none
	70102	ON	MOCH	05-ARN-02	BCCH	2004	RBM	8:50	none
	7280	ON	MOCH	05-MN-005	RBNU	2003	RMB	10:18	MOCH
	7280	ON	MOCH	05-MN-005	RBNU	2003	RMB	10:18	MOCH
	7280	ON	MOCH	05-MN-005	RBNU	2003	RMB	10:18	MOCH
	7280	ON	MOCH	05-MN-005	RBNU	2003	RMB	10:18	MOCH
	7280	ON	MOCH	05-MN-005	RBNU	2003	RMB	10:18	MOCH
	7280	ON	MOCH	05-MN-005	RBNU	2003	RMB	10:18	RBNU
	7280	ON	MOCH	05-MN-005	RBNU	2003	RMB	10:18	MOCH
	7280	ON	MOCH	05-MN-005	RBNU	2003	RMB	10:18	MOCH
	7280	ON	MOCH	05-MN-005	RBNU	2003	RMB	10:18	BCCH
	7280	ON	MOCH	05-MN-005	RBNU	2003	RMB	10:18	BCCH
	7280	ON	MOCH	05-MN-005	RBNU	2003	RMB	10:18	BCCH
	7280	ON	MOCH	05-MN-005	RBNU	2003	RMB	10:18	RBNU
	7280	ON	MOCH	05-MN-005	RBNU	2003	RMB	10:18	BCCH
	7280	ON	MOCH	05-MN-005	RBNU	2003	RMB	10:18	RBNU
	7280	ON	MOCH	05-MN-005	RBNU	2003	RMB	10:18	BCCH
	7280	ON	MOCH	05-MN-005	RBNU	2003	RMB	10:18	RBNU
	7280	ON	MOCH	05-MN-005	RBNU	2003	RMB	10:18	BCCH
	7280	ON	MOCH	05-MN-005	RBNU	2003	RMB	10:18	MOCH
ARN	7020	ON	MOCH	05-ARN-00	MOCH	2003	MRB	10:55	BCCH
ARN	7020	ON	MOCH	05-ARN-00	MOCH	2003	MRB	10:55	BCCH
ARN	7020	ON	MOCH	05-ARN-00	MOCH	2003	MRB	10:55	none
ARN	7020	ON	MOCH	05-ARN-00	MOCH	2003	MRB	10:55	RBNU
ARN	7020	ON	MOCH	05-ARN-00	MOCH	2003	MRB	10:55	MOCH
ARN	7020	ON	MOCH	05-ARN-00	MOCH	2003	MRB	10:55	MOCH

ARN	7020	ON	MOCH	05-ARN-00	MOCH	2003	MRB	10:55	BCCH
ARN	7020	ON	MOCH	05-ARN-00	MOCH	2003	MRB	10:55	BCCH
ARN	7020	ON	MOCH	05-ARN-00	MOCH	2003	MRB	10:55	RBNU
ARN	7020	ON	MOCH	05-ARN-00	MOCH	2003	MRB	10:55	RBNU
ARN	7020	ON	MOCH	05-ARN-00	MOCH	2003	MRB	10:55	BCCH
ARN	7020	ON	MOCH	05-ARN-00	MOCH	2003	MRB	10:55	BCCH
ARN	7020	ON	MOCH	05-ARN-00	MOCH	2003	MRB	10:55	BCCH
ARN	7020	ON	MOCH	05-ARN-00	MOCH	2003	MRB	10:55	BCCH
ARN	7020	ON	MOCH	05-ARN-00	MOCH	2003	MRB	10:55	MOCH
ARN	7020	ON	MOCH	05-ARN-00	MOCH	2003	MRB	10:55	MOCH
ARN	7020	ON	MOCH	05-ARN-00	MOCH	2003	MRB	10:55	BCCH
ARN	7020	ON	MOCH	05-ARN-00	MOCH	2003	MRB	10:55	RBNU
ARN	7020	ON	MOCH	05-ARN-00	MOCH	2003	MRB	10:55	BCCH
ARN	7020	ON	MOCH	05-ARN-00	MOCH	2003	MRB	10:55	BCCH
	7040	ON	MOCH	05-PAR-00	RBNU	2004	BMR	7:47	BCCH
	7040	ON	MOCH	05-PAR-00	RBNU	2004	BMR	7:47	BCCH
	7040	ON	MOCH	05-PAR-00	RBNU	2004	BMR	7:47	none
	7040	ON	MOCH	05-PAR-00	RBNU	2004	BMR	7:47	none
	7040	ON	MOCH	05-PAR-00	RBNU	2004	BMR	7:47	BCCH
	7040	ON	MOCH	05-PAR-00	RBNU	2004	BMR	7:47	BCCH
	7040	ON	MOCH	05-PAR-00	RBNU	2004	BMR	7:47	BCCH
	7040	ON	MOCH	05-PAR-00	RBNU	2004	BMR	7:47	MOCH
	7040	ON	MOCH	05-PAR-00	RBNU	2004	BMR	7:47	MOCH
	7040	ON	MOCH	05-PAR-00	RBNU	2004	BMR	7:47	MOCH
	7040	ON	MOCH	05-PAR-00	RBNU	2004	BMR	7:47	MOCH
	7040	ON	MOCH	05-PAR-00	RBNU	2004	BMR	7:47	MOCH
	7040	ON	MOCH	05-PAR-00	RBNU	2004	BMR	7:47	RBNU
	7040	ON	MOCH	05-PAR-00	RBNU	2004	BMR	7:47	RBNU
	7040	ON	MOCH	05-PAR-00	RBNU	2004	BMR	7:47	RBNU
	7040	ON	MOCH	05-PAR-00	RBNU	2004	BMR	7:47	MOCH
	7040	ON	MOCH	05-PAR-00	RBNU	2004	BMR	7:47	RBNU
	7040	ON	MOCH	05-PAR-00	RBNU	2004	BMR	7:47	MOCH
	7040	ON	MOCH	05-PAR-00	RBNU	2004	BMR	7:47	MOCH
	7040	ON	MOCH	05-PAR-00	RBNU	2004	BMR	7:47	MOCH
	7670	ON	MOCH	05-ARN-01	MOCH	2004	BRM	7:59	RBNU
	7670	ON	MOCH	05-ARN-01	MOCH	2004	BRM	7:59	MOCH
	7670	ON	MOCH	05-ARN-01	MOCH	2004	BRM	7:59	MOCH
	7670	ON	MOCH	05-ARN-01	MOCH	2004	BRM	7:59	MOCH
	7670	ON	MOCH	05-ARN-01	MOCH	2004	BRM	7:59	MOCH
	7670	ON	MOCH	05-ARN-01	MOCH	2004	BRM	7:59	none
	7670	ON	MOCH	05-ARN-01	MOCH	2004	BRM	7:59	BCCH
	7670	ON	MOCH	05-ARN-01	MOCH	2004	BRM	7:59	RBNU
	7670	ON	MOCH	05-ARN-01	MOCH	2004	BRM	7:59	RBNU
	7670	ON	MOCH	05-ARN-01	MOCH	2004	BRM	7:59	MOCH

7670	ON	MOCH	05-ARN-01	MOCH	2004	BRM	7:59	BCCH
7670	ON	MOCH	05-ARN-01	MOCH	2004	BRM	7:59	MOCH
7670	ON	MOCH	05-ARN-01	MOCH	2004	BRM	7:59	none
7670	ON	MOCH	05-ARN-01	MOCH	2004	BRM	7:59	RBNU
7670	ON	MOCH	05-ARN-01	MOCH	2004	BRM	7:59	MOCH
7855	ON	MOCH	05-RV-054	MOCH	2004	RMB	11:30	MOCH
7855	ON	MOCH	05-RV-054	MOCH	2004	RMB	11:30	BCCH
7855	ON	MOCH	05-RV-054	MOCH	2004	RMB	11:30	BCCH
7855	ON	MOCH	05-RV-054	MOCH	2004	RMB	11:30	BCCH
7855	ON	MOCH	05-RV-054	MOCH	2004	RMB	11:30	BCCH
7855	ON	MOCH	05-RV-054	MOCH	2004	RMB	11:30	MOCH
7855	ON	MOCH	05-RV-054	MOCH	2004	RMB	11:30	BCCH
7855	ON	MOCH	05-RV-054	MOCH	2004	RMB	11:30	BCCH
70502	ON	MOCH	05-ARN-01	RBNU	2004	RMB	12:13	RBNU
70502	ON	MOCH	05-ARN-01	RBNU	2004	RMB	12:13	MOCH
70502	ON	MOCH	05-ARN-01	RBNU	2004	RMB	12:13	BCCH
70502	ON	MOCH	05-ARN-01	RBNU	2004	RMB	12:13	BCCH
70502	ON	MOCH	05-ARN-01	RBNU	2004	RMB	12:13	MOCH
70502	ON	MOCH	05-ARN-01	RBNU	2004	RMB	12:13	BCCH
70502	ON	MOCH	05-ARN-01	RBNU	2004	RMB	12:13	BCCH
70502	ON	MOCH	05-ARN-01	RBNU	2004	RMB	12:13	RBNU
70502	ON	MOCH	05-ARN-01	RBNU	2004	RMB	12:13	RBNU
70502	ON	MOCH	05-ARN-01	RBNU	2004	RMB	12:13	none
7656	ON	MOCH	05-MN-013	MOCH	2004	MBR	8:30	BCCH
7656	ON	MOCH	05-MN-013	MOCH	2004	MBR	8:30	BCCH
7656	ON	MOCH	05-MN-013	MOCH	2004	MBR	8:30	MOCH
7656	ON	MOCH	05-MN-013	MOCH	2004	MBR	8:30	BCCH
7656	ON	MOCH	05-MN-013	MOCH	2004	MBR	8:30	RBNU
7656	ON	MOCH	05-MN-013	MOCH	2004	MBR	8:30	MOCH
7656	ON	MOCH	05-MN-013	MOCH	2004	MBR	8:30	MOCH
7656	ON	MOCH	05-MN-013	MOCH	2004	MBR	8:30	RBNU
7656	ON	MOCH	05-MN-013	MOCH	2004	MBR	8:30	BCCH
7656	ON	MOCH	05-MN-013	MOCH	2004	MBR	8:30	MOCH
7656	ON	MOCH	05-MN-013	MOCH	2004	MBR	8:30	MOCH
7656	ON	MOCH	05-MN-013	MOCH	2004	MBR	8:30	MOCH
7656	ON	MOCH	05-MN-013	MOCH	2004	MBR	8:30	RBNU
70734	ON	MOCH	05-ARN-00	DOWO	2004	MRB	8:57	BCCH
70734	ON	MOCH	05-ARN-00	DOWO	2004	MRB	8:57	MOCH
70734	ON	MOCH	05-ARN-00	DOWO	2004	MRB	8:57	MOCH
70734	ON	MOCH	05-ARN-00	DOWO	2004	MRB	8:57	MOCH
70734	ON	MOCH	05-ARN-00	DOWO	2004	MRB	8:57	MOCH
70734	ON	MOCH	05-ARN-00	DOWO	2004	MRB	8:57	MOCH
70734	ON	MOCH	05-ARN-00	DOWO	2004	MRB	8:57	MOCH
70734	ON	MOCH	05-ARN-00	DOWO	2004	MRB	8:57	RBNU

70734	ON	MOCH	05-ARN-00	DOWO	2004	MRB	8:57	none
70734	ON	MOCH	05-ARN-00	DOWO	2004	MRB	8:57	MOCH
70734	ON	MOCH	05-ARN-00	DOWO	2004	MRB	8:57	BCCH
70734	ON	MOCH	05-ARN-00	DOWO	2004	MRB	8:57	MOCH
70734	ON	MOCH	05-ARN-00	DOWO	2004	MRB	8:57	RBNU
70734	ON	MOCH	05-ARN-00	DOWO	2004	MRB	8:57	RBNU
70734	ON	MOCH	05-ARN-00	DOWO	2004	MRB	8:57	none
70734	ON	MOCH	05-ARN-00	DOWO	2004	MRB	8:57	MOCH
70734	ON	MOCH	05-ARN-00	DOWO	2004	MRB	8:57	MOCH
70110	ON	MOCH	05-CW-01(	DOWO	2004	MBR	12:50	BCCH
70110	ON	MOCH	05-CW-01(	DOWO	2004	MBR	12:50	MOCH
70110	ON	MOCH	05-CW-01(	DOWO	2004	MBR	12:50	BCCH
70110	ON	MOCH	05-CW-01(	DOWO	2004	MBR	12:50	MOCH
70110	ON	MOCH	05-CW-01(	DOWO	2004	MBR	12:50	MOCH
70110	ON	MOCH	05-CW-01(	DOWO	2004	MBR	12:50	MOCH
70110	ON	MOCH	05-CW-01(	DOWO	2004	MBR	12:50	BCCH
70110	ON	MOCH	05-CW-01(	DOWO	2004	MBR	12:50	RBNU
70110	ON	MOCH	05-CW-01(	DOWO	2004	MBR	12:50	BCCH
70110	ON	MOCH	05-CW-01(	DOWO	2004	MBR	12:50	MOCH
70110	ON	MOCH	05-CW-01(	DOWO	2004	MBR	12:50	MOCH
70110	ON	MOCH	05-CW-01(	DOWO	2004	MBR	12:50	BCCH
70110	ON	MOCH	05-CW-01(	DOWO	2004	MBR	12:50	RBNU
70110	ON	MOCH	05-CW-01(	DOWO	2004	MBR	12:50	MOCH
70110	ON	MOCH	05-CW-01(	DOWO	2004	MBR	12:50	MOCH
70110	ON	MOCH	05-CW-01(	DOWO	2004	MBR	12:50	RBNU
70110	ON	MOCH	05-CW-01(	DOWO	2004	MBR	12:50	RBNU
70110	ON	MOCH	05-CW-01(	DOWO	2004	MBR	12:50	BCCH
70110	ON	MOCH	05-CW-01(	DOWO	2004	MBR	12:50	BCCH
70110	ON	MOCH	05-CW-01(	DOWO	2004	MBR	12:50	BCCH
7255	ON	MOCH	05-PAR-01	MOCH	2004	RMB	15:15	MOCH
7255	ON	MOCH	05-PAR-01	MOCH	2004	RMB	15:15	MOCH
70702	ON	MOCH	05-PAR-01	MOCH	2004	RMB	15:15	MOCH
70702	ON	MOCH	05-PAR-01	MOCH	2004	RMB	15:15	MOCH
7255	ON	MOCH	05-PAR-01	MOCH	2004	RMB	15:15	RBNU
70702	ON	MOCH	05-PAR-01	MOCH	2004	RMB	15:15	RBNU
7255	ON	MOCH	05-PAR-01	MOCH	2004	RMB	15:15	none
7255	ON	MOCH	05-PAR-01	MOCH	2004	RMB	15:15	RBNU
7255	ON	MOCH	05-PAR-01	MOCH	2004	RMB	15:15	MOCH
70702	ON	MOCH	05-PAR-01	MOCH	2004	RMB	15:15	none
70702	ON	MOCH	05-PAR-01	MOCH	2004	RMB	15:15	RBNU
70702	ON	MOCH	05-PAR-01	MOCH	2004	RMB	15:15	MOCH
7744	ON	MOCH	05-MN-012	MOCH	2004	BMR	9:17	RBNU
7744	ON	MOCH	05-MN-012	MOCH	2004	BMR	9:17	MOCH
7825	ON	MOCH	05-ARN-00	BCCH	2004	RMB	11:25	MOCH

7825	ON	MOCH	05-ARN-00	BCCH	2004	RMB	11:25	BCCH
7825	ON	MOCH	05-ARN-00	BCCH	2004	RMB	11:25	BCCH
7825	ON	MOCH	05-ARN-00	BCCH	2004	RMB	11:25	BCCH
7825	ON	MOCH	05-ARN-00	BCCH	2004	RMB	11:25	BCCH
7825	ON	MOCH	05-ARN-00	BCCH	2004	RMB	11:25	BCCH
7825	ON	MOCH	05-ARN-00	BCCH	2004	RMB	11:25	MOCH
7825	ON	MOCH	05-ARN-00	BCCH	2004	RMB	11:25	MOCH
7825	ON	MOCH	05-ARN-00	BCCH	2004	RMB	11:25	BCCH
7825	ON	MOCH	05-ARN-00	BCCH	2004	RMB	11:25	none
7280	ON	MOCH	05-MN-005	RBNU	2003	RMB	9:51	MOCH
7280	ON	MOCH	05-MN-005	RBNU	2003	RMB	9:51	MOCH
7280	ON	MOCH	05-MN-005	RBNU	2003	RMB	9:51	MOCH
7280	ON	MOCH	05-MN-005	RBNU	2003	RMB	9:51	MOCH
7280	ON	MOCH	05-MN-005	RBNU	2003	RMB	9:51	MOCH
7020	ON	MOCH	05-ARN-00	MOCH	2003	RMB	11:05	MOCH
7020	ON	MOCH	05-ARN-00	MOCH	2003	RMB	11:05	MOCH
7020	ON	MOCH	05-ARN-00	MOCH	2003	RMB	11:05	MOCH
7020	ON	MOCH	05-ARN-00	MOCH	2003	RMB	11:05	MOCH
7020	ON	MOCH	05-ARN-00	MOCH	2003	RMB	11:05	MOCH
7020	ON	MOCH	05-ARN-00	MOCH	2003	RMB	11:05	BCCH
7020	ON	MOCH	05-ARN-00	MOCH	2003	RMB	11:05	MOCH
7020	ON	MOCH	05-ARN-00	MOCH	2003	RMB	11:05	none
7020	ON	MOCH	05-ARN-00	MOCH	2003	RMB	11:05	none
7020	ON	MOCH	05-ARN-00	MOCH	2003	RMB	11:05	RBNU
7020	ON	MOCH	05-ARN-00	MOCH	2003	RMB	11:05	MOCH
7482	ON	MOCH	05-MN-015	MOCH	2003	RMB	8:29	BCCH
7482	ON	MOCH	05-MN-015	MOCH	2003	RMB	8:29	BCCH
7482	ON	MOCH	05-MN-015	MOCH	2003	RMB	8:29	BCCH
7482	ON	MOCH	05-MN-015	MOCH	2003	RMB	8:29	MOCH
7482	ON	MOCH	05-MN-015	MOCH	2003	RMB	8:29	MOCH
7482	ON	MOCH	05-MN-015	MOCH	2003	RMB	8:29	BCCH
7482	ON	MOCH	05-MN-015	MOCH	2003	RMB	8:29	MOCH
7482	ON	MOCH	05-MN-015	MOCH	2003	RMB	8:29	MOCH
7482	ON	MOCH	05-MN-015	MOCH	2003	RMB	8:29	BCCH
7482	ON	MOCH	05-MN-015	MOCH	2003	RMB	8:29	BCCH
7482	ON	MOCH	05-MN-015	MOCH	2003	RMB	8:29	BCCH
70738	ON	MOCH	05-ARN-01	MOCH	2004	RBM	10:34	MOCH
70738	ON	MOCH	05-ARN-01	MOCH	2004	RBM	10:34	MOCH
70738	ON	MOCH	05-ARN-01	MOCH	2004	RBM	10:34	MOCH
70738	ON	MOCH	05-ARN-01	MOCH	2004	RBM	10:34	MOCH
70738	ON	MOCH	05-ARN-01	MOCH	2004	RBM	10:34	MOCH
70738	ON	MOCH	05-ARN-01	MOCH	2004	RBM	10:34	BCCH
70738	ON	MOCH	05-ARN-01	MOCH	2004	RBM	10:34	BCCH
70738	ON	MOCH	05-ARN-01	MOCH	2004	RBM	10:34	MOCH

70738	ON	MOCH	05-ARN-01	MOCH	2004	RBM	10:34	RBNU
70738	ON	MOCH	05-ARN-01	MOCH	2004	RBM	10:34	RBNU
70738	ON	MOCH	05-ARN-01	MOCH	2004	RBM	10:34	RBNU
70738	ON	MOCH	05-ARN-01	MOCH	2004	RBM	10:34	RBNU
70738	ON	MOCH	05-ARN-01	MOCH	2004	RBM	10:34	BCCH
70738	ON	MOCH	05-ARN-01	MOCH	2004	RBM	10:34	RBNU
70738	ON	MOCH	05-ARN-01	MOCH	2004	RBM	10:34	BCCH
70738	ON	MOCH	05-ARN-01	MOCH	2004	RBM	10:34	BCCH
70738	ON	MOCH	05-ARN-01	MOCH	2004	RBM	10:34	BCCH
70738	ON	MOCH	05-ARN-01	MOCH	2004	RBM	10:34	BCCH
7744	ON	MOCH	05-MN-012	MOCH	2004	BRM	8:04	BCCH
7744	ON	MOCH	05-MN-012	MOCH	2004	BRM	8:04	BCCH
7744	ON	MOCH	05-MN-012	MOCH	2004	BRM	8:04	BCCH
7744	ON	MOCH	05-MN-012	MOCH	2004	BRM	8:04	BCCH
7744	ON	MOCH	05-MN-012	MOCH	2004	BRM	8:04	BCCH
7744	ON	MOCH	05-MN-012	MOCH	2004	BRM	8:04	BCCH
7744	ON	MOCH	05-MN-012	MOCH	2004	BRM	8:04	BCCH
7744	ON	MOCH	05-MN-012	MOCH	2004	BRM	8:04	BCCH
7744	ON	MOCH	05-MN-012	MOCH	2004	BRM	8:04	BCCH
7744	ON	MOCH	05-MN-012	MOCH	2004	BRM	8:13	RBNU
7744	ON	MOCH	05-MN-012	MOCH	2004	BRM	8:13	RBNU
7744	ON	MOCH	05-MN-012	MOCH	2004	BRM	8:13	RBNU
7744	ON	MOCH	05-MN-012	MOCH	2004	BRM	8:13	RBNU
7744	ON	MOCH	05-MN-012	MOCH	2004	BRM	8:13	RBNU
7744	ON	MOCH	05-MN-012	MOCH	2004	BRM	8:22	MOCH
7744	ON	MOCH	05-MN-012	MOCH	2004	BRM	8:22	MOCH
7744	ON	MOCH	05-MN-012	MOCH	2004	BRM	8:22	MOCH
7744	ON	MOCH	05-MN-012	MOCH	2004	BRM	8:22	MOCH
7744	ON	MOCH	05-MN-012	MOCH	2004	BRM	8:22	MOCH
7744	ON	MOCH	05-MN-012	MOCH	2004	BRM	8:22	MOCH
7744	ON	MOCH	05-MN-012	MOCH	2004	BRM	8:22	MOCH
7744	ON	MOCH	05-MN-012	MOCH	2004	BRM	8:22	MOCH
7744	ON	MOCH	05-MN-012	MOCH	2004	BRM	8:22	MOCH
7744	ON	MOCH	05-MN-012	MOCH	2004	BRM	8:22	MOCH
7744	ON	MOCH	05-MN-012	MOCH	2004	BRM	8:22	MOCH
7744	ON	MOCH	05-MN-012	MOCH	2004	BRM	8:22	MOCH
7744	ON	MOCH	05-MN-012	MOCH	2004	BRM	8:22	MOCH
7744	ON	MOCH	05-MN-012	MOCH	2004	BRM	8:22	MOCH
7117	ON	MOCH	05-CW-017	TRES	2004	RMB	10:02	MOCH
7117	ON	MOCH	05-CW-017	TRES	2004	RMB	10:02	BCCH
7117	ON	MOCH	05-CW-017	TRES	2004	RMB	10:02	BCCH
7117	ON	MOCH	05-CW-017	TRES	2004	RMB	10:02	BCCH
7117	ON	MOCH	05-CW-017	TRES	2004	RMB	10:02	RBNU
7117	ON	MOCH	05-CW-017	TRES	2004	RMB	10:02	RBNU

7117	ON	MOCH	05-CW-017	TRES	2004	RMB	10:02	RBNU
7117	ON	MOCH	05-CW-017	TRES	2004	RMB	10:02	MOCH
7117	ON	MOCH	05-CW-017	TRES	2004	RMB	10:02	MOCH
7117	ON	MOCH	05-CW-017	TRES	2004	RMB	10:02	MOCH
7117	ON	MOCH	05-CW-017	TRES	2004	RMB	10:02	BCCH
7117	ON	MOCH	05-CW-017	TRES	2004	RMB	10:02	BCCH
7117	ON	MOCH	05-CW-017	TRES	2004	RMB	10:02	BCCH
7117	ON	MOCH	05-CW-017	TRES	2004	RMB	10:02	BCCH
7117	ON	MOCH	05-CW-017	TRES	2004	RMB	10:02	MOCH
7117	ON	MOCH	05-CW-017	TRES	2004	RMB	10:02	RBNU
7117	ON	MOCH	05-CW-017	TRES	2004	RMB	10:02	none
7748	ON	MOCH	05-PAR-03	MOCH	1999	RBM	11:52	MOCH
7748	ON	MOCH	05-PAR-03	MOCH	1999	RBM	11:52	MOCH
7748	ON	MOCH	05-PAR-03	MOCH	1999	RBM	11:52	RBNU
7748	ON	MOCH	05-PAR-03	MOCH	1999	RBM	11:52	MOCH
7748	ON	MOCH	05-PAR-03	MOCH	1999	RBM	11:52	MOCH
7748	ON	MOCH	05-PAR-03	MOCH	1999	RBM	11:52	RBNU
7972	ON	MOCH	05-PAR-049	RBNU	2004	BMR	5:56	RBNU
7972	ON	MOCH	05-PAR-049	RBNU	2004	BMR	5:56	none
7972	ON	MOCH	05-PAR-049	RBNU	2004	BMR	5:56	BCCH
7972	ON	MOCH	05-PAR-049	RBNU	2004	BMR	5:56	BCCH
7972	ON	MOCH	05-PAR-049	RBNU	2004	BMR	5:56	BCCH
7972	ON	MOCH	05-PAR-049	RBNU	2004	BMR	5:56	BCCH
7972	ON	MOCH	05-PAR-049	RBNU	2004	BMR	5:56	RBNU
7972	ON	MOCH	05-PAR-049	RBNU	2004	BMR	5:56	MOCH
7972	ON	MOCH	05-PAR-049	RBNU	2004	BMR	5:56	MOCH
7972	ON	MOCH	05-PAR-049	RBNU	2004	BMR	5:56	MOCH
7972	ON	MOCH	05-PAR-049	RBNU	2004	BMR	5:56	RBNU
7972	ON	MOCH	05-PAR-049	RBNU	2004	BMR	5:56	RBNU
7972	ON	MOCH	05-PAR-049	RBNU	2004	BMR	5:56	RBNU
7972	ON	MOCH	05-PAR-049	RBNU	2004	BMR	5:56	none
7972	ON	MOCH	05-PAR-049	RBNU	2004	BMR	5:56	RBNU
7972	ON	MOCH	05-PAR-049	RBNU	2004	BMR	5:56	RBNU
70133	ON	MOCH	05-ARN-02	MOCH	2004	BRM	8:23	BCCH
70133	ON	MOCH	05-ARN-02	MOCH	2004	BRM	8:23	BCCH
70133	ON	MOCH	05-ARN-02	MOCH	2004	BRM	8:23	BCCH
70133	ON	MOCH	05-ARN-02	MOCH	2004	BRM	8:23	BCCH
70133	ON	MOCH	05-ARN-02	MOCH	2004	BRM	8:23	RBNU
70133	ON	MOCH	05-ARN-02	MOCH	2004	BRM	8:23	BCCH
70133	ON	MOCH	05-ARN-02	MOCH	2004	BRM	8:23	BCCH
70133	ON	MOCH	05-ARN-02	MOCH	2004	BRM	8:23	RBNU
70133	ON	MOCH	05-ARN-02	MOCH	2004	BRM	8:23	RBNU
70133	ON	MOCH	05-ARN-02	MOCH	2004	BRM	8:23	MOCH
70133	ON	MOCH	05-ARN-02	MOCH	2004	BRM	8:23	MOCH

70133	ON	MOCH	05-ARN-02	MOCH	2004	BRM	8:23	MOCH
70133	ON	MOCH	05-ARN-02	MOCH	2004	BRM	8:23	MOCH
70133	ON	MOCH	05-ARN-02	MOCH	2004	BRM	8:23	MOCH
70133	ON	MOCH	05-ARN-02	MOCH	2004	BRM	8:23	BCCH
70133	ON	MOCH	05-ARN-02	MOCH	2004	BRM	8:23	RBNU
70133	ON	MOCH	05-ARN-02	MOCH	2004	BRM	8:23	BCCH
70133	ON	MOCH	05-ARN-02	MOCH	2004	BRM	8:23	BCCH
70133	ON	MOCH	05-ARN-02	MOCH	2004	BRM	8:23	MOCH
70133	ON	MOCH	05-ARN-02	MOCH	2004	BRM	8:23	MOCH
7408	ON	MOCH	05-PAR-04	RBNU	2004	BRM	8:24	BCCH
7408	ON	MOCH	05-PAR-04	RBNU	2004	BRM	8:24	none
7408	ON	MOCH	05-PAR-04	RBNU	2004	BRM	8:24	none
7408	ON	MOCH	05-PAR-04	RBNU	2004	BRM	8:24	none
7408	ON	MOCH	05-PAR-04	RBNU	2004	BRM	8:24	BCCH
7408	ON	MOCH	05-PAR-04	RBNU	2004	BRM	8:24	MOCH
7408	ON	MOCH	05-PAR-04	RBNU	2004	BRM	9:06	MOCH
7408	ON	MOCH	05-PAR-04	RBNU	2004	BRM	9:06	MOCH
7408	ON	MOCH	05-PAR-04	RBNU	2004	BRM	9:06	MOCH
7408	ON	MOCH	05-PAR-04	RBNU	2004	BRM	9:06	MOCH
7408	ON	MOCH	05-PAR-04	RBNU	2004	BRM	9:06	MOCH
7408	ON	MOCH	05-PAR-04	RBNU	2004	BRM	9:06	MOCH
7408	ON	MOCH	05-PAR-04	RBNU	2004	BRM	9:06	MOCH
7408	ON	MOCH	05-PAR-04	RBNU	2004	BRM	9:06	MOCH
7408	ON	MOCH	05-PAR-04	RBNU	2004	BRM	9:06	MOCH
70102	ON	MOCH	05-ARN-02	BCCH	2004	BRM	8:29	BCCH
70102	ON	MOCH	05-ARN-02	BCCH	2004	BRM	8:29	RBNU
70102	ON	MOCH	05-ARN-02	BCCH	2004	BRM	8:29	MOCH
70102	ON	MOCH	05-ARN-02	BCCH	2004	BRM	8:29	MOCH
70102	ON	MOCH	05-ARN-02	BCCH	2004	BRM	8:29	BCCH
70102	ON	MOCH	05-ARN-02	BCCH	2004	BRM	8:29	RBNU
70102	ON	MOCH	05-ARN-02	BCCH	2004	BRM	8:29	RBNU
70102	ON	MOCH	05-ARN-02	BCCH	2004	BRM	8:29	MOCH
70102	ON	MOCH	05-ARN-02	BCCH	2004	BRM	8:29	BCCH
70102	ON	MOCH	05-ARN-02	BCCH	2004	BRM	8:29	RBNU
70102	ON	MOCH	05-ARN-02	BCCH	2004	BRM	8:29	RBNU
70102	ON	MOCH	05-ARN-02	BCCH	2004	BRM	8:29	RBNU
70102	ON	MOCH	05-ARN-02	BCCH	2004	BRM	8:29	RBNU
70102	ON	MOCH	05-ARN-02	BCCH	2004	BRM	8:29	RBNU
70102	ON	MOCH	05-ARN-02	BCCH	2004	BRM	8:29	RBNU
70102	ON	MOCH	05-ARN-02	BCCH	2004	BRM	8:29	RBNU
70102	ON	MOCH	05-ARN-02	BCCH	2004	BRM	8:29	BCCH
70102	ON	MOCH	05-ARN-02	BCCH	2004	BRM	8:29	MOCH
7595	ON	MOCH	05-MN-025	RBNU	2004	MRB	6:50	MOCH
7595	ON	MOCH	05-MN-025	RBNU	2004	MRB	6:50	MOCH

7595	ON	MOCH	05-MN-025	RBNU	2004	MRB	6:50	MOCH
7595	ON	MOCH	05-MN-025	RBNU	2004	MRB	6:50	MOCH
7595	ON	MOCH	05-MN-025	RBNU	2004	MRB	6:50	MOCH
7595	ON	MOCH	05-MN-025	RBNU	2004	MRB	6:50	BCCH
7595	ON	MOCH	05-MN-025	RBNU	2004	MRB	6:50	BCCH
7595	ON	MOCH	05-MN-025	RBNU	2004	MRB	6:50	RBNU
7595	ON	MOCH	05-MN-025	RBNU	2004	MRB	6:50	RBNU
7595	ON	MOCH	05-MN-025	RBNU	2004	MRB	6:50	RBNU
7595	ON	MOCH	05-MN-025	RBNU	2004	MRB	6:50	MOCH
7595	ON	MOCH	05-MN-025	RBNU	2004	MRB	6:50	RBNU
7595	ON	MOCH	05-MN-025	RBNU	2004	MRB	6:50	BCCH
7595	ON	MOCH	05-MN-025	RBNU	2004	MRB	6:50	none
7595	ON	MOCH	05-MN-025	RBNU	2004	MRB	6:50	MOCH
7595	ON	MOCH	05-MN-025	RBNU	2004	MRB	6:50	RBNU
7595	ON	MOCH	05-MN-025	RBNU	2004	MRB	6:50	RBNU
7595	ON	MOCH	05-MN-025	RBNU	2004	MRB	6:50	RBNU
7595	ON	MOCH	05-MN-025	RBNU	2004	MRB	6:50	RBNU
7595	ON	MOCH	05-MN-025	RBNU	2004	MRB	6:50	RBNU
7839	ON	MOCH	05-PAR-05	MOCH	2004	BRM	7:40	MOCH
7839	ON	MOCH	05-PAR-05	MOCH	2004	BRM	7:40	MOCH
7839	ON	MOCH	05-PAR-05	MOCH	2004	BRM	7:40	MOCH
7839	ON	MOCH	05-PAR-05	MOCH	2004	BRM	7:40	BCCH
7440	ON	MOCH	05-ARN-02	MOCH	2002	MRB	11:02	MOCH
7440	ON	MOCH	05-ARN-02	MOCH	2002	MRB	11:02	BCCH
7440	ON	MOCH	05-ARN-02	MOCH	2002	MRB	11:02	MOCH
7440	ON	MOCH	05-ARN-02	MOCH	2002	MRB	11:02	MOCH
7440	ON	MOCH	05-ARN-02	MOCH	2002	MRB	11:02	MOCH
7440	ON	MOCH	05-ARN-02	MOCH	2002	MRB	11:02	MOCH
7440	ON	MOCH	05-ARN-02	MOCH	2002	MRB	11:02	RBNU
7440	ON	MOCH	05-ARN-02	MOCH	2002	MRB	11:02	BCCH
7440	ON	MOCH	05-ARN-02	MOCH	2002	MRB	11:02	BCCH
7440	ON	MOCH	05-ARN-02	MOCH	2002	MRB	11:02	MOCH
7440	ON	MOCH	05-ARN-02	MOCH	2002	MRB	11:02	MOCH
7440	ON	MOCH	05-ARN-02	MOCH	2002	MRB	11:02	MOCH
7440	ON	MOCH	05-ARN-02	MOCH	2002	MRB	11:02	RBNU
7440	ON	MOCH	05-ARN-02	MOCH	2002	MRB	11:02	RBNU
7440	ON	MOCH	05-ARN-02	MOCH	2002	MRB	11:02	RBNU
7440	ON	MOCH	05-ARN-02	MOCH	2002	MRB	11:02	BCCH
7440	ON	MOCH	05-ARN-02	MOCH	2002	MRB	11:02	MOCH
7440	ON	MOCH	05-ARN-02	MOCH	2002	MRB	11:02	RBNU
7440	ON	MOCH	05-ARN-02	MOCH	2002	MRB	11:02	RBNU
7440	ON	MOCH	05-ARN-02	MOCH	2002	MRB	11:02	MOCH
7440	ON	MOCH	05-ARN-02	MOCH	2002	MRB	11:02	none
70703	ON	MOCH	05-PAR-06	RBNU	2004	RBM	9:07	BCCH

70703	ON	MOCH	05-PAR-06	RBNU	2004	RBM	9:07	MOCH
70703	ON	MOCH	05-PAR-06	RBNU	2004	RBM	9:07	none
70703	ON	MOCH	05-PAR-06	RBNU	2004	RBM	9:07	none
70703	ON	MOCH	05-PAR-06	RBNU	2004	RBM	9:07	MOCH
70703	ON	MOCH	05-PAR-06	RBNU	2004	RBM	9:07	MOCH
70703	ON	MOCH	05-PAR-06	RBNU	2004	RBM	9:07	none
70703	ON	MOCH	05-PAR-06	RBNU	2004	RBM	9:07	MOCH
70703	ON	MOCH	05-PAR-06	RBNU	2004	RBM	9:07	none
70703	ON	MOCH	05-PAR-06	RBNU	2004	RBM	9:07	none
70703	ON	MOCH	05-PAR-06	RBNU	2004	RBM	9:07	MOCH
70703	ON	MOCH	05-PAR-06	RBNU	2004	RBM	9:07	MOCH
70703	ON	MOCH	05-PAR-06	RBNU	2004	RBM	9:07	MOCH
70703	ON	MOCH	05-PAR-06	RBNU	2004	RBM	9:07	none
70703	ON	MOCH	05-PAR-06	RBNU	2004	RBM	9:07	MOCH
70703	ON	MOCH	05-PAR-06	RBNU	2004	RBM	9:07	RBNU
7656	ON	MOCH	05-MN-013	MOCH	2004	BMR	12:24	BCCH
7656	ON	MOCH	05-MN-013	MOCH	2004	BMR	12:24	MOCH
7656	ON	MOCH	05-MN-013	MOCH	2004	BMR	12:24	MOCH
7656	ON	MOCH	05-MN-013	MOCH	2004	BMR	12:24	BCCH
7656	ON	MOCH	05-MN-013	MOCH	2004	BMR	12:24	none
7656	ON	MOCH	05-MN-013	MOCH	2004	BMR	12:24	BCCH
7656	ON	MOCH	05-MN-013	MOCH	2004	BMR	12:24	BCCH
7656	ON	MOCH	05-MN-013	MOCH	2004	BMR	12:24	BCCH
7656	ON	MOCH	05-MN-013	MOCH	2004	BMR	12:24	BCCH
7656	ON	MOCH	05-MN-013	MOCH	2004	BMR	12:24	BCCH
7656	ON	MOCH	05-MN-013	MOCH	2004	BMR	12:24	none
7656	ON	MOCH	05-MN-013	MOCH	2004	BMR	12:24	MOCH
7656	ON	MOCH	05-MN-013	MOCH	2004	BMR	12:24	MOCH
7656	ON	MOCH	05-MN-013	MOCH	2004	BMR	12:24	RBNU
7656	ON	MOCH	05-MN-013	MOCH	2004	BMR	12:24	RBNU
7656	ON	MOCH	05-MN-013	MOCH	2004	BMR	12:24	MOCH
7656	ON	MOCH	05-MN-013	MOCH	2004	BMR	12:24	MOCH
7656	ON	MOCH	05-MN-013	MOCH	2004	BMR	12:24	RBNU
7656	ON	MOCH	05-MN-013	MOCH	2004	BMR	12:24	none
7656	ON	MOCH	05-MN-013	MOCH	2004	BMR	12:24	MOCH
7656	ON	MOCH	05-MN-013	MOCH	2004	BMR	12:24	MOCH
7656	ON	MOCH	05-MN-013	MOCH	2004	BMR	12:24	RBNU
7656	ON	MOCH	05-MN-013	MOCH	2004	BMR	12:24	none
7656	ON	MOCH	05-MN-013	MOCH	2004	BMR	12:24	none
7825	ON	MOCH	05-ARN-00	BCCH	2004	RMB	8:40	RBNU
7825	ON	MOCH	05-ARN-00	BCCH	2004	RMB	8:40	RBNU
7825	ON	MOCH	05-ARN-00	BCCH	2004	RMB	8:40	RBNU
7825	ON	MOCH	05-ARN-00	BCCH	2004	RMB	8:40	RBNU
7825	ON	MOCH	05-ARN-00	BCCH	2004	RMB	8:40	BCCH

7825	ON	MOCH	05-ARN-00	BCCH	2004	RMB	8:40	RBNU
7825	ON	MOCH	05-ARN-00	BCCH	2004	RMB	8:40	RBNU
7825	ON	MOCH	05-ARN-00	BCCH	2004	RMB	8:40	MOCH
7825	ON	MOCH	05-ARN-00	BCCH	2004	RMB	8:40	MOCH
7825	ON	MOCH	05-ARN-00	BCCH	2004	RMB	8:40	MOCH
7825	ON	MOCH	05-ARN-00	BCCH	2004	RMB	8:40	MOCH
7280	ON	MOCH	05-MN-005	RBNU	2003	RBM	13:58	MOCH
7280	ON	MOCH	05-MN-005	RBNU	2003	RBM	13:58	MOCH
7280	ON	MOCH	05-MN-005	RBNU	2003	RBM	13:58	BCCH
7280	ON	MOCH	05-MN-005	RBNU	2003	RBM	13:58	BCCH
7280	ON	MOCH	05-MN-005	RBNU	2003	RBM	13:58	none
7280	ON	MOCH	05-MN-005	RBNU	2003	RBM	13:58	MOCH
7280	ON	MOCH	05-MN-005	RBNU	2003	RBM	13:58	none
7280	ON	MOCH	05-MN-005	RBNU	2003	RBM	13:58	RBNU
7280	ON	MOCH	05-MN-005	RBNU	2003	RBM	13:58	BCCH
7280	ON	MOCH	05-MN-005	RBNU	2003	RBM	13:58	BCCH
7280	ON	MOCH	05-MN-005	RBNU	2003	RBM	13:58	MOCH
7280	ON	MOCH	05-MN-005	RBNU	2003	RBM	13:58	none
7280	ON	MOCH	05-MN-005	RBNU	2003	RBM	13:58	BCCH
7280	ON	MOCH	05-MN-005	RBNU	2003	RBM	13:58	BCCH
7280	ON	MOCH	05-MN-005	RBNU	2003	RBM	13:58	BCCH
7280	ON	MOCH	05-MN-005	RBNU	2003	RBM	13:58	RBNU
7280	ON	MOCH	05-MN-005	RBNU	2003	RBM	13:58	RBNU
7280	ON	MOCH	05-MN-005	RBNU	2003	RBM	13:58	none
7280	ON	MOCH	05-MN-005	RBNU	2003	RBM	13:58	RBNU
7280	ON	MOCH	05-MN-005	RBNU	2003	RBM	13:58	none
7117	ON	MOCH	05-CW-017	TRES	2004	BRM	9:04	none
7117	ON	MOCH	05-CW-017	TRES	2004	BRM	9:04	BCCH
7117	ON	MOCH	05-CW-017	TRES	2004	BRM	9:04	RBNU
7117	ON	MOCH	05-CW-017	TRES	2004	BRM	9:04	RBNU
7117	ON	MOCH	05-CW-017	TRES	2004	BRM	9:04	RBNU
7117	ON	MOCH	05-CW-017	TRES	2004	BRM	9:04	RBNU
7117	ON	MOCH	05-CW-017	TRES	2004	BRM	9:04	MOCH
7117	ON	MOCH	05-CW-017	TRES	2004	BRM	9:04	MOCH
7117	ON	MOCH	05-CW-017	TRES	2004	BRM	9:04	RBNU
7117	ON	MOCH	05-CW-017	TRES	2004	BRM	9:04	RBNU
7117	ON	MOCH	05-CW-017	TRES	2004	BRM	9:04	BCCH
7117	ON	MOCH	05-CW-017	TRES	2004	BRM	9:04	BCCH
7117	ON	MOCH	05-CW-017	TRES	2004	BRM	9:04	BCCH
7117	ON	MOCH	05-CW-017	TRES	2004	BRM	9:04	MOCH
7117	ON	MOCH	05-CW-017	TRES	2004	BRM	9:04	BCCH
7117	ON	MOCH	05-CW-017	TRES	2004	BRM	9:04	RBNU
7117	ON	MOCH	05-CW-017	TRES	2004	BRM	9:04	RBNU
7117	ON	MOCH	05-CW-017	TRES	2004	BRM	9:04	MOCH

7117	ON	MOCH	05-CW-017	TRES	2004	BRM	9:04	MOCH
7117	ON	MOCH	05-CW-017	TRES	2004	BRM	9:04	none
7117	ON	MOCH	05-CW-017	TRES	2004	BRM	9:04	BCCH
7117	ON	MOCH	05-CW-017	TRES	2004	BRM	9:04	MOCH
7117	ON	MOCH	05-CW-017	TRES	2004	BRM	9:04	RBNU
7117	ON	MOCH	05-CW-017	TRES	2004	BRM	9:04	MOCH
7020	ON	MOCH	05-ARN-00	MOCH	2003	RMB	9:39	MOCH
7020	ON	MOCH	05-ARN-00	MOCH	2003	RMB	9:39	BCCH
7020	ON	MOCH	05-ARN-00	MOCH	2003	RMB	9:39	RBNU
7020	ON	MOCH	05-ARN-00	MOCH	2003	RMB	9:39	BCCH
7020	ON	MOCH	05-ARN-00	MOCH	2003	RMB	9:39	MOCH
7020	ON	MOCH	05-ARN-00	MOCH	2003	RMB	9:39	RBNU
7020	ON	MOCH	05-ARN-00	MOCH	2003	RMB	9:39	RBNU
7020	ON	MOCH	05-ARN-00	MOCH	2003	RMB	9:39	RBNU
7020	ON	MOCH	05-ARN-00	MOCH	2003	RMB	9:39	MOCH
7020	ON	MOCH	05-ARN-00	MOCH	2003	RMB	9:39	none
7020	ON	MOCH	05-ARN-00	MOCH	2003	RMB	9:39	none
7020	ON	MOCH	05-ARN-00	MOCH	2003	RMB	9:39	RBNU
7020	ON	MOCH	05-ARN-00	MOCH	2003	RMB	9:39	MOCH
7839	ON	MOCH	05-PAR-05	MOCH	2004	BRM	9:29	MOCH
7839	ON	MOCH	05-PAR-05	MOCH	2004	BRM	9:29	MOCH
7839	ON	MOCH	05-PAR-05	MOCH	2004	BRM	9:29	MOCH
7839	ON	MOCH	05-PAR-05	MOCH	2004	BRM	9:29	MOCH
7839	ON	MOCH	05-PAR-05	MOCH	2004	BRM	9:29	MOCH
7839	ON	MOCH	05-PAR-05	MOCH	2004	BRM	9:29	none
7839	ON	MOCH	05-PAR-05	MOCH	2004	BRM	9:29	none
7839	ON	MOCH	05-PAR-05	MOCH	2004	BRM	9:29	none
7839	ON	MOCH	05-PAR-05	MOCH	2004	BRM	9:29	BCCH
7839	ON	MOCH	05-PAR-05	MOCH	2004	BRM	9:29	RBNU
7839	ON	MOCH	05-PAR-05	MOCH	2004	BRM	9:29	RBNU
7839	ON	MOCH	05-PAR-05	MOCH	2004	BRM	9:29	BCCH
7839	ON	MOCH	05-PAR-05	MOCH	2004	BRM	9:29	RBNU
7595	ON	MOCH	05-MN-025	RBNU	2004	MRB	9:55	BCCH
7595	ON	MOCH	05-MN-025	RBNU	2004	MRB	9:55	BCCH
7595	ON	MOCH	05-MN-025	RBNU	2004	MRB	9:55	BCCH
7595	ON	MOCH	05-MN-025	RBNU	2004	MRB	9:55	BCCH
7595	ON	MOCH	05-MN-025	RBNU	2004	MRB	9:55	MOCH
7595	ON	MOCH	05-MN-025	RBNU	2004	MRB	9:55	none
7595	ON	MOCH	05-MN-025	RBNU	2004	MRB	9:55	none
7595	ON	MOCH	05-MN-025	RBNU	2004	MRB	9:55	none
7595	ON	MOCH	05-MN-025	RBNU	2004	MRB	9:55	none
7595	ON	MOCH	05-MN-025	RBNU	2004	MRB	9:55	none
7595	ON	MOCH	05-MN-025	RBNU	2004	MRB	9:55	RBNU
7595	ON	MOCH	05-MN-025	RBNU	2004	MRB	9:55	RBNU

7595	ON	MOCH	05-MN-025	RBNU	2004	MRB	9:55	RBNU
7595	ON	MOCH	05-MN-025	RBNU	2004	MRB	9:55	RBNU
7595	ON	MOCH	05-MN-025	RBNU	2004	MRB	9:55	BCCH
7595	ON	MOCH	05-MN-025	RBNU	2004	MRB	9:55	BCCH
7595	ON	MOCH	05-MN-025	RBNU	2004	MRB	9:55	BCCH
7595	ON	MOCH	05-MN-025	RBNU	2004	MRB	9:55	BCCH
7595	ON	MOCH	05-MN-025	RBNU	2004	MRB	9:55	BCCH
7595	ON	MOCH	05-MN-025	RBNU	2004	MRB	9:55	RBNU
7595	ON	MOCH	05-MN-025	RBNU	2004	MRB	9:55	BCCH
70502	ON	MOCH	05-ARN-01	RBNU	2004	RMB	15:36	MOCH
70502	ON	MOCH	05-ARN-01	RBNU	2004	RMB	15:36	MOCH
70502	ON	MOCH	05-ARN-01	RBNU	2004	RMB	15:36	MOCH
70502	ON	MOCH	05-ARN-01	RBNU	2004	RMB	15:36	MOCH
70502	ON	MOCH	05-ARN-01	RBNU	2004	RMB	15:36	MOCH
70502	ON	MOCH	05-ARN-01	RBNU	2004	RMB	15:36	BCCH
70502	ON	MOCH	05-ARN-01	RBNU	2004	RMB	15:36	none
70502	ON	MOCH	05-ARN-01	RBNU	2004	RMB	15:36	none
70502	ON	MOCH	05-ARN-01	RBNU	2004	RMB	15:36	RBNU
70502	ON	MOCH	05-ARN-01	RBNU	2004	RMB	15:36	RBNU
70502	ON	MOCH	05-ARN-01	RBNU	2004	RMB	15:36	RBNU
70502	ON	MOCH	05-ARN-01	RBNU	2004	RMB	15:36	RBNU
70502	ON	MOCH	05-ARN-01	RBNU	2004	RMB	15:36	none
70502	ON	MOCH	05-ARN-01	RBNU	2004	RMB	15:36	none
70502	ON	MOCH	05-ARN-01	RBNU	2004	RMB	15:36	none
70502	ON	MOCH	05-ARN-01	RBNU	2004	RMB	15:36	BCCH
70502	ON	MOCH	05-ARN-01	RBNU	2004	RMB	15:36	BCCH
70502	ON	MOCH	05-ARN-01	RBNU	2004	RMB	15:36	MOCH
70502	ON	MOCH	05-ARN-01	RBNU	2004	RMB	15:36	none
70502	ON	MOCH	05-ARN-01	RBNU	2004	RMB	15:36	BCCH
70502	ON	MOCH	05-ARN-01	RBNU	2004	RMB	15:36	BCCH
70502	ON	MOCH	05-ARN-01	RBNU	2004	RMB	15:36	none
70502	ON	MOCH	05-ARN-01	RBNU	2004	RMB	15:36	none
70502	ON	MOCH	05-ARN-01	RBNU	2004	RMB	15:36	BCCH
70502	ON	MOCH	05-ARN-01	RBNU	2004	RMB	15:36	none
70502	ON	MOCH	05-ARN-01	RBNU	2004	RMB	15:36	none
70502	ON	MOCH	05-ARN-01	RBNU	2004	RMB	15:36	RBNU
7974	ON	MOCH	05-PAR-09	RBNU	2004	RBM	10:33	BCCH
7974	ON	MOCH	05-PAR-09	RBNU	2004	RBM	10:33	BCCH
7974	ON	MOCH	05-PAR-09	RBNU	2004	RBM	10:33	MOCH
7974	ON	MOCH	05-PAR-09	RBNU	2004	RBM	10:33	BCCH
7974	ON	MOCH	05-PAR-09	RBNU	2004	RBM	10:33	BCCH
7974	ON	MOCH	05-PAR-09	RBNU	2004	RBM	10:33	MOCH
7974	ON	MOCH	05-PAR-09	RBNU	2004	RBM	10:33	RBNU
7974	ON	MOCH	05-PAR-09	RBNU	2004	RBM	10:33	RBNU

7974	ON	MOCH	05-PAR-09	RBNU	2004	RBM	10:33	RBNU
7974	ON	MOCH	05-PAR-09	RBNU	2004	RBM	10:33	MOCH
7974	ON	MOCH	05-PAR-09	RBNU	2004	RBM	10:33	RBNU
7974	ON	MOCH	05-PAR-09	RBNU	2004	RBM	10:33	BCCH
70102	ON	MOCH	06-LD-001	MOCH	2005	RBM	10:40	MOCH
70102	ON	MOCH	06-LD-001	MOCH	2005	RBM	10:40	RBNU
70102	ON	MOCH	06-LD-001	MOCH	2005	RBM	10:40	RBNU
70102	ON	MOCH	06-LD-001	MOCH	2005	RBM	10:40	BCCH
70102	ON	MOCH	06-LD-001	MOCH	2005	RBM	10:40	BCCH
70102	ON	MOCH	06-LD-001	MOCH	2005	RBM	10:40	MOCH
70102	ON	MOCH	06-LD-001	MOCH	2005	RBM	10:40	MOCH
70102	ON	MOCH	06-LD-001	MOCH	2005	RBM	10:40	MOCH
70102	ON	MOCH	06-LD-001	MOCH	2005	RBM	10:40	BCCH
70102	ON	MOCH	06-LD-001	MOCH	2005	RBM	10:40	MOCH
70102	ON	MOCH	06-LD-001	MOCH	2005	RBM	10:40	BCCH
7280	ON	MOCH	06-LD-013	MOCH	2005	BMR	9:57	BCCH
7280	ON	MOCH	06-LD-013	MOCH	2005	BMR	9:57	MOCH
7280	ON	MOCH	06-LD-013	MOCH	2005	BMR	9:57	MOCH
7280	ON	MOCH	06-LD-013	MOCH	2005	BMR	9:57	MOCH
7280	ON	MOCH	06-LD-013	MOCH	2005	BMR	9:57	RBNU
7280	ON	MOCH	06-LD-013	MOCH	2005	BMR	9:57	BCCH
70413	ON	MOCH	06-ARN-01	RBNU	2005	MBR	11:05	MOCH
70413	ON	MOCH	06-ARN-01	RBNU	2005	MBR	11:05	BCCH
70413	ON	MOCH	06-ARN-01	RBNU	2005	MBR	11:05	RBNU
70413	ON	MOCH	06-ARN-01	RBNU	2005	MBR	11:05	BCCH
70413	ON	MOCH	06-ARN-01	RBNU	2005	MBR	11:05	MOCH
70413	ON	MOCH	06-ARN-01	RBNU	2005	MBR	11:05	MOCH
70413	ON	MOCH	06-ARN-01	RBNU	2005	MBR	11:05	MOCH
70413	ON	MOCH	06-ARN-01	RBNU	2005	MBR	11:05	BCCH
70413	ON	MOCH	06-ARN-01	RBNU	2005	MBR	11:05	BCCH
70413	ON	MOCH	06-ARN-01	RBNU	2005	MBR	11:05	RBNU
70413	ON	MOCH	06-ARN-01	RBNU	2005	MBR	11:05	RBNU
70413	ON	MOCH	06-ARN-01	RBNU	2005	MBR	11:05	BCCH
70413	ON	MOCH	06-ARN-01	RBNU	2005	MBR	11:05	RBNU
70413	ON	MOCH	06-ARN-01	RBNU	2005	MBR	11:05	RBNU
7860	ON	MOCH	06-LD-015	RNSA	2003	RMB	11:20	MOCH
7860	ON	MOCH	06-LD-015	RNSA	2003	RMB	11:20	MOCH
7860	ON	MOCH	06-LD-015	RNSA	2003	RMB	11:20	MOCH
7860	ON	MOCH	06-LD-015	RNSA	2003	RMB	11:20	MOCH
7860	ON	MOCH	06-LD-015	RNSA	2003	RMB	11:20	MOCH
7860	ON	MOCH	06-LD-015	RNSA	2003	RMB	11:20	MOCH
7860	ON	MOCH	06-LD-015	RNSA	2003	RMB	11:20	MOCH
7860	ON	MOCH	06-LD-015	RNSA	2003	RMB	11:20	BCCH
7860	ON	MOCH	06-LD-015	RNSA	2003	RMB	11:20	none

7860	ON	MOCH	06-LD-015	RNSA	2003	RMB	11:20	RBNU
7860	ON	MOCH	06-LD-015	RNSA	2003	RMB	11:20	RBNU
7860	ON	MOCH	06-LD-015	RNSA	2003	RMB	11:20	RBNU
7860	ON	MOCH	06-LD-015	RNSA	2003	RMB	11:20	MOCH
70102	ON	MOCH	06-LD-001	MOCH	2005	RBM	9:01	none
70102	ON	MOCH	06-LD-001	MOCH	2005	RBM	9:01	RBNU
70102	ON	MOCH	06-LD-001	MOCH	2005	RBM	9:01	BCCH
70102	ON	MOCH	06-LD-001	MOCH	2005	RBM	9:01	BCCH
70102	ON	MOCH	06-LD-001	MOCH	2005	RBM	9:01	MOCH
70102	ON	MOCH	06-LD-001	MOCH	2005	RBM	9:01	MOCH
70102	ON	MOCH	06-LD-001	MOCH	2005	RBM	9:01	RBNU
70102	ON	MOCH	06-LD-001	MOCH	2005	RBM	9:01	MOCH
7280	ON	MOCH	06-LD-013	MOCH	2005	MRB	11:05	BCCH
7280	ON	MOCH	06-LD-013	MOCH	2005	MRB	11:05	BCCH
7280	ON	MOCH	06-LD-013	MOCH	2005	MRB	11:05	RBNU
7280	ON	MOCH	06-LD-013	MOCH	2005	MRB	11:05	BCCH
7280	ON	MOCH	06-LD-013	MOCH	2005	MRB	11:05	MOCH
7280	ON	MOCH	06-LD-013	MOCH	2005	MRB	11:05	RBNU
7280	ON	MOCH	06-LD-013	MOCH	2005	MRB	11:05	RBNU
7280	ON	MOCH	06-LD-013	MOCH	2005	MRB	11:05	MOCH
7280	ON	MOCH	06-LD-013	MOCH	2005	MRB	11:05	BCCH
7280	ON	MOCH	06-LD-013	MOCH	2005	MRB	11:05	MOCH
7280	ON	MOCH	06-LD-013	MOCH	2005	MRB	11:05	RBNU
7280	ON	MOCH	06-LD-013	MOCH	2005	MRB	11:05	BCCH
7744	ON	MOCH	06-ARN-01	MOCH	2005	RMB	9:19	MOCH
7744	ON	MOCH	06-ARN-01	MOCH	2005	RMB	9:19	RBNU
7744	ON	MOCH	06-ARN-01	MOCH	2005	RMB	9:19	RBNU
7744	ON	MOCH	06-ARN-01	MOCH	2005	RMB	9:19	BCCH
7744	ON	MOCH	06-ARN-01	MOCH	2005	RMB	9:19	BCCH
7744	ON	MOCH	06-ARN-01	MOCH	2005	RMB	9:19	BCCH
7744	ON	MOCH	06-ARN-01	MOCH	2005	RMB	9:19	MOCH
7744	ON	MOCH	06-ARN-01	MOCH	2005	RMB	9:19	MOCH
7744	ON	MOCH	06-ARN-01	MOCH	2005	RMB	9:19	BCCH
7744	ON	MOCH	06-ARN-01	MOCH	2005	RMB	9:19	RBNU
7744	ON	MOCH	06-ARN-01	MOCH	2005	RMB	9:19	MOCH
7744	ON	MOCH	06-ARN-01	MOCH	2005	RMB	9:19	BCCH
7852	ON	MOCH	06-SLD-01	MOCH	2005	MBR	8:07	MOCH
7852	ON	MOCH	06-SLD-01	MOCH	2005	MBR	8:07	MOCH
7852	ON	MOCH	06-SLD-01	MOCH	2005	MBR	8:07	BCCH
7852	ON	MOCH	06-SLD-01	MOCH	2005	MBR	8:07	RBNU
7852	ON	MOCH	06-SLD-01	MOCH	2005	MBR	8:07	MOCH
7852	ON	MOCH	06-SLD-01	MOCH	2005	MBR	8:07	BCCH
7852	ON	MOCH	06-SLD-01	MOCH	2005	MBR	8:07	RBNU
7440	ON	MOCH	06-ARN-01	MOCH	2005	MBR	8:19	MOCH

7440	ON	MOCH	06-ARN-01	MOCH	2005	MBR	8:19	MOCH
7440	ON	MOCH	06-ARN-01	MOCH	2005	MBR	8:19	BCCH
7440	ON	MOCH	06-ARN-01	MOCH	2005	MBR	8:19	MOCH
7440	ON	MOCH	06-ARN-01	MOCH	2005	MBR	8:19	MOCH
7440	ON	MOCH	06-ARN-01	MOCH	2005	MBR	8:19	RBNU
7440	ON	MOCH	06-ARN-01	MOCH	2005	MBR	8:19	MOCH
7440	ON	MOCH	06-ARN-01	MOCH	2005	MBR	8:19	BCCH
7440	ON	MOCH	06-ARN-01	MOCH	2005	MBR	8:19	RBNU
70128	ON	MOCH	06-ARN-01	MOCH	2005	MBR	9:50:00	MOCH
70128	ON	MOCH	06-ARN-01	MOCH	2005	MBR	9:50:00	MOCH
70128	ON	MOCH	06-ARN-01	MOCH	2005	MBR	9:50:00	BCCH
70128	ON	MOCH	06-ARN-01	MOCH	2005	MBR	9:50:00	RBNU
7840	ON	MOCH	06-CK-049	MOCH	2003	RBM	12:19	BCCH
7840	ON	MOCH	06-CK-049	MOCH	2003	RBM	12:19	MOCH
7840	ON	MOCH	06-CK-049	MOCH	2003	RBM	12:19	BCCH
7840	ON	MOCH	06-CK-049	MOCH	2003	RBM	12:19	BCCH
7840	ON	MOCH	06-CK-049	MOCH	2003	RBM	12:19	MOCH
7840	ON	MOCH	06-CK-049	MOCH	2003	RBM	12:19	RBNU
7840	ON	MOCH	06-CK-049	MOCH	2003	RBM	12:19	BCCH
7840	ON	MOCH	06-CK-049	MOCH	2003	RBM	12:19	MOCH
70153	ON	MOCH	06-KS-027	DOWO	2005	BMR	8:16	MOCH
70153	ON	MOCH	06-KS-027	DOWO	2005	BMR	8:16	BCCH
70153	ON	MOCH	06-KS-027	DOWO	2005	BMR	8:16	BCCH
70153	ON	MOCH	06-KS-027	DOWO	2005	BMR	8:16	RBNU
70153	ON	MOCH	06-KS-027	DOWO	2005	BMR	8:16	BCCH
70153	ON	MOCH	06-KS-027	DOWO	2005	BMR	8:16	BCCH
70153	ON	MOCH	06-KS-027	DOWO	2005	BMR	8:16	MOCH
70153	ON	MOCH	06-KS-027	DOWO	2005	BMR	8:16	RBNU
7862	ON	MOCH	06-KS-030	MOCH	2005	MBR	10:46	BCCH
7862	ON	MOCH	06-KS-030	MOCH	2005	MBR	10:46	BCCH
7862	ON	MOCH	06-KS-030	MOCH	2005	MBR	10:46	MOCH
7862	ON	MOCH	06-KS-030	MOCH	2005	MBR	10:46	MOCH
7862	ON	MOCH	06-KS-030	MOCH	2005	MBR	10:46	MOCH
7862	ON	MOCH	06-KS-030	MOCH	2005	MBR	10:46	MOCH
7862	ON	MOCH	06-KS-030	MOCH	2005	MBR	10:46	MOCH
7862	ON	MOCH	06-KS-030	MOCH	2005	MBR	10:46	MOCH
7862	ON	MOCH	06-KS-030	MOCH	2005	MBR	10:46	BCCH
7862	ON	MOCH	06-KS-030	MOCH	2005	MBR	10:46	MOCH
7862	ON	MOCH	06-KS-030	MOCH	2005	MBR	10:46	RBNU
7862	ON	MOCH	06-KS-030	MOCH	2005	MBR	10:46	
7288	ON	MOCH	06-CK-042	RBNU	2004	RBM	8:46	MOCH
7288	ON	MOCH	06-CK-042	RBNU	2004	RBM	8:46	BCCH
7288	ON	MOCH	06-CK-042	RBNU	2004	RBM	8:46	MOCH
7288	ON	MOCH	06-CK-042	RBNU	2004	RBM	8:46	MOCH

7288	ON	MOCH	06-CK-042	RBNU	2004	RBM	8:46	RBNU
7288	ON	MOCH	06-CK-042	RBNU	2004	RBM	8:46	RBNU
7288	ON	MOCH	06-CK-042	RBNU	2004	RBM	8:46	RBNU
7288	ON	MOCH	06-CK-042	RBNU	2004	RBM	8:46	
7288	ON	MOCH	06-CK-042	RBNU	2004	RBM	8:46	BCCH
7288	ON	MOCH	06-CK-042	RBNU	2004	RBM	8:46	MOCH
7288	ON	MOCH	06-CK-042	RBNU	2004	RBM	8:46	RBNU
7288	ON	MOCH	06-CK-042	RBNU	2004	RBM	8:46	
7744	ON	MOCH	06-ARN-01	MOCH	2005	MRB	9:34	BCCH
7744	ON	MOCH	06-ARN-01	MOCH	2005	MRB	9:34	BCCH
7744	ON	MOCH	06-ARN-01	MOCH	2005	MRB	9:34	MOCH
7744	ON	MOCH	06-ARN-01	MOCH	2005	MRB	9:34	MOCH
7744	ON	MOCH	06-ARN-01	MOCH	2005	MRB	9:34	RBNU
7744	ON	MOCH	06-ARN-01	MOCH	2005	MRB	9:34	RBNU
7744	ON	MOCH	06-ARN-01	MOCH	2005	MRB	9:34	RBNU
7744	ON	MOCH	06-ARN-01	MOCH	2005	MRB	9:34	RBNU
7744	ON	MOCH	06-ARN-01	MOCH	2005	MRB	9:34	MOCH
7744	ON	MOCH	06-ARN-01	MOCH	2005	MRB	9:34	BCCH
7744	ON	MOCH	06-ARN-01	MOCH	2005	MRB	9:34	RBNU
7744	ON	MOCH	06-ARN-01	MOCH	2005	MRB	9:34	BCCH
7744	ON	MOCH	06-ARN-01	MOCH	2005	MRB	9:34	RBNU
7744	ON	MOCH	06-ARN-01	MOCH	2005	MRB	9:34	BCCH
7744	ON	MOCH	06-ARN-01	MOCH	2005	MRB	9:34	BCCH
7744	ON	MOCH	06-ARN-01	MOCH	2005	MRB	9:34	BCCH
7744	ON	MOCH	06-ARN-01	MOCH	2005	MRB	9:34	BCCH
7744	ON	MOCH	06-ARN-01	MOCH	2005	MRB	9:34	MOCH
7744	ON	MOCH	06-ARN-01	MOCH	2005	MRB	9:34	MOCH
7744	ON	MOCH	06-ARN-01	MOCH	2005	MRB	9:34	RBNU
7744	ON	MOCH	06-ARN-01	MOCH	2005	MRB	9:34	BCCH
7744	ON	MOCH	06-ARN-01	MOCH	2005	MRB	9:34	MOCH
7744	ON	MOCH	06-ARN-01	MOCH	2005	MRB	9:34	RBNU
7744	ON	MOCH	06-ARN-01	MOCH	2005	MRB	9:34	
70102	ON	MOCH	06-LD-001	MOCH	2005	MBR	8:37	BCCH
70102	ON	MOCH	06-LD-001	MOCH	2005	MBR	8:37	BCCH
70102	ON	MOCH	06-LD-001	MOCH	2005	MBR	8:37	MOCH
70102	ON	MOCH	06-LD-001	MOCH	2005	MBR	8:37	MOCH
70102	ON	MOCH	06-LD-001	MOCH	2005	MBR	8:37	RBNU
70102	ON	MOCH	06-LD-001	MOCH	2005	MBR	8:37	RBNU
70102	ON	MOCH	06-LD-001	MOCH	2005	MBR	8:37	RBNU
70102	ON	MOCH	06-LD-001	MOCH	2005	MBR	8:37	BCCH
70102	ON	MOCH	06-LD-001	MOCH	2005	MBR	8:37	MOCH
70102	ON	MOCH	06-LD-001	MOCH	2005	MBR	8:37	RBNU
70102	ON	MOCH	06-LD-001	MOCH	2005	MBR	8:37	
70102	ON	MOCH	06-LD-001	MOCH	2005	MBR	8:37	RBNU

70413	ON	MOCH	06-ARN-01	RBNU	2005	MBR	10:17	RBNU
70413	ON	MOCH	06-ARN-01	RBNU	2005	MBR	10:17	RBNU
70413	ON	MOCH	06-ARN-01	RBNU	2005	MBR	10:17	BCCH
70413	ON	MOCH	06-ARN-01	RBNU	2005	MBR	10:17	RBNU
70413	ON	MOCH	06-ARN-01	RBNU	2005	MBR	10:17	BCCH
70413	ON	MOCH	06-ARN-01	RBNU	2005	MBR	10:17	BCCH
70413	ON	MOCH	06-ARN-01	RBNU	2005	MBR	10:17	MOCH
70413	ON	MOCH	06-ARN-01	RBNU	2005	MBR	10:17	RBNU
70413	ON	MOCH	06-ARN-01	RBNU	2005	MBR	10:17	
70413	ON	MOCH	06-ARN-01	RBNU	2005	MBR	10:17	BCCH
70413	ON	MOCH	06-ARN-01	RBNU	2005	MBR	10:17	RBNU
70413	ON	MOCH	06-ARN-01	RBNU	2005	MBR	10:17	BCCH
70413	ON	MOCH	06-ARN-01	RBNU	2005	MBR	10:17	MOCH
70413	ON	MOCH	06-ARN-01	RBNU	2005	MBR	10:17	BCCH
70413	ON	MOCH	06-ARN-01	RBNU	2005	MBR	10:17	MOCH
70413	ON	MOCH	06-ARN-01	RBNU	2005	MBR	10:17	RBNU
70413	ON	MOCH	06-ARN-01	RBNU	2005	MBR	10:17	
7440	ON	MOCH	06-ARN-01	MOCH	2005	MBR	9:25	MOCH
7440	ON	MOCH	06-ARN-01	MOCH	2005	MBR	9:25	MOCH
7440	ON	MOCH	06-ARN-01	MOCH	2005	MBR	9:25	BCCH
7440	ON	MOCH	06-ARN-01	MOCH	2005	MBR	9:25	BCCH
7440	ON	MOCH	06-ARN-01	MOCH	2005	MBR	9:25	MOCH
7440	ON	MOCH	06-ARN-01	MOCH	2005	MBR	9:25	MOCH
7440	ON	MOCH	06-ARN-01	MOCH	2005	MBR	9:25	MOCH
7440	ON	MOCH	06-ARN-01	MOCH	2005	MBR	9:25	RBNU
7440	ON	MOCH	06-ARN-01	MOCH	2005	MBR	9:25	RBNU
7440	ON	MOCH	06-ARN-01	MOCH	2005	MBR	9:25	RBNU
7440	ON	MOCH	06-ARN-01	MOCH	2005	MBR	9:25	RBNU
7440	ON	MOCH	06-ARN-01	MOCH	2005	MBR	9:25	MOCH
7440	ON	MOCH	06-ARN-01	MOCH	2005	MBR	9:25	MOCH
7440	ON	MOCH	06-ARN-01	MOCH	2005	MBR	9:25	BCCH
7440	ON	MOCH	06-ARN-01	MOCH	2005	MBR	9:25	BCCH
7440	ON	MOCH	06-ARN-01	MOCH	2005	MBR	9:25	MOCH
7440	ON	MOCH	06-ARN-01	MOCH	2005	MBR	9:25	RBNU
7440	ON	MOCH	06-ARN-01	MOCH	2005	MBR	9:25	RBNU
7440	ON	MOCH	06-ARN-01	MOCH	2005	MBR	9:25	
7840	ON	MOCH	06-CK-049	MOCH	2003	RBM	9:50	MOCH
7840	ON	MOCH	06-CK-049	MOCH	2003	RBM	9:50	RBNU
7840	ON	MOCH	06-CK-049	MOCH	2003	RBM	9:50	RBNU
7840	ON	MOCH	06-CK-049	MOCH	2003	RBM	9:50	RBNU
7840	ON	MOCH	06-CK-049	MOCH	2003	RBM	9:50	BCCH
7840	ON	MOCH	06-CK-049	MOCH	2003	RBM	9:50	MOCH
7840	ON	MOCH	06-CK-049	MOCH	2003	RBM	9:50	RBNU
7840	ON	MOCH	06-CK-049	MOCH	2003	RBM	9:50	

	7840	ON	MOCH	06-CK-049	MOCH	2003	RBM	9:50	BCCH
	7840	ON	MOCH	06-CK-049	MOCH	2003	RBM	9:50	MOCH
	7840	ON	MOCH	06-CK-049	MOCH	2003	RBM	9:50	RBNU
	7840	ON	MOCH	06-CK-049	MOCH	2003	RBM	9:50	
IB	7406	ON	MOCH	07-LP-015	GLSA	2006	BMR	11:23	none
IB	7406	ON	MOCH	07-LP-015	GLSA	2006	BMR	11:23	BCCH
IB	7406	ON	MOCH	07-LP-015	GLSA	2006	BMR	11:23	RBNU
IB	7406	ON	MOCH	07-LP-015	GLSA	2006	BMR	11:23	MOCH
IB	7406	ON	MOCH	07-LP-015	GLSA	2006	BMR	11:23	MOCH
IB	7406	ON	MOCH	07-LP-015	GLSA	2006	BMR	11:23	MOCH
IB	7406	ON	MOCH	07-LP-015	GLSA	2006	BMR	11:23	BCCH
IB	7406	ON	MOCH	07-LP-015	GLSA	2006	BMR	11:23	MOCH
IB	7406	ON	MOCH	07-LP-015	GLSA	2006	BMR	11:23	RBNU
IB	7860	ON	MOCH	07-TD-006	MOCH	2006	RMB	11:33	BCCH
IB	7860	ON	MOCH	07-TD-006	MOCH	2006	RMB	11:33	BCCH
IB	7860	ON	MOCH	07-TD-006	MOCH	2006	RMB	11:33	BCCH
IB	7860	ON	MOCH	07-TD-006	MOCH	2006	RMB	11:33	BCCH
IB	7860	ON	MOCH	07-TD-006	MOCH	2006	RMB	11:33	BCCH
IB	7860	ON	MOCH	07-TD-006	MOCH	2006	RMB	11:33	BCCH
IB	7860	ON	MOCH	07-TD-006	MOCH	2006	RMB	11:33	BCCH
IB	7860	ON	MOCH	07-TD-006	MOCH	2006	RMB	11:33	BCCH
IB	7860	ON	MOCH	07-TD-006	MOCH	2006	RMB	11:33	BCCH
IB	7860	ON	MOCH	07-TD-006	MOCH	2006	RMB	11:33	BCCH
IB	7860	ON	MOCH	07-TD-006	MOCH	2006	RMB	11:33	BCCH
IB	7860	ON	MOCH	07-TD-006	MOCH	2006	RMB	11:33	BCCH
IB	7860	ON	MOCH	07-TD-006	MOCH	2006	RMB	11:33	BCCH
IB	7860	ON	MOCH	07-TD-006	MOCH	2006	RMB	11:33	RBNU
IB	7860	ON	MOCH	07-TD-006	MOCH	2006	RMB	11:33	MOCH
IB	7860	ON	MOCH	07-TD-006	MOCH	2006	RMB	11:33	MOCH
IB	7860	ON	MOCH	07-TD-006	MOCH	2006	RMB	11:33	MOCH
IB	7860	ON	MOCH	07-TD-006	MOCH	2006	RMB	11:33	MOCH
IB	7860	ON	MOCH	07-TD-006	MOCH	2006	RMB	11:33	MOCH
IB	7860	ON	MOCH	07-TD-006	MOCH	2006	RMB	11:33	BCCH
IB	7860	ON	MOCH	07-TD-006	MOCH	2006	RMB	11:33	RBNU
IB	7860	ON	MOCH	07-TD-006	MOCH	2006	RMB	11:33	RBNU
IB	7860	ON	MOCH	07-TD-006	MOCH	2006	RMB	11:33	RBNU
IB	7860	ON	MOCH	07-TD-006	MOCH	2006	RMB	11:33	RBNU
IB	7860	ON	MOCH	07-TD-006	MOCH	2006	RMB	11:33	MOCH
IB	7860	ON	MOCH	07-TD-006	MOCH	2006	RMB	11:33	BCCH
IB	70721	ON	MOCH	07-IB-014	MOCH	2006	BMR	7:31	MOCH
IB	70721	ON	MOCH	07-IB-014	MOCH	2006	BMR	7:31	BCCH
IB	70721	ON	MOCH	07-IB-014	MOCH	2006	BMR	7:31	BCCH
IB	70721	ON	MOCH	07-IB-014	MOCH	2006	BMR	7:31	BCCH
IB	70721	ON	MOCH	07-IB-014	MOCH	2006	BMR	7:31	BCCH

IB	70721	ON	MOCH	07-IB-014	MOCH	2006	BMR	7:31	MOCH
IB	70721	ON	MOCH	07-IB-014	MOCH	2006	BMR	7:31	MOCH
IB	70721	ON	MOCH	07-IB-014	MOCH	2006	BMR	7:31	BCCH
IB	70721	ON	MOCH	07-IB-014	MOCH	2006	BMR	7:31	MOCH
IB	70721	ON	MOCH	07-IB-014	MOCH	2006	BMR	7:31	MOCH
IB	70721	ON	MOCH	07-IB-014	MOCH	2006	BMR	7:31	MOCH
IB	70721	ON	MOCH	07-IB-014	MOCH	2006	BMR	7:31	MOCH
IB	70721	ON	MOCH	07-IB-014	MOCH	2006	BMR	7:31	BCCH
IB	70721	ON	MOCH	07-IB-014	MOCH	2006	BMR	7:31	BCCH
IB	70721	ON	MOCH	07-IB-014	MOCH	2006	BMR	7:31	BCCH
IB	70721	ON	MOCH	07-IB-014	MOCH	2006	BMR	7:31	MOCH
IB	70721	ON	MOCH	07-IB-014	MOCH	2006	BMR	7:31	MOCH
IB	70721	ON	MOCH	07-IB-014	MOCH	2006	BMR	7:31	MOCH
IB	70721	ON	MOCH	07-IB-014	MOCH	2006	BMR	7:31	MOCH
IB	70721	ON	MOCH	07-IB-014	MOCH	2006	BMR	7:31	BCCH
IB	70721	ON	MOCH	07-IB-014	MOCH	2006	BMR	7:31	MOCH
IB	70721	ON	MOCH	07-IB-014	MOCH	2006	BMR	7:31	MOCH
IB	70721	ON	MOCH	07-IB-014	MOCH	2006	BMR	7:31	BCCH
IB	70721	ON	MOCH	07-IB-014	MOCH	2006	BMR	7:31	BCCH
IB	70721	ON	MOCH	07-IB-014	MOCH	2006	BMR	7:31	BCCH
IB	70721	ON	MOCH	07-IB-014	MOCH	2006	BMR	7:31	BCCH
IB	70721	ON	MOCH	07-IB-014	MOCH	2006	BMR	7:31	BCCH
IB	70721	ON	MOCH	07-IB-014	MOCH	2006	BMR	7:31	BCCH
IB	70721	ON	MOCH	07-IB-014	MOCH	2006	BMR	7:31	BCCH
IB	70721	ON	MOCH	07-IB-014	MOCH	2006	BMR	7:31	MOCH
IB	70721	ON	MOCH	07-IB-014	MOCH	2006	BMR	7:31	BCCH
IB	70721	ON	MOCH	07-IB-014	MOCH	2006	BMR	7:31	MOCH
IB	70721	ON	MOCH	07-IB-014	MOCH	2006	BMR	7:31	RBNU
IB	70102	ON	MOCH	07-AE-013	MOCH	2006	RBM	9:46	MOCH
IB	70102	ON	MOCH	07-AE-013	MOCH	2006	RBM	9:46	MOCH
IB	70102	ON	MOCH	07-AE-013	MOCH	2006	RBM	9:46	MOCH
IB	70102	ON	MOCH	07-AE-013	MOCH	2006	RBM	9:46	MOCH
IB	70102	ON	MOCH	07-AE-013	MOCH	2006	RBM	9:46	RBNU
IB	70102	ON	MOCH	07-AE-013	MOCH	2006	RBM	9:46	MOCH
IB	70102	ON	MOCH	07-AE-013	MOCH	2006	RBM	9:46	MOCH
IB	70102	ON	MOCH	07-AE-013	MOCH	2006	RBM	9:46	MOCH
IB	70102	ON	MOCH	07-AE-013	MOCH	2006	RBM	9:46	RBNU
IB	70102	ON	MOCH	07-AE-013	MOCH	2006	RBM	9:46	MOCH
IB	70102	ON	MOCH	07-AE-013	MOCH	2006	RBM	9:46	MOCH
IB	70102	ON	MOCH	07-AE-013	MOCH	2006	RBM	9:46	MOCH
IB	70102	ON	MOCH	07-AE-013	MOCH	2006	RBM	9:46	MOCH
IB	70102	ON	MOCH	07-AE-013	MOCH	2006	RBM	9:46	MOCH
IB	70102	ON	MOCH	07-AE-013	MOCH	2006	RBM	9:46	RBNU
IB	70102	ON	MOCH	07-AE-013	MOCH	2006	RBM	9:46	RBNU

IB	70102	ON	MOCH	07-AE-013	MOCH	2006	RBM	9:46	MOCH
IB	70102	ON	MOCH	07-AE-013	MOCH	2006	RBM	9:46	MOCH
IB	70102	ON	MOCH	07-AE-013	MOCH	2006	RBM	9:46	RBNU
IB	70102	ON	MOCH	07-AE-013	MOCH	2006	RBM	9:46	RBNU
IB	70102	ON	MOCH	07-AE-013	MOCH	2006	RBM	9:46	MOCH
IB	70102	ON	MOCH	07-AE-013	MOCH	2006	RBM	9:46	MOCH
IB	70102	ON	MOCH	07-AE-013	MOCH	2006	RBM	9:46	MOCH
IB	70102	ON	MOCH	07-AE-013	MOCH	2006	RBM	9:46	BCCH
IB	70102	ON	MOCH	07-AE-013	MOCH	2006	RBM	9:46	BCCH
IB	70102	ON	MOCH	07-AE-013	MOCH	2006	RBM	9:46	BCCH
IB	70102	ON	MOCH	07-AE-013	MOCH	2006	RBM	9:46	BCCH
IB	70102	ON	MOCH	07-AE-013	MOCH	2006	RBM	9:46	BCCH
IB	70102	ON	MOCH	07-AE-013	MOCH	2006	RBM	9:46	RBNU
IB	70102	ON	MOCH	07-AE-013	MOCH	2006	RBM	9:46	RBNU
IB	70102	ON	MOCH	07-AE-013	MOCH	2006	RBM	9:46	RBNU
IB	70102	ON	MOCH	07-AE-013	MOCH	2006	RBM	9:46	RBNU
IB	70102	ON	MOCH	07-AE-013	MOCH	2006	RBM	9:46	RBNU
IB	70102	ON	MOCH	07-AE-013	MOCH	2006	RBM	9:46	RBNU
IB	70102	ON	MOCH	07-AE-013	MOCH	2006	RBM	9:46	RBNU
IB	70102	ON	MOCH	07-AE-013	MOCH	2006	RBM	9:46	BCCH
IB	70102	ON	MOCH	07-AE-013	MOCH	2006	RBM	9:46	RBNU
IB	70102	ON	MOCH	07-AE-013	MOCH	2006	RBM	9:46	RBNU
IB	70102	ON	MOCH	07-AE-013	MOCH	2006	RBM	9:46	BCCH
IB	70102	ON	MOCH	07-AE-013	MOCH	2006	RBM	9:46	MOCH
IB	7028	ON	MOCH	07-LP-042	RBNU	2006	BMR	10:48	RBNU
IB	7028	ON	MOCH	07-LP-042	RBNU	2006	BMR	10:48	RBNU
IB	7028	ON	MOCH	07-LP-042	RBNU	2006	BMR	10:48	RBNU
IB	7028	ON	MOCH	07-LP-042	RBNU	2006	BMR	10:48	RBNU
IB	7028	ON	MOCH	07-LP-042	RBNU	2006	BMR	10:48	RBNU
IB	7028	ON	MOCH	07-LP-042	RBNU	2006	BMR	10:48	RBNU
IB	7028	ON	MOCH	07-LP-042	RBNU	2006	BMR	10:48	RBNU
IB	7028	ON	MOCH	07-LP-042	RBNU	2006	BMR	10:48	RBNU
IB	7028	ON	MOCH	07-LP-042	RBNU	2006	BMR	10:48	RBNU
IB	7028	ON	MOCH	07-LP-042	RBNU	2006	BMR	10:48	RBNU
IB	7028	ON	MOCH	07-LP-042	RBNU	2006	BMR	10:48	RBNU
IB	7028	ON	MOCH	07-LP-042	RBNU	2006	BMR	10:48	RBNU
IB	7028	ON	MOCH	07-LP-042	RBNU	2006	BMR	10:48	RBNU
IB	7028	ON	MOCH	07-LP-042	RBNU	2006	BMR	10:48	BCCH
IB	7028	ON	MOCH	07-LP-042	RBNU	2006	BMR	10:48	BCCH
IB	7028	ON	MOCH	07-LP-042	RBNU	2006	BMR	10:48	BCCH
IB	7028	ON	MOCH	07-LP-042	RBNU	2006	BMR	10:48	BCCH
IB	7028	ON	MOCH	07-LP-042	RBNU	2006	BMR	10:48	BCCH
IB	7028	ON	MOCH	07-LP-042	RBNU	2006	BMR	10:48	BCCH
IB	7028	ON	MOCH	07-LP-042	RBNU	2006	BMR	10:48	MOCH

IB	7028	ON	MOCH	07-LP-042	RBNU	2006	BMR	10:48	RBNU
IB	70122	ON	MOCH	08-AE-020	RBNU	2006	BMR	10:15	MOCH
IB	70122	ON	MOCH	08-AE-020	RBNU	2006	BMR	10:15	MOCH
IB	70122	ON	MOCH	08-AE-020	RBNU	2006	BMR	10:15	MOCH
IB	70122	ON	MOCH	08-AE-020	RBNU	2006	BMR	10:15	MOCH
IB	70122	ON	MOCH	08-AE-020	RBNU	2006	BMR	10:15	MOCH
IB	70122	ON	MOCH	08-AE-020	RBNU	2006	BMR	10:15	MOCH
IB	70122	ON	MOCH	08-AE-020	RBNU	2006	BMR	10:15	MOCH
IB	70122	ON	MOCH	08-AE-020	RBNU	2006	BMR	10:15	MOCH
IB	70122	ON	MOCH	08-AE-020	RBNU	2006	BMR	10:15	MOCH
IB	70122	ON	MOCH	08-AE-020	RBNU	2006	BMR	10:15	MOCH
IB	70122	ON	MOCH	08-AE-020	RBNU	2006	BMR	10:15	MOCH
IB	70122	ON	MOCH	08-AE-020	RBNU	2006	BMR	10:15	MOCH
IB	70122	ON	MOCH	08-AE-020	RBNU	2006	BMR	10:15	MOCH
IB	70122	ON	MOCH	08-AE-020	RBNU	2006	BMR	10:15	MOCH
IB	70122	ON	MOCH	08-AE-020	RBNU	2006	BMR	10:15	RBNU
IB	70122	ON	MOCH	08-AE-020	RBNU	2006	BMR	10:15	RBNU
IB	70122	ON	MOCH	08-AE-020	RBNU	2006	BMR	10:15	RBNU
IB	70122	ON	MOCH	08-AE-020	RBNU	2006	BMR	10:15	RBNU
IB	70122	ON	MOCH	08-AE-020	RBNU	2006	BMR	10:15	MOCH
IB	70122	ON	MOCH	08-AE-020	RBNU	2006	BMR	10:15	MOCH
IB	70122	ON	MOCH	08-AE-020	RBNU	2006	BMR	10:15	RBNU
IB	70122	ON	MOCH	08-AE-020	RBNU	2006	BMR	10:15	MOCH
IB	70122	ON	MOCH	08-AE-020	RBNU	2006	BMR	10:15	MOCH
IB	70122	ON	MOCH	08-AE-020	RBNU	2006	BMR	10:15	RBNU
IB	70122	ON	MOCH	08-AE-020	RBNU	2006	BMR	10:15	RBNU
IB	70122	ON	MOCH	08-AE-020	RBNU	2006	BMR	10:15	RBNU
IB	70122	ON	MOCH	08-AE-020	RBNU	2006	BMR	10:15	RBNU
IB	70122	ON	MOCH	08-AE-020	RBNU	2006	BMR	10:15	RBNU
IB	70122	ON	MOCH	08-AE-020	RBNU	2006	BMR	10:15	RBNU
IB	70122	ON	MOCH	08-AE-020	RBNU	2006	BMR	10:15	RBNU
IB	70122	ON	MOCH	08-AE-020	RBNU	2006	BMR	10:15	RBNU
IB	70122	ON	MOCH	08-AE-020	RBNU	2006	BMR	10:15	MOCH
IB	70122	ON	MOCH	08-AE-020	RBNU	2006	BMR	10:15	RBNU
IB	70122	ON	MOCH	08-AE-020	RBNU	2006	BMR	10:15	RBNU
IB	70122	ON	MOCH	08-AE-020	RBNU	2006	BMR	10:15	MOCH
IB	70122	ON	MOCH	08-AE-020	RBNU	2006	BMR	10:15	MOCH
IB	70122	ON	MOCH	08-AE-020	RBNU	2006	BMR	10:15	RBNU
IB	70122	ON	MOCH	08-AE-020	RBNU	2006	BMR	10:15	RBNU
IB	70122	ON	MOCH	08-AE-020	RBNU	2006	BMR	10:15	RBNU
IB	70122	ON	MOCH	08-AE-020	RBNU	2006	BMR	10:15	RBNU
IB	70122	ON	MOCH	08-AE-020	RBNU	2006	BMR	10:15	RBNU
IB	70122	ON	MOCH	08-AE-020	RBNU	2006	BMR	10:15	RBNU
IB	70122	ON	MOCH	08-AE-020	RBNU	2006	BMR	10:15	RBNU

IB	70122	ON	MOCH	08-AE-020	RBNU	2006	BMR	10:15	RBNU
IB	70122	ON	MOCH	08-AE-020	RBNU	2006	BMR	10:15	MOCH
IB	70122	ON	MOCH	08-AE-020	RBNU	2006	BMR	10:15	MOCH
IB	70122	ON	MOCH	08-AE-020	RBNU	2006	BMR	10:15	MOCH
IB	70122	ON	MOCH	08-AE-020	RBNU	2006	BMR	10:15	MOCH
IB	70122	ON	MOCH	08-AE-020	RBNU	2006	BMR	10:15	BCCH
IB	70122	ON	MOCH	08-AE-020	RBNU	2006	BMR	10:15	MOCH
IB	70122	ON	MOCH	08-AE-020	RBNU	2006	BMR	10:15	RBNU
ARN, IB	70147	ON	MOCH	07-AE-004	MOCH	2005	MRB	10:58 AM	RBNU
ARN, IB	70147	ON	MOCH	07-AE-004	MOCH	2005	MRB	10:58 AM	RBNU
ARN, IB	70147	ON	MOCH	07-AE-004	MOCH	2005	MRB	10:58 AM	BCCH
ARN, IB	70147	ON	MOCH	07-AE-004	MOCH	2005	MRB	10:58 AM	BCCH
ARN, IB	70147	ON	MOCH	07-AE-004	MOCH	2005	MRB	10:58 AM	BCCH
ARN, IB	70147	ON	MOCH	07-AE-004	MOCH	2005	MRB	10:58 AM	BCCH
ARN, IB	70147	ON	MOCH	07-AE-004	MOCH	2005	MRB	10:58 AM	BCCH
ARN, IB	70147	ON	MOCH	07-AE-004	MOCH	2005	MRB	10:58 AM	RBNU
ARN, IB	70147	ON	MOCH	07-AE-004	MOCH	2005	MRB	10:58 AM	RBNU
ARN, IB	70147	ON	MOCH	07-AE-004	MOCH	2005	MRB	10:58 AM	RBNU
ARN, IB	70147	ON	MOCH	07-AE-004	MOCH	2005	MRB	10:58 AM	MOCH
ARN, IB	70147	ON	MOCH	07-AE-004	MOCH	2005	MRB	10:58 AM	BCCH
ARN, IB	70147	ON	MOCH	07-AE-004	MOCH	2005	MRB	10:58 AM	BCCH
ARN, IB	70147	ON	MOCH	07-AE-004	MOCH	2005	MRB	10:58 AM	BCCH
ARN, IB	70147	ON	MOCH	07-AE-004	MOCH	2005	MRB	10:58 AM	MOCH
ARN, IB	70147	ON	MOCH	07-AE-004	MOCH	2005	MRB	10:58 AM	RBNU
ARN, IB	70147	ON	MOCH	07-AE-004	MOCH	2005	MRB	10:58 AM	BCCH
ARN, IB	7788	ON	MOCH	07-ARN-00	MOCH	2005	BMR	9:13 AM	BCCH
ARN, IB	7788	ON	MOCH	07-ARN-00	MOCH	2005	BMR	9:13 AM	BCCH
ARN, IB	7788	ON	MOCH	07-ARN-00	MOCH	2005	BMR	9:13 AM	BCCH
ARN, IB	7788	ON	MOCH	07-ARN-00	MOCH	2005	BMR	9:13 AM	MOCH
ARN, IB	7788	ON	MOCH	07-ARN-00	MOCH	2005	BMR	9:13 AM	MOCH
ARN, IB	7788	ON	MOCH	07-ARN-00	MOCH	2005	BMR	9:13 AM	MOCH
ARN, IB	7788	ON	MOCH	07-ARN-00	MOCH	2005	BMR	9:13 AM	BCCH
ARN, IB	7788	ON	MOCH	07-ARN-00	MOCH	2005	BMR	9:13 AM	BCCH
ARN, IB	7788	ON	MOCH	07-ARN-00	MOCH	2005	BMR	9:13 AM	MOCH
ARN, IB	7788	ON	MOCH	07-ARN-00	MOCH	2005	BMR	9:13 AM	MOCH
ARN, IB	7788	ON	MOCH	07-ARN-00	MOCH	2005	BMR	9:13 AM	MOCH
ARN, IB	7788	ON	MOCH	07-ARN-00	MOCH	2005	BMR	9:13 AM	MOCH
ARN, IB	7788	ON	MOCH	07-ARN-00	MOCH	2005	BMR	9:13 AM	BCCH
ARN, IB	7788	ON	MOCH	07-ARN-00	MOCH	2005	BMR	9:13 AM	RBNU
ARN, IB	7788	ON	MOCH	07-ARN-00	MOCH	2005	BMR	9:13 AM	RBNU
ARN, IB	7788	ON	MOCH	07-ARN-00	MOCH	2005	BMR	9:13 AM	BCCH
ARN, IB	7788	ON	MOCH	07-ARN-00	MOCH	2005	BMR	9:13 AM	BCCH
ARN, IB	7788	ON	MOCH	07-ARN-00	MOCH	2005	BMR	9:13 AM	RBNU
ARN, IB	7788	ON	MOCH	07-ARN-00	MOCH	2005	BMR	9:13 AM	RBNU

ARN, IB	7788	ON	MOCH	07-ARN-00	MOCH	2005	BMR	9:13 AM	MOCH
ARN, IB	7788	ON	MOCH	07-ARN-00	MOCH	2005	BMR	9:13 AM	BCCH
ARN, IB	7788	ON	MOCH	07-ARN-00	MOCH	2005	BMR	9:13 AM	RBNU
ARN, IB	7788	ON	MOCH	07-ARN-00	MOCH	2005	BMR	9:13 AM	MOCH
ARN, IB	7788	ON	MOCH	07-ARN-00	MOCH	2005	BMR	9:13 AM	BCCH
ARN, IB	7788	ON	MOCH	07-ARN-00	MOCH	2005	BMR	9:13 AM	MOCH
ARN, IB	7788	ON	MOCH	07-ARN-00	MOCH	2005	BMR	9:13 AM	RBNU
ARN, IB	7788	ON	MOCH	07-ARN-00	MOCH	2005	BMR	9:13 AM	MOCH
ARN, IB	70502	ON	MOCH	07-ARN-01	MOCH	2005	RMB	10:42 AM	BCCH
ARN, IB	70502	ON	MOCH	07-ARN-01	MOCH	2005	RMB	10:42 AM	MOCH
ARN, IB	70502	ON	MOCH	07-ARN-01	MOCH	2005	RMB	10:42 AM	MOCH
ARN, IB	70502	ON	MOCH	07-ARN-01	MOCH	2005	RMB	10:42 AM	BCCH
ARN, IB	70502	ON	MOCH	07-ARN-01	MOCH	2005	RMB	10:42 AM	MOCH
ARN, IB	70502	ON	MOCH	07-ARN-01	MOCH	2005	RMB	10:42 AM	MOCH
ARN, IB	70502	ON	MOCH	07-ARN-01	MOCH	2005	RMB	10:42 AM	MOCH
ARN, IB	70502	ON	MOCH	07-ARN-01	MOCH	2005	RMB	10:42 AM	MOCH
ARN, IB	70502	ON	MOCH	07-ARN-01	MOCH	2005	RMB	10:42 AM	MOCH
ARN, IB	70502	ON	MOCH	07-ARN-01	MOCH	2005	RMB	10:42 AM	RBNU
ARN, IB	70502	ON	MOCH	07-ARN-01	MOCH	2005	RMB	10:42 AM	MOCH
ARN, IB	70502	ON	MOCH	07-ARN-01	MOCH	2005	RMB	10:42 AM	MOCH
ARN, IB	70502	ON	MOCH	07-ARN-01	MOCH	2005	RMB	10:42 AM	BCCH
ARN, IB	70502	ON	MOCH	07-ARN-01	MOCH	2005	RMB	10:42 AM	BCCH
ARN, IB	70502	ON	MOCH	07-ARN-01	MOCH	2005	RMB	10:42 AM	BCCH
ARN, IB	70502	ON	MOCH	07-ARN-01	MOCH	2005	RMB	10:42 AM	BCCH
ARN, IB	70502	ON	MOCH	07-ARN-01	MOCH	2005	RMB	10:42 AM	RBNU
ARN, IB	70502	ON	MOCH	07-ARN-01	MOCH	2005	RMB	10:42 AM	RBNU
ARN, IB	70502	ON	MOCH	07-ARN-01	MOCH	2005	RMB	10:42 AM	RBNU
ARN, IB	70502	ON	MOCH	07-ARN-01	MOCH	2005	RMB	10:42 AM	RBNU
ARN, IB	70502	ON	MOCH	07-ARN-01	MOCH	2005	RMB	10:42 AM	RBNU
ARN, IB	70502	ON	MOCH	07-ARN-01	MOCH	2005	RMB	10:42 AM	RBNU
ARN, IB	70502	ON	MOCH	07-ARN-01	MOCH	2005	RMB	10:42 AM	RBNU
ARN, IB	70502	ON	MOCH	07-ARN-01	MOCH	2005	RMB	10:42 AM	MOCH
ARN, IB	70502	ON	MOCH	07-ARN-01	MOCH	2005	RMB	10:42 AM	MOCH
ARN, IB	70502	ON	MOCH	07-ARN-01	MOCH	2005	RMB	10:42 AM	BCCH
ARN, IB	70502	ON	MOCH	07-ARN-01	MOCH	2005	RMB	10:42 AM	RBNU
ARN, IB	70502	ON	MOCH	07-ARN-01	MOCH	2005	RMB	10:42 AM	MOCH
ARN, IB	70502	ON	MOCH	07-ARN-01	MOCH	2005	RMB	10:42 AM	RBNU
ARN, IB	70502	ON	MOCH	07-ARN-01	MOCH	2005	RMB	10:42 AM	MOCH
ARN, IB	70502	ON	MOCH	07-ARN-01	MOCH	2005	RMB	10:42 AM	BCCH
ARN, IB	70502	ON	MOCH	07-ARN-01	MOCH	2005	RMB	10:42 AM	RBNU
ARN, IB	70502	ON	MOCH	07-ARN-01	MOCH	2005	RMB	10:42 AM	RBNU
ARN, IB	70502	ON	MOCH	07-ARN-01	MOCH	2005	RMB	10:42 AM	RBNU
ARN, IB	7563	ON	MOCH	07-IB-002	MOCH	2004	BMR	10:36 AM	RBNU
ARN, IB	7563	ON	MOCH	07-IB-002	MOCH	2004	BMR	10:36 AM	BCCH

ARN, IB	7563	ON	MOCH	07-IB-002	MOCH	2004	BMR	10:36 AM	MOCH
ARN, IB	7563	ON	MOCH	07-IB-002	MOCH	2004	BMR	10:36 AM	MOCH
ARN, IB	7563	ON	MOCH	07-IB-002	MOCH	2004	BMR	10:36 AM	MOCH
ARN, IB	7563	ON	MOCH	07-IB-002	MOCH	2004	BMR	10:36 AM	MOCH
ARN, IB	7563	ON	MOCH	07-IB-002	MOCH	2004	BMR	10:36 AM	RBNU
ARN, IB	7563	ON	MOCH	07-IB-002	MOCH	2004	BMR	10:36 AM	BCCH
ARN, IB	7563	ON	MOCH	07-IB-002	MOCH	2004	BMR	10:36 AM	BCCH
ARN, IB	7563	ON	MOCH	07-IB-002	MOCH	2004	BMR	10:36 AM	MOCH
ARN, IB	7563	ON	MOCH	07-IB-002	MOCH	2004	BMR	10:36 AM	MOCH
ARN, IB	7563	ON	MOCH	07-IB-002	MOCH	2004	BMR	10:36 AM	MOCH
ARN, IB	7563	ON	MOCH	07-IB-002	MOCH	2004	BMR	10:36 AM	MOCH
ARN, IB	7563	ON	MOCH	07-IB-002	MOCH	2004	BMR	10:36 AM	MOCH
ARN, IB	7563	ON	MOCH	07-IB-002	MOCH	2004	BMR	10:36 AM	MOCH
ARN, IB	7563	ON	MOCH	07-IB-002	MOCH	2004	BMR	10:36 AM	RBNU
ARN, IB	7563	ON	MOCH	07-IB-002	MOCH	2004	BMR	10:36 AM	RBNU
ARN, IB	7563	ON	MOCH	07-IB-002	MOCH	2004	BMR	10:36 AM	MOCH
ARN, IB	7563	ON	MOCH	07-IB-002	MOCH	2004	BMR	10:36 AM	BCCH
ARN, IB	7563	ON	MOCH	07-IB-002	MOCH	2004	BMR	10:36 AM	MOCH
ARN, IB	7563	ON	MOCH	07-IB-002	MOCH	2004	BMR	10:36 AM	BCCH
ARN, IB	7563	ON	MOCH	07-IB-002	MOCH	2004	BMR	10:36 AM	MOCH
ARN, IB	7563	ON	MOCH	07-IB-002	MOCH	2004	BMR	10:36 AM	RBNU
IB	70122	ON	MOCH	07-AE-020	RBNU	2005	BMR	11:58 AM	MOCH
IB	70122	ON	MOCH	07-AE-020	RBNU	2005	BMR	11:58 AM	MOCH
IB	70122	ON	MOCH	07-AE-020	RBNU	2005	BMR	11:58 AM	MOCH
IB	70122	ON	MOCH	07-AE-020	RBNU	2005	BMR	11:58 AM	RBNU
IB	70122	ON	MOCH	07-AE-020	RBNU	2005	BMR	11:58 AM	RBNU
IB	70122	ON	MOCH	07-AE-020	RBNU	2005	BMR	11:58 AM	MOCH
IB	70122	ON	MOCH	07-AE-020	RBNU	2005	BMR	11:58 AM	RBNU
IB	70122	ON	MOCH	07-AE-020	RBNU	2005	BMR	11:58 AM	RBNU
IB	70122	ON	MOCH	07-AE-020	RBNU	2005	BMR	11:58 AM	RBNU
IB	70122	ON	MOCH	07-AE-020	RBNU	2005	BMR	11:58 AM	RBNU
IB	70122	ON	MOCH	07-AE-020	RBNU	2005	BMR	11:58 AM	RBNU
IB	70122	ON	MOCH	07-AE-020	RBNU	2005	BMR	11:58 AM	BCCH
IB	70122	ON	MOCH	07-AE-020	RBNU	2005	BMR	11:58 AM	BCCH
IB	70122	ON	MOCH	07-AE-020	RBNU	2005	BMR	11:58 AM	BCCH
IB	70122	ON	MOCH	07-AE-020	RBNU	2005	BMR	11:58 AM	BCCH
IB	70122	ON	MOCH	07-AE-020	RBNU	2005	BMR	11:58 AM	MOCH
IB	70122	ON	MOCH	07-AE-020	RBNU	2005	BMR	11:58 AM	RBNU
IB	70133	ON	MOCH	07-KS-046	MOCH	2005	MBR	11:23 AM	MOCH
IB	70133	ON	MOCH	07-KS-046	MOCH	2005	MBR	11:23 AM	MOCH
IB	70133	ON	MOCH	07-KS-046	MOCH	2005	MBR	11:23 AM	MOCH
IB	70133	ON	MOCH	07-KS-046	MOCH	2005	MBR	11:23 AM	MOCH
IB	70133	ON	MOCH	07-KS-046	MOCH	2005	MBR	11:23 AM	MOCH
IB	70133	ON	MOCH	07-KS-046	MOCH	2005	MBR	11:23 AM	MOCH

IB	70133	ON	MOCH	07-KS-046 MOCH	2005	MBR	11:23 AM MOCH
IB	70133	ON	MOCH	07-KS-046 MOCH	2005	MBR	11:23 AM MOCH
IB	70133	ON	MOCH	07-KS-046 MOCH	2005	MBR	11:23 AM MOCH
IB	70133	ON	MOCH	07-KS-046 MOCH	2005	MBR	11:23 AM MOCH
IB	70133	ON	MOCH	07-KS-046 MOCH	2005	MBR	11:23 AM MOCH
IB	70133	ON	MOCH	07-KS-046 MOCH	2005	MBR	11:23 AM MOCH
IB	70133	ON	MOCH	07-KS-046 MOCH	2005	MBR	11:23 AM MOCH
IB	70133	ON	MOCH	07-KS-046 MOCH	2005	MBR	11:23 AM MOCH
IB	70133	ON	MOCH	07-KS-046 MOCH	2005	MBR	11:23 AM MOCH
IB	70133	ON	MOCH	07-KS-046 MOCH	2005	MBR	11:23 AM MOCH
IB	70133	ON	MOCH	07-KS-046 MOCH	2005	MBR	11:23 AM MOCH
IB	70133	ON	MOCH	07-KS-046 MOCH	2005	MBR	11:23 AM MOCH
IB	70133	ON	MOCH	07-KS-046 MOCH	2005	MBR	11:23 AM MOCH
IB	70133	ON	MOCH	07-KS-046 MOCH	2005	MBR	11:23 AM MOCH
IB	70133	ON	MOCH	07-KS-046 MOCH	2005	MBR	11:23 AM MOCH
IB	70133	ON	MOCH	07-KS-046 MOCH	2005	MBR	11:23 AM MOCH
IB	70133	ON	MOCH	07-KS-046 MOCH	2005	MBR	11:23 AM BCCH
IB	70133	ON	MOCH	07-KS-046 MOCH	2005	MBR	11:23 AM RBNU
ARN	7744	ON	MOCH	07-KS-039 MOCH	2006	RBM	7:53 AM MOCH
ARN	7744	ON	MOCH	07-KS-039 MOCH	2006	RBM	7:53 AM BCCH
ARN	7744	ON	MOCH	07-KS-039 MOCH	2006	RBM	7:53 AM MOCH
ARN	7744	ON	MOCH	07-KS-039 MOCH	2006	RBM	7:53 AM MOCH
ARN	7744	ON	MOCH	07-KS-039 MOCH	2006	RBM	7:53 AM MOCH
ARN	7744	ON	MOCH	07-KS-039 MOCH	2006	RBM	7:53 AM BCCH
ARN	7744	ON	MOCH	07-KS-039 MOCH	2006	RBM	7:53 AM BCCH
ARN	7744	ON	MOCH	07-KS-039 MOCH	2006	RBM	7:53 AM BCCH
ARN	7744	ON	MOCH	07-KS-039 MOCH	2006	RBM	7:53 AM
ARN	7744	ON	MOCH	07-KS-039 MOCH	2006	RBM	7:53 AM BCCH
ARN	7744	ON	MOCH	07-KS-039 MOCH	2006	RBM	7:53 AM MOCH
ARN	7744	ON	MOCH	07-KS-039 MOCH	2006	RBM	7:53 AM RBNU
ARN	7744	ON	MOCH	07-KS-039 MOCH	2006	RBM	7:53 AM
IB	7440	ON	MOCH	07-KS-037 MOCH	2006	MBR	9:20 AM BCCH
IB	7440	ON	MOCH	07-KS-037 MOCH	2006	MBR	9:20 AM BCCH
IB	7440	ON	MOCH	07-KS-037 MOCH	2006	MBR	9:20 AM BCCH
IB	7440	ON	MOCH	07-KS-037 MOCH	2006	MBR	9:20 AM BCCH
IB	7440	ON	MOCH	07-KS-037 MOCH	2006	MBR	9:20 AM BCCH
IB	7440	ON	MOCH	07-KS-037 MOCH	2006	MBR	9:20 AM BCCH
IB	7440	ON	MOCH	07-KS-037 MOCH	2006	MBR	9:20 AM BCCH
IB	7440	ON	MOCH	07-KS-037 MOCH	2006	MBR	9:20 AM BCCH
IB	7440	ON	MOCH	07-KS-037 MOCH	2006	MBR	9:20 AM MOCH
IB	7440	ON	MOCH	07-KS-037 MOCH	2006	MBR	9:20 AM BCCH
IB	7440	ON	MOCH	07-KS-037 MOCH	2006	MBR	9:20 AM RBNU
IB	70102	ON	MOCH	07-AE-013 MOCH	2006	RBM	7:45 AM RBNU
IB	70102	ON	MOCH	07-AE-013 MOCH	2006	RBM	7:45 AM RBNU

IB	70102	ON	MOCH	07-AE-013	MOCH	2006	RBM	7:45 AM	RBNU
IB	70102	ON	MOCH	07-AE-013	MOCH	2006	RBM	7:45 AM	RBNU
IB	70102	ON	MOCH	07-AE-013	MOCH	2006	RBM	7:45 AM	RBNU
IB	70102	ON	MOCH	07-AE-013	MOCH	2006	RBM	7:45 AM	BCCH
IB	70102	ON	MOCH	07-AE-013	MOCH	2006	RBM	7:45 AM	MOCH
IB	70102	ON	MOCH	07-AE-013	MOCH	2006	RBM	7:45 AM	MOCH
IB	70102	ON	MOCH	07-AE-013	MOCH	2006	RBM	7:45 AM	MOCH
IB	70102	ON	MOCH	07-AE-013	MOCH	2006	RBM	7:45 AM	MOCH
IB	70102	ON	MOCH	07-AE-013	MOCH	2006	RBM	7:45 AM	RBNU
IB	70102	ON	MOCH	07-AE-013	MOCH	2006	RBM	7:45 AM	BCCH
IB	70102	ON	MOCH	07-AE-013	MOCH	2006	RBM	7:45 AM	MOCH
IB	7512	ON	MOCH	07-TD-016	RBNU	2004	RBM	10:22 AM	BCCH
IB	7512	ON	MOCH	07-TD-016	RBNU	2004	RBM	10:22 AM	BCCH
IB	7512	ON	MOCH	07-TD-016	RBNU	2004	RBM	10:22 AM	BCCH
IB	7512	ON	MOCH	07-TD-016	RBNU	2004	RBM	10:22 AM	BCCH
IB	7512	ON	MOCH	07-TD-016	RBNU	2004	RBM	10:22 AM	BCCH
IB	7512	ON	MOCH	07-TD-016	RBNU	2004	RBM	10:22 AM	MOCH
IB	7512	ON	MOCH	07-TD-016	RBNU	2004	RBM	10:22 AM	RBNU
IB	7512	ON	MOCH	07-TD-016	RBNU	2004	RBM	10:22 AM	RBNU
IB	7512	ON	MOCH	07-TD-016	RBNU	2004	RBM	10:22 AM	RBNU
IB	7512	ON	MOCH	07-TD-016	RBNU	2004	RBM	10:22 AM	RBNU
IB	7512	ON	MOCH	07-TD-016	RBNU	2004	RBM	10:22 AM	BCCH
IB	7512	ON	MOCH	07-TD-016	RBNU	2004	RBM	10:22 AM	BCCH
IB	7512	ON	MOCH	07-TD-016	RBNU	2004	RBM	10:22 AM	MOCH
IB	7512	ON	MOCH	07-TD-016	RBNU	2004	RBM	10:22 AM	MOCH
IB	7512	ON	MOCH	07-TD-016	RBNU	2004	RBM	10:22 AM	RBNU
IB	7512	ON	MOCH	07-TD-016	RBNU	2004	RBM	10:22 AM	RBNU
IB	7512	ON	MOCH	07-TD-016	RBNU	2004	RBM	10:22 AM	RBNU
IB	7512	ON	MOCH	07-TD-016	RBNU	2004	RBM	10:22 AM	MOCH
IB	7512	ON	MOCH	07-TD-016	RBNU	2004	RBM	10:22 AM	MOCH
IB	7512	ON	MOCH	07-TD-016	RBNU	2004	RBM	10:22 AM	MOCH
IB	7512	ON	MOCH	07-TD-016	RBNU	2004	RBM	10:22 AM	RBNU
IB	7512	ON	MOCH	07-TD-016	RBNU	2004	RBM	10:22 AM	BCCH
IB	7512	ON	MOCH	07-TD-016	RBNU	2004	RBM	10:22 AM	MOCH
IB	7063	ON	MOCH	07-IB-004	MOCH	2004	RBM	11:17 AM	MOCH
IB	7063	ON	MOCH	07-IB-004	MOCH	2004	RBM	11:17 AM	RBNU
IB	7063	ON	MOCH	07-IB-004	MOCH	2004	RBM	11:17 AM	BCCH
IB	7063	ON	MOCH	07-IB-004	MOCH	2004	RBM	11:17 AM	MOCH
IB	7063	ON	MOCH	07-IB-004	MOCH	2004	RBM	11:17 AM	BCCH
IB	7063	ON	MOCH	07-IB-004	MOCH	2004	RBM	11:17 AM	RBNU
IB	7063	ON	MOCH	07-IB-004	MOCH	2004	RBM	11:17 AM	BCCH
IB	7063	ON	MOCH	07-IB-004	MOCH	2004	RBM	11:17 AM	MOCH
IB	7563	ON	MOCH	07-IB-002	MOCH	2004	MBR	8:37 AM	MOCH
IB	7563	ON	MOCH	07-IB-002	MOCH	2004	MBR	8:37 AM	MOCH

IB	7563	ON	MOCH	07-IB-002	MOCH	2004	MBR	8:37	AM	MOCH
IB	7563	ON	MOCH	07-IB-002	MOCH	2004	MBR	8:37	AM	RBNU
IB	7563	ON	MOCH	07-IB-002	MOCH	2004	MBR	8:37	AM	RBNU
IB	7563	ON	MOCH	07-IB-002	MOCH	2004	MBR	8:37	AM	RBNU
IB	7563	ON	MOCH	07-IB-002	MOCH	2004	MBR	8:37	AM	BCCH
IB	7563	ON	MOCH	07-IB-002	MOCH	2004	MBR	8:37	AM	MOCH
IB	7563	ON	MOCH	07-IB-002	MOCH	2004	MBR	8:37	AM	RBNU
IB	7788	ON	MOCH	07-ARN-00	MOCH	2005	RBM	9:25	AM	BCCH
IB	7788	ON	MOCH	07-ARN-00	MOCH	2005	RBM	9:25	AM	MOCH
IB	7788	ON	MOCH	07-ARN-00	MOCH	2005	RBM	9:25	AM	MOCH
IB	7788	ON	MOCH	07-ARN-00	MOCH	2005	RBM	9:25	AM	RBNU
IB	7788	ON	MOCH	07-ARN-00	MOCH	2005	RBM	9:25	AM	MOCH
IB	7788	ON	MOCH	07-ARN-00	MOCH	2005	RBM	9:25	AM	MOCH
IB	7788	ON	MOCH	07-ARN-00	MOCH	2005	RBM	9:25	AM	MOCH
IB	7788	ON	MOCH	07-ARN-00	MOCH	2005	RBM	9:25	AM	BCCH
IB	7788	ON	MOCH	07-ARN-00	MOCH	2005	RBM	9:25	AM	MOCH
IB	7788	ON	MOCH	07-ARN-00	MOCH	2005	RBM	9:25	AM	MOCH
IB	7788	ON	MOCH	07-ARN-00	MOCH	2005	RBM	9:25	AM	RBNU
IB	7788	ON	MOCH	07-ARN-00	MOCH	2005	RBM	9:25	AM	RBNU
IB	7788	ON	MOCH	07-ARN-00	MOCH	2005	RBM	9:25	AM	RBNU
IB	7788	ON	MOCH	07-ARN-00	MOCH	2005	RBM	9:25	AM	BCCH
IB	7788	ON	MOCH	07-ARN-00	MOCH	2005	RBM	9:25	AM	MOCH
IB	7788	ON	MOCH	07-ARN-00	MOCH	2005	RBM	9:25	AM	RBNU
IB	70102	ON	MOCH	07-AE-013	MOCH	2006	BMR	7:45	AM	RBNU
IB	70102	ON	MOCH	07-AE-013	MOCH	2006	BMR	7:45	AM	RBNU
IB	70102	ON	MOCH	07-AE-013	MOCH	2006	BMR	7:45	AM	RBNU
IB	70102	ON	MOCH	07-AE-013	MOCH	2006	BMR	7:45	AM	RBNU
IB	70102	ON	MOCH	07-AE-013	MOCH	2006	BMR	7:45	AM	BCCH
IB	70102	ON	MOCH	07-AE-013	MOCH	2006	BMR	7:45	AM	BCCH
IB	70102	ON	MOCH	07-AE-013	MOCH	2006	BMR	7:45	AM	MOCH
IB	70102	ON	MOCH	07-AE-013	MOCH	2006	BMR	7:45	AM	RBNU
IB	70102	ON	MOCH	07-AE-013	MOCH	2006	BMR	7:45	AM	RBNU
IB	70102	ON	MOCH	07-AE-013	MOCH	2006	BMR	7:45	AM	RBNU
IB	70102	ON	MOCH	07-AE-013	MOCH	2006	BMR	7:45	AM	RBNU
IB	70102	ON	MOCH	07-AE-013	MOCH	2006	BMR	7:45	AM	RBNU
IB	70102	ON	MOCH	07-AE-013	MOCH	2006	BMR	7:45	AM	RBNU
IB	70102	ON	MOCH	07-AE-013	MOCH	2006	BMR	7:45	AM	RBNU
IB	70102	ON	MOCH	07-AE-013	MOCH	2006	BMR	7:45	AM	RBNU
IB	70102	ON	MOCH	07-AE-013	MOCH	2006	BMR	7:45	AM	RBNU
IB	70102	ON	MOCH	07-AE-013	MOCH	2006	BMR	7:45	AM	BCCH
IB	70102	ON	MOCH	07-AE-013	MOCH	2006	BMR	7:45	AM	BCCH
IB	70102	ON	MOCH	07-AE-013	MOCH	2006	BMR	7:45	AM	BCCH
IB	70102	ON	MOCH	07-AE-013	MOCH	2006	BMR	7:45	AM	RBNU
IB	70102	ON	MOCH	07-AE-013	MOCH	2006	BMR	7:45	AM	BCCH

IB	70102	ON	MOCH	07-AE-013	MOCH	2006	BMR	7:45 AM	MOCH
IB	70102	ON	MOCH	07-AE-013	MOCH	2006	BMR	7:45 AM	RBNU
IB	7440	ON	MOCH	07-KS-037	MOCH	2006	RMB	10:40 AM	BCCH
IB	7440	ON	MOCH	07-KS-037	MOCH	2006	RMB	10:40 AM	MOCH
IB	7440	ON	MOCH	07-KS-037	MOCH	2006	RMB	10:40 AM	RBNU
IB	7440	ON	MOCH	07-KS-037	MOCH	2006	RMB	10:40 AM	BCCH
HK	70153	ON	MOCH	08-HK-008	RBNU	2007	MBR	10:50	
HK	70153	ON	MOCH	08-HK-008	RBNU	2007	MBR	10:50	BCCH
HK	70153	ON	MOCH	08-HK-008	RBNU	2007	MBR	10:50	BCCH
HK	70153	ON	MOCH	08-HK-008	RBNU	2007	MBR	10:50	BCCH
HK	70153	ON	MOCH	08-HK-008	RBNU	2007	MBR	10:50	BCCH
HK	70153	ON	MOCH	08-HK-008	RBNU	2007	MBR	10:50	
HK	70153	ON	MOCH	08-HK-008	RBNU	2007	MBR	10:50	MOCH
HK	70153	ON	MOCH	08-HK-008	RBNU	2007	MBR	10:50	BCCH
HK	70153	ON	MOCH	08-HK-008	RBNU	2007	MBR	10:50	RBNU
HK	70153	ON	MOCH	08-HK-008	RBNU	2007	MBR	10:50	
HK	70153	ON	MOCH	08-HK-008	RBNU	2007	MBR	10:50	
HK	7839	ON	MOCH	08-ARN-02	MOCH	2007	BMR	9:58	MOCH
HK	7839	ON	MOCH	08-ARN-02	MOCH	2007	BMR	9:58	MOCH
HK	7839	ON	MOCH	08-ARN-02	MOCH	2007	BMR	9:58	MOCH
HK	7839	ON	MOCH	08-ARN-02	MOCH	2007	BMR	9:58	MOCH
HK	7839	ON	MOCH	08-ARN-02	MOCH	2007	BMR	9:58	MOCH
HK	7839	ON	MOCH	08-ARN-02	MOCH	2007	BMR	9:58	MOCH
HK	7839	ON	MOCH	08-ARN-02	MOCH	2007	BMR	9:58	MOCH
HK	7839	ON	MOCH	08-ARN-02	MOCH	2007	BMR	9:58	MOCH
HK	7839	ON	MOCH	08-ARN-02	MOCH	2007	BMR	9:58	MOCH
HK	7839	ON	MOCH	08-ARN-02	MOCH	2007	BMR	9:58	MOCH
HK	7839	ON	MOCH	08-ARN-02	MOCH	2007	BMR	9:58	MOCH
HK	7839	ON	MOCH	08-ARN-02	MOCH	2007	BMR	9:58	RBNU
HK	7839	ON	MOCH	08-ARN-02	MOCH	2007	BMR	9:58	RBNU
HK	7839	ON	MOCH	08-ARN-02	MOCH	2007	BMR	9:58	BCCH
HK	7839	ON	MOCH	08-ARN-02	MOCH	2007	BMR	9:58	MOCH
HK	7839	ON	MOCH	08-ARN-02	MOCH	2007	BMR	9:58	MOCH
HK	7839	ON	MOCH	08-ARN-02	MOCH	2007	BMR	9:58	MOCH
HK	7839	ON	MOCH	08-ARN-02	MOCH	2007	BMR	9:58	BCCH
HK	7839	ON	MOCH	08-ARN-02	MOCH	2007	BMR	9:58	RBNU
HK	7839	ON	MOCH	08-ARN-02	MOCH	2007	BMR	9:58	MOCH
HK	7839	ON	MOCH	08-ARN-02	MOCH	2007	BMR	9:58	RBNU
HK	7839	ON	MOCH	08-ARN-02	MOCH	2007	BMR	9:58	
HK	7839	ON	MOCH	08-ARN-02	MOCH	2007	BMR	9:58	MOCH
HK	7839	ON	MOCH	08-ARN-02	MOCH	2007	BMR	9:58	RBNU
HK	7839	ON	MOCH	08-ARN-02	MOCH	2007	BMR	9:58	BCCH
HK	7839	ON	MOCH	08-ARN-02	MOCH	2007	BMR	9:58	BCCH
HK	7839	ON	MOCH	08-ARN-02	MOCH	2007	BMR	9:58	MOCH

HK	7839	ON	MOCH	08-ARN-02	MOCH	2007	BMR	9:58	RBNU
HK	7839	ON	MOCH	08-ARN-02	MOCH	2007	BMR	9:58	
HK	7839	ON	MOCH	08-ARN-02	MOCH	2007	BMR	9:58	
HK	70102	ON	MOCH	08-LB-032	MOCH	2007	BRM	10:38	BCCH
HK	70102	ON	MOCH	08-LB-032	MOCH	2007	BRM	10:38	MOCH
HK	70102	ON	MOCH	08-LB-032	MOCH	2007	BRM	10:38	BCCH
HK	70102	ON	MOCH	08-LB-032	MOCH	2007	BRM	10:38	BCCH
HK	70102	ON	MOCH	08-LB-032	MOCH	2007	BRM	10:38	MOCH
HK	70102	ON	MOCH	08-LB-032	MOCH	2007	BRM	10:38	MOCH
HK	70102	ON	MOCH	08-LB-032	MOCH	2007	BRM	10:38	MOCH
HK	70102	ON	MOCH	08-LB-032	MOCH	2007	BRM	10:38	BCCH
HK	70102	ON	MOCH	08-LB-032	MOCH	2007	BRM	10:38	BCCH
HK	70102	ON	MOCH	08-LB-032	MOCH	2007	BRM	10:38	BCCH
HK	70102	ON	MOCH	08-LB-032	MOCH	2007	BRM	10:38	RBNU
HK	70102	ON	MOCH	08-LB-032	MOCH	2007	BRM	10:38	RBNU
HK	70102	ON	MOCH	08-LB-032	MOCH	2007	BRM	10:38	MOCH
HK	70102	ON	MOCH	08-LB-032	MOCH	2007	BRM	10:38	RBNU
HK	70102	ON	MOCH	08-LB-032	MOCH	2007	BRM	10:38	MOCH
HK	70102	ON	MOCH	08-LB-032	MOCH	2007	BRM	10:38	MOCH
HK	70102	ON	MOCH	08-LB-032	MOCH	2007	BRM	10:38	RBNU
HK	70102	ON	MOCH	08-LB-032	MOCH	2007	BRM	10:38	RBNU
HK	70102	ON	MOCH	08-LB-032	MOCH	2007	BRM	10:38	RBNU
HK	70102	ON	MOCH	08-LB-032	MOCH	2007	BRM	10:38	
HK	70102	ON	MOCH	08-LB-032	MOCH	2007	BRM	10:38	BCCH
HK	70102	ON	MOCH	08-LB-032	MOCH	2007	BRM	10:38	MOCH
HK	70102	ON	MOCH	08-LB-032	MOCH	2007	BRM	10:38	MOCH
HK	70102	ON	MOCH	08-LB-032	MOCH	2007	BRM	10:38	RBNU
HK	70102	ON	MOCH	08-LB-032	MOCH	2007	BRM	10:38	RBNU
HK	70102	ON	MOCH	08-LB-032	MOCH	2007	BRM	10:38	RBNU
HK	70102	ON	MOCH	08-LB-032	MOCH	2007	BRM	10:38	MOCH
HK	70102	ON	MOCH	08-LB-032	MOCH	2007	BRM	10:38	BCCH
HK	70102	ON	MOCH	08-LB-032	MOCH	2007	BRM	10:38	RBNU
HK	70102	ON	MOCH	08-LB-032	MOCH	2007	BRM	10:38	MOCH
HK	70102	ON	MOCH	08-LB-032	MOCH	2007	BRM	10:38	MOCH
HK	70102	ON	MOCH	08-LB-032	MOCH	2007	BRM	10:38	MOCH
HK	70102	ON	MOCH	08-LB-032	MOCH	2007	BRM	10:38	
HK	7974	ON	MOCH	08-ARN-01	RBNU	2007	RBM	9:19	BCCH
HK	7974	ON	MOCH	08-ARN-01	RBNU	2007	RBM	9:19	BCCH
HK	7974	ON	MOCH	08-ARN-01	RBNU	2007	RBM	9:19	BCCH
HK	7974	ON	MOCH	08-ARN-01	RBNU	2007	RBM	9:19	BCCH
HK	7974	ON	MOCH	08-ARN-01	RBNU	2007	RBM	9:19	MOCH
HK	7974	ON	MOCH	08-ARN-01	RBNU	2007	RBM	9:19	MOCH
HK	7974	ON	MOCH	08-ARN-01	RBNU	2007	RBM	9:19	MOCH
HK	7974	ON	MOCH	08-ARN-01	RBNU	2007	RBM	9:19	MOCH

HK	7974	ON	MOCH	08-ARN-01	RBNU	2007	RBM	9:19	BCCH
HK	7974	ON	MOCH	08-ARN-01	RBNU	2007	RBM	9:19	BCCH
HK	7974	ON	MOCH	08-ARN-01	RBNU	2007	RBM	9:19	MOCH
HK	7974	ON	MOCH	08-ARN-01	RBNU	2007	RBM	9:19	MOCH
HK	7974	ON	MOCH	08-ARN-01	RBNU	2007	RBM	9:19	MOCH
HK	7974	ON	MOCH	08-ARN-01	RBNU	2007	RBM	9:19	MOCH
HK	7974	ON	MOCH	08-ARN-01	RBNU	2007	RBM	9:19	BCCH
HK	7974	ON	MOCH	08-ARN-01	RBNU	2007	RBM	9:19	MOCH
HK	7974	ON	MOCH	08-ARN-01	RBNU	2007	RBM	9:19	MOCH
HK	7974	ON	MOCH	08-ARN-01	RBNU	2007	RBM	9:19	
HK	7974	ON	MOCH	08-ARN-01	RBNU	2007	RBM	9:19	RBNU
HK	7974	ON	MOCH	08-ARN-01	RBNU	2007	RBM	9:19	RBNU
HK	7974	ON	MOCH	08-ARN-01	RBNU	2007	RBM	9:19	BCCH
HK	7974	ON	MOCH	08-ARN-01	RBNU	2007	RBM	9:19	MOCH
HK	7974	ON	MOCH	08-ARN-01	RBNU	2007	RBM	9:19	MOCH
HK	7974	ON	MOCH	08-ARN-01	RBNU	2007	RBM	9:19	
HK	70813	ON	MOCH	08-AB-033	DOWO	2007	RMB	10:45	MOCH
HK	70813	ON	MOCH	08-AB-033	DOWO	2007	RMB	10:45	MOCH
HK	70813	ON	MOCH	08-AB-033	DOWO	2007	RMB	10:45	MOCH
HK	70813	ON	MOCH	08-AB-033	DOWO	2007	RMB	10:45	MOCH
HK	70813	ON	MOCH	08-AB-033	DOWO	2007	RMB	10:45	MOCH
HK	70813	ON	MOCH	08-AB-033	DOWO	2007	RMB	10:45	MOCH
HK	70813	ON	MOCH	08-AB-033	DOWO	2007	RMB	10:45	MOCH
HK	70813	ON	MOCH	08-AB-033	DOWO	2007	RMB	10:45	RBNU
HK	70813	ON	MOCH	08-AB-033	DOWO	2007	RMB	10:45	MOCH
HK	70813	ON	MOCH	08-AB-033	DOWO	2007	RMB	10:45	MOCH
HK	70813	ON	MOCH	08-AB-033	DOWO	2007	RMB	10:45	MOCH
HK	70813	ON	MOCH	08-AB-033	DOWO	2007	RMB	10:45	MOCH
HK	70813	ON	MOCH	08-AB-033	DOWO	2007	RMB	10:45	BCCH
HK	70813	ON	MOCH	08-AB-033	DOWO	2007	RMB	10:45	RBNU
HK	70813	ON	MOCH	08-AB-033	DOWO	2007	RMB	10:45	RBNU
HK	70813	ON	MOCH	08-AB-033	DOWO	2007	RMB	10:45	MOCH
HK	70813	ON	MOCH	08-AB-033	DOWO	2007	RMB	10:45	BCCH
HK	70813	ON	MOCH	08-AB-033	DOWO	2007	RMB	10:45	BCCH
HK	70813	ON	MOCH	08-AB-033	DOWO	2007	RMB	10:45	
HK	70733	ON	MOCH	08-ARN-04	RBNU	2005	BMR	9:16	MOCH
HK	70733	ON	MOCH	08-ARN-04	RBNU	2005	BMR	9:16	RBNU
HK	70733	ON	MOCH	08-ARN-04	RBNU	2005	BMR	9:16	BCCH
HK	70733	ON	MOCH	08-ARN-04	RBNU	2005	BMR	9:16	MOCH
HK	70733	ON	MOCH	08-ARN-04	RBNU	2005	BMR	9:16	RBNU
HK	70733	ON	MOCH	08-ARN-04	RBNU	2005	BMR	9:16	RBNU
HK	70733	ON	MOCH	08-ARN-04	RBNU	2005	BMR	9:16	
HK	70821	ON	MOCH	08-HK-005	MOCH	2007	BRM	10:55	RBNU
HK	70821	ON	MOCH	08-HK-005	MOCH	2007	BRM	10:55	BCCH

HK	70821	ON	MOCH	08-HK-005 MOCH	2007	BRM	10:55 RBNU
HK	70821	ON	MOCH	08-HK-005 MOCH	2007	BRM	10:55 RBNU
HK	70821	ON	MOCH	08-HK-005 MOCH	2007	BRM	10:55 BCCH
HK	70821	ON	MOCH	08-HK-005 MOCH	2007	BRM	10:55 RBNU
HK	70821	ON	MOCH	08-HK-005 MOCH	2007	BRM	10:55 MOCH
HK	70821	ON	MOCH	08-HK-005 MOCH	2007	BRM	10:55 MOCH
HK	70821	ON	MOCH	08-HK-005 MOCH	2007	BRM	10:55
HK	7839	ON	MOCH	08-ARN-02 MOCH	2007	BMR	12:03 BCCH
HK	7839	ON	MOCH	08-ARN-02 MOCH	2007	BMR	12:03 BCCH
HK	7839	ON	MOCH	08-ARN-02 MOCH	2007	BMR	12:03 BCCH
HK	7839	ON	MOCH	08-ARN-02 MOCH	2007	BMR	12:03 BCCH
HK	7839	ON	MOCH	08-ARN-02 MOCH	2007	BMR	12:03 BCCH
HK	7839	ON	MOCH	08-ARN-02 MOCH	2007	BMR	12:03 BCCH
HK	7839	ON	MOCH	08-ARN-02 MOCH	2007	BMR	12:03 BCCH
HK	7839	ON	MOCH	08-ARN-02 MOCH	2007	BMR	12:03 BCCH
HK	7839	ON	MOCH	08-ARN-02 MOCH	2007	BMR	12:03 BCCH
HK	7839	ON	MOCH	08-ARN-02 MOCH	2007	BMR	12:03 BCCH
HK	7839	ON	MOCH	08-ARN-02 MOCH	2007	BMR	12:03 BCCH
HK	7839	ON	MOCH	08-ARN-02 MOCH	2007	BMR	12:03 BCCH
HK	7839	ON	MOCH	08-ARN-02 MOCH	2007	BMR	12:03 BCCH
HK	7839	ON	MOCH	08-ARN-02 MOCH	2007	BMR	12:03 BCCH
HK	7839	ON	MOCH	08-ARN-02 MOCH	2007	BMR	12:03 MOCH
HK	7839	ON	MOCH	08-ARN-02 MOCH	2007	BMR	12:03 MOCH
HK	7839	ON	MOCH	08-ARN-02 MOCH	2007	BMR	12:03 MOCH
HK	7839	ON	MOCH	08-ARN-02 MOCH	2007	BMR	12:03 MOCH
HK	7839	ON	MOCH	08-ARN-02 MOCH	2007	BMR	12:03 MOCH
HK	7839	ON	MOCH	08-ARN-02 MOCH	2007	BMR	12:03 BCCH
HK	7839	ON	MOCH	08-ARN-02 MOCH	2007	BMR	12:03 BCCH
HK	7839	ON	MOCH	08-ARN-02 MOCH	2007	BMR	12:03 MOCH
HK	7839	ON	MOCH	08-ARN-02 MOCH	2007	BMR	12:03 RBNU
HK	7839	ON	MOCH	08-ARN-02 MOCH	2007	BMR	12:03 RBNU
HK	7839	ON	MOCH	08-ARN-02 MOCH	2007	BMR	12:03
HK	7974	ON	MOCH	08-ARN-01 RBNU	2007	RBM	8:13 MOCH
HK	7974	ON	MOCH	08-ARN-01 RBNU	2007	RBM	8:13 MOCH
HK	7974	ON	MOCH	08-ARN-01 RBNU	2007	RBM	8:13 MOCH
HK	7974	ON	MOCH	08-ARN-01 RBNU	2007	RBM	8:13 MOCH
HK	7974	ON	MOCH	08-ARN-01 RBNU	2007	RBM	8:13 MOCH
HK	7974	ON	MOCH	08-ARN-01 RBNU	2007	RBM	8:13 MOCH
HK	7974	ON	MOCH	08-ARN-01 RBNU	2007	RBM	8:13 MOCH
HK	7974	ON	MOCH	08-ARN-01 RBNU	2007	RBM	8:13 MOCH
HK	7974	ON	MOCH	08-ARN-01 RBNU	2007	RBM	8:13 MOCH
HK	7974	ON	MOCH	08-ARN-01 RBNU	2007	RBM	8:13 MOCH
HK	7974	ON	MOCH	08-ARN-01 RBNU	2007	RBM	8:13 MOCH
HK	7974	ON	MOCH	08-ARN-01 RBNU	2007	RBM	8:13 MOCH

HK	7974	ON	MOCH	08-ARN-01	RBNU	2007	RBM	8:13	MOCH
HK	7974	ON	MOCH	08-ARN-01	RBNU	2007	RBM	8:13	RBNU
HK	7974	ON	MOCH	08-ARN-01	RBNU	2007	RBM	8:13	RBNU
HK	7974	ON	MOCH	08-ARN-01	RBNU	2007	RBM	8:13	RBNU
HK	7974	ON	MOCH	08-ARN-01	RBNU	2007	RBM	8:13	MOCH
HK	7974	ON	MOCH	08-ARN-01	RBNU	2007	RBM	8:13	RBNU
HK	7974	ON	MOCH	08-ARN-01	RBNU	2007	RBM	8:13	MOCH
HK	7974	ON	MOCH	08-ARN-01	RBNU	2007	RBM	8:13	RBNU
HK	7974	ON	MOCH	08-ARN-01	RBNU	2007	RBM	8:13	MOCH
HK	7974	ON	MOCH	08-ARN-01	RBNU	2007	RBM	8:13	MOCH
HK	7974	ON	MOCH	08-ARN-01	RBNU	2007	RBM	8:13	
HK	7974	ON	MOCH	08-ARN-01	RBNU	2007	RBM	8:13	RBNU
HK	7974	ON	MOCH	08-ARN-01	RBNU	2007	RBM	8:13	MOCH
HK	7974	ON	MOCH	08-ARN-01	RBNU	2007	RBM	8:13	MOCH
HK	7974	ON	MOCH	08-ARN-01	RBNU	2007	RBM	8:13	MOCH
HK	7974	ON	MOCH	08-ARN-01	RBNU	2007	RBM	8:13	RBNU
HK	7974	ON	MOCH	08-ARN-01	RBNU	2007	RBM	8:13	
HK	70153	ON	MOCH	08-HK-008	RBNU	2007	RBM	9:39	BCCH
HK	70153	ON	MOCH	08-HK-008	RBNU	2007	RBM	9:39	BCCH
HK	70153	ON	MOCH	08-HK-008	RBNU	2007	RBM	9:39	BCCH
HK	70153	ON	MOCH	08-HK-008	RBNU	2007	RBM	9:39	BCCH
HK	70153	ON	MOCH	08-HK-008	RBNU	2007	RBM	9:39	BCCH
HK	70153	ON	MOCH	08-HK-008	RBNU	2007	RBM	9:39	BCCH
HK	70153	ON	MOCH	08-HK-008	RBNU	2007	RBM	9:39	MOCH
HK	70153	ON	MOCH	08-HK-008	RBNU	2007	RBM	9:39	BCCH
HK	70153	ON	MOCH	08-HK-008	RBNU	2007	RBM	9:39	BCCH
HK	70153	ON	MOCH	08-HK-008	RBNU	2007	RBM	9:39	BCCH
HK	70153	ON	MOCH	08-HK-008	RBNU	2007	RBM	9:39	BCCH
HK	70153	ON	MOCH	08-HK-008	RBNU	2007	RBM	9:39	MOCH
HK	70153	ON	MOCH	08-HK-008	RBNU	2007	RBM	9:39	MOCH
HK	70153	ON	MOCH	08-HK-008	RBNU	2007	RBM	9:39	MOCH
HK	70153	ON	MOCH	08-HK-008	RBNU	2007	RBM	9:39	MOCH
HK	70153	ON	MOCH	08-HK-008	RBNU	2007	RBM	9:39	RBNU
HK	70153	ON	MOCH	08-HK-008	RBNU	2007	RBM	9:39	RBNU
HK	70153	ON	MOCH	08-HK-008	RBNU	2007	RBM	9:39	RBNU
HK	70153	ON	MOCH	08-HK-008	RBNU	2007	RBM	9:39	RBNU
HK	70153	ON	MOCH	08-HK-008	RBNU	2007	RBM	9:39	RBNU
HK	70153	ON	MOCH	08-HK-008	RBNU	2007	RBM	9:39	RBNU
HK	70153	ON	MOCH	08-HK-008	RBNU	2007	RBM	9:39	MOCH
HK	70153	ON	MOCH	08-HK-008	RBNU	2007	RBM	9:39	BCCH
HK	70153	ON	MOCH	08-HK-008	RBNU	2007	RBM	9:39	RBNU
HK	70153	ON	MOCH	08-HK-008	RBNU	2007	RBM	9:39	RBNU
HK	70153	ON	MOCH	08-HK-008	RBNU	2007	RBM	9:39	RBNU
HK	70153	ON	MOCH	08-HK-008	RBNU	2007	RBM	9:39	RBNU

HK	70153	ON	MOCH	08-HK-008	RBNU	2007	RBM	9:39	RBNU
HK	70153	ON	MOCH	08-HK-008	RBNU	2007	RBM	9:39	BCCH
HK	70153	ON	MOCH	08-HK-008	RBNU	2007	RBM	9:39	RBNU
HK	70153	ON	MOCH	08-HK-008	RBNU	2007	RBM	9:39	RBNU
HK	70153	ON	MOCH	08-HK-008	RBNU	2007	RBM	9:39	RBNU
HK	70153	ON	MOCH	08-HK-008	RBNU	2007	RBM	9:39	RBNU
HK	70153	ON	MOCH	08-HK-008	RBNU	2007	RBM	9:39	RBNU
HK	70153	ON	MOCH	08-HK-008	RBNU	2007	RBM	9:39	BCCH
HK	70153	ON	MOCH	08-HK-008	RBNU	2007	RBM	9:39	BCCH
HK	70153	ON	MOCH	08-HK-008	RBNU	2007	RBM	9:39	MOCH
HK	70153	ON	MOCH	08-HK-008	RBNU	2007	RBM	9:39	BCCH
HK	70153	ON	MOCH	08-HK-008	RBNU	2007	RBM	9:39	BCCH
HK	70153	ON	MOCH	08-HK-008	RBNU	2007	RBM	9:39	MOCH
HK	70153	ON	MOCH	08-HK-008	RBNU	2007	RBM	9:39	BCCH
HK	70153	ON	MOCH	08-HK-008	RBNU	2007	RBM	9:39	RBNU
HK	70153	ON	MOCH	08-HK-008	RBNU	2007	RBM	9:39	RBNU
HK	70153	ON	MOCH	08-HK-008	RBNU	2007	RBM	9:39	BCCH
HK	70153	ON	MOCH	08-HK-008	RBNU	2007	RBM	9:39	MOCH
HK	70153	ON	MOCH	08-HK-008	RBNU	2007	RBM	9:39	MOCH
HK	70153	ON	MOCH	08-HK-008	RBNU	2007	RBM	9:39	
HK	7837	ON	MOCH	08-CA-027	TRES	2006	RMB	9:42	RBNU
HK	7837	ON	MOCH	08-CA-027	TRES	2006	RMB	9:42	BCCH
HK	7837	ON	MOCH	08-CA-027	TRES	2006	RMB	9:42	BCCH
HK	7837	ON	MOCH	08-CA-027	TRES	2006	RMB	9:42	RBNU
HK	7837	ON	MOCH	08-CA-027	TRES	2006	RMB	9:42	MOCH
HK	7837	ON	MOCH	08-CA-027	TRES	2006	RMB	9:42	BCCH
HK	7837	ON	MOCH	08-CA-027	TRES	2006	RMB	9:42	RBNU
HK	7837	ON	MOCH	08-CA-027	TRES	2006	RMB	9:42	MOCH
HK	7837	ON	MOCH	08-CA-027	TRES	2006	RMB	9:42	BCCH
HK	7837	ON	MOCH	08-CA-027	TRES	2006	RMB	9:42	RBNU
HK	7837	ON	MOCH	08-CA-027	TRES	2006	RMB	9:42	MOCH
HK	7837	ON	MOCH	08-CA-027	TRES	2006	RMB	9:42	MOCH
HK	7837	ON	MOCH	08-CA-027	TRES	2006	RMB	9:42	RBNU
HK	7837	ON	MOCH	08-CA-027	TRES	2006	RMB	9:42	MOCH
HK	7837	ON	MOCH	08-CA-027	TRES	2006	RMB	9:42	BCCH
HK	7837	ON	MOCH	08-CA-027	TRES	2006	RMB	9:42	BCCH
HK	7837	ON	MOCH	08-CA-027	TRES	2006	RMB	9:42	
HK	70770	ON	MOCH	08-ARN-02	MOCH	2007	MBR	11:27	MOCH
HK	70770	ON	MOCH	08-ARN-02	MOCH	2007	MBR	11:27	MOCH
HK	70770	ON	MOCH	08-ARN-02	MOCH	2007	MBR	11:27	MOCH
HK	70770	ON	MOCH	08-ARN-02	MOCH	2007	MBR	11:27	MOCH
HK	70770	ON	MOCH	08-ARN-02	MOCH	2007	MBR	11:27	MOCH
HK	70770	ON	MOCH	08-ARN-02	MOCH	2007	MBR	11:27	MOCH
HK	70770	ON	MOCH	08-ARN-02	MOCH	2007	MBR	11:27	MOCH

HK	70770	ON	MOCH	08-ARN-02	MOCH	2007	MBR	11:27	MOCH
HK	70770	ON	MOCH	08-ARN-02	MOCH	2007	MBR	11:27	MOCH
HK	70770	ON	MOCH	08-ARN-02	MOCH	2007	MBR	11:27	MOCH
HK	70770	ON	MOCH	08-ARN-02	MOCH	2007	MBR	11:27	MOCH
HK	70770	ON	MOCH	08-ARN-02	MOCH	2007	MBR	11:27	MOCH
HK	70770	ON	MOCH	08-ARN-02	MOCH	2007	MBR	11:27	MOCH
HK	70770	ON	MOCH	08-ARN-02	MOCH	2007	MBR	11:27	MOCH
HK	70770	ON	MOCH	08-ARN-02	MOCH	2007	MBR	11:27	MOCH
HK	70770	ON	MOCH	08-ARN-02	MOCH	2007	MBR	11:27	RBNU
HK	70770	ON	MOCH	08-ARN-02	MOCH	2007	MBR	11:27	BCCH
HK	70770	ON	MOCH	08-ARN-02	MOCH	2007	MBR	11:27	MOCH
HK	70770	ON	MOCH	08-ARN-02	MOCH	2007	MBR	11:27	RBNU
HK	70770	ON	MOCH	08-ARN-02	MOCH	2007	MBR	11:27	BCCH
HK	70770	ON	MOCH	08-ARN-02	MOCH	2007	MBR	11:27	RBNU
HK	70770	ON	MOCH	08-ARN-02	MOCH	2007	MBR	11:27	RBNU
HK	70770	ON	MOCH	08-ARN-02	MOCH	2007	MBR	11:27	RBNU
HK	70770	ON	MOCH	08-ARN-02	MOCH	2007	MBR	11:27	MOCH
HK	70770	ON	MOCH	08-ARN-02	MOCH	2007	MBR	11:27	BCCH
HK	70770	ON	MOCH	08-ARN-02	MOCH	2007	MBR	11:27	RBNU
HK	70770	ON	MOCH	08-ARN-02	MOCH	2007	MBR	11:27	RBNU
HK	70770	ON	MOCH	08-ARN-02	MOCH	2007	MBR	11:27	
HK	70770	ON	MOCH	08-ARN-02	MOCH	2007	RBM	10:21	RBNU
HK	70770	ON	MOCH	08-ARN-02	MOCH	2007	RBM	10:21	BCCH
HK	70770	ON	MOCH	08-ARN-02	MOCH	2007	RBM	10:21	BCCH
HK	70770	ON	MOCH	08-ARN-02	MOCH	2007	RBM	10:21	BCCH
HK	70770	ON	MOCH	08-ARN-02	MOCH	2007	RBM	10:21	BCCH
HK	70770	ON	MOCH	08-ARN-02	MOCH	2007	RBM	10:21	BCCH
HK	70770	ON	MOCH	08-ARN-02	MOCH	2007	RBM	10:21	BCCH
HK	70770	ON	MOCH	08-ARN-02	MOCH	2007	RBM	10:21	BCCH
HK	70770	ON	MOCH	08-ARN-02	MOCH	2007	RBM	10:21	BCCH
HK	70770	ON	MOCH	08-ARN-02	MOCH	2007	RBM	10:21	MOCH
HK	70770	ON	MOCH	08-ARN-02	MOCH	2007	RBM	10:21	MOCH
HK	70770	ON	MOCH	08-ARN-02	MOCH	2007	RBM	10:21	MOCH
HK	70770	ON	MOCH	08-ARN-02	MOCH	2007	RBM	10:21	MOCH
HK	70770	ON	MOCH	08-ARN-02	MOCH	2007	RBM	10:21	MOCH
HK	70770	ON	MOCH	08-ARN-02	MOCH	2007	RBM	10:21	MOCH
HK	70770	ON	MOCH	08-ARN-02	MOCH	2007	RBM	10:21	MOCH
HK	70770	ON	MOCH	08-ARN-02	MOCH	2007	RBM	10:21	MOCH
HK	70770	ON	MOCH	08-ARN-02	MOCH	2007	RBM	10:21	RBNU
HK	70770	ON	MOCH	08-ARN-02	MOCH	2007	RBM	10:21	MOCH
HK	70770	ON	MOCH	08-ARN-02	MOCH	2007	RBM	10:21	RBNU
HK	70770	ON	MOCH	08-ARN-02	MOCH	2007	RBM	10:21	RBNU
HK	70770	ON	MOCH	08-ARN-02	MOCH	2007	RBM	10:21	RBNU
HK	70770	ON	MOCH	08-ARN-02	MOCH	2007	RBM	10:21	RBNU

HK	70770	ON	MOCH	08-ARN-02	MOCH	2007	RBM	10:21	RBNU
HK	70770	ON	MOCH	08-ARN-02	MOCH	2007	RBM	10:21	RBNU
HK	70770	ON	MOCH	08-ARN-02	MOCH	2007	RBM	10:21	BCCH
HK	70770	ON	MOCH	08-ARN-02	MOCH	2007	RBM	10:21	MOCH
HK	70770	ON	MOCH	08-ARN-02	MOCH	2007	RBM	10:21	MOCH
HK	70770	ON	MOCH	08-ARN-02	MOCH	2007	RBM	10:21	
HK	7974	ON	MOCH	08-ARN-01	RBNU	2007	RBM	8:04	RBNU
HK	7974	ON	MOCH	08-ARN-01	RBNU	2007	RBM	8:04	BCCH
HK	7974	ON	MOCH	08-ARN-01	RBNU	2007	RBM	8:04	BCCH
HK	7974	ON	MOCH	08-ARN-01	RBNU	2007	RBM	8:04	BCCH
HK	7974	ON	MOCH	08-ARN-01	RBNU	2007	RBM	8:04	BCCH
HK	7974	ON	MOCH	08-ARN-01	RBNU	2007	RBM	8:04	BCCH
HK	7974	ON	MOCH	08-ARN-01	RBNU	2007	RBM	8:04	BCCH
HK	7974	ON	MOCH	08-ARN-01	RBNU	2007	RBM	8:04	BCCH
HK	7974	ON	MOCH	08-ARN-01	RBNU	2007	RBM	8:04	BCCH
HK	7974	ON	MOCH	08-ARN-01	RBNU	2007	RBM	8:04	MOCH
HK	7974	ON	MOCH	08-ARN-01	RBNU	2007	RBM	8:04	MOCH
HK	7974	ON	MOCH	08-ARN-01	RBNU	2007	RBM	8:04	MOCH
HK	7974	ON	MOCH	08-ARN-01	RBNU	2007	RBM	8:04	MOCH
HK	7974	ON	MOCH	08-ARN-01	RBNU	2007	RBM	8:04	MOCH
HK	7974	ON	MOCH	08-ARN-01	RBNU	2007	RBM	8:04	MOCH
HK	7974	ON	MOCH	08-ARN-01	RBNU	2007	RBM	8:04	MOCH
HK	7974	ON	MOCH	08-ARN-01	RBNU	2007	RBM	8:04	MOCH
HK	7974	ON	MOCH	08-ARN-01	RBNU	2007	RBM	8:04	MOCH
HK	7974	ON	MOCH	08-ARN-01	RBNU	2007	RBM	8:04	MOCH
HK	7974	ON	MOCH	08-ARN-01	RBNU	2007	RBM	8:04	MOCH
HK	7974	ON	MOCH	08-ARN-01	RBNU	2007	RBM	8:04	RBNU
HK	7974	ON	MOCH	08-ARN-01	RBNU	2007	RBM	8:04	MOCH
HK	7974	ON	MOCH	08-ARN-01	RBNU	2007	RBM	8:04	MOCH
HK	7974	ON	MOCH	08-ARN-01	RBNU	2007	RBM	8:04	BCCH
HK	7974	ON	MOCH	08-ARN-01	RBNU	2007	RBM	8:04	RBNU
HK	7974	ON	MOCH	08-ARN-01	RBNU	2007	RBM	8:04	MOCH
HK	7974	ON	MOCH	08-ARN-01	RBNU	2007	RBM	8:04	BCCH
HK	7974	ON	MOCH	08-ARN-01	RBNU	2007	RBM	8:04	RBNU
HK	7974	ON	MOCH	08-ARN-01	RBNU	2007	RBM	8:04	BCCH
HK	7974	ON	MOCH	08-ARN-01	RBNU	2007	RBM	8:04	MOCH
HK	7974	ON	MOCH	08-ARN-01	RBNU	2007	RBM	8:04	MOCH
HK	7974	ON	MOCH	08-ARN-01	RBNU	2007	RBM	8:04	
HK	70102	ON	MOCH	08-LB-032	MOCH	2007	BMR	8:37	
HK	70102	ON	MOCH	08-LB-032	MOCH	2007	BMR	8:37	
HK	70102	ON	MOCH	08-LB-032	MOCH	2007	BMR	8:37	BCCH
HK	70102	ON	MOCH	08-LB-032	MOCH	2007	BMR	8:37	BCCH
HK	70102	ON	MOCH	08-LB-032	MOCH	2007	BMR	8:37	BCCH
HK	70102	ON	MOCH	08-LB-032	MOCH	2007	BMR	8:37	MOCH

HK	70102	ON	MOCH	08-LB-032	MOCH	2007	BMR	8:37	MOCH
HK	70102	ON	MOCH	08-LB-032	MOCH	2007	BMR	8:37	MOCH
HK	70102	ON	MOCH	08-LB-032	MOCH	2007	BMR	8:37	MOCH
HK	70102	ON	MOCH	08-LB-032	MOCH	2007	BMR	8:37	RBNU
HK	70102	ON	MOCH	08-LB-032	MOCH	2007	BMR	8:37	RBNU
HK	70102	ON	MOCH	08-LB-032	MOCH	2007	BMR	8:37	BCCH
HK	70102	ON	MOCH	08-LB-032	MOCH	2007	BMR	8:37	MOCH
HK	70102	ON	MOCH	08-LB-032	MOCH	2007	BMR	8:37	BCCH
HK	70102	ON	MOCH	08-LB-032	MOCH	2007	BMR	8:37	BCCH
HK	70102	ON	MOCH	08-LB-032	MOCH	2007	BMR	8:37	BCCH
HK	70102	ON	MOCH	08-LB-032	MOCH	2007	BMR	8:37	MOCH
HK	70102	ON	MOCH	08-LB-032	MOCH	2007	BMR	8:37	MOCH
HK	70102	ON	MOCH	08-LB-032	MOCH	2007	BMR	8:37	RBNU
HK	70102	ON	MOCH	08-LB-032	MOCH	2007	BMR	8:37	BCCH
HK	70102	ON	MOCH	08-LB-032	MOCH	2007	BMR	8:37	MOCH
HK	70102	ON	MOCH	08-LB-032	MOCH	2007	BMR	8:37	MOCH
HK	70102	ON	MOCH	08-LB-032	MOCH	2007	BMR	8:37	BCCH
HK	70102	ON	MOCH	08-LB-032	MOCH	2007	BMR	8:37	BCCH
HK	70102	ON	MOCH	08-LB-032	MOCH	2007	BMR	8:37	BCCH
HK	70102	ON	MOCH	08-LB-032	MOCH	2007	BMR	8:37	MOCH
HK	70102	ON	MOCH	08-LB-032	MOCH	2007	BMR	8:37	RBNU
HK	70102	ON	MOCH	08-LB-032	MOCH	2007	BMR	8:37	RBNU
HK	70102	ON	MOCH	08-LB-032	MOCH	2007	BMR	8:37	
HK	70733	ON	MOCH	08-ARN-04	RBNU	2005	MRB	9:44	MOCH
HK	70733	ON	MOCH	08-ARN-04	RBNU	2005	MRB	9:44	RBNU
HK	70733	ON	MOCH	08-ARN-04	RBNU	2005	MRB	9:44	RBNU
HK	70733	ON	MOCH	08-ARN-04	RBNU	2005	MRB	9:44	MOCH
HK	70733	ON	MOCH	08-ARN-04	RBNU	2005	MRB	9:44	RBNU
HK	70733	ON	MOCH	08-ARN-04	RBNU	2005	MRB	9:44	MOCH
HK	70733	ON	MOCH	08-ARN-04	RBNU	2005	MRB	9:44	MOCH
HK	70733	ON	MOCH	08-ARN-04	RBNU	2005	MRB	9:44	RBNU
HK	70733	ON	MOCH	08-ARN-04	RBNU	2005	MRB	9:44	BCCH
HK	70733	ON	MOCH	08-ARN-04	RBNU	2005	MRB	9:44	BCCH
HK	70733	ON	MOCH	08-ARN-04	RBNU	2005	MRB	9:44	
AE	70821	ON	MOCH	08-HK-005	MOCH	2007	RMB	11:52 AM	BCCH
AE	70821	ON	MOCH	08-HK-005	MOCH	2007	RMB	11:52 AM	BCCH
AE	70821	ON	MOCH	08-HK-005	MOCH	2007	RMB	11:52 AM	BCCH
AE	70821	ON	MOCH	08-HK-005	MOCH	2007	RMB	11:52 AM	MOCH
AE	70821	ON	MOCH	08-HK-005	MOCH	2007	RMB	11:52 AM	BCCH
AE	70821	ON	MOCH	08-HK-005	MOCH	2007	RMB	11:52 AM	BCCH
AE	70821	ON	MOCH	08-HK-005	MOCH	2007	RMB	11:52 AM	RBNU
AE	70821	ON	MOCH	08-HK-005	MOCH	2007	RMB	11:52 AM	RBNU
AE	70821	ON	MOCH	08-HK-005	MOCH	2007	RMB	11:52 AM	RBNU
AE	70821	ON	MOCH	08-HK-005	MOCH	2007	RMB	11:52 AM	MOCH

AE	70821	ON	MOCH	08-HK-005	MOCH	2007	RMB	11:52 AM	MOCH
AE	70821	ON	MOCH	08-HK-005	MOCH	2007	RMB	11:52 AM	MOCH
AE	70821	ON	MOCH	08-HK-005	MOCH	2007	RMB	11:52 AM	BCCH
AE	70821	ON	MOCH	08-HK-005	MOCH	2007	RMB	11:52 AM	BCCH
AE	70821	ON	MOCH	08-HK-005	MOCH	2007	RMB	11:52 AM	BCCH
AE	70821	ON	MOCH	08-HK-005	MOCH	2007	RMB	11:52 AM	MOCH
AE	70821	ON	MOCH	08-HK-005	MOCH	2007	RMB	11:52 AM	MOCH
AE	70821	ON	MOCH	08-HK-005	MOCH	2007	RMB	11:52 AM	BCCH
AE	70821	ON	MOCH	08-HK-005	MOCH	2007	RMB	11:52 AM	BCCH
AE	70821	ON	MOCH	08-HK-005	MOCH	2007	RMB	11:52 AM	MOCH
AE	70821	ON	MOCH	08-HK-005	MOCH	2007	RMB	11:52 AM	MOCH
AE	70821	ON	MOCH	08-HK-005	MOCH	2007	RMB	11:52 AM	BCCH
AE	70821	ON	MOCH	08-HK-005	MOCH	2007	RMB	11:52 AM	MOCH
AE	70821	ON	MOCH	08-HK-005	MOCH	2007	RMB	11:52 AM	RBNU
AE	70821	ON	MOCH	08-HK-005	MOCH	2007	RMB	11:52 AM	MOCH
AE	70821	ON	MOCH	08-HK-005	MOCH	2007	RMB	11:52 AM	RBNU
AE	70821	ON	MOCH	08-HK-005	MOCH	2007	RMB	11:52 AM	MOCH
AE	70821	ON	MOCH	08-HK-005	MOCH	2007	RMB	11:52 AM	BCCH
HK	70153	ON	MOCH	08-HK-008	RBNU	2007	MRB	10:21	BCCH
HK	70153	ON	MOCH	08-HK-008	RBNU	2007	MRB	10:21	BCCH
HK	70153	ON	MOCH	08-HK-008	RBNU	2007	MRB	10:21	BCCH
HK	70153	ON	MOCH	08-HK-008	RBNU	2007	MRB	10:21	BCCH
HK	70153	ON	MOCH	08-HK-008	RBNU	2007	MRB	10:21	BCCH
HK	70153	ON	MOCH	08-HK-008	RBNU	2007	MRB	10:21	BCCH
HK	70153	ON	MOCH	08-HK-008	RBNU	2007	MRB	10:21	BCCH
HK	70153	ON	MOCH	08-HK-008	RBNU	2007	MRB	10:21	MOCH
HK	70153	ON	MOCH	08-HK-008	RBNU	2007	MRB	10:21	MOCH
HK	70153	ON	MOCH	08-HK-008	RBNU	2007	MRB	10:21	RBNU
HK	70153	ON	MOCH	08-HK-008	RBNU	2007	MRB	10:21	BCCH
HK	70153	ON	MOCH	08-HK-008	RBNU	2007	MRB	10:21	BCCH
HK	70153	ON	MOCH	08-HK-008	RBNU	2007	MRB	10:21	BCCH
HK	70153	ON	MOCH	08-HK-008	RBNU	2007	MRB	10:21	BCCH
HK	70153	ON	MOCH	08-HK-008	RBNU	2007	MRB	10:21	RBNU
HK	70153	ON	MOCH	08-HK-008	RBNU	2007	MRB	10:21	BCCH
HK	70153	ON	MOCH	08-HK-008	RBNU	2007	MRB	10:21	MOCH
HK	70153	ON	MOCH	08-HK-008	RBNU	2007	MRB	10:21	MOCH
HK	70153	ON	MOCH	08-HK-008	RBNU	2007	MRB	10:21	MOCH
HK	70153	ON	MOCH	08-HK-008	RBNU	2007	MRB	10:21	MOCH
HK	70153	ON	MOCH	08-HK-008	RBNU	2007	MRB	10:21	RBNU
HK	70153	ON	MOCH	08-HK-008	RBNU	2007	MRB	10:21	RBNU
HK	70153	ON	MOCH	08-HK-008	RBNU	2007	MRB	10:21	RBNU
HK	70153	ON	MOCH	08-HK-008	RBNU	2007	MRB	10:21	BCCH
HK	70153	ON	MOCH	08-HK-008	RBNU	2007	MRB	10:21	BCCH
HK	70153	ON	MOCH	08-HK-008	RBNU	2007	MRB	10:21	RBNU

HK	70153	ON	MOCH	08-HK-008	RBNU	2007	MRB	10:21	RBNU
HK	70153	ON	MOCH	08-HK-008	RBNU	2007	MRB	10:21	BCCH
HK	70153	ON	MOCH	08-HK-008	RBNU	2007	MRB	10:21	RBNU
HK	70153	ON	MOCH	08-HK-008	RBNU	2007	MRB	10:21	RBNU
HK	70153	ON	MOCH	08-HK-008	RBNU	2007	MRB	10:21	RBNU
HK	70153	ON	MOCH	08-HK-008	RBNU	2007	MRB	10:21	RBNU
HK	70153	ON	MOCH	08-HK-008	RBNU	2007	MRB	10:21	RBNU
HK	70153	ON	MOCH	08-HK-008	RBNU	2007	MRB	10:21	RBNU
HK	70153	ON	MOCH	08-HK-008	RBNU	2007	MRB	10:21	BCCH
HK	70153	ON	MOCH	08-HK-008	RBNU	2007	MRB	10:21	RBNU
HK	70153	ON	MOCH	08-HK-008	RBNU	2007	MRB	10:21	BCCH
HK	70153	ON	MOCH	08-HK-008	RBNU	2007	MRB	10:21	MOCH
HK	70153	ON	MOCH	08-HK-008	RBNU	2007	MRB	10:21	RBNU
HK	70153	ON	MOCH	08-HK-008	RBNU	2007	MRB	10:21	BCCH
HK	70153	ON	MOCH	08-HK-008	RBNU	2007	MRB	10:21	BCCH
HK	70153	ON	MOCH	08-HK-008	RBNU	2007	MRB	10:21	
AE	70831	ON	MOCH	08-AB-039	MOCH	2007	RMB	7:43 AM	BCCH
AE	70831	ON	MOCH	08-AB-039	MOCH	2007	RMB	7:43 AM	BCCH
AE	70831	ON	MOCH	08-AB-039	MOCH	2007	RMB	7:43 AM	RBNU
AE	70831	ON	MOCH	08-AB-039	MOCH	2007	RMB	7:43 AM	MOCH
AE	70831	ON	MOCH	08-AB-039	MOCH	2007	RMB	7:43 AM	MOCH
AE	70831	ON	MOCH	08-AB-039	MOCH	2007	RMB	7:43 AM	RBNU
AE	70831	ON	MOCH	08-AB-039	MOCH	2007	RMB	7:43 AM	RBNU
AE	70831	ON	MOCH	08-AB-039	MOCH	2007	RMB	7:43 AM	MOCH
AE	70831	ON	MOCH	08-AB-039	MOCH	2007	RMB	7:43 AM	BCCH
AE	70831	ON	MOCH	08-AB-039	MOCH	2007	RMB	7:43 AM	BCCH
AE	70831	ON	MOCH	08-AB-039	MOCH	2007	RMB	7:43 AM	MOCH
AE	70831	ON	MOCH	08-AB-039	MOCH	2007	RMB	7:43 AM	BCCH
AE	70831	ON	MOCH	08-AB-039	MOCH	2007	RMB	7:43 AM	MOCH
AE	70831	ON	MOCH	08-AB-039	MOCH	2007	RMB	7:43 AM	RBNU
AE	70831	ON	MOCH	08-AB-039	MOCH	2007	RMB	7:43 AM	RBNU
AE	70831	ON	MOCH	08-AB-039	MOCH	2007	RMB	7:43 AM	MOCH
AE	70831	ON	MOCH	08-AB-039	MOCH	2007	RMB	7:43 AM	MOCH
AE	70831	ON	MOCH	08-AB-039	MOCH	2007	RMB	7:43 AM	MOCH
AE	70831	ON	MOCH	08-AB-039	MOCH	2007	RMB	7:43 AM	MOCH
AE	70831	ON	MOCH	08-AB-039	MOCH	2007	RMB	7:43 AM	BCCH
AE	70831	ON	MOCH	08-AB-039	MOCH	2007	RMB	7:43 AM	RBNU
AE	70831	ON	MOCH	08-AB-039	MOCH	2007	RMB	7:43 AM	RBNU
AE	70831	ON	MOCH	08-AB-039	MOCH	2007	RMB	7:43 AM	RBNU
AE	70831	ON	MOCH	08-AB-039	MOCH	2007	RMB	7:43 AM	MOCH
AE	70831	ON	MOCH	08-AB-039	MOCH	2007	RMB	7:43 AM	BCCH
AE	70133	ON	MOCH	08-ARN-04	MOCH	2007	RBM	10:50 AM	BCCH
AE	70133	ON	MOCH	08-ARN-04	MOCH	2007	RBM	10:50 AM	MOCH
AE	70133	ON	MOCH	08-ARN-04	MOCH	2007	RBM	10:50 AM	MOCH

AE	70133	ON	MOCH	08-ARN-04	MOCH	2007	RBM	10:50 AM	MOCH
AE	70133	ON	MOCH	08-ARN-04	MOCH	2007	RBM	10:50 AM	MOCH
AE	70133	ON	MOCH	08-ARN-04	MOCH	2007	RBM	10:50 AM	MOCH
AE	70133	ON	MOCH	08-ARN-04	MOCH	2007	RBM	10:50 AM	MOCH
AE	70133	ON	MOCH	08-ARN-04	MOCH	2007	RBM	10:50 AM	MOCH
AE	70133	ON	MOCH	08-ARN-04	MOCH	2007	RBM	10:50 AM	MOCH
AE	70133	ON	MOCH	08-ARN-04	MOCH	2007	RBM	10:50 AM	MOCH
AE	70133	ON	MOCH	08-ARN-04	MOCH	2007	RBM	10:50 AM	MOCH
AE	70133	ON	MOCH	08-ARN-04	MOCH	2007	RBM	10:50 AM	RBNU
AE	70133	ON	MOCH	08-ARN-04	MOCH	2007	RBM	10:50 AM	BCCH
AE	70133	ON	MOCH	08-ARN-04	MOCH	2007	RBM	10:50 AM	BCCH
AE	70133	ON	MOCH	08-ARN-04	MOCH	2007	RBM	10:50 AM	BCCH
AE	70133	ON	MOCH	08-ARN-04	MOCH	2007	RBM	10:50 AM	BCCH
AE	70133	ON	MOCH	08-ARN-04	MOCH	2007	RBM	10:50 AM	BCCH
AE	70133	ON	MOCH	08-ARN-04	MOCH	2007	RBM	10:50 AM	MOCH
AE	70133	ON	MOCH	08-ARN-04	MOCH	2007	RBM	10:50 AM	BCCH
AE	70133	ON	MOCH	08-ARN-04	MOCH	2007	RBM	10:50 AM	RBNU
AE	70133	ON	MOCH	08-ARN-04	MOCH	2007	RBM	10:50 AM	MOCH
AE	70133	ON	MOCH	08-ARN-04	MOCH	2007	RBM	10:50 AM	MOCH
AE	70133	ON	MOCH	08-ARN-04	MOCH	2007	RBM	10:50 AM	BCCH
AE	70133	ON	MOCH	08-ARN-04	MOCH	2007	RBM	10:50 AM	MOCH
AE	70133	ON	MOCH	08-ARN-04	MOCH	2007	RBM	10:50 AM	RBNU
AE	70133	ON	MOCH	08-ARN-04	MOCH	2007	RBM	10:50 AM	RBNU
AE	70133	ON	MOCH	08-ARN-04	MOCH	2007	RBM	10:50 AM	RBNU
AE	70133	ON	MOCH	08-ARN-04	MOCH	2007	RBM	10:50 AM	RBNU
AE	70133	ON	MOCH	08-ARN-04	MOCH	2007	RBM	10:50 AM	RBNU
AE	70133	ON	MOCH	08-ARN-04	MOCH	2007	RBM	10:50 AM	RBNU
AE	70133	ON	MOCH	08-ARN-04	MOCH	2007	RBM	10:50 AM	BCCH
AE	70133	ON	MOCH	08-ARN-04	MOCH	2007	RBM	10:50 AM	BCCH
AE	70133	ON	MOCH	08-ARN-04	MOCH	2007	RBM	10:50 AM	BCCH
AE	70133	ON	MOCH	08-ARN-04	MOCH	2007	RBM	10:50 AM	BCCH
AE	70133	ON	MOCH	08-ARN-04	MOCH	2007	RBM	10:50 AM	MOCH
AE	70133	ON	MOCH	08-ARN-04	MOCH	2007	RBM	10:50 AM	RBNU
AE	70133	ON	MOCH	08-ARN-04	MOCH	2007	RBM	10:50 AM	BCCH
AE	70133	ON	MOCH	08-ARN-04	MOCH	2007	RBM	10:50 AM	MOCH
IB	7860	ON	MOCH	07-TD-006	MOCH	2006	RBM	8:30 AM	BCCH
IB	7860	ON	MOCH	07-TD-006	MOCH	2006	RBM	8:30 AM	RBNU
IB	7860	ON	MOCH	07-TD-006	MOCH	2006	RBM	8:30 AM	MOCH
IB	7860	ON	MOCH	07-TD-006	MOCH	2006	RBM	8:30 AM	RBNU
IB	7860	ON	MOCH	07-TD-006	MOCH	2006	RBM	8:30 AM	RBNU
IB	7860	ON	MOCH	07-TD-006	MOCH	2006	RBM	8:30 AM	RBNU
IB	7860	ON	MOCH	07-TD-006	MOCH	2006	RBM	8:30 AM	RBNU
IB	7860	ON	MOCH	07-TD-006	MOCH	2006	RBM	8:30 AM	RBNU
IB	7860	ON	MOCH	07-TD-006	MOCH	2006	RBM	8:30 AM	RBNU

IB	7860	ON	MOCH	07-TD-006	MOCH	2006	RBM	8:30 AM	RBNU
IB	7860	ON	MOCH	07-TD-006	MOCH	2006	RBM	8:30 AM	RBNU
IB	7860	ON	MOCH	07-TD-006	MOCH	2006	RBM	8:30 AM	RBNU
IB	7860	ON	MOCH	07-TD-006	MOCH	2006	RBM	8:30 AM	BCCH
IB	7860	ON	MOCH	07-TD-006	MOCH	2006	RBM	8:30 AM	BCCH
IB	7860	ON	MOCH	07-TD-006	MOCH	2006	RBM	8:30 AM	MOCH
IB	7860	ON	MOCH	07-TD-006	MOCH	2006	RBM	8:30 AM	MOCH
IB	7860	ON	MOCH	07-TD-006	MOCH	2006	RBM	8:30 AM	RBNU
IB	7860	ON	MOCH	07-TD-006	MOCH	2006	RBM	8:30 AM	BCCH
IB	7860	ON	MOCH	07-TD-006	MOCH	2006	RBM	8:30 AM	MOCH
IB	7860	ON	MOCH	07-TD-006	MOCH	2006	RBM	8:30 AM	RBNU
IB	7860	ON	MOCH	07-TD-006	MOCH	2006	RBM	8:30 AM	RBNU
	7974	ON	RBNU	04-ARN-05	RBNU	2003	R	12:24:38	RBNU
	7974	ON	RBNU	04-ARN-05	RBNU	2003	R	12:24:38	RBNU
	7974	ON	RBNU	04-ARN-05	RBNU	2003	R	12:24:38	RBNU
	7974	ON	RBNU	04-ARN-05	RBNU	2003	R	12:24:38	RBNU
	7974	ON	RBNU	04-ARN-05	RBNU	2003	R	12:24:38	RBNU
	7974	ON	RBNU	04-ARN-05	RBNU	2003	R	12:24:38	RBNU
	7974	ON	RBNU	04-ARN-05	RBNU	2003	R	12:24:38	RBNU
	7974	ON	RBNU	04-ARN-05	RBNU	2003	R	12:24:38	RBNU
	7974	ON	RBNU	04-ARN-05	RBNU	2003	R	12:24:38	RBNU
	7974	ON	RBNU	04-ARN-05	RBNU	2003	R	12:24:38	RBNU
	7974	ON	RBNU	04-ARN-05	RBNU	2003	R	12:24:38	RBNU
	7974	ON	RBNU	04-ARN-05	RBNU	2003	R	12:24:38	RBNU
	7974	ON	RBNU	04-ARN-05	RBNU	2003	R	12:24:38	RBNU
	7974	ON	RBNU	04-ARN-05	RBNU	2003	R	12:24:38	RBNU
	7974	ON	RBNU	04-ARN-05	RBNU	2003	R	12:24:38	RBNU
	7974	ON	RBNU	04-ARN-05	RBNU	2003	R	12:24:38	RBNU
	7974	ON	RBNU	04-ARN-05	RBNU	2003	R	12:24:38	RBNU
	7983	ON	RBNU	04-DG-055	RBNU	2002	RMB	11:12	BCCH
	7983	ON	RBNU	04-DG-055	RBNU	2002	RMB	11:12	RBNU
	7983	ON	RBNU	04-DG-055	RBNU	2002	RMB	11:12	RBNU
	7983	ON	RBNU	04-DG-055	RBNU	2002	RMB	11:12	RBNU
	7983	ON	RBNU	04-DG-055	RBNU	2002	RMB	11:12	RBNU
	7983	ON	RBNU	04-DG-055	RBNU	2002	RMB	11:12	RBNU
	7983	ON	RBNU	04-DG-055	RBNU	2002	RMB	11:12	RBNU
	7983	ON	RBNU	04-DG-055	RBNU	2002	RMB	11:12	RBNU
	7983	ON	RBNU	04-DG-055	RBNU	2002	RMB	11:12	RBNU
	7983	ON	RBNU	04-DG-055	RBNU	2002	RMB	11:12	BCCH
	7983	ON	RBNU	04-DG-055	RBNU	2002	RMB	11:12	MOCH
	7983	ON	RBNU	04-DG-055	RBNU	2002	RMB	11:12	BCCH
	7983	ON	RBNU	04-DG-055	RBNU	2002	RMB	11:12	MOCH
	7983	ON	RBNU	04-DG-055	RBNU	2002	RMB	11:12	MOCH
	7983	ON	RBNU	04-DG-055	RBNU	2002	RMB	11:12	MOCH

7983	ON	RBNU	04-DG-055	RBNU	2002	RMB	11:12	BCCH
7040	ON	RBNU		MOCH	2002	BRM	9:20	MOCH
7040	ON	RBNU		MOCH	2002	BRM	9:20	none
7040	ON	RBNU		MOCH	2002	BRM	9:20	none
7040	ON	RBNU		MOCH	2002	BRM	9:20	none
7040	ON	RBNU		MOCH	2002	BRM	9:20	none
7040	ON	RBNU		MOCH	2002	BRM	9:20	none
7040	ON	RBNU		MOCH	2002	BRM	9:20	RBNU
7040	ON	RBNU		MOCH	2002	BRM	9:20	RBNU
7040	ON	RBNU		MOCH	2002	BRM	9:20	MOCH
7040	ON	RBNU		MOCH	2002	BRM	9:20	MOCH
7040	ON	RBNU		MOCH	2002	BRM	9:20	MOCH
7040	ON	RBNU		MOCH	2002	BRM	9:20	RBNU
7775	ON	RBNU	04-NK-044	MOCH	2003	MBR	10:25	MOCH
7775	ON	RBNU	04-NK-044	MOCH	2003	MBR	10:25	BCCH
7974	ON	RBNU	04-ARN-05	RBNU	2003	MRB	8:00	BCCH
7974	ON	RBNU	04-ARN-05	RBNU	2003	MRB	8:00	BCCH
7974	ON	RBNU	04-ARN-05	RBNU	2003	MRB	8:00	BCCH
7974	ON	RBNU	04-ARN-05	RBNU	2003	MRB	8:00	BCCH
7974	ON	RBNU	04-ARN-05	RBNU	2003	MRB	8:00	BCCH
7974	ON	RBNU	04-ARN-05	RBNU	2003	MRB	8:00	BCCH
7974	ON	RBNU	04-ARN-05	RBNU	2003	MRB	8:00	RBNU
7974	ON	RBNU	04-ARN-05	RBNU	2003	MRB	8:00	RBNU
7974	ON	RBNU	04-ARN-05	RBNU	2003	MRB	8:00	RBNU
7974	ON	RBNU	04-ARN-05	RBNU	2003	MRB	8:00	RBNU
7974	ON	RBNU	04-ARN-05	RBNU	2003	MRB	8:00	RBNU
7974	ON	RBNU	04-ARN-05	RBNU	2003	MRB	8:00	RBNU
7974	ON	RBNU	04-ARN-05	RBNU	2003	MRB	8:00	BCCH
7974	ON	RBNU	04-ARN-05	RBNU	2003	MRB	8:00	BCCH
7974	ON	RBNU	04-ARN-05	RBNU	2003	MRB	8:00	BCCH
7974	ON	RBNU	04-ARN-05	RBNU	2003	MRB	8:00	BCCH
7974	ON	RBNU	04-ARN-05	RBNU	2003	MRB	8:00	BCCH
7974	ON	RBNU	04-ARN-05	RBNU	2003	MRB	8:00	RBNU
7974	ON	RBNU	04-ARN-05	RBNU	2003	MRB	8:00	RBNU
7974	ON	RBNU	04-ARN-05	RBNU	2003	MRB	8:00	RBNU
7974	ON	RBNU	04-ARN-05	RBNU	2003	MRB	8:00	BCCH
7974	ON	RBNU	04-ARN-05	RBNU	2003	MRB	8:00	BCCH
7974	ON	RBNU	04-ARN-05	RBNU	2003	MRB	8:00	RBNU
7974	ON	RBNU	04-ARN-05	RBNU	2003	MRB	8:00	BCCH
7974	ON	RBNU	04-ARN-05	RBNU	2003	MRB	8:00	RBNU
7974	ON	RBNU	04-ARN-05	RBNU	2003	MRB	8:00	BCCH
7974	ON	RBNU	04-ARN-05	RBNU	2003	MRB	8:00	RBNU
7974	ON	RBNU	04-ARN-05	RBNU	2003	MRB	8:00	RBNU
7974	ON	RBNU	04-ARN-05	RBNU	2003	MRB	8:00	MOCH

7137	ON	RBNU		MOCH	2003	BMR	8:00	BCCH
7137	ON	RBNU		MOCH	2003	BMR	8:00	BCCH
7137	ON	RBNU		MOCH	2003	BMR	8:00	BCCH
7137	ON	RBNU		MOCH	2003	BMR	8:00	BCCH
7137	ON	RBNU		MOCH	2003	BMR	8:00	BCCH
7137	ON	RBNU		MOCH	2003	BMR	8:00	BCCH
7137	ON	RBNU		MOCH	2003	BMR	8:00	BCCH
7137	ON	RBNU		MOCH	2003	BMR	8:00	BCCH
7137	ON	RBNU		MOCH	2003	BMR	8:00	BCCH
7137	ON	RBNU		MOCH	2003	BMR	8:00	BCCH
7137	ON	RBNU		MOCH	2003	BMR	8:00	BCCH
7137	ON	RBNU		MOCH	2003	BMR	8:00	BCCH
7137	ON	RBNU		MOCH	2003	BMR	8:00	RBNU
7137	ON	RBNU		MOCH	2003	BMR	8:00	BCCH
7137	ON	RBNU		MOCH	2003	BMR	8:00	BCCH
7137	ON	RBNU		MOCH	2003	BMR	8:00	RBNU
7137	ON	RBNU		MOCH	2003	BMR	8:00	RBNU
7137	ON	RBNU		MOCH	2003	BMR	8:00	BCCH
7137	ON	RBNU		MOCH	2003	BMR	8:00	MOCH
7137	ON	RBNU		MOCH	2003	BMR	8:00	RBNU
7137	ON	RBNU		MOCH	2003	BMR	8:00	BCCH
7137	ON	RBNU		MOCH	2003	BMR	8:00	RBNU
7512	ON	RBNU	04-PAR-06	MOCH	2004	MBR	9:30	none
7512	ON	RBNU	04-PAR-06	MOCH	2004	MBR	9:30	MOCH
7512	ON	RBNU	04-PAR-06	MOCH	2004	MBR	9:30	none
7512	ON	RBNU	04-PAR-06	MOCH	2004	MBR	9:30	BCCH
7512	ON	RBNU	04-PAR-06	MOCH	2004	MBR	9:30	MOCH
7512	ON	RBNU	04-PAR-06	MOCH	2004	MBR	9:30	RBNU
7512	ON	RBNU	04-PAR-06	MOCH	2004	MBR	9:30	RBNU
7040	ON	RBNU	04-ARN-03	MOCH	2002	MBR	8:40	RBNU
7040	ON	RBNU	04-ARN-03	MOCH	2002	MBR	8:40	RBNU
7040	ON	RBNU	04-ARN-03	MOCH	2002	MBR	8:40	RBNU
7040	ON	RBNU	04-ARN-03	MOCH	2002	MBR	8:40	MOCH
7040	ON	RBNU	04-ARN-03	MOCH	2002	MBR	8:40	BCCH
7040	ON	RBNU	04-ARN-03	MOCH	2002	MBR	8:40	BCCH
7040	ON	RBNU	04-ARN-03	MOCH	2002	MBR	8:40	BCCH
7040	ON	RBNU	04-ARN-03	MOCH	2002	MBR	8:40	BCCH
7040	ON	RBNU	04-ARN-03	MOCH	2002	MBR	8:40	RBNU
7040	ON	RBNU	04-ARN-03	MOCH	2002	MBR	8:40	RBNU
7040	ON	RBNU	04-ARN-03	MOCH	2002	MBR	8:40	RBNU
7040	ON	RBNU	04-ARN-03	MOCH	2002	MBR	8:40	BCCH
7040	ON	RBNU	04-ARN-03	MOCH	2002	MBR	8:40	RBNU
7040	ON	RBNU	04-ARN-03	MOCH	2002	MBR	8:40	RBNU
7983	ON	RBNU	04-DG-055	RBNU	2002	BRM	9:19	MOCH

7983	ON	RBNU	04-DG-055	RBNU	2002	BRM	9:19	BCCH
7983	ON	RBNU	04-DG-055	RBNU	2002	BRM	9:19	RBNU
7983	ON	RBNU	04-DG-055	RBNU	2002	BRM	9:19	RBNU
7983	ON	RBNU	04-DG-055	RBNU	2002	BRM	9:19	BCCH
7983	ON	RBNU	04-DG-055	RBNU	2002	BRM	9:19	BCCH
7983	ON	RBNU	04-DG-055	RBNU	2002	BRM	9:19	RBNU
7983	ON	RBNU	04-DG-055	RBNU	2002	BRM	9:19	RBNU
7983	ON	RBNU	04-DG-055	RBNU	2002	BRM	9:19	RBNU
7983	ON	RBNU	04-DG-055	RBNU	2002	BRM	9:19	RBNU
7983	ON	RBNU	04-DG-055	RBNU	2002	BRM	9:19	MOCH
7983	ON	RBNU	04-DG-055	RBNU	2002	BRM	9:19	RBNU
7983	ON	RBNU	04-DG-055	RBNU	2002	BRM	9:19	BCCH
7983	ON	RBNU	04-DG-055	RBNU	2002	BRM	9:19	BCCH
7983	ON	RBNU	04-DG-055	RBNU	2002	BRM	9:19	RBNU
70101	ON	RBNU	04-PAR-07	RBNU	2003	RBM	7:36	BCCH
70101	ON	RBNU	04-PAR-07	RBNU	2003	RBM	7:36	BCCH
70101	ON	RBNU	04-PAR-07	RBNU	2003	RBM	7:36	none
70101	ON	RBNU	04-PAR-07	RBNU	2003	RBM	7:36	none
70101	ON	RBNU	04-PAR-07	RBNU	2003	RBM	7:36	BCCH
70101	ON	RBNU	04-PAR-07	RBNU	2003	RBM	7:36	RBNU
70101	ON	RBNU	04-PAR-07	RBNU	2003	RBM	7:36	BCCH
70101	ON	RBNU	04-PAR-07	RBNU	2003	RBM	7:36	BCCH
70101	ON	RBNU	04-PAR-07	RBNU	2003	RBM	7:36	none
70101	ON	RBNU	04-PAR-07	RBNU	2003	RBM	7:36	none
70101	ON	RBNU	04-PAR-07	RBNU	2003	RBM	7:36	none
70101	ON	RBNU	04-PAR-07	RBNU	2003	RBM	7:36	RBNU
70101	ON	RBNU	04-PAR-07	RBNU	2003	RBM	7:36	RBNU
70101	ON	RBNU	04-PAR-07	RBNU	2003	RBM	7:36	RBNU
70101	ON	RBNU	04-PAR-07	RBNU	2003	RBM	7:36	RBNU
70101	ON	RBNU	04-PAR-07	RBNU	2003	RBM	7:36	BCCH
70101	ON	RBNU	04-PAR-07	RBNU	2003	RBM	7:36	BCCH
70101	ON	RBNU	04-PAR-07	RBNU	2003	RBM	7:36	BCCH
70101	ON	RBNU	04-PAR-07	RBNU	2003	RBM	7:36	BCCH
70101	ON	RBNU	04-PAR-07	RBNU	2003	RBM	7:36	BCCH
70101	ON	RBNU	04-PAR-07	RBNU	2003	RBM	7:36	BCCH
70101	ON	RBNU	04-PAR-07	RBNU	2003	RBM	7:36	BCCH
70101	ON	RBNU	04-PAR-07	RBNU	2003	RBM	7:36	none
70101	ON	RBNU	04-PAR-07	RBNU	2003	RBM	7:36	BCCH
70101	ON	RBNU	04-PAR-07	RBNU	2003	RBM	7:36	BCCH
7974	ON	RBNU	04-ARN-05	RBNU	2003	MRB	9:46:30	BCCH
7974	ON	RBNU	04-ARN-05	RBNU	2003	MRB	9:46:30	BCCH
7974	ON	RBNU	04-ARN-05	RBNU	2003	MRB	9:46:30	BCCH
7974	ON	RBNU	04-ARN-05	RBNU	2003	MRB	9:46:30	BCCH
7974	ON	RBNU	04-ARN-05	RBNU	2003	MRB	9:46:30	none

	7974	ON	RBNU	04-ARN-05	RBNU	2003	MRB	9:46:30	none
	7974	ON	RBNU	04-ARN-05	RBNU	2003	MRB	9:46:30	none
	7974	ON	RBNU	04-ARN-05	RBNU	2003	MRB	9:46:30	none
	7974	ON	RBNU	04-ARN-05	RBNU	2003	MRB	9:46:30	MOCH
	7974	ON	RBNU	04-ARN-05	RBNU	2003	MRB	9:46:30	MOCH
	7974	ON	RBNU	04-ARN-05	RBNU	2003	MRB	9:46:30	MOCH
	7974	ON	RBNU	04-ARN-05	RBNU	2003	MRB	9:46:30	MOCH
	7974	ON	RBNU	04-ARN-05	RBNU	2003	MRB	9:46:30	RBNU
	7974	ON	RBNU	04-ARN-05	RBNU	2003	MRB	9:46:30	RBNU
	7974	ON	RBNU	04-ARN-05	RBNU	2003	MRB	9:46:30	BCCH
	7974	ON	RBNU	04-ARN-05	RBNU	2003	MRB	9:46:30	BCCH
	7974	ON	RBNU	04-ARN-05	RBNU	2003	MRB	9:46:30	RBNU
	7974	ON	RBNU	04-ARN-05	RBNU	2003	MRB	9:46:30	BCCH
	7974	ON	RBNU	04-ARN-05	RBNU	2003	MRB	9:46:30	RBNU
	7974	ON	RBNU	04-ARN-05	RBNU	2003	MRB	9:46:30	RBNU
	7974	ON	RBNU	04-ARN-05	RBNU	2003	MRB	9:46:30	none
	7512	ON	RBNU	04-PAR-06	MOCH	2003	BRM	7:11	none
	7512	ON	RBNU	04-PAR-06	MOCH	2003	BRM	7:11	RBNU
	7512	ON	RBNU	04-PAR-06	MOCH	2003	BRM	7:11	RBNU
	7512	ON	RBNU	04-PAR-06	MOCH	2003	BRM	7:11	RBNU
	7512	ON	RBNU	04-PAR-06	MOCH	2003	BRM	7:11	MOCH
	7512	ON	RBNU	04-PAR-06	MOCH	2003	BRM	7:11	BCCH
	7512	ON	RBNU	04-PAR-06	MOCH	2003	BRM	7:11	RBNU
	7512	ON	RBNU	04-PAR-06	MOCH	2003	BRM	7:11	RBNU
	7512	ON	RBNU	04-PAR-06	MOCH	2003	BRM	7:11	RBNU
	7512	ON	RBNU	04-PAR-06	MOCH	2003	BRM	7:11	RBNU
	7512	ON	RBNU	04-PAR-06	MOCH	2003	BRM	7:11	RBNU
	7512	ON	RBNU	04-PAR-06	MOCH	2003	BRM	7:11	none
	7512	ON	RBNU	04-PAR-06	MOCH	2003	BRM	7:11	MOCH
ARN	7545	ON	RBNU	05-ARN-03	RBNU	2003	RBM	8:23	BCCH
ARN	7545	ON	RBNU	05-ARN-03	RBNU	2003	RBM	8:23	BCCH
ARN	7545	ON	RBNU	05-ARN-03	RBNU	2003	RBM	8:23	MOCH
ARN	7545	ON	RBNU	05-ARN-03	RBNU	2003	RBM	8:23	MOCH
ARN	7545	ON	RBNU	05-ARN-03	RBNU	2003	RBM	8:23	MOCH
ARN	7545	ON	RBNU	05-ARN-03	RBNU	2003	RBM	8:23	none
ARN	7545	ON	RBNU	05-ARN-03	RBNU	2003	RBM	8:23	BCCH
ARN	7545	ON	RBNU	05-ARN-03	RBNU	2003	RBM	8:23	RBNU
ARN	7545	ON	RBNU	05-ARN-03	RBNU	2003	RBM	8:23	MOCH
ARN	7545	ON	RBNU	05-ARN-03	RBNU	2003	RBM	8:23	RBNU
ARN	7545	ON	RBNU	05-ARN-03	RBNU	2003	RBM	8:23	MOCH
ARN	7545	ON	RBNU	05-ARN-03	RBNU	2003	RBM	8:23	none
ARN	7545	ON	RBNU	05-ARN-03	RBNU	2003	RBM	8:23	RBNU
ARN	7545	ON	RBNU	05-ARN-03	RBNU	2003	RBM	8:23	BCCH
ARN	7545	ON	RBNU	05-ARN-03	RBNU	2003	RBM	8:23	RBNU

70702	ON	RBNU	05-PAR-02	RBNU	2004	RMB	14:33	MOCH
70702	ON	RBNU	05-PAR-02	RBNU	2004	RMB	14:33	BCCH
7255	ON	RBNU	05-PAR-02	RBNU	2004	RMB	14:33	MOCH
7255	ON	RBNU	05-PAR-02	RBNU	2004	RMB	14:33	BCCH
70702	ON	RBNU	05-PAR-02	RBNU	2004	RMB	14:33	MOCH
7255	ON	RBNU	05-PAR-02	RBNU	2004	RMB	14:33	MOCH
70702	ON	RBNU	05-PAR-02	RBNU	2004	RMB	14:33	BCCH
70702	ON	RBNU	05-PAR-02	RBNU	2004	RMB	14:33	BCCH
7255	ON	RBNU	05-PAR-02	RBNU	2004	RMB	14:33	BCCH
7255	ON	RBNU	05-PAR-02	RBNU	2004	RMB	14:33	BCCH
70702	ON	RBNU	05-PAR-02	RBNU	2004	RMB	14:33	MOCH
7255	ON	RBNU	05-PAR-02	RBNU	2004	RMB	14:33	MOCH
70702	ON	RBNU	05-PAR-02	RBNU	2004	RMB	14:33	RBNU
7255	ON	RBNU	05-PAR-02	RBNU	2004	RMB	14:33	RBNU
70702	ON	RBNU	05-PAR-02	RBNU	2004	RMB	14:33	RBNU
70702	ON	RBNU	05-PAR-02	RBNU	2004	RMB	14:33	BCCH
7255	ON	RBNU	05-PAR-02	RBNU	2004	RMB	14:33	RBNU
7255	ON	RBNU	05-PAR-02	RBNU	2004	RMB	14:33	BCCH
70702	ON	RBNU	05-PAR-02	RBNU	2004	RMB	14:33	none
70702	ON	RBNU	05-PAR-02	RBNU	2004	RMB	14:33	MOCH
70702	ON	RBNU	05-PAR-02	RBNU	2004	RMB	14:33	BCCH
7255	ON	RBNU	05-PAR-02	RBNU	2004	RMB	14:33	none
7255	ON	RBNU	05-PAR-02	RBNU	2004	RMB	14:33	MOCH
7255	ON	RBNU	05-PAR-02	RBNU	2004	RMB	14:33	BCCH
70702	ON	RBNU	05-PAR-02	RBNU	2004	RMB	14:33	RBNU
70702	ON	RBNU	05-PAR-02	RBNU	2004	RMB	14:33	MOCH
70702	ON	RBNU	05-PAR-02	RBNU	2004	RMB	14:33	MOCH
70702	ON	RBNU	05-PAR-02	RBNU	2004	RMB	14:33	BCCH
7255	ON	RBNU	05-PAR-02	RBNU	2004	RMB	14:33	RBNU
7255	ON	RBNU	05-PAR-02	RBNU	2004	RMB	14:33	MOCH
7255	ON	RBNU	05-PAR-02	RBNU	2004	RMB	14:33	MOCH
7255	ON	RBNU	05-PAR-02	RBNU	2004	RMB	14:33	BCCH
70702	ON	RBNU	05-PAR-02	RBNU	2004	RMB	14:33	RBNU
70702	ON	RBNU	05-PAR-02	RBNU	2004	RMB	14:33	MOCH
70702	ON	RBNU	05-PAR-02	RBNU	2004	RMB	14:33	BCCH
70702	ON	RBNU	05-PAR-02	RBNU	2004	RMB	14:33	BCCH
7255	ON	RBNU	05-PAR-02	RBNU	2004	RMB	14:33	RBNU
7255	ON	RBNU	05-PAR-02	RBNU	2004	RMB	14:33	MOCH
7255	ON	RBNU	05-PAR-02	RBNU	2004	RMB	14:33	BCCH
7255	ON	RBNU	05-PAR-02	RBNU	2004	RMB	14:33	BCCH
70702	ON	RBNU	05-PAR-02	RBNU	2004	RMB	14:33	RBNU
7255	ON	RBNU	05-PAR-02	RBNU	2004	RMB	14:33	RBNU
70702	ON	RBNU	05-PAR-02	RBNU	2004	RMB	14:33	MOCH
7255	ON	RBNU	05-PAR-02	RBNU	2004	RMB	14:33	MOCH

7545	ON	RBNU	05-ARN-03	RBNU	2004	RMB	7:48	none
7545	ON	RBNU	05-ARN-03	RBNU	2004	RMB	7:48	none
7545	ON	RBNU	05-ARN-03	RBNU	2004	RMB	7:48	none
7545	ON	RBNU	05-ARN-03	RBNU	2004	RMB	7:48	none
7545	ON	RBNU	05-ARN-03	RBNU	2004	RMB	7:48	none
7545	ON	RBNU	05-ARN-03	RBNU	2004	RMB	7:48	none
7545	ON	RBNU	05-ARN-03	RBNU	2004	RMB	7:48	none
7545	ON	RBNU	05-ARN-03	RBNU	2004	RMB	7:48	none
7545	ON	RBNU	05-ARN-03	RBNU	2004	RMB	7:48	none
7545	ON	RBNU	05-ARN-03	RBNU	2004	RMB	7:48	none
7545	ON	RBNU	05-ARN-03	RBNU	2004	RMB	7:48	none
7545	ON	RBNU	05-ARN-03	RBNU	2004	RMB	7:48	none
7545	ON	RBNU	05-ARN-03	RBNU	2004	RMB	7:48	none
7545	ON	RBNU	05-ARN-03	RBNU	2004	RMB	7:48	none
7545	ON	RBNU	05-ARN-03	RBNU	2004	RMB	7:48	none
7545	ON	RBNU	05-ARN-03	RBNU	2004	RMB	7:48	none
7545	ON	RBNU	05-ARN-03	RBNU	2004	RMB	7:48	none
7545	ON	RBNU	05-ARN-03	RBNU	2004	RMB	7:48	none
7545	ON	RBNU	05-ARN-03	RBNU	2004	RMB	7:48	none
7545	ON	RBNU	05-ARN-03	RBNU	2004	RMB	7:48	none
7545	ON	RBNU	05-ARN-03	RBNU	2004	RMB	7:48	none
7545	ON	RBNU	05-ARN-03	RBNU	2004	RMB	7:48	none
7545	ON	RBNU	05-ARN-03	RBNU	2004	RMB	7:48	none
7579	ON	RBNU	05-MN-018	RBNU	2004	RBM	8:46	BCCH
7579	ON	RBNU	05-MN-018	RBNU	2004	RBM	8:46	MOCH
7579	ON	RBNU	05-MN-018	RBNU	2004	RBM	8:46	MOCH
7579	ON	RBNU	05-MN-018	RBNU	2004	RBM	8:46	RBNU
7579	ON	RBNU	05-MN-018	RBNU	2004	RBM	8:46	RBNU
7579	ON	RBNU	05-MN-018	RBNU	2004	RBM	8:46	RBNU
7579	ON	RBNU	05-MN-018	RBNU	2004	RBM	8:46	RBNU
7579	ON	RBNU	05-MN-018	RBNU	2004	RBM	8:46	RBNU
7579	ON	RBNU	05-MN-018	RBNU	2004	RBM	8:46	BCCH
7579	ON	RBNU	05-MN-018	RBNU	2004	RBM	8:46	MOCH
7579	ON	RBNU	05-MN-018	RBNU	2004	RBM	8:46	RBNU
7579	ON	RBNU	05-MN-018	RBNU	2004	RBM	8:46	BCCH
7579	ON	RBNU	05-MN-018	RBNU	2004	RBM	8:46	MOCH
7579	ON	RBNU	05-MN-018	RBNU	2004	RBM	8:46	MOCH
7894	ON	RBNU	05-CL-030	RBNU	2004	MRB	11:44	BCCH
7894	ON	RBNU	05-CL-030	RBNU	2004	MRB	11:44	BCCH
7894	ON	RBNU	05-CL-030	RBNU	2004	MRB	11:44	RBNU
7894	ON	RBNU	05-CL-030	RBNU	2004	MRB	11:44	BCCH
7894	ON	RBNU	05-CL-030	RBNU	2004	MRB	11:44	BCCH
7894	ON	RBNU	05-CL-030	RBNU	2004	MRB	11:44	none
7894	ON	RBNU	05-CL-030	RBNU	2004	MRB	12:13	RBNU

7894	ON	RBNU	05-CL-030	RBNU	2004	MRB	12:13	RBNU
7894	ON	RBNU	05-CL-030	RBNU	2004	MRB	12:13	BCCH
7815	ON	RBNU	05-ARN-03	RBNU	2004	MRB	12:20	MOCH
7815	ON	RBNU	05-ARN-03	RBNU	2004	MRB	12:20	MOCH
7815	ON	RBNU	05-ARN-03	RBNU	2004	MRB	12:20	RBNU
7815	ON	RBNU	05-ARN-03	RBNU	2004	MRB	12:20	BCCH
7815	ON	RBNU	05-ARN-03	RBNU	2004	MRB	12:20	MOCH
7815	ON	RBNU	05-ARN-03	RBNU	2004	MRB	12:20	BCCH
7815	ON	RBNU	05-ARN-03	RBNU	2004	MRB	12:20	RBNU
7815	ON	RBNU	05-ARN-03	RBNU	2004	MRB	12:20	RBNU
7815	ON	RBNU	05-ARN-03	RBNU	2004	MRB	12:20	BCCH
7374	ON	RBNU	05-CW-035	RBNU	2004	BMR	15:43	MOCH
7374	ON	RBNU	05-CW-035	RBNU	2004	BMR	15:43	MOCH
7374	ON	RBNU	05-CW-035	RBNU	2004	BMR	15:43	none
7374	ON	RBNU	05-CW-035	RBNU	2004	BMR	15:43	MOCH
7374	ON	RBNU	05-CW-035	RBNU	2004	BMR	15:43	RBNU
7374	ON	RBNU	05-CW-035	RBNU	2004	BMR	15:43	RBNU
7374	ON	RBNU	05-CW-035	RBNU	2004	BMR	15:43	BCCH
7374	ON	RBNU	05-CW-035	RBNU	2004	BMR	15:43	RBNU
7374	ON	RBNU	05-CW-035	RBNU	2004	BMR	15:43	RBNU
7374	ON	RBNU	05-CW-035	RBNU	2004	BMR	15:43	RBNU
7374	ON	RBNU	05-CW-035	RBNU	2004	BMR	15:43	RBNU
7374	ON	RBNU	05-CW-035	RBNU	2004	BMR	15:43	RBNU
7374	ON	RBNU	05-CW-035	RBNU	2004	BMR	15:43	none
7374	ON	RBNU	05-CW-035	RBNU	2004	BMR	15:43	RBNU
7374	ON	RBNU	05-CW-035	RBNU	2004	BMR	15:43	none
7374	ON	RBNU	05-CW-035	RBNU	2004	BMR	15:43	MOCH
7874	ON	RBNU	05-CW-033	RBNU	2004	BMR	16:23	MOCH
7874	ON	RBNU	05-CW-033	RBNU	2004	BMR	16:23	RBNU
7874	ON	RBNU	05-CW-033	RBNU	2004	BMR	16:23	RBNU
7874	ON	RBNU	05-CW-033	RBNU	2004	BMR	16:23	BCCH
7874	ON	RBNU	05-CW-033	RBNU	2004	BMR	16:23	RBNU
7874	ON	RBNU	05-CW-033	RBNU	2004	BMR	16:23	BCCH
7874	ON	RBNU	05-CW-033	RBNU	2004	BMR	16:23	none
7545	ON	RBNU	05-ARN-03	RBNU	2004	BMR	10:44	MOCH
7545	ON	RBNU	05-ARN-03	RBNU	2004	BMR	10:44	MOCH
7545	ON	RBNU	05-ARN-03	RBNU	2004	BMR	10:44	RBNU
7545	ON	RBNU	05-ARN-03	RBNU	2004	BMR	10:44	RBNU
7545	ON	RBNU	05-ARN-03	RBNU	2004	BMR	10:44	none
7545	ON	RBNU	05-ARN-03	RBNU	2004	BMR	10:44	BCCH
7545	ON	RBNU	05-ARN-03	RBNU	2004	BMR	10:44	BCCH
7545	ON	RBNU	05-ARN-03	RBNU	2004	BMR	10:44	BCCH
7545	ON	RBNU	05-ARN-03	RBNU	2004	BMR	10:44	none
7545	ON	RBNU	05-ARN-03	RBNU	2004	BMR	10:44	none

7545	ON	RBNU	05-ARN-03	RBNU	2004	BMR	10:44	BCCH
7545	ON	RBNU	05-ARN-03	RBNU	2004	BMR	10:44	MOCH
7545	ON	RBNU	05-ARN-03	RBNU	2004	BMR	10:44	RBNU
7545	ON	RBNU	05-ARN-03	RBNU	2004	BMR	10:44	RBNU
7545	ON	RBNU	05-ARN-03	RBNU	2004	BMR	10:44	RBNU
7545	ON	RBNU	05-ARN-03	RBNU	2004	BMR	10:44	RBNU
7545	ON	RBNU	05-ARN-03	RBNU	2004	BMR	10:44	BCCH
7545	ON	RBNU	05-ARN-03	RBNU	2004	BMR	10:44	MOCH
7545	ON	RBNU	05-ARN-03	RBNU	2004	BMR	10:44	MOCH
70410	ON	RBNU	05-PAR-08	MOCH	2004	BRM	11:00	MOCH
70410	ON	RBNU	05-PAR-08	MOCH	2004	BRM	11:00	RBNU
70410	ON	RBNU	05-PAR-08	MOCH	2004	BRM	11:00	RBNU
70410	ON	RBNU	05-PAR-08	MOCH	2004	BRM	11:00	RBNU
70410	ON	RBNU	05-PAR-08	MOCH	2004	BRM	11:00	BCCH
70410	ON	RBNU	05-PAR-08	MOCH	2004	BRM	11:00	BCCH
70410	ON	RBNU	05-PAR-08	MOCH	2004	BRM	11:00	RBNU
70733	ON	RBNU	05-CL-047	RNSA	2004	RMB	9:11	BCCH
70733	ON	RBNU	05-CL-047	RNSA	2004	RMB	9:11	RBNU
70733	ON	RBNU	05-CL-047	RNSA	2004	RMB	9:11	RBNU
70733	ON	RBNU	05-CL-047	RNSA	2004	RMB	9:11	RBNU
70733	ON	RBNU	05-CL-047	RNSA	2004	RMB	9:11	MOCH
70733	ON	RBNU	05-CL-047	RNSA	2004	RMB	9:11	BCCH
70733	ON	RBNU	05-CL-047	RNSA	2004	RMB	9:11	BCCH
70733	ON	RBNU	05-CL-047	RNSA	2004	RMB	9:11	MOCH
70733	ON	RBNU	05-CL-047	RNSA	2004	RMB	9:11	BCCH
70141	ON	RBNU	05-PAR-11	RBNU	2004	BMR	8:36	MOCH
70141	ON	RBNU	05-PAR-11	RBNU	2004	BMR	8:36	MOCH
70141	ON	RBNU	05-PAR-11	RBNU	2004	BMR	8:36	MOCH
70141	ON	RBNU	05-PAR-11	RBNU	2004	BMR	8:36	MOCH
70141	ON	RBNU	05-PAR-11	RBNU	2004	BMR	8:36	MOCH
70141	ON	RBNU	05-PAR-11	RBNU	2004	BMR	8:36	RBNU
70141	ON	RBNU	05-PAR-11	RBNU	2004	BMR	8:36	RBNU
70141	ON	RBNU	05-PAR-11	RBNU	2004	BMR	8:36	none
70141	ON	RBNU	05-PAR-11	RBNU	2004	BMR	8:36	RBNU
70141	ON	RBNU	05-PAR-11	RBNU	2004	BMR	8:36	RBNU
70141	ON	RBNU	05-PAR-11	RBNU	2004	BMR	8:36	BCCH
70141	ON	RBNU	05-PAR-11	RBNU	2004	BMR	8:36	BCCH
70141	ON	RBNU	05-PAR-11	RBNU	2004	BMR	8:36	RBNU
70141	ON	RBNU	05-PAR-11	RBNU	2004	BMR	8:36	none
70141	ON	RBNU	05-PAR-11	RBNU	2004	BMR	8:36	none
70141	ON	RBNU	05-PAR-11	RBNU	2004	BMR	8:36	none
70141	ON	RBNU	05-PAR-11	RBNU	2004	BMR	8:36	BCCH
70141	ON	RBNU	05-PAR-11	RBNU	2004	BMR	8:36	RBNU
70141	ON	RBNU	05-PAR-11	RBNU	2004	BMR	8:36	none

	70141	ON	RBNU	05-PAR-11	RBNU	2004	BMR	8:36	BCCH
	70141	ON	RBNU	05-PAR-11	RBNU	2004	BMR	8:36	MOCH
	70422	ON	RBNU	06-ARN-00	RBNU	2005	BMR	10:06	none
	70422	ON	RBNU	06-ARN-00	RBNU	2005	BMR	10:13	MOCH
	70422	ON	RBNU	06-ARN-00	RBNU	2005	BMR	10:13	MOCH
	70422	ON	RBNU	06-ARN-00	RBNU	2005	BMR	10:18	RBNU
	70422	ON	RBNU	06-ARN-00	RBNU	2005	BMR	10:18	RBNU
	70422	ON	RBNU	06-ARN-00	RBNU	2005	BMR	10:18	RBNU
	70422	ON	RBNU	06-ARN-00	RBNU	2005	BMR	10:18	RBNU
	70422	ON	RBNU	06-ARN-00	RBNU	2005	BMR	10:18	RBNU
	70422	ON	RBNU	06-ARN-00	RBNU	2005	BMR	10:08:003(	BCCH
	70770	ON	RBNU	06-ARN-02	RBNU	2005	RMB	11:26	RBNU
	70770	ON	RBNU	06-ARN-02	RBNU	2005	RMB	11:26	MOCH
	70770	ON	RBNU	06-ARN-02	RBNU	2005	RMB	11:26	MOCH
	70770	ON	RBNU	06-ARN-02	RBNU	2005	RMB	11:26	BCCH
	70770	ON	RBNU	06-ARN-02	RBNU	2005	RMB	11:26	BCCH
	70770	ON	RBNU	06-ARN-02	RBNU	2005	RMB	11:26	BCCH
	70770	ON	RBNU	06-ARN-02	RBNU	2005	RMB	11:26	BCCH
	70770	ON	RBNU	06-ARN-02	RBNU	2005	RMB	11:26	RBNU
	70770	ON	RBNU	06-ARN-02	RBNU	2005	RMB	11:26	RBNU
	70770	ON	RBNU	06-ARN-02	RBNU	2005	RMB	11:26	RBNU
	70770	ON	RBNU	06-ARN-02	RBNU	2005	RMB	11:26	RBNU
	70770	ON	RBNU	06-ARN-02	RBNU	2005	RMB	11:26	RBNU
	70770	ON	RBNU	06-ARN-02	RBNU	2005	RMB	11:26	RBNU
	70770	ON	RBNU	06-ARN-02	RBNU	2005	RMB	11:26	MOCH
	70770	ON	RBNU	06-ARN-02	RBNU	2005	RMB	11:26	BCCH
IB&ARN	7974	ON	RBNU	07-ARN-01	MOCH	2005	MBR	9:37	MOCH
IB&ARN	7974	ON	RBNU	07-ARN-01	MOCH	2005	MBR	9:37	BCCH
IB&ARN	7974	ON	RBNU	07-ARN-01	MOCH	2005	MBR	9:37	RBNU
IB&ARN	7974	ON	RBNU	07-ARN-01	MOCH	2005	MBR	9:37	MOCH
IB&ARN	7974	ON	RBNU	07-ARN-01	MOCH	2005	MBR	9:37	BCCH
IB&ARN	7974	ON	RBNU	07-ARN-01	MOCH	2005	MBR	9:37	MOCH
IB&ARN	7974	ON	RBNU	07-ARN-01	MOCH	2005	MBR	9:37	BCCH
IB&ARN	7974	ON	RBNU	07-ARN-01	MOCH	2005	MBR	9:37	BCCH
IB&ARN	7974	ON	RBNU	07-ARN-01	MOCH	2005	MBR	9:37	BCCH
IB&ARN	7974	ON	RBNU	07-ARN-01	MOCH	2005	MBR	9:37	BCCH
IB&ARN	7974	ON	RBNU	07-ARN-01	MOCH	2005	MBR	9:37	RBNU
IB&ARN	7974	ON	RBNU	07-ARN-01	MOCH	2005	MBR	9:37	RBNU
IB&ARN	7974	ON	RBNU	07-ARN-01	MOCH	2005	MBR	9:37	RBNU
IB&ARN	7974	ON	RBNU	07-ARN-01	MOCH	2005	MBR	9:37	RBNU
IB&ARN	7974	ON	RBNU	07-ARN-01	MOCH	2005	MBR	9:37	RBNU
IB&ARN	7974	ON	RBNU	07-ARN-01	MOCH	2005	MBR	9:37	RBNU
IB&ARN	7974	ON	RBNU	07-ARN-01	MOCH	2005	MBR	9:37	MOCH
IB&ARN	7974	ON	RBNU	07-ARN-01	MOCH	2005	MBR	9:37	BCCH

IB&ARN	7974	ON	RBNU	07-ARN-01	MOCH	2005	MBR	9:37	BCCH
IB&ARN	7974	ON	RBNU	07-ARN-01	MOCH	2005	MBR	9:37	MOCH
IB&ARN	7974	ON	RBNU	07-ARN-01	MOCH	2005	MBR	9:37	MOCH
IB&ARN	7974	ON	RBNU	07-ARN-01	MOCH	2005	MBR	9:37	RBNU
IB&ARN	7974	ON	RBNU	07-ARN-01	MOCH	2005	MBR	9:37	RBNU
IB&ARN	7974	ON	RBNU	07-ARN-01	MOCH	2005	MBR	9:37	MOCH
IB&ARN	7974	ON	RBNU	07-ARN-01	MOCH	2005	MBR	9:37	BCCH
IB&ARN	7974	ON	RBNU	07-ARN-01	MOCH	2005	MBR	9:37	MOCH
IB&ARN	7974	ON	RBNU	07-ARN-01	MOCH	2005	MBR	9:37	BCCH
IB&ARN	7974	ON	RBNU	07-ARN-01	MOCH	2005	MBR	9:37	RBNU
IB&ARN	7974	ON	RBNU	07-ARN-01	MOCH	2005	MBR	9:37	BCCH
IB&ARN	7974	ON	RBNU	07-ARN-01	MOCH	2005	MBR	9:37	RBNU
IB	70005	ON	RBNU	07-ARN-01	RBNU	2006	RMB	11:05	MOCH
IB	70005	ON	RBNU	07-ARN-01	RBNU	2006	RMB	11:05	MOCH
IB	70005	ON	RBNU	07-ARN-01	RBNU	2006	RMB	11:05	BCCH
IB	70005	ON	RBNU	07-ARN-01	RBNU	2006	RMB	11:05	MOCH
IB	70005	ON	RBNU	07-ARN-01	RBNU	2006	RMB	11:05	MOCH
IB	70005	ON	RBNU	07-ARN-01	RBNU	2006	RMB	11:05	MOCH
IB	70005	ON	RBNU	07-ARN-01	RBNU	2006	RMB	11:05	MOCH
IB	70005	ON	RBNU	07-ARN-01	RBNU	2006	RMB	11:05	BCCH
IB	70005	ON	RBNU	07-ARN-01	RBNU	2006	RMB	11:05	BCCH
IB	70005	ON	RBNU	07-ARN-01	RBNU	2006	RMB	11:05	MOCH
IB	70005	ON	RBNU	07-ARN-01	RBNU	2006	RMB	11:05	MOCH
IB	70005	ON	RBNU	07-ARN-01	RBNU	2006	RMB	11:05	MOCH
IB	70005	ON	RBNU	07-ARN-01	RBNU	2006	RMB	11:05	BCCH
IB	70005	ON	RBNU	07-ARN-01	RBNU	2006	RMB	11:05	MOCH
IB	70005	ON	RBNU	07-ARN-01	RBNU	2006	RMB	11:05	BCCH
IB	70005	ON	RBNU	07-ARN-01	RBNU	2006	RMB	11:05	BCCH
IB	70005	ON	RBNU	07-ARN-01	RBNU	2006	RMB	11:05	BCCH
IB	70005	ON	RBNU	07-ARN-01	RBNU	2006	RMB	11:05	RBNU
IB	70005	ON	RBNU	07-ARN-01	RBNU	2006	RMB	11:05	RBNU
IB	70005	ON	RBNU	07-ARN-01	RBNU	2006	RMB	11:05	RBNU
IB	70005	ON	RBNU	07-ARN-01	RBNU	2006	RMB	11:05	MOCH
IB	70005	ON	RBNU	07-ARN-01	RBNU	2006	RMB	11:05	MOCH
IB	70005	ON	RBNU	07-ARN-01	RBNU	2006	RMB	11:05	RBNU
IB	70005	ON	RBNU	07-ARN-01	RBNU	2006	RMB	11:05	MOCH
IB	70005	ON	RBNU	07-ARN-01	RBNU	2006	RMB	11:05	RBNU
IB	70005	ON	RBNU	07-ARN-01	RBNU	2006	RMB	11:05	RBNU
IB	70005	ON	RBNU	07-ARN-01	RBNU	2006	RMB	11:05	RBNU
IB	70005	ON	RBNU	07-ARN-01	RBNU	2006	RMB	11:05	RBNU
IB	70005	ON	RBNU	07-ARN-01	RBNU	2006	RMB	11:05	RBNU
IB	70005	ON	RBNU	07-ARN-01	RBNU	2006	RMB	11:05	RBNU
IB	70005	ON	RBNU	07-ARN-01	RBNU	2006	RMB	11:05	MOCH
IB	70005	ON	RBNU	07-ARN-01	RBNU	2006	RMB	11:05	MOCH

IB	70005	ON	RBNU	07-ARN-01	RBNU	2006	RMB	11:05	MOCH
IB	70005	ON	RBNU	07-ARN-01	RBNU	2006	RMB	11:05	MOCH
IB	70005	ON	RBNU	07-ARN-01	RBNU	2006	RMB	11:05	MOCH
IB	70005	ON	RBNU	07-ARN-01	RBNU	2006	RMB	11:05	BCCH
IB	70005	ON	RBNU	07-ARN-01	RBNU	2006	RMB	11:05	RBNU
IB	70005	ON	RBNU	07-ARN-01	RBNU	2006	RMB	11:05	RBNU
IB	70005	ON	RBNU	07-ARN-01	RBNU	2006	RMB	11:05	MOCH
IB	70005	ON	RBNU	07-ARN-01	RBNU	2006	RMB	11:05	BCCH
IB	70415	ON	RBNU	07-ARN-01	RBNU	2005	MRB	8:11	BCCH
IB	70415	ON	RBNU	07-ARN-01	RBNU	2005	MRB	8:11	BCCH
IB	70415	ON	RBNU	07-ARN-01	RBNU	2005	MRB	8:11	BCCH
IB	70415	ON	RBNU	07-ARN-01	RBNU	2005	MRB	8:11	BCCH
IB	70415	ON	RBNU	07-ARN-01	RBNU	2005	MRB	8:11	BCCH
IB	70415	ON	RBNU	07-ARN-01	RBNU	2005	MRB	8:11	BCCH
IB	70415	ON	RBNU	07-ARN-01	RBNU	2005	MRB	8:11	BCCH
IB	70415	ON	RBNU	07-ARN-01	RBNU	2005	MRB	8:11	BCCH
IB	70415	ON	RBNU	07-ARN-01	RBNU	2005	MRB	8:11	BCCH
IB	70415	ON	RBNU	07-ARN-01	RBNU	2005	MRB	8:11	RBNU
IB	70415	ON	RBNU	07-ARN-01	RBNU	2005	MRB	8:11	BCCH
IB	70415	ON	RBNU	07-ARN-01	RBNU	2005	MRB	8:11	MOCH
IB	70415	ON	RBNU	07-ARN-01	RBNU	2005	MRB	8:11	RBNU
IB	70415	ON	RBNU	07-ARN-01	RBNU	2005	MRB	8:11	MOCH
IB	70415	ON	RBNU	07-ARN-01	RBNU	2005	MRB	8:11	MOCH
IB	70415	ON	RBNU	07-ARN-01	RBNU	2005	MRB	8:11	RBNU
IB	70415	ON	RBNU	07-ARN-01	RBNU	2005	MRB	8:11	MOCH
IB	70415	ON	RBNU	07-ARN-01	RBNU	2005	MRB	8:11	MOCH
IB	70415	ON	RBNU	07-ARN-01	RBNU	2005	MRB	8:11	BCCH
IB	70415	ON	RBNU	07-ARN-01	RBNU	2005	MRB	8:11	MOCH
IB	70415	ON	RBNU	07-ARN-01	RBNU	2005	MRB	8:11	RBNU
IB	70415	ON	RBNU	07-ARN-01	RBNU	2005	MRB	8:11	RBNU
IB	70415	ON	RBNU	07-ARN-01	RBNU	2005	MRB	8:11	RBNU
IB	70415	ON	RBNU	07-ARN-01	RBNU	2005	MRB	8:11	RBNU
IB	70415	ON	RBNU	07-ARN-01	RBNU	2005	MRB	8:11	RBNU
IB	70415	ON	RBNU	07-ARN-01	RBNU	2005	MRB	8:11	RBNU
IB	70415	ON	RBNU	07-ARN-01	RBNU	2005	MRB	8:11	RBNU
IB	70415	ON	RBNU	07-ARN-01	RBNU	2005	MRB	8:11	RBNU
IB	70415	ON	RBNU	07-ARN-01	RBNU	2005	MRB	8:11	RBNU
IB	70415	ON	RBNU	07-ARN-01	RBNU	2005	MRB	8:11	RBNU
IB	70415	ON	RBNU	07-ARN-01	RBNU	2005	MRB	8:11	RBNU
IB	70415	ON	RBNU	07-ARN-01	RBNU	2005	MRB	8:11	RBNU
IB	70415	ON	RBNU	07-ARN-01	RBNU	2005	MRB	8:11	RBNU
IB	70415	ON	RBNU	07-ARN-01	RBNU	2005	MRB	8:11	RBNU
IB	70415	ON	RBNU	07-ARN-01	RBNU	2005	MRB	8:11	BCCH
IB	70415	ON	RBNU	07-ARN-01	RBNU	2005	MRB	8:11	MOCH

IB	70415	ON	RBNU	07-ARN-01	RBNU	2005	MRB	8:11	MOCH
IB	70415	ON	RBNU	07-ARN-01	RBNU	2005	MRB	8:11	MOCH
IB	70415	ON	RBNU	07-ARN-01	RBNU	2005	MRB	8:11	MOCH
IB	70415	ON	RBNU	07-ARN-01	RBNU	2005	MRB	8:11	MOCH
IB	70415	ON	RBNU	07-ARN-01	RBNU	2005	MRB	8:11	RBNU
IB	70415	ON	RBNU	07-ARN-01	RBNU	2005	MRB	8:11	BCCH
IB	7974	ON	RBNU	07-ARN-01	MOCH	2005	MBR	11:10	MOCH
IB	7974	ON	RBNU	07-ARN-01	MOCH	2005	MBR	11:10	RBNU
IB	7974	ON	RBNU	07-ARN-01	MOCH	2005	MBR	11:10	RBNU
IB	7974	ON	RBNU	07-ARN-01	MOCH	2005	MBR	11:10	RBNU
IB	7974	ON	RBNU	07-ARN-01	MOCH	2005	MBR	11:10	BCCH
IB	7974	ON	RBNU	07-ARN-01	MOCH	2005	MBR	11:10	RBNU
IB	7974	ON	RBNU	07-ARN-01	MOCH	2005	MBR	11:10	none
IB	7974	ON	RBNU	07-ARN-01	MOCH	2005	MBR	11:10	none
IB	7974	ON	RBNU	07-ARN-01	MOCH	2005	MBR	11:10	RBNU
IB	7974	ON	RBNU	07-ARN-01	MOCH	2005	MBR	11:10	RBNU
IB	7974	ON	RBNU	07-ARN-01	MOCH	2005	MBR	11:10	none
IB	7974	ON	RBNU	07-ARN-01	MOCH	2005	MBR	11:10	BCCH
IB	7974	ON	RBNU	07-ARN-01	MOCH	2005	MBR	11:10	RBNU
IB	7974	ON	RBNU	07-ARN-01	MOCH	2005	MBR	11:10	RBNU
IB	70198	ON	RBNU	07-ARN-03	RBNU	2006	BRM	10:06	MOCH
IB	70198	ON	RBNU	07-ARN-03	RBNU	2006	BRM	10:06	MOCH
IB	70198	ON	RBNU	07-ARN-03	RBNU	2006	BRM	10:06	MOCH
IB	70198	ON	RBNU	07-ARN-03	RBNU	2006	BRM	10:06	MOCH
IB	70198	ON	RBNU	07-ARN-03	RBNU	2006	BRM	10:06	MOCH
IB	70198	ON	RBNU	07-ARN-03	RBNU	2006	BRM	10:06	MOCH
IB	70198	ON	RBNU	07-ARN-03	RBNU	2006	BRM	10:06	MOCH
IB	70198	ON	RBNU	07-ARN-03	RBNU	2006	BRM	10:06	BCCH
IB	70198	ON	RBNU	07-ARN-03	RBNU	2006	BRM	10:06	RBNU
IB	70198	ON	RBNU	07-ARN-03	RBNU	2006	BRM	10:06	MOCH
IB	70198	ON	RBNU	07-ARN-03	RBNU	2006	BRM	10:06	MOCH
IB	70198	ON	RBNU	07-ARN-03	RBNU	2006	BRM	10:06	
ARN	7972	ON	RBNU	07-LP-024	MOCH	2005	BMR	8:50 AM	RBNU
ARN	7972	ON	RBNU	07-LP-024	MOCH	2005	BMR	8:50 AM	MOCH
ARN	7972	ON	RBNU	07-LP-024	MOCH	2005	BMR	8:50 AM	RBNU
ARN	7972	ON	RBNU	07-LP-024	MOCH	2005	BMR	8:50 AM	BCCH
ARN	7972	ON	RBNU	07-LP-024	MOCH	2005	BMR	8:50 AM	MOCH
ARN	7972	ON	RBNU	07-LP-024	MOCH	2005	BMR	8:50 AM	RBNU
ARN	7972	ON	RBNU	07-LP-024	MOCH	2005	BMR	8:50 AM	RBNU
ARN	7972	ON	RBNU	07-LP-024	MOCH	2005	BMR	8:50 AM	RBNU
ARN	7972	ON	RBNU	07-LP-024	MOCH	2005	BMR	8:50 AM	RBNU
ARN	7972	ON	RBNU	07-LP-024	MOCH	2005	BMR	8:50 AM	BCCH
ARN	7972	ON	RBNU	07-LP-024	MOCH	2005	BMR	8:50 AM	BCCH
ARN	7972	ON	RBNU	07-LP-024	MOCH	2005	BMR	8:50 AM	MOCH

ARN	7972	ON	RBNU	07-LP-024	MOCH	2005	BMR	8:50 AM	MOCH
ARN	7972	ON	RBNU	07-LP-024	MOCH	2005	BMR	8:50 AM	RBNU
ARN	7972	ON	RBNU	07-LP-024	MOCH	2005	BMR	8:50 AM	RBNU
ARN	7972	ON	RBNU	07-LP-024	MOCH	2005	BMR	8:50 AM	RBNU
ARN	7972	ON	RBNU	07-LP-024	MOCH	2005	BMR	8:50 AM	RBNU
ARN	7972	ON	RBNU	07-LP-024	MOCH	2005	BMR	8:50 AM	MOCH
ARN	7972	ON	RBNU	07-LP-024	MOCH	2005	BMR	8:50 AM	
ARN	7972	ON	RBNU	07-LP-024	MOCH	2005	BMR	8:50 AM	BCCH
ARN	7972	ON	RBNU	07-LP-024	MOCH	2005	BMR	8:50 AM	RBNU
ARN	7972	ON	RBNU	07-LP-024	MOCH	2005	BMR	8:50 AM	BCCH
ARN	7972	ON	RBNU	07-LP-024	MOCH	2005	BMR	8:50 AM	MOCH
ARN	7972	ON	RBNU	07-LP-024	MOCH	2005	BMR	8:50 AM	RBNU
ARN	7972	ON	RBNU	07-LP-024	MOCH	2005	BMR	8:50 AM	
IB	70005	ON	RBNU	07-ARN-01	RBNU	2006	RMB	8:01 AM	MOCH
IB	70005	ON	RBNU	07-ARN-01	RBNU	2006	RMB	8:01 AM	MOCH
IB	70005	ON	RBNU	07-ARN-01	RBNU	2006	RMB	8:01 AM	BCCH
IB	70005	ON	RBNU	07-ARN-01	RBNU	2006	RMB	8:01 AM	MOCH
IB	70005	ON	RBNU	07-ARN-01	RBNU	2006	RMB	8:01 AM	MOCH
IB	70005	ON	RBNU	07-ARN-01	RBNU	2006	RMB	8:01 AM	RBNU
IB	70005	ON	RBNU	07-ARN-01	RBNU	2006	RMB	8:01 AM	BCCH
IB	70005	ON	RBNU	07-ARN-01	RBNU	2006	RMB	8:01 AM	BCCH
IB	70005	ON	RBNU	07-ARN-01	RBNU	2006	RMB	8:01 AM	BCCH
IB	70005	ON	RBNU	07-ARN-01	RBNU	2006	RMB	8:01 AM	MOCH
IB	70005	ON	RBNU	07-ARN-01	RBNU	2006	RMB	8:01 AM	RBNU
IB	70005	ON	RBNU	07-ARN-01	RBNU	2006	RMB	8:01 AM	BCCH
IB	70005	ON	RBNU	07-ARN-01	RBNU	2006	RMB	8:01 AM	BCCH
IB	70005	ON	RBNU	07-ARN-01	RBNU	2006	RMB	8:01 AM	BCCH
IB	70005	ON	RBNU	07-ARN-01	RBNU	2006	RMB	8:01 AM	MOCH
IB	70005	ON	RBNU	07-ARN-01	RBNU	2006	RMB	8:01 AM	RBNU
IB	70005	ON	RBNU	07-ARN-01	RBNU	2006	RMB	8:01 AM	MOCH
ARN	70900	ON	RBNU	08-ARN-00	RBNU	2007	MBR	13:26	MOCH
ARN	70900	ON	RBNU	08-ARN-00	RBNU	2007	MBR	13:26	MOCH
ARN	70900	ON	RBNU	08-ARN-00	RBNU	2007	MBR	13:26	BCCH
ARN	70900	ON	RBNU	08-ARN-00	RBNU	2007	MBR	13:26	BCCH
ARN	70900	ON	RBNU	08-ARN-00	RBNU	2007	MBR	13:26	MOCH
ARN	70900	ON	RBNU	08-ARN-00	RBNU	2007	MBR	13:26	MOCH
ARN	70900	ON	RBNU	08-ARN-00	RBNU	2007	MBR	13:26	RBNU
ARN	70900	ON	RBNU	08-ARN-00	RBNU	2007	MBR	13:26	
ARN	70900	ON	RBNU	08-ARN-00	RBNU	2007	MBR	13:26	BCCH
ARN	70900	ON	RBNU	08-ARN-00	RBNU	2007	MBR	13:26	RBNU
ARN	70900	ON	RBNU	08-ARN-00	RBNU	2007	MBR	13:26	MOCH
ARN	70900	ON	RBNU	08-ARN-00	RBNU	2007	MBR	13:26	MOCH
ARN	70900	ON	RBNU	08-ARN-00	RBNU	2007	MBR	13:26	MOCH
ARN	70900	ON	RBNU	08-ARN-00	RBNU	2007	MBR	13:26	BCCH

ARN	70900	ON	RBNU	08-ARN-00	RBNU	2007	MBR	13:26	BCCH
ARN	70900	ON	RBNU	08-ARN-00	RBNU	2007	MBR	13:26	MOCH
ARN	70900	ON	RBNU	08-ARN-00	RBNU	2007	MBR	13:26	BCCH
ARN	70900	ON	RBNU	08-ARN-00	RBNU	2007	MBR	13:26	RBNU
ARN	70900	ON	RBNU	08-ARN-00	RBNU	2007	MBR	13:26	RBNU
ARN	70900	ON	RBNU	08-ARN-00	RBNU	2007	MBR	13:26	
HK	7063	ON	RBNU	08-ARN-03	MOCH	2007	RBM	10:01	BCCH
HK	7063	ON	RBNU	08-ARN-03	MOCH	2007	RBM	10:01	MOCH
HK	7063	ON	RBNU	08-ARN-03	MOCH	2007	RBM	10:01	MOCH
HK	7063	ON	RBNU	08-ARN-03	MOCH	2007	RBM	10:01	MOCH
HK	7063	ON	RBNU	08-ARN-03	MOCH	2007	RBM	10:01	BCCH
HK	7063	ON	RBNU	08-ARN-03	MOCH	2007	RBM	10:01	MOCH
HK	7063	ON	RBNU	08-ARN-03	MOCH	2007	RBM	10:01	
HK	7063	ON	RBNU	08-ARN-03	MOCH	2007	RBM	10:01	
HK	7063	ON	RBNU	08-ARN-03	MOCH	2007	RBM	10:01	
HK	7063	ON	RBNU	08-ARN-03	MOCH	2007	RBM	10:01	BCCH
HK	7063	ON	RBNU	08-ARN-03	MOCH	2007	RBM	10:01	MOCH
HK	7063	ON	RBNU	08-ARN-03	MOCH	2007	RBM	10:01	MOCH
HK	7063	ON	RBNU	08-ARN-03	MOCH	2007	RBM	10:01	MOCH
HK	7063	ON	RBNU	08-ARN-03	MOCH	2007	RBM	10:01	RBNU
HK	7063	ON	RBNU	08-ARN-03	MOCH	2007	RBM	10:01	BCCH
HK	7063	ON	RBNU	08-ARN-03	MOCH	2007	RBM	10:01	BCCH
HK	7063	ON	RBNU	08-ARN-03	MOCH	2007	RBM	10:01	MOCH
HK	7063	ON	RBNU	08-ARN-03	MOCH	2007	RBM	10:01	MOCH
HK	7063	ON	RBNU	08-ARN-03	MOCH	2007	RBM	10:01	MOCH
HK	7063	ON	RBNU	08-ARN-03	MOCH	2007	RBM	10:01	MOCH
HK	7063	ON	RBNU	08-ARN-03	MOCH	2007	RBM	10:01	MOCH
HK	7063	ON	RBNU	08-ARN-03	MOCH	2007	RBM	10:01	MOCH
HK	7063	ON	RBNU	08-ARN-03	MOCH	2007	RBM	10:01	BCCH
HK	7063	ON	RBNU	08-ARN-03	MOCH	2007	RBM	10:01	BCCH
HK	7063	ON	RBNU	08-ARN-03	MOCH	2007	RBM	10:01	BCCH
HK	7063	ON	RBNU	08-ARN-03	MOCH	2007	RBM	10:01	MOCH
HK	7063	ON	RBNU	08-ARN-03	MOCH	2007	RBM	10:01	
HK	7063	ON	RBNU	08-ARN-03	MOCH	2007	RBM	10:01	RBNU
HK	7063	ON	RBNU	08-ARN-03	MOCH	2007	RBM	10:01	BCCH
HK	7063	ON	RBNU	08-ARN-03	MOCH	2007	RBM	10:01	MOCH
HK	7063	ON	RBNU	08-ARN-03	MOCH	2007	RBM	10:01	MOCH
HK	7063	ON	RBNU	08-ARN-03	MOCH	2007	RBM	10:01	BCCH
HK	7063	ON	RBNU	08-ARN-03	MOCH	2007	RBM	10:01	
HK	70705	ON	RBNU	08-JT-001	MOCH	2005	RMB	11:00	BCCH
HK	70705	ON	RBNU	08-JT-001	MOCH	2005	RMB	11:00	BCCH
HK	70705	ON	RBNU	08-JT-001	MOCH	2005	RMB	11:00	BCCH
HK	70705	ON	RBNU	08-JT-001	MOCH	2005	RMB	11:00	RBNU
HK	70705	ON	RBNU	08-JT-001	MOCH	2005	RMB	11:00	RBNU

HK	70705	ON	RBNU	08-JT-001	MOCH	2005	RMB	11:00	RBNU
HK	70705	ON	RBNU	08-JT-001	MOCH	2005	RMB	11:00	RBNU
HK	70705	ON	RBNU	08-JT-001	MOCH	2005	RMB	11:00	RBNU
HK	70705	ON	RBNU	08-JT-001	MOCH	2005	RMB	11:00	MOCH
HK	70705	ON	RBNU	08-JT-001	MOCH	2005	RMB	11:00	MOCH
HK	70705	ON	RBNU	08-JT-001	MOCH	2005	RMB	11:00	RBNU
HK	70705	ON	RBNU	08-JT-001	MOCH	2005	RMB	11:00	MOCH
HK	70705	ON	RBNU	08-JT-001	MOCH	2005	RMB	11:00	MOCH
HK	70705	ON	RBNU	08-JT-001	MOCH	2005	RMB	11:00	RBNU
HK	70705	ON	RBNU	08-JT-001	MOCH	2005	RMB	11:00	MOCH
HK	70705	ON	RBNU	08-JT-001	MOCH	2005	RMB	11:00	MOCH
HK	70705	ON	RBNU	08-JT-001	MOCH	2005	RMB	11:00	RBNU
HK	70705	ON	RBNU	08-JT-001	MOCH	2005	RMB	11:00	BCCH
HK	70705	ON	RBNU	08-JT-001	MOCH	2005	RMB	11:00	RBNU
HK	70705	ON	RBNU	08-JT-001	MOCH	2005	RMB	11:00	RBNU
HK	70705	ON	RBNU	08-JT-001	MOCH	2005	RMB	11:00	MOCH
HK	70705	ON	RBNU	08-JT-001	MOCH	2005	RMB	11:00	MOCH
HK	70705	ON	RBNU	08-JT-001	MOCH	2005	RMB	11:00	MOCH
HK	70705	ON	RBNU	08-JT-001	MOCH	2005	RMB	11:00	MOCH
HK	70705	ON	RBNU	08-JT-001	MOCH	2005	RMB	11:00	BCCH
HK	70705	ON	RBNU	08-JT-001	MOCH	2005	RMB	11:00	RBNU
HK	70705	ON	RBNU	08-JT-001	MOCH	2005	RMB	11:00	RBNU
HK	70705	ON	RBNU	08-JT-001	MOCH	2005	RMB	11:00	MOCH
HK	70705	ON	RBNU	08-JT-001	MOCH	2005	RMB	11:00	BCCH
HK	70705	ON	RBNU	08-JT-001	MOCH	2005	RMB	11:00	BCCH
HK	70705	ON	RBNU	08-JT-001	MOCH	2005	RMB	11:00	RBNU
HK	70705	ON	RBNU	08-JT-001	MOCH	2005	RMB	11:00	MOCH
HK	70705	ON	RBNU	08-JT-001	MOCH	2005	RMB	11:00	MOCH
HK	70705	ON	RBNU	08-JT-001	MOCH	2005	RMB	11:00	MOCH
HK	70705	ON	RBNU	08-JT-001	MOCH	2005	RMB	11:00	BCCH
HK	70705	ON	RBNU	08-JT-001	MOCH	2005	RMB	11:00	MOCH
HK	70705	ON	RBNU	08-JT-001	MOCH	2005	RMB	11:00	MOCH
HK	70705	ON	RBNU	08-JT-001	MOCH	2005	RMB	11:00	BCCH
HK	70705	ON	RBNU	08-JT-001	MOCH	2005	RMB	11:00	BCCH
HK	70705	ON	RBNU	08-JT-001	MOCH	2005	RMB	11:00	BCCH
HK	70705	ON	RBNU	08-JT-001	MOCH	2005	RMB	11:00	RBNU
HK	70705	ON	RBNU	08-JT-001	MOCH	2005	RMB	11:00	RBNU
HK	70705	ON	RBNU	08-JT-001	MOCH	2005	RMB	11:00	RBNU
HK	70705	ON	RBNU	08-JT-001	MOCH	2005	RMB	11:00	MOCH
HK	70705	ON	RBNU	08-JT-001	MOCH	2005	RMB	11:00	BCCH
HK	70705	ON	RBNU	08-JT-001	MOCH	2005	RMB	11:00	BCCH
HK	70705	ON	RBNU	08-JT-001	MOCH	2005	RMB	11:00	
HK	70823	ON	RBNU	08-ARN-00	RBNU	2007	BMR	9:53	BCCH
HK	70823	ON	RBNU	08-ARN-00	RBNU	2007	BMR	9:53	BCCH

HK	70823	ON	RBNU	08-ARN-00 RBNU	2007	BMR	9:53	MOCH
HK	70823	ON	RBNU	08-ARN-00 RBNU	2007	BMR	9:53	MOCH
HK	70823	ON	RBNU	08-ARN-00 RBNU	2007	BMR	9:53	MOCH
HK	70823	ON	RBNU	08-ARN-00 RBNU	2007	BMR	9:53	MOCH
HK	70823	ON	RBNU	08-ARN-00 RBNU	2007	BMR	9:53	MOCH
HK	70823	ON	RBNU	08-ARN-00 RBNU	2007	BMR	9:53	MOCH
HK	70823	ON	RBNU	08-ARN-00 RBNU	2007	BMR	9:53	RBNU
HK	70823	ON	RBNU	08-ARN-00 RBNU	2007	BMR	9:53	MOCH
HK	70823	ON	RBNU	08-ARN-00 RBNU	2007	BMR	9:53	RBNU
HK	70823	ON	RBNU	08-ARN-00 RBNU	2007	BMR	9:53	BCCH
HK	70823	ON	RBNU	08-ARN-00 RBNU	2007	BMR	9:53	BCCH
HK	70823	ON	RBNU	08-ARN-00 RBNU	2007	BMR	9:53	MOCH
HK	70823	ON	RBNU	08-ARN-00 RBNU	2007	BMR	9:53	RBNU
HK	70823	ON	RBNU	08-ARN-00 RBNU	2007	BMR	9:53	RBNU
HK	70823	ON	RBNU	08-ARN-00 RBNU	2007	BMR	9:53	
HK	70829	ON	RBNU	08-ARN-01 RBNU	2007	BMR	10:06	RBNU
HK	70829	ON	RBNU	08-ARN-01 RBNU	2007	BMR	10:06	RBNU
HK	70829	ON	RBNU	08-ARN-01 RBNU	2007	BMR	10:06	RBNU
HK	70829	ON	RBNU	08-ARN-01 RBNU	2007	BMR	10:06	RBNU
HK	70829	ON	RBNU	08-ARN-01 RBNU	2007	BMR	10:06	RBNU
HK	70829	ON	RBNU	08-ARN-01 RBNU	2007	BMR	10:06	RBNU
HK	70829	ON	RBNU	08-ARN-01 RBNU	2007	BMR	10:06	RBNU
HK	70829	ON	RBNU	08-ARN-01 RBNU	2007	BMR	10:06	RBNU
HK	70829	ON	RBNU	08-ARN-01 RBNU	2007	BMR	10:06	RBNU
HK	70829	ON	RBNU	08-ARN-01 RBNU	2007	BMR	10:06	RBNU
HK	70829	ON	RBNU	08-ARN-01 RBNU	2007	BMR	10:06	RBNU
HK	70829	ON	RBNU	08-ARN-01 RBNU	2007	BMR	10:06	RBNU
HK	70829	ON	RBNU	08-ARN-01 RBNU	2007	BMR	10:06	RBNU
HK	70829	ON	RBNU	08-ARN-01 RBNU	2007	BMR	10:06	RBNU
HK	70829	ON	RBNU	08-ARN-01 RBNU	2007	BMR	10:06	RBNU
HK	70829	ON	RBNU	08-ARN-01 RBNU	2007	BMR	10:06	MOCH
HK	70829	ON	RBNU	08-ARN-01 RBNU	2007	BMR	10:06	MOCH
HK	70829	ON	RBNU	08-ARN-01 RBNU	2007	BMR	10:06	MOCH
HK	70829	ON	RBNU	08-ARN-01 RBNU	2007	BMR	10:06	MOCH
HK	70829	ON	RBNU	08-ARN-01 RBNU	2007	BMR	10:06	MOCH
HK	70829	ON	RBNU	08-ARN-01 RBNU	2007	BMR	10:06	RBNU
HK	70829	ON	RBNU	08-ARN-01 RBNU	2007	BMR	10:06	BCCH
HK	70829	ON	RBNU	08-ARN-01 RBNU	2007	BMR	10:06	MOCH
HK	70829	ON	RBNU	08-ARN-01 RBNU	2007	BMR	10:06	RBNU
HK	70829	ON	RBNU	08-ARN-01 RBNU	2007	BMR	10:06	RBNU
HK	70829	ON	RBNU	08-ARN-01 RBNU	2007	BMR	10:06	
HK	70003	ON	RBNU	08-ARN-04 RBNU	2006	MBR	10:17	BCCH
HK	70003	ON	RBNU	08-ARN-04 RBNU	2006	MBR	10:17	BCCH
HK	70003	ON	RBNU	08-ARN-04 RBNU	2006	MBR	10:17	BCCH

HK	70003	ON	RBNU	08-ARN-04	RBNU	2006	MBR	10:17	BCCH
HK	70003	ON	RBNU	08-ARN-04	RBNU	2006	MBR	10:17	BCCH
HK	70003	ON	RBNU	08-ARN-04	RBNU	2006	MBR	10:17	BCCH
HK	70003	ON	RBNU	08-ARN-04	RBNU	2006	MBR	10:17	BCCH
HK	70003	ON	RBNU	08-ARN-04	RBNU	2006	MBR	10:17	BCCH
HK	70003	ON	RBNU	08-ARN-04	RBNU	2006	MBR	10:17	BCCH
HK	70003	ON	RBNU	08-ARN-04	RBNU	2006	MBR	10:17	BCCH
HK	70003	ON	RBNU	08-ARN-04	RBNU	2006	MBR	10:17	BCCH
HK	70003	ON	RBNU	08-ARN-04	RBNU	2006	MBR	10:17	BCCH
HK	70003	ON	RBNU	08-ARN-04	RBNU	2006	MBR	10:17	BCCH
HK	70003	ON	RBNU	08-ARN-04	RBNU	2006	MBR	10:17	BCCH
HK	70003	ON	RBNU	08-ARN-04	RBNU	2006	MBR	10:17	BCCH
HK	70003	ON	RBNU	08-ARN-04	RBNU	2006	MBR	10:17	BCCH
HK	70003	ON	RBNU	08-ARN-04	RBNU	2006	MBR	10:17	BCCH
HK	70003	ON	RBNU	08-ARN-04	RBNU	2006	MBR	10:17	RBNU
HK	70003	ON	RBNU	08-ARN-04	RBNU	2006	MBR	10:17	RBNU
HK	70003	ON	RBNU	08-ARN-04	RBNU	2006	MBR	10:17	RBNU
HK	70003	ON	RBNU	08-ARN-04	RBNU	2006	MBR	10:17	BCCH
HK	70003	ON	RBNU	08-ARN-04	RBNU	2006	MBR	10:17	RBNU
HK	70003	ON	RBNU	08-ARN-04	RBNU	2006	MBR	10:17	MOCH
HK	70003	ON	RBNU	08-ARN-04	RBNU	2006	MBR	10:17	MOCH
HK	70003	ON	RBNU	08-ARN-04	RBNU	2006	MBR	10:17	MOCH
HK	70003	ON	RBNU	08-ARN-04	RBNU	2006	MBR	10:17	MOCH
HK	70003	ON	RBNU	08-ARN-04	RBNU	2006	MBR	10:17	MOCH
HK	70003	ON	RBNU	08-ARN-04	RBNU	2006	MBR	10:17	MOCH
HK	70003	ON	RBNU	08-ARN-04	RBNU	2006	MBR	10:17	MOCH
HK	70003	ON	RBNU	08-ARN-04	RBNU	2006	MBR	10:17	MOCH
HK	70003	ON	RBNU	08-ARN-04	RBNU	2006	MBR	10:17	MOCH
HK	70003	ON	RBNU	08-ARN-04	RBNU	2006	MBR	10:17	MOCH
HK	70003	ON	RBNU	08-ARN-04	RBNU	2006	MBR	10:17	MOCH
HK	70003	ON	RBNU	08-ARN-04	RBNU	2006	MBR	10:17	MOCH
HK	70003	ON	RBNU	08-ARN-04	RBNU	2006	MBR	10:17	MOCH
HK	70003	ON	RBNU	08-ARN-04	RBNU	2006	MBR	10:17	BCCH
HK	70003	ON	RBNU	08-ARN-04	RBNU	2006	MBR	10:17	MOCH
HK	70003	ON	RBNU	08-ARN-04	RBNU	2006	MBR	10:17	MOCH
HK	70003	ON	RBNU	08-ARN-04	RBNU	2006	MBR	10:17	MOCH
HK	70003	ON	RBNU	08-ARN-04	RBNU	2006	MBR	10:17	MOCH
HK	70003	ON	RBNU	08-ARN-04	RBNU	2006	MBR	10:17	MOCH
HK	70003	ON	RBNU	08-ARN-04	RBNU	2006	MBR	10:17	MOCH
HK	70003	ON	RBNU	08-ARN-04	RBNU	2006	MBR	10:17	MOCH
HK	70003	ON	RBNU	08-ARN-04	RBNU	2006	MBR	10:17	MOCH
HK	70003	ON	RBNU	08-ARN-04	RBNU	2006	MBR	10:17	MOCH
HK	70003	ON	RBNU	08-ARN-04	RBNU	2006	MBR	10:17	BCCH
HK	70003	ON	RBNU	08-ARN-04	RBNU	2006	MBR	10:17	BCCH

HK	70003	ON	RBNU	08-ARN-04	RBNU	2006	MBR	10:17	MOCH
HK	70003	ON	RBNU	08-ARN-04	RBNU	2006	MBR	10:17	BCCH
HK	70003	ON	RBNU	08-ARN-04	RBNU	2006	MBR	10:17	MOCH
HK	70003	ON	RBNU	08-ARN-04	RBNU	2006	MBR	10:17	MOCH
HK	70003	ON	RBNU	08-ARN-04	RBNU	2006	MBR	10:17	MOCH
HK	70003	ON	RBNU	08-ARN-04	RBNU	2006	MBR	10:17	MOCH
HK	70003	ON	RBNU	08-ARN-04	RBNU	2006	MBR	10:17	BCCH
HK	70003	ON	RBNU	08-ARN-04	RBNU	2006	MBR	10:17	RBNU
HK	70003	ON	RBNU	08-ARN-04	RBNU	2006	MBR	10:17	MOCH
HK	70003	ON	RBNU	08-ARN-04	RBNU	2006	MBR	10:17	MOCH
HK	70003	ON	RBNU	08-ARN-04	RBNU	2006	MBR	10:17	BCCH
HK	70003	ON	RBNU	08-ARN-04	RBNU	2006	MBR	10:17	BCCH
HK	70003	ON	RBNU	08-ARN-04	RBNU	2006	MBR	10:17	BCCH
HK	70003	ON	RBNU	08-ARN-04	RBNU	2006	MBR	10:17	MOCH
HK	70003	ON	RBNU	08-ARN-04	RBNU	2006	MBR	10:17	MOCH
HK	70003	ON	RBNU	08-ARN-04	RBNU	2006	MBR	10:17	BCCH
HK	70003	ON	RBNU	08-ARN-04	RBNU	2006	MBR	10:17	MOCH
HK	70003	ON	RBNU	08-ARN-04	RBNU	2006	MBR	10:17	MOCH
HK	70003	ON	RBNU	08-ARN-04	RBNU	2006	MBR	10:17	BCCH
HK	70003	ON	RBNU	08-ARN-04	RBNU	2006	MBR	10:17	RBNU
HK	70003	ON	RBNU	08-ARN-04	RBNU	2006	MBR	10:17	RBNU
HK	70003	ON	RBNU	08-ARN-04	RBNU	2006	MBR	10:17	RBNU
HK	70003	ON	RBNU	08-ARN-04	RBNU	2006	MBR	10:17	RBNU
HK	70003	ON	RBNU	08-ARN-04	RBNU	2006	MBR	10:17	MOCH
HK	70003	ON	RBNU	08-ARN-04	RBNU	2006	MBR	10:17	BCCH
HK	70003	ON	RBNU	08-ARN-04	RBNU	2006	MBR	10:17	RBNU
HK	70003	ON	RBNU	08-ARN-04	RBNU	2006	MBR	10:17	MOCH
HK	70003	ON	RBNU	08-ARN-04	RBNU	2006	MBR	10:17	BCCH
HK	70003	ON	RBNU	08-ARN-04	RBNU	2006	MBR	10:17	RBNU
HK	70003	ON	RBNU	08-ARN-04	RBNU	2006	MBR	10:17	RBNU
HK	70003	ON	RBNU	08-ARN-04	RBNU	2006	MBR	10:17	
HK	70832	ON	RBNU	08-AB-025	RBNU	2007	BRM	10:23	BCCH
HK	70832	ON	RBNU	08-AB-025	RBNU	2007	BRM	10:23	BCCH
HK	70832	ON	RBNU	08-AB-025	RBNU	2007	BRM	10:23	BCCH
HK	70832	ON	RBNU	08-AB-025	RBNU	2007	BRM	10:23	BCCH
HK	70832	ON	RBNU	08-AB-025	RBNU	2007	BRM	10:23	BCCH
HK	70832	ON	RBNU	08-AB-025	RBNU	2007	BRM	10:23	BCCH
HK	70832	ON	RBNU	08-AB-025	RBNU	2007	BRM	10:23	MOCH
HK	70832	ON	RBNU	08-AB-025	RBNU	2007	BRM	10:23	MOCH
HK	70832	ON	RBNU	08-AB-025	RBNU	2007	BRM	10:23	RBNU
HK	70832	ON	RBNU	08-AB-025	RBNU	2007	BRM	10:23	MOCH
HK	70832	ON	RBNU	08-AB-025	RBNU	2007	BRM	10:23	MOCH
HK	70832	ON	RBNU	08-AB-025	RBNU	2007	BRM	10:23	MOCH
HK	70832	ON	RBNU	08-AB-025	RBNU	2007	BRM	10:23	RBNU

HK	70832	ON	RBNU	08-AB-025	RBNU	2007	BRM	10:23	MOCH
HK	70832	ON	RBNU	08-AB-025	RBNU	2007	BRM	10:23	MOCH
HK	70832	ON	RBNU	08-AB-025	RBNU	2007	BRM	10:23	MOCH
HK	70832	ON	RBNU	08-AB-025	RBNU	2007	BRM	10:23	BCCH
HK	70832	ON	RBNU	08-AB-025	RBNU	2007	BRM	10:23	MOCH
HK	70832	ON	RBNU	08-AB-025	RBNU	2007	BRM	10:23	MOCH
HK	70832	ON	RBNU	08-AB-025	RBNU	2007	BRM	10:23	MOCH
HK	70832	ON	RBNU	08-AB-025	RBNU	2007	BRM	10:23	MOCH
HK	70832	ON	RBNU	08-AB-025	RBNU	2007	BRM	10:23	BCCH
HK	70832	ON	RBNU	08-AB-025	RBNU	2007	BRM	10:23	BCCH
HK	70832	ON	RBNU	08-AB-025	RBNU	2007	BRM	10:23	MOCH
HK	70832	ON	RBNU	08-AB-025	RBNU	2007	BRM	10:23	MOCH
HK	70832	ON	RBNU	08-AB-025	RBNU	2007	BRM	10:23	MOCH
HK	70832	ON	RBNU	08-AB-025	RBNU	2007	BRM	10:23	MOCH
HK	70832	ON	RBNU	08-AB-025	RBNU	2007	BRM	10:23	MOCH
HK	70832	ON	RBNU	08-AB-025	RBNU	2007	BRM	10:23	BCCH
HK	70832	ON	RBNU	08-AB-025	RBNU	2007	BRM	10:23	RBNU
HK	70832	ON	RBNU	08-AB-025	RBNU	2007	BRM	10:23	RBNU
HK	70832	ON	RBNU	08-AB-025	RBNU	2007	BRM	10:23	RBNU
HK	70832	ON	RBNU	08-AB-025	RBNU	2007	BRM	10:23	BCCH
HK	70832	ON	RBNU	08-AB-025	RBNU	2007	BRM	10:23	RBNU
HK	70832	ON	RBNU	08-AB-025	RBNU	2007	BRM	10:23	MOCH
HK	70832	ON	RBNU	08-AB-025	RBNU	2007	BRM	10:23	MOCH
HK	70832	ON	RBNU	08-AB-025	RBNU	2007	BRM	10:23	
HK	7063	ON	RBNU	08-ARN-03	MOCH	2007	RBM	8:41	RBNU
HK	7063	ON	RBNU	08-ARN-03	MOCH	2007	RBM	8:41	RBNU
HK	7063	ON	RBNU	08-ARN-03	MOCH	2007	RBM	8:41	RBNU
HK	7063	ON	RBNU	08-ARN-03	MOCH	2007	RBM	8:41	RBNU
HK	7063	ON	RBNU	08-ARN-03	MOCH	2007	RBM	8:41	RBNU
HK	7063	ON	RBNU	08-ARN-03	MOCH	2007	RBM	8:41	MOCH
HK	7063	ON	RBNU	08-ARN-03	MOCH	2007	RBM	8:41	RBNU
HK	7063	ON	RBNU	08-ARN-03	MOCH	2007	RBM	8:41	BCCH
HK	7063	ON	RBNU	08-ARN-03	MOCH	2007	RBM	8:41	MOCH
HK	7063	ON	RBNU	08-ARN-03	MOCH	2007	RBM	8:41	MOCH
HK	7063	ON	RBNU	08-ARN-03	MOCH	2007	RBM	8:41	
HK	70003	ON	RBNU	08-ARN-04	RBNU	2006	MRB	11:25	BCCH
HK	70003	ON	RBNU	08-ARN-04	RBNU	2006	MRB	11:25	BCCH
HK	70003	ON	RBNU	08-ARN-04	RBNU	2006	MRB	11:25	MOCH
HK	70003	ON	RBNU	08-ARN-04	RBNU	2006	MRB	11:25	
HK	70003	ON	RBNU	08-ARN-04	RBNU	2006	MRB	11:25	BCCH
HK	70003	ON	RBNU	08-ARN-04	RBNU	2006	MRB	11:25	MOCH
HK	70003	ON	RBNU	08-ARN-04	RBNU	2006	MRB	11:25	BCCH
HK	70003	ON	RBNU	08-ARN-04	RBNU	2006	MRB	11:25	BCCH
HK	70003	ON	RBNU	08-ARN-04	RBNU	2006	MRB	11:25	MOCH

HK	70003	ON	RBNU	08-ARN-04	RBNU	2006	MRB	11:25	MOCH
HK	70003	ON	RBNU	08-ARN-04	RBNU	2006	MRB	11:25	RBNU
HK	70003	ON	RBNU	08-ARN-04	RBNU	2006	MRB	11:25	BCCH
HK	70003	ON	RBNU	08-ARN-04	RBNU	2006	MRB	11:25	BCCH
HK	70003	ON	RBNU	08-ARN-04	RBNU	2006	MRB	11:25	
HK	70823	ON	RBNU	08-ARN-00	RBNU	2007	RBM	7:59	RBNU
HK	70823	ON	RBNU	08-ARN-00	RBNU	2007	RBM	7:59	RBNU
HK	70823	ON	RBNU	08-ARN-00	RBNU	2007	RBM	7:59	RBNU
HK	70823	ON	RBNU	08-ARN-00	RBNU	2007	RBM	7:59	BCCH
HK	70823	ON	RBNU	08-ARN-00	RBNU	2007	RBM	7:59	RBNU
HK	70823	ON	RBNU	08-ARN-00	RBNU	2007	RBM	7:59	RBNU
HK	70823	ON	RBNU	08-ARN-00	RBNU	2007	RBM	7:59	BCCH
HK	70823	ON	RBNU	08-ARN-00	RBNU	2007	RBM	7:59	MOCH
HK	70823	ON	RBNU	08-ARN-00	RBNU	2007	RBM	7:59	MOCH
HK	70823	ON	RBNU	08-ARN-00	RBNU	2007	RBM	7:59	
HK	70832	ON	RBNU	08-AB-025	RBNU	2007	MRB	7:57	BCCH
HK	70832	ON	RBNU	08-AB-025	RBNU	2007	MRB	7:57	BCCH
HK	70832	ON	RBNU	08-AB-025	RBNU	2007	MRB	7:57	BCCH
HK	70832	ON	RBNU	08-AB-025	RBNU	2007	MRB	7:57	MOCH
HK	70832	ON	RBNU	08-AB-025	RBNU	2007	MRB	7:57	BCCH
HK	70832	ON	RBNU	08-AB-025	RBNU	2007	MRB	7:57	MOCH
HK	70832	ON	RBNU	08-AB-025	RBNU	2007	MRB	7:57	MOCH
HK	70832	ON	RBNU	08-AB-025	RBNU	2007	MRB	7:57	MOCH
HK	70832	ON	RBNU	08-AB-025	RBNU	2007	MRB	7:57	MOCH
HK	70832	ON	RBNU	08-AB-025	RBNU	2007	MRB	7:57	RBNU
HK	70832	ON	RBNU	08-AB-025	RBNU	2007	MRB	7:57	RBNU
HK	70832	ON	RBNU	08-AB-025	RBNU	2007	MRB	7:57	RBNU
HK	70832	ON	RBNU	08-AB-025	RBNU	2007	MRB	7:57	RBNU
HK	70832	ON	RBNU	08-AB-025	RBNU	2007	MRB	7:57	BCCH
HK	70832	ON	RBNU	08-AB-025	RBNU	2007	MRB	7:57	BCCH
HK	70832	ON	RBNU	08-AB-025	RBNU	2007	MRB	7:57	MOCH
HK	70832	ON	RBNU	08-AB-025	RBNU	2007	MRB	7:57	RBNU
HK	70832	ON	RBNU	08-AB-025	RBNU	2007	MRB	7:57	
HK	70832	ON	RBNU	08-AB-025	RBNU	2007	MRB	7:57	BCCH
HK	70832	ON	RBNU	08-AB-025	RBNU	2007	MRB	7:57	MOCH
HK	70832	ON	RBNU	08-AB-025	RBNU	2007	MRB	7:57	RBNU
HK	70832	ON	RBNU	08-AB-025	RBNU	2007	MRB	7:57	BCCH
HK	70832	ON	RBNU	08-AB-025	RBNU	2007	MRB	7:57	BCCH
HK	70832	ON	RBNU	08-AB-025	RBNU	2007	MRB	7:57	
IB	70153	ON	RBNU W. M	07-AE-019	MOCH	2006	BMR	10:26 AM	BCCH
IB	70153	ON	RBNU W. M	07-AE-019	MOCH	2006	BMR	10:26 AM	BCCH
IB	70153	ON	RBNU W. M	07-AE-019	MOCH	2006	BMR	10:26 AM	BCCH
IB	70153	ON	RBNU W. M	07-AE-019	MOCH	2006	BMR	10:26 AM	BCCH
IB	70153	ON	RBNU W. M	07-AE-019	MOCH	2006	BMR	10:26 AM	MOCH

IB	70153	ON	RBNU W. M	07-AE-019	MOCH	2006	BMR	10:26 AM	MOCH
IB	70153	ON	RBNU W. M	07-AE-019	MOCH	2006	BMR	10:26 AM	MOCH
IB	70153	ON	RBNU W. M	07-AE-019	MOCH	2006	BMR	10:26 AM	RBNU
IB	70153	ON	RBNU W. M	07-AE-019	MOCH	2006	BMR	10:26 AM	RBNU
IB	70153	ON	RBNU W. M	07-AE-019	MOCH	2006	BMR	10:26 AM	RBNU
IB	70153	ON	RBNU W. M	07-AE-019	MOCH	2006	BMR	10:26 AM	RBNU
IB	70153	ON	RBNU W. M	07-AE-019	MOCH	2006	BMR	10:26 AM	RBNU
IB	70153	ON	RBNU W. M	07-AE-019	MOCH	2006	BMR	10:26 AM	BCCH
IB	70153	ON	RBNU W. M	07-AE-019	MOCH	2006	BMR	10:26 AM	RBNU
IB	70153	ON	RBNU W. M	07-AE-019	MOCH	2006	BMR	10:26 AM	MOCH
IB	70153	ON	RBNU W. M	07-AE-019	MOCH	2006	BMR	10:26 AM	MOCH
IB	70153	ON	RBNU W. M	07-AE-019	MOCH	2006	BMR	10:26 AM	BCCH
IB	70153	ON	RBNU W. M	07-AE-019	MOCH	2006	BMR	10:26 AM	MOCH
IB	70153	ON	RBNU W. M	07-AE-019	MOCH	2006	BMR	10:26 AM	BCCH
IB	70153	ON	RBNU W. M	07-AE-019	MOCH	2006	BMR	10:26 AM	BCCH
IB	70153	ON	RBNU W. M	07-AE-019	MOCH	2006	BMR	10:26 AM	RBNU
IB	70153	ON	RBNU W. M	07-AE-019	MOCH	2006	BMR	10:26 AM	RBNU
IB	70153	ON	RBNU W. M	07-AE-019	MOCH	2006	BMR	10:26 AM	RBNU
IB	70153	ON	RBNU W. M	07-AE-019	MOCH	2006	BMR	10:26 AM	BCCH
IB	70153	ON	RBNU W. M	07-AE-019	MOCH	2006	BMR	10:26 AM	RBNU
IB	70153	ON	RBNU W. M	07-AE-019	MOCH	2006	BMR	10:26 AM	BCCH
IB	70153	ON	RBNU W. M	07-AE-019	MOCH	2006	BMR	10:26 AM	RBNU
IB	70153	ON	RBNU W. M	07-AE-019	MOCH	2006	BMR	10:26 AM	RBNU
IB	70153	ON	RBNU W. M	07-AE-019	MOCH	2006	BMR	10:26 AM	BCCH
IB	70153	ON	RBNU W. M	07-AE-019	MOCH	2006	BMR	10:26 AM	RBNU
IB	70153	ON	RBNU W. M	07-AE-019	MOCH	2006	BMR	10:26 AM	BCCH
IB	70153	ON	RBNU W. M	07-AE-019	MOCH	2006	BMR	10:26 AM	MOCH
IB	70153	ON	RBNU W. M	07-AE-019	MOCH	2006	BMR	10:26 AM	RBNU
IB	70153	ON	RBNU W. M	07-AE-019	MOCH	2006	BMR	10:26 AM	RBNU
IB	70153	ON	RBNU W. M	07-AE-019	MOCH	2006	BMR	10:26 AM	RBNU
IB	70153	ON	RBNU W. M	07-AE-019	MOCH	2006	BMR	10:26 AM	RBNU
IB	70153	ON	RBNU W. M	07-AE-019	MOCH	2006	BMR	10:26 AM	BCCH
IB	70153	ON	RBNU W. M	07-AE-019	MOCH	2006	BMR	10:26 AM	MOCH
IB	70153	ON	RBNU W. M	07-AE-019	MOCH	2006	BMR	10:26 AM	MOCH
IB	70153	ON	RBNU W. M	07-AE-019	MOCH	2006	BMR	10:26 AM	RBNU
IB	70153	ON	RBNU W. M	07-AE-019	MOCH	2006	BMR	10:26 AM	BCCH
IB	70153	ON	RBNU W. M	07-AE-019	MOCH	2006	BMR	10:26 AM	MOCH
IB	70153	ON	RBNU W. M	07-AE-019	MOCH	2006	BMR	10:26 AM	RBNU
	7117	R	MOCH	05-CW-017	TRES	2004	BRM	8:34	BCCH
	7117	R	MOCH	05-CW-017	TRES	2004	BRM	8:34	BCCH
	7117	R	MOCH	05-CW-017	TRES	2004	BRM	8:34	MOCH
	7117	R	MOCH	05-CW-017	TRES	2004	BRM	8:34	BCCH
	7974	R	MOCH	06-ARN-01	MOCH	2005	MBR	10:29	BCCH
	7974	R	MOCH	06-ARN-01	MOCH	2005	MBR	10:29	BCCH

7974	R	MOCH	06-ARN-01	MOCH	2005	MBR	10:29	RBNU
7974	R	MOCH	06-ARN-01	MOCH	2005	MBR	10:29	MOCH
7974	R	MOCH	06-ARN-01	MOCH	2005	MBR	10:29	BCCH
7974	R	MOCH	06-ARN-01	MOCH	2005	MBR	10:29	MOCH
7974	R	MOCH	06-ARN-01	MOCH	2005	MBR	10:29	RBNU
7974	R	MOCH	06-ARN-01	MOCH	2005	MBR	10:29	MOCH
7974	R	MOCH	06-ARN-01	MOCH	2005	MBR	10:29	MOCH
7974	R	MOCH	06-ARN-01	MOCH	2005	MBR	10:29	RBNU
7974	R	MOCH	06-ARN-01	MOCH	2005	MBR	10:29	MOCH
7974	R	MOCH	06-ARN-01	MOCH	2005	MBR	10:29	BCCH
7974	R	MOCH	06-ARN-01	MOCH	2005	MBR	10:29	RBNU
70122	R	MOCH	06-KS-027	RBNU	2005	BMR	9:28	BCCH
70122	R	MOCH	06-KS-027	RBNU	2005	BMR	9:28	MOCH
70122	R	MOCH	06-KS-027	RBNU	2005	BMR	9:28	RBNU
7020	R	MOCH	06-LD-015	MOCH	2005	MRB	10:51	MOCH
7020	R	MOCH	06-LD-015	MOCH	2005	MRB	10:51	RBNU
7020	R	MOCH	06-LD-015	MOCH	2005	MRB	10:51	MOCH
7020	R	MOCH	06-LD-015	MOCH	2005	MRB	10:51	RBNU
7020	R	MOCH	06-LD-015	MOCH	2005	MRB	10:51	BCCH
7020	R	MOCH	06-LD-015	MOCH	2005	MRB	10:51	RBNU
7020	R	MOCH	06-LD-015	MOCH	2005	MRB	10:51	RBNU
7020	R	MOCH	06-LD-015	MOCH	2005	MRB	10:51	RBNU
7020	R	MOCH	06-LD-015	MOCH	2005	MRB	10:51	BCCH
7020	R	MOCH	06-LD-015	MOCH	2005	RMB	10:49	RBNU
7020	R	MOCH	06-LD-015	MOCH	2005	RMB	10:49	RBNU
7020	R	MOCH	06-LD-015	MOCH	2005	RMB	10:49	RBNU
7020	R	MOCH	06-LD-015	MOCH	2005	RMB	10:49	RBNU
7020	R	MOCH	06-LD-015	MOCH	2005	RMB	10:49	RBNU
7020	R	MOCH	06-LD-015	MOCH	2005	RMB	10:49	MOCH
7020	R	MOCH	06-LD-015	MOCH	2005	RMB	10:49	BCCH
7020	R	MOCH	06-LD-015	MOCH	2005	RMB	10:49	none
7020	R	RBNU	06-ARN-01	MOCH	2005	MRB	10:51	MOCH
7020	R	RBNU	06-ARN-01	MOCH	2005	MRB	10:51	RBNU
7020	R	RBNU	06-ARN-01	MOCH	2005	MRB	10:51	MOCH
7020	R	RBNU	06-ARN-01	MOCH	2005	MRB	10:51	RBNU
7020	R	RBNU	06-ARN-01	MOCH	2005	MRB	10:51	BCCH
7020	R	RBNU	06-ARN-01	MOCH	2005	MRB	10:51	RBNU
7020	R	RBNU	06-ARN-01	MOCH	2005	MRB	10:51	RBNU
7020	R	RBNU	06-ARN-01	MOCH	2005	MRB	10:51	RBNU
7020	R	RBNU	06-ARN-01	MOCH	2005	MRB	10:51	BCCH
7020	R	RBNU	06-ARN-01	MOCH	2005	RMB	10:49	RBNU
7020	R	RBNU	06-ARN-01	MOCH	2005	RMB	10:49	RBNU
7020	R	RBNU	06-ARN-01	MOCH	2005	RMB	10:49	RBNU
7020	R	RBNU	06-ARN-01	MOCH	2005	RMB	10:49	RBNU

	7020	R	RBNU	06-ARN-01	MOCH	2005	RMB	10:49	RBNU
	7020	R	RBNU	06-ARN-01	MOCH	2005	RMB	10:49	MOCH
	7020	R	RBNU	06-ARN-01	MOCH	2005	RMB	10:49	BCCH
	7020	R	RBNU	06-ARN-01	MOCH	2005	RMB	10:49	none
	7912	R	RBNU	05-CW-033	RBNU	2003	BMR	15:43	MOCH
	7912	R	RBNU	05-CW-033	RBNU	2003	BMR	15:43	BCCH
	7912	R	RBNU	05-CW-033	RBNU	2003	BMR	15:43	BCCH
	7912	R	RBNU	05-CW-033	RBNU	2003	BMR	15:43	MOCH
	7912	R	RBNU	05-CW-033	RBNU	2003	BMR	15:43	RBNU
	7912	R	RBNU	05-CW-033	RBNU	2003	BMR	15:43	MOCH
	7912	R	RBNU	05-CW-033	RBNU	2003	BMR	15:43	RBNU
	7912	R	RBNU	05-CW-033	RBNU	2003	BMR	15:43	BCCH
	7912	R	RBNU	05-CW-033	RBNU	2003	BMR	15:43	MOCH
	70733	R	RBNU	05-CL-047	RNSA	2004	RMB	9:43	RBNU
	70733	R	RBNU	05-CL-047	RNSA	2004	RMB	9:43	RBNU
	70733/701	R	RBNU	05-CL-047	RNSA	2004	RMB	9:43	RBNU
	70733	R	RBNU	05-CL-047	RNSA	2004	RMB	9:43	MOCH
	70733	R	RBNU	05-CL-047	RNSA	2004	RMB	9:43	BCCH
	70733	R	RBNU	05-CL-047	RNSA	2004	RMB	9:43	MOCH
	70733	R	RBNU	05-CL-047	RNSA	2004	RMB	9:43	RBNU
	70733	R	RBNU	05-CL-047	RNSA	2004	RMB	9:43	MOCH
	70733	R	RBNU	05-CL-047	RNSA	2004	RMB	9:43	BCCH
	70718	R	RBNU	06-ARN-02	RBNU	2004	RMB	12:26	RBNU
	70718	R	RBNU	06-ARN-02	RBNU	2004	RMB	12:26	RBNU
	70718	R	RBNU	06-ARN-02	RBNU	2004	RMB	12:26	RBNU
	70718	R	RBNU	06-ARN-02	RBNU	2004	RMB	12:26	MOCH
	70718	R	RBNU	06-ARN-02	RBNU	2004	RMB	12:26	BCCH
	70718	R	RBNU	06-ARN-02	RBNU	2004	RMB	12:26	RBNU
	70718	R	RBNU	06-ARN-02	RBNU	2004	RMB	12:26	RBNU
	70718	R	RBNU	06-ARN-02	RBNU	2004	RMB	12:26	MOCH
	70718	R	RBNU	06-ARN-02	RBNU	2004	RMB	12:26	BCCH
	70718	OR			RBNU	2004	RMB	10:34	BCCH
	70718	OR			RBNU	2004	RMB	10:34	BCCH
	70718	OR			RBNU	2004	RMB	10:34	MOCH
	70718	OR			RBNU	2004	RMB	10:34	RBNU
	70718	OR			RBNU	2004	RMB	10:34	BCCH
	70718	OR			RBNU	2004	RMB	10:34	MOCH
	70718	OR			RBNU	2004	RMB	10:34	RBNU
	70718	OR			RBNU	2004	RMB	10:34	
IB	70716	OR			RNSA	2006	RMB	11:03	BCCH
IB	70716	OR			RNSA	2006	RMB	11:03	BCCH
IB	70716	OR			RNSA	2006	RMB	11:03	BCCH
IB	70716	OR			RNSA	2006	RMB	11:03	BCCH
IB	70716	OR			RNSA	2006	RMB	11:03	BCCH

IB	70716	OR			RNSA	2006	RMB	11:03	BCCH
IB	70716	OR			RNSA	2006	RMB	11:03	BCCH
IB	70716	OR			RNSA	2006	RMB	11:03	BCCH
IB	70716	OR			RNSA	2006	RMB	11:03	BCCH
IB	70716	OR			RNSA	2006	RMB	11:03	BCCH
IB	70716	OR			RNSA	2006	RMB	11:03	BCCH
IB	70716	OR			RNSA	2006	RMB	11:03	RBNU
IB	70716	OR			RNSA	2006	RMB	11:03	RBNU
IB	70716	OR			RNSA	2006	RMB	11:03	MOCH
IB	70716	OR			RNSA	2006	RMB	11:03	BCCH
	70102	R	BCCH		NEW	2004	BRM	10:00	BCCH
	70102	R	BCCH		NEW	2004	BRM	10:00	RBNU
	70102	R	BCCH		NEW	2004	BRM	10:00	RBNU
	7825	R	BCCH		BCCH	2004	BRM	8:00	none
	7825	R	BCCH		BCCH	2004	BRM	8:00	none
	7825	R	BCCH		BCCH	2004	BRM	8:00	MOCH
	7694	R	BCCH	05-RV-026	MOCH	2004	MBR	11:57	RBNU
	7928	R	BCCH	05-MN-029	RBNU	2003	BMR	9:57	BCCH
	7928	R	BCCH	05-MN-029	RBNU	2003	BMR	9:57	BCCH
	7928	R	BCCH	05-MN-029	RBNU	2003	BMR	9:57	BCCH
	7928	R	BCCH	05-MN-029	RBNU	2003	BMR	9:57	BCCH
	7928	R	BCCH	05-MN-029	RBNU	2003	BMR	9:57	BCCH
	7928	R	BCCH	05-MN-029	RBNU	2003	BMR	9:57	BCCH
	7928	R	BCCH	05-MN-029	RBNU	2003	BMR	9:57	BCCH
	7928	R	BCCH	05-MN-029	RBNU	2003	BMR	9:57	RBNU
	7928	R	BCCH	05-MN-029	RBNU	2003	BMR	9:57	MOCH
	7928	R	BCCH	05-MN-029	RBNU	2003	BMR	9:57	RBNU
	7928	R	BCCH	05-MN-029	RBNU	2003	BMR	9:57	RBNU
	7928	R	BCCH	05-MN-029	RBNU	2003	BMR	9:57	MOCH
	7130	R	MOCH		DOWO	2003	MBR	10:02	none
	7130	R	MOCH		DOWO	2003	MBR	10:02	MOCH
	7130	R	MOCH		DOWO	2003	MBR	10:02	MOCH
	7130	R	MOCH		DOWO	2003	MBR	10:02	RBNU
	7130	R	MOCH		DOWO	2003	MBR	10:02	RBNU
	04-NK-024	R	MOCH		MOCH	2004	BRM	8:55	none
	04-NK-024	R	MOCH		MOCH	2004	BRM	8:55	RBNU
	04-NK-024	R	MOCH		MOCH	2004	BRM	8:55	RBNU
	04-NK-024	R	MOCH		MOCH	2004	BRM	8:55	RBNU
	04-NK-024	R	MOCH		MOCH	2004	BRM	8:55	RBNU
	7545	R	MOCH	05-ARN-00	RBNU	2004	RBM	10:35	MOCH
	7545	R	MOCH	05-ARN-00	RBNU	2004	RBM	10:35	MOCH
	7545	R	MOCH	05-ARN-00	RBNU	2004	RBM	10:35	MOCH
	7545	R	MOCH	05-ARN-00	RBNU	2004	RBM	10:35	BCCH
	7545	R	MOCH	05-ARN-00	RBNU	2004	RBM	10:35	MOCH

7545	R	MOCH	05-ARN-00	RBNU	2004	RBM	10:35	MOCH
7545	R	MOCH	05-ARN-00	RBNU	2004	RBM	10:35	BCCH
7545	R	MOCH	05-ARN-00	RBNU	2004	RBM	10:35	BCCH
7280	R	MOCH	05-MN-005	RBNU	2003	RMB	11:12	MOCH
7280	R	MOCH	05-MN-005	RBNU	2003	RMB	11:12	BCCH
7280	R	MOCH	05-MN-005	RBNU	2003	RMB	11:12	BCCH
7280	R	MOCH	05-MN-005	RBNU	2003	RMB	11:12	MOCH
7280	R	MOCH	05-MN-005	RBNU	2003	RMB	11:12	MOCH
7280	R	MOCH	05-MN-005	RBNU	2003	RMB	11:12	MOCH
7280	R	MOCH	05-MN-005	RBNU	2003	RMB	11:12	MOCH
7280	R	MOCH	05-MN-005	RBNU	2003	RMB	11:12	RBNU
7280	R	MOCH	05-MN-005	RBNU	2003	RMB	11:12	BCCH
7280	R	MOCH	05-MN-005	RBNU	2003	RMB	11:12	none
7280	R	MOCH	05-MN-005	RBNU	2003	RMB	11:12	none
7280	R	MOCH	05-MN-005	RBNU	2003	RMB	11:12	BCCH
7280	R	MOCH	05-MN-005	RBNU	2003	RMB	11:12	none
7020	R	MOCH	05-ARN-00	MOCH	2003	MRB	10:21	BCCH
7020	R	MOCH	05-ARN-00	MOCH	2003	MRB	10:21	BCCH
7020	R	MOCH	05-ARN-00	MOCH	2003	MRB	10:21	RBNU
7020	R	MOCH	05-ARN-00	MOCH	2003	MRB	10:21	none
7020	R	MOCH	05-ARN-00	MOCH	2003	MRB	10:21	MOCH
7020	R	MOCH	05-ARN-00	MOCH	2003	MRB	10:21	none
7020	R	MOCH	05-ARN-00	MOCH	2003	MRB	10:21	MOCH
7020	R	MOCH	05-ARN-00	MOCH	2003	MRB	10:21	RBNU
NEW	R	MOCH	05-MN-008	none	none	RMB	10:55	MOCH
NEW	R	MOCH	05-MN-008	none	none	RMB	10:55	MOCH
NEW	R	MOCH	05-MN-008	none	none	RMB	10:55	MOCH
NEW	R	MOCH	05-MN-008	none	none	RMB	10:55	BCCH
NEW	R	MOCH	05-MN-008	none	none	RMB	10:55	RBNU
NEW	R	MOCH	05-MN-008	none	none	RMB	10:55	BCCH
NEW	R	MOCH	05-MN-008	none	none	RMB	10:55	none
NEW	R	MOCH	05-MN-008	none	none	RMB	10:55	BCCH
NEW	R	MOCH	05-MN-008	none	none	RMB	10:55	BCCH
NEW	R	MOCH	05-MN-008	none	none	RMB	10:55	MOCH
NEW	R	MOCH	05-MN-008	none	none	RMB	10:55	RBNU
NEW	R	MOCH	05-MN-008	none	none	RMB	10:55	RBNU
NEW	R	MOCH	05-MN-008	none	none	RMB	10:55	RBNU
NEW	R	MOCH	05-MN-008	none	none	RMB	10:55	BCCH
NEW	R	MOCH	05-MN-008	none	none	RMB	10:55	BCCH
NEW	R	MOCH	05-MN-008	none	none	RMB	10:55	RBNU
NEW	R	MOCH	05-MN-008	none	none	RMB	10:55	RBNU
NEW	R	MOCH	05-MN-008	none	none	RMB	10:55	RBNU
7040	R	MOCH	05-PAR-00	RBNU	2004	BMR	8:34	MOCH
7040	R	MOCH	05-PAR-00	RBNU	2004	BMR	8:34	MOCH

7040	R	MOCH	05-PAR-00	RBNU	2004	BMR	8:34	MOCH
7040	R	MOCH	05-PAR-00	RBNU	2004	BMR	8:34	MOCH
7040	R	MOCH	05-PAR-00	RBNU	2004	BMR	8:34	MOCH
7040	R	MOCH	05-PAR-00	RBNU	2004	BMR	8:34	MOCH
7040	R	MOCH	05-PAR-00	RBNU	2004	BMR	8:34	MOCH
7040	R	MOCH	05-PAR-00	RBNU	2004	BMR	8:34	MOCH
7040	R	MOCH	05-PAR-00	RBNU	2004	BMR	8:34	MOCH
7040	R	MOCH	05-PAR-00	RBNU	2004	BMR	8:34	RBNU
7040	R	MOCH	05-PAR-00	RBNU	2004	BMR	8:34	RBNU
7670	R	MOCH	05-ARN-01	MOCH	2004	BRM	8:45	MOCH
7670	R	MOCH	05-ARN-01	MOCH	2004	BRM	8:45	MOCH
7670	R	MOCH	05-ARN-01	MOCH	2004	BRM	8:45	MOCH
7670	R	MOCH	05-ARN-01	MOCH	2004	BRM	8:45	MOCH
7670	R	MOCH	05-ARN-01	MOCH	2004	BRM	8:45	MOCH
7670	R	MOCH	05-ARN-01	MOCH	2004	BRM	8:45	MOCH
7670	R	MOCH	05-ARN-01	MOCH	2004	BRM	8:45	MOCH
7670	R	MOCH	05-ARN-01	MOCH	2004	BRM	8:45	BCCH
7670	R	MOCH	05-ARN-01	MOCH	2004	BRM	8:45	RBNU
70502/785	R	MOCH	05-ARN-01	RBNU/MO(	2004	RMB	11:00	MOCH
70502/785	R	MOCH	05-ARN-01	RBNU/MO(	2004	RMB	11:00	BCCH
70502/785	R	MOCH	05-ARN-01	RBNU/MO(	2004	RMB	11:00	MOCH
70502/785	R	MOCH	05-ARN-01	RBNU/MO(	2004	RMB	11:00	RBNU
70502/785	R	MOCH	05-ARN-01	RBNU/MO(	2004	RMB	11:00	MOCH
70502/785	R	MOCH	05-ARN-01	RBNU/MO(	2004	RMB	11:00	RBNU
70502/785	R	MOCH	05-ARN-01	RBNU/MO(	2004	RMB	11:00	RBNU
70734	R	MOCH	05-ARN-00	DOWO	2004	MRB	8:16	BCCH
70734	R	MOCH	05-ARN-00	DOWO	2004	MRB	8:16	BCCH
70734	R	MOCH	05-ARN-00	DOWO	2004	MRB	8:16	RBNU
70734	R	MOCH	05-ARN-00	DOWO	2004	MRB	8:16	MOCH
70734	R	MOCH	05-ARN-00	DOWO	2004	MRB	8:16	MOCH
70734	R	MOCH	05-ARN-00	DOWO	2004	MRB	8:16	BCCH
70734	R	MOCH	05-ARN-00	DOWO	2004	MRB	8:16	none
70110	R	MOCH	05-CW-01(	DOWO	2004	MBR	13:57	MOCH
70110	R	MOCH	05-CW-01(	DOWO	2004	MBR	13:57	BCCH
70110	R	MOCH	05-CW-01(	DOWO	2004	MBR	13:57	RBNU
70110	R	MOCH	05-CW-01(	DOWO	2004	MBR	13:57	MOCH
70110	R	MOCH	05-CW-01(	DOWO	2004	MBR	13:57	RBNU
70110	R	MOCH	05-CW-01(	DOWO	2004	MBR	13:57	RBNU
70110	R	MOCH	05-CW-01(	DOWO	2004	MBR	13:57	none
70110	R	MOCH	05-CW-01(	DOWO	2004	MBR	13:57	none
7744	R	MOCH	05-MN-012	MOCH	2004	BMR	8:37	MOCH
7744	R	MOCH	05-MN-012	MOCH	2004	BMR	8:37	MOCH
7744	R	MOCH	05-MN-012	MOCH	2004	BMR	8:37	RBNU
7744	R	MOCH	05-MN-012	MOCH	2004	BMR	8:37	RBNU

7744	R	MOCH	05-MN-012	MOCH	2004	BMR	8:37	BCCH
7744	R	MOCH	05-MN-012	MOCH	2004	BMR	8:37	BCCH
7744	R	MOCH	05-MN-012	MOCH	2004	BMR	8:37	RBNU
7744	R	MOCH	05-MN-012	MOCH	2004	BMR	8:37	RBNU
7744	R	MOCH	05-MN-012	MOCH	2004	BMR	8:37	none
7825	R	MOCH	05-ARN-00	BCCH	2004	RMB	10:09	MOCH
7545	R	MOCH		RBNU	2004	RMB	10:09	MOCH
NEW	R	MOCH	05-ARN-012			RMB	10:09	MOCH
7825	R	MOCH	05-ARN-00	BCCH	2004	RMB	10:09	MOCH
7545	R	MOCH		RBNU	2004	RMB	10:09	MOCH
NEW	R	MOCH	05-ARN-012			RMB	10:09	MOCH
7825	R	MOCH	05-ARN-00	BCCH	2004	RMB	10:09	none
7545	R	MOCH		RBNU	2004	RMB	10:09	none
NEW	R	MOCH	05-ARN-012			RMB	10:09	none
7825	R	MOCH	05-ARN-00	BCCH	2004	RMB	10:09	BCCH
7545	R	MOCH		RBNU	2004	RMB	10:09	BCCH
NEW	R	MOCH	05-ARN-012			RMB	10:09	BCCH
NEW	R	MOCH	05-ARN-014			RBM	14:56	BCCH
NEW	R	MOCH	05-ARN-014			RBM	14:56	MOCH
NEW	R	MOCH	05-ARN-014			RBM	14:56	MOCH
NEW	R	MOCH	05-ARN-014			RBM	14:56	BCCH
NEW	R	MOCH	05-ARN-014			RBM	14:56	BCCH
NEW	R	MOCH	05-ARN-014			RBM	14:56	RBNU
NEW	R	MOCH	05-ARN-014			RBM	14:56	BCCH
NEW	R	MOCH	05-ARN-014			RBM	14:56	MOCH
NEW	R	MOCH	05-ARN-014			RBM	14:56	MOCH
NEW	R	MOCH	05-ARN-014			RBM	14:56	MOCH
NEW	R	MOCH	05-ARN-014			RBM	14:56	RBNU
7280	R	MOCH	05-MN-005	RBNU	2003	RMB	8:47	BCCH
7280	R	MOCH	05-MN-005	RBNU	2003	RMB	8:47	MOCH
7280	R	MOCH	05-MN-005	RBNU	2003	RMB	8:47	BCCH
7280	R	MOCH	05-MN-005	RBNU	2003	RMB	8:47	BCCH
7280	R	MOCH	05-MN-005	RBNU	2003	RMB	8:47	MOCH
7280	R	MOCH	05-MN-005	RBNU	2003	RMB	8:47	BCCH
7280	R	MOCH	05-MN-005	RBNU	2003	RMB	8:47	BCCH
7280	R	MOCH	05-MN-005	RBNU	2003	RMB	8:47	none
7020	R	MOCH	05-ARN-007			RMB	11:43	none
7512	R	MOCH		RBNU	2004	BMR	8:38	MOCH
7512	R	MOCH		RBNU	2004	BMR	8:38	BCCH
7512	R	MOCH		RBNU	2004	BMR	8:38	RBNU
7512	R	MOCH		RBNU	2004	BMR	8:38	RBNU
7512	R	MOCH		RBNU	2004	BMR	8:38	RBNU
7512	R	MOCH		RBNU	2004	BMR	8:38	RBNU
7512	R	MOCH		RBNU	2004	BMR	8:38	RBNU

7482	R	MOCH	05-MN-015	MOCH	2003	RMB	9:19	MOCH
7482	R	MOCH	05-MN-015	MOCH	2003	RMB	9:19	BCCH
7482	R	MOCH	05-MN-015	MOCH	2003	RMB	9:19	MOCH
7482	R	MOCH	05-MN-015	MOCH	2003	RMB	9:19	MOCH
7482	R	MOCH	05-MN-015	MOCH	2003	RMB	9:19	BCCH
7482	R	MOCH	05-MN-015	MOCH	2003	RMB	9:19	MOCH
7482	R	MOCH	05-MN-015	MOCH	2003	RMB	9:19	MOCH
7482	R	MOCH	05-MN-015	MOCH	2003	RMB	9:19	BCCH
7482	R	MOCH	05-MN-015	MOCH	2003	RMB	9:19	BCCH
7482	R	MOCH	05-MN-015	MOCH	2003	RMB	9:19	BCCH
7482	R	MOCH	05-MN-015	MOCH	2003	RMB	9:19	BCCH
7482	R	MOCH	05-MN-015	MOCH	2003	RMB	9:19	BCCH
7482	R	MOCH	05-MN-015	MOCH	2003	RMB	9:19	RBNU
7482	R	MOCH	05-MN-015	MOCH	2003	RMB	9:19	RBNU
7482	R	MOCH	05-MN-015	MOCH	2003	RMB	9:19	BCCH
70738	R	MOCH	05-ARN-01	MOCH	2004	RBM	9:44	BCCH
70738	R	MOCH	05-ARN-01	MOCH	2004	RBM	9:44	BCCH
70738	R	MOCH	05-ARN-01	MOCH	2004	RBM	9:44	BCCH
70738	R	MOCH	05-ARN-01	MOCH	2004	RBM	9:44	BCCH
70738	R	MOCH	05-ARN-01	MOCH	2004	RBM	9:44	BCCH
70738	R	MOCH	05-ARN-01	MOCH	2004	RBM	9:44	BCCH
70738	R	MOCH	05-ARN-01	MOCH	2004	RBM	9:44	MOCH
70738	R	MOCH	05-ARN-01	MOCH	2004	RBM	9:44	MOCH
70738	R	MOCH	05-ARN-01	MOCH	2004	RBM	9:44	RBNU
70738	R	MOCH	05-ARN-01	MOCH	2004	RBM	9:44	MOCH
70738	R	MOCH	05-ARN-01	MOCH	2004	RBM	9:44	MOCH
70738	R	MOCH	05-ARN-01	MOCH	2004	RBM	9:44	RBNU
70738	R	MOCH	05-ARN-01	MOCH	2004	RBM	9:44	RBNU
70738	R	MOCH	05-ARN-01	MOCH	2004	RBM	9:44	BCCH
70738	R	MOCH	05-ARN-01	MOCH	2004	RBM	9:44	RBNU
70738	R	MOCH	05-ARN-01	MOCH	2004	RBM	9:44	BCCH
70738	R	MOCH	05-ARN-01	MOCH	2004	RBM	9:44	RBNU
70738	R	MOCH	05-ARN-01	MOCH	2004	RBM	9:44	MOCH
70738	R	MOCH	05-ARN-01	MOCH	2004	RBM	9:44	RBNU
70738	R	MOCH	05-ARN-01	MOCH	2004	RBM	9:44	BCCH
7117	R	MOCH	05-CW-017	TRES	2004	RMB	10:34	BCCH
7117	R	MOCH	05-CW-017	TRES	2004	RMB	10:34	BCCH
7117	R	MOCH	05-CW-017	TRES	2004	RMB	10:34	MOCH
7117	R	MOCH	05-CW-017	TRES	2004	RMB	10:34	MOCH
7117	R	MOCH	05-CW-017	TRES	2004	RMB	10:34	MOCH
7117	R	MOCH	05-CW-017	TRES	2004	RMB	10:34	BCCH
7117	R	MOCH	05-CW-017	TRES	2004	RMB	10:34	BCCH
7117	R	MOCH	05-CW-017	TRES	2004	RMB	10:34	MOCH
7117	R	MOCH	05-CW-017	TRES	2004	RMB	10:34	BCCH

7117	R	MOCH	05-CW-017	TRES	2004	RMB	10:34	BCCH
7748	R	MOCH	05-PAR-03	MOCH	1999	RBM	11:24	none
7972	R	MOCH	05-PAR-04	RBNU	2004	BMR	6:28	none
7972	R	MOCH	05-PAR-04	RBNU	2004	BMR	6:28	MOCH
7972	R	MOCH	05-PAR-04	RBNU	2004	BMR	6:28	MOCH
7972	R	MOCH	05-PAR-04	RBNU	2004	BMR	6:28	BCCH
7972	R	MOCH	05-PAR-04	RBNU	2004	BMR	6:28	RBNU
7972	R	MOCH	05-PAR-04	RBNU	2004	BMR	6:28	RBNU
7972	R	MOCH	05-PAR-04	RBNU	2004	BMR	6:28	RBNU
7972	R	MOCH	05-PAR-04	RBNU	2004	BMR	6:28	RBNU
7972	R	MOCH	05-PAR-04	RBNU	2004	BMR	6:28	RBNU
7972	R	MOCH	05-PAR-04	RBNU	2004	BMR	6:28	RBNU
7972	R	MOCH	05-PAR-04	RBNU	2004	BMR	6:28	none
7972	R	MOCH	05-PAR-04	RBNU	2004	BMR	6:28	MOCH
7408	R	MOCH	05-PAR-04	RBNU	2004	BRM	7:49	MOCH
7408	R	MOCH	05-PAR-04	RBNU	2004	BRM	7:49	MOCH
7408	R	MOCH	05-PAR-04	RBNU	2004	BRM	7:49	MOCH
7408	R	MOCH	05-PAR-04	RBNU	2004	BRM	7:49	MOCH
7408	R	MOCH	05-PAR-04	RBNU	2004	BRM	7:49	MOCH
7408	R	MOCH	05-PAR-04	RBNU	2004	BRM	7:49	MOCH
7408	R	MOCH	05-PAR-04	RBNU	2004	BRM	7:49	MOCH
7408	R	MOCH	05-PAR-04	RBNU	2004	BRM	7:49	MOCH
70133	R	MOCH	05-ARN-02	MOCH	2004	BRM	9:50	BCCH
70133	R	MOCH	05-ARN-02	MOCH	2004	BRM	9:50	RBNU
70133	R	MOCH	05-ARN-02	MOCH	2004	BRM	9:50	RBNU
70133	R	MOCH	05-ARN-02	MOCH	2004	BRM	9:50	MOCH
70133	R	MOCH	05-ARN-02	MOCH	2004	BRM	9:50	MOCH
70133	R	MOCH	05-ARN-02	MOCH	2004	BRM	9:50	MOCH
70133	R	MOCH	05-ARN-02	MOCH	2004	BRM	9:50	MOCH
70102	R	MOCH	05-ARN-02	BCCH	2004	BRM	9:10	MOCH
70102	R	MOCH	05-ARN-02	BCCH	2004	BRM	9:10	MOCH
70102	R	MOCH	05-ARN-02	BCCH	2004	BRM	9:10	MOCH
70102	R	MOCH	05-ARN-02	BCCH	2004	BRM	9:10	MOCH
70102	R	MOCH	05-ARN-02	BCCH	2004	BRM	9:10	MOCH
70102	R	MOCH	05-ARN-02	BCCH	2004	BRM	9:10	BCCH
70102	R	MOCH	05-ARN-02	BCCH	2004	BRM	9:10	BCCH
70102	R	MOCH	05-ARN-02	BCCH	2004	BRM	9:10	RBNU
7839	R	MOCH	05-PAR-05	MOCH	2004	BRM	6:57	BCCH
7839	R	MOCH	05-PAR-05	MOCH	2004	BRM	6:57	BCCH
7595	R	MOCH	05-MN-025	RBNU	2004	MRB	7:28	BCCH
7595	R	MOCH	05-MN-025	RBNU	2004	MRB	7:28	BCCH
7595	R	MOCH	05-MN-025	RBNU	2004	MRB	7:28	MOCH
7595	R	MOCH	05-MN-025	RBNU	2004	MRB	7:28	MOCH
7595	R	MOCH	05-MN-025	RBNU	2004	MRB	7:28	MOCH

	7595	R	MOCH	05-MN-025	RBNU		2004	MRB	7:28	MOCH
	7595	R	MOCH	05-MN-025	RBNU		2004	MRB	7:28	MOCH
	7595	R	MOCH	05-MN-025	RBNU		2004	MRB	7:28	none
	7595	R	MOCH	05-MN-025	RBNU		2004	MRB	7:28	none
	7595	R	MOCH	05-MN-025	RBNU		2004	MRB	7:28	RBNU
	7595	R	MOCH	05-MN-025	RBNU		2004	MRB	7:28	BCCH
	7595	R	MOCH	05-MN-025	RBNU		2004	MRB	7:28	BCCH
	7595	R	MOCH	05-MN-025	RBNU		2004	MRB	7:28	none
	7595	R	MOCH	05-MN-025	RBNU		2004	MRB	7:28	RBNU
	7595	R	MOCH	05-MN-025	RBNU		2004	MRB	7:28	none
?		R	MOCH	05-MN-026	RBNU	?		MBR	15:27	RBNU
?		R	MOCH	05-MN-026	RBNU	?		MBR	15:27	RBNU
	7440	R	MOCH	05-ARN-02	MOCH		2002	MRB	11:48	RBNU
	7440	R	MOCH	05-ARN-02	MOCH		2002	MRB	11:48	MOCH
	7440	R	MOCH	05-ARN-02	MOCH		2002	MRB	11:48	RBNU
	7440	R	MOCH	05-ARN-02	MOCH		2002	MRB	11:48	RBNU
	7440	R	MOCH	05-ARN-02	MOCH		2002	MRB	11:48	MOCH
	7440	R	MOCH	05-ARN-02	MOCH		2002	MRB	11:48	RBNU
	7440	R	MOCH	05-ARN-02	MOCH		2002	MRB	11:48	BCCH
	7440	R	MOCH	05-ARN-02	MOCH		2002	MRB	11:48	BCCH
	7440	R	MOCH	05-ARN-02	MOCH		2002	MRB	11:48	BCCH
	70703	R	MOCH	05-PAR-06	RBNU		2004	RBM	9:50	none
	70703	R	MOCH	05-PAR-06	RBNU		2004	RBM	9:50	RBNU
	70703	R	MOCH	05-PAR-06	RBNU		2004	RBM	9:50	MOCH
	70703	R	MOCH	05-PAR-06	RBNU		2004	RBM	9:50	RBNU
	70703	R	MOCH	05-PAR-06	RBNU		2004	RBM	9:50	RBNU
	70703	R	MOCH	05-PAR-06	RBNU		2004	RBM	9:50	RBNU
	70703	R	MOCH	05-PAR-06	RBNU		2004	RBM	9:50	RBNU
	70703	R	MOCH	05-PAR-06	RBNU		2004	RBM	9:50	BCCH
	70703	R	MOCH	05-PAR-06	RBNU		2004	RBM	9:50	MOCH
	70703	R	MOCH	05-PAR-06	RBNU		2004	RBM	9:50	MOCH
	70703	R	MOCH	05-PAR-06	RBNU		2004	RBM	9:50	MOCH
	70703	R	MOCH	05-PAR-06	RBNU		2004	RBM	9:50	BCCH
	7878	R	MOCH	05-PAR-06	DOWO		2004	BMR	15:43	MOCH
	7878	R	MOCH	05-PAR-06	DOWO		2004	BMR	15:43	BCCH
	7878	R	MOCH	05-PAR-06	DOWO		2004	BMR	15:43	BCCH
	7878	R	MOCH	05-PAR-06	DOWO		2004	BMR	15:43	MOCH
	7878	R	MOCH	05-PAR-06	DOWO		2004	BMR	15:43	RBNU
	7878	R	MOCH	05-PAR-06	DOWO		2004	BMR	15:43	MOCH
	7878	R	MOCH	05-PAR-06	DOWO		2004	BMR	15:43	RBNU
	7878	R	MOCH	05-PAR-06	DOWO		2004	BMR	15:43	BCCH
	7878	R	MOCH	05-PAR-06	DOWO		2004	BMR	15:43	MOCH
	7656	R	MOCH	05-MN-013	MOCH		2004	BMR	13:00	RBNU
	7825	R	MOCH	05-ARN-006				RMB	12:22	MOCH

	R	MOCH	05-ARN-013			RMB	12:22	MOCH
7825	R	MOCH	05-ARN-006			RMB	12:22	BCCH
7825	R	MOCH	05-ARN-006			RMB	12:22	MOCH
7825	R	MOCH	05-ARN-006			RMB	12:22	MOCH
	R	MOCH	05-ARN-013			RMB	12:22	BCCH
	R	MOCH	05-ARN-013			RMB	12:22	MOCH
	R	MOCH	05-ARN-013			RMB	12:22	MOCH
7825	R	MOCH	05-ARN-006			RMB	12:22	RBNU
	R	MOCH	05-ARN-013			RMB	12:22	RBNU
7825	R	MOCH	05-ARN-006			RMB	12:22	MOCH
	R	MOCH	05-ARN-013			RMB	12:22	MOCH
7825	R	MOCH	05-ARN-006			RMB	12:22	RBNU
	R	MOCH	05-ARN-013			RMB	12:22	RBNU
7280	R	MOCH	05-MN-005	RBNU	2003	RBM	14:27	MOCH
7280	R	MOCH	05-MN-005	RBNU	2003	RBM	14:27	MOCH
7280	R	MOCH	05-MN-005	RBNU	2003	RBM	14:27	MOCH
7280	R	MOCH	05-MN-005	RBNU	2003	RBM	14:27	MOCH
7280	R	MOCH	05-MN-005	RBNU	2003	RBM	14:27	BCCH
7280	R	MOCH	05-MN-005	RBNU	2003	RBM	14:27	MOCH
7280	R	MOCH	05-MN-005	RBNU	2003	RBM	14:27	MOCH
7280	R	MOCH	05-MN-005	RBNU	2003	RBM	14:27	MOCH
7280	R	MOCH	05-MN-005	RBNU	2003	RBM	14:27	BCCH
7280	R	MOCH	05-MN-005	RBNU	2003	RBM	14:27	MOCH
7280	R	MOCH	05-MN-005	RBNU	2003	RBM	14:27	RBNU
7280	R	MOCH	05-MN-005	RBNU	2003	RBM	14:27	RBNU
7280	R	MOCH	05-MN-005	RBNU	2003	RBM	14:27	BCCH
7280	R	MOCH	05-MN-005	RBNU	2003	RBM	14:27	MOCH
7117	R	MOCH	05-CW-017			BRM	8:34	BCCH
7020	R	MOCH	05-ARN-00	MOCH	2003	RMB	10:14	RBNU
7020	R	MOCH	05-ARN-00	MOCH	2003	RMB	10:14	RBNU
7020	R	MOCH	05-ARN-00	MOCH	2003	RMB	10:14	MOCH
7020	R	MOCH	05-ARN-00	MOCH	2003	RMB	10:14	RBNU
7020	R	MOCH	05-ARN-00	MOCH	2003	RMB	10:14	BCCH
7595	R	MOCH	05-MN-025	RBNU	2004	MRB	9:26	BCCH
7595	R	MOCH	05-MN-025	RBNU	2004	MRB	9:26	RBNU
7595	R	MOCH	05-MN-025	RBNU	2004	MRB	9:26	RBNU
7595	R	MOCH	05-MN-025	RBNU	2004	MRB	9:26	BCCH
7595	R	MOCH	05-MN-025	RBNU	2004	MRB	9:26	MOCH
7595	R	MOCH	05-MN-025	RBNU	2004	MRB	9:26	RBNU
7595	R	MOCH	05-MN-025	RBNU	2004	MRB	9:26	RBNU
7595	R	MOCH	05-MN-025	RBNU	2004	MRB	9:26	BCCH
7595	R	MOCH	05-MN-025	RBNU	2004	MRB	9:26	BCCH
7595	R	MOCH	05-MN-025	RBNU	2004	MRB	9:26	RBNU
7595	R	MOCH	05-MN-025	RBNU	2004	MRB	9:26	RBNU

7595	R	MOCH	05-MN-025	RBNU	2004	MRB	9:26	RBNU
7595	R	MOCH	05-MN-025	RBNU	2004	MRB	9:26	BCCH
7595	R	MOCH	05-MN-025	RBNU	2004	MRB	9:26	MOCH
7595	R	MOCH	05-MN-025	RBNU	2004	MRB	9:26	MOCH
7595	R	MOCH	05-MN-025	RBNU	2004	MRB	9:26	MOCH
7595	R	MOCH	05-MN-025	RBNU	2004	MRB	9:26	RBNU
7595	R	MOCH	05-MN-025	RBNU	2004	MRB	9:26	MOCH
7595	R	MOCH	05-MN-025	RBNU	2004	MRB	9:26	RBNU
7595	R	MOCH	05-MN-025	RBNU	2004	MRB	9:26	BCCH
7595	R	MOCH	05-MN-025	RBNU	2004	MRB	9:26	MOCH
7595	R	MOCH	05-MN-025	RBNU	2004	MRB	9:26	BCCH
7595	R	MOCH	05-MN-025	RBNU	2004	MRB	9:26	RBNU
7595	R	MOCH	05-MN-025	RBNU	2004	MRB	9:26	BCCH
NEW	R	MOCH	05-MN-008	none	none	RMB	13:41	RBNU
NEW	R	MOCH	05-MN-008	none	none	RMB	13:41	MOCH
NEW	R	MOCH	05-MN-008	none	none	RMB	13:41	MOCH
NEW	R	MOCH	05-MN-008	none	none	RMB	13:41	BCCH
NEW	R	MOCH	05-ARN-03	none	none	RBM	14:11	MOCH
70502	R	MOCH	05-ARN-018			RMB	16:09	RBNU
70502	R	MOCH	05-ARN-018			RMB	16:09	MOCH
7855	R	MOCH	05-RV-054			RMB	16:09	MOCH
7855	R	MOCH	05-RV-054			RMB	16:09	MOCH
70502	R	MOCH	05-ARN-018			RMB	16:09	RBNU
7855	R	MOCH	05-RV-054			RMB	16:09	MOCH
70502	R	MOCH	05-ARN-018			RMB	16:09	MOCH
7855	R	MOCH	05-RV-054			RMB	16:09	MOCH
7974	R	MOCH	05-PAR-09	RBNU	2004	RBM	11:08	BCCH
7974	R	MOCH	05-PAR-09	RBNU	2004	RBM	11:08	BCCH
7974	R	MOCH	05-PAR-09	RBNU	2004	RBM	11:08	BCCH
7974	R	MOCH	05-PAR-09	RBNU	2004	RBM	11:08	MOCH
7974	R	MOCH	05-PAR-09	RBNU	2004	RBM	11:08	MOCH
7974	R	MOCH	05-PAR-09	RBNU	2004	RBM	11:08	none
7974	R	MOCH	05-PAR-09	RBNU	2004	RBM	11:08	none
7974	R	MOCH	05-PAR-09	RBNU	2004	RBM	11:08	MOCH
7974	R	MOCH	05-PAR-09	RBNU	2004	RBM	11:08	MOCH
7974	R	MOCH	05-PAR-09	RBNU	2004	RBM	11:08	MOCH
7974	R	MOCH	05-PAR-09	RBNU	2004	RBM	11:08	RBNU
70413	R	MOCH	06-ARN-01	RBNU	2005	MBR	12:02	BCCH
70413	R	MOCH	06-ARN-01	RBNU	2005	MBR	12:02	MOCH
70413	R	MOCH	06-ARN-01	RBNU	2005	MBR	12:02	MOCH
70413	R	MOCH	06-ARN-01	RBNU	2005	MBR	12:02	MOCH
70413	R	MOCH	06-ARN-01	RBNU	2005	MBR	12:02	RBNU
70413	R	MOCH	06-ARN-01	RBNU	2005	MBR	12:02	RBNU
70413	R	MOCH	06-ARN-01	RBNU	2005	MBR	12:02	MOCH

	70102	R	MOCH	06-LD-001	MOCH	2005	RBM	9:34	RBNU
	7280	R	MOCH	06-LD-013	MOCH	2005	MRB	10:30	BCCH
	7280	R	MOCH	06-LD-013	MOCH	2005	MRB	10:30	BCCH
	7280	R	MOCH	06-LD-013	MOCH	2005	MRB	10:30	BCCH
	7280	R	MOCH	06-LD-013	MOCH	2005	MRB	10:30	BCCH
	7280	R	MOCH	06-LD-013	MOCH	2005	MRB	10:30	RBNU
	7280	R	MOCH	06-LD-013	MOCH	2005	MRB	10:30	MOCH
	7744	R	MOCH	06-ARN-01	MOCH	2005	RMB	8:54	BCCH
	7744	R	MOCH	06-ARN-01	MOCH	2005	RMB	8:54	BCCH
	7744	R	MOCH	06-ARN-01	MOCH	2005	RMB	8:54	MOCH
	7744	R	MOCH	06-ARN-01	MOCH	2005	RMB	8:54	MOCH
	7744	R	MOCH	06-ARN-01	MOCH	2005	RMB	8:54	RBNU
	7744	R	MOCH	06-ARN-01	MOCH	2005	RMB	8:54	MOCH
	7744	R	MOCH	06-ARN-01	MOCH	2005	RMB	8:54	BCCH
	7852	R	MOCH	06-SLD-01	MOCH	2005	MBR	8:34	BCCH
	7852	R	MOCH	06-SLD-01	MOCH	2005	MBR	8:34	MOCH
	7852	R	MOCH	06-SLD-01	MOCH	2005	MBR	8:34	BCCH
	7852	R	MOCH	06-SLD-01	MOCH	2005	MBR	8:34	RBNU
	7440	R	MOCH	06-ARN-01	MOCH	2005	MBR	7:51	MOCH
	7440	R	MOCH	06-ARN-01	MOCH	2005	MBR	7:51	BCCH
	7440	R	MOCH	06-ARN-01	MOCH	2005	MBR	7:51	RBNU
	CHECK	R	MOCH	06-CK-049	MOCH	CHECK	RBM	12:55	RBNU
	CHECK	R	MOCH	06-CK-049	MOCH	CHECK	RBM	12:55	BCCH
	CHECK	R	MOCH	06-CK-049	MOCH	CHECK	RBM	12:55	MOCH
	70413R	R	MOCH				MBR	10:44	RBNU
	70413R	R	MOCH				MBR	10:44	BCCH
	70413R	R	MOCH				MBR	10:44	MOCH
	70413R	R	MOCH				MBR	10:44	BCCH
	70413R	R	MOCH				MBR	10:44	MOCH
	70413R	R	MOCH				MBR	10:44	RBNU
	70413R	R	MOCH				MBR	10:44	
IB	7406	R	MOCH	07-LP-015	GLSA		BMR	10:54	BCCH
IB	7406	R	MOCH	07-LP-015	GLSA		BMR	10:54	BCCH
IB	7406	R	MOCH	07-LP-015	GLSA		BMR	10:54	BCCH
IB	7406	R	MOCH	07-LP-015	GLSA		BMR	10:54	BCCH
IB	7406	R	MOCH	07-LP-015	GLSA		BMR	10:54	BCCH
IB	7406	R	MOCH	07-LP-015	GLSA		BMR	10:54	BCCH
IB	7406	R	MOCH	07-LP-015	GLSA		BMR	10:54	BCCH
IB	7406	R	MOCH	07-LP-015	GLSA		BMR	10:54	BCCH
IB	7406	R	MOCH	07-LP-015	GLSA		BMR	10:54	BCCH
IB	7406	R	MOCH	07-LP-015	GLSA		BMR	10:54	BCCH
IB	7406	R	MOCH	07-LP-015	GLSA		BMR	10:54	BCCH
IB	7406	R	MOCH	07-LP-015	GLSA		BMR	10:54	MOCH
IB	7406	R	MOCH	07-LP-015	GLSA		BMR	10:54	MOCH

IB	7406	R	MOCH	07-LP-015	GLSA		BMR	10:54	MOCH
IB	7406	R	MOCH	07-LP-015	GLSA		BMR	10:54	MOCH
IB	7406	R	MOCH	07-LP-015	GLSA		BMR	10:54	BCCH
IB	7406	R	MOCH	07-LP-015	GLSA		BMR	10:54	MOCH
IB	7406	R	MOCH	07-LP-015	GLSA		BMR	10:54	MOCH
IB	7406	R	MOCH	07-LP-015	GLSA		BMR	10:54	MOCH
IB	7406	R	MOCH	07-LP-015	GLSA		BMR	10:54	MOCH
IB	7406	R	MOCH	07-LP-015	GLSA		BMR	10:54	MOCH
IB	7406	R	MOCH	07-LP-015	GLSA		BMR	10:54	MOCH
IB	7406	R	MOCH	07-LP-015	GLSA		BMR	10:54	MOCH
IB	7406	R	MOCH	07-LP-015	GLSA		BMR	10:54	MOCH
IB	7406	R	MOCH	07-LP-015	GLSA		BMR	10:54	MOCH
IB	7406	R	MOCH	07-LP-015	GLSA		BMR	10:54	RBNU
IB	7406	R	MOCH	07-LP-015	GLSA		BMR	10:54	RBNU
IB	7406	R	MOCH	07-LP-015	GLSA		BMR	10:54	RBNU
IB	7406	R	MOCH	07-LP-015	GLSA		BMR	10:54	RBNU
IB	7406	R	MOCH	07-LP-015	GLSA		BMR	10:54	RBNU
IB	7406	R	MOCH	07-LP-015	GLSA		BMR	10:54	RBNU
IB	7406	R	MOCH	07-LP-015	GLSA		BMR	10:54	RBNU
IB	7406	R	MOCH	07-LP-015	GLSA		BMR	10:54	BCCH
IB	7406	R	MOCH	07-LP-015	GLSA		BMR	10:54	RBNU
IB	7406	R	MOCH	07-LP-015	GLSA		BMR	10:54	RBNU
IB	7406	R	MOCH	07-LP-015	GLSA		BMR	10:54	MOCH
IB	7406	R	MOCH	07-LP-015	GLSA		BMR	10:54	RBNU
IB	70721	R	MOCH	07-IB-014	MOCH	2006	BMR	8:13	MOCH
IB	70721	R	MOCH	07-IB-014	MOCH	2006	BMR	8:13	MOCH
IB	70721	R	MOCH	07-IB-014	MOCH	2006	BMR	8:13	MOCH
IB	70721	R	MOCH	07-IB-014	MOCH	2006	BMR	8:13	MOCH
IB	70721	R	MOCH	07-IB-014	MOCH	2006	BMR	8:13	MOCH
IB	70721	R	MOCH	07-IB-014	MOCH	2006	BMR	8:13	BCCH
IB	70721	R	MOCH	07-IB-014	MOCH	2006	BMR	8:13	BCCH
IB	70721	R	MOCH	07-IB-014	MOCH	2006	BMR	8:13	BCCH
IB	70721	R	MOCH	07-IB-014	MOCH	2006	BMR	8:13	BCCH
IB	70721	R	MOCH	07-IB-014	MOCH	2006	BMR	8:13	BCCH
IB	70721	R	MOCH	07-IB-014	MOCH	2006	BMR	8:13	BCCH
IB	70721	R	MOCH	07-IB-014	MOCH	2006	BMR	8:13	BCCH
IB	70721	R	MOCH	07-IB-014	MOCH	2006	BMR	8:13	RBNU
IB	70721	R	MOCH	07-IB-014	MOCH	2006	BMR	8:13	RBNU
IB	70721	R	MOCH	07-IB-014	MOCH	2006	BMR	8:13	RBNU
IB	70721	R	MOCH	07-IB-014	MOCH	2006	BMR	8:13	BCCH
IB	70721	R	MOCH	07-IB-014	MOCH	2006	BMR	8:13	RBNU
IB	70721	R	MOCH	07-IB-014	MOCH	2006	BMR	8:13	RBNU
IB	70721	R	MOCH	07-IB-014	MOCH	2006	BMR	8:13	RBNU
IB	70721	R	MOCH	07-IB-014	MOCH	2006	BMR	8:13	RBNU

IB	70721	R	MOCH	07-IB-014	MOCH	2006	BMR	8:13	RBNU
IB	70721	R	MOCH	07-IB-014	MOCH	2006	BMR	8:13	RBNU
IB	70721	R	MOCH	07-IB-014	MOCH	2006	BMR	8:13	RBNU
IB	70721	R	MOCH	07-IB-014	MOCH	2006	BMR	8:13	RBNU
IB	70721	R	MOCH	07-IB-014	MOCH	2006	BMR	8:13	RBNU
IB	70721	R	MOCH	07-IB-014	MOCH	2006	BMR	8:13	BCCH
IB	70721	R	MOCH	07-IB-014	MOCH	2006	BMR	8:13	MOCH
IB	70721	R	MOCH	07-IB-014	MOCH	2006	BMR	8:13	RBNU
IB	70102	R	MOCH	07-AE-013	MOCH	2006	RBM	9:10	RBNU
IB	70102	R	MOCH	07-AE-013	MOCH	2006	RBM	9:10	RBNU
IB	70102	R	MOCH	07-AE-013	MOCH	2006	RBM	9:10	BCCH
IB	70102	R	MOCH	07-AE-013	MOCH	2006	RBM	9:10	RBNU
IB	70102	R	MOCH	07-AE-013	MOCH	2006	RBM	9:10	BCCH
IB	70102	R	MOCH	07-AE-013	MOCH	2006	RBM	9:10	BCCH
IB	70102	R	MOCH	07-AE-013	MOCH	2006	RBM	9:10	RBNU
IB	70102	R	MOCH	07-AE-013	MOCH	2006	RBM	9:10	BCCH
IB	70102	R	MOCH	07-AE-013	MOCH	2006	RBM	9:10	BCCH
IB	70102	R	MOCH	07-AE-013	MOCH	2006	RBM	9:10	MOCH
IB	70102	R	MOCH	07-AE-013	MOCH	2006	RBM	9:10	BCCH
IB	70102	R	MOCH	07-AE-013	MOCH	2006	RBM	9:10	MOCH
IB	70102	R	MOCH	07-AE-013	MOCH	2006	RBM	9:10	BCCH
IB	70102	R	MOCH	07-AE-013	MOCH	2006	RBM	9:10	BCCH
IB	70102	R	MOCH	07-AE-013	MOCH	2006	RBM	9:10	RBNU
IB	70102	R	MOCH	07-AE-013	MOCH	2006	RBM	9:10	BCCH
IB	70102	R	MOCH	07-AE-013	MOCH	2006	RBM	9:10	RBNU
IB	70102	R	MOCH	07-AE-013	MOCH	2006	RBM	9:10	BCCH
IB	70102	R	MOCH	07-AE-013	MOCH	2006	RBM	9:10	MOCH
IB	70102	R	MOCH	07-AE-013	MOCH	2006	RBM	9:10	MOCH
IB	70102	R	MOCH	07-AE-013	MOCH	2006	RBM	9:10	MOCH
IB	70102	R	MOCH	07-AE-013	MOCH	2006	RBM	9:10	RBNU
IB	70102	R	MOCH	07-AE-013	MOCH	2006	RBM	9:10	RBNU
IB	70102	R	MOCH	07-AE-013	MOCH	2006	RBM	9:10	RBNU
IB	70102	R	MOCH	07-AE-013	MOCH	2006	RBM	9:10	RBNU
IB	70102	R	MOCH	07-AE-013	MOCH	2006	RBM	9:10	BCCH
IB	70102	R	MOCH	07-AE-013	MOCH	2006	RBM	9:10	BCCH
IB	70102	R	MOCH	07-AE-013	MOCH	2006	RBM	9:10	BCCH
IB	70102	R	MOCH	07-AE-013	MOCH	2006	RBM	9:10	MOCH
IB	70102	R	MOCH	07-AE-013	MOCH	2006	RBM	9:10	MOCH
IB	70102	R	MOCH	07-AE-013	MOCH	2006	RBM	9:10	MOCH
IB	70102	R	MOCH	07-AE-013	MOCH	2006	RBM	9:10	MOCH
IB	70102	R	MOCH	07-AE-013	MOCH	2006	RBM	9:10	RBNU
IB	70102	R	MOCH	07-AE-013	MOCH	2006	RBM	9:10	BCCH
IB	70102	R	MOCH	07-AE-013	MOCH	2006	RBM	9:10	MOCH
IB	70122	R	MOCH	08-AE-020	RBNU	2006	BRM	9:15	MOCH

IB	70122	R	MOCH	08-AE-020	RBNU	2006	BRM	9:15	MOCH
IB	70122	R	MOCH	08-AE-020	RBNU	2006	BRM	9:15	MOCH
IB	70122	R	MOCH	08-AE-020	RBNU	2006	BRM	9:15	MOCH
IB	70122	R	MOCH	08-AE-020	RBNU	2006	BRM	9:15	MOCH
IB	70122	R	MOCH	08-AE-020	RBNU	2006	BRM	9:15	MOCH
IB	70122	R	MOCH	08-AE-020	RBNU	2006	BRM	9:15	MOCH
IB	70122	R	MOCH	08-AE-020	RBNU	2006	BRM	9:15	MOCH
IB	70122	R	MOCH	08-AE-020	RBNU	2006	BRM	9:15	RBNU
IB	70122	R	MOCH	08-AE-020	RBNU	2006	BRM	9:15	RBNU
IB	70122	R	MOCH	08-AE-020	RBNU	2006	BRM	9:15	RBNU
IB	70122	R	MOCH	08-AE-020	RBNU	2006	BRM	9:15	RBNU
IB	70122	R	MOCH	08-AE-020	RBNU	2006	BRM	9:15	RBNU
IB	70122	R	MOCH	08-AE-020	RBNU	2006	BRM	9:15	RBNU
IB	70122	R	MOCH	08-AE-020	RBNU	2006	BRM	9:15	RBNU
IB	70122	R	MOCH	08-AE-020	RBNU	2006	BRM	9:15	BCCH
IB	70122	R	MOCH	08-AE-020	RBNU	2006	BRM	9:15	BCCH
IB	70122	R	MOCH	08-AE-020	RBNU	2006	BRM	9:15	RBNU
IB	70122	R	MOCH	08-AE-020	RBNU	2006	BRM	9:15	RBNU
IB	70122	R	MOCH	08-AE-020	RBNU	2006	BRM	9:15	RBNU
IB	70122	R	MOCH	08-AE-020	RBNU	2006	BRM	9:15	RBNU
IB	70122	R	MOCH	08-AE-020	RBNU	2006	BRM	9:15	RBNU
IB	70122	R	MOCH	08-AE-020	RBNU	2006	BRM	9:15	RBNU
IB	70122	R	MOCH	08-AE-020	RBNU	2006	BRM	9:15	MOCH
IB	70122	R	MOCH	08-AE-020	RBNU	2006	BRM	9:15	RBNU
IB	70122	R	MOCH	08-AE-020	RBNU	2006	BRM	9:15	RBNU
IB	70122	R	MOCH	08-AE-020	RBNU	2006	BRM	9:15	RBNU
IB	70122	R	MOCH	08-AE-020	RBNU	2006	BRM	9:15	RBNU
IB	70122	R	MOCH	08-AE-020	RBNU	2006	BRM	9:15	BCCH
IB	70122	R	MOCH	08-AE-020	RBNU	2006	BRM	9:15	BCCH
IB	70122	R	MOCH	08-AE-020	RBNU	2006	BRM	9:15	RBNU
IB	70122	R	MOCH	08-AE-020	RBNU	2006	BRM	9:15	MOCH
AE	70821	R	MOCH	08-HK-005			RBM	1:05 PM	RBNU
AE	70821	R	MOCH	08-HK-005			RBM	1:05 PM	BCCH
AE	70821	R	MOCH	08-HK-005			RBM	1:05 PM	MOCH
AE	70831	R	MOCH	08-AB-039			RMB	8:35 AM	RBNU
AE	70831	R	MOCH	08-AB-039			RMB	8:35 AM	RBNU
AE	70831	R	MOCH	08-AB-039			RMB	8:35 AM	RBNU
AE	70831	R	MOCH	08-AB-039			RMB	8:35 AM	RBNU
AE	70831	R	MOCH	08-AB-039			RMB	8:35 AM	RBNU
AE	70831	R	MOCH	08-AB-039			RMB	8:35 AM	RBNU
AE	70831	R	MOCH	08-AB-039			RMB	8:35 AM	RBNU
AE	70831	R	MOCH	08-AB-039			RMB	8:35 AM	RBNU
AE	70831	R	MOCH	08-AB-039			RMB	8:35 AM	RBNU
AE	70831	R	MOCH	08-AB-039			RMB	8:35 AM	MOCH

AE	70831	R	MOCH	08-AB-039			RMB	8:35 AM	BCCH
AE	70831	R	MOCH	08-AB-039			RMB	8:35 AM	RBNU
AE	70831	R	MOCH	08-AB-039			RMB	8:35 AM	RBNU
AE	70831	R	MOCH	08-AB-039			RMB	8:35 AM	BCCH
AE	70831	R	MOCH	08-AB-039			RMB	8:35 AM	BCCH
AE	70831	R	MOCH	08-AB-039			RMB	8:35 AM	BCCH
AE	70831	R	MOCH	08-AB-039			RMB	8:35 AM	RBNU
AE	70831	R	MOCH	08-AB-039			RMB	8:35 AM	RBNU
AE	70831	R	MOCH	08-AB-039			RMB	8:35 AM	MOCH
AE	70831	R	MOCH	08-AB-039			RMB	8:35 AM	BCCH
AE	70133	R	MOCH	08-ARN-047			RBM	11:19 AM	MOCH
AE	70133	R	MOCH	08-ARN-047			RBM	11:19 AM	MOCH
AE	70133	R	MOCH	08-ARN-047			RBM	11:19 AM	MOCH
AE	70133	R	MOCH	08-ARN-047			RBM	11:19 AM	MOCH
AE	70133	R	MOCH	08-ARN-047			RBM	11:19 AM	MOCH
AE	70133	R	MOCH	08-ARN-047			RBM	11:19 AM	MOCH
AE	70133	R	MOCH	08-ARN-047			RBM	11:19 AM	MOCH
AE	70133	R	MOCH	08-ARN-047			RBM	11:19 AM	MOCH
AE	70133	R	MOCH	08-ARN-047			RBM	11:19 AM	MOCH
AE	70133	R	MOCH	08-ARN-047			RBM	11:19 AM	RBNU
AE	70133	R	MOCH	08-ARN-047			RBM	11:19 AM	RBNU
AE	70133	R	MOCH	08-ARN-047			RBM	11:19 AM	RBNU
AE	70133	R	MOCH	08-ARN-047			RBM	11:19 AM	BCCH
AE	70133	R	MOCH	08-ARN-047			RBM	11:19 AM	RBNU
AE	70133	R	MOCH	08-ARN-047			RBM	11:19 AM	RBNU
AE	70133	R	MOCH	08-ARN-047			RBM	11:19 AM	RBNU
AE	70133	R	MOCH	08-ARN-047			RBM	11:19 AM	RBNU
AE	70133	R	MOCH	08-ARN-047			RBM	11:19 AM	RBNU
AE	70133	R	MOCH	08-ARN-047			RBM	11:19 AM	RBNU
AE	70133	R	MOCH	08-ARN-047			RBM	11:19 AM	BCCH
AE	70133	R	MOCH	08-ARN-047			RBM	11:19 AM	RBNU
AE	70133	R	MOCH	08-ARN-047			RBM	11:19 AM	RBNU
AE	70133	R	MOCH	08-ARN-047			RBM	11:19 AM	BCCH
AE	70133	R	MOCH	08-ARN-047			RBM	11:19 AM	BCCH
AE	70133	R	MOCH	08-ARN-047			RBM	11:19 AM	MOCH
AE	70133	R	MOCH	08-ARN-047			RBM	11:19 AM	MOCH
AE	70133	R	MOCH	08-ARN-047			RBM	11:19 AM	RBNU
AE	70133	R	MOCH	08-ARN-047			RBM	11:19 AM	BCCH
AE	70133	R	MOCH	08-ARN-047			RBM	11:19 AM	MOCH
	7839	R	MOCH	05-PAR-05	MOCH	2004	BRM	10:07	BCCH
	7839	R	MOCH	05-PAR-05	MOCH	2004	BRM	10:07	BCCH
	7839	R	MOCH	05-PAR-05	MOCH	2004	BRM	10:07	MOCH
	7839	R	MOCH	05-PAR-05	MOCH	2004	BRM	10:07	MOCH
	7839	R	MOCH	05-PAR-05	MOCH	2004	BRM	10:07	MOCH

7839	R	MOCH	05-PAR-05	MOCH	2004	BRM	10:07	MOCH
7839	R	MOCH	05-PAR-05	MOCH	2004	BRM	10:07	MOCH
7839	R	MOCH	05-PAR-05	MOCH	2004	BRM	10:07	MOCH
7839	R	MOCH	05-PAR-05	MOCH	2004	BRM	10:07	RBNU
7839	R	MOCH	05-PAR-05	MOCH	2004	BRM	10:07	RBNU
7839	R	MOCH	05-PAR-05	MOCH	2004	BRM	10:07	RBNU
7839	R	MOCH	05-PAR-05	MOCH	2004	BRM	10:07	RBNU
7839	R	MOCH	05-PAR-05	MOCH	2004	BRM	10:07	RBNU
NEW	R	RBNU	05-PAR-05	MOCH	2004	BRM	10:07	BCCH
NEW	R	RBNU	05-PAR-05	MOCH	2004	BRM	10:07	BCCH
NEW	R	RBNU	05-PAR-05	MOCH	2004	BRM	10:07	MOCH
NEW	R	RBNU	05-PAR-05	MOCH	2004	BRM	10:07	MOCH
NEW	R	RBNU	05-PAR-05	MOCH	2004	BRM	10:07	MOCH
NEW	R	RBNU	05-PAR-05	MOCH	2004	BRM	10:07	MOCH
NEW	R	RBNU	05-PAR-05	MOCH	2004	BRM	10:07	MOCH
NEW	R	RBNU	05-PAR-05	MOCH	2004	BRM	10:07	MOCH
NEW	R	RBNU	05-PAR-05	MOCH	2004	BRM	10:07	RBNU
NEW	R	RBNU	05-PAR-05	MOCH	2004	BRM	10:07	RBNU
NEW	R	RBNU	05-PAR-05	MOCH	2004	BRM	10:07	RBNU
NEW	R	RBNU	05-PAR-05	MOCH	2004	BRM	10:07	RBNU
NEW	R	RBNU	05-PAR-05	MOCH	2004	BRM	10:07	RBNU
NEW	R		none	none	none	MBR	14:55	MOCH
NEW	R		none	none	none	MBR	14:55	MOCH
NEW	R		none	none	none	MBR	14:55	MOCH
NEW	R		none	none	none	MBR	14:55	MOCH
NEW	R		none	none	none	MBR	14:55	none
NEW	R		none	none	none	MBR	14:55	MOCH
NEW	R		none	none	none	MBR	14:55	MOCH
NEW	R		none	none	none	MBR	14:55	MOCH
NEW	R		none	none	none	MBR	14:55	none
NEW	R		none	none	none	MBR	14:55	MOCH
NEW	R		none	none	none	MBR	14:55	none
POSS NEW	R	RBNU	none	none	none	RBM	9:57	RBNU
POSS NEW	R	RBNU	none	none	none	RBM	9:57	RBNU
POSS NEW	R	RBNU	none	none	none	RBM	9:57	BCCH
POSS NEW	R	RBNU	none	none	none	RBM	9:57	MOCH
POSS NEW	R	RBNU	none	none	none	RBM	9:57	RBNU
POSS NEW	R	RBNU	none	none	none	RBM	9:57	RBNU
POSS NEW	R	RBNU	none	none	none	RBM	9:57	BCCH
POSS NEW	R	RBNU	none	none	none	RBM	9:57	RBNU
POSS NEW	R	RBNU	none	none	none	RBM	9:57	none
7040	R	RBNU		MOCH	2002	BRM	7:40	BCCH
7040	R	RBNU		MOCH	2002	BRM	7:40	BCCH
7040	R	RBNU		MOCH	2002	BRM	7:40	BCCH

7040	R	RBNU	MOCH	2002	BRM	7:40	BCCH
7040	R	RBNU	MOCH	2002	BRM	7:40	BCCH
7040	R	RBNU	MOCH	2002	BRM	7:40	RBNU
7040	R	RBNU	MOCH	2002	BRM	7:40	BCCH
7040	R	RBNU	MOCH	2002	BRM	7:40	BCCH
7040	R	RBNU	MOCH	2002	BRM	7:40	RBNU
7040	R	RBNU	MOCH	2002	BRM	7:40	BCCH
7040	R	RBNU	MOCH	2002	BRM	7:40	BCCH
7040	R	RBNU	MOCH	2002	BRM	7:40	MOCH
7040	R	RBNU	MOCH	2002	BRM	7:40	MOCH
7040	R	RBNU	MOCH	2002	BRM	7:40	RBNU
7040	R	RBNU	MOCH	2002	BRM	7:40	RBNU
7040	R	RBNU	MOCH	2002	BRM	7:40	RBNU
7040	R	RBNU	MOCH	2002	BRM	7:40	RBNU
7040	R	RBNU	MOCH	2002	BRM	7:40	RBNU
7040	R	RBNU	MOCH	2002	BRM	7:40	RBNU
7040	R	RBNU	MOCH	2002	BRM	7:40	RBNU
7040	R	RBNU	MOCH	2002	BRM	7:40	RBNU
7040	R	RBNU	MOCH	2002	BRM	7:40	RBNU
7040	R	RBNU	MOCH	2002	BRM	7:40	MOCH
7040	R	RBNU	MOCH	2002	BRM	7:40	MOCH
7040	R	RBNU	MOCH	2002	BRM	7:40	MOCH
7040	R	RBNU	MOCH	2002	BRM	7:40	MOCH
70502	R	RBNU	New	2004	BMR	9:07	RBNU
70502	R	RBNU	New	2004	BMR	9:07	RBNU
70502	R	RBNU	New	2004	BMR	9:07	RBNU
70502	R	RBNU	New	2004	BMR	9:07	RBNU
70502	R	RBNU	New	2004	BMR	9:07	RBNU
70502	R	RBNU	New	2004	BMR	9:07	RBNU
70502	R	RBNU	New	2004	BMR	9:07	RBNU
70502	R	RBNU	New	2004	BMR	9:07	RBNU
70502	R	RBNU	New	2004	BMR	9:07	RBNU
70502	R	RBNU	New	2004	BMR	9:07	RBNU
70502	R	RBNU	New	2004	BMR	9:07	RBNU
7512	R	RBNU	MOCH	2003	MBR	7:45	none
7512	R	RBNU	MOCH	2003	MBR	7:45	RBNU
7512	R	RBNU	MOCH	2003	MBR	7:45	RBNU
7512	R	RBNU	MOCH	2003	MBR	7:45	RBNU
7512	R	RBNU	MOCH	2003	MBR	7:45	none
7512	R	RBNU	MOCH	2003	MBR	7:45	none
7512	R	RBNU	MOCH	2003	MBR	7:45	MOCH
7512	R	RBNU	MOCH	2003	MBR	7:45	MOCH
7512	R	RBNU	MOCH	2003	MBR	7:45	BCCH
7512	R	RBNU	MOCH	2003	MBR	7:45	none

7512	R	RBNU		MOCH	2003	MBR	7:45	RBNU
70502	R	RBNU		NEW	2004	BRM	7:54:30	RBNU
70502	R	RBNU		NEW	2004	BRM	7:54:30	RBNU
7579	R	RBNU	05-MN-018	RBNU	2004	RBM	9:44	BCCH
7579	R	RBNU	05-MN-018	RBNU	2004	RBM	9:44	BCCH
7579	R	RBNU	05-MN-018	RBNU	2004	RBM	9:44	BCCH
7579	R	RBNU	05-MN-018	RBNU	2004	RBM	9:44	BCCH
7579	R	RBNU	05-MN-018	RBNU	2004	RBM	9:44	BCCH
7579	R	RBNU	05-MN-018	RBNU	2004	RBM	9:44	BCCH
7579	R	RBNU	05-MN-018	RBNU	2004	RBM	9:44	MOCH
7579	R	RBNU	05-MN-018	RBNU	2004	RBM	9:44	MOCH
7579	R	RBNU	05-MN-018	RBNU	2004	RBM	9:44	RBNU
7579	R	RBNU	05-MN-018	RBNU	2004	RBM	9:44	MOCH
7579	R	RBNU	05-MN-018	RBNU	2004	RBM	9:44	MOCH
7579	R	RBNU	05-MN-018	RBNU	2004	RBM	9:44	RBNU
7579	R	RBNU	05-MN-018	RBNU	2004	RBM	9:44	RBNU
7579	R	RBNU	05-MN-018	RBNU	2004	RBM	9:44	BCCH
7579	R	RBNU	05-MN-018	RBNU	2004	RBM	9:44	RBNU
7579	R	RBNU	05-MN-018	RBNU	2004	RBM	9:44	BCCH
7579	R	RBNU	05-MN-018	RBNU	2004	RBM	9:44	RBNU
7579	R	RBNU	05-MN-018	RBNU	2004	RBM	9:44	MOCH
7579	R	RBNU	05-MN-018	RBNU	2004	RBM	9:44	RBNU
7579	R	RBNU	05-MN-018	RBNU	2004	RBM	9:44	BCCH
7457	R	RBNU	05-CW-025			MBR	6:33	BCCH
7457	R	RBNU	05-CW-025			MBR	6:33	RBNU
7457	R	RBNU	05-CW-025			MBR	6:33	RBNU
7457	R	RBNU	05-CW-025			MBR	6:33	RBNU
7457	R	RBNU	05-CW-025			MBR	6:33	RBNU
7457	R	RBNU	05-CW-025			MBR	6:33	RBNU
7457	R	RBNU	05-CW-025			MBR	6:33	none
7457	R	RBNU	05-CW-025			MBR	6:33	MOCH
7457	R	RBNU	05-CW-025			MBR	6:33	RBNU
7457	R	RBNU	05-CW-025			MBR	6:33	RBNU
7457	R	RBNU	05-CW-025			MBR	6:33	MOCH
7457	R	RBNU	05-CW-025			MBR	6:33	BCCH
	R	RBNU	05-PAR-021			BMR	9:42	RBNU
	R	RBNU	05-PAR-021			BMR	9:42	RBNU
	R	RBNU	05-PAR-021			BMR	9:42	RBNU
	R	RBNU	05-PAR-021			BMR	9:42	RBNU
	R	RBNU	05-PAR-021			BMR	9:42	RBNU
	R	RBNU	05-PAR-021			BMR	9:42	none
	R	RBNU	05-PAR-021			BMR	9:42	BCCH
	R	RBNU	05-PAR-021			BMR	9:42	MOCH
	R	RBNU	05-PAR-021			BMR	9:42	RBNU

	R	RBNU	05-PAR-021			BMR	9:42	MOCH
7815	R	RBNU	05-ARN-031			MRB	12:52	BCCH
7815	R	RBNU	05-ARN-031			MRB	12:52	RBNU
7815	R	RBNU	05-ARN-031			MRB	12:52	RBNU
7815	R	RBNU	05-ARN-031			MRB	12:52	RBNU
7815	R	RBNU	05-ARN-031			MRB	12:52	BCCH
7815	R	RBNU	05-ARN-031			MRB	12:52	BCCH
7815	R	RBNU	05-ARN-031			MRB	12:52	BCCH
7815	R	RBNU	05-ARN-031			MRB	12:52	none
7374	R	RBNU	05-CW-035	RBNU	2004	BMR	15:10	BCCH
7374	R	RBNU	05-CW-035	RBNU	2004	BMR	15:10	BCCH
7374	R	RBNU	05-CW-035	RBNU	2004	BMR	15:10	BCCH
7374	R	RBNU	05-CW-035	RBNU	2004	BMR	15:10	RBNU
7374	R	RBNU	05-CW-035	RBNU	2004	BMR	15:10	RBNU
7374	R	RBNU	05-CW-035	RBNU	2004	BMR	15:10	RBNU
7374	R	RBNU	05-CW-035	RBNU	2004	BMR	15:10	RBNU
7374	R	RBNU	05-CW-035	RBNU	2004	BMR	15:10	RBNU
7374	R	RBNU	05-CW-035	RBNU	2004	BMR	15:10	RBNU
7374	R	RBNU	05-CW-035	RBNU	2004	BMR	15:10	RBNU
7374	R	RBNU	05-CW-035	RBNU	2004	BMR	15:10	none
7374	R	RBNU	05-CW-035	RBNU	2004	BMR	15:10	BCCH
7374	R	RBNU	05-CW-035	RBNU	2004	BMR	15:10	BCCH
7374	R	RBNU	05-CW-035	RBNU	2004	BMR	15:10	RBNU
7374	R	RBNU	05-CW-035	RBNU	2004	BMR	15:10	RBNU
7374	R	RBNU	05-CW-035	RBNU	2004	BMR	15:10	MOCH
7374	R	RBNU	05-CW-035	RBNU	2004	BMR	15:10	MOCH
	R	RBNU	05-ARN-033			RMB	12:22	MOCH
	R	RBNU	05-ARN-033			RMB	12:22	BCCH
	R	RBNU	05-ARN-033			RMB	12:22	MOCH
	R	RBNU	05-ARN-033			RMB	12:22	MOCH
	R	RBNU	05-ARN-033			RMB	12:22	RBNU
	R	RBNU	05-ARN-033			RMB	12:22	MOCH
	R	RBNU	05-ARN-033			RMB	12:22	RBNU
7545	R	RBNU	05-ARN-03	RBNU	2004	BMR	9:32	RBNU
7545	R	RBNU	05-ARN-03	RBNU	2004	BMR	9:32	RBNU
7545	R	RBNU	05-ARN-03	RBNU	2004	BMR	9:32	RBNU
7545	R	RBNU	05-ARN-03	RBNU	2004	BMR	9:32	RBNU
7545	R	RBNU	05-ARN-03	RBNU	2004	BMR	9:32	MOCH
7545	R	RBNU	05-ARN-03	RBNU	2004	BMR	9:32	BCCH
7545	R	RBNU	05-ARN-03	RBNU	2004	BMR	9:32	BCCH
7545	R	RBNU	05-ARN-03	RBNU	2004	BMR	9:32	MOCH
7545	R	RBNU	05-ARN-03	RBNU	2004	BMR	9:32	none
7545	R	RBNU	05-ARN-03	RBNU	2004	BMR	9:32	BCCH
7545	R	RBNU	05-ARN-03	RBNU	2004	BMR	9:32	RBNU

	7545	R	RBNU	05-ARN-03	RBNU	2004	BMR	9:32	MOCH
	7545	R	RBNU	05-ARN-03	RBNU	2004	BMR	9:32	MOCH
	7545	R	RBNU	05-ARN-03	RBNU	2004	BMR	9:32	RBNU
		R	RBNU	06-KS-024			BRM	10:10	BCCH
		R	RBNU	06-KS-024			BRM	10:10	BCCH
		R	RBNU	06-KS-024			BRM	10:10	BCCH
		R	RBNU	06-KS-024			BRM	10:10	BCCH
		R	RBNU	06-KS-024			BRM	10:10	RBNU
		R	RBNU	06-KS-024			BRM	10:10	MOCH
		R	RBNU	06-SLD-049			RMB	9:40	RBNU
		R	RBNU	06-SLD-049			RMB	9:40	RBNU
		R	RBNU	06-SLD-049			RMB	9:40	RBNU
		R	RBNU	06-SLD-049			RMB	9:40	RBNU
		R	RBNU	06-SLD-049			RMB	9:40	MOCH
		R	RBNU	06-SLD-049			RMB	9:40	MOCH
		R	RBNU	06-SLD-049			RMB	9:40	BCCH
		R	RBNU	06-SLD-049			RMB	9:40	MOCH
		R	RBNU	06-SLD-049			RMB	9:40	RBNU
		R	RBNU	06-SLD-049			RMB	9:40	RBNU
		R	RBNU	06-SLD-049			RMB	9:40	MOCH
		R	RBNU	06-SLD-049			RMB	9:40	BCCH
IB&ARN	7974	R	RBNU	07-ARN-01	MOCH	2005	MBR	11:25	RBNU
IB&ARN	7974	R	RBNU	07-ARN-01	MOCH	2005	MBR	11:25	MOCH
IB&ARN	7974	R	RBNU	07-ARN-01	MOCH	2005	MBR	11:25	MOCH
IB&ARN	7974	R	RBNU	07-ARN-01	MOCH	2005	MBR	11:25	MOCH
IB&ARN	7974	R	RBNU	07-ARN-01	MOCH	2005	MBR	11:25	BCCH
IB&ARN	7974	R	RBNU	07-ARN-01	MOCH	2005	MBR	11:25	BCCH
IB&ARN	7974	R	RBNU	07-ARN-01	MOCH	2005	MBR	11:25	MOCH
IB&ARN	7974	R	RBNU	07-ARN-01	MOCH	2005	MBR	11:25	BCCH
IB&ARN	7974	R	RBNU	07-ARN-01	MOCH	2005	MBR	11:25	MOCH
IB&ARN	7974	R	RBNU	07-ARN-01	MOCH	2005	MBR	11:25	MOCH
IB&ARN	7974	R	RBNU	07-ARN-01	MOCH	2005	MBR	11:25	MOCH
IB&ARN	7974	R	RBNU	07-ARN-01	MOCH	2005	MBR	11:25	MOCH
IB&ARN	7974	R	RBNU	07-ARN-01	MOCH	2005	MBR	11:25	MOCH
IB&ARN	7974	R	RBNU	07-ARN-01	MOCH	2005	MBR	11:25	MOCH
IB&ARN	7974	R	RBNU	07-ARN-01	MOCH	2005	MBR	11:25	MOCH
IB&ARN	7974	R	RBNU	07-ARN-01	MOCH	2005	MBR	11:25	BCCH
IB&ARN	7974	R	RBNU	07-ARN-01	MOCH	2005	MBR	11:25	MOCH
IB&ARN	7974	R	RBNU	07-ARN-01	MOCH	2005	MBR	11:25	MOCH
IB&ARN	7974	R	RBNU	07-ARN-01	MOCH	2005	MBR	11:25	MOCH
IB&ARN	7974	R	RBNU	07-ARN-01	MOCH	2005	MBR	11:25	MOCH
IB&ARN	7974	R	RBNU	07-ARN-01	MOCH	2005	MBR	11:25	MOCH
IB&ARN	7974	R	RBNU	07-ARN-01	MOCH	2005	MBR	11:25	MOCH
IB&ARN	7974	R	RBNU	07-ARN-01	MOCH	2005	MBR	11:25	BCCH

IB&ARN	7974	R	RBNU	07-ARN-01	MOCH	2005	MBR	11:25	RBNU
IB&ARN	7974	R	RBNU	07-ARN-01	MOCH	2005	MBR	11:25	RBNU
IB&ARN	7974	R	RBNU	07-ARN-01	MOCH	2005	MBR	11:25	RBNU
IB&ARN	7974	R	RBNU	07-ARN-01	MOCH	2005	MBR	11:25	RBNU
IB&ARN	7974	R	RBNU	07-ARN-01	MOCH	2005	MBR	11:25	RBNU
IB&ARN	7974	R	RBNU	07-ARN-01	MOCH	2005	MBR	11:25	BCCH
IB&ARN	7974	R	RBNU	07-ARN-01	MOCH	2005	MBR	11:25	RBNU
IB	70005	R	RBNU	07-ARN-01	RBNU	2006	RMB	11:39	MOCH
IB	70005	R	RBNU	07-ARN-01	RBNU	2006	RMB	11:39	MOCH
IB	70005	R	RBNU	07-ARN-01	RBNU	2006	RMB	11:39	MOCH
IB	70005	R	RBNU	07-ARN-01	RBNU	2006	RMB	11:39	MOCH
IB	70005	R	RBNU	07-ARN-01	RBNU	2006	RMB	11:39	MOCH
IB	70005	R	RBNU	07-ARN-01	RBNU	2006	RMB	11:39	MOCH
IB	70005	R	RBNU	07-ARN-01	RBNU	2006	RMB	11:39	BCCH
IB	70005	R	RBNU	07-ARN-01	RBNU	2006	RMB	11:39	MOCH
IB	70005	R	RBNU	07-ARN-01	RBNU	2006	RMB	11:39	MOCH
IB	70005	R	RBNU	07-ARN-01	RBNU	2006	RMB	11:39	MOCH
IB	70005	R	RBNU	07-ARN-01	RBNU	2006	RMB	11:39	MOCH
IB	70005	R	RBNU	07-ARN-01	RBNU	2006	RMB	11:39	BCCH
IB	70005	R	RBNU	07-ARN-01	RBNU	2006	RMB	11:39	MOCH
IB	70005	R	RBNU	07-ARN-01	RBNU	2006	RMB	11:39	MOCH
IB	70005	R	RBNU	07-ARN-01	RBNU	2006	RMB	11:39	MOCH
IB	70005	R	RBNU	07-ARN-01	RBNU	2006	RMB	11:39	MOCH
IB	70005	R	RBNU	07-ARN-01	RBNU	2006	RMB	11:39	MOCH
IB	70005	R	RBNU	07-ARN-01	RBNU	2006	RMB	11:39	MOCH
IB	70005	R	RBNU	07-ARN-01	RBNU	2006	RMB	11:39	MOCH
IB	70005	R	RBNU	07-ARN-01	RBNU	2006	RMB	11:39	MOCH
IB	70005	R	RBNU	07-ARN-01	RBNU	2006	RMB	11:39	RBNU
IB	70005	R	RBNU	07-ARN-01	RBNU	2006	RMB	11:39	RBNU
IB	70005	R	RBNU	07-ARN-01	RBNU	2006	RMB	11:39	RBNU
IB	70005	R	RBNU	07-ARN-01	RBNU	2006	RMB	11:39	RBNU
IB	70005	R	RBNU	07-ARN-01	RBNU	2006	RMB	11:39	MOCH
IB	70005	R	RBNU	07-ARN-01	RBNU	2006	RMB	11:39	BCCH
IB	70198	R	RBNU	07-ARN-036			BRM	10:34	BCCH
IB	70198	R	RBNU	07-ARN-036			BRM	10:34	MOCH
IB	70198	R	RBNU	07-ARN-036			BRM	10:34	MOCH
IB	70198	R	RBNU	07-ARN-036			BRM	10:34	MOCH
IB	70198	R	RBNU	07-ARN-036			BRM	10:34	MOCH
IB	70198	R	RBNU	07-ARN-036			BRM	10:34	MOCH
IB	70198	R	RBNU	07-ARN-036			BRM	10:34	BCCH
IB	70198	R	RBNU	07-ARN-036			BRM	10:34	RBNU
IB	70198	R	RBNU	07-ARN-036			BRM	10:34	MOCH
IB	7974	R	RBNU	07-ARN-01	MOCH	2005	RBM	10:45 AM	BCCH
IB	7974	R	RBNU	07-ARN-01	MOCH	2005	RBM	10:45 AM	BCCH

IB	7974	R	RBNU	07-ARN-01	MOCH	2005	RBM	10:45 AM	BCCH
IB	7974	R	RBNU	07-ARN-01	MOCH	2005	RBM	10:45 AM	RBNU
IB	7974	R	RBNU	07-ARN-01	MOCH	2005	RBM	10:45 AM	BCCH
IB	7974	R	RBNU	07-ARN-01	MOCH	2005	RBM	10:45 AM	BCCH
IB	7974	R	RBNU	07-ARN-01	MOCH	2005	RBM	10:45 AM	BCCH
IB	7974	R	RBNU	07-ARN-01	MOCH	2005	RBM	10:45 AM	RBNU
IB	7974	R	RBNU	07-ARN-01	MOCH	2005	RBM	10:45 AM	RBNU
IB	7974	R	RBNU	07-ARN-01	MOCH	2005	RBM	10:45 AM	RBNU
IB	7974	R	RBNU	07-ARN-01	MOCH	2005	RBM	10:45 AM	RBNU
IB	7974	R	RBNU	07-ARN-01	MOCH	2005	RBM	10:45 AM	RBNU
IB	7974	R	RBNU	07-ARN-01	MOCH	2005	RBM	10:45 AM	RBNU
IB	7974	R	RBNU	07-ARN-01	MOCH	2005	RBM	10:45 AM	RBNU
IB	7974	R	RBNU	07-ARN-01	MOCH	2005	RBM	10:45 AM	BCCH
IB	7974	R	RBNU	07-ARN-01	MOCH	2005	RBM	10:45 AM	RBNU
IB	7974	R	RBNU	07-ARN-01	MOCH	2005	RBM	10:45 AM	BCCH
IB	7974	R	RBNU	07-ARN-01	MOCH	2005	RBM	10:45 AM	MOCH
	7564	R			RBNU	2003	MRB	10:16	MOCH
	7564	R			RBNU	2003	MRB	10:16	MOCH
	7564	R			RBNU	2003	MRB	10:16	RBNU
	7564	R			RBNU	2003	MRB	10:16	MOCH
	7564	R			RBNU	2003	MRB	10:16	RBNU
	7564	R			RBNU	2003	MRB	10:16	MOCH
	7564	R			RBNU	2003	MRB	10:16	RBNU
	7564	R			RBNU	2003	MRB	10:16	BCCH
	7512	R			MOCH	2003	BMR	11:10	BCCH
	7512	R			MOCH	2003	BMR	11:10	BCCH
	7512	R			MOCH	2003	BMR	11:10	BCCH
	7512	R			MOCH	2003	BMR	11:10	MOCH
	7512	R			MOCH	2003	BMR	11:10	MOCH
	7512	R			MOCH	2003	BMR	11:10	MOCH
	7512	R			MOCH	2003	BMR	11:10	RBNU
	7512	R			MOCH	2003	BMR	11:10	MOCH
	7512	R			MOCH	2003	BMR	11:10	MOCH
	7512	R			MOCH	2003	BMR	11:10	RBNU
	7512	R			MOCH	2003	BMR	11:10	MOCH
	7512	R			MOCH	2003	BMR	11:10	RBNU
	7512	R			MOCH	2003	BMR	11:10	RBNU
	7512	R			MOCH	2003	BMR	11:10	RBNU
	7512	R			MOCH	2003	BMR	11:10	BCCH
	7512	R			MOCH	2003	BMR	11:10	RBNU
	7512	R			MOCH	2003	BMR	11:10	MOCH
	7512	R			MOCH	2003	BMR	11:10	BCCH
	7512	R			MOCH	2003	BMR	11:10	MOCH
	7512	R			MOCH	2003	BMR	11:10	BCCH

7512 R	MOCH	2003 BMR	11:10 RBNU
7512 R	MOCH	2003 BMR	11:10 RBNU
7512 R	MOCH	2003 BMR	11:10 BCCH
7512 R	MOCH	2003 BMR	11:10 RBNU
7512 R	MOCH	2003 BMR	11:10 BCCH
7656 R	RBNU	2001 BRM	7:30 BCCH
7656 R	RBNU	2001 BRM	7:30 BCCH
7656 R	RBNU	2001 BRM	7:30 RBNU
7656 R	RBNU	2001 BRM	7:30 RBNU
7656 R	RBNU	2001 BRM	7:30 RBNU
7656 R	RBNU	2001 BRM	7:30 RBNU
7656 R	RBNU	2001 BRM	7:30 RBNU
7656 R	RBNU	2001 BRM	7:30 RBNU
7656 R	RBNU	2001 BRM	7:30 RBNU
7656 R	RBNU	2001 BRM	7:30 MOCH
7656 R	RBNU	2001 BRM	7:30 MOCH
7825 R	BCCH	2003 BRM	10:00 BCCH
7825 R	BCCH	2003 BRM	10:00 BCCH
7825 R	BCCH	2003 BRM	10:00 BCCH
7825 R	BCCH	2003 BRM	10:00 none
7825 R	BCCH	2003 BRM	10:00 BCCH
7825 R	BCCH	2003 BRM	11:00 BCCH
7825 R	BCCH	2003 BRM	11:00 RBNU
7825 R	BCCH	2003 BRM	11:00 RBNU
7679 R	MOCH	2002 RMB	11:35 MOCH
7679 R	MOCH	2002 RMB	11:35 MOCH
7679 R	MOCH	2002 RMB	11:35 BCCH
7679 R	MOCH	2002 RMB	11:35 MOCH
7679 R	MOCH	2002 RMB	11:35 MOCH
7679 R	MOCH	2002 RMB	11:35 MOCH
7775 R	MOCH	2003 MBR	8:00 BCCH
7775 R	MOCH	2003 MBR	8:00 BCCH
7775 R	MOCH	2003 MBR	8:00 BCCH
7775 R	MOCH	2003 MBR	8:00 BCCH
7775 R	MOCH	2003 MBR	8:00 BCCH
7775 R	MOCH	2003 MBR	8:00 BCCH
7775 R	MOCH	2003 MBR	8:00 BCCH
7775 R	MOCH	2003 MBR	8:00 MOCH
7775 R	MOCH	2003 MBR	8:00 BCCH
7775 R	MOCH	2003 MBR	8:00 MOCH
7775 R	MOCH	2003 MBR	8:00 BCCH
7775 R	MOCH	2003 MBR	8:00 BCCH
7775 R	MOCH	2003 MBR	8:00 MOCH
7775 R	MOCH	2003 MBR	8:00 BCCH

7775	R	MOCH	2003	MBR	8:00	BCCH
7775	R	MOCH	2003	MBR	8:00	MOCH
7775	R	MOCH	2003	MBR	8:00	none
7775	R	MOCH	2003	MBR	8:00	RBNU
7775	R	MOCH	2003	MBR	8:00	MOCH
7775	R	MOCH	2003	MBR	8:00	BCCH
7775	R	MOCH	2003	MBR	8:00	BCCH
7775	R	MOCH	2003	MBR	8:00	RBNU
7775	R	MOCH	2003	MBR	8:00	MOCH
7775	R	MOCH	2003	MBR	8:00	RBNU
7974	R	RBNU	2003	MRB	10:40	BCCH
7974	R	RBNU	2003	MRB	10:40	BCCH
7974	R	RBNU	2003	MRB	10:40	BCCH
7974	R	RBNU	2003	MRB	10:40	BCCH
7974	R	RBNU	2003	MRB	10:40	BCCH
7974	R	RBNU	2003	MRB	10:40	BCCH
7974	R	RBNU	2003	MRB	10:40	BCCH
7974	R	RBNU	2003	MRB	10:40	BCCH
7974	R	RBNU	2003	MRB	10:40	BCCH
7974	R	RBNU	2003	MRB	10:40	BCCH
7974	R	RBNU	2003	MRB	10:40	BCCH
7974	R	RBNU	2003	MRB	10:40	BCCH
7974	R	RBNU	2003	MRB	10:40	none
7974	R	RBNU	2003	MRB	10:40	MOCH
7974	R	RBNU	2003	MRB	10:40	RBNU
7974	R	RBNU	2003	MRB	10:40	RBNU
7974	R	RBNU	2003	MRB	10:40	RBNU
7974	R	RBNU	2003	MRB	10:40	RBNU
7974	R	RBNU	2003	MRB	10:40	RBNU
7974	R	RBNU	2003	MRB	10:40	RBNU
7974	R	RBNU	2003	MRB	10:40	BCCH
7974	R	RBNU	2003	MRB	10:40	RBNU
7974	R	RBNU	2003	MRB	10:40	MOCH
7974	R	RBNU	2003	MRB	10:40	BCCH
7974	R	RBNU	2003	MRB	10:40	MOCH
7974	R	RBNU	2003	MRB	10:40	RBNU
7130	R	DOWO	2003	BRM	8:09	none
70102	R	MOCH	2003	BMR	9:01	RBNU
70102	R	MOCH	2003	BMR	9:01	RBNU
70102	R	MOCH	2003	BMR	9:01	MOCH
70102	R	MOCH	2003	BMR	9:01	MOCH
70102	R	MOCH	2003	BMR	9:01	none
70102	R	MOCH	2003	BMR	9:01	BCCH
7040	R	MOCH	2002	MBR	9:15:30	RBNU

7040	R	MOCH	2002	MBR	9:15:30	RBNU
7040	R	MOCH	2002	MBR	9:15:30	RBNU
7040	R	MOCH	2002	MBR	9:15:30	BCCH
7983	R	RBNU	2002	BRM	8:37	MOCH
7983	R	RBNU	2002	BRM	8:37	BCCH
7983	R	RBNU	2002	BRM	8:37	MOCH
7983	R	RBNU	2002	BRM	8:37	BCCH
7983	R	RBNU	2002	BRM	8:37	RBNU
7983	R	RBNU	2002	BRM	8:37	MOCH
7983	R	RBNU	2002	BRM	8:37	BCCH
7983	R	RBNU	2002	BRM	8:37	none
7983	R	RBNU	2002	BRM	8:37	BCCH
7983	R	RBNU	2002	BRM	8:37	RBNU
7983	R	RBNU	2002	BRM	8:37	RBNU
7983	R	RBNU	2002	BRM	8:37	MOCH
7983	R	RBNU	2002	BRM	8:37	RBNU
7983	R	RBNU	2002	BRM	8:37	RBNU
7983	R	RBNU	2002	BRM	8:37	none
7564	R	RBNU	2003	MBR	10:08	BCCH
7564	R	RBNU	2003	MBR	10:08	BCCH
7564	R	RBNU	2003	MBR	10:08	BCCH
7564	R	RBNU	2003	MBR	10:08	RBNU
7564	R	RBNU	2003	MBR	10:08	MOCH
70101	R			RBM	8:16	RBNU
70101	R			RBM	8:16	BCCH
70101	R			RBM	8:16	MOCH
70101	R			RBM	8:16	RBNU
70101	R			RBM	8:16	RBNU
70101	R			RBM	8:16	RBNU
70101	R			RBM	8:16	MOCH
7974	R	RBNU	2003	MRB	7:42:35	BCCH
7974	R	RBNU	2003	MRB	7:42:35	BCCH
7974	R	RBNU	2003	MRB	7:42:35	BCCH
7974	R	RBNU	2003	MRB	7:42:35	BCCH
7974	R	RBNU	2003	MRB	7:42:35	RBNU
7974	R	RBNU	2003	MRB	7:42:35	none
7974	R	RBNU	2003	MRB	7:42:35	none
7974	R	RBNU	2003	MRB	7:42:35	BCCH
7974	R	RBNU	2003	MRB	7:42:35	RBNU
70101	R			BRM	8:13:34	BCCH
70101	R			BRM	8:13:34	BCCH
70101	R			BRM	8:13:34	RBNU
70101	R			BRM	8:13:34	RBNU
70101	R			BRM	8:13:34	RBNU

70101	R			BRM	8:13:34	MOCH
70101	R			BRM	8:13:34	BCCH
70101	R			BRM	8:13:34	RBNU
70101	R			BRM	8:13:34	BCCH
70101	R			BRM	8:13:34	none
7512	R	MOCH	2003	BRM	7:55:30	none
7512	R	MOCH	2003	BRM	7:55:30	MOCH
7512	R	MOCH	2003	BRM	7:55:30	none
7512	R	MOCH	2003	BRM	7:55:30	BCCH
7512	R	MOCH	2003	BRM	7:55:30	RBNU
7512	R	MOCH	2003	BRM	7:55:30	MOCH
7512	R	MOCH	2003	BRM	7:55:30	MOCH
7512	R	MOCH	2003	BRM	7:55:30	MOCH
7512	R	MOCH	2003	BRM	7:55:30	none
7512	R	MOCH	2003	BRM	7:55:30	BCCH
7512	R	MOCH	2003	BRM	7:55:30	RBNU
7512	R	MOCH	2003	BRM	7:55:30	MOCH
7512	R	MOCH	2003	BRM	7:55:30	BCCH
7512	R	MOCH	2003	BRM	7:55:30	BCCH
7512	R	MOCH	2003	BRM	7:55:30	none
7512	R	MOCH	2003	BRM	7:55:30	RBNU
7512	R	MOCH	2003	BRM	7:55:30	none
70101	R			RMB	9:35	MOCH
70101	R			RMB	9:35	MOCH
70101	R			RMB	9:35	RBNU
70101	R			RMB	9:35	RBNU
70101	R			RMB	9:35	BCCH
70101	R			RMB	9:35	BCCH
70723	R	BCCH	2004	BMR	11:20	MOCH
70723	R	BCCH	2004	BMR	11:20	BCCH
70723	R	BCCH	2004	BMR	11:20	BCCH
70723	R	BCCH	2004	BMR	11:20	RBNU
70723	R	BCCH	2004	BMR	11:20	BCCH
70723	R	BCCH	2004	BMR	11:20	MOCH
70723	R	BCCH	2004	BMR	11:20	RBNU
70723	R	BCCH	2004	BMR	11:20	BCCH
70730	R	BCCH	2004	RBM	9:14	MOCH
70730	R	BCCH	2004	RBM	9:14	MOCH
70730	R	BCCH	2004	RBM	9:14	MOCH
70730	R	BCCH	2004	RBM	9:14	MOCH
70730	R	BCCH	2004	RBM	9:14	MOCH
70730	R	BCCH	2004	RBM	9:14	MOCH
70730	R	BCCH	2004	RBM	9:14	MOCH
7063	R	MOCH	2004	MBR	10:39	none

7063	R	MOCH	2004	MBR	10:39	none
7063	R	MOCH	2004	MBR	10:39	none
7063	R	MOCH	2004	MBR	10:39	none
NEW RBNL	R	-	-	BMR	10:18	none
NEW RBNL	R	-	-	BMR	10:18	none
NEW RBNL	R	-	-	BMR	10:18	BCCH
NEW RBNL	R	-	-	BMR	10:18	BCCH
NEW RBNL	R	-	-	BMR	10:18	BCCH
NEW RBNL	R	-	-	BMR	10:18	BCCH
NEW RBNL	R	-	-	BMR	10:18	RBNU
NEW RBNL	R	-	-	BMR	10:18	BCCH
NEW RBNL	R	-	-	BMR	10:18	RBNU
NEW RBNL	R	-	-	BMR	10:18	RBNU
NEW RBNL	R	-	-	BMR	10:18	RBNU
NEW RBNL	R	-	-	BMR	10:18	RBNU
NEW RBNL	R	-	-	BMR	10:18	RBNU
NEW RBNL	R	-	-	BMR	10:18	none
NEW RBNL	R	-	-	BMR	10:18	MOCH
NEW RBNL	R	-	-	BMR	10:18	none
7130	R	MOCH	1999	BMR	11:39	MOCH
7130	R	MOCH	1999	BMR	11:39	MOCH
7130	R	MOCH	1999	BMR	11:39	MOCH
7130	R	MOCH	1999	BMR	11:39	MOCH
7130	R	MOCH	1999	BMR	11:39	MOCH
7130	R	MOCH	1999	BMR	11:39	none
7130	R	MOCH	1999	BMR	11:39	BCCH
7130	R	MOCH	1999	BMR	11:39	BCCH
7130	R	MOCH	1999	BMR	11:39	BCCH
7130	R	MOCH	1999	BMR	11:39	MOCH
7130	R	MOCH	1999	BMR	11:39	none
7130	R	MOCH	1999	BMR	11:39	RBNU
7130	R	MOCH	1999	BMR	11:39	none
7656	R	MOCH	2004	MBR	7:50	MOCH
7656	R	MOCH	2004	MBR	7:50	MOCH
7656	R	MOCH	2004	MBR	7:50	BCCH
7656	R	MOCH	2004	MBR	7:50	BCCH
7656	R	MOCH	2004	MBR	7:50	MOCH
7656	R	MOCH	2004	MBR	7:50	BCCH
7656	R	MOCH	2004	MBR	7:50	none
7557	R			BRM	11:14	RBNU
check	R			RBM	9:06	RBNU
check	R			RBM	9:06	RBNU
check	R			RBM	9:06	none
check	R			RBM	9:06	BCCH

check	R	RBM	9:06	MOCH
check	R	RBM	9:06	MOCH
check	R	RBM	9:06	BCCH
	R	BMR	10:40	BCCH
	R	BMR	10:40	BCCH
	R	BMR	10:40	MOCH
	R	BMR	10:40	BCCH
	R	BMR	10:40	MOCH
	R	BMR	10:40	none
	R	BMR	10:40	BCCH
	R	BMR	10:40	RBNU
	R	BMR	10:40	RBNU
	R	BMR	10:40	RBNU
	R	BMR	10:40	MOCH
	R	BMR	10:40	RBNU
	R	BMR	10:40	RBNU
	R	BMR	10:40	RBNU
	R	BMR	10:40	RBNU
	R	BMR	10:40	RBNU
	R	BMR	10:40	MOCH
	R	BMR	10:40	MOCH
POND	R	BMR	10:05	BCCH
POND	R	BMR	10:05	BCCH
POND	R	BMR	10:05	BCCH
POND	R	BMR	10:05	BCCH
POND	R	BMR	10:18	MOCH
POND	R	BMR	10:25	RBNU
POND	R	BMR	10:25	RBNU
7595	R	MRB	9:26	BCCH
7595	R	MRB	9:26	RBNU
7595	R	MRB	9:26	RBNU
7595	R	MRB	9:26	RBNU
7595	R	MRB	9:26	RBNU
7595	R	MRB	9:26	BCCH
7595	R	MRB	9:26	MOCH
7595	R	MRB	9:26	MOCH
7595	R	MRB	9:26	RBNU
7862R	R	MBR	11:40	RBNU
7862R	R	MBR	11:40	MOCH
7862R	R	MBR	11:40	RBNU
7862R	R	MBR	11:40	RBNU
7862R	R	MBR	11:40	BCCH
7862R	R	MBR	11:40	RBNU
7862R	R	MBR	11:40	BCCH

	7862R	R			MBR	11:40	MOCH
	7862R	R			MBR	11:40	RBNU
	7862R	R			MBR	11:40	
	7440R	R			MBR	9:06	RBNU
	7440R	R			MBR	9:06	BCCH
	7440R	R			MBR	9:06	MOCH
	7440R	R			MBR	9:06	RBNU
	7440R	R			MBR	9:06	
		R			RMB	8:08	BCCH
		R			RMB	8:08	RBNU
		R			RMB	8:08	RBNU
		R			RMB	8:08	MOCH
		R			RMB	8:08	BCCH
		R			RMB	8:08	BCCH
		R			RMB	8:08	BCCH
		R			RMB	8:08	BCCH
		R			RMB	8:08	BCCH
		R			RMB	8:08	MOCH
		R			RMB	8:08	RBNU
		R			RMB	8:08	
		R			RBM	10:13	MOCH
		R			RBM	10:13	RBNU
		R			RBM	10:13	BCCH
		R			RBM	10:13	MOCH
		R			RBM	10:13	RBNU
		R			RBM	10:13	
IB	7374	R	RBNU	2005	RMB	10:44	RBNU
IB	7374	R	RBNU	2005	RMB	10:44	RBNU
IB	7374	R	RBNU	2005	RMB	10:44	RBNU
IB	7374	R	RBNU	2005	RMB	10:44	MOCH
IB	7374	R	RBNU	2005	RMB	10:44	BCCH
IB	70755	R	MOCH	2006	RMB	11:21	BCCH
IB	70755	R	MOCH	2006	RMB	11:21	BCCH
IB	70755	R	MOCH	2006	RMB	11:21	BCCH
IB	70755	R	MOCH	2006	RMB	11:21	BCCH
IB	70755	R	MOCH	2006	RMB	11:21	BCCH
IB	70755	R	MOCH	2006	RMB	11:21	BCCH
IB	70755	R	MOCH	2006	RMB	11:21	RBNU
IB	70755	R	MOCH	2006	RMB	11:21	MOCH
IB	70755	R	MOCH	2006	RMB	11:21	BCCH
IB	7408	R	MOCH	2006	MRB	9:50	BCCH
IB	7408	R	MOCH	2006	MRB	9:50	BCCH
IB	7408	R	MOCH	2006	MRB	9:50	BCCH
IB	7408	R	MOCH	2006	MRB	9:50	BCCH

IB	7408	R	MOCH	2006	MRB	9:50	BCCH
IB	7408	R	MOCH	2006	MRB	9:50	BCCH
IB	7408	R	MOCH	2006	MRB	9:50	BCCH
IB	7408	R	MOCH	2006	MRB	9:50	BCCH
IB	7408	R	MOCH	2006	MRB	9:50	BCCH
IB	7408	R	MOCH	2006	MRB	9:50	BCCH
IB	7408	R	MOCH	2006	MRB	9:50	BCCH
IB	7408	R	MOCH	2006	MRB	9:50	BCCH
IB	7408	R	MOCH	2006	MRB	9:50	BCCH
IB	7408	R	MOCH	2006	MRB	9:50	RBNU
IB	7408	R	MOCH	2006	MRB	9:50	BCCH
IB	7408	R	MOCH	2006	MRB	9:50	RBNU
IB	7408	R	MOCH	2006	MRB	9:50	RBNU
IB	7408	R	MOCH	2006	MRB	9:50	MOCH
IB	7280	R	MOCH	2006	MRB	9:46	MOCH
IB	7280	R	MOCH	2006	MRB	9:46	MOCH
IB	7280	R	MOCH	2006	MRB	9:46	MOCH
IB	7280	R	MOCH	2006	MRB	9:46	MOCH
IB	7280	R	MOCH	2006	MRB	9:46	MOCH
IB	7280	R	MOCH	2006	MRB	9:46	MOCH
IB	7280	R	MOCH	2006	MRB	9:46	MOCH
IB	7280	R	MOCH	2006	MRB	9:46	MOCH
IB	7280	R	MOCH	2006	MRB	9:46	MOCH
IB	7280	R	MOCH	2006	MRB	9:46	MOCH
IB	7280	R	MOCH	2006	MRB	9:46	MOCH
IB	7280	R	MOCH	2006	MRB	9:46	MOCH
IB	7280	R	MOCH	2006	MRB	9:46	RBNU
IB	7280	R	MOCH	2006	MRB	9:46	RBNU
IB	7280	R	MOCH	2006	MRB	9:46	RBNU
IB	7280	R	MOCH	2006	MRB	9:46	BCCH
IB	7280	R	MOCH	2006	MRB	9:46	BCCH
IB	7280	R	MOCH	2006	MRB	9:46	BCCH
IB	7280	R	MOCH	2006	MRB	9:46	MOCH
IB	7280	R	MOCH	2006	MRB	9:46	RBNU
IB	7280	R	MOCH	2006	MRB	9:46	BCCH
IB	70703	R	RBNU	2006	MBR	9:32	BCCH
IB	70703	R	RBNU	2006	MBR	9:32	BCCH
IB	70703	R	RBNU	2006	MBR	9:32	BCCH
IB	70703	R	RBNU	2006	MBR	9:32	BCCH
IB	70703	R	RBNU	2006	MBR	9:32	BCCH
IB	70703	R	RBNU	2006	MBR	9:32	BCCH
IB	70703	R	RBNU	2006	MBR	9:32	BCCH
IB	70703	R	RBNU	2006	MBR	9:32	RBNU
IB	70703	R	RBNU	2006	MBR	9:32	BCCH

IB	70703	R		RBNU	2006	MBR	9:32	RBNU
IB	70703	R		RBNU	2006	MBR	9:32	RBNU
IB	70703	R		RBNU	2006	MBR	9:32	RBNU
IB	70703	R		RBNU	2006	MBR	9:32	MOCH
IB	70703	R		RBNU	2006	MBR	9:32	BCCH
IB	70703	R		RBNU	2006	MBR	9:32	BCCH
IB	70703	R		RBNU	2006	MBR	9:32	BCCH
IB	70703	R		RBNU	2006	MBR	9:32	RBNU
IB	70703	R		RBNU	2006	MBR	9:32	MOCH
IB	70703	R		RBNU	2006	MBR	9:32	MOCH
IB	70703	R		RBNU	2006	MBR	9:32	RBNU
IB	70703	R		RBNU	2006	MBR	9:32	MOCH
IB	70703	R		RBNU	2006	MBR	9:32	MOCH
IB	70703	R		RBNU	2006	MBR	9:32	MOCH
IB	70703	R		RBNU	2006	MBR	9:32	MOCH
IB	70703	R		RBNU	2006	MBR	9:32	MOCH
IB	70703	R		RBNU	2006	MBR	9:32	BCCH
IB	70703	R		RBNU	2006	MBR	9:32	MOCH
IB	70703	R		RBNU	2006	MBR	9:32	MOCH
IB	70703	R		RBNU	2006	MBR	9:32	MOCH
IB	70703	R		RBNU	2006	MBR	9:32	BCCH
IB	70703	R		RBNU	2006	MBR	9:32	RBNU
IB	70110	R	05-PAR-11	MOCH	2005	RMB	11:23	BCCH
IB	70110	R	05-PAR-11	MOCH	2005	RMB	11:23	RBNU
IB	70110	R	05-PAR-11	MOCH	2005	RMB	11:23	RBNU
IB	70110	R	05-PAR-11	MOCH	2005	RMB	11:23	RBNU
IB	70110	R	05-PAR-11	MOCH	2005	RMB	11:23	RBNU
IB	70110	R	05-PAR-11	MOCH	2005	RMB	11:23	RBNU
IB	70110	R	05-PAR-11	MOCH	2005	RMB	11:23	RBNU
IB	70110	R	05-PAR-11	MOCH	2005	RMB	11:23	RBNU
IB	70110	R	05-PAR-11	MOCH	2005	RMB	11:23	MOCH
IB	70110	R	05-PAR-11	MOCH	2005	RMB	11:23	MOCH
IB	70110	R	05-PAR-11	MOCH	2005	RMB	11:23	MOCH
IB	70110	R	05-PAR-11	MOCH	2005	RMB	11:23	MOCH
IB	70110	R	05-PAR-11	MOCH	2005	RMB	11:23	RBNU
IB	70110	R	05-PAR-11	MOCH	2005	RMB	11:23	MOCH
IB	70110	R	05-PAR-11	MOCH	2005	RMB	11:23	BCCH
IB	06-SLD-06	R		RBNU	2006	BRM	9:52	MOCH
IB	06-SLD-06	R		RBNU	2006	BRM	9:52	MOCH
IB	06-SLD-06	R		RBNU	2006	BRM	9:52	MOCH
IB	06-SLD-06	R		RBNU	2006	BRM	9:52	MOCH
IB	06-SLD-06	R		RBNU	2006	BRM	9:52	MOCH
IB	06-SLD-06	R		RBNU	2006	BRM	9:52	MOCH
IB	06-SLD-06	R		RBNU	2006	BRM	9:52	MOCH

IB	06-SLD-06	R	RBNU	2006	BRM	9:52	MOCH
IB	06-SLD-06	R	RBNU	2006	BRM	9:52	MOCH
IB	06-SLD-06	R	RBNU	2006	BRM	9:52	MOCH
IB	06-SLD-06	R	RBNU	2006	BRM	9:52	MOCH
IB	06-SLD-06	R	RBNU	2006	BRM	9:52	RBNU
IB	06-SLD-06	R	RBNU	2006	BRM	9:52	RBNU
IB	06-SLD-06	R	RBNU	2006	BRM	9:52	MOCH
IB	06-SLD-06	R	RBNU	2006	BRM	9:52	RBNU
IB	06-SLD-06	R	RBNU	2006	BRM	9:52	RBNU
IB	06-SLD-06	R	RBNU	2006	BRM	9:52	MOCH
IB	06-SLD-06	R	RBNU	2006	BRM	9:52	BCCH
IB	06-SLD-06	R	RBNU	2006	BRM	9:52	RBNU
IB	06-SLD-06	R	RBNU	2006	BRM	9:52	MOCH
IB	06-SLD-04	R	?	?	RMB	10:52	RBNU
IB	06-SLD-04	R			RMB	10:52	MOCH
IB	06-SLD-04	R			RMB	10:52	BCCH
IB	70418	R	MOCH	2005	RBM	9:34	MOCH
IB	70418	R	MOCH	2005	RBM	9:34	MOCH
IB	70418	R	MOCH	2005	RBM	9:34	MOCH
IB	70418	R	MOCH	2005	RBM	9:34	MOCH
IB	70418	R	MOCH	2005	RBM	9:34	MOCH
IB	70418	R	MOCH	2005	RBM	9:34	MOCH
IB	70418	R	MOCH	2005	RBM	9:34	MOCH
IB	70418	R	MOCH	2005	RBM	9:34	RBNU
IB	70418	R	MOCH	2005	RBM	9:34	RBNU
IB	70418	R	MOCH	2005	RBM	9:34	RBNU
IB	70418	R	MOCH	2005	RBM	9:34	RBNU
IB	70418	R	MOCH	2005	RBM	9:34	MOCH
IB	70418	R	MOCH	2005	RBM	9:34	RBNU
IB	70418	R	MOCH	2005	RBM	9:34	BCCH
IB	70418	R	MOCH	2005	RBM	9:34	MOCH
IB	70694	R	MOCH	2006	MRB	11:30	BCCH
IB	70694	R	MOCH	2006	MRB	11:30	BCCH
IB	70694	R	MOCH	2006	MRB	11:30	BCCH
IB	70694	R	MOCH	2006	MRB	11:30	MOCH
IB	70694	R	MOCH	2006	MRB	11:30	MOCH
IB	70694	R	MOCH	2006	MRB	11:30	MOCH
IB	70694	R	MOCH	2006	MRB	11:30	MOCH
IB	70694	R	MOCH	2006	MRB	11:30	MOCH
IB	70694	R	MOCH	2006	MRB	11:30	MOCH
IB	70694	R	MOCH	2006	MRB	11:30	MOCH
IB	70694	R	MOCH	2006	MRB	11:30	MOCH
IB	70694	R	MOCH	2006	MRB	11:30	MOCH
IB	70694	R	MOCH	2006	MRB	11:30	MOCH

IB	70694	R	MOCH	2006	MRB	11:30	RBNU
IB	70694	R	MOCH	2006	MRB	11:30	RBNU
IB	70694	R	MOCH	2006	MRB	11:30	RBNU
IB	70694	R	MOCH	2006	MRB	11:30	BCCH
IB	70694	R	MOCH	2006	MRB	11:30	BCCH
IB	70694	R	MOCH	2006	MRB	11:30	BCCH
IB	70694	R	MOCH	2006	MRB	11:30	BCCH
IB	70694	R	MOCH	2006	MRB	11:30	BCCH
IB	70694	R	MOCH	2006	MRB	11:30	BCCH
IB	70694	R	MOCH	2006	MRB	11:30	BCCH
IB	70694	R	MOCH	2006	MRB	11:30	BCCH
IB	70694	R	MOCH	2006	MRB	11:30	BCCH
IB	70694	R	MOCH	2006	MRB	11:30	MOCH
IB	70694	R	MOCH	2006	MRB	11:30	RBNU
IB	70694	R	MOCH	2006	MRB	11:30	RBNU
IB	70694	R	MOCH	2006	MRB	11:30	RBNU
IB	70694	R	MOCH	2006	MRB	11:30	BCCH
IB	70694	R	MOCH	2006	MRB	11:30	RBNU
IB	70694	R	MOCH	2006	MRB	11:30	RBNU
IB	70694	R	MOCH	2006	MRB	11:30	BCCH
IB	70694	R	MOCH	2006	MRB	11:30	BCCH
IB	70694	R	MOCH	2006	MRB	11:30	MOCH
IB	70694	R	MOCH	2006	MRB	11:30	RBNU
IB	70694	R	MOCH	2006	MRB	11:30	RBNU
IB	70694	R	MOCH	2006	MRB	11:30	BCCH
IB	70415	R			MRB	9:11	MOCH
IB	70415	R			MRB	9:11	RBNU
IB	70415	R			MRB	9:11	RBNU
IB	70415	R			MRB	9:11	RBNU
IB	70415	R			MRB	9:11	RBNU
IB	70415	R			MRB	9:11	RBNU
IB	70415	R			MRB	9:11	MOCH
IB	70415	R			MRB	9:11	MOCH
IB	70415	R			MRB	9:11	RBNU
IB	70415	R			MRB	9:11	MOCH
IB	70415	R			MRB	9:11	MOCH
IB	70415	R			MRB	9:11	RBNU
IB	70415	R			MRB	9:11	RBNU
IB	70415	R			MRB	9:11	MOCH
IB	70415	R			MRB	9:11	RBNU
IB	70415	R			MRB	9:11	BCCH
IB	70415	R			MRB	9:11	BCCH
IB	70415	R			MRB	9:11	BCCH
IB	7974	R			MBR	10:49	MOCH

IB	7974	R	MBR	10:49	MOCH
IB	7974	R	MBR	10:49	RBNU
IB	7974	R	MBR	10:49	RBNU
IB	7974	R	MBR	10:49	RBNU
IB	7974	R	MBR	10:49	MOCH
IB	7974	R	MBR	10:49	BCCH
IB	7974	R	MBR	10:49	RBNU
IB	7974	R	MBR	10:49	RBNU
IB	70735	R	RMB	7:50	BCCH
IB	70735	R	RMB	7:50	BCCH
IB	70735	R	RMB	7:50	BCCH
IB	70735	R	RMB	7:50	BCCH
IB	70735	R	RMB	7:50	BCCH
IB	70735	R	RMB	7:50	BCCH
IB	70735	R	RMB	7:50	MOCH
IB	70735	R	RMB	7:50	MOCH
IB	70735	R	RMB	7:50	BCCH
IB	70735	R	RMB	7:50	BCCH
IB	70735	R	RMB	7:50	BCCH
IB	70735	R	RMB	7:50	BCCH
IB	70735	R	RMB	7:50	RBNU
IB	70735	R	RMB	7:50	BCCH
IB	70735	R	RMB	7:50	MOCH
IB	70735	R	RMB	7:50	RBNU
IB	70735	R	RMB	7:50	RBNU
IB	70735	R	RMB	7:50	RBNU
IB	70735	R	RMB	7:50	RBNU
IB	70735	R	RMB	7:50	RBNU
IB	70735	R	RMB	7:50	RBNU
IB	70735	R	RMB	7:50	RBNU
IB	70735	R	RMB	7:50	RBNU
IB	70735	R	RMB	7:50	RBNU
IB	70735	R	RMB	7:50	RBNU
IB	70735	R	RMB	7:50	RBNU
IB	70735	R	RMB	7:50	RBNU
IB	70735	R	RMB	7:50	RBNU
IB	70735	R	RMB	7:50	RBNU
IB	70735	R	RMB	7:50	RBNU
IB	70735	R	RMB	7:50	RBNU
IB	70735	R	RMB	7:50	MOCH
IB	70735	R	RMB	7:50	MOCH
IB	70735	R	RMB	7:50	MOCH
IB	70735	R	RMB	7:50	MOCH
IB	70735	R	RMB	7:50	BCCH

IB	70735	R			RMB	7:50	BCCH
IB	70735	R			RMB	7:50	RBNU
IB	70735	R			RMB	7:50	RBNU
IB	70735	R			RMB	7:50	RBNU
IB	70735	R			RMB	7:50	RBNU
IB	70735	R			RMB	7:50	RBNU
IB	70735	R			RMB	7:50	RBNU
IB	70735	R			RMB	7:50	RBNU
IB	70735	R			RMB	7:50	RBNU
IB	70735	R			RMB	7:50	RBNU
IB	70735	R			RMB	7:50	RBNU
IB	70735	R			RMB	7:50	RBNU
IB	70735	R			RMB	7:50	RBNU
IB	70735	R			RMB	7:50	RBNU
IB	70735	R			RMB	7:50	RBNU
IB	70735	R			RMB	7:50	RBNU
IB	70735	R			RMB	7:50	RBNU
IB	70735	R			RMB	7:50	RBNU
IB	70735	R			RMB	7:50	BCCH
IB	70735	R			RMB	7:50	BCCH
IB	70735	R			RMB	7:50	MOCH
IB	70735	R			RMB	7:50	MOCH
IB	70735	R			RMB	7:50	MOCH
IB	70735	R			RMB	7:50	BCCH
IB	70735	R			RMB	7:50	BCCH
IB	70735	R			RMB	7:50	MOCH
IB	70735	R			RMB	7:50	BCCH
IB	70735	R			RMB	7:50	RBNU
IB	70735	R			RMB	7:50	MOCH
IB	70735	R			RMB	7:50	BCCH
IB	70735	R			RMB	7:50	BCCH
IB	70735	R			RMB	7:50	
IB	70420	R	RBNU	2006	BMR	11:18	RBNU
IB	70420	R	RBNU	2006	BMR	11:18	RBNU
IB	70420	R	RBNU	2006	BMR	11:18	RBNU
IB	70420	R	RBNU	2006	BMR	11:18	RBNU
IB	70420	R	RBNU	2006	BMR	11:18	RBNU
IB	70420	R	RBNU	2006	BMR	11:18	MOCH
IB	70420	R	RBNU	2006	BMR	11:18	MOCH
IB	70420	R	RBNU	2006	BMR	11:18	MOCH
IB	70420	R	RBNU	2006	BMR	11:18	MOCH
IB	70420	R	RBNU	2006	BMR	11:18	BCCH
IB	70420	R	RBNU	2006	BMR	11:18	MOCH
IB	70420	R	RBNU	2006	BMR	11:18	RBNU

IB	70405	R	RBNU	2005	BMR	9:58	MOCH
IB	70405	R	RBNU	2005	BMR	9:58	MOCH
IB	70405	R	RBNU	2005	BMR	9:58	MOCH
IB	70405	R	RBNU	2005	BMR	9:58	MOCH
IB	70405	R	RBNU	2005	BMR	9:58	MOCH
IB	70405	R	RBNU	2005	BMR	9:58	MOCH
IB	70405	R	RBNU	2005	BMR	9:58	BCCH
IB	70405	R	RBNU	2005	BMR	9:58	BCCH
IB	70405	R	RBNU	2005	BMR	9:58	RBNU
IB	70405	R	RBNU	2005	BMR	9:58	RBNU
IB	70405	R	RBNU	2005	BMR	9:58	RBNU
IB	70405	R	RBNU	2005	BMR	9:58	RBNU
IB	70405	R	RBNU	2005	BMR	9:58	RBNU
IB	70405	R	RBNU	2005	BMR	9:58	BCCH
IB	70405	R	RBNU	2005	BMR	9:58	RBNU
IB	70405	R	RBNU	2005	BMR	9:58	RBNU
IB	70405	R	RBNU	2005	BMR	9:58	RBNU
IB	70405	R	RBNU	2005	BMR	9:58	RBNU
IB	70405	R	RBNU	2005	BMR	9:58	BCCH
IB	70405	R	RBNU	2005	BMR	9:58	MOCH
IB	70405	R	RBNU	2005	BMR	9:58	MOCH
IB	70405	R	RBNU	2005	BMR	9:58	BCCH
IB	70405	R	RBNU	2005	BMR	9:58	BCCH
IB	70405	R	RBNU	2005	BMR	9:58	BCCH
IB	70405	R	RBNU	2005	BMR	9:58	BCCH
IB	70405	R	RBNU	2005	BMR	9:58	BCCH
IB	70405	R	RBNU	2005	BMR	9:58	MOCH
IB	70405	R	RBNU	2005	BMR	9:58	RBNU
IB	7888	R	TRES	2006	RBM	10:25	BCCH
IB	7888	R	TRES	2006	RBM	10:25	RBNU
IB	7888	R	TRES	2006	RBM	10:25	RBNU
IB	7888	R	TRES	2006	RBM	10:25	RBNU
IB	7888	R	TRES	2006	RBM	10:25	RBNU
IB	7888	R	TRES	2006	RBM	10:25	RBNU
IB	7888	R	TRES	2006	RBM	10:25	RBNU
IB	7888	R	TRES	2006	RBM	10:25	RBNU
IB	7888	R	TRES	2006	RBM	10:25	RBNU
IB	7888	R	TRES	2006	RBM	10:25	BCCH
IB	7888	R	TRES	2006	RBM	10:25	MOCH
IB	70147R	R			MRB	10:15	BCCH
ARN, IB		R			MRB	10:15 AM	BCCH
IB	70147R	R			MRB	10:15	BCCH
ARN, IB		R			MRB	10:15 AM	BCCH
IB	70147R	R			MRB	10:15	BCCH

ARN, IB		R	MRB	10:15 AM	BCCH
IB	70147R	R	MRB	10:15	BCCH
ARN, IB		R	MRB	10:15 AM	BCCH
IB	70147R	R	MRB	10:15	RBNU
ARN, IB		R	MRB	10:15 AM	RBNU
IB	70147R	R	MRB	10:15	BCCH
ARN, IB		R	MRB	10:15 AM	BCCH
IB	70147R	R	MRB	10:15	MOCH
IB	70147R	R	MRB	10:15	MOCH
ARN, IB		R	MRB	10:15 AM	MOCH
IB	70147R	R	MRB	10:15	MOCH
IB	70147R	R	MRB	10:15	MOCH
IB	70147R	R	MRB	10:15	MOCH
ARN, IB		R	MRB	10:15 AM	MOCH
ARN, IB		R	MRB	10:15 AM	MOCH
ARN, IB		R	MRB	10:15 AM	MOCH
IB	70147R	R	MRB	10:15	RBNU
IB	70147R	R	MRB	10:15	BCCH
ARN, IB		R	MRB	10:15 AM	RBNU
ARN, IB		R	MRB	10:15 AM	BCCH
IB	70147R	R	MRB	10:15	BCCH
ARN, IB		R	MRB	10:15 AM	BCCH
IB	70147R	R	MRB	10:15	RBNU
ARN, IB		R	MRB	10:15 AM	RBNU
IB	70147R	R	MRB	10:15	RBNU
ARN, IB		R	MRB	10:15 AM	RBNU
IB	70147R	R	MRB	10:15	RBNU
ARN, IB		R	MRB	10:15 AM	RBNU
IB	70147R	R	MRB	10:15	BCCH
ARN, IB		R	MRB	10:15 AM	BCCH
IB	70147R	R	MRB	10:15	BCCH
ARN, IB		R	MRB	10:15 AM	BCCH
IB	70147R	R	MRB	10:15	MOCH
IB	70147R	R	MRB	10:15	MOCH
IB	70147R	R	MRB	10:15	BCCH
ARN, IB		R	MRB	10:15 AM	MOCH
ARN, IB		R	MRB	10:15 AM	MOCH
ARN, IB		R	MRB	10:15 AM	BCCH
IB	70147R	R	MRB	10:15	RBNU
IB	70147R	R	MRB	10:15	BCCH
ARN, IB		R	MRB	10:15 AM	MOCH
ARN, IB		R	MRB	10:15 AM	RBNU
ARN, IB		R	MRB	10:15 AM	BCCH
IB	70147R	R	MRB	10:15	BCCH

IB	70147R	R	MRB	10:15	
ARN, IB		R	BMR	9:45 AM	BCCH
ARN, IB		R	BMR	9:45 AM	BCCH
ARN, IB		R	BMR	9:45 AM	BCCH
ARN, IB		R	BMR	9:45 AM	MOCH
ARN, IB		R	BMR	9:45 AM	MOCH
ARN, IB		R	BMR	9:45 AM	MOCH
ARN, IB		R	BMR	9:45 AM	MOCH
ARN, IB		R	BMR	9:45 AM	MOCH
ARN, IB		R	BMR	9:45 AM	MOCH
ARN, IB		R	BMR	9:45 AM	MOCH
ARN, IB		R	BMR	9:45 AM	MOCH
ARN, IB		R	BMR	9:45 AM	MOCH
ARN, IB		R	BMR	9:45 AM	RBNU
ARN, IB		R	BMR	9:45 AM	MOCH
ARN, IB		R	BMR	9:45 AM	RBNU
ARN, IB		R	BMR	9:45 AM	RBNU
ARN, IB		R	BMR	9:45 AM	RBNU
ARN, IB		R	BMR	9:45 AM	BCCH
ARN, IB		R	BMR	9:45 AM	BCCH
ARN, IB		R	BMR	9:45 AM	BCCH
ARN, IB		R	BMR	9:45 AM	BCCH
ARN, IB		R	BMR	9:45 AM	BCCH
ARN, IB		R	BMR	9:45 AM	MOCH
ARN, IB		R	BMR	9:45 AM	RBNU
ARN, IB		R	BMR	9:45 AM	RBNU
ARN, IB		R	BMR	9:45 AM	MOCH
ARN, IB		R	BMR	9:45 AM	RBNU
ARN, IB		R	BMR	9:45 AM	RBNU
ARN, IB		R	BMR	9:45 AM	BCCH
ARN, IB		R	BMR	9:45 AM	MOCH
ARN, IB		R	BMR	9:45 AM	RBNU
ARN, IB		R	BMR	9:45 AM	MOCH
IB	7563R	R	BMR	9:57	MOCH
ARN, IB	7563R	R	BMR	9:57 AM	MOCH
IB	7563R	R	BMR	9:57	BCCH
ARN, IB	7563R	R	BMR	9:57 AM	BCCH
IB	7563R	R	BMR	9:57	MOCH
IB	7563R	R	BMR	9:57	RBNU
ARN, IB	7563R	R	BMR	9:57 AM	RBNU
IB	7563R	R	BMR	9:57	RBNU
ARN, IB	7563R	R	BMR	9:57 AM	RBNU
IB	7563R	R	BMR	9:57	RBNU
ARN, IB	7563R	R	BMR	9:57 AM	RBNU

IB	7563R	R	BMR	9:57	BCCH
ARN, IB	7563R	R	BMR	9:57 AM	BCCH
IB	7563R	R	BMR	9:57	RBNU
ARN, IB	7563R	R	BMR	9:57 AM	RBNU
IB	7563R	R	BMR	9:57	BCCH
ARN, IB	7563R	R	BMR	9:57 AM	BCCH
IB	7563R	R	BMR	9:57	BCCH
IB	7563R	R	BMR	9:57	MOCH
IB	7563R	R	BMR	9:57	RBNU
ARN, IB	7563R	R	BMR	9:57 AM	BCCH
ARN, IB	7563R	R	BMR	9:57 AM	MOCH
ARN, IB	7563R	R	BMR	9:57 AM	RBNU
IB	7563R	R	BMR	9:57	RBNU
IB	7563R	R	BMR	9:57	
IB	7406R	R	RMB	8:20 AM	BCCH
IB	7406R	R	RMB	8:20 AM	BCCH
IB	7406R	R	RMB	8:20 AM	BCCH
IB	7406R	R	RMB	8:20 AM	BCCH
IB	7406R	R	RMB	8:20 AM	BCCH
IB	7406R	R	RMB	8:20 AM	MOCH
IB	7406R	R	RMB	8:20 AM	MOCH
IB	7406R	R	RMB	8:20 AM	MOCH
IB	7406R	R	RMB	8:20 AM	BCCH
IB	7406R	R	RMB	8:20 AM	MOCH
IB	7406R	R	RMB	8:20 AM	MOCH
IB	7406R	R	RMB	8:20 AM	MOCH
IB	7406R	R	RMB	8:20 AM	MOCH
IB	7406R	R	RMB	8:20 AM	MOCH
IB	7406R	R	RMB	8:20 AM	MOCH
IB	7406R	R	RMB	8:20 AM	MOCH
IB	7406R	R	RMB	8:20 AM	BCCH
IB	7406R	R	RMB	8:20 AM	MOCH
IB	7406R	R	RMB	8:20 AM	MOCH
IB	7406R	R	RMB	8:20 AM	BCCH
IB	7406R	R	RMB	8:20 AM	RBNU
IB	7406R	R	RMB	8:20 AM	MOCH
IB	7406R	R	RMB	8:20 AM	BCCH
IB	70004R	R	MBR		MOCH
IB	70004R	R	MBR		MOCH
IB	70004R	R	MBR		BCCH
IB	70004R	R	MBR		BCCH
IB	70004R	R	MBR		MOCH
IB	70004R	R	MBR		BCCH
IB	70004R	R	MBR		BCCH

IB	70004R	R	MBR		MOCH
IB	70004R	R	MBR		MOCH
IB	70004R	R	MBR		BCCH
IB	70004R	R	MBR		BCCH
IB	70004R	R	MBR		MOCH
IB	70004R	R	MBR		BCCH
IB	70004R	R	MBR		BCCH
IB	70004R	R	MBR		MOCH
IB	70004R	R	MBR		BCCH
IB	70004R	R	MBR		BCCH
IB	70004R	R	MBR		BCCH
IB	70004R	R	MBR		BCCH
IB	70004R	R	MBR		BCCH
IB	70004R	R	MBR		BCCH
IB	70004R	R	MBR		BCCH
IB	70004R	R	MBR		BCCH
IB	70004R	R	MBR		BCCH
IB	70004R	R	MBR		BCCH
IB	70004R	R	MBR		BCCH
IB	70004R	R	MBR		BCCH
IB	70004R	R	MBR		MOCH
IB	70004R	R	MBR		MOCH
IB	70004R	R	MBR		BCCH
IB	70004R	R	MBR		BCCH
IB	70004R	R	MBR		BCCH
IB	70004R	R	MBR		MOCH
IB	70004R	R	MBR		BCCH
IB	70004R	R	MBR		BCCH
IB	70004R	R	MBR		MOCH
IB	70004R	R	MBR		BCCH
IB	7865R	R	BMR	9:46 AM	BCCH
IB	7865R	R	BMR	9:46 AM	MOCH
IB	7865R	R	BMR	9:46 AM	MOCH
IB	7865R	R	BMR	9:46 AM	MOCH
IB	7865R	R	BMR	9:46 AM	MOCH
IB	7865R	R	BMR	9:46 AM	MOCH
IB	7865R	R	BMR	9:46 AM	MOCH
IB	7865R	R	BMR	9:46 AM	MOCH
IB	7865R	R	BMR	9:46 AM	MOCH
IB	7865R	R	BMR	9:46 AM	RBNU
IB	7865R	R	BMR	9:46 AM	RBNU
IB	7865R	R	BMR	9:46 AM	RBNU
IB	7865R	R	BMR	9:46 AM	MOCH
IB	7865R	R	BMR	9:46 AM	RBNU

IB	7865R	R	BMR	9:46 AM	RBNU
IB	7865R	R	BMR	9:46 AM	MOCH
IB	7865R	R	BMR	9:46 AM	RBNU
IB	7865R	R	BMR	9:46 AM	BCCH
IB	7865R	R	BMR	9:46 AM	MOCH
IB	7865R	R	BMR	9:46 AM	RBNU
IB	70003R	R	BMR	11:00 AM	MOCH
IB	70003R	R	BMR	11:00 AM	MOCH
IB	70003R	R	BMR	11:00 AM	MOCH
IB	70003R	R	BMR	11:00 AM	MOCH
IB	70003R	R	BMR	11:00 AM	MOCH
IB	70003R	R	BMR	11:00 AM	MOCH
IB	70003R	R	BMR	11:00 AM	MOCH
IB	70003R	R	BMR	11:00 AM	MOCH
IB	70003R	R	BMR	11:00 AM	MOCH
IB	70003R	R	BMR	11:00 AM	MOCH
IB	70003R	R	BMR	11:00 AM	MOCH
IB	70003R	R	BMR	11:00 AM	MOCH
IB	70003R	R	BMR	11:00 AM	MOCH
IB	70003R	R	BMR	11:00 AM	MOCH
IB	70003R	R	BMR	11:00 AM	MOCH
IB	70003R	R	BMR	11:00 AM	MOCH
IB	70003R	R	BMR	11:00 AM	MOCH
IB	70003R	R	BMR	11:00 AM	MOCH
IB	70003R	R	BMR	11:00 AM	MOCH
IB	70003R	R	BMR	11:00 AM	MOCH
IB	70003R	R	BMR	11:00 AM	MOCH
IB	70003R	R	BMR	11:00 AM	MOCH
IB	70003R	R	BMR	11:00 AM	MOCH
IB	70003R	R	BMR	11:00 AM	MOCH
IB	70003R	R	BMR	11:00 AM	BCCH
IB	70003R	R	BMR	11:00 AM	MOCH
ARN	7972R	R	BMR	9:40 AM	RBNU
ARN	7972R	R	BMR	9:40 AM	RBNU
ARN	7972R	R	BMR	9:40 AM	RBNU
ARN	7972R	R	BMR	9:40 AM	RBNU
ARN	7972R	R	BMR	9:40 AM	RBNU
ARN	7972R	R	BMR	9:40 AM	MOCH
ARN	7972R	R	BMR	9:40 AM	MOCH
ARN	7972R	R	BMR	9:40 AM	MOCH
ARN	7972R	R	BMR	9:40 AM	RBNU
ARN	7972R	R	BMR	9:40 AM	BCCH
ARN	7972R	R	BMR	9:40 AM	RBNU
ARN	7972R	R	BMR	9:40 AM	BCCH
ARN	7972R	R	BMR	9:40 AM	MOCH

ARN	7972R	R			BMR	9:40 AM	RBNU
ARN	7972R	R			BMR	9:40 AM	RBNU
ARN	7972R	R			BMR	9:40 AM	BCCH
ARN	7972R	R			BMR	9:40 AM	MOCH
ARN	7972R	R			BMR	9:40 AM	RBNU
ARN	7972R	R			BMR	9:40 AM	
IB	70133R	R			MBR	11:00 AM	BCCH
IB	70133R	R			MBR	11:00 AM	RBNU
IB	70133R	R			MBR	11:00 AM	MOCH
IB	70133R	R			MBR	11:00 AM	BCCH
IB	70133R	R			MBR	11:00 AM	RBNU
IB	70163R	R			RMB		RBNU
IB	70163R	R			RMB		RBNU
IB	70163R	R			RMB		RBNU
IB	70163R	R			RMB		RBNU
IB	70163R	R			RMB		RBNU
IB	70163R	R			RMB		RBNU
IB	70163R	R			RMB		MOCH
IB	70163R	R			RMB		MOCH
IB	70163R	R			RMB		RBNU
IB	70163R	R			RMB		MOCH
IB	70163R	R			RMB		BCCH
IB	70163R	R			RMB		BCCH
IB	70163R	R			RMB		RBNU
IB	70163R	R			RMB		MOCH
IB	70163R	R			RMB		RBNU
IB	70163R	R			RMB		MOCH
IB	70163R	R			RMB		BCCH
IB	7080/7360	R	MOCH	1995	BRM	7:42 AM	MOCH
IB	7080/7360	R	MOCH	1995	BRM	7:42 AM	MOCH
IB	7080/7360	R	MOCH	1995	BRM	7:42 AM	BCCH
IB	7080/7360	R	MOCH	1995	BRM	7:42 AM	MOCH
IB	7080/7360	R	MOCH	1995	BRM	7:42 AM	RBNU
IB	7080/7360	R	MOCH	1995	BRM	7:42 AM	RBNU
IB	7080/7360	R	MOCH	1995	BRM	7:42 AM	RBNU
IB	7080/7360	R	MOCH	1995	BRM	7:42 AM	RBNU
IB	7080/7360	R	MOCH	1995	BRM	7:42 AM	RBNU
IB	7080/7360	R	MOCH	1995	BRM	7:42 AM	RBNU
IB	7080/7360	R	MOCH	1995	BRM	7:42 AM	MOCH
IB	7080/7360	R	MOCH	1995	BRM	7:42 AM	MOCH
IB	7080/7360	R	MOCH	1995	BRM	7:42 AM	RBNU
IB	7080/7360	R	MOCH	1995	BRM	7:42 AM	MOCH
IB	7080/7360	R	MOCH	1995	BRM	7:42 AM	RBNU
IB	7080/7360	R	MOCH	1995	BRM	7:42 AM	RBNU

IB	7080/7360	R	MOCH	1995	BRM	7:42 AM	MOCH
IB	7080/7360	R	MOCH	1995	BRM	7:42 AM	MOCH
IB	7080/7360	R	MOCH	1995	BRM	7:42 AM	MOCH
IB	7080/7360	R	MOCH	1995	BRM	7:42 AM	MOCH
IB	7080/7360	R	MOCH	1995	BRM	7:42 AM	MOCH
IB	7080/7360	R	MOCH	1995	BRM	7:42 AM	RBNU
IB	7080/7360	R	MOCH	1995	BRM	7:42 AM	MOCH
IB	7080/7360	R	MOCH	1995	BRM	7:42 AM	MOCH
IB	7080/7360	R	MOCH	1995	BRM	7:42 AM	MOCH
IB	7080/7360	R	MOCH	1995	BRM	7:42 AM	MOCH
IB	7080/7360	R	MOCH	1995	BRM	7:42 AM	RBNU
IB	7080/7360	R	MOCH	1995	BRM	7:42 AM	RBNU
IB	7080/7360	R	MOCH	1995	BRM	7:42 AM	MOCH
IB	7080/7360	R	MOCH	1995	BRM	7:42 AM	MOCH
IB	7080/7360	R	MOCH	1995	BRM	7:42 AM	BCCH
IB	7080/7360	R	MOCH	1995	BRM	7:42 AM	RBNU
IB	7080/7360	R	MOCH	1995	BRM	7:42 AM	MOCH
ARN	7744R	R			RBM	8:26 AM	MOCH
ARN	7744R	R			RBM	8:26 AM	BCCH
ARN	7744R	R			RBM	8:26 AM	BCCH
ARN	7744R	R			RBM	8:26 AM	RBNU
ARN	7744R	R			RBM	8:26 AM	BCCH
ARN	7744R	R			RBM	8:26 AM	MOCH
ARN	7744R	R			RBM	8:26 AM	RBNU
ARN	7349R	R				9:33 AM	RBNU
ARN	7349R	R				9:33 AM	MOCH
ARN	7349R	R				9:33 AM	MOCH
ARN	7349R	R				9:33 AM	MOCH
ARN	7349R	R				9:33 AM	MOCH
ARN	7349R	R				9:33 AM	MOCH
ARN	7349R	R				9:33 AM	BCCH
ARN	7349R	R				9:33 AM	MOCH
ARN	7349R	R				9:33 AM	RBNU
ARN	7349R	R				9:33 AM	
IB	7827/7440	R	RBNU	2003	MBR	9:58 AM	MOCH
IB	7827/7440	R	RBNU	2003	MBR	9:58 AM	BCCH
IB	7827/7440	R	RBNU	2003	MBR	9:58 AM	RBNU
IB	7512R	R			RBM	10:00 AM	MOCH
IB	7512R	R			RBM	10:00 AM	MOCH
IB	7512R	R			RBM	10:00 AM	MOCH
IB	7512R	R			RBM	10:00 AM	RBNU
IB	7512R	R			RBM	10:00 AM	BCCH
IB	7512R	R			RBM	10:00 AM	MOCH
IB	7840R	R			MBR	8:57 AM	BCCH

IB	7840R	R	MBR	8:57 AM	BCCH
IB	7840R	R	MBR	8:57 AM	BCCH
IB	7840R	R	MBR	8:57 AM	BCCH
IB	7840R	R	MBR	8:57 AM	BCCH
IB	7840R	R	MBR	8:57 AM	BCCH
IB	7840R	R	MBR	8:57 AM	BCCH
IB	7840R	R	MBR	8:57 AM	BCCH
IB	7840R	R	MBR	8:57 AM	BCCH
IB	7840R	R	MBR	8:57 AM	BCCH
IB	7840R	R	MBR	8:57 AM	BCCH
IB	7840R	R	MBR	8:57 AM	MOCH
IB	7840R	R	MBR	8:57 AM	MOCH
IB	7840R	R	MBR	8:57 AM	BCCH
IB	7840R	R	MBR	8:57 AM	RBNU
IB	70735R	R	MBR	8:09 AM	MOCH
IB	70735R	R	MBR	8:09 AM	RBNU
IB	70735R	R	MBR	8:09 AM	RBNU
IB	70735R	R	MBR	8:09 AM	RBNU
IB	70735R	R	MBR	8:09 AM	RBNU
IB	70735R	R	MBR	8:09 AM	BCCH
IB	70735R	R	MBR	8:09 AM	MOCH
IB	70735R	R	MBR	8:09 AM	RBNU
IB	70900R	R	MRB	11:03 AM	RBNU
IB	70900R	R	MRB	11:03 AM	BCCH
IB	70900R	R	MRB	11:03 AM	RBNU
IB	70900R	R	MRB	11:03 AM	RBNU
IB	70900R	R	MRB	11:03 AM	RBNU
IB	70900R	R	MRB	11:03 AM	RBNU
IB	70900R	R	MRB	11:03 AM	BCCH
IB	70900R	R	MRB	11:03 AM	BCCH
IB	70900R	R	MRB	11:03 AM	BCCH
IB	70900R	R	MRB	11:03 AM	RBNU
IB	70900R	R	MRB	11:03 AM	RBNU
IB	70900R	R	MRB	11:03 AM	RBNU
IB	70900R	R	MRB	11:03 AM	RBNU
IB	70900R	R	MRB	11:03 AM	RBNU
IB	70900R	R	MRB	11:03 AM	RBNU
IB	70900R	R	MRB	11:03 AM	BCCH
IB	70900R	R	MRB	11:03 AM	RBNU
IB	70900R	R	MRB	11:03 AM	RBNU
IB	70900R	R	MRB	11:03 AM	MOCH
IB	70900R	R	MRB	11:03 AM	BCCH
IB	70900R	R	MRB	11:03 AM	BCCH
IB	70900R	R	MRB	11:03 AM	BCCH

IB	70900R	R	MRB	11:03 AM	RBNU
IB	70900R	R	MRB	11:03 AM	RBNU
IB	70900R	R	MRB	11:03 AM	MOCH
IB	70900R	R	MRB	11:03 AM	RBNU
IB	70005R?	R	RMB	8:30 AM	RBNU
IB	70005R?	R	RMB	8:30 AM	RBNU
IB	70005R?	R	RMB	8:30 AM	RBNU
IB	70005R?	R	RMB	8:30 AM	BCCH
IB	70005R?	R	RMB	8:30 AM	MOCH
IB	70005R?	R	RMB	8:30 AM	RBNU
IB		R	RBM	9:07 AM	BCCH
IB		R	RBM	9:07 AM	BCCH
IB		R	RBM	9:07 AM	BCCH
IB		R	RBM	9:07 AM	MOCH
IB		R	RBM	9:07 AM	RBNU
IB		R	RBM	9:07 AM	RBNU
IB	70102R	R	BMR	8:10 AM	BCCH
IB	70102R	R	BMR	8:10 AM	MOCH
IB	70102R	R	BMR	8:10 AM	RBNU
IB	7360R	R	BMR	9:38 AM	MOCH
IB	7360R	R	BMR	9:38 AM	MOCH
IB	7360R	R	BMR	9:38 AM	MOCH
IB	7360R	R	BMR	9:38 AM	BCCH
IB	7360R	R	BMR	9:38 AM	MOCH
IB	7360R	R	BMR	9:38 AM	RBNU
IB	7360R	R	BMR	9:38 AM	RBNU
IB	7440R	R	RMB	11:00 AM	BCCH
IB	7440R	R	RMB	11:00 AM	MOCH
IB	7440R	R	RMB	11:00 AM	RBNU
HK	7839R	R	BMR	11:18	BCCH
HK	7839R	R	BMR	11:18	MOCH
HK	7839R	R	BMR	11:18	MOCH
HK	7839R	R	BMR	11:18	MOCH
HK	7839R	R	BMR	11:18	MOCH
HK	7839R	R	BMR	11:18	MOCH
HK	7839R	R	BMR	11:18	MOCH
HK	7839R	R	BMR	11:18	MOCH
HK	7839R	R	BMR	11:18	
HK	7839R	R	BMR	11:18	MOCH
HK	7839R	R	BMR	11:18	BCCH
HK	7839R	R	BMR	11:18	RBNU
HK	7839R	R	BMR	11:18	BCCH
HK	7839R	R	BMR	11:18	BCCH
HK	7839R	R	BMR	11:18	MOCH

HK	7839R	R			BMR	11:18	RBNU
HK	7839R	R			BMR	11:18	RBNU
HK	7839R	R			BMR	11:18	RBNU
HK	7839R	R			BMR	11:18	
HK	70102R/ 7(	R	MOBL	2006	BRM	11:55	BCCH
HK	70102R/ 7(	R	MOBL	2006	BRM	11:55	MOCH
HK	70102R/ 7(	R	MOBL	2006	BRM	11:55	MOCH
HK	70102R/ 7(	R	MOBL	2006	BRM	11:55	BCCH
HK	70102R/ 7(	R	MOBL	2006	BRM	11:55	BCCH
HK	70102R/ 7(	R	MOBL	2006	BRM	11:55	BCCH
HK	70102R/ 7(	R	MOBL	2006	BRM	11:55	BCCH
HK	70102R/ 7(	R	MOBL	2006	BRM	11:55	RBNU
HK	70102R/ 7(	R	MOBL	2006	BRM	11:55	MOCH
HK	70102R/ 7(	R	MOBL	2006	BRM	11:55	MOCH
HK	70102R/ 7(	R	MOBL	2006	BRM	11:55	BCCH
HK	70102R/ 7(	R	MOBL	2006	BRM	11:55	
HK	7403R	R			BRM	10:23	RBNU
HK	7403R	R			BRM	10:23	RBNU
HK	7403R	R			BRM	10:23	RBNU
HK	7403R	R			BRM	10:23	BCCH
HK	7403R	R			BRM	10:23	MOCH
HK	7403R	R			BRM	10:23	MOCH
HK	7403R	R			BRM	10:23	MOCH
HK	7403R	R			BRM	10:23	MOCH
HK	7403R	R			BRM	10:23	MOCH
HK	7403R	R			BRM	10:23	MOCH
HK	7403R	R			BRM	10:23	MOCH
HK	7403R	R			BRM	10:23	MOCH
HK	7403R	R			BRM	10:23	MOCH
HK	7403R	R			BRM	10:23	RBNU
HK	7403R	R			BRM	10:23	RBNU
HK	7403R	R			BRM	10:23	RBNU
HK	7403R	R			BRM	10:23	RBNU
HK	7403R	R			BRM	10:23	RBNU
HK	7403R	R			BRM	10:23	RBNU
HK	7403R	R			BRM	10:23	RBNU
HK	7403R	R			BRM	10:23	RBNU
HK	7403R	R			BRM	10:23	RBNU
HK	7403R	R			BRM	10:23	MOCH
HK	7403R	R			BRM	10:23	MOCH
HK	7403R	R			BRM	10:23	RBNU
HK	7403R	R			BRM	10:23	MOCH
HK	7403R	R			BRM	10:23	RBNU
HK	7403R	R			BRM	10:23	BCCH

HK	7403R	R	BRM	10:23	RBNU
HK	7403R	R	BRM	10:23	MOCH
HK	7403R	R	BRM	10:23	MOCH
HK	7403R	R	BRM	10:23	
HK	7028R	R	RBM	12:03	MOCH
HK	7028R	R	RBM	12:03	RBNU
HK	7028R	R	RBM	12:03	MOCH
HK	7028R	R	RBM	12:03	MOCH
HK	7028R	R	RBM	12:03	BCCH
HK	7028R	R	RBM	12:03	BCCH
HK	7028R	R	RBM	12:03	BCCH
HK	7028R	R	RBM	12:03	BCCH
HK	7028R	R	RBM	12:03	BCCH
HK	7028R	R	RBM	12:03	MOCH
HK	7028R	R	RBM	12:03	MOCH
HK	7028R	R	RBM	12:03	BCCH
HK	7028R	R	RBM	12:03	BCCH
HK	7028R	R	RBM	12:03	MOCH
HK	7028R	R	RBM	12:03	RBNU
HK	7028R	R	RBM	12:03	RBNU
HK	7028R	R	RBM	12:03	RBNU
HK	7028R	R	RBM	12:03	RBNU
HK	7028R	R	RBM	12:03	RBNU
HK	7028R	R	RBM	12:03	RBNU
HK	7028R	R	RBM	12:03	RBNU
HK	7028R	R	RBM	12:03	RBNU
HK	7028R	R	RBM	12:03	RBNU
HK	7028R	R	RBM	12:03	BCCH
HK	7028R	R	RBM	12:03	MOCH
HK	7028R	R	RBM	12:03	MOCH
HK	7028R	R	RBM	12:03	
HK	70196R	R	MRB	9:06	BCCH
HK	70196R	R	MRB	9:06	MOCH
HK	70196R	R	MRB	9:06	MOCH
HK	70196R	R	MRB	9:06	BCCH
HK	70196R	R	MRB	9:06	MOCH
HK	70196R	R	MRB	9:06	MOCH
HK	70196R	R	MRB	9:06	MOCH
HK	70196R	R	MRB	9:06	MOCH
HK	70196R	R	MRB	9:06	MOCH
HK	70196R	R	MRB	9:06	RBNU
HK	70196R	R	MRB	9:06	MOCH
HK	70196R	R	MRB	9:06	RBNU
HK	70196R	R	MRB	9:06	BCCH

HK	70196R	R	MRB	9:06	BCCH
HK	70196R	R	MRB	9:06	RBNU
HK	70196R	R	MRB	9:06	RBNU
HK	70196R	R	MRB	9:06	RBNU
HK	70196R	R	MRB	9:06	RBNU
HK	70196R	R	MRB	9:06	RBNU
HK	70196R	R	MRB	9:06	BCCH
HK	70196R	R	MRB	9:06	RBNU
HK	70196R	R	MRB	9:06	BCCH
HK	70196R	R	MRB	9:06	RBNU
HK	70196R	R	MRB	9:06	RBNU
HK	70196R	R	MRB	9:06	RBNU
HK	70196R	R	MRB	9:06	RBNU
HK	70196R	R	MRB	9:06	BCCH
HK	70196R	R	MRB	9:06	RBNU
HK	70196R	R	MRB	9:06	BCCH
HK	70196R	R	MRB	9:06	BCCH
HK	70196R	R	MRB	9:06	MOCH
HK	70196R	R	MRB	9:06	BCCH
HK	70196R	R	MRB	9:06	BCCH
HK	70196R	R	MRB	9:06	MOCH
HK	70196R	R	MRB	9:06	RBNU
HK	70196R	R	MRB	9:06	BCCH
HK	70196R	R	MRB	9:06	BCCH
HK	70196R	R	MRB	9:06	
HK	7063R	R	RBM	10:36	BCCH
HK	7063R	R	RBM	10:36	BCCH
HK	7063R	R	RBM	10:36	MOCH
HK	7063R	R	RBM	10:36	MOCH
HK	7063R	R	RBM	10:36	RBNU
HK	7063R	R	RBM	10:36	RBNU
HK	7063R	R	RBM	10:36	RBNU
HK	7063R	R	RBM	10:36	RBNU
HK	7063R	R	RBM	10:36	BCCH
HK	7063R	R	RBM	10:36	BCCH
HK	7063R	R	RBM	10:36	MOCH
HK	7063R	R	RBM	10:36	MOCH
HK	7063R	R	RBM	10:36	BCCH
HK	7063R	R	RBM	10:36	MOCH
HK	7063R	R	RBM	10:36	RBNU
HK	7063R	R	RBM	10:36	BCCH
HK	7063R	R	RBM	10:36	MOCH
HK	7063R	R	RBM	10:36	MOCH
HK	7063R	R	RBM	10:36	

HK	AFC R	R	MRB	10:37	BCCH
HK	AFC R	R	MRB	10:37	BCCH
HK	AFC R	R	MRB	10:37	MOCH
HK	AFC R	R	MRB	10:37	MOCH
HK	AFC R	R	MRB	10:37	MOCH
HK	AFC R	R	MRB	10:37	BCCH
HK	AFC R	R	MRB	10:37	MOCH
HK	AFC R	R	MRB	10:37	BCCH
HK	AFC R	R	MRB	10:37	MOCH
HK	AFC R	R	MRB	10:37	BCCH
HK	AFC R	R	MRB	10:37	RBNU
HK	AFC R	R	MRB	10:37	BCCH
HK	AFC R	R	MRB	10:37	RBNU
HK	AFC R	R	MRB	10:37	RBNU
HK	AFC R	R	MRB	10:37	RBNU
HK	AFC R	R	MRB	10:37	MOCH
HK	AFC R	R	MRB	10:37	RBNU
HK	AFC R	R	MRB	10:37	BCCH
HK	AFC R	R	MRB	10:37	BCCH
HK	AFC R	R	MRB	10:37	
HK	2006 RBNU	R	RBM	12:42	MOCH
HK	2006 RBNU	R	RBM	12:42	MOCH
HK	2006 RBNU	R	RBM	12:42	MOCH
HK	2006 RBNU	R	RBM	12:42	BCCH
HK	2006 RBNU	R	RBM	12:42	BCCH
HK	2006 RBNU	R	RBM	12:42	BCCH
HK	2006 RBNU	R	RBM	12:42	BCCH
HK	2006 RBNU	R	RBM	12:42	MOCH
HK	2006 RBNU	R	RBM	12:42	BCCH
HK	2006 RBNU	R	RBM	12:42	MOCH
HK	2006 RBNU	R	RBM	12:42	MOCH
HK	2006 RBNU	R	RBM	12:42	BCCH
HK	2006 RBNU	R	RBM	12:42	BCCH
HK	2006 RBNU	R	RBM	12:42	MOCH
HK	2006 RBNU	R	RBM	12:42	MOCH
HK	2006 RBNU	R	RBM	12:42	BCCH
HK	2006 RBNU	R	RBM	12:42	BCCH
HK	2006 RBNU	R	RBM	12:42	MOCH
HK	2006 RBNU	R	RBM	12:42	RBNU
HK	2006 RBNU	R	RBM	12:42	BCCH
HK	2006 RBNU	R	RBM	12:42	BCCH
HK	2006 RBNU	R	RBM	12:42	MOCH
HK	2006 RBNU	R	RBM	12:42	RBNU
HK	2006 RBNU	R	RBM	12:42	RBNU

HK	2006 RBNU	R	RBM	12:42	RBNU
HK	2006 RBNU	R	RBM	12:42	BCCH
HK	2006 RBNU	R	RBM	12:42	MOCH
HK	2006 RBNU	R	RBM	12:42	MOCH
HK	2006 RBNU	R	RBM	12:42	RBNU
HK	2006 RBNU	R	RBM	12:42	RBNU
HK	2006 RBNU	R	RBM	12:42	BCCH
HK	2006 RBNU	R	RBM	12:42	MOCH
HK	2006 RBNU	R	RBM	12:42	MOCH
HK	2006 RBNU	R	RBM	12:42	
HK	new RBNU	R	MRB	10:15	BCCH
HK	new RBNU	R	MRB	10:15	BCCH
HK	new RBNU	R	MRB	10:15	BCCH
HK	new RBNU	R	MRB	10:15	RBNU
HK	new RBNU	R	MRB	10:15	RBNU
HK	new RBNU	R	MRB	10:15	RBNU
HK	new RBNU	R	MRB	10:15	RBNU
HK	new RBNU	R	MRB	10:15	BCCH
HK	new RBNU	R	MRB	10:15	RBNU
HK	new RBNU	R	MRB	10:15	MOCH
HK	new RBNU	R	MRB	10:15	RBNU
HK	new RBNU	R	MRB	10:15	MOCH
HK	new RBNU	R	MRB	10:15	BCCH
HK	new RBNU	R	MRB	10:15	MOCH
HK	new RBNU	R	MRB	10:15	MOCH
HK	new RBNU	R	MRB	10:15	RBNU
HK	new RBNU	R	MRB	10:15	RBNU
HK	new RBNU	R	MRB	10:15	BCCH
HK	new RBNU	R	MRB	10:15	BCCH
HK	new RBNU	R	MRB	10:15	BCCH
HK	new RBNU	R	MRB	10:15	MOCH
HK	new RBNU	R	MRB	10:15	RBNU
HK	new RBNU	R	MRB	10:15	BCCH
HK	new RBNU	R	MRB	10:15	BCCH
HK	new RBNU	R	MRB	10:15	
HK	70705R	R	RMB	11:34	MOCH
HK	70705R	R	RMB	11:34	RBNU
HK	70705R	R	RMB	11:34	RBNU
HK	70705R	R	RMB	11:34	RBNU
HK	70705R	R	RMB	11:34	RBNU
HK	70705R	R	RMB	11:34	RBNU
HK	70705R	R	RMB	11:34	RBNU
HK	70705R	R	RMB	11:34	RBNU
HK	70705R	R	RMB	11:34	RBNU

HK	70705R	R	RMB	11:34	MOCH
HK	70705R	R	RMB	11:34	RBNU
HK	70705R	R	RMB	11:34	MOCH
HK	70705R	R	RMB	11:34	MOCH
HK	70705R	R	RMB	11:34	RBNU
HK	70705R	R	RMB	11:34	MOCH
HK	70705R	R	RMB	11:34	BCCH
HK	70705R	R	RMB	11:34	MOCH
HK	70705R	R	RMB	11:34	
HK	70415R	R	MBR	9:46	MOCH
HK	70415R	R	MBR	9:46	MOCH
HK	70415R	R	MBR	9:46	MOCH
HK	70415R	R	MBR	9:46	BCCH
HK	70415R	R	MBR	9:46	MOCH
HK	70415R	R	MBR	9:46	MOCH
HK	70415R	R	MBR	9:46	MOCH
HK	70415R	R	MBR	9:46	MOCH
HK	70415R	R	MBR	9:46	
HK	70415R	R	MBR	9:46	RBNU
HK	70415R	R	MBR	9:46	BCCH
HK	70415R	R	MBR	9:46	BCCH
HK	70415R	R	MBR	9:46	RBNU
HK	70415R	R	MBR	9:46	RBNU
HK	70415R	R	MBR	9:46	MOCH
HK	70415R	R	MBR	9:46	BCCH
HK	70415R	R	MBR	9:46	RBNU
HK	70415R	R	MBR	9:46	RBNU
HK	70415R	R	MBR	9:46	
HK	70813R	R	RMB	11:14	BCCH
HK	70813R	R	RMB	11:14	
HK	70813R	R	RMB	11:14	RBNU
HK	70813R	R	RMB	11:14	RBNU
HK	70813R	R	RMB	11:14	RBNU
HK	70813R	R	RMB	11:14	RBNU
HK	70813R	R	RMB	11:14	RBNU
HK	70813R	R	RMB	11:14	BCCH
HK	70813R	R	RMB	11:14	RBNU
HK	70813R	R	RMB	11:14	MOCH
HK	70813R	R	RMB	11:14	MOCH
HK	70813R	R	RMB	11:14	RBNU
HK	70813R	R	RMB	11:14	MOCH
HK	70813R	R	RMB	11:14	BCCH
HK	70813R	R	RMB	11:14	BCCH
HK	70813R	R	RMB	11:14	

HK	7086R	R	MRB	8:48	MOCH
HK	7086R	R	MRB	8:48	RBNU
HK	7086R	R	MRB	8:48	MOCH
HK	7086R	R	MRB	8:48	MOCH
HK	7086R	R	MRB	8:48	MOCH
HK	7086R	R	MRB	8:48	MOCH
HK	7086R	R	MRB	8:48	MOCH
HK	7086R	R	MRB	8:48	MOCH
HK	7086R	R	MRB	8:48	RBNU
HK	7086R	R	MRB	8:48	RBNU
HK	7086R	R	MRB	8:48	RBNU
HK	7086R	R	MRB	8:48	RBNU
HK	7086R	R	MRB	8:48	RBNU
HK	7086R	R	MRB	8:48	RBNU
HK	7086R	R	MRB	8:48	RBNU
HK	7086R	R	MRB	8:48	RBNU
HK	7086R	R	MRB	8:48	RBNU
HK	7086R	R	MRB	8:48	RBNU
HK	7086R	R	MRB	8:48	BCCH
HK	7086R	R	MRB	8:48	BCCH
HK	7086R	R	MRB	8:48	MOCH
HK	7086R	R	MRB	8:48	MOCH
HK	7086R	R	MRB	8:48	MOCH
HK	7086R	R	MRB	8:48	RBNU
HK	7086R	R	MRB	8:48	BCCH
HK	7086R	R	MRB	8:48	BCCH
HK	7086R	R	MRB	8:48	RBNU
HK	7086R	R	MRB	8:48	RBNU
HK	7086R	R	MRB	8:48	RBNU
HK	7086R	R	MRB	8:48	
HK	7803R	R	MBR	10:27	BCCH
HK	7803R	R	MBR	10:27	BCCH
HK	7803R	R	MBR	10:27	BCCH
HK	7803R	R	MBR	10:27	BCCH
HK	7803R	R	MBR	10:27	BCCH
HK	7803R	R	MBR	10:27	BCCH
HK	7803R	R	MBR	10:27	BCCH
HK	7803R	R	MBR	10:27	BCCH
HK	7803R	R	MBR	10:27	BCCH
HK	7803R	R	MBR	10:27	BCCH
HK	7803R	R	MBR	10:27	BCCH
HK	7803R	R	MBR	10:27	BCCH
HK	7803R	R	MBR	10:27	BCCH
HK	7803R	R	MBR	10:27	BCCH

HK	7803R	R	MBR	10:27	BCCH
HK	7803R	R	MBR	10:27	BCCH
HK	7803R	R	MBR	10:27	BCCH
HK	7803R	R	MBR	10:27	BCCH
HK	7803R	R	MBR	10:27	RBNU
HK	7803R	R	MBR	10:27	BCCH
HK	7803R	R	MBR	10:27	BCCH
HK	7803R	R	MBR	10:27	MOCH
HK	7803R	R	MBR	10:27	MOCH
HK	7803R	R	MBR	10:27	MOCH
HK	7803R	R	MBR	10:27	MOCH
HK	7803R	R	MBR	10:27	BCCH
HK	7803R	R	MBR	10:27	MOCH
HK	7803R	R	MBR	10:27	BCCH
HK	7803R	R	MBR	10:27	BCCH
HK	7803R	R	MBR	10:27	MOCH
HK	7803R	R	MBR	10:27	BCCH
HK	7803R	R	MBR	10:27	RBNU
HK	7803R	R	MBR	10:27	RBNU
HK	7803R	R	MBR	10:27	
HK	7972R/708	R	BMR	9:15	MOCH
HK	7972R/708	R	BMR	9:15	BCCH
HK	7972R/708	R	BMR	9:15	BCCH
HK	7972R/708	R	BMR	9:15	BCCH
HK	7972R/708	R	BMR	9:15	MOCH
HK	7972R/708	R	BMR	9:15	MOCH
HK	7972R/708	R	BMR	9:15	BCCH
HK	7972R/708	R	BMR	9:15	BCCH
HK	7972R/708	R	BMR	9:15	MOCH
HK	7972R/708	R	BMR	9:15	MOCH
HK	7972R/708	R	BMR	9:15	MOCH
HK	7972R/708	R	BMR	9:15	BCCH
HK	7972R/708	R	BMR	9:15	BCCH
HK	7972R/708	R	BMR	9:15	RBNU
HK	7972R/708	R	BMR	9:15	RBNU
HK	7972R/708	R	BMR	9:15	RBNU
HK	7972R/708	R	BMR	9:15	RBNU
HK	7972R/708	R	BMR	9:15	BCCH
HK	7972R/708	R	BMR	9:15	MOCH
HK	7972R/708	R	BMR	9:15	RBNU
HK	7972R/708	R	BMR	9:15	RBNU
HK	7972R/708	R	BMR	9:15	
HK	70821R	R	BRM	11:37	BCCH
HK	70821R	R	BRM	11:37	RBNU

HK	70821R	R			BRM	11:37	MOCH
HK	70821R	R			BRM	11:37	MOCH
HK	70821R	R			BRM	11:37	
HK	70829R	R			BMR	10:33	RBNU
HK	70829R	R			BMR	10:33	RBNU
HK	70829R	R			BMR	10:33	RBNU
HK	70829R	R			BMR	10:33	RBNU
HK	70829R	R			BMR	10:33	RBNU
HK	70829R	R			BMR	10:33	RBNU
HK	70829R	R			BMR	10:33	RBNU
HK	70829R	R			BMR	10:33	RBNU
HK	70829R	R			BMR	10:33	RBNU
HK	70829R	R			BMR	10:33	RBNU
HK	70829R	R			BMR	10:33	RBNU
HK	70829R	R			BMR	10:33	BCCH
HK	70829R	R			BMR	10:33	MOCH
HK	70829R	R			BMR	10:33	RBNU
HK	70829R	R			BMR	10:33	RBNU
HK	70829R	R			BMR	10:33	
HK		R	BCCH	08-ARN-017	BRM	9:34	MOCH
HK		R	BCCH	08-ARN-017	BRM	9:34	MOCH
HK		R	BCCH	08-ARN-017	BRM	9:34	MOCH
HK		R	BCCH	08-ARN-017	BRM	9:34	MOCH
HK		R	BCCH	08-ARN-017	BRM	9:34	MOCH
HK		R	BCCH	08-ARN-017	BRM	9:34	MOCH
HK		R	BCCH	08-ARN-017	BRM	9:34	MOCH
HK		R	BCCH	08-ARN-017	BRM	9:34	MOCH
HK		R	BCCH	08-ARN-017	BRM	9:34	MOCH
HK		R	BCCH	08-ARN-017	BRM	9:34	RBNU
HK		R	BCCH	08-ARN-017	BRM	9:34	MOCH
HK		R	BCCH	08-ARN-017	BRM	9:34	RBNU
HK		R	BCCH	08-ARN-017	BRM	9:34	MOCH
HK		R	BCCH	08-ARN-017	BRM	9:34	MOCH
HK		R	BCCH	08-ARN-017	BRM	9:34	RBNU
HK		R	BCCH	08-ARN-017	BRM	9:34	RBNU
HK		R	BCCH	08-ARN-017	BRM	9:34	MOCH
HK		R	BCCH	08-ARN-017	BRM	9:34	MOCH
HK		R	BCCH	08-ARN-017	BRM	9:34	BCCH
HK		R	BCCH	08-ARN-017	BRM	9:34	RBNU
HK		R	BCCH	08-ARN-017	BRM	9:34	MOCH
HK		R	BCCH	08-ARN-017	BRM	9:34	MOCH
HK		R	BCCH	08-ARN-017	BRM	9:34	
HK	7839R	R			BMR	12:37	RBNU
HK	7839R	R			BMR	12:37	RBNU

HK	7839R	R	BMR	12:37	RBNU
HK	7839R	R	BMR	12:37	RBNU
HK	7839R	R	BMR	12:37	RBNU
HK	7839R	R	BMR	12:37	RBNU
HK	7839R	R	BMR	12:37	RBNU
HK	7839R	R	BMR	12:37	RBNU
HK	7839R	R	BMR	12:37	RBNU
HK	7839R	R	BMR	12:37	BCCH
HK	7839R	R	BMR	12:37	MOCH
HK	7839R	R	BMR	12:37	RBNU
HK	7839R	R	BMR	12:37	RBNU
HK	7839R	R	BMR	12:37	
HK	7063R	R	RBM	8:49	BCCH
HK	7063R	R	RBM	8:49	BCCH
HK	7063R	R	RBM	8:49	MOCH
HK	7063R	R	RBM	8:49	BCCH
HK	7063R	R	RBM	8:49	RBNU
HK	7063R	R	RBM	8:49	RBNU
HK	7063R	R	RBM	8:49	RBNU
HK	7063R	R	RBM	8:49	RBNU
HK	7063R	R	RBM	8:49	RBNU
HK	7063R	R	RBM	8:49	BCCH
HK	7063R	R	RBM	8:49	
HK	7063R	R	RBM	8:49	BCCH
HK	7063R	R	RBM	8:49	RBNU
HK	7063R	R	RBM	8:49	RBNU
HK	7063R	R	RBM	8:49	RBNU
HK	7063R	R	RBM	8:49	BCCH
HK	7063R	R	RBM	8:49	MOCH
HK	7063R	R	RBM	8:49	MOCH
HK	7063R	R	RBM	8:49	
HK	70770R/70	R	MBR	12:11	BCCH
HK	70770R/70	R	MBR	12:11	MOCH
HK	70770R/70	R	MBR	12:11	MOCH
HK	70770R/70	R	MBR	12:11	BCCH
HK	70770R/70	R	MBR	12:11	RBNU
HK	70770R/70	R	MBR	12:11	RBNU
HK	70770R/70	R	MBR	12:11	
HK	70525R	R	BMR	9:02	RBNU
HK	70525R	R	BMR	9:02	MOCH
HK	70525R	R	BMR	9:02	MOCH
HK	70525R	R	BMR	9:02	RBNU
HK	70525R	R	BMR	9:02	RBNU
HK	70525R	R	BMR	9:02	RBNU

HK	70525R	R	BMR	9:02	RBNU
HK	70525R	R	BMR	9:02	BCCH
HK	70525R	R	BMR	9:02	MOCH
HK	70525R	R	BMR	9:02	RBNU
HK	70525R	R	BMR	9:02	BCCH
HK	70525R	R	BMR	9:02	MOCH
HK	70525R	R	BMR	9:02	RBNU
HK	70525R	R	BMR	9:02	RBNU
HK	70525R	R	BMR	9:02	
AE	70717R	R	BMR	10:06	BCCH
AE	70717R	R	BMR	10:06	MOCH
AE	70717R	R	BMR	10:06	MOCH
AE	70717R	R	BMR	10:06	MOCH
AE	70717R	R	BMR	10:06	MOCH
AE	70717R	R	BMR	10:06	MOCH
AE	70717R	R	BMR	10:06	MOCH
AE	70717R	R	BMR	10:06	RBNU
AE	70717R	R	BMR	10:06	MOCH
AE	70717R	R	BMR	10:06	RBNU
AE	70717R	R	BMR	10:06	RBNU
AE	70717R	R	BMR	10:06	RBNU
AE	70717R	R	BMR	10:06	MOCH
AE	70717R	R	BMR	10:06	MOCH
AE	70717R	R	BMR	10:06	BCCH
AE	70717R	R	BMR	10:06	BCCH
AE	70717R	R	BMR	10:06	BCCH
AE	70717R	R	BMR	10:06	MOCH
AE	70717R	R	BMR	10:06	RBNU
AE	70717R	R	BMR	10:06	RBNU
AE	70717R	R	BMR	10:06	
HK	7839R	R	MRB	8:27	MOCH
HK	7839R	R	MRB	8:27	RBNU
HK	7839R	R	MRB	8:27	RBNU
HK	7839R	R	MRB	8:27	RBNU
HK	7839R	R	MRB	8:27	RBNU
HK	7839R	R	MRB	8:27	RBNU
HK	7839R	R	MRB	8:27	RBNU
HK	7839R	R	MRB	8:27	RBNU
HK	7839R	R	MRB	8:27	BCCH
HK	7839R	R	MRB	8:27	MOCH
HK	7839R	R	MRB	8:27	RBNU
HK	7839R	R	MRB	8:27	BCCH
HK	7839R	R	MRB	8:27	BCCH
HK	7839R	R	MRB	8:27	MOCH

HK	7839R	R			MRB	8:27	
HK	7837R/707	R			BRM	9:58	RBNU
HK	7837R/707	R			BRM	9:58	RBNU
HK	7837R/707	R			BRM	9:58	BCCH
HK	7837R/707	R			BRM	9:58	RBNU
HK	7837R/707	R			BRM	9:58	MOCH
HK	7837R/707	R			BRM	9:58	MOCH
HK	7837R/707	R			BRM	9:58	
HK	08-ARN-01	R	BCCH	08-ARN-017	BRM	11:48	BCCH
HK	08-ARN-01	R	BCCH	08-ARN-017	BRM	11:48	BCCH
HK	08-ARN-01	R	BCCH	08-ARN-017	BRM	11:48	BCCH
HK	08-ARN-01	R	BCCH	08-ARN-017	BRM	11:48	BCCH
HK	08-ARN-01	R	BCCH	08-ARN-017	BRM	11:48	BCCH
HK	08-ARN-01	R	BCCH	08-ARN-017	BRM	11:48	BCCH
HK	08-ARN-01	R	BCCH	08-ARN-017	BRM	11:48	BCCH
HK	08-ARN-01	R	BCCH	08-ARN-017	BRM	11:48	BCCH
HK	08-ARN-01	R	BCCH	08-ARN-017	BRM	11:48	BCCH
HK	08-ARN-01	R	BCCH	08-ARN-017	BRM	11:48	BCCH
HK	08-ARN-01	R	BCCH	08-ARN-017	BRM	11:48	BCCH
HK	08-ARN-01	R	BCCH	08-ARN-017	BRM	11:48	BCCH
HK	08-ARN-01	R	BCCH	08-ARN-017	BRM	11:48	BCCH
HK	08-ARN-01	R	BCCH	08-ARN-017	BRM	11:48	BCCH
HK	08-ARN-01	R	BCCH	08-ARN-017	BRM	11:48	BCCH
HK	08-ARN-01	R	BCCH	08-ARN-017	BRM	11:48	BCCH
HK	08-ARN-01	R	BCCH	08-ARN-017	BRM	11:48	BCCH
HK	08-ARN-01	R	BCCH	08-ARN-017	BRM	11:48	BCCH
HK	08-ARN-01	R	BCCH	08-ARN-017	BRM	11:48	BCCH
HK	08-ARN-01	R	BCCH	08-ARN-017	BRM	11:48	RBNU
HK	08-ARN-01	R	BCCH	08-ARN-017	BRM	11:48	BCCH
HK	08-ARN-01	R	BCCH	08-ARN-017	BRM	11:48	RBNU
HK	08-ARN-01	R	BCCH	08-ARN-017	BRM	11:48	RBNU
HK	08-ARN-01	R	BCCH	08-ARN-017	BRM	11:48	MOCH
HK	08-ARN-01	R	BCCH	08-ARN-017	BRM	11:48	RBNU
HK	08-ARN-01	R	BCCH	08-ARN-017	BRM	11:48	RBNU
HK	08-ARN-01	R	BCCH	08-ARN-017	BRM	11:48	BCCH
HK	08-ARN-01	R	BCCH	08-ARN-017	BRM	11:48	BCCH
HK	08-ARN-01	R	BCCH	08-ARN-017	BRM	11:48	RBNU
HK	08-ARN-01	R	BCCH	08-ARN-017	BRM	11:48	MOCH
HK	08-ARN-01	R	BCCH	08-ARN-017	BRM	11:48	MOCH
HK	08-ARN-01	R	BCCH	08-ARN-017	BRM	11:48	BCCH
HK	08-ARN-01	R	BCCH	08-ARN-017	BRM	11:48	
HK	7063R/797	R			RMB	9:09	RBNU
HK	7063R/797	R			RMB	9:09	MOCH
HK	7063R/797	R			RMB	9:09	MOCH

HK	7063R/797	R	RMB	9:09	MOCH
HK	7063R/797	R	RMB	9:09	RBNU
HK	7063R/797	R	RMB	9:09	RBNU
HK	7063R/797	R	RMB	9:09	MOCH
HK	7063R/797	R	RMB	9:09	BCCH
HK	7063R/797	R	RMB	9:09	BCCH
HK	7063R/797	R	RMB	9:09	
HK	70102R	R	BMR	9:10	MOCH
HK	70102R	R	BMR	9:10	MOCH
HK	70102R	R	BMR	9:10	RBNU
HK	70102R	R	BMR	9:10	RBNU
HK	70102R	R	BMR	9:10	RBNU
HK	70102R	R	BMR	9:10	RBNU
HK	70102R	R	BMR	9:10	RBNU
HK	70102R	R	BMR	9:10	RBNU
HK	70102R	R	BMR	9:10	RBNU
HK	70102R	R	BMR	9:10	BCCH
HK	70102R	R	BMR	9:10	BCCH
HK	70102R	R	BMR	9:10	BCCH
HK	70102R	R	BMR	9:10	MOCH
HK	70102R	R	BMR	9:10	RBNU
HK	70102R	R	BMR	9:10	RBNU
HK	70102R	R	BMR	9:10	
HK	70733R	R	BRM	10:17	
HK	70733R	R	BRM	10:17	RBNU
HK	70733R	R	BRM	10:17	RBNU
HK	70733R	R	BRM	10:17	RBNU
HK	70733R	R	BRM	10:17	BCCH
HK	70733R	R	BRM	10:17	RBNU
HK	70733R	R	BRM	10:17	MOCH
HK	70733R	R	BRM	10:17	MOCH
HK	70733R	R	BRM	10:17	
HK	70153R	R	RMB	10:55	RBNU
HK	70153R	R	RMB	10:55	MOCH
HK	70153R	R	RMB	10:55	BCCH
HK	70153R	R	RMB	10:55	BCCH
HK	70153R	R	RMB	10:55	RBNU
HK	70153R	R	RMB	10:55	MOCH
HK	70153R	R	RMB	10:55	BCCH
HK	70153R	R	RMB	10:55	BCCH
HK	70153R	R	RMB	10:55	
HK	70823R	R	MRB	8:29	MOCH
HK	70823R	R	MRB	8:29	RBNU
HK	70823R	R	MRB	8:29	RBNU

HK	70823R	R			MRB	8:29	BCCH
HK	70823R	R			MRB	8:29	MOCH
HK	70823R	R			MRB	8:29	RBNU
HK	70823R	R			MRB	8:29	BCCH
HK	70823R	R			MRB	8:29	BCCH
HK	70823R	R			MRB	8:29	
HK	70525R/08	R	RBNU	08-ARN-039	BRM	9:44	BCCH
HK	70525R/08	R	RBNU	08-ARN-039	BRM	9:44	BCCH
HK	70525R/08	R	RBNU	08-ARN-039	BRM	9:44	BCCH
HK	70525R/08	R	RBNU	08-ARN-039	BRM	9:44	BCCH
HK	70525R/08	R	RBNU	08-ARN-039	BRM	9:44	BCCH
HK	70525R/08	R	RBNU	08-ARN-039	BRM	9:44	BCCH
HK	70525R/08	R	RBNU	08-ARN-039	BRM	9:44	BCCH
HK	70525R/08	R	RBNU	08-ARN-039	BRM	9:44	BCCH
HK	70525R/08	R	RBNU	08-ARN-039	BRM	9:44	BCCH
HK	70525R/08	R	RBNU	08-ARN-039	BRM	9:44	BCCH
HK	70525R/08	R	RBNU	08-ARN-039	BRM	9:44	BCCH
HK	70525R/08	R	RBNU	08-ARN-039	BRM	9:44	BCCH
HK	70525R/08	R	RBNU	08-ARN-039	BRM	9:44	BCCH
HK	70525R/08	R	RBNU	08-ARN-039	BRM	9:44	BCCH
HK	70525R/08	R	RBNU	08-ARN-039	BRM	9:44	RBNU
HK	70525R/08	R	RBNU	08-ARN-039	BRM	9:44	RBNU
HK	70525R/08	R	RBNU	08-ARN-039	BRM	9:44	MOCH
HK	70525R/08	R	RBNU	08-ARN-039	BRM	9:44	RBNU
HK	70525R/08	R	RBNU	08-ARN-039	BRM	9:44	RBNU
HK	70525R/08	R	RBNU	08-ARN-039	BRM	9:44	BCCH
HK	70525R/08	R	RBNU	08-ARN-039	BRM	9:44	RBNU
HK	70525R/08	R	RBNU	08-ARN-039	BRM	9:44	MOCH
HK	70525R/08	R	RBNU	08-ARN-039	BRM	9:44	MOCH
HK	70525R/08	R	RBNU	08-ARN-039	BRM	9:44	
HK	70717R	R			RMB	11:50	BCCH
HK	70717R	R			RMB	11:50	RBNU
HK	70717R	R			RMB	11:50	RBNU
HK	70717R	R			RMB	11:50	RBNU
HK	70717R	R			RMB	11:50	MOCH
HK	70717R	R			RMB	11:50	BCCH
HK	70717R	R			RMB	11:50	BCCH
HK	70717R	R			RMB	11:50	
HK	70832R	R			MRB	8:23	BCCH
HK	70832R	R			MRB	8:23	MOCH
HK	70832R	R			MRB	8:23	BCCH
HK	70832R	R			MRB	8:23	RBNU
HK	70832R	R			MRB	8:23	MOCH
HK	70832R	R			MRB	8:23	RBNU

HK	70832R	R			MRB	8:23	BCCH
HK	70832R	R			MRB	8:23	BCCH
HK	70832R	R			MRB	8:23	
IB		R			BMR	9:45	BCCH
IB		R			BMR	9:45	BCCH
IB		R			BMR	9:45	BCCH
IB		R			BMR	9:45	MOCH
IB		R			BMR	9:45	MOCH
IB		R			BMR	9:45	MOCH
IB		R			BMR	9:45	MOCH
IB		R			BMR	9:45	MOCH
IB		R			BMR	9:45	MOCH
IB		R			BMR	9:45	MOCH
IB		R			BMR	9:45	MOCH
IB		R			BMR	9:45	MOCH
IB		R			BMR	9:45	MOCH
IB		R			BMR	9:45	RBNU
IB		R			BMR	9:45	RBNU
IB		R			BMR	9:45	RBNU
IB		R			BMR	9:45	RBNU
IB		R			BMR	9:45	BCCH
IB		R			BMR	9:45	BCCH
IB		R			BMR	9:45	BCCH
IB		R			BMR	9:45	BCCH
IB		R			BMR	9:45	BCCH
IB		R			BMR	9:45	MOCH
IB		R			BMR	9:45	RBNU
IB		R			BMR	9:45	RBNU
IB		R			BMR	9:45	MOCH
IB		R			BMR	9:45	RBNU
IB		R			BMR	9:45	RBNU
IB		R			BMR	9:45	BCCH
IB		R			BMR	9:45	MOCH
IB		R			BMR	9:45	RBNU
IB		R			BMR	9:45	RBNU
IB		R			BMR	9:45	MOCH
IB	?????	R			BMR	9:45	
IB		R			RBM	8:58 AM	BCCH
IB		R			RBM	8:58 AM	BCCH
IB		R			RBM	8:58 AM	BCCH
IB		R			RBM	8:58 AM	BCCH
IB		R			RBM	8:58 AM	MOCH
IB		R			RBM	8:58 AM	RBNU
	70730	R - TEST	BCCH	2004	RBM	8:44	none

70730	R - TEST			BCCH	2004	RBM	8:44	none
70730	R - TEST			BCCH	2004	RBM	8:44	none
7280	TEST	MOCH	05-MN-005	RBNU	2003	RMB	8:50	MOCH
7280	TEST	MOCH	05-MN-005	RBNU	2003	RMB	8:50	BCCH
7280	TEST	MOCH	05-MN-005	RBNU	2003	RMB	8:50	BCCH
7280	TEST	MOCH	05-MN-005	RBNU	2003	RMB	8:50	BCCH
7280	TEST	MOCH	05-MN-005	RBNU	2003	RMB	8:50	RBNU
7280	TEST	MOCH	05-MN-005	RBNU	2003	RMB	8:50	RBNU
7280	TEST	MOCH	05-MN-005	RBNU	2003	RMB	8:50	MOCH
7280	TEST	MOCH	05-MN-005	RBNU	2003	RMB	8:50	MOCH
7280	TEST	MOCH	05-MN-005	RBNU	2003	RMB	8:50	MOCH
7280	TEST	MOCH	05-MN-005	RBNU	2003	RMB	8:50	MOCH
7280	TEST	MOCH	05-MN-005	RBNU	2003	RMB	8:50	MOCH
7280	TEST	MOCH	05-MN-005	RBNU	2003	RMB	8:50	BCCH
7280	TEST	MOCH	05-MN-005	RBNU	2003	RMB	8:50	RBNU
7545	TEST			RBNU	2003	RBM	8:02	none
70721	TEST			MOBL	2004	RBM	8:37	MOCH
70721	TEST			MOBL	2004	RBM	8:37	MOCH
70721	TEST			MOBL	2004	RBM	8:37	MOCH
70721	TEST			MOBL	2004	RBM	8:37	BCCH

Track	Time	Stopwatch	Stopwatch	Stopwatch	Species	Sex	Band	Age	No.ads.pre
	7:35:10			5:10	BCCH	U			1
	7:35:40			5:40	BCCH	M			1
	7:36:20			6:20	BCCH	M			1
	7:39:20			9:20	BCCH	M			1
	7:45:10			15:10	BCCH	F			2
	7:48:00			18:00	BCCH	M			2
	7:53:10			23:10	BCCH	M			2
	7:57:50			27:50:00	BCCH	M			2
	7:57:51			27:51:00	BCCH	F			2
	7:55:30			25:30:00	BCCH	M			2
	7:45:10			15:10	BCCH	M			2
	7:42:00			12:00	BCCH	M			2
	7:42:00			12:00	BCCH	F			2
	7:46:30			16:30	BCCH	M			2
	7:49:10			19:10	BCCH	M			2
	7:56:30			26:30:00	BCCH	M			2
	7:52:00			22:00	BCCH	M			2
	7:30:00			0:00	NOFL	U			1
	7:46:30			16:30	RBNU	U			1
	7:45:50			15:50	RBNU	U			1
				0:13:00	BCCH	M			2
				0:02:02	BCCH	M,F			2
				0:07:28	BCCH	M			1
				0:02:24	BCCH				
				0:02:30	BCCH				
				0:03:10	BCCH				
				0:08:00	BCCH	M			1
				0:09:10	BCCH	M			1
				0:11:20	BCCH	M			2
				0:14:40	BCCH	M			2
				0:16:00	BCCH	M			2
				0:20:45	BCCH	M			2
				0:20:45	BCCH	F			2
				0:22:20	BCCH	M			2
				0:24:00	BCCH	F			2
				0:01:15	BCCH	F			2
				0:01:15	BCCH	M			2
				0:01:50	BCCH	M			2
				0:03:00	BCCH	F			2
				0:02:02	BCCH				
				0:08:20	BCCH	M			1
				0:09:20	BCCH	F			2
				0:23:00	BCCH	M			2

				0:00:00	BCCH	M	2
				0:23:20	BCCH	M	2
				0:03:00	BCCH	M	2
				0:00:10	BCCH		1
				0:24:19	BCCH	M	2
				0:07:00	BCCH	M	1
				0:24:20	BCCH	M	2
				0:24:20	BCCH	F	2
				0:24:30	BCCH	M	2
				0:20:00	START		
				0:01:00	START		2
	9:50	0	0	0:00:00			
5	9:19	15	41	0:15:41	BCCH	M	1
5	9:19	15	15	0:15:15	BCCH	M	1
5	9:20	16	40	0:16:40	BCCH	M	1
3	9:09	5	39	0:05:39	BCCH	M	1
3	9:11	7	11	0:07:11	BCCH	M	1
3	9:09	5	7	0:05:07	BCCH	M	1
3	9:10	6	21	0:06:21	BCCH	M	1
5	9:22	18	35	0:18:35	BCCH	M	1
3	9:08	4	33	0:04:33	BCCH	M	1
3	9:12	8	2	0:08:02	MOCH	M	1
11	9:13	9	25	0:09:25	MOCH	M	1
3	9:09	5	20	0:05:20	RBNU		1
3	9:06	2	49	0:02:49	start		
11	9:13	9	8	0:09:08	start		
5	9:18	14	0	0:14:00	start		
5	9:23	19	8	0:19:08	stop		
	9:04	0	0	0:00:00			
11	11:35	13	50	0:13:50	BCCH		1
11	11:36	14	23	0:14:23	BCCH	M	1
5	11:25	3	55	0:03:55	BCCH		1
5	11:28	6	26	0:06:26	BCCH		1
11	11:36	14	33	0:14:33	BCCH	F	2
11	11:36	14	40	0:14:40	BCCH	F	2
11	11:36	14	52	0:14:52	BCCH	M	1
11	11:37	15	5	0:15:05	BCCH	M	1
11	11:36	14	52	0:14:52	RBNU	M	1
11	11:37	15	5	0:15:05	RBNU	M	1
11	11:37	15	21	0:15:21	RBNU	M	1
11	11:35	13	59	0:13:59	RBNU		1
11	11:37	15	21	0:15:21	RBNU		2
5	11:22	0	52	0:00:52	start		
3	11:29	7	35	0:07:35	start		

11	11:34	12	50	0:12:50	start		
11	11:39	17	57	0:17:57	stop		
	11:22	0	0	0:00:00			
5	11:50	0	10	0:00:10	BOCH	U	1
5	11:50	0	22	0:00:22	BOCH	U	1
5	11:50	0	50	0:00:50	BOCH	U	1
5	11:52	2	23	0:02:23	BOCH	U	1
5	11:54	4	6	0:04:06	BOCH	U	1
5	11:54	4	40	0:04:40	BOCH	U	1
5	11:55	5	2	0:05:02	BOCH	U	1
5	11:55	5	20	0:05:20	BOCH	U	1
5	11:50	0	0	0:00:00	START		
1	11:56	6	0	0:06:00	START		
13	12:00	10	10	0:10:10	START		
	8:30:50			15:50	BCCH	M	1
	8:32:20			17:20	BCCH	M	1
	8:25:00			10:00	BCCH	M	1
	8:21:00			6:00	BCCH	M	1
	8:19:20			4:20	MOCH	U	1
	8:20:20			5:20	MOCH	U	1
	8:22:20			7:20	MOCH	U	1
	8:23:35			8:35	MOCH	U	1
	8:27:10			12:10	MOCH	U	1
	8:28:40			13:40	MOCH	U	1
	8:29:10			14:10	MOCH	U	1
	8:30:20			15:20	MOCH	U	1
	8:31:40			16:40	MOCH	U	1
	8:32:50			17:50	MOCH	U	1
	8:36:20			21:20	MOCH	U	1
	8:40:10			25:10:00	MOCH	U	1
	8:44:10			29:10:00	MOCH	U	1
	8:18:00			3:00	RBNU	U	1
	8:59:40			16:40	MOCH	M	2
	9:03:40			20:40	MOCH	M	2
	8:50:00			7:00	MOCH	M	2
	8:56:52			13:52	MOCH	M	2
	8:43:00			0:00	MOCH	F	2
	8:55:00			12:00	MOCH	F	2
	8:49:30			6:30	MOCH	M	2
	8:58:00			15:00	MOCH	U	3
	8:50:20			7:20	MOCH	F	2
	9:03:40			20:40	MOCH	F	2
	8:43:00			0:00	MOCH	M	2
	9:06:00			23:00	MOCH	B	2

8:43:04	0:04	MOCH	F	2
8:52:30	9:30	MOCH	U	2
8:58:30	15:30	MOCH	M	3
8:49:28	6:28	RBNU	U	2
8:49:00	6:00	RBNU	U	2
8:50:00	7:00	RBNU	U	2
8:52:50	9:50	RBNU	U	2
8:56:20	13:20	RBNU	U	2
9:07:00	24:00:00	RBNU	U	2
10:55:00	15:00	MOCH	M	2
10:52:40	12:40	MOCH	M	2
10:54:00	14:00	MOCH	M	2
10:54:00	14:00	MOCH	F	2
10:56:20	16:20	MOCH	M	2
10:48:20	8:20	MOCH	M	2
10:52:45	12:45	MOCH	M	2
10:48:00	8:00	MOCH	M	2
11:00:45	20:45	MOCH	M	2
10:48:10	8:10	RBNU	M	1
10:50:30	10:30	RBNU	M	1
10:57:00	17:00	RBNU	M	1
11:03:10	23:10	RBNU	M	1
10:41:00	1:00	RBNU	M	1
14:12:30	9:30	BCCH	M	1
14:12:00	9:00	BCCH	M	1
14:19:40	16:40	BCCH	M	1
14:11:30	8:30	BCCH	M	1
14:23:10	20:10	BCCH	M	1
14:20:20	17:20	BCCH	M	1
14:10:50	7:50	BCCH	M	1
14:45:00	42:00:00	MOCH	F	2
14:03:53	0:53	MOCH	U	2
14:04:20	1:20	MOCH	F	2
14:08:45	5:45	MOCH	F	2
14:15:10	12:10	MOCH	U	1
14:15:30	12:30	MOCH	F	1
14:32:45	29:45:00	MOCH	F	2
14:31:10	28:10:00	MOCH	M	2
14:03:53	0:53	MOCH	U	2
14:32:00	29:00:00	MOCH	M	2
14:03:00	0:00	MOCH	M	2
14:04:00	1:00	RBNU	U	1
14:52:40	47:40:00	MOCH	M	2
15:08:50	3:50	MOCH	M	2

15:08:50	3:50	MOCH	M	2
14:52:20	47:20:00	MOCH	M	2
14:50:49	45:49:00	MOCH	M	2
14:54:50	49:50:00	MOCH	M	2
15:08:00	3:00	MOCH	M	2
15:10:36	5:36	MOCH	M	2
15:21:00	16:00	MOCH	M	2
14:55:30	50:30:00	MOCH	M	2
14:55:50	50:50:00	MOCH	M	2
15:08:25	3:25	MOCH	M	2
15:10:50	5:50	MOCH	M	2
15:16:00	11:00	RBNU	M	1
15:17:38	12:38	RBNU	M	1
13:26:00	14:00	MOCH	U	1
13:26:15	14:15	MOCH	U	1
13:35:00	23:00	MOCH	U	1
13:35:15	23:15	MOCH	U	1
13:23:40	11:40	MOCH	U	1
13:35:30	23:30	MOCH	U	1
13:34:00	22:00	MOCH	U	1
13:19:40	7:40	MOCH	U	1
13:17:00	5:00	RBNU	M	1
13:33:50	21:50	RBNU	U	1
13:25:00	13:00	RBNU	U	1
13:16:10	4:10	RBNU	M	1
13:20:30	8:30	RBNU	U	1
13:55:01	20:01	MOCH	M	2
13:54:20	19:20	MOCH	M	2
13:36:43	1:43	MOCH	U	2
13:40:00	5:00	MOCH	U	2
13:40:41	5:41	MOCH	U	2
13:53:10	18:10	MOCH	M	2
13:54:20	19:20	MOCH	F	2
13:55:00	20:00	MOCH	M	2
13:57:00	22:00	MOCH	M	2
13:52:52	17:52	MOCH	M	2
14:30:38	25:38:00	BCCH	M	1
14:27:48	22:48	BCCH	M	1
14:29:16	24:16:00	BCCH	M	1
14:28:50	23:50	BCCH	M	1
14:31:50	26:50:00	BCCH	M	1
14:22:49	17:49	MOCH	U	2
14:31:26	26:26:00	MOCH	U	2
14:10:30	5:30	MOCH	U	2

	14:27:20			22:20	MOCH	U	2
	14:27:55			22:55	MOCH	U	2
	14:11:29			6:29	MOCH	U	2
	14:26:46			21:46	MOCH	U	2
	14:26:25			21:25	MOCH	U	2
	14:10:30			5:30	MOCH	U	2
	14:13:11			8:11	MOCH	U	2
	14:23:40			18:40	MOCH	U	2
	14:25:10			20:10	MOCH	U	2
	14:19:55			14:55	MOCH	B	2
	14:20:59			15:59	MOCH	U	2
	14:05:04			0:04	MOCH	M	2
	14:05:36			0:36	MOCH	F	2
	14:09:15			4:15	RBNU	U	1
11		17	0	0:17:00	END		
1		9	0	0:09:00	MOCH	F	1
5		3	22	0:03:22	MOCH		1
1		7	20	0:07:20	START		
5		3	20	0:03:20	START		
11		12	20	0:12:20	START		
	8:15	0	0	0:00:00	START		
1	9:36	19	4	0:19:04	BCCH	M	2
1	9:36	19	27	0:19:27	BCCH	M	2
5	9:25	9	35	0:09:35	BCCH	M	1
1	9:32	16	25	0:16:25	BCCH	M	1
1	9:33	17	11	0:17:11	BCCH	M	1
13	9:21	5	35	0:05:35	BCCH	M	1
5	9:24	8	4	0:08:04	BCCH	M	1
5	9:27	11	18	0:11:18	BCCH	M	1
5	9:29	13	0	0:13:00	BCCH	M	1
1	9:31	15	17	0:15:17	BCCH	M	1
1	9:35	18	0	0:18:00	BCCH	M	2
1	9:34	17	28	0:17:28	MOCH	U	1
13	9:16	0	0	0:00:00	START		
5	9:23	7	30	0:07:30	START		
1	9:31	15	0	0:15:00	START		
13	8:35	15	10	0:15:10	MOCH		1
5	8:43	23	44	0:23:44	MOCH	M	1
5	8:45	25	7	0:25:07	MOCH	M	1
13	8:28	8	11	0:08:11	RBNU	M	1
13	8:27	7	13	0:07:13	RBNU	M	1
13	8:26	6	51	0:06:51	RBNU	M	1
13	8:27	7	34	0:07:34	RBNU	M	1
13	8:27	7	59	0:07:59	RBNU	M	1

13	8:26	6	36	0:06:36	RBNU	M	1
13	8:28	8	42	0:08:42	RBNU	M	1
13	8:26	6	22	0:06:22	RBNU	M	1
13	8:29	9	46	0:09:46	RBNU	M	1
5	8:49	29	0	0:29:00	RBNU	M	1
13	8:30	10	10	0:10:10	RBNU	M	1
5	8:45	25	7	0:25:07	RBNU	M	1
13	8:32	12	37	0:12:37	RBNU	M	1
13	8:34	14	23	0:14:23	RBNU	M	1
13	8:35	15	0	0:15:00	RBNU	M	1
5	8:40	20	11	0:20:11	RBNU	M	1
5	8:40	20	47	0:20:47	RBNU	M	1
5	8:41	21	58	0:21:58	RBNU	M	1
5	8:42	22	44	0:22:44	RBNU	M	1
5	8:43	23	6	0:23:06	RBNU	M	1
1	8:21	1	40	0:01:40	RBNU	M	1
1	8:22	2	56	0:02:56	RBNU	M	1
1	8:24	4	9	0:04:08	RBNU	M	1
13	8:26	6	11	0:06:11	RBNU	M	1
1	8:20	0	39	0:00:39	RBNU	M	1
1	8:20	0	0	0:00:00	START		
13	8:26	6	0	0:06:00	START		
5	8:40	20	0	0:20:00	START		
13	10:25	18	0	0:18:00	END		
1	10:13	6	49	0:06:49	MOCH	F	1
5	10:07	0	39	0:00:39	MOCH	U	1
5	10:08	1	54	0:01:54	MOCH	U	1
5	10:11	4	10	0:04:10	MOCH	U	1
5	10:11	4	47	0:04:47	MOCH	U	1
13	10:19	12	59	0:12:59	MOCH	F	1
13	10:20	13	20	0:13:20	MOCH	F	1
5	10:10	3	28	0:03:28	MOCH	U	1
13	10:20	13	40	0:13:40	MOCH	F	1
1	10:13	6	48	0:06:48	START		
5	10:07	0	35	0:00:35	START		
13	10:19	12	53	0:12:53	START		
	10:07	0	0	0:00:00	START		
1	9:21	1	22	0:01:22	MOCH	U	1
5	9:27	7	13	0:07:13	MOCH	M	2
5	9:27	7	28	0:07:28	MOCH	F	2
1	9:22	2	37	0:02:37	MOCH	M	1
5	9:28	8	12	0:08:12	MOCH	M	1
1	9:25	5	0	0:05:00	MOCH	M	1
5	9:28	8	6	0:08:06	MOCH	M	1

5	9:31	11	24	0:11:24	MOCH	M	1
5	9:27	7	35	0:07:35	MOCH	M	2
5	9:27	7	35	0:07:35	MOCH	F	2
1	9:21	1	26	0:01:26	RBNU	M	1
1	9:20	0	0	0:00:00	START		
5	9:26	6	30	0:06:30	START		
13	9:32	12	4	0:12:04	START		
3	10:04	12	5	0:12:05	BCCH	M	1
3	10:05	13	12	0:13:12	BCCH	M	1
3	10:06	14	51	0:14:51	BCCH	M	1
3	10:08	16	25	0:16:25	BCCH	M	1
11	9:58	6	56	0:06:56	CHMK		1
5	10:14	22	48	0:22:48	HAWO		1
	9:53	1	15	0:01:15	MOCH		2
11	9:55	3	30	0:03:30	MOCH		1
11	9:55	3	50	0:03:50	MOCH		1
11	9:56	4	7	0:04:07	MOCH		2
11	9:56	4	50	0:04:50	MOCH		2
11	9:57	5	30	0:05:30	MOCH		1
11	9:59	7	13	0:07:13	MOCH		1
5	10:09	17	15	0:17:15	MOCH		1
11	9:55	3	26	0:03:26	MOCH		1
11	9:57	5	13	0:05:13	MOCH		1
11	9:56	4	36	0:04:36	MOCH		2
11	9:59	7	14	0:07:14	MOCH		1
5	10:10	18	54	0:18:54	MOCH	M	1
11	9:55	3	2	0:03:02	RBNU		1
11	9:54	2	46	0:02:46	RBNU		1
11	9:54	2	30	0:02:30	RBNU		1
11	9:55	3	14	0:03:14	RBNU		1
11	9:55	3	50	0:03:50	RBNU		1
11	9:56	4	21	0:04:21	RBNU		1
11	9:57	5	13	0:05:13	RBNU		1
11	9:56	4	58	0:04:58	RBNU		1
11	9:57	5	58	0:05:58	RBNU		1
11	10:00	8	36	0:08:36	RBNU		1
3	10:03	11	42	0:11:42	RBNU		1
11	9:54	2	10	0:02:10	start		
3	10:01	9	32	0:09:32	start		
5	10:09	17	3	0:17:03	start		
5	10:16	24	39	0:24:39	stop		
5	10:09	17	40	0:17:40	TRES		3
	9:52	0	0	0:00:00			
3	9:54	17	30	0:17:30	BCCH	M	1

3	9:53	16	40	0:16:40	MOCH	F		1
3	9:53	16	45	0:16:45	MOCH	F		1
11	9:42	5	0	0:05:00	MOCH			1
5	9:49	12	40	0:12:40	MOCH	M		1
5	9:48	11	30	0:11:30	MOCH	M		1
5	9:46	9	40	0:09:40	MOCH	M		1
5	9:50	13	50	0:13:50	MOCH	M		1
5	9:51	14	30	0:14:30	MOCH	M		2
11	9:39	2	30	0:02:30	MOCH			1
11	9:41	4	0	0:04:00	RBNU	M		1
11	9:43	6	10	0:06:10	RBNU	M		1
5	9:50	13	30	0:13:30	RBNU			1
5	9:47	10	30	0:10:30	RBNU			1
5	9:49	12	50	0:12:50	RBNU			1
11	9:39	2	40	0:02:40	RBNU			1
3	9:55	18	45	0:18:45	RBNU			1
3	9:57	20	45	0:20:45	RBNU			1
11	9:38	1	55	0:01:55	start			
5	9:46	9	8	0:09:08	start			
3	9:53	16	30	0:16:30	start			
3	9:58	21	30	0:21:30	stop			
	9:37	0	0	0:00:00				
11	9:05	10	7	0:10:07	MOCH			2
11	9:05	10	41	0:10:41	MOCH			2
11	9:05	10	41	0:10:41	MOCH			2
11	9:06	11	5	0:11:05	MOCH			2
5	9:10	15	59	0:15:59	MOCH	M	AK/GO	2
5	9:11	16	10	0:16:10	MOCH	M	AK/GO	2
5	9:12	17	39	0:17:39	MOCH	M	AK/GO	2
5	9:12	17	45	0:17:45	MOCH	M	AK/GO	2
5	9:12	17	50	0:17:50	MOCH	M	AK/GO	2
5	9:13	18	1	0:18:01	MOCH	M	AK/GO	2
5	9:13	18	10	0:18:10	MOCH	M	AK/GO	2
3	8:59	4	21	0:04:21	RBNU			1
3	9:00	5	3	0:05:03	RBNU			1
11	9:04	9	10	0:09:10	RBNU			1
3	8:56	1	37	0:01:37	RBNU			1
3	8:55	0	29	0:00:29	start			
11	9:02	7	57	0:07:57	start			
5	9:10	15	0	0:15:00	start			
5	9:16	21	47	0:21:47	stop			
	8:55	0	0	0:00:00				
11	9:42	15	5	0:15:05	MOCH			1
5	9:44	17	52	0:17:52	MOCH			2

5	9:45	18	25	0:18:25	MOCH			2
3	9:30	3	3	0:03:03	MOCH			2
3	9:30	3	10	0:03:10	MOCH			2
3	9:30	3	15	0:03:15	MOCH			2
3	9:32	5	13	0:05:13	MOCH			1
3	9:32	5	15	0:05:15	MOCH			1
3	9:33	6	58	0:06:58	MOCH			1
11	9:38	11	19	0:11:19	MOCH			2
11	9:38	11	21	0:11:21	MOCH			2
11	9:38	11	23	0:11:23	MOCH			2
11	9:38	11	31	0:11:31	MOCH			1
11	9:39	12	32	0:12:32	MOCH			1
11	9:39	12	48	0:12:48	MOCH			1
11	9:42	15	10	0:15:10	MOCH			1
11	9:42	15	26	0:15:26	MOCH			1
5	9:45	18	0	0:18:00	MOCH			2
5	9:45	18	5	0:18:05	MOCH			2
5	9:45	18	25	0:18:25	MOCH			2
5	9:47	20	26	0:20:26	MOCH			1
5	9:47	20	40	0:20:40	MOCH			1
5	9:47	20	41	0:20:41	MOCH			2
5	9:47	20	58	0:20:58	MOCH			2
5	9:48	21	49	0:21:49	MOCH			2
5	9:49	22	7	0:22:07	MOCH			2
3	9:29	2	57	0:02:57	MOCH			1
11	9:39	12	5	0:12:05	RBNU			1
11	9:37	10	22	0:10:22	RBNU			1
3	9:31	4	56	0:04:56	RBNU			1
3	9:27	0	57	0:00:57	start			
11	9:35	8	42	0:08:42	start			
5	9:42	15	37	0:15:37	start			
5	9:49	22	36	0:22:36	stop			
	9:27	0	0	0:00:00				
	11:23	0	0	0:00:00	MOCH	F	AY/OW	1
5	11:27	4	16	0:04:16	RBNU			1
5	11:26	3	34	0:03:34	start			
3	11:31	8	4	0:08:04	start			
11	11:35	12	40	0:12:40	start			
11	11:39	16	36	0:16:36	stop			
	11:23	0	0	0:00:00				
	11:03:40				BCCH	F		2
	11:03:40				BCCH	M		2
	11:02:40				BCCH	M		1
	11:01:30				BCCH	U		1

11:11:50	BCCH	M	2
11:11:50	BCCH	F	2
11:01:11	BCCH	M	1
10:55:30	RBNU	M	1
10:59:30	RBNU	U	1
11:03:07	RBNU	U	1
11:06:30	RBNU	M	2
11:06:40	RBNU	F	2
11:08:30	RBNU	F	2
11:08:30	RBNU	M	2
11:11:00	RBNU	F	1
11:13:24	RBNU	M	1
11:05:20	RBNU	M	1
8:52:26	MOCH	U	1
8:30:31	MOCH	U	1
8:47:20	MOCH	U	1
8:44:20	RBNU	U	3
8:44:20	RBNU	M	3
8:46:19	RBNU	U	3
8:45:21	RBNU	F	3
8:55:50	RBNU	U	3
8:54:40	RBNU	U	3
8:43:18	RBNU	U	3
8:45:50	RBNU	U	3
8:48:30	RBNU	M	3
8:57:20	RBNU	U	3
8:36:20	TAHU	U	1
7:44:00	BCCH	U	1
7:36:34	BCCH	U	2
7:45:20	BCCH	M	2
7:45:20	BCCH	F	2
7:41:30	BCCH	M	1
7:35:25	BCCH	U	1
7:38:39	BCCH	U	1
10:27:30	BCCH	M	2
10:25:20	BCCH	U	2
10:25:20	BCCH	M	2
10:17:30	BCCH	U	1
10:15:20	BCCH	U	1
10:23:30	RBNU	M	1
10:28:50	RBNU	M	1
10:30:40	RBNU	M	1
10:32:30	RBNU	M	1
10:34:20	RBNU	M	1

10:28:20		RBNU	M	1
10:27:20		RBNU	M	1
10:18:28		RBNU	U	1
10:20:45		RBNU	U	1
11:14:30	14:30	MOCH	U	
11:15:20	15:20	MOCH	U	
11:16:30	16:30	MOCH	M	
11:18:00	18:00	MOCH	M	
11:21:40	21:40	MOCH	M	
11:11:58	11:58	MOCH	M	
11:13:20	13:20	MOCH	M	
11:14:45	14:45	RBNU	F	
11:23:15	23:15	RBNU	F	
11:00:00	0:00	RBNU	F	
11:18:30	18:30	RBNU	F	
11:20:00	20:00	RBNU	F	
11:22:10	22:10	RBNU	F	
11:22:10	22:10	RBNU	F	
11:23:40	23:40	RBNU	F	
11:15:20	15:20	RBNU	F	
11:18:00	18:00	RBNU	F	
11:04:41	4:41	RBNU	M	
11:23:15	23:15	RBNU	F	
11:16:40	16:40	RBNU	F	
11:14:30	14:30	RBNU	F	
11:15:30	15:30	RBNU	F	
8:59:00	13:00	MOCH	U	2
8:59:20	13:20	MOCH	M	2
9:00:00	14:00	MOCH	F	2
8:48:00	2:00	MOCH	M	
9:01:50	15:50	RBNU	U	2
9:08:45	22:45	RBNU	U	2
9:06:50	20:50	RBNU	U	2
8:51:17	5:17	RBNU	U	2
8:51:20	5:20	RBNU	U	2
8:51:30	5:30	RBNU	U	2
8:56:40	10:40	RBNU	U	2
8:58:40	12:40	RBNU	U	2
9:06:00	20:00	RBNU	U	2
9:09:40	23:40	RBNU	U	2
8:50:40	4:40	RBNU	U	
8:47:00	1:00	RBNU	U	
8:51:30	5:30	RBNU	U	2
8:59:00	13:00	RBNU	U	2

9:01:40	15:40	RBNU	U	2
7:38:05	26:05:00	RBNU	U	2
7:37:30	25:30:00	RBNU	U	2
7:34:16	22:16	RBNU	U	2
7:28:13	16:13	RBNU	U	2
7:26:32	14:32	RBNU	U	2
7:27:00	15:00	RBNU	U	2
7:25:25	13:25	RBNU	U	2
7:28:13	16:13	RBNU	U	2
7:22:45	10:45	RBNU	U	2
7:34:30	22:30	RBNU	U	2
7:38:05	26:05:00	RBNU	U	2
7:18:20	6:20	RBNU	U	2
8:49:40	13:40	MOCH	M	2
8:51:50	15:50	MOCH	M	2
8:42:25	6:25	MOCH	M	1
8:37:00	1:00	MOCH	M	1
8:45:45	9:45	MOCH	M	1
8:50:10	14:10	RBNU	M	2
8:49:00	13:00	RBNU	M	2
8:54:30	18:30	RBNU	M	2
8:41:47	5:47	RBNU	M	2
8:59:18	23:18	RBNU	M	2
8:55:30	19:30	RBNU	M	2
8:58:30	22:30	RBNU	M	2
8:41:00	5:00	RBNU	M	2
8:47:35	11:35	RBNU	M	2
8:58:00	22:00	RBNU	M	2
8:36:00	0:00	RBNU	M	2
9:21:45	12:45	MOCH	U	2
9:14:55	5:55	MOCH	U	2
9:11:25	2:25	MOCH	M	2
9:22:12	13:12	RBNU	M	2
9:25:25	16:25	RBNU	M	2
9:29:40	20:40	RBNU	M	2
9:28:42	19:42	RBNU	M	2
10:27:40	18:40	RBNU	M	2
10:27:10	18:10	RBNU	M	2
10:26:40	17:40	RBNU	M	2
10:26:21	17:21	RBNU	M	2
10:21:20	12:20	RBNU	M	2
10:34:30	25:30:00	RBNU	M	2
10:13:00	4:00	RBNU	M	2
10:23:00	14:00	RBNU	M	2

	10:12:20	3:20	RBNU	F	2
	10:12:20	3:20	RBNU	M	2
	10:15:20	6:20	RBNU	F	2
	10:22:18	13:18	RBNU	M	2
	10:23:45	14:45	RBNU	M	2
	10:16:20	7:20	RBNU	M	2
	10:17:50	8:50	RBNU	M	2
	10:16:45	7:45	RBNU	M	2
	10:09:00	0:00	RBNU	M	2
	15:30:18	28:18:00	MOCH	M	1
	15:27:34	25:34:00	MOCH	M	1
	15:30:08	28:08:00	MOCH	M	1
	15:30:50	28:50:00	MOCH	M	1
	15:27:40	25:40:00	MOCH	M	1
	15:28:40	26:40:00	MOCH	M	1
	15:26:55	24:55:00	MOCH	M	1
	15:31:20	29:20:00	MOCH	M	1
	15:25:45	23:45	MOCH	M	1
	15:12:30	10:30	RBNU	U	2
	15:11:00	9:00	RBNU	U	2
	15:10:50	0:08:50	RBNU	U	2
	15:15:40	13:40	RBNU	U	2
	15:04:30	2:30	RBNU	U	2
	15:07:00	5:00	RBNU	U	2
	15:14:00	12:00	RBNU	U	2
	15:15:55	13:55	RBNU	U	2
	15:17:30	15:30	RBNU	U	2
	15:18:15	16:15	RBNU	U	2
	15:18:19	16:19	RBNU	U	2
	15:14:36	12:36	RBNU	U	2
	15:04:55	2:55	RBNU	U	2
	15:12:35	10:35	RBNU	U	2
	15:26:26	24:26:00	RBNU	U	2
1		0:03:00	RBNU	F	1
11		0:08:20	RBNU	F	1
5		0:12:50	RBNU	F	1
1		0:05:20	RBNU	U	2
1		0:03:00	START		1
11		0:08:20	START		1
5		0:12:50	START		1
11		0:03:40	RBNU	M	2
1		0:17:00	RBNU	M	2
11		0:03:30	RBNU	M	2
		0:00:30	RBNU	M	2

11			0:07:00	RBNU	M	2
5			0:09:00	RBNU	M	2
5			0:14:20	RBNU	M	2
5			0:14:30	RBNU	M	2
11			0:02:59	START		
5			0:09:00	START		
1			0:15:30	START		
5	17	0	0:17:00	END		
1	7	15	0:07:15	RBNU	M	2
1	10	0	0:10:00	RBNU	M	1
5	12	0	0:12:00	RBNU	F	1
5	13	0	0:13:00	RBNU	M	1
5	14	0	0:14:00	RBNU	M	1
5	15	40	0:15:40	RBNU	M	1
11	2	10	0:02:10	RBNU	M	1
	0	35	0:00:35	RBNU	M	2
	0	38	0:00:38	RBNU	F	2
11	6	0	0:06:00	RBNU	M	1
1	7	20	0:07:20	RBNU	F	2
1	9	0	0:09:00	RBNU	M	1
5	12	20	0:12:20	RBNU	M,F	2
1	7	10	0:07:10	START		
5	11	40	0:11:40	START		
11	1	30	0:01:30	START		
	0	0	0:00:00	START		
1	18	0	0:18:00	END		
5	10	30	0:10:30	MOCH	M	1
1	13	52	0:13:52	MOCH	M	1
5	8	50	0:08:50	MOCH		1
5	9	20	0:09:20	MOCH	M	2
5	9	20	0:09:20	MOCH		2
11	5	44	0:05:44	RBNU	M	1
11	3	25	0:03:25	RBNU	M	1
1	13	25	0:13:25	RBNU	M	1
1	15	20	0:15:20	RBNU	M	1
5	9	30	0:09:30	RBNU	M	1
5	12	17	0:12:17	RBNU	M	1
1	13	13	0:13:13	START		
5	8	10	0:08:10	START		
11	3	20	0:03:20	START		
	0	0	0:00:00	START		
1	18	0	0:18:00	END		
1	13	20	0:13:20	MOCH		1
5	7	30	0:07:30	MOCH		1

5		10	30	0:10:30	MOCH		1
1		13	30	0:13:30	MOCH		1
1		14	0	0:14:00	MOCH		1
11		4	25	0:04:25	MOCH	M	1
11		2	0	0:02:00	RBNU	M	1
1		11	30	0:11:30	START		
5		6	15	0:06:15	START		
11		1	0	0:01:00	START		
	10:07	0	0	0:00:00	START		
1		18	0	0:18:00	END		
5		8	0	0:08:00	RBNU	M	1
11		0	35	0:00:35	RBNU	M	1
11		5	0	0:05:00	RBNU	M	1
5		12	30	0:12:30	RBNU	M	1
1		16	15	0:16:15	RBNU	M	1
1		13	20	0:13:20	RBNU	M,F	2
11		0	35	0:00:35	RBNU	F	1
1		13	10	0:13:10	START		
5		8	0	0:08:00	START		
11		0	20	0:00:20	START		
	8:50	0	0	0:00:00	START		
3	10:00	16	47	0:16:47	BCCH	M	1
3	10:00	16	8	0:16:08	BCCH	M	1
3	9:59	15	9	0:15:09	BCCH	M	1
11	9:53	9	0	0:09:00	RBNU		2
11	9:56	12	43	0:12:43	RBNU		1
11	9:51	7	58	0:07:58	RBNU		2
5	9:48	4	24	0:04:24	RBNU		1
5	9:47	3	30	0:03:30	RBNU		1
5	9:48	4	52	0:04:52	RBNU		1
11	9:51	7	12	0:07:12	RBNU		1
3	10:02	18	2	0:18:02	RBNU		1
	9:44	0	0	0:00:00	RBNU		1
5	9:46	2	0	0:02:00	start		
11	9:50	6	57	0:06:57	start		
3	9:58	14	3	0:14:03	start		
3	10:04	20	22	0:20:22	stop		
	9:44	0	0	0:00:00			
5	9:38	16	34	0:16:34	BCCH	M	1
3	9:36	14	0	0:14:00	BCCH	M	1
5	9:41	19	9	0:19:09	RBNU	M	1
5	9:39	17	26	0:17:26	RBNU	M	1
11	9:24	2	17	0:02:17	RBNU	M	1
5	9:39	17	49	0:17:49	RBNU	M	1

11	9:28	6	15	0:06:15	RBNU	M	1
11	9:29	7	0	0:07:00	RBNU	M	1
3	9:35	13	2	0:13:02	RBNU	M	1
3	9:36	14	12	0:14:12	RBNU	M	1
3	9:34	12	44	0:12:44	RBNU	M	1
3	9:35	13	40	0:13:40	RBNU	M	1
5	9:41	19	10	0:19:10	RBNU	M	1
5	9:41	19	43	0:19:43	RBNU	M	1
11	9:29	7	28	0:07:28	RBNU		2
11	9:23	1	2	0:01:02	start		
3	9:30	8	31	0:08:31	start		
5	9:38	16	4	0:16:04	start		
5	9:43	21	53	0:21:53	stop		
5	9:42	20	19	0:20:19			1
	9:22	0	0	0:00:00			
3	8:37	9	52	0:09:52	BCCH	M	1
3	8:41	13	8	0:13:08	BCCH	M	1
3	8:43	15	1	0:15:01	BCCH	M	1
3	8:43	15	27	0:15:27	BCCH	M	1
5	8:31	3	16	0:03:16	MOCH		1
5	8:31	3	37	0:03:37	MOCH		1
5	8:32	4	14	0:04:14	MOCH		2
3	8:37	9	52	0:09:52	MOCH		1
5	8:31	3	6	0:03:06	MOCH		1
5	8:33	5	19	0:05:19	MOCH	M	2
5	8:35	7	37	0:07:37	MOCH	M	2
3	8:38	10	49	0:10:49	MOCH		1
3	8:38	10	59	0:10:59	MOCH		1
5	8:32	4	2	0:04:02	MOCH	M	2
3	8:39	11	15	0:11:15	MOCH		1
3	8:39	11	46	0:11:46	MOCH	M	2
11	8:47	19	29	0:19:29	RBNU		2
11	8:47	19	17	0:19:17	RBNU		2
11	8:47	19	58	0:19:58	RBNU		2
11	8:46	18	59	0:18:59	RBNU		1
11	8:46	18	37	0:18:37	RBNU		1
11	8:48	20	16	0:20:16	RBNU		2
3	8:44	16	20	0:16:20	RBNU		1
3	8:44	16	30	0:16:30	RBNU		2
3	8:44	16	32	0:16:32	RBNU		2
11	8:46	18	2	0:18:02	RBNU		1
11	8:48	20	26	0:20:26	RBNU		2
11	8:48	20	35	0:20:35	RBNU		2
11	8:48	20	35	0:20:35	RBNU	M	2

11	8:50	22	40	0:22:40	RBNU		2
11	8:48	20	52	0:20:52	RBNU		2
11	8:49	21	20	0:21:20	RBNU		3
11	8:50	22	53	0:22:53	RBNU		2
11	8:47	19	7	0:19:07	RBNU		2
11	8:49	21	18	0:21:18	RBNU		2
11	8:52	24	34	0:24:34	RBNU		1
11	8:49	21	18	0:21:18	RBNU		2
11	8:53	25	8	0:25:08	RBNU		1
5	8:30	2	25	0:02:25	RUGR		1
5	8:29	1	39	0:01:39	start		
3	8:37	9	50	0:09:50	start		
11	8:46	18	0	0:18:00	start		
11	8:53	25	25	0:25:25	stop		
	8:28	0	0	0:00:00			
3	9:43	9	14	0:09:14	RBNU	F	1
3	9:46	12	51	0:12:51	RBNU	F	1
11	9:49	15	10	0:15:10	RBNU	M	1
	9:34	0	0	0:00:00	RBNU	F	2
5	9:37	3	59	0:03:59	RBNU	F	1
5	9:39	5	3	0:05:03	RBNU	F	1
11	9:49	15	45	0:15:45	RBNU	F	1
11	9:50	16	41	0:16:41	RBNU	M	1
11	9:50	16	53	0:16:53	RBNU	M	1
11	9:50	16	55	0:16:55	RBNU	F	1
11	9:51	17	3	0:17:03	RBNU	F	1
11	9:52	18	17	0:18:17	RBNU	F	1
11	9:52	18	25	0:18:25	RBNU	F	1
	9:34	0	0	0:00:00	RBNU	M	2
5	9:39	5	10	0:05:10	RBNU	F	1
11	9:50	16	41	0:16:41	RBNU	F	1
5	9:37	3	42	0:03:42	start		
3	9:42	8	44	0:08:44	start		
11	9:48	14	50	0:14:50	start		
11	9:53	19	56	0:19:56	stop		
	9:34	0	0	0:00:00			
11	10:27	3	48	0:03:48	RBNU	M	1
11	10:27	3	57	0:03:57	RBNU	M	1
11	10:28	4	24	0:04:24	RBNU	M	1
11	10:28	4	38	0:04:38	RBNU	M	1
11	10:28	4	43	0:04:43	RBNU	M	1
11	10:28	4	54	0:04:54	RBNU	M	1
11	10:27	3	28	0:03:28	RBNU	M	1
5	10:39	15	20	0:15:20	RBNU	M	1

11	10:25	1	54	0:01:54	RBNU		1
5	10:37	13	31	0:13:31	RBNU		1
11	10:25	1	15	0:01:15	start		
3	10:31	7	14	0:07:14	start		
5	10:36	12	7	0:12:07	start		
5	10:41	17	9	0:17:09	stop		
	10:24	0	0	0:00:00			
3	11:01	10	52	0:10:52	RBNU	M	1
3	11:00	9	6	0:09:06	RBNU	M	1
3	11:00	9	43	0:09:43	RBNU	M	1
3	11:01	10	54	0:10:54	RBNU	M	1
3	11:02	11	44	0:11:44	RBNU	M	1
11	11:05	14	30	0:14:30	RBNU	M	1
11	11:06	15	2	0:15:02	RBNU	M	1
11	11:07	16	29	0:16:29	RBNU	F	1
11	11:07	16	59	0:16:59	RBNU	M	1
11	11:04	13	5	0:13:05	RBNU	F	1
11	11:07	16	14	0:16:14	RBNU	M	1
11	11:07	16	14	0:16:14	RBNU	F	1
11	11:07	16	29	0:16:29	RBNU	M	1
11	11:04	13	31	0:13:31	RBNU	F	1
11	11:05	14	2	0:14:02	RBNU	F	1
11	11:06	15	50	0:15:50	RBNU	M	1
11	11:06	15	18	0:15:18	RBNU	M	1
11	11:08	17	12	0:17:12	RBNU	M	1
5	10:52	1	20	0:01:20	start		
3	10:57	6	29	0:06:29	start		
11	11:03	12	43	0:12:43	start		
11	11:10	19	35	0:19:35	stop		
	10:51	0	0	0:00:00			
1	9:24	3	30	0:03:30	RBNU	M	1
13	9:32	11	4	0:11:04	RBNU	M	1
13	9:26	5	37	0:05:37	RBNU	M	1
13	9:30	9	30	0:09:30	RBNU	M	1
13	9:32	11	20	0:11:20	RBNU	M	1
1	9:24	3	16	0:03:16	RBNU	M	1
1	9:23	2	45	0:02:45	RBNU	M	1
5	9:36	15	17	0:15:17	RBNU	M	1
13	9:33	12	53	0:12:53	RBNU	M	1
5	9:35	14	22	0:14:22	RBNU	M	1
5	9:37	16	1	0:16:01	RBNU	M	1
5	9:37	16	42	0:16:42	RBNU	M	1
5	9:35	14	56	0:14:56	RBNU	M	1
1	9:21	0	0	0:00:00	START		

5	9:34	13	30	0:13:30	START		
13	9:26	5	30	0:05:30	START		
1	9:13	4	6	0:04:06	BCCH	M	1
1	9:12	3	47	0:03:47	BCCH	M	1
1	9:12	3	56	0:03:56	BCCH	M	1
1	9:12	3	11	0:03:11	BCCH	M	1
1	9:13	4	22	0:04:22	BCCH	M	1
1	9:14	5	0	0:05:00	BCCH	M	1
1	9:11	2	51	0:02:51	BCCH	M	1
1	9:11	2	8	0:02:08	BCCH	M	1
1	9:10	1	47	0:01:47	BCCH	M	1
1	9:10	1	34	0:01:34	BCCH	M	1
1	9:10	1	5	0:01:05	BCCH	M	1
13	9:27	18	4	0:18	DOWO	M	1
13	9:30	21	13	0:21	DOWO	M	1
13	9:30	21	54	0:21:54	DOWO	M	1
5	9:19	10	25	0:10:25	MOCH	M	1
5	9:22	13	51	0:13:51	MOCH	M	2
5	9:22	13	2	0:13:02	MOCH	M	2
5	9:18	9	10	0:09:10	MOCH	M	1
5	9:19	10	14	0:10:14	MOCH	M	2
5	9:20	11	42	0:11:42	MOCH	M	2
5	9:20	11	11	0:11:11	MOCH	M	2
5	9:17	8	49	0:08:49	MOCH	M	1
5	9:22	13	30	0:13:30	MOCH	M	2
5	9:18	9	31	0:09:31	MOCH	F	2
5	9:19	10	21	0:10:21	MOCH	M	2
5	9:23	14	49	0:14:49	MOCH	M	1
5	9:24	15	30	0:15:30	MOCH	M	1
5	9:19	10	34	0:10:34	MOCH	M	2
13	9:29	20	18	0:20:18	RBNU	M	1
13	9:29	20	22	0:20:22	RBNU	M	1
13	9:27	18	53	0:18:53	RBNU	M	1
13	9:28	19	30	0:19:30	RBNU	M	1
13	9:27	18	37	0:18:37	RBNU	M	1
13	9:29	20	46	0:20:46	RBNU	M	1
13	9:28	19	23	0:19:23	RBNU	M	1
13	9:27	18	24	0:18:24	RBNU	M	1
13	9:30	21	23	0:21:23	RBNU	M	1
13	9:31	22	28	0:22:28	RBNU	M	1
13	9:33	24	10	0:24:10	RBNU	M	1
13	9:34	25	59	0:25:59	RBNU	M	1
13	9:35	26	49	0:26:49	RBNU	M	1
13	9:37	28	0	0:28:00	RBNU	M	1

5	9:21	12	11	0:12:11	RBNU	M	1
1	9:09	0	0	0:00:00	START		
5	9:16	7	30	0:07:30	START		
13	9:26	17	30	0:17:30	START		
	13:01:34			9:34	MOCH	M	1
	13:00:08			8:08	MOCH	M	1
	13:03:00			11:00	MOCH	M	1
	12:54:17			2:17	MOCH	M	1
	12:59:00			7:00	MOCH	M	1
	13:06:55			14:55	RBNU	M	1
	13:06:50			14:50	RBNU	M	1
	13:08:00			16:00	RBNU	M	1
	13:08:01			16:01	RBNU	M	1
	13:09:11			17:11	RBNU	M	1
	13:10:20			18:20	RBNU	M	1
	10:28:30			3:30	RBNU	M	1
	10:28:08			3:08	RBNU	M	1
	10:30:45			5:45	RBNU	M	1
	10:25:00			0:00	RBNU	M	1
	10:31:20			6:20	RBNU	M	1
	10:34:40			9:40	RBNU	M	1
	10:34:46			9:46	RBNU	M	1
	10:36:00			11:00	RBNU	M	1
	10:38:21			13:21	RBNU	M	1
	7:55:00				RBNU	M	1
	7:54:00				RBNU	M	1
	7:58:00				RBNU	M	1
	8:00:00				RBNU	M	1
	14:21:00				MOCH	U	1
	14:57:00				MOCH	U	1
	15:04:10				RBNU	M	1
	15:01:20				RBNU	M	1
	15:03:01				RBNU	M	1
	15:10:00				RBNU	M	1
	15:11:40				RBNU	M	1
	15:12:20				RBNU	M	1
	15:08:40				RBNU	M	1
	15:13:05				RBNU	M	1
	13:35:30				MOCH	U	1
	13:35:55				MOCH	U	1
	13:49:50				RBNU	U	1
	9:17:16				MOCH	U	1
	9:27:15				RBNU	M	1
	9:32:50				RBNU	M	1

9:58:40	8:40	RBNU	U	1
10:15:30	25:30:00	RBNU	U	1
10:08:50	18:50	TAHU	U	1
8:25:30	25:30:00	BCCH	U	1
8:27:20	27:20:00	BCCH	U	1
8:22:05	22:05	BCCH	U	1
8:22:50	22:50	BCCH	M	1
8:24:35	24:35:00	BCCH	M	1
8:22:15	22:15	BCCH	U	1
8:07:00	7:00	MOCH	U	1
8:06:10	6:10	MOCH	U	1
8:14:10	9:10	BCCH	U	1
8:19:30	14:30	RBNU	M	1
8:19:10	14:10	RBNU	M	1
8:19:00	14:00	RBNU	M	1
8:22:20	17:20	RBNU	M	1
8:22:50	17:50	RBNU	M	1
8:15:10	10:10	RBNU	U	1
8:09:00	4:00	TAHU	U	1
10:55:25	18:25	RBNU	U	1
9:44:20	6:20	BCCH	M	2
9:47:30	9:30	BCCH	U	3
9:44:05	6:05	BCCH	M	1
9:44:50	6:50	BCCH	U	1
9:49:10	11:10	BCCH	U	2
9:44:20	6:20	BCCH	U	2
9:45:00	7:00	BCCH	U	3
9:54:25	16:25	BCCH	U	1
9:58:10	20:10	BCCH	M	1
9:59:40	21:40	BCCH	M	1
9:52:50	14:50	RBNU	M	1
9:53:00	15:00	RBNU	M	1
9:52:30	14:30	RBNU	M	1
9:51:40	13:40	RBNU	U	1
9:53:20	15:20	RBNU	M	1
9:55:05	17:05	RBNU	U	1
9:58:20	20:20	RBNU	U	1
9:56:15	18:15	RBNU	U	1
10:40:30	16:30	MOCH	U	1
10:33:35	9:35	MOCH	U	1
10:40:00	16:00	RBNU	U	1
9:26:30	16:30	BCCH	U	1
9:25:30	15:30	BCCH	U	1
9:27:00	17:00	BCCH	U	1

9:28:50			18:50	BCCH	U	1
9:32:20			22:20	BCCH	C	1
9:29:50			19:50	BCCH	M	2
9:32:37			22:37	RBNU	M	1
9:37:35			27:35:00	RBNU	M	1
9:37:37			27:37:00	RBNU	M	1
9:32:35			22:35	RBNU	M	1
9:33:10			23:10	RBNU	M	1
9:38:30			28:30:00	RBNU	M	1
9:42:12			27:12:00	MOCH	U	1
9:24:50			9:50	RBNU	U	1
9:30:20			15:20	RBNU	U	1
8:46:00	16	0	16:00	BCCH	U	2
8:50:00	20	0	20:00	BCCH	U	2
8:53:00	23	0	23:00	BCCH	U	2
8:48:20	18	20	18:20	BCCH	U	2
8:48:40	18	40	18:40	BCCH	U	2
8:48:00	18	0	18:00	BCCH	U	2
8:51:30	21	30	21:30	BCCH	U	2
8:37:20	7	20	7:20	BCCH	U	2
8:53:50	23	50	23:50	BCCH	U	2
8:36:30	6	30	6:30	BCCH	U	2
8:37:50	7	50	7:50	BCCH	U	2
8:37:00	7	0	7:00	BCCH	U	2
8:52:20	22	20	22:20	BCCH	M	2
8:40:30	10	30	10:30	BCCH	U	2
8:39:20	9	20	9:20	BCCH	U	2
8:45:40	15	40	15:40	BCCH	U	2
8:46:40	16	40	16:40	BCCH	U	2
8:47:40	17	40	17:40	BCCH	U	2
8:53:20	23	20	23:20	BCCH	U	2
8:30:25	0	25	0:25	MOCH	U	2
8:32:00	2	0	2:00	MOCH	U	2
8:32:00	2	0	2:00	MOCH	U	2
8:36:20	6	20	6:20	MOCH	U	2
8:44:00	14	0	14:00	MOCH	U	2
8:53:20	23	20	23:20	MOCH	U	1
8:46:20	16	20	16:20	MOCH	U	2
8:46:40	16	40	16:40	MOCH	U	2
8:45:00				MOCH	U	1
9:02:11				MOCH	M	1
9:07:45				TRES	U	1
10:48:15				NIL		
11:29:31				BCCH	U	2

11:34:10	BCCH	U	2
11:36:09	BCCH	M	2
11:29:30	BCCH	U	2
11:27:50	BCCH	M	2
11:35:00	BCCH	M	2
11:27:00	BCCH	U	2
11:40:15	BCCH	M	2
11:37:02	BCCH	M	2
8:56:00	BCCH	M	2
8:57:00	BCCH	M	2
8:58:29	BCCH	M	2
8:57:00	BCCH	F	2
8:58:29	BCCH	F	2
8:56:10	BCCH	F	2
8:52:50	BCCH	M	1
8:54:50	BCCH	M	1
8:41:00	BCCH	U	1
8:45:10	BCCH	M	1
8:46:00	MOCH	U	1
8:42:30	MOCH	U	1
8:45:00	RBNU	M	1
8:44:00	RBNU	M	1
8:47:00	RBNU	M	1
8:53:30	RBNU	M	1
8:54:50	RBNU	M	1
8:56:10	RBNU	M	1
9:14:40	BCCH	U	1
9:30:17	DOWO	F	1
9:42:20	DOWO	U	1
9:22:20	MOCH	U	2
9:21:50	MOCH	U	2
9:15:30	MOCH	U	2
9:21:10	MOCH	U	2
9:39:10	MOCH	U	1
9:40:00	MOCH	U	2
9:38:10	RBNU	M	2
9:31:51	RBNU	U	2
9:33:40	RBNU	M	2
9:35:00	RBNU	M	2
9:35:00	RBNU	F	2
9:42:10	RBNU	M	2
9:28:40	RBNU	M	1
9:28:20	RBNU	M	1
9:28:40	RBNU	U	2

9:30:50		RBNU	U		1
9:18:04		RBNU	U		1
9:25:30		TAHU	U		1
9:09:00		RBNU	U		1
8:50:00		TAHU	U		1
0:22:25	22:25	BCCH	M		4
0:18:04	18:04	BCCH	U		4
0:21:30	21:30	BCCH	B		4
0:07:02	7:02	BCCH	M		4
0:11:05	11:05	BCCH	M		4
0:14:00	14:00	BCCH	U		4
0:14:15	14:15	BCCH	M		4
0:24:20	24:20:00	BCCH	M		4
0:26:00	26:00:00	BCCH	U		4
0:19:20	19:20	BCCH	B		4
0:27:00	27:00:00	BCCH	B		4
9:41:00	2:00	BCCH	U		4
0:09:00	9:00	BCCH	M		
0:17:45	17:45	BCCH	M		
0:13:00	13:00	TRES	U		
0:25:30	25:30:00	TRES	U		
11:29:00	15:00	BCCH	M		
11:24:00	10:00	MOCH	M		
11:19:40	5:40	MOCH	U		
11:20:30	6:30	MOCH	M		
11:22:41	8:41	MOCH	M		2
11:22:41	8:41	MOCH			2
11:30:40	16:40	MOCH	M		
11:37:00	23:00	MOCH	M		
10:17:30	14:30	TRES	U		2
10:13:47	10:47	TRES	U		2
10:17:30	14:30	TRES	U		2
10:09:00	6:00	TRES	B		2
10:20:00	17:00		-	-	
10:50:00	8:00	HAWO	U		
11:03:30	21:30	MOBL	M		
10:45:20	3:20	MOBL	U		
10:44:30	2:30	MOCH	U	-	
10:49:00	7:00	MOCH	M		
10:49:20	7:20	MOCH	M		
12:33:00	14:00	MOCH	M		2
12:33:15	14:15	MOCH	F		2
12:19:30	0:30	MOCH	B		2
12:26:00	7:00	MOCH	B		2

	12:39:30			20:30	TRES		4
	9:56:00			3:00	MOCH	M	2
	9:56:00			3:00	MOCH	F	2
	10:18:50			25:50:00	MOCH	U	1
	9:53:00			0:00	MOCH	M	2
	10:53:00			0:00	MOCH	F	2
	10:15:18			22:18	MOCH	U	2
	10:15:20			22:20	MOCH	U	2
	10:19:40			26:40:00	MOCH	M	1
	10:12:20			19:20	RBNU	U	1
	9:12:28			2:28	MOCH	U	1
	9:12:30			2:30	MOCH	U	2
	9:13:02			3:02	MOCH	U	2
	9:14:20			4:20	MOCH	U	2
	9:14:50			4:50	MOCH	U	2
	9:13:51			3:51	MOCH	U	2
	9:15:00			5:00	MOCH	U	2
	9:25:50			15:50	MOCH	M	1
	9:24:40			14:40	MOCH	U	1
	9:17:20			7:20	RBNU	U	1
	9:20:15			10:15	RBNU	U	1
	9:23:30			13:30	RBNU	U	1
	10:58:20			24:20:00	MOCH	U	1
	10:59:00			25:00:00	MOCH	U	1
	10:47:00			13:00	MOCH	U	1
	10:47:40			13:40	MOCH	U	1
	10:53:30			19:30	MOCH	U	1
	10:54:20			20:20	MOCH	U	1
	10:57:40			23:40	MOCH	U	1
	10:46:40			12:40	MOCH	U	1
	10:56:00			22:00	MOCH	U	1
	10:57:00			23:00	MOCH	U	1
	9:24	0	0	0	NIL		0
5	9:27			0:03:00	NIL		0
11				0:13:30	RBNU	M	1
11				0:13:00	RBNU	M	1
11				0:11:15	RBNU	M	1
11				0:11:30	RBNU	M	1
1				0:19:00	RBNU	M	1
11	9:34			0:11:00	RBNU	M	1
11				0:14:00	RBNU	M	2
11				0:14:30	RBNU	F	2
11				0:13:40	RNSA	U	1
				0:00:00	MOCH	U	1

				0:01:50	RBNU	U			1
5				0:05:50	MOCH	M			1
5				0:05:00	MOCH	M			1
5				0:04:20	MOCH	M			1
5				0:04:00	MOCH	M			1
1				0:12:00	MOCH	U			1
1				0:12:30	RBNU	U			1
11				0:19:00	RBNU	M			1
11				0:22:50	RBNU	M			1
11				0:18:40	RBNU	M			1
11				0:17:50	RBNU	M			1
11				0:17:00	RBNU	M			1
11				0:26:00	RBNU	M			1
5				0:07:50	BCCH	M			1
5				0:06:50	MOCH	M			2
5				0:05:38	MOCH	B			2
5				0:07:10	MOCH	M			2
5				0:04:50	MOCH	B			2
13				0:12:00	MOCH	B			2
1				0:19:30	MOCH	B			2
13				0:12:50	RBNU	M			1
				0:00:00	RBNU	B			2
5				0:03:12	RBNU	B			2
13				0:14:50	RBNU	M			1
13				0:16:00	RBNU	M			1
1				0:18:41	RBNU	M			1
1				0:25:00	RBNU	M			1
				0:00:00	BCCH	B	RPAG	ATY	2
1				0:08:30	MOCH	M			1
1				0:09:00	MOCH	M			1
1				0:05:30	MOCH	M			1
					NIL				
					NIL				
11		14	0	0:14:00	END				
1		6	20	0:06:20	START				
5		2	0	0:02:00	START				
11		10	0	0:10:00	START				
	8:37	0	0	0:00:00	START				
5		7	0	0:07:00	BCCH	M			2
13		2	50	0:02:50	DEJU	U			1
5		15	28	0:15:28	MOCH	M			2
5		15	0	0:15:10	MOCH	M			2
5		15	55	0:15:55	MOCH	M			2
5		13	44	0:13:44	MOCH	M			2

5	15	40	0:15:40	MOCH	F	2
5	16	20	0:16:20	MOCH	M	2
5	16	20	0:16:20	MOCH	F	2
5	17	5	0:17:05	MOCH	M	2
5	17	5	0:17:05	MOCH	F	2
5	13	30	0:13:30	MOCH	M	2
1	18	15	0:18:15	MOCH	M	2
1	19	50	0:19:50	MOCH	F	2
5	12	34	0:12:34	MOCH	M	2
5	9	30	0:09:30	MOCH	M	2
5	11	6	0:11:06	MOCH	M	2
5	9	9	0:09:09	MOCH	M	2
5	12	10	0:12:10	MOCH	F	2
5	9	10	0:09	MOCH	F	2
1	21	47	0:21:47	MOCH	U	2
1	22	48	0:22:48	MOCH	F	2
1	24	5	0:24:05	MOCH	M	2
5	8	30	0:08:30	MOCH	M	1
5	8	12	0:08:12	MOCH	M	1
1	28	0	0:28:00	MOCH	F	2
13	0	0	0:00:00	START		
5	7	0	0:07:00	START		
1	18	15	0:18:15	START		
5	8	20	0:08:20	MOCH	U	1
5	9	50	0:09:50	MOCH	M	2
5	12	12	0:12:12	MOCH	M	2
5	12	12	0:12:12	MOCH	F	2
5	9	0	0:09:00	MOCH	M	1
13	6	30	0:06:30	MOCH	U	1
1	15	0	0:15:00	START		
5	0	0	0:00:00	MOCH	M	1
3	15	1	0:15:01	MOCH	M	1
5	3	9	0:03:09	MOCH	M	1
5	4	20	0:04:20	MOCH	M	1
13	14	2	0:14:02	MOCH	M	1
3	15	1	0:15:01	MOCH	M	1
3	19	29	0:19:20	MOCH	M	1
13	9	55	0:09:55	RBNU	M	1
13	10	20	0:10:20	RBNU	M	1
13	9	46	0:09:46	RNSA	U	1
13	7	30	0:07:30	START		
3	15	0	0:15:00	START		
1	17	35	0:17:35	MOCH	U	1
5	2	32	0:02:32	MOCH	M	1

5	3	34	0:03:34	MOCH	M	1
5	5	27	0:05:27	MOCH	M	1
5	5	52	0:05:52	MOCH	M	1
1	16	27	0:16:27	MOCH	U	1
1	16	50	0:16:50	MOCH	U	1
1	17	10	0:17:10	MOCH	U	1
5	2	42	0:02:42	MOCH	M	1
5	3	28	0:03:28	MOCH	M	1
5	4	58	0:04:58	MOCH	M	1
5	5	10	0:05:10	MOCH	M	1
5	5	39	0:05:39	MOCH	M	1
1	16	6	0:16:06	MOCH	U	1
1	16	11	0:16:11	MOCH	U	1
1	18	0	0:18:00	MOCH	U	1
5	2	27	0:02:27	MOCH	M	3
1	16	32	0:16:32	MOCH	U	1
5	6	27	0:06:27	MOCH	M	1
1	18	13	0:18:13	MOCH	U	1
5	2	9	0:02:09	MOCH	M	1
1	17	22	0:17:22	MOCH	U	1
5	1	56	0:01:56	MOCH	M	1
5	1	3	0:01:03	MOCH	M	1
5	1	32	0:01:32	MOCH	M	1
5	0	25	0:00:25	MOCH	M	1
5	3	59	0:03:59	MOCH	M	1
1	18	24	0:18:24	MOCH	U	1
1	18	44	0:18:44	MOCH	U	1
1	19	54	0:19:54	MOCH	U	1
13	8	1	0:08:01	MOCH	M	1
13	9	7	0:09:07	MOCH	M	1
13	9	13	0:09:13	MOCH	U	1
1	15	49	0:15:49	MOCH	U	1
13	10	18	0:10:18	MOCH	M	2
13	11	53	0:11:53	MOCH	M	2
13	10	48	0:10:48	MOCH	M	2
13	12	11	0:12:11	MOCH	M	2
5	0	3	0:00:03	MOCH	U	1
5	0	0	0:00:00	START		
13	7	40	0:07:40	START		
1	15	0	0:15:00	START		
5	4	21	0:04:21	BCCH	M	1
5	7	10	0:07:10	BCCH	M	1
1	10	11	0:10:11	BCCH	M	1
1	10	46	0:10:46	BCCH	M	1

1	11	26	0:11:26	BCCH	M	1
1	11	50	0:11:50	BCCH	M	1
1	9	7	0:09:07	BCCH	M	1
5	7	22	0:07:22	BCCH	M	1
1	8	3	0:08:03	BCCH	M	1
1	13	3	0:13:03	BCCH	M	1
1	13	18	0:13:18	BCCH	M	1
5	0	41	0:00:41	BOCH	M	1
5	0	35	0:00:35	BOCH	M	1
1	8	35	0:08:35	BOCH	U	1
1	9	58	0:09:58	BOCH	U	1
5	3	33	0:03:33	BOCH	M	1
1	13	3	0:13:03	BOCH	U	1
13	18	53	0:18:53	CHIC	U	1
5	0	10	0:00:10	MOCH	F	1
5	3	33	0:03:33	MOCH	F	1
5	6	50	0:06:50	MOCH	F	1
5	5	39	0:05:39	MOCH	F	1
13	19	53	0:19:53	MOCH	M	1
5	0	0	0:00:00	START		
1	7	30	0:07:30	START		
13	15	0	0:15:00	START		
1	18	53	0:18:53	BCCH	M	1
5	10	7	0:10:07	BOCH	M	1
5	9	1	0:09:01	BOCH	M	1
1	17	27	0:17:27	BOCH	M	1
5	12	16	0:12:16	BOCH	M	1
1	18	25	0:18:25	BOCH	M	1
5	9	1	0:09:01	MOCH	F	1
5	10	46	0:10:46	MOCH	F	2
5	10	46	0:10:46	MOCH	M	2
1	17	45	0:17:45	MOCH	F	1
13	4	18	0:04:18	MOCH	U	1
13	5	3	0:05:03	MOCH	U	1
1	18	25	0:18:25	MOCH	F	1
1	18	53	0:18:53	MOCH	F	1
5	9	1	0:09:01	MOCH	F	1
13	2	26	0:02:26	RBNU	M	1
13	2	22	0:02:22	RBNU	M	1
13	2	59	0:02:59	RBNU	M	1
13	3	37	0:03:37	RBNU	M	1
13	3	56	0:03:56	RBNU	U	2
13	3	56	0:03:56	RBNU	M	2
13	4	44	0:04:44	RBNU	U	2

13	2	37	0:02:37	RBNU	M	1
5	12	36	0:12:36	RBNU	M	1
5	10	10	0:10:10	RBNU	M	1
5	8	32	0:08:32	RBNU	F	1
13	0	0	0:00:00	START		
5	8	32	0:08:32	START		
1	15	3	0:15:03	START	U	
13	9	56	0:09:56	RBNU	M	1
13	10	28	0:10:28	RBNU	M	1
13	10	36	0:10:36	RBNU	M	1
13	11	34	0:11:34	RBNU	U	2
13	11	7	0:11:07	RBNU	U	2
13	12	2	0:12:02	RBNU	M	1
13	12	30	0:12:30	RBNU	M	1
13	13	22	0:13:22	RBNU	M	1
13	14	11	0:14:11	RBNU	M	1
13	15	3	0:15:03	RBNU	M	1
5	18	12	0:18	RBNU	M	1
5	18	30	0:19:30	RBNU	M	1
5	19	52	0:19:52	RBNU	M	1
5	20	33	0:20:33	RBNU	M	1
5	21	3	0:21:03	RBNU	M	1
1	0	0	0:00:00	RBNU	M	1
13	7	30	0:07:30	start		
5	19	0	0:19:00	MOCH	M	1
1	30	38	0:30:38	MOCH	M	1
5	17	52	0:17:52	MOCH	M	1
1	28	50	0:28:50	MOCH	M	1
1	31	22	0:31:22	MOCH	M	1
5	11	1	0:11:01	MOCH	M	1
5	13	27	0:13:27	MOCH	M	1
5	14	11	0:14:11	MOCH	M	1
5	10	7	0:10:07	MOCH	M	1
5	13	42	0:13:42	MOCH	M	1
5	16	29	0:16:29	MOCH	M	1
1	28	58	0:28:58	MOCH	F	2
1	33	13	0:33:13	MOCH	M	1
1	34	9	0:34:09	MOCH	M	1
1	36	0	0:36:00	MOCH	M	1
5	21	3	0:21:03	MOCH	F	2
5	21	19	0:21:19	MOCH	M	1
5	23	7	0:23:07	MOCH	M	1
5	24	3	0:24:03	MOCH	M	1
1	29	13	0:29:13	MOCH	F	2

5	9	1	0:09:01	MOCH	M	1
5	12	7	0:12:07	MOCH	M	1
5	13	4	0:13:04	MOCH	M	1
5	17	2	0:17:02	MOCH	F	2
1	29	41	0:29:41	MOCH	F	2
1	30	51	0:30:51	MOCH	M	1
1	30	5	0:30:05	MOCH	M	1
1	36	39	0:36:39	MOCH	M	1
1	37	4	0:37:04	MOCH	M	1
5	8	20	0:08:20	MOCH	M	1
5	24	35	0:24:35	MOCH	M	1
1	37	7	0:37:07	MOCH	M	1
5	8	3	0:08:03	MOCH	M	1
1	27	36	0:27:36	MOCH	M	1
5	26	0	0:26:00	MOCH	M	
1	37	30	0:37:30	MOCH	M	
13	6	38	0:06:38	RBNU		1
13	0	0	0:00:00	start		
5	7	30	0:07:30	start		
1	27	0	0:27:00	start		
13	1	30	0:01:30	U		1
13	0	0	0:00:00	start		
1	7	30	0:07:30	start		
5	15	0	0:15:00	start		
5	2	20	0:02:20	MOCH	M	2
5	2	28	0:02:28	MOCH	M	2
1	15	55	0:15:55	MOCH	M	1
5	1	52	0:01:52	MOCH	M	2
1	16	13	0:16:13	MOCH	M	1
5	1	41	0:01:41	MOCH	F	2
1	15	30	0:15:30	MOCH	M	1
5	1	24	0:01:24	MOCH	M	1
13	7	43	0:07:43	MOCH	M	1
13	11	14	0:11:14	MOCH	U	1
5	2	46	0:02:46	MOCH	M	2
5	3	1	0:03:01	MOCH	M	2
5	3	1	0:03:01	MOCH	F	2
1	17	7	0:17:07	MOCH	M	2
1	19	37	0:19:37	MOCH	U	2
5	1	1	0:01:01	MOCH	M	1
5	3	32	0:03:32	MOCH	M	2
5	3	32	0:03:32	MOCH	F	2
13	8	22	0:08:22	MOCH	M	1
13	10	3	0:10:03	MOCH	U	1

5		0	23	0:00:23	MOCH	M	1
13		7	59	0:07:59	MOCH	M	1
1		20	35	0:20:35	MOCH	M	1
5		5	12	0:05:12	MOCH	U	1
5		4	3	0:04:03	MOCH	U	1
1		16	4	0:16:41	MOCH	M	2
1		17	43	0:17:43	MOCH	M	2
1		16	28	0:16:28	MOCH	U	2
5		0	0	0:00:00	start		
13		7	28	0:07:28	start		
1		15	0	0:15:00	start		
5	9:25	13	40	0:13:40	CHIC	U	1
5	9:22	10	43	0:10:43	MOCH	M	2
5	9:20	8	50	0:08:50	MOCH	M	1
5	9:21	9	57	0:09:57	MOCH	M	1
5	9:22	10	43	0:10:43	MOCH	U	2
1	9:28	16	8	0:16:08	MOCH	M	1
1	9:28	16	41	0:16:41	MOCH	M	1
1	9:29	17	13	0:17:13	MOCH	M	1
1	9:31	19	19	0:19:19	MOCH	M	1
5	9:20	8	0	0:08:00	MOCH	M	1
1	9:32	20	37	0:20:37	MOCH	M	1
13	9:14	2	39	0:02:39	RBNU	M	1
13	9:15	3	31	0:03:31	RBNU	M	1
13	9:16	4	23	0:04:23	RBNU	M	1
13	9:18	6	20	0:06:20	RBNU	M	1
5	9:25	13	18	0:13:18	RBNU	M	1
1	9:30	18	8	0:18:08	RBNU	M	1
1	9:31	19	19	0:19:19	RBNU	M, F	2
13	9:12	0	0	0:00:00	START		
5	9:19	7	30	0:07:30	START		
1	9:27	15	0	0:15:00	START		
1	9:35	23	0	0:23:00	STOP		
	9:12	0	0	0:00:00			
1	0:03	3	11	0:03:11	BCCH	M	1
5	0:08	8	31	0:08:31	MOCH	M	1
13	0:16	16	36	0:16:36	RBNU	F	1
13	0:17	17	13	0:17:13	RBNU	M	2
13	0:18	18	5	0:18:05	RBNU	M	2
13	0:16	16	52	0:16:52	RBNU	F	1
13	0:18	18	37	0:18:37	RBNU	F	2
13	0:19	19	12	0:19:12	RBNU	M	2
1	11:48	0	0	0:00:00	START		
5	0:07	7	30	0:07:30	START		

13	0:15	15	0	0:15:00	START		
1	0:02	2	7	0:02:07	BCCH	M	1
1	0:02	2	47	0:02:47	BCCH	M	1
1	0:03	3	28	0:03:28	BCCH	M	1
1	0:01	1	42	0:01:42	BCCH	M	1
1	0:01	1	7	0:01:07	BCCH	M	1
1	0:00	0	24	0:00:24	BCCH	M	1
5	0:08	8	31	0:08:31	BCCH	M	1
5	0:09	9	30	0:09:30	BCCH	M	1
5	0:09	9	44	0:09:44	BCCH	M	1
13	0:17	17	16	0:17:16	RBNU	M	2
13	0:17	17	16	0:17:16	RBNU	F	2
13	0:18	18	23	0:18:23	RBNU	B	2
13	0:18	18	54	0:18:54	RBNU	B	2
13	0:19	19	4	0:19:04	RBNU	B	2
1	0:03	3	46	0:03:46	RBNU	M	2
1	0:04	4	55	0:04:55	RBNU	M	2
1	0:06	6	5	0:06:05	RBNU	M	2
5	0:07	7	40	0:07:40	RBNU	M	1
13	0:20	20	15	0:20:15	RBNU	B	2
13	0:21	21	0	0:21	RBNU	M	2
1	0:02	2	17	0:02:17	RBNU	M	1
5	0:10	10	7	0:10:07	RBNU	M	1
5	0:10	10	59	0:10:59	RBNU	M	1
5	0:11	11	41	0:11:41	RBNU	M	1
13	0:19	19	10	0:19:10	RBNU	U	3
1	10:32	0	0	0:00:00	START		
5	0:07	7	39	0:07:39	START		
13	0:15	15	0	0:15:00	START		
5	#VALUE!	2	12	0:02:12	MOCH	M	1
5	#VALUE!	7	30	0:07:30	MOCH	M	2
5	#VALUE!	7	55	0:07:55	MOCH	M	1
5	#VALUE!	10	58	0:10:58	MOCH	M	2
13	#VALUE!	19	31	0:19:31	MOCH	M	2
1	#VALUE!	25	46	0:25:46	MOCH	M	1
5	#VALUE!	12	37	0:12:37	MOCH	M	2
13	#VALUE!	14	0	0:14:00	MOCH	M	2
13	#VALUE!	14	51	0:14:51	MOCH	M	2
13	#VALUE!	16	6	0:16:06	MOCH	M	2
13	#VALUE!	17	29	0:17:29	MOCH	M	2
5	#VALUE!	2	33	0:02:33	MOCH	M	2
5	#VALUE!	4	19	0:04:19	MOCH	M	2
5	#VALUE!	5	55	0:05:55	MOCH	M	2
5	#VALUE!	0	0	0:00:00	START		

13	#VALUE!	13	30	0:13:30	START		
1	#VALUE!	21	0	0:21:00	START		
5	9:13	5	4	0:05:04	MOCH	M	1
5	9:13	5	56	0:05:56	MOCH	M	1
5	9:14	6	17	0:06:17	MOCH	M	1
5	9:14	6	41	0:06:41	MOCH	M	1
5	9:12	4	51	0:04:51	MOCH	M	1
5	9:16	8	34	0:08:34	MOCH	M	2
5	9:18	10	36	0:10:36	MOCH	M	2
5	9:12	4	20	0:04:20	MOCH	M	1
5	9:15	7	0	0:07:00	MOCH	M	2
1	9:29	21	54	0:21:54	MOCH	M	1
5	9:08	0	0	0:00:00	START		
13	9:21	13	0	0:13:00	START		
1	9:28	20	30	0:20:30	START		
1	9:31	23	26	0:23:26	TRES	U	1
1	10:25	11	2	0:11:02	BCCH	F	2
1	10:24	10	34	0:10:34	BCCH	M	2
1	10:25	11	50	0:11:50	BCCH	M	2
1	10:23	9	50	0:09:50	BCCH	M	1
1	10:22	8	0	0:08:00	BCCH	M	1
1	10:23	9	34	0:09:34	BCCH	M	1
1	10:25	11	35	0:11:35	MOCH	M	1
5	10:33	19	15	0:19:15	MOCH	M	1
13	10:18	4	42	0:04:42	RBNU	M	2
13	10:19	5	5	0:05:05	RBNU	M	2
13	10:19	5	20	0:05:20	RBNU	M	2
1	10:22	8	50	0:08:50	RBNU	M	1
13	10:18	4	51	0:04:51	RBNU	M	2
1	10:25	11	17	0:11:17	RBNU	M	1
	10:14	0	0	0:00:00	RBNU	M	1
13	10:19	5	10	0:05:10	RBNU	M	2
13	10:16	2	55	0:02:55	RBNU	M	1
13	10:18	4	28	0:04:28	RBNU	F	2
13	10:14	0	0	0:00:00	START		
1	10:21	7	59	0:07:59	START		
5	10:29	15	30	0:15:30	START		
11	10:53	17	52	0:17:52	DOWO		1
1	10:36	0	28	0:00:28	MOCH		1
5	10:45	9	6	0:09:06	MOCH	M	2
5	10:48	12	37	0:12:37	MOCH	F	2
5	10:48	12	43	0:12:43	MOCH	M	2
11	10:52	16	31	0:16:31	MOCH	F	2
5	10:44	8	51	0:08:51	MOCH	M	2

5	10:47	11	1	0:11:01	MOCH	M	1
5	10:48	12	26	0:12:26	MOCH	M	1
5	10:48	12	34	0:12:34	MOCH	M	1
5	10:49	13	48	0:13:48	MOCH	M	2
5	10:44	8	24	0:08:24	MOCH	M	2
5	10:44	8	11	0:08:11	MOCH	M	2
1	10:39	3	38	0:03:38	MOCH	M	1
5	10:45	9	27	0:09:27	MOCH	F	2
11	10:52	16	45	0:16:45	RBNU	M	1
11	10:53	17	53	0:17:53	RBNU	M	1
11	10:55	19	48	0:19:48	RBNU	M	1
5	10:47	11	28	0:11:28	RBNU	M	1
1	10:42	6	4	0:06:04	RBNU		1
5	10:46	10	19	0:10:19	RBNU	M	1
1	10:36	0	0	0:00:00	start		
5	10:43	7	30	0:07:30	start		
11	10:51	15	0	0:15:00	start		
11	10:56	20	56	0:20:56	stop		
	10:36	0	0	0:00:00			
1	11:14	7	10	0:07:10	BCCH	M	1
1	11:15	8	1	0:08:01	BCCH	M	1
1	11:15	8	52	0:08:52	BCCH	M	1
1	11:13	6	29	0:06:29	BCCH	M	1
13	11:24	17	2	0:17:02	MOCH	U	1
5	11:10	2	0	0:02:00	MOCH	M	1
5	11:11	3	17	0:03:17	MOCH	M	1
13	11:20	13	30	0:13:30	MOCH	M	1
5	11:12	5	0	0:05:00	MOCH	M	2
5	11:12	5	0	0:05:00	MOCH	M	2
13	11:22	15	15	0:15:15	RBNU	M	1
13	11:20	13	13	0:13:13	RBNU	M	1
13	11:19	12	42	0:12:42	RBNU	M	1
13	11:19	12	10	0:12:10	RBNU	M	1
13	11:19	12	15	0:12:15	RBNU	M	1
13	11:21	14	58	0:14:58	RBNU	M	1
13	11:18	11	56	0:11:56	RBNU	M	1
13	11:21	14	1	0:14:01	RBNU	M	1
13	11:18	11	44	0:11:44	RBNU	M	1
13	11:22	15	47	0:15:47	RBNU	M	1
13	11:23	16	0	0:16:00	RBNU	M	1
13	11:23	16	56	0:16:56	RBNU	M	1
13	11:24	17	50	0:17:50	RBNU	M	1
13	11:24	17	51	0:17:51	RBNU	M	1
5	11:08	0	25	0:00:25	RBNU	M	1

5	11:11	3	39	0:03:39	RBNU	M	1
1	11:13	6	50	0:06:50	RBNU	M	1
5	11:08	0	0	0:00:00	START		
1	11:12	5	30	0:05:30	START		
13	11:18	11	0	0:11:00	START		
5	7:35	15	5	0:15:05	MOCH	M	2
5	7:36	16	17	0:16:17	MOCH	U	2
5	7:36	16	17	0:16:17	MOCH	U	2
5	7:36	16	32	0:16:32	MOCH	M	2
5	7:34	14	37	0:14:37	MOCH	U	2
5	7:34	14	1	0:14:01	MOCH	U	1
13	7:25	5	37	0:05:37	MOCH	M	1
13	7:24	4	1	0:04:01	RBNU	M	1
13	7:22	2	34	0:02:34	RBNU	M	1
13	7:21	1	49	0:01:49	RBNU	M	1
13	7:22	2	7	0:02:07	RBNU	M	1
13	7:23	3	4	0:03:04	RBNU	M	1
13	7:20	0	46	0:00:46	RBNU	M	1
13	7:21	1	30	0:01:30	RBNU	M	1
13	7:24	4	10	0:04:10	RBNU	M	1
13	7:25	5	2	0:05:02	RBNU	M	1
13	7:26	6	0	0:06:00	RBNU	M	1
5	7:35	15	43	0:15:43	RBNU	M	1
13	7:20	0	0	0:00:00	START		
1	7:27	7	30	0:07:30	START		
5	7:33	13	0	0:13:00	START		
5	9:20	13	27	0:13:27	MOCH	U	1
13	9:17	10	1	0:10:02	RBNU	M	1
1	9:07	0	0	0:00:00	START		
13	9:12	5	39	0:05:39	START		
5	9:19	12	0	0:12:00	START		
5	9:03	8	54	0:08:54	MOCH	M	1
5	9:04	9	16	0:09:16	MOCH	M	1
5	9:04	9	57	0:09:57	MOCH	M	1
5	9:05	10	51	0:10:51	MOCH	M	1
5	9:07	12	7	0:12:07	MOCH	M	1
5	9:08	13	3	0:13:03	MOCH	M	1
1	9:10	15	18	0:15:18	MOCH	M	1
1	9:10	15	37	0:15:37	MOCH	M	1
13	8:56	1	30	0:01:30	MOCH	U	1
13	8:57	2	2	0:02:02	RBNU	M	1
13	8:57	2	26	0:02:26	RBNU	M	1
13	8:58	3	16	0:03:16	RBNU	M	1
13	9:00	5	5	0:05:05	RBNU	M	1

5	9:07	12	12	0:12:12	RBNU	M	1
5	9:08	13	3	0:13:03	RBNU	M	1
1	9:11	16	48	0:16:48	RBNU	M	1
13	9:01	6	2	0:06:02	RBNU	M	1
13	8:55	0	0	0:00:00	START		
5	9:03	8	30	0:08:30	START		
1	9:10	15	0	0:15:00	START		
13	8:15	0	0	0:00:00	START		
1	8:20	5	0	0:05:00	START		
5	8:25	10	0	0:10:00	START		
5		12	40	0:12:40	MOCH		1
5		11	0	0:11:00	MOCH		1
5		13	35	0:13:35	MOCH		1
3		19	30	0:19:30	MOCH		1
11		6	20	0:06:20	RBNU		1
11		3	50	0:03:50	RBNU		1
11		8	54	0:08:54	RBNU		2
11		4	0	0:04:00	RBNU		1
11		2	15	0:02:15	start		
5		9	58	0:09:58	start		
3		18	4	0:18:04	start		
3	11:06	23	30	0:23:30	stop		
	10:46	0	0	0:00:00			
11	9:50	9	50	0:09:50	BCCH		2
11	9:54	13	21	0:13:21	BCCH		1
3	9:44	3	57	0:03:57	BCCH	M	1
3	9:42	1	51	0:01:51	BCCH		1
3	9:47	6	51	0:06:51	MOCH		1
11	9:53	12	32	0:12:32	MOCH	M	1
11	9:52	11	43	0:11:43	MOCH	M	1
5	10:00	19	25	0:19:25	MOCH		1
5	10:00	19	52	0:19:52	MOCH		1
5	10:02	21	1	0:21:01	MOCH		1
3	9:44	3	30	0:03:30	RBNU		1
3	9:41	0	55	0:00:55	start		
11	9:49	8	30	0:08:30	start		
5	9:58	17	10	0:17:10	start		
5	10:04	23	45	0:23:45	stop		
	9:41	0	0	0:00:00			
11	11:31	3	49	0:03:49	RBNU		1
11	11:32	4	52	0:04:52	RBNU		1
11	11:31	3	36	0:03:36	RBNU		1
11	11:33	5	49	0:05:49	RBNU		1
3	11:37	9	40	0:09:40	RBNU		1

5	11:44	16	23	0:16:23	RBNU		1
11	11:30	2	41	0:02:41	RBNU		1
5	11:43	15	48	0:15:48	RBNU		1
11	11:29	1	42	0:01:42	start		
3	11:36	8	57	0:08:57	start		
5	11:42	14	33	0:14:33	start		
5	11:46	18	54	0:18:54	stop		
	11:28	0	0	0:00:00			
11	10:13	20	26	0:20:26	RBNU	M	1
11	10:13	20	10	0:20:10	RBNU	M	1
11	10:12	19	40	0:19:40	RBNU	M	1
11	10:11	18	54	0:18:54	RBNU	M	1
11	10:12	19	30	0:19:30	RBNU	M	1
11	10:13	20	54	0:20:54	RBNU	M	1
11	10:16	23	20	0:23:20	RBNU	M	1
5	9:56	3	15	0:03:15	RBNU		1
5	9:55	2	46	0:02:46	start		
3	10:03	10	50	0:10:50	start		
11	10:11	18	35	0:18:35	start		
11	10:18	25	0	0:25:00	stop		
	9:53	0	0	0:00:00			
3	10:14	30	45	0:30:45	MOCH		1
3	10:14	30	13	0:30:13	MOCH		1
11	10:03	19	12	0:19:12	MOCH		1
3	10:11	27	6	0:27:06	MOCH	M	1
5	9:56	12	0	0:12:00	MOCH	M	2
5	9:56	12	42	0:12:42	MOCH	M	2
5	9:56	12	57	0:12:57	MOCH	M	2
5	9:57	13	29	0:13:29	MOCH	M	2
5	9:57	13	29	0:13:29	MOCH		2
5	9:57	13	50	0:13:50	MOCH		2
5	9:58	14	42	0:14:42	MOCH		2
5	9:59	15	15	0:15:15	MOCH	M	2
5	9:59	15	32	0:15:32	MOCH	M	2
5	9:59	15	56	0:15:56	MOCH	M	2
3	10:09	25	9	0:25:09	MOCH	M	1
3	10:09	25	23	0:25:23	MOCH	M	1
5	9:55	11	39	0:11:39	MOCH	M	2
5	9:55	11	47	0:11:47	MOCH	M	2
11	10:03	19	55	0:19:55	MOCH		1
3	10:08	24	33	0:24:33	MOCH		2
5	9:54	10	58	0:10:58	MOCH	M	1
5	9:55	11	22	0:11:22	MOCH		2
5	9:58	14	9	0:14:09	MOCH		2

3	10:08	24	33	0:24:51	MOCH		2
3	10:11	27	50	0:27:50	MOCH		2
5	9:56	12	30	0:12:30	MOCH		2
11	10:05	21	23	0:21:23	MOCH		1
3	10:10	26	26	0:26:26	MOCH	M	1
5	9:57	13	17	0:13:17	MOCH	M	2
5	9:58	14	20	0:14:20	MOCH	M	2
11	10:02	18	48	0:18:48	MOCH	M	1
3	10:12	28	31	0:28:31	MOCH	M	1
11	10:01	17	59	0:17:59	RBNU		2
11	10:03	19	12	0:19:12	RBNU		1
3	10:08	24	51	0:24:51	RBNU		1
3	10:11	27	27	0:27:27	RBNU		2
5	10:01	17	11	0:17:11	RBNU		1
11	10:01	17	48	0:17:48	RBNU	M	2
3	10:10	26	8	0:26:08	RBNU		1
3	10:11	27	27	0:27:27	RBNU		2
11	10:01	17	48	0:17:48	RBNU		2
11	10:03	19	36	0:19:36	RBNU		1
5	9:57	13	7	0:13:07	RBNU		1
3	10:13	29	26	0:29:26	RBNU		1
5	9:54	10	20	0:10:20	start		
11	10:01	17	22	0:17:22	start		
3	10:08	24	4	0:24:04	start		
3	10:15	31	33	0:31:33	stop		
	9:44	0	0	0:00:00			
3	12:07	14	32	0:14:32	BCCH	M	1
3	12:05	12	5	0:12:05	BCCH	M	1
3	12:01	8	55	0:08:55	BCCH	M	1
3	12:02	9	26	0:09:26	BCCH	M	1
3	12:04	11	49	0:11:49	BCCH	M	1
3	12:09	16	32	0:16:32	BCCH	M	1
3	12:02	9	15	0:09:15	BCCH	M	1
3	12:03	10	13	0:10:13	BCCH	M	1
3	12:03	10	53	0:10:53	BCCH	M	1
3	12:04	11	29	0:11:29	BCCH	M	1
3	12:06	13	29	0:13:29	BCCH	M	1
3	12:07	14	6	0:14:06	BCCH	M	1
3	12:10	17	28	0:17:28	BCCH	M	1
3	12:10	17	54	0:17:54	BCCH	M	1
5	12:16	23	5	0:23:05	BCCH	M	1
3	12:01	8	29	0:08:29	BCCH	M	1
11	11:55	2	28	0:02:28	MOCH		1
3	12:09	16	47	0:16:47	MOCH	M	1

3	12:13	20	23	0:20:23	MOCH	M	1
5	12:16	23	13	0:23:13	MOCH	M	1
11	11:56	3	35	0:03:35	RBNU		1
11	11:57	4	35	0:04:35	RBNU		2
3	12:03	10	40	0:10:40	RBNU		1
3	12:04	11	7	0:11:07	RBNU		1
3	12:06	13	0	0:13:00	RBNU		1
11	11:57	4	35	0:04:35	RBNU		2
11	11:54	1	54	0:01:54	start		
3	12:01	8	8	0:08:08	start		
5	12:13	20	25	0:20:25	start		
5	12:21	28	4	0:28:04	stop		
	11:53	0	0	0:00:00			
3	9:26	12	12	0:12:12	BCCH		1
3	9:27	13	5	0:13:05	BCCH		1
3	9:26	12	44	0:12:44	BCCH		1
3	9:26	12	53	0:12:53	BCCH		1
3	9:27	13	34	0:13:34	BCCH		1
3	9:27	13	58	0:13:58	MOCH		1
11	9:33	19	30	0:19:30	RBNU		2
11	9:33	19	16	0:19:16	RBNU		1
11	9:32	18	50	0:18:50	RBNU		1
11	9:33	19	41	0:19:41	RBNU		2
11	9:33	19	52	0:19:52	RBNU		2
11	9:34	20	10	0:20:10	RBNU		2
11	9:34	20	15	0:20:15	RBNU		2
11	9:31	17	30	0:17:30	RBNU		1
5	9:19	5	36	0:05:36	start		
3	9:24	10	55	0:10:55	start		
11	9:31	17	29	0:17:29	start		
11	9:37	23	16	0:23:16	stop		
	9:14	0	0	0:00:00			
5	8:11	5	44	0:05:44	MOCH	M	1
5	8:12	6	34	0:06:34	MOCH	M	1
5	8:12	6	25	0:06:25	MOCH	M	1
5	8:12	6	47	0:06:47	MOCH	M	1
5	8:12	6	59	0:06:59	MOCH	M	1
5	8:12	6	10	0:06:10	MOCH	M	1
5	8:10	4	4	0:04:04	MOCH	M	1
5	8:10	4	30	0:04:30	MOCH	M	1
5	8:13	7	17	0:07:17	MOCH	M	1
5	8:17	11	44	0:11:44	MOCH	M	1
5	8:18	12	11	0:12:11	MOCH	M	1
5	8:14	8	29	0:08:29	MOCH	M	1

3	8:32	26	45	0:26:45	MOCH		1
11	8:25	19	26	0:19:26	RBNU		1
11	8:25	19	3	0:19:03	RBNU		1
11	8:24	18	21	0:18:21	RBNU		1
11	8:25	19	13	0:19:13	RBNU		1
11	8:26	20	20	0:20:20	RBNU		1
11	8:22	16	57	0:16:57	RBNU		1
11	8:23	17	9	0:17:09	RBNU		1
11	8:23	17	30	0:17:30	RBNU		1
11	8:24	18	32	0:18:32	RBNU		1
11	8:24	18	0	0:18:00	RBNU		1
11	8:23	17	16	0:17:16	RBNU		1
11	8:23	17	51	0:17:51	RBNU		1
11	8:26	20	35	0:20:35	RBNU		1
11	8:24	18	45	0:18:45	RBNU		1
11	8:26	20	0	0:20:00	RBNU		1
11	8:26	20	52	0:20:52	RBNU		1
11	8:27	21	35	0:21:35	RBNU		1
11	8:28	22	30	0:22:30	RBNU		1
11	8:29	23	4	0:23:04	RBNU		1
11	8:29	23	24	0:23:24	RBNU		1
11	8:22	16	29	0:16:29	RBNU		1
11	8:22	16	44	0:16:44	RBNU		1
3	8:30	24	30	0:24:30	RBNU		1
3	8:34	28	42	0:28:42	RBNU		1
3	8:31	25	11	0:25:11	RBNU		1
5	8:14	8	38	0:08:38	RBNU		1
3	8:32	26	52	0:26:52	RBNU		1
5	8:16	10	49	0:10:49	RBNU		1
5	8:09	3	44	0:03:44	start		
11	8:21	15	35	0:15:35	start		
3	8:30	24	14	0:24:14	start		
3	8:35	29	30	0:29:30	stop		
	8:06	0	0	0:00:00			
	10:00	0	0	0:00:00	BCCH	M	1
3	10:07	7	46	0:07:46	BCCH		1
11	10:16	16	53	0:16:53	BCCH	M	1
11	10:17	17	5	0:17:05	BCCH	M	2
11	10:13	13	46	0:13:46	BCCH	M	1
5	10:03	3	16	0:03:16	BCCH	M	1
3	10:12	12	37	0:12:37	BCCH	M	1
11	10:17	17	33	0:17:33	BCCH		2
3	10:06	6	48	0:06:48	BCCH		1
3	10:09	9	5	0:09:05	MOCH		1

3	10:09	9	0	0:09:00	MOCH		1
3	10:09	9	16	0:09:16	MOCH		1
3	10:09	9	27	0:09:27	MOCH		1
3	10:08	8	51	0:08:51	MOCH		1
3	10:06	6	24	0:06:24	MOCH		1
3	10:08	8	38	0:08:38	MOCH		1
3	10:09	9	48	0:09:48	MOCH		1
5	10:05	5	58	0:05:58	MOCH		1
3	10:10	10	28	0:10:28	MOCH		1
5	10:03	3	38	0:03:38	MOCH		1
5	10:01	1	29	0:01:29	MOCH		1
5	10:02	2	57	0:02:57	MOCH		1
3	10:06	6	38	0:06:38	MOCH		1
3	10:08	8	19	0:08:19	MOCH		1
3	10:11	11	11	0:11:11	MOCH		1
3	10:07	7	10	0:07:10	MOCH		1
5	10:00	0	13	0:00:13	start		
3	10:06	6	0	0:06:00	start		
11	10:12	12	38	0:12:38	start		
11	10:18	18	0	0:18:00	stop		
5	10:04	4	28	0:04:28	YRWA		1
	10:00	0	0	0:00:00			
5	8:52	12	16	0:12:16	BCCH		1
3	8:47	7	18	0:07:18	BCCH		1
5	8:50	10	40	0:10:40	MOCH	M	1
5	8:52	12	58	0:12:58	MOCH	M	1
11	8:54	14	22	0:14:22	MOCH	M	1
11	8:55	15	30	0:15:30	RBNU		1
11	8:55	15	50	0:15:50	RBNU		1
11	8:56	16	20	0:16:20	RBNU		1
11	8:56	16	24	0:16:24	RBNU		1
3	8:46	6	52	0:06:52	RBNU		1
5	8:52	12	33	0:12:33	RBNU		1
3	8:41	1	1	0:01:01	start		
5	8:48	8	9	0:08:09	start		
11	8:54	14	10	0:14:10	start		
11	8:59	19	5	0:19:05	stop		
	8:40	0	0	0:00:00			
3	9:20	16	30	0:16:30	BCCH	M	1
3	9:21	17	0	0:17:00	BCCH	M	1
3	9:23	19	15	0:19:15	BCCH	M	1
3	9:23	19	41	0:19:41	BCCH	M	1
3	9:21	17	44	0:17:44	BCCH	M	1
3	9:20	16	7	0:16:07	BCCH	M	1

3	9:24	20	0	0:20:00	BCCH	M	1
3	9:19	15	30	0:15:30	CAVI	M	1
5	9:09	5	41	0:05:41	MOCH		1
3	9:21	17	0	0:17:00	MOCH		1
5	9:08	4	57	0:04:57	MOCH		1
3	9:21	17	21	0:17:21	MOCH		1
11	9:12	8	6	0:08:06	RBNU		1
11	9:11	7	52	0:07:52	RBNU		1
11	9:12	8	25	0:08:25	RBNU	M	2
11	9:12	8	25	0:08:25	RBNU		2
11	9:11	7	44	0:07:44	RBNU		1
11	9:12	8	55	0:08:55	RBNU	M	2
11	9:12	8	55	0:08:55	RBNU		2
11	9:13	9	11	0:09:11	RBNU	M	2
11	9:13	9	11	0:09:11	RBNU		2
11	9:13	9	27	0:09:27	RBNU		2
11	9:13	9	27	0:09:27	RBNU	M	2
11	9:14	10	24	0:10:24	RBNU		2
11	9:14	10	57	0:10:57	RBNU		3
11	9:16	12	25	0:12:25	RBNU	M	2
11	9:17	13	14	0:13:14	RBNU		2
11	9:15	11	27	0:11:27	RBNU		2
3	9:18	14	48	0:14:48	RBNU		1
11	9:14	10	34	0:10:34	RBNU		3
11	9:16	12	25	0:12:25	RBNU		2
3	9:24	20	20	0:20:20	RNSA		1
5	9:05	1	46	0:01:46	start		
11	9:11	7	1	0:07:01	start		
3	9:17	13	59	0:13:59	start		
3	9:25	21	54	0:21:54	stop		
	9:04	0	0	0:00:00			
3	11:06	24	4	0:24:04	BCCH		1
5	11:01	19	16	0:19:16	DOWO		1
5	10:58	16	21	0:16:21	MOCH	M	1
3	11:04	22	44	0:22:44	MOCH	M	1
5	10:56	14	11	0:14:11	MOCH	M	1
5	10:57	15	28	0:15:28	MOCH	M	1
5	10:55	13	6	0:13:06	MOCH	M	1
5	10:55	13	38	0:13:38	MOCH	M	1
5	10:57	15	34	0:15:34	MOCH	M	1
5	10:54	12	34	0:12:34	MOCH	M	1
5	10:54	12	0	0:12:00	MOCH		1
5	10:57	15	2	0:15:02	MOCH	F	1
13	10:48	6	0	0:06:00	MOCH	M	1

3	11:04	22	25	0:22:25	MOCH	M	1
3	11:06	24	4	0:24:04	MOCH	M	1
3	11:06	24	49	0:24:49	MOCH	M	1
13	10:45	3	40	0:03:40	RBNU	M	1
13	10:45	3	59	0:03:59	RBNU	M	1
13	10:44	2	33	0:02:33	RBNU	M	1
13	10:49	7	18	0:07:18	RBNU	M	1
13	10:44	2	11	0:02:11	RBNU	M	1
13	10:47	5	7	0:05:07	RBNU	M	1
13	10:50	8	57	0:08:57	RBNU	M	1
5	10:57	15	36	0:15:36	RBNU	M	1
5	10:54	12	37	0:12:37	RBNU	M	1
3	11:07	25	0	0:25:00	RBNU		1
13	10:48	6	13	0:06:13	RBNU	M	1
13	10:47	5	41	0:05:41	RBNU	M	1
5	10:57	15	16	0:15:16	RBNU	M	1
13	10:42	0	0	0:00:00	start		
5	10:54	12	0	0:12:00	start		
3	11:03	21	30	0:21:30	start		
3	11:07	25	0	0:25:00	stop		
13	10:43	1	21	0:01:21	TRES	F	1
13	10:51	9	28	0:09:28	TRES		1
	10:42	0	0	0:00:00			
1	9:14	1	14	0:01:14	BCCH	M	1
1	9:17	4	2	0:04:02	BCCH	M	1
1	9:14	1	20	0:01:20	CHIC		1
5	9:22	9	39	0:09:39	MOCH	M	1
5	9:22	9	39	0:09:39	MOCH	F	1
5	9:23	10	15	0:10:15	MOCH	M	1
5	9:23	10	15	0:10:15	MOCH	M	1
1	9:14	1	45	0:01:45	MOCH	M	1
5	9:21	8	52	0:08:52	MOCH	M	1
5	9:21	8	26	0:08:26	MOCH	M	1
5	9:23	10	52	0:10:52	MOCH	M	1
5	9:23	10	52	0:10:52	MOCH	F	1
1	9:13	0	30	0:00:30	MOCH	M	1
1	9:16	3	34	0:03:34	MOCH	M	1
11	9:33	20	6	0:20:06	MOCH	M	1
11	9:34	21	41	0:21:41	MOCH	M	1
1	9:15	2	34	0:02:34	MOCH	M	1
1	9:18	5	48	0:05:48	MOCH	M	1
1	9:19	6	37	0:06:37	MOCH	M	1
11	9:31	18	9	0:18:09	MOCH	M	1
11	9:32	19	47	0:19:47	MOCH	M	1

1	9:13	0	0	0:00:00	MOCH		
5	9:25	12	46	0:12:46	MOCH		1
1	9:14	1	59	0:01:59	RNSA	M	1
11	9:30	17	34	0:17:34	RNSA		1
11	9:28	15	8	0:15:08	RNSA		
1	9:14	1	59	0:01:59	RNSA	M	1
5	9:21	8	12	0:08:12	RNSA		1
1	9:13	0	0	0:00:00	start		
5	9:20	7	30	0:07:30	start		
11	9:28	15	0	0:15:00	start		
11	9:34	21	41	0:21:41	stop		
5	9:20	7	50	0:07:50	TRES		1
	9:13	0	0	0:00:00			
	8:25:15			10:15	BCCH	U	1
	8:26:30			11:30	BCCH	M	2
	8:26:30			11:30	BCCH	F	2
	8:30:40			15:40	BCCH	U	1
	8:31:30			16:30	BCCH	U	2
	8:39:20			24:20:00	BCCH	U	2
	8:43:19			28:19:00	BCCH	U	2
	8:27:30			12:30	BCCH	U	2
	8:29:10			14:10	BCCH	U	1
	8:39:40			24:40:00	BCCH	U	2
	8:42:20			27:20:00	BCCH	U	2
	8:26:05			11:05	BCCH	M	1
	8:23:30			8:30	BCCH	U	1
	8:23:40			8:40	BCCH	M	1
	8:25:55			10:55	BCCH	U	1
	8:27:30			12:30	BCCH	U	2
	8:33:00			18:00	BCCH	U	2
	8:39:00			24:00:00	BCCH	M	1
	8:40:20			25:20:00	BCCH	U	2
	8:25:40			10:40	BCCH	U	1
	8:21:00			6:00	MOBL	M	1
	8:34:50			19:50	RNSA	U	1
	8:50:40	15	40	15:40	BCCH	U	2
	8:46:20	11	20	11:20	BCCH	U	2
	8:48:00	13	0	13:00	BCCH	U	2
	8:45:00	10	0	10:00	BCCH	U	2
	8:45:00	10	0	10:00	BCCH	U	2
	8:52:40	17	40	17:40	BCCH	U	2
	9:02:30	27	30	27:30:00	BCCH	U	2
	9:02:50	27	50	27:50:00	BCCH	U	2
	8:35:30	0	30	0:30	BCCH	U	1

8:49:30	14	30	14:30	BCCH	U	2
8:38:00	3	0	3:00	BCCH	U	1
8:41:00	6	0	6:00	BCCH	U	2
8:41:00	6	0	6:00	BCCH	U	2
8:49:00	14	0	14:00	BCCH	U	2
8:51:20	16	20	16:20	BCCH	U	2
8:52:15	17	15	17:15	BCCH	U	2
8:56:30	21	30	21:30	BCCH	U	2
9:05:30	30	30	30:30:00	BCCH	U	2
9:05:30	30	30	30:30:00	BCCH	U	2
8:44:10	9	10	9:10	BCCH	U	2
8:44:10	9	10	9:10	BCCH	U	2
8:50:00	15	0	15:00	BCCH	U	2
9:03:05	28	5	28:05:00	BCCH	U	2
9:03:30	28	30	28:30:00	BCCH	U	2
9:03:50	28	50	28:50:00	BCCH	U	2
8:46:30	11	30	11:30	TAHU	U	1
8:39:30	4	30	4:30	UID	U	1
9:44:00				BCCH	F	1
9:59:50				BCCH	F	1
10:05:00				BCCH	F	2
10:05:25				BCCH	M	2
10:04:00				BCCH	F	2
10:04:00				BCCH	M	2
9:52:00				BCCH	U	2
9:52:50				BCCH	U	2
9:59:00				BCCH	F	1
10:05:10				BCCH	U	2
9:55:50				BCCH	M	1
10:07:45				BCCH	U	2
10:08:00				BCCH	U	2
9:50:44				BCCH	M	1
10:03:40				RBNU	M	1
10:07:30				RBNU	M	1
9:59:50				RBNU	U	1
9:51:40				RBNU	U	1
11:39:10			15:10	BCCH	M	2
11:34:10			10:10	BCCH	M	2
11:33:30			9:30	BCCH	M	2
11:34:00			10:00	BCCH	M	2
11:38:10			14:10	BCCH	M	2
11:39:00			15:00	BCCH	M	2
11:37:40			13:40	BCCH	M	2
11:38:40			14:40	BCCH	M	2

	11:33:50			9:50	BCCH	M	2
	11:40:20			16:20	BCCH	M	2
	11:33:20			9:20	BCCH	M	2
	9:30:10			8:10	BCCH	U	2
	9:34:20			12:20	BCCH	U	2
	9:35:00			13:00	BCCH	U	2
	9:35:10			13:10	BCCH	U	2
	9:29:30			7:30	BCCH	U	2
	9:28:35			6:35	BCCH	U	2
	9:42:10			20:10	BCCH	U	2
	9:42:17			20:17	BCCH	U	2
	9:22:37			0:37	BCCH	U	2
	9:25:10			3:10	BCCH	B	2
	9:43:50			21:50	RBNU	M	1
	9:43:30			21:30	RBNU	M	1
	9:44:50			22:50	RBNU	M	1
1	9:54	14	5	0:14:05	BCCH	M	1
13	10:06	26	13	0:26:13	BCCH	U	1
1	9:53	13	21	0:13:21	BCCH	M	1
1	9:53	13	51	0:13:51	BCCH	M	1
13	10:07	27	0	0:27:00	BCCH	U	1
1	9:57	17	7	0:17:07	BCCH	M	1
13	10:04	24	53	0:24:53	BCCH	M	1
1	9:52	12	53	0:12:53	BCCH	M	1
5	9:44	4	47	0:04:47	MOCH	M	2
5	9:44	4	50	0:04:50	MOCH	M	2
5	9:45	5	2	0:05:02	MOCH	M	2
5	9:47	7	3	0:07:03	MOCH	M	2
1	9:52	12	38	0:12:38	MOCH	M	1
5	9:46	6	2	0:06:02	MOCH	M	2
5	9:47	7	39	0:07:39	MOCH	M	2
13	10:00	20	45	0:20:45	MOCH	M	1
5	9:44	4	42	0:04:42	MOCH	M	2
1	9:54	14	5	0:14:05	MOCH	M	1
1	9:52	12	19	0:12:19	MOCH	M	1
5	9:42	2	30	0:02:30	MOCH	M	1
5	9:44	4	33	0:04:33	MOCH	M	2
13	10:00	20	21	0:20:21	MOCH	M	1
5	9:43	3	3	0:03:03	MOCH	M	2
5	9:44	4	1	0:04:01	MOCH	M	2
5	9:49	9	29	0:09:29	MOCH	M	1
13	10:07	27	10	0:27:10	MOCH	M	1
5	9:40	0	28	0:00:28	MOCH	M	1
5	9:42	2	39	0:02:39	MOCH	M	2

13	10:02	22	13	0:22:13	RBNU	M	2
13	10:01	21	0	0:21:00	RBNU	M	1
13	10:01	21	37	0:21:37	RBNU	F	2
13	10:01	21	2	0:21:02	RBNU	F	2
13	10:02	22	39	0:22:39	RBNU	M	2
13	10:03	23	3	0:23:03	RBNU	M	2
13	10:04	24	36	0:24:36	RBNU	M	1
1	9:54	14	5	0:14:05	RBNU	M	1
13	10:05	25	47	0:25:47	RBNU	M	1
13	10:07	27	10	0:27:10	RBNU	M	1
5	9:48	8	6	0:08:06	RBNU	M	1
13	10:03	23	3	0:23:03	RBNU	F	2
5	9:40	0	0	0:00:00	START		
1	9:51	11	30	0:11:30	START		
13	9:59	19	30	0:19:30	START		
1	8:43	13	43	0:13:43	BCCH	F	2
1	8:42	12	48	0:12:48	BCCH	F	2
1	8:38	8	22	0:08:22	BCCH	F	2
1	8:36	6	22	0:06:22	BCCH	M	2
1	8:38	8	28	0:08:28	BCCH	M	2
1	8:36	6	18	0:06:18	BCCH	M	2
1	8:36	6	42	0:06:42	BCCH	M	2
1	8:37	7	21	0:07:21	BCCH	M	2
1	8:37	7	40	0:07:40	BCCH	F	2
1	8:39	9	0	0:09:00	BCCH	M	2
1	8:39	9	0	0:09:00	BCCH	F	2
1	8:39	9	33	0:09:33	BCCH	U	2
1	8:40	10	13	0:10:13	BCCH	M	2
1	8:40	10	13	0:10:13	BCCH	F	2
1	8:41	11	39	0:11:39	BCCH	M	2
1	8:36	6	13	0:06:13	BCCH	M	2
1	8:36	6	13	0:06:13	BCCH	F	2
1	8:44	14	2	0:14:02	BCCH	F	2
1	8:45	15	3	0:15:03	BCCH	M	2
5	8:30	0	0	0:00:00	START		
1	8:35	5	0	0:05:00	START		
13	8:46	16	8	0:16:08	START		
13	10:39	12	40	0:12:40	BCCH		1
1	10:45	18	59	0:18:59	BCCH		1
1	10:46	19	16	0:19:16	BCCH		1
1	10:46	19	59	0:19:59	BCCH		1
1	10:47	20	5	0:20:05	BCCH		1
1	10:48	21	40	0:21:40	BCCH		1
13	10:42	15	1	0:15:01	BCCH	M	1

13	10:42	15	1	0:15:01	BCCH	F	1
5	10:30	3	1	0:03:01	MOCH	M	1
5	10:28	1	28	0:01:28	MOCH	M	1
13	10:36	9	29	0:09:29	MOCH	M	1
5	10:29	2	13	0:02:13	MOCH	M	1
5	10:32	5	0	0:05:00	MOCH	M	1
5	10:32	5	46	0:05:46	MOCH	M	1
13	10:40	13	55	0:13:55	MOCH	M	1
5	10:30	3	27	0:03:27	MOCH	M	1
5	10:33	6	12	0:06:12	MOCH		2
13	10:37	10	36	0:10:36	RBNU	M	1
13	10:39	12	38	0:12:38	RBNU	M	1
13	10:37	10	19	0:10:19	RBNU	M	1
13	10:36	9	15	0:09:15	RBNU	M	1
13	10:37	10	50	0:10:50	RBNU	M	1
13	10:41	14	10	0:14:10	RBNU	M	1
13	10:35	8	52	0:08:52	RBNU	M	1
1	10:48	21	34	0:21:34	RBNU	M	1
1	10:49	22	44	0:22:44	RBNU	M	1
13	10:36	9	29	0:09:29	RBNU	M	1
13	10:42	15	1	0:15:01	RBNU	U	1
1	10:45	18	0	0:18:00	START		
5	10:27	0	0	0:00:00	START		
13	10:35	8	3	0:08:03	START		
5	12:44	6	56	0:06:56	MOCH	M	2
5	12:44	6	20	0:06:20	MOCH	M	1
5	12:42	4	10	0:04:10	MOCH	M	1
5	12:42	4	48	0:04:48	MOCH	M	1
5	12:42	4	0	0:04:00	MOCH	M	1
5	12:47	9	40	0:09:40	MOCH	F	2
5	12:47	9	43	0:09:43	MOCH	M	2
5	12:40	2	38	0:02:38	MOCH	M	1
3	12:54	16	10	0:16:10	MOCH	F	2
5	12:44	6	48	0:06:48	MOCH	F	2
11	13:05	27	45	0:27:45	RBNU		1
11	13:01	23	58	0:23:58	RBNU		1
5	12:41	3	39	0:03:39	RBNU		1
3	12:53	15	38	0:15:38	start		
11	13:00	22	45	0:22:45	start		
11	13:08	30	0	0:30:00	stop		
11	13:04	26	15	0:26:15	TRES	F	1
	12:38	0	0	0:00:00			
5	12:38	0	45	0:00:45			
	9:16:20			6:20	MOCH	U	1

9:23:10	13:10	MOCH	U	1
9:27:40	17:40	MOCH	U	1
9:38:30	28:30:00	MOCH	U	1
9:16:00	6:00	MOCH	U	1
9:24:00	14:00	MOCH	U	1
9:11:20	1:20	MOCH	U	1
9:12:40	2:40	MOCH	U	1
10:07:00	17:00	MOCH	U	1
9:52:40	2:40	MOCH	U	2
9:59:40	9:40	MOCH	U	1
10:04:20	14:20	MOCH	U	1
10:05:50	15:50	MOCH	U	1
10:14:50	24:50:00	MOCH	U	2
10:14:50	24:50:00	MOCH	U	2
10:01:40	11:40	MOCH	U	1
10:06:20	16:20	MOCH	U	1
9:59:40	9:40	RBNU	M	1
9:58:00	8:00	RBNU	M	1
9:58:10	8:10	RBNU	M	1
9:57:00	7:00	RBNU	M	1
9:56:15	6:15	RBNU	M	1
9:56:50	6:50	RBNU	M	1
10:00:30	10:30	RBNU	M	1
10:04:50	14:50	RBNU	M	1
9:55:50	5:50	RBNU	M	1
9:56:05	6:05	RBNU	M	1
9:56:25	6:25	RBNU	M	1
10:01:00	11:00	RBNU	M	1
10:01:40	11:40	RBNU	M	1
10:07:30	17:30	RBNU	M	1
10:09:00	19:00	RBNU	M	1
10:06:50	16:50	RBNU	U	1
10:17:15	27:15:00	RBNU	U	1
8:54:00	0:09:00	BCCH	M	2
8:54:00	0:09:00	BCCH	F	2
8:52:30	0:07:30	DOWO	M	1
9:09:30	24:30:00	DOWO	M	2
9:09:30	24:30:00	DOWO	F	2
8:59:00	0:14:00	DOWO	M	1
8:48:30	0:03:30	MOCH	U	2
8:48:30	0:03:30	MOCH	U	2
8:49:50	0:04:50	MOCH	U	2
8:50:50	0:05:50	MOCH	U	2
8:51:20	0:06:20	MOCH	U	2

8:58:20	0:13:20	MOCH	U	1
9:05:50	0:20:50	MOCH	M	2
9:05:50	0:20:50	MOCH	F	2
9:08:40	0:23:40	MOCH	U	2
8:48:30	0:03:30	MOCH	U	2
8:49:10	0:04:10	MOCH	U	2
9:08:45	0:23:45	MOCH	U	2
9:22:30		MOCH	U	1
9:28:30		MOCH	U	1
9:47:30		MOCH	U	1
9:50:00		MOCH	U	1
9:47:45		MOCH	U	1
9:12:20	10:20	MOCH	F	
9:14:55	12:55	MOCH	M	
9:18:00	16:00	MOCH	F	
9:27:00	25:00:00	MOCH	M	
9:14:45	12:45	MOCH	M	
9:22:15	20:15	MOCH	M	
9:22:50	20:50	MOCH	M	
9:07:59	5:59	MOCH	U	
9:09:00	7:00	MOCH	F	
9:09:40	7:40	MOCH	F	
9:14:01	12:01	MOCH	M	
9:07:01	5:01	MOCH	M	
9:21:30	19:30	MOCH	M	
9:14:00	12:00	MOCH	B	
9:23:50	21:50	MOCH	F	
9:06:59	4:59	MOCH	M	
9:11:00	9:00	MOCH	B	
8:18:30	6:30	MOCH	M	
8:19:30	7:30	MOCH	M	2
8:19:30	7:30	MOCH	F	2
8:20:00	8:00	MOCH	F	2
8:18:30	6:30	MOCH	F	2
8:18:10	6:10	MOCH	M	
8:16:50	4:50	MOCH	M	
8:17:30	5:30	MOCH	M	
8:13:00	1:00	MOCH	M	
8:38:40	26:40:00	MOCH	M	
8:20:40	8:40	MOCH	B	2
8:27:00	15:00	RBNU	U	2
8:29:30	17:30	RBNU	U	2
8:30:00	18:00	RBNU	U	2
8:18:20	6:20	RBNU	U	

8:22:00	10:00	RBNU	U		1
8:14:38	2:38	RBNU	U		
10:43:38	14:38	MOCH	M		2
10:49:01	20:01	MOCH	M		2
10:49:10	20:10	MOCH	M		2
10:41:31	12:31	MOCH	M		2
10:41:31	12:31	MOCH	F	SY	2
10:42:20	13:20	MOCH	M		2
10:30:20	1:20	MOCH	F	SY	2
11:30:20	1:20	MOCH	M		2
10:38:25	9:25	MOCH	U		1
8:56:20	6:20	MOCH	U		2
9:02:10	12:10	MOCH	M		2
9:07:40	17:40	MOCH	M		2
8:56:30	6:30	MOCH	F		2
9:02:45	12:45	MOCH	F		2
8:53:00	3:00	MOCH	F		2
8:54:00	4:00	MOCH	M		2
8:51:02	1:02	MOCH	M		2
8:52:40	2:40	MOCH	M		2
10:26:50	8:50	MOCH	M		2
10:27:00	9:00	MOCH	M		2
10:26:20	8:20	MOCH	F		2
10:26:20	8:20	MOCH	M		2
10:27:30	9:30	MOCH	M		2
10:19:00	1:00	MOCH	U		2
10:25:30	7:30	MOCH	F		2
10:25:30	7:30	MOCH	M		2
10:32:15	14:15	MOCH	F		2
10:36:13	18:13	MOCH	F		2
10:38:00	20:00	MOCH	F		3
10:19:00	1:00	MOCH	U		2
10:38:00	20:00	MOCH	M		3
10:21:40	3:40	MOCH	M		2
10:35:27	17:27	MOCH	M		2
10:22:45	4:45	MOCH	M		2
10:35:27	17:27	MOCH	M		3
10:30:45	12:45	MOCH	M		2
11:17:00	22:00	BCCH	M		1
11:15:00	20:00	BCCH	M		1
10:58:00	3:00	BCCH	M		1
11:10:30	15:30	BCCH	M		1
11:03:45	8:45	BCCH	M		1
11:02:00	7:00	MOCH	M		2

11:16:00	21:00	MOCH	M	2
11:15:55	20:55	MOCH	M	2
11:08:40	13:40	MOCH	F	2
11:09:50	14:50	MOCH	F	2
11:15:50	20:50	MOCH	F	2
11:16:15	21:15	MOCH	F	2
11:16:30	21:30	MOCH	B	2
11:16:35	21:35	MOCH	F	2
11:01:50	6:50	MOCH	B	2
11:03:40	8:40	MOCH	M	2
11:15:45	20:45	MOCH	B	2
11:07:00	12:00	MOCH	M	2
11:21:10	26:10:00	MOCH	M	2
11:17:00	22:00	MOCH	M	2
7:55:00	8:00	MOCH	M	2
7:53:58	6:58	MOCH	M	2
7:51:00	4:00	MOCH	F	2
7:51:00	4:00	MOCH	M	2
7:53:00	6:00	MOCH	M	2
7:53:20	6:20	MOCH	M	2
7:56:50	9:50	MOCH	M	2
8:01:00	14:00	MOCH	F	2
8:03:40	16:40	MOCH	F	1
8:05:10	18:10	MOCH	M	2
8:07:28	20:28	MOCH	M	2
8:07:28	20:28	MOCH	F	2
8:08:00	21:00	MOCH	F	2
8:09:20	22:20	MOCH	F	2
8:12:20	25:20:00	MOCH	M	2
8:01:20	14:20	MOCH	M	2
8:08:40	21:40	MOCH	M	2
8:01:40	14:40	MOCH	M	2
8:02:00	15:00	MOCH	F	2
8:03:20	16:20	MOCH	B	2
8:15:50	16:50	DOWO	M	1
8:26:20	27:20:00	DOWO	M	1
8:26:00	27:00:00	MOCH	M	2
8:25:20	26:20:00	MOCH	M	2
8:24:40	25:40:00	MOCH	M	2
7:59:00	0:00	MOCH	F	1
8:12:20	13:20	MOCH	M	2
8:15:20	16:20	MOCH	M	2
8:15:25	16:25	MOCH	F	2
8:25:28	26:28:00	MOCH	F	2

8:09:40	10:40	MOCH	M	2
8:30:00	31:00:00	MOCH	M	2
8:08:00	9:00	MOCH	M	2
8:15:30	16:30	MOCH	B	2
8:26:20	27:20:00	MOCH	B	2
11:49:40	19:40	MOCH	B	2
11:51:00	21:00	MOCH	F	2
11:50:40	20:40	MOCH	M	2
11:50:28	20:28	MOCH	M	2
11:52:00	22:00	MOCH	M	2
11:48:40	18:40	MOCH	U	2
11:53:00	23:00	MOCH	F	2
11:53:10	23:10	MOCH	B	2
12:22:00	9:00	MOCH	F	1
12:29:00	16:00	MOCH	M	2
12:33:20	20:20	MOCH	M	2
12:35:40	22:40	MOCH	M	2
12:28:30	15:30	MOCH	M	2
12:37:00	24:00:00	MOCH	M	3
12:37:00	24:00:00	MOCH	M	3
12:24:40	11:40	MOCH	M	2
12:19:50	6:50	RBNU	U	1
12:15:00	2:00	RBNU	U	1
8:42:20	12:20	MOCH	M	2
8:42:26	12:26	MOCH	M	2
8:35:20	5:20	MOCH	F	2
8:43:45	13:45	MOCH	F	2
8:53:20	23:20	MOCH	F	2
8:35:20	5:20	MOCH	M	2
8:35:50	5:50	MOCH	M	2
8:54:00	24:00:00	MOCH	M	2
8:43:00	13:00	MOCH	M	2
8:36:30	6:30	MOCH	M	2
8:37:45	7:45	MOCH	M	3
8:36:05	6:05	MOCH	M	2
8:54:14	24:14:00	MOCH	M	2
9:21:50	24:50:00	BCCH	M	2
9:05:30	8:30	MOCH	M	2
9:05:44	8:44	MOCH	M	2
9:06:16	9:16	MOCH	M	2
9:07:05	10:05	MOCH	M	2
9:07:37	10:37	MOCH	M	2
9:08:30	11:30	MOCH	M	2
9:10:10	13:10	MOCH	M	2

8:57:00	0:00	MOCH	F	2
9:06:40	9:40	MOCH	F	2
9:17:05	20:05	MOCH	M	2
9:05:25	8:25	MOCH	M	2
9:11:45	14:45	MOCH	M	2
9:12:45	15:45	MOCH	M	2
8:59:30	2:30	MOCH	M	2
9:02:05	5:05	MOCH	F	2
9:02:30	5:30	MOCH	M	2
13:01:20	11:20	BCCH	U	1
12:55:00	5:00	MOCH	M	2
13:01:10	11:10	MOCH	M	2
12:53:30	3:30	MOCH	M	2
12:51:30	1:30	MOCH	M	2
12:56:30	6:30	MOCH	M	2
12:59:00	9:00	MOCH	M	2
13:07:30	17:30	MOCH	M	2
12:59:30	9:30	MOCH	M	2
12:50:00	0:00	MOCH	F	2
12:55:30	5:30	MOCH	F	2
12:59:00	9:00	MOCH	F	2
13:08:20	18:20	MOCH	M	2
12:50:19	0:19	MOCH	M	2
12:52:20	2:20	MOCH	F	2
13:07:20	17:20	MOCH	M	2
13:12:00	22:00	MOCH	M	2
13:03:00	13:00	MOCH	M	2
13:01:30	11:30	MOCH	M	2
13:01:35	11:35	MOCH	M	2
15:33:17	18:17	MOCH	M	2
15:34:00	19:00	MOCH	M	2
15:33:17	18:17	MOCH	M	2
15:34:00	19:00	MOCH	M	2
15:21:30	6:30	RBNU	M	2
15:21:30	6:30	RBNU	M	2
15:18:58	3:58	RBNU	M	2
15:27:18	12:18	RBNU	M	2
15:29:20	14:20	RBNU	M	2
15:18:58	3:58	RBNU	M	2
15:27:18	12:18	RBNU	M	2
15:29:20	14:20	RBNU	M	2
9:38:50	21:50	MOCH	U	1
9:31:49	14:49	MOCH	U	1
11:39:40	14:40	MOCH	M	2

11:45:50	20:50	MOCH	M		2
11:46:40	21:40	MOCH	M		2
11:45:40	20:40	MOCH	F		2
11:46:00	21:00	MOCH	M		2
11:48:45	23:45	MOCH	F		2
11:42:50	17:50	MOCH	M		2
11:42:50	17:50	MOCH	F		2
11:48:00	23:00	MOCH	M		2
11:25:00	0:00	MOCH	M		2
9:51:10	0:10	MOCH	M		2
9:53:50	2:50	MOCH	M		2
9:54:00	3:00	MOCH	F		2
9:54:05	3:05	MOCH	M		2
9:54:35	3:35	MOCH	M		2
11:22:00	17:00	MOCH	M		2
11:21:20	16:20	MOCH	M		2
11:18:30	13:30	MOCH	M		2
11:19:50	14:50	MOCH	M		2
11:20:27	15:27	MOCH	M		2
11:25:10	20:10	MOCH	M		2
11:18:50	13:50	MOCH	M		2
11:06:00	1:00	MOCH	F		2
11:06:00	1:00	MOCH	M		2
11:10:30	5:30	MOCH	M		2
11:18:05	13:05	MOCH	M		2
8:49:50	20:50	MOCH	F		2
8:50:00	21:00	MOCH	F		2
8:51:19	22:19	MOCH	F		2
8:46:18	17:18	MOCH	F		2
8:46:45	17:45	MOCH	M		2
8:49:30	20:30	MOCH	F		2
8:43:30	14:30	MOCH	F		2
8:46:28	17:28	MOCH	M		2
8:53:05	24:05:00	MOCH	M		2
8:54:45	25:45:00	MOCH	M		2
8:53:40	24:40:00	MOCH	M		3
11:01:00	27:00:00	MOCH	M	SY	2
11:00:00	26:00:00	MOCH	M	SY	2
11:00:20	26:20:00	MOCH	M	SY	2
11:02:00	28:00:00	MOCH	M	SY	2
11:05:00	31:00:00	MOCH	M	SY	2
10:47:00	13:00	MOCH	F		1
10:48:00	14:00	MOCH	F		1
11:09:00	35:00:00	MOCH	M	SY	2

10:41:30	7:30	RBNU	M	1
10:39:48	5:48	RBNU	M	1
10:40:20	6:20	RBNU	M	1
10:41:00	7:00	RBNU	M	1
10:47:00	13:00	RBNU	M	1
10:39:20	5:20	RBNU	M	1
10:49:30	15:30	RBNU	M	1
10:49:00	15:00	RBNU	M	2
10:49:00	15:00	RBNU	F	2
10:52:50	18:50	RBNU	M	1
8:05:00	1:00	MOCH	U	2
8:06:50	2:50	MOCH	U	2
8:07:00	3:00	MOCH	U	2
8:07:03	3:03	MOCH	U	2
8:07:40	3:40	MOCH	U	2
8:05:05	1:05	MOCH	U	2
8:06:00	2:00	MOCH	U	2
8:07:10	3:10	MOCH	U	2
8:07:45	3:45	MOCH	U	2
8:13:00	0:00	MOCH	M	2
8:17:25	4:25	MOCH	U	2
8:17:30	4:30	MOCH	U	2
8:17:33	4:33	MOCH	U	2
8:14:00	1:00	MOCH	M	2
8:22:50	0:50	MOCH	U	2
8:23:00	1:00	MOCH	U	2
8:23:50	1:50	MOCH	U	2
8:24:56	2:56	MOCH	U	2
8:25:55	3:55	MOCH	U	2
8:26:30	4:30	MOCH	U	2
8:26:37	4:37	MOCH	U	2
8:26:45	4:45	MOCH	U	2
8:27:10	5:10	MOCH	F	2
8:28:00	6:00	MOCH	M	2
8:22:00	0:00	MOCH	U	2
8:24:30	2:30	MOCH	B	2
8:26:55	4:55	MOCH	M	2
8:24:40	2:40	MOCH	U	2
10:17:30	15:30	MOCH	M	2
10:24:30	22:30	MOCH	F	2
10:24:30	22:30	MOCH	M	2
10:24:40	22:40	MOCH	M	2
10:09:30	7:30	MOCH	M	1
10:14:20	12:20	MOCH	M	1

10:17:00	15:00	MOCH	M		1
10:17:20	15:20	MOCH	M		1
10:18:00	16:00	MOCH	F		2
10:18:00	16:00	MOCH	M		2
10:23:10	21:10	MOCH	F		2
10:23:45	21:45	MOCH	F		2
10:24:00	22:00	MOCH	M		2
10:25:45	23:45	MOCH	F		2
10:17:30	15:30	MOCH	F		2
10:09:55	7:55	MOCH	M		1
10:03:47	1:47	MOCH	M		1
12:19:10	27:10:00	MOCH	M	SY	2
12:22:20	30:20:00	MOCH	M	SY	2
11:58:10	6:10	MOCH	F		1
12:13:30	21:30	MOCH	M	SY	2
12:15:00	23:00	MOCH	M	SY	2
12:01:00	9:00	MOCH	M	SY	2
6:17:35	21:35	MOCH	M		2
5:56:50	0:50	MOCH	F		1
6:01:20	5:20	MOCH	F		1
6:01:40	5:40	MOCH	F		1
6:01:50	5:50	MOCH	F		1
6:05:30	9:30	MOCH	M		2
6:17:00	21:00	MOCH	M		2
6:09:00	13:00	MOCH	M		2
6:12:50	16:50	MOCH	M		2
6:13:00	17:00	MOCH	M		2
6:15:40	19:40	MOCH	M		2
6:17:30	21:30	RBNU	U		1
6:19:10	23:10	RBNU	U		1
5:58:00	2:00	RBNU	U		1
6:18:00	22:00	RBNU	U		1
6:15:20	19:20	RBNU	U		1
8:32:45	9:45	BCCH	M		1
8:32:50	9:50	BCCH	M		1
8:32:00	9:00	BCCH	M		1
8:31:40	8:40	BCCH	M		1
8:42:30	19:30	BCCH	M		1
8:30:30	7:30	MOCH	F		1
8:30:45	7:45	MOCH	F		1
8:40:00	17:00	MOCH	U		1
8:41:50	18:50	MOCH	U		1
9:22:30	59:30:00	MOCH	F		1
9:23:00	0:00	MOCH	F		1

9:39:00	16:00	MOCH	M	2
9:39:14	16:14	MOCH	M	2
9:41:40	18:40	MOCH	F	2
8:35:50	12:50	MOCH	U	1
8:42:20	19:20	MOCH	U	1
8:35:40	12:40	MOCH	U	1
8:31:30	8:30	MOCH	F	1
9:24:20	1:20	MOCH	F	1
9:31:40	8:40	MOCH	F	1
8:32:50	8:50	BCCH	M	1
8:24:09	0:09	MOCH	F	2
8:24:00	0:00	MOCH	M	2
8:24:10	0:10	MOCH	M	2
8:29:20	5:20	MOCH	M	2
8:44:18	20:18	none		1
9:09:00	3:00	MOCH	F	2
9:11:50	5:50	MOCH	F	2
9:12:04	6:04	MOCH	F	2
9:12:04	6:04	MOCH	M	2
9:06:10	0:10	MOCH	F	2
9:07:30	1:30	MOCH	B	2
9:06:00	0:00	MOCH	M	2
9:09:30	3:30	MOCH	M	2
9:10:00	4:00	MOCH	M	2
8:34:15	5:15	MOCH	F	2
8:48:49	19:49	MOCH	F	2
8:50:00	21:00	MOCH	M	2
8:51:30	22:30	MOCH	M	2
8:34:30	5:30	MOCH	M	2
8:43:15	14:15	MOCH	M	2
8:48:30	19:30	MOCH	F	2
8:50:10	21:10	MOCH	M	2
8:36:40	7:40	MOCH	M	2
8:42:00	13:00	MOCH	F	2
8:43:40	14:40	MOCH	M	2
8:43:37	14:37	MOCH	F	2
8:45:20	16:20	MOCH	B	2
8:46:23	17:23	MOCH	B	2
8:41:40	12:40	MOCH	M	2
8:41:55	12:55	MOCH	M	2
8:40:59	11:59	MOCH	M	2
8:53:47	24:47:00	MOCH	M	2
6:58:45	8:45	MOCH	M	2
7:00:45	10:45	MOCH	M	2

7:00:50	10:50	MOCH	M	2
7:01:29	11:29	MOCH	F	2
7:02:00	12:00	MOCH	M	2
7:11:05	21:05	MOCH	M	2
7:12:30	22:30	MOCH	M	2
7:06:55	16:55	MOCH	M	2
7:09:00	19:00	MOCH	M	2
7:03:50	13:50	MOCH	M	2
6:57:09	7:09	MOCH	M	2
7:03:30	13:30	MOCH	M	2
7:13:50	23:50	MOCH	M	2
6:50:00	0:00	MOCH	U	2
7:01:00	11:00	MOCH	F	2
7:04:40	14:40	RBNU	M	2
7:04:20	14:20	RBNU	M	2
7:03:50	13:50	RBNU	M	2
7:03:50	13:50	RBNU	U	2
7:07:15	17:15	RBNU	U	2
8:01:40	21:40	MOCH	U	1
8:01:00	21:00	MOCH	U	1
8:00:30	20:30	MOCH	U	1
7:52:29	12:29	MOCH	U	1
11:14:00	12:00	MOCH	M	2
11:34:00	32:00:00	MOCH	M	2
11:07:42	5:42	MOCH	M	2
11:07:57	5:57	MOCH	M	2
11:13:05	11:05	MOCH	M	2
11:18:50	16:50	MOCH	M	2
11:25:30	23:30	MOCH	M	2
11:32:10	30:10:00	MOCH	M	2
11:33:15	31:15:00	MOCH	F	2
11:07:40	5:40	MOCH	F	1
11:13:50	11:50	MOCH	F	2
11:13:55	11:55	MOCH	M	2
11:25:00	23:00	MOCH	M	2
11:26:30	24:30:00	MOCH	M	2
11:31:20	29:20:00	MOCH	F	2
11:32:01	30:01:00	MOCH	F	2
11:13:00	11:00	MOCH	F	2
11:25:45	23:45	MOCH	M	2
11:25:30	23:30	MOCH	F	2
11:08:00	6:00	MOCH	F	2
11:02:45	0:45	MOCH	U	1
9:22:52	15:52	MOCH	M	2

9:33:16	26:16:00	MOCH	M	2
9:24:53	17:53	MOCH	M	2
9:24:40	17:40	MOCH	M	2
9:32:30	25:30:00	MOCH	M	3
9:35:08	28:08:00	MOCH	M	2
9:09:00	2:00	MOCH	U	2
9:35:27	28:27:00	MOCH	M	2
9:09:30	2:30	MOCH	U	2
9:27:24	20:24	MOCH	M	3
9:28:30	21:30	MOCH	M	3
9:29:30	22:30	MOCH	M	3
9:30:40	23:40	MOCH	M	3
9:26:26	19:26	MOCH	M	3
9:30:40	23:40	MOCH	M	3
9:15:40	8:40	MOCH	M	2
12:31:28	7:28	MOCH	U	2
12:37:10	13:10	MOCH	U	2
12:39:55	15:55	MOCH	U	2
12:30:20	6:20	MOCH	U	2
12:28:13	4:13	MOCH	U	2
12:29:50	5:50	MOCH	U	2
12:30:00	6:00	MOCH	U	2
12:30:26	6:26	MOCH	U	2
12:30:37	6:37	MOCH	U	2
12:30:50	6:50	MOCH	U	2
12:33:00	9:00	MOCH	U	2
12:36:22	12:22	MOCH	U	2
12:39:12	15:12	MOCH	U	2
12:45:55	21:55	MOCH	U	2
12:47:38	23:38	MOCH	U	2
12:37:50	13:50	MOCH	U	2
12:36:45	12:45	MOCH	U	2
12:45:25	21:25	MOCH	U	2
12:26:25	2:25	MOCH	U	2
12:39:26	15:26	MOCH	U	2
12:38:26	14:26	MOCH	U	2
12:45:40	21:40	MOCH	U	2
12:32:55	8:55	MOCH	U	2
12:33:00	9:00	MOCH	U	2
8:45:30	5:30	MOCH	F	1
8:48:20	8:20	MOCH	F	1
8:55:10	15:10	MOCH	F	2
8:55:30	15:30	MOCH	M	2
9:08:50	28:50:00	MOCH	M	2

8:55:00	15:00	MOCH	M	2
8:47:00	7:00	MOCH	F	1
9:00:00	20:00	MOCH	F	2
8:57:00	17:00	MOCH	M	2
8:59:00	19:00	MOCH	M	2
9:01:00	21:00	MOCH	M	2
14:20:34	22:34	MOCH	M	2
14:19:58	21:58	MOCH	M	2
14:12:29	14:29	MOCH	U	2
14:12:44	14:44	MOCH	U	2
14:20:40	22:40	MOCH	M	2
14:19:30	21:30	MOCH	M	2
14:01:25	3:25	MOCH	U	2
14:08:50	10:50	MOCH	U	2
14:13:19	15:19	MOCH	U	2
14:15:41	17:41	MOCH	U	2
14:19:15	21:15	MOCH	M	2
14:21:16	23:16	MOCH	M	2
14:11:40	13:40	MOCH	U	2
14:11:50	13:50	MOCH	U	2
14:15:00	17:00	MOCH	U	2
14:03:37	5:37	MOCH	U	2
14:10:07	12:07	MOCH	U	2
14:02:10	4:10	MOCH	M	2
14:09:50	11:50	MOCH	M	2
14:22:55	24:55:00	RBNU	U	2
9:06:30	2:30	MOCH	U	2
9:12:44	8:44	MOCH	U	2
9:19:29	15:29	MOCH	U	2
9:19:29	15:29	MOCH	U	2
9:19:40	15:40	MOCH	U	2
9:23:20	19:20	MOCH	U	2
9:26:25	22:25	MOCH	U	2
9:28:08	24:08:00	MOCH	U	2
9:16:20	12:20	MOCH	U	2
9:19:12	15:12	MOCH	U	2
9:08:00	4:00	MOCH	U	2
9:08:30	4:30	MOCH	U	2
9:09:30	5:30	MOCH	U	2
9:24:30	20:30	MOCH	U	2
9:13:45	9:45	MOCH	U	2
9:14:11	10:11	MOCH	U	2
9:23:17	19:17	MOCH	U	2
9:26:45	22:45	MOCH	U	2

9:29:00	25:00:00	MOCH	U	2
9:06:35	2:35	MOCH	U	2
9:11:50	7:50	MOCH	U	2
9:24:20	20:20	MOCH	U	2
9:17:00	13:00	RBNU	U	1
9:30:22	26:22:00	RBNU	U	2
9:53:00	14:00	MOCH	U	2
10:01:36	22:36	MOCH	U	2
9:41:23	2:23	MOCH	U	2
10:01:10	22:10	MOCH	U	2
9:52:40	13:40	MOCH	U	2
9:40:20	1:20	MOCH	U	2
9:41:25	2:25	MOCH	U	2
9:43:57	4:57	MOCH	U	2
9:52:20	13:20	MOCH	U	2
9:55:22	16:22	MOCH	U	2
9:56:14	17:14	MOCH	U	2
9:42:25	3:25	RBNU	U	1
9:48:35	9:35	RBNU	U	2
9:54:00	25:00:00	MOCH	U	2
9:52:00	23:00	MOCH	U	2
9:51:00	22:00	MOCH	U	2
9:49:00	20:00	MOCH	U	2
9:48:40	19:40	MOCH	U	2
9:30:14	1:14	MOCH	U	2
9:30:30	1:30	MOCH	U	2
9:32:44	3:44	MOCH	U	2
9:36:50	7:50	MOCH	U	2
9:44:45	15:45	MOCH	U	2
9:46:00	17:00	MOCH	U	2
9:36:30	7:30	MOCH	U	2
9:45:00	16:00	MOCH	U	2
10:12:00	17:00	BCCH	M	1
10:15:00	20:00	BCCH	M	1
10:14:00	19:00	BCCH	M	2
10:10:55	15:55	BCCH	M	1
9:58:55	3:55	MOCH	M	2
9:59:40	4:40	MOCH	M	2
9:59:40	4:40	MOCH	F	2
10:01:00	6:00	MOCH	M	2
10:00:50	5:50	MOCH	F	2
10:04:47	9:47	MOCH	M	2
10:06:13	11:13	MOCH	M	2
10:06:45	11:45	MOCH	F	2

10:06:55	11:55	MOCH	M	2
10:09:22	14:22	MOCH	M	2
10:10:34	15:34	MOCH	M	2
10:11:00	16:00	MOCH	M	2
10:12:46	17:46	MOCH	F	2
10:14:55	19:55	MOCH	U	2
10:16:30	21:30	MOCH	U	1
10:09:34	14:34	MOCH	M	2
10:13:00	18:00	MOCH	M	2
15:50:45	14:45	MOCH	U	2
15:49:45	13:45	MOCH	U	2
15:50:37	14:37	MOCH	U	2
15:49:00	13:00	MOCH	U	2
15:50:30	14:30	MOCH	B	2
15:58:11	22:11	MOCH	M	2
15:36:06	0:06	MOCH	F	2
15:38:03	2:03	MOCH	F	2
15:43:46	7:46	MOCH	U	2
15:44:04	8:04	MOCH	U	2
15:44:17	8:17	MOCH	U	2
15:47:46	11:46	MOCH	U	2
15:52:20	16:20	MOCH	U	2
15:52:43	16:43	MOCH	U	2
15:54:49	18:49	MOCH	M	2
15:58:07	22:07	MOCH	M	2
15:59:29	23:29	MOCH	M	2
15:49:30	13:30	MOCH	U	2
15:50:50	14:50	MOCH	U	2
15:57:42	21:42	MOCH	M	2
15:59:49	23:49	MOCH	M	2
15:54:15	18:15	MOCH	M	2
15:52:25	16:25	MOCH	M	2
16:00:44	24:44:00	MOCH	M	2
15:36:26	0:26	MOCH	M	2
15:52:00	16:00	MOCH	M	2
15:46:28	10:28	MOCH	M	2
10:48:40	15:40	BCCH	M	1
10:46:34	13:34	BCCH	M	1
10:56:00	23:00	BCCH	M	2
10:50:15	17:15	BCCH	M	1
10:45:50	12:50	BCCH	M	1
10:55:10	22:10	MOCH	M	2
10:37:30	4:30	MOCH	F	2
10:39:35	6:35	MOCH	F	2

	10:40:14	7:14	MOCH	F		2
	10:54:40	21:40	MOCH	M		2
	10:38:01	5:01	MOCH	F		2
	10:51:16	18:16	MOCH	M		2
5		0:29:45	MOCH	M		2
13		0:07:00	MOCH	B		2
13		0:09:00	MOCH	U		1
1		0:19:30	MOCH	U		1
1		0:21:00	MOCH	U		1
5		0:27:00	MOCH	F		2
5		0:27:00	MOCH	M		2
5		0:29:00	MOCH	M		2
1		0:17:45	MOCH	U		1
5		0:25:50	MOCH	B		2
1		0:21:30	MOCH	U		2
1		0:03:35	BCCH	M		1
5		0:11:00	MOCH	M		2
5		0:09:40	MOCH	M		2
5		0:12:00	MOCH	M		2
11		0:17:01	MOCH	B		2
1		0:04:00	MOCH	U		1
5		0:06:30	MOCH	M	SY	2
1		0:14:40	MOCH	M	SY	2
11		0:24:30	MOCH	M	SY	2
1		0:15:40	MOCH	M	SY	2
5		0:03:00	MOCH	M	SY	2
5		0:02:00	MOCH	M	SY	2
5		0:06:30	MOCH	F		2
1		0:15:05	MOCH	F		2
1		0:15:28	MOCH	M	SY	2
11		0:23:00	MOCH	M	SY	2
11		0:27:30	MOCH	F		2
1		0:20:20	MOCH	M	SY	2
11		0:21:20	MOCH	M	SY	2
11		0:24:40	MOCH	F		2
5		0:12:50	BCCH	M		1
5		0:12:50	MOCH	M		2
5		0:11:50	MOCH	F		2
5		0:12:35	MOCH	M		2
5		0:11:50	MOCH	M		2
5		0:11:00	MOCH	U		1
5		0:12:55	MOCH	U		3
1		0:23:00	MOCH	M		2
		0:00:00	MOCH	U		1

11	0:03:40	RBNU	M		2
11	0:05:50	RBNU	F		2
11	0:05:20	RBNU	M		2
5	0:11:30	RBNU	M		2
	0:00:50	MOCH	M		2
11	0:06:00	MOCH	F		2
1	0:11:50	MOCH	M		2
1	0:12:30	MOCH	M		2
5	0:19:05	MOCH	F		2
5	0:19:20	MOCH	F		2
11	0:04:30	MOCH	M		2
5	0:21:00	MOCH	M		2
1	0:27:00	BCCH	M		2
1	0:27:00	BCCH	F		2
11	0:23:50	BCCH	M		1
1	0:28:00	BCCH	M		2
5	0:15:50	BCCH	M		1
11	0:22:40	BCCH	M		1
11	0:22:00	BCCH	M		1
5	0:15:10	MOCH	M		2
1	0:25:50	MOCH	M		1
5	0:15:00	START			
11	0:19:30	START			
1	0:24:50	START			
5	0:08:20	MOCH	M		2
11	0:04:10	MOCH	M		2
11	0:05:00	MOCH	M		2
1	0:15:40	MOCH	M		2
1	0:18:45	MOCH	M		2
1	0:14:40	MOCH	M		2
5	0:10:40	MOCH	M		2
5	0:11:00	MOCH	M		2
1	0:15:20	MOCH	M		2
11	0:02:40	START			
5	0:07:50	START			
1	0:14:00	START			
5	0:04:10	MOCH	M		2
5	0:04:00	MOCH	M		2
1	0:10:40	MOCH	M		2
11	0:14:00	MOCH	M		2
5	0:02:45	START			
1	0:09:15	START			
11	0:13:30	START			
5	0:03:11	MOCH	M	ASY	2

5				0:02:50	MOCH	M	ASY	2
1				0:09:15	MOCH	M	ASY	2
5				0:02:40	MOCH	M	ASY	2
5				0:00:35	MOCH	B		2
11				0:17:40	MOCH	M	ASY	2
5				0:00:30	START			
1				0:09:00	START			
11				0:16:00	START			
5				0:01:00	MOCH	U		1
5				0:00:00	START			
1				0:07:30	START			
11				0:13:16	START			
1				0:17:10	MOCH	F		2
5				0:24:00	MOCH	F		2
1				0:17:00	MOCH	M		2
1				0:17:20	MOCH	F		2
5				0:19:45	MOCH	B		2
11				0:02:55	START			
1				0:15:50	START			
5				0:19:40	START			
5				0:07:25	MOCH	M		1
1				0:02:40	MOCH	U		1
1				0:06:40	MOCH	U		1
11				0:17:58	MOCH	U		1
1				0:03:10	MOCH	U		1
1				0:01:45	START			
5				0:07:20	START			
11				0:13:45	START			
1		11	30	0:11:30	BCCH	M		1
1		9	30	0:09:30	BCCH	M		1
5		5	0	0:05:00	MOCH	M		1
5		6	50	0:06:50	MOCH			2
5		4	50	0:04:50	MOCH	M		1
5		4	40	0:04:40	MOCH	M		1
5		7	10	0:07:10	MOCH	M		2
5		7	20	0:07:20	RBNU			1
1		9	8	0:09:08	START			
5		3	0	0:03:00	START			
11		15	50	0:15:50	START			
	10:46	0	0	0:00:00	START			
5		16	0	0:16:00	END			
1		10	50	0:10:50	MOCH			1
5		13	25	0:13:25	MOCH	M		1
5		13	30	0:13:30	MOCH			1

11	2	50	0:02:50	MOCH		1
11	3	30	0:03:30	MOCH		1
11	4	20	0:04:20	MOCH		1
	0	59	0:00:59	MOCH		1
1	6	35	0:06:35	START		
5	11	40	0:11:40	START		
11	2	0	0:02:00	START		
	0	0	0:00:00	START		
1	22	20	0:22:20	END		
1	21	0	0:21:00	MOCH		1
5	3	0	0:03:00	MOCH	M	2
5	7	20	0:07:20	MOCH	M	2
11	10	0	0:10:00	MOCH	F	2
11	11	0	0:11:00	MOCH	F	2
11	14	0	0:14:00	MOCH	F	1
11	14	20	0:14:20	MOCH	M	1
5	2	30	0:02:30	MOCH		1
1	20	30	0:20:30	MOCH	M	1
11	8	32	0:08:32	MOCH	M	2
1	15	55	0:15:55	MOCH	M	1
11	11	20	0:11:20	MOCH	M	1
1	21	20	0:21:20	MOCH		1
1	21	30	0:21:30	MOCH		1
1	22	0	0:22:00	MOCH		1
1	22	10	0:22:10	MOCH		1
5	3	0	0:03:00	MOCH	F	2
5	7	20	0:07:20	MOCH	F	2
11	8	32	0:08:32	MOCH	F	2
1	15	50	0:15:50	START		
5	2	0	0:02:00	START		
11	8	30	0:08:30	START		
	0	0	0:00:00	START		
1	11	5	0:11:05	MOCH	M	1
1	11	10	0:11:10	MOCH	F	1
5	7	0	0:07:00	MOCH	M	1
5	8	0	0:08:00	MOCH	M	1
11	20	1	0:20:01	MOCH	F	1
11	21	40	0:21:40	MOCH	M	1
11	23	0	0:23:00	MOCH	M	1
1	11	0	0:11:00	START		
5	6	0	0:06:00	START		
11	20	0	0:20:00	START		
	0	0	0:00:00	START		
11	25	0	0:25:00			

11	18	0	0:18:00	BCCH	M	1
11	21	0	0:21:00	END		
1	9	40	0:09:40	MOCH	M	1
11	19	20	0:19:20	MOCH	F	1
1	10	30	0:10:30	MOCH	M	1
1	13	40	0:13:40	MOCH	F	1
5	7	50	0:07:50	MOCH		1
11	19	50	0:19:50	MOCH	F	1
	0	10	0:00:10	MOCH		1
1	11	20	0:11:20	MOCH	M	2
11	15	30	0:15:30	MOCH	M	1
1	9	0	0:09:00	MOCH		1
5	3	0	0:03:00	MOCH	M	1
1	8	0	0:08:00	START		
5	2	0	0:02:00	START		
11	14	40	0:14:40	START		
	0	0	0:00:00	START		
5	3	20	0:03:20	MOCH	M	1
5	3	0	0:03:00	MOCH	M	1
1	20	40	0:20:40	MOCH	M	1
1	21	40	0:21:40	MOCH	F	1
5	18	0	0:18:00	MOCH	F	1
5	18	10	0:18:10	MOCH	F	1
5	19	40	0:19:40	MOCH		1
11	25	40	0:25:40	MOCH	F	2
11	25	42	0:25:42	MOCH	M	2
11	26	10	0:26:10	MOCH	M	2
11	26	50	0:26:50	MOCH	F	2
5	2	30	0:02:30	MOCH	M	1
5	1	55	0:01:55	MOCH	M	1
1	22	0	0:22:00	MOCH	M	1
1	20	30	0:20:30	START		
5	1	40	0:01:40	START		
11	25	20	0:25:20	START		
11	29	0	0:29:00	START		
	0	0	0:00:00	START		
5	17	0	0:17:00	END		
11	6	7	0:06:07	MOCH	F	1
11	4	35	0:04:35	MOCH		1
11	4	10	0:04:10	MOCH	M	1
1	7	20	0:07:20	MOCH	M,F	2
5	15	0	0:15:00	MOCH	M	1
11	3	30	0:03:30	MOCH	M	1
	0	38	0:00:38	MOCH	M	1

1		7	10	0:07:10	START		
5		12	30	0:12:30	START		
11		1	50	0:01:50	START		
	9:50	0	0	0:00:00	START		
		0	0	0:00:00	MOCH	B	2
1		0	10	0:00:10	MOCH	U	1
13		21	48	0:21:48	MOCH	M	1
5		8	2	0:08:02	MOCH	M	1
5		9	14	0:09:14	MOCH	M	1
5		10	0	0:10:00	MOCH	M	1
1		0	0	0:00:00	START		
5		7	30	0:07:30	START		
13		14	58	0:14:58	START		
1		18	8	0:18:08	BCCH	U	2
1		18	12	0:18:12	BCCH	U	2
1		18	41	0:18:41	BCCH	M	2
1		17	31	0:17:31	BCCH	M	1
1		18	52	0:18:52	BCCH	U	2
1		17	19	0:17:19	BCCH	M	1
1		17	54	0:17:54	BCCH	M	1
1		19	17	0:19:17	BCCH	M	2
1		16	49	0:16:49	BCCH	M	1
1		20	11	0:20:11	BCCH	B	2
1		20	28	0:20:28	BCCH	B	2
1		16	0	0:16:00	BCCH	M	1
1		16	24	0:16:24	BCCH	M	1
13		5	36	0:05:36	CHIC	U	1
5		10	9	0:10:09	MOCH	F	1
5		10	33	0:10:33	MOCH	F	1
5		11	1	0:11:01	MOCH	F	1
5		8	53	0:08:53	MOCH	F	1
5		9	30	0:09:30	MOCH	M	1
1		21	9	0:21:09	MOCH	U	1
13		2	45	0:02:45	MOCH	M	1
13		3	52	0:03:52	MOCH	M	1
13		4	6	0:04:06	MOCH	M	1
13		0	0	0:00:00	START		
5		7	38	0:07:38	START		
1		15	2	0:15:02	START		
5		11	59	0:11:59	MOCH	M	1
1		3	22	0:03:22	MOCH	M	2
1		3	58	0:03:58	MOCH	M	2
1		4	43	0:04:43	MOCH	F	2
1		5	1	0:05:01	MOCH	F	2

5	10	43	0:10:43	MOCH	M	2
5	11	17	0:11:17	MOCH	M	1
1	3	28	0:03:28	MOCH	M	2
5	10	43	0:10:43	MOCH	F	2
5	14	22	0:14:22	MOCH	M	1
5	14	38	0:14:38	MOCH	M	1
5	18	15	0:18:15	MOCH	M	1
1	4	30	0:04:30	MOCH	M	2
1	3	11	0:03:11	MOCH	F	2
1	2	48	0:02:48	MOCH	M	2
5	10	38	0:10:38	MOCH	M	1
5	11	1	0:11:01	MOCH	U	2
5	18	40	0:18:40	MOCH	M	1
5	10	24	0:10:24	MOCH	M	1
1	2	10	0:02:10	MOCH	M	2
5	10	12	0:10:12	MOCH	M	1
5	11	35	0:11:35	MOCH	M	1
1	2	10	0:02:10	MOCH	U	2
1	7	3	0:07:03	MOCH	M	2
1	7	3	0:07:03	MOCH	F	2
1	6	37	0:06:37	MOCH	M	2
1	6	4	0:06:04	MOCH	F	2
1	4	58	0:04:58	MOCH	M	2
1	3	49	0:03:49	MOCH	M	2
5	12	12	0:12:12	RBNU	M	1
1	0	0	0:00	start		
5	10	0	0:10:00	start		
13	20	24	0:20:24	start		
5	28	1	0:28:01	MOCH	F	2
5	28	23	0:28:23	MOCH	M	2
5	28	23	0:28:23	MOCH	F	2
5	29	20	0:29:20	MOCH	F	2
13	7	6	0:07:06	MOCH	?	1
5	26	39	0:26:39	MOCH	F	2
5	27	47	0:27:47	MOCH	F	2
5	28	59	0:28:59	MOCH	F	2
13	7	58	0:07:58	MOCH	?	1
5	30	1	0:30:01	MOCH	M	2
5	30	1	0:30:01	MOCH	F	2
5	31	30	0:31:30	MOCH	F	2
5	32	54	0:32:54	MOCH	M	2
5	32	54	0:32:54	MOCH	F	2
13	8	40	0:08:40	MOCH	M	2
13	8	40	0:08:40	MOCH	F	2

5		28	28	0:28:28	MOCH	M	2
5		28	28	0:28:28	MOCH	F	2
13		6	0	0:06:00	MOCH	M	2
13		6	0	0:06:00	MOCH	F	2
5		23	2	0:23:02	MOCH	F	1
5		24	7	0:24:07	MOCH	M	2
5		24	7	0:24:07	MOCH	F	2
1		18	26	0:18:26	MOCH	M	2
1		18	26	1:18:26	MOCH	F	2
1		16	56	0:16:56	MOCH	?	1
1		15	3	0:15:03	MOCH	?	1
1		15	57	0:15:57	MOCH	?	1
13		2	57	0:02:57	RBNU	F	1
13		3	6	0:03:06	RBNU	F	1
13		3	9	0:03:09	RBNU	F	1
13		1	56	0:01:56	RBNU	F	1
13		3	30	0:03:30	RBNU	F	1
13		4	20	0:04:20	RBNU	F	1
13		1	42	0:01:42	RBNU	F	1
1		11	43	0:11:43	RBNU	?	1
13		5	39	0:05:39	RBNU	F	
13		9	46	0:09:46	start		
1		11	0	0:11:00	start		
5		21	30	0:21:30	start		
13	0:16	16	0	0:16:00	RBNU	M	2
13	0:16	16	12	0:16:12	RBNU	M	2
13	0:16	16	26	0:16:26	RBNU	M	2
13	0:18	18	6	0:18:06	RBNU	M	2
13	0:18	18	30	0:18:30	RBNU	F	2
13	0:18	18	43	0:18:43	RBNU	M	2
13	0:16	16	36	0:16:36	RBNU	M	2
13	0:16	16	52	0:16:52	RBNU	M	2
13	0:16	16	53	0:16:53	RBNU	F	2
13	0:18	18	2	0:18:02	RBNU	F	2
13	0:19	19	20	0:19:20	RBNU	B	2
13	0:24	24	0	0:24	RBNU	B	2
13	0:19	19	37	0:19:37	RBNU	B	2
1	0:02	2	22	0:02:22	RBNU	M	1
1	0:03	3	41	0:03:41	RBNU	M	1
1	0:04	4	54	0:04:54	RBNU	F	2
1	0:00	0	29	0:00:29	RBNU	M	1
1	0:00	0	33	0:00:33	RBNU	M	1
1	10:48	0	0	0:00:00	START		
5	0:07	7	30	0:07:30	START		

13	0:15	15	15	0:15:15	START		
5	#VALUE!	15	2	0:15:02	MOCH	M	1
5	#VALUE!	7	47	0:07:47	MOCH	M	1
5	#VALUE!	7	52	0:07:52	MOCH	M	1
5	#VALUE!	8	42	0:08:42	MOCH	M	1
5	#VALUE!	9	3	0:09:03	MOCH	M	1
5	#VALUE!	10	9	0:10:09	MOCH	M	1
5	#VALUE!	11	28	0:11:28	MOCH	M	1
5	#VALUE!	18	59	0:18:59	MOCH	M	1
5	#VALUE!	21	27	0:21:27	MOCH	M	1
5	#VALUE!	7	42	0:07:42	MOCH	M	1
5	#VALUE!	13	43	0:13:43	MOCH	M	1
5	#VALUE!	15	1	0:15:01	MOCH	M	1
5	#VALUE!	15	18	0:15:18	MOCH	M	1
5	#VALUE!	18	30	0:18:30	MOCH	M	1
13	#VALUE!	27	3	0:27:03	MOCH	M	2
13	#VALUE!	27	45	0:27:45	MOCH	M	2
13	#VALUE!	30	11	0:30:11	MOCH	F	2
13	#VALUE!	31	25	0:31:25	MOCH	F	2
5	#VALUE!	15	41	0:15:41	MOCH	M	1
5	#VALUE!	16	10	0:16:10	MOCH	F	2
13	#VALUE!	35	33	0:35:33	MOCH	M	2
5	#VALUE!	7	25	0:07:25	MOCH	M	1
5	#VALUE!	20	1	0:20:01	MOCH	M	1
13	#VALUE!	34	1	0:34:01	MOCH	F	2
13	#VALUE!	34	28	0:34:28	MOCH	F	2
13	#VALUE!	28	11	0:28:11	MOCH	M	2
13	#VALUE!	28	41	0:28:41	MOCH	M	2
13	#VALUE!	29	14	0:29:14	MOCH	M	2
13	#VALUE!	29	45	0:29:45	MOCH	B	2
13	#VALUE!	35	41	0:35:41	MOCH	F	2
13	#VALUE!	26	22	0:26:22	MOCH	B	2
5	#VALUE!	23	48	0:23:48	MOCH	M	1
13	#VALUE!	33	51	0:33:51	MOCH	M	2
13	#VALUE!	34	48	0:34:48	MOCH	M	2
5	#VALUE!	22	51	0:22:51	MOCH	M	1
5	#VALUE!	6	54	0:06:54	MOCH	M	1
13	#VALUE!	27	7	0:27:07	RBNU	M	1
13	#VALUE!	26	51	0:26:51	RBNU	M	1
13	#VALUE!	26	59	0:26:59	RBNU	M	1
13	#VALUE!	26	36	0:26:36	RBNU	M	1
13	#VALUE!	26	2	0:26:02	RBNU	M	1
13	#VALUE!	32	42	0:32:42	RBNU	M	1
13	#VALUE!	25	43	0:25:43	RBNU	M	1

13	#VALUE!	33	34	0:33:34	RBNU	M	1
5	#VALUE!	20	35	0:20:35	RBNU	M	1
5	#VALUE!	11	39	0:11:39	RBNU	M	1
5	#VALUE!	12	13	0:12:13	RBNU	M	1
5	#VALUE!	11	13	0:11:13	RBNU	M	1
1	#VALUE!	0	0	0:00:00	START		
5	#VALUE!	6	38	0:06:38	START		
13	#VALUE!	25	30	0:25:30	START		
15	11:10	12	55	0:12:55	AMRO	U	1
15	11:07	9	2	0:09:02	AMRO	U	1
1	11:20	22	19	0:22:19	MOCH	M	1
1	11:14	16	51	0:16:51	MOCH	F	1
1	11:21	23	44	0:23:44	MOCH	M	1
1	11:19	21	46	0:21:46	MOCH	M	1
1	11:22	24	13	0:24:13	MOCH	M	1
15	11:06	8	8	0:08:08	MOCH	U	1
15	11:06	8	32	0:08:32	MOCH	U	2
15	11:12	14	13	0:14:13	MOCH	U	1
5	10:58	0	24	0:00:24	MOCH	U	1
1	11:16	18	3	0:18:03	MOCH	F	1
1	11:18	20	24	0:20:24	RBNU	M	1
1	11:17	19	39	0:19:39	RBNU	M	1
5	10:58	0	0	0:00	START		
15	11:05	7	36	0:07:36	START		
1	11:14	16	0	0:16:00	START		
1	9:14	1	14	0:01:14	BCCH	M	1
1	9:13	0	30	0:00:30	BCCH	M	1
1	9:14	1	20	0:01:20	CHIC	U	1
5	9:21	9	39	0:09:39	MOCH	M,F	2
5	9:22	10	15	0:10:15	MOCH	M	2
5	9:22	10	15	0:10:15	MOCH	F	2
1	9:13	0	0	0:00:00	MOCH	U	1
1	9:14	1	45	0:01:45	MOCH	M	1
5	9:20	8	52	0:08:52	MOCH	M	2
5	9:22	10	52	0:10:52	MOCH	F	2
5	9:20	8	26	0:08:26	MOCH	M	1
5	9:22	10	52	0:10:52	MOCH	M	2
1	9:16	3	34	0:03:34	MOCH	M	1
13	9:31	20	6	0:20:06	MOCH	M	1
13	9:32	21	41	0:21:41	MOCH	M	3
1	9:15	2	34	0:02:34	MOCH	M	1
1	9:18	5	48	0:05:48	MOCH	M	1
13	9:29	18	9	0:18:09	MOCH	M	1
13	9:30	19	47	0:19:47	MOCH	M	1

5	9:23	12	46	0:12:46	MOCH	U	2
1	9:14	1	59	0:01:59	RNSA	M	2
13	9:26	15	8	0:15:08	RNSA	U	1
5	9:20	8	12	0:08:12	RNSA	U	1
1	9:13	0	0	0:00:00	START		
5	9:19	7	30	0:07:30	START		
13	9:26	15	0	0:15:00	START		
5	9:19	7	50	0:07:50	TRES	U	1
1	11:06	24	4	0:24:04	BCCH	U	1
5	11:01	19	16	0:19:16	DOWO		1
5	10:58	16	21	0:15:21	MOCH	M	1
1	11:04	22	44	0:22:44	MOCH	M	2
5	10:56	14	11	0:14:11	MOCH	M	1
5	10:57	15	28	0:15:28	MOCH	M	1
5	10:55	13	6	0:13:06	MOCH	M	1
5	10:55	13	38	0:13:38	MOCH	M	1
5	10:57	15	34	0:15:34	MOCH	M	1
13	10:48	6	0	0:06:00	MOCH	M	1
5	10:54	12	34	0:12:34	MOCH	M	1
5	10:57	15	2	0:15:02	MOCH	F	2
1	11:04	22	25	0:22:25	MOCH	M	2
1	11:04	22	25	0:22:25	MOCH	M	2
1	11:06	24	4	0:24:04	MOCH	M	1
1	11:06	24	49	0:24:49	MOCH	M	
13	10:45	3	40	0:03:40	RBNU	M	1
13	10:45	3	59	0:03:59	RBNU	M	1
13	10:44	2	33	0:02:33	RBNU	M	1
13	10:49	7	18	0:07:18	RBNU	M	1
13	10:44	2	11	0:02:11	RBNU	M	1
13	10:47	5	7	0:05:07	RBNU	M	1
13	10:50	8	57	0:08:57	RBNU	M	1
5	10:57	15	36	0:15:36	RBNU	M	1
5	10:54	12	37	0:12:37	RBNU	M	1
1	11:07	25	0	0:25:00	RBNU		1
13	10:48	6	13	0:06:13	RBNU	M	1
5	10:57	15	16	0:15:16	RBNU	M	1
13	10:42	0	0	0:00:00	START		
5	10:54	12	0	0:12:00	START		
1	11:03	21	30	0:21:30	START		
13	10:43	1	21	0:01:21	TRES	U	2
13	10:43	1	21	0:01:21	TRES	U	2
13	10:51	9	28	0:09:28	TRES	U	1
13	10:53	17	52	0:17:52	DOWO	U	1
1	10:36	0	28	0:00:28	MOCH	U	1

5	10:45	9	6	0:09:06	MOCH	M	1
5	10:45	9	27	0:09:27	MOCH	F	1
5	10:48	12	37	0:12:37	MOCH	F	2
5	10:48	12	43	0:12:43	MOCH	M	2
13	10:52	16	31	0:16:31	MOCH	F	2
1	10:39	3	38	0:03:38	MOCH	M	1
1	10:41	5	24	0:05:24	MOCH	M	1
5	10:44	8	51	0:08:51	MOCH	M	1
5	10:47	11	1	0:11:01	MOCH	M	1
5	10:48	12	26	0:12:26	MOCH	M	1
5	10:49	13	48	0:13:48	MOCH	M	2
5	10:44	8	24	0:08:24	MOCH	M	1
5	10:44	8	11	0:08:11	MOCH	M	1
13	10:52	16	45	0:16:45	RBNU	M	1
13	10:55	19	48	0:19:48	RBNU	M	1
5	10:47	11	28	0:11:28	RBNU	M	1
1	10:42	6	4	0:06:04	RBNU	U	1
5	10:46	10	19	0:10:19	RBNU	M	1
1	10:36	0	0	0:00:00	START		
5	10:43	7	30	0:07:30	START		
13	10:51	15	0	0:15:00	START		
5	12:06	8	15	0:08:15	MOCH	M	1
5	12:06	8	47	0:08:47	MOCH	M	1
5	12:05	7	29	0:07:29	MOCH	M	1
13	12:10	12	4	0:12:04	MOCH	M	1
13	12:11	13	36	0:13:36	MOCH	M	1
5	12:08	10	1	0:10:01	MOCH	M	1
13	12:11	13	38	0:13:38	RBNU	M	1
13	12:12	14	2	0:14:02	RBNU	M	1
13	12:13	15	17	0:15:17	RBNU	M	1
13	12:10	12	50	0:12:50	RBNU	M	1
13	12:10	12	18	0:12:18	RBNU	M	1
1	12:02	4	46	0:04:46	RBNU	M	1
1	12:00	2	51	0:02:51	RBNU	M	1
1	11:59	1	52	0:01:52	RBNU	M	1
1	11:58	0	0	0:00:00	START		
5	12:04	6	0	0:06:00	START		
13	12:10	12	0	0:12:00	START		
5	11:24	1	2	0:01:02	MOCH	M	1
5	11:23	0	58	0:00:58	MOCH	M	1
5	11:27	4	3	0:04:03	MOCH	M	2
5	11:27	4	41	0:04:41	MOCH	F	2
5	11:28	5	33	0:05:33	MOCH	F	1
5	11:34	11	43	0:11:43	MOCH	M	2

5	11:23	0	30	0:00:30	MOCH	M	1
5	11:24	1	30	0:01:30	MOCH	M	1
5	11:25	2	51	0:02:51	MOCH	M	1
5	11:26	3	18	0:03:18	MOCH	M	1
5	11:27	4	3	0:04:03	MOCH	F	2
5	11:29	6	14	0:06:14	MOCH	M	2
5	11:31	8	4	0:08:04	MOCH	M	2
5	11:34	11	20	0:11:20	MOCH	M	2
5	11:32	9	50	0:09:50	MOCH	M	2
5	11:33	10	2	0:10:02	MOCH	M	2
5	11:33	10	18	0:10:18	MOCH	M	2
5	11:23	0	23	0:00:23	MOCH	M	1
5	11:35	12	5	0:12:05	MOCH	M	2
5	11:28	5	29	0:05:29	MOCH	M	2
5	11:35	12	38	0:12:38	RBNU	M	1
5	11:23	0	0	0:00:00	START		
1	11:37	14	0	0:14:00	START		
13	11:42	19	0	0:19:00	START		
5	8:16	23	0	0:23:00	END		
1	8:02	9	30	0:09:30	MOCH	F	1
5	8:12	19	0	0:19:00	MOCH	F	1
5	8:10	17	40	0:17:40	MOCH	F	1
5	8:11	18	0	0:18:00	MOCH	F	1
1	8:02	9	35	0:09:35	MOCH	F	1
1	8:04	11	0	0:11:00	MOCH	F	1
1	8:04	11	45	0:11:45	MOCH	F	1
	7:53	0	0	0:00:00	MOCH	F	1
1	8:02	9	18	0:09:18	START		
5	8:09	16	58	0:16:58	START		
13	7:56	3	10	0:03:30	START		
	7:53	0	0	0:00:00	START		
1	8:24	4	27	0:04:27	BCCH	M	1
1	8:26	6	33	0:06:33	BCCH	M	1
1	8:28	8	47	0:08:47	BCCH	M	1
1	8:25	5	13	0:05:13	BCCH	M	1
1	8:26	6	54	0:06:54	BCCH	M	1
1	8:29	9	5	0:09:05	BCCH	M	1
1	8:34	14	13	0:14:13	BCCH	M	1
1	8:24	4	10	0:04:10	BCCH	M	1
5	8:20	0	0	0:00:00	START		
1	8:24	4	0	0:04:00	START		
13	8:35	15	0	0:15:00	START		
13	8:00	11	41	0:11:41	MOCH	F	1
13	7:48	3	46	0:03:46	MOCH	M	1

13	7:49	4	10	0:04:10	MOCH	M	1
13	8:01	12	30	0:12:30	MOCH	F	1
13	8:03	14	2	0:14:02	MOCH	F	1
1	8:06	17	47	0:17:47	MOCH	F	1
5	8:08	19	1	0:19:01	MOCH	F	1
5	8:08	19	4	0:19:04	MOCH	F	1
5	8:09	20	24	0:20:24	MOCH	F	1
5	8:09	20	48	0:20:48	MOCH	F	1
13	7:45	0	0	0:00:00	START		
1	8:04	15	0	0:15:00	START		
5	8:08	19	0	0:19:00	START		
1	10:34	12	31	0:12:31	BCCH	M	1
1	10:34	12	5	0:12:05	BCCH	M	1
1	10:33	11	34	0:11:34	BCCH	M	1
1	10:33	11	11	0:11:11	BCCH	M	1
1	10:34	12	53	0:12:53	BCCH	M	1
5	10:41	19	0	0:19:00	CHIC	U	1
13	10:28	6	2	0:06:02	MOCH	F	3
13	10:25	3	51	0:03:51	MOCH	M	1
13	10:28	6	0	0:06:00	MOCH	M	1
13	10:30	8	11	0:08:11	MOCH	M	3
1	10:32	10	29	0:10:29	MOCH	M	1
1	10:36	14	12	0:14:12	MOCH	M	1
5	10:37	15	15	0:15:15	MOCH	M	1
5	10:37	15	27	0:15:27	MOCH	M	2
13	10:29	7	40	0:07:40	MOCH	M	3
13	10:30	8	54	0:08:54	MOCH	M	3
13	10:30	8	54	0:08:54	MOCH	F	3
5	10:38	16	30	0:16:30	RBNU	M	1
5	10:39	17	35	0:17:35	RBNU	M	1
5	10:41	19	0	0:19:00	RBNU	M	1
13	10:22	0	0	0:00:00	START		
1	10:32	10	0	0:10:00	START		
5	10:37	15	0	0:15:00	START		
5	11:28	11	7	0:11:07	MOCH	M	1
13	11:19	2	14	0:02:14	RBNU	M	1
1	11:22	5	22	0:05:22	RBNU	M	1
5	11:27	10	13	0:10:13	RBNU	M	1
1	11:24	7	29	0:07:29	RBNU	M	1
13	11:17	0	0	0:00:00	START		
1	11:22	5	0	0:05:00	START		
5	11:27	10	0	0:10:00	START		
5	8:39	2	7	0:02:07	MOCH	M	1
5	8:37	0	42	0:00:42	MOCH	M	1

5	8:41	4	36	0:04:36	MOCH	M	1
13	8:51	14	24	0:14:24	MOCH	M	2
13	8:50	13	34	0:13:34	MOCH	M	2
13	8:50	13	34	0:13:34	MOCH	F	2
1	8:43	6	0	0:06:00	START		
5	8:37	0	0	0:00:00	START		
13	8:47	10	0	0:10:00	START		
1	9:34	9	28	0:09:28	BCCH	M	1
5	9:42	17	11	0:17:11	BCCH	M	1
5	9:41	16	4	0:16:04	BCCH	M	1
13	9:31	6	0	0:06:00	BCCH	M	1
5	9:40	15	22	0:15:22	MOCH	M	1
5	9:41	16	33	0:16:33	MOCH	M	1
5	9:42	17	11	0:17:11	MOCH	M	1
1	9:34	9	6	0:09:06	MOCH	U	1
5	9:45	20	16	0:20:16	MOCH	M	1
5	9:38	13	23	0:13:23	MOCH	M	1
13	9:25	0	0	0:00:00	MOCH	M	1
13	9:29	4	30	0:04:30	MOCH	M	1
13	9:27	2	26	0:02:26	MOCH	M	1
1	9:31	6	40	0:06:40	START		
5	9:37	12	30	0:12:30	START		
13	9:25	0	0	0:00:00	START		
13	8:00	15	57	0:15:57	BCCH	M	1
13	7:56	11	45	0:11:45	MOCH	F	2
13	8:03	18	12	0:18:12	MOCH	U	2
13	8:03	18	12	0:18:12	MOCH	U	2
1	7:46	1	44	0:01:44	MOCH	M	2
1	7:46	1	44	0:01:44	MOCH	F	2
5	7:50	5	44	0:05:44	MOCH	M	2
13	7:59	14	30	0:14:30	MOCH	U	2
13	8:00	15	3	0:15:03	MOCH	U	2
13	8:02	17	3	0:17:03	MOCH	U	2
13	8:02	17	51	0:17:51	MOCH	U	2
13	7:59	14	25	0:14:25	RBNU	M	1
13	8:01	16	42	0:16:42	RBNU	M	1
13	7:56	11	45	0:11:45	RBNU	M	1
13	7:57	12	20	0:12:20	RBNU	M	1
13	7:59	14	42	0:14:42	RBNU	M	1
1	7:46	1	7	0:01:07	RBNU	M	1
1	7:46	1	44	0:01:44	RBNU	M	1
1	7:48	3	34	0:03:34	RBNU	M	1
13	8:03	18	12	0:18:12	RBNU	M	1
1	7:45	0	0	0:00:00	START		

5	7:50	5	0	0:05:00	START		
13	7:55	10	0	0:10:00	START		
1	10:50	10	0	0:10:00	START		
5	10:45	5	0	0:05:00	START		
13	10:40	0	0	0:00:00	START		
1	10:53	13	13	0:13:13			
	10:50	0	0	0	MOCH		1
3	11:01	11	0	0:11:00	MOCH		1
3	11:01	11	15	0:11:15	MOCH		1
3	11:01	11	36	0:11:36	MOCH		1
3	11:01	11	51	0:11:51	MOCH		1
	10:50	0	0	0	RBNU		1
5	10:52	2	11	0:02:11	start		
3	10:58	8	4	0:08:04	start		
11	11:05	15	30	0:15:30	start		
	11:10	20	7	0:20:07	stop		
	10:50	0	0	0			
5	10:11	13	30	0:13:30	MOCH	M	1
5	10:10	12	51	0:12:51	MOCH	M	2
5	10:11	13	12	0:13:12	MOCH	M	1
5	10:08	10	10	0:10:10	MOCH		1
5	10:08	10	28	0:10:28	MOCH	M	1
5	10:07	9	53	0:09:53	MOCH		1
5	10:07	9	20	0:09:20	MOCH		1
5	10:13	15	36	0:15:36	MOCH	M	1
5	10:13	15	57	0:15:57	MOCH	M	1
5	10:14	16	14	0:16:14	MOCH	M	1
5	10:15	17	7	0:17:07	MOCH	M	1
11	10:20	22	19	0:22:19	MOCH		2
11	10:21	23	0	0:23:00	MOCH		2
3	10:03	5	1	0:05:01	MOCH		1
5	10:07	9	15	0:09:15	MOCH		1
5	10:09	11	40	0:11:40	MOCH	M	2
5	10:10	12	29	0:12:29	MOCH	M	2
3	10:04	6	42	0:06:42	MOCH		1
11	10:19	21	19	0:21:19	MOCH	M	2
5	10:17	19	42	0:19:42	MOCH	M	2
11	10:18	20	31	0:20:31	MOCH	M	2
	9:58	0	45	0:00:45	MOCH	M	1
5	10:15	17	35	0:17:35	MOCH	M	2
11	10:26	28	15	0:28:15	RBNU		1
3	10:02	4	2	0:04:02	RNSA		1
3	9:59	1	35	0:01:35	start		
5	10:07	9	6	0:09:06	start		

11	10:18	20	0	0:20:00	start			
	10:26	28	15	0:28:15	stop			
	9:58	0	0	0				
3	10:41	3	55	0:03:55	BCCH			1
5	10:59	21	40	0:21:40	BCCH			1
3	10:41	3	36	0:03:36	BCCH			1
3	10:41	3	20	0:03:20	BCCH			1
5	10:59	21	15	0:21:15	BCCH			2
5	10:59	21	15	0:21:15	BCCH			2
5	10:59	21	39	0:21:39	BCCH			1
3	10:40	2	50	0:02:50	BCCH			1
3	10:42	4	10	0:04:10	BCCH			1
3	10:43	5	4	0:05:04	BCCH			3
11	10:50	12	52	0:12:52	BCCH	M		2
11	10:52	14	56	0:14:56	BCCH	M		2
5	10:57	19	45	0:19:45	BCCH	M		1
11	10:49	11	21	0:11:21	BCCH	M		1
5	10:56	18	11	0:18:11	BCCH	M		1
5	10:58	20	20	0:20:20	BCCH	M		2
11	10:49	11	44	0:11:44	BCCH	M		2
11	10:50	12	53	0:12:52	BCCH	M		2
11	10:52	14	17	0:14:17	BCCH	M		1
	10:38	0	0	0	MOCH			1
3	10:46	8	0	0:08:00	MOCH	M		1
5	10:59	21	40	0:21:40	MOCH		AK/PY	1
5	11:03	25	40	0:25:40	MOCH		AK/PY	1
11	10:48	10	34	0:10:34	MOCH			1
11	10:48	10	50	0:10:50	MOCH			1
11	10:49	11	16	0:11:16	MOCH			1
5	10:57	19	0	0:19:00	MOCH	M		1
3	10:40	2	40	0:02:40	start			
11	10:48	10	9	0:10:09	start			
5	10:56	18	10	0:18:10	start			
5	11:04	26	3	0:26:03	stop			
5	10:59	21	20	0:21:20	TRES			1
	10:38	0	0	0				
3	9:30	11	11	0:11:11	BCCH			1
3	9:33	14	3	0:14:03	MOCH	M	AK/YP	1
3	9:31	12	15	0:12:15	MOCH	M	AK/YP	1
3	9:31	12	43	0:12:43	MOCH	M	AK/YP	1
5	9:36	17	52	0:17:52	MOCH	M	AK/YP	1
5	9:37	18	3	0:18:03	MOCH	M	AK/YP	1
5	9:37	18	28	0:18:28	MOCH	M	AK/YP	1
5	9:38	19	9	0:19:09	MOCH	M	AK/YP	2

3	9:30	11	34	0:11:34	MOCH	M	AK/YP	1
3	9:36	17	30	0:17:30	MOCH	M	AK/YP	1
5	9:37	18	38	0:18:38	MOCH	M	AK/YP	1
5	9:37	18	48	0:18:48	MOCH			2
5	9:38	19	58	0:19:58	MOCH			2
5	9:42	23	41	0:23:41	MOCH			2
3	9:34	15	43	0:15:43	MOCH	M	AK/YP	1
5	9:40	21	45	0:21:45	MOCH			2
5	9:39	20	43	0:20:43	RBNU			1
	9:19	0	0	0:00:00	RBNU			1
11	9:26	7	31	0:07:31	RBNU			1
11	9:23	4	4	0:04:03	start			
3	9:29	10	8	0:10:08	start			
5	9:36	17	41	0:17:41	start			
5	9:44	25	20	0:25:20	stop			
	9:19	0	0	0:00:00				
5	10:56	11	18	0:11:18	MOCH	M		1
5	10:52	7	50	0:07:50	MOCH	M		1
5	10:54	9	50	0:09:50	MOCH	M		1
5	10:52	7	55	0:07:55	MOCH	M		1
5	10:55	10	8	0:10:08	MOCH	M		1
5	10:56	11	38	0:11:38	MOCH	M		1
5	10:57	12	44	0:12:44	MOCH	M		1
11	10:49	4	21	0:04:21	RBNU			1
5	10:59	14	0	0:14:00	RBNU			1
5	10:56	11	4	0:11:04	RBNU			1
5	10:56	11	38	0:11:38	RBNU			1
5	10:57	12	43	0:12:43	RBNU			1
3	11:02	17	17	0:17:17	RBNU			1
11	10:48	3	49	0:03:49	RBNU			1
11	10:46	1	6	0:01:06	start			
5	10:51	6	55	0:06:55	start			
3	10:59	14	47	0:14:47	start			
3	11:04	19	31	0:19:31	stop			
	10:45	0	0	0:00:00				
5	9:24	8	13	0:08:13	BCCH			1
11	9:27	11	54	0:11:54	RBNU			1
3	9:17	1	18	0:01:18	start			
5	9:21	5	34	0:05:34	start			
11	9:26	10	47	0:10:47	start			
11	9:30	14	28	0:14:28	stop			
	9:16	0	0	0:00:00				
11	11:05	10	40	0:10:40	DOWO			1
3	10:57	2	48	0:02:48	MOCH			1

11	11:05	10	30	0:10:30	RBNU			1
11	11:02	7	20	0:07:20	RBNU			1
3	10:57	2	10	0:02:10	start			
11	11:01	6	59	0:06:59	start			
5	11:08	13	48	0:13:48	start			
5	11:13	18	40	0:18:40	stop			
	10:55	0	0	0:00:00				
3	12:07	4	32	0:04:32	BCCH			1
3	12:10	7	52	0:07:52	MOCH			1
3	12:10	7	9	0:07:09	MOCH			1
3	12:10	7	29	0:07:29	MOCH			1
3	12:11	8	28	0:08:28	MOCH			1
3	12:09	6	50	0:06:50	MOCH			1
3	12:11	8	52	0:08:52	MOCH			1
3	12:12	9	27	0:09:27	MOCH			1
3	12:07	4	52	0:04:52	MOCH			1
3	12:08	5	39	0:05:39	MOCH			1
3	12:08	5	58	0:05:58	MOCH			1
3	12:09	6	21	0:06:21	MOCH			1
3	12:12	9	45	0:09:45	MOCH			1
3	12:13	10	1	0:10:01	MOCH			1
5	12:15	12	54	0:12:54	MOCH			2
5	12:16	13	21	0:13:21	MOCH			2
5	12:17	14	43	0:14:43	MOCH			2
5	12:15	12	37	0:12:37	MOCH	M		2
5	12:16	13	34	0:13:34	MOCH	M		2
3	12:12	9	34	0:09:34	RBNU			1
3	12:06	3	45	0:03:45	start			
5	12:15	12	2	0:12:02	start			
11	12:20	17	18	0:17:18	start			
11	12:25	22	30	0:22:30	stop			
	12:03	0	0	0:00:00				
3	8:22	11	44	0:11:44	BCCH			1
3	8:23	12	48	0:12:48	BCCH			1
3	8:26	15	36	0:15:36	BCCH	M		1
5	8:27	16	32	0:16:32	BCCH	M		1
3	8:25	14	19	0:14:19	BCCH			1
3	8:21	10	54	0:10:54	MOCH		AK/YP	1
3	8:22	11	18	0:11:18	MOCH		AK/YP	1
3	8:20	9	46	0:09:46	MOCH		AK/YP	1
3	8:22	11	33	0:11:33	MOCH		AK/YP	1
5	8:27	17	19	0:17:19	MOCH			1
3	8:25	14	28	0:14:28	MOCH			1
5	8:27	17	39	0:17:39	MOCH			2

5	8:28	18	30	0:18:30	MOCH		1
11	8:15	2	36	0:02:36	RBNU		2
11	8:16	3	9	0:03:09	RBNU		1
11	8:17	4	50	0:04:50	RBNU		1
5	8:31	21	3	0:21:03	RBNU		1
11	8:15	2	36	0:02:36	RBNU		2
5	8:30	20	19	0:20:19	RBNU		1
11	8:18	5	40	0:05:40	RBNU		1
3	8:25	14	36	0:14:36	RBNU		1
5	8:28	18	38	0:18:38	RBNU		1
	8:13	0	0	0:00:00	RBNU		
11	8:15	2	14	0:02:14	start		
3	8:19	8	24	0:08:24	start		
5	8:26	15	57	0:15:57	start		
5	8:32	22	34	0:22:34	stop		
11	8:16	3	40	0:03:40	UKWO		1
	8:13	0	0	0:00:00			
3	9:55	16	7	0:16:07	BCCH	M	1
3	9:52	13	23	0:13:23	BCCH	M	1
3	9:52	13	9	0:13:09	BCCH	M	1
3	9:52	13	46	0:13:46	BCCH	M	1
3	9:53	14	12	0:14:12	BCCH	M	1
3	9:53	14	27	0:14:27	BCCH	M	1
5	9:56	17	16	0:17:16	BCCH	M	1
3	9:51	12	40	0:12:40	BCCH	M	1
3	9:53	14	40	0:14:40	BCCH	M	1
3	9:50	11	20	0:11:20	BCCH	M	1
3	9:49	10	29	0:10:29	BCCH	M	1
5	9:56	17	49	0:17:49	BCCH	M	1
5	9:57	18	8	0:18:08	BCCH	M	1
5	9:58	19	16	0:19:16	BCCH	M	1
5	10:01	22	56	0:22:56	BCCH	M	1
11	9:47	8	20	0:08:20	MOCH	F	2
11	9:47	8	20	0:08:20	MOCH	M	2
11	9:45	6	28	0:06:28	MOCH	M	1
11	9:47	8	9	0:08:09	MOCH	M	1
11	9:46	7	42	0:07:42	RBNU	M	1
11	9:45	6	40	0:06:40	RBNU	M	1
5	9:56	17	23	0:17:23	RBNU		1
3	9:53	14	51	0:14:51	RBNU		1
11	9:41	2	45	0:02:45	RBNU	M	1
11	9:41	2	58	0:02:58	RBNU	M	1
11	9:41	2	24	0:02:24	RBNU	M	1
11	9:43	4	52	0:04:52	RBNU	M	1

11	9:44	5	10	0:05:10	RBNU	M	1
3	9:53	14	40	0:14:40	RBNU		1
11	9:41	2	17	0:02:17	RBNU	M	1
11	9:41	2	37	0:02:37	RBNU	M	1
11	9:42	3	29	0:03:29	RBNU	M	1
11	9:42	3	40	0:03:40	RBNU	M	1
11	9:41	2	1	0:02:01	RBNU	M	1
3	9:48	9	59	0:09:59	RBNU		1
3	9:50	11	8	0:11:08	RBNU		1
5	9:56	17	39	0:17:39	RBNU		1
3	9:50	11	30	0:11:30	RBNU		1
3	9:54	15	0	0:15:00	RBNU		1
5	9:58	19	1	0:19:01	RBNU		1
3	9:51	12	27	0:12:27	RBNU		2
11	9:43	4	0	0:04:00	RBNU		2
11	9:40	1	42	0:01:42	start		
3	9:48	9	36	0:09:36	start		
5	9:56	17	7	0:17:07	start		
5	10:03	24	53	0:24:53	stop		
	9:39	0	0	0:00:00			
11	9:47	5	47	0:05:47	MOCH		2
3	9:56	14	57	0:14:57	MOCH		1
3	9:59	17	43	0:17:43	MOCH		1
11	9:47	5	47	0:05:47	MOCH		2
5	9:49	7	37	0:07:37	MOCH		2
3	9:55	13	6	0:13:06	MOCH		1
11	9:45	3	57	0:03:57	RBNU		1
5	9:51	9	59	0:09:59	RBNU		1
3	9:57	15	58	0:15:58	RBNU		1
11	9:45	3	1	0:03:01	RBNU		1
5	9:51	9	2	0:09:02	RBNU		1
5	9:53	11	55	0:11:55	RNSA		1
11	9:43	1	13	0:01:13	start		
5	9:49	7	10	0:07:10	start		
3	9:54	12	47	0:12:47	start		
3	10:01	19	0	0:19:00	stop		
	9:42	0	0	0:00:00			
5	11:31	4	18	0:04:18	MOCH	M	1
5	11:32	5	51	0:05:51	MOCH	M	2
5	11:33	6	2	0:06:02	MOCH		2
5	11:33	6	28	0:06:28	MOCH		2
5	11:33	6	57	0:06:57	MOCH		3
5	11:34	7	27	0:07:27	MOCH		3
5	11:34	7	15	0:07:15	MOCH		3

5	11:30	3	53	0:03:53	MOCH	M	1
5	11:32	5	28	0:05:28	MOCH		2
5	11:34	7	37	0:07:37	MOCH		3
5	11:31	4	31	0:04:31	MOCH	M	1
5	11:31	4	44	0:04:44	MOCH	M	1
5	11:33	6	45	0:06:45	MOCH		3
5	11:34	7	51	0:07:51	MOCH		3
5	11:35	8	32	0:08:32	MOCH		3
11	11:44	17	48	0:17:48	MOCH		1
3	11:38	11	40	0:11:40	MOCH		1
5	11:35	8	58	0:08:58	MOCH		3
11	11:45	18	31	0:18:31	MOCH		1
3	11:39	12	55	0:12:55	MOCH		1
11	11:45	18	13	0:18:13	RBNU		1
11	11:44	17	19	0:17:19	RBNU		1
11	11:43	16	31	0:16:31	RBNU		1
5	11:29	2	12	0:02:12	start		
3	11:36	9	38	0:09:38	start		
11	11:42	15	31	0:15:31	start		
11	11:47	20	11	0:20:11	stop		
	11:27	0	0	0:00:00			
11	10:23	2	31	0:02:31	MOCH		1
3	10:30	9	26	0:09:26	MOCH		1
3	10:30	9	58	0:09:58	MOCH		1
3	10:31	10	12	0:10:12	MOCH		1
3	10:32	11	43	0:11:43	MOCH		1
3	10:32	11	52	0:11:52	MOCH		1
3	10:33	12	49	0:12:49	MOCH		1
3	10:34	13	5	0:13:05	MOCH		1
3	10:34	13	31	0:13:31	MOCH		1
5	10:37	16	7	0:16:07	MOCH		1
5	10:39	18	6	0:18:06	MOCH		1
5	10:39	18	13	0:18:13	MOCH		1
5	10:39	18	41	0:18:41	MOCH		1
5	10:39	18	52	0:18:52	MOCH		1
5	10:40	19	49	0:19:49	MOCH		1
5	10:40	19	59	0:19:59	MOCH		1
5	10:41	20	8	0:20:08	MOCH		
11	10:23	2	0	0:02:00	MOCH		1
5	10:38	17	52	0:17:52	MOCH		1
11	10:23	2	55	0:02:55	RBNU	M	1
11	10:25	4	58	0:04:58	RBNU		2
11	10:25	4	14	0:04:14	RBNU		2
11	10:28	7	50	0:07:05	RBNU	F	1

11	10:24	3	35	0:03:35	RBNU			2
11	10:22	1	32	0:01:32	start			
3	10:29	8	14	0:08:14	start			
5	10:36	15	45	0:15:45	start			
5	10:41	20	59	0:20:59	stop			
	10:21	0	0	0:00:00				
11	8:08	4	36	0:04:36	MOCH			1
3	8:12	8	28	0:08:28	MOCH	F		1
3	8:13	9	19	0:09:19	MOCH			1
3	8:15	11	55	0:11:55	MOCH			1
3	8:16	12	28	0:12:28	MOCH			1
3	8:17	13	47	0:13:47	MOCH			1
3	8:18	14	1	0:14:01	MOCH			1
3	8:19	15	10	0:15:10	MOCH			1
3	8:19	15	19	0:15:19	MOCH			1
5	8:22	18	31	0:18:31	MOCH	M	AK/YP	1
5	8:22	18	43	0:18:43	MOCH	M	AK/YP	1
5	8:24	20	10	0:20:10	MOCH	M	AK/YP	2
5	8:24	20	25	0:20:25	MOCH	M	AK/YP	2
5	8:24	20	25	0:20:25	MOCH	F		2
5	8:25	21	40	0:21:40	MOCH	M	AK/YP	1
5	8:23	19	53	0:19:53	MOCH	M	AK/YP	2
5	8:23	19	53	0:19:53	MOCH	F		2
5	8:25	21	34	0:21:34	MOCH	M	AK/YP	1
5	8:21	17	58	0:17:58	MOCH	M	AK/YP	1
5	8:25	21	23	0:21:23	MOCH	M	AK/YP	1
11	8:11	7	3	0:07:03	MOCH	F		2
5	8:23	19	2	0:19:02	MOCH	M	AK/YP	1
5	8:21	17	31	0:17:31	MOCH	M	AK/YP	1
3	8:18	14	1	0:14:01	RBNU			1
11	8:07	3	9	0:03:09	RBNU			1
5	8:24	20	43	0:20:43	RBNU			1
3	8:15	11	26	0:11:26	RBNU			1
11	8:05	1	5	0:01:05	start			
3	8:12	8	17	0:08:17	start			
5	8:19	15	49	0:15:49	start			
5	8:26	22	16	0:22:16	stop			
	8:04	0	0	0:00:00				
	8:38	1	31	0:01:31	MOCH	M	AB/OB	2
	8:38	1	31	0:01:31	MOCH	F	AK/PY	2
3	8:45	8	45	0:08:45	MOCH	F	AK/PY	2
3	8:47	10	5	0:10:05	MOCH	F	AK/PY	2
3	8:48	11	18	0:11:18	MOCH	F	AK/PY	2
5	8:48	11	27	0:11:27	MOCH	F	AK/PY	2

5	8:51	14	7	0:14:07	MOCH	M	AB/OB	1
5	8:51	14	25	0:14:25	MOCH	M	AB/OB	1
5	8:52	15	20	0:15:20	MOCH	F	AK/PY	1
11	8:57	20	40	0:20:40	MOCH	M	AB/OB	1
11	8:57	20	50	0:20:50	MOCH	M	AB/OB	1
3	8:47	10	29	0:10:29	MOCH	M	AB/OB	1
5	8:49	12	55	0:12:55	MOCH	M	AB/OB	1
3	8:42	5	31	0:05:31	MOCH	M	AB/OB	1
3	8:42	5	59	0:05:59	MOCH	M	AB/OB	1
3	8:41	4	34	0:04:34	MOCH	M	AB/OB	1
5	8:49	12	46	0:12:46	MOCH	M	AB/OB	1
5	8:52	15	7	0:15:07	MOCH	F	AK/PY	1
11	8:57	20	32	0:20:32	MOCH	M	AB/OB	1
3	8:43	6	29	0:06:29	MOCH	M		2
5	8:50	13	22	0:13:22	MOCH	M	AB/OB	1
5	8:50	13	38	0:13:38	MOCH	M	AB/OB	1
3	8:44	7	27	0:07:27	MOCH	M	AB/OB	2
3	8:44	7	27	0:07:27	MOCH	M		2
3	8:40	3	53	0:03:53	start			
5	8:48	11	26	0:11:26	start			
11	8:54	17	59	0:17:59	start			
11	9:01	24	14	0:24:14	stop			
	8:37	0	0	0:00:00				
5	9:44	0	43	0:00:43	MOCH			1
11	9:51	7	36	0:07:36	MOCH			2
11	9:54	10	57	0:10:57	MOCH			2
5	9:49	5	17	0:05:17	MOCH			2
11	9:56	12	57	0:12:57	RBNU			1
5	9:47	3	16	0:03:16	RCKI			1
5	9:44	0	22	0:00:22	start			
11	9:51	7	17	0:07:17	start			
3	9:59	15	27	0:15:27	start			
3	10:03	19	24	0:19:24	stop			
	9:44	0	0	0:00:00				
3		13	40	0:13:40	DOWO	U		2
3		17	44	0:17:44	MOCH	M		2
3		18	6	0:18:06	MOCH	M		2
5		7	12	0:07:12	MOCH	M		2
3		18	47	0:18:47	MOCH	M		2
3		18	49	0:18:49	MOCH	M		2
11		0	10	0:00:10	MOCH	F		1
11		4	40	0:04:40	MOCH	F		2
11		4	45	0:04:45	MOCH	M		2
5		8	40	0:08:40	MOCH	F		2

5		9	34	0:09:34	MOCH	M	2
5		9	34	0:09:34	MOCH	F	2
3		17	8	0:17:08	MOCH	M	2
3		19	28	0:19:28	MOCH	F	2
3		19	30	0:19:30	MOCH	F	2
5		8	41	0:08:41	MOCH	F	2
5		9	23	0:09:23	MOCH	M	2
3		13	14	0:13:14	MOCH	M	2
3		16	39	0:15:15	MOCH	F	2
5		10	13	0:10:13	MOCH	M	2
5		8	50	0:08:50	MOCH	F	2
3		14	28	0:14:28	MOCH	M	2
5		9	10	0:09:10	MOCH	M	2
11		3	43	0:03:43	MOCH	M	2
5		7	10	0:07:10	MOCH	M	2
11		0	0	0:00:00	START		
5		6	20	0:06:20	START		
3		12	8	0:12:08	START		
3	10:37	18	26	0:18:26	BCCH	M	1
3	10:41	22	25	0:22:25	BCCH	M	1
3	10:37	18	57	0:18:57	BCCH	M	1
3	10:38	19	47	0:19:47	BCCH	M	1
3	10:40	21	0	0:21:00	BCCH	M	1
3	10:41	22	38	0:22:38	BCCH	M	1
3	10:36	17	15	0:17:15	BCCH	M	1
5	10:25	4	5	0:04:05	MOCH	M	1
5	10:26	6	36	0:06:36	MOCH		1
11	10:32	13	6	0:13:06	MOCH		1
3	10:38	19	23	0:19:23	MOCH		1
3	10:40	21	0	0:21:00	MOCH		1
3	10:42	23	34	0:23:34	MOCH		1
3	10:42	23	50	0:23:50	MOCH		1
11	10:34	15	2	0:15:02	MOCH		1
3	10:39	20	9	0:20:09	MOCH		1
5	10:24	3	34	0:03:34	MOCH	M	1
5	10:26	6	1	0:06:01	MOCH	M	1
5	10:26	6	30	0:06:30	MOCH		1
5	10:26	6	30	0:06:30	MOCH		1
11	10:33	14	34	0:14:34	MOCH		1
11	10:35	16	10	0:16:10	MOCH		2
11	10:35	16	10	0:16:10	MOCH		2
3	10:36	17	27	0:17:27	MOCH		1
3	10:37	18	57	0:18:57	MOCH		1
11	10:31	12	30	0:12:30	MOCH		1

11	10:30	11	34	0:11:34	MOCH		1
3	10:35	16	53	0:16:53	MOCH		1
11	10:30	11	58	0:11:58	MOCH		1
11	10:29	10	0	0:10:00	RBNU	M	1
11	10:29	10	32	0:10:32	RBNU	M	1
11	10:28	9	48	0:09:48	RBNU	M	1
11	10:28	9	32	0:09:32	RBNU	M	1
11	10:29	10	36	0:10:36	RBNU	M	1
3	10:41	22	38	0:22:38	RBNU		1
11	10:32	13	49	0:13:49	RBNU		1
3	10:40	21	33	0:21:33	RBNU		1
5	10:22	1	22	0:01:22	start		
11	10:27	8	43	0:08:43	start		
3	10:35	16	21	0:16:21	start		
3	10:42	23	56	0:23:56	stop		
	10:21	0	0	0:00:00			
3		19	33	0:19:33	BCCH	M	1
3		17	40	0:17:40	BCCH	M	1
11		3	0	0:03:00	MOCH	U	1
5		9	1	0:09:01	MOCH		2
5		9	13	0:09:13	MOCH	U	2
11		0	42	0:00:42	MOCH	U	1
11		3	6	0:03:06	MOCH	U	1
5		13	36	0:13:36	MOCH	U	2
3		15	5	0:15:05	MOCH	U	2
3		22	33	0:22:33	MOCH	U	2
5		8	43	0:08:43	MOCH	U	2
3		23	7	0:23:07	MOCH	U	2
5		9	23	0:19:23	MOCH	U	2
11		1	3	0:01:03	MOCH	U	1
11		2	0	0:02:00	MOCH	U	1
5		7	40	0:07:40	MOCH	U	1
5		9	33	0:09:33	MOCH	U	2
5		9	55	0:09:55	MOCH	U	2
5		10	20	0:10:20	MOCH	M	2
3		15	20	0:15:20	MOCH	U	2
11		1	30	0:01:30	RBNU	U	1
11		3	43	0:03:43	RBNU	U	1
11		0	0	0:00:00	START		
5		7	14	0:07:14	START		
3		14	51	0:14:51	START		
3		10	25	0:10:25	BCCH	U	1
5		17	35	0:17:35	CHIC	U	1
5		19	45	0:19:45	MOCH	U	2

5		19	27	0:19:27	MOCH	U	2
5		18	50	0:18:50	MOCH	U	2
5		19	0	0:19:00	MOCH	U	2
5		18	33	0:18:33	MOCH	U	2
5		20	10	0:20:10	MOCH	U	2
5		20	45	0:20:45	MOCH	U	2
5		17	46	0:17:46	MOCH	U	2
5		20	46	0:20:46	MOCH	U	2
11		4	15	0:04:15	MOCH	U	1
3		9	2	0:09:02	MOCH	U	1
3		9	30	0:09:30	MOCH	U	2
3		11	45	0:11:45	MOCH	F	2
3		10	6	0:10:06	MOCH	U	2
3		12	15	0:12:15	MOCH	M	2
5		22	10	0:22:10	MOCH	U	2
3		8	2	0:08:02	MOCH	U	1
11		4	8	0:04:08	MOCH	U	1
5		17	9	0:17:09	MOCH	U	2
5		16	57	0:16:57	MOCH	U	2
3		14	4	0:14:04	MOCH	U	2
5		22	28	0:22:28	MOCH	U	2
11		3	10	0:03:10	RBNU	M	1
11		5	27	0:05:27	RBNU	M	2
11		6	28	0:06:28	RBNU	M	2
11		6	41	0:06:41	RBNU	F	2
11		7	17	0:07:17	RBNU	F	2
11		3	30	0:03:30	RBNU	F	2
3		8	38	0:08:38	RBNU	U	2
3		9	55	0:09:55	RBNU	U	2
3		10	57	0:10:57	RBNU	U	1
3		10	44	0:10:44	RBNU	U	1
5		15	30	0:15:30	RBNU	U	1
11		0	37	0:37:00	START		
3		8	0	0:08:00	START		
5		15	30	0:15:30	START		
1	8:39	9	40	0:09:40	MOCH		1
13	8:34	4	49	0:04:49	MOCH	U	1
5	8:46	16	17	0:16:17	MOCH		1
13	8:35	5	20	0:05:20	MOCH	U	1
13	8:33	3	5	0:03:05	RBNU	M	1
13	8:32	2	23	0:02:23	RBNU	M	1
13	8:32	2	47	0:02:47	RBNU	M	1
13	8:33	3	42	0:03:42	RBNU	M	1
13	8:34	4	4	0:04:04	RBNU	M	1

13	8:33	3	29	0:03:29	RBNU	M	1
13	8:34	4	26	0:04:26	RBNU	M	1
13	8:34	4	45	0:04:45	RBNU	M	1
1	8:40	10	16	0:10:16	RBNU	M	1
1	8:41	11	28	0:11:28	RBNU	M	1
5	8:42	12	17	0:12:17	RBNU	M	1
5	8:46	16	50	0:16:50	RBNU	M	1
13	8:36	6	58	0:06:58	RBNU	M	1
1	8:38	8	0	0:08:00	START		
5	8:42	12	0	0:12:00	START		
13	8:30	0	0	0:00:00	START		
13	8:31	1	2	0:01:02			1
	12:26:35				MOCH	U	1
	12:26:20				MOCH	U	1
	12:32:50				RBNU	F	2
	12:32:15				RBNU	M	1
	12:32:50				RBNU	M	2
	12:33:20				RBNU	F	2
	12:33:38				RBNU	F	2
	12:33:38				RBNU	M	2
	12:34:00				RBNU	F	2
	12:34:01				RBNU	F	2
	12:34:55				RBNU	F	2
	12:31:50				RBNU	M	1
	12:32:10				RBNU	M	1
	12:33:50				RBNU	M	2
	12:31:40				RBNU	M	1
	12:35:22				RBNU	M	2
	12:37:00				RBNU	M	1
	11:41:50			29:50:00	MOCH	U	1
	11:14:40			2:40	RBNU	M	1
	11:12:24			0:24	RBNU	M	1
	11:12:58			0:58	RBNU	M	1
	11:13:20			1:20	RBNU	M	1
	11:12:42			0:42	RBNU	M	1
	11:12:05			0:05	RBNU	M	1
	11:15:50			3:50	RBNU	M	1
	11:16:40			4:40	RBNU	M	1
	11:40:50			28:50:00	RBNU	M	1
	11:25:40			13:40	RBNU	M	1
	11:35:30			23:30	RBNU	M	1
	11:26:40			14:40	RBNU	M	1
	11:33:50			21:50	RBNU	M	1
	11:28:30			16:30	RBNU	M	1

11:35:00	23:00	TAHU	U	1
9:44:00	24:00:00	MOCH	U	1
9:21:40	0:01:40	RBNU	M	2
9:21:40	0:01:40	RBNU	F	2
9:23:20	3:20	RBNU	M	2
9:23:55	3:55	RBNU	M	2
9:24:20	4:20	RBNU	M	2
9:39:50	19:50	RBNU	M	2
9:39:50	19:50	RBNU	F	2
9:46:10	26:10:00	RBNU	M	2
9:46:10	26:10:00	RBNU	F	2
9:48:10	28:10:00	RBNU	M	2
9:35:30	15:30	WAVI	U	1
10:31:20	6:20	BCCH	U	1
10:43:50	18:50	RBNU	U	1
8:22:50	22:50	BCCH	M	1
8:22:30	22:30	BCCH	M	1
8:23:45	23:45	BCCH	M	1
8:27:10	27:10:00	BCCH	M	1
8:21:15	21:15	BCCH	M	1
8:25:00	25:00:00	BCCH	M	1
8:14:50	14:50	RBNU	M	1
8:15:00	15:00	RBNU	M	1
8:14:10	14:10	RBNU	M	1
8:13:50	13:50	RBNU	M	1
8:14:20	14:20	RBNU	M	1
8:14:25	14:25	RBNU	M	1
8:21:50	21:50	RBNU	M	2
8:21:50	21:50	RBNU	F	2
8:23:10	23:10	RBNU	M	2
8:27:10	27:10:00	RBNU	M	2
8:27:10	27:10:00	RBNU	F	2
8:15:40	15:40	RBNU	M	1
8:16:00	16:00	RBNU	M	1
8:18:00	18:00	RBNU	M	1
8:23:50	23:50	RBNU	M	1
8:22:50	22:50	RBNU	M	2
8:18:10	18:10	RBNU	M	1
8:24:40	24:40:00	RBNU	M	1
8:18:20	18:20	RBNU	M	1
8:27:00	27:00:00	RBNU	M	2
8:20:00	20:00	RBNU	M	2
8:20:00	20:00	RBNU	F	2
8:10:00	10:00	RBNU	U	1

8:10:30			10:30	BCCH	U	2
8:10:30			10:30	BCCH	U	2
8:07:20			7:20	BCCH	U	2
8:08:00			8:00	BCCH	U	1
8:09:15			9:15	BCCH	U	1
8:10:00			10:00	BCCH	U	2
8:10:00			10:00	BCCH	U	2
8:07:00			7:00	BCCH	U	2
8:07:00			7:00	BCCH	U	2
8:06:00			6:00	BCCH	U	1
8:11:30			11:30	BCCH	U	2
8:11:30			11:30	BCCH	U	2
8:25:20			25:20:00	RBNU	M	1
8:10:40			10:40	RBNU	M	1
8:10:50			10:50	RBNU	M	1
8:24:40			24:40:00	RBNU	M	1
10:20:00			20:00	RBNU	M	1
8:11:30			11:30	RBNU	M	1
8:15:00			15:00	RBNU	M	1
8:22:00			22:00	RBNU	M	1
8:06:00			6:00	RBNU	U	1
8:26:00			26:00:00	RBNU	M	1
9:32:20	2	20	2:20	MOCH	U	1
9:42:00	12	0	12:00	MOCH	U	1
9:32:30	2	30	2:30	RBNU	U	1
9:46:00	16	0	16:00	RBNU	U	1
9:39:50	9	50	9:50	RBNU	U	1
9:55:20	25	20	25:20:00	RBNU	M	1
9:57:00	27	0	27:00:00	RBNU	U	1
9:03:10				RBNU	M	2
9:04:20				RBNU	M	1
9:03:50				RBNU	M	2
8:47:40				RBNU	M	1
8:53:00				RBNU	U	1
8:57:20				RBNU	M	1
8:59:19				RBNU	M	1
9:00:30				RBNU	F	1
9:01:45				RBNU	M	1
9:02:46				RBNU	F	1
9:05:20				RBNU	M	1
8:56:55				RBNU	U	1
9:03:10				RBNU	F	2
9:03:50				RBNU	F	2
9:46:55				MOCH	U	1

9:33:00	MOCH	U	1
9:36:40	RBNU	M	1
9:36:30	RBNU	M	1
9:25:57	RBNU	M	1
9:29:00	RBNU	U	1
9:34:30	RBNU	M	1
9:37:50	RBNU	F	2
9:38:20	RBNU	F	1
9:40:20	RBNU	U	1
9:41:50	RBNU	U	1
9:34:00	RBNU	U	1
9:25:20	RBNU	M	1
9:33:00	RBNU	U	1
9:37:50	RBNU	M	2
7:51:15	BCCH	M	1
7:53:06	BCCH	M	1
7:36:00	BCCH	U	2
7:36:00	BCCH	U	2
8:00:00	BCCH	U	1
7:44:36	BCCH	U	2
7:50:10	BCCH	M	1
7:50:30	BCCH	M	1
7:40:20	MOCH	U	1
7:36:30	RBNU	M	2
7:36:30	RBNU	F	2
7:47:00	RBNU	M	2
7:47:00	RBNU	F	2
7:49:00	RBNU	M	2
7:49:00	RBNU	F	2
7:52:07	RBNU	M	2
7:52:07	RBNU	F	2
7:53:30	RBNU	M	2
7:53:30	RBNU	F	2
7:51:10	RBNU	M	1
7:51:20	RBNU	M	1
7:52:30	RBNU	M	1
7:38:40	RBNU	U	1
8:03:00	RNSA	U	1
7:56:40	TAHU	U	1
10:13:50	BCCH	U	1
10:11:40	BCCH	M	1
10:10:40	BCCH	M	1
10:13:20	BCCH	U	1
9:47:05	RBNU	F	2

9:48:15		RBNU	M		2
9:51:30		RBNU	F		2
9:51:31		RBNU	M		2
9:54:20		RBNU	F		2
9:55:40		RBNU	F		2
9:56:00		RBNU	F		2
9:56:45		RBNU	M		2
10:01:00		RBNU	U		2
10:01:30		RBNU	U		2
10:10:20		RBNU	M		1
10:14:50		RBNU	M		1
10:02:30		RBNU	M		2
10:11:50		RBNU	M		1
10:01:10		RBNU	U		2
10:02:10		RBNU	U		2
9:51:00		RBNU	U		3
7:12:45		RBNU	M		1
7:25:30		RBNU	F		1
7:26:40		RBNU	M		1
7:29:30		RBNU	U		1
7:39:00		RBNU	M		1
7:22:30		RBNU	M		1
7:25:00		RBNU	M		1
7:28:00		RBNU	M		1
7:30:20		RBNU	M		1
7:31:00		RBNU	U		2
7:29:10		RBNU	U		1
7:11:01		TAHU	U		1
7:36:30		TAHU	U		1
#VALUE!	15:12	MOCH	M	AGR	2
#VALUE!	15:20	MOCH	F	GAPO	2
#VALUE!	20:20	MOCH	M	AGR	2
#VALUE!	20:50	MOCH	M	AGR	2
#VALUE!	20:25	MOCH	M	AGR	2
8:24:40	1:40	MOCH			2
#VALUE!	16:15	MOCH	F	GAPO	2
8:24:59	1:59	MOCH	F	GAPO	2
#VALUE!	22:50	MOCH	B		2
#VALUE!	7:00	MOCH	M	AGR	2
#VALUE!	19:32	MOCH	M	AGR	2
8:23:00	0:00	MOCH	B		2
#VALUE!	6:00	MOCH	M	AGR	2
#VALUE!	14:50	MOCH	U		2
#VALUE!	8:00	MOCH	B		2

14:50:10	17:10	MOCH	U	2
14:55:21	22:21	MOCH	U	2
14:50:10	17:10	MOCH	U	2
14:55:21	22:21	MOCH	U	2
14:48:30	15:30	MOCH	U	2
14:48:30	15:30	MOCH	U	2
14:58:50	25:50:00	MOCH	F	2
14:58:50	25:50:00	MOCH	M	2
14:58:50	25:50:00	MOCH	F	2
14:58:50	25:50:00	MOCH	M	2
14:48:00	15:00	MOCH	U	2
14:48:00	15:00	MOCH	U	2
14:40:30	7:30	RBNU	M	2
14:40:30	7:30	RBNU	M	2
14:39:50	6:50	RBNU	M	2
14:56:50	23:50	RBNU	M	2
14:39:50	6:50	RBNU	M	2
14:56:50	23:50	RBNU	M	2
14:33:00	0:00	RBNU	F	1
14:47:40	14:40	RBNU	M	2
14:57:20	24:20:00	RBNU	M	2
14:33:00	0:00	RBNU	F	1
14:47:40	14:40	RBNU	M	2
14:57:20	24:20:00	RBNU	M	2
14:39:30	6:30	RBNU	M	2
14:48:18	15:18	RBNU	M	2
14:50:30	17:30	RBNU	M	2
14:55:21	22:21	RBNU	M	2
14:39:30	6:30	RBNU	M	2
14:48:18	15:18	RBNU	M	2
14:50:30	17:30	RBNU	M	2
14:55:21	22:21	RBNU	M	2
14:45:00	12:00	RBNU	M	2
14:46:50	13:50	RBNU	M	2
15:00:30	27:30:00	RBNU	M	2
15:00:50	27:50:00	RBNU	M	2
14:45:00	12:00	RBNU	M	2
14:46:50	13:50	RBNU	M	2
15:00:30	27:30:00	RBNU	M	2
15:00:50	27:50:00	RBNU	M	2
14:38:50	5:50	RBNU	M	2
14:38:50	5:50	RBNU	M	2
14:52:10	19:10	RBNU	M	2
14:52:10	19:10	RBNU	M	2

8:12:00	24:00:00	BCCH	M	1
8:10:45	22:45	BCCH	M	1
7:50:00	2:00	BCCH	M	1
7:55:00	7:00	MOCH	U	1
7:58:00	10:00	MOCH	M	1
8:02:40	14:40	MOCH	M	1
7:48:20	0:20	MOCH	M	1
8:03:20	15:20	MOCH	M	1
7:54:48	6:48	RBNU	M	1
7:55:28	7:28	RBNU	M	1
8:09:40	21:40	RBNU	M	1
8:10:30	22:30	RBNU	M	1
8:05:00	17:00	RBNU	M	1
8:09:00	21:00	RBNU	M	1
8:02:00	14:00	RBNU	M	1
7:53:30	5:30	RBNU	M	1
8:05:25	17:25	RBNU	M	1
8:08:10	20:10	RBNU	M	1
8:11:00	23:00	RBNU	M	1
8:00:40	12:40	RBNU	M	1
7:58:20	10:20	RBNU	M	1
7:48:00	0:00	RBNU	M	1
8:03:12	15:12	RBNU	M	1
8:54:00	8:00	MOCH	U	1
9:03:45	17:45	MOCH	U	1
9:04:30	18:30	MOCH	U	1
8:46:35	0:35	RBNU	M	2
8:46:25	0:25	RBNU	M	2
8:47:00	1:00	RBNU	M	2
8:48:00	2:00	RBNU	M	2
8:50:00	4:00	RBNU	F	2
8:55:30	9:30	RBNU	M	2
9:02:00	16:00	RBNU	M	2
8:51:00	5:00	RBNU	M	2
8:54:00	8:00	RBNU	M	2
9:04:00	18:00	RBNU	M	1
9:01:40	15:40	RBNU	M	2
12:06:00	22:00	BCCH	M	1
12:07:11	23:11	BCCH	M	1
11:56:50	12:50	RBNU	M	2
12:04:30	20:30	RBNU	M	2
12:03:01	19:01	RBNU	M	2
11:45:30	1:30	RBNU	M	2
12:27:00	14:00	RBNU	U	1

12:26:51	13:51	RBNU	U	1
12:35:45	22:45	RBNU	U	1
12:24:15	4:15	MOCH	M	1
12:26:00	6:00	MOCH	M	1
12:32:00	12:00	RBNU	M	2
12:37:20	17:20	RBNU	M	2
12:25:00	5:00	RBNU	M	2
12:38:00	18:00	RBNU	M	2
12:30:30	10:30	RBNU	M	2
12:34:55	14:55	RBNU	M	2
12:41:50	21:50	RBNU	M	2
15:59:47	16:47	MOCH	M	1
15:57:36	14:36	MOCH	M	1
16:00:16	17:16	MOCH	M	1
15:56:25	13:25	MOCH	M	1
16:05:40	22:40	MOCH	M	1
16:06:00	23:00	MOCH	U	2
15:52:45	0:09:45	MOCH	M	1
16:04:10	21:10	RBNU	M	2
16:02:55	19:55	RBNU	M	2
16:05:00	22:00	RBNU	M	2
16:05:30	22:30	RBNU	M	2
16:04:50	21:50	RBNU	F	2
15:43:50	0:50	RBNU	M	2
16:04:00	21:00	RBNU	M	2
15:45:28	2:28	RBNU	M	2
15:55:50	12:50	RBNU	M	2
16:35:30	12:30	MOCH	M	1
16:43:20	20:20	RBNU	M	2
16:42:50	19:50	RBNU	M	2
16:30:30	7:30	RBNU	M	2
16:42:15	19:15	RBNU	M	2
16:29:10	6:10	RBNU	M	2
16:24:50	1:50	RBNU	M	2
10:56:27	12:27	MOCH	M	2
10:57:48	13:48	MOCH	M	2
11:06:30	22:30	MOCH	U	2
11:08:20	24:20:00	MOCH	U	2
10:45:30	1:30	MOCH	U	2
10:50:55	6:55	MOCH	U	2
10:51:27	7:27	MOCH	M	2
10:53:05	9:05	MOCH	M	2
10:45:10	1:10	MOCH	U	2
10:47:30	3:30	MOCH	U	2

10:51:56	7:56	MOCH	U	2
11:00:35	16:35	MOCH	U	2
11:04:49	20:49	RBNU	M	1
11:07:00	23:00	RBNU	M	1
11:09:40	25:40:00	RBNU	M	1
11:10:45	26:45:00	RBNU	M	1
10:51:58	7:58	RBNU	M	1
10:59:20	15:20	RBNU	M	1
10:57:14	13:14	RBNU	M	1
11:13:30	13:30	BCCH	U	1
11:06:10	6:10	RBNU	M	2
11:08:25	8:25	RBNU	M	2
11:08:40	8:40	RBNU	M	2
11:05:20	5:20	RBNU	U	2
11:03:45	3:45	RBNU	U	2
11:11:20	11:20	RBNU	M	2
9:29:01	18:01	RBNU	M	2
9:17:10	6:10	RBNU	M	2
9:17:58	6:58	RBNU	M	2
9:21:05	10:05	RBNU	M	2
9:22:50	11:50	RBNU	M	2
9:31:10	20:10	RBNU	M	2
9:32:39	21:39	RBNU	M	2
9:28:15	17:15	RBNU	M	2
9:33:36	22:36	RBNU	M	2
8:55:00	19:00	MOCH	M	2
8:48:40	12:40	MOCH	M	2
8:49:00	13:00	MOCH	M	2
8:54:55	18:55	MOCH	M	2
8:55:30	19:30	MOCH	U	2
8:57:10	21:10	MOCH	M	2
9:01:50	25:50:00	MOCH	M	2
8:37:35	1:35	MOCH	U	2
9:02:50	26:50:00	RBNU	M	2
9:02:40	26:40:00	RBNU	M	2
8:42:00	6:00	RBNU	M	2
8:43:00	7:00	RBNU	M	2
9:01:40	25:40:00	RBNU	M	2
9:04:00	28:00:00	RBNU	M	2
9:04:20	28:20:00	RBNU	F	2
9:05:30	29:30:00	RBNU	F	2
8:40:29	4:29	RBNU	M	2
9:02:10	26:10:00	RBNU	M	2
8:39:47	3:47	RBNU	U	2

	8:46:53			10:53	RBNU	M	2
	8:49:30			13:30	RBNU	M	2
				0:00:00	RBNU	U	1
5				0:07:40	BCCH	U	2
5				0:07:30	RBNU	U	1
11				0:12:33	RBNU	M	2
11				0:14:10	RBNU	M	2
11				0:13:02	RBNU	M	2
11				0:12:40	RBNU	M	2
11				0:16:30	RBNU	M	2
1				0:02:30	RBNU	U	1
11				0:02:00	RBNU	M	2
5				0:08:45	RBNU	M	2
5				0:10:00	RBNU	M	2
1				0:15:30	RBNU	F	2
1				0:16:30	RBNU	M	2
1				0:19:00	RBNU	M	2
1				0:19:40	RBNU	M	2
11				0:01:40	RBNU	M	2
11				0:00:30	RBNU	M	2
11				0:00:45	RBNU	M	2
11				0:06:30	RBNU	M	2
11				0:06:30	RBNU	F	2
11				0:00:25	START		
5				0:07:30	START		
1				0:14:38	START		
5		1	0	0:01:00	MOCH	M	1
1		0	10	0:00:10	MOCH	M	2
11		1	19	0:01:19	MOCH	M	2
5		2	0	0:02:00	MOCH	M	1
1		0	5	0:00:05	MOCH	M	2
5		3	0	0:03:00	MOCH	M	1
1		1	50	0:01:50	MOCH	M	2
1		2	0	0:02:00	MOCH	F	2
1		2	30	0:02:30	MOCH	M	2
1		4	40	0:04:40	MOCH	F	2
11		2	20	0:02:20	MOCH	M	2
11		2	30	0:02:30	MOCH	F	2
11		3	0	0:03:00	MOCH	F	2
11		3	30	0:03:30	MOCH	M	2
11		4	0	0:04:00	MOCH	M	2
11		6	30	0:06:30	MOCH	F	2
5		0	40	0:00:40	MOCH	M	1
1		1	30	0:01:30	MOCH	F	2

1	3	20	0:03:20	MOCH	M	2
5	0	0	0:00:00	MOCH	M	1
5	0	10	0:00:10	MOCH	M	1
11	1	15	0:01:15	MOCH	F	2
11	1	50	0:01:50	MOCH	M	2
5	3	30	0:03:30	MOCH	M	1
1	5	30	0:05:30	MOCH	M	2
5	4	0	0:04:00	MOCH	M	1
1	7	0	0:07:00	MOCH	M	2
11	0	50	0:00:50	MOCH	M	2
1	10	9	0:10:09	START		
11	10	21	0:10:21	START		
5	11	30	0:11:30	MOCH	M	4
5	11	30	0:11:30	MOCH	F	4
1	17	59	0:17:59	MOCH	?	2
5	8	12	0:08:12	MOCH	M	1
5	8	30	0:08:30	MOCH	M	1
5	9	31	0:09:31	MOCH	M	1
5	8	52	0:08:52	MOCH	M	1
1	17	10	0:17:10	MOCH	F	2
1	17	38	0:17:38	MOCH	F	2
5	13	4	0:13:04	MOCH	M	4
5	13	19	0:13:19	MOCH	F	4
5	14	31	0:14:31	MOCH	M	2
1	16	45	0:16:45	MOCH	M	1
5	15	29	0:15:29	MOCH	M	2
1	18	28	0:18:28	MOCH	?	2
1	20	6	0:20:06	MOCH	M	2
1	20	41	0:20:41	MOCH	F	3
13	4	48	0:04:48	RBNU	?	1
13	1	22	0:01:22	RBNU	M	3
13	0	53	0:00:53	RBNU	M	3
5	11	30	0:11:30	RBNU	M	4
5	11	30	0:11:30	RBNU	F	4
13	1	2	0:01:02	RBNU	M	3
5	11	55	0:11:55	RBNU	M	4
13	0	31	0:00:31	RBNU	M	3
13	1	56	0:01:56	RBNU	M	3
13	2	12	0:02:12	RBNU	M	3
13	0	10	0:00:10	RBNU	M	3
13	2	51	0:02:51	RBNU	M	3
13	3	20	0:03:20	RBNU	M	3
5	12	25	0:12:25	RBNU	M	4
5	8	0	0:08:00	RBNU	?	1

5	13	47	0:13:47	RBNU	M	3
5	14	12	0:14:12	RBNU	M	3
5	15	29	0:15:29	RBNU	M	2
1	18	28	0:18:28	RBNU	?	2
13	5	5	0:05:05	RBNU	?	1
13	0	0	0:00:00	start		
5	8	0	0:08:00	start		
1	16	30	0:16:30	start		
1	23	20	0:23:20	BCCH	M	1
1	24	52	0:24:52	BCCH	M	2
1	26	4	0:26:04	BCCH	M	2
1	26	4	0:26:04	BCCH	M	2
1	23	9	0:23:09	BCCH	M	1
1	25	13	0:25:13	BCCH	M	2
1	29	5	0:29:05	BCCH	M	2
1	29	44	0:29:44	BCCH	M	2
1	24	2	0:24:02	BCCH	M	2
13	13	27	0:13:27	MOCH	F	1
1	30	59	0:30:59	MOCH	M	2
5	2	24	0:02:24	MOCH	M	2
13	10	38	0:10:38	MOCH	?	1
5	2	40	0:02:40	MOCH	M	2
5	3	9	0:03:09	MOCH	M	2
13	10	30	0:10:30	MOCH	?	2
5	3	48	0:03:48	MOCH	M	2
5	3	57	0:03:57	MOCH	M	2
1	23	38	0:23:38	MOCH	M	1
5	1	59	0:01:59	MOCH	M	2
13	10	20	0:10:20	MOCH	M	1
13	9	52	0:09:52	RBNU	M	3
13	16	14	0:16:14	RBNU	F	2
13	15	3	0:15:03	RBNU	F	2
13	10	14	0:10:14	RBNU	M	1
13	9	52	1:09:52	RBNU	?	3
13	9	52	2:09:52	RBNU	?	3
13	16	30	0:16:30	RBNU	F	2
13	8	3	0:08:03	RBNU	M	1
13	9	42	0:09:42	RBNU	M	1
13	14	43	0:14:43	RBNU	M	2
13	18	25	0:18:25	RBNU	M	3
13	18	25	0:18:25	RBNU	M	3
13	18	25	0:18:25	RBNU	F	3
1	22	47	0:22:47	RBNU	M	1
5	5	42	0:05:42	RBNU	M	2

5		7	20	0:07:20	RBNU	M	2
5		1	22	0:01:22	RBNU	M	1
5		2	52	0:02:52	RBNU	M	2
5		0	0	0:00:00	start		
13		7	30	0:07:30	start		
1		21	0	0:21:00	start		
5	11:14	4	13	0:04:13	MOCH	M	1
13	11:31	21	9	0:21:09	RBNU	M	2
13	11:27	17	52	0:17:52	RBNU	M	1
13	11:28	18	47	0:18:47	RBNU	M	1
1	11:20	10	1	0:10:01	RBNU	M	1
13	11:29	19	7	0:19:07	RBNU	M	2
	11:10	0	0	0:00:00	RBNU	F?	2
	11:10	0	0	0:00:00	RBNU	M	2
13	11:29	19	33	0:19:33	RBNU	F?	3
13	11:31	21	22	0:21:22	RBNU		2
	11:10	0	0	0:00:00	START		
1	11:17	7	28	0:07:28	START		
13	11:25	15	0	0:15:00	START		
13	11:32	22	41	0:22:41	STOP?		
5	10:22	16	27	0:16:27	MOCH	M	1
5	10:23	17	30	0:17:30	MOCH	M	1
5	10:23	17	58	0:17:58	MOCH	M	1
5	10:24	18	3	0:18:03	MOCH	M	1
5	10:24	18	7	0:18:07	MOCH	M	2
5	10:21	15	48	0:15:48	MOCH	M	1
5	10:26	20	0	0:20:00	MOCH	M, F	2
1	10:06	0	42	0:00:42	START		
13	10:13	7	28	0:07:28	START		
5	10:21	15	0	0:15:00	START		
5	10:30	24	0	0:24:00	STOP		
	10:06	0	0	0:00:00			
14	9:11	21	0	0:21:00	END		
5	8:58	8	45	0:08:45	MOCH	M	1
14	9:04	14	50	0:14:50	MOCH		1
1	8:55	5	15	0:05:15	MOCH	M	1
5	8:56	6	32	0:06:32	MOCH	M	1
14	9:06	16	30	0:16:30	MOCH	M	1
14	9:05	15	20	0:15:20	RBNU	M	2
14	9:06	16	40	0:16:40	RBNU	M	3
14	9:06	16	10	0:16:10	RBNU	M	3
1	8:52	2	58	0:02:58	RBNU	M	2
1	8:53	3	20	0:03:20	RBNU	M	2
5	8:57	7	0	0:07:00	RBNU	M	2

5	8:57	7	50	0:07:50	RBNU	M	2
14	9:04	14	30	0:14:30	RBNU	M	2
14	9:06	16	40	0:16:40	RBNU	M	3
14	9:10	20	0	0:20:00	RBNU	F	2
14	9:08	18	0	0:18:00	RBNU	M	3
5	9:00	10	50	0:10:50	RBNU	M	2
	8:50	0	45	0:00:45	RBNU	M	2
1	8:53	3	30	0:03:30	RBNU	M	2
14	9:06	16	20	0:16:20	RBNU	M	3
1	8:52	2	0	0:02:00	START		
5	8:56	6	30	0:06:30	START		
14	9:04	14	0	0:14:00	START		
	8:50	0	0	0:00:00	START		
5	8:08	7	11	0:07:11	MOCH	M	1
5	8:09	8	10	0:08:10	MOCH	M	1
1	8:19	18	26	0:18:26	MOCH	U	1
5	8:07	6	52	0:06:52	MOCH	M	1
5	8:10	9	17	0:09:17	RBNU	F	1
13	8:01	0	23	0:00:23	RBNU	M	1
1	8:16	15	44	0:15:44	RBNU		2
1	8:17	16	51	0:16:51	RBNU	M	2
1	8:17	16	51	0:16:51	RBNU	F	2
5	8:15	14	35	0:14:35	RBNU	F	1
13	8:05	4	15	0:04:15	RBNU	M	1
1	8:18	17	28	0:17:28	RBNU	M	2
1	8:18	17	28	0:17:28	RBNU	F	2
1	8:16	15	30	0:15:30	START		
5	8:07	6	0	0:06:00	START		
13	8:01	0	0	0:00:00	START		
5	8:09	8	40	0:08:40			
5	13:31	5	45	0:05:45	MOCH		1
5	13:31	5	25	0:05:25	MOCH		1
3	13:38	12	42	0:12:42	MOCH	M	1
3	13:40	14	20	0:14:20	MOCH	M	1
5	13:36	10	30	0:10:30	MOCH	M	1
5	13:30	4	10	0:04:10	MOCH	M	1
11	13:43	17	40	0:17:40	RBNU	M	1
	13:26	0	27	0:00:27	RBNU		1
3	13:41	15	50	0:15:50	RBNU	M	1
11	13:47	21	20	0:21:20	RBNU	M	1
5	13:32	6	0	0:06:00	RBNU	M	1
5	13:32	6	30	0:06:30	RBNU	M	1
5	13:35	9	0	0:09:00	RBNU	M	1
3	13:40	14	35	0:14:35	RBNU	M	1

3	13:38	12	30	0:12:30	RBNU	M	1
5	13:29	3	10	0:03:10	start		
3	13:36	10	48	0:10:48	start		
11	13:43	17	30	0:17:30	start		
11	13:48	22	0	0:22:00	stop		
	13:26	0	0	0:00:00			
3	10:10	9	25	0:09:25	BCCH		1
5	10:17	16	44	0:16:44	MOCH		1
5	10:18	17	6	0:17:06	MOCH		3
5	10:19	18	2	0:18:02	MOCH		1
3	10:11	10	29	0:10:29	RBNU		2
5	10:19	18	9	0:18:09	RBNU		2
	10:01	0	0	0:00:00	RBNU		2
	10:01	0	0	0:00:00	RBNU		2
	10:01	0	0	0:00:00	RBNU		2
3	10:11	10	45	0:10:45	RBNU		2
5	10:20	19	5	0:19:05	RBNU		2
5	10:23	22	22	0:22:22	RBNU		2
5	10:23	22	31	0:22:31	RBNU		2
11	10:02	1	30	0:01:30	RBNU		2
3	10:13	12	6	0:12:06	RBNU		1
3	10:16	15	57	0:15:57	RBNU		1
5	10:17	16	26	0:16:26	RBNU		1
5	10:18	17	36	0:17:36	RBNU		1
5	10:21	20	19	0:20:19	RBNU		2
5	10:25	23	10	0:23:10	RBNU		3
5	10:20	19	45	0:19:45	RBNU	M	2
5	10:20	19	45	0:19:45	RBNU		2
3	10:13	12	39	0:12:39	RBNU		1
3	10:10	9	59	0:09:59	RBNU		2
3	10:12	11	1	0:11:01	RBNU		2
5	10:23	22	3	0:22:03	RBNU		2
	10:01	0	0	0:00:00	RNSA		2
11	10:02	1	12	0:01:12	start		
3	10:09	8	35	0:08:35	start		
5	10:17	16	14	0:16:14	start		
5	10:25	23	51	0:23:51	stop		
3	10:12	11	46	0:11:46	TRES		1
	10:01	0	0	0:00:00			
3	11:17	17	45	0:17:45	BCCH	M	1
3	11:16	16	41	0:16:41	BCCH	M	1
3	11:21	21	40	0:21:40	BCCH	M	1
11	11:03	3	16	0:03:16	RBNU		2
11	11:03	3	10	0:03:10	RBNU		2

11	11:03	3	14	0:03:14	RBNU		2
11	11:03	3	4	0:03:04	RBNU		2
11	11:06	6	38	0:06:38	RBNU		2
5	11:13	13	40	0:13:40	RBNU		2
5	11:13	13	41	0:13:41	RBNU		2
11	11:06	6	24	0:06:24	RBNU		2
5	11:10	10	34	0:10:34	RBNU		2
5	11:10	10	53	0:10:53	RBNU		2
11	11:02	2	4	0:02:04	RBNU		2
5	11:10	10	59	0:10:59	RBNU		2
5	11:11	11	11	0:11:11	RBNU		2
11	11:08	8	9	0:08:09	RBNU		3
3	11:17	17	30	0:17:30	RBNU		2
11	11:02	2	10	0:02:10	RBNU		2
11	11:02	2	40	0:02:40	RBNU		2
5	11:11	11	25	0:11:25	RBNU		2
5	11:11	11	35	0:11:35	RBNU		2
5	11:13	13	31	0:13:31	RBNU		2
5	11:14	14	31	0:14:31	RBNU		2
3	11:16	16	15	0:16:15	RBNU		2
11	11:02	2	28	0:02:28	RBNU		2
11	11:04	4	33	0:04:33	RBNU		2
5	11:11	11	45	0:11:45	RBNU		2
3	11:16	16	35	0:16:35	RBNU		2
3	11:18	18	13	0:18:13	RBNU		2
11	11:02	2	56	0:02:56	RBNU		2
5	11:11	11	47	0:11:47	RBNU		2
5	11:12	12	16	0:12:16	RBNU		2
5	11:12	12	27	0:12:27	RBNU		2
3	11:20	20	34	0:20:34	RBNU		2
5	11:12	12	41	0:12:41	RBNU		2
5	11:14	14	40	0:14:40	RBNU		2
3	11:22	22	6	0:22:06	RBNU		1
3	11:19	19	48	0:19:48	RBNU		2
3	11:20	20	46	0:20:46	RBNU		3
11	11:07	7	1	0:07:01	RBNU		3
11	11:07	7	44	0:07:44	RBNU		3
11	11:01	1	29	0:01:29	start		
5	11:09	9	19	0:09:19	start		
3	11:15	15	58	0:15:58	start		
3	11:22	22	57	0:22:57	stop		
	11:00	0	0	0:00:00			
3	9:58	5	41	0:05:41	BCCH	M	1
3	10:00	7	10	0:07:10	BCCH	M	1

5	10:04	11	27	0:11:27	RBNU		1
5	10:04	11	14	0:11:14	RBNU		1
5	10:04	11	20	0:11:20	RBNU		1
5	10:03	10	0	0:10:00	RBNU		2
5	10:03	10	30	0:10:30	RBNU		2
5	10:04	11	10	0:11:10	RBNU		2
11	10:06	14	20	0:14:20	RBNU		1
5	10:04	11	50	0:11:50	RBNU		1
11	10:07	15	30	0:15:30	RBNU		1
3	9:58	5	55	0:05:55	RBNU		1
3	9:55	2	41	0:02:41	start		
5	10:02	9	7	0:09:07	start		
11	10:05	13	45	0:13:45	start		
11	10:11	19	12	0:19:12	stop		
	9:53	0	0	0:00:00			
11	10:21	15	0	0:15:00	CHMK		1
11	10:18	12	59	0:12:59	RBNU		1
11	10:19	13	36	0:13:36	RBNU		1
11	10:18	12	6	0:12:06	RBNU		1
11	10:18	12	8	0:12:08	RBNU		1
11	10:18	12	24	0:12:24	RBNU		1
11	10:19	13	13	0:13:13	RBNU		1
11	10:18	12	43	0:12:43	RBNU		1
11	10:17	11	56	0:11:56	RBNU		1
11	10:19	13	22	0:13:22	RBNU		1
11	10:16	10	50	0:10:50	RBNU		1
11	10:17	11	18	0:11:18	RBNU		1
11	10:17	11	20	0:11:20	RBNU		1
11	10:20	14	24	0:14:24	RBNU		2
11	10:21	15	32	0:15:32	RBNU		1
5	10:13	7	23	0:07:23	RBNU		1
5	10:14	8	17	0:08:17	RBNU		1
5	10:15	9	0	0:09:00	RBNU		1
5	10:16	10	11	0:10:11	RBNU		1
5	10:13	7	27	0:07:27	RBNU		1
11	10:20	14	11	0:14:11	RBNU		2
3	10:07	1	4	0:01:04	start		
5	10:11	5	17	0:05:17	start		
11	10:16	10	16	0:10:16	start		
11	10:23	17	41	0:17:41	stop		
	10:06	0	0	0:00:00			
3	10:34	17	19	0:17:19	BCCH	M	1
3	10:30	13	7	0:13:07	BCCH	M	1
3	10:30	13	18	0:13:18	BCCH	M	1

3	10:32	15	11	0:15:11	BCCH	M	1
3	10:31	14	19	0:14:19	BCCH	M	1
3	10:31	14	36	0:14:36	BCCH	M	1
3	10:33	16	46	0:16:46	BCCH	M	1
3	10:35	18	14	0:18:14	BCCH	M	1
3	10:29	12	56	0:12:56	BCCH	M	1
3	10:30	13	28	0:13:28	BCCH	M	1
3	10:31	14	1	0:14:01	BCCH	M	1
3	10:32	15	56	0:15:56	BCCH	M	1
3	10:30	13	47	0:13:47	BCCH	M	1
3	10:36	19	9	0:19:09	BCCH	M	1
3	10:28	11	6	0:11:06	BCCH	M	1
3	10:29	12	15	0:12:15	BCCH	M	1
3	10:31	14	51	0:14:51	BCCH	M	1
11	10:41	24	10	0:24:10	BCCH	M	1
11	10:42	25	33	0:25:33	BCCH	M	1
11	10:43	26	55	0:26:55	BCCH	M	1
3	10:36	19	40	0:19:40	BCCH	M	1
11	10:41	24	41	0:24:41	BCCH	M	1
5	10:20	3	44	0:03:44	MOCH	M	1
5	10:21	4	26	0:04:26	MOCH	M	1
5	10:21	4	58	0:04:58	MOCH		2
5	10:22	5	9	0:05:09	MOCH		2
5	10:22	5	54	0:05:54	MOCH		2
5	10:23	6	5	0:06:05	MOCH		2
5	10:23	6	5	0:06:05	MOCH		2
5	10:23	6	32	0:06:32	MOCH		2
5	10:23	6	58	0:06:58	MOCH		2
5	10:24	7	13	0:07:13	MOCH		2
5	10:24	7	49	0:07:49	MOCH		1
5	10:25	8	50	0:08:50	MOCH	M	1
5	10:27	10	4	0:10:04	MOCH	M	1
3	10:27	10	50	0:10:50	MOCH	M	1
5	10:21	4	7	0:04:07	MOCH	M	1
5	10:22	5	9	0:05:09	MOCH		2
5	10:23	6	41	0:06:41	MOCH		2
5	10:21	4	34	0:04:34	MOCH		2
5	10:22	5	23	0:05:23	MOCH		2
5	10:23	6	17	0:06:17	MOCH		2
5	10:23	6	41	0:06:41	MOCH		2
5	10:25	8	16	0:08:16	MOCH		1
5	10:25	8	33	0:08:33	MOCH	M	1
3	10:27	10	57	0:10:57	MOCH	M	1
3	10:37	20	46	0:20:46	MOCH		1

5	10:22	5	23	0:05:23	MOCH		2
3	10:27	10	40	0:10:40	MOCH	M	1
5	10:19	2	52	0:02:52	MOCH	M	1
5	10:20	3	9	0:03:09	MOCH	M	1
5	10:23	6	17	0:06:17	MOCH		2
5	10:19	2	32	0:02:32	MOCH	M	1
3	10:28	11	22	0:11:22	MOCH	M	1
11	10:43	26	21	0:26:21	MOCH	M	1
5	10:21	4	42	0:04:42	MOCH	M	3
5	10:25	8	33	0:08:33	RBNU		1
3	10:32	15	0	0:15:00	RBNU		1
3	10:32	15	55	0:15:55	RBNU		1
3	10:33	16	32	0:16:32	RBNU		1
5	10:24	7	19	0:07:19	RBNU	M	2
5	10:24	7	19	0:07:19	RBNU	F	2
3	10:29	12	1	0:12:01	RBNU		1
5	10:19	2	15	0:02:15	RBNU		1
5	10:20	3	19	0:03:19	RBNU		1
3	10:28	11	45	0:11:45	RBNU		1
11	10:42	25	19	0:25:19	RBNU		1
11	10:42	25	58	0:25:58	RBNU		1
11	10:45	28	51	0:28:51	RBNU		3
11	10:44	27	6	0:27:06	RBNU		1
5	10:24	7	32	0:07:32	RBNU		2
3	10:38	21	47	0:21:47	RBNU		1
11	10:45	28	10	0:28:10	RBNU		2
5	10:18	1	52	0:01:52	start		
3	10:27	10	6	0:10:06	start		
11	10:40	23	8	0:23:08	start		
11	10:46	29	30	0:29:30	stop		
	10:17	0	0	0:00:00			
3	10:30	7	31	0:07:31	BCCH	M	1
3	10:33	10	38	0:10:38	BCCH	M	2
3	10:31	8	27	0:08:27	BCCH	M	1
3	10:32	9	2	0:09:02	BCCH	M	2
3	10:30	7	1	0:07:01	BCCH	M	1
3	10:32	9	2	0:09:02	BCCH	M	2
5	10:43	20	29	0:20:29	MOCH	M	1
5	10:44	21	15	0:21:15	MOCH	M	1
11	10:36	13	15	0:13:15	RBNU	M	1
5	10:41	18	1	0:18:01	RBNU		1
5	10:41	18	49	0:18:49	RBNU		1
5	10:45	22	4	0:22:04	RBNU		1
11	10:35	12	14	0:12:14	RBNU	M	1

5	10:45	22	18	0:22:18	RBNU		1
5	10:42	19	35	0:19:35	RBNU		1
5	10:44	21	40	0:21:40	RBNU		1
3	10:31	8	27	0:08:27	RBNU		1
5	10:42	19	3	0:19:03	RBNU		1
5	10:47	24	10	0:24:10	RBNU		1
5	10:48	25	29	0:25:29	RBNU		1
5	10:43	20	48	0:20:48	RBNU		1
3	10:30	7	54	0:07:54	RBNU		1
3	10:31	8	17	0:08:17	RBNU		1
5	10:42	19	49	0:19:49	RBNU		1
5	10:47	24	28	0:24:28	RBNU		1
5	10:47	24	58	0:24:58	RBNU		1
5	10:45	22	36	0:22:36	RBNU		1
5	10:48	25	35	0:25:35	RBNU		1
3	10:31	8	42	0:08:42	RBNU		1
11	10:36	13	29	0:13:29	RBNU		2
11	10:37	14	33	0:14:33	RBNU		2
11	10:39	16	32	0:16:32	RBNU		2
3	10:28	5	4	0:05:04	start		
11	10:34	11	37	0:11:37	start		
5	10:40	17	50	0:17:50	start		
5	10:49	26	2	0:26:02	stop		
	10:23	0	0	0:00:00			
11	8:43	2	39	0:02:39	RBNU	F	2
11	8:45	4	55	0:04:55	RBNU	M	2
11	8:47	6	44	0:06:44	RBNU	M	2
11	8:46	5	23	0:05:23	RBNU	M	2
11	8:41	0	50	0:00:50	RNSA	M	1
5	8:55	14	36	0:14:36	RNSA	M	1
11	8:41	0	44	0:00:44	start		
3	8:47	6	54	0:06:54	start		
5	8:52	11	32	0:11:32	start		
5	8:59	18	40	0:18:40	stop		
	8:41	0	0	0:00:00			
3	11:37	12	10	0:12:10	BCCH	M	1
3	11:38	13	56	0:13:56	BCCH	M	1
5	11:29	4	32	0:04:32	RBNU		2
	11:25	0	0	0:00:00	RBNU		1
3	11:36	11	55	0:11:55	RBNU		1
5	11:28	3	53	0:03:53	RBNU		1
3	11:38	13	4	0:13:04	RBNU		1
3	11:39	14	54	0:14:54	RBNU		1
5	11:26	1	24	0:01:24	RTHA		1

5	11:25	0	50	0:00:50	start		
11	11:31	6	27	0:06:27	start		
3	11:35	10	45	0:10:45	start		
3	11:40	15	17	0:15:17	stop		
	11:25	0	0	0:00:00			
11	8:02	3	42	0:03:42	RBNU		1
11	8:02	3	10	0:03:10	RBNU		1
11	8:03	4	47	0:04:47	RBNU		1
3	8:08	9	39	0:09:39	RBNU		1
11	8:03	4	57	0:04:57	RBNU		2
11	8:01	2	12	0:02:12	start		
3	8:06	7	5	0:07:05	start		
5	8:11	12	22	0:12:22	start		
5	8:15	16	31	0:16:31	stop		
	7:59	0	0	0:00:00			
3	8:12	15	45	0:15:45	BCCH	M	1
3	8:13	16	12	0:16:12	BCCH	M	1
3	8:11	14	54	0:14:54	BCCH	M	1
5	7:58	1	45	0:01:45	MOCH	M	1
3	8:13	16	10	0:16:10	RBNU	M	2
5	8:00	3	6	0:03:06	RBNU	M	1
5	8:00	3	14	0:03:14	RBNU	M	1
5	8:02	5	7	0:05:07	RBNU	M	2
5	8:02	5	38	0:05:38	RBNU	F	2
11	8:06	9	20	0:09:20	RBNU	F	2
11	8:06	9	20	0:09:20	RBNU	M	2
11	8:06	9	49	0:09:49	RBNU	F	2
11	8:07	10	1	0:10:01	RBNU	M	2
3	8:12	15	17	0:15:17	RBNU	M	2
3	8:12	15	33	0:15:33	RBNU	F	2
5	8:00	3	54	0:03:54	RBNU		2
11	8:07	10	5	0:10:05	RBNU	F	2
	7:57	0	41	0:00:41	RBNU		1
3	8:13	16	20	0:16:20	RBNU	M	2
5	7:58	1	3	0:01:03	start		
11	8:04	7	12	0:07:12	start		
3	8:10	13	18	0:13:18	start		
3	8:15	18	55	0:18:55	stop		
	7:57	0	0	0:00:00			
1	10:27	1	6	0:01:06	BCCH	M	1
1	10:30	4	52	0:04:52	BCCH	M	2
1	10:32	6	34	0:06:34	BCCH	M	1
1	10:34	8	58	0:08:58	BCCH	M	1
5	10:35	9	54	0:09:54	BCCH	M	1

5	10:37	11	8	0:11:08	BCCH	M			1
5	10:39	13	18	0:13:18	BCCH	M			1
13	10:44	18	26	0:18:26	BCCH	M			1
13	10:45	19	20	0:19:20	BCCH	M			1
13	10:46	20	52	0:20:52	BCCH	M			1
13	10:47	21	51	0:21:51	BCCH	M			1
13	10:52	26	7	0:26:07	BCCH	M			1
1	10:26	0	44	0:00:44	BCCH	M			1
13	10:50	24	16	0:24:16	CHIC	U			1
5	10:37	11	8	0:11:08	MOCH	M			1
5	10:36	10	17	0:10:17	MOCH	M			1
1	10:31	5	1	0:05:01	MOCH	M			1
5	10:39	13	58	0:13:58	MOCH	M			1
1	10:28	2	32	0:02:32	MOCH	M			2
1	10:28	2	32	0:02:32	MOCH	M			2
13	10:45	19	27	0:19:27	RBNU	M			1
13	10:45	19	16	0:19:16	RBNU	M			1
13	10:44	18	59	0:18:59	RBNU	M			1
1	10:28	2	30	0:02:30	RBNU	M			1
13	10:44	18	24	0:18:24	RBNU	M			1
1	10:27	1	59	0:01:59	RBNU	M			1
13	10:43	17	43	0:17:43	RBNU	M			1
13	10:44	18	5	0:18:05	RBNU	M			1
1	10:27	1	10	0:01:10	RBNU	M			1
13	10:48	22	41	0:22:41	RBNU	F			1
1	10:27	1	41	0:01:41	RBNU	M			1
5	10:40	14	17	0:14:17	RBNU	M			1
13	10:43	17	13	0:17:13	RBNU	M			1
13	10:51	25	46	0:25:46	RBNU	M			1
13	10:48	22	8	0:22:08	RBNU	M			1
13	10:49	23	29	0:23:29	RBNU	M			2
1	10:33	7	42	0:07:42	RBNU	M			2
5	10:37	11	40	0:11:40	RBNU	M			1
5	10:38	12	1	0:12:01	RBNU	M			1
13	10:46	20	1	0:20:01	RBNU	U			2
1	10:26	0	0	0:00:00	START				
5	10:35	9	30	0:09:30	START				
13	10:43	17	0	0:17:00	START				
	8:41:10			7:10	MOCH	M			1
	8:42:10			8:10	MOCH	M			1
	8:56:00			22:00	MOCH	M			1
	8:41:15			7:15	MOCH	M			1
1				0:10:00	MOCH	M			1
1				0:10:20	MOCH	M			1

11	0:14:40	MOCH	M	1
5	0:04:10	MOCH	M	1
1	0:08:10	MOCH	M	1
5	0:03:30	MOCH	M	1
11	0:16:10	MOCH	M	1
5	0:03:00	MOCH	M	1
5	0:06:50	MOCH	M	1
11	0:17:00	MOCH	F	2
5	0:01:00	START		
1	0:07:45	START		
11	0:14:30	START		
1	0:01:30	START		
5	0:06:30	START		
11	0:11:00	START		
5	0:01:30	MOCH	U	1
11	0:07:35	MOCH	U	1
5	0:01:00	MOCH	U	1
11	0:13:00	MOCH	U	1
1	0:19:01	MOCH	B	2
11	0:13:40	RBNU	M	2
11	0:13:50	RBNU	F	2
11	0:16:00	RBNU	M	2
1	0:22:10	RBNU	M	2
11	0:05:00	MOCH	U	1
11	0:04:05	RBNU	M	2
11	0:04:00	RBNU	M	2
11	0:03:20	RBNU	M	2
11	0:06:20	RBNU	M	2
5	0:10:10	RBNU	M	2
1	0:17:30	RBNU	M	2
	0:00:00	RBNU	M	2
5	0:01:30	MOCH	U	1
11	0:07:35	MOCH	U	1
5	0:01:00	MOCH	U	1
11	0:13:00	MOCH	U	1
1	0:19:01	MOCH	B	2
11	0:13:40	RBNU	M	2
11	0:13:50	RBNU	F	2
11	0:16:00	RBNU	M	2
1	0:22:10	RBNU	M	2
11	0:05:00	MOCH	U	1
11	0:04:05	RBNU	M	2
11	0:04:00	RBNU	M	2
11	0:03:20	RBNU	M	2

11				0:06:20	RBNU	M	2
5				0:10:10	RBNU	M	2
1				0:17:30	RBNU	M	2
				0:00:00	RBNU	M	2
	15:59:30			16:30	MOCH	M	1
	15:47:50			4:50	MOCH	M	1
	15:49:00			6:00	MOCH	M	1
	15:56:00			13:00	MOCH	M	1
	16:03:00			20:00	MOCH	M	1
	15:53:00			10:00	MOCH	M	1
	16:05:10			22:10	MOCH	M	1
	15:50:40			7:40	MOCH	M	1
	15:55:00			12:00	MOCH	M	1
	9:45:40			2:40	RBNU	M	1
	9:44:56			1:56	RBNU	M	1
	9:44:46			1:46	RBNU	M	1
	9:57:27			14:27	RBNU	M	1
	9:58:39			15:39	RBNU	M	1
	9:53:13			10:13	RBNU	M	1
	9:48:13			5:13	RBNU	M	1
	9:50:40			7:40	RBNU	M	1
	10:04:39			21:39	RBNU	M	1
11				0:01:40	RBNU	M	1
11				0:02:20	RBNU	M	1
11				0:02:50	RBNU	M	1
5				0:07:00	RBNU	M	1
1				0:14:40	RBNU	M	1
11				0:05:00	RBNU	M	1
11				0:00:25	START		
5				0:06:20	START		
1				0:11:15	START		
1		15	0	0:15:00	END		
1		12	0	0:12:00	MOCH		1
5		6	0	0:06:00	MOCH	M	1
11		3	30	0:03:30	RBNU		1
1		10	25	0:10:25	START		
5		5	45	0:05:45	START		
11		1	45	0:01:45	START		
	10:34	0	0	0:00:00	START		
1		15	32	0:15:32	BCCH	F	2
1		16	47	0:16:47	BCCH	F	2
1		17	8	0:17:08	BCCH	U	2
1		16	23	0:16:23	BCCH	M	2
1		15	31	0:15:31	BCCH	M	2

1		17	21	0:17:21	BCCH	U		1
1		18	12	0:18:12	MOCH	M		1
1		18	48	0:18:48	MOCH	U		1
1		19	41	0:19:41	MOCH	M		1
1		20	44	0:20:44	MOCH	M		1
1		21	30	0:21:30	MOCH	M		1
13		4	21	0:04:21	MOCH	U		1
13		0	0	0:00:00	START			
5		7	41	0:07:41	START			
1		15	0	0:15:00	START			
	10:08:20			0:08:20	BCCH	U		1
	10:14:20			14:20	BCCH	U		1
	10:15:00			15:00	MOCH	U		1
	8:03:50	3	50	3:50	BCCH	U		1
	8:03:20	3	20	3:20	BCCH	U		1
	8:26:20	26	20	26:20:00	MOCH	U		1
	12:08:20			11:20	BCCH	U		1
	10:03:45			6:45	BCCH	M		1
	10:01:09			4:09	BCCH	M		1
	10:00:53			3:53	BCCH	M		1
	10:00:43			3:43	BCCH	M		1
	10:01:45			4:45	BCCH	M		1
	10:03:35			6:35	BCCH	M		1
	9:59:45			2:45	BCCH	M		1
	10:14:50			17:50	BCCH	M		1
	10:08:22			11:22	BCCH	M		1
	10:13:10			16:10	RBNU	M		1
	10:12:48			15:48	RBNU	M		1
	10:11:30			14:30	RBNU	M		1
	10:04:30			2:30	RBNU	U		1
	10:11:20			9:20	RBNU	U		1
	10:14:30			12:30	RBNU	U		1
	10:25:15			23:15	RBNU	U		1
	10:26:50			24:50:00	RBNU	U		1
	8:58:00			3:00	BCCH	U		2
	9:13:30			18:30	MOCH	U		1
	9:13:10			18:10	MOCH	U		1
	9:11:15			16:15	RBNU	U		1
	9:13:30			18:30	RBNU	U		1
	0:20:10			20:10	MOCH	M	AGR	2
	0:22:30			22:30	MOCH	M	AGR	2
	0:20:10			20:10	MOCH	F	GAPO	2
	0:16:00			16:00	MOCH	B		2
	0:20:05			20:05	MOCH	M	AGR	2

0:25:00	25:00:00	MOCH	B		2
0:14:00	14:00	MOCH	M	AGR	2
0:14:00	14:00	MOCH	F	GAPO	2
11:25:15	13:15	MOCH	M		2
11:31:40	19:40	MOCH	M		1
11:32:10	20:10	MOCH	M		1
11:25:00	13:00	MOCH	M		2
11:25:15	13:15	MOCH	F		2
11:27:10	15:10	MOCH	M		2
11:27:10	15:10	MOCH	F		2
11:17:00	5:00	MOCH	U		1
11:30:30	18:30	MOCH	M		2
11:16:00	4:00	MOCH	M		2
12:16:00	4:00	MOCH	F		2
11:32:50	20:50	MOCH	M		1
11:14:47	2:47	MOCH	U		2
10:44:10	23:10	BCCH	M		1
10:42:40	21:40	BCCH	M		1
10:35:00	14:00	BCCH	U		1
10:25:20	4:20	BCCH	M		1
10:27:00	6:00	MOCH	M		1
10:24:43	3:43	RBNU	U		1
10:27:10	6:10	RBNU	U		1
10:36:40	15:40	RBNU	U		1
11:12:30	17:30	MOCH	M		2
11:12:40	17:40	MOCH	M		2
11:09:40	14:40	MOCH	M		2
11:17:30	22:30	MOCH	M		2
11:01:20	6:20	MOCH	U		1
11:16:30	21:30	MOCH	M		2
10:57:22	2:22	MOCH	U		1
11:19:10	24:10:00	MOCH	M		2
11:22:00	27:00:00	MOCH	M		2
11:13:00	18:00	MOCH	M		2
11:04:26	9:26	RBNU	U		2
11:00:40	5:40	RBNU	U		2
11:07:00	12:00	RBNU	U		2
11:23:40	28:40:00	RBNU	M		2
11:17:42	22:42	RBNU	U		2
11:02:56	7:56	RBNU	U		2
11:05:00	10:00	RBNU	U		2
11:05:00	10:00	TAHU	U		2
8:53:30	19:30	MOCH	U		1
8:53:00	19:00	MOCH	U		1

8:52:25	18:25	MOCH	U	1
8:48:40	14:40	MOCH	U	1
8:50:30	16:30	MOCH	U	1
8:51:10	17:10	MOCH	U	1
8:48:20	14:20	MOCH	U	1
8:54:45	20:45	MOCH	U	1
8:48:00	14:00	MOCH	U	1
8:56:00	22:00	MOCH	U	1
8:59:45	25:45:00	MOCH	M	1
9:12:20	27:20:00	MOCH	M	2
9:10:30	25:30:00	MOCH	M	2
9:09:00	24:00:00	MOCH	M	2
9:08:00	23:00	MOCH	M	1
9:15:00	30:00:00	MOCH	F	2
9:08:30	23:30	MOCH	F	2
9:14:00	29:00:00	MOCH	M	2
8:55:50	10:50	MOCH	M	1
9:04:58	19:58	MOCH	M	1
11:19:50	19:50	MOCH	M	2
11:20:10	20:10	MOCH	M	2
11:19:00	19:00	MOCH	B	2
11:05:50	5:50	MOCH	U	1
11:16:15	16:15	MOCH	M	2
11:05:50	5:50	MOCH	M	1
11:10:55	10:55	RBNU	U	1
8:38:00	22:00	BCCH	U	1
8:39:00	23:00	BCCH	U	1
8:31:00	15:00	MOCH	M	1
8:25:00	9:00	MOCH	M	1
8:23:30	7:30	MOCH	M	1
8:44:00	28:00:00	MOCH	M	1
8:18:49	2:49	MOCH	M	1
14:04:15	7:15	MOCH	M	1
14:09:35	12:35	MOCH	M	1
14:15:00	18:00	MOCH	M	1
14:02:45	5:45	MOCH	M	1
14:15:30	18:30	MOCH	M	1
14:16:47	19:47	MOCH	M	1
14:05:09	8:09	MOCH	M	1
14:10:20	13:20	MOCH	M	1
8:50:55	13:55	MOCH	U	1
8:50:20	13:20	MOCH	U	1
8:58:10	21:10	MOCH	U	1
8:57:40	20:40	MOCH	U	1

8:44:20			7:20	MOCH	U	1
8:43:10			6:10	MOCH	U	1
8:59:06			22:06	MOCH	U	1
9:02:10			25:10:00	MOCH	U	1
8:41:10			4:10	MOCH	U	1
10:17:30			8:30	MOCH	M	1
10:17:30			8:30	MOCH	M	1
10:17:30	30:00:00	MOCH	8:30	MOCH	M	1
10:20:48			11:48	MOCH	M	1
10:20:48			11:48	MOCH	M	1
10:20:48	48:00:00	MOCH	11:48	MOCH	M	1
10:11:50			2:50	MOCH	M	1
10:11:50			2:50	MOCH	M	1
10:11:50	50:00:00	MOCH	2:50	MOCH	M	1
10:26:25			17:25	RBNU	U	1
10:26:25			17:25	RBNU	U	1
10:26:25	25:00:00	RBNU	17:25	RBNU	U	1
15:10:20			14:20	BCCH	M	2
15:19:20			23:20	BCCH	U	1
15:20:40			24:40:00	BCCH	U	1
15:10:40			14:40	BCCH	F	2
15:10:00			14:00	BCCH	F	2
15:08:00			12:00	BCCH	U	1
15:10:00			14:00	BCCH	M	2
15:22:50			26:50:00	MOCH	U	1
15:17:20			21:20	MOCH	U	1
15:16:10			20:10	MOCH	U	1
15:01:45			5:45	RBNU	U	1
9:07:17			20:17	MOCH	U	1
9:02:00			15:00	MOCH	U	1
9:07:30			20:30	MOCH	U	1
9:08:00			21:00	MOCH	U	2
9:01:50			14:50	MOCH	U	1
9:08:30			21:30	MOCH	U	2
9:09:30			22:30	MOCH	U	2
8:51:00			4:00	MOCH	M	1
11:43:00			0:00	NIL		
8:56:50			18:50	MOCH	U	1
8:44:00			6:00	MOCH	U	1
9:01:20			23:20	MOCH	M	1
8:59:20			21:20	RBNU	M	1
9:00:30			22:30	RBNU	M	1
8:58:00			20:00	RBNU	M	1
9:02:40			24:40:00	RBNU	U	1

9:28:50	9:50	MOCH	M	1
9:31:50	12:50	MOCH	M	1
9:27:40	8:40	MOCH	M	1
9:26:50	7:50	MOCH	M	1
9:30:50	11:50	MOCH	M	1
9:23:30	4:30	MOCH	M	1
9:24:50	5:50	MOCH	M	1
9:33:40	14:40	MOCH	M	1
9:36:10	17:10	MOCH	M	1
9:34:16	15:16	MOCH	M	1
9:37:40	18:40	MOCH	M	1
9:39:00	20:00	MOCH	M	1
9:21:50	2:50	MOCH	M	2
9:22:00	3:00	MOCH	M	2
9:37:25	18:25	MOCH	M	2
10:01:35	17:35	BCCH	M	1
10:02:00	18:00	BCCH	M	1
10:01:30	17:30	BCCH	M	1
10:01:00	17:00	BCCH	M	1
9:59:40	15:40	BCCH	M	1
10:04:50	20:50	BCCH	M	1
10:08:20	24:20:00	MOCH	U	1
10:09:00	25:00:00	MOCH	U	1
9:56:40	12:40	MOCH	U	1
10:12:30	28:30:00	MOCH	U	1
10:13:00	29:00:00	MOCH	U	1
9:52:45	8:45	RBNU	M	1
9:53:20	9:20	RBNU	M	1
10:02:10	18:10	RBNU	M	1
9:52:00	8:00	RBNU	M	1
10:01:40	17:40	RBNU	M	1
9:56:40	12:40	RBNU	M	1
10:08:00	24:00:00	RBNU	M	1
9:55:00	11:00	RBNU	M	1
10:03:50	19:50	RBNU	M	1
10:58:20	24:20:00	MOCH	U	1
10:59:00	25:00:00	MOCH	U	1
10:47:00	13:00	MOCH	U	1
10:47:40	13:40	MOCH	U	1
10:53:30	19:30	MOCH	U	1
10:54:20	20:20	MOCH	U	1
10:57:40	23:40	MOCH	U	1
10:46:40	12:40	MOCH	U	1
10:56:00	22:00	MOCH	U	1

10:57:00	23:00	MOCH	U		1
11:47:30	23:30	NIL			
6:30:40	2:40	MOCH	U		1
6:40:50	12:50	MOCH	U		1
6:40:20	12:20	MOCH	U		1
6:33:00	5:00	MOCH	U		1
6:52:40	24:40:00	MOCH	U		1
6:47:40	19:40	RBNU	U		1
6:48:20	20:20	RBNU	M		1
6:49:00	21:00	RBNU	M		1
6:47:10	19:10	RBNU	M		1
6:49:10	21:10	RBNU	U		2
6:31:00	3:00	RBNU	U		1
6:42:30	14:30	RBNU	U		1
8:11:28	22:28	MOCH	U		2
8:11:39	22:39	MOCH	M	SY	3
8:08:20	19:20	MOCH	M	SY	1
8:09:00	20:00	MOCH	M	SY	1
8:11:00	22:00	MOCH	U		1
8:13:00	24:00:00	MOCH	M	SY	3
8:15:20	26:20:00	MOCH	U		2
8:09:30	20:30	MOCH	U		1
9:57:55	7:55	MOCH	U		1
10:06:00	16:00	RBNU	U		1
10:08:20	18:20	RBNU	U		1
10:10:00	20:00	RBNU	U		1
10:12:00	22:00	RBNU	U		1
10:13:20	23:20	RBNU	U		1
10:15:00	25:00:00	RBNU	U		1
9:30:30	20:30	MOCH	M		2
9:31:57	21:57	MOCH	U		2
9:30:30	20:30	MOCH	F		2
9:29:19	19:19	MOCH	M		2
9:32:37	22:37	MOCH	B		2
9:14:15	4:15	MOCH	U		2
9:14:20	4:20	MOCH	M		2
9:23:25	13:25	MOCH	M		2
7:02:30	0:05:30	DOWO	U		1
7:07:00	10:00	RBNU	U		1
7:51:40	23:40	BCCH	M		1
7:55:00	27:00:00	BCCH	M		1
7:38:10	10:10	MOCH	U		2
7:37:10	9:10	MOCH	U		2
7:39:53	11:53	MOCH	U		2

7:35:32	7:32	MOCH	U		2
7:36:40	8:40	MOCH	U		2
7:30:00	2:00	MOCH	B		2
7:28:55	0:55	MOCH	U		2
7:41:50	13:50	MOCH	U		2
7:52:54	24:54:00	MOCH	U		2
7:50:43	22:43	MOCH	M		2
7:29:30	1:30	MOCH	M		2
7:45:20	17:20	MOCH	U		2
7:28:58	0:58	RBNU	U		1
15:42:50	15:50	RBNU	M		1
15:40:10	13:10	RBNU	M		1
12:04:30	16:30	MOCH	M		1
11:55:40	7:40	MOCH	M		1
11:58:40	10:40	MOCH	M		1
11:59:40	11:40	MOCH	M		1
11:53:00	5:00	MOCH	M		1
12:01:40	13:40	MOCH	M		1
12:10:10	22:10	MOCH	M		1
12:10:11	22:11	MOCH	M		1
12:13:30	25:30:00	MOCH	M		1
9:53:00	3:00	MOCH	U		1
9:58:20	8:20	MOCH	U		1
10:14:15	24:15:00	MOCH	U		1
9:56:51	6:51	RBNU	M		1
9:56:10	6:10	RBNU	M		1
9:57:39	7:39	RBNU	M		1
9:58:50	8:50	RBNU	M		1
10:01:56	11:56	RBNU	M		1
10:10:40	20:40	RBNU	M		1
10:14:20	24:20:00	RBNU	M		1
10:07:50	17:50	RBNU	M		1
10:04:30	14:30	RBNU	M		1
15:59:30	16:30	MOCH	M		1
15:47:50	4:50	MOCH	M		1
15:49:00	6:00	MOCH	M		1
15:56:00	13:00	MOCH	M		1
16:03:00	20:00	MOCH	M		1
15:53:00	10:00	MOCH	M		1
16:05:10	22:10	MOCH	M		1
15:50:40	7:40	MOCH	M		1
15:55:00	12:00	MOCH	M		1
13:16:36	16:36	MOCH	M		2
12:45:15	23:15	MOCH	M	AGR	1

12:45:15	23:15	MOCH	M	AGR	1
12:42:40	20:40	MOCH	U		1
12:44:55	22:55	MOCH	M	AGR	1
12:45:50	23:50	MOCH	F		2
12:42:40	20:40	MOCH	U		1
12:44:55	22:55	MOCH	M	AGR	1
12:45:50	23:50	MOCH	F		2
12:26:50	4:50	MOCH	U		1
12:26:50	4:50	MOCH	U		1
12:46:00	24:00:00	MOCH	M		2
12:46:00	24:00:00	MOCH	M		2
12:28:10	6:10	MOCH	M		1
12:28:10	6:10	MOCH	M		1
14:49:29	22:29	MOCH	M		2
14:48:50	21:50	MOCH	M		2
14:47:35	20:35	MOCH	M		2
14:39:31	12:31	MOCH	M		2
14:33:07	6:07	MOCH	M		2
14:38:50	11:50	MOCH	M		2
14:44:21	17:21	MOCH	M		2
14:44:49	17:49	MOCH	M		2
14:32:55	5:55	MOCH	M		2
14:39:11	12:11	MOCH	M		2
14:29:59	2:59	MOCH	U		2
14:30:29	3:29	MOCH	U		2
14:33:43	6:43	MOCH	M		2
14:38:22	11:22	MOCH	M		2
8:34:00	0:00				
10:15:35	1:35	RBNU	U		1
10:14:50	0:50	RBNU	U		1
10:24:35	10:35	RBNU	U		1
10:19:00	5:00	RBNU	U		1
10:27:02	13:02	RBNU	U		1
9:43:40	17:40	MOCH	M		2
9:38:24	12:24	MOCH	M		2
9:40:30	14:30	MOCH	M		2
9:42:49	16:49	MOCH	M		2
9:32:45	6:45	MOCH	M		2
9:35:20	9:20	MOCH	M		2
9:37:20	11:20	MOCH	M		2
9:43:20	17:20	MOCH	M		2
9:44:00	18:00	MOCH	M		2
9:34:40	8:40	MOCH	M		2
9:36:10	10:10	MOCH	M		2

	9:39:40	13:40	MOCH	M		2
	9:42:15	16:15	MOCH	M		2
	9:26:50	0:50	MOCH	M		2
	9:27:30	1:30	MOCH	M		2
	9:28:56	2:56	MOCH	M		2
	9:39:45	13:45	MOCH	F		2
	9:27:30	1:30	MOCH	F		2
	9:39:15	13:15	MOCH	F		2
	9:45:50	19:50	MOCH	M		2
	9:28:38	2:38	MOCH	F		2
	9:43:40	17:40	MOCH	F		2
	9:38:10	12:10	MOCH	F		2
	9:46:05	20:05	MOCH	F		2
	13:45:03	4:03	MOCH	U		1
	13:46:41	5:41	MOCH	U		1
	13:48:35	7:35	MOCH	U		1
	13:54:50	13:50	RBNU	U		1
	14:22:50	11:50	MOCH	U		2
	16:10:45	1:45	MOCH	M		2
	16:20:15	11:15	MOCH	M		2
	16:10:45	1:45	MOCH	M		2
	16:20:15	11:15	MOCH	M		2
	16:13:38	4:38	MOCH	M		2
	16:13:38	4:38	MOCH	M		2
	16:16:36	7:36	MOCH	M		2
	16:16:36	7:36	MOCH	M		2
	11:21:16	13:16	BCCH	M		1
	11:20:18	12:18	BCCH	M		1
	11:18:50	10:50	BCCH	M		1
	11:35:08	27:08:00	MOCH	M		1
	11:28:16	20:16	MOCH	M		1
	11:35:20	27:20:00	MOCH	M		1
	11:35:45	27:45:00	MOCH	M		1
	11:27:00	19:00	MOCH	M		1
	11:30:50	22:50	MOCH	M		1
	11:25:53	17:53	MOCH	M		1
	11:16:10	8:10	MOCH	U		1
1		0:11:55	MOCH	M	SY	2
5		0:09:30	MOCH	M	SY	1
5		0:04:20	MOCH	M	SY	1
5		0:06:00	MOCH	M	SY	1
11		0:21:00	MOCH	B		2
11		0:22:30	MOCH	M	SY	2
5		0:06:30	MOCH	F		2

11			0:04:00	MOCH	M	1
1			0:12:50	BCCH	M	1
1			0:13:00	BCCH	M	1
1			0:12:40	BCCH	M	1
1			0:12:20	BCCH	M	1
11			0:07:30	MOCH	U	1
5			0:04:30	MOCH	U	1
1			0:12:30	MOCH	M	2
1			0:13:20	MOCH	M	2
5			0:08:20	MOCH	M	2
5			0:07:45	MOCH	M	2
11			0:02:25	START		
5			0:06:30	START		
1			0:12:20	START		
1			0:08:00	RBNU	U	1
5			0:02:00	START		
1			0:07:00	START		
11			0:12:00	START		
5			0:01:50	START		
1			0:06:40	START		
11			0:11:40	START		
11			0:01:20	START		
1			0:04:25	START		
5			0:08:10	START		
11	14	0	0:14:00	END		
1	7	30	0:07:30	MOCH	M,F	2
5	1	30	0:01:30	MOCH	M,F	2
1	6	30	0:06:30	START		
5	1	0	0:01:00	START		
11	10	30	0:10:30	START		
	0	0	0:00:00	START		
3	3	7	0:03:07	BCCH	M	1
3	2	52	0:02:52	BCCH	M	1
3	4	9	0:04:09	BCCH	M	1
3	4	20	0:04:20	BCCH	M	1
3	2	12	0:02:12	BCCH	M	1
3	1	42	0:01:42	BCCH	M	1
3	4	58	0:04:58	BCCH	M	2
3	4	33	0:04:33	BCCH	F	2
3	5	3	0:05:03	BCCH	F	2
3	5	42	0:05:42	BCCH	M	2
3	6	22	0:06:22	BCCH	M	2
5	9	24	0:09:24	BCCH	M	2
5	10	21	0:10:21	BCCH	M	2

5	13	5	0:13:05	BCCH	M	1
5	14	25	0:14:25	BCCH	M	1
3	2	30	0:02:30	BCCH	M	1
5	9	47	0:09:47	MOCH	M	1
5	11	34	0:11:34	MOCH	M	1
5	13	30	0:13:30	MOCH	M	1
5	11	8	0:11:08	MOCH	M	1
5	12	39	0:12:39	MOCH	M	1
5	15	2	0:15:02	MOCH	M	1
5	9	36	0:09:36	MOCH	M	1
5	9	11	0:09:11	MOCH	M	1
5	8	50	0:08:50	MOCH	M	1
13	17	3	0:17:03	MOCH	M	1
13	19	12	0:19:12	MOCH	M	1
13	17	58	0:17:58	MOCH	M	1
13	20	20	0:20:20	MOCH	M	1
13	18	11	0:18:11	MOCH	M	1
13	20	37	0:20:37	MOCH	M	1
13	21	47	0:21:47	MOCH	M	1
3	5	44	0:05:44	MOCH	M	1
13	18	21	0:18:21	RBNU	U	1
13	19	2	0:19:02	RBNU	U	1
5	7	35	0:07:35	START		
13	15	9	0:15:09	START		
5	8	55	0:08:55	MOCH	M	1
5	9	5	0:09:05	MOCH	M	1
5	8	17	0:08:17	MOCH	M	1
5	8	13	0:08:13	MOCH	M	1
5	8	22	0:08:22	MOCH	M	1
1	0	15	0:00:15	MOCH	U	1
1	0	43	0:00:43	MOCH	U	1
1	0	30	0:00:30	MOCH	U	1
1	3	7	0:03:07	MOCH	M	2
1	4	32	0:04:32	MOCH	M	2
1	1	2	0:01:02	MOCH	U	1
1	1	21	0:01:21	MOCH	U	1
13	18	18	0:18:18	MOCH	M	1
13	20	32	0:20:32	MOCH	M	1
13	21	31	0:21:31	MOCH	M	1
1	2	26	0:02:26	MOCH	U	2
13	15	55	0:15:55	RBNU	M	1
13	15	34	0:15:34	RBNU	M	1
13	15	21	0:15:21	RBNU	M	1
13	16	17	0:16:17	RBNU	M	1

13		16	44	0:16:44	RBNU	M	1
13		17	3	0:17:03	RBNU	M	1
13		17	11	0:17:11	RBNU	M	1
13		20	57	0:20:57	RBNU	M	1
13		22	30	0:22:30	RBNU	M	1
1		0	0	0:00:00	start		
5		7	30	0:07:30	start		
13		15	0	0:15:00	start		
13		1	27	0:01:27	MOCH	M	1
13		1	48	0:01:48	MOCH	M	1
1		9	43	0:09:43	MOCH	M	2
13		1	3	0:01:03	MOCH	M	1
1		9	27	0:09:27	MOCH	M	2
1		10	34	0:10:34	MOCH	M	2
13		0	49	0:00:49	MOCH	M	1
1		11	13	0:11:13	MOCH	M	2
1		11	13	0:11:13	MOCH	F	2
5		15	40	0:15:40	MOCH	M	1
1		9	27	0:09:27	MOCH	F	2
5		15	50	0:15:50	MOCH	M	1
1		11	27	0:11:27	MOCH	M	2
1		11	27	0:11:27	MOCH	F	2
13		2	9	0:02:09	MOCH	M	1
1		9	21	0:09:21	MOCH	M	1
13		3	7	0:03:07	MOCH	M	2
1		9	5	0:09:05	MOCH	M	1
5		16	33	0:16:33	MOCH	M	1
5		17	10	0:17:10	MOCH	M	1
5		18	1	0:18:01	MOCH	M	1
13		2	59	0:02:59	MOCH	M	2
13		4	23	0:04:23	MOCH	M	2
13		5	20	0:05:20	MOCH	M	1
13		7	13	0:07:13	MOCH	M	1
1		8	54	0:08:54	MOCH	M	1
1		14	38	0:14:38	MOCH	M	1
1		13	13	0:13:13	MOCH	M	1
5		18	19	0:18:19	MOCH	M	1
5		19	11	0:19:11	MOCH	M	1
5		19	31	0:19:31	MOCH	M	1
5		20	49	0:20:49	MOCH	M	1
13		0	0	0:00:00	start		
1		7	43	0:07:43	start		
5		15	0	0:15:00	start		
5	9:39	24	30	0:24:30	MOCH	M	1

5	9:41	26	8	0:26:08	MOCH	M	1
5	9:42	27	35	0:27	MOCH	M	1
5	9:38	23	38	0:23:38	MOCH	M	1
5	9:37	22	54	0:22:54	MOCH	M	1
5	9:43	28	40	0:28:40	MOCH	M	1
5	9:44	29	20	0:29:20	MOCH	M	1
5	9:37	22	22	0:22:22	MOCH	M	1
13	9:26	11	22	0:11:22	RBNU	M	1
13	9:25	10	6	0:10:06	RBNU	M	1
13	9:26	11	4	0:11:04	RBNU	M	1
13	9:25	10	42	0:10:42	RBNU	M	1
13	9:24	9	1	0:09:01	RBNU	M	1
13	9:23	8	48	0:08:48	RBNU	M	1
13	9:23	8	13	0:08:13	RBNU	M	1
1	9:16	1	15	0:01	RBNU	M	1
1	9:16	1	21	0:01:21	RBNU	M	1
13	9:22	7	59	0:07:59	RBNU	M	1
13	9:27	12	44	0:12:44	RBNU	M	1
13	9:28	13	38	0:13:38	RBNU	M	1
13	9:29	14	20	0:14:20	RBNU	M	1
13	9:22	7	46	0:07:46	RBNU	M	1
13	9:32	17	9	0:17:09	RBNU	M	1
5	9:37	22	9	0:22	RBNU	M	1
13	9:30	15	0	0:15:00	RBNU	M	1
13	9:31	16	30	0:16:30	RBNU	M	1
13	9:34	19	6	0:19:06	RBNU	M	1
13	9:35	20	55	0:20:55	RBNU	M	1
1	9:19	4	49	0:04:49	RBNU	M	1
1	9:15	0	0	0:00:00	START		
13	9:22	7	30	0:07:30	START		
5	9:37	22	8	0:22:08	START		
11		0	0	0:00:00	START		
3		4	42	0:04:42	START		
5		8	46	0:08:46	START		
11		5	59	0:05:59	MOBL	F	1
11		1	15	0:01:15	RBNU	M	1
11		2	3	0:02:03	RBNU	M	1
11		3	15	0:03:15	RBNU	M	3
11		5	1	0:05:01	RBNU	M	3
11		5	1	0:05:01	RBNU	F	3
11		5	59	0:05:59	RBNU	F	3
11		6	39	0:06:39	RBNU	M	3
11		6	39	0:06:39	RBNU	F	3
5		11	40	0:11:40	RBNU	M	2

3		15	26	0:15:26	RBNU	M	2
11		4	4	0:04:04	RBNU	U	3
11		4	19	0:04:19	RBNU	U	3
3		16	41	0:16:41	RBNU	U	2
3		16	50	0:16:50	RBNU	U	2
3		19	49	0:19:49	RBNU	U	2
11		2	32	0:02:32	RBNU	U	2
11		0	0	0:00:00	START		
5		8	21	0:08:21	START		
3		14	12	0:14:12	START		
5		17	2	0:17:02	MOCH	M	2
5		18	26	0:18:26	MOCH	U	2
5		16	37	0:16:37	MOCH	M	2
5		19	35	0:19:35	MOCH	U	2
5		20	53	0:20:53	MOCH	U	2
5		16	19	0:16:19	MOCH	M	2
5		15	28	0:15:28	MOCH	M	2
5		14	40	0:14:40	MOCH	M	2
5		15	13	0:15:13	MOCH	M	2
11		4	12	0:04:12	MOCH	U	2
11		4	42	0:04:42	MOCH	U	2
11		6	26	0:06:26	MOCH	U	2
4		10	39	0:10:39	MOCH	U	2
11		6	1	0:06:01	MOCH	U	2
11		1	8	0:01:08	MOCH	U	2
11		2	5	0:02:05	MOCH	U	2
11		0	49	0:00:49	MOCH	U	1
11		5	0	0:05:00	MOCH	U	2
11		3	30	0:03:30	RBNU	U	1
4		9	5	0:09:05	RBNU	U	1
11		3	54	0:03:54	RBNU	U	2
11		4	11	0:04:11	RBNU	U	2
4		9	54	0:09:54	RBNU	U	2
4		13	15	0:13:15	RBNU	U	2
5		17	27	0:17:27	RBNU	U	1
5		19	13	0:19:13	RBNU	U	1
11		0	20	0:00:20	START		
4		7	24	0:07:24	START		
5		14	12	0:14:12	START		
	10:12:20			5:20	BCCH	U	1
	10:13:10			6:10	BCCH	U	1
	10:26:10			19:10	BCCH	M	1
	10:27:51			20:51	MOCH	M	1
	10:26:50			19:50	MOCH	M	1

10:27:50	20:50	MOCH	M	1
10:25:30	18:30	MOCH	M	1
10:29:00	22:00	MOCH	M	1
10:18:30	11:30	RBNU	M	1
10:18:00	11:00	RBNU	M	1
10:17:40	10:40	RBNU	M	1
10:20:00	13:00	RBNU	M	1
10:20:56	13:56	RBNU	M	1
10:12:20	5:20	BCCH	U	1
10:13:10	6:10	BCCH	U	1
10:26:10	19:10	BCCH	M	1
10:27:51	20:51	MOCH	M	1
10:26:50	19:50	MOCH	M	1
10:27:50	20:50	MOCH	M	1
10:25:30	18:30	MOCH	M	1
10:29:00	22:00	MOCH	M	1
10:18:30	11:30	RBNU	M	1
10:18:00	11:00	RBNU	M	1
10:17:40	10:40	RBNU	M	1
10:20:00	13:00	RBNU	M	1
10:20:56	13:56	RBNU	M	1
15:00:20	5:20	RBNU	M	1
15:00:39	5:39	RBNU	M	1
14:59:40	4:40	RBNU	M	1
14:59:01	4:01	RBNU	M	1
14:57:16	2:16	RBNU	M	1
14:59:00	4:00	RBNU	M	1
15:01:25	6:25	RBNU	M	1
15:01:00	6:00	RBNU	M	1
14:56:47	1:47	RBNU	M	1
15:02:30	7:30	RBNU	M	1
14:55:01	0:01	RBNU	M	1
10:06:20	9:20	RBNU	M	1
10:05:10	8:10	RBNU	M	1
10:16:10	19:10	RBNU	M	1
10:16:20	19:20	RBNU	M	1
10:08:30	11:30	RBNU	M	1
10:10:59	13:59	RBNU	M	1
10:11:01	14:01	RBNU	M	1
10:03:35	6:35	RBNU	M	1
10:03:00	6:00	RBNU	M	1
7:47:10	7:10	MOCH	U	1
7:46:35	6:35	MOCH	U	1
7:45:40	5:40	MOCH	U	1

7:47:05			7:05	MOCH	U	1
7:47:50			7:50	MOCH	U	1
7:53:30			13:30	MOCH	U	1
7:48:15			8:15	MOCH	U	1
7:46:10			6:10	MOCH	U	1
7:53:10			13:10	MOCH	U	1
7:46:45			6:45	MOCH	U	1
7:48:25			8:25	MOCH	U	1
8:04:00			24:00:00	MOCH	U	1
8:06:35			26:35:00	MOCH	U	1
7:55:30			15:30	RBNU	M	1
7:55:15			15:15	RBNU	M	1
7:55:50			15:50	RBNU	M	1
7:56:30			16:30	RBNU	M	1
7:54:45			14:45	RBNU	M	1
7:54:30			14:30	RBNU	M	1
7:57:20			17:20	RBNU	M	1
7:58:00			18:00	RBNU	M	1
7:58:30			18:30	RBNU	M	1
8:02:50			22:50	RBNU	M	1
8:00:50			20:50	RBNU	M	1
8:01:50			21:50	RBNU	M	1
8:06:35			26:35:00	RBNU	M	1
9:29:30	22	30	22:30	RBNU	M	1
9:31:40	24	40	24:40:00	RBNU	M	1
9:33:20	26	20	26:20:00	RBNU	M	1
9:30:15	23	15	23:15	RBNU	M	1
9:31:15	24	15	24:15:00	RBNU	M	1
9:28:45	21	45	21:45	RBNU	M	1
9:28:10	21	10	21:10	RBNU	M	1
9:32:20	25	20	25:20:00	RBNU	M	1
9:33:50	26	50	26:50:00	RBNU	M	1
9:28:00	21	0	21:00	RBNU	M	1
9:28:50	21	50	21:50	RBNU	M	1
7:50:10	5	10	5:10	MOCH	U	1
8:06:40	21	40	21:40	RBNU	U	2
8:07:30	22	30	22:30	RBNU	M	2
8:08:40	23	40	23:40	RBNU	U	2
7:48:10	3	10	3:10	RBNU	U	1
7:48:30	3	30	3:30	RBNU	U	2
7:53:15	8	15	8:15	RBNU	U	2
7:54:55	9	55	9:55	RBNU	U	2
7:58:20	13	20	13:20	RBNU	U	2
7:47:30	2	30	2:30	RBNU	U	1

8:10:00	25	0	25:00:00	RBNU	U	2
8:09:20				RBNU	M	1
8:11:00				RBNU	M	1
10:01:35			17:35	BCCH	M	1
10:02:00			18:00	BCCH	M	1
10:01:30			17:30	BCCH	M	1
10:01:00			17:00	BCCH	M	1
9:59:40			15:40	BCCH	M	1
10:04:50			20:50	BCCH	M	1
10:08:20			24:20:00	MOCH	U	1
10:09:00			25:00:00	MOCH	U	1
9:56:40			12:40	MOCH	U	1
10:12:30			28:30:00	MOCH	U	1
10:13:00			29:00:00	MOCH	U	1
9:52:45			8:45	RBNU	M	1
9:53:20			9:20	RBNU	M	1
10:02:10			18:10	RBNU	M	1
9:52:00			8:00	RBNU	M	1
10:01:40			17:40	RBNU	M	1
9:56:40			12:40	RBNU	M	1
10:08:00			24:00:00	RBNU	M	1
9:55:00			11:00	RBNU	M	1
10:03:50			19:50	RBNU	M	1
6:47:06			14:06	BCCH	M	1
6:57:35			24:35:00	MOCH	M	2
6:54:40			21:40	MOCH	M	2
6:55:00			22:00	MOCH	M	2
6:58:45			25:45:00	MOCH	M	2
6:52:40			19:40	RBNU	U	2
6:33:00			0:00	RBNU	U	1
6:41:45			8:45	RBNU	U	1
6:54:40			21:40	RBNU	U	2
6:57:45			24:45:00	RBNU	U	2
6:43:01			10:01	RBNU	U	1
6:51:05			18:05	RBNU	U	1
9:58:20			16:20	RBNU	M	2
9:58:30			16:30	RBNU	F	2
10:01:00			19:00	RBNU	M	2
10:01:00			19:00	RBNU	F	2
10:03:20			21:20	RBNU	M	2
9:42:29			0:29	RBNU	U	1
9:46:40			4:40	RBNU	U	1
9:54:50			12:50	RBNU	U	1
9:57:20			15:20	RBNU	U	1

9:54:20	12:20	RBNU	U		1
13:07:20	15:20	MOCH	M		1
13:01:40	9:40	RBNU	M		1
13:01:20	9:20	RBNU	M		1
13:00:30	8:30	RBNU	M		1
13:06:10	14:10	RBNU	M		1
13:07:20	15:20	RBNU	M		1
13:08:00	16:00	RBNU	M		1
12:52:00	0:00	RBNU	M		1
15:16:50	6:50	BCCH	M		1
15:18:00	8:00	BCCH	M		1
15:16:18	6:18	BCCH	M		1
15:30:55	20:55	MOCH	M		1
15:31:30	21:30	MOCH	M		1
15:32:45	22:45	MOCH	M		1
15:30:15	20:15	MOCH	M		1
15:29:00	19:00	MOCH	M		1
15:31:45	21:45	RBNU	U		1
15:31:20	21:20	RBNU	U		1
15:11:02	1:02	RBNU	U		1
15:17:35	7:35	RBNU	U		1
15:16:20	6:20	RBNU	U		1
15:34:00	24:00:00	RBNU	U		1
15:29:05	19:05	RBNU	U		1
15:24:26	14:26	RBNU	U		1
15:27:55	17:55	RBNU	U		1
12:45:15	23:15	MOCH	M	AGR	1
12:42:40	20:40	MOCH	U		1
12:44:55	22:55	MOCH	M	AGR	1
12:45:50	23:50	MOCH	F		2
12:26:50	4:50	MOCH	U		1
12:46:00	24:00:00	MOCH	M		2
12:28:10	6:10	MOCH	M		1
9:58:28	26:28:00	MOCH	M		1
10:00:10	28:10:00	MOCH	M		1
10:00:30	28:30:00	MOCH	M		1
9:55:00	23:00	MOCH	M		1
9:45:50	13:50	MOCH	M		1
9:39:05	7:05	RBNU	M		1
9:41:00	9:00	RBNU	M		1
9:46:50	14:50	RBNU	M		1
9:34:58	2:58	RBNU	U		1
9:37:49	5:49	RBNU	U		1
9:57:45	25:45:00	RBNU	U		1

	9:45:58			13:58	RBNU	U	1
	9:51:24			19:24	RBNU	U	1
	9:59:00			27:00:00	RBNU	U	1
1				0:03:40	RBNU	U	1
1				0:04:20	RBNU	U	2
1				0:02:50	RBNU	U	1
1				0:00:00	START		
11				0:05:10	START		
5				0:09:10	START		
11				0:03:35	RBNU	F	2
11				0:04:40	RBNU	F	2
11				0:05:30	RBNU	M	2
11				0:06:10	RBNU	F	2
5				0:10:30	RBNU	M	2
5				0:14:00	RBNU	M	2
1				0:21:40	RBNU	M	2
5				0:10:40	RBNU	M	2
11				0:04:30	RBNU	M	2
11				0:01:57	START		
5				0:09:50	START		
1				0:17:25	START		
11		26	11	0:26:11	DOWO	U	1
5		1	26	0:01:26	MOCH	M	1
5		3	0	0:03:00	MOCH	M	2
5		10	50	0:10:50	MOCH	M	2
1		16	29	0:16:29	MOCH	M	1
1		17	16	0:17:16	MOCH	M	1
5		1	42	0:01:42	MOCH	M	1
1		16	59	0:16:59	MOCH	M	1
5		10	5	0:10:05	MOCH	M	2
5		2	20	0:02:20	MOCH	F	2
5		0	10	0:00:10	MOCH	M	1
5		0	34	0:00:34	MOCH	M	1
5		1	0	0:01	MOCH	M	1
5		9	15	0:09:15	MOCH	M	2
5		10	40	0:10:40	MOCH	M	2
1		16	28	0:16:28	MOCH	M	1
5		2	50	0:02:50	MOCH	F	2
5		8	55	0:08:55	MOCH	M	2
5		10	40	0:10:40	MOCH	F	2
5		12	0	0:12:00	MOCH	M	2
5		3	40	0:03:40	MOCH	M	2
5		8	0	0:08:00	MOCH	M	2
1		20	50	0:20:50	MOCH	M	1

11		26	27	0:26:27	MOCH	U	1
11		28	12	0:28:12	MOCH	M	1
11		31	0	0:31:00	MOCH	M	1
11		25	47	0:25:47	MOCH	M	1
11		26	58	0:26:58	MOCH	M	1
1		15	52	0:15:52	START		
11		23	7	0:23:07	START		
5		11	29	0:11:29	MOCH	M	1
5		10	26	0:10:26	MOCH	M	1
5		12	0	0:12:00	MOCH	M	1
5		11	20	0:11:20	MOCH	M	1
5		12	14	0:12:14	MOCH	M	1
5		10	19	0:10:19	MOCH	M	1
1		17	35	0:17:35	MOCH	M	1
5		11	39	0:11:39	MOCH	M	1
5		10	3	0:10:03	MOCH	M	1
5		12	35	0:12:35	MOCH	M	1
5		12	50	0:12:50	MOCH	M	1
1		18	47	0:18:47	MOCH	M	1
5		13	26	0:13:26	MOCH	M	2
5		16	21	0:16:21	MOCH	M	2
5		14	22	0:14:22	MOCH	M	2
5		15	27	0:15:27	MOCH	M	2
5		13	44	0:13:44	MOCH	M	2
5		14	22	0:14:22	MOCH	M	2
5		15	2	0:15:02	MOCH	M	2
5		15	23	0:15:23	MOCH	M	2
13		0	11	0:00:11	RBNU	M	1
13		1	15	0:01:15	RBNU	M	1
13		2	31	0:02:31	RBNU	M	1
13		1	58	0:01:58	RBNU	M	1
5		9	42	0:09:42	start		
1		17	30	0:17:30	start		
1	0:01	1	35	0:01:35	BCCH	M	1
5	0:16	16	43	0:16:43	MOCH	M	1
5	0:16	16	35	0:16:35	MOCH	M	1
5	0:17	17	15	0:17:15	MOCH	M	1
5	0:19	19	5	0:19:05	MOCH	M	1
5	0:21	21	45	0:21:45	MOCH	M	2
1	10:34	0	0	0:00:00	START		
13	0:07	7	30	0:07:30	START		
5	0:15	15	0	0:15:00	START		
1	10:54	9	49	0:09:49	MOCH	M	1
1	10:55	10	42	0:10:42	MOCH	M	1

1	10:58	13	7	0:13:07	MOCH	M	1
13	10:46	1	46	0:01:46	RBNU	M	1
1	10:56	11	27	0:11:27	RBNU	M	1
1	10:56	11	50	0:11:50	RBNU	M	1
1	10:57	12	43	0:12:43	RBNU	M	1
13	10:47	2	10	0:02:10	RBNU	M	1
13	10:46	1	2	0:01:02	RBNU	M	1
13	10:45	0	29	0:00:29	RBNU	M	1
13	10:46	1	30	0:01:30	RBNU	M	1
13	10:47	2	0	0:02:00	RBNU	M	1
13	10:47	2	37	0:02:37	RBNU	F	2
13	10:47	2	37	0:02:37	RBNU	M	2
1	10:57	12	55	0:12:55	RBNU	M	1
13	10:45	0	0	0:00:00	START		
1	10:54	9	30	0:09:30	START		
5	10:59	14	30	0:14:30	START		
	10:18:50				MOCH	U	1
	10:20:20				MOCH	U	1
	10:31:40				MOCH	U	1
	10:25:00				MOCH	U	1
	10:38:00				RBNU	M	1
	10:19:15				RBNU	M	1
	10:28:00				RBNU	M	1
	10:48:00				RBNU	U	1
	11:11:30			1:30	MOCH	U	1
	11:11:50			1:50	MOCH	U	1
	11:14:20			4:20	MOCH	U	1
	11:23:30			13:30	MOCH	M	2
	11:23:30			13:30	MOCH	F	2
	11:25:15			15:15	MOCH	U	1
	11:33:30			23:30	MOCH	U	1
	11:21:00			11:00	MOCH	U	1
	11:21:30			11:30	MOCH	U	1
	11:33:00			23:00	MOCH	U	1
	11:27:00			17:00	MOCH	M	1
	11:31:20			21:20	RBNU	M	1
	11:31:40			21:40	RBNU	M	1
	11:34:40			24:40:00	RBNU	U	1
	11:19:50			9:50	RBNU	U	1
	11:34:20			24:20:00	RBNU	U	1
	11:26:00			16:00	RBNU	U	1
	11:10:20			0:20	RBNU	U	1
	11:22:10			12:10	RBNU	U	1
	11:18:40			8:40	TAHU	U	1

11:35:25	25:25:00	TAHU	U	1
11:37:20	27:20:00	TAHU	U	1
11:15:30	5:30	TAHU	U	1
11:38:20	28:20:00	TAHU	U	1
11:10:30	0:30	TAHU	U	1
7:35:15	5:15	CORA	U	1
7:40:00	10:00	NOFL	U	1
7:46:20	16:20	RBNU	M	1
7:45:50	15:50	RBNU	M	1
7:46:40	16:40	RBNU	M	1
7:44:40	14:40	RBNU	M	1
7:47:40	17:40	RBNU	M	1
7:44:05	14:05	RBNU	M	1
7:50:15	20:15	RBNU	M	1
7:53:20	23:20	RBNU	U	1
7:54:00	24:00:00	RBNU	U	1
10:07:30	7:30	BCCH	M	1
10:08:50	8:50	BCCH	M	1
10:09:10	9:10	BCCH	U	1
10:01:00	1:00	MOCH	U	1
10:09:00	9:00	RBNU	U	1
11:09:40	9:40	BCCH	U	1
11:19:00	19:00	BCCH	U	1
11:17:00	17:00	MOCH	U	1
11:50:05	15:05	MOCH	U	1
11:51:20	16:20	MOCH	U	2
11:59:50	24:50:00	MOCH	U	2
11:49:30	14:30	MOCH	U	1
11:55:00	20:00	MOCH	U	2
11:48:15	13:15	MOCH	U	1
8:18:40	18:40	BCCH	M	2
8:18:40	18:40	BCCH	F	2
8:14:50	14:50	BCCH	U	1
8:15:40	15:40	BCCH	U	1
8:16:30	16:30	BCCH	U	1
8:20:20	20:20	BCCH	M	2
8:20:20	20:20	BCCH	F	2
8:07:30	7:30	BCCH	U	1
8:17:00	17:00	BCCH	U	2
8:07:00	7:00	BCCH	U	1
8:14:32	14:32	BCCH	U	1
8:18:00	18:00	BCCH	U	2
8:06:30	6:30	BCCH	U	1
8:14:30	14:30	BCCH	U	1

8:15:00	15:00	BCCH	U	1
8:07:50	7:50	BCCH	U	1
8:03:20	3:20	BCCH	U	1
8:22:10	22:10	BCCH	U	2
8:09:30	9:30	BCCH	U	1
8:13:30	13:30	BCCH	M	1
8:14:00	14:00	BCCH	U	1
8:24:50	24:50:00	BCCH	U	2
8:11:00	11:00	BCCH	M	1
8:23:00	23:00	RBNU	U	1
11:07:30	27:30:00	BCCH	M	1
11:07:50	27:50:00	BCCH	M	1
11:02:20	22:20	BCCH	M	1
11:03:20	23:20	BCCH	M	1
11:03:50	23:50	BCCH	M	1
11:04:30	24:30:00	BCCH	M	1
11:05:50	25:50:00	BCCH	M	1
11:05:00	25:00:00	BCCH	M	1
11:07:00	27:00:00	BCCH	M	1
11:02:00	22:00	BCCH	M	1
11:06:10	26:10:00	BCCH	M	1
11:01:20	21:20	BCCH	M	1
10:44:20	4:20	MOCH	U	1
10:52:20	12:20	MOCH	U	1
10:54:00	14:00	RBNU	M	1
10:54:20	14:20	RBNU	M	1
10:53:38	13:38	RBNU	M	1
10:53:30	13:30	RBNU	M	1
10:53:50	13:50	RBNU	M	1
10:55:20	15:20	RBNU	M	1
11:02:50	22:50	RBNU	U	1
10:54:50	14:50	RBNU	M	1
10:46:30	6:30	RBNU	U	1
11:01:40	21:40	RBNU	U	1
10:49:20	9:20	RBNU	U	1
10:57:20	17:20	RBNU	U	2
8:09:24		MOCH	M	1
9:23:40		BCCH	U	1
9:28:30		BCCH	U	1
9:19:01		BCCH	U	1
9:19:47		BCCH	M	1
9:03:20		BCCH	M	1
9:07:50		MOCH	U	1
9:40:35		MOCH	U	1

9:37:20	RBNU	M	1
9:43:00	RBNU	U	1
9:34:00	RBNU	U	1
9:00:00	BCCH	M	1
8:47:00	BCCH	M	1
8:59:30	BCCH	M	1
8:43:20	BCCH	U	1
8:52:50	BCCH	M	1
9:03:12	BCCH	M	1
8:44:30	MOCH	U	1
8:38:40	MOCH	U	1
8:42:30	MOCH	U	1
8:51:30	RBNU	M	1
8:50:00	RBNU	M	1
9:00:31	RBNU	U	1
8:53:16	RBNU	M	1
8:54:30	RBNU	M	1
8:37:30	TAHU	U	1
10:29:00	BCCH	M	1
10:25:00	BCCH	M	1
10:23:40	BCCH	M	1
10:36:30	BCCH	M	1
10:14:12	RBNU	U	1
8:25:40	MOCH	U	1
8:28:40	MOCH	U	1
8:42:40	MOCH	U	1
8:22:20	RBNU	M	1
8:22:00	RBNU	M	1
8:27:40	RBNU	U	1
8:39:10	RBNU	U	1
8:10:30	BCCH	U	1
8:09:45	BCCH	U	1
8:08:05	MOCH	U	1
8:08:55	MOCH	U	1
8:01:10	RBNU	U	1
7:43:20	RBNU	U	1
7:47:20	TAHU	U	1
8:04:30	TAHU	U	1
8:00:55	TAHU	U	1
8:26:17	BCCH	M	2
8:26:17	BCCH	F	2
8:28:45	BCCH	U	2
8:28:45	BCCH	U	2
8:31:04	BCCH	U	2

	8:34:15		BCCH	U	2
	8:25:38		BCCH	U	1
	8:28:15		BCCH	U	1
	8:23:05		BCCH	M	1
	8:13:34		TAHU	U	1
	7:59:00		BCCH	U	1
	8:21:02		MOCH	U	1
	7:55:35		MOCH	U	5
	8:01:50		MOCH	U	1
	8:13:00		MOCH	U	1
	8:21:10		RBNU	M	1
	8:23:47		RBNU	M	1
	8:24:30		RBNU	M	1
	7:55:35		RBNU	M	1
	8:09:50		RBNU	M	1
	8:10:20		RBNU	M	1
	8:20:05		RBNU	U	1
	8:07:20		RBNU	U	1
	8:06:11		TAHU	U	1
	7:58:50		TAHU	U	1
	8:11:20		TAHU	U	1
	7:55:40		TAHU	U	1
	9:50:20		BCCH	U	2
	9:50:20		BCCH	U	2
	9:45:47		BCCH	M	2
	9:45:47		BCCH	F	2
	9:58:10		RBNU	M	1
	9:57:40		RBNU	M	1
3	0:15:30	15:30	BCCH	M	
7	0:11:40	11:40	BCCH	M	
7	0:10:30	10:30	BCCH	M	
	0:20:01	20:01	BCCH	M	
7	0:07:00	7:00	BCCH	M	
3	0:16:00	16:00	MOCH	M	
	0:21:00	21:00	MOCH	M	
7	0:12:29	12:29	MOCH	M	
	9:43:44	29:44:00	MOCH	M	
	9:40:35	26:35:00	MOCH	M	
	9:42:40	28:40:00	MOCH	M	
	9:41:30	27:30:00	MOCH	M	
	9:40:30	26:30:00	MOCH	M	
	9:43:30	29:30:00	MOCH	M	
	9:37:33	23:33	MOCH	M	
	10:39:00	0:00	BCCH	M	

10:42:00	3:00	MOCH	M	
10:40:30	1:30	MOCH	M	
10:40:00	1:00	RBNU	U	
10:22:30	4:30	BCCH	M	
10:21:46	3:46	BCCH	M	
10:29:30	11:30	MOCH	F	2
10:29:30	11:30	MOCH	M	2
10:28:40	10:40	MOCH	M	
10:30:45	12:45	MOCH	M	2
10:44:00	26:00:00	MOCH	M	
10:26:25	8:25	MOCH	M	
10:41:00	23:00	MOCH	M	
10:40:50	22:50	RBNU	F	
10:40:20	22:20	RBNU	F	
10:40:00	22:00	RBNU	U	
10:45:00	27:00:00	RBNU	F	
10:49:00	31:00:00	RBNU	F	
10:33:50	15:50	RBNU	U	
10:20:11	2:11	TAHU	U	
11:56:20	17:20	MOCH	M	2
11:58:10	19:10	MOCH	M	2
11:54:55	15:55	MOCH	M	2
11:57:50	18:50	MOCH	F	2
11:54:20	15:20	MOCH	U	2
11:39:00	0:00	MOCH	M	2
11:46:10	7:10	MOCH	U	2
11:47:20	8:20	MOCH	B	2
11:53:30	14:30	MOCH	B	2
12:01:20	22:20	MOCH	B	2
11:39:00	0:00	MOCH	F	2
12:03:30	24:30:00	RBNU	U	1
12:09:30	30:30:00	RBNU	U	1
7:56:00	6:00	MOCH	M	1
7:56:20	6:20	MOCH	M	1
8:01:40	11:40	MOCH	M	1
8:01:20	11:20	MOCH	M	1
7:56:30	6:30	MOCH	M	1
8:02:15	12:15	MOCH	M	1
7:53:10	3:10	MOCH	M	1
11:33:00	19:00	MOCH	M	1
9:12:11	6:11	RBNU	M	1
9:11:00	5:00	RBNU	M	1
9:06:00	0:00	RBNU	U	1
9:19:40	13:40	RBNU	U	1

	9:29:30			23:30	RBNU	U	1
	9:27:50			21:50	RBNU	U	1
	9:21:40			15:40	RBNU	U	1
	10:49:50			9:50	BCCH	M	1
	10:46:00			6:00	BCCH	M	1
	10:51:00			11:00	BCCH	M	1
	10:44:10			4:10	BCCH	M	1
	10:52:40			12:40	BCCH	M	1
	10:40:00			0:00	BCCH	U	1
	10:43:20			3:20	BCCH	M	1
	11:01:00			21:00	BCCH	U	2
	11:05:00			25:00:00	BCCH	M	2
	11:05:00			25:00:00	BCCH	F	2
	10:57:10			17:10	BCCH	M	1
	11:00:00			20:00	RBNU	M	1
	10:59:00			19:00	RBNU	M	1
	11:00:50			20:50	RBNU	M	1
	10:58:30			18:30	RBNU	M	1
	11:05:00			25:00:00	RBNU	M	1
	10:55:36			15:36	RBNU	U	1
	10:51:48			11:48	RBNU	U	1
1				0:04:00	BCCH	F	2
1				0:04:20	BCCH	M	2
1				0:08:30	BCCH	M	2
1				0:03:00	BCCH	M	2
5				0:13:20	BCCH	B	2
13				0:20:10	BCCH	F	2
13				0:22:00	BCCH	M	2
1				0:25:00	BCCH	U	1
13				0:15:10	MOCH	U	1
13				0:12:31	MOCH	U	1
13				0:13:00	RBNU	M	1
13				0:17:30	RBNU	M	1
1				0:20:51	RBNU	M	1
5				0:08:00	RBNU	M	1
5				0:05:51	RBNU	M	1
13				0:12:50	RBNU	U	1
11		19	0	0:19:00	END		
5		2	50	0:02:50	MOCH		1
11		15	20	0:15:20	RBNU	M	1
11		14	40	0:14:40	RBNU	M	1
1		8	30	0:08:30	RBNU	M	1
11		14	50	0:14:50	RBNU	M	1
1		7	50	0:07:50	START		

5		1	50	0:01:50	START		
11		13	45	0:13:45	START		
		0	0	0:00:00	START		
11		14	0	0:14:00	END		
1		6	20	0:06:20	START		
5		1	45	0:01:45	START		
11		10	10	0:10:10	START		
		0	0	0:00:00	START		
1		17	0	0:17:00	END		
11		1	37	0:01:37	RBNU	M	1
11		4	0	0:04:00	RBNU	M	1
5		10	40	0:10:40	RBNU	M,F	1
1		15	0	0:15:00	RBNU		1
1		13	20	0:13:20	RBNU	M	1
1		13	20	0:13:20	RBNU	F	1
1		15	0	0:15:00	RBNU	F	1
1		12	55	0:12:55	START		
5		7	40	0:07:40	START		
11		1	20	0:01:20	START		
	8:08	0	0	0:00:00	START		
5		15	0	0:15:00	END		
11		2	10	0:02:10	MOCH	M	1
1		6	45	0:06:45	START		
5		10	50	0:10:50	START		
11		1	0	0:01:00	START		
	10:13	0	0	0:00:00	START		
13		0	0	0:00	RBNU	M	1
13		0	0	0:00:10	RBNU	M	1
13		0	0	0:00:00	START		
5		7	30	0:07:30	START		
1		15	0	0:15:00	START		
1		18	0	0:18:00	BCCH	M	1
1		19	20	0:19	BCCH	M	1
1		20	51	20:51	BCCH	M	1
1		21	30	21:30	BCCH	F	2
1		22	20	22:20	BCCH	M	2
1		17	0	0:17:00	MOCH	M	1
13		0	0	0:00:00	START		
5		7	30	0:07:30	START		
1		15	0	0:15:00	START		
3		16	28	0:16:28	BCCH	M	1
3		16	48	0:16:48	BCCH	M	1
3		17	40	0:17:40	BCCH	M	2
3		18	40	0:18:40	BCCH	M	2

3	19	5	0:19:05	BCCH	M	2
3	19	20	0:19:20	BCCH	M	2
3	17	40	0:17:40	BCCH	M	2
3	18	40	0:18:40	BCCH	M	2
3	19	33	0:19	BCCH	M	2
3	20	48	0:20:48	BCCH	M	2
3	21	43	0:21:43	BCCH	M	2
3	22	28	0:22:28	BCCH	M	2
3	23	44	0:23:44	BCCH	M	2
13	7	30	0:07:30	START		
3	15	3	0:15:03	START		
13	8	20	0:08:20	TRES	U	2
13	10	0	0:10:00	TRES	U	2
5	4	7	0:04:07	TRES	U	4
5	1	31	0:01:31	MOCH	M	1
5	3	1	0:03:01	MOCH	M	1
5	3	42	0:03:42	MOCH	M	1
5	3	20	0:03:20	MOCH	M	1
5	0	37	0:00:37	MOCH	M	1
5	2	15	0:02:15	MOCH	M	1
5	1	13	0:01:13	MOCH	M	1
5	4	10	0:04:10	MOCH	M	2
5	4	45	0:04:45	MOCH	M	2
5	5	26	0:05:26	MOCH	M	2
5	6	3	0:06:03	MOCH	M	2
5	7	5	0:07:05	MOCH	M	2
13	14	13	0:14:13	MOCH	M	1
13	15	26	0:15:26	MOCH	M	1
13	15	57	0:15:57	MOCH	M	1
1	20	56	0:20:56	MOCH	M	1
1	21	27	0:21:27	MOCH	M	2
1	22	20	0:22:20	MOCH	M	2
5	0	0	0:00:00	START		
13	11	13	0:11:13	START		
1	18	16	0:18:16	START		
1	9	22	0:09:22	BCCH	M	1
1	9	47	0:09:47	BCCH	M	1
1	10	26	0:10:26	BCCH	M	1
1	10	49	0:10:49	BCCH	M	1
1	11	2	0:11:02	BCCH	M	1
1	8	21	0:08:21	BCCH	M	1
1	8	36	0:08:36	BCCH	F	1
13	15	3	0:15:03	BCCH	M	1
1	7	56	0:07:56	BCCH	M	1

13	18	46	0:18:46	BCCH	M	1
13	20	1	0:20:01	BCCH	M	1
13	21	8	0:21:08	BCCH	M	1
5	1	55	0:01:55	BCCH	M	1
1	12	2	0:12:02	BCCH	M	1
1	13	19	0:13:19	BCCH	M	1
1	14	32	0:14:32	BCCH	M	1
13	18	7	0:18:07	BCCH	M	1
5	2	16	0:02:16	BOCH	U	1
5	5	42	0:05:42	BOCH	U	1
13	17	52	0:17:52	CHIC	U	1
5	3	15	0:03:15	MOCH	F	1
5	3	7	0:03:07	MOCH	F	1
5	4	13	0:04:13	MOCH	F	1
5	4	38	0:04:38	MOCH	F	1
5	5	42	0:05:42	MOCH	F	1
1	8	40	0:08:40	MOCH	M	1
5	6	29	0:06:29	MOCH	F	1
5	7	14	0:07:14	MOCH	F	1
5	0	0	0:00:00	START		
1	7	32	0:07:32	START		
13	15	2	0:15:02	START		
1	21	44	0:21:44	CHIC	U	1
13	3	35	0:03:35	RBNU	M	2
13	3	3	0:03:03	RBNU	M	2
13	2	12	0:02:12	RBNU	F	2
13	2	23	0:02:23	RBNU	F	2
13	1	27	0:01:27	RBNU	F	2
13	1	7	0:01:07	RBNU	M	1
13	4	52	0:04:52	RBNU	M	2
5	13	30	0:13:30	RBNU	M	3
5	13	30	0:13:30	RBNU	U	3
5	13	30	0:13:30	RBNU	U	3
5	12	59	0:12:59	RBNU	M	1
13	0	0	0:00:00	START		
5	7	40	0:07:40	START		
1	14	55	0:14:55	START		
5	17	41	0:17:41	MOCH	M	1
5	16	16	0:16:16	MOCH	M	1
5	17	12	0:17:12	MOCH	M	1
5	17	33	0:17:33	MOCH	M	1
5	15	55	0:15:55	MOCH	M	1
5	15	37	0:15:37	MOCH	M	1
5	18	50	0:19:50	MOCH	M	1

5	19	36	0:19:36	MOCH	M	1
5	20	30	0:20:30	MOCH	M	1
5	21	21	0:21:21	MOCH	M	1
5	22	0	0:22:00	MOCH	M	1
13	12	3	0:12:03	RBNU	M	1
13	13	8	0:13:08	RBNU	M	1
5	20	58	0:29:58	RBNU	U	1
13	10	44	0:10:44	RBNU	M	1
13	11	28	0:11:28	RBNU	M	1
5	22	0	0:22:00	RBNU	M	1
1	0	0	0:00:00	start		
13	7	30	0:07:30	start		
5	15	0	0:15:00	start		
13	0	0	0:00:00	start		
5	7	30	0:07:30	start		
1	15	2	0:15:02	start		
5	19	27	0:19:27	MOCH	M	1
5	16	24	0:16:24	MOCH	M	1
5	17	1	0:17:01	MOCH	M	1
5	17	40	0:17:40	MOCH	M	1
5	17	53	0:17:53	MOCH	M	1
5	16	0	0:16:00	MOCH	M	1
5	15	43	0:15:43	MOCH	M	1
13	2	33	0:02:33	MOCH	M	1
13	2	58	0:02:58	MOCH	M	1
13	6	3	0:06:03	MOCH	M	1
13	6	22	0:06:22	MOCH	M	1
5	20	3	0:20:03	MOCH	M	1
13	0	0	0:00:00	start		
1	7	30	0:07:30	start		
5	12	30	0:12:30	start		
1	16	7	0:16:07	BCCH	M	1
1	18	38	0:18:38	BCCH	M	2
1	15	41	0:15:41	BCCH	M	1
5	2	52	0:02:52	MOCH	M	2
5	3	20	0:03:20	MOCH	M	2
5	3	20	0:03:20	MOCH	U	2
5	5	15	0:05:15	MOCH	M	2
5	5	15	0:05:15	MOCH	U	2
5	1	13	0:01:13	MOCH	U	1
5	6	25	0:06:25	MOCH	M	2
5	6	25	0:06:25	MOCH	U	2
5	0	0	0:00:00	MOCH	U	1
5	0	37	0:00:37	MOCH	U	1

13		9	8	0:09:08	MOCH	U	2
13		13	18	0:13:18	MOCH	M	2
13		13	18	0:13:18	MOCH	U	2
1		17	42	0:17:42	MOCH	M	2
1		18	51	0:18:51	MOCH	M	1
1		19	12	0:19:12	MOCH	M	2
1		19	12	0:19:12	MOCH	M	2
1		19	31	0:19:31	MOCH	M	2
1		19	57	0:19:57	MOCH	M	2
1		20	44	0:20:44	MOCH	M	2
1		20	44	0:20:44	MOCH	M	2
1		20	59	0:20:59	MOCH	M	2
5		3	30	0:03:30	MOCH	U	3
13		8	3	0:08:03	MOCH	M	1
13		9	30	0:09:30	MOCH	M	3
13		9	30	0:09:30	MOCH	U	3
1		21	31	0:21:31	MOCH	M	2
13		10	34	0:10:34	MOCH	M	1
13		11	59	0:11:59	MOCH	M	1
1		22	21	0:22:21	MOCH	M	2
1		22	50	0:22:50	MOCH	M	1
5		1	48	0:01:48	MOCH	M	2
13		12	27	0:12:27	MOCH	M	1
13		7	40	0:07:40	start		
1		15	1	0:15:01	start		
5	9:15	4	16	0:04:16	CHIC	U	1
13	9:19	8	26	0:08:26	MOCH	M	1
13	9:20	9	40	0:09:40	MOCH	M	1
13	9:20	9	5	0:09:05	RBNU	M	1
13	9:20	9	58	0:09:58	RBNU	M	1
13	9:22	11	9	0:11:09	RBNU	M	2
5	9:15	4	41	0:04:41	RBNU	M	1
5	9:15	4	51	0:04:51	RBNU	M	1
13	9:18	7	41	0:07:41	RBNU	M	1
5	9:11	0	51	0:00:51	RBNU	M	1
5	9:12	1	20	0:01:20	RBNU	M	1
13	9:21	10	35	0:10:35	RBNU	M	2
13	9:22	11	9	0:11:09	RBNU	M	2
5	9:11	0	0	0:00:00	start		
13	9:18	7	30	0:07:30	START		
1	9:26	15	0	0:15:00	start		
1	9:30	19	35	0:19:35	stop		
1	9:28	17	4	0:17:04	YRWA	U	1
5	10:53	4	37	0:04:37	CHIC		2

5	10:51	2	51	0:02:51	CHIC		1
13	11:06	17	51	0:17:51	RBNU	M	1
13	11:05	18	54	0:18:54	RBNU	M	1
13	11:06	19	17	0:19:17	RBNU	M	1
5	10:49	0	0	0:00:00	START		
1	10:56	7	30	0:07:30	START		
13	11:04	15	0	0:15:00	START		
13	11:07	20	0	0:20:00	STOP		
3	8:36	46	30	0:46:30	BCCH	M	1
3	8:33	43	36	0:43:36	BCCH	M	1
3	8:37	47	39	0:47:39	BCCH	M	1
3	8:33	43	0	0:43:00	BCCH	M	1
3	8:39	49	48	0:49:48	BCCH	M	1
3	8:33	43	53	0:43:53	MOCH	M	1
5	8:30	40	32	0:40:32	MOCH	M	1
5	8:31	41	32	0:41:32	MOCH	M	1
3	8:35	45	50	0:45:50	MOCH	M	1
3	8:36	46	10	0:46:10	MOCH	M	1
3	8:35	45	0	0:45:00	MOCH	M	1
3	8:39	49	48	0:49:48	MOCH	M	1
13	7:54	4	38	0:04:38	MOCH	M	1
3	8:32	42	30	0:42:30	MOCH	M	1
5	8:27	37	20	0:37:20	MOCH	M	1
13	7:52	2	14	0:02:14	RBNU	M	1
13	7:54	4	13	0:04:13	RBNU	M	1
13	7:52	2	26	0:02:26	RBNU	M	1
13	7:53	3	22	0:03:22	RBNU	M	1
13	7:55	5	0	0:05:00	RBNU	M	1
13	7:55	5	21	0:05:21	RBNU	M	1
13	7:56	6	13	0:06:13	RBNU	M	1
13	7:58	8	25	0:08:25	RBNU	M	1
13	7:58	8	56	0:08:56	RBNU	M	1
13	7:59	9	37	0:09:37	RBNU	M	1
13	8:02	12	13	0:12:13	RBNU	M	1
13	8:02	12	30	0:12:30	RBNU	M	1
13	8:05	15	42	0:15:42	RBNU	M	2
13	8:06	16	28	0:16:28	RBNU	M	2
13	8:11	21	26	0:21:26	RBNU	F	2
13	8:14	24	10	0:24:10	RBNU	F	2
5	8:26	36	10	0:36:10	RBNU	M	2
5	8:26	36	43	0:36:43	RBNU	M	2
5	8:31	41	45	0:41:45	RBNU	M	1
5	8:32	42	0	0:42:00	RBNU	M	1
3	8:40	50	47	0:50:47	RBNU	M	1

3	8:41	51	27	0:51:27	RBNU	M	1
13	7:51	1	23	0:01:23	RBNU	M	1
13	7:51	1	55	0:01:55	RBNU	M	1
13	7:53	3	38	0:03:38	RBNU	M	1
13	8:01	11	55	0:11:55	RBNU	M	1
13	8:03	13	43	0:13:43	RBNU	F	2
13	8:05	15	42	0:15:42	RBNU	M	2
13	7:50	0	56	0:00:56	RBNU	M	1
13	8:03	13	38	0:13:38	RBNU	F	2
13	8:20	30	0	0:30:00	RBNU		2
13	7:50	0	24	0:00:24	RBNU	M	1
13	8:17	27	42	0:27:42	RBNU	F	1
13	7:56	6	2	0:06:02	RBNU	M	1
13	8:06	16	28	0:16:28	RBNU	F	2
13	8:07	17	56	0:17:56	RBNU	M	2
13	8:07	17	56	0:17:56	RBNU	F	2
13	8:12	22	31	0:22:21	RBNU	F	2
13	8:18	28	10	0:28:10	RBNU	F	1
3	8:37	47	36	0:47:36	RBNU	M	1
3	8:38	48	14	0:48:14	RBNU	M	1
5	8:30	40	29	0:40:29	RBNU	M	1
5	8:29	39	48	0:39:48	RBNU	M	1
5	8:26	36	43	0:36:43	RBNU	F?	2
3	8:43	53	13	0:53:13	RBNU	M	2
3	8:39	49	48	0:49:48	RBNU	M	1
5	8:31	41	2	0:41:02	RBNU	M	1
3	8:34	44	2	0:44:02	RBNU	M	1
13	7:50	0	0	0:00:00	START		
5	8:25	35	0	0:35:00	START		
3	8:32	42	0	0:42:00	START		
3	8:50	60	0	1:00:00	stop		
	7:50	0	0	0:00:00			
13	0:16	16	0	0:16:00	RBNU	M	1
13	0:19	19	47	0:19:47	RBNU	M	1
13	0:15	15	20	0:15:20	RBNU	M	1
13	0:17	17	25	0:17:25	RBNU	M	1
13	0:19	19	10	0:19:10	RBNU	M	1
5	0:08	8	37	0:08:37	RBNU	M	1
5	0:09	9	19	0:09:19	RBNU	M	1
5	0:10	10	23	0:10:23	RBNU	M	1
5	0:12	12	4	0:12	RBNU	M	1
1	11:18	0	0	0:00:00	START		
5	0:07	7	30	0:07:30	START		
13	0:15	15	0	0:15:00	START		

5	0:09	9	32	0:09:32	BCCH	B	2
5	0:08	8	19	0:08:19	BCCH	M	1
5	0:09	9	14	0:09:14	BCCH	F	2
5	0:10	10	38	0:10:38	BCCH	B	2
5	0:11	11	7	0:11:07	BCCH	M	2
5	0:07	7	49	0:07:49	BCCH	M	1
1	0:00	0	31	0:00:31	BCCH	M	1
1	0:01	1	59	0:01:59	BCCH	M	1
13	10:23				END		
13	0:16	16	42	0:16:42	RBNU	M	1
13	0:17	17	0	0:17:00	RBNU	M	1
13	0:17	17	22	0:17:22	RBNU	M	1
13	0:16	16	33	0:16:33	RBNU	M	1
1	0:04	4	2	0:04:02	RBNU	M	1
13	0:17	17	23	0:17:23	RBNU	B	2
13	0:18	18	9	0:18:09	RBNU	M	2
13	0:18	18	31	0:18:31	RBNU	M	2
13	0:19	19	11	0:19:11	RBNU	M	2
1	0:03	3	36	0:03:36	RBNU	M	1
5	0:12	12	20	0:12:20	RBNU	M	1
5	0:11	11	6	0:11:06	RBNU	M	1
1	0:00	0	51	0:00:51	RBNU	M	1
1	0:01	1	37	0:01:37	RBNU	M	1
1	0:02	2	18	0:02:18	RBNU	M	1
1	0:03	3	4	0:03:04	RBNU	M	2
1	9:58	0	0	0:00:00	START		
5	0:07	7	30	0:07:30	START		
13	0:15	15	7	0:15:07	START		
1	10:35	10	32	0:10:32	MOCH	M	1
13	10:26	1	8	0:01:08	RBNU	F	2
13	10:27	2	17	0:02:17	RBNU	M	2
13	10:27	2	37	0:02:37	RBNU	M	2
13	10:28	3	8	0:03:08	RBNU	B	2
13	10:29	4	49	0:04:49	RBNU	B	2
13	10:26	1	4	0:01:04	RBNU	M	2
13	10:31	6	55	0:06:55	RBNU	B	2
13	10:14	0	0	0:00:00	START		
1	10:32	7	30	0:07:30	START		
5	10:39	14	0	0:14:00	START		
1	10:34	19	40	0:19:40	BCCH	M	1
1	10:34	19	40	0:19:40	BCCH	M	1
1	10:39	24	25	0:24:25	BCCH	M	1
1	10:39	24	25	0:24:25	BCCH	M	1
1	10:32	17	33	0:17:33	BCCH	M	1

1	10:32	17	33	0:17:33	BCCH	M	1
1	10:40	25	20	0:25:20	BCCH		1
1	10:40	25	20	0:25:20	BCCH	U	1
13	10:27	12	18	0:12:18	HAWO	M	1
13	10:27	12	18	0:12:18	HAWO	M	1
1	10:31	16	25	0:16:25	HAWO	F	1
1	10:31	16	25	0:16:25	MAWO	F	1
5	10:17	2	54	0:02:54	MOCH	M	1
5	10:18	3	1	0:03:01	MOCH	M	1
5	10:17 AM	2	54	0:02:54	MOCH	M	1
5	10:16	1	24	0:01:24	MOCH	M	1
5	10:17	2	10	0:02:10	MOCH	M	1
5	10:20	5	1	0:05:01	MOCH	M	1
5	10:16 AM	1	24	0:01:24	MOCH	M	1
5	10:17 AM	2	10	0:02:10	MOCH	M	1
5	10:20 AM	5	1	0:05:01	MOCH	M	1
13	10:29	14	44	0:14:44	MOCH	M	1
1	10:31	16	4	0:16:04	MOCH	M	1
13	10:29	14	44	0:14:44	MOCH	M	1
1	10:31	16	4	0:16:04	MOCH	M	1
1	10:33	18	1	0:18:01	PIWO		1
1	10:33	18	1	0:18:01	PIWO	U	1
13	10:25	10	2	0:10:02	RBNU	M	1
13	10:25	10	2	0:10:02	RBNU	M	1
13	10:24	9	26	0:09:26	RBNU	M	1
13	10:24	9	26	0:09:26	RBNU	M	1
13	10:26	11	12	0:11:12	RBNU	M	1
13	10:26	11	12	0:11:12	RBNU	M	1
1	10:36	21	47	0:21:47	RBNU		2
1	10:36	21	47	0:21:47	RBNU	U	2
1	10:35	20	35	0:20:35	RBNU	M	1
1	10:35	20	35	0:20:35	RBNU	M	1
5	10:15	0	47	0:00:47	RBNU		1
5	10:18	3	39	0:03:39	RBNU	M	1
1	10:32	17	1	0:17:01	RBNU	M	1
5	10:15 AM	0	47	0:00:47	RBNU	U	1
5	10:18 AM	3	39	0:03:39	RBNU	M	1
1	10:32	17	1	0:17:01	RBNU	M	1
13	10:22	7	30	0:07:30	start		
1	10:30	15	0	0:15:00	start		
5	10:15 AM	0	0	0:00:00	START		
13	10:22 AM	7	30	0:07:30	START	U	
1	10:30	15	0	0:15:00	START		
1	10:40	25	20	0:25:20	stop		

	10:15	0	0	0:00:00			
1	9:46	1	51	0:01:51	BCCH	M	1
1	9:47	2	43	0:02:43	BCCH	M	1
1	9:48	3	48	0:03:48	BCCH	M	1
5	9:56	11	51	0:11:51	BCCH	M	1
5	9:53	8	11	0:08:11	BCCH	M	2
5	9:57	12	22	0:12:22	MOCH	M	2
5	9:54	9	8	0:09:08	MOCH	M	1
5	9:55	10	48	0:10:48	MOCH	M	1
5	9:57	12	44	0:12:44	MOCH	M	2
5	9:54	9	55	0:09:55	MOCH	M	1
5	9:55	10	23	0:10:23	MOCH	M	1
5	9:58	13	23	0:13:23	MOCH	M	2
13	10:01	16	40	0:16:40	MOCH	M	2
5	9:58	13	36	0:13:36	MOCH	M	2
13	10:03	18	30	0:18:30	MOCH	M	1
13	10:00	15	51	0:15:51	MOCH	M	2
13	10:05	20	34	0:20:34	MOCH	M	2
1	9:48	3	1	0:03:01	MOCH	U	1
1	9:47	2	0	0:02:00	MOCH	M	1
1	9:50	5	34	0:05:34	MOCH	M	2
1	9:48	3	48	0:03:48	MOCH	M	2
1	9:50	5	34	0:05:34	MOCH	M	2
5	9:53	8	3	0:08:03	MOCH	M	2
13	10:00	15	36	0:15:36	MOCH	M	2
13	10:05	20	34	0:20:34	MOCH	M	2
5	9:57	12	22	0:12:22	MOCH	M	2
13	10:04	19	31	0:19:31	RBNU	M	1
13	10:07	22	11	0:22:11	RBNU	M	1
1	9:45	0	0	0:00:00	START		
5	9:52	7	30	0:07:30	START		
13	10:00	15	15	0:15:15	START		
5	9:53	8	35	0:08:35	TRES	U	1
5	10:03	6	59	0:06:59	MOCH		1
5	10:03	6	59	0:06:59	MOCH	U	1
1	9:59	2	54	0:02:54	MOCH	M	1
1	9:59	2	54	0:02:54	MOCH	M	1
5	10:04	7	30	0:07:30	MOCH		1
13	10:09	12	58	0:12:58	RBNU	M	1
13	10:09	12	58	0:12:58	RBNU	M	1
13	10:11	14	5	0:14:05	RBNU	M	1
13	10:11	14	5	0:14:05	RBNU	M	1
13	10:11	14	34	0:14:34	RBNU	M	1
13	10:11	14	34	0:14:34	RBNU	M	1

1	10:01	4	0	0:04:00	RBNU	M	1
1	10:01	4	0	0:04:00	RBNU	M	1
13	10:12	15	33	0:15:33	RBNU	M	1
13	10:12	15	33	0:15:33	RBNU	M	1
1	9:58	1	21	0:01:21	RNSA	M	1
1	9:58	1	21	0:01:21	RNSA	M	1
1	9:57	0	0	0:00:00	start		
5	10:02	5	40	0:05:40	start		
13	10:09	12	21	0:12:21	start		
1	9:57	0	0	0:00:00	START		
5	10:02	5	40	0:05:40	START		
13	10:09	12	21	0:12:21	START		
13	10:12	15	33	0:15:33	stop		
	9:57	0	0	0:00:00			
1	8:37	17	24	0:17:24	BCCH	M	1
1	8:37	17	8	0:17:08	BCCH	M	1
1	8:36	16	30	0:16:30	BCCH	M	1
1	8:39	19	1	0:19:01	BCCH	M	1
1	8:35	15	48	0:15:48	BCCH	M	1
5	8:30	10	12	0:10:12	MOCH	M	2
5	8:30	10	50	0:10:50	MOCH	M	2
5	8:29	9	56	0:09:56	MOCH	M	2
1	8:37	17	8	0:17:08	MOCH	M	2
5	8:29	9	13	0:09:13	MOCH	M	1
5	8:29	9	38	0:09:38	MOCH	M	1
5	8:32	12	43	0:12:43	MOCH	M	2
5	8:29	9	50	0:09:50	MOCH	F	2
5	8:31	11	19	0:11:19	MOCH	F	2
5	8:33	13	10	0:13:10	MOCH	F	2
5	8:34	14	40	0:14:40	MOCH	F	2
1	8:36	16	30	0:16:30	MOCH	M	2
5	8:32	12	43	0:12:43	MOCH	F	2
5	8:33	13	10	0:13:10	MOCH	M	2
1	8:39	19	30	0:19:30	MOCH	M	1
13	8:20	0	0	0:00:00	START		
5	8:27	7	30	0:07:30	START		
1	8:35	15	0	0:15:00	START		
5		1	47	0:01:47	BCCH	M	1
5		0	22	0:00:22	MOCH	M	1
1		9	4	0:09:04	MOCH	M	2
1		9	4	0:09:04	MOCH	F	2
5		2	20	0:02:20	MOCH	M	1
1		7	30	0:07:30	MOCH	M	1
1		17	38	0:17:38	MOCH	M	1

5		1	56	0:01:56	MOCH	M	1
5		6	21	0:06:21	MOCH	M	1
1		19	20	0:19:20	MOCH	M	2
1		14	21	0:14:21	MOCH	M	1
5		1	47	0:01:47	MOCH	M	1
1		10	19	0:10:19	MOCH	M	2
1		10	19	0:10:19	MOCH	F	2
5		0	22	0:00:22	RBNU	M	1
1		18	31	0:18:31	RBNU	M	1
1		18	28	0:18:28	RBNU	M	1
1		11	51	0:11:51	RBNU	M	1
1		12	11	0:12:11	RBNU	M	1
1		11	5	0:11:05	RBNU	M	1
1		8	0	0:08:00	RBNU	M	1
1		20	11	0:20:11	RBNU	M	2
1		16	37	0:16:37	RBNU	M	1
1		17	13	0:17:13	RBNU	M	1
1		18	7	0:18:07	RBNU	M	1
1		9	45	0:09:45	RBNU	M	1
1		10	19	0:10:19	RBNU	M	1
5		5	2	0:05:02	RBNU	M	1
5		4	28	0:04:28	RBNU	M	1
1		8	21	0:08:21	RBNU	M	1
1		21	0	0:21:00	RBNU	M	1
1		21	38	0:21:38	RBNU	M	1
5		5	36	0:05:36	RBNU	M	1
1		20	11	0:20:11	RBNU	M	2
1		20	30	0:20:30	RBNU	M	2
5		0	0	0:00:00	START		
1		7	30	0:07:30	START		
1	9:47	1	44	0:01:44	BCCH	M	1
5	10:01	15	17	0:15:17	MOCH	M	1
5	9:56	10	30	0:10:30	MOCH	M	1
5	10:01	15	55	0:15:55	MOCH	M	1
5	9:57	11	50	0:11:50	MOCH	M	1
5	10:05	19	21	0:19:21	MOCH	M	1
5	9:58	12	31	0:12:31	MOCH	M	1
5	10:03	17	3	0:17:03	MOCH	M	1
5	10:04	18	4	0:18:04	MOCH	M	1
13	10:10	24	23	0:24:23	MOCH	U	1
13	10:13	27	37	0:27:37	MOCH	U	2
13	10:12	26	38	0:26:38	MOCH	M	1
5	10:07	21	35	0:21:35	MOCH	M	1
13	10:09	23	7	0:23:07	RBNU	M	1

13	10:08	22	38	0:22:38	RBNU	M	1
5	10:01	15	1	0:15:01	RBNU	U	1
13	10:11	25	20	0:25:20	RBNU	M	1
1	9:46	0	0	0:00:00	START		
5	9:51	5	0	0:05:00	START		
13	10:08	22	15	0:22:15	START		
5	11:07	7	0	0:07:00	MOCH	M	1
5	11:08	8	2	0:08:02	MOCH	M	1
5	11:09	9	5	0:09:05	MOCH	M	1
5	11:09	9	29	0:09:29	MOCH	M	1
5	11:10	10	15	0:10:15	MOCH	M	1
5	11:12	12	30	0:12:30	MOCH	M	1
5	11:05	5	57	0:05:57	MOCH	M	1
5	11:11	11	21	0:11:21	MOCH	M	1
5	11:14	14	17	0:14:17	RBNU	M	2
5	11:14	14	51	0:14:51	RBNU	M	2
5	11:16	16	42	0:16:42	RBNU	M	1
5	11:12	12	53	0:12:53	RBNU	M	1
5	11:15	15	50	0:15:50	RBNU	M	1
5	11:07	7	31	0:07:31	RBNU	M	1
5	11:15	15	2	0:15:02	RBNU	M	1
5	11:10	10	15	0:10:15	RBNU	M	1
5	11:13	13	58	0:13:58	RBNU	U	2
5	11:06	6	25	0:06:25	RBNU	M	1
5	11:09	9	18	0:09:18	RBNU	M	1
5	11:11	11	36	0:11:36	RBNU	M	1
5	11:13	13	13	0:13:13	RBNU	M	1
5	11:05	5	28	0:05:28	RBNU	M	1
5	11:18	18	1	0:18:01	RBNU	M	1
1	11:00	0	0	0:00:00	START		
5	11:05	5	0	0:05:00	START		
13	9:57	17	0	0:17:00	END		
13	9:53	13	0	0:13:00	RBNU	M	1
13	9:52	12	20	0:12:20	RBNU	M	1
13	9:52	12	0	0:12:00	RBNU	M	1
13	9:54	14	40	0:14:40	RBNU	M	2
5	9:45	5	28	0:05:28	RBNU	M	1
5	9:47	7	10	0:07:10	RBNU	M	1
5	9:48	8	50	0:08:50	RBNU	M	1
13	9:51	11	40	0:11:40	RBNU	M	1
1	9:43	3	40	0:03:40	RBNU	M	1
13	9:55	15	50	0:15:50	RBNU	M	2
1	9:42	2	20	0:02:20	RBNU	M	1
5	9:48	8	20	0:08:20	RBNU	M	1

13	9:53	13	40	0:13:40	RBNU	M	2
13	9:55	15	50	0:15:50	RBNU	M	2
1	9:40	0	35	0:00:35	START		
5	9:45	5	0	0:05:00	START		
13	9:51	11	28	0:11:28	START		
	9:40	0	0	0:00:00	START		
1	11:06	6	59	0:06:59	BCCH	M	1
13	11:10	10	6	0:10:06	MOCH	M	1
5	11:00	0	0	0:00:00	START		
1	11:04	4	0	0:04:00	START		
13	11:09	9	45	0:09:45	START		
13		0	58	0:00:58	RBNU	M	1
13		1	31	0:01:31	RBNU	M	1
13		2	2	0:02:02	RBNU	F	2
13		0	51	0:00:51	RBNU	M	1
13		3	14	0:03:14	RBNU	M	1
13		4	29	0:04:29	RBNU	M	1
5		7	40	0:07:40	RBNU	M	1
5		8	37	0:08:37	RBNU	M	1
13		5	17	0:05:17	RBNU	M	1
5		11	7	0:11:07	RBNU	M	1
1		16	10	0:16:10	RBNU	M	1
1		17	19	0:17:19	RBNU	M	1
13		2	17	0:02:17	RBNU	F	2
5		10	3	0:10:03	RBNU	M	1
13		0	0	0:00:00	START		
5		7	30	0:07:30	START		
1		15	2	0:15:02	START		
5	7:58	16	55	0:16:55	MOCH	M	1
5	7:57	15	30	0:15:30	MOCH	U	1
1	7:42	0	46	0:00:46	MOCH	U	1
5	8:00	18	1	0:18:01	MOCH	M	1
13	7:50	8	47	0:08:47	RBNU	M	1
13	7:50	8	21	0:08:21	RBNU	M	1
13	7:49	7	36	0:07:36	RBNU	M	1
13	7:48	6	18	0:06:18	RBNU	M	1
13	7:48	6	31	0:06:31	RBNU	M	1
13	7:50	8	35	0:08:35	RBNU	M	1
5	7:59	17	13	0:17:13	RBNU	M	1
5	7:58	16	58	0:16:58	RBNU	M	1
13	7:47	5	44	0:05:44	RBNU	M	1
5	8:00	18	38	0:18:38	RBNU	M	1
13	7:47	5	37	0:05:37	RBNU	M	1
13	7:49	7	7	0:07:07	RBNU	M	1

5	7:58	16	3	0:16:03	RBNU	M	1
5	8:03	21	53	0:21:53	RBNU	M	1
5	8:05	23	16	0:23:16	RBNU	M	1
5	7:57	15	15	0:15:15	RBNU	M	1
5	8:01	19	35	0:19:35	RBNU	M	1
13	7:54	12	1	0:12:01	RBNU	M	1
5	7:56	14	58	0:14:58	RBNU	M	1
5	8:01	19	46	0:19:46	RBNU	M	1
5	8:06	24	20	0:24:20	RBNU	M	1
5	8:13	31	12	0:31:12	RBNU	M	1
13	7:52	10	45	0:10:45	RBNU	M	1
13	7:53	11	19	0:11:19	RBNU	M	1
5	8:06	24	8	0:24:08	RBNU	M	1
5	8:10	28	8	0:28:08	RBNU	M	1
1	7:42	0	0	0:00:00	START		
13	7:47	5	0	0:05:00	START		
5	7:56	14	30	0:14:30	START		
5	8:41	15	0	0:15:00	END		
1	8:32	6	50	0:06:50	MOCH	U	1
1	8:34	8	50	0:08:50	MOCH	U	1
13	8:29	3	40	0:03:40	MOCH	U	1
1	8:31	5	33	0:05:33	START		
5	8:36	10	13	0:10:13	START		
13	8:26	0	0	0:00:00	START		
13	9:50	17	0	0:17:00	END		
5	9:36	3	50	0:03:50	MOCH	U	1
5	9:37	4	20	0:04:20	MOCH	U	1
5	9:36	3	10	0:03:10	MOCH	U	1
5	9:36	3	0	0:03:00	MOCH	U	1
5	9:39	6	0	0:06:00	MOCH	U	1
1	9:40	7	16	0:07:16	START		
5	9:34	1	10	0:01:10	START		
13	9:46	13	8	0:13:08	START		
	9:33	0	0	0:00:00	START		
5	9:58	0	0	0:00:00	START		
1	10:03	5	0	0:05:00	START		
13	10:07	9	10	0:09:10	START		
5	10:11	11	17	0:11:17	MOCH	M	1
5	10:11	11	38	0:11:38	MOCH	M	1
5	10:13	13	17	0:13:17	RBNU	M	1
13	10:00	0	0	0:00:00	START		
1	10:05	5	0	0:05:00	START		
5	10:09	9	0	0:09:00	START		
1	9:04	7	49	0:07:49	BCCH	M	1

1	9:04	7	55	0:07:55	BCCH	M	1
1	9:05	8	51	0:08:51	BCCH	M	1
1	9:04	7	30	0:07:30	BCCH	M	1
1	9:04	7	10	0:07:10	BCCH	M	1
1	9:02	5	41	0:05:41	BCCH	M	1
1	9:06	9	6	0:09:06	BCCH	M	1
1	9:07	10	1	0:10:01	BCCH	M	1
1	9:03	6	4	0:06:04	BCCH	M	1
1	9:03	6	51	0:06:51	BCCH	M	
1	9:03	6	57	0:06:57	BCCH	M	1
5	8:58	1	37	0:01:37	RNSA		1
5	8:57	0	0	0:00:00	START		
1	9:01	4	45	0:04:45	START		
13	9:09	12	5	0:12:05	START		
5	8:10	1	56	0:01:56	MOCH	M	1
13	8:20	11	30	0:11:30	RBNU	M	1
13	8:22	13	22	0:13:22	RBNU	M	1
13	8:23	14	25	0:14:25	RBNU	M	1
13	8:19	10	39	0:10:39	RBNU	M	1
1	8:14	5	0	0:05:00	START		
5	8:09	0	0	0:00:00	START		
13	8:18	9	0	0:09:00	START		
13	11:10	7	32	0:07:32	RBNU	M	2
1	11:24	21	13	0:21:13	RBNU	M	2
13	11:11	8	7	0:08:07	RBNU	M	2
13	11:13	10	20	0:10:20	RBNU	M	2
13	11:14	11	35	0:11:35	RBNU	M	2
13	11:18	15	44	0:15:44	RBNU	M	2
1	11:24	21	33	0:21:33	RBNU	M	2
1	11:23	20	10	0:20:10	RBNU	F	2
1	11:25	22	51	0:22:51	RBNU	M	2
13	11:09	6	55	0:06:55	RBNU	M	2
13	11:09	6	55	0:06:55	RBNU	F	2
13	11:10	7	6	0:07:06	RBNU	F	2
13	11:11	8	46	0:08:46	RBNU	M	2
13	11:17	14	2	0:14:02	RBNU	M	2
13	11:15	12	37	0:12:37	RBNU	M	2
1	11:24	21	54	0:21:54	RBNU	M	2
13	11:19	16	2	0:16:02	RBNU	M	2
13	11:08	5	2	0:05:02	RBNU	U	1
5	11:05	2	25	0:02:25	RBNU	M	1
1	11:23	20	10	0:20:10	RBNU	M	2
1	11:25	22	11	0:22:11	RBNU	M	2
1	11:26	23	43	0:23:43	RBNU	M	2

13	11:20	17	28	0:17:28	RBNU	M	2
13	11:21	18	5	0:18:05	RBNU	M	2
5	11:03	0	0	0:00:00	START		
13	11:08	5	0	0:05:00	START		
13	8:31	1	13	0:01:13	RBNU	M	1
13	8:32	2	46	0:02:46	RBNU	M	1
13	8:30	0	44	0:00:44	RBNU	M	1
1	8:40	10	0	0:10:00	START		
5	8:35	5	0	0:05:00	START		
13	8:30	0	0	0:00:00	START		
1	9:11	4	48	0:04:48	BCCH	M	1
1	9:12	6	20	0:06:20	BCCH	M	1
1	9:11	4	0	0:04:00	START		
5	9:14	8	30	0:08:30	START		
13	9:07	0	0	0:00:00	START		
13	9:07	0	25	0:00:25	TRES	U	1
1	8:10	0	0	0:00:00	START		
5	8:14	4	0	0:04:00	START		
13	8:18	8	15	0:08:15	START		
5	9:42	4	26	0:04:26	MOCH	M	1
5	9:43	5	41	0:05:41	MOCH	M	1
5	9:44	6	38	0:06:38	MOCH	M	1
1	9:38	0	0	0:00:00	START		
5	9:42	4	0	0:04:00	START		
13	9:47	9	0	0:09:00	START		
13	9:50	12	4	0:12:04			
1	11:10	10	0	0:10:00	START		
5	11:05	5	0	0:05:00	START		
13	11:00	0	0	0:00:00	START		
3	11:20	2	50	0:02:50	AMRO		1
5	11:29	11	10	0:11:10	MOCH		1
5	11:29	11	8	0:11:08	MOCH		1
5	11:29	11	17	0:11:17	MOCH		1
5	11:28	10	50	0:10:50	MOCH		1
5	11:30	12	3	0:12:03	MOCH		2
5	11:29	11	35	0:11:35	MOCH		1
5	11:33	15	32	0:15:32	MOCH		2
	11:18	0	27	0:00:27	RBNU		1
5	11:27	9	25	0:09:25	RBNU		1
3	11:20	2	4	0:02:04	RBNU		1
11	11:40	22	28	0:22:28	RBNU		1
3	11:26	8	48	0:08:48	RCKI		1
3	11:19	1	37	0:01:37	start		
5	11:27	9	6	0:09:06	start		

11	11:35	17	17	0:17:17	start		
11	11:41	23	51	0:23:51	stop		
11	11:36	18	50	0:18:50	YRWA		1
	11:18	0	0	0			
3	11:58	3	20	0:03:20	BCCH		1
5	12:10	15	30	0:15:30	MOCH		2
5	12:08	13	0	0:13:00	MOCH		2
3	12:02	7	48	0:07:48	MOCH		2
3	12:02	7	48	0:07:48	MOCH		2
3	11:59	4	17	0:04:17	RCKI		1
3	11:56	1	45	0:01:45	start		
11	12:03	8	50	0:08:50	start		
5	12:08	13	0	0:13:00	start		
5	12:12	17	0	0:17:00	stop		
3	12:01	6	45	0:06:45	YRWA		1
	11:55	0	0	0:00:00			
11	10:32	9	12	0:09:12	BCCH		1
11	10:34	11	4	0:11:04	BCCH		2
11	10:31	8	0	0:08:00	BCCH		1
3	10:26	3	31	0:03:31	BCCH	M	1
5	10:43	20	0	0:20:00	MOCH	M	2
5	10:38	15	20	0:15:20	MOCH		1
5	10:38	15	39	0:15:39	MOCH	M	1
5	10:41	18	54	0:18:54	MOCH		2
5	10:43	20	27	0:20:27	MOCH	M	2
5	10:42	19	46	0:19:46	MOCH		2
5	10:40	17	18	0:17:18	MOCH		2
5	10:41	18	59	0:18:59	MOCH	M	2
5	10:44	21	47	0:21:47	MOCH	M	2
11	10:35	12	45	0:12:45	MOCH	M	1
11	10:36	13	43	0:13:43	MOCH	M	1
11	10:33	10	8	0:10:08	MOCH	M	1
11	10:32	9	47	0:09:47	RBNU		1
11	10:31	8	43	0:08:43	RBNU		1
11	10:32	9	35	0:09:35	RBNU		1
11	10:33	10	48	0:10:48	RBNU		1
11	10:34	11	4	0:11:48	RBNU	M	1
11	10:35	12	17	0:12:17	RBNU		1
5	10:42	19	15	0:19:15	RBNU		1
5	10:38	15	39	0:15:39	RBNU		1
11	10:36	13	11	0:13:11	RBNU		1
5	10:41	18	32	0:18:32	RBNU		1
11	10:36	13	43	0:13:43	RBNU		1
3	10:25	2	35	0:02:35	start		

11	10:30	7	41	0:07:41	start		
5	10:37	14	37	0:14:37	start		
5	10:45	22	14	0:22:14	stop		
	10:23	0	0	0:00:00			
5	12:22	19	29	0:19:29	BCCH		1
11	12:08	5	46	0:05:46	BCCH		1
5	12:20	17	3	0:17:03	MOCH	M	1
5	12:21	18	13	0:18:13	MOCH	M	1
3	12:14	11	45	0:11:45	MOCH		2
3	12:12	9	52	0:09:52	MOCH		1
3	12:13	10	26	0:10:26	MOCH		1
3	12:15	12	55	0:12:55	MOCH		2
3	12:12	9	5	0:09:05	MOCH		1
5	12:19	16	52	0:16:52	MOCH	M	1
5	12:22	19	44	0:19:44	MOCH	M	1
3	12:13	10	42	0:10:42	MOCH		2
3	12:14	11	21	0:11:21	MOCH		2
5	12:23	20	5	0:20:05	MOCH	M	1
11	12:05	2	3	0:02:03	RBNU		1
11	12:06	3	36	0:03:36	RBNU		2
11	12:06	3	57	0:03:57	RBNU		1
11	12:09	6	28	0:06:28	RBNU		1
11	12:05	2	55	0:02:55	RBNU		2
11	12:06	3	18	0:03:18	RBNU		2
11	12:04	1	38	0:01:38	RBNU		1
11	12:07	4	13	0:04:13	RBNU		1
11	12:04	1	14	0:01:14	start		
3	12:11	8	21	0:08:21	start		
5	12:18	15	32	0:15:32	start		
5	12:24	21	51	0:21:51	stop		
	12:03	0	0	0:00:00			
3	9:24	18	58	0:18:58	BCCH	M	1
5	9:09	3	39	0:03:39	MOCH	M	1
5	9:10	4	45	0:04:45	MOCH	M	1
3	9:29	23	30	0:23:30	MOCH		1
5	9:09	3	6	0:03:06	MOCH	M	1
5	9:07	1	56	0:01:56	MOCH	M	1
5	9:08	2	31	0:02:31	MOCH	M	1
5	9:11	5	10	0:05:10	MOCH	M	1
5	9:11	5	30	0:05:30	MOCH	M	1
11	9:17	11	34	0:11:34	MOCH	M	1
5	9:10	4	30	0:04:30	MOCH	M	1
11	9:14	8	50	0:08:50	MOCH		1
3	9:25	19	52	0:19:52	MOCH	M	1

3	9:27	21	22	0:21:22	MOCH	M	1
11	9:19	13	58	0:13:58	RBNU		1
11	9:16	10	37	0:10:37	RBNU		1
11	9:19	13	18	0:13:18	RBNU		1
11	9:19	13	42	0:13:42	RBNU		1
11	9:20	14	10	0:14:10	RBNU		1
3	9:22	16	53	0:16:53	RBNU		1
11	9:16	10	9	0:10:09	RBNU		1
3	9:23	17	0	0:17:00	RBNU		1
11	9:17	11	44	0:11:44	RBNU		1
11	9:17	11	0	0:11:00	RBNU		1
11	9:17	11	19	0:11:19	RBNU		1
11	9:19	13	6	0:13:06	RBNU		1
3	9:23	17	29	0:17:29	RBNU		1
11	9:15	9	43	0:09:43	RBNU		1
3	9:25	19	29	0:19:29	RBNU		1
3	9:24	18	39	0:18:39	RBNU		1
5	9:12	6	8	0:06:08	RBNU		1
3	9:27	21	39	0:21:39	RBNU		1
3	9:28	22	14	0:22:14	RBNU		2
5	9:07	1	15	0:01:15	start		
11	9:14	8	30	0:08:30	start		
3	9:22	16	30	0:16:30	start		
3	9:30	24	22	0:24:22	stop		
	9:06	0	0	0:00:00			
3	10:46	10	57	0:10:57	MOCH	M	1
3	10:48	12	10	0:12:10	MOCH	M	2
5	10:52	16	45	0:16:45	MOCH	M	1
5	10:53	17	11	0:17:11	MOCH	M	1
11	10:38	2	56	0:02:56	RBNU		1
11	10:41	5	24	0:05:24	RBNU		1
11	10:38	2	3	0:02:03	RBNU		1
11	10:37	1	41	0:01:41	RBNU		1
3	10:44	8	1	0:08:01	RBNU		1
3	10:44	8	29	0:08:29	RBNU		1
5	10:51	15	44	0:15:44	RBNU		1
5	10:53	17	23	0:17:23	RBNU		1
3	10:46	10	13	0:10:13	RBNU		1
5	10:53	17	56	0:17:56	RBNU		1
11	10:36	0	37	0:00:37	start		
3	10:43	7	55	0:07:55	start		
5	10:51	15	16	0:15:16	start		
5	10:56	20	35	0:20:35	stop		
	10:36	0	0	0:00:00			

3	10:53	16	15	0:16:15	BCCH	M	1
3	10:55	18	35	0:18:35	BCCH	M	1
5	10:41	4	20	0:04:20	MOCH	M	1
5	10:40	3	9	0:03:09	MOCH	M	1
5	10:42	5	2	0:05:02	MOCH	M	1
3	10:56	19	11	0:19:11	MOCH	M	1
5	10:39	2	48	0:02:48	MOCH	M	1
3	10:55	18	31	0:18:31	MOCH	M	1
5	10:39	2	22	0:02:22	MOCH	M	1
3	10:54	17	49	0:17:49	MOCH	M	1
11	10:49	12	3	0:12:03	RBNU		2
3	10:55	18	12	0:18:12	RBNU		1
11	10:48	11	25	0:11:25	RBNU		2
11	10:48	11	15	0:11:15	RBNU		1
11	10:51	14	42	0:14:42	RBNU		2
5	10:38	1	46	0:01:46	start		
11	10:45	8	15	0:08:15	start		
3	10:52	15	30	0:15:30	start		
3	10:57	20	37	0:20:37	stop		
	10:37	0	0	0:00:00			
5	13:01	19	9	0:19:09	BCCH		2
5	12:59	17	13	0:17:13	BCCH		2
5	12:57	15	7	0:15:07	BCCH		2
3	12:52	10	58	0:10:58	BCCH	M	1
3	12:54	12	43	0:12:43	BCCH		2
3	12:52	10	8	0:10:08	BCCH	M	2
3	12:52	10	19	0:10:19	BCCH	M	2
5	12:59	17	57	0:17:57	BCCH		2
3	12:51	9	44	0:09:44	BCCH	M	2
5	13:00	18	23	0:18:23	BCCH		2
5	12:57	15	32	0:15:32	BCCH		2
3	12:51	9	15	0:09:15	BCCH	M	2
3	12:50	8	36	0:08:36	BCCH	M	2
5	13:00	18	34	0:18:34	BCCH		2
5	12:57	15	52	0:15:52	BCCH		2
3	12:50	8	0	0:08:00	BCCH	M	2
3	12:52	10	33	0:10:33	BCCH		1
5	12:58	16	8	0:16:08	BCCH		2
11	12:48	6	30	0:06:30	BCCH		1
3	12:49	7	28	0:07:28	BCCH		1
3	12:50	8	53	0:08:53	BCCH		2
5	12:59	17	36	0:17:36	BCCH		2
11	12:45	3	49	0:03:49	RBNU		1
11	12:45	3	8	0:03:08	RBNU		1

11	12:47	5	44	0:05:44	RBNU		1
3	12:53	11	9	0:11:09	RBNU		1
5	12:57	15	19	0:15:19	RBNU		1
5	12:58	16	53	0:16:53	RBNU		1
11	12:43	1	46	0:01:46	RBNU		1
11	12:42	0	57	0:00:57	start		
3	12:48	6	44	0:06:44	start		
5	12:56	14	17	0:14:17	start		
5	13:01	19	28	0:19:28	stop		
	12:42	0:00	0:00	0:00:00			
3	10:29	14	54	0:14:54	BCCH	M	1
3	10:29	14	24	0:14:24	BCCH	M	1
3	10:31	16	0	0:16:00	BCCH	M	1
11	10:23	8	8	0:08:08	RBNU		2
11	10:23	8	28	0:08:28	RBNU		2
11	10:24	9	56	0:09:56	RBNU		2
11	10:26	11	32	0:11:32	RBNU		2
3	10:28	13	13	0:13:13	RBNU		2
11	10:25	10	17	0:10:17	RBNU		2
5	10:17	2	25	0:02:25	RBNU		1
11	10:22	7	1	0:07:01	RBNU		2
5	10:16	1	21	0:01:21	RBNU		1
3	10:30	15	10	0:15:10	RBNU		1
5	10:19	4	14	0:04:14	RBNU		2
5	10:20	5	12	0:05:12	RBNU		2
11	10:23	8	43	0:08:43	RBNU		2
11	10:26	11	48	0:11:48	RBNU		2
3	10:31	16	39	0:16:39	RBNU		2
3	10:33	18	1	0:18:01	RBNU		2
3	10:33	18	17	0:18:17	RBNU		2
5	10:15	0	52	0:00:52	start		
11	10:20	5	56	0:05:56	start		
3	10:28	13	1	0:13:01	start		
3	10:34	19	21	0:19:21	stop		
	10:15	0	0	0:00:00			
5	11:44	10	21	0:10:21	BCCH	M	1
11	11:35	1	59	0:01:59	RBNU		1
11	11:38	4	0	0:04:00	RBNU		2
11	11:36	2	51	0:02:51	RBNU		2
11	11:37	3	10	0:03:10	RBNU		2
11	11:38	4	41	0:04:41	RBNU		2
11	11:40	6	2	0:06:02	RBNU		3
11	11:37	3	46	0:03:46	RBNU		2
11	11:39	5	5	0:05:05	RBNU		2

5	11:42	8	24	0:08:24	RBNU		2
11	11:38	4	52	0:04:52	RBNU		2
5	11:43	9	13	0:09:13	RBNU		2
5	11:44	10	3	0:10:03	RBNU		2
11	11:34	0	55	0:00:55	start		
5	11:41	7	19	0:07:19	start		
3	11:46	12	15	0:12:15	start		
5	11:52	18	17	0:18:17	stop		
	11:34	0	0	0:00:00			
5	9:51	5	14	0:05:14	MOCH	M	1
5	9:50	4	41	0:04:41	MOCH	M	1
5	9:51	5	37	0:05:37	MOCH	M	1
3	9:58	12	20	0:12:20	MOCH	M	1
5	9:55	9	55	0:09:55	MOCH	M	1
5	9:52	6	0	0:06:00	MOCH	M	1
5	9:52	6	13	0:06:13	MOCH	M	1
5	9:53	7	28	0:07:28	MOCH	M	1
	9:49	3	53	0:03:53	RBNU		1
11	10:08	22	18	0:22:18	RBNU		1
3	10:00	14	48	0:14:48	RBNU		1
3	10:01	15	34	0:15:34	RBNU		1
11	10:04	18	55	0:18:55	RBNU		1
11	10:06	20	53	0:20:53	RBNU		2
5	9:50	4	8	0:04:08	start		
3	9:58	12	13	0:12:13	start		
11	10:04	18	9	0:18:09	start		
11	10:10	23	38	0:23:38	stop		
	9:46	0	0	0:00:00			
3	11:29	15	13	0:15:13	BCCH	M	1
	11:15	1	15	0:01:15	MOCH	M	1
11	11:18	4	22	0:04:22	MOCH	M	1
11	11:19	5	31	0:05:31	RBNU		1
11	11:17	3	13	0:03:13	RBNU		1
11	11:20	6	52	0:06:52	RBNU		1
11	11:16	2	35	0:02:35	RBNU		1
3	11:29	15	44	0:15:44	RBNU		1
11	11:20	6	17	0:06:17	RBNU		1
5	11:22	8	31	0:08:31	RBNU		1
5	11:24	10	4	0:10:04	RBNU		1
11	11:15	1	42	0:01:42	start		
5	11:22	8	13	0:08:13	start		
3	11:27	13	37	0:13:37	start		
3	11:31	17	55	0:17:55	stop		
	11:14	0	0	0:00:00			

5	8:51	3	11	0:03:11 MOCH		1
11	8:55	7	32	0:07:32 MOCH		1
5	8:50	2	48	0:02:48 MOCH		1
5	8:50	2	51	0:02:51 MOCH		1
5	8:51	3	23	0:03:23 MOCH		1
5	8:52	4	37	0:04:37 MOCH		1
5	8:51	3	40	0:03:40 MOCH		1
5	8:52	4	15	0:04:15 MOCH		1
11	8:56	8	0	0:08:00 MOCH	M	2
11	8:56	8	15	0:08:15 MOCH	M	2
11	8:55	7	18	0:07:18 RBNU		1
11	8:56	8	24	0:08:24 RBNU		1
11	8:54	6	50	0:06:50 RBNU		1
11	8:59	9	59	0:09:59 RBNU		1
11	8:56	8	42	0:08:42 RBNU		1
11	8:54	6	31	0:06:31 RBNU		1
11	9:01	11	50	0:11:50 RBNU		1
11	8:59	9	20	0:09:20 RBNU		1
3	9:02	12	44	0:12:44 RBNU		1
3	9:03	13	43	0:13:43 RBNU		1
5	8:52	4	24	0:04:24 RBNU		1
5	8:51	3	59	0:03:59 RBNU		1
5	8:48	0	33	0:00:33 start		
11	8:53	5	56	0:05:56 start		
3	9:01	11	56	0:11:56 start		
3	9:06	16	49	0:16:49 stop		
11	9:00	10	5	0:10:05 TRES		2
11	8:59	9	44	0:09:44 TRES		2
11	9:01	11	53	0:11:53 TRES		2
	8:48	0	0	0:00:00		
3	10:38	11	15	0:11:15 BCCH		2
3	10:38	11	26	0:11:26 BCCH		2
3	10:38	11	26	0:11:26 BCCH		2
3	10:38	11	30	0:11:30 BCCH		2
3	10:38	11	45	0:11:45 BCCH		2
3	10:39	12	2	0:12:02 BCCH		2
3	10:39	12	2	0:12:02 BCCH		2
3	10:39	12	52	0:12:52 BCCH		2
3	10:40	13	18	0:13:18 BCCH		2
3	10:40	13	31	0:13:31 BCCH		2
3	10:37	10	44	0:10:44 BCCH		2
3	10:37	10	53	0:10:53 BCCH		2
3	10:39	12	28	0:12:28 BCCH		2
3	10:40	13	31	0:13:31 BCCH		2

3	10:39	12	28	0:12:28	BCCH		2
3	10:39	12	52	0:12:52	BCCH		2
3	10:36	9	50	0:09:50	BCCH		1
3	10:37	10	25	0:10:25	BCCH		2
11	10:44	17	12	0:17:12	BCCH		2
3	10:37	10	12	0:10:12	BCCH		1
3	10:35	8	32	0:08:32	MOCH		1
5	10:32	5	53	0:05:53	MOCH		1
5	10:34	7	14	0:07:14	MOCH		1
5	10:32	5	25	0:05:25	MOCH		1
5	10:30	3	40	0:03:40	MOCH		1
3	10:35	8	35	0:08:35	MOCH		1
5	10:29	2	17	0:02:17	MOCH		1
3	10:35	8	58	0:08:58	MOCH		1
3	10:36	9	35	0:09:35	MOCH		1
5	10:27	0	58	0:00:58	start		
3	10:35	8	14	0:08:14	start		
11	10:41	14	54	0:14:54	start		
11	10:45	18	22	0:18:22	stop		
	10:27	0	0	0:00:00			
5	9:26	11	53	0:11:53	BCCH	M	1
3	9:20	5	22	0:05:22	BCCH	M	1
3	9:20	5	50	0:05:50	BCCH	M	1
3	9:22	7	20	0:07:20	BCCH	M	1
5	9:27	12	50	0:12:50	BCCH	M	1
5	9:28	13	35	0:13:35	BCCH	M	1
3	9:19	4	52	0:04:52	BCCH	M	1
3	9:23	8	15	0:08:15	BCCH	M	1
5	9:25	10	59	0:10:59	BCCH	M	1
5	9:28	13	54	0:13:54	BCCH	M	1
5	9:29	14	4	0:14:04	BCCH	M	1
3	9:19	4	13	0:04:13	BCCH	M	1
3	9:23	8	16	0:08:16	RBNU		1
11	9:33	18	3	0:18:03	RBNU		1
11	9:35	20	26	0:20:26	RBNU		1
11	9:35	20	42	0:20:42	RBNU		2
11	9:35	20	42	0:20:42	RBNU		2
3	9:18	3	31	0:03:31	start		
5	9:25	10	30	0:10:30	start		
11	9:31	16	30	0:16:30	start		
11	9:36	21	26	0:21:26	stop		
	9:15	0	0	0:00:00			
3	11:38	1	18	0:01:18	start		
11	11:43	6	0	0:06:00	start		

5	11:48	11	29	0:11:29	start		
5	11:54	16	13	0:16:13	stop		
	11:37	0	0	0:00:00			
11	10:42	9	52	0:09:52	RBNU		1
11	10:43	10	15	0:10:15	RBNU		1
11	10:42	9	27	0:09:27	RBNU		1
11	10:42	9	40	0:09:40	RBNU		1
11	10:43	10	20	0:10:20	RBNU		1
11	10:43	10	41	0:10:41	RBNU		1
11	10:43	10	42	0:10:42	RBNU		1
11	10:43	10	46	0:10:46	RBNU		1
11	10:44	11	4	0:11:04	RBNU		1
11	10:44	11	22	0:11:22	RBNU		1
11	10:45	12	4	0:12:04	RBNU		1
3	10:33	0	42	0:00:42	start		
5	10:37	4	35	0:04:35	start		
11	10:41	8	40	0:08:40	start		
11	10:45	12	56	0:12:56	stop		
	10:33	0	0	0:00:00			
5	9:52	18	34	0:18:34	BCCH		1
5	9:51	17	18	0:17:18	MOCH	M	1
5	9:52	18	16	0:18:16	MOCH	M	1
5	9:51	17	51	0:17:51	MOCH	M	1
5	9:50	16	45	0:16:45	MOCH	M	1
5	9:52	18	1	0:18:01	MOCH	M	1
5	9:50	16	30	0:16:30	MOCH	M	1
5	9:53	19	19	0:19:19	MOCH	M	1
5	9:55	21	2	0:21:02	MOCH	M	1
11	9:42	8	45	0:08:45	RBNU		1
5	9:54	20	10	0:20:10	RBNU		1
11	9:43	9	12	0:09:12	RBNU		1
5	9:51	17	18	0:17:18	RBNU		1
5	9:55	21	2	0:21:02	RBNU		1
11	9:41	7	38	0:07:38	RBNU		1
11	9:45	11	6	0:11:06	RBNU		1
5	9:49	15	1	0:15:01	RBNU		1
5	9:49	15	38	0:15:38	RBNU		1
3	9:35	1	23	0:01:23	start		
11	9:40	6	53	0:06:53	start		
5	9:47	13	46	0:13:46	start		
5	9:55	21	28	0:21:28	stop		
	9:34	0	0	0:00:00			
11	12:50	13	35	0:13:35	RBNU		1
11	12:50	13	50	0:13:50	RBNU		1

11	12:51	14	56	0:14:56	RBNU			2
11	12:51	14	30	0:14:30	RBNU			1
11	12:51	14	43	0:14:43	RBNU			2
11	12:50	13	18	0:13:18	RBNU			1
11	12:52	15	16	0:15:16	RBNU			2
11	12:53	16	0	0:16:00	RBNU			2
11	12:52	15	47	0:15:47	RBNU			2
3	12:37	0	37	0:00:37	start			
5	12:43	6	36	0:06:36	start			
11	12:49	12	11	0:12:11	start			
11	12:55	18	21	0:18:21	stop			
	12:37	0	0	0:00:00				
3	9:02	13	39	0:13:39	BCCH			1
3	9:05	16	34	0:16:34	BCCH	M		1
5	9:06	17	4	0:17:04	BCCH	M		1
3	9:01	12	39	0:12:39	BCCH			1
11	8:52	3	29	0:03:29	RBNU	M		1
11	8:51	2	9	0:02:09	RBNU	M		1
11	8:52	3	12	0:03:12	RBNU	M		2
11	8:53	4	8	0:04:08	RBNU	M		1
11	8:53	4	38	0:04:38	RBNU	M		2
3	9:00	11	57	0:11:57	RBNU			1
	8:49	0	23	0:00:23	RBNU			1
3	8:59	10	30	0:10:30	RBNU			1
11	8:53	4	18	0:04:18	RBNU			2
11	8:55	6	16	0:06:16	RBNU			2
11	8:50	1	43	0:01:43	start			
3	8:58	9	23	0:09:23	start			
5	9:05	16	57	0:16:57	start			
5	9:11	22	20	0:22:20	stop			
	8:49	0	0	0:00:00				
3	12:19	8	31	0:08:31	DUFL			1
5	12:16	5	28	0:05:28	RBNU			1
5	12:14	3	5	0:03:05	start			
3	12:18	7	47	0:07:47	start			
11	12:23	12	32	0:12:32	start			
11	12:27	16	4	0:16:04	stop			
	12:11	0	0	0:00:00				
11	9:19	17	24	0:17:24	BCCH	M		1
5	9:13	11	39	0:11:39	BCCH			1
5	9:10	8	15	0:08:15	MOCH	M		1
11	9:16	14	47	0:14:47	RBNU			2
11	9:16	14	47	0:14:47	RBNU	M	AB/YK	2
11	9:20	18	37	0:18:37	RBNU	M	AB/YK	2

11	9:15	13	16	0:13:16	RBNU		2
3	9:07	5	23	0:05:23	RBNU		1
5	9:12	10	43	0:10:43	RBNU		1
11	9:15	13	6	0:13:06	RBNU		1
3	9:04	2	25	0:02:25	start		
5	9:09	7	24	0:07:24	start		
11	9:14	12	28	0:12:28	start		
11	9:21	19	7	0:19:07	stop		
	9:02	0	0	0:00:00			
3	10:07	1	45	0:01:45	BCCH		1
5	10:13	7	55	0:07:55	MOCH	M	1
5	10:14	8	15	0:08:15	MOCH	M	1
5	10:15	9	9	0:09:09	MOCH	M	1
5	10:15	9	30	0:09:30	MOCH	M	1
5	10:15	9	55	0:09:55	MOCH	M	1
5	10:13	7	19	0:07:19	MOCH	M	1
11	10:18	13	30	0:13:30	MOCH		1
5	10:12	6	0	0:06:00	MOCH	M	1
11	10:17	12	30	0:12:30	RBNU		2
11	10:19	14	50	0:14:50	RBNU		1
11	10:16	11	30	0:11:30	RBNU		1
5	10:12	6	50	0:06:50	RBNU		1
5	10:12	6	5	0:06:05	RBNU		1
3	10:09	3	10	0:03:10	RBNU		1
3	10:11	5	10	0:05:10	RBNU		1
3	10:06	0	21	0:00:21	start		
5	10:11	5	25	0:05:25	start		
11	10:16	11	26	0:11:26	start		
11	10:22	17	50	0:17:50	stop		
	10:06	0	0	0:00:00			
5	8:30	3	29	0:03:29	PISI		1
11	8:35	8	36	0:08:36	RBNU	M	2
11	8:36	9	35	0:09:35	RBNU	M	2
11	8:35	8	3	0:08:03	RBNU	M	2
11	8:36	9	53	0:09:53	RBNU	M	2
11	8:34	7	13	0:07:13	RBNU		1
11	8:35	8	10	0:08:10	RBNU		2
11	8:36	9	18	0:09:18	RBNU		2
3	8:42	15	14	0:15:14	RBNU		1
5	8:27	0	48	0:00:48	start		
11	8:33	6	8	0:06:08	start		
3	8:39	12	2	0:12:02	start		
3	8:43	16	28	0:16:28	stop		
5	8:29	2	57	0:02:57	WAVI		1

	8:27	0	0	0:00:00			
11	10:07	9	13	0:09:13	RBNU	M	1
11	10:04	6	14	0:06:14	RBNU		1
3	9:59	1	14	0:01:14	start		
11	10:03	5	15	0:05:15	start		
5	10:08	10	48	0:10:48	start		
5	10:12	14	34	0:14:34	stop		
	9:58	0	0	0:00:00			
3	11:56	8	30	0:08:30	BCCH	M	1
3	11:55	7	17	0:07:17	BCCH	M	1
3	11:55	7	29	0:07:29	BCCH	M	1
3	11:54	6	53	0:06:53	BCCH	M	1
3	11:50	2	2	0:02:02	BCCH	M	1
3	11:49	1	56	0:01:56	BCCH	M	1
3	11:50	2	38	0:02:38	BCCH	M	1
3	11:51	3	35	0:03:35	BCCH	M	1
3	11:52	4	22	0:04:22	BCCH	M	1
3	11:53	5	51	0:05:51	BCCH	M	1
3	11:50	2	15	0:02:15	BCCH	M	1
3	11:51	3	5	0:03:05	BCCH	M	1
3	11:57	9	27	0:09:27	BCCH	M	1
3	11:56	8	11	0:08:11	BCCH	M	1
3	11:57	9	3	0:09:03	BCCH	M	1
3	11:49	1	46	0:01:46	BCCH	M	1
3	11:58	10	13	0:10:13	BCCH	M	1
3	11:58	10	43	0:10:43	BCCH	M	1
3	12:00	12	40	0:12:40	BCCH	M	1
11	12:05	17	17	0:17:17	BCCH	M	1
3	11:59	11	21	0:11:21	BCCH	M	1
11	12:06	18	32	0:18:32	BCCH	M	1
11	12:07	19	5	0:19:05	BCCH	M	1
3	12:12	24	12	0:24:12	BCCH	M	1
11	12:04	16	53	0:16:53	RBNU		1
11	12:05	17	59	0:17:59	RBNU		1
3	11:55	7	39	0:07:39	RBNU		1
3	11:48	0	52	0:00:52	start		
11	12:03	15	50	0:15:50	start		
3	21:09	21	16	0:21:16	start		
3	12:14	26	17	0:26:17	stop		
3	11:53	5	24	0:05:24	U		1
	11:48	0	0	0:00:00			
11	9:12	3	38	0:03:38	BCCH		1
5	9:19	10	31	0:10:31	MOCH	M	1
5	9:20	11	12	0:11:12	RBNU		1

5	9:20	11	42	0:11:42	RBNU			1
11	9:10	1	16	0:01:16	RBNU			1
11	9:10	1	7	0:01:07	start			
5	9:16	7	5	0:07:05	start			
3	9:22	13	1	0:13:01	start			
3	9:26	17	13	0:17:13	stop			
	9:09	0	0	0:00:00				
5	9:20	10	19	0:10:19	MOCH	F	AK/PY	1
5	9:20	10	50	0:10:50	MOCH	F	AK/PY	1
11	9:24	14	24	0:14:24	RBNU	M		1
11	9:26	16	6	0:16:06	RBNU	M		1
11	9:24	14	57	0:14:57	RBNU	M		1
11	9:26	16	36	0:16:36	RBNU	M		1
11	9:25	15	20	0:15:20	RBNU	M		1
11	9:27	17	15	0:17:15	RBNU	M		1
11	9:28	18	11	0:18:11	RBNU	M		1
3	9:15	5	28	0:05:28	RBNU			1
3	9:11	1	26	0:01:26	RBNU			1
3	9:10	0	38	0:00:38	start			
5	9:16	6	5	0:06:05	start			
11	9:22	12	51	0:12:51	start			
11	9:29	19	3	0:19:03	stop			
	9:10	0	0	0:00:00				
	10:17	0	0	0:00:00	MOCH			1
11	10:25	8	18	0:08:18	RBNU			1
11	10:24	7	25	0:07:25	RBNU			1
11	10:26	9	59	0:09:59	RBNU			1
3	10:19	2	13	0:02:13	start			
11	10:23	6	52	0:06:52	start			
5	10:28	11	57	0:11:57	start			
5	10:32	15	57	0:15:57	stop			
	10:17	0	0	0:00:00				
11	11:00	5	58	0:05:58	RBNU			1
5	11:06	11	48	0:11:48	RBNU			1
3	11:10	15	17	0:15:17	RBNU			1
3	11:12	17	4	0:17:04	RBNU			1
11	10:55	0	53	0:00:53	start			
5	11:02	7	24	0:07:24	start			
3	11:08	13	51	0:13:51	start			
3	11:14	19	22	0:19:22	stop			
	10:55	0	0	0:00:00				
5	8:33	4	8	0:04:08	BCCH			1
11	8:35	6	14	0:06:14	RBNU			1
11	8:36	7	54	0:07:54	RBNU			1

3	8:39	10	33	0:10:33	RBNU			1
5	8:29	0	34	0:00:34	start			
11	8:34	5	11	0:05:11	start			
3	8:38	9	3	0:09:03	start			
3	8:41	12	32	0:12:32	stop			
	8:29	0	0	0:00:00				
3	9:45	1	24	0:01:24	BCCH	M		1
3	9:46	2	2	0:02:02	BCCH	M		1
3	9:46	2	9	0:02:09	BCCH	F		2
3	9:45	1	47	0:01:47	BCCH	M		1
3	9:46	2	9	0:02:09	BCCH	M		2
3	9:47	3	12	0:03:12	BCCH	M		2
3	9:48	4	27	0:04:27	BCCH	M		2
3	9:48	4	42	0:04:42	BCCH	M		2
3	9:45	1	11	0:01:11	BCCH	M		1
3	9:48	4	20	0:04:20	BCCH	M		2
3	9:48	4	58	0:04:58	BCCH	M		2
3	9:48	4	58	0:04:58	BCCH	F		2
3	9:49	5	12	0:05:12	BCCH	M		2
3	9:50	6	8	0:06:08	BCCH	M		2
11	9:54	10	23	0:10:23	RBNU	F		1
11	9:52	8	19	0:08:19	RBNU			2
5	10:01	17	24	0:17:24	RBNU			1
11	9:53	9	24	0:09:24	RBNU			1
11	9:52	8	19	0:08:19	RBNU			2
3	9:44	0	38	0:00:38	start			
11	9:51	7	58	0:07:58	start			
5	9:58	14	37	0:14:37	start			
5	10:04	20	0	0:20:00	stop			
	9:44	0	0	0:00:00				
3	12:05	15	44	0:15:44	BCCH	M		1
11	11:53	3	50	0:03:50	RBNU	M	AB/YK ?	1
11	11:54	4	22	0:04:22	RBNU	M	AB/YK ?	1
11	11:51	1	6	0:01:06	start			
5	11:57	7	50	0:07:50	start			
3	12:02	12	52	0:12:52	start			
3	12:08	18	15	0:18:15	stop			
	11:50	0	0	0:00:00				
3	8:38	15	58	0:15:58	BCCH	M		1
5	8:27	4	0	0:04:00	DUFL			2
3	8:38	15	21	0:15:21	RBNU			1
11	8:31	8	7	0:08:07	RBNU			1
5	8:24	1	38	0:01:38	start			
11	8:30	7	3	0:07:03	start			

3	8:35	12	24	0:12:24	start		
3	8:40	17	8	0:17:08	stop		
	8:23	0	0	0:00:00			
1	9:46	1	51	0:01:51	BCCH	M	1
1	9:47	2	43	0:02:43	BCCH	M	1
1	9:48	3	48	0:03:48	BCCH	M	1
5	9:53	8	11	0:08:11	BCCH	M	1
5	9:56	11	51	0:11:51	BCCH	M	1
5	9:57	12	22	0:12:22	MOCH	M	2
5	9:54	9	8	0:09:08	MOCH	M	1
5	9:55	10	48	0:10:48	MOCH	M	1
5	9:57	12	44	0:12:44	MOCH	M	2
5	9:54	9	55	0:09:55	MOCH	M	1
5	9:55	10	23	0:10:23	MOCH	M	1
5	9:58	13	23	0:13:23	MOCH	M	2
5	9:58	13	36	0:13:36	MOCH	M	2
13	10:02	18	30	0:18:30	MOCH		2
13	9:59	15	51	0:15:51	MOCH	M	2
13	10:00	16	40	0:16:40	MOCH	M	2
13	10:04	20	34	0:20:34	MOCH	M	2
1	9:48	3	1	0:03:01	MOCH		1
1	9:47	2	0	0:02:00	MOCH	M	1
1	9:50	5	34	0:05:34	MOCH	M	2
1	9:48	3	48	0:03:48	MOCH	M	2
1	9:50	5	34	0:05:34	MOCH	M	2
5	9:53	8	3	0:08:03	MOCH	M	2
13	9:59	15	36	0:15:36	MOCH	M	2
13	10:04	20	34	0:20:34	MOCH	M	2
5	9:57	12	22	0:12:22	MOCH	M	2
13	10:03	19	31	0:19:31	RBNU	M	1
13	10:06	22	11	0:22:11	RBNU		1
1	9:45	0	0	0:00:00	start		
5	9:52	7	30	0:07:30	start		
13	9:59	15	15	0:15:15	start		
13	10:06	22	11	0:22:11	stop		
5	9:53	8	35	0:08:35	TRES		1
	9:45	0	0	0:00:00			
1	9:04	6	3	0:06:03	BCCH	M	1
1	9:04	6	52	0:06:52	BCCH	M	1
1	9:06	8	6	0:08:06	BCCH	M	1
1	9:03	5	0	0:05:00	START		
5	9:08	10	0	0:10:00	START		
13	8:58	0	0	0:00:00	START		
	8:44:46			0:46	MOCH	M	

8:48:50	4:50	MOCH		
8:47:00	3:00	TAHU	U	
9:04:10	14:10	MOCH	U	2
9:09:25	19:25	MOCH	M	2
9:09:20	19:20	MOCH	M	2
9:10:00	20:00	MOCH	M	2
9:00:40	10:40	MOCH	U	1
9:00:40	10:40	MOCH	U	1
9:05:30	15:30	MOCH	M	2
9:04:00	14:00	MOCH	U	2
9:05:10	15:10	MOCH	M	2
9:05:30	15:30	MOCH	M	2
9:03:00	13:00	MOCH	U	1
9:12:30	22:30	MOCH	B	2
9:03:00	13:00	MOCH	M	1
-	-		-	-
9:04:30	27:30:00	MOBL	B	
8:58:12	21:12	MOCH	M	
8:57:51	20:51	MOCH	M	
8:52:00	15:00	MOCH	M	

Behaviour1	Distance.m	Nest.status	Fledg.pres	Nrst.Nest.s	Comments	Behaviour2	Behaviour3	Behaviour4	RANK1
FL	1								5
C	1								9
S	1								13
L	1								10
EN	1								8
FC	1					FO			4
FC	1								4
FC	1								4
FL	1								5
L	1.5								10
AR	2								
S	3								13
wing flutter	3					EN			
FO	10								1
S	15								13
FO	15				FO on At leaves				1
S	60								13
C	40	E			NOFL nest nearby				9
C	30								9
C	60								9
A	0	PN	no						
A	0	PN	no		BOTH ATTACK				
S	0.5	PN	no						
LIP	1	PN	no						
EN	1	PN	no						
EXC	1	PN	no						
N,S	1	PN	no						
SW	1	PN	no						
N	1	PN	no						
EN	1	PN	no						
EXC	1	PN	no						
LIP	1	PN	no						
EN	1	PN	no						
SW	1	PN	no						
EXC	1	PN	no						
SR	1	PN	no						
EXC	1	PN	no						
N	1	PN	no						
EXC	1	PN	no						
N	3	PN	no						
AC,S	3	PN	no		carrying fur				
AC,EXC	3	PN	no						
SR	3	PN	no						

S	4	PN	no
S	8	PN	no
S	10	PN	no
C	15	PN	no
S	15	PN	no
LIP	25	PN	no
S	40	PN	no
C	40	PN	no
AC	40	PN	no
		PN	no
		PN	no
		PN	no
A	0	E	no
FL	2	E	no
FLY	10	E	no
S	15	E	no
S	15	E	no
S	20	E	no
S	20	E	no
C	20	E	no
S	30	E	no
S	20	E	no
S	20	E	no
LC	30	E	no
		E	no
		E	no
		E	no
		E	no
		E	no
SR	3	C	no
S	3	C	no
EN	3.5	C	no
FL	3.5	C	no
EN	3.5	C	no
FL	3.5	C	no
SR	5	C	no
FLY	15	C	no
SR	6	C	no
SR	7	C	no
FLY	15	C	no
C	20	C	no
LC	20	C	no
		C	no
		C	no

			C	no				
			C	no				
			C	no				
FL, C		3		NO				
C		5		NO				
C		7		NO				
C		7		NO				
C		7		NO				
C		10		NO				
C		15		NO				
C		20		NO				
				NO				
				NO				
				NO				
S		10	C					13
S		10	C					13
S		15	C					13
S		20	C					13
C		2	C			FC		9
EN		2	C					8
FC		2	C		Feeds chic LIP		L	10
EN		2	C					8
FC		2	C					4
FC		2	C					4
FC		2	C					4
FC		2	C					4
FC		2	C					4
FC		2	C					4
FC		2	C					4
FC		2	C					4
FC		2	C					4
C		15	C					9
SW,AC		0	PN					
AC,SR		1	PN					
C		1.5	PN					
AC,SR		1.5	PN					
IN		2	PN					
LIP,EN		2	PN					
C		3	PN					
C,SR		3	PN					
C		4	PN					
C		8	PN					
C		10	PN					
FL	25+		PN		Both MOCH fly off			

FL		PN	
CH		PN	MOCH CH RBNU off
CH		PN	M MOCH CH 3rd MOCH away
SR	1	PN	
SR	4	PN	
SR	4	PN	
SC	10	PN	
LC	15	PN	
LC	20	PN	
A	0	E	
FC	1	E	
AC	1	E	
FL	1	E	
S	2	E	
SR,C	3	E	
CB	6	E	MOCH chases RBNU away
C	40	E	
S	60	E	
SR	3	E	
LC	6	E	
LC	15	E	
LC	15	E	
C	60	E	
N,S	1.5	E	
N,S	3	E	
S	4	E	
N,S	5	E	
S	6	E	
S	8	E	
S	15	E	
A	0	E	
N,C	2	E	
EN	2	E	
FL	2	E	both fly off
SR	2.5	E	
EN	2.5	E	
SR	4	E	
FO	5	E	
C	10	E	
S	15	E	
AR	25	E	
LC	10	E	
S	0.5	E	
SR	1	E	

SWAY	1	E	
S	1.5	E	
AR	4	E	had to wait for ad to come, male brings food
FC	4	E	
FC	4	E	Had to wait for male to be around, Feeds Female
LIP	4	E	
FC	4	E	Feeds Female
S,FO	10	E	Female flushes
CH WAVI	15	E	Chases WAVI
C	15	E	
FL	20	E	
LIP	4	E	
LC	8	E	
FC, EN	1.5	C	
FL	1.5	C	
FC	1.5	C	
FL	1.5	C	
C	2	C	
CB	4	C	MOCH chased RBNU
C	6	C	
C	8	C	
SR	2	C	
AR	4	C	
C	6	C	
SR	8	C	
LC	20	C	
SW	0.25	I	
N,SR	1	I	
EN	2	I	
EXT	2	I	
EN	2	I	
LIP,FC	2	I	Feeds female
HD	2	I	
LIP	2	I	
C	3	I	
S	8	I	
S	1	C	
C	10	C	
S	10	C	
S	25	C	
S	50	C	
FC	0.5	C	ORANGE WORM
EN	0.5	C	
L	1	C	2ND MOCH-HAS FOOD

L	1	C		
C	1	C		
C	1.5	C		
L	1.5	C		
SR	2.5	C		
L	3	C		HAS FOOD
C	3	C		
C	3	C		
C	3	C		
SR,L	4	C		
C	4	C		
S	15	C		
C	30			
LC	70	C		

FC	6			
FC	6			

S, N	10		NO	
S	15		NO	
S	25		NO	
S	25		NO	
S	25		NO	
S	35		NO	
S	35		NO	
S, FO	35		NO	
S	35		NO	
S	40		NO	
S	50		NO	
CHASE BCCH			NO	
			NO	
			NO	
			NO	
FIGHT WITI	3		NO	
S	30		NO	
S	30		NO	
A	0		NO	
SW	0.1		NO	
SW	0.25		NO	
L, N	0.5		NO	
L	1		NO	

L	2		NO	
N	2.5		NO	
N	5		NO	
S, N	6		NO	
S	15		NO	
S	20		NO	SHORT FIGHT WITH UKFL NESTING IN SAME TREE
S	30		NO	
S	40		NO	
S	40		NO	
S	40		NO	
S	40		NO	
S	40		NO	
S	40		NO	
S	40		NO	
S	40		NO	
S	50		NO	
S	50		NO	
S	50		NO	
S	50		NO	
S			NO	
			NO	
			NO	
			NO	
		INC	NO	
HD	3	INC	NO	STAYS IN CAV
N, WF, LIP	3	INC	NO	
FLY, WF	3	INC	NO	
AC, LIP	3	INC	NO	
EN	3	INC	NO	
HD	3	INC	NO	
FL	3	INC	NO	
C	5	INC	NO	
C, FLY	5-100	INC	NO	
		INC	NO	
		INC	NO	
		INC	NO	
		INC	NO	
FO	3.5	C	NO	
FC, EN	3.5	C	NO	
FC, EN	3.5	C	NO	
S	8	C	NO	
C, S	8	C	NO	
S	40	C	NO	
S	40	C	NO	

S	40	C	NO	
FLY		C	NO	
FLY		C	NO	
S	35	C	NO	
		C	NO	
		C	NO	
		C	NO	
S	10	E	no	
S, SR	10	E	no	
S, SR	15	E	no	
S, SR	17	E	no	chases RBNU
FO	10	E	no	
drumming	15	E	no	
L	4	E	no	
L, EN	4	E	no	
looks out fr	4	E	no	
L	4	E	no	
EN	4	E	no	
LIP	4	E	no	
LIP	4	E	no	
L	4	E	no	chases RBNU
N	5	E	no	
SR	5	E	no	
FL, CB	10	E	no	
FLY	20	E	no	
S	30	E	no	
SR	1	E	no	
SR	2	E	no	
N	4	E	no	
SR	4	E	no	
SR	5	E	no	
SR	8	E	no	
AC	10	E	no	
SR	15	E	no	
AC	15	E	no	
AC	15	E	no	
C	15	E	no	
		E	no	
		E	no	
		E	no	
		E	no	
CB	12	E	no	
		E	no	
S	30	E	no	

FL	3	E	no	
LIP, EN	3	E	no	
LIP, EN	8	E	no	7 eggs
FLY	10	E	no	
S	12	E	no	
S	15	E	no	
FO	15	E	no	
S	20	E	no	
C	25	E	no	
SR, AC	10	E	no	
C	15	E	no	
LC	15	E	no	
C	20	E	no	
C	20	E	no	
LC	40	E	no	
C	40	E	no	
C	40	E	no	
		E	no	
		E	no	
		E	no	
		E	no	
		E	no	
C	8	C	no	
FL	8	C	no	
EN	8	C	no	
C	8	C	no	
FLY	8	C	no	
FC	8	C	no	
FL	8	C	no	
LIP	8	C	no	
SR	8	C	no	
LIP	8	C	no	
FLY	10	C	no	
FO	15	C	no	
FO	15	C	no	
LC	25	C	no	
LC	40	C	no	
		C	no	
		C	no	
		C	no	
		C	no	
		C	no	
N	5.5	C	no	
N	5.5	C	no	carrying food

N	5.5	C	no	
LIP, FC	6	C	no	
N	6	C	no	
LIP, FC	6	C	no	
LIP, EN	6	C	no	
FL	6	C	no	
EN	6	C	no	
LIP	6	C	no	carrying food
FL	6	C	no	
EN	6	C	no	
FL	6	C	no	
LIP, EN	6	C	no	
FL	6	C	no	
EN	6	C	no	
FL	6	C	no	
FL	6	C	no	
EN	6	C	no	
FL	6	C	no	
LIP, EN	6	C	no	carrying food
FL	6	C	no	
LIP, EN	6	C	no	
FL	6	C	no	
LIP, EN	6	C	no	
FL	6	C	no	
FLY	10	C	no	these behaviours seem to be just feeding - not in respons
LC, SR	15	C	no	
C	25	C	no	
LC	40	C	no	
		C	no	
		C	no	
		C	no	
		C	no	
		C	no	
C	15	C	no	carrying food
LC	40	C	no	
		C	no	
		C	no	
		C	no	
		C	no	
		C	no	4 chicks 9-10 days old
A,AC	0	C	Y	
A,AC	0	C	Y	
S	5	C	Y	
C	20	C	Y	

FO	20	C	Y		
FO	20	C	Y		
S	40	C			
EN,FC	5	C		Orange worms	
EN,FC	5	C			
EN,FC	5	C			
EN,FC	5	C			
EN,FC	5	C			
EN,FC	5	C			
EN,FC	5	C			
EN,FC	5	C			
EN,FC	5	C			
AR	15	C		AR with food; RBNU pr. Seem apprehensive around aggres	
C	40				9
C	60				9
C	60				9
FO	15				1
CB	15				14
C	15				9
CC	20				
C	40				9
C	70				9
N				Three RBNI CB	6
N				All 3 birds on nest tree	6
FC					4
FL				Adult FL as DG approaches nest tree	5
C	15				9
C	10	F			
C	15	F			
S	15	F	Y		
C	15	F	Y		
S,C	40	F			
C	50	F			
C	80	F			
S	8	C			
C	15	C			
S	20	C			
C	30	C			
C	40	C			
LIP,FC	2	C			
EN,FC	2	C			
LIP,FC	2	C		Bright orange worm	
FC	2	C			
N	3	C			

CB		8	C	Chased BCCHs away
AR		10	C	
LC		20	C	
LC		30	C	
FL		3	PN	
FL		3	PN	
S		15	PN	
S		20	PN	
S		30	PN	
S		50	PN	
S		60	PN	
A		0	PN	
SR,N		1	PN	
EX		1.5	PN	F RBNU EX today
EN		1.5	PN	
FL		1.5	PN	
LIP		1.5	PN	
EN		1.5	PN	
EX		1.5	PN	
FL		3	PN	
N		4	PN	
LC		15	PN	
SR		15	PN	
LC		20	PN	
CH	-		PN	CH MOCH
CH	-		PN	CH MOCH
SR		4	PN	
S		10	PN	
C		15	PN	
S		60	PN	
SW		0	PN	
N		1	PN	
N		3	PN	
EN		5	PN	
N		5	PN	2 RBNU AND 2 MOCH
LIP		5	PN	
AC		5	PN	
LC		5	PN	
LIP,C		5	PN	
FL		5	PN	
SC		7	PN	
C		20	PN	
FL	-		PN	
CH	-		PN	CHASED OFF BY RBNU

C	-	PN	
LC	6	E	
LC	8	E	
LC	10	E	
LC	20	E	
LC	25	E	
LC	25	E	Another RBNU C from different direction
LC	40	E	
LC	40	E	Other RBNU C from different direction
LC	50	E	
LC	50	E	Other RBNU
LC	50	E	
LC	60	E	
C	15	E	
C	20	E	
C	25	E	
S	30	E	
C	30	E	
SR	2.25	E	
SR	5	E	
N	7	E	Pecking on tree
LC	10	E	
LC	10	E	
SC	15	E	
SC	15	E	
LC	25	E	
LC	25	E	
SC	25	E	
LC	30	E	Female IN
SR	10	E	
C	20	E	
C	30	E	
SR	3	E	
LC	10	E	
LC	15	E	
LC	17	E	
SW	0	I	Knocked model off stand
N	1.5	I	
SR	2.5	I	
SR	3	I	
N,SR	4	I	
LC	4	I	
AC	5	I	
FO	7	I	

FL	8	I	
N,LIP	8	I	
EN	8	I	
LC,N	8	I	
FC	8	I	
LC	9	I	
AC	9	I	
CB	10	I	RBNU chased RNSA
C	15	I	
A	0	?	
L	0.5	?	
L	0.5	?	
SR	0.5	?	SR FOR MODEL
N	0.75	?	
C,FL	0.75	?	
SR,L	5	?	
FL,C	6	?	no RBNU went near nest - nest probably abandoned
SR	10	?	
N	1.5	?	
SR	2	?	LOOKS LIKE MALE RBNU BUT NOT SURE
SR	8	?	STARTED RBNU PB AGAIN
LC	15	?	
SC/LC	20	?	WEIRD CALL
SC/LC	20	?	WEIRD CALL
LC	20	?	
LC	20	?	
LC	30	?	
SC	30	?	2ND RBNU
LC	30	?	
LC	60	?	2ND RBNU
LC	80	?	
LC	80	?	2ND RBNU
LC	80	?	
IN	5	INC	
IN	5	INC	
IN	5	INC	
LC	20		

SW	0.25	INC
L	1.5	INC
SR	2	INC
FC	3	INC

LC,N	3	INC
N	3	INC
LIP	3	INC
LC	12	INC

N, L	4
FC	4
FC	4
FC	4
FC	4
FC	4
LC	4
FC	4
FC	4
N, LC	6
WF	10
LC	10
FO	10

S	10
S	20
C	40
S	40
AR	
SR	3
L	6
LC	15
LC	15
LC	15
LC	15

FC	4
FC	4

FC	4
L, SC	5
C	30
S	40
PR	8

LC	6
LC	6
LC	6
C, FO	10
LC	15
FO	20
AR	

S	10	PN	no	
S	15	PN	no	
S	25	PN	no	
LC	20	PN	no	
LC	20	PN	no	MOCH enters and re-enters cavity many times until 25:30
LC	30	PN	no	
LC	40	PN	no	
LC	50	PN	no	
LC	50	PN	no	
LC	50	PN	no	
LC	50	PN	no	
N		PN	no	
		PN	no	
		PN	no	
		PN	no	
		PN	no	
		PN	no	
S	20	E	no	
S	25	E	no	
N	3	E	no	
L	4	E	no	
SR	5	E	no	
SR	6	E	no	

FLY	10	E	no
FO	10	E	no
SR	10	E	no
LC	10	E	no
FO	15	E	no
SR, LC	15	E	no
FLY	15	E	no
FO	15	E	no
LC	30	E	no
		E	no
		E	no
		E	no
		E	no
CB	15	E	no
		E	no
S	15	E	no
S	20	E	no
C	20	E	no
C	25	E	no
SR	1.5	E	no
C	1.5	E	no
C, SR	1.5	E	no
SR	4	E	no
SR	5	E	no
S	5	E	no
C	15	E	no
SR	15	E	no
C	15	E	no
S	20	E	no
FO	20	E	no
S	25	E	no
SR	1	E	no
SR	2	E	no
SR	2	E	no
SR	3	E	no
SR, N	5	E	no
N, SR	6	E	no
C, N	7	E	no
FL	7	E	no
FC	7	E	no
N	7	E	no
LIP	7	E	no
N	7	E	no
N	7	E	no

N, C	8	E	no	
CB, AC	10	E	no	
SR	10	E	no	
C, SR	12	E	no	
LC	20	E	no	
AC	20	E	no	
LC	20	E	no	
AC	25	E	no	
LC	25	E	no	
NR	15	E	no	
		E	no	
		E	no	
		E	no	
		E	no	
		E	no	
EN, FL	3	C	no	
EN, FL	3	C	no	
EN, FL	3	C	no	
N	4	C	no	carrying food
N	4	C	no	
SR	4	C	no	
SR, N	4	C	no	carrying food
EN	4	C	no	
FL	4	C	no	
EN	4	C	no	
FL	4	C	no	
EN	4	C	no	
FL	4	C	no	
FO	7	C	no	
FLY	10	C	no	
FLY	10	C	no	
		C	no	
		C	no	
		C	no	
		C	no	
		C	no	
SR	1	C	no	
SR, N	1	C	no	
SR	1	C	no	
SR, N	4	C	no	
SR, N	6	C	no	
SR, C	7	C	no	
SR	12	C	no	
FLY	12	C	no	

LC	30	C	no
LC	30	C	no
		C	no
		C	no
		C	no
		C	no
		C	no
N	8	C	no
EN, FC	9	C	no
FL	9	C	no
EN, FC	9	C	no
FL, FLY	9	C	no
EN	9	C	no
FL, C	9	C	no
EN, FC	9	C	no
LIP	9	C	no
SR, WF, FL'	12	C	no
WF	12	C	no
WF	12	C	no
LC	12	C	no
WF	13	C	no
LC	15	C	no
C	15	C	no
C	20	C	no
LC	30	C	no
		C	no
		C	no
		C	no
		C	no
		C	no
LIP, FEEDII	7		NO
N, L	7		NO
L	8		NO
PR	8		NO
AR	8		NO
L	9		NO
L	10		NO
S	10		NO
AR	10		NO
L	12		NO
	15		NO
S	30		NO
FEEDS F			NO
			NO

		NO	
		NO	
SR,S	4		
SR,S	5		
SR,S	5		
SR,S	8		
S	10		
STOP S	10		
S	15		
S	20		
S	40		
S	60		
S	100		
L	15		
C	15		
C	20		
A	0		
SW	0.25		
SW	0.5		
SW	0.75		
SW	0.75		
SW	0.75		
S	1		
S	2		
FO	2.5		FO on spruce
AR	3		
S	4		
C	10		
AR	25		
S	100		
SW	0.5		
L	1		
SR	2		
AR	2		
SR	2.5		
WF	2.5		
N	3		
SR	4		
C	4		
SC	4		
AC	4		
AC	20		
AC	20		
STOP AC	20		

S	100					
S	6					
S	10					
FO	15					
C	20					
FL,CH	20			Being chased or chasing other bird species		
SR	1					
N,SR	2					
LIP	2.5					
FL	10			Chased by other bird species		
LC	15					
LC	20			Then chased away again		
SR	0.5					
SR	1					
FO	2					
C	10					
LC	10					
LC	10					
LC	10					
LC	15					
LC	15					
N	1	N	N	trial		6
C	10	N	N	trial		9
C	20	N	N	trial		9
C	50	N	N	trial		9
C	20	N	N	trial		9
C		N	N	trial		9
N	2	N	N	trial		6
N	6	N	N	trial		6
N	6	N	N	trial	L	10
C	10	N	N	trial		9
FO	10	N	N	trial		1
C	10	N	N	trial		9
C	20	N	N	trial		9
C	30	N	N	trial		9
N	2	N	N	trial		6
L	2	N	N	trial		10
	15	N	N	trial		
FO	30	N	N	noise	C	9
C	40	N	N	noise		9
C	60	N	N	noise		9

C	70	N	N				9
C	90	N	N				9
C	30	N	N				9
FLY	1	N	N	MOCH-E			2
FLY	1	N	N	MOCH-E			2
C	4	N	N	MOCH-E			9
C	4	N	N	MOCH-E		S	13
C	8	N	N	MOCH-E		S	13
C	10	N	N	MOCH-E			9
C	10	N	N	MOCH-E			9
C	15	N	N	MOCH-E			9
C	100						9
SR	1						11
N	3						6
N	5						6
AR	5				Looking in all cavities in nearby trees		
C	30						9
C	100						9
C	40						9
C	80			MOCH-E			9
S	1						13
S	5					C	13
S	10						13
AR	10						
FO	10				FO in nest tree (At)		1
AR	15						
S	20					C	13
C	20						9
S	20						13
S	30						13
AR	2						
N	2						6
AR	6						
AR	10						
AR	10						
C	15						9
C	40						9
AR	50						
FO	20				FO on At leaves		1
C	30						9
C	40						9
C	10						9
C	15						9
C	15						9

C	15						9
C	40						9
S	100						13
AR	5						
AR	5						
AR	5			EN cav in new nest tree nearby			
AR	10						
FLY	10						2
C	10						9
FO	15			FO in At			1
C	60						9
C	80						9
L	0.1						10
S	1				N		13
S	1						13
SR	3			Both adults	SR		11
C	3			With food	N	S	13
AR	5						
S	5						13
S	8						13
S	8			FO on aspe	FO		13
C	10						9
S	10						13
S	15						13
S	15						13
C	20						9
S	30						13
N					LIP		6
SR							11
N					SR		6
N							6
C	15				FO		9
C	15				FO		9
C	20				FO		9
C	20						9
C	20			2 adults C	FO		9
C	20						9
N							6
SR							11
C	25		MOCH-C				
S	60		MOCH-C				
C	20		MOCH-C				
			RBNU-E				
A	0	Y	RBNU-C				

A	0	Y	RBNU-C	
N,S,SR	0	Y	RBNU-C	
AC	2	Y	RBNU-C	
S	3	Y	RBNU-C	
S	5	Y	RBNU-C	
AR	10	Y	RBNU-C	BCCH pr. With 4+ yng out of nest
S	15	Y	RBNU-C	
S	30	Y	RBNU-C	
A,S,AC	0	Y		
A,S,AC	0	Y		
S	0	Y		
AC	1	Y		
AC	1	Y		
C	5	Y		
S	10	Y		
S	10	Y		
C	20	Y		Followed BCCH pr. To family of 4 yng, recently fledged nr.
S	40	Y		
C	20			
C	60			
A	0			
N,LIP	5			
FO,C	10			
LC,AC	10			
AC	10			
AC	10			
C	40		RBNU-F	
C,DR	15			
FO	30			
L,SR,LIP	0.5	Y		
L,C	8	Y		
C,FO	10	Y		Pr FO with 4 HYs on MPB tree
C,FO	10	Y		Pr FO with 4 HYs
C	20	Y		
FO,C	30	Y		family FO on FD
LC	10	Y		
CC	15	Y		Female SW at DOWO
AC	20	Y		
CC	20	Y		Pr with 3 HYs
CC	20	Y		
CC,FO	20	Y		family FO on MPB trees
LC	30	Y		
C	40	Y		
C	60	Y		

AC	60	Y		
C	75			
C	30			
C	10		RBNU-C	
C	30		RBNU-C	
S	0.25			
N	1			
FO	1			
S	3			
S	3			
C	5			
L,S	5			
S	5			
C	10			
FO	15			
FO	20			
FO	30			
S	60			
S	60			
LIP	6			
LIP	6			
C,FO	30			
S,N	2			
SR	3			
S	4			
S	4			
S	5			
S	20			
S	50			
SW	0.5			
LIP	3.5			
EN	3.5			
N	5			
-	-			can't compare - TRES aggressive, CH and RBNU not aroun
AR	4			
AR	3			
AR	2 from N			
N	2			
AC	8			
S	8			
SR	8			
C	8			
FO	20			
FO	60			

FL		4 TRES flying around
C,FO	10	
C,FO	10	
C	15	
C,FO	20	
C,FO	20	
C	20	
C	20	
S	30	
C	80	
LIP	6	
EN	6	
LIP	6	
FL	6	
N	8	
C	15	
C	15	
S	25	
C	30	
C	35	
AC	35	
AC	35	
SW	0.25	
SW	0.25	
L	1	
AR	1	
AR	1	
SWAY	1	
SR	1	
N,SR	4	
C	4	
C,FO	8	
NR		
NR		
SW	1	
N	2	
N	4	
LC,WF	4	
AC	6	AC for 1 minute
SR	8	
LC	40	
SC	40	
LIP	3	RNSA peeks in cav
C	20	

C	60	
AC	1	
S,N	3	
S	15	
S	30	
C	20	
C	60	
SW	0.25	
N,SR	1	
LIP	1.5	
N	3	
SR	10	
LC	10	
S	60	
L	0.25	
SR	1	
S	2	
L	3	
NR	5	
NR	30	
SR	4	
EX	5	
NR	5	
N	5	
LC,N	5	
LC	12	
LC	12	
FO	10	
C	8	
S	8	
S	30	

AR	20	
AR	5	
SW	0	NO
L	1	NO
L	1	NO
S	1.5	NO

FO	1.5	NO
AR	1.5	NO
AR	1.5	NO
AR	2	NO
AR	2	NO
S	3	NO
AR	4	NO
AR	4	NO
S	7	NO
S	8	NO
S	8	NO
S	15	NO
C	15	NO
C	20	NO
AR	20	NO
FO	25	NO
AR	25	NO
S	30	NO
S	40	NO
FO	40	NO

AR	15	NO
AR	15	NO
FA	20	NO
FA	20	NO
C	50	NO
C	100	NO
NR		
STOP S	100	NO
S	100	NO
S	150	NO
STOP S	150	NO
S	150	NO
S	150	NO
S	150	NO
LC	200	NO
STOP S	200	NO
AR		

N	0.5	NO
N,LIP	1	NO

N,L	1	NO
N	1	NO
N	1	NO
N	1	NO
N	1	NO
N	1	NO
L	2	NO
N,LIP	2	NO
C	2	NO
LIP,N	2	NO
N	2	NO
N	2	NO
L	2	NO
N	2	NO
N	3	NO
L	3	NO
AR	4	NO
L	4	NO
L	5	NO
L	5	NO
L	6	NO
L	7	NO
FO	7	NO
L,AR	10	NO
AR	10	NO
C	10	NO
FO	12	NO
FO	12	NO
FO	15	NO
C	15	NO
FO	15	NO
L	15	NO
S	25	NO
S	25	NO
S	30	NO
S	30	NO
C	40	NO

AR	10
C	10
S	10
S	10

S	10
NR	10
S	20
S	30
S	30
AR	30
AR	50
A	0
SW	1.5
C	10
C	10
AR	20
AR	30
C	150
C	20
AR	20
C	25
C	30
S	150

NOT RECO	20
not recorde	0.5
C	2
N	3
FO	4
NOT RECO	20
C	0.5
C	3
L	3
L	4
C	10
C	10
NOT RECO	20
NOT RECO	25
SW	not recorded
N	2
N	3
S	5
S	10
C	10
S	10
C	20

S	50		
S	50		
S	100		
not recorde	not recorded		
N,L	8	NO	
L	10	NO	
S	10	NO	
C	20	NO	
C	30	NO	
S	40	NO	
S	40	NO	
S	40	NO	on and off S
S	40	NO	on and off S
stop S	60	NO	
S	60	NO	on and off S
stop S	60	NO	
S	60	NO	just once
S	60	NO	on and off S
C	60	NO	
stop S	120	NO	top broken off
NR		NO	
A	0	NO	
A	0	NO	
N,L,AC	1	NO	
N	1	NO	
AR,L	1	NO	
L,AC	1.5	NO	
N,L,AC	1.5	NO	
N,L,AC	1.5	NO	
L	2	NO	
L,AC	2	NO	
AC	2	NO	
N	2	NO	
S,N	2	NO	
S	2.5	NO	
S	2.5	NO	
AR	3	NO	F took off
S,N	3	NO	
S,N	3	NO	
S,N	3	NO	
LIP	3	NO	

AC,N	4	NO	
L,AC	4	NO	
L,AC	4	NO	
C	4	NO	F took off
N	4	NO	took off
N	4	NO	
AR	5	NO	
FO	5	NO	
WF,CC	6	NO	
L,C	7	NO	
stop S	8	NO	
WF,CC	8	NO	
C	15	NO	
AR	20	NO	
		NO	took off
stop S,C		NO	took off
C,N	4	NO	just for a sec on N
		NO	
		NO	
		NO	
N	4	NO	just for a sec on N
NR		NO	
NR		NO	
NR		NO	
AC,N	1	NO	
AC	2	NO	
L,AC	2	NO	
L	3	NO	
L,AC,N	3	NO	
L	4	NO	
AC	6	NO	
S	8	NO	
AC	10	NO	
C	10	NO	
S	15	NO	
WF,S	15	NO	
WF	15	NO	
S	15	NO	
AC	15	NO	
S	20	NO	
AC,C,WF	20	NO	
AC,C,WF	20	NO	
FO	20	NO	
PR	20	NO	

S	30	NO	
S	30	NO	
S	30	NO	still S, when I left
AC	35	NO	took off
AC	40	NO	
S	40	NO	
S	40	NO	
AR		NO	chasing e.o. in AE
		NO	
		NO	
		NO	
C	40		
C	30		
S	40		
STOP S	40		
C	40		
S	40		
S	40		
S	40		
S	40		
S	50		
STOP S			
S	40		
S	40		
S	40		
S	40		
S	40		
S	40		
"TALKING"	40		

S	80
S	40
WF,N,L	3
AR	4
C	4
AR	5
AR	10
LC,SC	10

S	6
S	7
STOP S	7
S	10
S	30
S	50
S	50
S	50
STOP S	50
LC	15
FO	15
AR	15
FO	15
FO	15
LC	30
SC	40
C	40
SC	40
FA	40
LC	40
LC	50
LC	50
LC	50
LC	50
LC	80

S	40
S	40
S	40
S	40
S	40
C	40
C	50
C	50
C	50
S	50
AR	50
S	60
S	60
S	60

N,C	11		
C	11		
FO,S	12		
S,N	12		
C	15		
S	40		
S	40		
C	60		
S	80		
S	100		

AR	25	
C	25	
S	30	
AC	35	
S	40	
S	80	
S	80	
S	60	
S	100	
L	6	
LIP	7	
EXC	7	
LIP	7	
AC	8	
CB	10	RBNU chases BCCH
SC	20	
C	20	
LC	40	
C	40	

LIP	12	C?	no?
EN	7	C?	no?
SR	7	C?	no?
EN	7	C?	no?
LIP	7	C?	no?
LIP	7	C?	no?
SR, L	8	C?	no?

S	10	C?	no?
S	10	C?	no?
stop S	10	C?	no?
S	12	C?	no?
S	20	C?	no?
S	30	C?	no?
CC		C?	no?
FL		C?	no?
S, N	9	C?	no?
stop S	9		
FO	10		
S	15	C?	no?
SC	20	C?	no?
S	20	C?	no?
		C?	no?
		C?	no?
		C?	no?
		C?	no?
S	12		NO
S	12		NO
L	12		NO
S	30		NO
C	30		NO
S	40		NO
S	40		NO
S	40		NO
S	70		NO
S	70		NO
SW	0.1		NO
SW	0.5		NO
SW	1		NO
N	1		NO
N, SR	2		NO
SR, L	2		NO
SR, N, L	3		NO
C	4		NO
N	6		NO
L	7		NO
S	8		NO
S	8		NO
S	20		NO
FLY, S	40		NO
S	60		NO

S	60	NO	
S	60	NO	
		NO	
		NO	
		NO	
S	25	NO	
C	25	NO	
C	25	NO	
S	25	NO	
C	30	NO	
C	40	NO	
S	60	NO	
N, L	2	NO	
N,SR, L	3	NO	
N	4	NO	
SR, L	4	NO	
SR, L	5	NO	
FLY	6	NO	
N, L, SR	6	NO	
SR, L	7	NO	
S	30	NO	
S	30	NO	
S	50	NO	
		NO	
		NO	
		NO	
C	50	NO	
S	40	NO	
		NO	
		NO	
		NO	
FO	10	NO	
S	10	NO	
S	12	NO	
S	15	NO	
S	20	NO	
S	20	NO	
S	30	NO	
S	40	NO	
LIP		NO	FEEDS OTHER
FLY	6	NO	
N, L	10	NO	
WF, SC	12	NO	
S	12	NO	

S	30	NO	
S	30	NO	
S	30	NO	
FLY		NO	
		NO	
		NO	
		NO	
		NO	
		NO	
		NO	FLYCATCHER AR
LIP	2	no	chases RBNU
FO, C	12	no	both RBNU on nest tree
FO	12	no	
C	20	no	
L	4	no	
N	5	no	
LC	50	no	
FA		no	
		no	
		no	
		no	
		no	
		no	
SR	5	no	
C	5	no	
S	20	no	
C	30	no	MOCH seen nearby, RCKI heard throughout playback
C	8	no	
S	8	no	
S	10	no	
L	10	no	
S	10	no	
C	30	no	
LC	40	no	
		no	
		no	
		no	
		no	
		no	
LC	3	no	
LC	5	no	
N	8	no	
LC	10	no	
LC	15	no	

LC	20		no	
LC	40		no	
LC	45		no	
			no	7028 is between 7403 and 7403R
			no	
			no	
			no	
			no	RCKI heard throughout playback (30m)
SW, N	2		no	
SW	3		no	
N, SR	4		no	
NR	5		no	
L	5		no	
LC	20		no	
LC	20		no	
C	30		no	
			no	
			no	
			no	
			no	
			no	
A	0	N	no	flies away (no longer visible)
SR	0.5	N	no	enters cavity with fur
SR	1	N	no	
SR	1	N	no	enters cavity with fur
SR	2	N	no	
SR	2	N	no	
S	2	N	no	
SR	2	N	no	
SR	2	N	no	
FLY	2	N	no	
N	2	N	no	chases BCCH
S	2	N	no	
N	2	N	no	chases BCCH
SR	2	N	no	
SR	2	N	no	
N,SR	2	N	no	
S	3	N	no	
C	3	N	no	
SR	3	N	no	
SR	3	N	no	
S	5	N	no	
SR	5	N	no	lands on nest tree with food in beak
N	5	N	no	

N	6	N	no	
FL	8	N	no	
C	10	N	no	
FO	10	N	no	
S	20	N	no	
S	30	N	no	
S	30	N	no	
S	30	N	no	
S	30	N	no	enters cavity with fur
A	0	N	no	
SR	2	N	no	
SR	2	N	no	
SR	4	N	no	
C	5	N	no	
N,SR	5	N	no	
SR	5	N	no	
SR	5	N	no	
N,SR	6	N	no	
C	8	N	no	
C	10	N	no	
CB	10	N	no	
		N	no	
		N	no	
		N	no	
		N	no	
		N	no	
A	0	N	no	
S	0.1	N	no	
SW	0.5	N	no	
S	0.5	N	no	
S	0.5	N	no	
SR	0.5	N	no	
S	1	N	no	
L	1	N	no	
S	1	N	no	
SR	1	N	no	
S	1	N	no	
S	2	N	no	
SR	3	N	no	
N	10	N	no	
N	12	N	no	
S	20	N	no	
FLY	10	N	no	
S	30	N	no	

S	30	N	no	
S	30	N	no	
FLY	10	N	no	
LC	10	N	no	
LC	20	N	no	
LC	30	N	no	
LC	30	N	no	
LC	50	N	no	
		N	no	
		N	no	
		N	no	
		N	no	
		N	no	
FO	6	N	no	
C	10	N	no	
SR	12	N	no	
FLY	12	N	no	
C	20	N	no	
C	15	N	no	
SR	5	N	no	
SR	8	N	no	
SR	12	N	no	
SR	20	N	no	
LC	20	N	no	
FLY	20	N	no	
LC	20	N	no	
LC	30	N	no	
		N	no	
		N	no	
		N	no	
		N	no	
		N	no	
A	0	N	no	
SR	0.1	N	no	
SR	1	N	no	
S	1	N	no	
N	3	N	no	
N, S	4	N	no	
S	5	N	no	
SR	5	N	no	MOCH (possible female) calls in area, RBNU (possible fer
SR, S	8	N	no	
S	10	N	no	
FLY	10	N	no	
S	15	N	no	

C	20	N	no	
A	0	N	no	
SW	0.1	N	no	
SW	0.5	N	no	
SR	0.5	N	no	
SR	0.5	N	no	
SR	1	N	no	
L	1	N	no	
N	1	N	no	
N	1	N	no	
N	2	N	no	
SR	3	N	no	
SR	3	N	no	
N	3	N	no	
SR	4	N	no	
SR	5	N	no	
AC	5	N	no	
puffs feath	5	N	no	
N, AC	6	N	no	
N	8	N	no	chases MOCH
AC	8	N	no	
SR	10	N	no	
SR	10	N	no	
AC	12	N	no	
AC	15	N	no	
AC	20	N	no	
LC	30	N	no	
AC	35	N	no	
LC	40	N	no	
		N	no	
		N	no	
		N	no	AC to other RBNU
		N	no	
		N	no	
S	10	N	no	
SR	10	N	no	
SR	10	N	no	
FO	10	N	no	
SR	12	N	no	feeds other MOCH in cavity
C	15	N	no	
S	15	N	no	
FO	15	N	no	
S	30	N	no	
SR	0.3	N	no	

SR	1	N	no	
AC	1	N	no	
SR	1	N	no	
N	3	N	no	
SR	4	N	no	
AC, SR	4	N	no	
SR	4	N	no	
N	5	N	no	
SR	5	N	no	
SR	6	N	no	
SR	8	N	no	
C	8	N	no	
SR	10	N	no	
AC	10	N	no	
SR	12	N	no	
C	15	N	no	
		N	no	
		N	no	
		N	no	
		N	no	
FO	10	N	no	
		N	no	
C	20	N	no	
AC	40	N	no	
S	15	N	no	
S	15	N	no	
S	15	N	no	
N	10	N	no	
SR	15	N	no	
FLY	15	N	no	
LC	40	N	no	
LC	50	N	no	
LC	50	N	no	2 males present
		N	no	
		N	no	
		N	no	
		N	no	
		N	no	
CB	10	N	no	
S	10	N	no	
FLY	10	N	no	
S	10	N	no	
S	12	N	no	
S	15	N	no	

S	20	N	no	possible BCCH: pair excavating, male singing, BCCH chas
S	10	N	no	
N	12	N	no	
SR	12	N	no	
C	15	N	no	
FLY	20	N	no	
L	0.5	N	no	
SR	1	N	no	
SR	1	N	no	
SR	2	N	no	
SR	4	N	no	
SR	5	N	no	
SR	8	N	no	
SR	8	N	no	
SR	8	N	no	
SR	10	N	no	
SR	10	N	no	
CB	12	N	no	
LC	12	N	no	
LC	15	N	no	
AC	15	N	no	
CB	20	N	no	
LC	30	N	no	
LC	50	N	no	
LC	50	N	no	chases other BCCH
FLY	6	N	no	
		N	no	
		N	no	
		N	no	
		N	no	
		N	no	
N	7			
in AR				
A, AC	0			
A	0			
SW	0.25			
N	0.5			
S	1			
S, AC	1			
AC	1			
S	10			
C	20			
C	20			
SR, L	80			

S		
SR,L		
in AR		
L, SR	0.5	
NR	0.5	
SR, L	1	
LIP, SR	1	
C	2	
SR, L	3	
S	5	
S	5	
S	20	
LC	30	
SR, L	95	
FLY AWAY		
in AR		
NR	10	
FLY		
S	15	
stop S	15	RNSA, TRES in Area
C	50	
L,C, AC	5.5	
L,C, AC	5.5	
C, L, WF	7	
C, L, WF	7	
LIP	10	
AC	10	
L	15	
FLY	20	
FLY	20	
S	30	
S	30	
S	30	
S	30	
S	40	
S	40	
stop S	40	
S	40	
S	40	

C
drumming 15
20
25
NR
drumming

LIP, N

EN	1	E	N	FL	8
LIP	1	E	N		7
LIP	1	E	N		7
EN	1	E	N	EN with small green caterpillar	8
N	1	E	N		6
EN	1	E	N		8
IN	1	E	N		
C	3	E	N		9
C	5	E	N		9
FO	5	E	N		1
FO	5	E	N		1
S	8	E	N		13
C	10	E	N		9
S	10	E	N		13
C	10	E	N		9
C	10	E	N		9
FO	10	E	N		1
S	10	E	N		13
FO	10	E	N		1
FO	15	E	N		1
FLY	15	E	N	Chicks hatching	2
C	10	E	N		9
L	1	C			10
AR	2	C		Flys towards nest but does not enter with food	
AR	2	C			
AR	3	C			
AR	3	C			
AR	4	C			
AR	4	C		With food	
AR	4	C		Second adult also with food	
C	5	C			9

AR	6	C		
AR	10	C		
C	10	C	One BCCH looks at ARN, start of mp3 delayed	9
C	10	C		9
FO	10	C	FO on aspen	1
FO	10	C	Both adults FO on aspen	1
FO	10	C	Both adults FO on aspen	1
FO	10	C	Both adults FO on aspen	1
FO	5	C	Both adults FO	1
FO	5	C		1
C		C		9
C		C		9
FC		C	Exits with feacal sac	4
FC		C	Exits with feacal sac	4
FC		C	Second adult FC and exits with feacal sac	4
LIP		C	L	10
C	20	C		9
CB	10	C	Unidenitifed chases BCCH	14
EN,FC	2	C		
EN,FC	2	C		
FC	2	C		
FC	2	C		
N	3	C		
AR	4	C	Food in beak	
C,FO	10	C	FO on At leaves	
C,FO	10	C	FO on At leaves	
C	10	C		
CB	10	C	Chases RBNU male	
AR	15	C	Orange worms in beak	
FO	15	C		
CB	15	C		
S	70	C		
AR	10	C		
S	15	C	Singing from old nest tree 7278	
C	50	C		
C	80	C		
SW	0.25	I		
L	0.5	I		
LIP	1	I		
FC,N	1	I		
LIP	1	I		
LIP	1	I		
L	3	I		
S	3	I		

N,S	4	I		
S	10	I		
S	15	I		
SR,N	1	I		
FLY	1	I		FLIES WITHIN 1M OF MODEL
L	2	I		
L	2	I		
AR	5	I		2ND MOCH W/ FOOD-ATE IT
LIP,EN	6	I		W/ FOOD
LIP	6	I		
LIP	6	I		STILL AT LIP
C	10	I		
FO	15	I		
SR	4	I		
SR	8	I		
FO	10	I		CAV EXCAVATED BY BCCH PR THIS YEAR 2005
N	4		NO	
SR	4		NO	
FLY	5		NO	
FEEDS OTH	6		NO	
FO	10		NO	
S	20		NO	
S	20		NO	
S	50		NO	
SW	0.5		NO	
S	0.5		NO	
S	0.5		NO	
SW	0.5		NO	
S	0.5		NO	
S,N	1		NO	
S,N	2		NO	
	4		NO	
S	8		NO	
S	8		NO	
S	10		NO	
AC	12		NO	
S	12		NO	
	12		NO	
S	20		NO	
S	20		NO	
S	30		NO	
S	50		NO	NO RBNU TRACK RECORDED FOR THIS PLAYBACK
S	60		NO	
S	75		NO	

SW	1	NO
SR, L	3	NO
SR, LIP	4	NO
FLY	6	NO
S	8	NO
FLY, C	30	NO
S	30	NO
S	40	NO
S	40	NO
S	50	NO
S	60	NO
FLY		NO
		NO
		NO
		NO
EN	2	NO
N	3	NO
	5	NO
N	6	NO
S	6	NO
FEEDS F	10	NO
S	10	NO
S	10	NO
C	10	NO
FLY	15	NO
FLY	15	NO
C	15	NO
FO	15	NO
FO	15	NO
S	15	NO
AR		NO
AR		NO
LIP	2	NO
FEEDS F	2	NO
		NO
		NO
		NO
EN	5.5	NO
EN		NO
FL		NO
EN		NO
FL		NO
WF, FC		NO
CH RBNU		NO

CH RBNU			NO
FO	3		NO
N	4		NO
W/ FOOD	4		NO
FO	5		NO
S	6		NO
S	10		NO
CH RBNU	10		NO
FLY			NO
FLY			NO
N	0.5		NO
N	0.5		NO
SW	1		NO
SW	1.5		NO
SR	4		NO
N	5		NO
L, N	7		NO
S	40		NO
S	40		NO
			NO
FLY			NO
			NO
			NO
			NO
A	0	PN	no
SW	0.5	PN	no
L	1.5	PN	no
S, N	1.5	PN	no
N	2	PN	no
WF	3	PN	no
FO	3	PN	no
S	4	PN	no
EN	20	PN	no
C	25	PN	no
LC	10	PN	no
LC	25	PN	no
LC	40	PN	no
		PN	no
		PN	no
		PN	no
LIP	3	PN	no
		PN	no
		PN	no
EN	1	E	N

FL	1	E	N		5
EN	1	E	N		8
FL	1	E	N		5
FO	2	E	N		1
C	3	E	N		9
FO	15	E	N	Chicks hatching	1
C	20	E	N		9
FO	1	E	N	FO on Aspe Wing fluttering	1
EN	2	E	N	EN After being fed by other MOCH	8
HD	2	E	N		
HD	2	E	N		
FL	2	E	N		5
EN	2	E	N		8
LIP	2	E	N	Feeds mat FC	7
C	10	E	N		9
AR	10	E	N		
C	0.25	E	N		9
L	0.5	E	N		10
SR	2	E	N	Searching around base of nest tree	11
L	3	E	N		10
AR	5	E	N		
L	5	E	N		10
FO	5	E	N		1
AR	5	E	N		
FLY	10	E	N		2
N	10	E	N		6
AR	10	E	N		
C	10	E	N		9
C	10	E	N		9
C	15	E	N		9
C	40	E	N		9
C	50	E	N		9
C	60	E	N		9
FO	10	E			1
FO	10	E			1
C	5	E			9
AR	10	E		LIP of DOWO nest nearby, pair switched at cav	
AR	10	E		LIP of DOWO nest nearby, pair switched at cav	
D	20	E		drumming	
FL	2	E			5
LIP	2	E			7
EN	2	E			8
HD	2	E			
HD	2	E			

FC	2	E		4
FC	2	E	feeds mate out of cav	4
wing flutter	2	E	EN	
FC	2	E		4
C	15	E		9
FO	20	E	FO on At leaves	1
FO	20	E	FO in Aspen	1
EN,FC	2	C		
EN,FC	2	C		
EN,FC	3	C		
EN,FC	3	C		
wing flutter	4	C		
A	0	PN		
A	0	PN		
EN	0	PN		
A	0	PN		
SR	0.25	PN		
LIP	0.5	PN		
S	0.5	PN		
N	1	PN		
N, LIP	1	PN		
EN	1	PN		
N	1	PN		
C	2	PN		
C	2	PN		
SR	3	PN		
C	3	PN		
C	5	PN	AGR and GAPO	
pr.cop.	-	PN		
S,N	2	L		
N,LIP	2	L		
N	2	L		
EN	2	L		
C	5	L		
S	25	L		
C	40	L		
S	40	L		
C,FO	50	L		
S	70	L		
FL		L		
SR	5	L		
LC	8	L		
LC	15	L		
LC	40	L		

LC	60	L	
C	70	L	
A,AC	0	PN	
S,A	0	PN	
S,AC	1	PN	
L,SR,N	1.5	PN	
EN	1.5	PN	
S	2	PN	
C	15	PN	
S	15	PN	
C	70	PN	
A	0	PN	
A	0	PN	
A	0	PN	
EN	1	PN	
FL	1	PN	
C	2	PN	
C,FO	5	PN	
C	15	PN	Test of Pederson songs
S	100	PN	
SR	0	PN	
A	0	PN	
SR	2	PN	
SR	2	PN	
S	4	PN	
LIP	5	PN	
HD	5	PN	
N,SR	5	PN	
EN	5	PN	
HD	5	PN	
FL	5	PN	
C	6	PN	
CB	10	PN	Pr. Copulates 20mins after playback
S	20	PN	
S	20	PN	
S	80	PN	
S	80	PN	
S	100	PN	
AC	15	PN	AS MOCH CHASES HIM AWAY
C	20	PN	
C	30	PN	
C	30	PN	
C	50	PN	
A	0	PN	

SR	2	PN	
SR	5	PN	
EN	6	PN	
EXT	6	PN	
EN	6	PN	
EXT	6	PN	
LIP	6	PN	
LIP	6	PN	
AR	7	PN	
S,N	7	PN	
FL	8	PN	
AR	10	PN	
S	10	PN	
CH	15	PN	CHASES BCCH
SW	0.1	L	
SW	0.3	L	
IN	2	L	Nest has 8 eggs
Feeds F	2	L	
LIP	2	L	
SR	2	L	
Feeds F	2	L	
EXT	2	L	F pokes head out and exits cav
LIP,EN	2	L	
LIP, Feeds	2	L	
LIP, Feeds	2	L	
EXT, FL	2	L	pr. FL off together
LIP	2	L	
EN	2	L	
LIP, FL	2	L	
Feeds F	3	L	
C	4	L	
C,S	10	L	
C	15	L	
FO	15	L	
AR	5	E	
NR Nest		E	
A	0	E	M banded AYRW?
S	1	E	
S	3	E	
IN	5	E	8 eggs
C	5	E	
C	5	E	
FL	5	E	FL in
IN	5	E	

S		10	E	
S		15	E	
S		100	E	
FL			E	Both FL out
C			E	Pr. Very upset
FL,LIP		1.75	PN	
pokes heac		1.75	PN	
N		2.5	PN	
SR		4	PN	
SR		4	PN	
C		70	PN	Prev. saw MOCH EN
EXT			PN	
FL			PN	Pr. Flies off
IN		2.25	PN	
S		10	PN	
S		10	PN	
C,AC		15	PN	
S		20	PN	
AC		20	PN	M MOCH CH another MOCH
CH		20	PN	F still in cav, 2 MOCHs still fighting
S		50	PN	
C		50	PN	
C		60	PN	
SW		0	PN	
A		0	PN	
IN		0.3	PN	IN cav for entire PB
EXT		0.3	PN	
LIP,EN		0.3	PN	
SR		0.5	PN	
S		0.5	PN	
C		1.5	PN	
S		3	PN	
S		5	PN	
AR		10	PN	2nd M FL over
A	O		PN	
FL			PN	FL off
S		40	L	
SW		0.25	L	
AC		0.25	L	
AC		0.5	L	
AC		1	L	
S		1.75	L	
S		1.75	L	
SR		1.75	L	

IN	4.5	L	9 EGGS
EN	4.5	L	
LIP, Feeds	4.5	L	
AC	5	L	
S	6	L	
S	6	L	
C	10	L	
EXT	10	L	
S	10	L	
SR	7	E	
A	0	E	
A	0	E	
SW	0.25	E	
AC,SR	0.5	E	
S	1	E	
S	1	E	
SR	1	E	
SR	1.5	E	
IN	2	E	
EN	2	E	
IN	2	E	
S	5	E	
N,SR	7	E	
FL,C	8	E	
SR	8	E	
C	10	E	
S	15	E	
CB	30	E	MOCH chases BCCH away
S	30	E	
C	25	E	
S	25	E	
C	25	E	
S	25	E	
SR	10	E	
SR	10	E	
LC	40	E	
LC	40	E	
LC	40	E	
LC	40	E	
LC	40	E	
LC	40	E	
N	7	E	
N,SR	8	E	
SW	0	E	

SW	0	E	
A	0	E	
EN	1	E	
LIP	1	E	
FL	1	E	
C	2	E	
FL,C	2	E	
SR,C	2	E	
S	30	E	
L	5	E	
S	5	E	
FL	5	E	
CO	5	E	
AR	25	E	Pr. Fly off together
A	0	E	
SW	0.1	E	
SR	1	E	
L	1	E	
S	1	E	
SR	1	E	
S	1.45	E	
FL	6	E	
AR	6	E	Male fluttered wings
SR	10	E	
SR	10	E	
LIP	1	E	
EN	1	E	
HD	1	E	
C	2	E	
FC	2	E	Feeds Female
SR	2.5	E	
EXT, C	4	E	
S	6	E	
C	15	E	
S	20	E	(3rd MOCH-S, AR)
S	80	E	
SW	0.5	E	
AC	2	E	
N,AC	2	E	
FO	2	E	
AC	2	E	
HD	5	E	
FL,EN	5	E	
S	15	E	

A	0	E	
N,SR	1	E	
LC	1	E	
FB	1	E	
LIP	5	E	
SR	8	E	
LC	8	E	
C	10	E	
C	10	E	
LC	15	E	
EN, HD	5	E	8 eggs, 1 hatching
C	5	E	
EN	5	E	
FL	5	E	
LIP	5	E	(feeds chicks?)
FL	9	E	
FO	10	E	
FO	10	E	
FO	10	E	
S,LIP	5	E	
C	5	E	
EN	5	E	
EXT	5	E	
FO	10	E	
C	5	E	
EN	5	E	
EXT	5	E	
EN	5	E	
C	5	E	C from inside cav
EXT	5	E	
EN	5	E	
EXT	5	E	
EN	5	E	
LIP, FC	5	E	Feeds Female
SR	7	E	
FO	7	E	Then fly off
FC	8	E	Feeds Female
C	10	E	
A	0	E	
SW	0	E	
SW	0	E	
A	0	E	
FL	1	E	
EN	1	E	

FL	1	E	
AC,N	1	E	
EN	1	E	
S,N	1	E	
FL	1	E	
BWD	1	E	
SR,N	1	E	
EN	1	E	
AR	2	E	
AC	5	E	
C	30	E	
A	0	I	
S	1.5	I	
HD	4	I	
SR	7	I	
AC	7	I	
S	60	I	
N	6	I	
FC	8	I	
HD	8	I	
FL	8	I	
EN	8	I	
FC	8	I	
LIP,FC	8	I	
N	9	I	
S,N	9	I	
LIP	9	I	
C	10	I	
N	6	I	
LC,AC	10	I	
C	20	I	
AC	20	I	
C	30	I	
S	3	I	
FO	3	I	Took a lrg lepidopteran (poss. Tent caterp) off At tree, brok
N,SR	3.5	I	
S	8	I	
S	60	I	
HD	2	I	
FL	2	I	
EN	2	I	
FC	2	I	
HD	2	I	
FL	2	I	

S,FC		2	I	
IN		2	I	
LIP		2	I	
N		4	I	
FO		5	I	
C		8	I	
C		15	I	
C		15	I	
C		20	I	
S		60	E	
EN		5.5	E	
N, FC		6.5	E	Feeds Female
AR		8	E	
SR		8	E	
NA	NA		E	M not around, TRES at LIP
EN		5.5	E	
EXT		5.5	E	
C		8	E	Preening
C		8	E	
EXT,C		12	E	
FO		12	E	
S		15	E	Had to restart MOCH since M was not around
S		15	E	
S		30	E	
IN, HD		1	E	
EN		1	E	
LIP		1	E	
L		1	E	
AR		1.5	E	Has a green worm
FC		1.5	E	Feeds Female
N		1.5	E	
SR		1.5	E	
L		2	E	
FL, C		2	E	
C		2	E	
C		2.5	E	
FO		3	E	
C,FO		3	E	
S		6	E	
C		6	E	
FO		9	E	
FO		10	E	
S		4	E	
S,N		5.5	E	

LIP, FC	5.5	E	Feeds Female
EN	5.5	E	
S	10	E	
C	10	E	
C	10	E	
C	12	E	
C	12	E	
SR	13	E	
S	20	E	
S	20	E	
FO	20	E	
AR,C	50	E	Could not look in cav, but ad. IN cav (incubating)
EXT		E	
N	3	E	
SR	5	E	
SR	10	E	
LC	30	E	2ND RBNU
SC	50	E	
L	1	I	
L	2	I	
SR,N	4	I	
C	20	I	
A	0	I	
A	0	I	
SR	1	I	
AC	1	I	
AC,SR	1	I	
S	1	I	
L	1	I	
SR,SWAY	1	I	
SR	1.5	I	
HD	3	I	
IN,C	3	I	
FC,LIP	3	I	
SR	3	I	
LIP	3	I	
EN	3	I	
HD	3	I	
N,C	5	I	
AC	10	I	
FL,C	15	I	
FL,C	16	I	
C	30	I	
A	0	E	

N,L	0.25	E	
N,SR	0.5	E	
SR	0.75	E	
FO	1	E	Found a large caterpillar
N	1	E	
LIP	3	E	Female IN
LIP	3	E	
S	4	E	
S	4	E	
L	4	E	
SWAY	4	E	Swaying head back and forth
SWAY,SR	4	E	
S	30	E	3rd MOCH in area
S	40	E	3rd MOCH in area
S	60	E	
A	0	C	
SW	0	C	
A	0	C	
SW	0.1	C	
EN	0.3	C	
HD-EXT	0.3	C	
LIP	0.3	C	2nd MOCH
LIP,L	0.3	C	
EN	0.3	C	Enters w/ food
IN,HD,L	0.3	C	
IN	0.3	C	
EXT	0.3	C	
LIP,EN	0.3	C	EN w/ food
EN	0.3	C	
EN, EXT	0.3	C	Enters cav w/ food, exits with fecal sac
C	1.5	C	
SR	2.5	C	
SR	2.5	C	MOCH has food in beak
AR	3	C	ad has food
C	3	C	
AC	4	C	
AC	8	C	2nd MOCH
C	15	C	
FO	20	C	
HD	1	C	
EN	1	C	
FL	1	C	
FC	1	C	
FC	1	C	All feeding trips male brought BWs; bad weather day

C	2	C	
FL	3	C	
AR	3	C	
SR	4	C	
Preening	4	C	
FO	6	C	
A	0	C	
SW	0.25	C	
SR	3	C	
AC	3	C	
S	3	C	
SR	4	C	
EN	4.5	C	EN w/ food
EN,EXT	4.5	C	EN w/ food
EN,EXT	4.5	C	2nd MOCH EN w/ food
EN,EXT	4.5	C	EN w/ food
EN,EXT	4.5	C	
S,N	4.5	C	
SR	5	C	
C	5	C	
FO	15	C	
EXT,FL	20	C	
C	20	C	
S	40	C	
S	40	C	
LC	100	C	
EN	0.7	C	W/ FOOD
HD	0.7	C	MOCH IN CAV
EXT	0.7	C	
EN,FC	0.7	C	2ND ADULT
EXT,FL	0.7	C	
EN,FC	0.7	C	
EN,FC	0.7	C	
EN	0.7	C	
LIP	1.25	C	
N,C	2	C	
SR	2.5	C	
SWAY	2.5	C	(BROKEN WING)
SWAY	2.5	C	
AR	2.5	C	
C	3	C	
SR	3	C	
AR	3	C	
FL,FO	10	C	

FL,FO	10	C	
EXT,FL		C	2ND MOCH FLIES OFF
FL		C	FLUTTERS WINGS THEN FLIES OFF
FL		C	FLIES OFF
LC	100	C	
SC	100	C	
A	0	C	
A	0	C	
L	0.5	C	
SR	1.5	C	
SR	3	C	
SR	5	C	
EN,FC	6	C	
FC	6	C	orange worm
FC	6	C	had to start MOCH PB again
HD-EXT	6	C	
FC	6	C	
LC	60	C	
LC	60	C	no MOCH around
A	0	C	
L	0.5	C	
L	2	C	
L	3	C	
SR	4	C	
EN	8	C	
EXT	8	C	
EN,FC	8	C	
EN,FC	8	C	
EN,FC	8	C	
FC	8	C	2ND MOCH
AR	9	C	W/ FOOD
AR	9	C	2ND MOCH
L	0.5	C	
S	1.5	C	
S	2	C	
C	10	C	
A	0	C	
S	2	C	
N,C	4	C	
S,FO	4	C	
FC	5	C	
FC	5	C	
SR,N	5	C	
EN	5	C	

EN	5	C	
HD,EXT	5	C	
N	5	C	Has food in beak
SR,N	5	C	
FC	5	C	
FC	5	C	
FC,C	5	C	
C	10	C	
S	10	C	
A	0	C	
SW	0.1	C	
SW	0.1	C	2ND MOCH
SR	1	C	
L	1	C	
L	1	C	
EN	1.9	C	
EXT	1.9	C	
LIP,SR	1.9	C	
EN	1.9	C	
HD	1.9	C	
EXT	1.9	C	
LIP	1.9	C	
EN	1.9	C	
LIP,EN	1.9	C	
LIP	1.9	C	
LIP,EN	1.9	C	
SR	2	C	2ND MOCH W/ FOOD
C	2	C	
SR,N	3	C	HAS FOOD
EXT	4	C	
S,N	5.5	C	HAS FOOD
S	10	C	
S	15	C	
S	20	C	
S	20	C	
S	70	C	
N,SR	5	I	
S	8	I	
C,N	10	I	
S	12	I	
S	70	I	
AC,SR	1	I	
HD	3	I	
EN	3	I	

HD	3	I
SR	6	I
EXT,C	12	I
C	30	I
A	0	PN
SR	1	PN
LIP	1	PN
LIP	1	PN
EN	1	PN
LIP	1	PN
S	1	PN
AC	1	PN
C,SR	2	PN
AR	2	PN
C	15	PN
S	60	PN
C	1.5	PN
AC	3	PN
AC	3	PN
NR	3	PN
L	4	PN
A	0	I
SW	0.25	I
SW	0.25	I
SWAY	1.5	I
SR	3	I
N	4	I
FL	4	I
EN	4	I
LIP	4	I
FC	4	I
EN	4	I
FO	5	I
FO	5	I
FO	8	I
S	50	U
L	1	INC
EN	3	INC
AC	3	INC
L,N	4	INC
C	6	INC
AR	6	INC
C	10	INC
C	40	INC

A	0	LAY	
AR	10	LAY	
AC	10	LAY	
LC	30	LAY	
FC	1	I	
EN	1	I	
C	1	I	
LIP	1	I	
HD	1	I	
FL	1	I	
C	1.5	I	
AC	5	I	
A	0	I	
C	5	I	
S	10	I	
CB	10	I	Chased MOCH
S	15	I	
S	20	I	
S	40	I	
C	5	I	
FC,N	5	I	
		I	
		I	
		I	
S	4	C	MOCH seems to match tune & pitch of notes on CD for M(
FC	5	C	
FO	5	C	FO on At
FC	5	C	
FC	5	C	
N	6	C	
S	10	C	
S	10	C	
FO	10	C	
		C	
		C	
		C	
C	3	I	
C	20	I	
C	20	I	
C	30	I	
		I	
		I	
		I	
A	0	I	

S	1	I	
SWAY	1	I	
AC	2	I	
C	10	I	
C,FO	20	I	
		I	
		I	
		I	
C	8	I	
		I	
		I	
		I	
C,LIP	2.5	I	
EN	3	I	
C	15	I	
FA	15	I	
C,FO	15	I	
		I	
		I	
		I	
S	4	C	SANG UNTIL 9:50
LIP	5	C	
FC	5	C	
FC	5	C	
FA	20	C	
		C	
		C	
		C	
S	20	C	
S	40	C	
S	3	C	
EN	7	C	
S	8	C	
S	15	C	
S	40	C	
LC	60	C	
		C	
		C	
		C	
		C	
FC	5		
S	5		
FC	5		

FC	5
FC	5
FC	5
FC	5

FC	6
S	6
FO	6
FC	6
LIP	6
FC	6
FC	6
C	7
FC	8
S	8
S	10
S	10
FC	
FC	
FC	
FC	
AR	
FO	
FO	

L	1
LIP	1
S	1
EN	1
L	1
FC	1
FC	1

S	60
N, L	3
N, L	3
FC	4
FC	4
FC	4
LIP	4
FC	4
N	5
NR	6
FL, FA	20
S	50
AC	0
S, AC	1.5
N, sway	4
LIP	4
FC	4
FC	4
FC	4
HD	4
FC	4
LIP, L	4
EN	4
AC	6
S	10
FLY	40
FC	3
C	10
S	30
C, FO	40
S	50
S	50
S	50

WF,CO	20	PN	NO
C	20	PN	NO
S	40	PN	NO
S	80	PN	NO
S	80	PN	NO
STOP S	80	PN	NO
		PN	
		PN	
		PN	
N	1	PN	
L	1	PN	
L	1	PN	
N	2	PN	
LIP	3	PN	
L	4	PN	
S	4	PN	
N	10	PN	
S	15	PN	
AR	15	PN	
FA	30	PN	
S	70	PN	
S	70	PN	
C	35	PN	
L	2	PN	
C	4	PN	
L	4	PN	
L	6	PN	
S	40	PN	
C	40	PN	
S	100	PN	
S	100	PN	
S	100	PN	
		PN	
		PN	
		PN	
A	0	PN	NO
L,WF	0.5	PN	NO
SR	0.5	PN	NO
L	0.5	PN	NO
LIP,EN	0.5	PN	NO

LIP		0.5	PN	NO	
L,LIP		0.5	PN	NO	
L		0.7	PN	NO	
L		1	PN	NO	on ground and took off
AR		1	PN	NO	came back to A!
AC		1	PN	NO	took off
S		2	PN	NO	came back to A!
L		3	PN	NO	
AR		4	PN	NO	
L		6	PN	NO	
L		6	PN	NO	
C		6	PN	NO	
S		7	PN	NO	took off
N		8	PN	NO	
S		10	PN	NO	
L		10	PN	NO	
S		10	PN	NO	
AR		12	PN	NO	
FO		15	PN	NO	
FO		15	PN	NO	
C		25	PN	NO	
C,FO		40	PN	NO	
S		60	PN	NO	
LIP	?		PN	NO	
S		80	PN	NO	
			PN	NO	
			PN	NO	
NR			PN	NO	
WF,C,N		0.5	PN	NO	
CO		1	PN	NO	
CO		1	PN	NO	
L,WF		1	PN	NO	
N,L		2	PN	NO	
LIP,EN		2.5	PN	NO	
FL		2.5	PN	NO	
LIP,EN		2.5	PN	NO	
N		3	PN	NO	
FO		4	PN	NO	
FO		4	PN	NO	
N		4	PN	NO	
AR		9	PN	NO	
AR		9	PN	NO	
WF,C,FO		10	PN	NO	
WF,C,FO		10	PN	NO	

AR	10	PN	NO	
AR	10	PN	NO	
WF,FO	12.5	PN	NO	
WF,FO	12.5	PN	NO	
FO	15	PN	NO	
FO	15	PN	NO	
FO	15	PN	NO	
FO,AR	17.5	PN	NO	
FO,AR	17.5	PN	NO	
FO	25	PN	NO	
C	30	PN	NO	
C	30	PN	NO	on and off
C,L,N	1.5	PN	NO	
C	2	PN	NO	
C	3	PN	NO	
C	6	PN	NO	
C	10	PN	NO	
C	15	PN	NO	
C	20	PN	NO	
C	30	PN	NO	
stop C		PN	NO	took off
		PN	NO	
		PN	NO	
		PN	NO	
L	3	PN		
L	5	PN		
L	6	PN		
AC	7	PN		
SR,EN	7	PN		
AC	7	PN		
WF,LC	8	PN		
WF,LC	8	PN		
SR	8	PN		
EN	10	PN		
N,SR	10	PN		
EXC	10	PN		
FA	30	PN		
SC	40	PN		
SC	40	PN		
FO	40	PN		
LC	60	PN		
STOP S	60	PN		
		PN		
		PN		

		PN	
A	0	E	
L	1	E	
AC	1	E	
S	1	E	
L	1	E	
S	1	E	
S	1	E	
SR,S	1	E	
S	1	E	
EN	2	E	
S	2	E	
L	2	E	
AC	2	E	
SR	2	E	
AR	2	E	
C	2	E	
N	2	E	
L	2	E	
AC	2.5	E	
WF	2.5	E	
L	2.5	E	
N,SR	3	E	
S	3	E	
N	3	E	
LIP	3	E	
CB	5	E	MOCH chases RBNU
CB	5	E	MOCH chases RBNU
C	5	E	MOCH chases RBNU
CB	5	E	MOCH pair chases RBNU
AR	5	E	
L	6	E	
S	10	E	
AR	10	E	
FO	10	E	
S	15	E	
S	50	E	
SR	1	E	
AR	3	E	
SR	3	E	
AR	4	E	
SR	5	E	
CB	5	E	
L	10	E	

WF,LC	10	E		
LC	40	E		
LC	50	E		
STOP S	50	E		
LC	80	E		
		E		
		E		
		E		
L	5		NO	E
L	6		NO	E
AC	1		NO	E
LIP, L	1.5		NO	E
C	1.5		NO	E
C	2		NO	E
N	2		NO	E
C	3		NO	E
L	15		NO	E
C	20		NO	E
C	40		NO	E
EN			NO	E
S	80		NO	E
S	100		NO	E
			NO	E
			NO	E
			NO	E
S	15		NO	
S	30		NO	
C	50		NO	
L, C, AC	5		NO	
C, L	7		NO	
C, L	7		NO	
EN	10		NO	
LIP	10		NO	
AC	10		NO	
L	10		NO	
L	15		NO	
L	20		NO	
S	30		NO	
S	30		NO	
S	30		NO	
S	40		NO	
S	40		NO	
S	40		NO	
S	40		NO	

C			NO
D	15		NO
	25		NO
D			NO
			NO
			NO
			NO
LIP	10		NO
N	7	L	NO
		L	NO
A	0	L	NO
A	0	L	NO
SW	0.25	L	NO
N	0.5	L	NO
S	1	L	NO
S, AC	1	L	NO
AC	1	L	NO
SR, L	8	L	NO
S	10	L	NO
C	20	L	NO
S	50	L	NO
S	50	L	NO
SR, L		L	NO
		L	NO
L, SR	0.5	L	NO
N	0.5	L	NO
SR, L	1	L	NO
LIP, SR	1	L	NO
C	2	L	NO
SR, L	3	L	NO
SR, L	5	L	NO
S	5	L	NO
S	20	L	NO
C	30	L	NO
SR, L	95	L	NO
		L	NO
		L	NO
		L	NO
		L	NO
	8	L	NO
	10	L	NO
FO		L	NO
L	12		NO
EN	7		NO

SR, L	7	NO
FL	7	NO
EN	7	NO
LIP	7	NO
LIP	7	NO
CC	8	NO
AR	8	NO
SR, L	8	NO
S	10	NO
S	10	NO
S	12	NO
S	20	NO
S	30	NO
S, N	9	NO
FO	10	NO
S	15	NO
SC	20	NO
S	20	NO
		NO
		NO
		NO
FLY	8	NO
S	15	NO
S	20	NO
S	20	NO
S	20	NO
CH UKWA		NO
L, SR	1	NO
L, SR	2	NO
SR, L	3.5	NO
	4	NO
FLY	8	NO
S	30	NO
S	40	NO
S	50	NO
		NO
		NO
		NO
A	0	NO
SW	0.5	NO
N	3	NO
C	3	NO
EN	3	NO
FEEDS F	3	NO

L, SR	4		NO
S, N	4		NO
L, SR	4		NO
S, N	4		NO
N	4		NO
S, N	4		NO
N, S	4		NO
S, C	5		NO
FO	7		NO
AC	7		NO
S	7		NO
L, SR	8		NO
FO	15		NO
FLY			NO
S	40		NO
			NO
			NO
			NO
		INC	NO
FL	4	INC	NO
EN	5	INC	NO
C, N	6	INC	NO
C, FO	7	INC	NO
C	8	INC	NO
C	15	INC	NO
C	20	INC	NO
IN		INC	NO
		INC	NO
		INC	NO
		INC	NO
		INC	NO
S, N	4		NO
S, N	6		NO
S	7		NO
S, N	10		NO
S, N	15		NO
S	15		NO
FLY	15		NO
FLY			NO
			NO
			NO
			NO
C, N, L	1		NO
C, ARRIVES WITH FOOD			NO

FLY		NO
EN		NO
FLY		NO
EN		NO
L FROM CAV		NO
EN, L		NO
EN, L		NO
IN		NO
		NO
		NO
		NO
C	8	NO
C	10	NO
C	20	NO
S	40	NO
FLY		NO
C	30	NO
C, FL	3	NO
S	60	NO
S	60	NO
S	60	NO
S	60	NO
S	60	NO
S	60	NO
S	60	NO
FEEDS F, C		NO
FLY		NO
FLY		NO
S	80	NO
S	80	NO
S	80	NO
		NO
		NO
		NO
S	60	NO
S	30	NO
S	30	NO
S	50	NO
S	60	NO
		NO
		NO
		NO
S	30	NO
S	40	NO

S	60		NO
S	60		NO
FC			NO
FC			NO
			NO
			NO
			NO
S	10		NO
S	20		NO
S	30		NO
S	40		NO
S	5		NO
FO	5		NO
FO	10		NO
C	15		NO
S	15		NO
S	30		NO
S	30		NO
S	35		NO
LIP, FEED F IN CAV			NO
			NO
			NO
			NO
SW	20	C	NO
EN	2	C	NO
C	2	C	NO
C	4	C	NO
FEED	10	C	NO
FEED	10	C	NO
FL, S	10	C	NO
AC	10	C	NO
AC		C	NO
EN		C	NO
CH RBNU		C	NO
SW	0.25	C	NO
	1	C	NO
WF, L	2	C	NO
L	2	C	NO
WF, L	2	C	NO
WF, SC	10	C	NO
S	10	C	NO
SS	10	C	NO
SC	10	C	NO
		C	NO

		C	NO	
		C	NO	
		C	NO	
		C	NO	
		C	NO	
NR		C	NO	
EN	6	PN	no	
EN	6	PN	no	
FL	6	PN	no	
EN	6	PN	no	
FL	6	PN	no	
C	10	PN	no	
		PN	no	
		PN	no	
		PN	no	
		PN	no	
		PN	no	
A	0	PN	no	
SW	0.5	PN	no	
L	0.5	PN	no	
SR	1	PN	no	
C	1	PN	no	
SR	2	PN	no	
N	5	PN	no	
N	5	PN	no	
S	5	PN	no	
SR	5	PN	no	
S	5	PN	no	
EN	8	PN	no	
EN	8	PN	no	chases BCCH
	10	PN	no	
	10	PN	no	
S	10	PN	no	
SR	10	PN	no	
	12	PN	no	
S	12	PN	no	
S	15	PN	no	
S	15	PN	no	
S	20	PN	no	
S	20	PN	no	
LC	25	PN	no	
drumming	40	PN	no	
		PN	no	
		PN	no	

		PN	no	
		PN	no	
		PN	no	
AC	0.1	PN	no	
SR	0.1	PN	no	
S	0.5	PN	no	
SR	1	PN	no	
SR	1	PN	no	
SR	1	PN	no	
LIP	2	PN	no	
FLY	4	PN	no	
C	4	PN	no	
CB	10	PN	no	
S	10	PN	no	
S	10	PN	no	
S	10	PN	no	
S	15	PN	no	
S	15	PN	no	
S	15	PN	no	
S	20	PN	no	
S	20	PN	no	
S	20	PN	no	
EN	0	PN	no	with nest mat
S	4	PN	no	
SR	5	PN	no	
SR	5	PN	no	
C	15	PN	no	
C	15	PN	no	
C	20	PN	no	
S	50	PN	no	
		PN	no	
		PN	no	
		PN	no	
		PN	no	
CB	1	PN	no	
		PN	no	
C	20	PN	no	
AC,A	0	PN	no	
SR	1	PN	no	
C	1	PN	no	
SR, WF	1	PN	no	
S	1	PN	no	
S	1	PN	no	
S	1	PN	no	calling from withing cavity

SR	2	PN	no	
C	2	PN	no	
S	2	PN	no	
SR	3	PN	no	
SR	5	PN	no	
FLY, S	5	PN	no	
S	8	PN	no	
FLY	12	PN	no	
AC	15	PN	no	
LC	25	PN	no	6 eggs; 08-ARN-003 with 6+ chicks 8-9 days old
AC	30	PN	no	
		PN	no	
		PN	no	
		PN	no	
		PN	no	chases BCCH
		PN	no	
S	10	PN	no	
N	12	PN	no	
LIP	12	PN	no	
S	15	PN	no	
S	15	PN	no	
C	15	PN	no	
C	15	PN	no	
SR	20	PN	no	
LC	30	PN	no	
LC	40	PN	no	
LC	40	PN	no	
LC	40	PN	no	
LC	50	PN	no	
LC	60	PN	no	
		PN	no	
		PN	no	
		PN	no	
		PN	no	
		PN	no	fur in cavity
C	20	E	no	
LC	50	E	no	
		E	no	
		E	no	
		E	no	
		E	no	
		E	no	7 eggs, MOCH in cavity, DOWO on nest tree
N	5	E	no	
C	10	E	no	

FO	20	E	no	
C	25	E	no	
		E	no	
		E	no	
		E	no	
		E	no	
		E	no	
C	20	L	no	
SR	0.5	L	no	
SR	1	L	no	continues to 0:14:10
L	1	L	no	
SR	2	L	no	
SR	2.5	L	no	continues to 0:14:10
SR	3	L	no	
SR	4	L	no	
N	6	L	no	
SR	6	L	no	
C	6	L	no	
C	6	L	no	
C	6	L	no	
EN	10	L	no	two searching together at 1 m
FL	10	L	no	
SR	10	L	no	
FLY	15	L	no	
S	30	L	no	chases BCCH
S	50	L	no	banded: AK/PY
LC	40	L	no	
		L	no	
		L	no	
		L	no	
		L	no	random at nest tree 70692
		L	no	
C	10	E	no	
C	10	E	no	
S	15	E	no	
S	15	E	no	
C	20	E	no	
SR	5	E	no	
FLY	6	E	no	carrying fur
SR	10	E	no	
SR	10	E	no	
C	10	E	no	
SR	15	E	no	
SR	15	E	no	

C	15	E	no	
LC	15	E	no	
LC	15	E	no	
C	15	E	no	
C	15	E	no	
LC	20	E	no	
C	20	E	no	
C	25	E	no	
C	25	E	no	
C	25	E	no	
		E	no	
		E	no	
		E	no	
		E	no	
		E	no	
drumming	15	E	no	
		E	no	
L, AC	0.1	E	no	
L, AC	0.2	E	no	
AC	0.5	E	no	
AC	1	E	no	
SR	1	E	no	
SR	1	E	no	
N, SR	2	E	no	
S	4	E	no	
SR	5	E	no	
S	8	E	no	
S	10	E	no	
SR	10	E	no	
SR	12	E	no	
CB	12	E	no	
CB	12	E	no	in 70830
C, N	5.5	E	no	
N	8	E	no	
S	15	E	no	
S	15	E	no	
SR, L	0.05	E	no	
SR, AC, L	0.1	E	no	
SR	0.1	E	no	
L	0.2	E	no	
SR	0.5	E	no	
SR, AC	0.5	E	no	
SR	1	E	no	
L, SR	1	E	no	

SR, AC, WF	1	E	no
SR	1	E	no
SW	2	E	no
N, SR	2	E	no
N, SR	3	E	no
N, AC	3	E	no
N, SR	5	E	no
AC, SR	5	E	no
CB	10	E	no
FLY	10	E	no
AC	12	E	no
FLY	15	E	no
LC	20	E	no
AC	30	E	no
AC	50	E	no
		E	no
		E	no
		E	no
		E	no
		E	no
LIP	6	E	no
SR	6	E	no
PR	6	E	no
C	7	E	no
SR	7	E	no
SR	7	E	no
LC	20	E	no
LC	30	E	no
LC	30	E	no
LC	40	E	no
LC	40	E	no
C	30	E	no
		E	no
		E	no
		E	no
		E	no
		E	no
SR	0.5	E	no
SW	0.5	E	no
CB	0.5	E	no
SR	0.5	E	no
CB, AC	1	E	no
L	1	E	no
CB, AC	2	E	no

SR	4	E	no	
SR	4	E	no	
AC	4	E	no	
SR	5	E	no	
S	5	E	no	
SR	5	E	no	
CB	6	E	no	
EN	6	E	no	
LIP	6	E	no	
FO	8	E	no	
AC	10	E	no	
SR	10	E	no	
FO	12	E	no	
SR	5	E	no	
SR	6	E	no	
SR	15	E	no	
		E	no	
		E	no	
		E	no	
		E	no	
		E	no	
SR	5	C	no	carrying food
LIP	5	C	no	
EN	5	C	no	carrying food
FL	5	C	no	
EN	5	C	no	carrying food
FL	5	C	no	
EN	5	C	no	
FL	5	C	no	
EN	5	C	no	
FL	5	C	no	
EN	5	C	no	carrying food
FL	5	C	no	
EN	5	C	no	
FL	5	C	no	
EN	5	C	no	
FL	5	C	no	
chicks hea	5	C	no	
SR	6	C	no	
SR	8	C	no	
LIP, SR	5	C	no	
SR	6	C	no	
FLY	10	C	no	
FO	20	C	no	

LC	50	C	no	
		C	no	
		C	no	
		C	no	
		C	no	banded male AW/KY seen FC after playback
		C	no	
EN	4	C	no	
LIP, EN	4	C	no	
FL	4	C	no	
EN	4	C	no	
L	4	C	no	
EN	4	C	no	
FL	4	C	no	
EN	4	C	no	
FL	4	C	no	
EN	4	C	no	
FL	4	C	no	
EN	4	C	no	
FL	4	C	no	
EN	4	C	no	
LIP	4	C	no	
SR	5	C	no	
SR	5	C	no	
C	5	C	no	
WF	8	C	no	carrying lep
FO	10	C	no	
C	12	C	no	
FO	15	C	no	
FO	20	C	no	
LC	20	C	no	
LC	30	C	no	
LC	30	C	no	
LC	50	C	no	
		C	no	
		C	no	
		C	no	
		C	no	
		C	no	
C	1.5	C	no	
FL	1.5	C	no	
EN	1.5	C	no	
FL	1.5	C	no	
EN	1.5	C	no	
FL	1.5	C	no	

LIP, EN	1.5	C	no	
FL	1.5	C	no	
EN, FL	1.5	C	no	
EN	1.5	C	no	
FL	1.5	C	no	
C, N	2	C	no	
N, SR	2	C	no	
C, SR	3	C	no	
C, SR, N	4	C	no	
C	5	C	no	
N	5	C	no	
SR	5	C	no	
SR	5	C	no	
S	10	C	no	new unbanded male
FLY	10	C	no	
C	10	C	no	
FLY	15	C	no	
SR	15	C	no	
		C	no	
		C	no	
		C	no	
		C	no	
		C	no	
L	8	E	no	looks from cavity
LIP, EN, FL	8	E	no	
FO	12	E	no	
FO	15	E	no	no bands
LC	50	E	no	
C	10	E	no	
		E	no	
		E	no	
		E	no	
		E	no	
		E	no	
CB	7	C	NO	DOWO chases MOCH (M)
SR, S	1	C	NO	
SR, S	2	C	NO	
SR	3	C	NO	
SR, S	3	C	NO	
FLY	3	C	NO	
SR	4	C	NO	
FL	4	C	NO	
FC	4	C	NO	
FL	4	C	NO	

EN	4	C	NO	
N	4	C	NO	
SR	4	C	NO	
FLY	4	C	NO	
EN	4	C	NO	
SR	6	C	NO	
SR	6	C	NO	
SR	6	C	NO	
FL	6	C	NO	
SR	7	C	NO	
L	10	C	NO	
SR	10	C	NO	
SR	15	C	NO	
C	40	C	NO	
FL		C	NO	
		C	NO	
		C	NO	
		C	NO	
S, C	6	C	no	
S	8	C	no	
S, C	10	C	no	
S	10	C	no	
S	10	C	no	
S	12	C	no	
S	15	C	no	
S	6	C	no	
EN	6	C	no	
SR	6	C	no	carrying food
EN, FC	6	C	no	
EN, FC	6	C	no	
FO	6	C	no	
EN	6	C	no	
SR	8	C	no	
SR	8	C	no	
S	10	C	no	
C	10	C	no	
WF	10	C	no	
C	10	C	no	
FLY	10	C	no	
WF	10	C	no	
WF	10	C	no	
CB	10	C	no	chases BCCH
WF, BWD, S	10	C	no	
FO	15	C	no	

C	20	C	no
C	20	C	no
C	30	C	no
N, SR	0.5	C	no
N, SR	2	C	no
N, SR	3	C	no
N, SR	8	C	no
FLY	15	C	no
LC	15	C	no
LC	20	C	no
LC	20	C	no
		C	no
		C	no
		C	no
		C	no
		C	no
S	60	C	NO
S	70	C	NO
FLY	1	C	NO
SR	1	C	NO
SR	2	C	NO
FC	3.5	C	NO
FC	3.5	C	NO
FC	3.5	C	NO
EN	3.5	C	NO
EN	3.5	C	NO
C	7	C	NO
FO	7	C	NO
C	10	C	NO
FL	15	C	NO
FLY	15	C	NO
SR	20	C	NO
FLY	20	C	NO
C	20	C	NO
S	20	C	NO
FL	30	C	NO
C	50	C	NO
C	50	C	NO
			NO
		C	NO
		C	NO
SR	8	C	NO
FLY	4	C	NO
FLY, FO	0.2	C	NO

SR	0.5	C	NO	
SR	2	C	NO	
SR,FLY	2	C	NO	
SR	3	C	NO	
FO	3	C	NO	
FO	4	C	NO	
SR	5	C	NO	
FLY, FO	5	C	NO	
LIP	6	C	NO	
FC	6	C	NO	
SR	6	C	NO	
FC,N	6	C	NO	
FLY, SR	8	C	NO	
AR	8	C	NO	HAS FOOD
FO	8	C	NO	
C	9	C	NO	
FLY	10	C	NO	
SR	10	C	NO	HAS FOOD
SR	15	C	NO	HAS FOOD
C	30	C	NO	
C	50	C	NO	
LIP	6	C	NO	
LIP	6	C	NO	
N	6	C	NO	
AC	10	C	NO	
C	10	C	NO	
C	12	C	NO	
C	15	C	NO	
C	20	C	NO	
C	25	C	NO	
AC	30	C	NO	
C	30	C	NO	
		C	NO	
		C	NO	
FEED	4		NO	
FEED	4		NO	
FEED			NO	
FEED			NO	
L	0.5		NO	
N	1		NO	
L	2		NO	
SR, L	3		NO	
N	4		NO	

AR	6		NO		
SR, L	7		NO		
SR, L	12		NO		
S	40		NO		
S	40		NO		
S	40		NO		
S	40		NO		
SR, L	40		NO		
			NO		
			NO		
			NO		
EN WITH FOOD			NO		
N	5	PL	N	trial	6
C	20	PL	N	trial	9
N	2	PL	N	trial	6
LIP	3	PL	N	trial	7
EN	3	PL	N	trial	8
LIP	3	PL	N	trial	7
EN	3	PL	N	trial	8
LIP	3	PL	N	trial	7
FL	3	PL	N	trial	5
EN	3	PL	N	trial	8
LIP	3	PL	N	trial	7
N	4	PL	N	trial	6
C	4	PL	N	trial	9
FLY	4	PL	N	trial	2
N	5	PL	N	trial	6
	10	PL	N	trial	
C	20	PL	N	trial	9
C	15	PN	N		9
A	0	PN	N		16
SW	0.5	PN	N		15
SW	1	PN	N		15
SR	1	PN	N		11
C	2	PN	N		9
N	5	PN	N		6
C	5	PN	N		9
LIP	5	PN	N		7
N	5	PN	N		6
C	6	PN	N		9
C	6	PN	N		9
C	10	PN	N		9
FO	10	PN	N		1
C	20	PN	N		9

C		PN	N			9
S	20	E				13
LIP	2	E			FC	7
HD	2	E				
FC	2	E				4
FC	2	E				4
FC	2	E				4
FC	2	E				4
HD	2	E				
FC	2	E				4
FL	2	E				5
LIP	2	E			C	9
		E		Calling very loudly during playback		
AR	10	E				
FL	3	E				5
FLY	6	E		Being chased by RBNU		2
S	10	E				13
N	15	E			S	13
S	15	E				13
S	20	E				13
S	20	E				13
SW	0	E				15
SW	0	E				15
S	1	E				13
LIP	3	E				7
LIP	3	E				7
L	3	E				10
LIP	3	E				7
HD	3	E				
LIP	3	E				7
FC	3	E				4
HD	3	E				
N	4	E				6
N	5	E				6
N	5	E				6
C	5	E				9
CB	6	E		Chases BCCH		14
N	10	E				6
C	10	E				9
C	15	E				9
FO	15	E				1
AR	25	E				
AR	25	E				
C	60	E				9

A	0	E	N		Both BCCH attack model		16
A	0	E	N				16
C	0.5	E	N			S	13
N	1	E	N			S	13
S	2	E	N				13
AR	2	E	N				
AR	2	E	N				
N	5	E	N				6
C	5	E	N				9
S	10	E	N				13
AR	20	E	N				
AR	20	E	N				
A	0	E	N		Knocks model to the ground		16
Displays	0.5	E	N				
CB	0.5	E	N		RBNU chases away BCCH after they attack m		14
SR	0.5	E	N				11
L	2	E	N				10
C	5	E	N				9
C	5	E	N		Still calling		9
AR	8	E	N				
AR	10	E	N	?	New nest tree within 7137 plot		
S	15	E	N				13
S	20	E					13
S	40	E					13
C	40	E					9
C	60	E					9
LIP		E					7
N		E				LIP	6
N		E				LIP	6
C,S	0.5	C					
S	0.5	C					
S	2	C					
EN,FC	3	C					
EN,FC	3	C					
N,FC,C	3	C					
N,FC,C	3	C					
F,FC	3	C					
N,LIP	3	C					
N,FC	3	C					
LIP	3	C					
C	10	C					
C	10	C					
C	10	C					
C	30	C					

C		C	
CC	0.5	C	
N,SR	2	C	
EN,FC	5	C	
EN,FC	5	C	
N,SR	5	C	
EN	5	C	
FL	5	C	
EN	5	C	
EN	5	C	
C	10	C	
FO	15	C	FO on Fd
C	20	C	
AR	20	C	
S	5	E	
S	10	E	
FO,C	20	E	
FO,C	20	E	
C	20	E	
C,FO	30	E	
S	30	E	
S	70	E	
C,FO	40	E	
LIP,FC	2	E	Feeds female in cav
IN	2	E	
LIP,FC	2	E	
IN	2	E	
LIP,FC	2	E	
IN	2	E	
FC	2	E	
IN	2	E	
LIP,FC	2	E	
IN	2	E	
N	3	E	
CB	5	E	Chased BCCH
FO	5	E	
C	60	E	
C	20	E	
C	20	E	
C	8	C	
S	10	C	
S	20	C	
C	30	C	
EN,FC	2	C	

LIP	2	C	
FL	2	C	
EN,FC	2	C	
EN,FC	2	C	
HD	2	C	
LIP,L	2	C	
LIP	2	C	
EN	2	C	
FL	2	C	
FC	2	C	
EN,FC	2	C	
AC	20	C	
LC	30	C	
C	40	C	
C	40	C	
C	50	C	
FC	6	C	
FC	6	C	
EN,FC	6	C	
FC	6	C	Lrg. Winged insect
FC	6	C	Orange worm
CC	10	C	
L	10	C	Food in beak
C	10	C	
C	20	C	
AR	75	C	
C	100	C	
C	40	C	
C	40	C	
A	0	PN	
A	0	PN	
A	0	PN	
S	1	PN	
S	2	PN	
LIP	3	PN	
EN	4.5	PN	
LIP	5	PN	
pr.cop.	5	PN	
C	7	PN	
SR	7	PN	
FO	10	PN	RBNU nest in this tree but different cav in 2004, too
C	10	PN	
SR	10	PN	
F	25	PN	

C	8	E	
C	8	E	
C	8	E	
C	8	E	
C	10	E	
C	10	E	
C	15	E	
C	15	E	
C	15	E	
C	15	E	
C	20	E	
C	20	E	
SW	0.1	E	
SW	0.1	E	
N,SR	2	E	
AC,CB	2	E	RBNU chased MOCH away
N,SR	2	E	
AC,CB	2	E	RBNU chased MOCH away
IN	3	E	
SR	3	E	
LIP	3	E	
IN	3	E	
SR	3	E	
LIP	3	E	
N,SR	4	E	
LC	4	E	
AC	4	E	
AC	4	E	
N,SR	4	E	
LC	4	E	
AC	4	E	
AC	4	E	
LC	5	E	
SR	5	E	
AC	5	E	
LC	5	E	
LC	5	E	
SR	5	E	
AC	5	E	
LC	5	E	
SR	6	E	
SR	6	E	
LC	10	E	
LC	10	E	

S	30	PN	
S	50	PN	
S	80	PN	
C	10	PN	
C	10	PN	
C	12	PN	
S	15	PN	
C	20	PN	
N,SR	3	PN	
LC,AC	3	PN	
LIP	3	PN	
LIP	3	PN	drumming on lip of 04-RBNU cavity
N,SR,LIP	4	PN	
SR	4	PN	
LIP	5	PN	
SR	6	PN	
N,LC	6	PN	
LC,N	6	PN	
LC,N	6	PN	
CB	10	PN	
CB	15	PN	
LC	20	PN	Male RBNU excavating new cav in tree and calling AR
C	20	PN	
SR	10	E	
SR	15	E	
FO	15	E	
A	0	E	
N,SR	1	E	
LC	3	E	
LIP	6	E	
EN	6	E	
LIP	6	E	
FC	6	E	
CB	8	E	Chased YRWA off N tree
SR	10	E	
FO	10	E	
FO	15	E	
S	15	E	
S	20	E	
SR	4	E	
SR	6	E	
SR	7	E	
LC	30	E	
SR	2	E	

SR	5	E	
LC	30	E	
AC	4	I	
S	15	I	
SR,C	0.1	I	
SR	3	I	
CB	4	I	RBNU chased MOCH
LIP	4	I	
SR	6	I	
LC	8	I	
AC	8	I	
A	0	E	
SW	0.25	E	Has food in beak
N,SR	1	E	Looking for model MOCH
SR	3	E	
S	15	E	
LC	15	E	
C	25	E	
N	3	E	
SR	4	E	
LC	4	E	
N	4	E	
EXT,C	5	E	
LC	40	E	Female IN
LC	40	E	
LC	50	E	
SC	70	E	
S	50	I	
A	0	I	
SR	3	I	
FC	4	I	
N,LIP,SR	4	I	
C	30	I	
C	50	I	
S	15	E	
S	15	E	
FO,C	15	E	Bubbly call
FO,C	15	E	
C	20	E	Bubbly call -FO?
C	20	E	
S	20	E	
S	20	E	
C	25	E	
C	25	E	

C	25	E	
C	30	E	
SR	4	E	
L	4	E	
AR	4	E	2 DEJU neraby
LC	9	E	
LC	15	E	
FO	20	E	
LC	60	E	
C	50	C	
N,SR	3	C	
CH	4	C	Chases CHSP
LC,AC	4	C	
FC	6	C	
EN	8	C	
CH,N	10	C	Chases DOWO
SR	2.5	E	
SR	3	E	
N	7	E	
LIP	7	E	F IN CAV
SR	7	E	
SC,LIP	7	E	
LIP,FC	7	E	FEEDS FEMALE
LC	10	E	
LC	20	E	
SW	0.25	PN	
S	1	PN	
AC	1	PN	
S	1	PN	
C	8	PN	2ND MOCH
S	10	PN	
S	10	PN	
C	35	PN	Fight b/t M MOCH + M RBNU yesterday
A	0	PN	
SR	0.5	PN	
N,SR	4	PN	
FB	4	PN	
AC	4	PN	
LC	4	PN	
C	4	PN	
EN	4	PN	
N	5	PN	
N	5	PN	
LC	6	PN	

WING FLUT	6	PN	
C	8	PN	
NR	10	PN	
NR	20	PN	
NR	10	PN	
SW	0	PN	
A	0	PN	
N	1	PN	
N	3	PN	
LC,SC	4	PN	
NR	10	PN	
SR	3	I	
LIP,FC	4	I	
EN	4	I	
LC,IN	4	I	
LIP	4	I	
FC	4	I	
FC	4	I	
SR	6	I	
SR	8	I	
C	8	I	
LC	12	I	
SC	12	I	
		I	
		I	
		I	
A	0	PN	NO
A	0	PN	NO
A	0	PN	NO
S,N	1	PN	NO
N,S	1	PN	NO
S,N	2	PN	NO
S	2	PN	NO
FL	2	PN	NO
S	2	PN	NO
EN,EX	2	PN	NO
LIP	2	PN	NO
N,LIP	2	PN	NO
EN,EX	2	PN	NO
S	2	PN	NO
STOP S	2	PN	NO
EX	2	PN	NO
S	2.5	PN	NO
EN	3	PN	NO

S	3	PN	NO	
S	4	PN	NO	
S	4	PN	NO	
AR	4	PN	NO	
S	4	PN	NO	
S	5	PN	NO	
S	5	PN	NO	
S	8	PN	NO	
S	10	PN	NO	
AR	10	PN	NO	
		PN	NO	
		PN	NO	
CB	4	PN	NO	
CB	4	PN	NO	
N	8	PN	NO	took off
C	10	PN	NO	
S	12	PN	NO	
S	12	PN	NO	
S	15	PN	NO	
C	15	PN	NO	
C	20	PN	NO	
S	30	PN	NO	
C	30	PN	NO	
S	30	PN	NO	
S	30	PN	NO	
S	40	PN	NO	
C	50	PN	NO	
S	50	PN	NO	
C	50	PN	NO	
N,WF,C	2	PN	NO	
N,SR,L	3	PN	NO	
L,N	4	PN	NO	
CB	4	PN	NO	
CB	4	PN	NO	
N,SR	5	PN	NO	
N,S	5	PN	NO	
SR,L,N	6	PN	NO	
N,SR	6	PN	NO	
N,L	7	PN	NO	
AR	8	PN	NO	
C	8	PN	NO	
CB	10	PN	NO	
S	12	PN	NO	
AR	15	PN	NO	

S	20	PN	NO	
stop S	20	PN	NO	
S	35	PN	NO	
C	50	PN	NO	
		PN	NO	took off
		PN	NO	3 RBNU AR
		PN	NO	
		PN	NO	
S	8	PN	NO	
AC	10	PN	NO	
SR,L,AC	10	PN	NO	
SR,L,AC	10	PN	NO	
S	12	PN	NO	
AR	12	PN	NO	
AR	15	PN	NO	both took off
AR,AC	20	PN	NO	
S	30	PN	NO	
L,AR	7	PN	NO	
C	7	PN	NO	took off
S,L	8	PN	NO	
WF	12	PN	NO	took off
S	15	PN	NO	
S	15	PN	NO	
C	15	PN	NO	
S	20	PN	NO	
C	25	PN	NO	
S	40	PN	NO	
S	50	PN	NO	
S,AR	50	PN	NO	
N	2	PN	NO	
L,C	4	PN	NO	
L	6	PN	NO	
AR	10	PN	NO	2 took off
AR	12	PN	NO	
AR	12	PN	NO	
AR	12	PN	NO	took off
S	40	PN	NO	
S	40	PN	NO	
AR	40	PN	NO	
AR	40	PN	NO	
AR	40	PN	NO	
AR	40	PN	NO	
S	40	PN	NO	on and off
S	70	PN	NO	

S	70	PN	NO	on and off
S	100	PN	NO	
S	100	PN	NO	
		PN	NO	
		PN	NO	
		PN	NO	
S	120	PN		
L	15	PN		
S	40	PN		
S	40	PN		
S	80	PN		
S	80	PN		
FL		PN		
AR		PN		
AR		PN		
FLY AWAY		PN		
		PN		
		PN		
		PN		
		PN		
S	15	PN		
STOP S	15	PN		
C	15	PN		
S	15	PN		
C	15	PN		
S	35	PN		
FLY AWAY		PN		
		PN		
		PN		
		PN		
		PN		
		PN		
		INC	NO	
S	15	INC	NO	TO 11:40
C	25	INC	NO	
S	80	INC	NO	
S	80	INC	NO	
SR	80	INC	NO	
N, SR	3	INC	NO	
LC	3	INC	NO	BOTH
SR	6	INC	NO	
L	10	INC	NO	
FC	10	INC	NO	
FC	10	INC	NO	

LIP	10	INC	NO	
N	10	INC	NO	
LC	10	INC	NO	
FL	10	INC	NO	
LC	15	INC	NO	TO 20:30
LC	25	INC	NO	
LC	30	INC	NO	F IN
FLY	40	INC	NO	
CH OTHER RBNU		INC	NO	
		INC	NO	
		INC	NO	
		INC	NO	
S	20		NO	
S	25		NO	
C	40		NO	
S	50		NO	
L	5		NO	
L	7		NO	
L	8		NO	
SC	10		NO	
SC	10		NO	
FLY	15		NO	
FLY	30		NO	
FLY			NO	
FLY			NO	
			NO	
			NO	
			NO	
	30		NO	
SR	4	E	no	
SR	6	E	no	
S	15	E	no	
FO	15	E	no	
S	25	E	no	
S	50	E	no	
A	0	E	no	
EN	3	E	no	
FC	3	E	no	
N	3	E	no	
SR	4	E	no	
CB	5	E	no	
CB	5	E	no	
FO	15	E	no	

LC	40	E	no	
		E	no	
		E	no	
		E	no	
		E	no	
		E	no	
C	20	PN	no	
NR	8	PN	no	
NR	10	PN	no	
FLY	10	PN	no	chases MOCH
SR	2	PN	no	
AC	2	PN	no	
LIP	6	PN	no	
N	8	PN	no	
LC	8	PN	no	
N,SR	8	PN	no	
SR	8	PN	no	
N	8	PN	no	
CB	8	PN	no	
LC	10	PN	no	
C	10	PN	no	
L	10	PN	no	
C,N	10	PN	no	
CB	10	PN	no	
AC	10	PN	no	
CB	10	PN	no	
SR	12	PN	no	
SR	12	PN	no	
C	15	PN	no	
C	30	PN	no	
FLY	30	PN	no	
C	30	PN	no	
NR	10	PN	no	
		PN	no	
		PN	no	
		PN	no	
		PN	no	
enters near	12	PN	no	
		PN	no	
S	10	E	no	
S	15	E	no	
S	15	E	no	
A	0	E	no	
SW	1	E	no	chases MOCH

SR	1	E	no	
LIP	3.5	E	no	chases MOCH
EN	3.5	E	no	
LIP	3.5	E	no	
EN	3.5	E	no	
SR	4	E	no	
SR	4	E	no	
L	4	E	no	
AC	5	E	no	
SR	5	E	no	
L	5	E	no	
FLY	6	E	no	
CB	6	E	no	
SR	8	E	no	
N, SR	8	E	no	
N, LC	8	E	no	
SR	8	E	no	
N	9	E	no	chases BCCH
N, SR, LC	9	E	no	
N, SR	9	E	no	
LC	10	E	no	chases MOCH
LC	10	E	no	chases MOCH
FLY	10	E	no	female inside cavity
SR	11	E	no	
SR	12	E	no	
SR	15	E	no	
FLY	15	E	no	
LC	15	E	no	
SR	15	E	no	
LC	15	E	no	
SR	20	E	no	carrying nest material?
LC	20	E	no	
LC	20	E	no	
LC	30	E	no	
LC	45	E	no	
LC	60	E	no	
AC	60	E	no	
		E	no	
		E	no	
		E	no	
		E	no	
		E	no	
S	40	E	no	
S	40	E	no	

SR	4	E	no	
N	5	E	no	
N	8	E	no	
FL	10	E	no	
N	10	E	no	
SR	15	E	no	
SR	15	E	no	
FLY	20	E	no	
FLY	30	E	no	chases RBNU
LC	50	E	no	
		E	no	
		E	no	
		E	no	
		E	no	
		E	no	
FO	2	E	no	
SR	1	E	no	
SR	1	E	no	
SR	2	E	no	
N	2	E	no	
SR	2	E	no	
SR	2	E	no	
SR	2.5	E	no	
SR	3	E	no	
SR	3	E	no	
SR	5	E	no	
FLY	5	E	no	
SR	5	E	no	
LC	5	E	no	
SR	5	E	no	
FLY	20	E	no	
LC	20	E	no	
LC	20	E	no	
LC	20	E	no	
LC	25	E	no	
LC	30	E	no	
		E	no	
		E	no	
		E	no	
		E	no	
		E	no	
A	0	L		
SR, S	0.1	L		
AC	0.1	L		

SW, AC	0.1	L	
S, SR	0.5	L	
SW	0.5	L	
AC, L	0.5	L	
SR	0.5	L	
SR, S	1	L	
SR, S	1	L	
S	1	L	
AC	1	L	
S, SR	2	L	
SR	2	L	
S, SR	5	L	
S	5	L	
SR	6	L	
S	20	L	
C	20	L	
C	20	L	MOCH heard nearby
FLY	25	L	
FLY	25	L	
SR	1	L	
L, SR	1	L	
SR	1	L	chases MOCH
SR	1	L	
SW	1	L	
SR	1	L	same tree as MOCH - no fight
SR	1	L	feeds female
SR	1	L	
SR	1	L	
SR	1	L	
SR	1	L	
SR	1	L	
SW	1	L	
SR	1	L	
SR	2	L	
SR	2	L	
SR	3	L	
SR	4	L	
SR	4	L	
SR	5	L	
SR	5	L	MOCH S 10m, RBNU LC before playback start
SR	5	L	
S	5	L	
SR	5	L	
C, SR	5	L	

SR	6	L		
SR	6	L		
N	8	L		
N, SR	8	L		
FLY	15	L		
S	20	L		
SR	20	L		
S	25	L		
S	30	L		chases MOCH
FLY	5	L		
SR	5	L		
CB	5	L		
CB	6	L		
N	7	L		
N	7	L		
EN	7	L		
N	8	L		
N	8	L		
N	8	L		
N	8	L		chases MOCH
N, SR	8	L		
FL	8	L		
LC	15	L		
FLY, LC	20	L		
AC	20	L		
LC	30	L		
		L		
		L		
		L		
		L		
		L		
S	15	E	no	
S	15	E	no	
S	20	E	no	
S	20	E	no	
S	40	E	no	
S	40	E	no	
S	10	E	no	
C	15	E	no	
SR	4	E	no	
SR	4	E	no	
N, SR	4	E	no	
SR	5	E	no	
SR	6	E	no	

FO	6	E	no	
SR	7	E	no	
SR	7	E	no	
LIP	8	E	no	
SR	8	E	no	
LIP, FC	8	E	no	
LIP, FC	8	E	no	
SR	9	E	no	
SR	10	E	no	
CB	10	E	no	
SR	10	E	no	
AC	10	E	no	
AC	10	E	no	
FLY	15	E	no	
FLY	15	E	no	
FLY	20	E	no	
LC	40	E	no	
LC	50	E	no	RCKI heard 30m away throughout playback
LC	50	E	no	
		E	no	
		E	no	
		E	no	
		E	no	
		E	no	
LC	7	E	no	calls from cavity
FC	7	E	no	feeds female
FC	7	E	no	feeds female
SR	10	E	no	
SR	5	E	no	
FC in nearb	8	E	no	
		E	no	
		E	no	
		E	no	
		E	no	
		E	no	
S	35	E	no	
S	35	E	no	
FL	8	E	no	
LC	20	E	no	
C	20	E	no	
LC	30	E	no	
AC	30	E	no	
AC	30	E	no	
C	100	E	no	

		E	no	
		E	no	
		E	no	
		E	no	
		E	no	
AC	15	C, F	yes	
C	20	C, F	yes	
C	20	C, F	yes	
C	20	C, F	yes	
C	30	C, F	yes	
		C, F	yes	
		C, F	yes	
		C, F	yes	
		C, F	yes	
		C, F	yes	1 chick (of 4 remaining in cavity) fledges several minutes a
AC,S,SR	5	C	no	
S	15	C	no	
S	20	C	no	
S	15	C	no	
CB	5	C	no	chases BCCH
EN	8	C	no	
FL	8	C	no	
EN, FL	8	C	no	
N, EN	8	C	no	
FL	8	C	no	
EN	8	C	no	
FO	8	C	no	
FL	8	C	no	
EN, FL	8	C	no	
EN	8	C	no	
LC	10	C	no	
LC	10	C	no	
LC	15	C	no	
CB	15	C	no	chases BCCH
		C	no	
		C	no	
		C	no	
		C	no	
		C	no	5 chicks
S	6		NO	
S	30		NO	
S	30		NO	
S	30		NO	
S	30		NO	

S	30	NO	
S	30	NO	
SC	30	NO	
S	30	NO	
S	30	NO	
S	30	NO	
S	30	NO	
S	40	NO	
C	15	NO	
S	10	NO	
S	12	NO	
S	30	NO	
S	30	NO	
S	40	NO	2 BIRDS SINGING AT 40 M
S	40	NO	
N, L	1	NO	
L, SR	2	NO	
L, SR	3	NO	
AC	4	NO	
L	4	NO	
SC	5	NO	
N	6	NO	
L	6	NO	
L	7	NO	
SC	8	NO	
SC	10	NO	
SC	10	NO	
SC	10	NO	
S	15	NO	
S	40	NO	
S	40	NO	
FEEDS FEMALE		NO	
CH MOCH		NO	
LIP		NO	
FLY		NO	
		NO	
		NO	
		NO	
AR	7 C		
S	20 C		
C	30 C		
FL	50 C		
SW	0.25 I		
N,SR	1 I		

N,SR	1	I	
N	2	I	
L	2	I	
L	3	I	
S	5	I	
N	6	I	
FO	6	I	
C	15	I	

L	1	PN	
N	2	PN	
FO	4	PN	
AC	4	PN	
FO	10	PN	
SR	5	PN	
AR	5	PN	pr copulates
LC	10	PN	
LC	20	PN	
C	20	I	
A	0	L	
N	1	L	
AC	10	L	
LC	12	L	
LC	12	L	
LC	12	L	
SC	20	L	
L	1	PN	
N	2	PN	
FO	4	PN	
AC	4	PN	
FO	10	PN	
SR	5	PN	
AR	5	PN	pr copulates
LC	10	PN	
LC	20	PN	
C	20	I	
A	0	L	
N	1	L	
AC	10	L	

LC	12	L	
LC	12	L	
LC	12	L	
SC	20	L	
A	0	I	
SR,FO	1	I	
N,L	1	I	
SR	1	I	
N,SR	1	I	
SR	2	I	
FO	2	I	
FO	3	I	
FO	6	I	
SR	1	E	
LIP,R	1.5	E	looks in cavity
N,SR	4	E	*Random done at 70101 - old nest tree
SC	15	E	
SC	15	E	
SC,AC	20	E	
LC	25	E	
SC	25	E	
LC	30	E	
SW	2	I	
SR	4	I	
LC	6	I	
LC	20	I	
LC	20	I	
LC	50	I	
C	20		
S	40		
LC	10		
C	10		
L	15		
C	20		
S	25		
S	30		

L	30				
S	50				
C	70				
S	80				
S	80				
STOP S	80				
C	100	NO			
C	40		BCCH-E		9
C	80		BCCH-E		9
C	80		BCCH-E		9
C	15				9
C	30				9
C	40				9
C	40 I				
AC	0.25 I			On model stand - pecking on stand	
N	1.5 I				
C	2 I				
C	3 I				
FO	3 I			FO on spruce	
FO	4 I				
SR	8 I				
C	15 I				
C	20 I				
N,SR	2.5 I				
SR	10 I				
LC	50 I				
C	30		MOCH-E		9
C	40		MOCH-E		9
C	40		MOCH-E		9
C	80		MOCH-E		9
C	80		MOCH-E		9
FO	20		MOCH-C		1
C	30		MOCH-C		9
C	50		MOCH-C		9
C	60		MOCH-C		9
C	60		MOCH-C		9
A	0 PN				
S	1 PN				
C	2 PN				
C,FO	4 PN				
FL	5 PN				

FO	5	PN	
C	10	PN	/AGR (M)
C	10	PN	GAPO (F)
A	0	PN	
SR	1	PN	
S	1	PN	
SR	2	PN	
AR	3	PN	
FO	4	PN	
FO	4	PN	
FO	8	PN	
AR	10	PN	MOCH flies away then returns
FO,C	15	PN	
FO,C	15	PN	
AR	25	PN	MOCH flies away
C	40	PN	
S	8	PN	
S	10	PN	
S	30	PN	
S	70	PN	
C,S	50	PN	
C	50	PN	
C	60	PN	
C	70	PN	
SR	2	PN	
N	4	PN	
SR	6	PN	
N	6	PN	
C	8	PN	
C	10	PN	
C,FO	15	PN	
C	30	PN	
S	60	PN	
FL		PN	
N,SR	1	PN	
N	2	PN	
C	20	PN	
C	20	PN	
C	60	PN	
C	80	PN	
FL		PN	
CH	10	PN	2 TAHU CH each other
SW	0	N	
N,SR	0.5	N	

N		1	N	
N		1.5	N	
L		1.5	N	
N,SR		1.5	N	
N		2	N	
N		2	N	
AR		7	N	
FO		10	N	
S		15	N	
A		0	N	AYRW
S,N		1	N	F C
S		1.5	N	AYRW
S		5	N	
C		5	N	
C		8	N	
S		10	N	AYRW
S		80	N	
S		80	N	
AC		25	PN	
C		25	PN	
cop, FO		30	PN	
C		60	PN	Random plot for both 70502 and 7855
S		60	PN	
S		80	PN	
C		80	PN	
N,SR		1	N	SY (wing flutter)
S		1	N	BCCH diong wing flutter like F, but singing
SR		0.5	N	
N		1	N	
SR		8	N	Banded APOG
S		15	N	(was windy day and overcast)
C		50	N	
A		0	E	
A		0	E	
SR		1	E	
SR		5	E	
FL		15	E	Flys off
S		25	E	
SR	-		E	on model stand looking for model MOCH
SR	-		E	on model stand looking for model BCCH
L		0.5	E	
L,SR		1	E	
SR		2	E	
SR		2.5	E	

SR	3.5	E	
SR,N	8	E	
FO	8	E	
C	15	E	
C	25	E	
S	20	E	
S	20	E	
S	20	E	
S	30	E	
S	30	E	
S	30	E	
S	50	E	
S	50	E	
S	50	E	
C	70	E	
C	70	E	
C	70	E	
A	0	E	
SR	2.5	E	Sitting <1m away from MOCH
CB	3	E	BCCH chases MOCH
S	5	E	
C	10	E	
C	25	E	
S	25	E	
AR	1	E	
SR	3	E	Sitting <1m away from BCCH
SR	15	E	
LC	40	E	
SR	0.5	E	
SR	1.5	E	
SR	5	E	
C	5	E	
SR	6	E	
C	10	E	
S	25	E	
S	50	E	
C	30	PN	
C	40	PN	
S	40	PN	
N,SR	1	PN	
FB	3	PN	Fluffs up & flakes bark
N,SR	4	PN	
C	20	PN	

SR	1	E	
SR	1	E	
S	2	E	
L	2.5	E	
SR	2.5	E	
S	3	E	
S	3	E	
S	4	E	
FO	5	E	
S,FO	6	E	
FO	7	E	
S	7	E	
S	20	E	F near nest w/ M
FC	20	E	Feeds Female
FC	20	E	Feeds Female
SW	0.25		
S	1		
S	2		
S	5		
S	8		
FO,S	8		
SR	3	E	
AC	3	E	
HD	4	E	
SR	5	E	
C	10	E	
A	0	E	
LC	1	E	
N,SR	1	E	
N,SR	3	E	
SR	3	E	
LIP	4	E	
LC	8	E	
LC	20	E	
LC	30	E	
SW	0.25	E	
SW	0.25	E	
L	1	E	
AR	1	E	
AR	1	E	
SWAY	1	E	
SR	1	E	
N,SR	4	E	
C	4	E	

C,FO	8	E	
C	10	I	
AC,CB	10	I	Chases MOCH?
SR	12	I	
AR	15	I	
C	15	I	
SR	1.5	I	
AC	2	I	
LC,AC	3	I	
SR	5	I	
LC	10	I	
C	30	I	
C	40	I	
AC	20	I	
S	30	I	
S	35	I	
S	40	I	
C	40	I	
S	40	I	
C	40	I	
C	50	I	
AR	9	I	
SR	4	I	
CC	6	I	
AR	6	I	
Preening	7	I	
LC	10	I	
LC	10	I	
AC	0.5	E	
L	0.5	E	
C	3	E	
S	4	E	
FO	5	E	
C	10	E	
S	10	E	
C	40	E	
AR	15	I	
C	100	I	
S	2	E	
S,FO	10	E	
FO	4	E	
SR,N	6	E	
FO	6	E	

C,N	12	E	
C,N	12	E	has a worm
FO	15	E	
C	20	E	
C	20	E	
C	20	E	2nd MOCH
S	40	E	
S	50	E	2nd male singing
C	50	E	
LC	25	E	
LC	8	E	
LC	13	E	
L	1	I	
SR	1.5	I	
SR	2	I	
N	2	I	
SR,N	3	I	With food
FO	3	I	
N,SR	3	I	
FO	3	I	
C,FO	20	I	
C	40	E	
C	40	E	
C	40	E	
SR	5	E	
N,SR	10	E	
LC	12	E	
AC	12	E	
LC	12	E	
LC	25	E	
SC	25	E	
LC	50	E	
LC	60	E	
A	0	C	
SR,FO	1	C	
N,L	1	C	
SR	1	C	
N,SR	1	C	
SR	2	C	
FO	2	C	
FO	3	C	
FO	6	C	
S	35		Singing from clump where nest is
S,N	1	C	

S,N	1	C	
FO	3	C	FO on dead Pines, getting orange lep larvae
S	3	C	
C	3	C	
FO	3	C	FO on dead Pines, getting orange lep larvae
S	3	C	
C	3	C	
C,FO	10	C	
C,FO	10	C	
S	15	C	
S	15	C	
S	60	C	
S	60	C	
A	0		
AC,SW	0.1		
SW	0.2		
S	1		
N	1.5		
S	1.5		
S	2		
AC	2		
SR	3		
S	3		
FO	4		
FO	4		2nd MOCH
S	4		
SR	4		
SR,N	3.5	C	
N	7	C	On N, near excavation
LC	20	C	
LC	40	C	
LC	50	C	
SW	0.25	C	
S	1	C	
FL	1	C	
SR	1	C	
SR	2	C	Searching on ground
SR	2	C	
S	2	C	
S	2	C	
S	3	C	
N	4	C	
S	4	C	

S	4	C	
S	4	C	
SR	5	C	
S	5	C	
S	5	C	
C,FO	6	C	
SR	7	C	
C	8	C	
FO,S	8	C	
FL	10	C	
FO	10	C	
C	15	C	
C	15	C	
C	10	C	
C	15	C	
S	20	C	
LC	30	C	
C	50	I	
S	10	C	
S	10	C	
S	10	C	
S	10	C	
S	40	C	
S	40	C	
S	50	C	
S	50	C	
S	4	I	Really young - 2nd year
S	8	I	
S	10	I	
A	0	I	
N,L	0.75	I	
C	1	I	
SR	1	I	Searching on ground
C	5	I	
N	5	I	
SR	6	I	
C	50	I	
A	0	I	
SW	0.25	I	
N,LIP	1	I	
S	3	I	
FO	20	I	
S	40	I	
C	50	I	

FO	15	I	
SW	0.25	U	
WF	1	U	
S	8	U	
S	10	U	
C	5	I	
FO	8	I	
N,SR,L	1	C	
S	4		
S	15	C	
S	30	C	
LC	50		
FO	20		
FO	20		
WF	0	PN	NO
WF	2	PN	NO
WF	2	PN	NO
S	5	PN	NO
S	10	PN	NO
S	15	PN	NO
S	20	PN	NO
C	30	PN	NO
C	30	PN	NO
S	30	PN	NO
S	30	PN	NO
S	30	PN	NO
S	30	PN	NO

S	40	PN	NO	
S	50	PN	NO	
S	100	PN	NO	
L	1	PN	NO	
S	1	PN	NO	
S	1	PN	NO	
S	1.5	PN	NO	
FO	2.5	PN	NO	
S	3	PN	NO	
S	4	PN	NO	
S	12	PN	NO	
S	15	PN	NO	
S	15	PN	NO	
C	15	PN	NO	
S	20	PN	NO	
C	20	PN	NO	
S	30	PN	NO	
S	30	PN	NO	
S	30	PN	NO	
S	40	PN	NO	
AR	8	PN	NO	
AR	10	PN	NO	
N,LIP	0.5	PN	NO	
N,L	1	PN	NO	
N	2	PN	NO	
N	4	PN	NO	
L	6	PN	NO	
N	8	PN	NO	
C,N	8	PN	NO	
L	10	PN	NO	
S	20	PN	NO	
stop S	20	PN	NO	
AR	30	PN	NO	
AR	40	PN	NO	
C	50	PN	NO	once!
S	60	PN	NO	
stop S	60	PN	NO	
C	70	PN	NO	
S,SR	5	PN	NO	
L,SR	7	PN	NO	
AR	8	PN	NO	
S	10	PN	NO	

S	30	PN	NO	
S	40	PN	NO	
stop S	40	PN	NO	
S	80	PN	NO	
stop S	80	PN	NO	
		PN	NO	
		PN	NO	
		PN	NO	
L	3	PN	NO	
L	4	PN	NO	
L,N	4	PN	NO	
L,SR	5	PN	NO	
N,C	5	PN	NO	
AR	5	PN	NO	
C	8	PN	NO	
N,WF	8	PN	NO	
N,WF	8	PN	NO	
L	8	PN	NO	
AR	10	PN	NO	
S	12	PN	NO	
AR	15	PN	NO	both took off
AR	15	PN	NO	lost them!
S	20	PN	NO	
C	20	PN	NO	
S	30	PN	NO	
C	30	PN	NO	
S	30	PN	NO	
S	30	PN	NO	
stop S	30	PN	NO	took off
C	40	PN	NO	
S	60	PN	NO	both on and off S!
S	80	PN	NO	
S	80	PN	NO	
stop S	80	PN	NO	
S	80	PN	NO	on and off S
S	100	PN	NO	
S	100	PN	NO	
S	100	PN	NO	on and off S
S	100	PN	NO	
stop S	100	PN	NO	
		PN	NO	
		PN	NO	
		PN	NO	
S,SR	3			

S	3
S	7
S	20
S	30
S	30
S	30
S	50
N,WF,SR	4
N,SR	5
AR	5
WF	6
N,SR,WF	7
N,SR,WF	9
N,SR,WF	10
LC	15
AR	15
WF	15
LC,WF	15
SC	15
SC	15
WF	18
SC	20
FO	20
SC	30
STOP S	30
SC	30
STOP S	30
LC	40

		N	NO	
		N	NO	
		N	NO	
C	13	N	NO	MOBL calls at RBNU
SR	7	N	NO	
SR	10	N	NO	
C	12	N	NO	
FO	12	N	NO	
FO	13	N	NO	
C	13	N	NO	
FO	13	N	NO	
FO	13	N	NO	
FO	13	N	NO	

FO	15	N	NO
C	40	N	NO
C	40	N	NO
C	40	N	NO
C	40	N	NO
C	50	N	NO
C	80	N	NO
			NO
		N	NO
		N	NO
FO	5		NO
FO	6		NO
FLY,SR	7		NO
FO	7		NO
FO	8		NO
FLY,SR	10		NO
FLY,SR	15		NO
S	18		NO
SR,S	18		NO
C	20		NO
C,FO	20		NO
C	20		NO
C	20		NO
C	25		NO
C	30		NO
C	30		NO
C	35		NO
FLY	40		NO
C	20		NO
LC	20		NO
AC	30		NO
LC	30		NO
C	40		NO
C	40		NO
C	40		NO
C	40		NO
			NO
			NO
C	10	C	
FO	10	C	
S	50	C	
A	0	C	
SR	0.5	C	

N,SR	1	C				
SR	1.5	C				
S	5	C				
SR	1	C				
SR	2	C				
N	2.5	C				
FO	4	C				
LC	15	C				
C	10	C				
FO	10	C				
S	50	C				
A	0	C				
SR	0.5	C				
N,SR	1	C				
SR	1.5	C				
S	5	C				
SR	1	C				
SR	2	C				
N	2.5	C				
FO	4	C				
LC	15	C				
SR	1.5	PN				
LC	4	PN				
LC	6	PN				
SR	7	PN				
N,LIP,EXC	8.5	PN				
LIP	8.5	PN				
EXC	8.5	PN				
LC	9	PN				
LC	15	PN				
LC	15	PN		For BCCH and RBNU, RBNU not around - will redo PB at la		
LC	30	PN		New RBNU excavation, incomplete		
N,LC	6	PN				
LC	8	PN				
SC	9	PN				
SC	9	PN		No female AR - cavity not done yet -> male looks like SY-b		
AC	20	PN				
SC	20	PN				
SC	20	PN				
LC	30	PN				
LC	60	PN				
C	0.5		MOCH-C		L	10
AR	1		MOCH-C			
L	2		MOCH-C	has food		10

AR	2	MOCH-C				
S	4	MOCH-C	FO in Aspe	FO		13
AR	5	MOCH-C				
S	6	MOCH-C	FO in Aspe	FO		13
AR	10	MOCH-C				
AR	10	MOCH-C				
AR	20	MOCH-C				
S	20	MOCH-C				13
C	20	MOCH-C				9
C	40	MOCH-C				9
L	1	MOCH-C				10
N	2	MOCH-C				6
FB	2	MOCH-C				12
SR	2	MOCH-C				11
L	3	MOCH-C				10
L	5	MOCH-C				10
S	5	MOCH-C				13
FO	5	MOCH-C	FO on PL bark			1
PR	5	MOCH-C				3
C	10	MOCH-C				9
C	20	MOCH-C				9
C	30	MOCH-C				9
C	30	MOCH-C				9
A	0	RBNU-I				16
SR	1	RBNU-I		L		11
L	1	RBNU-I		SR	N	11
AR	2	RBNU-I				
AR	2	RBNU-I				
SR	4	RBNU-I				11
AR	5	RBNU-I				
FO	5	RBNU-I	FO on PL with BI, gleaning bark			1
AR	5	RBNU-I				
AR	10	RBNU-I	This is a new tree, nest ID 04-ARN-045			
N		RBNU-I		SR	L	11
C	20					9
C	6					9
C	10		FO on youn	FO		9
C	15					9
C	20					9
C	20					9
C	20					9
C	20					9
C	30		RBNU continuing calling on and off for next 7 r			9
C	40					9

C	40			9
AR	10	C	New nest tree, 04-ARN-045	
AR	18	C		
SW	0.25			
S	1			
S	2			
S	5			
S	8			
FO,S	8			
SR	3	E		
AC	3	E		
HD	4	E		
SR	5	E		
C	10	E		
A	0	E		
LC	1	E		
N,SR	1	E		
N,SR	3	E		
SR	3	E		
LIP	4	E		
LC	8	E		
LC	20	E		
LC	30	E		
S	50	E		
S	15	E		
S	20	E		
S	20	E		
C,FO	20	E	(2 MOCH FO, C)	
LC	30	E		
LC	60	E		
LC	60	E		
LC	60	E		
LC	60	E		
AC	80	E		
LC	80	E		
SR	5	I		
SR	5	I		
SC	10	I		
CC	10	I		
LC	15	I		
C	30	I		
LC	30	I		
LC	30	I		
LC	30	I		

LC	60	I	
S	20	I	
SR	1	I	
SR	1.75	I	
SR	3	I	
SR	3	I	
N,SR	4	I	
LC,N	6	I	
C	40	I	
S	6	E	
S	10	E	
S	15	E	
S	15	E	
S	15	E	
S	15	E	
S	20	E	
C	30	E	
N	1	E	
SR	2	E	
SC	20	E	
LC	20	E	
LC	25	E	
LC	25	E	
LC	30	E	
LC	50	E	
LC	50	E	
S,N	1	L	
FO	3	L	FO on dead Pines, getting orange lep larvae
S	3	L	
C	3	L	
C,FO	10	L	
S	15	L	
S	60	L	
C	20	E	
C,FO	20	E	
S,FO	20	E	
S	50	E	
S	70	E	
SR	3	E	
FO	6	E	
FO	6	E	
LC	50	E	Female IN?
LC	50	E	
LC	50	E	

LC	60	E	
LC	60	E	
LC	70	E	
LC	20		
LC	20		
LC	40		

SC	10	I	
SC	10	I	
LC	10	I	
SC	15	I	
SC	15	I	
SC	15	I	
LC	15	I	
LC	30	I	
LC	40	I	

C	40	PN	NO
A	0	PN	NO
S	0	PN	NO
S	0	PN	NO
A	0	PN	NO
S	0	PN	NO
S	2	PN	NO
S	2	PN	NO
S	3	PN	NO
AR	4	PN	NO
AR	5	PN	NO
S	5	PN	NO
S	5	PN	NO
S	5	PN	NO
AC	6	PN	NO
AR	6	PN	NO
AR	10	PN	NO
S	10	PN	NO
AR	15	PN	NO
STOP S	15	PN	NO
S	20	PN	NO
S	20	PN	NO
S	30	PN	NO

C	30	PN	NO
S	40	PN	NO
S	80	PN	NO
S	100	PN	NO
S	100	PN	NO
N,S	3	PN	NO
N,S	4	PN	NO
S	6	PN	NO
L	8	PN	NO
S	8	PN	NO
S	10	PN	NO
FO	10	PN	NO
S	12	PN	NO
S	16	PN	NO
S	30	PN	NO
S	30	PN	NO
FO	30	PN	NO
C	40	PN	NO
C	40	PN	NO
S	50	PN	NO
S	50	PN	NO
S	80	PN	NO
S	80	PN	NO
stop S	80	PN	NO
C	80	PN	NO
L	20	PN	NO
L,WF	20	PN	NO
AR	30	PN	NO
S	50	PN	NO
		PN	NO
		PN	NO
S	70	PN	
S	8	PN	
S	10	PN	
S	25	PN	
C	25	PN	
C	60	PN	
		PN	
		PN	
		PN	
S	80		NO
S	80		NO

S	80		NO			
SR, L	3		NO			
N	4		NO			
SC	4		NO			
S	4		NO			
N, S	5		NO			
S	6		NO			
N	7		NO			
N	7		NO			
	15		NO			
C			NO			
FLY			NO			
FLY			NO			
			NO			
			NO			
			NO			
L	3	N	N			10
FO	10	N	N			1
C	10	N	N			9
FO	20	N	N			1
FO	10	N	N			1
C	20	N	N			9
C	20	N	N			9
C	30	N	N			9
C	10	N	N			9
C	10	N	N			9
C	15	N	N			9
CB	20	N	N			14
CB	20	N	N			14
FO	20	N	N		foraging in FD crown	1
C	20	N	N	N		9
C	40	N	N			9
C	40	N	N			9
C	50	N	N	N		9
C	60	N	N	N		9
C	10	N	N	N		9
C	20	N	N	N		9
C	40	N	N	N		9
C	60	N	N			9
C	60	N	N	N		9
C	70	N	N			9
C	100	N	N			9
C	100	N	N			9
C	5	N	N			9

C	15	N	N	N					9
C	15	N	N	N					9
C	30	N	N						9
C	40	N	N	N					9
C	50	N	N						9
N	15	N	N	MOCH-E					6
N	7	N	N	MOCH-E					6
N	2	N	N	MOCH-E		SR			11
N	5	N	N	MOCH-E		L			10
N	5	N	N	MOCH-E		SR			11
N	6	N	N	MOCH-E					6
C	8	N	N	MOCH-E					9
N	10	N	N	MOCH-E					6
FO	30	N	N	MOCH-E					1
C	30	N	N	MOCH-E					9
C	30	N	N	MOCH-E					9
S	15	N	N	BCCH-E					13
S	15	N	N	BCCH-E					13
FO	25	N	N	BCCH-E	FO on Aspen bark				1
C	30	N	N	BCCH-E					9
C	100	N	N	BCCH-E					9
C	50	N	N	BCCH-E					9
C	50	N	N	BCCH-E					9
C	30	N	N	BCCH-E					9
C	15			MOCH-E					9
FO	15			MOCH-E	FO on Pine	C			9
FO	15			MOCH-E	FO on Aspen				1
C	20			MOCH-E					9
C	20			MOCH-E					9
C	30			MOCH-E					9
A	0			RBNU-E		C			16
A	0			RBNU-E					16
SR	0.1			RBNU-E					11
L	0.2			RBNU-E		N			10
C	0.2			RBNU-E		L	SR	N	11
COP	1			RBNU-E					
COP	1			RBNU-E					
AR	2			RBNU-E					
AR	2			RBNU-E					
AR	3			RBNU-E					
L	3			RBNU-E					10
FO	3			RBNU-E	FO in At				1
AR	5			RBNU-E					
AR	5			RBNU-E					

C	8	RBNU-E			9
AR	10	RBNU-E			
FO	15	RBNU-E	FO in At		1
FO	15	RBNU-E			1
C	20	RBNU-E			9
S	20	RBNU-E			13
C	20	RBNU-E		FO	9
FO	20	RBNU-E			1
S	50	RBNU-E			13
N	15	RBNU-E			6
A	0	RBNU-PN			16
SR	1	RBNU-PN			11
N	2	RBNU-PN			6
AR	2	RBNU-PN			
S	2	RBNU-PN			13
AC	2	RBNU-PN			
L	2	RBNU-PN			10
SR	3	RBNU-PN		N	11
S	3	RBNU-PN			13
N	5	RBNU-PN			6
S	5	RBNU-PN			13
S	15	RBNU-PN			13
FO	10	RBNU-PN	FO on Fd buds		1
FO	15	RBNU-PN			1
SW	0	RBNU-PN			15
SW	0	RBNU-PN			15
AR	1	RBNU-PN			
AR	3	RBNU-PN			
FLY	4	RBNU-PN			2
SR	5	RBNU-PN		N	11
AR	5	RBNU-PN			
AR	10	RBNU-PN			
FO	20	RBNU-PN	FO on Fd bark		1
AR	20	RBNU-PN			
C	30	RBNU-PN			9
C	60	RBNU-PN			9
S	60	MOCH-C			
C	20				
C	20				
C	60				
S	60				
S	80	BCCH-C			
C	60				
C	30	MOCH-C			

CC,S	15		MOCH-C	
CC	15		MOCH-C	
CC	30		MOCH-C	
S	15		RBNU-C	
S	30		RBNU-C	
S	30		RBNU-C	
S	40		RBNU-C	
S	40		RBNU-C	
S	40		RBNU-C	
C,S	5		RBNU-C	Was chased by a HAFL
C	15		RBNU-C	
C	15		RBNU-C	
S	1		RBNU-C	
AR	5		RBNU-C	Was chased by HAFL
LC	15		RBNU-C	
FO	20		RBNU-C	
AC	20		RBNU-C	
C	20		RBNU-C	
S,N,SR	1	Y	RBNU-C	
S,N,SR	5	Y	RBNU-C	Looks in all cavs in area
S	15	Y	RBNU-C	
S	30	Y	RBNU-C	
LC,CC	10		RBNU-C	
C	15		RBNU-E	
C	15		RBNU-E	
C	25		RBNU-E	
SR	5		RBNU-E	
SR	10		RBNU-E	
LC	15		RBNU-E	
C	50		RBNU-E	
C,FO	15		RBNU-C	FO on MPB tree branches
C	40		RBNU-C	
C	20		RBNU-C	
C	50		RBNU-C	
CC	50		RBNU-C	
CC			RBNU-C	
C	30		RBNU-C	
C	40		RBNU-C	
C	50		RBNU-C	
S	15		RBNU-F	
C	15		RBNU-F	
C	20	Y	RBNU-F	Presume 2 ads and 2 yng - circling area, not approaching
C	20	Y	RBNU-F	Presume 2 ads and 2 yng - circling area, not approaching
C	20	Y	RBNU-F	Presume 2 ads and 2 yng - circling area, not approaching

C	20		Y	RBNU-F	
C	30			RBNU-F	
C	40			RBNU-F	
S	60			RBNU-F	
C	20			RBNU-F	
C	40				
C,FO	20				
C	30				
C	40				
C	50				
LC	30				
C	30				
LC	30				
LC	50				
LC	50				
LC	50				
C	50				
LC	60				
C	10				
C	30				
C	30				
C	40				
AC	8		Y		
FO	10		Y		
C,FO	15		Y		
C,FO	15		Y		
LC	8				
C	15				
C	15	PN			
C	20	PN			
C	30	PN			
C	40	PN			
S	100	PN			
S	20	PN			
C	20	PN			
S	40	PN			
A	0	PN			ASY -tried to band
SR	1.5	PN			
S	1.5	PN			
N	2.5	PN			
SR	3	PN			
FL	5	PN			
S	10	PN			
S	40	PN			

C	50	PN	no action
S	80	PN	
C	100	PN	
C	20	PN	
S	50	PN	
C	15	PN	
S	15	PN	
S	40	PN	
S	40	PN	
S	40	PN	
S	60	PN	
S, C	60	PN	
N,SR	5	PN	
SR	6	PN	
FL	15	PN	
FL	20	PN	
LC	20	PN	
C	60	PN	
AR	25	PN	
SW	0	PN	
A	0	PN	
SR	1	PN	
C, wing flut	1	PN	
SR	10	PN	
FO	15	PN	on Sx tree
C	15	PN	
FO	15	PN	
FO	15	PN	
FO	15	PN	
C	25	PN	
SR	4	PN	MOCH pr. FO and copulating during PB
LC	15	PN	after end of playback
A	0	PN	
S	1.5	PN	
S	1.5	PN	
S	10	PN	
S	20	PN	
FL,S	20	PN	
S	100	PN	
C	25	PN	MOCH pr. AR collecting fur, but no action
SR,N	2.5	PN	
SR	8	PN	
C	15	PN	
C	50	PN	

C	50	PN
C	70	PN
C	100	PN
S	1.5	PN
S	2	PN
SR	3	PN
S	5	PN
S	8	PN
C	15	PN
S	15	PN
C	15	PN
S	15	PN
C	15	PN
S	20	PN
SW	0.25	PN
N,SR	1.5	PN
LC	3	PN
N,SR	5	PN
LC	8	PN
C	15	PN
C	30	PN
L	3	
AC	3	
S	3	
S	4	
FO	5	
C	15	
S	15	
C	25	PN
C	15	PN
C	20	PN
SW	0.25	PN
LC,SC	15	PN
LC,SC	15	PN
C	25	PN
EX	40	PN
SC	40	PN
C	20	
SW	2	
SR	10	
N	12	
LIP	12	

LC	10		
AC	10		
FO	12		
LC	15		
LC	30		
FO			
FO			
S	60		
SC	100	PN	NO
STOP C	100	PN	NO
NR			
NR			
N	2	PN	NO
S	7	PN	NO
S,N	15	PN	NO
C	50	PN	NO
S	60	PN	NO
S	50	PN	NO
NR			
NR			
S	5	PN	NO
S	10	PN	NO
S	10	PN	NO
S	20	PN	NO

STOP S	25	PN	NO	
FA	25	PN	NO	
S	100	PN	NO	
S	100	PN	NO	
S	100	PN	NO	
S	100	PN	NO	
S	150	PN	NO	
S	150	PN	NO	
S	150	PN	NO	
AR	6	PN	NO	
LIP	6	PN	NO	
AR	15	PN	NO	
L,N	1	PN	NO	
L	1	PN	NO	
LIP,N	1	PN	NO	
L	1.5	PN	NO	
N	2	PN	NO	
L	2	PN	NO	
L	2.5	PN	NO	
S	20	PN	NO	
S	40	PN	NO	
S	40	PN	NO	
S	40	PN	NO	
S	40	PN	NO	
C	50	PN	NO	
S	50	PN	NO	
STOP S	50	PN	NO	
S	50	PN	NO	
S	50	PN	NO	2 MALES SINGING AT 50M
STOP S				
S	10			
S	15			
S	15			
C	20			
FO	20			
S	25			
C	30			
FO	30			
S	50			

S	50
stopped S	50
C	70
S	100
S	100
S	100
stopped S	100
S	100
C	2
AR	12
L	10
C	0
SW	2
AR	2.5
AR	10
AR	12
S	15
S	100
S	100

C	20
A	0
L	1.5
SR	2
L	2
AR	5
C	10
S	30
S	50
C	50
C	50
S	100

S	15	PN	NO
S	20	PN	NO
S	20	PN	NO
S	20	PN	NO
S	25	PN	NO
S	30	PN	NO
S	30	PN	NO

S	30	PN	NO	
S	40	PN	NO	
S	40	PN	NO	
stop S		PN	NO	
S	100	PN	NO	on and off S
stop S	100	PN	NO	
C	100	PN	NO	
S	120	PN	NO	on and off S
stop S		PN	NO	
stop S		PN	NO	
NR		PN	NO	
		PN	NO	
		PN	NO	
NR		PN	NO	
NR		PN	NO	
NR		PN	NO	
FO	8	PN	NO	
S	10	PN	NO	
stop S	10	PN	NO	
S	12	PN	NO	
stop S	12	PN	NO	
S	25	PN	NO	
S	30	PN	NO	
S	40	PN	NO	
stop S	40	PN	NO	
S	60	PN	NO	same direction
stop S	60	PN	NO	
		PN	NO	took off
		PN	NO	
NR		PN	NO	
		PN	NO	
S	50	PN	NO	
stop S	50	PN	NO	
S	60	PN	NO	
AC,N	8	PN	NO	
C,AC	10	PN	NO	
C,AC	10	PN	NO	poss. Sex F
C,AC	13	PN	NO	
C,AC	13	PN	NO	poss. Sex F
C	15	PN	NO	
C,AC	30	PN	NO	
C,AC	30	PN	NO	poss. Sex F
C	40	PN	NO	start
C	40	PN	NO	

C	40	PN	NO	poss. Sex F
C,AC	40	PN	NO	chasing e.o.
C,AC	40	PN	NO	poss. Sex F
C,AC	40	PN	NO	
S	40	PN	NO	
S	40	PN	NO	
S	40	PN	NO	
C	40	PN	NO	
S	40	PN	NO	
S,C	40	PN	NO	on and off
S,C	40	PN	NO	on and off
stop S	40	PN	NO	
C	45	PN	NO	on and off
S	45	PN	NO	
C,AC	45	PN	NO	
C,AC	45	PN	NO	poss. Sex F
C	50	PN	NO	
S	60	PN	NO	
C	60	PN	NO	
S	60	PN	NO	
S,AR	60	PN	NO	on and off
S	80	PN	NO	
S	90	PN	NO	
		PN	NO	
		PN	NO	
FO	20			
S	40			
S	45			
S	60			
S	75			
S	75			
S	80			
stopped S	80			
S	80			
S	100			
stopped S	100			
S	120			
S	120			
AR				
C	80			

C	100	
S	40	
S	90	
S	100	
S	20	
S	30	
S	30	
S	35	
S	40	
S	10	
S	20	
S	20	
C	35	
S	35	
S	40	
S	40	
S	50	
S	IN AREA	
CB?		CHASES RBNU?
N	4	
WF	4	
S	5	
LIP	5	
EN	5	
drumming (	5	
LIP, EN, KN	5	
LIP, EN, KN	5	
AC	5	
EN	5	
EN, KN	5	
LIP, EN	5	
EXCAVATIN	5	
EXCAVATIN	5	
EN, EXCAV	5	
EXCAVATIN	5	
EXCAVATIN	5	
C, LIP	5	
C, LIP	5	
EXCAVATIN	5	
LIP, EN	5	

EXCAVATIN	5	
L	6	
L	6	
S	6	
CLEANING	6	
FO	6	
WF	6	
L	7	
C	7	
N, FO	7	
L	8	
FO	8	
FLY	10	
FLY	10	
FLY	10	
FLY	10	
FLY	10	
FO	10	
S	10	
S	10	
C	15	
S	20	
C	?	
S,C	5?	
C	IN AREA	
STOP C		
CB		CHASES M BCCH

L	15
C	15
AR	20
S	20
STOP S	20
LC	50
LC	50
LC	60
LC	60

N	12			
S	15			
AR	15			
CB	15			2 BCCHs chasing each other
AC	15			
S	20			
S	50			
S	50			
N	7			
WF,LC	10			
SR	10			
N	12			
SC	15			
C	15			
CB	15			2 MALE RBNUs chase each other
AC	20			
LC	20			both MALEs calling
AR	30			
LC	30			
LC	40			
LC	50			
STOP S	50			
LC	50			
C	100			
S	80			
L	10			
AR	10			
SR	10			
CC	10			
FO,L	10			
LC	15			
FO	20			
S	20 N	no		
S	20	NO	E	
AR	30 N	no		
S	30	NO	E	
S	50 N	no		

S	50		NO	E
C	70	N	no	
C	70		NO	E
NR	15	N	no	
	15		NO	
NR		N	no	
			NO	E
S	25	N	no	
stop S	25	N	no	
S	25		NO	
S	40	N	no	
S	40	N	no	
S	40	N	no	
S	40		NO	
S	40		NO	
S	40		NO	
S	50	N	no	
S	50	N	no	
S	50		NO	
S	50		NO	
NR	40	N	no	
	40		NO	E
N,L,SR	4	N	no	
N, L, SR	4		NO	
L	5	N	no	
L	5		NO	
FO	15	N	no	
FO	15		NO	
C	40	N	no	
C	40		NO	E
S	50	N	no	
S	50		NO	E
C	80	N	no	
S	80	N	no	
S	80	N	no	
C	80		NO	
S	80		NO	
S	80		NO	E
		N	no	
		N	no	
			NO	
			NO	
			NO	
		E	no	

	N	no
S	30	NO
S	30	NO
S	30	NO
S	40	NO
S	60	NO
L	2	NO
SR	4	NO
AC	5	NO
S	5	NO
AC	6	NO
AC, L	7	NO
S	7	NO
S	8	NO
S	10	NO
S	10	NO
S	15	NO
S	15	NO
C	30	NO
S	40	NO
S	40	NO
S	60	NO
S	60	NO
S	60	NO
S	60	NO
S	60	NO
S	70	NO
S	15	NO
S	60	NO
		NO
		NO
		NO
LIP, N		NO
FLY	10	
	10	NO
S	60	
S	60	NO
FLY AWAY		
S	10	
S	10	NO
NR	15	
AR	15	NO
S	30	
S	30	NO

S	40	
S	40	NO
FLY AWAY		
FLY		NO
in cavity	10	
AR	10	NO
		NO
		NO
		NO
SW	1	NO
S	8	NO
S	15	NO
S	15	NO
S	20	NO
L, SR	4	NO
C	5	NO
C	8	NO
S	10	NO
L, SR	15	NO
AC, C	15	NO
C	15	NO
C, FO	20	NO
C, FO	20	NO
C	20	NO
C, FLY	20	NO
S	20	NO
FLY		NO
FLY		NO
FLY		NO
		NO
		NO
		NO
CH MOCH		NO
A	0	NO
FLY	6	NO
FLY	6	NO
CH BCCH	8	NO
S	8	NO
S	8	NO

S	10	NO	
C	10	NO	
S	15	NO	
S	20	NO	
CH BCCH		NO	
FLY		NO	
FLY		NO	
A	0	NO	
A	0	NO	RBNU TRACK PLAYING HERE?
SW	0.1	NO	
SW	0.25	NO	
SW	0.25	NO	
N	0.5	NO	
N	2	NO	
	2	NO	
N	2.5	NO	
L, N	2.5	NO	
L	2.5	NO	
N	3	NO	
L, SR	3	NO	
EN	3.5	NO	
S	4	NO	
SR, WF	4	NO	
AC	4	NO	
S	4	NO	
S	10	NO	
	50	NO	
FLY		NO	
		NO	
		NO	
V	40	NO	
SW	0.5	NO	
SR, L	1	NO	
N	1	NO	
FO, L	1.5	NO	
FO	1.5	NO	
FO	2	NO	
FO	2	NO	
FO	2	NO	
C	8	NO	
C	20	NO	
S	25	NO	
FLY		NO	
L	6	NO	

FLY	10	NO	
C	30	NO	
FLY		NO	
		NO	
		NO	
		NO	
S	10	NO	
S	12	NO	
S	12	NO	
S	20	NO	
S	20	NO	
C	20	NO	
S	40	NO	
S	40	NO	
L	0.5	NO	
SW	1	NO	
SR, L	1	NO	
N, SR	4	NO	
N, L	4	NO	
S	5	NO	
L	6	NO	
S	30	NO	
C	40	NO	
S	50	NO	
S	50	NO	
S	50	NO	
S	50	NO	
S	60	NO	
FLY		NO	
		NO	RBNU track not played?
		NO	
	INC	NO	
N, SR	3	NO	
N, SR	5	NO	
N, SR	6	NO	
SC	10	NO	
LC	15	NO	
LC	15	NO	
FO	15	NO	
SR	15	NO	
LC	20	NO	
LC	20	NO	
LC	25	NO	TO 3:20
FC	35	NO	

LC	40	NO
LC	40	NO
		NO
		NO
		NO
		NO
S	40	NO
S	40	NO
		NO
		NO
		NO
L, SR	2	NO
N	3	NO
SR	4	NO
FLY	6	NO
SR	10	NO
SC	20	NO
SC	20	NO
C	20	NO
SC	30	NO
C	30	NO
S	30	NO
S	30	NO
FLY		NO
C		NO
		NO
		NO
		NO
S, N	10	NO
C	15	NO
C	30	NO
FLY, S	30	NO
A	0	NO
SW	0.1	NO
SW	0.25	NO
SW	0.5	NO
L	0.5	NO
N, L	0.5	NO
S, N	4	NO
LIP	5	NO
SR, L	6	NO
S, N	6	NO
SR, L, N	8	NO
N	8	NO

FLY, N	8	NO	
N, S	9	NO	
S	9	NO	
S	20	NO	
S	20	NO	
S	30	NO	
S	30	NO	
S	30	NO	
S	30	NO	
S	50	NO	
N		NO	
FLY		NO	
FLY		NO	
FLY		NO	
		NO	
		NO	
		NO	
		NO	
C	35	NO	
C	35	NO	
C	35	NO	
		NO	
		NO	
		NO	
		NO	
C, SR	2	NO	
C, FO	2.5	NO	
N, C	3	NO	
L, SR	4	NO	
FO	5	NO	IN AT WS
		NO	
		NO	
		NO	
		NO	
		NO	
		NO	
		NO	
N, L	10	NO	
FLY		NO	
S	8	NO	
		NO	
		NO	
		NO	
N	1	NO	

S	1	NO	
L	2	NO	
S	3	NO	
S	5	NO	
S	10	NO	
S	15	NO	
S	15	NO	
S	30	NO	
		NO	
S		NO	
FLY		NO	
		NO	
		NO	
		NO	
S	50	NO	
L	10	NO	
S	10	NO	
S	20	NO	
S	80	NO	
		NO	
		NO	Poss RBNU
		NO	
SW	0.5	NO	
L	1	NO	
L	1	NO	
L	1	NO	
N	1	NO	
SW	1	NO	
SR	3	NO	
C	4	NO	
PR	4	NO	
N	4	NO	
N	4	NO	
EN	4	NO	
L, N	4	NO	
N	4	NO	
WF, S	5	NO	
L	8	NO	
	10	NO	
SC	12	NO	
S	40	NO	
LIP		NO	
LIP, FLY		NO	
FLY		NO	

PR			NO	
N, FLY			NO	
			NO	
			NO	
S	12		NO	
	12		NO	
S	30		NO	
			NO	
			NO	
			NO	
L, S	12		NO	
FLY			NO	
			NO	
			NO	
			NO	
FEEDS IN AR			NO	EVERY 25 SEC
			NO	
			NO	
			NO	
S	30	C	NO	
S	40	C	NO	
S	40	C	NO	
		C	NO	
		C	NO	
		C	NO	
NR		C	NO	
			NO	
			NO	
			NO	
FO	20	N	no	
SR	0.5	N	no	
SR	1	N	no	
SR	5	N	no	
SR	10	N	no	
S	15	N	no	
S	20	N	no	
S	20	N	no	
LC	20	N	no	
LC	30	N	no	
LC	40	N	no	
LC	40	N	no	
S	20	N	no	
		N	no	
		N	no	

		N	no
		N	no
FO	15	N	no
		N	no
AC	25	N	no
SR	8	N	no
SR	10	N	no
SR	20	N	no
SR	20	N	no
S	30	N	no
		N	no
		N	no
		N	no
		N	no
FO	15	N	no
		N	no
SR	7	N	no
SR	7	N	no
SR	10	N	no
S	40	N	no
S	2	N	no
SR	3	N	no
S	3	N	no
SR	3	N	no
S	3	N	no
SR	4	N	no
SR	5	N	no
S	5	N	no
S	6	N	no
S	15	N	no
S	20	N	no
S	35	N	no
LC	3	N	no
LC	5	N	no
N, C	5	N	no
L	5	N	no
S	5	N	no
S	8	N	no
LC	10	N	no
LC	15	N	no
S	20	N	no
LC	20	N	no
S	30	N	no
		N	no

		N	no	
		N	no	
		N	no	
		N	no	
C	10	N	no	
C	20	N	no	
S	1	N	no	
SR	1	N	no	
SR	1.5	N	no	
SR	2	N	no	
C	2	N	no	
N	2	N	no	
C	5	N	no	
SR	5	N	no	
C	5	N	no	
C	10	N	no	
SR	10	N	no	
C	20	N	no	
SR	1	N	no	
SR	1	N	no	
SR	1	N	no	
SR	1	N	no	
SR	2	N	no	
SR	5	N	no	
LC	10	N	no	
FLY	15	N	no	
		N	no	
		N	no	
		N	no	
		N	no	grass nest inside cavity, pitch around outside
		N	no	
S	20	N	no	
S,L	2	N	no	
C	5	N	no	
SR	5	N	no	
S,L	8	N	no	
C	10	N	no	
C	10	N	no	food in beak
S	10	N	no	
S	10	N	no	
S	15	N	no	
S	30	N	no	
C	30	N	no	
S	30	N	no	

S	40	N	no
SR	0.5	N	no
SR	1	N	no
FLY	1	N	no
L	1	N	no
LC	1	N	no
SW	1	N	no
L	2	N	no
SR	2	N	no
SR	3	N	no
FLY,SR	5	N	no
L	5	N	no
SR	5	N	no
LC	5	N	no
N	6	N	no
LC	8	N	no
LC	10	N	no
LC	25	N	no
LC	30	N	no
LC	30	N	no
		N	no
		N	no
		N	no
		N	no
		N	no
S	20	N	no
S	30	N	no
S	30	N	no
S	40	N	no
SR	1	N	no
L	1	N	no
SR	2	N	no
N	5	N	no
C	10	N	no
C	15	N	no
AC	15	N	no
AC	15	N	no
AC	25	N	no
AC	30	N	no
		N	no
		N	no
		N	no
		N	no
		N	no

S	30	N	no
S	30	N	no
S	10	N	no
S	15	N	no
S	15	N	no
S	15	N	no
S	20	N	no
S	20	N	no
S	30	N	no
S	30	N	no
AC	30	N	no
AC	30	N	no
AC	40	N	no
AC	50	N	no
LC	50	N	no
		N	no
		N	no
		N	no
		N	no
		N	no
A	0	N	no
AC	0.5	N	no
SR	2	N	no
S	3	N	no
SR	3	N	no
FLY	4	N	no
S	4	N	no
SR	4	N	no
S	5	N	no
FO	5	N	no
SR	6	N	no
S	8	N	no
S	10	N	no
C	10	N	no
SR	12	N	no
S	15	N	no
C	15	N	no
SR	15	N	no
C	20	N	no
C	20	N	no
C	20	N	no
C	20	N	no
LC	10	N	no
LC	20	N	no

LC	20	N	no	
LC	20	N	no	
LC	20	N	no	
LC	20	N	no	
LC	40	N	no	
		N	no	
		N	no	
		N	no	
		N	no	
		N	no	6+ eggs
S	15	N	no	
S	20	N	no	
S	20	N	no	
N, SR	6	N	no	
N, LC	6	N	no	
SR	6	N	no	
LC	10	N	no	
LC	15	N	no	
FLY	20	N	no	
LC	30	N	no	
LC	30	N	no	
LC	40	N	no	
LC	40	N	no	
LC	50	N	no	
LC	50	N	no	
LC	50	N	no	
LC	50	N	no	
LC	50	N	no	
LC	50	N	no	
LC	50	N	no	
		N	no	
		N	no	
		N	no	
		N	no	
		N	no	near old nest trees 7076 and 7488
S	20	N	no	
N, SR	10	N	no	
SR	10	N	no	
SR	15	N	no	
AC	15	N	no	
SR	15	N	no	
AC	15	N	no	
FLY	30	N	no	
AC	30	N	no	

LC	30	N	no	
LC	40	N	no	feeding sounds
LC	50	N	no	
LC	50	N	no	
		N	no	
		N	no	
		N	no	
		N	no	
		N	no	
SR	10	N	no	
S	15	N	no	
S	15	N	no	
S	15	N	no	
C	20	N	no	
S	25	N	no	
S	40	N	no	
S	40	N	no	
LC	30	N	no	
AC	40	N	no	5 eggs, male incubating
LC	50	N	no	
LC	50	N	no	
LC	50	N	no	
LC	50	N	no	
		N	no	
		N	no	
		N	no	
		N	no	
		N	no	
S	50	N	no	chases MOCH
S	40	N	no	
S	40	N	no	
SR	8	N	no	
SR	12	N	no	
SR	15	N	no	
LC	40	N	no	
LC	40	N	no	
LC	50	N	no	
LC	50	N	no	
LC	50	N	no	
		N	no	
		N	no	
		N	no	
		N	no	
		N	no	

N, SR	3	N	no	
N, SR	5	N	no	
SR	6	N	no	
LIP	6	N	no	fur in cavity
SR	8	N	no	
SR, C	12	N	no	
SR	15	N	no	
C	15	N	no	
S	20	N	no	
FLY	20	N	no	
SR	2	N	no	
SR	2	N	no	
N, SR	5	N	no	
N	5	N	no	
SR	6	N	no	
N, SR	10	N	no	
N	10	N	no	
AC	12	N	no	
AC	20	N	no	5 eggs
AC	25	N	no	
AC	30	N	no	
LC	50	N	no	
		N	no	chases other RBNU
		N	no	
		N	no	
		N	no	
CB	5	N	no	
NR	10	N	no	
CB	10	N	no	
		N	no	chases other RBNU
SW	0.5	N	no	
SR	0.5	N	no	
SR	1	N	no	
AC	1	N	no	
CB	1	N	no	
N, SR	2	N	no	
SR	2	N	no	
SR	2	N	no	chases 2 other TRES
N	2	N	no	
SR	2	N	no	
L	3	N	no	
AC	3	N	no	
FO	3	N	no	
SR	3	N	no	

FO	4	N	no	
FO	7	N	no	
FO	10	N	no	
SR	10	N	no	
FO	10	N	no	
SR	15	N	no	
N	1	N	no	
SR	2	N	no	
PR	2	N	no	
SR	3	N	no	
AC	4	N	no	
SR	4	N	no	
SR	7	N	no	
FLY	10	N	no	
AC	15	N	no	
		N	no	
		N	no	chases RBNU
		N	no	
		N	no	
		N	no	
SR	10	N	no	
SR	15	N	no	
C	15	N	no	
SR, C	15	N	no	
C	15	N	no	
S	15	N	no	
SR	20	N	no	chases RBNU
C	20	N	no	
C	20	N	no	
SR, S	20	N	no	
FLY	20	N	no	
C	40	N	no	
LC	30	N	no	
LC	50	N	no	
LC	50	N	no	
LC	50	N	no	
LC	50	N	no	
		N	no	
		N	no	
		N	no	continues to sing until 19:36
		N	no	
		N	no	
		N	no	
		N	no	

		N	no	
		N	no	
		N	no	
SR	3	N	no	
FB	3	N	no	
N	4	N	no	
LC	5	N	no	
FLY, LC	10	N	no	
SR	10	N	no	
FLY	15	N	no	
LC	15	N	no	
LC	20	N	no	
LC	25	N	no	
LC	30	N	no	
		N	no	
		N	no	
		N	no	
		N	no	
		N	no	
CB	10	N	no	
S	1	N	no	
SR	1	N	no	
S	1.5	N	no	
SR, S	2	N	no	
S, SR	2	N	no	enters cavity with nest supplies
SR	8	N	no	
S	10	N	no	
S	15	N	no	
LC	10	N	no	
AC	10	N	no	
LC	15	N	no	
LC	15	N	no	
AC	15	N	no	
LC	20	N	no	
LC	20	N	no	
LC	25	N	no	
LC	40	N	no	
		N	no	
		N	no	
		N	no	RBNU seen nearby
		N	no	chases BCCH
		N	no	
SR	3	N	no	
FLY	3	N	no	

AC	6	N	no
SR	8	N	no
CB, AC	8	N	no
SR	10	N	no
FLY	20	N	no
LC	20	N	no
LC	30	N	no
		N	no
		N	no
		N	no
		N	no
		N	no
C	15	N	no
S	15	N	no
S	15	N	no
C	30	N	no
N	2.5	N	no
SR	5	N	no
CB	5	N	no
SR	7	N	no
SR	10	N	no
C	25	N	no
C	30	N	no
AC	30	N	no
LC	40	N	no
LC	40	N	no
		N	no
		N	no
		N	no
		N	no
		N	no
N, C	15	N	no
LC	50	N	no
		N	no
		N	no
		N	no
		N	no
		N	no
S	20	N	no
C	25	N	no
S	15	N	no
SR	2	N	no
SR	3	N	no
FLY	12	N	no

LC	30	N	no
LC	40	N	no
LC	50	N	no
LC	50	N	no
		N	no
		N	no
		N	no
		N	no
		N	no
C	35	N	no
SR	1	N	no
S	1	N	no
FLY	3	N	no
N, SR	3	N	no
FLY	12	N	no
FLY	15	N	no
C, SR	25	N	no
S	30	N	no
SR, AC	10	N	no
SR	15	N	no
AC	20	N	no
AC	25	N	no
C	35	N	no
LC	50	N	no
LC	50	N	no
		N	no
		N	no
		N	no
		N	no
		N	no
C	10	N	no
SR	5	N	no
SR	6	N	no
SR	10	N	no
FLY	20	N	no
LC	30	N	no
LC	30	N	no
LC	30	N	no
C	40	N	no
		N	no
		N	no
		N	no
		N	no
S	10	N	no

		N	no	
C	15	N	no	
LC	40	N	no	
		N	no	
		N	no	
		N	no	
		N	no	
		N	no	
A	0	N	no	
S, SR	0.5	N	no	
FB	0.5	N	no	
S, SR	1	N	no	
SR	2	N	no	
N	3	N	no	
SR, FO	3	N	no	
S, SR	3	N	no	
N, SR	3	N	no	
SR	3	N	no	
S, SR	4	N	no	
S, SR	4	N	no	carrying food
S, SR	4	N	no	
S, SR	5	N	no	
S	5	N	no	
SR	8	N	no	same BCCH present throughout
FO	10	N	no	
S	10	N	no	
S	10	N	no	
S	10	N	no	
S, FO	12	N	no	
S	15	N	no	
S	15	N	no	
S	15	N	no	
LC	15	N	no	
LC	15	N	no	
LC	20	N	no	
		N	no	
		N	no	
		N	no	
		N	no	
CB	3	N	no	unknown bird chases BCCH
		N	no	
C	25	N	no	
S	40	N	no	
LC	30	N	no	

LC	30	N	no
LC	40	N	no
		N	no
		N	no
		N	no
		N	no
		N	no
FO	5	N	no
FO, C	8	N	no
LC, N	8	N	no
LC	8	N	no
LC	10	N	no
LC	10	N	no
LC	12	N	no
C, SR	15	N	no
C	15	N	no
LC	30	N	no
LC	50	N	no
		N	no
		N	no
		N	no
		N	no
		N	no
C	20	N	no
LC	18	N	no
LC	25	N	no
LC	50	N	no
		N	no
		N	no
		N	no
		N	no
		N	no
C	20	N	no
AC	30	N	no
AC	30	N	no
AC	30	N	no
		N	no
		N	no
		N	no
		N	no
		N	no
C	35	N	no
LC	50	N	no
AC	50	N	no

LC	50	N	no
		N	no
		N	no
		N	no
		N	no
		N	no
SW, S	0.5	N	no
N	1	N	no
SR	2	N	no
AC, L, WF	5	N	no
L	5	N	no
S	5	N	no
S	10	N	no
C	10	N	no
N, S	12	N	no
S	15	N	no
FO	15	N	no
FO	15	N	no
S	15	N	no
S, FLY	20	N	no
SR, N	4	N	no
SR, C	12	N	no
C	30	N	no
LC	35	N	no
LC	40	N	no
		N	no
		N	no
		N	no
		N	no
		N	no
S	25	N	no
SR	8	N	no
SR	10	N	no
		N	no
		N	no
		N	no
		N	no
		N	no
S	40	N	no
SR	10	N	no
LC	20	N	no
LC	50	N	no
		N	no
		N	no

		N	no
		N	no
		N	no
S	30		
S	30		
S	30		
S	40		
S	40		
L	2		
SR	4		
AC	5		
S	5		
AC	6		
AC,L	7		
S	7		
S	10		
S	10		
S	15		
S	15		
S	15		
C	30		
S	40		
S	40		
S	60		
S	60		
S	60		
S	60		
S	60		
S	70		
S	15		
	60		
N, LIP			
C	20		NO
S	20		NO
S	30		NO
			NO
			NO
			NO
C	15	PN	

S		80	PN	no responses to recordings
AR		5	PN	
A		0	PN	
A		0	PN	
N		0.5	PN	
S		3	PN	
EN		5	PN	
FL		5	PN	
S		5	PN	
AR		8	PN	
C		8	PN	
FO		10	PN	
AR		15	PN	
FO		20	PN	
C		60	PN	
-	-		PN	can't compare - MOCH pr. Not around at all
SW	-		PN	can't compare - MOBL nest in tree - swoops model!
S		1	PN	
S		2	PN	
C		40	PN	

RANK2	RANK3	RANK4
1		

4

7 4

e to playback?

ssive BCCHs

6

1

9
9

9

9

9 6

1

7790

ıd

nale) calls in area

sed MOCH away

5

7

4

‹e up, sang, then flew off while MOCH watched

[illegible]CH & BCCH songs

4

7

6

9
6

7
7

after playback ends - 2 others had probably already fledged

ter time

uff brown back instead of slate/gray

9

1

1

10
10

10 6

1

6
6
6

1

9

6
10 9 6

1

6

6

model
model
model
