# Supplementary material for: Cavity nesting birds show behavioural plasticity to simulated territorial intrusions in response to natural resource pulses
Source: Sci Rep. 2025 Mar 18;15:9338. doi: 10.1038/s41598-025-93109-y (PMC11920054; doi:10.1038/s41598-025-93109-y)
Supplement: Supplementary file 5 — Supplementary Material 5 [file 41598_2025_93109_MOESM5_ESM.pdf]

```
#   Table S2. R script to accompany the manuscript, Cavity-nesting bird species show behavioural plasticity to simulated territorial intrusions
#   in response to natural resource pulses. Script includes instructions for analysis of data included in Supplementary Table S1.


rm(list = ls())
PB <- read.csv("Playbackdata_no_header.csv")

PB <- data.frame(Date = PB$Date, Year = PB$Year, Site = PB$Site, OBS = PB$OBS, Plot = PB$Plot, NOR = PB$N.O.R, Nestspp = PB$Current.nest.spp, NestID = PB$Nest.ID, Lastnestspp = PB$Last.nest.spp, Lastnstyr = PB$Last.nest.yr, Order = PB$Play.order, Start = PB$Start.time, Model = PB$Model, Track = PB$Track, Time = PB$Time, Stoptime = PB$Stopwatch.time, Spp = PB$Species, Sex = PB$Sex, Num = PB$No.ads.pres, Behav = PB$Behaviour1, Dist = PB$Distance.m, Nest.status = PB$Nest.status, Nrnest.stat = PB$Nrst.Nest.status, AT = PB$LiveAT.ha, BI = PB$LP2BI, Mden = PB$MOCH, Bden = PB$BCCH, Rden = PB$RBNU, TAHU = PB$TAHU)


#combine ON with N and OR with O#

PB$NOR1 <- ifelse(PB$NOR =="ON", "N", "")
PB$NOR2 <- ifelse(PB$NOR =="OR", "O", "")
PB$NOR3a <- ifelse(PB$NOR =="R", "A", "")
PB$NOR4 <- ifelse(PB$NOR == "N", "N", "")
PB$NOR5 <- ifelse(PB$NOR == "O", "O", "")


PB$NOR3 <- do.call(paste, c(PB[c("NOR1", "NOR2", "NOR3a", "NOR4", "NOR5")], sep = ""))

PB3 <- subset(PB, PB$NOR3 != "")




#combine blank with none models#

PB3$Model1 <- ifelse(PB3$Model == "", "anone", "")
PB3$Model2a <- ifelse(PB3$Model == "none", "anone", "")
PB3$Model3a <- ifelse(PB3$Model == "BCCH", "BCCH", "")
PB3$Model3b <- ifelse(PB3$Model == "MOCH", "MOCH", "")
PB3$Model3c <- ifelse(PB3$Model == "RBNU", "RBNU", "")

PB3$Model2 <- do.call(paste, c(PB3[c("Model1", "Model2a", "Model3a", "Model3b", "Model3c")], sep = ""))

PB4 <- subset(PB3, PB3$Model2 != "")



#take only MOCH/RBNU/BCCH species responses#

PB4$Spp1 <- ifelse(PB4$Spp == "MOCH", "MOCH", "")
PB4$Spp2 <- ifelse(PB4$Spp == "RBNU", "RBNU", "")
PB4$Spp3 <- ifelse(PB4$Spp == "BCCH", "BCCH", "")

PB4$Spp4 <-  do.call(paste, c(PB4[c("Spp1", "Spp2", "Spp3")], sep = ""))

PB4 <- subset(PB4, Spp4 != "")


write.csv(PB4, file="OutputPlaybacks1.csv")
```

```
#Clean up dist data#

PB4a <- subset(PB4, Dist != "-")
PB4b <- subset(PB4a, Dist != "?")
PB4c <- subset(PB4b, Dist != "IN AREA")
PB4d <- subset(PB4c, Dist != "not recorded")

PB4d$Dista <- ifelse(PB4d$Dist == "25+", "25", "")
PB4d$Distb <- ifelse(PB4d$Dist == "5-100", "5", "")
PB4d$Distc <- ifelse(PB4d$Dist == "5?", "5", "")

PB4e <- subset(PB4d, Dist != "25+")
PB4f <- subset(PB4e, Dist != "5-100")
PB4g <- subset(PB4f, Dist != "5?")

PB4g$Dist2 <-  do.call(paste, c(PB4g[c("Dist", "Dista", "Distb", "Distc")], sep = ""))

write.csv(PB4g, file="OutputPlaybacks2.csv")





#use dplyr to extract min distances

library(dplyr)

min.dist <- PB4g %>%
  group_by(Model2, NOR3, Spp4, Site, Date, Start, Year, Mden, Rden, Bden, TAHU, BI, AT)
%>%
  summarize(
    min_value = min(Dist2, na.rm = TRUE),
    count = n(),
    .groups = 'drop'  # Optional: Ungroup after summarizing
  )

# Subsetting the results
min.dist2 <- min.dist %>%
  filter(!is.na(min_value))  # Change this to min_value then back to Dist2.min
min.dist2$Dist2.min <- min.dist2$min_value

write.csv(min.dist2, file="OutputMinDist.csv")


#convert min distances to numeric, and others to factors####


min.dist2$Dist2.min2 <- as.numeric(min.dist2$Dist2.min)
min.dist2$Spp4 <- as.factor(min.dist2$Spp4)
min.dist2$Model2 <- as.factor(min.dist2$Model2)
min.dist2$NOR3 <- as.factor(min.dist2$NOR3)
min.dist2$Year <- as.factor(min.dist2$Year)








#######EXPORT TO HERE for figs, models scripts########
```

```
#include Order, Current.nest.spp and Nest.status ###



min.dist <- PB4g %>%
  group_by(Model2, NOR3, Spp4, Site, Date, Start, Year, Mden, Rden, Bden, BI, AT, TAHU,
Nest.status, Order, Nestspp, Plot) %>%
  summarize(
    Dist2_min = min(Dist2, na.rm = TRUE),
    count = n(),
    .groups = 'drop'  # Optional: Ungroup after summarizing
  )

# Subsetting the results
min.dist2 <- min.dist %>%
  filter(Dist2_min != "")


#convert min distances to numeric, and others to factors####

min.dist2$Dist2.min2 <- as.numeric(min.dist2$Dist2_min)
min.dist2$Spp4 <- as.factor(min.dist2$Spp4)
min.dist2$Model2 <- as.factor(min.dist2$Model2)
min.dist2$NOR3 <- as.factor(min.dist2$NOR3)
min.dist2$Year <- as.factor(min.dist2$Year)








#View data##



head(min.dist2)

plot(min.dist2$Spp4, min.dist2$Dist2.min2)

plot(min.dist$Dist2.length, min.dist$Dist2.min)
```
